# Light has a principal role in the Arabidopsis transcriptomic response to the spaceflight environment

**DOI:** 10.1038/s41526-024-00417-0

**Published:** 2024-08-06

**Authors:** Mingqi Zhou, Robert J. Ferl, Anna-Lisa Paul

**Affiliations:** 1 Department of Horticultural Sciences, University of Florida, 2550 Hull Road, Fifield Hall, Gainesville, FL 32611 USA; 2 UF Research, University of Florida, 1523 Union Rd, Grinter Hall, Gainesville, FL 32611 USA; 3 Interdisciplinary Center for Biotechnology Research, University of Florida, 2033 Mowry Road, Gainesville, FL 32610 USA

**Keywords:** Plant sciences, Molecular biology

## Abstract

The Characterizing Arabidopsis Root Attractions (CARA) spaceflight experiment provides comparative transcriptome analyses of plants grown in both light and dark conditions within the same spaceflight. CARA compared three genotypes of Arabidopsis grown in ambient light and in the dark on board the International Space Station (ISS); Col-0, Ws, and *phyD*, a phytochrome D mutant in the Col-0 background. In all genotypes, leaves responded to spaceflight with a higher number of differentially expressed genes (DEGs) than root tips, and each genotype displayed distinct light / dark transcriptomic patterns that were unique to the spaceflight environment. The Col-0 leaves exhibited a substantial dichotomy, with ten-times as many spaceflight DEGs exhibited in light-grown plants versus dark-grown plants. Although the total number of DEGs in *phyD* leaves is not very different from Col-0, *phyD* altered the manner in which light-grown leaves respond to spaceflight, and many genes associated with the physiological adaptation of Col-0 to spaceflight were not represented. This result is in contrast to root tips, where a previous CARA study showed that *phyD* substantially reduced the number of DEGs. There were few DEGs, but a series of space-altered gene categories, common to genotypes and lighting conditions. This commonality indicates that key spaceflight genes are associated with signal transduction for light, defense, and oxidative stress responses. However, these key signaling pathways enriched from DEGs showed opposite regulatory direction in response to spaceflight under light and dark conditions, suggesting a complex interaction between light as a signal, and light-signaling genes in acclimation to spaceflight.

## Introduction

Plants have utilized light and gravity as environmental cues throughout their evolutionary history to navigate and shape their growth and development^1,2^. Microgravity spaceflight environments are being explored with considerable effort to inform understanding of growth signals as well as the initiative to create suitable habitats and approaches for including plants in long-term space missions^3,4^. On orbit, where gravity does not act as a primary tropic force applied on plants, light is used as an orienting tropism for plant growth^5,6^. Plants remodel their growth strategy in different light conditions during spaceflight^5,7^. In the Advanced Plant Experiment 01 (APEX-01) on board the International Space Station (ISS), Arabidopsis (*Arabidopsis thaliana*) seedlings utilized the established light gradient within the growth hardware to direct their tropic growth^5,8^. Experiments have also revealed the dramatic impact of light wavelengths on the morphology of Arabidopsis seedlings grown in microgravity and fractional gravity^6,9–11^. Due to space facility limitations, as well as payload capacity in spaceflight, most of plant transcriptomic analyses have been performed using either constant light or constant dark conditions^8,12–15^. While extensive analyses have been conducted on plants cultivated in various spaceflight-associated environments, including some experiments without light, direct comparisons of transcriptomic responses to spaceflight under light and dark conditions in the same flight are still limited. As one of the very few examples, Villacampa et al. ^16^ grew Arabidopsis seedlings onboard ISS in a light/dark cycle for 4 days, prior to treatment with constant red light or dark for 2 days^16^. Compared with dark treatment, photo-stimulation of red light enhanced the adaptive response to 0.33 *g* by upregulating multiple stress-responsive pathways. In contrast, the CARA experiment comprised prolonged plant growth in constant light or in constant dark environments after germination to reveal the role of light in spaceflight-associated transcriptome regulation^17^.

The Characterizing Arabidopsis Root Attractions (CARA) experiment set up a side-by-side comparison between the plants grown in the light and plants kept dark in the same location on the ISS. Half of the plants were exposed to ambient cabin light to provide a primarily non-directional source of light for growth and development. The other half were kept wrapped in blackout cloth. This comparison thereby facilitated the direct dissection of the role of light in plant molecular responses in space^17^. Three genotypes of Arabidopsis were used: Col-0 and Ws wildtypes, and a mutant in the Col-0 background for the phytochrome D (*PHYD*) gene, which encodes a member of phytochrome family, the plant red/far-red photoreceptors^18^. Previous studies of the roots from CARA plants identified the genes and signaling pathways engaged by the root tip cells on orbit^17^. The influences of genotype on the plant physiological adaptation to spaceflight were measured by transcriptomic patterns. Presented here is using the same set of plants as reported by Paul et al. ^17^ to perform a comprehensive analysis of the impact of light on the genome-wide transcriptional response in Arabidopsis leaves in the spaceflight environment, showing genotype- and ecotype-related signaling pathways and gene categories that are changed in response to spaceflight in the light and in the dark. The comparison of tissue-related transcriptomic responses to space between leaves and root tips is discussed. The present work has revealed a sharp contrast between leaf transcriptomic responses to orbital flight in the light and the responses that occur in the dark.

## Results

### Leaves showed stronger transcriptional responses to spaceflight than root tips

**Fig. 1:**
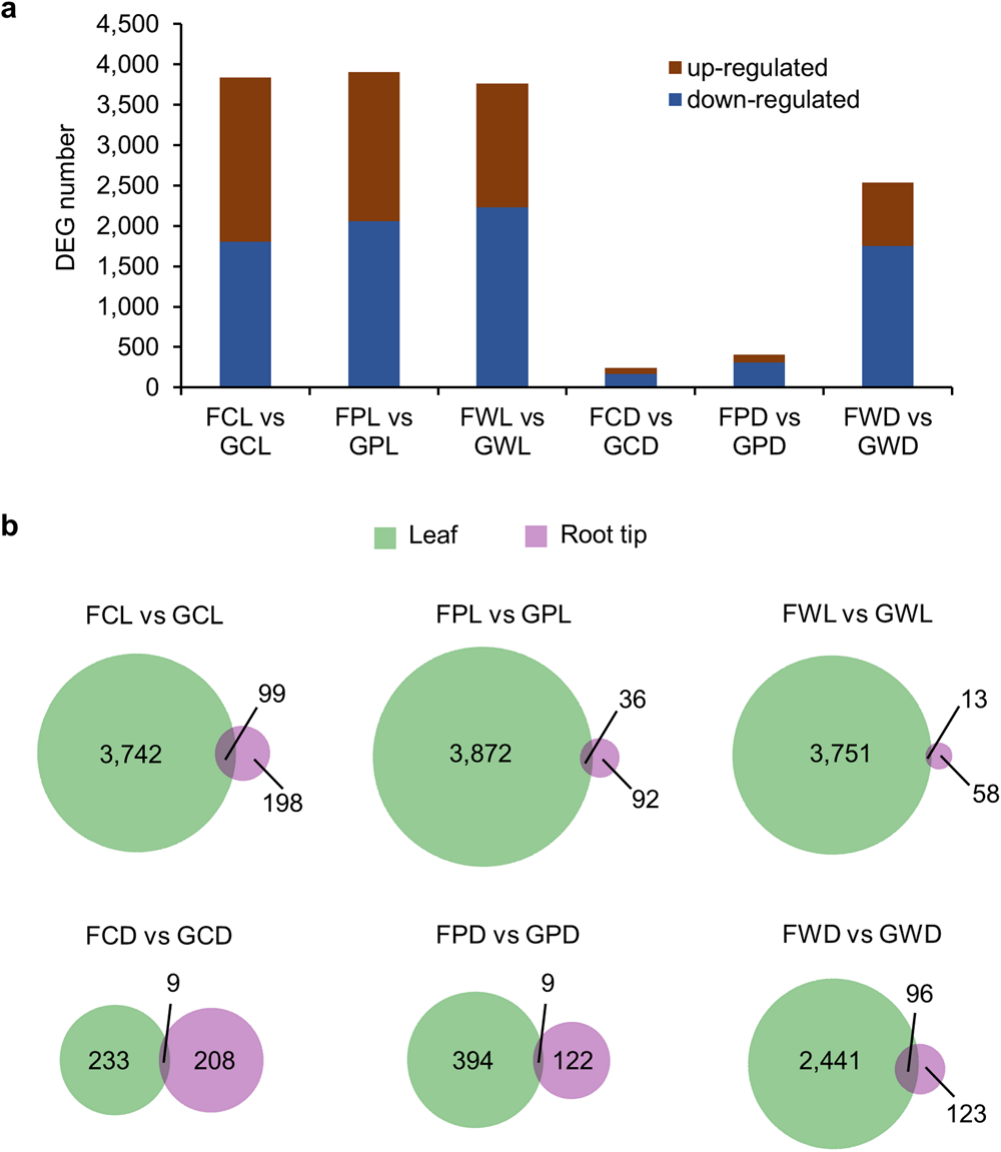
DEGs detected in Arabidopsis leaves in the CARA experiment. Samples include Flight (F) or Ground Control (G) for Arabidopsis with three genotypes, Col-0 (C), *phyD* (P) and Ws (W), grown in the light (L) or in the dark (D) conditions. The threshold is log2(fold-change) >1 or < −1, Padj <0.05. **a** Numbers of DEGs detected in pair-wise comparisons between samples of Flight (F) and Ground Control (G). **b** Venn diagrams show overlap between DEGs detected in leaves and root tips for each comparison.

As described in Paul et al. ^17^, the CARA experiment deployed Flight (F) or Ground Control (G) samples composed of Arabidopsis plants with three genotypes, Col-0 (C), *phyD* (P) and Ws (W), grown in the light (L) or in the dark (D) conditions^17^. Figure 1 summarizes the differential analyses of the transcriptome in leaf tissues. The comparative analysis generated differentially expressed genes (DEGs) with significant changes that were more than two-fold in the transcription level (log2(fold-change) >1 or < -1, and FDR adjusted *P* value (Padj) < 0.05) between samples of Flight and Ground Control (Fig. 1a). More DEGs were detected in the light than in the dark for each genotype in leaves, while there was an opposite trend of DEG number in the comparison between the light and the dark in root tips (Fig. 1b). Overall, leaf and root tip tissues showed little overlap of DEGs in both the light and the dark. Substantially more DEGs were detected in leaves than in root tips, suggesting that leaves are more sensitive in transcriptional response to spaceflight than root tips, especially in the light (Fig. 1b).

### Light condition is a critical factor affecting transcriptional regulation in plant response to spaceflight

**Fig. 2:**
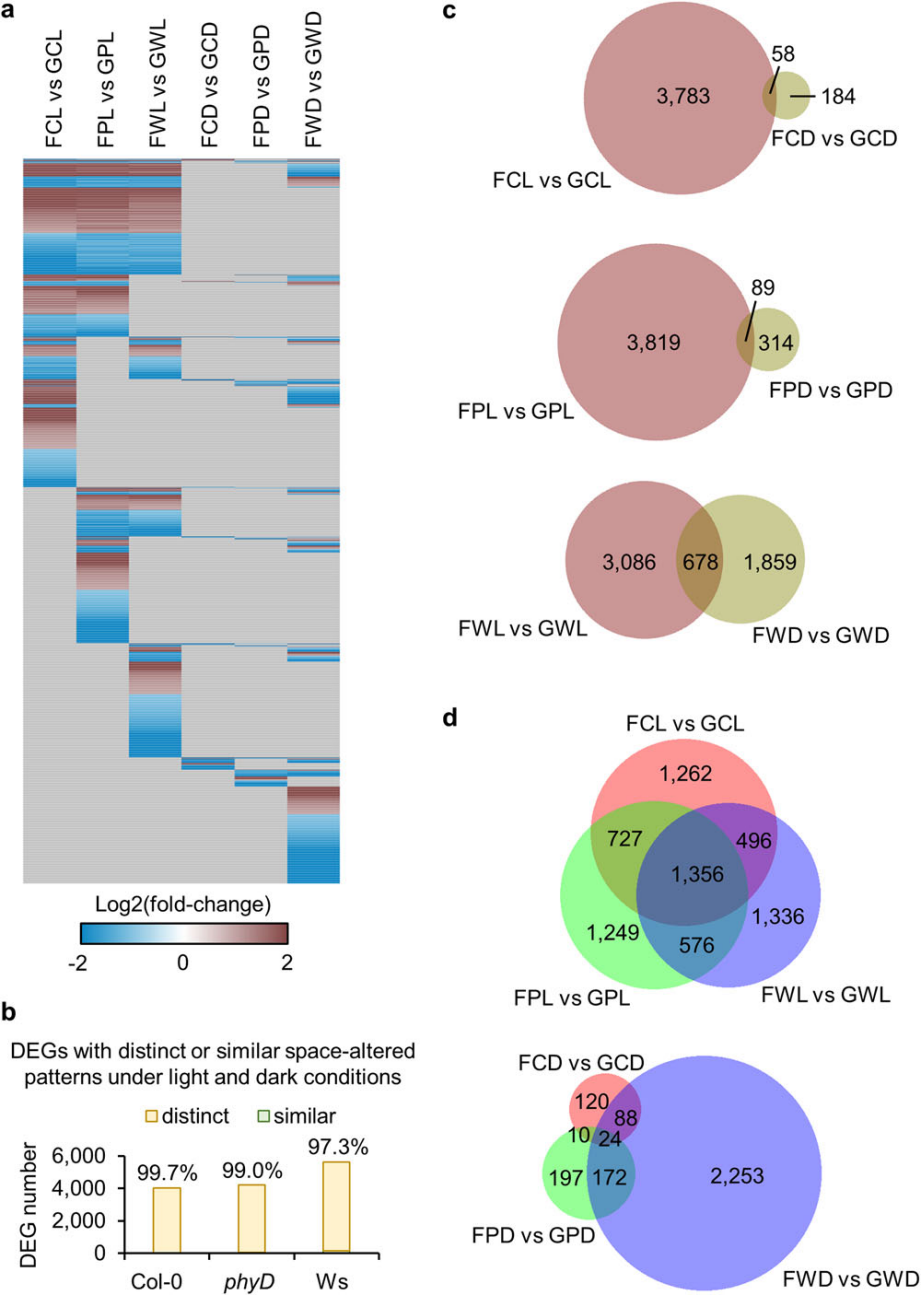
Differential expression of CARA DEGs between Flight and Ground Control in Arabidopsis leaves. The threshold is log2(fold-change) >1 or < -1, Padj <0.05. **a** Expression pattern of 8477 DEGs that were significantly changed between Flight (F) and Ground Control (G) in at least one comparison for Col-0 (C), *phyD* (P) or Ws (W) in the light or in the dark. Hierarchical clustering of the heatmap is done using one minus cosine similarity. Genes with no significant changes or no expression data detected in RNA-seq are indicated in gray. **b** DEGs with distinct or similar space-altered patterns in response to spaceflight for leaves grown in the light and in the dark. There are 4014 out of 4025 (99.7%), 4179 out of 4222 (99%) and 5470 out of 5623 (97.3%) DEGs showing distinct space-altered expression patterns in the light and in the dark for Col-0, *phyD* and Ws, respectively. Venn diagrams show the overlap of DEGs between leaf responses to spaceflight in the light and in the dark for each genotype (**c**), and the overlap of DEGs among leaf responses to spaceflight in three genotypes in the light or in the dark (**d**).

**Fig. 3:**
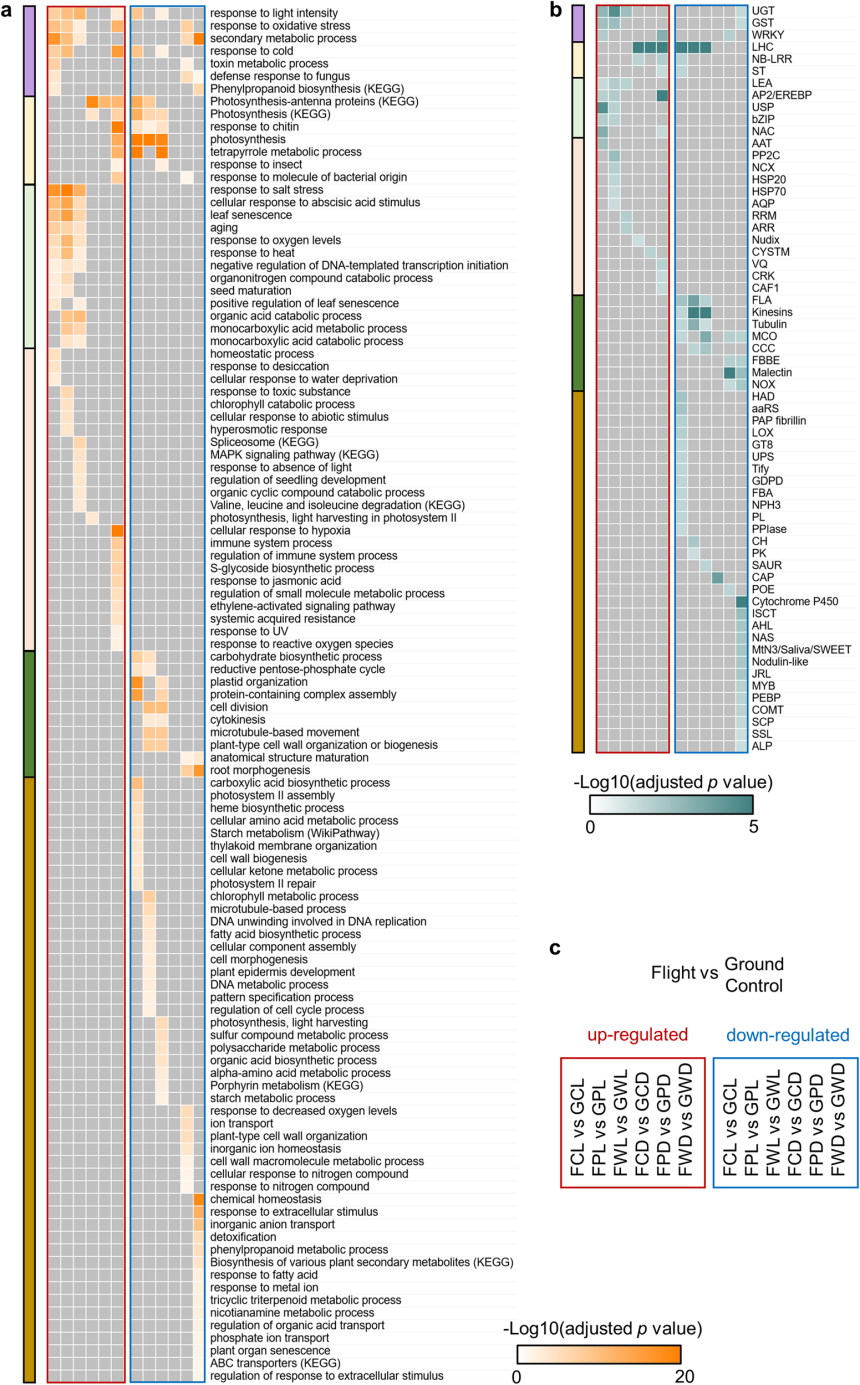
Enrichment analyses of Differentially Expressed Genes (DEGs) reveal biological pathways assocaiated with the spaceflight response. (**a**) and gene family (**b**) using CARA leaf DEGs between Flight and Ground Control in different comparisons. The threshold for DEGs is log2(fold-change) >1 or < -1, Padj <0.05, and for enrichment is Padj <0.05. Heatmaps are showing the -log10(Padj) for each significantly enriched item. Significantly enriched pathways or gene families are clustered according to the enrichment pattern. Clusters are indicated using colored bars on the left of the heatmaps. **c** The order of the comparisons used to generate the DEGs for enrichment analysis is shown. Each column in the heatmaps indicates one comparison. Heatmaps in (**a**) and (**b**) use the same order as shown in (**c**).

In leaves, 8477 DEGs were detected between Flight and Ground Control in at least one comparison for Col-0, *phyD* or Ws ecotype in the light or in the dark (Fig. 2a and Supplementary Table 1). Distinct patterns of spaceflight responses were observed between plants grown in the light (left three columns of heatmap in Fig. 2a) and the dark (right three columns of heatmap in Fig. 2a). The significant regulation of DEGs in the same direction in both the light and the dark were defined as “similar” space-altered patterns, otherwise they were designated as “distinct” patterns. There were 99.7%, 99% and 97.3% DEGs in Col-0, *phyD* and Ws showed distinct space-altered patterns in the light and in the dark, respectively (Fig. 2b). Although there were 58, 89 and 678 DEGs presented in both the light and the dark in three genotypes, only 11, 43 and 153 DEGs showed similar space-altered patterns, respectively (Fig. 2b, c). In other words, 81%, 52% and 77% DEGs in three genotypes shown in both the light and the dark were regulated in opposite direction by spaceflight under the two conditions. Overall, the overlap of spaceflight responsive DEGs under light and dark environments was also smaller than that between different genotypes (Fig. 2c, d). Enrichment analyses of gene ontology and classification were performed on DEGs in each condition to reveal signaling pathways and gene families that were significantly altered in response to spaceflight (Fig. 3a, b). The orientation of comparisons was shown in Fig. 3c. Distinct pathways were altered by spaceflight in the light (left three columns of each heatmap in Fig. 3a) and in the dark (right three columns of each heatmap in Fig. 3a). Responses to abiotic stresses including salt, heat and abnormal oxygen levels as well as aging pathways were enriched for up-regulated genes of all three genotypes only in the light. More cell wall organization and biogenesis related pathways were repressed in the light than in the dark. In Ws, there were unique dark-responses typically associated with hypoxic and immune responses that were up-regulated, and chemical homeostasis and ion transport related pathways that were down-regulated. In contrast, no down-regulated pathway was enriched for Col-0 in the dark. There were also some similar signaling pathways deployed for response to spaceflight in both the light and the dark, but with different regulatory patterns. For instance, some photosynthesis related pathways were repressed in the light but induced in the dark for both Col-0 and Ws. Defense responses to chitin and insect were also repressed in the light but induced in the dark for Ws, while secondary metabolic process was induced in the light but repressed in the dark for Ws and *phyD*. Accordingly, the enriched gene families related to each signaling pathway were identified (Fig. 3b). Genes with significant responses to spaceflight in both the light and the dark could also be regulated in different patterns. As an example, light-harvesting chlorophyll a/b-binding (LHC) genes functioning in photosynthesis and stress responses^19^ were repressed in the light and induced in the dark in all three genotypes. The repression of genes encoding chlorophyll a/b binding proteins was among the first transcriptome responses associated with the spaceflight^20^.

**Fig. 4:**
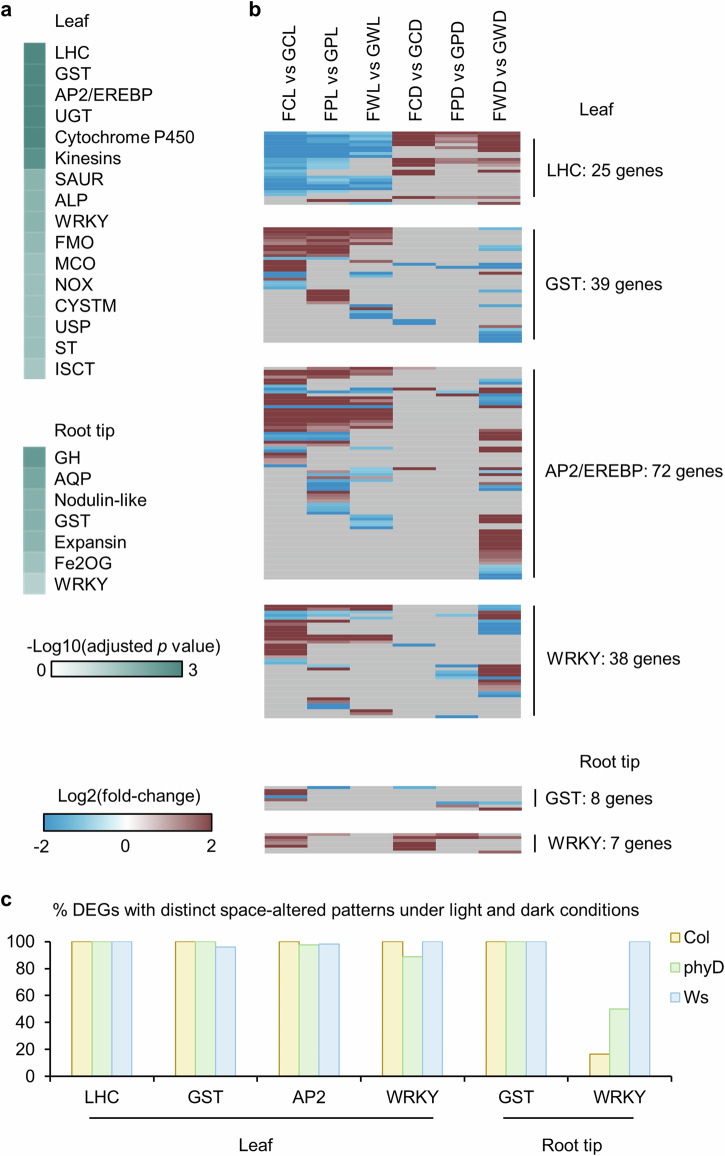
Light condition-determined expression pattern of genes from key spaceflight-altered gene families. **a** Gene family enrichment analysis for 8477 leaf DEGs and 716 root tip DEGs between Flight and Ground Control in at least one comparison of the CARA experiments. The threshold for DEGs is log2(fold-change) >1 or < –1, Padj <0.05, and for enrichment is Padj <0.05. Heatmap is showing the -log10(Padj) for each significantly enriched gene family. **b** The differential expression pattern of genes from LHC, GST, AP2/EREBP and WRKY families in leaves, and GST and WRKY families in root tips. The heatmap is plotted in the same way as Fig. 2c. **c** Percentage of DEGs from listed gene families with distinct space-altered patterns in response to spaceflight under light and dark conditions in leaves and root tips, respectively.

The expression patterns of DEGs from gene families enriched in spaceflight were compared between the light and the dark in both leaves and root tips. Gene family enrichment analyses were performed on 8477 DEGs from leaves and 716 DEGs from root tips^17^ DEGs between Flight and Ground Control (Fig. 4a). Glutathione-S-Transferase (GST) and WRKY families involved in stress response and metabolism signaling^21,22^ were enriched in both tissues. Differential expression patterns in response to spaceflight were plotted for leaf DEGs from GST, WRKY gene families, and the most highly enriched families of LHC and APETALA2/ethylene-responsive element binding protein (AP2/EREBP), as well as root tip DEGs from GST and WRKY families (Fig. 4b). Consistent with the overall transcriptomic responses to spaceflight in leaves, the majority of DEGs from these enriched gene families showed distinct space-altered patterns in the light (left three columns of each heatmap in Fig. 4b) and the dark (right three columns of each heatmap in Fig. 4b). Especially for LHC, none of the 25 genes showed significant regulation in the same direction in any of three genotypes (Fig. 4c). More than 90% DEGs from GST, AP2/EREBP and WRKY in leaves as well as GST in root tips showed distinct space-altered patterns under light and dark conditions in all three genotypes. The only exception is WRKY genes in root tips, exhibiting 17% and 50% DEGs with distinct space-altered patterns in the light and in the dark for Col-0 and *phyD*, respectively, implying the light-independent regulation of WRKY genes in the ecotype of Col-0.

### PHYD-dependent spaceflight-altered genes

**Fig. 5:**
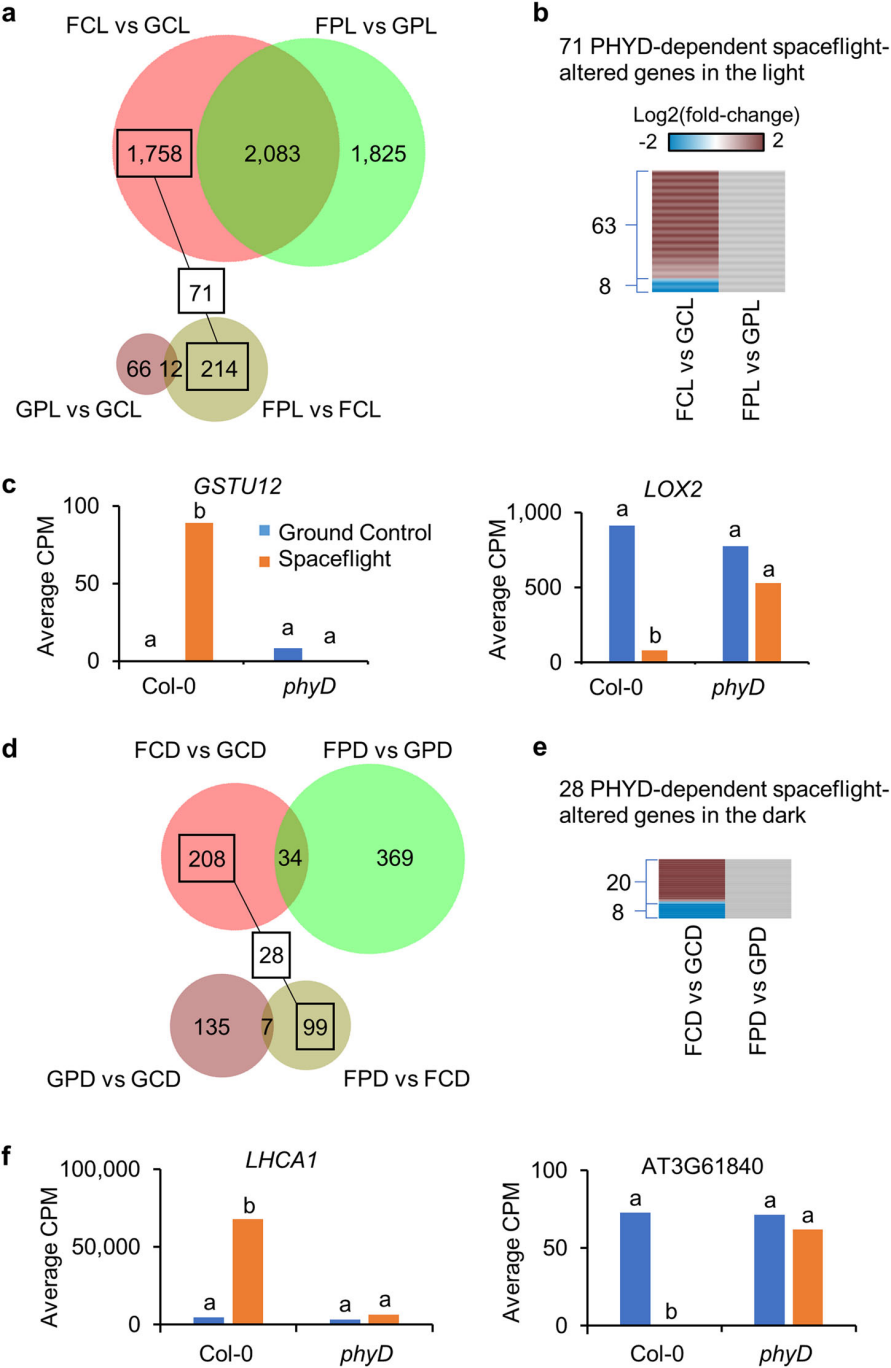
PHYD-dependent spaceflight-altered genes. **a** 71 PHYD-dependent spaceflight-altered genes identified using overlap analysis for DEGs in the light. These genes are significantly changed in transcription by spaceflight in Col-0, but not in *phyD*, and they only show significant difference of transcription between Col-0 and *phyD* in space, not on earth. **b** Differential expression pattern of 71 genes from (**a**) between Flight and Ground Control in Col-0 and *phyD*. **c** Examples of PHYD-dependent spaceflight-altered genes in the light, which were significantly induced (*GSTU12*) or repressed (*LOX2*) by spaceflight in Col-0, but not in *phyD*. **d** 28 PHYD-dependent spaceflight-altered genes are identified using overlap analysis for DEGs in the dark. **e** Differential expression pattern of 28 genes from (**d**) between Flight and Ground Control in Col-0 and *phyD*. **f** Examples of PHYD-dependent spaceflight-altered genes in the dark, which were significantly induced (*LHCA1*) or repressed (AT3G61840) by spaceflight in Col-0, but not in *phyD*. In histograms, gene expression level is indicated by average count per million mapped reads (CPM) in RNA-seq. Lowercase letters indicate significant differences between samples (Padj <0.05 in DESeq2). The heatmap is plotted in the same way as Fig. 2c.

PHYD functions in red/far-red light sensing in Col-0, but Ws is naturally deficient in the gene encoding PHYD^18^. In root tips, mutation of PHYD in Col-0 disrupted the regulation of multiple gene categories during spaceflight, many associated with cell wall metabolism and defense^17^. In leaves, pathways related to cell wall metabolism and defense were also influenced in the spaceflight response in Col-0 but not in the *phyD* mutants (Fig. 3a), suggesting that both root tips and leaves share a set of key pathways regulated by PHYD in response to spaceflight. There were a set of DEGs with significant changes between Flight and Ground Control only in Col-0, but not in *phyD*, as well as significant changes between *phyD* and Col-0 only in spaceflight, but not on earth (Fig. 5a). These genes were defined as PHYD-dependent spaceflight-altered genes, which showed similar expression levels between Col-0 and *phyD* on the ground, but had significant responses only in wild type plants in space (Supplementary Table 2). In the light, there were 71 PHYD-dependent space-altered genes, with 63 up-regulated and 8 down-regulated only in Col-0 (Fig. 5b). *GSTU12* from GST family^21^ and a lipoxygenase gene *LOX2*^23^ were examples showing the up- and down-regulation in space response of Col-0 but not *phyD*, respectively (Fig. 5c). In the dark, 28 PHYD-dependent space-altered genes were identified (Fig. 5d), with 20 up-regulated and 8 down-regulated only in Col-0 (Fig. 5e). *LHCA1* from LHC family^24^ and an auxin-responsive gene AT3G61840^25^ were examples of PHYD-dependent space-altered genes in the dark (Fig. 5f).

### Ecotype-related spaceflight-altered genes in Col-0 or Ws

**Fig. 6:**
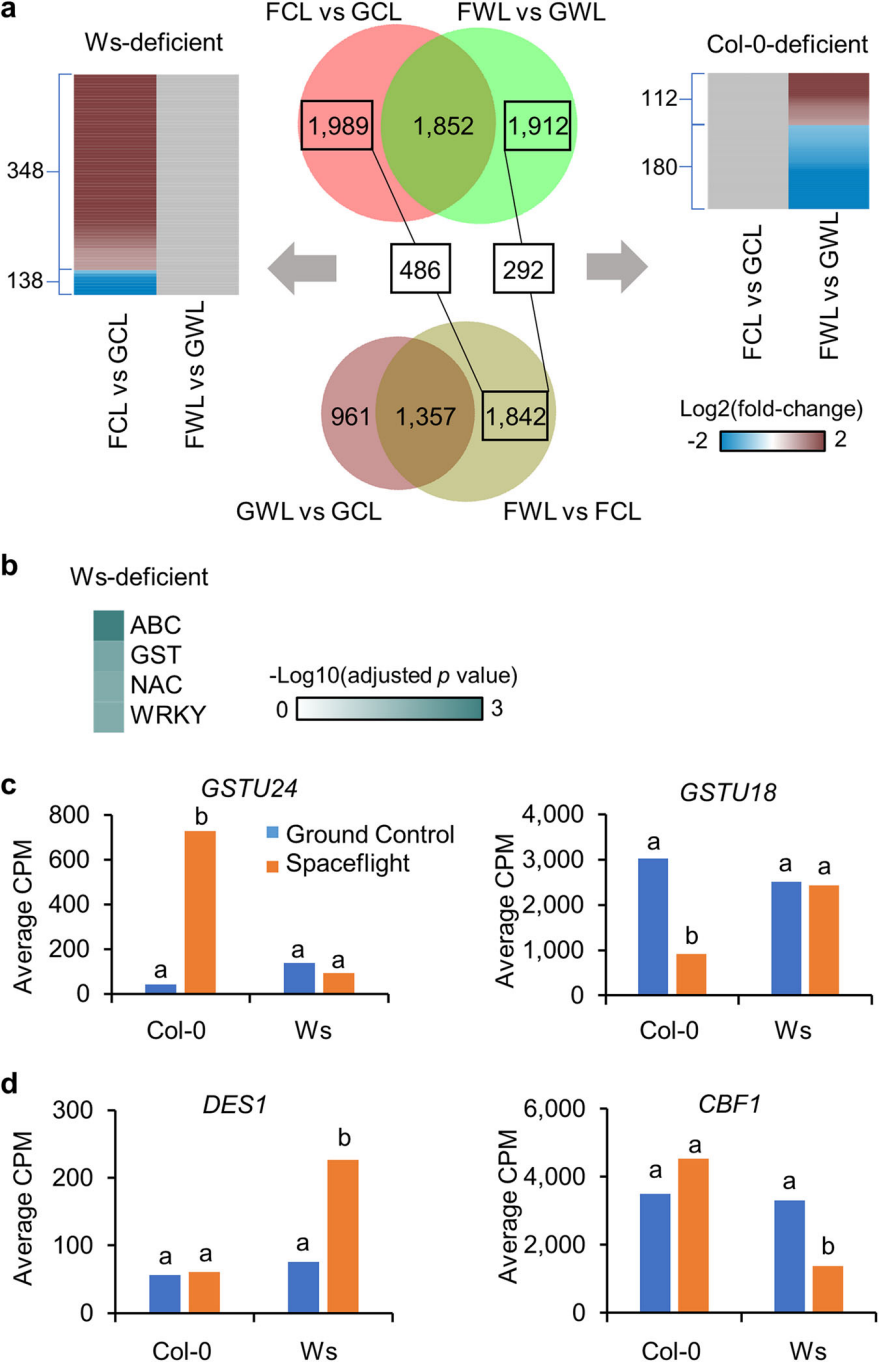
Ecotype-related spaceflight-altered genes in the light. **a** 486 Ws-deficient and 292 Col-0-deficient spaceflight-altered genes were identified in overlap analysis in the samples of the light. These genes are significantly changed in transcription by spaceflight either in Col-0 or in Ws, not in both, and they only show significant difference of transcription between Col-0 and Ws in space, not on earth. Differential expression patterns between Flight and Ground Control in Col-0 and Ws are shown for Ws-deficient and Col-0-deficient genes. The heatmap is plotted in the same way as Fig. 2c. **b** Gene family enrichment analysis using 486 Ws-deficient spaceflight-altered genes. Heatmap is plotted in the same way of Fig. 4a. **c** Examples of Ws-deficient spaceflight-altered genes, which were significantly induced (*GSTU24*) or repressed (*GSTU18*) by spaceflight in Col-0, but not in Ws. **d** Examples of Col-0-deficient spaceflight-altered genes, which were significantly induced (*DES1*) or repressed (*CBF1*) by spaceflight in Ws, but not in Col-0. Gene expression level is presented in the same way as Fig. 5c.

**Fig. 7:**
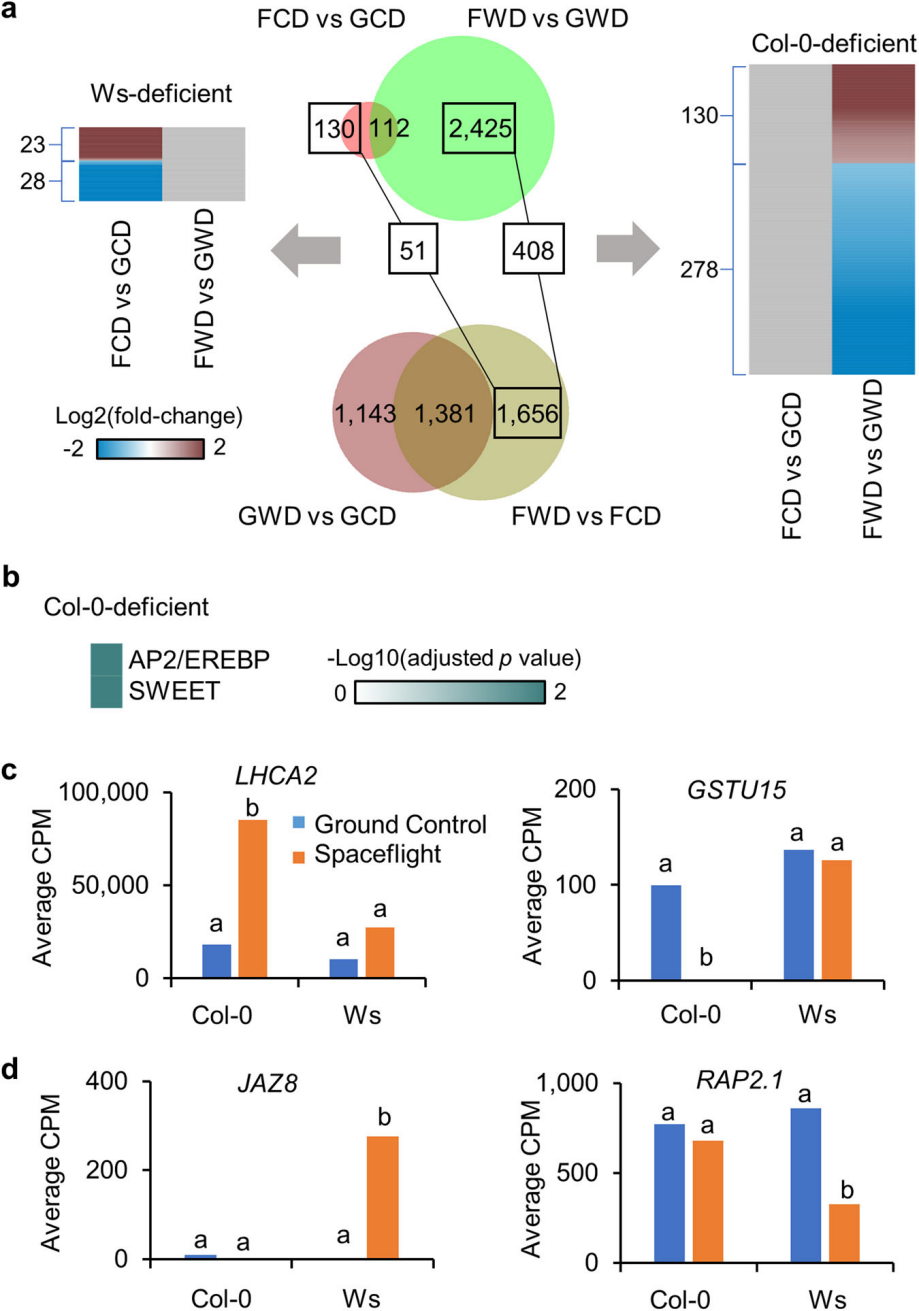
Ecotype-related spaceflight-altered genes in the dark. **a** 51 Ws-deficient and 408 Col-0-deficient spaceflight-altered genes were identified in overlap analysis in the samples of the dark. Differential expression patterns between Flight and Ground Control in Col-0 and Ws are shown for Ws-deficient and Col-0-deficient genes. The heatmap is plotted in the same way as Fig. 2c. **b** Gene family enrichment analysis using 408 Col-0-deficient spaceflight-altered genes. The heatmap is plotted in the same way as Fig. 4a. **c** Examples of Ws-deficient spaceflight-altered genes, which were significantly induced (*LHCA2*) or repressed (*GSTU15*) by spaceflight in Col-0, but not in Ws. **d** Examples of Col-0-deficient spaceflight-altered genes, which were significantly induced (*JAZ8*) or repressed (*RAP2.1*) by spaceflight in Ws, but not in Col-0. Gene expression level is presented in the same way as Fig. 5c.

Next, ecotype-related spaceflight-altered genes were identified through overlap analysis of DEGs in response to spaceflight from Col-0 and Ws. These identified genes showed similar expression levels between Col-0 and Ws on the ground, and had significant differential responses in only one of these two ecotypes in space (Supplementary Table 2). In the light, 486 spaceflight-altered genes presented in Col-0 were not significantly changed in the response of Ws to spaceflight (Fig. 6a). These genes differentially expressed in Col-0 but not in Ws in space, which were defined as Ws-deficient spaceflight-altered genes. They were enriched in four gene families functioning in plant developmental processes and stress responses, including ATP-binding cassette (ABC) transporter^26^, GST^21^, NAC^27^ and WRKY^22^ (Fig. 6b). *GSTU24* and *GSTU18* were two examples of this kind (Fig. 6c). Meanwhile, there were 292 genes altered in Ws but not in Col-0 in response to spaceflight, and they had similar expression levels between Col-0 and Ws on earth (Fig. 6a). They were defined as Col-0-deficient spaceflight-altered genes. No gene family was enriched for these Col-0-deficient spaceflight-altered genes, but abiotic stress responsive genes were observed, such as *DES1*^28,29^ that was only induced in Ws, and *CBF1*^30^ that was only repressed in Ws (Fig. 6d). In the dark, there were 51 and 408 spaceflight-altered genes without space response in Ws and Col-0, respectively (Fig. 7a). The 408 Col-0-deficient spaceflight-altered genes were enriched in AP2/EREBP and SWEET families functioning in plant development and abiotic stress responses^31,32^ (Fig. 7b). *JAZ8* and *RAP2.1* were two examples of this kind (Fig. 7d). No gene family was enriched for 51 Ws-deficient spaceflight-altered genes, but *LHC* and *GST* genes were observed (Fig. 7c).

### PHYD-dependent and ecotype-related spaceflight-altered genes were regulated in a light-dependent manner in response to spaceflight

**Fig. 8:**
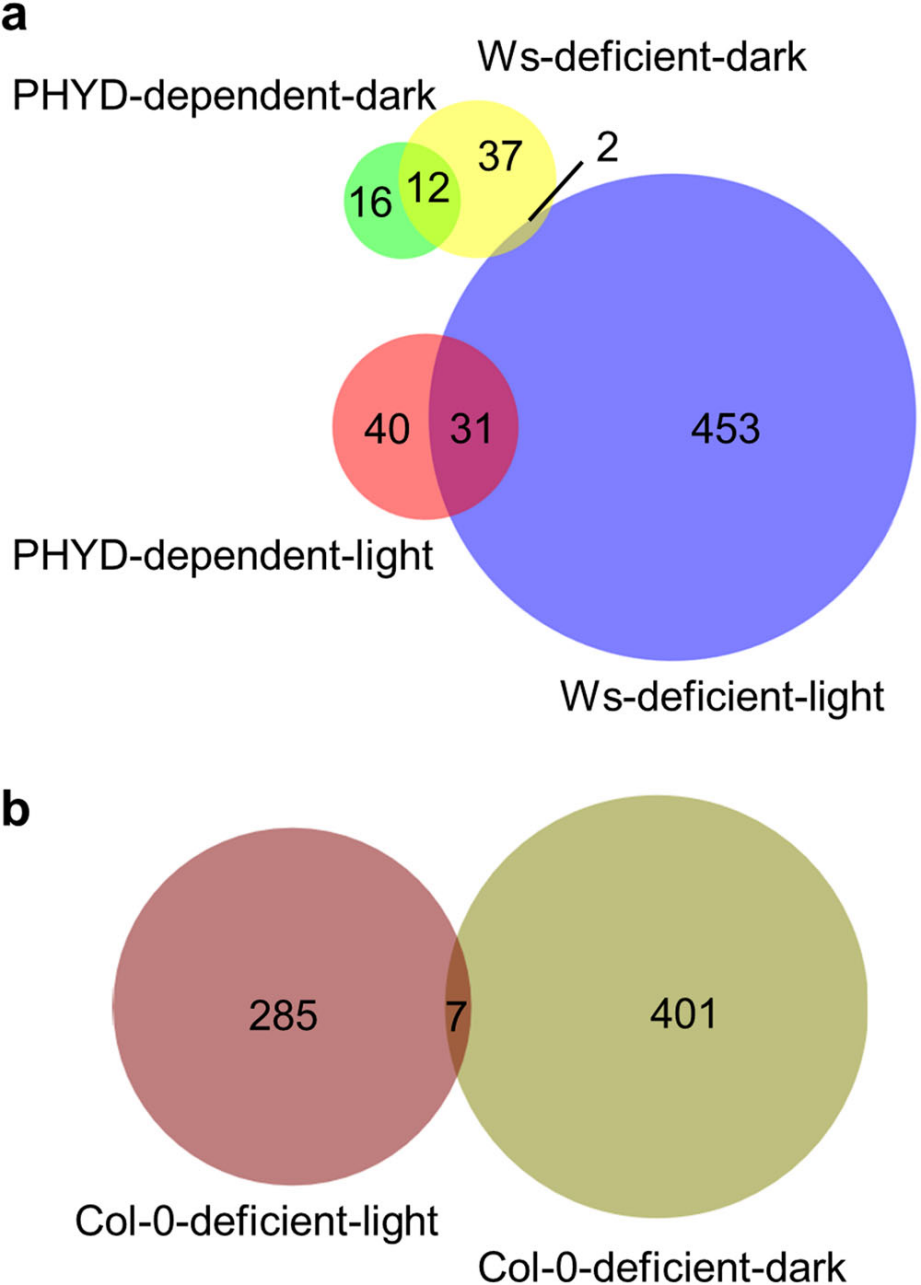
Overlap analysis for PHYD-dependent and ecotype-related spaceflight-altered genes. These genes are identified in Figs. 5a, 6a, and 7a. **a** Pair-wise overlap between PHYD-dependent and Ws-deficient spaceflight-altered genes in the light and in the dark. **b** Overlap between Col-0-deficient spaceflight-altered genes in the light and in the dark.

Between Arabidopsis leaves grown in the light and in the dark, zero PHYD-dependent space-altered genes were overlapped, indicating that PHYD regulated distinct transcriptional downstream sigaling of adaptive responses to space under light and dark environments (Fig. 8a). Similarly, there was also a small overlap for ecotype-related spaceflight-altered genes in leaves under light and dark conditions (Fig. 8b). In contrast, there were ~43% (31 out of 71 in the light and 12 out of 28 in the dark) PHYD-dependent spaceflight-altered genes were also Ws-deficient in response to spaceflight in both light and dark conditions (Fig. 8a and Supplementary Table 2). The shared space-regulated DEGs between *phyD* and Ws, which is naturally deficient in PHYD, indicated a role of PHYD in regulating spaceflight-altered genes.

## Discussion

**Fig. 9:**
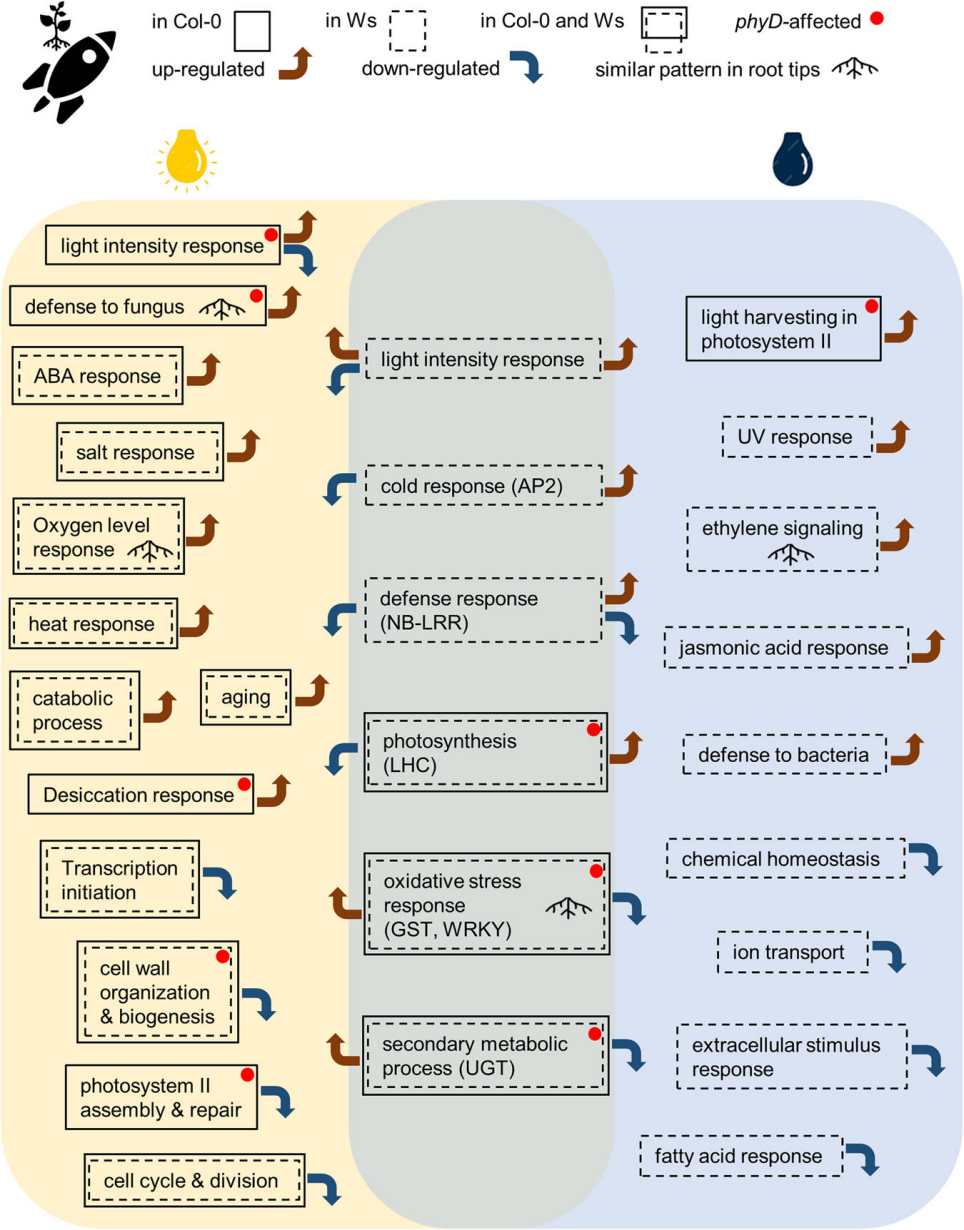
Summary of signaling pathways enriched using DEGs of Arabidopsis leaves in response to spaceflight in the light and in the dark, respectively. Up- and down-regulation of pathways, ecotypes showing the enrichment of the pathways, pathway enrichment affected by *phyD* mutation, and similar patterns observed in root tips are indicated as labeled.

Light conditions greatly influence growth, development, and environmental stress responses of plants^33–36^. While plant responses to terrestrial abiotic and biotic stresses can be strongly affected by both light intensity and quality^36^, there are also many key stress-associated genes that respond to the stress regardless of the lighting environment. The degree of induction or repression of these key stress responsive genes can vary among plants and instances, but these stress responsive genes typically show a consistent direction of response (induced or repressed) irrespective of the lighting status of the stress environment. Examples of this kind of key responsive genes are observed across multiple abiotic and biotic stress responses in terrestrial environments, such as *HY5*, *CBF* and *COR* genes in cold response^37–40^, *PIF4* and *HSP70* in heat response^41,42^, *RAP2.4* in drought and salt responses^43^, and *PR1* in response to tobacco mosaic virus^44^, etc. In contrast, there is no single set of genes showing universally consistent patterns of response to the spaceflight environment, and there is substantial variability among flight experiments, even when identical genotypes and tissues are sampled. Rather, there is a set of key categories of gene families and pathways that are commonly engaged in plant responses to the spaceflight environment. The genes comprising these families and pathways are widely observed in previous spaceflight experiments^15,45^, as well as in both the light and the dark in the CARA flight leaves (Fig. 9).

The major categories of spaceflight responsive genes that are differentially expressed in both the light and the dark in CARA represent pathways related to defense, oxidative stress response, light signaling/photosynthesis, and secondary metabolism. These categories of genes are very typical of the spaceflight response in plants and have been seen in experiments representing a wide variety of growth hardware and spaceflight environments. For example, among Arabidopsis spaceflight experiments grown on the ISS in diverse environments such as the BRIC (Biological Research in Canisters) hardware (both light- and dark-grown), the Vegetable production system (VEGGIE, directional light-grown), the Advanced Biological Research System (ABRS, directional light-grown), the European Modular Cultivation System (EMCS, controlled white, blue, or red light, or dark), and even on the ISS without plant-growth hardware (the bulkhead-grown CARA experiment presented here), these key gene categories are represented in the differentially expressed genes^8,12,15,46–56^. The connection can even be extended to completely different spaceflight platforms, which show that these key gene categories are also engaged by seedlings grown on the SJ-10 satellite (16 h light/8 h dark cycle) and the TG-2 orbital laboratory (light-grown)^57,58^. CARA with both light and dark growth conditions, therefore in many ways is typical of plant spaceflight responses.

The CARA experiment was, however, specifically designed to compare directly the impact of the lighting environment on spaceflight grown plants of three genotypes. CARA conducted parallel, concomitant light and dark growth conditions in the same flight experiment, thereby enabling side-by-side comparison of space-altered transcriptome in the light and in the dark. The data presented here suggest that variations in light conditions among spaceflight experiments have a substantial impact on the transcriptional patterns of key spaceflight-associated genes. In CARA leaves, the vast majority of genes differentially expressed in space (more than 99% of the DEGs in Col-0 and *phyD*, and more than 97% of the DEGs in Ws) have a distinct light-dependent response to spaceflight (Fig. 2b). Significant differential expression of key space-responsive gene families, such as LHC, were prominently represented in the CARA leaf transcriptomes (Fig. 4a), but the representatives from the key gene families are regulated in opposite directions with respect to the lighting environment; in most cases, if a key gene is up-regulated in the light, it is down-regulated or not significantly regulated in the dark, and vice versa (Fig. 4b). This trend is also observed for most pathway categorizations that are enriched in both the light and the dark in response to spaceflight (center column, Fig. 9); again, if the common pathway shows up-regulation in the light, it shows down-regulation in the dark (Fig. 9).

The nature of the lighting environment may also play a role in spaceflight associated gene expression. In the CARA experiment, plants were grown in the ambient, indirect lighting of the Destiny module on the ISS. The diffuse ambient lighting would provide general light signaling cues to plants, but might not serve as a powerful directional cue compared to plant growth facilities with strong and directional lighting^17^. The combination of an environment lacking a directional cue for gravity with a diminished directional cue for light may have contributed to the complexity of the leaf transcriptome in CARA compared to the leaf spaceflight transcriptomes for Ws and Col-0 plants grown in habitats with strong directional lighting^8,54–56^. The transcriptomes from dark-grown plants, which lack both gravity and light cues, are substantially different than the transcriptomes from light-grown leaves. In addition to the opposite direction of regulation in the key set of spaceflight-associated genes in light versus dark plants (Fig. 4), there were also far more genes differentially expressed in the light than in the dark (Figs. 1a, 3a), which might indicate the sensitivity of leaves to small variation in ambient light. These DEGs represented a larger number of metabolic pathways under light condition (Fig. 9). In the light, more stress-responsive signaling pathways were activated, and more pathways related to cell wall and cell cycle were repressed. In the dark, more genes involved in ethylene and jasmonic acid signaling were induced, while more ion transport and chemical homeostasis pathways were down-regulated in the absence of both light and gravity. In contrast, for root tips that can be more sensitive to environmental cues of tropic growth, more DEGs were detected in the dark with neither light nor gravity cues in spaceflight compared with the 1 *g* condition on the ground.

Phytochromes play a key role in light sensing of plants during spaceflight on the ISS^1,6,9,10,47^, and the CARA data suggest that the phytochrome gene family may play a broader role in an environment that relies on light as the primary tropic cue. In addition to modulating light-signaling responses, the *phyD* mutation influenced the space-altered modulation of defense and oxidative stress in both leaves and root tips, and desiccation response, cell wall metabolism and secondary metabolic process in leaves (Fig. 9). Thus, phytochrome D appeared to be involved in the modulatory network of stress responsive signaling and metabolic processes in response to spaceflight, in which the regulatory patterns of downstream genes are related to light conditions. The loss-of-function mutation of *PHYD* in Col-0 or the natural deficiency of *PHYD* in Ws did not severely compromise processes engaged in physiological adaptation of Arabidopsis to spaceflight. The elimination or adjustment of some of these genes may not compromise the plant health in space but can potentially reduce the metabolic cost. Given the largely altered transcription pattern in response to spaceflight (Fig. 2a), there were still considerably overlapped DEGs in Col-0, *phyD* and Ws, especially in the light (Fig. 2d). The *phyD* mutant altered the manner in which light-grown leaves responded to spaceflight, and many DEGs associated with the physiological adaptation of Col-0 to spaceflight were absent in the *phyD* plants. The lack of *PHYD* created the necessity for the plants to adjust the regulatory network required to deploy similar signaling pathways to establish physiological adaptation (Fig. 3a, pathways labeled by colored bars of light yellow, light green and dark green on the left).

The key genes that were differentially expressed in CARA are typical of the Arabidopsis spaceflight response and represent pathways identified by the mega-analyses conducted by Barker and colleagues across a variety of spaceflight environments^15^. However, the unique experimental design of CARA added another layer of perspective, by providing direct tissue-specific, genotype-specific and lighting-specific expression patterns in response to spaceflight. There was a much stronger transcriptomic response to spaceflight in leaves than in root tips as measured by DEG numbers (Fig. 1b). Meanwhile, these two tissues shared some space-altered gene categories, such as genes from GST and WRKY families (Fig. 4a), and genes enriched in pathways of defense, cell wall metabolism, and oxidative stress response^17^. However, the DEGs of these common space-altered gene categories were not always coordinately expressed in the two tissues (Fig. 3a, Fig. 9). There were many ecotype-dependent patterns of gene expression associated with spaceflight adaptation. Col-0 and Ws both engaged pathways involved in developmental regulation and abiotic stress responses, but each expressed different genes representing these categories (Fig. 6, Fig. 7, Fig. 9). And all of these tissue-specific and ecotype-related responses to spaceflight exhibit distinctly different patterns of expression between light and dark environments. (Fig. 2c, Fig. 8).

The transcriptomic profiles of the CARA biology suggest a dominant role of the light environment in the plant physiological adaptation to spaceflight. This study illustrates the distinct behaviors of genes deployed in plant responses to spaceflight under light and dark environments. The presence or absence of light greatly influences the genome-wide regulation of transcription across plant tissues and ecotypes. Different plant tissues, ecotypes or mutants with genetic deficiency in certain genes, can engage different sets of genes from a series of key categories in response to spaceflight-associated habitats. The genes from these key categories would be significantly changed by spaceflight, but their expression patterns are dependent on the light conditions. In comparison, the light-dependent influence on plant transcriptomic responses in orbital spaceflight is more overwhelming than other effects from genotypes or ecotypes. Future experimental design with respect to genetic engineering or genome editing for improved adaptation of plants to spaceflight habitats will require coordination in the selection of genetically modified targets and the establishment of optimal light conditions.

## Methods

### CARA experiment operations

The CARA (Characterizing Arabidopsis Root Attractions) experiment performed on the ISS has been documented in Paul et al. ^17^. Briefly, the experiment launched on SpaceX CRS-3 under the NASA operations nomenclature (OpNom) of “Petri Plants” and was one of the ARK1 payloads flown by the Center for Advancement of Science in Space (CASIS) (https://www.nasa.gov/mission/station/research-explorer/investigation/?#id=1020).

Three lines of *Arabidopsis thaliana* (Arabidopsis) including two wild-types, Columbia-0 (Col-0) and Wassilewskija (Ws), and a Col-0 *PHYD* mutant (*phyD*, SALK_027956C), were grown in phytagel plates on orbit or at Kennedy Space Center (KSC) within the ISS Environment Simulator (ISSES) Chamber for 11 days. Six plates were used for each genotype and 11 seedlings were planted in each plate. Seedlings were grown in the ambient, diffuse light (4–6 μmol m^–2^ s^−1^) of the ISS cabin wall, or in the dark (plates were unwrapped for 4 h to activate germination, then re-wrapped by black-out cloth, which were also affixed to the wall). The ground control in the ISSES (ISS Environmental Simulation) chamber of KSC were performed 24 h later than spaceflight samples, allowing the manipulation of lighting and atmospheric composition for the ISSES to mimic the conditions that plants were experiencing on the ISS. The 11-day old plants were harvested into RNAlater in Kennedy Space Center Fixation Tubes (KFTs) and kept for 12–18 h at ambient temperature to ensure perfusion before being transferred to the MELFI freezer for storage on orbit. The KFTs were also kept frozen in transit to Earth and KSC, and during the transportation back to UF Space Plants Lab.

### RNA extraction and sequencing

Seedlings frozen in RNAlater were thawed at 4 °C and then leaf tissues were dissected from the hypocotyl using a dissecting microscope and collected. The plants used in this study were from the same plates as those used for the collection of root tips in Paul et al. ^17^. All leaves from one plate were pooled for RNA extraction, thus one plate comprised a biological replicate. RNA extraction was conducted using RNeasy Plant Mini Kit (Qiagen, Germantown, USA). Three biological replicates were used for RNA-seq. RNA samples were multiplexed and sequenced in the NovaSeq 6000 platform with 2 × 150 bp reads at the Interdisciplinary Center for Biotechnology Research (ICBR), University of Florida, including Core of Gene Expression and Genotyping (RRID:SCR_019145) for library construction and Core of NextGen DNA sequencing (RRID:SCR_019152) for sequencing. More than 60 million paired-end reads were obtained per library.

### Transcriptome data analysis

After demultiplexing, sequencing adaptors and low-quality bases with quality phred-like score <20 were trimmed using the cutadapt program^59^. Reads that were shorter than 40 bases were excluded from RNA-seq analysis. RNA-seq analysis was using the genome of *Arabidopsis thaliana* (version TAIR10.51) from TAIR (The Arabidopsis Information Resource) as the reference. The cleaned reads of each sample were mapped to the reference sequences using the read mapper of the STAR package (Spliced Transcripts Alignment to a Reference, v2.7.9a)^60^. The resulted mapping data were processed through HTSeq (High-Throughput Sequence Analysis in Python, v0.11.2)^61^, samtools, and scripts developed in house at ICBR to remove potential PCR duplicates, filter and count uniquely mapped reads for transcriptomic analysis^62^. The counted reads for each gene were analyzed by a DESeq2-based R pipeline. Differentially expressed genes (DEGs) with statistical significance were selected using a threshold of log2(fold-change) >1 or < −1, and FDR adjusted *P* value (Padj) < 0.05. (ICBR Bioinformatics Core, RRID:SCR_019120).

Process and pathway enrichment analysis was done using Metascape (https://metascape.org/gp/index.html#/main/step1)^63^. Ontology sources used in Metascape included GO Biological Processes, KEGG Pathways and WikiPathways. Gene family enrichment was performed using GenFam (https://www.mandadilab.com/genfam/)^64^. In these enrichment tests, *P* values were calculated and adjusted using default settings. The threshold for significant enrichment was Padj < 0.05. Venn diagrams were plotted using DeepVenn (http://www.deepvenn.com/).

### Supplementary information

Supplementary Information

## Data Availability

RNA-seq data of CARA leaves samples have been deposited with the accession number of PRJNA1069768 in BioProject (https://www.ncbi.nlm.nih.gov/bioproject/) of National Center for Biotechnology Information (NCBI) and with the OSD identifier of OSD-678 (DOI: 10.26030/65t6-7p29) in NASA GeneLab (https://genelab.nasa.gov/). CARA root tips transcriptome data and phenotype photos are deposited under OSD-120 (DOI: 10.26030/0w7t-3128) in NASA GeneLab.

## Code availability

All codes used in this work have been described in methods section. No previously unpublished codes were used.
