# Supplementary material for: Light has a principal role in the Arabidopsis transcriptomic response to the spaceflight environment
Source: NPJ Microgravity. 2024 Aug 6;10:82. doi: 10.1038/s41526-024-00417-0 (PMC11303767; doi:10.1038/s41526-024-00417-0)
Supplement: Supplementary file 1 — Supplementary Information [file 41526_2024_417_MOESM1_ESM.pdf]

Light has a principal role in the Arabidopsis transcriptomic response to the spaceflight environment

Mingqi Zhou[1], Robert Ferl[1,2,*] and Anna-Lisa Paul[1,3,*]

[1]Department of Horticultural Sciences, University of Florida, 2550 Hull Road, Fifield Hall, Gainesville FL, 32611, USA

[2]UF Research, University of Florida, 1523 Union Rd, Grinter Hall, Gainesville, FL, 32611, USA

[3]Interdisciplinary Center for Biotechnology Research, University of Florida, 2033 Mowry Road, Gainesville FL, 32610, USA

*Correspondance: Anna-Lisa Paul (alp@ufl.edu), Robert J. Ferl (robferl@ufl.edu)

Supplementary Table 1. The differentially expressed genes (DEGs) between Flight and Ground Control in Col-0, *phyD* or Ws in the light or in the dark. Log2FC numbers highlighted in yellow indicate significant changes (Padj < 0.05). Red indicates upregulation (Log2FC > 1) and blue indicates downregulation (Log2FC < -1).

Gene	Gene name	FCL vs GCL	Padj	FPL vs GPL	Padj	FWL vs GWL	Padj	FCD vs GCD	Padj	FPD vs GPD	Padj	FWD vs GWD	Padj
AT1G64110	DAA1	5.151505268	1.98235E-60	5.829330551	1.47424E-18	5.096605823	8.92455E-14	-1.818658052	2.90134E-07	-1.409881947	1.2961E-13	-3.139153235	6.58835E-65
AT1G16850	AT1G16850	3.298560069	1.58053E-09	5.140407618	2.79236E-30	3.200427608	6.12898E-08	-1.547460778	0.034723544	-1.161783327	1.71159E-05	-2.314598063	1.48657E-39
AT1G22990	HIPP22	2.222927343	0.009324125	2.983532534	9.51951E-08	2.77323319	0.000858848	-2.537865118	0.000505277	-1.411897979	0.005790177	-2.227420045	7.19792E-10
AT2G05070	LHCB2.2	-1.787611488	3.92341E-05	-1.093612979	1.57055E-17	-1.130549176	3.07455E-15	4.33649333	0.000963371	1.912956212	0.010209078	1.832461721	2.9538E-31
AT1G15820	LHCB6	-2.016345879	1.62157E-07	-1.640075718	4.43911E-29	-1.820956725	2.89867E-27	2.169926284	0.033270547	1.384800295	2.5343E-11	1.955780213	1.07381E-45
AT4G10340	LHCB5	-2.198234453	2.45813E-10	-1.64285649	3.30671E-18	-1.1544272	2.39627E-40	3.013169598	0.008252774	1.112136945	0.012283914	1.922292569	3.09587E-40
AT1G03130	PSAD-2	-2.302916044	2.52607E-08	-1.482822588	2.88432E-18	-1.662103558	4.02879E-42	2.639541365	0.027983987	1.085703777	4.15313E-06	1.181691665	5.94937E-09
AT5G54270	LHCB3	-2.546064705	2.20283E-10	-2.040633501	2.69209E-19	-1.439964448	2.86847E-41	4.231639732	0.00096406	1.489957813	0.00316976	2.223896054	4.02154E-41
AT5G12940	AT5G12940	-1.874454694	2.85578E-05	-2.346400968	0.000102214	-3.327384073	5.27613E-18	-1.516826483	1.22634E-05	-1.196410022	1.32594E-06	-0.081654863	0.765533353
AT2G23110	AT2G23110	7.355088121	6.23803E-12	13.49362728	8.4598E-13	7.544287544	2.27814E-17	-1.84568538	0.026523867	-0.211792183	0.909240131	-2.680662677	5.0357E-12
AT1G73540	NUDT21	-1.35698519	0.004598105	-1.019700955	3.96083E-05	-1.233259275	0.005816702	2.219581961	0.025169391	-0.585689216	0.116463958	3.452563694	1.5501E-176
AT4G13395	RTFL12	-1.521082304	0.001135647	-1.504741217	4.17273E-08	-1.163497449	0.000210389	1.952529102	0.006700442	0.176827518	0.857077081	4.174093731	3.0237E-199
AT5G64040	PSAN	-1.577811849	0.000271676	-1.157882575	6.08341E-16	-1.131416078	4.02523E-20	2.934996287	0.022106181	0.853492068	6.72792E-05	1.02335401	2.58597E-09
AT1G06680	PSBP-1	-1.695050283	4.10705E-06	-1.181780614	5.22576E-11	-1.146048564	2.18335E-34	2.74858762	0.024208681	0.739955131	0.000258358	1.287318441	3.5375E-16
AT3G46780	PTAC16	-2.308770521	1.16059E-09	-1.53891853	7.04909E-13	-1.235332277	2.72367E-31	3.019663013	0.004787614	0.786199329	0.007773052	2.075253738	3.76403E-38
AT3G54890	LHCA1	-2.774192132	1.04045E-11	-2.275807457	1.22526E-30	-1.56825696	9.55197E-41	4.342168359	0.002130111	1.055442393	0.067161964	2.125810326	1.24409E-43
AT1G21500	AT1G21500	-3.026409942	1.07165E-20	-2.563398808	5.64049E-31	-2.333529312	1.52175E-55	2.389276512	0.024641754	0.528490786	0.401563983	2.344842822	3.00316E-16
AT5G14470	AT5G14470	2.049651667	0.000129599	2.055915986	2.4542E-15	2.162606085	2.67651E-38	3.134174289	0.023745764	0.074873938	0.958119929	0.162179816	0.578100653
AT1G60190	PUB19	4.057399366	4.54349E-24	6.441367686	1.08548E-37	2.185447598	3.5292E-24	2.99084787	0.033270547	-0.610947166	0.491449838	-0.136232228	0.903127494
AT1G21400	AT1G21400	1.698438552	5.34123E-05	1.337059611	2.89026E-14	2.409372729	1.39121E-37	2.922543511	0.041186577	0.417830934	0.115814285	0.262301024	0.164590676
AT3G45290	MLO3	2.778705829	1.03973E-09	2.611658012	6.25666E-12	2.969696501	1.78127E-13	2.405310277	0.030255469	0.278770705	0.652699183	-0.036684074	0.937495644
AT3G22961	AT3G22961	1.369301345	0.000947705	1.591834272	0.002650417	1.329908923	0.02795584	2.291078246	0.014996721	0.082985062	0.962421525	-0.50320929	0.428531423
AT4G15550	IAGLU	3.133213784	2.13134E-16	3.660746252	7.64784E-57	2.329938745	3.03203E-67	1.565809631	0.016187243	0.757262564	1.15088E-06	0.419554319	0.003676235
AT4G33467	AT4G33467	4.504232549	7.53715E-10	6.143587411	2.06153E-26	3.854728592	4.68585E-13	5.214447168	NA	3.443600865	0.041984649	-2.066963255	0.000911476
AT3G24420	AT3G24420	1.735867251	3.42385E-05	1.895170204	1.93035E-06	1.413070582	1.2805E-10	-0.913875206	0.646747644	-1.044682822	0.043651375	-2.461871767	1.34074E-25
AT3G49120	PRXCB	2.16078581	4.88891E-10	1.77517629	6.10384E-06	1.612853163	0.001812349	-0.82830764	0.66686842	-1.101574078	7.39761E-10	-1.121896558	1.0015E-18
AT1G30250	AT1G30250	5.850116423	0.007741014	5.336428803	6.5594E-34	2.960073851	5.32362E-42	0.614084887	0.834485313	-2.365523333	6.57872E-08	-1.996300446	1.11023E-14
AT3G22640	PAP85	7.591230969	4.88533E-13	5.88876556	0.000944272	6.067268821	1.58982E-66	-0.731690973	0.936758504	-3.887387521	0.031058829	-1.737814111	3.84879E-10
AT1G29910	CAB3	-3.61741886	1.39302E-22	-2.948212093	1.49246E-27	-2.918074365	5.2285E-224	3.615493562	0.303656308	1.443133469	2.76019E-06	2.272652457	0.001058683
AT2G24600	AT2G24600	-2.403224014	3.62613E-09	-1.2233659	0.013663852	-2.296003476	1.27939E-11	-0.629590079	0.75214745	-1.614761443	0.004660737	4.01849124	6.00604E-81
AT4G17490	ERF6	-1.461884271	0.001940439	-1.918093389	1.6715E-12	-1.565131155	9.67289E-16	1.923170662	0.180725221	-1.116480159	0.010877765	3.046403573	3.1486E-132
AT4G24570	DIC2	-1.633749517	0.000214932	-2.086963855	8.668E-16	-1.656902819	1.36035E-08	1.40878866	0.273353269	-1.510861264	6.92642E-06	3.031631023	1.45819E-89
AT5G45340	CYP707A3	-1.531743393	0.002733045	-2.518043569	1.35596E-17	-1.092108712	9.97876E-05	0.322044074	0.893088744	-2.044243919	1.16966E-16	2.978256519	1.14981E-69
AT4G08040	ACS11	-2.233598163	0.000117842	-3.164586787	7.78776E-06	-1.910477945	0.046618459	1.464300012	0.633717484	-1.830516471	0.013634164	2.119718597	1.23021E-09
AT1G19770	PUP14	-1.518929656	0.000248093	-1.34321615	2.79011E-17	-1.418465585	7.96114E-13	0.981742764	0.56497814	-1.006241817	2.12856E-09	1.57775267	1.12104E-39
AT4G38770	PRP4	-2.37168533	8.00514E-11	-1.41590485	2.77179E-09	-2.700137255	1.14524E-43	-0.376499591	0.835442106	-1.699849975	2.89146E-28	-1.042296772	6.46411E-12
AT1G29660	AT1G29660	-2.880827799	1.75412E-14	-2.184126078	2.47569E-16	-2.630767373	5.51776E-18	-1.207011633	0.313609174	-1.232229771	2.18469E-06	-1.453954132	7.89839E-21
AT1G68740	PHO1;H1	-1.650573204	0.019703195	-1.913720133	0.000277386	-1.896240534	0.037519782	-1.367201989	0.640704059	-1.092903582	0.006064026	-2.040451067	5.95773E-28
AT1G07180	NDA1	-2.309966752	0.000144821	-1.452620795	2.74105E-10	-1.514878919	5.3622E-09	3.689324386	NA	7.695955311	0.017433604	-0.938854079	0.560408869
AT3G26690	NUDX13	2.286905111	1.52486E-10	2.475930155	3.51125E-15	2.526840397	9.66196E-40	0.801344302	0.294468892	1.237547095	1.15193E-13	0.376825013	0.066075117
AT3G55500	EXPA16	1.272031388	0.026388677	1.053603601	0.010210889	1.021731021	2.583E-07	1.466213963	0.547026175	1.081891164	0.030515906	-0.014189218	0.979780542
AT4G15700	AT4G15700	2.327806248	1.16832E-08	2.01204072	1.05727E-06	1.172784334	0.006785702	0.984882979	0.598901706	1.078262264	0.000825337	0.630837937	0.027912683
AT5G63180	AT5G63180	-1.46338674	0.009979158	-1.352826257	0.048326588	-1.279248718	0.000152964	-0.819572069	0.66924865	-1.022075242	1.94054E-05	0.198239567	0.194826078
AT4G12730	FLA2	-1.53150793	2.81185E-06	-1.965316757	5.13264E-07	-1.427054632	4.28322E-13	-1.079620152	0.337914365	-1.028368783	9.67221E-08	-0.113344231	0.639751545
AT4G18197	PUP7	-1.749315131	2.38143E-05	-1.166383712	9.39809E-05	-1.426861399	0.000624031	0.045292589	0.976635648	-1.030858576	0.036145051	0.681537705	0.000962907
AT5G49630	AAP6	-1.341928317	0.006386128	-1.396155152	0.007473859	-1.308801203	0.000579298	-0.421546297	0.867000021	-1.122804248	0.000403932	0.226521016	0.296945056
AT1G76240	AT1G76240	-2.783928645	1.26585E-06	-2.582276678	2.89747E-08	-2.69307823	1.83716E-11	-0.097110417	0.966082349	-1.139439147	0.001554924	0.319733245	0.350774644
AT4G28780	AT4G28780	-2.176190578	8.87677E-09	-1.817494479	0.009688891	-1.448934734	0.000995869	0.025966998	0.993804788	-1.200822723	0.002887237	-0.845924578	0.000349111
AT1G12110	NRT1.1	-1.34363424	7.57829E-05	-1.625810457	7.99295E-05	-1.750359091	2.20523E-07	-1.117956022	0.518021029	-1.272423856	2.00423E-16	-0.931205499	1.30004E-08
AT3G15356	AT3G15356	-2.918663998	5.84254E-17	-2.16415127	6.68204E-27	-3.394377967	3.45028E-06	-0.237230729	0.929506087	-1.324828386	1.50088E-09	-0.092184658	0.682499849
AT4G22690	CYP706A1	-1.460649922	0.000457768	-1.159567052	0.000272836	-1.623644236	1.18147E-22	0.399490705	0.727213714	-1.400334915	0.000120228	-0.028612468	0.906389603
AT5G01190	LAC10	-5.06184351	0.00220755	-4.805863912	0.016853948	-7.03532979	8.74643E-05	-1.892143027	0.677952867	-2.43953436	0.003378266	-0.222607134	0.669113881
AT1G61340	FBS1	1.413494225	0.003073638	1.568908374	2.45767E-06	1.055663438	0.000125978	1.106049179	0.647947756	-0.498402285	0.543580441	2.204173236	7.70687E-14
AT5G03210	DIP2	4.517500194	1.50752E-21	5.6354224	2.17139E-19	3.107109173	1.22076E-05	1.37728471	0.55451616	0.410905957	0.589336718	1.769445438	2.84053E-05
AT2G15880	AT2G15880	1.744431822	3.63273E-05	1.486710451	3.01195E-33	2.021310175	0.000104476	2.682939493	0.072226197	0.503348416	0.128437935	1.386154328	3.80582E-20
AT1G15330	AT1G15330	1.302549046	0.005635341	1.544682642	5.61376E-07	2.015177527	5.49086E-17	1.931441972	0.210785317	0.773234519	0.185411782	1.169010902	8.61744E-07
AT5G47610	AT5G47610	1.833841807	0.000706764	2.861965383	2.49695E-17	1.134556702	0.000761279	2.259130743	0.183329115	0.247844792	0.796178186	1.095281866	0.027640458
AT1G77120	ADH1	3.36770297	1.68111E-19	4.225401343	3.43322E-26	2.006206983	2.66046E-07	-0.284973615	0.758662788	-0.543835454	0.387327222	1.002022497	5.85743E-07
AT1G49450	AT1G49450	2.434239429	0.000260285	3.370285975	4.06189E-11	1.768257209	0.000272121	-1.112951717	0.62738272	-0.483277855	0.786582627	-1.005016721	0.044249486
AT5G47810	PFK2	1.387903878	0.000875843	1.18911618	6.47045E-05	1.803809211	1.87528E-11	0.276157461	0.773629186	0.440890796	0.178708196	-1.012652808	3.62164E-08
AT5G44790	RAN1	1.998861662	2.70682E-17	1.301498032	1.54559E-14	1.318546155	9.6976E-17	-0.441034177	0.670950811	-0.283825418	0.258926293	-1.024582512	7.27858E-20
AT5G03240	UBQ3	3.092549857	2.31198E-39	2.991952369	1.14517E-61	2.504069574	2.13528E-60	-0.257084914	0.878717171	-0.075207182	0.849024218	-1.027604108	3.15396E-22
AT5G19875	AT5G19875	2.823193842	1.16226E-08	2.982216525	8.03203E-12	1.923285639	0.002264669	-0.411420648	0.685495817	-0.348880219	0.540774196	-1.034226068	1.76783E-06
AT1G69252	AT1G69252	2.746150803	2.29902E-19	2.074142478	2.23237E-22	1.62399324	2.26156E-25	0.153400393	0.914259529	0.241768216	0.371229642	-1.040413185	1.65786E-09
AT1G23040	AT1G23040	1.759214063	0.0005625	3.394957267	1.31722E-08	1.428962155	0.01265809	-1.233705034	0.65378996	-0.592403816	0.12916102	-1.041764241	3.67939E-08
AT3G03170	AT3G03170	2.993759784	5.22799E-12	3.651773777	2.07651E-13	1.802679588	0.00035897	-0.33260668	0.695103554	-0.719613234	0.093774581	-1.048804679	0.000635077
AT3G57770	AT3G57770	1.356642957	0.004209343	1.340404956	0.01871142	1.123300358	0.000290514	1.469828794	0.598901706	-0.057363931	0.98143759	-1.048918424	0.024777224
AT3G16990	AT3G16990	2.039061465	2.01134E-05	2.994440439	1.29927E-17	2.326588712	1.64465E-09	0.608476995	0.707112033	0.046769567	0.970305421	-1.051378009	0.006535087
AT1G52342	AT1G52342	1.919857848	0.000102339	2.07457329	9.08423E-11	1.594109647	7.00207E-08	0.279003853	0.854215164	0.749238861	0.072389536	-1.062080198	0.002834553
AT1G79270	ECT8	2.013624692	4.71782E-06	3.601000322	2.88689E-43	1.685196071	1.31954E-16	0.7924019	0.664635521	-0.100094866	0.870402458	-1.066420216	2.07861E-10
AT1G11175	AT1G11175	2.673417828	9.12327E-11	2.177040062	3.44981E-10	1.310898777	1.13603E-11	-0.503985211	0.586228813	0.091991377	0.875046829	-1.082007631	8.89732E-17
AT4G13530	AT4G13530	2.187072404	4.45664E-08	1.801110734	5.44047E-16	1.265593883	1.8476E-16	0.722372804	0.65378996	0.035798556	0.958308469	-1.088517144	0.026622522
AT1G54575	AT1G54575	5.142840813	5.74268E-23	5.060335349	4.74502E-63	4.83657246	4.46079E-16	0.960634493	0.616300683	-0.478042653	0.485182841	-1.102744609	5.63815E-06
AT3G10450	SCPL7	2.551091437	2.15375E-11	3.897654521	6.13083E-18	3.076318543	1.35497E-26	-0.988317272	0.00042072	-0.260681755	0.548823276	-1.106264235	1.2503E-12
AT2G31945	AT2G31945	2.053808757	0.000414711	2.558410849	5.91572E-08	1.70692791	1.24315E-06	0.552261924	0.797614335	0.103951543	0.951111828	-1.108314261	0.00058914
AT3G01520	AT3G01520	1.700863862	1.72845E-08	1.437493467	2.92388E-19	1.199853563	5.39548E-18	0.0899247	0.956802484	-0.207881184	0.427220454	-1.114012963	7.36402E-17
AT1G50290	AT1G50290	1.955738728	1.52425E-05	1.494236197	9.56788E-06	1.244554234	2.71758E-29	-1.908605805	0.385221483	0.113250825	0.934226173	-1.120963149	2.76127E-16
AT1G07985	AT1G07985	4.938926857	6.97625E-30	7.280760432	8.03858E-37	3.8209288	2.9118E-10	0.118886947	0.967195835	-0.006270431	0.99575645	-1.128462805	0.001270727
AT1G58030	CAT2	1.849807861	3.47219E-14	1.485564629	9.32224E-24	2.086475572	4.79315E-53	0.026291871	0.985904881	-0.123387398	0.578542261	-1.137358907	4.00565E-13
AT1G10140	AT1G10140	2.491957303	6.38373E-13	2.312066407	6.01593E-16	2.211090984	1.277E-49	0.084698084	0.956852966	-0.034286635	0.954643598	-1.168494512	1.721E-07
AT4G23050	AT4G23050	1.920345796	2.27745E-11	2.21981584	1.53384E-11	1.703094001	3.30255E-13	-0.126445804	0.943633329	-0.300245946	0.383688353	-1.168967433	5.11288E-12
AT5G66052	AT5G66052	2.581653069	0.023391877	1.238278841	0.012659998	1.375405296	1.97529E-16	-0.370345652	0.771422703	0.015591862	0.989242512	-1.171106836	2.36871E-14
AT2G35060	KUP11	1.296117568	2.23669E-05	2.083617898	3.56596E-25	2.043626356	2.48204E-48	0.165969521	0.941415027	-0.469929673	0.04070249	-1.209083921	2.85588E-13
AT4G16190	AT4G16190	3.108219973	5.81303E-27	3.273620746	9.17E-50	1.814964888	4.66256E-31	0.441776436	0.77590923	-0.191217657	0.493875814	-1.210428953	1.05282E-28
AT5G49690	AT5G49690	4.389136414	6.60034E-18	4.603837574	3.76645E-27	4.170413774	2.50067E-14	0.361263398	0.797390149	0.144801715	0.857349753	-1.220745909	1.47579E-05
AT3G57540	AT3G57540	3.605498559	1.35994E-08	3.787491964	2.72832E-19	1.98065765	1.05706E-06	-0.252608341	0.939540455	-1.023827807	0.233184169	-1.220896395	0.01864954
AT1G58190	RLP9	2.739371999	5.45122E-08	2.921873528	4.08295E-14	3.41699872	1.85711E-34	1.817027195	0.281913411	0.049566941	0.962120014	-1.221772084	2.16788E-05
AT4G02280	SUS3	3.733801612	3.93087E-35	5.250305659	1.53716E-57	4.431456066	1.40817E-13	-0.809946239	0.170461508	-0.36162542	0.25776244	-1.225834137	1.34627E-11

AT3G20660	4-10月	1.627336935	7.03112E-06	2.118311735	5.27674E-08	1.783544447	5.0776E-09	-0.498040803	0.653026084	-0.24860579	0.550184567	-1.235740791	7.2347E-08
AT3G18280	AT3G18280	1.860615405	0.00021848	1.821243659	3.2215E-11	2.464435661	6.80469E-20	-1.143883105	0.541068634	-0.63112612	0.006941051	-1.259321246	1.59355E-21
AT2G15480	UGT73B5	2.30568941	2.73056E-06	2.718033748	6.88287E-19	2.824172119	4.34949E-06	0.105293327	0.973864665	0.574069954	0.219096394	-1.264711207	1.37863E-05
AT4G11910	AT4G11910	4.508494314	0.000559884	3.946762531	1.58954E-10	3.777425523	4.24324E-22	-0.365916905	0.876585375	-0.026226399	0.988021272	-1.266666419	0.003124545
AT2G37870	AT2G37870	2.343276809	0.02369181	5.562913482	5.16959E-13	4.568795525	3.06095E-11	-1.453237818	0.579644733	-0.939203445	0.038816205	-1.267561921	1.93244E-07
AT3G26170	CYP71B19	2.658886813	2.87835E-05	2.541644901	4.39808E-11	1.988398929	5.95966E-08	0.608144464	0.63192307	0.051996935	0.957672256	-1.273585012	2.50438E-08
AT2G46690	AT2G46690	1.7699522	1.15439E-06	1.717890508	2.84868E-09	1.531601505	7.06737E-09	0.731855463	0.540789146	0.056200613	0.924627441	-1.275903846	8.66461E-10
AT1G05340	AT1G05340	2.86456546	1.90896E-15	3.647300701	1.22046E-25	3.092048417	5.94681E-08	0.702595737	0.695103554	0.116102243	0.859192131	-1.278953754	7.41836E-11
AT1G62510	AT1G62510	1.408846069	0.03619454	1.63497071	0.000587721	1.721769107	1.5537E-28	0.873065843	0.681315477	0.065537868	0.963419708	-1.280689702	3.52553E-11
AT1G52855	AT1G52855	3.681021762	0.007350149	6.143193261	3.95247E-07	4.474313167	2.96763E-23	-0.286910873	0.909287719	-0.73957857	0.381649427	-1.285531761	0.000507997
AT2G29490	GSTU1	3.683165322	2.74508E-21	3.726179228	1.08671E-16	3.416946526	1.59766E-52	-0.36377367	0.72243276	0.450538125	0.08501642	-1.304942194	3.53554E-19
AT4G16000	AT4G16000	1.10916942	0.007232153	1.260296938	0.000401303	1.416025705	2.20761E-12	1.803102943	0.419746227	0.115215559	0.913269747	-1.325506723	6.16917E-08
AT2G15490	UGT73B4	3.342313713	4.6564E-19	3.502040703	1.83565E-13	3.590948814	4.9101E-12	0.009609124	0.997591659	0.058496455	0.950071203	-1.329297343	4.11253E-14
AT4G31877	ath-MIR156c	3.743102072	2.32282E-08	2.132849706	0.013486258	1.303699027	0.001883836	-0.962180373	0.279591669	-0.35495223	0.607258954	-1.341261594	2.77594E-09
AT5G58320	NET4A	1.581230166	0.041086655	1.779585392	0.00287853	1.088574775	0.001074969	-0.606454998	0.632534636	-0.657571314	0.0570932	-1.341562289	1.64574E-06
AT5G24530	DMR6	1.65272531	7.91052E-05	1.87863135	4.81943E-13	2.802017068	1.4835E-155	1.696566207	0.380380211	0.024030398	0.976992907	-1.34539837	5.35272E-16
AT1G15100	RHA2A	2.358403489	2.21654E-10	1.626818449	0.00050281	1.483877252	2.98637E-05	-0.587949671	0.577728436	-0.364124743	0.331809276	-1.362842874	6.51296E-13
AT4G05100	MYB74	4.624262351	9.9434E-32	6.234855092	1.6338E-10	4.129530137	1.97669E-22	-0.151078216	0.972495118	-0.632865843	0.572051703	-1.363618451	0.001823084
AT4G03320	Tic20-IV	1.762096632	0.013817071	1.422615148	0.031706878	1.709722966	5.63383E-07	-0.285110446	0.830658776	-0.477095677	0.318380163	-1.367952863	1.88564E-11
AT3G57010	AT3G57010	4.213750521	6.56137E-14	4.446724468	4.31798E-10	3.168089873	1.76097E-06	-1.235005888	0.611321113	-0.223777441	0.563104532	-1.368302862	2.01238E-19
AT3G61900	AT3G61900	2.212694921	0.018287678	2.765603084	1.00199E-05	1.959080937	0.014146234	0.211667682	0.953380212	-0.075446581	0.97926944	-1.374467867	0.017362617
AT5G05110	AT5G05110	2.399415354	1.07956E-14	1.944406891	5.57329E-14	1.028503458	4.53752E-05	-0.124159535	0.911509594	0.048520836	0.929239543	-1.380254618	1.98494E-18
AT2G37170	PIP2B	2.383094484	5.78769E-13	2.481456019	9.89047E-37	2.029266204	3.26979E-25	0.026912246	0.98791139	-0.83754113	6.04017E-17	-1.391692083	7.73073E-17
AT5G07010	ST2A	1.682275213	4.66594E-05	1.385433768	3.12347E-07	1.992334159	3.4084E-07	-0.770252377	0.521765323	-0.460949776	0.506912308	-1.393466818	1.19921E-36
AT3G03470	CYP89A9	2.55039478	4.01986E-19	3.408994636	6.13601E-28	3.985680128	1.8193E-17	-0.048558668	0.966082349	-0.427287402	0.119988448	-1.397024278	8.93532E-17
AT2G24260	LRL1	2.163724286	1.77361E-08	1.925790793	1.48538E-07	1.404547571	9.23193E-18	-1.006637952	0.327175594	0.100531257	0.83609719	-1.403623993	1.4843E-23
AT3G51440	AT3G51440	2.182719515	0.032083109	1.611583455	0.043653803	2.506670866	0.000212835	-0.20947863	0.936758504	0.206750638	0.854653576	-1.448347559	3.66194E-07
AT5G39520	AT5G39520	9.292976326	4.81188E-05	8.315829812	1.24158E-12	7.47927479	9.95375E-07	0.874500744	0.759702709	-0.274868547	0.739243805	-1.450059953	8.96161E-11
AT4G17550	G3Pp4	2.393236555	2.05872E-09	2.574522967	1.15057E-10	1.598603572	1.01075E-06	-0.294802711	0.697104641	-0.600489675	0.091556107	-1.452861696	9.85245E-08
AT1G67920	AT1G67920	3.428808819	2.91058E-06	4.212733859	2.09263E-13	2.611286253	0.000408535	0.351076834	0.890241742	0.058822981	0.975858601	-1.461716886	0.006864777
AT1G79520	AT1G79520	2.416891174	3.69107E-07	3.669160092	1.7067E-36	2.506298461	3.01059E-15	0.510307351	0.721649216	-0.359180735	0.596924946	-1.46443786	1.94111E-06
AT3G01970	WRKY45	3.742460923	1.25871E-13	4.652452124	2.32199E-15	2.530471853	1.01203E-14	0.537869775	0.685495817	0.704895583	0.034089739	-1.464471611	1.24334E-14
AT5G14180	MPL1	3.700542424	1.0744E-16	4.223628279	8.84569E-15	4.098817128	2.07348E-36	-1.412411525	0.082687285	-0.494382003	0.128956485	-1.46546821	1.26243E-28
AT2G40390	AT2G40390	2.746436328	0.000137906	1.689796997	0.012752036	1.687031327	2.26786E-05	0.61260542	0.66808962	-0.095031594	0.950400259	-1.468324726	6.37092E-05
AT2G46680	HB-7	4.067063802	5.19354E-21	5.36487664	4.96316E-65	3.40371659	7.23079E-13	0.048472576	0.985080874	-0.088956862	0.896222696	-1.468915932	0.005251601
AT1G17020	SRG1	7.92844661	1.81052E-10	4.579289478	5.43037E-05	4.13568667	3.42041E-09	-0.835582793	0.721140128	-0.142233452	0.866906715	-1.476143673	6.80422E-14
AT2G21560	AT2G21560	2.836010509	3.19902E-22	2.013254975	2.91052E-07	1.693788859	9.23824E-10	-0.399111648	0.715199899	-0.298139661	0.491449838	-1.483550195	9.25024E-18
AT2G18050	HIS1-3	3.810348029	3.91648E-26	3.826962671	4.62164E-62	2.453550177	9.98916E-27	1.027143602	0.589531904	-0.107605564	0.916110833	-1.491765548	0.001041877
AT1G31820	PUT1	3.17182283	3.01111E-16	3.878220274	4.87338E-24	2.58740219	2.5224E-08	0.544911176	0.652047062	0.164543995	0.866906715	-1.496753632	2.12918E-06
AT2G47180	GolS1	2.172632293	3.17541E-14	3.640215949	6.47051E-24	2.235440894	4.6615E-22	-0.174372931	0.896927975	-0.261516005	0.534073839	-1.499173151	3.54547E-27
AT5G50720	HVA22E	2.028511571	2.81359E-05	1.593451833	0.000177621	1.309037087	0.003200994	0.606794677	0.698266436	0.284520708	0.659063827	-1.501665902	2.21805E-06
AT2G41280	M10	5.150858676	6.89793E-08	5.815767999	9.26122E-29	4.942676386	2.06165E-23	0.421493096	0.855427008	0.786097927	0.58081406	-1.511567756	0.015027844
AT3G28007	SWEET4	3.875147092	4.6564E-19	4.81377017	9.75187E-23	3.517661486	8.0076E-11	1.148859096	0.637815859	-0.045396553	0.986818647	-1.533249605	9.27691E-06
AT3G48740	SWEET11	1.395086069	1.99584E-05	1.022017603	5.55102E-08	1.447992817	3.51591E-31	0.626317229	0.704470077	0.136604771	0.619494542	-1.559508812	6.98524E-24
AT2G47270	UPB1	2.637355833	4.73771E-06	3.933606191	1.45476E-21	2.789836415	2.72475E-14	0.167793605	0.911720308	0.389779067	0.535760946	-1.564170392	1.24577E-06
AT2G33380	RD20	3.328618693	2.18936E-08	4.66742577	2.73814E-08	3.758161734	0.000575069	0.489077282	0.876015705	-0.193577476	0.824058587	-1.56927384	3.34706E-07
AT1G76590	AT1G76590	1.873565016	4.38496E-05	2.146156723	1.60715E-15	2.08349115	3.89265E-22	0.594332753	0.779496598	-0.426428084	0.23980685	-1.573092542	8.17536E-10
AT2G47260	WRKY23	2.237748191	2.59551E-05	1.458485027	0.032246814	2.404510197	3.2745E-07	-0.719643898	0.600370118	0.283280888	0.652699183	-1.589001649	1.03638E-19
AT1G02460	AT1G02460	3.902054982	0.004990733	2.5936564	0.000646024	3.752203078	0.001037487	2.16401401	0.652047062	-1.200159757	0.717454016	-1.595343627	0.04365382
AT3G05640	AT3G05640	3.754103224	2.87916E-25	4.66206632	1.54018E-56	4.062203303	2.19457E-18	-0.158953795	0.936758504	-0.680683717	0.030439069	-1.615325778	1.05494E-43
AT4G23450	AIRP1	1.887167516	0.00113	2.734823919	3.43606E-05	1.642174108	5.66616E-05	0.140572428	0.951485287	0.169445325	0.909436033	-1.62449252	0.000162597
AT2G37770	ChlAKR	2.281883378	0.017873246	6.210771177	1.6442E-06	3.910859315	0.002345235	-0.64398138	0.918286956	2.213710267	0.116914646	-1.637520877	0.022339549
AT3G12580	HSP70	3.095842003	3.57108E-12	4.201955228	2.146E-12	2.947171105	7.02542E-22	-0.575457353	0.540789146	-0.444463293	0.073570501	-1.655124558	2.89143E-20
AT1G49900	AT1G49900	3.284713362	0.022184885	4.964631796	5.75176E-06	3.512448284	7.24005E-05	-0.086679003	0.982183273	-0.181367259	0.927507051	-1.656977131	0.021112832
AT1G62570	FMO GS-OX4	4.554301467	5.51377E-36	6.22360125	1.52844E-83	1.699281409	0.000329683	0.648446286	0.809632507	-0.967852133	0.032546089	-1.657100923	1.25661E-13
AT5G56100	AT5G56100	2.296021562	5.59835E-09	2.735052287	5.54384E-18	1.606924744	5.09959E-15	1.594990282	0.366891789	0.42894993	0.295775985	-1.67170055	0.005159891
AT4G30460	AT4G30460	4.171715341	7.94863E-16	4.441129404	4.45896E-16	1.703529708	0.000469761	-1.6056208	0.253109653	0.017334347	0.990170591	-1.671778931	0.000965832
AT3G61430	PIP1A	1.23148625	0.000500059	1.555528602	8.5671E-20	1.185790185	3.59298E-16	0.265933749	0.871661081	-0.712885031	1.80215E-05	-1.682007202	1.4129E-27
AT1G78290	SNRK2-8	2.370674196	1.43901E-10	1.252793893	0.002200936	1.323336446	7.66583E-12	-0.300488723	0.823318742	0.114489504	0.896222696	-1.686116379	3.26134E-14
AT1G65970	TPX2	10.36200205	1.88258E-11	6.118568655	4.74453E-07	5.910553115	6.2635E-296	-0.282056705	0.901814827	0.670983521	0.712668629	-1.709999655	8.30012E-56
AT2G45570	CYP76C2	1.993388164	0.001782423	5.991371701	3.16977E-24	4.542518206	6.68099E-05	-1.670683285	0.635731984	0.938859854	0.563104532	-1.716418544	0.001315798
AT3G60140	DIN2	5.608874339	3.56732E-25	5.081658874	7.07692E-07	6.141424771	8.41957E-15	0.036670324	0.992231761	-0.549532694	0.647824775	-1.726755542	2.06991E-19
AT1G60740	AT1G60740	10.32302739	2.23324E-11	5.425555888	2.75355E-07	6.477145546	1.42774E-61	-1.11320328	0.340875713	-0.731615627	0.612379475	-1.736236499	7.99515E-14
AT5G13370	AT5G13370	2.424066433	4.17259E-11	3.743600848	2.51173E-33	3.496875126	3.16584E-48	-0.030302257	0.991559366	0.09645363	0.851437944	-1.740966166	2.72087E-43
AT1G46768	RAP2.1	1.783082951	2.80312E-09	2.198665904	3.28023E-05	1.279393412	0.00014148	0.097415236	0.976635648	-0.133652274	0.865593881	-1.754101346	2.42062E-10
AT1G67810	SUFE2	3.732175583	7.23242E-12	1.589099302	0.000786833	2.157268818	1.25201E-06	-1.819902895	0.380505605	0.121005201	0.91048199	-1.782495602	4.47311E-37
AT2G45210	SAUR36	1.120835751	0.018162296	1.172298899	0.00226133	2.333413916	4.9072E-13	0.071130009	0.972495118	-0.048321301	0.957093674	-1.79275165	1.89931E-25
AT3G17790	PAP17	1.876314916	9.86447E-07	2.548771104	3.63444E-20	1.164332684	0.012860509	-0.072817154	0.964175862	-0.163195631	0.854600248	-1.810320798	7.03447E-29
AT2G47485	AT2G47485	1.952422801	1.65305E-06	1.707696907	6.05018E-05	1.128533955	2.48338E-05	0.088581522	0.961358666	-0.689375808	0.187294793	-1.821721937	1.2439E-06
AT1G80160	GLYI7	5.368248837	4.61477E-22	6.898908179	4.63241E-17	7.399443122	9.69883E-22	0.920640573	0.663291724	0.579188687	0.37256248	-1.831903722	4.95372E-11
AT5G04080	AT5G04080	2.584586805	2.45773E-15	2.476895819	2.89942E-21	1.910813628	1.96717E-23	0.097501306	0.958184471	-0.117273768	0.822128113	-1.84139346	1.17918E-35
AT1G20160	ATSBT5.2	2.599629079	2.39987E-20	2.575960174	6.10656E-13	1.637357473	8.74182E-13	-0.958200979	0.452963543	-0.350401052	0.445236462	-1.843397979	3.50773E-44
AT3G05937	AT3G05937	2.094271923	1.22415E-07	2.972862673	8.99752E-15	1.223008801	0.007410562	-0.619682843	0.689484332	0.216790539	0.761744816	-1.871146769	8.53802E-10
AT2G38800	AT2G38800	2.140177805	1.38347E-20	1.470279491	1.15721E-10	1.089714679	1.54469E-16	-0.239293384	0.852062083	-0.07183699	0.883116962	-1.901774716	6.49928E-86
AT4G15610	AT4G15610	5.088462859	1.98198E-66	3.323575815	1.23551E-38	3.361949738	7.49203E-52	0.32798324	0.861051144	-0.334018762	0.327045125	-1.92984327	7.04402E-17
AT5G44005	AT5G44005	1.322717157	0.014890666	1.703866685	0.000243935	1.089494493	0.000127678	0.932779252	0.704225272	-0.692115087	0.548231503	-1.94454532	2.76895E-08
AT2G21820	AT2G21820	4.459066798	0.018919786	6.14902445	2.35351E-31	4.443707795	2.27892E-08	0.220144879	0.951061044	0.276213496	0.89611977	-2.008411013	0.000606202
AT1G48000	MYB112	3.333355353	0.024309989	6.664850164	0.009643067	6.850709873	4.99877E-11	1.234417819	0.62738272	-0.175721957	0.958669515	-2.03083448	0.003470932
AT1G03790	SOM	4.256635448	0.003073638	6.224854525	3.60302E-09	4.788936966	2.22748E-25	-0.793827647	0.481130123	-0.55849718	0.047903155	-2.036168578	2.25397E-09
AT1G29640	AT1G29640	3.936318925	0.002484598	5.283679701	3.7477E-05	3.3120544	7.69978E-08	-0.955401407	0.532231419	-0.785218353	0.642169321	-2.043918672	5.8936E-06
AT2G17500	AT2G17500	2.559629222	8.23837E-07	1.673104314	6.36898E-05	1.639180193	2.58331E-05	-1.066686185	0.606948021	-0.077098143	0.869626899	-2.048600989	6.71201E-32
AT4G37220	AT4G37220	2.820315526	1.0222E-14	2.258238773	5.52925E-11	2.198794679	2.00368E-64	-0.067934021	0.985484378	-0.390245102	0.35525666	-2.101296972	0.000578253
AT1G07430	HAI2	5.008506971	0.023370912	7.185594342	1.24056E-52	3.342816312	8.08908E-10	-0.022593033	0.996799452	-0.20727714	0.918766875	-2.105856998	0.000219469
AT1G73260	KTI1	5.016792504	4.91391E-23	2.545877931	5.77899E-11	1.30070166	3.7934E-07	-0.646837493	0.765067714	-0.383390029	0.663111026	-2.139234586	1.49873E-71
AT2G38400	AGT3	1.553664786	4.33344E-06	1.439123518	1.44257E-06	1.966065793	5.07122E-25	0.126257452	0.968467209	-0.543314425	0.069289533	-2.143159877	3.43076E-27
AT3G44300	NIT2	4.821473868	1.74027E-55	6.190406191	9.65744E-12	5.386258716	4.23603E-11	-1.646979468	0.066666426	0.052095517	0.953576013	-2.20129733	1.25373E-47
AT4G01060	CPL3	2.416206337	0.000482781	2.294628977	8.45585E-06	2.076173777	1.24356E-06	-0.735516518	0.66808962	-0.331858582	0.773861036	-2.237477754	2.06902E-05
AT3G48510	AT3G48510	4.270715819	1.75137E-10	4.842193833	0.006283953	3.490253488	4.34704E-09	-0.404941399	0.785453357	-0.440665141	0.703592556	-2.285811643	7.01081E-09
AT2G35070	AT2G35070	3.749925214	2.63684E-12	6.207281524	1.71798E-64	5.616924542	3.83899E-09	0.991919496	0.693791019	0.000953588	0.99989203	-2.295566806	4.02151E-12
AT2G20880	ERF53	4.190244203	9.46282E-24	5.09953959	0.001220203	4.683712828	0.000186643	2.964460237	0.06223917	0.373523064	0.885377618	-2.304390911	0.02061099
AT5G06760	LEA4-5	4.407768449	1.48564E-34	10.27431538	3.18987E-32	5.565230922	5.64932E-07	-0.299062326	0.812482986	0.192967539	0.896222696	-2.321738823	5.85353E-11
AT5G63350	AT5G63350	6.718536839	0.000131663	7.725581159	1.34214E-29	4.222341915	0.0001182	0.269215102	0.944280349	0.389970889	0.715410636	-2.45675381	2.27446E-10
AT3G48020	AT3G48020	2.909047579	0.040156664	2.519837973	0.007463379	2.186957248	0.000473749	-2.211971954	0.586991747	-0.705266686	0.091095588	-2.48480356	1.18899E-27
AT3G03341	AT3G03341	4.408118831	0.037648524	7.620273367	1.179E-07	4.468953842	0.000623429	-0.595351697	0.878985819	-1.406553334	0.516634069	-2.486306611	0.000473825

AT3G59930	AT3G59930	7.163425612	0.000105526	9.340682175	1.05716E-12	5.699011641	0.048728435	-0.36546466	0.913641552	0.147337493	0.857737893	-2.554928637	5.71517E-09
AT2G26355	AT2G26355	2.917310197	4.89524E-22	3.072390617	3.46775E-17	2.524010586	0.000117481	-0.389697224	0.633049961	0.188998908	0.63650345	-2.590146897	2.39595E-30
AT5G26146	AT5G26146	5.157299277	1.0346E-05	2.929576727	0.001330972	2.605514056	0.003596103	0.414492439	0.782300491	0.211071893	0.848343766	-2.621125481	0.00520383
AT3G50980	XERO1	9.74265308	4.20835E-11	12.45488117	1.09215E-19	5.413441926	2.27909E-09	-2.298226355	0.616300683	0.645625947	0.759165288	-2.663526729	1.71514E-09
AT2G47770	TSPO	6.632628277	0.000903251	8.481483861	2.12839E-18	5.356111057	2.32633E-05	-0.471763826	0.823318742	-0.539590864	0.521243345	-2.705879675	2.03092E-15
AT5G43840	HSFA6A	3.859133332	9.82807E-16	5.953133295	2.65534E-61	3.092726809	2.72846E-15	1.594156394	0.56571725	0.061531691	0.973216634	-2.794108718	0.00037986
AT5G52300	LTI65	5.666407342	2.8494E-36	9.536142948	1.89979E-13	4.741513764	2.45644E-08	0.586665557	0.826486125	-1.015748343	0.485857955	-2.826770557	0.000247898
AT2G35300	LEA18	12.80251665	3.5957E-21	8.365798732	5.60255E-07	5.704411298	1.21132E-06	-0.139125876	0.980939287	0.825403686	0.58081406	-3.060791033	1.0199E-09
AT3G21720	ICL	5.798250881	1.94445E-77	7.496989153	1.50821E-08	7.281070458	5.77842E-36	0.503243696	0.875558325	0.735466087	0.59780496	-3.067919873	2.22698E-64
AT3G27250	AT3G27250	2.0675468	0.005342706	3.596785747	5.08515E-20	2.171123004	9.73293E-06	0.803819223	0.718141493	-1.244638214	0.087042206	-3.105043142	3.73409E-23
AT2G19900	NADP-ME1	2.85022439	0.0005515	6.424603671	0.000381801	5.859729751	2.26731E-05	-1.822142621	0.420586313	0.030872105	0.986818647	-3.206590723	1.47143E-09
AT2G36640	ECP63	4.705928602	4.45349E-05	12.42754535	8.86305E-20	5.624269219	1.19382E-13	1.29812958	0.887763386	1.639802678	0.869002246	-3.243355554	0.001643808
AT1G17810	BETA-TIP	4.209197684	0.042110744	4.43752089	0.000198981	5.372848075	1.12909E-12	-1.276894666	0.793480908	0.036238604	0.98877807	-3.283934936	0.04459263
AT5G66400	RAB18	5.58987078	1.35033E-38	8.901681001	1.03937E-37	5.64082967	3.70223E-09	0.837278052	0.732322487	-0.56798877	0.774140454	-3.508288837	3.79178E-05
AT5G62490	HVA22B	8.546003325	9.43265E-05	6.896884097	4.09142E-08	8.132436226	6.60721E-08	-6.804943611	0.319520931	-6.23917037	0.1688939	-3.657299697	0.000193413
AT3G03620	AT3G03620	9.17416029	3.77217E-06	11.18501852	2.91941E-12	7.314708338	1.15181E-05	-3.06068154	0.395098242	1.271607161	0.686941393	-3.899019363	1.41743E-06
AT4G36600	AT4G36600	5.374289882	0.000361474	6.785888549	6.8002E-09	5.455561413	9.55634E-05	3.809943406	NA	0.04917823	0.996678199	-4.387765497	0.005614452
AT3G02480	AT3G02480	4.403125151	1.40343E-11	7.951544032	6.30355E-09	6.728472639	6.35587E-09	-1.675762925	0.414437927	-1.134420775	0.45858949	-4.620970568	3.66439E-67
AT1G52690	LEA7	7.331331835	1.61186E-10	11.83520446	4.85066E-12	7.29184191	9.6643E-10	-0.592272692	0.873245463	-0.897335362	0.624504205	-4.664207015	3.6738E-24
AT2G42560	AT2G42560	3.264680281	0.026549449	9.255159136	0.000277451	8.385430022	0.002070486	-5.492537671	0.156029601	5.342861444	0.515629348	-5.443384336	1.3477E-08
AT3G51810	EM1	4.734642057	0.010944069	5.010969633	8.8316E-05	8.118551526	1.22076E-05	-3.088240695	0.718141493	0.083503068	0.988021272	-6.393736664	0.022468911
AT2G41260	M17	6.297868927	5.63054E-06	12.32185967	1.92841E-09	10.71360894	1.20478E-13	-1.123202869	0.667958595	-0.493112209	0.896410026	-8.859052463	8.65736E-09
AT1G32450	NRT1.5	1.366852347	0.001260511	-1.065658839	0.000508619	-4.825050206	1.01997E-52	-1.540065928	0.565728752	0.030526443	0.986774932	-1.607700199	8.51944E-36
AT4G28410	AT4G28410	9.788681831	4.38404E-08	-3.162312011	0.049652665	-6.707376964	0.049532338	-1.28029812	0.747011433	-0.664410109	0.356290239	-2.750733389	7.42709E-20
AT5G38100	AT5G38100	8.448194018	0.000250527	-6.693356933	0.043223091	-7.749606376	0.000106604	-2.577509933	0.494722635	-1.828705862	0.090340494	-3.400536285	7.09814E-13
AT4G37400	CYP81F3	7.268426653	0.0257923	-7.269831361	0.019347637	-6.612090354	0.018776126	-1.37039433	0.721140128	-0.861507259	0.256781773	-1.481662821	2.99149E-09
AT4G13235	EDA21	8.040456792	0.001114989	-8.097882279	0.000265866	-6.639823269	0.013891131	-1.337209029	0.731041294	-0.151641733	0.905535933	-2.075822145	1.09669E-07
AT1G33760	AT1G33760	-4.05813988	2.54264E-18	-3.349295263	3.44074E-06	-2.811515537	0.003192292	2.040544704	0.501211524	0.865256492	0.91692451	6.486252457	4.12762E-20
AT2G32200	AT2G32200	-2.7065294	0.000605413	-1.777613752	0.001918882	-2.9897851	0.011071504	0.050414383	0.988164996	-0.725725328	0.857963506	4.655173591	5.1207E-19
AT4G27654	AT4G27654	-1.587212705	0.012579269	-2.008207909	4.32542E-05	-1.072877068	0.037281014	0.986203756	0.718141493	2.418738972	0.125608293	4.522460984	5.06707E-50
AT3G46490	AT3G46490	-2.931244887	2.24966E-05	-2.688376167	0.000259525	-3.067167749	1.01938E-12	0.854579724	0.622850451	-0.177763485	0.897002087	4.173625755	1.10354E-20
AT1G76650	CML38	-1.146791742	0.038514716	-1.212158269	9.32756E-07	-1.009107742	0.000255032	2.310890891	0.062555197	-0.394822002	0.625323462	3.823889521	7.18609E-63
AT5G19240	AT5G19240	-2.734743497	5.7242E-08	-2.079922863	5.08469E-17	-3.47020946	2.07842E-05	1.059345354	0.663046749	-1.212706412	0.05483456	3.547978861	9.79634E-37
AT3G20600	NDR1	-2.084931906	5.26906E-06	-1.10007841	0.000275713	-1.43019011	3.84189E-06	1.429314605	0.575462403	-0.88694038	0.288680005	3.108562496	1.67593E-56
AT2G35930	PUB23	-2.86269728	7.71502E-07	-3.336503612	3.74706E-20	-1.731999709	0.049445013	0.501950438	0.802533514	-1.342990023	0.134739551	3.033282296	1.89198E-51
AT1G72910	AT1G72910	-2.214679478	0.001519673	-2.448785434	1.59541E-06	-2.659526047	7.29859E-18	0.841042341	0.858510044	-1.387651683	0.823358742	2.988604753	6.00371E-19
AT2G35710	PGSIP7	-2.073343209	0.00011289	-2.198460535	3.07336E-06	-2.320392696	6.96459E-09	-0.426100675	0.711255369	-0.734640101	0.186913581	2.52939068	1.53242E-47
AT5G48490	AT5G48490	-2.667509422	2.14682E-06	-2.097813995	7.75291E-24	-2.61133084	1.48076E-19	3.042609306	0.275929155	0.312239073	0.905907141	2.433150906	1.92941E-09
AT3G27690	LHCB2.3	-4.600310394	2.68837E-23	-3.386346066	5.19696E-77	-2.18954859	3.39004E-29	3.197291386	0.092759749	-0.228709783	0.802638226	2.326048538	7.08966E-26
AT5G47500	PME5	-2.529097666	1.10588E-06	-2.555721061	9.30712E-39	-1.55721786	7.4065E-13	0.763346023	0.547026175	0.813725165	0.022744601	2.206297855	2.50639E-22
AT2G34620	AT2G34620	-2.906456798	2.97447E-10	-1.837436698	2.53833E-22	-1.845681101	9.66594E-19	2.330457004	0.326457456	0.378261082	0.827181504	2.189190783	9.94084E-20
AT1G19380	AT1G19380	-1.129404766	0.019816185	-1.371666619	1.82778E-07	-1.375559527	4.48501E-05	0.801893025	0.618005886	-0.633388602	0.048017645	2.172238889	3.49632E-17
AT2G31880	SOBIR1	-1.622683092	9.1353E-05	-1.004746078	2.11397E-06	-1.338499816	1.33754E-06	0.747288946	0.637815859	-0.831730243	0.006327601	2.149901833	4.02154E-41
AT5G07690	MYB29	-3.676963788	0.008054364	-1.799254815	0.043812972	-7.97386396	5.36957E-05	-3.874701946	0.690158649	2.035040022	0.798169865	2.144826456	0.003564742
AT2G05940	RIPK	-1.620266559	1.25307E-05	-1.407361896	0.000418812	-1.166346615	0.032676842	-0.284763739	0.721579398	-0.288076017	0.596685937	2.142943264	1.28414E-37
AT5G66650	AT5G66650	-1.550557023	0.00052166	-2.191169283	8.15825E-19	-1.354420825	7.92798E-09	0.617054768	0.647947756	-0.792545728	0.012283914	2.100464448	4.2306E-23
AT2G47750	GH3.9	-2.765533257	0.026011732	-1.447167347	0.001200298	-2.589514409	4.89066E-06	-3.681626024	0.587300556	1.633154976	0.33608583	2.043871711	0.002837176
AT5G06320	NHL3	-1.354830907	0.000664303	-1.025076695	0.000102522	-1.446829641	4.72834E-08	0.19712413	0.930598641	-0.935685351	0.009414101	2.013994924	3.81227E-88
AT1G67470	AT1G67470	-1.815067966	0.002016579	-1.169938463	0.045061119	-1.68238756	6.18727E-05	-0.278166844	0.808237728	-0.824718049	0.104629999	1.950171828	6.50618E-24
AT4G01250	WRKY22	-1.410574036	0.00613367	-1.068664058	6.67763E-05	-1.140852098	7.55178E-05	0.854180543	0.653026084	-0.43039664	0.354547118	1.867842854	1.33878E-20
AT3G05370	RLP31	-2.743839029	2.61344E-07	-2.339788729	2.61195E-11	-1.889165787	2.24739E-10	0.187168587	0.932561267	-0.031252492	0.98143759	1.863979038	6.19488E-09
AT2G26530	AR781	-1.347632193	0.002224808	-1.222350806	4.6815E-08	-1.671797801	9.17302E-06	0.815565398	0.705076831	-0.88038377	0.070974858	1.84684932	1.01715E-24
AT2G17780	MCA2	-2.590467362	1.0232E-09	-2.932024301	1.19775E-08	-1.715537146	4.63447E-06	0.364641498	0.795048376	0.300976821	0.823666413	1.83241557	4.61095E-08
AT4G36280	CRH1	-4.399519847	3.01633E-08	-2.963953402	1.49224E-05	-9.329860154	9.66854E-12	-0.917794765	0.865681813	1.523963807	0.221106445	1.781216071	1.63312E-06
AT2G41340	RPB5D	-1.720173742	0.019816185	-2.084252494	0.02562143	-2.003603154	1.32221E-05	1.277011244	0.07535494	0.754927473	0.350174136	1.739124386	0.009349756
AT5G46690	bHLH071	-2.50339192	0.000373956	-3.273330243	0.000178924	-2.860205363	1.11853E-06	0.693221616	0.664635521	0.477830733	0.388772141	1.674989183	9.68364E-21
AT4G39710	PnsL4	-2.06776252	1.10699E-08	-2.091819585	2.56646E-07	-1.833592946	4.10689E-19	1.174366479	0.087323788	0.568906931	0.15990855	1.63837693	4.65118E-15
AT2G39470	PnsL1	-3.005299114	1.01869E-18	-2.426300278	3.75353E-13	-1.515527304	2.30787E-25	0.954343835	0.589474071	0.175996829	0.810176655	1.632471796	3.58057E-21
AT4G21280	PSBQA	-2.518681186	7.21432E-20	-1.701548853	7.67676E-15	-1.702307298	2.14179E-49	1.360216666	0.196045896	0.560432797	0.391124836	1.628341072	5.71405E-57
AT2G01180	PAP1	-2.2027639	2.45306E-12	-1.833066416	1.88142E-07	-1.544042627	8.67306E-06	-0.28121036	0.75100724	-0.894281326	0.031111468	1.623024564	8.38465E-21
AT1G09750	AT1G09750	-1.327566769	1.92356E-05	-1.727973486	8.27108E-17	-1.01181207	1.67049E-15	-0.287829218	0.796538797	-0.50438282	0.003571563	1.51476989	8.20126E-05
AT1G53520	FAP3	-2.566553029	7.90971E-14	-2.393446055	4.11287E-06	-2.173278593	6.61768E-28	-0.117550884	0.963956318	0.489985181	0.193727895	1.508745348	4.88693E-17
AT1G30380	PSAK	-2.039100496	2.29703E-08	-1.840695834	1.41247E-24	-1.672938167	2.42952E-30	1.822441752	0.21014474	0.903132982	2.29823E-05	1.508547259	2.94391E-14
AT1G52230	PSAH2	-3.149480257	1.58868E-21	-2.102748016	1.1634E-59	-2.451485319	4.46079E-36	0.884480276	0.48633216	0.670853751	0.035699	1.48650023	3.1948E-19
AT5G27290	AT5G27290	-3.520571732	6.60034E-18	-2.188607067	0.000178924	-2.306049698	1.8621E-45	2.63929979	0.16556514	0.939082089	0.287120998	1.479948649	0.000131636
AT3G52450	PUB22	-2.249731063	2.61344E-07	-2.397980563	9.94038E-06	-1.769956132	1.57481E-06	0.458786019	0.753910599	-0.913827542	0.013770802	1.477475844	4.95229E-21
AT5G46710	AT5G46710	-1.515320545	0.001370199	-1.344302513	0.001816747	-1.370486096	1.50707E-06	0.230314858	0.839330979	-0.707798009	0.21409659	1.468251268	5.05343E-23
AT4G12800	PSAL	-2.285452792	2.51538E-11	-1.512244344	2.07238E-19	-1.266243606	1.30577E-37	1.978461531	0.086492111	0.752592416	0.242445371	1.464321018	4.87509E-28
AT4G39970	AT4G39970	-2.907096239	3.45933E-20	-2.323638571	1.35596E-17	-1.860116388	4.04781E-39	0.550690828	0.626508471	-0.061663775	0.958308469	1.446920658	1.05612E-15
AT1G52220	AT1G52220	-1.54371488	9.41156E-07	-1.395258142	1.68613E-14	-1.409367302	1.25508E-38	1.44755262	0.127697587	0.868815857	0.013509288	1.439412457	3.05029E-28
AT3G63140	CSP41A	-3.700434517	3.54893E-42	-2.888915716	4.27437E-27	-3.098910459	1.074E-135	1.21980606	0.255573307	0.165167005	0.810706099	1.435424938	1.11458E-12
AT5G38700	AT5G38700	-4.616361711	0.011706788	-10.71017839	1.27201E-13	-3.588248239	0.033147804	-2.896018251	0.563204569	-3.330463143	0.067570199	1.425361816	0.019537201
AT4G09010	TL29	-2.486408528	7.36758E-22	-1.776995577	3.4459E-20	-1.815981679	4.44241E-54	0.85927571	0.350541127	0.542376748	0.032962938	1.41429641	1.16582E-30
AT1G66940	AT1G66940	-2.515605992	0.000131916	-2.926257575	9.44485E-05	-3.681847032	1.5047E-70	-0.151138347	0.966973374	-0.217704474	0.70029725	1.41166225	5.08918E-15
AT2G02500	ISPD	-1.759771742	1.79582E-05	-2.189466619	2.59227E-06	-1.830065552	5.26078E-28	0.161897384	0.950313494	0.357003546	0.481806699	1.404989749	3.56229E-13
AT3G55840	AT3G55840	-1.395958057	0.024035348	-1.291049733	0.014512452	-1.666528319	5.46716E-06	-0.565385639	0.827683763	-0.605824474	0.421679589	1.403117096	4.02561E-13
AT3G23480	AT3G23480	-1.906709319	2.77473E-05	-1.242902999	0.022707849	-1.987848448	4.0585E-08	0.417775306	0.736609833	0.932312392	0.084118875	1.382611718	9.3236E-13
AT3G21055	PSBTN	-2.311284574	6.93763E-09	-1.452999668	1.49154E-18	-1.437899508	3.22309E-44	1.848955794	0.099695852	0.447146152	0.088665114	1.382608648	2.73097E-14
AT1G17650	GLYR2	-2.42759977	1.57075E-13	-1.783842812	7.27346E-09	-1.941823509	6.34027E-19	0.641896301	0.644839236	0.007098977	0.993373477	1.37260739	6.20162E-11
AT5G38990	AT5G38990	-1.634852569	0.000115839	-1.155626951	0.012907604	-1.366207579	3.10919E-05	0.829422249	0.626508471	0.121560375	0.934574943	1.369353334	2.48072E-07
AT4G02770	PSAD-1	-2.060969716	1.46342E-08	-1.426173792	6.06703E-19	-1.542544048	3.17124E-56	2.266868281	0.05013994	0.692889423	0.001398585	1.357479313	1.92018E-16
AT5G19500	AT5G19500	-1.907588661	6.52823E-06	-2.112389111	3.62347E-05	-2.019890206	6.23813E-05	1.498339672	0.565728752	0.644254129	0.511188955	1.354581278	0.001746477
AT5G47910	RBOHD	-1.538588958	0.000195467	-1.444437562	1.21927E-05	-1.418703526	1.39911E-08	-0.932192169	0.031910209	-0.950596122	0.000345208	1.315014048	1.319E-22
AT2G43560	AT2G43560	-2.482207599	2.02751E-14	-1.874598482	2.89508E-23	-1.5908498	6.48998E-29	1.019944272	0.343358673	0.530884833	0.090746558	1.302408121	1.92798E-10
AT3G26060	PRXQ	-1.894380954	5.98562E-23	-1.336084299	0.000289738	-1.289133872	3.98665E-28	0.54347217	0.602119156	0.406901816	0.178708196	1.291974666	6.38026E-11
AT3G16250	PnsB3	-5.040389952	3.28952E-22	-3.556164825	4.10167E-13	-3.160548432	5.19209E-42	-0.133673376	0.973896678	-0.160444694	0.935882353	1.291130235	3.57684E-05
AT3G25920	RPL15	-1.814774974	9.11093E-23	-1.171346496	5.83359E-05	-1.489100171	3.48013E-40	0.594707955	0.373396563	0.496231785	0.011075397	1.286050142	2.03582E-28
AT1G08380	PSAO	-2.017204463	3.79688E-09	-1.622306858	2.87273E-26	-1.809851694	1.72323E-60	2.15103578	0.109029762	0.795003166	0.000123753	1.28591899	3.54732E-23
AT5G56850	AT5G56850	-2.937778569	6.14157E-09	-1.584525857	0.000493303	-2.253210054	1.20339E-16	-0.46312807	0.799598167	0.461024999	0.479421473	1.273238159	5.69685E-07
AT1G57990	PUP18	-1.814713753	1.68723E-05	-1.91864204	3.65194E-15	-1.216845228	1.47252E-05	0.938096851	0.632534636	-0.865745852	3.91866E-05	1.267100689	7.70244E-22
AT1G20340	DRT112	-1.623959679	2.4425E-05	-1.167320518	1.13253E-13	-1.653780147	8.16632E-39	1.759095427	0.159498309	0.607841091	0.293352221	1.257523335	1.63212E-14
AT3G18890	Tic62	-3.435479894	1.04081E-38	-2.215613037	2.87716E-12	-2.298365092	5.19872E-29	0.43829904	0.598901706	0.253367898	0.566484832	1.252816889	2.77401E-10
AT1G78995	AT1G78995	-1.551554295	0.021662378	-1.650389168	0.01622941	-1.346713682	0.003029431	2.407801482	0.776796299	-0.376113502	0.936554664	1.250622659	0.006583041
AT2G42220	AT2G42220	-1.982107352	2.20221E-09	-1.714958811	4.39661E-20	-1.254938647	1.02662E-22	1.904771047	0.147119358	0.455596697	0.135547064	1.249528605	2.33647E-24

AT3G29030	EXPA5	-2.067968637	0.001672164	-1.581664811	0.01987913	-3.73925964	0.000196317	-0.382439172	0.916159273	-0.990119049	0.091710539	1.240786238	0.000146406
AT1G33600	AT1G33600	-1.541000061	0.000679178	-1.028128055	1.75812E-11	-1.107596339	4.65584E-07	0.743494427	0.66524984	-0.825532944	0.00150107	1.239812516	1.14448E-11
AT1G54820	AT1G54820	-3.651726859	3.80582E-19	-3.829643854	2.19191E-38	-3.072333197	2.77848E-30	1.035614386	0.598901706	-1.138021751	0.342414779	1.239585911	1.8384E-05
AT3G61870	AT3G61870	-2.276373742	3.01195E-15	-1.530573807	3.51715E-07	-1.678185135	1.36218E-23	0.734010948	0.639875152	-0.001802513	0.998594549	1.234731534	3.41983E-16
AT3G01440	PnsL3	-2.696744402	0.000148179	-4.346870086	8.17469E-05	-3.692643659	1.06844E-24	-1.118639897	0.808705576	0.468581668	0.803280676	1.229591203	0.027241123
AT2G32640	AT2G32640	-2.083456587	0.000121931	-1.591511919	1.01449E-05	-1.403488325	0.001038105	0.625190689	0.718553996	0.483922756	0.488929906	1.228655457	3.46379E-05
AT5G64850	AT5G64850	-2.619948689	4.0758E-08	-3.698437962	3.52775E-27	-1.905039521	3.52412E-08	-0.114139268	0.949945368	-0.157513705	0.889117268	1.225177474	6.1155E-06
AT3G62030	ROC4	-3.431852831	9.19449E-21	-2.294952104	0.00733223	-3.384857647	5.3367E-150	-0.172844275	0.932705464	0.187959658	0.532591859	1.21095654	5.83165E-28
AT5G01040	LAC8	-2.030685438	3.34582E-06	-1.9935751	0.001350857	-1.236536739	2.78612E-05	-0.663793374	0.602119156	-0.459465484	0.352772432	1.201712788	9.53666E-07
AT3G28340	GATL10	-1.504548033	0.001935551	-1.778739956	0.00011003	-1.834180647	1.09807E-12	-0.419263817	0.653363031	-0.738697747	0.002250063	1.198281155	5.53171E-17
AT2G30695	AT2G30695	-1.521023218	7.48765E-06	-1.542609783	1.88882E-05	-1.174977569	2.55403E-08	0.356685777	0.704225272	0.271378542	0.537208356	1.196998821	6.08786E-16
AT1G72940	AT1G72940	-1.740805902	0.000970302	-1.526018355	1.58358E-06	-1.357296737	9.71567E-09	0.396850817	0.890241742	-0.933525554	0.244409692	1.188644466	1.39207E-09
AT4G28750	PSAE-1	-2.226572117	2.24608E-18	-1.270836363	4.49471E-15	-1.238185979	9.90735E-43	2.547803882	0.092759749	0.717688395	0.101256397	1.162258927	4.25571E-12
AT4G33260	CDC20.2	-2.192539272	0.000566857	-1.87383621	0.000257035	-1.255304374	0.01085602	-0.002235585	0.999583915	0.465723738	0.167528994	1.155094552	6.39308E-12
AT5G06290	2-Cys Prx B	-1.875849162	1.20453E-17	-1.495960346	1.01848E-07	-1.548879628	1.0237E-30	0.65763718	0.495190613	0.209316764	0.474596951	1.13190701	4.81138E-15
AT5G16400	TRXF2	-2.374270473	2.08967E-12	-2.518021608	3.28481E-26	-2.545414261	7.39982E-55	0.709297374	0.637815859	0.307727828	0.525334184	1.122408693	7.01086E-09
AT4G26520	FBA7	-1.809348663	7.16922E-09	-1.335939392	7.22275E-07	-1.449323175	2.57039E-14	0.330520574	0.782971455	0.560515018	0.397816908	1.118553446	5.54187E-09
AT1G18060	AT1G18060	-3.010111328	1.4623E-15	-2.235320809	2.09688E-19	-2.113983313	1.72623E-22	0.875924887	0.591481547	0.412398694	0.42662725	1.116716085	6.88804E-08
AT4G04570	CRK40	-2.531537079	6.94902E-07	-1.677820433	0.000466021	-2.570941174	6.30806E-23	-0.521595748	0.702246294	-0.079202514	0.941042784	1.11558092	0.000212697
AT2G01760	RR14	-1.771253257	1.64891E-05	-2.114503184	0.000192797	-1.436349031	4.51589E-05	0.796853973	0.626700633	0.499265229	0.487040982	1.11427528	2.18502E-06
AT1G20020	FNR2	-3.279191208	1.61613E-27	-2.672797606	1.01619E-24	-1.902537506	9.5486E-84	1.378579625	0.271829457	0.516507457	0.07135089	1.108171924	1.44389E-06
AT1G54780	TLP18.3	-2.522615846	1.43068E-17	-1.715866846	1.23511E-26	-1.930756832	3.87635E-46	0.728226418	0.611085146	0.147123903	0.786685207	1.093548757	6.40916E-11
AT3G02730	TRXF1	-2.721981324	7.96751E-13	-2.051299264	1.45716E-26	-2.643660568	4.35432E-44	1.072945605	0.393926565	0.386531651	0.59228116	1.087071357	7.18953E-08
AT5G66530	AT5G66530	-1.96949006	7.72215E-16	-1.745622541	3.70058E-06	-1.905610211	1.13108E-12	0.655665334	0.393926565	0.191381989	0.721359573	1.083668914	7.57005E-11
AT1G32200	ATS1	-1.787959588	1.77904E-07	-1.254042943	3.74839E-08	-1.038552125	8.22059E-15	0.589595354	0.474958926	0.1243033	0.820217829	1.077416465	8.016E-12
AT1G78490	CYP708A3	-1.590015778	0.011598476	-1.765430778	0.000784079	-2.313613433	2.34048E-05	0.152691236	0.966694285	-0.231537021	0.555959885	1.077166462	2.37183E-08
AT5G35735	AT5G35735	-1.481455299	0.000305421	-1.581380166	2.11795E-10	-1.318461567	0.019190397	0.370676539	0.847546649	-0.634320955	0.037424693	1.076253313	7.04169E-12
AT3G59080	AT3G59080	-1.368792968	0.00280363	-1.317612616	4.91511E-05	-1.190662799	0.00065902	0.315497587	0.847660958	-0.553833675	0.329484853	1.069397779	4.05192E-11
AT2G41090	AT2G41090	-1.978400493	4.84971E-10	-1.36481865	0.009875637	-3.056134625	4.4827E-30	-0.642806376	0.685495817	0.149020871	0.755176072	1.067084449	2.24529E-11
AT1G35680	RPL21C	-1.853059532	1.13875E-21	-1.30133392	2.79158E-08	-1.308699343	5.83281E-27	0.445179989	0.602119156	0.467042578	0.017433604	1.065093679	1.02847E-09
AT5G18660	PCB2	-3.1277047	7.78772E-12	-2.275488081	5.53571E-06	-3.002110407	2.95258E-51	0.332324095	0.783775657	0.445160027	0.378776441	1.058465921	6.82103E-10
AT2G33450	PRPL28	-2.076728902	6.51954E-10	-1.154196053	2.2655E-05	-1.45692472	4.61662E-28	0.17023508	0.897050311	0.067225413	0.938703599	1.056468478	1.40319E-11
AT5G45680	FKBP13	-1.816607889	1.01274E-08	-1.070252962	6.75459E-06	-1.185864673	5.13003E-09	1.541458691	0.078050132	0.405277031	0.476338504	1.054696158	1.4052E-09
AT4G13500	AT4G13500	-1.67515801	0.000207811	-1.51175819	0.000976262	-1.025121307	0.000290169	0.102455696	0.970302657	0.188748859	0.773789213	1.053719242	0.000251297
AT1G66430	AT1G66430	-1.545055579	0.000167839	-1.669737408	0.001712857	-1.748815697	9.22041E-17	0.339956006	0.731041294	0.12858242	0.804786811	1.051265375	1.35786E-20
AT1G74070	AT1G74070	-1.804532499	0.000261353	-1.258645195	8.70156E-07	-1.42244325	1.59102E-13	1.253503272	0.204145338	0.488582765	0.487040982	1.046998227	8.73373E-08
AT5G42240	scpl42	-1.113836788	0.003921537	-1.317231151	5.89352E-06	-2.108095162	2.02136E-17	0.419507966	0.646729152	0.164940577	0.683203027	1.046429005	1.41599E-12
AT1G60600	ABC4	-1.625157232	0.002001238	-1.394575041	0.001142892	-1.305189718	6.92249E-08	0.051196235	0.980301604	0.296458839	0.575454666	1.045841379	2.16788E-05
AT1G05020	AT1G05020	-1.892978697	0.000701735	-1.380367684	0.021232191	-1.040665481	0.01968547	1.42730592	0.461515802	0.739877567	0.256694103	1.045013372	0.010201319
AT1G51805	AT1G51805	-2.055009739	0.000437687	-2.043878928	2.01467E-12	-2.386043096	1.6963E-25	0.337329593	0.799369545	-0.383033977	0.630466974	1.044120353	6.37092E-05
AT3G48730	GSA2	-2.52365381	5.0192E-13	-1.991410912	0.004448109	-2.261492107	1.10379E-37	0.081281812	0.978281551	0.282630436	0.315471019	1.042566913	9.27231E-12
AT1G32990	PRPL11	-2.346405264	6.76753E-34	-1.528540385	9.13409E-08	-2.067722376	7.00181E-46	0.13642463	0.923573229	0.195324714	0.543411985	1.040689451	4.81564E-10
AT3G55800	SBPASE	-2.438603972	4.84803E-31	-1.903409318	2.88809E-10	-1.553016525	4.66103E-53	0.979060078	0.313609174	0.398778011	0.093661005	1.025925308	5.37302E-12
AT5G27390	AT5G27390	-1.731086294	7.96635E-06	-1.432770422	1.10341E-09	-1.246685385	9.57802E-13	-0.236507557	0.886793538	0.158425666	0.740181504	1.0239383	1.20351E-09
AT4G01150	AT4G01150	-2.018883489	1.58972E-14	-1.463819019	6.02643E-12	-1.700439248	2.35453E-90	1.407049527	0.105968094	0.347106525	0.206674418	1.018519167	5.31284E-15
AT2G24090	PRPL35	-1.687268878	9.82807E-16	-1.081355125	4.11866E-05	-1.411470896	1.99255E-27	0.564656588	0.502960535	0.300600707	0.404931229	1.012756992	9.77021E-14
AT1G69830	AMY3	-1.613316668	2.25964E-08	-1.503536769	3.69469E-08	-1.214042164	1.44466E-08	0.909627534	0.433558429	0.119430648	0.824058587	1.009337446	4.62951E-13
AT5G23910	AT5G23910	-1.98225982	0.02173335	-2.106904117	1.26882E-05	-2.189241945	2.83618E-05	-0.092087017	0.978041029	0.034156839	0.97926944	1.008071685	9.24215E-08
AT2G37660	AT2G37660	-1.94260257	4.2907E-18	-1.567916829	3.92302E-11	-2.097863693	2.36085E-56	0.266731093	0.712045709	0.341357991	0.122203706	1.007305442	1.39896E-24
AT3G10060	AT3G10060	-3.164216134	1.83507E-24	-1.900653837	8.00746E-17	-2.236905507	2.6346E-26	0.923512515	0.318251123	-0.047403402	0.97746335	1.003963422	0.000109596
AT1G64150	AT1G64150	-2.08682647	6.82644E-08	-1.755037677	0.000104454	-1.083957049	1.60314E-13	-0.59370269	0.62738272	0.95875567	0.087156448	1.001408863	4.5944E-12
AT5G62630	HIPL2	-1.049380187	0.017532089	-1.87571402	0.014887247	-3.001540259	1.91185E-15	-1.124372556	0.326457456	-0.526598985	0.168222434	-1.11006657	4.09336E-08
AT4G11211	AT4G11211	-1.126726054	0.024749491	-2.599691888	2.0128E-10	-1.209919281	0.001643737	0.17288011	0.939540455	0.250479835	0.513456138	-1.130043037	2.30342E-10
AT3G20370	AT3G20370	-2.666777483	2.8192E-13	-2.971602927	1.27301E-19	-3.255204125	1.09734E-27	-0.936885193	0.409180759	-0.147247704	0.764985315	-1.142177289	3.31679E-17
AT3G06370	NHX4	-2.286220584	0.001148035	-2.104612449	3.62623E-05	-3.782297756	0.000174174	-0.493682487	0.751883669	-0.928722277	0.117868539	-1.253043807	2.34387E-08
AT2G14247	AT2G14247	-11.48876484	3.76778E-16	-3.92470947	0.040561916	-5.388504873	6.06795E-67	-4.466919504	0.242292325	-3.226754681	0.227633707	-1.283996596	0.024275432
AT4G04955	ALN	-1.271516717	0.001400866	-2.562274484	2.57242E-09	-1.032107867	3.54869E-06	-0.027862631	0.990915497	0.051674032	0.954116505	-1.315563682	6.84886E-06
AT5G05250	AT5G05250	-1.450459789	0.023254017	-2.433647629	3.63811E-06	-3.109052473	1.38092E-11	-0.744736418	0.676109289	-1.088722085	0.085265414	-1.68506832	2.3513E-05
AT1G13950	ELF5A-1	-1.693528465	0.020672913	-1.512294632	0.032477495	-2.104032512	0.001297878	-1.053890201	0.634112695	-0.225980813	0.792965164	-1.705643485	3.93505E-09
AT4G32280	IAA29	-2.985721427	7.27542E-31	-2.802412105	2.89747E-30	-2.596999163	1.5087E-19	-0.89650825	0.585601259	-0.797005531	0.001978373	-1.763223665	1.16439E-33
AT5G47240	NUDT8	-1.399058793	0.002282486	-1.469089057	0.001118892	-3.091084634	2.09919E-08	-0.633302164	0.702246294	-0.012143364	0.994809352	-2.18478847	1.45997E-08
AT5G02180	AT5G02180	-2.191921422	0.000710765	-2.039006461	0.001377188	-1.474375657	0.041626114	1.403831901	0.77461156	-0.127746727	0.97257894	-2.537820134	3.75058E-08
AT1G71250	AT1G71250	10.00106958	1.99949E-08	9.409472255	5.27877E-07	9.335412998	3.15651E-10	1.591187288	0.876015705	0.162131517	0.985787245	-2.24256301	0.184963875
AT5G50360	AT5G50360	9.7075514	1.46346E-35	7.507817352	5.26053E-05	3.902600618	5.92613E-06	1.095660348	0.647947756	-0.849714287	0.531396702	-1.044481933	0.128983795
AT2G33160	AT2G33160	9.444437933	6.36479E-08	9.000808052	7.13976E-06	4.115058038	0.009857892	-4.155093527	0.624615508	1.752169302	0.616726708	2.510680823	0.307008243
AT5G07330	AT5G07330	9.205043494	4.46197E-07	6.402453767	6.0852E-10	3.699196946	3.80346E-07	-7.276736786	0.140170159	-1.331848123	0.788747686	-1.602706275	0.134229601
AT5G40790	AT5G40790	9.103396861	1.76786E-06	9.993882215	3.18387E-10	5.135574563	0.000716737	-0.693448958	0.966021495	-6.222171664	0.334261823	-0.917390538	0.764046035
AT1G19530	AT1G19530	6.587991362	9.74897E-68	4.570504277	1.55128E-16	3.68321694	3.18967E-09	1.691590685	0.319072725	0.385087772	0.584309227	-0.541132078	0.301301792
AT4G04450	WRKY42	5.975371457	1.07326E-06	3.009378063	9.49176E-05	3.008574565	1.65372E-09	1.911191921	0.596296903	-0.716624557	0.88172163	0.619501814	0.527441043
AT5G54165	AT5G54165	5.807000248	0.000816549	7.07530431	1.7675E-15	5.664871712	1.15404E-29	-0.273546046	0.961276546	1.354657557	0.306223133	0.152789659	0.870855605
AT4G12410	AT4G12410	5.518325672	8.6083E-10	2.944562926	0.000268634	1.927106014	0.002522142	-1.412403686	0.477936046	0.248291416	0.86223157	-0.038658286	0.958947944
AT4G22620	AT4G22620	5.398854875	0.000638498	5.94683074	2.84614E-05	4.25892527	3.83705E-05	-2.111203552	0.755543384	-0.687511664	0.799785412	-0.818799373	0.334293692
AT2G47190	MYB2	5.371795132	1.73312E-08	6.555423491	0.004880792	4.162004626	0.034586201	-0.003136587	0.999198381	-0.320557602	0.924140734	-1.223411586	0.109522448
AT4G25580	AT4G25580	5.069612351	2.74172E-29	4.879545611	2.37348E-84	3.130776812	5.31139E-81	2.926735321	0.120855166	0.386113045	0.464679378	-0.551542819	0.314902887
AT4G28140	AT4G28140	4.875985047	1.25716E-19	6.412888306	9.12783E-70	4.377556163	0.01583944	3.863384554	0.088918508	0.520144557	0.837269281	-0.722446982	0.541000606
AT3G63060	EDL3	4.845871644	4.0486E-21	5.460715665	5.58119E-24	3.262337604	2.89279E-08	0.013405459	0.997245719	-0.002834079	0.999664963	-0.237730816	0.763113464
AT1G69480	AT1G69480	4.770288143	0.019110058	10.59902398	5.7942E-34	2.199121973	5.3611E-10	1.03773421	0.637614678	-0.35127248	0.887567656	-0.10434898	0.747902488
AT1G05100	MAPKKK18	4.738411484	0.026448667	6.494873532	2.3959E-33	2.863286947	2.22496E-07	1.482991398	0.664635521	-0.55691108	0.713346329	0.47955121	0.420391669
AT5G59220	HAI1	4.732350302	4.02974E-43	6.651284117	9.11076E-22	4.004481488	2.84927E-06	0.455829186	0.782971455	0.484446572	0.176850826	-0.41335727	0.125064874
AT5G05220	AT5G05220	4.687338913	0.015947329	7.867684032	0.00381827	3.73996835	1.44529E-07	1.956094268	0.66524984	-6.815552855	0.109885231	-0.277427239	0.836415396
AT5G06570	AT5G06570	4.628245683	8.67548E-41	3.129720518	1.35263E-18	4.988708365	1.0159E-114	0.756157489	0.629389168	0.131876055	0.836350418	-0.997248828	2.21812E-05
AT3G28740	CYP81D11	4.421431375	1.81795E-22	5.080255697	1.2755E-137	4.10556515	4.056E-168	1.695712227	0.260795959	0.568055673	0.019566611	-0.093926465	0.708651436
AT5G61820	AT5G61820	4.415346217	7.07355E-39	4.918914712	1.23366E-82	3.873884122	1.72695E-83	0.768412306	0.637815859	0.063984899	0.871060407	-0.469637556	0.009732274
AT1G43160	RAP2.6	4.365521628	0.030355471	7.763019023	1.36858E-15	9.771221213	1.16099E-16	1.000506988	0.770575751	-1.264249712	0.661955695	-0.399329166	0.763488011
AT1G52890	NAC019	4.212796779	3.91961E-25	6.89339744	1.11953E-19	3.417432448	1.24987E-07	2.4914813	0.255573307	0.068496847	0.966726249	0.80020145	0.063389071
AT1G02470	AT1G02470	4.200897785	0.001149447	2.221339566	0.018778358	2.306417759	0.031854624	-0.128503284	0.959868884	0.345196533	0.657343215	-0.683498422	0.000340721
AT2G41190	AT2G41190	4.079845839	1.21101E-20	5.560749988	2.2864E-99	4.053177034	5.4188E-22	0.693697611	0.783684933	-0.582576978	0.56302106	-0.591605559	0.003813733
AT1G30220	INT2	4.027809609	0.00159004	4.120924907	5.92944E-17	3.061074394	5.61142E-05	0.127721449	0.93436394	0.20199405	0.86247291	-0.223430466	0.684873307
AT5G02020	SIS	3.999306754	6.32521E-29	4.566704801	8.2214E-193	3.727272283	6.3041E-111	0.982340736	0.616300683	0.3805422	0.274886133	-0.206278442	0.528365762
AT5G16960	AT5G16960	3.925639049	0.042791183	5.345986528	1.28757E-06	4.483931129	6.48998E-29	-0.640610377	NA	1.005706345	0.757778879	0.549373116	0.84860456
AT4G01026	PYL7	3.894839068	3.92887E-07	3.946672652	6.14917E-23	3.223434799	8.459E-77	1.560892207	0.542178894	0.177700293	0.904845876	0.134732341	0.714546216
AT4G33150	AT4G33150	3.865401573	7.21546E-21	4.227648615	2.1656E-81	4.075599702	1.6351E-130	1.241141795	0.569893363	-0.239266046	0.368755836	-0.979834591	2.465E-11
AT5G02580	AT5G02580	3.804519214	1.19603E-05	1.771069762	2.43867E-05	1.886661229	2.40665E-07	0.173788701	0.938741823	0.651030766	0.000619757	-0.157257978	0.498848383
AT2G46790	PRR9	3.788278453	1.33932E-19	3.505956358	1.09215E-19	2.589007929	1.31971E-31	2.43730679	0.104814163	0.153745043	0.927507051	0.107922618	0.917053199

AT2G05380	GRP3S	3.77704666	4.91802E-20	2.843796014	2.2401E-197	2.248596382	1.83351E-06	0.311592801	0.757212383	0.605221238	0.000142413	-0.547271914	0.610830572
AT4G27410	RD26	3.751555463	1.06889E-19	4.43859505	1.8001E-46	2.62147696	1.04519E-26	1.772701748	0.352794607	-0.004673045	0.996204637	-0.336730581	0.288817654
AT3G48240	AT3G48240	3.750827366	1.48365E-10	3.900055816	3.40209E-10	2.741395019	1.12735E-11	2.24978391	0.641679337	0.329039229	0.954114031	-0.215679749	0.867663122
AT1G21520	AT1G21520	3.716797319	6.71963E-15	2.539526526	7.50032E-12	4.653129113	1.88473E-06	-0.002854203	0.999044845	0.84324463	0.004372012	0.055014963	0.858765458
AT1G01520	ASG4	3.707684635	1.01632E-08	5.364343576	1.09096E-18	2.305062173	2.32372E-06	3.836379176	0.062498458	-0.779221521	0.817744169	-0.67745986	0.465795806
AT1G75750	GASA1	3.655908666	0.002572742	3.366060931	2.12734E-44	3.201637185	1.39684E-61	1.020200577	0.474876218	-0.232093279	0.471547396	-0.508857091	0.005586378
AT3G24500	MBF1C	3.644585081	1.5358E-21	4.225963728	4.88836E-24	2.749952005	1.7691E-32	0.271773667	0.731041294	-0.135035218	0.871564236	0.210042283	0.383401945
AT2G36270	ABI5	3.623013958	1.39409E-26	3.866730627	4.5658E-31	2.561189746	3.59548E-14	-0.025738242	0.991271129	-0.178401341	0.751599754	-0.788358885	0.001439752
AT5G39610	NAC6	3.555131336	1.919E-20	3.434599328	2.4858E-38	2.536256863	4.89592E-23	0.823428643	0.575462403	0.064565511	0.91692451	0.205840768	0.24840968
AT3G04070	NAC047	3.50714871	4.12832E-17	3.260105647	2.41082E-15	2.936986978	6.66573E-35	0.548613381	0.662219342	0.697947093	0.200001282	0.073181723	0.840651271
AT1G77450	NAC032	3.492378636	1.94756E-22	4.737531546	7.02285E-42	2.894361633	2.62978E-24	0.54425992	0.66808962	0.059842224	0.923979325	0.510657465	0.000136695
AT5G13330	Rap2.6L	3.464293768	2.24909E-25	3.194600845	2.83566E-28	4.332607805	3.26643E-62	-0.274710394	0.697407195	0.025318713	0.964792889	-0.471000217	0.001594693
AT5G66170	STR18	3.454857155	1.03055E-25	1.80768622	3.60293E-09	1.305310241	0.000888042	-0.413264034	0.777104408	0.823756772	0.002250063	-0.505550949	4.10188E-05
AT4G36900	RAP2.10	3.422925337	1.77335E-14	2.682410011	3.33233E-17	2.340628974	5.86315E-18	-0.158218813	0.939022589	0.559369415	0.284725078	-1.819642577	0.051224332
AT3G16120	AT3G16120	3.376061067	4.33902E-06	3.218974582	9.10006E-05	2.200336404	2.08964E-11	2.41833811	0.632278424	0.325733963	0.918766875	0.127556659	0.916591182
AT3G20340	AT3G20340	3.369513633	1.97503E-10	3.265590788	1.89632E-11	2.181257205	2.77056E-07	-0.130552575	0.951485287	-0.312090517	0.536643581	-0.35557099	0.178436326
AT1G02850	BGLU11	3.345755709	3.36097E-17	4.916993646	9.54567E-17	4.066144254	8.25045E-90	0.625499449	0.633628949	0.455392114	0.012255952	-0.558562027	9.62553E-06
AT3G51430	YLS2	3.330391238	3.43296E-20	3.18626054	1.27544E-48	3.121855374	2.893E-28	0.974331843	0.426758148	0.363834858	0.427495393	0.021825684	0.931924361
AT5G13880	AT5G13880	3.324949786	1.43304E-08	2.326175422	2.96627E-06	1.720711859	0.0384445	-0.089273082	0.967195835	-0.354005163	0.648153416	-0.828231726	0.012460093
AT5G05410	DREB2A	3.317031678	1.40491E-16	3.641972026	2.79604E-10	1.48591535	0.011237654	0.057033113	0.982055475	-0.629254153	0.597382991	0.751175724	0.176614112
AT3G16770	EBP	3.286346427	3.62575E-35	2.06533961	5.09769E-22	3.057428154	1.12555E-46	0.215464258	0.696184854	0.165381519	0.644774623	-0.565324246	0.001366669
AT2G31980	CYS2	3.281966109	7.60067E-31	3.385623578	5.49773E-22	3.252990354	7.82962E-37	-0.67960968	0.205816502	-0.287983493	0.359502101	-0.827612348	9.59321E-05
AT3G61890	HB-12	3.281828565	1.07534E-16	3.145897242	4.54947E-24	1.935741242	1.43588E-17	1.50119888	0.433558429	0.136249889	0.847697457	-0.221281175	0.800238759
AT5G15190	AT5G15190	3.240644225	4.12187E-09	3.902196199	9.10962E-30	3.514264933	3.74857E-07	-0.844459478	0.672344553	0.052294384	0.968338614	-0.913882929	0.280483633
AT2G39800	P5CS1	3.219376763	6.83234E-20	4.827266442	1.2878E-149	3.268324474	1.10249E-11	1.426439187	0.602119156	-0.722681762	0.227789856	-0.673570683	0.000126587
AT5G57910	AT5G57910	3.189933665	3.30843E-17	4.353913924	7.20438E-41	3.557060367	7.69208E-22	0.999424467	0.404349383	0.4422054	0.262547221	-0.642073228	0.003506549
AT5G57050	ABI2	3.125885193	1.3721E-14	4.389975102	8.39857E-59	3.408929711	1.59495E-44	-0.148755498	0.938937153	0.203884116	0.68117884	-0.973466898	2.29507E-05
AT4G26080	ABI1	3.118320717	1.88754E-18	3.439999467	3.063E-59	2.442502103	8.18694E-09	0.918302822	0.601921549	0.138061386	0.673297356	-0.173488252	0.599215829
AT5G47640	NF-YB2	3.111473556	1.37663E-12	3.369517155	3.99164E-21	1.448004576	7.98077E-07	0.065375949	0.978803133	-0.388217726	0.479007201	-0.620384621	0.029931724
AT4G39955	AT4G39955	3.079618567	2.66717E-11	3.626868812	3.29777E-19	2.835975268	1.26322E-11	-0.113235867	0.953801756	-0.133586362	0.856243901	-0.311937058	0.295697874
AT3G14990	DJ1A	3.062502219	1.10731E-26	2.677416947	2.91793E-71	2.269107116	6.19591E-42	0.687639435	0.616300683	0.265926447	0.257757062	-0.164679695	0.153136615
AT2G20560	AT2G20560	3.054688126	6.33391E-10	3.574774777	5.73376E-11	1.977738706	1.9768E-05	-0.304757276	0.901065817	-0.299964493	0.498449509	-0.452536744	0.050708785
AT1G24600	AT1G24600	3.053071384	0.040664797	3.991345572	4.93165E-08	2.96336243	2.76276E-09	-1.743146634	0.379838512	0.417896116	0.950768103	0.072583361	0.908223376
AT5G07100	WRKY26	3.04499697	3.27644E-10	2.442448788	6.71961E-14	4.027941454	7.43503E-86	1.661257584	0.186649478	-0.364542855	0.40552082	-0.862850975	0.000424973
AT1G19540	AT1G19540	3.030406803	2.34374E-12	2.597117545	6.82191E-48	3.798850749	5.72087E-13	2.28494314	0.133800827	0.000189494	0.99989203	0.803919025	1.46865E-05
AT4G15490	UGT84A3	2.996495381	1.35328E-06	3.617355481	8.76354E-43	2.130537213	1.06097E-39	0.620773236	0.742807747	-0.147966827	0.887266124	-0.238947698	0.084513934
AT1G13990	AT1G13990	2.971357536	1.80166E-10	3.850592111	3.90947E-39	3.180382706	1.80965E-27	1.089109795	0.521759167	0.657551671	0.009504015	-0.706070747	0.005925481
AT5G03230	AT5G03230	2.941192976	3.72793E-22	3.552782498	1.70694E-19	1.446531936	4.74385E-12	-0.05920675	0.965812559	-0.023367955	0.965027891	-0.411885405	0.050775856
AT1G64660	MGL	2.931571261	1.10488E-26	3.257475661	6.45055E-76	2.137387529	2.32714E-41	0.249359742	0.816446062	-0.091942633	0.801085181	-0.09287312	0.612078167
AT1G05330	AT1G05330	2.927713363	3.64846E-08	4.300792405	2.70056E-12	2.004486131	0.000489202	1.034712095	0.735907718	0.202623121	0.904202655	0.492646136	0.578108517
AT1G14200	AT1G14200	2.912985781	6.31469E-18	2.480336262	2.84011E-09	1.292262857	9.45502E-06	-0.671325871	0.518021029	-0.180471666	0.758891775	-0.461978033	0.054033889
AT3G20810	JMJD5	2.909742544	1.25584E-07	2.402376156	1.26123E-22	2.103901915	3.75651E-07	0.080892173	0.937935718	0.156684079	0.713614817	0.309309993	0.152439021
AT1G69260	AFP1	2.887734131	3.9033E-12	3.536292168	9.40926E-33	1.803070441	2.05713E-11	0.549698358	0.687176366	0.01563413	0.986818647	-0.665112987	0.003062601
AT4G34000	ABF3	2.86071426	5.69018E-17	3.503422648	1.481E-104	2.987656091	5.13592E-17	0.932322276	0.565728752	0.140132431	0.691792577	-0.235560048	0.424255611
AT4G16680	AT4G16680	2.848317517	3.64198E-13	2.699375756	1.27584E-26	1.663370471	5.39324E-09	1.292407032	0.477936046	0.231963407	0.613407287	-0.746209071	4.43896E-05
AT1G08630	THA1	2.844776053	1.04045E-11	1.897873499	3.23228E-17	3.479316993	1.1415E-109	4.467629949	0.073310574	0.993046798	0.053503415	0.175227731	0.411151111
AT4G05020	NDB2	2.835857674	3.38974E-14	3.39153086	2.23566E-23	2.404534898	1.7882E-08	-0.489237666	0.720626114	-0.063253048	0.943490436	-0.839110947	2.54474E-05
AT1G01470	LEA14	2.801802731	1.67977E-13	3.688744495	6.71403E-35	2.747126883	1.26014E-34	1.584404914	0.451782821	-0.58952481	0.019886905	-0.380206772	0.013118481
AT1G10070	BCAT-2	2.797450426	3.29495E-10	2.155916022	1.72591E-30	2.861185262	1.34865E-59	2.331264311	0.147564873	0.037261636	0.94746792	-0.325471352	0.094985326
AT1G21680	AT1G21680	2.784185752	6.62266E-16	2.665941477	1.53544E-40	1.964251748	2.89658E-06	1.474025803	0.433558429	-0.022997957	0.96653787	-0.631194017	9.5851E-05
AT1G05680	UGT74E2	2.767706023	0.000100221	5.96349347	2.5517E-39	5.423886251	5.26074E-36	0.330977422	0.899467197	-0.260520552	0.83836677	-0.712370107	0.22836003
AT5G57900	SKIP1	2.767065703	3.96012E-22	3.445553973	1.15777E-38	2.24564735	2.57995E-30	0.80180455	0.579644733	0.125899617	0.796567249	-0.44412381	0.029060911
AT2G15830	AT2G15830	2.763597413	1.66473E-05	1.818777906	0.007819427	1.456132276	0.035055776	-0.189367637	0.935898864	0.450863054	0.674518066	-0.36173625	0.534270204
AT2G46270	GBF3	2.753064647	4.18715E-14	3.536188344	4.16865E-47	2.303021445	3.76163E-29	0.443401872	0.800300732	-0.210143288	0.430539275	-0.347622219	0.082220665
AT1G47960	C/VIF1	2.738870324	3.12702E-10	3.425040573	1.05407E-38	1.992372127	8.12088E-17	0.118403451	0.95660961	0.023235041	0.969622714	-0.833248692	1.8277E-07
AT5G42900	COR27	2.737219129	0.000167814	2.684733428	2.89508E-23	3.274471384	1.22209E-06	0.233561323	0.906077763	-0.05086539	0.956275704	-0.816858385	1.32883E-07
AT1G54100	ALDH7B4	2.729460711	2.50694E-18	3.210035597	1.89915E-57	3.124631785	1.48999E-55	1.012079813	0.587820092	-0.0315854	0.945235704	-0.747915533	3.95085E-09
AT2G22470	AGP2	2.700436329	3.58697E-06	3.475077733	1.42133E-12	3.475138534	7.54704E-10	-1.415610351	0.083549925	-0.871683395	0.005792273	-0.998973981	0.001344819
AT1G80110	PP2-B11	2.699452956	7.65361E-09	4.444118683	4.19076E-24	3.336022404	1.71417E-36	0.357147765	0.911737107	-0.137082833	0.87530671	-0.280190343	0.299262542
AT1G73370	SUS6	2.697951517	6.92991E-06	3.063085229	6.89066E-06	1.480482259	0.027878123	-0.296042539	0.888051184	-0.010606258	0.994584402	0.416682152	0.227709792
AT5G24735	AT5G24735	2.695721238	1.77335E-14	2.085088462	8.6582E-30	1.907444828	4.22716E-27	0.627457247	0.616300683	0.193780324	0.555808116	-0.842117472	2.18497E-05
AT5G64510	TIN1	2.690255071	0.000305421	3.074331941	8.94204E-07	2.201183142	1.75647E-06	-0.375108103	0.855277825	0.172301553	0.923509746	-0.599498197	0.017746624
AT3G61410	AT3G61410	2.68792081	4.16866E-05	2.785451944	0.001867666	2.088371124	4.38285E-05	-1.864060988	0.66524984	-0.76596286	0.513456138	-0.388698312	0.519097607
AT5G45310	AT5G45310	2.681683755	2.70682E-10	4.247731143	2.93607E-54	2.243458456	1.98025E-29	1.58769835	0.496127053	-0.073357526	0.957156296	0.223479838	0.662236256
AT4G35720	AT4G35720	2.680293169	2.1507E-13	2.904573109	6.09461E-24	2.242829834	1.32146E-14	-0.622143421	0.694798261	-0.065936257	0.824246731	-0.949379988	1.58999E-05
AT1G07400	AT1G07400	2.675662407	0.008605535	3.547466204	3.44795E-09	3.472919642	2.16275E-05	-0.711820249	0.677952867	-0.402366193	0.693364203	0.074584441	0.908766922
AT3G51000	AT3G51000	2.664637747	6.80282E-12	2.676439698	3.6321E-30	1.831024349	6.10877E-32	1.512610302	0.343779816	0.040596833	0.948497787	0.416789561	0.000936132
AT2G15960	AT2G15960	2.662454202	6.4326E-10	1.7641836	3.69892E-18	1.698667667	7.38465E-26	1.711705631	0.268420306	0.699729792	0.000124569	-0.507426258	0.290561499
AT3G29575	AFP3	2.642596556	2.39778E-17	2.894657196	7.40369E-26	2.588743338	6.96769E-28	0.320700283	0.790756089	0.016339726	0.975427751	-0.477501373	0.057629685
AT1G45976	SBP1	2.628147409	3.27361E-15	1.81346692	4.2848E-26	1.866752025	1.50116E-39	0.799614823	0.62738272	0.111535898	0.795808621	-0.576878233	0.000311946
AT5G47590	AT5G47590	2.621064935	8.95152E-09	2.310907676	1.14669E-17	1.696209417	3.02198E-19	1.838546796	0.17999167	0.491122722	0.152974337	-0.091188861	0.803671717
AT3G54820	PIP2;5	2.614484705	2.92594E-09	1.550872695	4.05768E-07	1.88851814	5.30458E-13	-0.39707102	0.65378996	-0.34187161	0.318380163	-0.265900978	0.180397316
AT1G26665	AT1G26665	2.572240609	8.89401E-17	1.944782996	1.05257E-41	2.354764655	8.7933E-43	0.493682458	0.619462504	0.231975298	0.252204904	0.019063317	0.912574926
AT1G17745	PGDH	2.556327648	0.036399839	2.365263675	0.000331336	1.908333235	0.028084134	-0.771269691	0.783775657	-0.067563701	0.930954102	-0.61874501	0.003533801
AT5G37720	ALY4	2.551859481	3.72354E-12	1.636826892	7.71338E-14	1.413259383	4.59618E-11	0.075713739	0.973896678	0.364389968	0.228038221	-0.501173231	0.575867865
AT2G37180	RD28	2.539877967	2.35065E-11	3.385940916	6.87856E-44	3.245008641	2.13649E-63	0.35375936	0.833174899	-0.808875903	2.65419E-06	-0.53417592	0.004091489
AT4G34135	UGT73B2	2.539055894	6.40728E-13	2.03380953	6.19922E-13	2.325655724	3.50418E-20	0.323295195	0.676109289	0.28258088	0.160507774	0.395585614	0.006096228
AT1G45145	TRX5	2.527762251	2.23324E-11	2.840541362	3.00768E-19	2.181067982	1.96717E-23	-0.379389663	0.78630406	0.438852736	0.094913021	-0.856355156	1.60431E-06
AT5G53970	TAT7	2.524910997	2.19659E-10	3.205983696	4.34594E-37	2.385122132	4.9563E-61	2.309136887	0.186649778	0.260702745	0.56302106	-0.379905996	0.071668188
AT3G14610	CYP72A7	2.522682359	7.96594E-11	1.598494517	1.61025E-09	1.18615699	0.000805895	-0.351341489	0.716554635	0.200258583	0.493686501	-0.243096893	0.109024116
AT3G21250	ABCC8	2.520502624	4.1747E-12	3.030044194	4.19078E-25	2.514348329	2.31593E-37	1.386496024	0.107090587	0.420629903	0.01770954	0.040582169	0.8560923
AT5G10625	AT5G10625	2.519326772	4.17002E-12	2.279981747	1.42331E-12	1.516209048	5.0228E-25	0.682526845	0.695103554	-0.029587683	0.966179231	-0.675633795	0.000280897
AT3G51860	CAX3	2.51193756	2.67706E-10	4.19736466	2.77952E-20	2.797686027	1.43052E-21	-0.583575347	0.512070232	-0.208349807	0.705122473	-0.076512355	0.761927143
AT1G48840	AT1G48840	2.491416342	1.98478E-08	2.751535644	3.46783E-16	1.381734085	2.89867E-27	0.786609651	0.66808962	0.397105677	0.366910832	0.036937283	0.877826292
AT1G06570	PDS1	2.489700152	1.55195E-11	2.672364772	5.68762E-56	2.630244361	1.57711E-88	2.180270067	0.180725221	-0.068334892	0.87530671	-0.668054498	3.76711E-06
AT1G01720	ATAF1	2.48408981	1.19824E-10	3.581951738	1.69857E-27	2.370831921	4.81432E-52	0.360255674	0.816446062	0.067821467	0.877301402	0.236782039	0.141453991
AT4G34131	UGT73B3	2.480765784	1.53222E-09	2.070273013	8.04744E-08	1.523318992	0.00063757	0.154939389	0.823181035	-0.168097056	0.822542295	-0.971292294	1.58021E-05
AT3G09390	MT2A	2.480061581	3.37419E-13	2.29685762	3.12585E-34	1.247432953	3.89859E-09	0.011167996	0.992662394	0.352533064	0.075136703	-0.396118961	0.061980972
AT1G45249	ABF2	2.478026134	4.1641E-10	3.331212255	5.80953E-26	1.5834624	2.43121E-08	0.111125997	0.923573229	0.080052114	0.866906715	-0.680316445	0.015350791
AT3G45300	IVD	2.475780594	1.07502E-10	2.457084392	2.16429E-40	2.501023759	6.73629E-67	1.637555123	0.268420306	0.387510863	0.053208839	-0.146242743	0.525320751
AT1G01010	NAC001	2.464342509	1.96992E-06	2.050809954	5.78837E-05	5.047857661	1.0586E-161	-0.41264685	0.782073003	-0.410688621	0.331465476	-0.186521094	0.357923498
AT5G17450	HIPP21	2.461775279	3.07267E-06	3.174870163	2.88432E-18	1.787368792	4.33838E-05	0.493165201	0.712887303	0.503934242	0.579707687	-0.139410537	0.791409046
AT5G54080	HGO	2.45963723	4.02819E-12	2.74174584	1.73483E-56	2.212221453	7.66835E-51	1.844117476	0.242292325	0.150005436	0.685800226	-0.073168524	0.702678138
AT4G35560	DAW1	2.451123563	9.90997E-10	3.003619008	9.86509E-24	2.560297482	1.47379E-45	0.649920836	0.641679337	0.079773577	0.905843881	0.121563608	0.608133281

AT2G38530	LTP2	2.445567714	0.01301791	2.462341724	1.15774E-46	3.179545877	3.39238E-59	-1.224558426	0.465437463	-0.345334899	0.035174141	-0.402321928	0.002063527
AT5G01600	FER1	2.41794561	8.27668E-21	2.807805128	6.87412E-40	3.103018918	1.88059E-14	0.410904841	0.767355318	0.07722964	0.857138563	0.138770127	0.278237473
AT3G26210	CYP71B23	2.414092177	2.49844E-15	1.602642587	0.001951113	1.090698536	0.026997823	0.609963443	0.514833076	0.531926137	0.194395077	-0.352139506	0.25269069
AT3G11410	PP2CA	2.408829336	7.47011E-08	3.933990064	8.76354E-43	2.168554713	5.77389E-17	0.353991021	0.867112468	-0.530419696	0.075784836	-0.980587814	3.65924E-05
AT3G48115	AT3G48115	2.396609031	5.26972E-12	1.501346194	2.20414E-13	1.614949025	1.58767E-23	0.109985175	0.882425056	0.23162569	0.28536795	-0.731855892	1.83799E-07
AT4G02520	GSTF2	2.390420025	0.006391573	2.471077313	0.007609203	1.849548378	2.26146E-13	0.349397768	0.868407292	0.03628153	0.986818647	0.742310739	3.98331E-10
AT4G15670	AT4G15670	2.361588057	8.50717E-07	2.577622691	3.21406E-06	1.963840048	9.35275E-05	0.520585107	0.807506486	0.214785892	0.776086194	-0.012711562	0.979079581
AT3G19300	AT3G19300	2.35644522	6.29066E-05	3.712958909	4.61522E-12	1.260141135	0.005529804	0.549070863	0.696427231	-0.392544479	0.530549155	0.1639691	0.639116582
AT4G16690	MES16	2.350556696	2.35662E-07	3.638931427	5.02572E-26	3.831867895	7.51929E-81	0.842445827	0.672844426	-0.114182322	0.838708765	-0.84381876	1.33921E-07
AT3G11020	DREB2B	2.334515096	0.040750375	2.834028435	0.01360474	1.610044642	0.003556156	1.291237857	0.814101614	-0.851826901	0.592200811	-0.201142549	0.560709396
AT4G34030	MCCB	2.330370282	3.3298E-11	2.502728795	1.14686E-54	2.606696889	1.11722E-84	2.146481937	0.204145338	0.15439842	0.615469901	-0.134704036	0.563331175
AT3G10500	NAC053	2.323980429	1.10528E-09	1.672010953	2.11987E-08	1.605143944	5.01991E-16	-0.073406842	0.965260868	-0.155170488	0.729103822	-0.756707118	1.80192E-05
AT4G21534	SPHK2	2.304589559	3.45355E-09	1.674514201	4.51915E-12	2.014482899	4.7053E-14	0.395096713	0.823849926	-0.300798975	0.382648726	-0.693876596	0.003433807
AT2G37410	TIM17-2	2.303014881	3.78253E-21	1.315192784	2.22319E-16	1.427605301	2.59224E-16	0.315680083	0.716289725	0.325732439	0.0818318	-0.581751265	0.000716512
AT2G39350	ABCG1	2.300683158	5.86959E-06	2.656983797	1.71575E-09	2.276625927	4.38558E-11	-0.615995594	0.164934579	-0.229142007	0.532315551	-0.528176781	0.001460849
AT1G20450	ERD10	2.300078961	9.7123E-09	2.972124315	5.37398E-55	1.288600769	1.23164E-28	1.922379353	0.294865885	-0.430025699	0.05047913	0.325525994	0.064539726
AT1G69410	ELF5A-3	2.293891116	3.16214E-13	2.675979179	1.61749E-23	1.929635849	9.43252E-17	0.222444222	0.744250473	0.141615894	0.70029725	-0.41257928	0.031178777
AT2G38820	AT2G38820	2.286245378	2.37743E-08	2.289302204	1.27685E-15	1.31550087	2.99211E-13	1.548832246	0.409180759	0.574252675	0.091180997	-0.171900248	0.621085479
AT4G21323	AT4G21323	2.27145913	3.05199E-05	1.466940574	0.04066659	1.641726584	0.000158737	0.312781868	0.853062846	-0.058271704	0.973725013	0.179235713	0.719365777
AT2G31990	AT2G31990	2.271192157	0.000305421	2.051300806	0.001893111	1.560352766	0.001342347	-0.162544595	0.944575187	-0.072650099	0.957093674	-0.776833942	0.001611792
AT1G73066	AT1G73066	2.264766706	0.001683674	2.184856006	7.41833E-05	1.852130482	2.80155E-06	1.008506593	0.597380545	-0.697070243	0.575644885	0.765515259	0.064805646
AT1G78070	AT1G78070	2.26219824	7.02195E-09	3.222522487	5.29067E-24	1.353491609	5.76032E-06	1.268865911	0.616300683	-0.597114271	0.050769647	0.45803757	0.048773274
AT5G16080	CXE17	2.261245843	3.87207E-08	2.515002639	2.20414E-13	1.969721289	2.25578E-09	0.927051169	0.701052583	-0.371935722	0.593339308	0.306443737	0.503447575
AT5G01520	AIRP2	2.257530638	4.03882E-09	3.595668731	3.09461E-39	3.058298794	1.42724E-39	0.6584286	0.709586436	-0.25948159	0.524761417	-0.852896733	0.000467104
AT4G15530	PPDK	2.254950871	6.49711E-09	2.293393814	2.96407E-18	2.943123488	2.66488E-62	2.597113158	0.058413663	-0.012540559	0.988694176	-0.632193147	0.003753377
AT5G52450	AT5G52450	2.24701533	0.00027265	1.995242444	1.87183E-06	2.035861372	2.5695E-05	0.28896953	0.79553587	-0.412162045	0.343296444	-0.200359935	0.315543495
AT1G13740	AFP2	2.243002849	2.12654E-09	1.432781311	0.009951866	1.132881411	0.000776787	-0.353284808	0.753910599	0.239762426	0.713614817	-0.841839583	0.000769822
AT4G14430	IBR10	2.240984684	1.77335E-14	2.002839777	3.6732E-17	1.778371285	5.81239E-21	0.292406138	0.831161184	0.030134436	0.963419708	-0.510842621	0.005912629
AT5G53120	SPDS3	2.234682322	3.30962E-11	2.531326307	1.52862E-15	3.022911804	5.37856E-24	-0.211658745	0.845839675	-0.21677213	0.565049692	-0.253435983	0.171555282
AT5G22290	NAC089	2.233478354	1.58651E-12	3.275047846	5.04802E-29	2.32263936	4.3894E-25	0.1944815	0.936877812	-0.143639702	0.797128275	-0.63496091	0.002976715
AT1G53180	AT1G53180	2.232727439	9.15124E-09	2.563089438	2.54686E-13	2.811107515	5.70091E-24	-0.057174303	0.972931218	0.052691807	0.919937283	0.040808349	0.896595909
AT4G38060	AT4G38060	2.228949544	3.79786E-10	1.683624399	1.07655E-16	1.666534052	3.52758E-13	0.567851456	0.735695447	0.575628138	0.056779167	-0.835767875	0.02427087
AT5G26340	MSS1	2.224156738	4.24466E-09	3.095554557	7.91885E-29	1.80881606	5.44343E-07	0.342795624	0.89610223	-0.589800493	0.178164429	-0.926460131	4.70847E-07
AT2G45920	AT2G45920	2.220543505	9.6009E-08	2.331169925	1.63559E-11	2.659283804	6.64573E-44	-0.054522546	0.981525175	0.197344715	0.55299139	-0.823637503	0.000222033
AT1G11210	AT1G11210	2.216098299	5.04855E-07	3.030634682	1.12384E-17	2.021042977	9.95721E-13	0.255157743	0.906077763	-0.49858638	0.145164419	-0.575010836	0.027934132
AT1G72770	HAB1	2.214904806	1.24375E-09	3.354917492	3.04954E-38	2.687359457	9.95161E-33	0.544235341	0.711996909	-0.050875176	0.918426331	-0.555216052	0.00073514
AT5G63030	GRXC1	2.212771094	5.14073E-11	2.082883706	6.89071E-28	1.230084689	1.99681E-05	0.994546796	0.234234306	0.041482577	0.945176019	-0.500748025	0.026242696
AT1G22985	CRF7	2.212218843	0.001042319	1.668582957	3.21228E-06	1.182370365	0.000298518	0.406935577	0.874228094	0.130927993	0.89093772	-0.746928573	0.041989712
AT1G77380	AAP3	2.209715905	1.54402E-07	1.748823046	4.44445E-07	1.055588968	0.008690801	0.09155359	0.93664894	-0.318228646	0.36366911	-0.77858314	5.45326E-09
AT4G12400	Hop3	2.207305894	0.002011686	2.044648962	0.000285992	1.56984443	2.57127E-09	-0.425663587	0.716554635	-0.148876053	0.852285116	-0.573849286	0.005963393
AT2G28400	AT2G28400	2.203842084	2.12511E-06	2.490234627	7.69951E-18	2.536708685	1.33449E-27	1.132914651	0.536956184	0.324688772	0.525334184	0.349955237	0.098881204
AT1G79360	2-10月	2.201503452	7.34547E-05	1.165444856	0.000839421	2.462648535	5.52188E-74	2.508334043	0.14744465	0.109215503	0.930662149	-0.098244998	0.761927143
AT2G32120	HSP70T-2	2.196106974	2.7355E-06	1.982074108	2.27841E-06	1.611341244	8.61697E-08	0.334256058	0.842959841	-0.034609317	0.98143759	-0.526008545	0.028454881
AT1G64950	CYP89A5	2.193397635	3.16321E-07	2.69655118	1.41898E-21	1.079444271	0.003160105	1.037396365	0.488977185	-0.162476403	0.752244106	-0.466628894	0.041167205
AT4G13540	AT4G13540	2.184276578	0.000291892	2.259188107	1.68233E-07	2.880245783	1.18302E-18	0.777594433	0.647947756	0.064849903	0.930903945	-0.371343788	0.36033647
AT4G01610	AT4G01610	2.172442412	5.23114E-22	2.100758818	2.62049E-19	1.610596972	2.68477E-14	-0.373492562	0.616300683	-0.159175837	0.603158863	-0.738488979	3.3813E-09
AT1G72680	CAD1	2.169387596	5.82124E-15	3.082356275	3.8704E-25	2.633471246	4.61205E-28	0.343500686	0.785453357	0.367416058	0.145348244	-0.020228562	0.942204773
AT1G17530	TIM23-1	2.165190006	2.60657E-22	1.633204299	7.30572E-13	1.413673165	4.74498E-19	-0.051008568	0.981229441	0.229175047	0.327759772	-0.526412081	0.005149636
AT1G55530	AT1G55530	2.161771079	9.7793E-22	2.248379552	3.0069E-29	1.824822135	2.41232E-25	0.38873491	0.687176366	0.43664427	0.073201051	-0.163217531	0.422195534
AT5G46180	DELTA-OAT	2.160415636	1.76574E-14	2.681600043	1.06364E-31	2.228808674	1.08854E-41	0.244512446	0.81577697	0.200544784	0.455415792	-0.494282665	0.000247222
AT2G22660	AT2G22660	2.159430986	2.20894E-12	1.783759313	1.06843E-36	1.601352772	1.3395E-28	0.37943144	0.732202251	-0.02402221	0.963495178	-0.107977667	0.693610471
AT4G15660	AT4G15660	2.159329689	3.21675E-06	3.33112724	0.005461156	1.688529056	0.002081866	0.141936497	0.969302142	0.680712613	0.040889919	-0.646137924	0.072006788
AT1G69270	RPK1	2.157879697	0.047129565	2.778120001	1.46175E-06	2.102115837	1.86042E-18	1.271580279	0.662219342	0.699738907	0.616062875	-0.071436587	0.814315114
AT2G01010	AT2G01010	2.150748323	9.73089E-13	1.949160365	1.99109E-24	1.028135253	2.87514E-07	-0.251352946	0.865029651	0.362780337	0.618550064	-0.707848224	0.523280751
AT3G14560	AT3G14560	2.141564392	1.03838E-09	2.725499873	1.93803E-10	1.889756364	8.24231E-25	-0.041051716	0.981525175	0.438004095	0.174037213	-0.345781617	0.220730644
AT5G06370	AT5G06370	2.138630814	4.58322E-11	2.383109129	1.83238E-37	1.821507287	3.90508E-27	0.252323453	0.854231696	0.029679497	0.954114031	-0.792419374	1.78115E-12
AT2G41210	PIP5K5	2.136610296	4.60025E-08	2.849569517	1.13834E-16	2.710411342	3.39813E-28	0.36482055	0.832157671	-0.021311768	0.980663768	-0.971708187	1.46778E-05
AT1G35720	ANNAT1	2.136347852	1.55884E-11	2.223601841	3.28205E-24	1.78560687	2.59445E-19	-0.831028723	0.572888325	-0.301324371	0.09955352	-0.148865081	0.245352642
AT1G14890	AT1G14890	2.1211404	1.96581E-13	1.987009719	1.86998E-19	1.458453541	5.78197E-09	0.181131361	0.836588087	0.212965417	0.271501627	-0.460315412	0.000931487
AT4G14270	AT4G14270	2.110035454	3.26623E-14	2.113376741	1.81062E-34	1.460903499	1.96176E-15	0.144643361	0.908772285	0.689277868	7.75567E-05	0.235821531	0.404029055
AT5G16600	MYB43	2.106664416	8.60071E-05	1.953437912	8.99283E-08	1.038993978	0.017304506	-0.165770864	0.945609936	0.202004917	0.641639	-0.005727308	0.987386736
AT3G19290	ABF4	2.105785609	1.12923E-10	2.462876276	3.76645E-27	2.039751905	1.27E-28	0.670267692	0.680482607	-0.076627673	0.890869479	-0.541253342	0.034851589
AT5G13820	TBP1	2.103756767	0.000185138	2.707278745	1.78148E-09	1.507646067	0.002037613	-0.431130407	0.726635812	-0.262179958	0.613826202	-0.596097121	0.03358386
AT4G22820	AT4G22820	2.096102452	2.34995E-13	2.362644978	5.07981E-15	1.182641296	1.05506E-07	-0.409424231	0.554617999	-0.396025934	0.147168146	-0.539837651	3.39014E-05
AT5G48160	OBE2	2.093858743	3.6858E-13	1.461263889	3.8498E-24	1.727952542	7.75726E-40	0.604744628	0.685495817	0.084392847	0.798314629	-0.714619992	1.34477E-05
AT3G14590	NTMC2T6.2	2.084465134	1.54975E-05	2.907514804	1.22324E-13	2.232467515	7.06979E-06	-0.009731312	0.997515054	-0.111975631	0.889397186	-0.686317866	0.011822982
AT5G10860	CBSX3	2.084105734	1.99189E-12	1.672809173	9.14926E-28	1.333965026	4.4203E-16	0.653957213	0.582068518	0.191190206	0.363015761	-0.134916485	0.380506538
AT1G77000	SKP2B	2.079787475	3.35373E-05	2.826908184	1.1832E-13	1.889010314	3.10409E-08	0.359373793	0.662219342	0.432227274	0.219497123	0.045284895	0.879980604
AT1G49032	AT1G49032	2.078378159	1.19691E-08	1.669190128	2.55285E-12	1.084180385	3.67802E-07	0.850048075	0.542342434	0.235900667	0.417835615	-0.884564432	5.91385E-06
AT3G19990	AT3G19990	2.072872656	2.3006E-06	1.381425581	1.41716E-11	1.565628438	1.84905E-15	1.210455114	0.482795025	0.181771391	0.670869081	-0.394455849	0.208047335
AT1G55850	CSLE1	2.072251828	8.77522E-16	1.411854564	4.55859E-12	1.335027556	3.17801E-14	0.006768167	0.996262282	0.072884019	0.842607116	-0.607983752	0.000621938
AT2G42790	CSY3	2.071024913	2.2769E-10	2.612229311	7.3944E-35	1.933131629	1.43052E-21	0.26521419	0.87813535	-0.392949493	0.098024026	-0.575427553	5.78736E-06
AT1G75770	AT1G75770	2.066074606	0.000226772	1.998554934	7.26421E-09	1.389374471	1.13024E-10	0.563581383	0.702246294	0.242515293	0.780558679	-0.730016261	0.002868787
AT1G77680	AT1G77680	2.060836486	1.44481E-09	2.296550584	1.32286E-11	1.495064512	8.87421E-12	0.151471405	0.938741823	-0.370417809	0.131410428	-0.496968342	0.000865429
AT2G16700	ADF5	2.059296134	2.43961E-07	1.878187233	2.59531E-11	1.630006818	2.50233E-15	-0.130579935	0.937178328	-0.011945216	0.98877807	-0.700887878	0.002047111
AT1G50420	SCL3	2.058963414	4.22551E-06	1.651684005	4.52499E-06	1.372119429	1.07929E-09	0.128794802	0.924925439	0.10301238	0.834745508	-0.476113542	0.000981542
AT3G51890	AT3G51890	2.057913794	3.23145E-05	2.234611329	6.91057E-13	1.691889602	9.95072E-26	0.5548731	0.668028007	-0.081332829	0.911423117	-0.374801415	0.132224051
AT3G48580	XTH11	2.05590559	0.016553665	3.191047833	0.011918846	2.645487863	6.28802E-14	0.987257479	0.704225272	0.117701895	0.958007814	-0.106814417	0.853631454
AT2G16586	AT2G16586	2.052085903	0.001715641	3.155306987	3.66385E-10	2.493935024	1.21132E-06	-0.146288411	0.962521414	0.043790019	0.98143759	0.611519441	0.254393253
AT4G27970	SLAH2	2.040897349	0.016431781	2.163511028	0.000472508	2.900404695	0.001405581	-0.804538627	0.379538351	-0.704085163	0.085087943	-0.017148663	0.972585708
AT4G11660	AT-HSFB2B	2.040736688	0.00026541	2.543073953	3.06356E-10	1.336086795	6.20595E-05	-0.304849396	0.810393493	0.19458353	0.811222926	-0.115705028	0.794612983
AT4G01870	AT4G01870	2.039912352	3.03267E-07	3.05706564	4.7558E-34	2.247723863	3.5459E-108	1.528043568	0.430181703	-0.126762306	0.77201177	0.174678432	0.287306826
AT1G73480	AT1G73480	2.030868097	4.37166E-07	2.883662404	2.88438E-26	2.001546213	1.10127E-26	1.268685116	0.574062337	-0.124553009	0.854600248	0.05243207	0.843810536
AT3G13065	SRF4	2.016718156	0.000354813	1.812252291	0.000406074	2.154634379	1.11577E-14	1.749398836	0.451223032	0.783011076	0.445236462	-0.020126247	0.971547415
AT3G26220	CYP71B3	2.014004402	4.79668E-06	1.915755522	5.92379E-19	1.584980188	1.42102E-09	1.200748912	0.317624416	0.164270021	0.758461089	-0.318111493	0.143348621
AT2G21130	AT2G21130	2.004150749	0.00228187	2.3675266	2.6206E-11	1.072686939	0.00263397	0.512600718	0.598901706	-0.06430484	0.924627441	-0.354093578	0.042497493
AT4G13110	AT4G13110	2.003665032	0.000901994	2.263460277	7.68444E-07	1.136737324	0.00014612	0.341905868	0.857378455	-0.220791554	0.805320688	-0.515136866	0.009558738
AT1G03470	NET3A	1.9944057	1.78195E-05	1.719137884	3.27043E-07	1.232753308	7.54971E-06	0.466224398	0.646729152	0.528609756	0.208866119	-0.018401712	0.968386723
AT3G62650	AT3G62650	1.98390456	5.46905E-08	1.60453187	1.35596E-17	1.39388403	4.78067E-12	0.878091179	0.519283767	0.229486548	0.302207375	-0.715426668	0.121611282
AT3G12320	AT3G12320	1.981814575	2.81818E-05	2.56477714	5.57329E-14	1.158741517	0.029445692	1.457615335	0.260795959	0.391793758	0.199674929	0.708160735	7.6934E-05
AT5G59590	UGT76E2	1.978203283	2.07407E-06	1.598059229	0.011003699	1.21675586	0.020078201	-0.427513403	0.680482607	-0.479535761	0.268092126	-0.521042593	0.082344487
AT4G31420	AT4G31420	1.977982834	1.67208E-12	2.136858728	2.85039E-27	1.403878402	1.88537E-24	0.574616099	0.647883443	-0.00977001	0.986774932	-0.441114182	0.000962195
AT5G05965	AT5G05965	1.972598795	3.56122E-07	2.269550109	1.67318E-11	1.907181871	1.0267E-14	0.008632522	0.996799452	0.143345501	0.801085181	-0.71053167	0.000138305
AT5G11260	HY5	1.969495841	0.0146101	1.382401161	0.005662031	1.290778371	0.000776751	-0.437008543	0.622600949	0.121463963	0.846410046	-0.586702966	0.011927587

AT1G12780	UGE1	1.967449985	2.29532E-08	1.953323236	2.17617E-35	2.002916445	1.22568E-75	1.331824074	0.474958589	0.057123347	0.888329824	-0.374276185	0.000712768
AT2G41870	AT2G41870	1.967065974	0.000144274	2.726123113	4.00743E-22	1.015327248	7.32957E-06	1.987331353	0.281913411	-0.480757486	0.430488323	-0.948310961	0.000523168
AT1G53170	ERF8	1.959603797	9.30873E-07	2.898503869	2.69209E-19	1.621322653	6.3359E-16	1.006963348	0.606948021	0.424096998	0.272079517	0.985289027	1.42346E-10
AT1G07150	MAPKKK13	1.951608701	7.3622E-08	2.546977534	3.02511E-07	1.09918207	0.000705733	0.202590392	0.953774208	-0.151930667	0.898446957	-0.289521861	0.450406599
AT1G58360	AAP1	1.95104465	9.09685E-10	2.296845367	8.81201E-20	1.426771889	4.64642E-17	0.322143397	0.828990118	-0.435510625	0.043651375	-0.738028699	1.99258E-06
AT1G80570	AT1G80570	1.950959333	0.000111661	1.800948883	2.50016E-13	1.438641819	6.92299E-11	0.599934308	0.712887303	-0.006805733	0.994163341	0.248971281	0.232570838
AT2G24300	AT2G24300	1.950090199	0.022276225	2.058622713	1.64478E-06	1.933636664	1.36451E-10	-0.135336287	0.947977983	-0.233895617	0.511188955	-0.183835689	0.557211813
AT4G11220	BTI2	1.945337995	3.37864E-16	2.155374666	6.07331E-12	1.268283224	3.16317E-08	-0.194537225	0.806096089	-0.08172061	0.852285116	-0.637690047	0.000103456
AT5G23050	AAE17	1.938141858	2.8541E-10	1.863261094	6.14917E-23	1.636375854	2.66143E-26	0.939661698	0.532520266	0.342878814	0.242790905	0.481135669	0.000136721
AT2G36770	AT2G36770	1.927665827	0.000289617	3.308636641	2.73225E-16	1.81114313	0.006332289	0.289624193	0.937313144	-0.859104005	0.36223266	-0.6377422	0.298845114
AT4G04620	ATG8B	1.927216975	2.57393E-10	1.647467061	2.15443E-13	1.885384429	1.66589E-41	0.586460633	0.622600949	0.251752914	0.319571819	0.093348609	0.557302819
AT2G21620	RD2	1.921344472	4.1991E-22	2.327446909	1.94744E-14	1.357024983	3.78317E-11	-0.376755855	0.429222482	-0.00772651	0.986818647	-0.555172708	0.000418528
AT1G07040	AT1G07040	1.915582838	6.37617E-08	2.075937019	3.39287E-19	2.485505143	2.71294E-28	1.656827409	0.2995105	-0.034790146	0.946850229	-0.270515191	0.075272875
AT5G04250	AT5G04250	1.897325461	4.05927E-05	2.694785707	1.78332E-08	2.289283266	3.2107E-05	0.19062148	0.93556951	0.42245636	0.510621252	-0.283524797	0.635573767
AT5G10930	CIPK5	1.892575224	2.80251E-06	2.768207493	8.67106E-15	1.391992331	0.000511545	-0.512691572	0.433558429	-0.354512189	0.385100337	-0.538189537	0.021464296
AT2G30140	UGT87A2	1.890210657	9.57562E-09	3.157286664	8.01364E-31	2.208965334	8.38979E-22	-0.031274329	0.994162268	0.342563041	0.510723249	-0.368342769	0.011495485
AT3G11580	AT3G11580	1.889372778	0.010053476	3.022089773	0.000314936	2.194365765	1.11418E-08	-1.073007386	0.340875713	-0.458117657	0.627293967	-0.665172129	0.068584752
AT4G01120	GBF2	1.882266015	1.10767E-07	2.176750048	2.64778E-28	1.334452513	4.58832E-22	0.802221897	0.598901706	-0.009946885	0.986818647	-0.180272422	0.239187998
AT1G17830	AT1G17830	1.879556125	0.002050893	1.888311633	0.000744836	1.638124641	0.000521845	-0.825195992	0.365746137	-0.097541665	0.930903945	0.258006014	0.38673658
AT3G03440	AT3G03440	1.87561779	0.010294082	2.574657051	1.64478E-06	1.446738355	0.028987235	-1.019995739	0.494599505	-0.073913593	0.959215642	-0.714000667	0.029691673
AT1G08230	AT1G08230	1.868395046	4.13499E-08	2.061810707	8.00899E-18	1.190726792	4.36925E-07	0.476962295	0.606948021	0.214518432	0.485888437	-0.226371208	0.413316443
AT5G39050	PMAT1	1.841500325	6.82315E-07	2.68878803	2.40695E-33	1.971406561	1.33823E-23	0.26227299	0.652047062	0.282713605	0.366726987	-0.166529511	0.512629735
AT2G02390	GSTZ1	1.838494327	4.01762E-10	2.561586173	1.21855E-31	1.675993692	1.11789E-21	0.263749001	0.855277825	-0.032717323	0.958669515	-0.200211784	0.17677859
AT2G42890	ML2	1.836694797	7.02195E-09	1.754026552	7.20962E-27	1.990600057	1.63725E-23	0.517941954	0.721771703	-0.059140089	0.89791993	-0.591598423	0.00069751
AT3G51500	AT3G51500	1.824611381	7.29598E-08	1.591650141	2.47604E-11	1.671583503	3.42377E-18	1.018861426	0.408213729	0.268403714	0.321965943	-0.157069033	0.442713722
AT2G37760	AKR4C8	1.820752266	9.60424E-13	3.431200397	1.14409E-39	1.488569997	1.8251E-07	-0.281999886	0.676109289	0.282047666	0.211913228	-0.535613969	0.00052053
AT1G73390	AT1G73390	1.819316783	5.69404E-06	2.638311703	1.00896E-32	1.490046345	2.83048E-15	0.860215206	0.604422229	0.046186694	0.953851164	-0.236595408	0.276397463
AT4G24040	TRE1	1.818109682	0.001167083	1.688496879	6.12051E-07	2.049451788	4.50515E-20	0.240153902	0.935432099	-0.291129334	0.570555085	-0.668682043	0.000156607
AT5G14240	AT5G14240	1.808539422	3.94247E-08	1.367499819	2.92346E-19	1.150343442	6.63437E-13	0.494838554	0.611321113	0.189994039	0.516852689	-0.265384629	0.154018091
AT4G31750	WIN2	1.802767902	1.94806E-05	1.479952932	7.47884E-05	1.154875818	3.0009E-06	0.204052953	0.855081587	-0.078929397	0.905220501	-0.449297068	0.07019549
AT4G24960	HVA22D	1.791736598	2.48155E-07	2.648762264	7.05346E-17	1.349722244	4.12979E-06	0.248031187	0.709338752	-0.13790459	0.78520308	-0.153571246	0.533639437
AT2G32520	AT2G32520	1.78973291	3.4813E-15	1.35037282	6.37425E-15	1.278698865	2.95587E-22	0.247192479	0.769724647	0.23596676	0.420207266	0.348458931	0.015886342
AT4G19880	AT4G19880	1.789091122	5.46662E-08	1.505033235	4.02881E-19	1.723946179	1.77818E-26	0.092564503	0.953923605	-0.269458335	0.288524312	-0.594761846	1.34628E-07
AT5G12840	NF-YA1	1.781863446	8.26874E-07	2.01051659	5.1099E-11	1.296021364	1.88334E-13	0.645838377	0.604415921	0.19650217	0.638629847	0.131094297	0.634453188
AT2G36900	MEMB11	1.780109469	8.12399E-09	1.459999493	3.3061E-10	1.430730253	4.97023E-15	0.569286286	0.62738272	0.43529471	0.010735268	-0.063785919	0.77899148
AT5G56870	BGAL4	1.775794659	8.49762E-08	1.091667004	4.51163E-05	2.852966167	4.9006E-43	1.324624142	0.565728752	-0.4252717	0.085226407	-0.972319243	6.77752E-12
AT3G23920	BAM1	1.775070061	7.17572E-06	2.831203048	6.22519E-07	1.708777943	1.93061E-14	0.26703715	0.867112468	-0.214359021	0.551816216	0.063121099	0.761927143
AT3G07560	PEX13	1.768970974	2.03252E-08	1.337715565	2.01806E-20	1.263813649	1.12595E-22	0.435521038	0.731445776	0.051794674	0.896356173	-0.705842588	0.000285778
AT5G07920	DGK1	1.768809269	0.000111886	1.443791238	1.8311E-05	1.153788683	6.07609E-06	-0.456709934	0.486378875	-0.306410378	0.261253548	-0.461484513	0.005388746
AT5G11090	AT5G11090	1.768323416	1.19891E-07	1.71258318	7.59943E-15	1.075557413	1.34163E-14	0.639047039	0.637815859	-0.061769347	0.873441029	-0.913198314	4.45567E-12
AT1G03290	AT1G03290	1.760121076	1.88999E-06	1.834540082	6.17529E-20	1.979208953	5.27664E-31	0.885967307	0.574350959	0.234801904	0.462927792	-0.311603115	0.147319937
AT2G28570	AT2G28570	1.752099587	0.00019142	1.599765834	0.000271594	1.267261053	0.002589825	0.22799764	0.825486791	0.539777289	0.199396735	0.035802747	0.940783936
AT1G22640	MYB3	1.748549554	5.18364E-06	1.791222205	2.51028E-17	1.454215678	3.09369E-27	0.758959841	0.662219342	0.023402887	0.966196677	-0.252379187	0.32035885
AT5G66040	STR16	1.748176469	2.77803E-10	1.616962553	1.2134E-23	1.209369895	1.62301E-09	0.348674071	0.69623624	-0.32699638	0.078571119	-0.79927605	1.83383E-09
AT4G30550	GGP3	1.747621413	1.68661E-07	1.389472614	6.155E-16	1.544932946	1.9163E-27	0.972538281	0.437207517	0.235578785	0.431964416	0.101956289	0.639116582
AT1G65980	TPX1	1.74138312	1.75852E-13	1.636454152	1.70428E-15	1.373809003	9.3321E-30	0.064848784	0.963574073	0.194822825	0.467058937	-0.246664671	0.119514148
AT4G01000	AT4G01000	1.739735799	8.03016E-09	1.421398169	4.63121E-20	1.379156005	2.02709E-23	0.807737979	0.616300683	0.115204118	0.669273821	-0.471029579	0.022068191
AT4G29840	MTO2	1.73646741	2.27928E-11	1.164393323	1.6772E-10	1.451033722	3.89311E-28	0.31409947	0.709586436	0.284101519	0.211889393	-0.155325411	0.266453963
AT2G21240	BPC4	1.726385912	2.91534E-10	1.293286136	3.63167E-23	1.379001401	2.49833E-28	0.938847535	0.454333948	0.470843587	0.005266251	-0.084127461	0.701561709
AT4G38810	AT4G38810	1.710497714	3.07613E-08	1.863076375	1.23764E-19	1.584071232	1.09016E-24	1.195601903	0.473406779	-0.064677115	0.921453678	-0.68477289	7.69114E-05
AT1G50300	TAF15	1.707130186	0.000199034	1.29982892	7.35948E-05	1.142980705	2.1854E-09	0.457733003	0.723884274	0.352836951	0.374801421	-0.356188261	0.239864578
AT3G12490	CYSB	1.705802862	2.28552E-08	1.548629754	5.49122E-17	1.214522827	9.38451E-16	0.300199771	0.66524984	0.27063567	0.327045125	0.149826933	0.346696624
AT1G71730	AT1G71730	1.695177003	1.64956E-10	1.219812047	1.19411E-12	1.478724742	4.55438E-20	0.262927456	0.672844426	0.402830926	0.061893219	-0.042536766	0.853588587
AT2G22680	WAVH1	1.689285629	5.04541E-05	1.797034641	5.32813E-13	2.088338578	1.31185E-25	-0.346570182	0.696374342	-0.267783679	0.291736964	-0.50734143	0.002709413
AT1G52565	AT1G52565	1.689026572	0.03149068	3.55451642	5.48781E-11	2.625645831	2.77829E-05	1.078429083	0.536986632	0.739416916	0.453793716	0.513590322	0.145307043
AT1G80130	AT1G80130	1.683484604	0.000357841	4.030872626	4.48143E-44	2.292370729	9.9328E-14	0.038629736	0.983305599	0.052065165	0.951805765	-0.352295377	0.225808959
AT3G02990	HSFA1E	1.681412841	0.005199716	2.817079248	2.72178E-05	1.163952529	0.001149023	-0.887866902	0.228792905	-0.15923702	0.793709109	-0.289880428	0.229040945
AT4G32040	KNAT5	1.680431159	6.48568E-07	1.889045052	1.93923E-16	1.639673043	4.22868E-34	0.533327806	0.726900342	-0.090942694	0.856490679	-0.456142056	0.002025521
AT1G53580	GLY3	1.672522486	1.54366E-05	2.092023975	2.6436E-34	2.136265181	2.6659E-37	0.9538329	0.611226747	-0.084022387	0.866906715	-0.439370452	0.012001999
AT5G04750	AT5G04750	1.666593143	1.06273E-07	2.007898727	5.51527E-22	1.4954831	5.31812E-27	0.082821088	0.954232379	0.037775447	0.95555756	-0.616125341	0.008784916
AT3G61630	CRF6	1.666540266	5.47526E-08	1.219071734	1.57413E-05	1.929004924	1.71249E-34	0.496369544	0.598901706	0.024384631	0.97061131	-0.555687905	0.001931713
AT3G21600	AT3G21600	1.666028506	0.000240201	1.864972005	5.06482E-15	1.878248788	3.78916E-13	0.616342179	0.662900207	-0.172263061	0.772094392	-0.421495143	0.206519985
AT1G29330	ERD2	1.665944493	0.000302307	1.908696013	7.14346E-10	1.336360785	8.22472E-06	-0.02771748	0.98423633	-0.137298494	0.816953676	-0.071948874	0.760556638
AT4G37300	MEE59	1.658592554	2.34276E-05	1.498777399	7.25762E-23	1.63985247	1.86126E-39	1.26224046	0.374967877	0.028952764	0.94946668	0.260909552	0.049365129
AT4G02380	SAG21	1.657168935	6.11799E-05	2.257631567	3.81645E-06	2.470789339	6.32831E-64	0.265143859	0.808442563	0.8223708	0.08501642	-0.050218836	0.861492643
AT1G22930	AT1G22930	1.656809391	5.70345E-07	1.545995004	6.78068E-16	1.615392705	3.36365E-31	0.68829875	0.628007131	-0.022552309	0.958742629	-0.607688966	0.00066411
AT2G36460	FBA6	1.653334194	5.58353E-07	1.604110009	4.91625E-40	1.05328322	2.09817E-12	0.458387584	0.677952867	0.166915021	0.508159229	-0.172069557	0.262087327
AT5G49665	AT5G49665	1.643691556	9.25243E-08	1.576445899	5.32782E-06	1.245925053	1.08897E-07	-0.16322029	0.800300732	0.134375625	0.805320688	-0.421160096	0.001803559
AT4G16146	AT4G16146	1.643640754	0.015093145	2.673510217	4.86747E-19	2.07623151	1.96091E-08	0.770084666	0.640704059	0.141225416	0.883751355	-0.483887744	0.209862001
AT5G14780	FDH	1.638010001	2.33669E-08	2.254463766	3.10907E-25	1.100047609	2.93258E-14	0.437879204	0.663046749	-0.290165822	0.112283574	0.179791016	0.155249915
AT1G16240	SYP51	1.637682331	7.8104E-08	1.393825815	4.18153E-15	1.144854586	8.18697E-17	0.538797776	0.667070517	0.207110753	0.393308596	-0.316535018	0.023806743
AT3G26510	AT3G26510	1.634839336	0.000116939	1.882279004	8.809E-10	1.954943009	1.01611E-46	1.289426184	0.43724862	0.314065824	0.484106076	-0.643128432	0.000109499
AT2G01100	AT2G01100	1.633879694	2.97447E-10	1.069922274	9.78427E-14	1.58822495	2.87373E-32	0.50416141	0.695103554	0.272365822	0.211889393	-0.379106385	0.088629275
AT3G14660	CYP72A13	1.63139524	2.1201E-06	1.865678262	1.18485E-33	1.765352478	3.46413E-72	1.286705002	0.379538351	0.171507794	0.36930157	0.102185472	0.490396181
AT5G01350	AT5G01350	1.628767871	5.18995E-08	1.183023637	2.24637E-11	1.234497063	5.55515E-29	0.323395521	0.721140128	0.225271606	0.310704083	-0.26993241	0.063075367
AT2G34900	IMB1	1.628519043	1.16311E-10	1.474910231	6.63598E-12	1.476532038	2.73576E-25	0.487678589	0.618907586	0.646953764	0.000602496	-0.111311055	0.711588739
AT1G67300	AT1G67300	1.627046849	0.000138246	2.389439037	9.24388E-08	1.08194939	0.004790146	-0.253666658	0.796278925	0.317595688	0.486297808	-0.4880751	0.056947748
AT5G64230	AT5G64230	1.6262127	0.018043483	2.484071387	1.89532E-09	1.340965386	0.008465748	-0.505063739	0.820406788	-0.393636835	0.660210785	-0.8341212	0.015833713
AT4G32150	VAMP711	1.625489597	2.18485E-12	1.346998873	1.81104E-17	1.525613298	4.9624E-35	0.095126661	0.946164167	-0.253202419	0.141780121	-0.984197615	1.87807E-25
AT4G35300	TMT2	1.624366646	1.53501E-10	2.054264122	4.84894E-14	1.512582331	3.37267E-09	-0.600569512	0.196757929	-0.199597897	0.507709391	-0.736944959	0.000181267
AT2G47710	AT2G47710	1.621398697	3.97355E-08	1.719446989	6.023E-14	1.072421767	1.61874E-07	0.73492128	0.559275497	0.346254973	0.091710539	0.146680277	0.451468396
AT1G71080	AT1G71080	1.620450133	6.5007E-05	1.700923801	2.40814E-22	1.366794924	7.0368E-22	0.530600471	0.580627708	0.019865895	0.966453077	-0.575914739	0.000307769
AT3G20250	PUM5	1.615137951	9.57849E-09	1.800648627	4.04943E-14	1.556643007	2.00647E-27	-0.064286952	0.957410897	-0.249525548	0.366910832	-0.749359314	3.15603E-09
AT4G22920	NYE1	1.614415671	0.000290638	2.111014062	3.96083E-05	1.097852587	0.000429722	0.331423776	0.809893299	0.054201387	0.939595436	-0.617470249	0.014860133
AT1G55510	BCDH BETA1	1.612438343	3.2877E-05	1.605105882	5.67574E-15	2.170418436	1.57149E-37	1.464443107	0.474740724	0.267722478	0.520788216	0.202772343	0.281620971
AT3G21090	ABCG15	1.610468632	5.05239E-08	2.090851686	1.21275E-18	1.96333015	3.64602E-13	-0.049604605	0.972225981	0.097188904	0.831323166	-0.50716633	0.000211345
AT4G15690	AT4G15690	1.609837216	0.000439561	3.017076684	8.53667E-08	1.309366654	5.64026E-05	0.68694855	0.70850021	0.771631831	0.054272756	0.876514385	0.000459244
AT3G54950	pPLAIIIbeta	1.606447548	3.49729E-05	2.109454483	1.78797E-05	1.909778026	1.26061E-08	0.016630177	0.994162268	0.011015332	0.994163341	-0.654710993	0.005055691
AT1G23860	RSZ21	1.604328252	3.75837E-09	1.339133968	3.11766E-16	1.394308731	3.16451E-22	0.201749401	0.863039271	0.358171768	0.140308565	-0.23031509	0.087755899
AT3G20550	DDL	1.604245347	2.22726E-05	1.208175489	4.95492E-10	1.067859055	7.80961E-09	0.555249963	0.629389432	0.082309335	0.880732693	-0.317712487	0.225808959
AT4G12000	AT4G12000	1.600354863	0.011919362	2.738424892	7.79548E-08	1.260638478	0.000301176	-0.28701222	0.902675758	-0.158743703	0.900340026	-0.309193032	0.301253552
AT2G41200	AT2G41200	1.596698851	0.004352698	1.570002932	9.18821E-06	1.579289009	5.39091E-05	0.28146419	0.880294241	0.185867824	0.710593111	-0.49915468	0.055867691
AT3G17000	UBC32	1.595904076	2.59596E-06	1.556691421	4.31798E-10	1.28244892	6.0684E-14	0.379089464	0.760754635	-0.118181118	0.805320688	-0.377236328	0.076510929
AT1G55675	AT1G55675	1.594679775	1.3223E-05	1.110483114	7.41922E-06	1.011114663	0.001022718	0.649456554	0.636072176	0.456639627	0.217289538	-0.241058179	0.396611736

AT1G21410	SKP2A	1.592641518	0.000139053	3.371426406	1.66735E-33	1.73810365	4.84541E-17	0.761980239	0.647947756	0.492227368	0.195004065	-0.270694801	0.312302375
AT4G34860	A/N-InvB	1.59155281	4.94518E-11	2.267399673	1.76829E-17	1.893957803	1.22734E-09	0.168458086	0.865561711	0.397384126	0.093156434	0.055335673	0.814315114
AT2G26210	AT2G26210	1.591218806	6.05533E-07	1.350100455	6.1415E-17	1.555057144	6.12912E-18	0.686849696	0.575257546	0.24377436	0.299725468	-0.274636407	0.020669383
AT4G27130	AT4G27130	1.58059412	1.58972E-14	1.187449865	4.00805E-11	1.419041322	7.76682E-24	0.036352883	0.967291937	0.028994768	0.933194561	-0.332124411	0.032019992
AT1G32130	IWS1	1.569886602	2.40868E-07	1.234300633	7.95947E-18	1.274269576	1.62966E-24	0.566046074	0.685495817	-0.052728127	0.888681236	-0.537349462	9.61413E-05
AT5G01670	AT5G01670	1.567489468	8.90298E-07	1.757238164	1.04562E-09	1.263514748	1.52353E-05	0.639483721	0.644839236	0.225123845	0.612690792	-0.454054136	0.005848407
AT4G21320	HSA32	1.565239305	0.000472786	2.030645291	1.82582E-08	1.935023846	1.14514E-13	0.128814388	0.938741823	0.290726753	0.615469901	-0.177435282	0.571812536
AT5G48180	NSP5	1.564674971	2.01382E-06	1.116503847	3.55311E-06	1.011739326	2.2835E-06	0.048340479	0.984889186	0.344248974	0.161363536	-0.908847407	0.09787353
AT2G01490	PAHX	1.546069828	2.24078E-07	1.931963296	1.15179E-21	1.201589509	5.5092E-24	0.514623261	0.657369817	-0.02163648	0.957019716	-0.187878623	0.158114372
AT4G35550	WOX13	1.544568189	0.000416499	1.186502915	0.015103072	1.847529699	2.57666E-20	0.5281988	0.715099832	-0.012902806	0.989242512	-0.36217682	0.107674795
AT3G54680	AT3G54680	1.544243333	0.00052268	2.127956765	1.36474E-13	1.684474804	9.67431E-13	-0.280514711	0.697407195	-0.430749948	0.108412471	-0.673809968	9.28232E-06
AT4G32910	AT4G32910	1.541648222	1.39936E-09	1.34741632	1.342E-09	1.255062919	9.35468E-16	-0.065991546	0.961358666	0.341948162	0.041856703	-0.109693653	0.567773503
AT1G75170	AT1G75170	1.541100326	0.000103983	1.83509867	5.0346E-06	1.559156502	3.73943E-05	-0.114124463	0.927465214	-0.107071411	0.835618895	0.12771681	0.546016863
AT3G14690	CYP72A15	1.539509248	9.06056E-05	1.75119976	9.13858E-33	1.614344851	5.06849E-40	1.159423683	0.536986632	-0.089174781	0.851585386	-0.478027159	0.000329632
AT1G02305	AT1G02305	1.537525966	2.04481E-07	1.284120529	3.99043E-10	1.180628044	6.72117E-14	0.300027046	0.816625165	-0.193158674	0.510789648	-0.575720062	2.13794E-08
AT3G21870	CYCP2;1	1.533337375	0.000486879	1.938972347	4.24059E-10	1.905492646	1.74157E-45	2.716311203	0.144925486	0.083113922	0.95946592	-0.361872595	0.163651506
AT4G24690	NBR1	1.530120356	7.50243E-10	1.276239995	1.50453E-34	1.331466205	1.35038E-18	0.47412521	0.666277279	-0.095332509	0.709607102	-0.360826741	0.001876979
AT5G10300	MES5	1.523687609	0.001467324	3.033028794	4.69796E-06	1.381690039	0.006524515	-1.006274515	0.611039849	-0.588429556	0.279605916	-0.864267638	1.19669E-05
AT5G54130	AT5G54130	1.517884535	9.69808E-05	1.321986899	0.000434029	1.223384508	3.66158E-05	0.394231042	0.704225272	-0.164215135	0.786263879	-0.180404853	0.491183478
AT5G16970	AER	1.516874716	0.000116903	1.83321882	4.59284E-14	1.449966634	6.0807E-49	1.25642366	0.413284704	0.557492396	0.008165642	-0.192061678	0.350260246
AT3G12300	BUG22	1.515644955	1.56912E-05	1.599139457	3.03087E-05	1.040643419	4.50914E-06	0.867023784	0.511010999	0.572317753	0.048901218	0.158277421	0.47457662
AT3G19910	AT3G19910	1.514729805	3.06631E-08	1.18377901	1.15101E-08	1.41759481	6.95621E-22	0.649866684	0.568467773	0.401619468	0.060895008	0.079093185	0.70969945
AT4G02760	AT4G02760	1.510307065	0.005698906	1.325996215	0.000626294	1.073532176	0.01824411	0.459596324	0.662219342	-0.273652585	0.641639	0.077656715	0.819675079
AT5G20260	AT5G20260	1.504752585	0.016326401	1.058618039	0.038299696	1.395709272	5.61175E-05	0.38813852	0.926511921	0.081807935	0.968338614	0.726963664	0.080278636
AT4G21580	AT4G21580	1.503074766	4.03141E-06	1.7829328	5.88377E-22	1.081373679	3.44647E-07	0.310960977	0.784493718	-0.054548671	0.921538071	-0.36343864	0.063247925
AT3G16910	AAE7	1.500377843	1.53801E-07	1.80246124	5.78095E-11	1.029465163	1.13812E-07	0.132906825	0.937367978	-0.32658968	0.202269101	-0.360635792	0.00924947
AT5G59570	BOA	1.499832917	0.000804302	1.290674345	0.004946621	1.039542203	0.007435446	-0.870260268	0.437721723	0.178908976	0.734451454	0.074888902	0.761927143
AT3G11830	AT3G11830	1.494641702	2.60403E-14	1.175984171	6.56913E-10	1.268712007	5.83958E-25	-0.118196155	0.820789421	0.155621709	0.493875814	-0.021283985	0.896947242
AT4G28830	AT4G28830	1.493962291	0.00057888	1.706137074	1.1167E-09	1.134717106	2.13584E-09	0.481279969	0.602119156	0.40962038	0.195419415	0.152733773	0.366416733
AT3G07780	OBE1	1.490931608	1.58966E-05	1.03452884	6.18147E-19	1.238397584	3.06123E-26	0.910299244	0.611321113	-0.067166117	0.846818973	-0.39336154	0.119903629
AT5G66460	MAN7	1.489021184	0.000824822	1.268119677	7.98449E-05	1.133547811	1.26021E-05	0.264992768	0.841135253	-0.464812314	0.20936192	0.122006198	0.582597399
AT3G02340	AT3G02340	1.484811433	0.0005233	1.174100494	7.12046E-06	1.115875696	1.27561E-09	0.852884719	0.613859108	-0.006308636	0.993586481	-0.118412653	0.684092236
AT3G48990	AAE3	1.484458473	5.22074E-06	2.219970554	1.47124E-55	2.057265814	1.21428E-60	0.772405741	0.602119156	-0.138216957	0.641639	-0.357218396	0.001109697
AT1G59640	BPEp	1.483382988	0.03236735	2.193789763	0.000665354	1.826770199	4.88491E-05	-0.419801732	0.798808149	0.544970041	0.497254714	-0.031127471	0.937881422
AT1G19050	ARR7	1.482297777	0.000262929	1.035077559	0.000105863	2.405677568	1.33247E-34	0.237837256	0.793776337	0.003121583	0.997020455	0.026228854	0.922840403
AT1G15110	PSS1	1.481893588	2.14761E-06	1.167140844	5.40189E-05	1.650228638	1.08555E-09	0.094929849	0.955404265	-0.15988599	0.778222196	-0.512852859	0.00211636
AT5G17760	AT5G17760	1.481879786	0.002225178	1.848902086	0.000200261	1.982638901	5.64954E-08	-0.970673153	0.598901706	-0.150613762	0.768442912	-0.11891537	0.653680913
AT1G06180	MYB13	1.479658084	0.000262115	2.14285311	2.28744E-09	2.369002678	2.04928E-13	1.08507763	0.058170834	0.716821999	0.067910142	0.698824794	0.00010928
AT5G51040	SDHAF2	1.471587487	0.000197371	1.860400095	4.00229E-15	1.313853936	1.629E-13	0.163209303	0.911955093	0.172761571	0.662851223	-0.03880643	0.871619987
AT1G15400	AT1G15400	1.471210569	2.73471E-07	1.156353411	3.27907E-10	1.264187903	3.4807E-23	0.133357772	0.898305058	-0.040245322	0.919720416	-0.947454521	5.95306E-10
AT2G38640	AT2G38640	1.463316652	0.006227458	1.854449594	9.73931E-06	1.324754019	1.76357E-05	0.251275897	0.885120518	-0.430197636	0.567682504	-0.513030975	0.180258245
AT1G21780	AT1G21780	1.46185913	6.35971E-05	1.927546431	1.23179E-31	1.65048766	5.01043E-31	0.539854591	0.705044136	-0.015086051	0.981554934	-0.380501859	0.038616358
AT3G09850	AT3G09850	1.461042684	1.81589E-05	1.364760647	1.68906E-13	1.311931199	1.18876E-13	0.737709686	0.619569853	0.279535276	0.298368981	-0.403298212	0.013433968
AT1G78670	GGH3	1.459845762	8.15444E-06	1.673866169	8.30268E-16	1.183697964	1.77057E-11	0.52156081	0.647947756	-0.26294965	0.251428363	-0.58302501	3.16856E-07
AT5G25770	AT5G25770	1.457354127	1.39104E-06	1.147070662	0.004045021	1.362257507	2.87071E-05	-0.260448108	0.784493718	-0.089382135	0.862129805	-0.94204283	1.45823E-05
AT4G32920	AT4G32920	1.454478109	7.50847E-07	1.890166333	2.0045E-06	1.531635828	6.2575E-05	-0.585457508	0.66524984	-0.185940317	0.657855928	-0.244238444	0.201659274
AT2G29450	GSTU5	1.451419399	0.000227742	2.520699708	1.32203E-15	1.091454302	2.26E-05	0.06393463	0.980596414	-0.022444074	0.97061131	-0.432910165	0.008967652
AT1G33055	AT1G33055	1.449274748	0.004023149	1.757795949	1.336E-06	1.431250686	2.97641E-05	0.354862108	0.763069171	0.010970113	0.992427854	-0.246275208	0.492460069
AT4G16790	AT4G16790	1.448664017	0.000823319	1.176563283	0.000224973	1.573540896	4.69997E-10	-0.310948501	0.774577099	-0.240804501	0.696090718	-0.856775587	2.54288E-06
AT5G18110	NCBP	1.446445189	1.14148E-08	1.323734907	4.79277E-17	1.366768116	1.98822E-24	0.853593583	0.465456099	0.237853908	0.258496511	0.090121186	0.530512017
AT2G26430	RCY1	1.442762437	2.06102E-06	1.372734335	3.17805E-11	1.585017818	1.52412E-26	0.703891792	0.6208322	0.111022015	0.717765433	-0.104571436	0.559917015
AT1G60170	emb1220	1.441277608	3.14815E-05	1.22067617	1.09658E-12	1.319254258	9.62521E-19	0.680420105	0.534532914	0.174496434	0.470740576	-0.050782609	0.791640653
AT5G66180	AT5G66180	1.436748974	6.54821E-08	1.167856174	6.75928E-07	1.199036462	1.08942E-10	0.344813435	0.726900342	0.026419929	0.963954074	0.037370231	0.867604777
AT3G13224	AT3G13224	1.42962553	0.000177249	1.545414675	1.11094E-05	1.638262533	2.85002E-18	0.208110523	0.90670454	0.265448477	0.473873568	-0.338104621	0.08942883
AT5G35330	MBD02	1.427546615	6.64096E-06	1.1594038	1.88173E-11	1.155947006	2.11239E-17	0.650000008	0.533068231	-0.009058023	0.987053629	0.126164632	0.435079399
AT2G39980	AT2G39980	1.424959458	0.000237924	1.925196748	1.13058E-30	1.850724119	5.94977E-38	1.473949697	0.477936046	0.193878378	0.732125294	0.567589871	3.50267E-05
AT1G32790	CID11	1.424798202	9.47371E-07	1.092155167	5.69645E-22	1.161638352	6.65087E-19	0.744360653	0.596785539	0.221769339	0.269856688	0.088013999	0.542140764
AT3G22440	AT3G22440	1.419757437	2.29369E-13	1.241006701	2.11496E-15	1.15625633	4.82764E-27	-0.041090834	0.975645787	-0.152609726	0.441613827	-0.427535367	8.272E-06
AT5G66440	AT5G66440	1.419381924	0.002319318	1.235326883	1.56346E-05	1.559232134	5.6785E-10	0.459324416	0.685495817	0.110508688	0.894924841	-0.113462956	0.737031037
AT3G06290	SAC3B	1.418316499	4.054E-06	1.259218798	8.7658E-05	1.728472775	4.00284E-30	0.472027521	0.684184988	-0.04703537	0.929192932	-0.265891562	0.111166454
AT3G03310	LCAT3	1.411958225	2.82151E-07	1.508572563	2.0825E-05	1.554807456	5.62562E-11	0.154220137	0.851334862	-0.096619177	0.817392602	-0.348125195	0.152038413
AT5G29000	PHL1	1.411446779	0.001298964	1.914275794	2.09975E-10	1.095427317	0.002788083	0.112882001	0.929026631	0.030985706	0.964180223	-0.336611157	0.051255117
AT4G15260	AT4G15260	1.401565092	0.00040116	1.408281542	4.90731E-10	1.397420285	2.70964E-17	0.459664665	0.735833376	0.037331039	0.954684273	-0.758222932	0.000362932
AT1G56090	AT1G56090	1.397064133	3.1858E-05	1.137798985	0.000117293	1.014579385	0.000102608	0.635953652	0.632278424	0.316711777	0.521171371	-0.48556485	0.01713858
AT5G06450	AT5G06450	1.394559454	0.0042018	1.269592094	4.49478E-05	1.068955028	1.9495E-07	0.336986526	0.731041294	0.510760243	0.120900727	-0.086176124	0.736354335
AT5G63190	AT5G63190	1.393588765	1.06749E-05	1.427302428	1.30043E-11	1.495326365	1.26363E-18	0.30948658	0.786782066	-0.143932743	0.656106268	-0.792569051	2.20711E-08
AT5G06360	AT5G06360	1.392466485	8.31779E-11	1.137297205	7.29872E-18	1.245358913	1.28518E-22	0.423414982	0.587695712	0.223655423	0.1393487	0.178181662	0.279841313
AT1G35660	AT1G35660	1.388153638	0.000468172	1.219475158	1.94512E-06	1.498318377	1.23535E-13	0.41371873	0.7370188	0.281501442	0.529833936	-0.131602607	0.60285828
AT2G24100	ASG1	1.388067812	8.64353E-05	1.431581777	5.68151E-08	1.59465629	9.45442E-25	0.538943341	0.604422229	0.176631193	0.596685937	0.132008051	0.415995807
AT4G26060	AT4G26060	1.387143278	0.000444342	1.570368358	0.000152136	1.087338465	0.004189752	0.080457061	0.963509602	-0.286916324	0.646618265	-0.851727209	0.003390978
AT3G26040	AT3G26040	1.384551457	0.009643383	2.145558103	5.35441E-05	1.373211754	0.000562717	0.418447935	0.82814234	-0.016511287	0.992611192	0.024048609	0.963938415
AT4G27450	AT4G27450	1.383872599	5.55097E-06	1.750399336	1.42669E-23	1.602697256	9.93095E-54	1.006209853	0.431282679	0.433247423	0.034660889	0.465167664	0.000418992
AT5G16880	AT5G16880	1.381886769	1.63137E-06	1.343333264	1.1738E-22	1.471631246	2.50026E-24	0.442406626	0.697744369	-0.087719713	0.728724954	-0.395610318	0.0008994
AT5G56140	AT5G56140	1.379377521	2.50776E-07	1.050800238	2.68806E-17	1.355658491	3.15693E-23	0.39200218	0.76277889	-0.114920225	0.614818375	-0.503436393	0.002085359
AT1G07350	SR45a	1.379058038	7.56629E-07	2.345753043	4.97568E-26	2.163864473	7.13334E-49	0.009199755	0.994162268	0.257976151	0.289824876	0.143957136	0.301253552
AT1G66580	SAG24	1.377104511	1.68287E-08	1.106055399	6.09898E-15	1.59436977	1.08258E-45	0.23419654	0.692385923	0.532441676	4.82388E-06	0.207760994	0.102398496
AT5G22000	RHF2A	1.376081637	4.94333E-07	1.487865285	3.18274E-24	1.318965771	1.10394E-20	0.376952638	0.691895802	0.129252626	0.576242501	-0.575452269	0.000439023
AT3G54850	PUB14	1.371422215	1.12858E-05	1.151488501	9.35992E-07	1.290813826	1.29115E-11	0.427130684	0.753311426	0.012323757	0.985585094	-0.26057642	0.287397439
AT5G25280	AT5G25280	1.371421752	2.38444E-07	1.607069785	7.47376E-10	1.349726684	1.10466E-40	0.503879101	0.691895802	0.210591264	0.234902133	-0.432062963	8.0519E-05
AT3G44310	NIT1	1.367600473	2.88151E-12	2.403845728	2.71376E-90	1.307409515	1.17171E-23	0.253391643	0.732322487	0.136962006	0.663410884	0.044166221	0.768357562
AT5G50320	ELO3	1.363688162	3.13602E-08	1.180516571	3.51544E-07	1.04965223	4.85959E-18	0.33684712	0.764845027	0.342479019	0.136053891	0.005600486	0.97989514
AT1G33080	AT1G33080	1.363339967	0.01752694	1.597876507	2.27303E-06	1.222457321	6.00854E-06	1.078631781	0.585524552	0.314913739	0.589186505	0.164300022	0.712923435
AT1G75400	AT1G75400	1.359972322	9.04241E-07	1.701276452	1.04976E-12	1.332524291	9.35128E-10	-0.12656784	0.936848796	0.039677814	0.947435313	-0.421209516	0.002743261
AT4G19390	AT4G19390	1.35820937	9.76597E-06	1.385003154	1.9946E-05	1.17371892	0.000157801	0.070081212	0.978043477	0.044579204	0.950790869	-0.581377112	0.032216278
AT5G58350	WNK4	1.356396483	0.000181772	1.711305546	3.56269E-10	1.874655232	8.48904E-32	0.358101145	0.751883669	-0.437140017	0.151525675	0.019568616	0.960633256
AT4G30470	AT4G30470	1.351509746	3.80482E-05	2.833921326	2.91779E-49	1.909932065	7.40516E-09	-0.275611984	0.68789331	-0.374118825	0.130444887	-0.386922529	0.037995899
AT5G03490	AT5G03490	1.34974065	0.001162046	2.117104907	4.95595E-09	1.032053351	0.00330154	0.454727632	0.731041294	-0.053907557	0.961537869	-0.834909112	0.008475506
AT1G52710	AT1G52710	1.345382714	0.000141215	1.53827153	2.0809E-07	1.594110613	6.28711E-10	0.289933568	0.753910599	0.343887219	0.258282444	-0.238919409	0.471676199
AT1G15350	AT1G15350	1.34497633	1.0928E-07	1.207731783	5.64128E-14	1.447693514	3.83309E-20	-0.067802321	0.931240576	-0.075596379	0.846389857	-0.327482303	0.006632333
AT5G60360	ALP	1.344058495	1.43845E-05	1.999219296	8.87456E-21	1.161010346	1.34279E-12	0.413553267	0.705693673	-0.185140399	0.437782126	-0.789074871	2.14054E-07
AT5G65280	GCL1	1.340887711	0.000201831	2.048090477	4.24325E-05	1.643463309	4.6193E-06	0.003833431	0.997835552	-0.220746956	0.78917956	-0.696733017	0.013642489
AT3G53180	NodGS	1.338305645	6.88923E-05	2.111786543	5.19587E-13	2.083100258	5.77786E-33	0.279914632	0.809502311	0.093923195	0.811087455	-0.097166805	0.620437128
AT3G63260	ATMRK1	1.337769195	1.29489E-05	1.196975664	1.75222E-07	1.3793025	3.57359E-28	0.148613462	0.916159273	-0.196017176	0.501605226	-0.634473353	3.9254E-07
AT2G35680	AT2G35680	1.333209269	0.001376479	1.137722461	2.84868E-09	1.331512612	1.27701E-22	0.574397364	0.637815859	0.032976145	0.946325579	-0.242415545	0.191898471

AT2G46260	LRB1	1.332100592	6.52447E-08	1.231413171	1.60315E-11	1.280432535	1.35857E-20	0.485389508	0.702246294	0.074604642	0.793709109	-0.168530154	0.243277413
AT3G12400	ELC	1.327329133	7.98453E-05	1.493902568	7.9733E-17	1.533644285	1.82826E-32	0.933421017	0.604422229	-0.332018892	0.138192411	-0.238068156	0.088784298
AT3G56310	AT3G56310	1.325462025	1.67582E-09	1.759623548	5.15668E-14	1.292248421	4.90361E-17	0.025840833	0.988471189	-0.292701165	0.145902074	-0.644568551	2.85365E-06
AT1G45150	AT1G45150	1.324042088	0.002464921	1.071609532	1.79601E-06	1.450424786	8.16658E-08	0.769020203	0.607986058	0.319514008	0.490474456	-0.256153508	0.448863807
AT1G06265	AT1G06265	1.321368786	0.03091173	1.127825785	0.010074012	1.16695536	0.011620175	1.586453348	0.281687194	0.592452898	0.323397243	0.103612441	0.80413053
AT1G21450	SCL1	1.316357359	5.23389E-05	1.154518374	1.60595E-13	1.030404114	3.13046E-12	0.443001435	0.786973304	-0.182107934	0.564980934	-0.508248681	5.50313E-05
AT4G25500	RS40	1.315917417	3.71437E-05	1.042563042	3.54695E-18	1.12635944	1.71206E-11	0.527085605	0.676109289	0.108667378	0.666368662	-0.137305841	0.450529821
AT5G18040	AT5G18040	1.314156332	0.008235032	2.226885877	8.14155E-08	2.285090603	9.03717E-16	0.330232059	0.849668819	-0.012967081	0.992180597	-0.495318152	0.171041725
AT3G62860	AT3G62860	1.314123643	0.001395474	1.35982998	7.08545E-09	2.084144292	1.83628E-59	0.042267777	0.9905016	-0.175720535	0.659063827	-0.040782614	0.837784722
AT4G16150	AT4G16150	1.313313382	1.59154E-06	1.292784473	1.15777E-21	1.159638525	9.06837E-12	0.624181016	0.649556345	-0.191653322	0.374913973	-0.302729116	0.03183855
AT5G40720	AT5G40720	1.311965252	0.022580923	1.152644249	0.001319016	1.112065375	0.000214616	0.756600946	0.602119156	0.214023174	0.664790835	-0.438485672	0.065917929
AT3G25910	AT3G25910	1.308969263	4.20514E-07	1.096442556	4.89263E-11	1.092143736	3.52869E-11	0.285193123	0.697407195	0.234400939	0.227994936	-0.306554225	0.036939676
AT1G03080	NET1D	1.304361974	5.73014E-07	1.209878923	9.42833E-10	1.308141877	1.69905E-13	0.237541691	0.835120667	-0.093255968	0.801085181	-0.545448582	1.48623E-05
AT5G57655	AT5G57655	1.303400842	0.000147264	1.036672456	1.23233E-07	1.628442309	2.47202E-17	1.005819519	0.534010844	0.06996538	0.789622862	-0.110618681	0.466880405
AT4G15680	AT4G15680	1.303232739	0.010242859	2.904635193	1.06383E-07	1.543648369	1.19138E-06	0.164045977	0.964544073	0.15813533	0.869002246	0.287398573	0.436122744
AT1G23440	AT1G23440	1.293545095	0.000206337	1.298773269	1.47672E-06	1.160150679	5.52906E-10	0.041586888	0.977477438	0.182999922	0.656025221	-0.75810757	1.39638E-05
AT5G45360	AT5G45360	1.293276725	2.95763E-05	1.158813527	5.67015E-12	1.300756906	3.46984E-17	0.687651018	0.590549357	0.264725937	0.254066296	-0.206519439	0.125104077
AT1G60140	TPS10	1.292593698	0.001602596	1.467161374	5.57329E-14	1.469440016	4.49228E-16	0.426551788	0.721140128	-0.015272955	0.971701608	-0.473752464	0.001478875
AT3G22460	OASA2	1.292246375	0.003803187	1.909367432	6.68163E-07	1.954834236	1.03787E-15	-0.30129336	0.789887378	-0.139696844	0.875452188	-0.654274498	0.021720406
AT2G31280	CPUORF7	1.289203182	6.07816E-08	1.067394874	1.93208E-05	1.101314366	2.86178E-10	0.214686548	0.832109912	-0.02421033	0.964601576	-0.699613708	5.60964E-08
AT4G19230	CYP707A1	1.285052289	0.003480693	2.358840872	3.21245E-11	2.677602113	1.16296E-18	-0.900199094	0.253109653	-0.459330569	0.400769075	-0.39096028	0.070868299
AT1G59700	GSTU16	1.283189263	0.013851552	1.663993445	2.98073E-18	1.850913073	1.65559E-30	0.658078798	0.639605292	0.554265307	0.071991501	-0.144508199	0.553651462
AT4G19700	RING	1.279846847	0.01246395	1.626925115	4.40697E-09	1.123518291	5.80429E-07	-0.08983211	0.93664894	0.291197123	0.398388739	-0.185708664	0.326567001
AT4G34138	UGT73B1	1.270557573	0.001774834	1.285512838	1.05716E-12	1.854146288	2.00459E-63	1.856891518	0.1682865	0.279422451	0.307645689	0.356189714	0.016949833
AT3G62330	AT3G62330	1.266891734	0.000105526	1.202508754	6.28957E-08	1.25592309	1.50237E-06	0.444720696	0.743698216	0.268528925	0.367749288	-0.020760657	0.929072622
AT3G53420	PIP2A	1.26558581	0.001522227	1.79391945	2.03629E-16	1.298385634	3.58197E-25	0.409618647	0.770575751	-0.586845265	9.14114E-06	-0.309665967	0.003485665
AT4G28390	AAC3	1.263837963	0.016565942	1.6647734	1.43424E-05	1.242971174	0.000274539	0.072228032	0.953801758	0.266244173	0.680252138	-0.07155281	0.764383302
AT1G65660	SMP1	1.260601724	4.97168E-06	1.154794574	1.64141E-11	1.316448925	8.40622E-26	0.904514239	0.420106526	0.202167485	0.530145612	-0.103268021	0.587536917
AT1G74310	HSP101	1.258141899	0.00744213	1.609471213	0.000897531	1.120401298	0.016931479	-0.265152239	0.909900566	-0.181124068	0.689528364	-0.258289368	0.241406197
AT4G38440	IYO	1.252385233	9.6939E-07	1.069972919	7.0792E-06	1.580499766	2.49445E-17	0.512247952	0.665778009	0.079674379	0.875421612	-0.170194732	0.424255611
AT4G01410	AT4G01410	1.252115559	0.006554944	1.385622893	5.36642E-06	1.863632159	5.5596E-13	0.119928532	0.907260297	-0.042962644	0.936190346	-0.094607528	0.668453445
AT1G04300	AT1G04300	1.24494371	7.84698E-05	1.196332485	1.00621E-14	1.545624995	3.65335E-45	0.693967583	0.665778009	-0.087128256	0.788926828	-0.344483309	0.040761704
AT5G58575	AT5G58575	1.238637372	7.89898E-05	1.133656821	0.000172854	1.423395214	3.29737E-15	0.41665021	0.685495817	0.189929475	0.582043304	-0.329556732	0.009151291
AT1G20620	CAT3	1.237161619	3.54175E-09	1.194535526	2.35642E-35	1.124816894	2.16984E-09	0.137235729	0.899694612	-0.300290543	0.042444463	0.029714073	0.893737286
AT1G27980	DPL1	1.237138734	0.004245478	1.345117619	1.62716E-05	1.918258045	4.98116E-21	0.426947513	0.691824202	0.045102672	0.924543846	-0.064410813	0.790404189
AT5G47180	AT5G47180	1.237053272	0.00017749	1.279835821	4.51374E-12	1.387405494	1.07939E-16	0.160771291	0.908477916	-0.194547209	0.622827463	-0.623247818	1.45444E-07
AT5G40390	SIP1	1.231755586	0.001284349	2.309637993	3.24204E-08	1.474161992	7.68955E-10	-1.060093635	0.48633216	-0.569681028	0.01863789	-0.284545092	0.117726764
AT3G13200	EMB2769	1.23015366	8.31287E-06	1.193632627	3.24675E-11	1.227904399	7.04106E-24	0.348962967	0.692108738	0.222892554	0.308281389	-0.195902757	0.35514748
AT5G16370	AAE5	1.229061204	0.002424273	1.192771736	5.37099E-10	1.443413565	8.49621E-44	1.880318489	0.218297828	0.09405024	0.792965164	-0.591765077	0.00029246
AT5G20830	SUS1	1.227845526	0.003459185	1.774044618	7.61212E-06	1.197952734	2.50052E-05	-0.467425204	0.818940427	-0.20017402	0.675290032	-0.457146312	0.017101072
AT3G15780	AT3G15780	1.227748503	0.013438451	1.877646456	2.47353E-07	1.076110236	2.2926E-05	1.089605917	0.66524984	0.449860881	0.58050477	-0.786852493	0.002190226
AT2G26690	AT2G26690	1.217137445	0.000116939	1.875876712	1.64332E-22	1.301781843	5.79327E-17	-0.45438356	0.413866874	-0.363786517	0.105435145	-0.139991788	0.300340911
AT3G23560	ALF5	1.215698969	2.67488E-06	1.418122235	0.000275186	2.162088674	9.72814E-53	-0.101975546	0.914733185	0.424581621	0.155596055	0.785056748	3.61776E-08
AT1G55310	SCL33	1.213982331	2.10931E-06	1.380602286	8.45724E-11	1.640532643	3.17213E-33	0.898081708	0.332045541	0.304285842	0.218083341	0.304122939	0.09281456
AT2G46830	CCA1	1.207641617	0.016805953	2.253963404	1.34995E-11	2.080850538	4.83782E-06	0.646623125	0.604422229	0.150232044	0.632768672	-0.189959034	0.189081775
AT4G33540	AT4G33540	1.207620948	5.25031E-05	2.424674596	1.63204E-24	1.674546304	1.3735E-10	0.583523089	0.575462403	0.259150738	0.500045281	0.3107147	0.056641618
AT1G13190	AT1G13190	1.204258604	0.002912245	1.223258841	1.90139E-13	1.060494964	1.33268E-11	0.469402552	0.703109879	0.012741736	0.982729789	-0.490730061	0.010201319
AT2G25964	AT2G25964	1.204024004	0.004114528	1.250645247	0.000685894	1.444825174	4.93965E-28	-1.323398963	0.060737207	-0.047078946	0.936116842	-0.623354326	4.43195E-07
AT5G03560	AT5G03560	1.203278432	0.000857797	1.565260746	1.97348E-09	1.39326074	5.6972E-09	0.185536845	0.875058989	0.323828014	0.27174856	-0.604693437	0.000922162
AT5G03340	AtCDC48C	1.201524768	0.000165985	1.142714391	5.61236E-10	1.150940371	7.64925E-12	0.37596733	0.790596125	-0.247674937	0.456284977	0.001074793	0.995816011
AT3G51840	ACX4	1.200383297	0.001269562	1.066418456	1.76027E-13	1.239389989	1.0986E-27	1.272540479	0.394836051	0.149881262	0.498449509	-0.163186823	0.266519555
AT5G15260	AT5G15260	1.199060557	0.022994983	1.120909504	0.001504625	1.036235786	1.13152E-05	0.277887691	0.870511882	0.494493994	0.351079807	-0.301099038	0.165192298
AT1G15800	AT1G15800	1.193128737	0.005391221	1.017427145	0.000171251	1.088697709	1.33389E-06	0.146149239	0.92489468	0.219522283	0.59029983	-0.106319934	0.674766726
AT5G05480	AT5G05480	1.189872405	0.000492968	1.368252383	7.59785E-11	1.244823524	1.77962E-07	0.338433997	0.791199667	-0.045845771	0.950034363	-0.353285173	0.020199988
AT3G58640	AT3G58640	1.189404485	0.000713759	1.319135413	2.93957E-20	1.384008427	6.24309E-22	0.660221794	0.586846304	0.153971549	0.525334184	-0.087284175	0.707309909
AT4G24220	VEP1	1.185459589	2.08082E-06	1.325819186	3.77008E-20	1.126379625	9.62858E-14	0.267447057	0.827716472	-0.002962883	0.99453777	-0.762100397	8.92546E-09
AT1G33700	AT1G33700	1.182369909	0.006772724	1.873763673	1.14472E-07	1.554711212	5.05544E-12	-0.211696055	0.883898287	-0.331542658	0.271606183	-0.897748962	2.52821E-06
AT3G07350	F21O3.6	1.180742454	0.001585442	2.303268639	1.35177E-14	1.3729819	2.87206E-25	1.019571772	0.566699047	0.505845194	0.11791682	0.12193147	0.567172849
AT1G13710	CYP78A5	1.179928917	1.56912E-05	1.221111815	0.000864448	1.058969349	0.000539871	0.158814176	0.770543849	-0.185199418	0.595839201	-0.239885911	0.128710897
AT3G17810	PYD1	1.172048425	5.73257E-05	2.010548937	2.74877E-17	1.150081102	1.34344E-16	0.1287397	0.892672223	-0.095558521	0.805285979	0.070013225	0.714909394
AT5G47860	AT5G47860	1.167933713	0.00107112	1.743868006	1.59188E-13	1.552707262	4.72737E-21	0.524199297	0.697407195	0.262611665	0.503946392	-0.309539287	0.032102306
AT5G11390	WIT1	1.163754239	0.000433489	1.340835645	6.76661E-06	1.084475529	1.73197E-09	0.131085304	0.921176923	0.178271752	0.621230833	-0.188030793	0.350167837
AT1G09430	ACLA-3	1.159950126	0.000487887	1.131138876	4.50386E-18	1.314841691	1.87495E-13	1.067814581	0.319520931	0.179060129	0.418093903	0.059179323	0.751157972
AT1G50480	THFS	1.158626117	1.25027E-06	1.175741778	5.21031E-24	1.079551782	4.83709E-18	0.463834602	0.5728621	-0.006476261	0.989242512	-0.095722245	0.504525717
AT3G14595	AT3G14595	1.158068111	0.001591924	1.757547353	9.47654E-11	1.089310641	4.03204E-07	0.599459756	0.645642919	0.021167645	0.973216634	-0.582770091	0.002111724
AT3G48690	CXE12	1.15683129	0.000757589	1.609851638	3.37367E-15	1.195987402	2.35964E-19	0.69652287	0.674100217	0.073529334	0.845936736	-0.401129379	0.009954633
AT1G34370	STOP1	1.156659326	0.002639814	1.42400987	1.90613E-38	1.348904845	1.7934E-23	1.146467336	0.458341345	0.01718523	0.969038524	-0.062304442	0.754176524
AT4G10040	CYTC-2	1.154303163	0.001058344	2.18756461	9.28086E-11	1.331797856	0.001624777	-0.900500939	0.465456099	0.147304832	0.831317898	-0.491979859	0.071153684
AT5G03180	AT5G03180	1.148770392	0.004348787	2.05877082	5.6718E-08	2.32293674	9.17966E-27	-0.24645486	0.924835736	0.014966124	0.987936516	-0.231098135	0.424035322
AT1G50260	NTMC2T5.1	1.148359572	0.016081677	1.9891183	1.13777E-07	1.280447203	0.000413925	0.087283172	0.958628776	0.542263457	0.263073498	-0.369279131	0.177734883
AT5G05930	GC1	1.144804403	0.001564877	1.464394955	2.12892E-12	1.448800749	7.20155E-18	1.00658374	0.587695712	-0.011770347	0.986818647	-0.266942623	0.127463479
AT2G31350	GLX2-5	1.144307297	6.42398E-05	1.412747123	4.37858E-12	1.460173404	3.16082E-27	-0.129869509	0.92868457	-0.122063517	0.694108459	-0.836563031	8.7903E-13
AT5G27600	LACS7	1.142097005	0.001353561	1.268301551	6.97773E-06	1.026937894	7.56248E-07	-0.0905454	0.908591052	0.248295175	0.398166246	0.368442928	0.007644247
AT1G28260	AT1G28260	1.141857961	0.007253088	1.420816037	2.08808E-19	1.145815259	1.91107E-14	1.073968431	0.563804992	-0.330984257	0.25395818	-0.16606739	0.443244629
AT4G37520	AT4G37520	1.140699231	2.03903E-05	1.455452373	2.03335E-07	1.985417762	1.21015E-13	-0.068225319	0.956216588	-0.391720558	0.061668516	-0.048607604	0.816481581
AT1G64740	TUA1	1.137576154	0.001585897	1.731038253	5.72127E-27	1.1036286	4.41606E-21	0.584860652	0.704004387	-0.45350238	0.069027805	-0.602012873	8.66344E-07
AT2G30250	WRKY25	1.136738925	0.003874299	1.473712273	1.16426E-11	1.088514314	2.49138E-10	0.557884303	0.691824202	-0.12273417	0.847662387	-0.787489233	6.0435E-06
AT1G03090	MCCA	1.135061573	0.007238696	1.189006283	6.38732E-12	1.715296452	1.04114E-29	2.574893681	0.097747988	0.056161427	0.923979325	0.297301331	0.138721679
AT5G66820	AT5G66820	1.132385374	0.003073638	1.207859823	7.79554E-08	1.163473769	2.89506E-05	0.797942914	0.397639034	0.183465551	0.657765347	-0.101486027	0.708566231
AT5G03500	AT5G03500	1.130139371	0.001494866	1.183812178	7.66692E-06	1.268308163	9.77964E-13	0.623289994	0.608678066	0.157167926	0.693633289	-0.015435563	0.956944681
AT1G30320	AT1G30320	1.129804538	0.007021643	1.229969215	5.51932E-07	1.083815786	1.68949E-13	0.470801784	0.705076831	-0.051327941	0.927080141	-0.0886456	0.705077442
AT3G03490	PEX19-1	1.12858114	0.000201058	1.348479584	6.60544E-07	1.137781914	4.57595E-12	0.26683357	0.770575751	0.138470898	0.788443469	-0.678601984	0.000225615
AT1G10095	AT1G10095	1.127544902	0.039417019	1.120618469	0.00597706	1.027025126	0.00073364	1.010906537	0.498228536	0.052403973	0.958308469	0.170860945	0.540317986
AT1G68620	AT1G68620	1.124167128	0.019299352	1.599318076	4.52423E-06	1.132183484	0.000327513	-0.277026564	0.873147282	-0.35043344	0.572694598	-0.934571005	8.99634E-05
AT4G05460	AT4G05460	1.121950916	0.000364935	1.086844933	5.78992E-05	1.137406638	6.78992E-10	0.326592904	0.81577697	0.286970586	0.320305287	0.1439818	0.454738502
AT5G38530	TSBtype2	1.1204331	0.040668489	1.730867717	8.96373E-06	1.103981993	8.13182E-06	-0.494330753	0.601921549	-0.027739599	0.963419708	-0.047527753	0.850734375
AT4G02880	AT4G02880	1.114681833	0.001194506	1.48769144	5.39426E-12	1.29992167	1.61328E-13	0.129059059	0.914546909	-0.02452959	0.963495178	-0.268158446	0.150889499
AT2G46800	ZAT	1.111131621	0.000208562	1.672666086	7.04085E-13	1.10445694	3.96278E-11	0.170405652	0.881741852	0.095422126	0.749532733	0.075559909	0.665111248
AT2G17520	IRE1A	1.106932128	0.000155506	1.261168517	3.09562E-09	1.103753747	1.42406E-17	0.23604627	0.842538979	-0.145956044	0.661273265	-0.672769436	1.26698E-06
AT3G22430	AT3G22430	1.101050188	0.022473191	1.080367745	0.00093355	1.047410438	4.52308E-05	0.633956657	0.66808962	0.292975628	0.67431887	-0.271770763	0.330238336
AT1G55110	IDD7	1.094340869	0.008239826	1.408379079	9.26566E-12	1.642548963	2.60157E-36	1.534178162	0.191565372	-0.066918527	0.911423117	0.401847367	0.05296275
AT5G67600	WIH1	1.093047178	0.000424865	1.490023074	4.14279E-10	1.390778665	6.88068E-08	-0.408046995	0.709586436	0.084155444	0.865454224	-0.270527338	0.163886894
AT1G02750	AT1G02750	1.092115787	0.028379993	1.097061421	9.88642E-06	1.079668826	1.13648E-09	0.88906873	0.565728752	0.247101948	0.611423968	-0.137934185	0.655577041
AT1G47128	RD21A	1.088754164	0.000710016	1.901869509	3.6998E-14	1.0760923	1.62457E-08	0.368720603	0.731644221	0.097215115	0.680864607	-0.406748701	0.008249972

AT5G58720	AT5G58720	1.084038297	0.001095233	1.074154763	4.83643E-06	1.093983093	5.33192E-12	0.557509729	0.685495817	0.181658433	0.518859406	-0.215622046	0.35070255
AT3G06190	BPM2	1.076122265	0.000275452	1.239133662	1.49995E-11	1.371692584	3.86114E-18	0.794238068	0.516270934	0.166960673	0.544231331	-0.069283635	0.747804361
AT5G26600	AT5G26600	1.074654794	0.008607441	1.447676537	1.21802E-05	1.067976911	5.86659E-05	0.007977505	0.996693402	0.14088815	0.710941849	-0.499530845	0.00366947
AT3G61860	RS31	1.070425797	6.83234E-05	1.09814116	7.69391E-07	1.481648257	4.75898E-23	0.448998396	0.6657277	0.170100115	0.570054815	0.234490789	0.116425609
AT1G17550	HAB2	1.06480389	0.000354964	1.443327884	2.05299E-06	1.003285716	5.30109E-05	0.01743538	0.989846968	-0.104741845	0.801490404	-0.453602479	0.015926527
AT5G18850	AT5G18850	1.064746903	0.044590382	1.190354699	0.003415811	1.385521927	6.58594E-12	0.08629138	0.95447214	-0.098269358	0.857530571	-0.811946876	7.49851E-06
AT1G49540	ELP2	1.064349242	0.010165824	1.035082175	0.00073608	1.02172157	2.12781E-06	0.174867941	0.864295062	0.344538319	0.347502908	0.206845013	0.31205866
AT4G35420	DRL1	1.049258678	0.024921781	1.540691487	0.000588276	1.69726225	8.32904E-10	0.701544449	0.597380545	0.421500555	0.493493798	0.644727036	0.005104184
AT5G66580	AT5G66580	1.046052993	0.009460269	2.020313824	2.29922E-06	1.018203384	0.001732154	0.131576194	0.926124658	0.017977964	0.988021272	-0.117936464	0.778663979
AT5G51070	ERD1	1.036006345	0.001603749	1.188600731	2.72584E-07	1.329575611	1.74959E-18	0.836730285	0.58993184	-0.019273036	0.968000197	-0.674507465	4.70356E-09
AT4G27830	BGLU10	1.035231528	0.002541301	2.125718702	4.61266E-11	1.305601468	7.00955E-08	-0.068469612	0.963509602	0.001226287	0.999095849	-0.519944643	0.009609337
AT3G11420	AT3G11420	1.029959735	0.017228029	1.533720725	1.49188E-08	1.461406991	2.6319E-06	-0.620014276	0.648919095	-0.757005466	0.000911504	-0.815233837	4.42823E-09
AT5G65380	AT5G65380	1.024887486	0.008773364	1.525539397	9.48192E-14	1.412615447	4.45146E-29	0.611155781	0.691824202	0.245310421	0.262710181	-0.374309197	0.023569067
AT3G20770	EIN3	1.024727812	0.000199196	1.095280055	2.06826E-24	1.001836721	5.33901E-21	0.423501772	0.676109289	-0.036457054	0.911423117	-0.41672954	0.000118062
AT3G03950	ECT1	1.021545648	0.000104721	1.027277178	0.000255006	1.079996558	1.48189E-09	0.074772849	0.95660961	0.044304245	0.932632067	-0.127779099	0.509347091
AT4G29900	ACA10	1.020173797	0.000900324	1.460055073	2.97383E-11	1.461608025	2.4155E-33	0.385257114	0.721140128	0.145160481	0.550428211	-0.272152737	0.068219723
AT3G51370	AT3G51370	1.019451293	0.000801698	1.005580294	2.43092E-08	1.278511686	1.90627E-18	0.246900432	0.818777262	0.072942198	0.821415128	-0.706596735	8.88508E-07
AT2G02710	PLPB	1.018067618	0.004732113	1.472414191	5.95878E-14	1.274286043	7.33656E-31	1.022742266	0.565728752	0.224911414	0.421779218	-0.223505159	0.239653808
AT1G08460	HDA08	1.017623787	0.00029498	1.344419299	1.26102E-09	1.020451885	1.07749E-07	0.482608723	0.694676172	0.061033389	0.903043766	-0.199569186	0.401621798
AT1G33050	AT1G33050	1.016565195	0.00069656	1.011958402	1.12354E-08	1.284891224	2.98347E-18	0.940002564	0.584219873	0.039020305	0.924938189	-0.599567982	1.82342E-06
AT3G10770	AT3G10770	1.014689608	0.000677887	1.204344679	1.14312E-15	1.516432615	6.52416E-17	0.934177515	0.513430931	-0.038746013	0.914359122	-0.039702182	0.831602852
AT4G34140	AT4G34140	1.014466615	0.004793648	1.234847297	5.19123E-05	1.191620131	3.06747E-08	0.477093705	0.706203289	0.201897266	0.633532624	-0.066652506	0.834174854
AT1G61690	AT1G61690	1.013965663	0.000626694	1.083084593	1.87648E-10	1.187203918	4.41445E-16	0.219594432	0.84196754	0.179448286	0.559527671	-0.181438464	0.330265139
AT1G09520	AT1G09520	1.008932593	0.004175575	1.326050794	2.90775E-10	1.129644208	9.83104E-10	0.389722058	0.739621931	0.341787221	0.42895719	0.304433273	0.071403954
AT1G33680	AT1G33680	1.008134212	3.74837E-05	1.07274284	8.10452E-17	1.466548742	7.70307E-23	0.846581327	0.547026175	0.088286899	0.776194084	-0.036028801	0.857119124
AT1G29760	AT1G29760	1.007720814	0.024469651	1.074637383	5.98403E-07	1.471904307	2.18453E-22	0.58973833	0.669344191	0.181444315	0.685606577	-0.313137151	0.037624154
AT1G21000	AT1G21000	1.006451989	0.006873746	1.336976716	1.94265E-19	1.414414647	3.20509E-31	1.017008012	0.482795025	-0.06418575	0.880587778	-0.195790608	0.401553813
AT2G43820	UGT74F2	1.002940874	0.00352019	1.373357055	6.20323E-13	1.239962902	1.10779E-16	0.69914262	0.652047062	-0.055668	0.913563814	-0.617912149	6.22483E-08
AT3G46450	AT3G46450	1.002429961	0.004585989	1.767149218	3.4487E-13	1.130421309	3.36274E-14	-0.052398525	0.972614304	-0.345714662	0.228145578	-0.382934899	0.005956868
AT5G03170	FLA11	-1.000955866	0.045265665	-1.820116366	6.79573E-05	-1.878331333	0.012408949	-1.350465115	0.448215878	-0.671090733	0.063561172	-0.128316411	0.719365777
AT1G50010	TUA2	-1.026903537	0.00478677	-1.207241767	2.64164E-07	-1.006758442	5.31522E-10	-0.38065911	0.69828863	-0.078050396	0.759890373	0.66957451	1.43112E-05
AT1G75500	WAT1	-1.030206583	0.004143988	-1.30753816	3.39607E-06	-1.229777375	9.24615E-11	-0.70453925	0.541614164	-0.539536261	0.000805258	-0.556129603	0.000397024
AT4G09460	MYB6	-1.043318474	0.010325677	-1.64274921	2.2398E-09	-1.22146097	0.000103594	-0.272217294	0.735695447	0.184958568	0.566279262	0.19090812	0.296792631
AT4G34290	AT4G34290	-1.045471299	0.041357749	-1.351479847	0.000397399	-1.439425287	5.9847E-09	0.115845324	0.966114207	0.053127862	0.944178317	0.742240446	0.001314775
AT5G48830	AT5G48830	-1.04701931	0.035708598	-1.269146576	0.018179536	-1.092245456	2.38009E-05	0.298980844	0.810169925	0.229270886	0.631435158	0.412780551	0.137120603
AT3G52400	SYP122	-1.051395468	0.011000642	-1.385744666	1.92418E-08	-1.250630364	5.64393E-06	0.632591355	0.697179773	-0.980127373	0.002368604	0.886663421	0.00070853
AT2G42040	AT2G42040	-1.053280453	0.000766723	-1.526326287	1.14602E-06	-1.340467567	1.5483E-07	0.363450341	0.755988967	0.026356513	0.974021484	-0.212267243	0.489720529
AT5G08330	TCP11	-1.055867511	0.002996987	-1.588992565	1.14482E-11	-1.450928313	1.0426E-15	0.15932989	0.924925439	0.164676018	0.638845866	0.409565645	0.141190594
AT5G51545	LPA2	-1.059410307	0.004595872	-1.095914115	1.52579E-06	-1.222576467	7.36387E-10	0.562604633	0.625225271	-0.020535974	0.979393839	0.0644335	0.834957913
AT1G72416	AT1G72416	-1.065120571	0.006258924	-1.307104337	3.12467E-06	-1.491936103	4.96038E-11	0.616231051	0.472046758	0.035968657	0.971700698	0.732500347	0.017595233
AT3G14840	AT3G14840	-1.073273753	0.004256634	-1.591931444	1.1753E-05	-1.629566464	6.37487E-11	-0.579696334	0.691895802	-0.269510386	0.142463471	0.433553331	0.001519102
AT1G55370	NDF5	-1.074030399	0.029566363	-1.567199684	5.51415E-07	-1.138462695	5.58049E-08	0.665018447	0.677154607	0.1433548	0.897477433	-0.035162145	0.949832062
AT3G43600	AAO2	-1.075128935	0.003031254	-1.491508122	6.56913E-10	-1.028822713	9.05783E-06	-0.366349297	0.704225272	-0.12998872	0.728271334	-0.153832236	0.501519547
AT5G19140	AILP1	-1.078862682	0.002585925	-1.164081149	1.25956E-11	-1.147294339	4.16215E-13	0.86398239	0.518021029	-0.047344766	0.900602859	0.486998282	1.22391E-06
AT5G53490	AT5G53490	-1.088953158	0.004784019	-1.110976056	2.63233E-13	-1.077700161	1.3641E-21	1.227852802	0.246235822	0.287684064	0.272503743	0.437568462	0.012092185
AT5G65920	AT5G65920	-1.093655957	0.005449304	-1.801387168	7.11426E-05	-1.18969157	0.000158921	0.010387423	0.99546073	0.266977467	0.624504205	0.310638619	0.125104077
AT1G24170	LGT9	-1.101140586	0.014153211	-1.717550994	4.02676E-08	-1.581557471	8.67358E-07	-0.135619104	0.951485287	-0.228341017	0.464942555	0.031742671	0.891061184
AT1G12460	AT1G12460	-1.104103232	0.010540261	-2.141695866	0.000612739	-1.354057583	0.003543057	-0.133709641	0.932330263	-0.16429948	0.83088478	-0.118240019	0.724470735
AT3G07470	AT3G07470	-1.109261046	0.000110373	-1.412531614	1.93254E-10	-1.384275239	3.87941E-10	-0.472205025	0.531419697	-0.413520835	0.035528844	-0.262582876	0.202423987
AT5G46290	KASI	-1.116291199	0.000579508	-1.281549254	6.39015E-06	-1.511853802	5.51605E-27	-0.289869917	0.768328791	-0.128996792	0.594079186	0.487614715	3.33822E-06
AT4G34220	AT4G34220	-1.119125237	0.000103619	-1.230777415	6.63149E-13	-1.242241069	1.89203E-13	0.187187846	0.771422703	-0.356190374	0.161681323	0.461779055	0.051532778
AT2G23290	MYB70	-1.121362755	0.03690326	-1.853542191	3.93189E-08	-1.12049111	0.000379142	-0.049565081	0.982734229	0.011586498	0.989337643	0.400502293	0.182362611
AT2G14050	MCM9	-1.124694486	0.036046453	-1.017198935	0.03019428	-1.283287132	0.001600896	0.280721942	0.861673753	0.662330554	0.249608675	-0.075995991	0.872128265
AT2G03350	AT2G03350	-1.125648042	0.042641054	-1.621155164	0.001574251	-1.598672258	2.46039E-13	0.045927145	0.978345558	-0.081983468	0.895021414	0.100327489	0.744425821
AT3G62110	AT3G62110	-1.143608981	0.006620369	-1.328955049	1.25171E-05	-1.443350182	3.75035E-10	-0.176093383	0.93480387	0.003525913	0.995671131	0.420303504	0.004420239
AT5G46800	BOU	-1.147881924	0.000229999	-1.087286983	2.64687E-07	-1.191423756	9.50022E-15	-0.085873612	0.924925439	0.362968159	0.151031228	0.195728911	0.283677676
AT2G28630	KCS12	-1.148566235	0.003645703	-1.058733532	0.020281168	-1.951784066	2.47463E-25	-0.126019236	0.953045007	-0.662038347	0.112405654	0.295273434	0.087218715
AT5G65700	BAM1	-1.152899808	0.017489256	-2.105848673	1.00222E-07	-1.215031467	1.45611E-05	-0.479652598	0.627500883	-0.312547488	0.195022122	-0.032851361	0.910814
AT1G13110	CYP71B7	-1.156386353	2.14416E-07	-1.601434671	1.89872E-06	-1.490846522	6.1769E-11	-0.064641541	0.947241707	-0.762430545	4.72023E-05	-0.179416918	0.332652637
AT5G04020	AT5G04020	-1.159648699	0.018380837	-1.22610696	0.002485263	-1.109978529	0.001438668	-0.285077347	0.874570817	-0.417572293	0.188546576	-0.579777502	0.000773114
AT1G29890	RWA4	-1.160734626	0.003676902	-1.783694013	0.005281008	-1.282006079	5.15325E-07	-0.457178059	0.676109289	-0.382577587	0.223683458	0.210795796	0.278259298
AT3G46630	AT3G46630	-1.167138681	0.00039348	-1.255079997	0.001996741	-1.677735396	1.34759E-16	0.612318565	0.532959875	0.59658916	0.141162872	0.552718034	0.039659735
AT4G00730	ANL2	-1.16773407	7.95616E-08	-1.686325692	1.2438E-08	-1.340634595	2.0108E-12	0.128186101	0.876684611	0.17392385	0.509409433	0.17328348	0.232550783
AT5G46580	AT5G46580	-1.17722357	0.00231244	-1.01936147	0.000654743	-1.535635495	4.92577E-10	0.064697592	0.968021479	0.280607035	0.356679881	-0.002025728	0.993906767
AT5G56260	AT5G56260	-1.179995567	0.004022577	-1.621054508	5.02242E-05	-1.629545529	1.08942E-10	-0.333871532	0.848497759	0.150830371	0.848936053	0.135407016	0.672808657
AT3G50270	AT3G50270	-1.184217614	0.035653767	-1.682402665	5.21772E-08	-1.166923338	0.001767304	1.772374174	0.441302192	0.025623097	0.989242512	0.609510393	0.332641753
AT1G22550	AT1G22550	-1.194893915	0.027512847	-2.071542138	0.001209459	-1.046468545	0.004166804	-0.635988245	0.598901706	-0.352716407	0.553336532	0.580708994	0.005685286
AT4G24275	AT4G24275	-1.203665179	0.047369489	-1.35495228	0.017290635	-2.143857584	7.22609E-06	-0.674038547	0.657369817	-0.139522385	0.747458101	0.468790201	0.032968665
AT1G11350	SD1-13	-1.205442527	0.000134819	-1.147873721	0.000196435	-1.134648929	5.05722E-07	0.5266285	0.604422229	-0.082289432	0.898728824	0.716818573	0.00213739
AT1G25440	BBX15	-1.21052562	3.74609E-06	-1.517526746	1.44045E-08	-1.413322906	4.1979E-17	0.654563436	0.613859108	0.318500517	0.421936299	0.268715315	0.119775757
AT3G08920	AT3G08920	-1.22052245	4.13923E-05	-1.272219683	1.29186E-09	-1.102201696	8.26625E-06	1.097075022	0.146103977	0.704146955	0.039753935	0.507061923	0.004163998
AT2G23380	CLF	-1.227566594	0.043066287	-1.392686403	0.015564984	-1.419114283	1.44935E-09	-0.725152684	0.711751731	0.092184732	0.858565321	0.232778109	0.193978205
AT1G61170	AT1G61170	-1.230979398	0.020692544	-1.916733995	3.56364E-05	-1.733176544	3.30179E-05	-0.748895014	0.561935767	0.174698498	0.761516542	0.182957727	0.569262556
AT2G24020	AT2G24020	-1.231721792	1.85758E-05	-1.14645884	0.00173975	-1.352301772	4.19605E-22	0.722276092	0.470210027	0.281568961	0.461865804	0.899581038	2.82337E-10
AT1G57980	AT1G57980	-1.240819418	0.008449777	-1.444457736	7.13094E-06	-1.101374223	0.00363951	-0.172512988	0.926730556	-0.210689814	0.835639362	-0.27555964	0.539081632
AT1G54220	AT1G54220	-1.242702114	0.002045226	-1.139647155	0.001313191	-1.651143568	1.07078E-10	-0.268295645	0.868036566	0.010338504	0.992165706	0.25680179	0.210810182
AT1G60800	NIK3	-1.242750562	2.45156E-05	-1.549639169	2.40294E-11	-1.071534292	3.6053E-05	-0.109583774	0.936758504	-0.014096238	0.98143759	0.354687389	0.034713458
AT1G05470	CVP2	-1.244579696	0.038253392	-1.631900254	2.80809E-07	-1.00360153	0.000117362	0.182728785	0.871383083	0.027303383	0.975386078	0.388852856	0.057461886
AT3G62660	GATL7	-1.246454848	4.77881E-07	-1.694163244	6.21243E-19	-1.442935927	8.66396E-17	-0.264867638	0.737072243	-0.231668304	0.50618714	-0.549057856	9.9999E-05
AT1G11670	AT1G11670	-1.260249809	0.045816104	-2.237408667	1.28408E-05	-1.615917804	2.181E-05	-0.915096106	0.559215018	-0.382628768	0.438575468	0.246132991	0.333162917
AT5G55480	SVL1	-1.26035198	0.026554099	-1.731447934	0.004275418	-1.044281593	0.003646466	-1.027010861	0.563804992	-0.782915106	0.001928768	0.103249913	0.683095096
AT5G40610	GPDHp	-1.263346605	8.42464E-05	-1.219207195	0.00034906	-1.247435858	0.00015987	0.428849189	0.589474071	0.525508156	0.108285109	0.504043115	0.002971864
AT2G01420	PIN4	-1.263443659	5.36511E-07	-1.189863688	4.45371E-14	-1.237850998	2.92829E-20	-0.279764115	0.62738272	-0.092678316	0.798599938	-0.229003299	0.087441854
AT2G46330	AGP16	-1.265300917	1.07236E-06	-1.761130598	6.43462E-19	-1.380284062	1.64746E-12	-0.36759464	0.632278424	-0.0683041	0.865842332	0.389060388	0.009384868
AT2G18910	AT2G18910	-1.270859022	0.001715641	-1.654180428	0.002790986	-1.482782665	8.06089E-12	-0.345032464	0.849512513	-0.080542902	0.896222696	0.352347953	0.075222386
AT2G36310	URH1	-1.276811032	0.002283795	-1.200042693	0.000127401	-1.436764077	1.53791E-09	-0.81545053	0.662900207	-0.32555293	0.485309912	-0.075166226	0.753002162
AT1G20190	EXPA11	-1.279093286	0.019031036	-1.13988344	8.381E-07	-2.329752086	2.74622E-28	-1.323350131	0.505950107	-0.716594635	1.18834E-05	-0.088143485	0.791640653
AT3G57560	NAGK	-1.279295649	0.00044115	-1.192374066	2.4304E-06	-1.149379068	1.62696E-09	-0.052868967	0.982734229	0.170619731	0.686407369	0.423290992	0.042892252
AT2G27820	PD1	-1.282932525	0.000855064	-1.002044479	0.004316635	-1.164949439	1.10449E-08	-0.120781506	0.947537872	0.038323493	0.949416132	0.352503997	0.041371789
AT5G27550	AT5G27550	-1.300450035	0.036379001	-2.171214619	1.25645E-06	-1.235920805	0.040215121	-0.358777714	0.732322487	0.010383615	0.98996705	0.371268662	0.082326872
AT1G74690	IQD31	-1.315282173	0.001415635	-1.332663136	5.57735E-07	-1.02360734	7.74057E-06	-0.126635239	0.945609936	-0.09375968	0.702760422	0.256545052	0.140320118
AT5G51460	ATTPPA	-1.318536191	0.001214665	-1.397170387	0.00050552	-1.335466758	7.49002E-05	-0.025340265	0.989481773	0.084248531	0.926800209	0.390545339	0.182387605
AT2G13690	AT2G13690	-1.320905982	0.020546422	-2.137306513	0.002217547	-1.150786161	0.003933889	-0.227060793	0.936447266	0.184839187	0.820057591	-0.105879665	0.761998212
AT3G05750	TRM6	-1.320979332	0.010490576	-1.685576446	3.35131E-06	-1.281574229	0.000846342	0.096620915	0.956970681	0.397985084	0.357040463	0.232087853	0.367663557

AT3G19400	AT3G19400	-1.32192134	0.011646678	-1.917514293	3.1017E-06	-1.842045582	8.66542E-07	-0.328686362	0.885844797	-0.007338328	0.993586481	-0.056308944	0.845485094
AT2G20340	AAS	-1.322097364	0.015989343	-1.187644303	0.017738459	-1.814063953	0.003633879	-0.316464182	0.869069601	-0.921749893	0.18824835	0.160535203	0.658728072
AT1G70210	CYCD1;1	-1.32900971	0.007928778	-1.839949119	1.42306E-07	-1.426343505	1.00007E-09	-0.422667083	0.763737566	0.184170184	0.566059569	0.167512365	0.286121287
AT5G49160	MET1	-1.333090929	0.002603272	-1.369790582	0.001273291	-1.242375339	9.93715E-07	-0.266818419	0.870136387	-0.148764857	0.704826102	0.375375004	0.013161004
AT4G05520	EHD2	-1.33856496	0.0165455	-1.867439779	0.008272638	-1.208246697	0.003365558	-0.210581546	0.926730556	0.380682031	0.556988648	0.75126222	1.56745E-05
AT3G52500	AT3G52500	-1.338796711	6.5583E-10	-1.032283044	0.002769119	-1.055513622	2.66294E-06	-0.350421495	0.671810924	-0.2768701	0.2553851	0.54844377	0.001549578
AT2G25480	AT2G25480	-1.339051442	0.00106039	-1.084558671	1.48275E-05	-1.246742711	1.45237E-07	-0.231099968	0.870759043	0.108421493	0.841586178	0.412874503	0.015418205
AT5G43760	KCS20	-1.341437071	2.39563E-07	-1.213588079	9.6945E-14	-1.180002572	6.12561E-11	-0.870913694	0.128407019	-0.791362387	2.28868E-07	-0.857417473	3.14983E-11
AT1G32120	AT1G32120	-1.342964035	0.026297833	-2.443353774	2.05599E-07	-1.015137455	0.007765422	0.924020606	0.514939801	0.279903593	0.731476924	0.376856135	0.047775674
AT4G18340	AT4G18340	-1.343700816	0.006213701	-1.776266767	0.0007189	-1.939504914	2.73986E-05	0.140127379	0.948761383	0.150827742	0.813042846	0.486626691	0.10756892
AT2G44040	AT2G44040	-1.344351035	0.00155076	-1.138933241	0.023760461	-1.842439772	3.79625E-10	-0.181649753	0.935083701	0.411596291	0.187623269	0.886796743	6.98084E-10
AT5G48460	AT5G48460	-1.348672101	0.044949109	-2.042554714	7.99833E-13	-1.549829035	1.59841E-09	-0.531455973	0.794188368	-0.35050195	0.265754101	0.158968302	0.343956583
AT4G21970	AT4G21970	-1.359553726	0.008300031	-1.856151571	0.012487249	-1.010457202	0.04551682	1.63796068	0.429222482	-0.491100152	0.723920667	0.746400713	0.076622943
AT5G23820	AT5G23820	-1.360979509	0.035311806	-2.471754445	2.7721E-11	-2.37114487	3.68815E-27	-0.476574355	0.751883669	0.10730344	0.798386362	0.462672685	0.003514869
AT3G09580	AT3G09580	-1.361106197	0.000141847	-1.627002973	0.000267407	-1.106469885	1.62379E-05	0.589805826	0.637815859	0.067368778	0.917278038	0.301246252	0.260518382
AT2G26550	HO2	-1.363324117	5.92543E-05	-1.474222417	0.000405893	-1.009979945	1.59643E-06	0.289613187	0.791701102	0.029661114	0.968000197	0.329862002	0.052419549
AT2G05790	AT2G05790	-1.363501697	0.005858688	-1.872773835	1.48985E-09	-1.298131732	2.12114E-30	-0.327916857	0.820186281	-0.006932116	0.988694176	0.57048597	0.000306604
AT5G55280	FTSZ1-1	-1.364012869	0.004690889	-1.317527612	2.41347E-07	-1.348340198	8.45688E-12	-0.041985654	0.982623916	0.06831366	0.866906715	0.547014714	0.000549402
AT1G04820	TUA4	-1.367616919	0.0031657	-1.518934806	1.09154E-06	-1.247179812	3.76846E-11	-0.475640716	0.735695447	-0.219911865	0.15117005	0.631043316	0.000381763
AT1G14310	AT1G14310	-1.372245478	0.00508906	-1.170837303	0.006365303	-1.205307751	0.000706582	0.412100077	0.728134899	0.265307498	0.68357422	0.508663976	0.046587406
AT4G33680	AGD2	-1.380021613	2.2466E-07	-1.28529275	8.06438E-05	-1.597944361	7.97535E-26	-0.127830037	0.918503553	0.075467747	0.861957219	0.744119086	6.00312E-07
AT2G34090	MEE18	-1.383881755	0.001719576	-1.543291834	0.002151809	-2.058586393	1.71206E-11	0.204741707	0.927465214	-0.002521228	0.998877638	0.262604326	0.338322856
AT3G16000	MFP1	-1.388227798	4.15774E-08	-1.071487794	1.05228E-08	-1.28157139	1.24724E-13	0.642990858	0.405880771	0.348343062	0.263073498	0.750270174	7.78866E-09
AT3G47960	GTR1	-1.389982919	0.00132765	-1.178709785	0.000803133	-1.509160788	3.94084E-05	-1.073134551	0.374967877	-0.793774993	0.070110076	0.087282084	0.765161582
AT3G15190	PRPS20	-1.395104344	6.28082E-09	-1.058417767	0.000610272	-1.48404653	4.38246E-53	0.274575697	0.637815859	0.293743349	0.35863867	0.908495667	3.25223E-08
AT5G24314	PTAC7	-1.395815373	0.000193048	-1.146479043	0.023281515	-1.475542079	9.26563E-18	0.137698394	0.926969568	0.350196492	0.447139821	0.644154592	8.57838E-05
AT1G26570	UGD1	-1.405400381	0.037755352	-1.516139864	0.000646197	-1.25158974	9.28363E-05	-0.476104369	0.961358666	-0.187398607	0.708747273	0.848100999	0.000167076
AT3G24480	AT3G24480	-1.406136311	3.13793E-05	-1.154418496	0.000201293	-1.325451949	4.32913E-09	-1.01258832	0.134411276	-0.961224916	1.53345E-06	-0.515166854	0.000322754
AT5G57440	GS1	-1.408492965	0.017609186	-2.177150057	2.19008E-06	-1.451953834	3.05929E-11	-0.116137057	0.962872831	0.104246769	0.870101981	0.81261013	4.81061E-05
AT1G09200	AT1G09200	-1.413978527	0.019960402	-1.826347659	6.65444E-09	-2.038968573	8.5652E-12	-0.560322043	0.732595931	-0.226133298	0.43793265	0.259663238	0.213444335
AT5G51550	EXL3	-1.417136598	3.53428E-06	-1.244427161	2.05034E-16	-1.145850248	3.20665E-11	0.490269728	0.624478206	-0.531578305	0.000983778	-0.001597872	0.995980165
AT4G37510	AT4G37510	-1.417458676	2.03252E-08	-1.080649027	1.83565E-13	-1.092400801	8.48687E-14	0.1873861	0.788170174	0.092748546	0.803294274	0.507898943	0.003845538
AT3G49670	BAM2	-1.424512739	0.009457496	-2.151225598	7.27346E-09	-2.081806423	6.21208E-12	-0.498370094	0.706790319	-0.21566736	0.537982096	0.294805672	0.185839314
AT2G47590	PHR2	-1.424650163	0.000139785	-1.052083701	3.62268E-05	-1.591879397	2.03639E-27	-0.061847447	0.970302657	-0.037436363	0.938675699	0.81885551	1.58796E-10
AT2G44500	AT2G44500	-1.425529947	9.7753E-06	-1.520676636	1.84288E-06	-1.353446209	5.89245E-06	-0.162393665	0.890591808	-0.466305154	0.049663039	0.633974159	3.19916E-07
AT3G05320	AT3G05320	-1.426988866	0.02004097	-2.09537037	0.03533403	-2.683547137	0.000339218	-0.304746217	0.905404361	-0.164365199	0.911400203	-0.02139771	0.976020785
AT1G20850	XCP2	-1.428815987	0.000261434	-1.395888368	0.02496612	-1.029221425	0.000835872	-0.372955038	0.712887303	-0.519999229	0.067570199	-0.156762355	0.442073554
AT5G36890	BGLU42	-1.43083429	0.035426816	-1.505452657	0.042126957	-1.741520884	6.68308E-05	-0.852145316	0.641679337	-0.303419265	0.512980933	0.330248667	0.13863628
AT4G39770	TPPH	-1.430974813	0.010318894	-1.473961361	6.43793E-05	-2.029664361	1.75735E-06	-0.442256148	0.857472027	-0.698393827	0.156921618	-0.205821479	0.63789371
AT3G62630	F26K9_60	-1.433192411	0.031896036	-2.375137538	1.03695E-10	-2.005133176	1.23219E-08	-0.264626669	0.902115605	0.451609535	0.086842332	0.262342505	0.251500393
AT1G12244	AT1G12244	-1.438935557	0.001577378	-1.576618316	0.015991928	-1.176207914	2.4493E-05	-0.011205635	0.996799452	0.219003007	0.84792397	0.528402954	0.028779994
AT5G52280	AT5G52280	-1.441292388	7.69019E-05	-1.234574613	8.93838E-05	-1.026258125	3.03212E-06	-0.133541579	0.930511033	-0.098809154	0.817199908	-0.110680574	0.564984661
AT1G62950	AT1G62950	-1.451096268	0.035280632	-1.633448024	0.006454718	-2.162183347	0.002438599	0.25225194	0.885120518	0.011450415	0.992575938	0.088261137	0.834374862
AT5G53500	AT5G53500	-1.453464372	2.12553E-05	-1.89884877	3.14254E-11	-1.190129167	6.62891E-05	-0.219917379	0.850582824	0.026735524	0.975386078	0.435152979	0.015350083
AT5G24810	AT5G24810	-1.471422781	0.000112089	-1.046500101	0.001562845	-1.234910062	1.01862E-07	-0.744430027	0.585445788	-0.322951624	0.310782919	-0.056944873	0.857733604
AT4G34610	BLH6	-1.473403144	1.64853E-05	-1.694756885	5.29102E-08	-1.726599098	1.2114E-09	0.029533111	0.990231864	0.162873678	0.83365656	0.563599583	0.000486833
AT4G13840	AT4G13840	-1.47352546	4.71469E-06	-1.394486292	6.22119E-07	-1.902379438	7.24398E-19	0.075942593	0.950356778	0.012472813	0.986818647	0.716276919	8.32591E-08
AT5G01220	SQD2	-1.474431867	6.4367E-07	-1.323241286	2.67859E-06	-1.950052669	8.81513E-30	0.297981069	0.782971455	0.060502072	0.933465014	0.131144175	0.584024373
AT3G44020	AT3G44020	-1.474735065	0.001259895	-1.306734154	0.00040585	-1.016624765	0.013747653	1.332430492	0.498460747	0.37221267	0.759890373	0.831163594	0.004730853
AT1G75280	AT1G75280	-1.475194608	0.000709388	-1.069031149	0.001910457	-1.645445481	9.91917E-12	-0.375360609	0.800906229	-0.605179681	0.014807791	-0.005417868	0.988233344
AT2G22230	AT2G22230	-1.475374768	0.001340114	-1.708887265	0.002847321	-1.646557959	2.27772E-21	-0.094095692	0.953801756	-0.144243739	0.805100296	0.550946394	0.015374929
AT5G23350	AT5G23350	-1.487166364	0.002480392	-2.145754393	0.001006758	-2.07037932	9.28265E-06	0.244242109	0.874183021	0.021663663	0.986818647	0.495656219	0.09771885
AT1G48350	EMB3105	-1.489911442	7.96751E-13	-1.11530094	7.77482E-07	-1.315041654	1.86845E-29	0.464930608	0.44746329	0.440359466	0.142159834	0.985364396	5.09444E-17
AT1G23080	PIN7	-1.490413933	3.73656E-12	-1.398918441	6.51583E-10	-1.291072293	1.33033E-16	-0.297449738	0.632278424	-0.241954307	0.366326255	-0.114829884	0.455607217
AT1G22650	A/N-InvD	-1.498348237	1.47838E-05	-1.484315547	0.006433085	-1.756294734	3.89315E-08	0.550621069	0.691895802	-0.454991307	0.554072186	0.615333777	0.041989712
AT1G09390	AT1G09390	-1.503670508	0.002651694	-1.329004608	0.04339685	-1.310101339	0.000209984	-0.338147276	0.820941695	-0.045360756	0.95271487	0.087764389	0.729188227
AT3G53260	PAL2	-1.515345945	7.73314E-08	-1.305801531	5.79201E-08	-1.347880235	6.63101E-11	-0.178387097	0.901544633	-0.321125189	0.186425171	-0.07792415	0.746673211
AT3G11945	HST	-1.515926222	1.00063E-05	-1.187919637	0.000184828	-1.707756901	1.08043E-12	0.478490613	0.568009156	0.121183657	0.8608857	0.698037287	0.000900721
AT2G31890	RAP	-1.519482324	0.000430476	-1.330938537	0.012205526	-1.184572442	5.13125E-09	0.018608083	0.994744545	0.135295346	0.857077081	0.409803781	0.014927667
AT5G16390	CAC1	-1.522118891	9.74263E-10	-1.122914408	1.99609E-09	-1.085327934	9.76672E-12	-0.087622749	0.939540455	-0.124901235	0.77266756	0.384174555	0.023157499
AT5G61270	PIF7	-1.525152611	0.002381725	-1.044718803	0.003229273	-1.319100759	1.12197E-05	2.596362845	0.611039849	0.209889004	0.964274323	-1.113993585	0.310350277
AT3G49650	AT3G49650	-1.534978901	0.002000389	-1.518043511	0.000118093	-1.142475622	0.000795134	-0.520651735	0.733019473	-0.168568951	0.832170536	0.806384842	0.004232447
AT1G12500	AT1G12500	-1.53792634	0.00058176	-1.405979439	2.61601E-10	-1.09485368	2.11272E-06	-0.75047637	0.582068518	-0.517403041	0.019794356	-0.170990679	0.36028053
AT1G56050	AT1G56050	-1.539031796	0.00220396	-1.022979698	0.00385796	-2.191596362	3.18943E-15	-0.402801385	0.652047062	0.178664562	0.739480447	0.681440732	2.16788E-05
AT2G39280	AT2G39280	-1.541546626	0.000353663	-1.561618741	2.23106E-05	-1.305822625	6.78626E-07	-0.009844934	0.994162268	0.326691275	0.2553851	0.061263321	0.807125305
AT2G40840	DPE2	-1.548827458	3.10265E-12	-1.173788058	3.2864E-10	-1.842184799	9.40392E-40	-0.121843469	0.936447266	0.217288251	0.430029101	0.266925077	0.079775116
AT3G18500	AT3G18500	-1.552908833	0.004910045	-1.841218805	0.000646006	-1.536530154	1.06003E-06	0.816113164	0.669477598	0.0468745	0.972994743	0.61399831	0.049969425
AT5G58250	EMB3143	-1.556019847	4.20881E-11	-1.063730816	8.07371E-06	-1.334855668	1.40989E-19	0.444213388	0.632278424	0.040694271	0.948625602	0.95139558	2.77596E-05
AT3G11700	FLA18	-1.557317195	5.56203E-09	-1.24534456	1.13082E-17	-1.616863394	2.34447E-18	-0.759070036	0.379538351	-0.402219059	0.084468991	0.095228145	0.580293788
AT4G32570	TIFY8	-1.562610833	1.34464E-06	-1.219571287	6.26797E-05	-1.125569782	1.96847E-10	0.144027915	0.875558325	-0.077701954	0.863621528	0.695419898	1.92784E-07
AT1G25450	KCS5	-1.563850496	2.28965E-05	-1.262438632	0.001418386	-1.345286318	0.000426662	-0.591372555	0.693517591	-0.529303016	0.158543269	-0.724863247	0.002398083
AT2G07690	MCM5	-1.565823445	0.024122638	-1.285449571	5.15045E-05	-1.158233676	0.001507397	-0.027729562	0.993846273	-0.249181683	0.644247015	0.086904239	0.743874616
AT1G70370	PG2	-1.567240022	1.71426E-06	-1.053801668	0.000564503	-1.716702366	1.98238E-32	-0.709874641	0.611226747	-0.516078433	0.005070854	0.10773239	0.645082103
AT1G48480	RKL1	-1.567377232	0.000202689	-1.904851393	9.88954E-08	-1.647387326	3.83622E-07	-0.562960963	0.606464418	-0.431808971	0.110300088	0.052227965	0.797247615
AT4G11820	HMGS	-1.571467412	4.22551E-06	-2.006647364	1.51553E-18	-1.597555209	5.23482E-13	-0.58893886	0.632278424	-0.19375478	0.639577631	0.43137324	0.00090095
AT5G18570	EMB269	-1.572405224	1.51298E-08	-1.098237996	0.000843596	-1.5134879	0.000958045	0.085256516	0.97476204	0.329401214	0.436704713	0.397330025	0.012041063
AT5G41050	AT5G41050	-1.572516689	0.003221454	-1.450319011	0.000326035	-2.221234597	2.62239E-15	-0.73095418	0.721140128	0.32195158	0.723574445	0.700349619	0.08547011
AT2G40690	GLY1	-1.579139369	0.004112294	-1.418676482	0.000425342	-1.644997752	2.39218E-05	-0.366139866	0.906561784	0.114206723	0.886523736	0.94327147	1.44367E-05
AT1G68890	PHYLLO	-1.582860173	0.000189613	-1.514821731	1.75227E-05	-1.142251502	1.90586E-09	0.222154909	0.813446107	0.037828208	0.954114031	0.698659903	0.000256325
AT5G22390	AT5G22390	-1.585721477	0.000170275	-2.7647824	6.64533E-05	-1.113176801	0.024212245	-0.541800685	0.899163675	1.326621308	0.139706229	0.851620772	0.274602138
AT3G17510	CIPK1	-1.589744333	5.70584E-12	-1.400662891	2.07842E-16	-1.155545635	7.13119E-19	-0.017614598	0.990915497	0.027183524	0.964378377	-0.293327067	0.097579614
AT5G48880	KAT5	-1.598370166	0.013855838	-1.647988624	0.000304298	-2.01349942	2.12139E-07	0.887940385	0.87100943	-0.133119175	0.944342934	-0.025545094	0.975107592
AT1G49780	PUB26	-1.601607833	0.002518178	-1.140781827	0.004997598	-1.002108304	0.001126364	0.10676966	0.937910278	-0.622742117	0.187623269	-0.279707132	0.274002633
AT1G56720	AT1G56720	-1.607448616	0.003087371	-2.192739556	5.27911E-10	-1.835904114	1.47596E-08	-0.51720359	0.759786486	-0.125944958	0.784525395	0.555259556	0.000359814
AT3G19450	ATCAD4	-1.612097204	3.31009E-05	-1.238985383	0.000202307	-1.708858154	7.92012E-11	-0.322382629	0.742776611	-0.33920886	0.420125039	-0.616254272	0.027914143
AT3G17640	AT3G17640	-1.615339273	0.00556042	-2.688398401	0.038819071	-3.278577117	4.3155E-06	-1.01129633	0.610291087	-1.286196459	0.057158059	0.355271682	0.443286354
AT2G31040	AT2G31040	-1.615697718	5.18822E-09	-1.20433623	9.8998E-10	-1.380505192	1.38564E-14	0.547409105	0.712887303	0.090260634	0.876125588	0.598847657	0.000161246
AT4G12420	SKU5	-1.620434972	1.5874E-06	-1.925091443	1.82073E-12	-1.314853951	1.07843E-12	-0.765686638	0.302357822	-0.342727955	0.212574616	0.199661993	0.259087267
AT4G30020	AT4G30020	-1.624475573	0.001895932	-1.042555966	0.007332687	-1.568342202	4.52784E-09	-0.855199146	0.532231419	-0.318406284	0.226218163	0.199610428	0.376064952
AT2G37950	AT2G37950	-1.624647748	0.012375507	-2.549250771	0.001549681	-2.664338737	4.15117E-06	-1.332400113	0.730749258	0.662033584	0.475859633	-0.722242174	0.153538753
AT3G46550	SOS5	-1.62995461	0.013762388	-2.079285914	0.000142111	-1.36123893	0.000148458	-0.543584941	0.685495817	-0.677008639	0.006392008	-0.046997849	0.865883226
AT3G50240	KICP-02	-1.638901467	3.32693E-08	-1.265367501	1.76958E-06	-1.30306643	8.358E-16	1.270836461	0.457226926	0.201741413	0.635351512	0.393427764	0.051384653
AT4G27430	CIP7	-1.6402465	6.51489E-10	-1.599891043	2.71875E-14	-1.265682116	3.11292E-16	-0.154713768	0.79822243	-0.311823724	0.243461821	-0.036395863	0.864438644
AT1G34000	OHP2	-1.651023586	0.000172807	-1.318359247	2.39393E-06	-1.223773283	2.88187E-08	0.952556404	0.576657372	0.335774949	0.47709135	0.222008985	0.442659586

AT5G22620	AT5G22620	-1.652113965	2.2176E-10	-1.43054488	1.40077E-10	-1.097115687	0.00010497	0.631532615	0.6208322	-0.031305001	0.967816739	0.325032724	0.235283903
AT5G22800	EMB1030	-1.652190238	3.02536E-08	-1.233895714	2.81512E-06	-1.544703899	1.13886E-11	0.473991326	0.700347186	0.361935453	0.377377981	0.320403279	0.032749367
AT1G50900	GDC1	-1.652613097	7.99393E-07	-1.136260464	0.006233765	-1.851490993	5.91106E-19	-0.376404466	0.769907555	-0.142212542	0.887420847	0.818711366	0.016735536
AT1G69740	HEMB1	-1.65511574	1.33932E-19	-1.094028045	1.15274E-09	-1.52584975	9.82615E-37	-0.126937848	0.874288094	0.08800388	0.752481505	0.547737113	0.000290665
AT4G37240	AT4G37240	-1.655303337	0.007312396	-4.020668051	0.000172109	-2.817192511	0.000324337	-1.111012836	0.437207517	-0.268426071	0.72447373	0.486742319	0.053750944
AT3G61550	AT3G61550	-1.662578937	6.64096E-06	-1.41337367	0.009321338	-1.260592724	2.7737E-06	-0.274303907	0.777552737	-0.631177466	0.049406238	-0.340223874	0.167597498
AT3G51150	AT3G51150	-1.663118126	1.93927E-05	-1.704433906	6.11871E-07	-1.099440053	2.67404E-06	-0.167422367	0.904609828	0.044977672	0.957133906	0.063111407	0.821833228
AT4G19020	CMT2	-1.663709583	0.00344377	-2.126776938	1.55991E-11	-1.157482678	2.99866E-09	-0.366729561	0.726635812	0.358964299	0.348918221	0.451949902	0.008963937
AT5G15050	AT5G15050	-1.669884821	0.032564204	-1.447430893	0.018007796	-1.414531525	0.000214441	-0.771100872	0.565728752	-0.068361207	0.93295262	0.11475359	0.692936819
AT4G37040	MAP1D	-1.671187061	2.31151E-07	-1.171936317	0.000298746	-1.113742149	1.50759E-10	-0.242345762	0.870136387	0.138084645	0.792363715	0.246279017	0.198830481
AT3G45850	AT3G45850	-1.671679514	6.31089E-06	-1.758003833	1.53346E-06	-1.110744432	1.54996E-09	-0.029098748	0.986517811	0.031675042	0.958107537	0.520954908	0.000846196
AT5G44030	CESA4	-1.673514345	0.001511818	-1.396979064	0.029102463	-1.167815913	0.024839862	-0.354076718	0.726635812	-0.488336321	0.337356272	-0.173758943	0.512567433
AT2G30570	PSBW	-1.678858871	6.84574E-06	-1.311234192	2.26844E-22	-1.306018789	5.22522E-28	2.084391016	0.099695852	0.692693862	0.002887237	0.839466783	3.66746E-07
AT3G20350	AT3G20350	-1.679152578	0.001151784	-1.397713082	0.029111859	-1.740522226	1.4224E-11	0.08662337	0.973990229	-0.420205307	0.423442669	0.806282744	0.000203102
AT5G61440	ACHT5	-1.68076549	7.34242E-06	-1.888000675	0.001883226	-1.301612499	8.50087E-11	-0.019081747	0.991695516	0.275549399	0.529833936	0.991121572	2.41222E-10
AT3G27160	GHS1	-1.684634624	7.83683E-13	-1.180810848	3.18626E-06	-1.59144152	2.84993E-39	0.526392837	0.62738272	0.390713863	0.146549515	0.843272248	6.07473E-08
AT1G45207	AT1G45207	-1.68591843	0.046152319	-1.942376219	0.000133681	-1.381189679	0.000345379	-0.111572383	0.965260868	0.682047372	0.090011354	0.728852584	4.46182E-06
AT1G64980	CDI	-1.68787919	3.49183E-07	-1.388007815	0.000109141	-1.500755747	7.9175E-07	-0.235712581	0.870759043	-0.235782956	0.536643581	0.583847555	0.000409877
AT2G04780	FLA7	-1.691827097	0.034049903	-1.549099561	0.004624555	-3.286936149	1.72197E-34	-0.944619636	0.651064692	-0.649792456	0.000976546	0.425502729	0.030252184
AT4G18205	AT4G18205	-1.692786832	2.93081E-05	-1.793768492	8.89504E-10	-1.307924709	2.67281E-05	0.888110245	0.572584637	-0.516025888	0.258496511	0.734823965	0.003227727
AT5G45290	AT5G45290	-1.69304992	0.000506147	-1.081737927	0.001860038	-1.026751479	6.36779E-05	-0.237068919	0.901081775	-0.060511791	0.90943989	-0.039827082	0.855927192
AT3G12930	AT3G12930	-1.694748796	1.91575E-05	-1.570714761	5.71492E-05	-1.928541095	1.50064E-18	0.097603517	0.953801756	0.389114622	0.249491868	0.194715079	0.365922673
AT2G33570	GALS1	-1.695198905	0.000801195	-1.20351375	2.96846E-06	-1.890329956	4.27815E-12	-0.752905414	0.637614678	-0.48832533	0.084978281	0.569547014	0.007011127
AT5G41140	AT5G41140	-1.695955392	0.001744972	-1.838029044	0.001702575	-1.764312527	6.0207E-09	-0.856168336	0.663046749	-0.227061723	0.474364938	-0.207686759	0.227293842
AT3G49720	AT3G49720	-1.696103591	3.90691E-09	-1.126367508	3.82017E-05	-1.011576649	7.98077E-07	-0.745425576	0.591481547	-0.300139147	0.232663375	0.230044522	0.188628979
AT2G40490	HEME2	-1.696502002	1.89681E-17	-1.567366575	3.98934E-08	-1.533373784	9.25135E-18	0.236179523	0.803996013	0.35333136	0.160507774	0.882631906	2.55693E-14
AT4G39640	GGT1	-1.701886915	1.60243E-11	-1.098901422	0.000174831	-1.197220268	7.8902E-09	-0.321694689	0.680482607	-0.425975234	0.327570049	0.563328206	0.000267272
AT5G20140	HBP5	-1.703310776	0.00011237	-1.123312705	0.002680717	-1.511482533	1.83925E-06	0.401499131	0.798269242	0.43831761	0.497254714	0.993459975	0.000659474
AT5G13840	FZR3	-1.704609588	0.000138246	-1.663994348	1.83578E-09	-1.062512802	0.002249148	-0.01396802	0.995383822	0.371709586	0.434595032	0.927573883	4.37861E-09
AT1G55140	AT1G55140	-1.704913389	0.000307219	-1.220405795	7.71125E-05	-1.171146738	0.000200179	0.45703615	0.721140128	0.147352479	0.832272733	0.447202737	0.069984745
AT4G32830	AUR1	-1.704969049	0.019994529	-2.544078127	0.001637543	-1.686187002	0.022106781	-0.377644688	0.954584875	-0.018746316	0.987053629	0.811471765	8.27522E-07
AT5G14660	PDF1B	-1.706209667	3.61268E-06	-1.035056159	0.001358733	-1.415813889	2.50776E-10	0.575235665	0.59446528	0.140970854	0.805285979	0.751160057	4.52815E-05
AT5G16590	AT5G16590	-1.713053012	0.00014647	-1.884706533	9.92191E-08	-1.214182794	4.40091E-07	-0.669567466	0.532231419	-0.574046078	0.015830812	-0.003602387	0.989307115
AT2G46160	AT2G46160	-1.720138015	0.001260773	-2.084283133	1.20441E-05	-2.076761752	3.47305E-07	-0.261311526	0.895706838	0.063391929	0.963495178	-0.344060806	0.408248365
AT4G04640	ATPC1	-1.721226615	8.01706E-09	-1.077663502	7.71324E-15	-1.27100641	1.23993E-36	1.12044808	0.480814999	0.217798847	0.513584451	0.468115856	0.004074814
AT2G47860	SETH6	-1.722195663	0.016760985	-1.398626254	0.017937103	-1.501201179	0.000273551	-1.039146795	0.6112702	-0.225694881	0.721359573	-0.918271669	1.17027E-06
AT3G12110	ACT11	-1.724282507	0.003842647	-1.533497189	0.00018772	-2.064363565	0.015956554	-0.098512999	0.958708047	-0.522866215	0.176596188	0.398310025	0.11900923
AT5G62790	DXR	-1.725480607	1.56655E-12	-1.113907918	1.48046E-09	-1.112657425	2.23883E-14	0.104689584	0.936758504	0.032686762	0.945410042	0.418269298	0.002350723
AT2G38310	PYL4	-1.726733348	7.07125E-08	-2.204765995	0.002334444	-2.025081413	0.000756751	-0.247223734	0.862037276	-0.116212203	0.697262551	0.151335243	0.648394706
AT2G29360	AT2G29360	-1.728723425	0.002124237	-1.475884263	1.78082E-10	-1.002212274	2.18127E-11	1.42147195	0.446367117	0.371240933	0.730359238	0.278299306	0.663868766
AT4G39460	SAMC1	-1.728963742	1.26948E-11	-1.321863542	8.78754E-11	-1.2829074	6.22045E-11	0.279629443	0.736631974	0.466744725	0.048245282	0.722137763	2.06514E-06
AT3G48610	NPC6	-1.730990909	6.29638E-06	-1.06923621	0.010654768	-1.519085206	4.93355E-07	-0.741017956	0.603726755	-0.52978793	0.195419415	-0.064939036	0.828812018
AT4G18970	AT4G18970	-1.731623056	0.000159299	-1.231904363	0.001158601	-1.708929605	2.27445E-14	-1.110460548	0.615358268	-0.643430637	2.14984E-05	0.403186696	0.006123544
AT1G18730	PnsB4	-1.732328158	0.000447641	-1.417015164	8.06311E-07	-1.662306919	4.29232E-19	1.037956359	0.549158023	0.246872754	0.735365463	0.28256261	0.377993158
AT5G19250	AT5G19250	-1.73618162	7.02195E-09	-1.26394975	5.56949E-06	-1.728834541	2.22243E-24	-0.399655553	0.66808962	-0.50969352	0.039484958	0.796106964	1.95423E-08
AT5G46570	BSK2	-1.742012675	0.007640362	-1.927427117	0.03287384	-1.404441419	9.0252E-05	-0.092381518	0.981845063	0.179176712	0.73812521	0.127521809	0.6151518
AT1G33811	AT1G33811	-1.745554633	4.25137E-08	-1.783136928	3.89449E-06	-2.867101075	1.92354E-26	-0.059907492	0.978989178	0.57949204	0.134697145	0.697198032	0.004098555
AT1G64760	AT1G64760	-1.745884656	3.00906E-08	-1.569251184	8.88498E-08	-1.594173799	4.82747E-08	-0.287878009	0.870238882	-0.137249305	0.812750957	0.87416507	6.64522E-06
AT4G24780	AT4G24780	-1.746934628	0.024035348	-1.924702305	1.36282E-12	-1.627659237	4.20062E-19	-0.239165855	0.926066852	-0.523350695	0.017459628	-0.825113372	0.000529782
AT4G21870	AT4G21870	-1.750616523	1.10819E-07	-3.310811883	3.51063E-14	-2.29691888	7.52511E-21	-0.158735965	0.953801756	0.450664891	0.590113348	0.76731909	0.017346461
AT4G31820	ENP	-1.751480881	4.92236E-12	-1.986458983	1.81624E-15	-1.590472407	6.0955E-25	0.272021666	0.682142539	0.233204284	0.433812805	0.332011718	0.025339825
AT4G34900	XDH2	-1.760293937	0.009376267	-2.094050834	8.1487E-10	-1.339070535	7.01651E-12	-0.092270092	0.957474117	0.009051307	0.992163494	0.618728261	4.12331E-05
AT4G34260	FUC95A	-1.760685523	1.3202E-06	-1.487166407	8.71038E-09	-1.013451591	2.50139E-12	-0.668196484	0.552973265	-0.111876469	0.719271526	0.340343667	0.016069934
AT2G22830	SQE2	-1.763083174	0.00795184	-1.847911556	0.002015194	-1.974984581	2.41845E-08	0.145628344	0.923225365	0.307563685	0.584915906	0.86809091	0.000277552
AT2G36050	OFP15	-1.7645393	0.000580108	-2.461561371	2.32032E-11	-1.60291377	6.57876E-07	0.125309402	0.938741823	-0.018148143	0.988694176	0.395512331	0.119220104
AT2G48070	RPH1	-1.767066368	5.24804E-08	-1.225243227	0.000117293	-1.258735295	5.15309E-11	-0.124695079	0.903468584	-0.230739742	0.572682273	0.652333673	0.001398627
AT1G31190	IMPL1	-1.772594991	4.25409E-05	-1.06046879	0.021322211	-1.036988846	0.000345175	-0.064315605	0.976040445	-0.019641318	0.984413209	0.052828476	0.890657428
AT5G52970	AT5G52970	-1.774732291	4.15527E-10	-1.295798242	3.51544E-07	-1.578519827	4.23653E-15	0.20637551	0.899191689	0.387638519	0.430288194	0.510601096	0.017501368
AT5G41410	BEL1	-1.776310591	0.003662679	-1.902189051	0.000260976	-1.626045903	7.4357E-11	-0.483680646	0.842538979	-0.376138143	0.579109484	-0.506776325	0.021317356
AT5G42650	AOS	-1.783230657	1.21533E-07	-1.526370446	1.60027E-15	-2.120889568	9.13447E-52	0.125561153	0.936447266	-0.189045969	0.675096916	0.557946639	5.09876E-05
AT3G18110	EMB1270	-1.789274448	0.003945059	-1.386966319	0.00856059	-1.278648822	4.31627E-05	-0.382498522	0.820360389	0.19503221	0.682011223	0.475780923	0.002730385
AT1G10830	Z-ISO	-1.792801808	0.000206765	-1.225399061	0.001732997	-1.1665878	0.000413154	-0.399089309	0.795944563	0.153494127	0.817744169	0.231367978	0.30548127
AT2G46225	ABIL1	-1.795273999	2.91058E-06	-1.327966983	3.84288E-07	-1.444285004	0.000264467	0.10250529	0.908634744	-0.104005466	0.857138563	0.678985174	9.03883E-05
AT5G23210	SCPL34	-1.798642064	1.0424E-07	-1.612307697	2.34604E-10	-1.105275303	0.000293977	-0.312292523	0.70850021	-0.353470201	0.261998738	0.035993867	0.877826292
AT1G32550	FdC2	-1.799257577	1.41528E-13	-1.341921917	7.56292E-08	-1.603067026	6.81768E-20	-0.116982739	0.962521414	0.590481459	0.113985559	0.551255946	0.011046891
AT5G40150	AT5G40150	-1.803212787	0.005568609	-2.367750837	0.003165524	-2.268694899	5.69814E-07	-0.524903286	0.754260213	-0.249979186	0.738186904	0.517724371	0.051352117
AT3G52960	AT3G52960	-1.805248266	1.65717E-10	-1.148885628	5.38886E-05	-1.54651447	1.42478E-49	0.061837844	0.95447214	0.01921431	0.975104725	0.595788702	4.80597E-07
AT5G15350	ENODL17	-1.805607632	3.91155E-06	-2.106005861	5.1779E-16	-1.625233718	2.01421E-09	-0.666957798	0.616300683	-0.517928555	0.003310922	0.160175151	0.435559662
AT3G05625	AT3G05625	-1.806238065	0.003390414	-1.509564884	0.013277805	-1.135818299	6.61522E-07	1.19710693	0.437721723	-0.022379405	0.985120284	0.87245886	9.34503E-05
AT5G04160	AT5G04160	-1.806851426	0.039699012	-3.628179287	0.003512977	-3.036013333	6.53007E-19	-0.613912089	0.686315536	-0.76902186	0.021397751	0.355395434	0.07513813
AT1G49975	AT1G49975	-1.809176931	6.25577E-05	-1.237114702	1.34028E-07	-1.264439057	7.14961E-08	0.510701051	0.721140128	0.650437514	0.191727682	0.779723067	6.29257E-05
AT2G42770	AT2G42770	-1.809367557	9.84582E-06	-1.15955809	3.39171E-05	-1.858187562	7.15313E-14	-0.168892428	0.919751416	0.311983057	0.564635774	0.299613264	0.268562815
AT1G20010	TUB5	-1.812652534	2.73554E-07	-1.805213033	0.007442193	-1.380807207	9.70978E-12	-0.266897981	0.751308246	-0.136695618	0.591655486	0.778690826	8.37649E-12
AT5G46270	AT5G46270	-1.815424349	0.009323916	-1.61046673	0.000113062	-1.530503091	1.6673E-05	1.156260704	0.633171846	-0.227048773	0.914287384	-0.229587879	0.50112546
AT3G59830	DEG8	-1.823473001	3.2476E-10	-1.010542905	1.05808E-05	-1.135526046	1.97865E-09	0.129784284	0.938292433	0.053794356	0.929498792	0.284259245	0.169292674
AT1G13210	ACA.l	-1.834435366	8.05084E-07	-1.355615536	0.003367878	-1.715942701	2.29567E-07	-0.608873395	0.144925486	-0.543479991	0.021013634	-0.08882083	0.739957182
AT1G78970	LUP1	-1.839039972	0.036169336	-9.401160184	2.55845E-14	-2.072649461	0.000131977	-0.457109953	0.792619066	-0.141496561	0.834876738	0.581574665	0.002748339
AT2G19400	AT2G19400	-1.841396857	0.008874226	-1.286209305	0.015935313	-1.39844489	0.004329192	0.470493543	0.844060401	-0.259745584	0.780129216	0.580986299	0.081762565
AT1G61580	RPL3B	-1.84212425	4.29531E-05	-1.91942144	1.17047E-07	-3.010218478	1.03908E-26	-0.150929264	0.937178328	-0.352716329	0.24016607	0.282360799	0.245089179
AT3G16530	AT3G16530	-1.849515155	0.007606206	-2.035010949	0.0001716	-2.79066008	1.71191E-09	-0.909186921	0.6158031	-1.046068224	0.172905467	-0.076804841	0.761927143
AT2G46820	PSI-P	-1.852564955	9.70654E-08	-1.357749445	1.11211E-27	-1.443631536	1.65661E-21	1.129784685	0.359733116	0.494876743	0.003157691	0.62834069	2.20337E-05
AT5G21060	AT5G21060	-1.853516509	0.000257938	-1.278947108	0.011159316	-1.72192314	6.80913E-15	-0.381608971	0.850582814	0.192738578	0.72447373	0.523161043	0.001093573
AT3G28180	CSLC04	-1.85944824	1.83776E-06	-1.991015308	6.40794E-13	-1.362207627	1.57217E-10	-1.058445382	0.183329115	-0.830894268	9.05806E-07	-0.061445904	0.786896594
AT2G24060	AT2G24060	-1.86019627	1.12814E-11	-1.030572678	0.00121771	-1.473291999	8.12368E-19	-0.102105898	0.911431059	-0.005675208	0.99349199	0.712493053	2.82491E-06
AT1G04680	AT1G04680	-1.866159873	0.009920704	-1.049667298	0.047899299	-1.278828991	2.5292E-05	-1.499569354	0.357892576	-0.857933424	2.78622E-10	-0.217831669	0.25483083
AT4G19985	AT4G19985	-1.874401331	0.000375001	-1.203201069	0.000774708	-1.836793929	7.95775E-13	0.110496341	0.971795048	0.159475593	0.820855399	0.85242672	1.47064E-05
AT5G54380	THE1	-1.891232685	4.15774E-08	-1.61832758	6.20599E-10	-1.103858701	0.000312253	-0.810780401	0.17999167	-0.696512702	0.001347568	-0.447032785	0.006777905
AT3G44620	AT3G44620	-1.893268243	4.51364E-06	-1.085625779	1.05834E-05	-1.31729103	6.18532E-10	-0.094422401	0.953774208	0.469395174	0.392285533	0.427141835	0.038176438
AT5G05740	EGY2	-1.895929504	9.94324E-16	-1.337119197	0.000335354	-1.747910172	3.63612E-32	0.348228902	0.639875152	0.06636073	0.908849666	0.746869883	4.82523E-07
AT1G70090	LGT8	-1.89605763	9.26577E-11	-2.100377253	5.36471E-14	-1.170229396	8.87247E-05	-0.352225095	0.6657277	0.1334462	0.730262291	0.465947696	0.078324223
AT1G45474	Lhca5	-1.898764007	2.54772E-08	-1.037886878	1.1042E-07	-1.598781744	7.44374E-13	0.199911589	0.926809464	0.11622966	0.868982135	0.415385187	0.115100412
AT3G18750	WNK6	-1.899920751	0.000479573	-2.146280012	0.012459497	-1.186009054	0.001021316	0.103771679	0.965260868	0.020664869	0.985862786	0.762104214	0.000162336
AT1G18550	AT1G18550	-1.904972599	0.002095621	-2.156459746	8.89428E-06	-1.861059698	0.0002986	-0.649906755	0.637815859	-0.210309906	0.770008565	0.518564472	0.023815555
AT5G63310	NDPK2	-1.906072458	8.98211E-16	-1.233390948	8.05929E-07	-1.808945381	5.79873E-35	0.17443334	0.874183021	0.293018684	0.252047337	0.714269432	3.1935E-07

AT4G10300	AT4G10300	-1.925162627	2.82954E-08	-1.326531472	1.16051E-07	-2.112220823	3.01626E-32	0.227594605	0.823064801	0.408381666	0.31206941	0.255504213	0.359138227
AT1G76100	PETE1	-1.925512468	2.96304E-09	-1.493332762	4.99313E-25	-1.558968048	1.29944E-16	0.644179723	0.653363031	0.281715441	0.485857955	0.457064531	0.00176219
AT4G17600	LIL3:1	-1.9267708	1.83416E-19	-1.378367875	0.000817203	-2.1727683	8.83472E-34	0.192238804	0.897609815	0.273174641	0.501566494	0.698329453	1.41743E-06
AT3G63410	APG1	-1.939681845	2.20628E-20	-1.226284234	2.66743E-09	-1.654282098	5.46298E-47	0.204835092	0.798570031	0.195437896	0.381537559	0.621189626	0.00069978
AT1G74730	AT1G74730	-1.943573068	1.75452E-10	-1.07980859	0.00103265	-1.851530301	7.03494E-18	0.312137417	0.839941064	0.520124792	0.371485466	0.734701913	3.25996E-06
AT5G17230	PSY	-1.946181714	9.41851E-10	-1.026909439	2.31386E-09	-1.287937274	9.14681E-10	0.419668349	0.711255369	0.186671087	0.576874111	0.540425313	2.77435E-05
AT3G18710	PUB29	-1.947703604	3.53428E-06	-2.078940843	6.85654E-06	-1.989584954	5.8616E-05	-0.28038248	0.828028997	0.05227068	0.964274323	0.920112519	2.68644E-08
AT5G28020	CYSD2	-1.949914971	2.10416E-13	-1.748008831	8.97574E-11	-1.769527439	2.94491E-14	0.085659081	0.918195684	-0.102454585	0.82339615	-0.271022391	0.229922658
AT1G15175	AT1G15175	-1.958529152	0.000848548	-1.242821033	0.004460287	-1.417160041	1.02231E-05	1.769089756	0.704225272	0.20379391	0.939586734	0.887243778	0.237929793
AT3G63490	EMB3126	-1.963288932	7.1721E-14	-1.172467086	1.06695E-10	-1.20779793	3.05877E-29	0.569523579	0.601921549	0.22144968	0.288283378	0.739035654	4.20452E-07
AT5G08280	HEMC	-1.966686686	1.59157E-18	-1.449440621	3.50845E-08	-2.178961691	1.29805E-55	-0.057424291	0.968021479	0.105130584	0.722577259	0.834715149	3.91027E-07
AT3G55760	AT3G55760	-1.966789081	5.74554E-07	-1.806796319	1.17937E-05	-2.02567111	2.60543E-11	-0.264010389	0.858622982	0.056868166	0.963495178	-0.367818968	0.13842478
AT1G76080	CDSP32	-1.974198856	2.99143E-07	-1.047253755	3.51755E-07	-1.474447916	2.34048E-25	1.141558198	0.552449115	0.227521799	0.741681948	0.357260402	0.201130155
AT3G44890	RPL9	-1.974921965	2.67227E-17	-1.251584059	0.000123637	-1.562365502	1.79502E-30	0.087667322	0.953801756	0.121524061	0.727051106	0.85970884	3.04397E-09
AT3G47070	AT3G47070	-1.976939154	6.91046E-06	-1.074782324	2.78331E-07	-1.719978074	2.6176E-22	0.767075662	0.517229302	0.415983427	0.355931055	0.508062161	0.072177876
AT5G23730	RUP2	-1.979399101	0.013178935	-2.119908829	0.000221401	-3.105159596	0.005504412	-0.25972235	0.929706774	1.048267019	0.157209744	0.058388685	0.915908135
AT1G35350	AT1G35350	-1.980448234	0.000827363	-1.772413582	1.15932E-11	-1.295851132	0.000405976	0.859191945	0.597380545	-0.329002624	0.563189301	0.048583842	0.912112487
AT3G55350	AT3G55350	-1.982984273	0.010669302	-1.437332052	0.00400022	-1.355771627	0.00390121	0.154757547	0.951334464	-0.083640277	0.936554664	0.612713705	0.002771955
AT5G62360	AT5G62360	-1.983865356	0.000321423	-3.837598032	6.19417E-20	-4.649948751	6.18596E-15	1.096684287	0.575257546	-1.105312614	0.371173545	0.515183818	0.319212196
AT2G26540	HEMD	-1.996629297	2.74221E-08	-1.717471467	2.91287E-09	-1.25369347	3.50867E-07	0.089354536	0.961276546	0.327667134	0.423442669	0.915910005	4.85341E-06
AT3G52150	PSRP2	-1.998285011	1.35637E-22	-1.306868394	2.4362E-08	-1.783451785	4.21764E-32	0.342604206	0.66808962	0.29590407	0.268885385	0.835750374	2.58297E-10
AT3G23805	RALFL24	-1.998618121	6.79883E-05	-1.400215397	0.000175408	-2.682656286	5.38271E-12	-0.341241846	0.866896535	0.239659585	0.578599682	0.518074016	0.046227737
AT4G17460	HAT1	-2.002546173	8.9218E-09	-2.096510239	1.68456E-13	-1.10703964	0.000992552	0.572801116	0.62738272	-0.158277478	0.831323166	0.311173649	0.144157988
AT2G17230	EXL5	-2.006921395	1.21128E-09	-2.374732038	1.61184E-08	-1.30400623	0.000576449	-0.478849363	0.608678066	-0.512587154	0.096474964	-0.481159692	0.073146518
AT1G77590	LACS9	-2.007493465	0.001887202	-1.961373678	4.36195E-08	-1.749907035	2.06103E-15	-0.359774842	0.758174418	0.034211107	0.958669515	0.559224132	3.50857E-05
AT1G70940	PIN3	-2.014420893	5.49047E-12	-1.466147992	1.14584E-18	-1.069659428	2.38721E-20	-0.647201892	0.073310574	-0.388570234	0.041856703	-0.374921878	0.010418116
AT3G06770	AT3G06770	-2.017856455	0.000502151	-1.882450051	9.07801E-10	-1.363309064	0.000873308	-0.520733235	0.691239307	-0.174925121	0.712688665	0.413367227	0.007296468
AT2G34860	EDA3	-2.021340761	1.53071E-16	-1.12610784	5.19454E-09	-1.765313306	1.10907E-42	0.244044002	0.807833592	0.113216758	0.807923242	0.469540696	0.015180377
AT3G07010	AT3G07010	-2.02167279	0.00029498	-1.859703949	0.000667366	-1.040624976	0.000485096	-0.761964315	0.610227484	-0.63923177	0.00514006	0.088617049	0.666580432
AT3G10940	LSF2	-2.02485136	0.000543372	-1.643667235	0.001598523	-3.145179046	1.7965E-10	-0.1940986	0.978216445	0.156765023	0.802413998	0.698730027	0.003836459
AT2G21370	XK-1	-2.027642673	2.07207E-08	-1.834171785	1.13795E-11	-1.188999051	5.71286E-10	0.206963193	0.883128604	0.243625634	0.74053286	0.79961174	1.63591E-05
AT1G75460	AT1G75460	-2.028551888	2.5688E-08	-1.532445114	2.11158E-10	-1.325985704	1.52728E-17	0.966858397	0.691895802	-0.602227887	0.323767852	-0.38367416	0.200086098
AT4G18480	CHLI1	-2.033123637	5.78102E-26	-1.29239402	2.4054E-14	-1.853093371	5.2811E-24	0.303841623	0.735038565	0.23259274	0.426711349	0.524494145	4.20258E-05
AT1G78915	AT1G78915	-2.044793829	2.64721E-09	-1.53723729	0.001295121	-1.203842733	2.1854E-09	0.391248341	0.719629226	0.278800863	0.662985311	0.754456807	6.35208E-05
AT4G37080	AT4G37080	-2.052085283	3.27093E-14	-2.332691075	1.68182E-05	-1.524226835	3.28872E-09	-0.099578715	0.95447214	-0.144020357	0.745446425	0.000735313	0.997852969
AT4G36530	AT4G36530	-2.054213233	1.34628E-08	-1.314544387	9.94292E-05	-1.114084377	2.6638E-10	0.39886177	0.731041294	0.10743272	0.885413453	-0.010023506	0.977013861
AT1G66970	SVL2	-2.063770676	2.56631E-08	-1.2611102	5.06122E-07	-2.193927134	2.54256E-24	0.897528381	0.541614164	0.009601071	0.992575938	0.994430275	0.000444833
AT1G76160	sks5	-2.072025002	6.00473E-19	-1.493763034	2.58736E-07	-1.152308671	5.60959E-08	-0.899247298	0.433558429	-0.472807567	0.020225889	0.377830185	0.031582005
AT1G23480	CSLA03	-2.075786763	2.07167E-07	-2.857060679	6.25562E-10	-1.612299988	1.60216E-05	-0.240699804	0.870309077	-0.282320776	0.494228383	0.473518043	0.002770756
AT1G50320	THX	-2.079476232	1.27465E-10	-1.068859211	3.01807E-08	-1.25300747	2.86178E-10	0.06355484	0.972495118	0.203091883	0.695325627	0.222900508	0.380071138
AT2G47440	AT2G47440	-2.081219505	1.17575E-06	-1.760489626	6.83011E-12	-1.028638751	0.002001699	-0.661187513	0.598901706	-0.296712259	0.452099003	0.097773449	0.774642389
AT4G10770	OPT7	-2.082326572	1.99545E-07	-1.787545046	0.001947018	-2.49763446	1.1264E-13	-0.704727987	0.735038565	-0.095613671	0.917278038	0.266529583	0.227478712
AT2G19170	SLP3	-2.085790905	0.000171669	-1.832793652	0.00168929	-2.418747496	1.49371E-05	-0.734742251	0.708641472	-0.195436694	0.809048267	0.489245302	0.009524537
AT3G55330	PPL1	-2.086675579	4.82776E-09	-1.77220247	1.42815E-18	-1.436939214	3.80736E-26	0.736798389	0.589531904	0.243372103	0.592311926	0.874956903	1.34297E-06
AT5G64290	DIT2.1	-2.08691847	4.43108E-13	-1.107238616	1.6595E-06	-1.756579589	5.34385E-19	-0.224277502	0.8208148	-0.371268078	0.098116414	0.272325008	0.197373814
AT3G21580	ABCI12	-2.088336748	0.001596484	-1.611612837	0.015140197	-1.303106109	2.6663E-05	-0.321825466	0.820406788	-0.389163223	0.64641767	0.04882305	0.913318762
AT2G34430	LHB1B1	-2.08980287	1.00609E-06	-2.271380013	5.838E-34	-1.161199453	3.96764E-08	5.906359269	0.173318686	1.185799167	0.498449509	0.63902965	0.385722432
AT3G06140	LUL4	-2.094617479	0.02176655	-3.257302905	0.006744073	-1.62406203	4.25261E-05	-0.089569344	0.972495118	-0.199311341	0.834876738	0.656999571	0.017220865
AT5G54600	RPL24	-2.102739455	8.21947E-23	-1.137808191	6.74707E-11	-1.330397636	2.51565E-27	0.399750467	0.611226747	0.256172225	0.491449838	0.802040167	2.35781E-07
AT1G29530	AT1G29530	-2.106117413	6.75089E-06	-3.742246597	0.000978818	-2.509496262	2.64927E-09	0.098636283	0.976635648	0.342477747	0.625207384	0.703586123	0.02006053
AT5G01590	AT5G01590	-2.10770627	2.32676E-17	-1.372440238	5.71901E-05	-1.385165156	2.67484E-24	-0.188600286	0.8853319	-0.04823148	0.914359122	0.494837603	8.65079E-06
AT3G15520	AT3G15520	-2.109713432	6.94046E-10	-1.270496091	1.29186E-09	-1.645005772	5.52608E-26	-0.194878855	0.869437373	0.062193337	0.951707362	0.759582644	0.000250571
AT1G73110	AT1G73110	-2.116857953	3.53509E-08	-1.075963977	0.006166992	-1.096495597	2.22051E-06	-0.015435368	0.995959328	0.389485264	0.354763354	0.503754687	0.004738956
AT5G13770	AT5G13770	-2.118931637	5.73017E-05	-1.466157827	1.43439E-18	-1.514856342	7.92012E-11	1.637668581	0.455598103	0.439788735	0.553652001	-0.007406812	0.988971078
AT4G27700	AT4G27700	-2.124732589	1.63926E-07	-1.406898754	3.00204E-14	-1.800442975	6.12912E-18	1.088489051	0.55451616	0.257107769	0.608418199	0.523767408	0.007236345
AT5G28500	AT5G28500	-2.125403136	3.12612E-19	-1.413206939	9.14878E-09	-1.799460227	1.8737E-26	0.158722093	0.863039271	0.282133621	0.239346148	0.199110749	0.142915702
AT2G40520	AT2G40520	-2.130320378	0.009267539	-2.040638095	0.006003503	-1.558500355	0.0001205	0.854402299	0.665832077	0.699865787	0.397712133	-0.122473223	0.838054433
AT5G65120	AT5G65120	-2.135060164	9.74488E-05	-1.739727014	0.019817217	-1.523313036	0.042232039	0.243111275	0.936848796	0.496073478	0.483585041	-0.149370497	0.693084646
AT3G24430	HCF101	-2.15215526	2.58809E-16	-1.525722758	8.83468E-07	-1.60585001	5.8394E-42	0.613998234	0.594299218	0.192523944	0.625031289	0.933068339	1.33759E-09
AT2G33330	PDLP3	-2.154599644	0.001500041	-3.174427252	8.09656E-06	-1.884715223	8.6756E-10	-0.808504723	0.758815467	-0.031500046	0.963874517	0.482775962	0.008165482
AT2G36430	AT2G36430	-2.158347718	3.3671E-15	-1.683315059	2.11708E-07	-1.901349167	1.68401E-06	-0.313945874	0.76896842	-0.114624373	0.861976294	0.021061518	0.960024088
AT5G19600	SULTR3;5	-2.164394115	0.012845249	-2.659217228	0.000122595	-1.566895825	0.000110693	-0.496903405	0.837258505	-0.456236499	0.158543269	-0.581246455	0.000245675
AT5G57170	AT5G57170	-2.168073239	0.000206824	-1.396230487	0.000486858	-1.877450219	5.29634E-12	0.063255353	0.982749202	0.156380945	0.883566526	0.704038774	0.0045265
AT2G23130	AGP17	-2.170345335	2.6577E-05	-1.402859066	0.00154575	-1.183497479	0.024283256	-0.299835027	0.73148555	-0.362220623	0.544656196	-0.113383591	0.849218537
AT2G43550	AT2G43550	-2.17049435	7.2271E-08	-2.149701346	0.000512076	-2.327021718	3.85651E-17	0.488117112	0.718095285	-0.254085776	0.856243901	-0.050114942	0.922593242
AT2G19800	MIOX2	-2.171109247	1.28611E-06	-2.859357254	0.003378323	-1.401362157	0.007987279	1.392493521	0.393926565	0.014346285	0.985862786	-0.000173882	0.99962369
AT1G01790	KEA1	-2.173315308	7.53557E-11	-1.869068903	6.17823E-14	-1.106920595	3.68338E-19	0.356566537	0.837694719	0.292705116	0.39039795	0.258768556	0.225850344
AT2G32100	OFP16	-2.185254972	0.000725901	-1.849776918	0.005843818	-2.203975072	7.75237E-08	0.748995267	0.732322487	0.427966282	0.779388526	0.729716076	0.121567179
AT5G52882	AT5G52882	-2.190345127	1.89523E-09	-2.683909603	0.000144657	-1.248629836	1.65997E-14	-0.549032032	0.721140128	-0.318501216	0.254066296	-0.055723716	0.771382959
AT3G16370	AT3G16370	-2.192613294	1.63354E-13	-1.475845306	1.67416E-07	-1.409456412	1.01898E-31	-1.026729647	0.326457456	-0.877089391	0.076998555	-0.329414305	0.081412227
AT5G60020	LAC17	-2.198267606	0.003556985	-1.345730939	0.040026795	-1.294042876	0.034809296	0.266747616	0.827716472	-0.453923851	0.53583656	-0.257917214	0.660939303
AT3G27830	RPL12-A	-2.198856614	2.52517E-18	-1.34842253	6.24843E-19	-1.545551741	4.04499E-31	0.243105912	0.748481821	0.098804784	0.856778872	0.79889935	2.35572E-05
AT1G23030	AT1G23030	-2.19941649	1.56625E-06	-1.913469871	3.80233E-05	-1.251915406	0.000132116	-0.159791643	0.851674502	-0.309565349	0.345339837	0.243738693	0.127088817
AT3G15110	AT3G15110	-2.203092101	3.35273E-13	-1.223799947	0.00105532	-1.045078501	1.22925E-09	0.670128268	0.6107328	-0.304838085	0.56302106	0.596864127	0.044510291
AT5G57700	AT5G57700	-2.215263317	0.000521249	-1.361182651	0.018082302	-1.836140043	2.8124E-13	-0.153824564	0.966434558	-0.392269849	0.319571819	0.356229054	0.017969013
AT3G11090	LBD21	-2.220221623	4.77583E-05	-1.968486462	0.003886211	-2.371679592	7.95797E-08	-0.873220927	0.697179773	0.434779586	0.788234057	-0.406791811	0.611046505
AT2G04039	AT2G04039	-2.22365549	1.13575E-07	-1.250550423	0.000741621	-1.288244034	7.17387E-07	0.652727744	0.777104408	-0.112624615	0.958669515	0.253951734	0.600693778
AT1G75690	LQY1	-2.223789417	1.53704E-13	-1.421026515	4.087E-14	-1.686739077	1.85711E-34	1.172317088	0.430181703	0.6158088	0.062240516	0.852167902	1.04476E-07
AT4G28720	YUC8	-2.228605705	0.000141823	-2.514647125	1.365E-05	-1.894846181	1.55208E-13	-0.627218185	0.726214276	0.359935965	0.515641671	-0.61750941	0.004220858
AT3G56650	PPD6	-2.230616198	6.73041E-08	-1.0758372	1.17735E-06	-1.379036307	1.04547E-22	0.448060752	0.702246294	0.519336927	0.347196014	0.761648833	6.30121E-05
AT1G26810	GALT1	-2.242752674	0.001298964	-1.615865312	0.012560236	-1.22911242	0.008400707	-0.030356772	0.994744545	0.196885704	0.797919251	0.501099606	0.024122815
AT2G45470	FLA8	-2.242790893	2.95236E-08	-2.063049344	2.16206E-10	-2.588499607	2.38506E-65	-1.042406999	0.533620946	-0.82628481	3.71168E-06	0.376803733	0.12564353
AT4G25050	ACP4	-2.245689573	9.3545E-24	-1.598185085	8.14714E-30	-2.211049205	3.74816E-22	0.197450837	0.793923375	0.166521086	0.579588108	0.457118448	0.006185917
AT3G24515	UBC37	-2.251361467	0.012632146	-1.521642173	0.039946783	-2.861523905	0.026892429	-0.608593927	0.647947756	0.134984203	0.907594619	0.266079907	0.657946163
AT3G01810	AT3G01810	-2.257344729	3.02536E-08	-2.165133554	0.000699222	-1.858086166	8.28736E-22	-0.32380874	0.776988689	-0.073315663	0.911846326	0.938096457	1.3412E-09
AT2G24390	AT2G24390	-2.258914399	0.000819988	-1.817780614	0.006336628	-1.381429615	0.00182271	0.58262243	0.816446062	0.004047814	0.998107647	0.241911681	0.513027724
AT5G35630	GS2	-2.262577076	1.09821E-27	-1.398671445	1.30522E-08	-1.498881357	2.87486E-50	0.479017104	0.680482607	0.121997592	0.686941393	0.939749839	3.4017E-13
AT1G49750	AT1G49750	-2.265042189	2.94608E-15	-1.501110704	3.30267E-06	-2.109420878	2.30678E-28	0.162719544	0.911509594	-0.035115666	0.95946592	0.541615524	0.006814628
AT1G22690	AT1G22690	-2.266485118	1.89828E-05	-2.203600327	1.28372E-14	-2.719821997	1.72218E-33	-0.222032422	0.943633329	0.122020747	0.888052136	0.701832306	0.012383254
AT1G68590	PSRP3/1	-2.275291425	9.95434E-22	-1.371681067	0.000793451	-2.197138177	8.72468E-39	-0.035010935	0.989481773	0.261728826	0.542006423	0.872077113	2.75318E-05
AT1G03870	FLA9	-2.27529923	1.2206E-06	-2.386369412	0.008767675	-1.843719522	0.003204996	-0.902892372	0.413866874	-0.904355739	2.57497E-06	0.126490402	0.600693778
AT5G55220	AT5G55220	-2.277737157	3.86864E-32	-1.522413838	3.7811E-08	-1.838797624	1.15773E-31	0.120531602	0.953774208	0.322280323	0.115937316	0.643294195	2.32542E-09
AT2G28760	UXS6	-2.286655274	0.030059128	-2.125673697	0.00010772	-1.993730164	0.00886843	-0.740907064	0.885840009	-0.490691436	0.177595922	-0.054090348	0.827435441
AT4G22890	PGR5-LIKE A	-2.289724131	5.39944E-11	-1.541011434	5.79337E-33	-1.479463221	2.36624E-30	1.40498241	0.279591669	0.350782692	0.218260485	0.55512339	1.42374E-05

AT4G04330	RbcX1	-2.292828622	0.000224233	-2.176190674	6.19688E-16	-2.026154738	1.71515E-13	3.658280869	0.459472858	-0.173610537	0.983142472	-1.155179105	0.531926123
AT1G56190	AT1G56190	-2.29316041	1.10465E-12	-1.383091468	5.4848E-05	-1.66887007	3.90436E-28	0.093633088	0.947061206	0.079506353	0.873118803	0.63441834	2.86071E-06
AT1G22630	AT1G22630	-2.295753199	0.000638904	-1.81987822	6.44039E-08	-2.280166559	1.07933E-15	1.312308222	0.373684124	-0.330912852	0.7682641	0.162324624	0.790324824
AT1G31800	CYP97A3	-2.29601595	4.1747E-12	-1.691445745	1.51919E-07	-1.475511752	2.3102E-10	0.243253825	0.845932274	0.020301941	0.979763766	0.890473684	1.03648E-06
AT1G27460	NPGR1	-2.298229673	3.12649E-11	-2.498099129	2.61329E-23	-1.042287339	1.8991E-06	-0.042053033	0.982749202	0.123261602	0.805320688	0.485323539	0.008117977
AT2G06520	PSBX	-2.299689575	1.2574E-09	-1.577120606	4.01673E-26	-1.377085969	1.83448E-39	2.021145671	0.118851064	0.388047973	0.204941626	0.907661121	1.68778E-07
AT2G03750	AT2G03750	-2.305044732	2.49354E-07	-2.014435786	1.42077E-10	-1.508836053	1.3397E-07	1.067538869	0.684321129	-0.387556634	0.650438754	0.799450221	0.003069603
AT4G14680	APS3	-2.30880649	1.81367E-09	-1.538304827	0.00689583	-2.631806727	5.62711E-15	0.198334659	0.899858481	-0.032410791	0.975427751	0.481044277	0.049560441
AT1G23750	AT1G23750	-2.314373559	0.004054865	-2.784340953	0.000679077	-1.484064343	5.30412E-07	0.432584955	0.870926643	0.404795965	0.618270343	0.298546863	0.26693317
AT5G45930	CHLI2	-2.323276269	6.98225E-18	-1.543403507	0.008398015	-2.215158089	3.4386E-25	0.036226396	0.982734229	0.470629337	0.133573568	0.84082418	1.85608E-07
AT4G32590	AT4G32590	-2.324420061	2.60948E-10	-2.011087347	2.14572E-11	-1.446307427	2.01044E-24	0.809521316	0.652047062	0.596345493	0.326937807	0.749555536	0.002273568
AT3G11630	AT3G11630	-2.338791041	3.91336E-25	-1.34005782	1.91264E-06	-1.748413141	3.83354E-35	0.300481339	0.704470077	0.128251487	0.647096367	0.968110841	1.62461E-06
AT2G10940	AT2G10940	-2.344812826	9.07728E-13	-1.838637252	4.50023E-12	-1.646233674	4.35475E-21	-1.102709155	0.29756507	-0.928993027	8.46438E-09	-0.1200992	0.600118624
AT1G05630	5PTASE13	-2.349847673	5.48764E-05	-1.518447234	0.022557563	-1.364324453	4.17662E-05	-0.239844988	0.886397192	0.114810261	0.857138563	0.232959152	0.314697952
AT3G12780	PGK1	-2.364338479	3.68937E-29	-1.487769791	1.64417E-11	-1.698778596	1.23808E-84	0.629687474	0.598901706	0.143435006	0.67277392	0.767157676	2.29071E-09
AT4G18760	RLP51	-2.406731599	0.000153518	-2.544368094	0.000314923	-1.615985718	0.001804013	-0.719551908	0.63192307	-0.586463093	0.112330215	-0.225904213	0.522126605
AT3G03780	MS2	-2.409547198	0.000213255	-1.0865111	0.031137319	-2.16892195	2.06095E-10	-0.630976451	0.815270201	-0.690839253	0.000602496	0.24642797	0.257867235
AT1G43560	ty2	-2.412192079	7.31057E-15	-1.53877385	1.91151E-12	-1.847896125	4.01324E-17	0.045336363	0.977607985	-0.026339098	0.969038524	0.208981593	0.479443633
AT3G01480	CYP38	-2.419964801	3.76094E-22	-1.975714097	3.83151E-30	-1.651977833	2.90482E-41	0.806382178	0.507877122	0.175173552	0.55798645	0.887227515	4.96245E-12
AT1G14150	PnsL2	-2.429415964	1.96934E-10	-2.513094375	1.21701E-07	-1.85997955	4.75485E-40	0.314045106	0.956442215	-0.267475884	0.865063654	0.804734136	0.070820413
AT3G02870	VTC4	-2.434660506	4.79316E-07	-1.613622446	1.28367E-05	-1.584106443	7.65699E-05	-0.614554005	0.732322487	0.121696152	0.845336685	0.311832028	0.095237113
AT3G52520	AT3G52520	-2.434931858	0.000619564	-1.988992609	0.007295982	-3.993986442	0.000170258	-0.229632628	0.935532809	-0.949467206	0.155596055	-0.008863329	0.989331152
AT3G58120	BZIP61	-2.439910711	2.29369E-13	-3.133308106	4.77039E-05	-2.162587956	0.002521835	-0.17271795	0.861588972	-0.20560666	0.489234293	-0.200287817	0.326952551
AT1G44000	AT1G44000	-2.458537562	5.77727E-09	-1.693137148	1.48034E-08	-2.025084689	6.06714E-11	0.329450804	0.731041294	0.445449738	0.206705742	0.535711757	0.002802331
AT1G04160	XIB	-2.460894013	8.17996E-05	-2.710676716	6.67179E-06	-2.936070757	1.9354E-11	-0.698089595	0.622596296	-0.532066617	0.266105627	0.207616319	0.442661691
AT2G21280	SULA	-2.461924564	8.7698E-13	-1.116413981	0.000300968	-2.064375669	1.13718E-33	0.398738134	0.721140128	0.508634465	0.380878661	0.984514435	4.49558E-07
AT4G12980	AT4G12980	-2.465386264	6.94902E-07	-2.048538815	0.000239165	-1.714453953	6.93482E-15	0.127693071	0.950437376	-0.403937828	0.524973096	-0.535006761	0.095742677
AT2G19650	AT2G19650	-2.466674029	0.003397158	-2.364542774	3.84637E-05	-2.631702565	9.47132E-05	-0.480439093	0.855427008	-0.128731319	0.958669515	0.066365766	0.908125061
AT2G34060	AT2G34060	-2.475844498	7.61908E-05	-1.635727933	0.012281731	-1.408331286	0.009208288	0.357456976	0.766791062	0.190807014	0.857245979	-0.216350196	0.637764107
AT1G16720	HCF173	-2.480645556	6.74328E-13	-1.645221587	7.57195E-15	-1.550147401	1.70374E-24	0.917093563	0.587820092	-0.185932656	0.738824444	0.259941738	0.182362611
AT4G24930	AT4G24930	-2.491124103	1.90229E-16	-1.256370752	1.95085E-05	-1.990967956	2.71512E-16	0.278224327	0.833592548	0.149875556	0.800997359	0.994806237	5.15907E-06
AT2G42130	AT2G42130	-2.491353376	1.04337E-08	-1.112967583	9.67402E-07	-1.813872752	1.14419E-14	-0.132057667	0.942141085	0.103566134	0.868188276	0.82423224	1.48061E-07
AT3G04790	EMB3119	-2.515829042	5.93531E-16	-1.631902945	0.000455767	-2.695330986	1.38506E-34	-0.192354528	0.85072665	-0.179444146	0.662608549	0.318011904	0.057916918
AT2G03590	UPS1	-2.520629098	0.013438451	-3.654933383	1.31516E-06	-1.530626643	3.92482E-05	-0.074167473	0.982529985	-0.549269531	0.592718867	-0.040898231	0.939455384
AT1G78230	AT1G78230	-2.531491131	1.0928E-07	-1.732639749	3.0561E-05	-2.096261564	3.01576E-06	-1.185472588	0.602119156	-0.740378749	0.423442669	-0.732959666	0.036539543
AT1G10930	RECQ4A	-2.540441673	9.71638E-05	-1.345810335	7.74689E-06	-1.606962936	4.28056E-09	-0.657652552	0.751308246	-0.192964504	0.790281754	0.257297672	0.208912128
AT5G66190	FNR1	-2.555720955	1.00555E-31	-1.631069585	6.84414E-35	-1.783609473	1.4203E-39	0.517591214	0.539736869	0.233805941	0.405816794	0.563502449	6.30842E-06
AT3G53900	UPP	-2.557541233	3.39837E-06	-2.353470471	1.2521E-10	-3.434070285	1.84592E-50	0.229471406	0.93556951	-0.05315295	0.963495178	0.890663272	1.85899E-05
AT5G07020	AT5G07020	-2.559120339	2.26224E-23	-1.633259618	1.16806E-16	-2.11245384	7.75535E-65	0.221310391	0.855628531	0.47426448	0.185313217	0.665257703	2.50542E-06
AT1G55480	ZKT	-2.567914241	1.03067E-15	-1.721564032	1.62442E-12	-1.709056505	1.71666E-15	0.967358739	0.598901706	0.271680499	0.573776086	0.947980807	1.1892E-08
AT5G05580	FAD8	-2.56928262	4.74201E-08	-2.277663865	0.003446119	-2.076828664	6.83352E-07	-0.415742511	0.8351738	-0.289624586	0.67569451	0.749595868	0.017841859
AT2G01590	CRR3	-2.579607142	1.21067E-09	-1.491473931	3.47718E-05	-1.740929159	3.51078E-14	-0.401081517	0.813275626	-0.685309189	0.445806954	0.43687827	0.165100333
AT1G03630	POR C	-2.582255229	6.92037E-25	-1.841915149	7.89153E-10	-2.342566034	4.32561E-24	0.810221544	0.553691236	-0.111080409	0.883027727	0.979332293	2.76895E-08
AT5G35970	AT5G35970	-2.610552513	1.46489E-15	-2.026824489	1.50744E-25	-1.409242288	3.12468E-25	1.393789888	0.437207517	0.144394962	0.794004103	-0.27327263	0.266355986
AT3G52750	FTSZ2-2	-2.62055958	2.60656E-05	-1.620941633	5.08941E-05	-2.381139821	8.44946E-17	-0.246287515	0.888695133	0.291627683	0.593522002	0.674557828	2.1966E-05
AT1G68260	AT1G68260	-2.638724804	3.67898E-09	-2.185347564	0.002334444	-2.055074982	1.53171E-08	-0.053680799	0.981281754	-0.137377489	0.878562017	0.365262442	0.314902887
AT2G03530	UPS2	-2.64226158	2.74756E-11	-1.964386941	5.1099E-11	-2.246975003	2.15782E-21	-0.773326889	0.599399361	-0.538369295	0.447139821	-0.637017489	0.077164626
AT4G38970	FBA2	-2.65354674	4.3539E-16	-1.636779912	1.68805E-17	-1.103978211	4.62457E-25	1.093043628	0.50266835	0.251299724	0.373750819	0.598422617	4.28302E-05
AT3G10840	AT3G10840	-2.6597534	1.84037E-05	-1.994294593	0.008807511	-2.666639116	3.72085E-09	-0.716438651	0.89521463	-0.210511118	0.848756997	0.772685407	0.012771285
AT5G36120	CCB3	-2.661945241	4.56606E-12	-2.577161327	8.27926E-05	-3.75636144	3.58098E-31	-0.164921047	0.951502645	-0.211452725	0.888052136	0.866563059	0.003810721
AT2G32560	AT2G32560	-2.670897082	1.40869E-12	-2.163475575	7.32332E-07	-2.295070311	1.55727E-16	0.050698127	0.982749202	-0.06596432	0.938703599	0.17160282	0.638526864
AT1G29700	AT1G29700	-2.673830548	1.39845E-08	-1.3652144	0.000180025	-1.549412828	1.63885E-12	1.334328727	0.409180759	0.045310959	0.97061131	0.474096987	0.277372605
AT3G54050	HCEF1	-2.685201343	3.27357E-17	-2.041487501	5.37879E-30	-1.717717014	3.19616E-44	1.986733511	0.133670218	0.613085922	0.041984649	0.747567149	0.001369676
AT5G21430	NdhU	-2.716585912	4.19333E-08	-2.421192809	2.67099E-12	-1.584887472	9.81938E-24	1.010135914	0.775318056	-0.415488546	0.739028089	1.090259451	0.161309091
AT5G02540	AT5G02540	-2.718444598	1.71946E-12	-1.485955471	0.001563968	-2.6777651	2.07309E-08	0.33068372	0.837207287	0.964920355	6.19886E-12	-0.183903381	0.370430043
AT2G39730	RCA	-2.730182839	8.24251E-17	-1.564969807	1.64196E-32	-1.52422579	1.93495E-26	1.174761136	0.527816001	0.176808633	0.574844643	0.633425283	2.40049E-05
AT3G22060	AT3G22060	-2.730291255	5.18592E-09	-2.989057718	4.86885E-16	-2.219415186	4.61299E-31	2.165102277	0.647947756	1.651481406	0.53583656	-0.484070908	0.599215829
AT1G12900	GAPA-2	-2.733497317	9.47269E-19	-1.906048937	6.13601E-28	-1.365527223	5.46777E-24	1.039538086	0.367739121	0.425910976	0.053599348	0.867480395	5.31495E-08
AT1G42970	GAPB	-2.738520903	4.67145E-24	-1.772017345	1.75929E-26	-1.154588648	1.69257E-18	0.787438006	0.561207188	0.431820157	0.071042761	0.620405164	1.98242E-05
AT1G22330	AT1G22330	-2.758969045	1.89724E-05	-3.590675007	4.83404E-18	-2.380367595	0.003455121	-0.313291704	0.820789421	-0.260385715	0.589186505	0.734807424	0.00069978
AT1G29920	CAB2	-2.81153071	7.14307E-13	-2.337554822	5.30318E-67	-1.509662107	3.97705E-21	4.362223521	0.10723022	1.305769907	0.251947034	1.128640932	0.22921049
AT1G52510	AT1G52510	-2.815713107	7.60117E-16	-2.138837596	1.36147E-10	-1.812822502	1.66751E-33	1.161295522	0.48633216	0.141942183	0.878918096	0.5445437	0.018913332
AT5G39860	PRE1	-2.820781505	2.95467E-08	-2.153087404	8.83152E-07	-1.897136297	6.34554E-08	-0.285851817	0.930136802	-0.338465756	0.811222926	-0.145904764	0.609217851
AT1G76740	AT1G76740	-2.831358233	0.00157606	-3.415218967	0.007974686	-2.310370039	0.006332777	0.5576416	0.928888991	0.591297992	0.630424977	0.100765339	0.800776293
AT2G33530	scpl46	-2.85029984	5.83079E-07	-1.476052557	0.005118303	-2.123172007	1.31386E-07	-0.333675173	0.85072665	-0.281287068	0.721359573	-0.34770252	0.136293911
AT5G64770	RGF9	-2.853696016	3.13602E-08	-2.777983543	7.53725E-12	-3.072532057	1.03481E-34	1.266523884	0.48198562	-0.56230711	0.711491646	0.063483987	0.90073822
AT5G38420	RBCS2B	-2.87140788	1.86788E-16	-1.581820167	0.00255727	-1.284760114	5.94888E-22	1.196238329	0.366891789	0.583212612	0.224470834	0.688494732	0.000921074
AT1G61190	AT1G61190	-2.887622177	2.5466E-08	-1.731108044	1.2042E-14	-1.307321578	0.000484782	0.777293993	0.64010149	0.017154985	0.991477195	0.455795104	0.015274754
AT5G48300	ADG1	-2.889184117	7.60117E-16	-2.270366439	3.48394E-13	-2.98118785	2.17299E-42	0.440679457	0.597380545	0.266902918	0.451926386	0.923811459	1.20695E-11
AT5G50950	FUM2	-2.90405185	4.21155E-06	-2.531769789	3.93929E-21	-2.377346174	8.45688E-12	1.239663683	0.663725004	-0.249415322	0.820854563	0.277120798	0.521696098
AT3G53680	AT3G53680	-2.908307255	1.0504E-07	-3.007642507	0.015980825	-2.138796655	0.002431929	0.026482443	0.993804788	0.207048804	0.838103967	0.171312977	0.671352376
AT1G16500	AT1G16500	-2.908617222	0.000147764	-3.193443716	0.008648397	-3.529993285	0.000501332	-1.292562878	0.714886717	-0.030823091	0.985862786	-0.328578403	0.616626748
AT4G37770	ACS8	-2.915193581	5.00802E-13	-2.421427637	0.001485635	-3.64983949	3.28375E-15	-0.150745684	0.963305035	0.30770364	0.603418453	-0.155090182	0.702965815
AT1G73870	BBX16	-2.922472239	8.57028E-08	-2.729369201	1.16051E-07	-2.946835208	3.58085E-12	3.047275209	0.574003303	1.277374321	0.871401797	3.036350088	0.633477293
AT1G09340	CRB	-2.933788919	1.40105E-16	-1.856577133	4.66672E-18	-1.872443024	2.67035E-46	1.110995263	0.50075507	0.162044543	0.695385801	0.929935086	1.87145E-09
AT4G09650	ATPD	-2.951635997	1.59542E-26	-1.845744036	3.36845E-18	-2.56315437	7.12767E-45	0.26707581	0.782181302	0.43540475	0.566973384	0.544305992	0.000909057
AT5G20935	CRR42	-2.95268016	0.008512584	-2.402886073	0.013363032	-1.811904466	0.001757499	0.380759043	0.948761383	-0.105283258	0.97325662	-0.000639664	0.99943512
AT4G28660	PSB28	-2.960281188	9.01725E-22	-2.025846014	3.81325E-10	-2.478105617	1.00954E-40	0.743123221	0.585445788	0.331203216	0.578901234	0.58659147	0.006226693
AT5G44680	AT5G44680	-2.973293908	2.92594E-09	-3.727030154	6.70077E-23	-2.261870165	7.78919E-29	-0.413772145	0.808272271	0.103106283	0.824002281	-0.030557965	0.884676064
AT3G06145	AT3G06145	-2.986237845	0.017418413	-9.194608684	8.50454E-08	-1.753399689	0.046223558	0.182777607	0.968021479	1.116067367	0.312439002	0.262908014	0.57342539
AT4G24350	AT4G24350	-3.008264493	5.38888E-09	-4.665285003	2.99329E-08	-1.235514808	2.78034E-06	1.887465444	0.086497474	-0.870988236	0.525334184	-0.254881038	0.710929795
AT4G30610	BRS1	-3.020019761	7.49046E-06	-2.578780099	6.72065E-06	-2.578355296	2.24542E-08	-0.655431131	0.637815859	-0.497507704	0.310305499	0.384258072	0.234142331
AT2G26870	NPC2	-3.028491365	9.58704E-07	-3.571749476	0.004380353	-3.06889741	1.50001E-06	-0.37637432	0.870968248	-0.475026169	0.597798092	0.77442393	0.01888549
AT1G19150	Lhca6	-3.057440339	2.5759E-14	-2.371204163	5.60278E-06	-2.859847521	1.08908E-32	0.01065613	0.99694718	0.177554886	0.83609719	0.842977828	0.007152394
AT2G42380	BZIP34	-3.087175164	1.01492E-16	-2.805718808	2.47702E-26	-2.092580342	7.69088E-06	-0.601014401	0.637815859	-0.190018937	0.734451454	-0.056298591	0.852921765
AT2G40610	EXPA8	-3.10337186	4.33173E-12	-1.778159991	3.56887E-05	-1.500882246	1.76138E-05	-0.255215941	0.80141891	-0.854448541	0.003091936	-0.873306352	1.67601E-09
AT3G19480	AT3G19480	-3.157217554	1.49237E-08	-1.409747187	0.001893111	-1.80952075	4.37026E-14	0.771178318	0.747061282	0.217085786	0.926851583	-0.576446595	0.321184984
AT1G65230	AT1G65230	-3.183087875	1.36317E-06	-1.207851561	0.010769527	-2.014632079	1.16312E-27	-0.413456584	0.918531026	0.417322681	0.76508716	0.976132723	0.000119391
AT1G21270	WAK2	-3.183349381	7.29343E-07	-2.423282617	0.000249769	-2.666975151	6.80043E-06	0.71219528	0.647947756	-0.077093431	0.959320035	0.781163896	0.004902301
AT3G10520	HB2	-3.207719983	1.21017E-09	-2.262879932	3.17962E-08	-3.702253445	3.3447E-46	-0.244728921	0.915508397	-0.172029792	0.821441621	0.198126773	0.55564725
AT4G33010	GLDP1	-3.228410436	6.712E-25	-1.855472437	2.43459E-23	-1.52992562	8.36439E-25	0.44733203	0.748481821	-0.476708769	0.013140762	0.252151679	0.265487031
AT4G37925	NdhM	-3.25695904	9.72777E-16	-2.291650375	2.44682E-10	-2.120602557	2.46943E-14	0.371971776	0.818724846	0.612469321	0.45459785	0.93489578	0.000404047
AT4G22830	AT4G22830	-3.269506555	4.43344E-10	-2.101910076	0.001860931	-2.450951973	2.46541E-10	-0.320908107	0.926730556	0.773668298	0.252047337	0.661013065	0.07257278
AT2G36145	AT2G36145	-3.281735933	1.32675E-09	-2.024220274	6.87867E-10	-2.889535299	1.39305E-16	2.008121023	0.65378996	0.522581812	0.820477747	0.31755041	0.471961447

AT1G22590	AGL87	-3.338076791	4.43231E-12	-2.372126439	0.02795569	-1.90794003	1.1819E-15	-0.194297823	0.946800339	0.222417874	0.87552726	-0.048416031	0.947652614
AT3G48720	DCF	-3.36411776	1.68821E-16	-2.727017554	1.50433E-18	-2.290519331	8.49475E-22	0.124947804	0.960666604	-0.47232446	0.275068751	-0.187450143	0.624396354
AT2G35960	NHL12	-3.38234685	1.55889E-06	-2.382825992	0.003307492	-3.666196412	9.38915E-16	-0.807435067	0.907704517	0.990925914	0.727760991	0.300570276	0.732369173
AT5G36910	THI2.2	-3.403253587	1.67954E-09	-2.808415534	1.50559E-17	-2.769102398	8.38218E-07	7.217042175	0.465456099	3.721338465	NA	5.387323791	0.072385764
AT5G58260	NdhN	-3.420031005	8.85474E-13	-1.771452175	2.64513E-05	-2.771325065	2.46692E-31	0.52427154	0.738801192	0.143202055	0.916204809	0.971338422	0.002433344
AT4G37930	SHM1	-3.427794426	3.11054E-33	-2.342644866	1.77015E-40	-2.031699585	5.01089E-71	0.933336779	0.563758673	0.193688025	0.562789691	0.738767501	1.16117E-05
AT3G28220	AT3G28220	-3.431000882	5.96272E-16	-1.94055046	0.002663902	-2.922802245	7.0509E-06	3.624346162	0.651371841	-0.821095123	0.890943193	-3.809204375	0.20731794
AT4G23290	CRK21	-3.431093875	4.3539E-16	-2.713665797	1.05421E-07	-1.397010318	8.48481E-05	1.129671014	0.697744369	0.309600544	0.890884678	-0.581574464	0.557759257
AT1G44575	NPQ4	-3.465813809	1.53155E-44	-2.470531243	3.81634E-85	-2.261919954	2.59858E-35	0.493755721	0.709586436	0.353755798	0.166216897	0.847085791	2.23688E-08
AT1G27480	AT1G27480	-3.483174116	2.21602E-13	-2.508535435	0.001342947	-2.443021711	1.28808E-11	-1.240754919	0.574003303	0.299102487	0.68232732	0.694065393	0.035937027
AT1G32220	AT1G32220	-3.535387528	8.02762E-30	-2.630769746	3.83668E-12	-2.66335586	8.99779E-52	0.378043989	0.741519211	0.219744651	0.719986639	0.42443451	0.041837879
AT4G36220	FAH1	-3.547885189	2.66366E-28	-2.733943545	3.71401E-13	-1.633864613	8.82039E-24	-0.322084334	0.81670535	-0.110745691	0.883796441	0.540637115	0.000480212
AT1G69730	AT1G69730	-3.594725003	0.006637421	-3.292991264	0.020140874	-5.498155	1.68117E-06	-0.755824186	0.967291937	0.058869697	0.993586481	-1.373293905	0.622102072
AT5G38430	RBCS1B	-3.617282887	4.87509E-21	-1.887337518	5.95889E-10	-3.479856872	9.5608E-12	-0.010766063	0.997796217	-0.838565471	0.478838042	1.12094426	0.281023881
AT1G15980	PnsB1	-3.736132753	5.06722E-46	-3.106336275	1.71353E-45	-3.055364743	4.11003E-35	-0.413011309	0.741769967	-0.202859454	0.821484152	0.715657182	0.009321847
AT3G56060	AT3G56060	-3.747053355	4.59569E-15	-2.073076844	7.14374E-10	-2.026233745	2.51481E-10	-0.025094418	0.992379592	-0.931723081	0.187623269	-0.337538597	0.403805113
AT1G57770	AT1G57770	-3.751719191	2.45583E-21	-3.189698651	5.1383E-10	-1.839426362	1.09131E-13	0.815443813	0.435291031	0.776370972	0.17086402	0.074880996	0.888137484
AT5G43750	PnsB5	-3.764292142	3.04426E-27	-2.696323603	5.6042E-06	-2.408905091	1.30266E-30	-0.798040481	0.544222418	0.473055199	0.26177131	0.758016035	0.006569248
AT2G30010	TBL45	-3.795840319	3.93683E-12	-3.30838374	6.89848E-14	-4.155610301	1.85031E-54	-0.802367426	0.597380545	-0.685561341	0.03465168	0.755046914	0.000826524
AT5G08030	GDPD6	-3.84491467	0.003538019	-8.493291273	2.02889E-05	-3.675099355	0.000483209	-2.130469655	NA	3.158872313	NA	2.722115881	0.134229601
AT1G26761	AT1G26761	-3.898495772	2.14476E-15	-3.351650628	8.06024E-08	-2.19026558	9.39396E-11	0.418477139	0.977607985	-1.493915171	0.672750383	0.988383652	0.170330258
AT1G10657	AT1G10657	-3.901533248	5.00536E-09	-3.645879144	0.002769119	-2.534874691	0.000202013	0.402996107	NA	1.595421717	0.682197544	1.250090232	0.122407179
AT5G54710	AT5G54710	-3.99239197	8.60751E-11	-1.763508427	0.002906476	-3.798034681	6.57767E-12	-1.313772435	0.769907555	-0.707659841	0.499220242	0.912178336	0.169849644
AT4G12830	AT4G12830	-4.00346239	1.6302E-09	-2.827645712	0.000115269	-3.288795993	5.55527E-25	0.178820552	0.982734229	-0.036416905	0.987706343	1.011923929	0.342444935
AT4G04840	MSRB6	-4.017402566	1.5335E-13	-3.265088048	3.7874E-14	-4.39847885	5.28764E-24	0.774450134	0.92099795	0.646799479	0.870860893	-0.935598192	0.668279596
AT4G19810	ChiC	-4.185782952	0.00046042	-3.167120714	0.036671458	-3.510284307	0.015368884	-1.597370496	0.685591568	-1.380083708	0.151779445	-0.776781594	0.669590322
AT5G38410	RBCS3B	-4.287736103	7.0936E-47	-2.954529932	2.86166E-26	-2.663344576	3.0723E-87	0.803512131	0.606948021	0.387497826	0.576874111	0.927506655	9.95409E-07
AT3G48420	AT3G48420	-4.627807814	3.10731E-32	-3.092049852	1.38642E-37	-3.295800821	4.5983E-30	-0.060511075	0.984084429	0.030749421	0.975259737	0.790511722	0.005938017
AT1G32080	LrgB	-4.635968674	1.29562E-47	-3.428658545	9.84067E-14	-2.921854927	3.20892E-40	-0.070428984	0.977596393	0.080380287	0.930662149	0.042223165	0.891627279
AT4G31870	GPX7	-4.692285238	0.011704531	-8.577844638	0.000243813	-4.28434335	0.004069445	2.923155814	0.718989301	5.843364777	0.455120097	0.717156711	0.82928832
AT3G01500	CA1	-5.095132172	7.46549E-50	-4.373041234	5.89616E-79	-2.733618835	2.46681E-68	1.634852536	NA	0.864742279	0.029329363	-0.70200139	0.326058022
AT3G20200	AT3G20200	-5.238383269	6.82908E-05	-4.767963049	0.004164368	-1.750252622	0.005727145	-0.961762744	0.932561267	0.875383738	0.574116593	0.977666287	0.062989002
AT1G70820	AT1G70820	-5.913611838	1.44855E-78	-4.509321406	2.81226E-29	-4.377478089	1.64617E-07	-2.533230108	0.786649032	0.071632741	0.993370461	-0.165743348	0.958043778
AT5G24420	PGL5	-6.281982559	5.81365E-14	-5.994882988	2.57619E-06	-10.77161347	7.62885E-16	3.820813816	0.542984302	-1.075110377	0.750566091	1.004586865	0.801020903
AT4G26530	FBA5	-6.526504056	1.17973E-48	-4.467029127	1.25916E-92	-3.648780935	9.9782E-11	2.432225084	0.637614678	-6.429357135	0.192417027	0.877170701	0.795445638
AT1G07450	AT1G07450	-7.104641153	0.02302441	-7.234923332	0.010839938	-3.933237756	0.015077512			4.120646097	NA	-1.377867238	NA
AT3G04210	AT3G04210	-7.258311858	5.66905E-07	-3.295685066	2.06103E-06	-8.143114543	4.50164E-06	0.34421492	0.982749202	5.950511512	0.42538701	1.452406385	NA
AT3G07000	AT3G07000	-7.637416532	0.00321948	-7.331284996	0.004843064	-6.903984628	0.003926084	0.441119744	0.953923605	-2.137320978	0.132245706	0.377684822	0.662932811
AT1G32910	AT1G32910	-7.795617538	0.010659607	-7.902019869	0.01345376	-6.553392376	0.014085739					3.067189115	0.529345966
AT1G29490	AT1G29490	-8.372252033	0.000226772	-6.670226375	0.041154319	-7.621842365	0.000438122	-0.931748844	0.890733612	-0.093731918	0.983138034	0.262560198	0.958043778
AT5G10250	DOT3	-8.381128476	0.000247925	-7.799070559	0.018268477	-8.804835499	8.5572E-08						
AT2G27420	AT2G27420	-9.014384765	0.000934922	-7.963159879	0.00030281	-3.723616208	0.027491006	6.023955062	0.619354152			3.409230996	0.583687476
AT1G24070	CSLA10	-9.140427229	3.13461E-07	-9.240041075	1.10044E-07	-8.410278899	1.32796E-06	0.615142522	0.936758504	0.392426261	0.851437944	0.756204386	0.096807574
AT2G41240	BHLH100	-9.342670673	3.87112E-07	-10.29156407	3.38236E-11	-11.66620821	1.05365E-18	-1.36301855	0.916159273	1.245975689	0.919937283	-3.873158937	0.107073972
AT2G29170	AT2G29170	-10.42826713	1.69592E-11	-9.930555801	6.89293E-11	-9.525012602	1.01804E-10	4.337135227	0.704004387			-5.870465328	0.137733582
AT5G59130	AT5G59130	-11.18786716	1.57093E-13	-9.90880792	1.22486E-10	-9.826151833	3.86286E-12					3.451173535	0.578100653
AT5G15960	KIN1	3.650276707	1.68573E-19	6.482222196	6.172E-19	1.389899001	NA	1.898377355	0.587695712	-1.680032624	0.001785542	-2.886542497	4.06905E-15
AT5G52310	LTI78	3.413838161	4.2907E-18	4.165432183	3.21156E-18	1.213850396	0.258011989	2.451211384	0.564319346	-1.831783371	6.74989E-11	-2.205421984	4.30759E-23
AT4G37070	PLP1	3.17434882	0.000235265	-3.585752575	0.043818137	2.049145087	0.602744214	-1.155191348	0.542252854	-1.260230638	0.000674342	-1.869660797	1.90602E-12
AT5G15500	AT5G15500	5.307648067	0.001162046	6.320900768	7.28861E-14	4.266953631	0.083126148	-1.066943533	0.862037276	8.157053641	0.000896375	-2.084774562	0.12591397
AT5G42060	AT5G42060	1.65682083	1.80198E-05	1.110852658	0.024118232	0.800459512	0.075705874	0.122209109	0.899858481	1.201758231	1.32594E-06	-0.499725659	0.096617637
AT3G04720	PR4	2.14316731	2.47855E-05	1.113493426	0.002385093	0.612789287	0.462000355	0.111944342	0.970946609	-2.270962879	0.033879158	0.352662821	0.136522071
AT3G07255	None	12.14707251	3.90288E-18	14.5136864	9.95782E-16			-3.606821838	NA	-7.410463899	0.03118522		
AT4G25480	DREB1A	2.308401412	0.000318594	1.317050444	5.66788E-06	-0.753461055	0.200861455	2.116305822	0.546537795	-0.823406039	0.575644885	1.640349053	0.000707931
AT1G72900	AT1G72900	1.367088123	0.003743266	1.179785883	1.43259E-05	-0.361017908	0.240452145	1.700398536	0.149173732	-0.264038341	0.618550064	1.604525522	2.78961E-07
AT3G62260	AT3G62260	1.862433933	1.34296E-05	2.355154218	3.75237E-12	0.703421504	0.360164759	0.806831466	0.62738272	-0.710780104	0.096474964	1.326162003	1.07865E-11
AT5G63130	AT5G63130	1.484149333	0.00026318	1.999899644	5.67251E-10	0.410980588	0.51676422	0.693797108	0.805408547	-0.438157434	0.706765606	1.169817116	0.002669935
AT5G63790	NAC102	1.400921293	0.001339185	2.357167892	1.46185E-11	0.89672245	2.54681E-05	0.764927843	0.642227114	0.188133289	0.465367001	1.142195124	5.08293E-21
AT1G23710	AT1G23710	1.112125314	0.016742418	1.36147494	4.77572E-05	0.234305188	0.434077245	0.475359625	0.820186281	-0.524998919	0.029678733	1.106816977	1.36242E-05
AT4G09600	GASA3	8.119369969	0.00221496	9.272361524	6.00257E-06	4.49428671	0.405997827	2.439316494	NA	-1.02742701	0.883566526	-4.623010143	5.62338E-05
AT4G13280	TPS12	8.221987393	0.000445702	7.925530522	0.018445806	1.539932728	0.626537555	0.759683248	0.913085739	-0.285743311	0.898419137	-1.247197398	0.006053801
AT3G49580	LSU1	10.32816594	3.19804E-10	7.628077548	0.003037292	1.74726995	0.641966602	-0.08200563	0.994744545	0.688559687	0.933021213	-1.832972543	0.012087837
AT2G47780	AT2G47780	2.468441856	0.019745768	5.256413405	0.007512867	0.880314729	0.366694488	-1.311749192	0.172855349	-0.275205888	0.225543512	-1.117363098	4.13529E-11
AT1G56600	GolS2	2.467195915	5.95942E-07	4.807811926	4.92546E-28	1.105591206	0.102210068	0.185694573	0.935083701	-0.839818095	0.35947423	-2.635130302	9.23612E-07
AT4G39700	AT4G39700	2.232621264	0.032217249	4.584730621	0.041147095	1.897282196	0.08201938	0.170925973	0.953275639	-0.382342853	0.802042097	-1.442926111	0.00514616
AT2G32510	MAPKKK17	3.511604478	0.012403777	4.274773552	0.000786007	1.307369341	0.292292797	-4.181232626	0.379135329	-0.497132987	0.827504892	-1.522439764	0.009609337
AT3G50970	LTI30	3.610648143	5.5669E-19	4.179675786	9.22703E-37	0.826968159	0.324069692	1.220369888	0.383872027	-0.274155773	0.737533924	-2.899486562	2.79601E-12
AT5G56795	MT1B	4.81866274	0.00271341	3.795084015	0.000171251	1.053468976	0.363412729	0.103577159	0.982034793	0.848682131	0.175666318	-1.741014724	0.002701715
AT2G28110	FRA8	3.532552421	2.5466E-08	2.961906515	2.66882E-18	1.125428535	0.170998213	-0.642720218	0.588988003	-0.67957841	0.011670901	-2.269434091	5.16023E-24
AT2G05510	AT2G05510	7.899013059	2.0956E-56	2.924697075	7.26069E-08	4.686774351	NA	-1.221940198	0.539736869	0.688374708	0.570854222	-3.259109692	0.000762794
AT3G23180	AT3G23180	2.591738058	0.013854731	2.916625124	1.96577E-05	1.040247124	0.679285878	-0.764361014	0.256760439	-0.690939078	0.225543512	-2.43848898	6.36484E-09
AT2G02930	GSTF3	4.027116617	0.000145464	2.788957521	0.000623511	1.284017659	0.095350792	0.411427472	0.855162677	-0.529093038	0.590311111	-1.205587014	0.015299145
AT1G07590	AT1G07590	2.092610239	1.42318E-14	2.726821035	2.79188E-15	0.811464398	0.010988954	-0.253576652	0.777083998	0.275477989	0.472395039	-1.903957763	5.77563E-05
AT3G57380	AT3G57380	5.682301431	0.000223673	2.594143436	0.028416664	-0.194961847	0.957969971	-0.665914808	0.691895802	-0.545660774	0.575644885	-1.075447072	0.007294021
AT5G13080	WRKY75	2.989293653	0.001213784	2.56471346	0.000109141	-0.326328091	0.852033036	-0.342300913	0.892627205	0.316087373	0.802042097	-1.545343113	1.53988E-06
AT5G03890	AT5G03890	3.14401932	2.05486E-06	2.432143927	7.60687E-07	0.945568141	0.098209856	0.317887444	0.855277825	0.630851892	0.510789648	-1.448282823	0.001494764
AT1G67980	CCOAMT	10.20139771	7.24472E-11	2.325583465	0.011546891	-0.903517462	0.721953783	-0.81881278	0.680482607	-0.115860259	0.953898607	-2.442373014	7.90466E-12
AT5G25240	AT5G25240	1.932737377	0.009886025	2.246755244	0.00014219	0.879266738	0.023585222	-0.671107294	0.676109289	-0.946361179	0.127016651	-1.300594505	0.001508416
AT1G62770	AT1G62770	2.823260805	6.60589E-17	2.198424533	3.3003E-11	1.096952994	0.084225527	0.17675617	0.908372568	-0.444028113	0.034469941	-1.117511154	2.07542E-14
AT1G17170	GSTU24	4.366903247	0.025918606	2.19758845	0.000303813	-0.743772137	0.207553495	-1.782304374	0.15552114	-0.15290568	0.828846482	-1.400705607	2.9414E-07
AT2G31585	AT2G31585	1.980457713	1.5168E-08	2.154515455	4.00109E-08	0.927241319	9.29357E-07	-0.643155056	0.646729152	-0.12530188	0.844918508	-1.337060267	2.0438E-29
AT2G07698	AT2G07698	1.505868368	0.011544032	2.124497582	0.000637608	0.192938878	0.809618896	-0.653443437	0.663662043	-1.152071722	0.100287424	-2.883259327	1.4369E-16
AT3G14060	AT3G14060	2.062457208	0.005391726	2.004551532	0.008808356	1.515121703	0.215843135	-1.688281901	0.2905498	-0.147522635	0.927080141	-1.072617375	0.000968761
AT1G28230	PUP1	2.552747358	0.000338261	1.998139437	0.000674303	0.765056736	0.113380813	0.069547852	0.987576531	0.492195451	0.548752327	-1.122974519	0.009628142
AT4G29905	AT4G29905	1.785890049	6.51846E-07	1.982053729	4.36989E-12	0.807892555	0.002892761	-0.37331536	0.680482607	-0.309362612	0.246696538	-1.314463294	6.34208E-11
AT2G45600	AT2G45600	1.041539556	0.040965257	1.944084387	4.59781E-08	0.449643115	0.071678786	0.108542659	0.977607985	-0.108301615	0.919937283	-1.043584922	3.18652E-06
AT1G62810	CuAO1	1.934564758	8.36357E-13	1.929789402	1.67382E-11	0.524919853	0.01254872	0.466333828	0.665673432	-0.146730273	0.659704779	-1.324853672	7.7531E-21
AT4G24480	AT4G24480	1.470416839	0.000314169	1.843284518	2.26948E-08	0.771460498	0.005282475	-0.6011044	0.433301204	-0.451314242	0.292750658	-1.549679997	9.28851E-17
AT4G39675	AT4G39675	4.050478041	1.02288E-27	1.778032282	0.000204894	-0.759329967	0.650967777	-1.210011905	0.471997679	-0.426367859	0.329353694	-1.944701901	4.44161E-15
AT5G54840	SGP1	1.759852527	0.002838566	1.757848197	0.001064293	0.100180982	0.900344019	-0.360805486	0.818515078	-0.283114619	0.679561967	-1.301814233	0.000864252
AT2G01530	MLP329	5.050230736	9.60281E-22	1.74218313	1.07197E-05	2.731920363	NA	-0.672963174	0.712887303	0.25207553	0.584383167	-2.487784679	5.90824E-33
AT2G34500	CYP710A1	4.113696824	4.52983E-10	1.719723688	0.003888106	1.44284158	0.266909404	-1.166804801	0.602119156	-0.437609599	0.311504292	-1.623412263	6.47933E-17
AT1G52827	CDT1	1.456558288	0.000102422	1.688465957	0.000223534	0.743638913	0.320325767	0.31142782	0.809492797	-0.203119682	0.790560461	-1.049214506	0.032723175
AT3G55880	SUE4	1.094746227	0.034233955	1.666463338	0.00021273	0.233787845	0.461243915	-0.531187661	0.654880495	-0.074970256	0.94851991	-1.214121912	1.1937E-13

AT3G49780	PSK4	2.492348308	4.23381E-11	1.647238423	4.09298E-07	0.77731675	0.021460237	-0.79283816	0.602119156	0.489639086	0.145348244	-1.644448692	1.78173E-18
AT5G09440	EXL4	1.559321198	4.81478E-08	1.621411297	6.74267E-12	0.810034351	0.203330966	-0.084045121	0.932578056	-0.634527781	0.000133788	-1.023359351	1.32737E-14
AT1G23850	AT1G23850	1.491828238	0.020677566	1.606931764	0.037279503	-0.055897089	0.952606907	-1.145385197	0.644839236	-0.104149761	0.930810942	-1.484934461	3.16757E-08
AT5G23980	FRO4	2.172095614	0.022741178	1.590377966	0.020644452	0.016563877	0.984592396	-0.067378492	0.984778736	-1.098366089	0.405629162	-2.307497751	2.78735E-08
AT3G12090	TET6	1.631801993	0.003673851	1.496480977	8.61162E-05	0.346943073	0.562748396	-0.115055398	0.965471639	0.258552316	0.713259931	-1.43740262	6.25538E-10
AT1G23010	LPR1	1.716821038	0.023584086	1.483454477	0.048488808	1.176315706	0.307898602	-0.409551388	0.644839236	-0.76778528	0.086698345	-1.284132656	2.66695E-05
AT1G08890	AT1G08890	1.277949876	0.002860586	1.481134731	5.16732E-08	0.204635382	0.496873153	0.093896821	0.971723999	-0.401605913	0.101075064	-1.108905105	8.79337E-08
AT4G32870	AT4G32870	3.560713286	0.003444134	1.444872827	0.025617849	1.370614376	0.071249855	0.117181485	0.972495118	0.213634515	0.848910819	-2.40787498	1.94035E-12
AT3G61160	AT3G61160	2.000556046	6.16668E-07	1.439445933	4.8382E-05	0.964049619	0.000340417	-0.461879701	0.527892502	-0.462946817	0.08501642	-1.278650371	3.57068E-12
AT4G02090	AT4G02090	1.73184374	0.020001984	1.422723395	0.044345263	0.775444916	0.39787036	-0.888416668	0.657369817	0.20441274	0.729643184	-1.178974707	0.013775267
AT4G01480	PPa5	2.053256906	4.95637E-05	1.418848226	0.008465106	-0.148413399	0.891736873	-1.253309062	0.218297828	-0.689523683	0.035196714	-1.429059953	4.92449E-20
AT1G49470	AT1G49470	1.742325901	0.025297349	1.400808004	0.018658397	-0.456238364	0.606859712	-1.08887684	0.647947756	-0.967972479	0.02495494	-1.334136568	1.56047E-08
AT5G66490	AT5G66490	1.305823343	0.006659879	1.319277625	0.00214243	0.19732223	0.599055973	0.434339823	0.706790319	-0.221515338	0.721384039	-1.362414939	5.82234E-08
AT4G23410	TET5	1.667048616	0.021956162	1.292356527	0.01908896	0.520076735	0.285932195	-0.532937223	0.709790565	0.355729306	0.485234994	-1.016978835	9.85137E-06
AT4G32940	GAMMA-VPE	1.34072016	1.09025E-05	1.281386991	3.99191E-07	0.821159007	0.001537897	-0.18871772	0.870759043	-0.318260103	0.145348244	-1.053827966	1.36861E-14
AT3G24070	AT3G24070	1.689133211	6.35971E-05	1.276341139	3.24562E-08	0.84119357	0.032375157	0.188471728	0.914644162	0.220766981	0.601357925	-1.31033743	1.58616E-05
AT1G30760	AT1G30760	4.753680895	2.1864E-46	1.275745768	0.000808138	1.021938507	0.319976681	-1.278675006	0.606948021	0.189946815	0.632912363	-3.233341851	7.5091E-181
AT3G30390	AT3G30390	1.667082751	1.18356E-10	1.235744395	5.67974E-08	0.338643585	0.05567012	-0.380046643	0.663046749	-0.476787677	0.00591378	-1.528348472	4.65868E-53
AT1G21360	GLTP2	1.717930472	0.001894765	1.163893234	0.019689992	1.104731202	0.080531389	0.161316913	0.924925439	-0.33878514	0.550968731	-1.045320623	0.009771233
AT2G29660	AT2G29660	1.729270586	4.72875E-10	1.144371136	0.003711484	0.973130896	0.000540786	-0.576652933	0.330569741	-0.223251496	0.434700701	-1.101378157	9.22832E-12
AT3G61990	OMTF3	1.38601132	0.017893525	1.096634425	0.006167681	0.808776632	0.180366262	-0.445929763	0.650313705	-0.503821869	0.171283271	-1.448185886	5.18764E-15
AT4G13030	AT4G13030	1.43333061	4.89608E-05	1.053437419	0.009696026	0.681332321	0.106913302	0.069421011	0.962872831	-0.422197129	0.258496511	-1.707962414	1.28023E-16
AT1G56010	NAC1	1.612394066	0.002568239	1.019072644	0.012981989	0.673496651	0.386064994	-0.492538475	0.721140128	-0.385978421	0.292089408	-1.337853119	6.41793E-21
AT5G09480	AT5G09480	3.540902238	0.007167159	-4.424688365	0.015390554	2.311013259	NA	-1.125511806	0.743023983	-0.613410885	0.309143999	-1.740979125	8.56567E-18
AT4G22810	AT4G22810	5.125834376	0.008228155	-7.232581455	0.010559222	-0.551715597	0.853331771	-1.330219744	0.214402425	-0.744894283	0.018070532	-2.608015897	2.40635E-31
AT5G42630	ATS	8.009248912	0.001167962	-7.672705886	0.001512062	-5.800240489	0.086403419	-0.791929934	0.894928184	2.380401842	0.244633007	-2.127450551	0.000250571
AT4G05180	PSBQ-2	-1.503167435	0.000246662	-1.043957747	4.35982E-10	-0.667592626	1.07857E-08	3.46339711	0.002726728	1.128578222	0.030329172	1.628555297	3.75215E-22
AT3G47470	LHCA4	-1.588574076	6.13311E-05	-1.240013767	1.11514E-16	-0.869539813	9.30857E-19	3.812116474	0.002226402	1.337746577	4.7504E-05	1.762321279	9.91631E-72
AT2G05100	LHCB2.1	-1.64292922	0.000131985	-1.108197944	3.60056E-10	-0.858868602	4.69126E-09	3.053342344	0.005286142	1.4354243	0.032013489	1.416259163	2.10496E-30
AT2G17880	AT2G17880	-1.033964743	0.011935427	-1.248138684	1.15496E-06	-0.737529473	4.96363E-05	2.732291923	0.024641754	0.559190238	0.097143159	1.334419228	2.26983E-08
AT1G18300	NUDT4	-1.186105591	0.013234608	-1.273831638	6.40696E-06	-0.736344142	0.178714949	2.114611547	0.032209306	-0.172568521	0.775703028	2.844458576	9.97326E-58
AT1G61520	LHCA3	-1.475579482	0.000253862	-1.005975808	4.66601E-09	-0.752621621	6.10208E-11	3.655097908	0.002726728	0.860985001	3.27055E-07	1.079954024	5.86217E-10
AT5G04360	LDA	-1.590835309	0.001914765	-1.231104435	3.4898E-05	-0.215984329	0.618777234	2.322841403	0.030255469	0.310094078	0.615893075	1.46231643	1.23885E-11
AT4G13495	AT4G13495	-1.87644717	3.92341E-05	-1.39149739	3.08657E-31	-0.440769663	0.109039631	3.777602319	1.07514E-05	1.162356272	0.174224683	1.573391008	4.58083E-18
AT4G19530	AT4G19530	-1.653690024	0.000209112	-1.497754088	5.82339E-20	-0.294885019	0.336431885	3.625233322	0.004459823	0.201258446	0.852285116	0.575991304	0.137639665
AT2G18480	AT2G18480	-4.755197473	0.00756225	-8.065817996	0.000267097	-1.244205359	0.530755364	-5.510629362	0.037611276	-1.913034584	0.583243151	-0.987793265	0.292079802
AT3G05727	AT3G05727	-1.142080273	0.008567332	-3.429189228	7.3862E-08	-0.78900782	0.023100354	0.223392784	0.937127708	1.090131864	0.038047851	1.365437953	2.26687E-12
AT2G33580	LYK5	-1.223179925	0.027300053	-1.011993995	0.045276423	0.369749022	0.779382496	-0.407520719	0.740082034	-1.1199325	0.005231588	2.394058936	3.29378E-34
AT1G80840	WRKY40	-1.664542475	0.00101796	-1.254535354	7.55884E-07	-0.865466378	0.15418701	2.108741147	0.208292228	-1.162354873	0.003814696	3.777746258	7.8855E-104
AT4G29780	AT4G29780	-1.068528861	0.02075665	-1.118724283	1.23554E-07	-0.785922436	0.212854638	1.755359134	0.196045896	-1.317050307	1.5652E-08	3.471882741	3.9813E-286
AT1G72520	LOX4	-1.633529796	0.002600511	-1.197950144	0.000455295	-0.942775096	0.209425441	0.570983976	0.835453631	-1.488162981	0.013492699	3.052456732	2.49508E-56
AT2G32500	AT2G32500	-1.572967327	2.27195E-07	-1.835974729	0.00015424	-0.885818487	1.97387E-05	1.391839665	0.186649778	1.042783804	0.045923958	0.996530401	0.000286375
AT4G13340	LRX3	-2.116386143	1.22865E-07	-1.445454895	1.62961E-13	-0.996415294	0.002999346	0.064989573	0.972495118	-1.015701888	9.45894E-06	-0.321349092	0.235282223
AT3G08500	MYB83	-5.62724814	0.003338662	-4.113624947	0.048426319	-0.238978874	0.946405917	-1.158306394	0.898525314	-3.433685253	0.002911784	0.388817053	0.706489951
AT4G34410	RRTF1	-3.299711131	3.13262E-07	-3.003802998	0.01172356	-0.541532646	0.700190682	2.824432423	NA	-6.399179105	0.123746952	7.37622951	2.5996E-203
AT1G66090	AT1G66090	-2.523817858	1.83776E-06	-1.614443913	0.000112108	4.685505135	0.063898624	0.161704662	0.972495118	-2.511907199	0.053350595	7.241986367	3.93767E-05
AT1G12610	DDF1	-1.59302882	0.00603499	-1.635677016	1.24885E-05	-0.468221135	0.727196239	0.998297816	0.698438613	-2.245747628	0.051913119	5.818379552	8.69728E-71
AT2G44840	ERF13	-1.809070078	0.007226179	-1.901926092	0.004844779	-0.756644787	0.281394865	0.97791698	0.208312184	-1.509775889	0.070974858	4.727856493	6.90478E-79
AT1G17420	LOX3	-3.263512494	3.47278E-09	-1.535625858	3.12731E-10	-1.676177576	0.085344112	1.939531381	0.570943682	-1.279678243	0.214388348	4.607206896	1.80383E-67
AT3G02840	AT3G02840	-1.341915969	0.031918691	-1.49059558	0.000753533	-0.419443271	0.74811193	1.979790197	0.584219873	-0.87888125	0.591264783	4.419287372	1.40351E-57
AT1G74450	AT1G74450	-1.04341824	0.039324073	-1.110407321	0.0001716	-0.780754216	0.03110106	1.267528643	0.458341345	-0.973835461	0.00438313	2.607158636	7.15656E-44
AT1G72920	AT1G72920	-2.527211926	0.00062455	-2.061136681	0.032830439	-2.183275736	0.354395095	0.521692401	0.847915568	-1.532089629	0.422600941	2.162637813	4.83815E-08
AT5G36925	AT5G36925	-3.678221386	0.003328273	-1.454297533	0.049230156	-0.607052617	0.792743906	-0.149281368	0.977607985	0.187330996	0.968000197	2.148357264	0.001965169
AT4G13493	ath-MIR850	-1.606128766	0.000243536	-1.604486591	1.95117E-08	-0.544335865	0.179259427	4.312666924	0.146028134	1.517440597	0.653834792	2.00301859	0.000105429
AT1G68840	RAV2	-1.278768113	0.000949236	-1.06168062	1.48732E-05	-0.834874397	3.75124E-07	1.439350776	0.070401	0.28146862	0.212842369	1.960623902	1.82792E-96
AT5G64660	CMPG2	-1.213486747	0.00107568	-1.377864245	1.49486E-05	-1.157909349	0.141567434	-0.17923045	0.86582927	-0.377565592	0.455183937	1.869894653	2.16204E-31
AT1G05540	AT1G05540	-4.337403522	0.004995295	-7.30028202	0.000270613	-1.829476841	0.298248185	0.092633158	NA	0.561287595	0.869183581	1.818234679	0.025289847
AT1G35140	PHI-1	-1.670146607	0.002954726	-2.104991208	2.51832E-14	-0.513272543	0.52670862	3.088867641	0.066290973	-0.704247528	0.211092572	1.529649019	0.011755772
AT1G13670	AT1G13670	-1.142980139	0.022580042	-1.485376385	0.001506372	-1.264755133	0.055708792	0.876637301	0.095349885	0.286016252	0.682217361	1.478985718	8.35079E-11
AT3G16860	COBL8	-1.319673039	0.032059491	-1.496075496	0.003827953	-1.336275084	0.254338268	0.892973375	0.676109289	-0.605620316	0.544520295	1.477257927	1.1534E-05
AT1G77490	TAPX	-2.329481904	2.13382E-21	-1.948998126	1.37849E-11	-0.966145224	1.11602E-08	1.281242007	0.394836051	0.483144006	0.056375962	1.453612295	1.41035E-17
AT5G11070	AT5G11070	-1.150018181	0.016331592	-1.106705743	2.3877E-08	-0.740805777	0.003869018	1.291131767	0.413866874	-0.185282062	0.724680249	1.37709241	2.31109E-19
AT4G01580	AT4G01580	-1.231045266	0.001121181	-1.386992737	0.000312312	0.187454424	0.748366796	0.729579594	0.576334999	0.937669449	0.003814696	1.350621892	5.07377E-17
AT4G27370	VIIIB	-1.812965848	2.96157E-05	-1.325952424	0.010756525	-0.851867277	0.019508761	0.633630216	0.569286428	0.243583115	0.762855447	1.302741303	3.94366E-11
AT1G30520	AAE14	-1.735838964	1.06979E-06	-1.621329969	3.18387E-10	-0.80142201	0.004015997	1.602301484	0.433558429	0.659708118	0.216258497	1.302720658	1.66527E-07
AT5G52860	ABCG8	-2.436848049	0.002319318	-1.63172591	0.029485556	0.499004807	0.636648751	0.373369679	0.884201248	0.257477392	0.689600848	1.296407646	0.000348444
AT4G33000	CBL10	-2.257778959	1.19011E-06	-1.894582031	7.24233E-08	-0.810190553	0.001332536	0.689553427	0.594193754	0.348128675	0.52118972	1.281832309	1.58408E-09
AT3G22760	SOL1	-1.323206412	0.020029711	-2.601046888	0.000296635	-0.352474764	0.434077245	0.24097377	0.914147923	0.491896733	0.298693478	1.266335585	4.00565E-13
AT3G54810	BME3	-1.917941215	8.26874E-07	-1.592546013	1.09551E-06	-0.94820574	0.003171764	0.344842973	0.691118215	-0.591950827	7.21839E-05	1.251854828	4.32721E-19
AT1G12860	SCRM2	-1.058895494	0.00670601	-1.048300628	5.1311E-07	-0.620982372	0.006059835	1.061746883	0.074082734	0.005186509	0.994809352	1.19299608	7.00853E-10
AT2G46640	AT2G46640	-3.578712942	1.80118E-05	-2.898630804	7.27302E-05	-3.128351816	0.079936799	2.619151342	0.627492921	1.709071054	0.190482229	1.192955711	0.014244085
AT3G59310	AT3G59310	-2.043941283	1.73394E-06	-1.875721423	4.18093E-05	-0.594559654	0.051870828	-0.371743592	0.783038559	0.258742981	0.74403739	1.160154568	3.98481E-08
AT5G38140	NF-YC12	-1.152736439	0.006637421	-1.362974694	3.57362E-05	0.461041629	0.137516776	0.899622789	0.315755736	0.610562242	0.109783343	1.120625877	3.33258E-06
AT1G22570	AT1G22570	-1.371956635	0.00136626	-1.072228074	3.30793E-05	-0.412648647	0.293676449	1.044094145	0.558178127	-0.411615432	0.551816216	1.11316405	0.000243006
AT1G32470	AT1G32470	-1.453857177	2.04236E-08	-1.236011658	3.00775E-07	-0.661408071	7.80279E-08	1.182019846	0.343851903	0.433074144	0.195022122	1.111965744	4.4039E-12
AT2G35450	AT2G35450	-1.142848287	0.000617895	-1.206495383	0.00098922	-0.689536879	0.000134577	0.448223622	0.579644733	0.020911005	0.98143759	1.110590974	4.08777E-10
AT1G67090	RBCS1A	-1.972360071	6.5406E-09	-1.044822589	8.78352E-13	-0.850168738	1.61579E-17	1.546162208	0.263628258	0.369116736	0.551155382	1.042245845	4.68411E-08
AT4G38620	MYB4	-1.25825352	0.02612945	-2.030998965	0.000604371	-0.603427749	0.204449049	-0.774504964	0.644839236	-0.293751194	0.514096491	1.016128649	8.40355E-16
AT3G53800	Fes1B	-1.518491988	0.000897128	-1.364895759	1.55917E-10	-0.993750506	1.00111E-06	1.722622306	0.143428011	0.840031607	0.096500101	1.007163129	0.000235634
AT5G49730	FRO6	-3.246609812	3.50658E-12	-2.719493447	3.3861E-12	-0.81645744	3.57458E-06	3.760659964	0.587820092	-0.004367098	0.999129744	-1.045354496	0.034820662
AT5G51790	AT5G51790	-1.693016477	0.000301277	-2.928927522	8.04391E-14	-0.945148905	0.079246337	0.058287658	0.981525175	0.280555589	0.809020022	-1.12878244	0.000272107
AT5G66080	AT5G66080	-1.553925867	0.000207811	-1.298919375	0.001306328	-0.135508242	0.69859617	-0.939775201	0.532231419	-0.273420372	0.56843464	-1.169995999	6.07473E-08
AT3G16690	SWEET16	-1.121683681	0.04407365	-1.029216885	0.011866186	-0.912817814	0.147781522	-0.114341464	0.954865063	-0.041718606	0.966304751	-1.696425329	1.28781E-07
AT5G04150	BHLH101	-1.831653545	0.000761913	-2.968203712	1.15813E-05	-3.418992735	0.074415043	0.831367232	0.70068163	0.416470275	0.880732693	-2.330221204	0.018763822
AT3G60970	ABCC15	5.454748516	0.013826711	9.422324989	2.30848E-06			0.60701101	0.96946629	1.152692141	0.920286072		
AT5G64210	AOX2	8.41529731	0.000120366	9.113640517	1.24885E-05	1.784310195	0.429698401	-1.152297147	0.93480387	-2.415955678	0.570854222	-2.696939916	0.080218228
AT3G08435	None	3.909811083	0.027471801	9.067531471	9.44878E-09			6.845251653	0.575257546	-0.689290612	0.955786471		
AT1G07873	None	4.710906765	0.010755824	8.054713556	2.27182E-07			0.929272418	0.960530601	-0.212913162	0.988021272		
AT5G12180	CPK17	3.488874775	0.04324124	7.422972062	5.78992E-05	3.468856549	0.114960389	1.195854161	0.630222114	0.016450422	0.998625192	1.611546953	0.238005098
AT1G01530	AGL28	7.215060381	0.025845956	6.473239995	0.000255148	6.273904482	0.102270292					0.049811487	0.988582661
AT2G05155	None	8.71590861	4.239E-05	6.344205671	0.018859116			-5.998735832	0.619569853	-1.041817956	0.935378386		
AT1G09163	None	9.149317752	2.05947E-06	6.314162309	0.017239266			0.551130907	0.977477438	-5.991084688	0.323767852		
AT2G02700	AT2G02700	4.784251588	0.004571257	6.261223226	0.023822054	1.431727136	0.45356011	0.090495492	0.994028221	-5.434463515	0.478338322	-0.730053877	0.092313841
AT5G08975	None	4.853871681	0.00380052	4.94553178	1.27774E-05			-0.42899491	0.981893161	2.466811251	0.511188955		

AT1G54095	AT1G54095	3.53078835	0.029123329	4.914700163	4.73916E-06	2.838466688	0.1001797	1.073554706	0.733019473	-1.118539198	0.67277392	-1.324776075	0.19237664
AT4G35180	LHT7	8.680395098	4.30781E-05	4.748054215	0.030742444	0.8531641	0.878089188	-7.412964998	0.386146892	2.599968521	0.51599066	0.314352345	0.824357435
AT5G17460	AT5G17460	3.760818226	8.35439E-21	4.463034661	1.70899E-39	1.061411956	0.327629306	1.639016605	0.429222482	-0.290249681	0.682175964	-0.543391686	0.014879501
AT5G23790	GolS5	4.635366792	0.021933754	4.403942913	0.035132991	4.391775924	0.19212097	5.849353334	0.616300683	-6.727304204	0.121816506	-0.661306278	0.90073822
AT3G63052	AT3G63052	2.400726897	0.029139527	4.281660532	0.005269127	4.958816351	0.354398923	-6.760535896	0.548879103	2.410291794	0.805320688	0.216187079	0.979326029
AT2G33130	RALFL18	5.319868704	0.002481323	4.135222152	0.033810071	1.864063456	0.567890807	-6.057051184	0.511442094	-1.530807197	0.629811151	-0.093898971	0.981463641
AT4G10960	UGE5	2.586698274	2.64096E-05	4.100015147	1.16737E-09			-0.839104334	0.615454805	0.130359609	0.941740389		
AT5G06230	TBL9	2.497696703	0.016584932	4.010684892	0.009679572	-0.972584069	0.754881385	-1.56787469	0.823401939	-0.238909557	0.926264746	-0.56019407	0.530165776
AT1G48100	AT1G48100	2.986349103	0.026846773	3.665140715	1.94097E-05	0.579215381	0.285496623	-2.0916371	0.644839236	0.586706714	0.953054857	0.225159218	0.38673658
AT3G06345	None	2.468909629	4.49644E-14	3.613569824	2.50926E-47			0.736665486	0.604698639	0.407654565	0.239373546		
AT1G69490	NAP	3.050913541	1.53926E-13	3.611956676	6.29915E-28	0.757745531	0.002556395	1.017639728	0.574453322	0.319755664	0.635351512	0.964385764	7.73683E-08
AT1G03100	AT1G03100	3.974083822	2.29116E-07	3.603915387	5.55405E-12	0.591082926	0.092088205	0.539890291	0.899163675	0.359892867	0.846410046	0.179546398	0.582923936
AT5G15970	KIN2	2.701470717	6.16571E-12	3.569456353	1.40265E-56	0.046443162	0.9516594	1.865640783	0.429222482	-0.448147211	0.012255952	-0.378334482	0.154093107
AT2G03855	None	4.089981728	0.000380681	3.525848409	3.08391E-07			1.223111044	0.685495817	0.99662749	0.51084463		
AT1G19490	AT1G19490	2.284523989	8.46834E-06	3.519917175	5.91961E-11	0.911367185	0.283756636	-0.436632391	0.791359656	0.631320691	0.286030229	-0.467174017	0.133275424
AT3G14620	CYP72A8	1.794714791	4.02713E-05	3.518530059	8.79065E-11	-0.389464374	0.504132441	0.400689073	0.767987589	0.564363353	0.395790828	0.201367649	0.754655423
AT3G14440	NCED3	2.530196966	6.22557E-09	3.354431299	4.01501E-19	0.864812741	0.000300317	0.827493619	0.731041294	-0.074860108	0.946299836	-0.138980207	0.718310107
AT1G67365	AT1G67365	3.349247824	1.89828E-05	3.337804235	0.001232624			-0.279697415	0.894017469	0.723664612	0.332641641		
AT2G31940	AT2G31940	4.358502107	0.020484468	3.324416959	0.007635982	1.367501674	0.374243542	0.612839701	0.911737107	0.798925863	0.67743073	-0.798944136	0.246275682
AT3G23840	AT3G23840	2.026433957	0.003044863	3.109580732	1.30448E-10	0.462900133	0.635755819	1.030202327	0.715099832	0.345938525	0.801700319	0.62726443	0.564224933
AT1G04047	None	2.583530135	6.83707E-06	2.9998779	5.17481E-12			1.599053366	0.502514446	1.253632765	0.528003994		
AT1G01310	AT1G01310	2.127192821	0.001815087	2.953844663	0.001713425	0.203524217	0.928930133	-0.97527744	0.91867804	3.367964401	0.242445371	1.719488629	0.228742249
AT4G12290	AT4G12290	2.292274917	1.57435E-07	2.953634623	9.35694E-21	1.004502085	0.132569663	-0.437315689	0.779600001	0.300487294	0.704395024	-0.678522044	0.009494269
AT3G21510	AHP1	2.341658499	6.02829E-05	2.886504357	0.000124358	1.177208336	0.070350323	-0.074078958	0.967187969	0.323050824	0.626431573	-0.491410493	0.094716629
AT5G20710	BGAL7	2.877425568	1.01437E-06	2.858211436	0.001702874	3.419980722	0.396516945	-0.599708251	0.822046726	-0.611209641	0.122955746	-0.31771378	0.761995254
AT4G25670	AT4G25670	2.455288688	1.30504E-11	2.851238053	1.86438E-34	0.802716708	0.820204552	0.655219159	0.680482607	0.11699433	0.802042097	0.839036322	0.826410016
AT4G37370	CYP81D8	1.168520656	0.018655892	2.821253607	6.10359E-10	0.447048704	0.580254235	-0.332674618	0.832843792	-0.181482622	0.818982935	0.075748998	0.878292965
AT5G03285	AT5G03285	2.307485455	0.000258875	2.791590499	0.001433118	2.009821325	0.08623393	0.087022047	0.982749202	0.507687566	0.618023749	-0.872018054	0.192936409
AT3G05650	RLP32	2.657488999	0.002810855	2.648649607	0.000181739	1.596956446	0.154911425	1.717525725	0.681538103	-0.509471971	0.775246054	0.888181174	0.013112338
AT3G09440	AT3G09440	1.958577883	1.64783E-08	2.643900626	2.51497E-24	0.952922212	0.000111043	-0.652806763	0.45375294	-0.476361738	0.000951482	-0.51790443	0.009383871
AT5G47650	NUDT2	1.79559458	7.21851E-06	2.620698588	0.003569149	0.89191434	0.001686631	0.416118634	0.820789421	0.085913138	0.914359122	0.138024628	0.615238191
AT1G12420	ACR8	2.52495997	1.98307E-12	2.615109109	6.10971E-09	0.830920242	0.04572117	-0.297933145	0.799604234	-0.092221357	0.898742935	-0.471896917	0.012181092
AT4G05010	FBS3	2.295994103	7.31086E-06	2.59834541	1.08515E-09	1.070087875	0.064724719	-0.403531993	0.696374342	0.028244815	0.979610562	0.240308757	0.550952411
AT2G39795	AT2G39795	2.606243781	1.60507E-05	2.597154809	2.34378E-11	0.121196912	0.741695288	0.385278426	0.680482607	0.08737036	0.893744923	0.16931571	0.55688725
AT1G77145	AT1G77145	3.385632582	0.011615367	2.552255747	0.000224459	0.752692511	0.456019464	0.863568174	0.672844426	1.088213352	0.104168293	-0.817812946	0.288179519
AT3G15500	NAC3	2.117029411	1.08621E-06	2.518115397	0.000801823	0.392413065	0.029127587	1.244644853	0.532818693	0.08474502	0.936116842	0.206937308	0.632898424
AT3G11080	RLP35	1.928039532	0.046078243	2.502817309	0.006865552	0.323969005	0.811454035	-0.834099732	0.883412909	-0.291192616	0.865842332	-0.704028893	0.387705991
AT5G43380	TOPP6	2.436036253	1.51713E-05	2.499431113	1.97271E-09	0.66174337	0.244061941	0.317309396	0.835994723	0.02286257	0.986818647	0.145367563	0.661891749
AT1G71960	ABCG25	2.07214143	6.45218E-07	2.495807597	9.38781E-12	0.816801387	0.009004256	-0.16637593	0.899163675	0.131110859	0.841586178	-0.000825121	0.997491107
AT4G25690	AT4G25690	2.201003688	1.44646E-10	2.479662368	6.86287E-23			0.609461246	0.662219342	0.482557291	0.019886905		
AT4G16750	AT4G16750	2.514463028	3.65115E-05	2.474589918	1.25096E-05	-1.133563851	0.076744906	-0.318248397	0.818896527	-0.345079836	0.652699183	-0.906515728	0.002225121
AT1G30260	AT1G30260	1.456245744	0.030990013	2.315666452	7.90701E-07	0.375166568	0.491274468	0.846884985	0.767987589	-0.692355891	0.50473905	-0.696193884	0.113868203
AT3G07090	AT3G07090	1.640287121	3.27373E-09	2.310353806	4.34818E-13	0.743124722	0.010631179	-0.53495539	0.608418107	-0.048463507	0.927107216	0.122043703	0.532344692
AT5G65990	AT5G65990	1.259062057	0.000232673	2.283933228	5.88979E-10	0.72381883	0.028621012	-0.215724839	0.801106443	-0.36713341	0.343087632	-0.397955528	0.054493441
AT3G19580	ZF2	1.723956541	0.000130436	2.266729261	6.64606E-14	0.562755707	0.558310667	0.291985677	0.905595539	-0.027735469	0.976570624	0.608285411	0.000526249
AT4G35190	LOG5	1.81254636	9.28916E-05	2.255852136	1.65539E-06	0.737627733	0.013095738	-1.393822052	0.385221483	0.333575646	0.672199109	-0.271869187	0.467016899
AT5G22270	AT5G22270	1.99856695	1.06606E-07	2.246504563	1.65893E-10	0.573555344	0.003107895	1.575039064	0.248219642	0.18390876	0.84188769	0.544704944	0.111444817
AT3G03270	AT3G03270	2.155119943	0.000639758	2.244068824	4.88675E-16	0.902859716	0.003572564	-0.235311841	0.752522032	0.072880747	0.921127795	-0.598115747	0.02898448
AT2G21180	AT2G21180	2.313364335	1.12294E-06	2.237165674	4.56763E-07	0.941454782	0.002508647	0.043626667	0.984928214	0.515973861	0.350174136	-0.786873684	0.004232447
AT1G58520	AT1G58525	1.335319466	0.019513223	2.221970608	1.87578E-08	0.619162424	0.058724383	0.783480825	0.646747644	-0.214741268	0.821510242	-0.217257135	0.591230596
AT3G10910	AT3G10910	1.565901873	0.009482051	2.204643159	0.001832828			-1.060768311	0.416717256	-0.288196842	0.58081406		
AT3G15440	AT3G15440	3.848329092	0.001641485	2.192802458	0.001681745	0.551789117	0.609139032	0.388949876	0.88950523	1.008628676	0.447139821	0.206156787	0.759096149
AT4G34881	AT4G34881	1.891937026	7.86778E-11	2.187098021	1.34912E-21			0.149106431	0.853012539	-0.060499984	0.929239543		
AT1G33102	AT1G33102	1.838413191	0.035531249	2.170003781	0.040813616			-0.030686198	0.99976672	1.882337687	0.363828665		
AT2G04350	LACS8	1.419109585	0.000224744	2.16212362	4.35655E-11	0.417292012	0.401675394	-0.365576289	0.75047646	-0.446169377	0.310699059	-0.823694785	4.59291E-07
AT1G01725	AT1G01725	3.04652611	2.64894E-06	2.161325102	0.001671713	0.559849312	0.226374719	-0.09938152	0.989178363	0.699696588	0.664623616	0.249718539	0.487350133
AT2G29440	GSTU6	1.490004114	0.020052313	2.150326323	0.001404544	-1.620146416	0.116032287	-0.76388991	0.882425056	-0.165891344	0.827003175	-0.510008177	0.055580019
AT4G30490	AT4G30490	1.047792871	0.002812187	2.148239051	3.95472E-10	0.85148466	0.011043149	0.256205423	0.816625165	0.284594751	0.409256816	0.525991086	0.001353452
AT1G08500	ENODL18	1.528789729	0.004539605	2.144048148	1.17673E-06	0.072063934	0.93189409	-0.850453486	0.486378875	-0.866292815	0.021748341	-0.663698912	0.001836777
AT1G73920	AT1G73920	1.407058805	2.44682E-05	2.138669399	1.67505E-11	0.870984743	9.81931E-07	0.181168639	0.858713803	-0.098441509	0.798412774	-0.229135651	0.109733516
AT2G29340	AT2G29340	1.130558889	0.007032814	2.120898748	9.15433E-28	0.837163386	4.85252E-06	1.950238868	0.210076168	0.617554102	0.060037937	0.27738646	0.275650327
AT5G13990	EXO70C2	1.603278365	0.013513086	2.116280127	0.004887103	-0.652287596	0.675973981	-1.035771557	0.685495817	0.607339232	0.615342952	0.188978823	0.812522158
AT4G28240	AT4G28240	2.011411629	5.55882E-09	2.111738959	4.46599E-10	0.891676379	1.28577E-08	0.742814208	0.601921549	0.005832846	0.992128549	-0.411922639	0.002645909
AT2G05520	GRP-3	1.837598057	3.98384E-07	2.104237547	1.01813E-20	0.935167842	0.121042165	-0.860559721	0.294468892	-0.403917645	0.06573481	-1.360367752	0.081150388
AT4G11600	GPX6	2.192267997	6.3592E-11	2.07595059	1.64332E-22	0.260815925	0.401849692	0.377019488	0.732957619	-0.06246357	0.882725602	-0.233908742	0.116254984
AT3G43270	AT3G43270	1.148743987	0.023127016	2.069876967	1.57055E-17	0.573666677	0.030226397	-0.728271836	0.079118837	-0.704944568	0.024399647	-0.215878147	0.368190222
AT2G15970	COR413-PM1	1.733361792	7.29166E-06	2.068966689	4.0563E-19	0.599512137	0.02330497	1.265655998	0.578723847	-0.738056441	2.7452E-05	-0.582083608	2.94953E-06
AT1G28520	VOZ1	1.441692252	1.42907E-07	2.006451621	5.59002E-25	0.935249637	3.00008E-07	0.334230822	0.706790319	0.610168527	0.000951482	-0.026193157	0.921669451
AT1G67360	AT1G67360	1.362069655	0.0414772	2.005346385	1.7418E-05	-0.377452659	0.641370775	-0.198873308	0.885844797	-0.54038703	0.27712645	-0.279011299	0.224345065
AT1G75390	bZIP44	2.154227294	0.009061495	1.996703383	1.38741E-05	1.068255291	0.756478003	-0.001185417	0.99972467	0.221858022	0.576874111	-0.096638934	0.974455978
AT1G58340	ZF14	1.458811903	0.000654374	1.972731823	0.00130398	1.086311293	0.095966437	-1.185113275	0.183329115	-0.187451401	0.854600248	-0.1700982	0.666971664
AT1G04310	ERS2	1.889242067	2.45358E-10	1.972646315	1.00983E-12	0.901128024	0.005005937	0.815073368	0.594074706	0.587390935	0.042252787	0.487284941	0.027606758
AT5G13200	AT5G13200	1.839487354	0.000644535	1.966246754	0.000102759	0.896076632	0.004619004	-0.974301513	0.631596962	-0.563736389	0.414151773	-0.724137715	0.001459428
AT1G73120	AT1G73120	1.46332856	3.17204E-05	1.957152108	1.91333E-05	0.241516338	0.275790174	-0.370688306	0.839619231	0.427745767	0.054093853	-0.788140369	0.000224374
AT2G36330	AT2G36330	1.326413035	0.027801327	1.955621036	0.004494831	-0.151494566	0.788134307	-0.124802786	0.963341405	-0.160615183	0.83609719	-0.374903036	0.082436438
AT4G13180	AT4G13180	1.743342587	7.62353E-09	1.948797303	7.22395E-13	0.962368486	0.000453624	0.430271518	0.582068518	0.408507663	0.070066227	0.688763114	0.000565211
AT5G59480	AT5G59480	1.493839069	3.37268E-06	1.937595178	1.17045E-09	0.887890818	9.57498E-07	-0.425761192	0.591481547	-0.080951838	0.834505061	0.040549121	0.867767394
AT4G02890	UBQ14	1.220452067	0.000110373	1.937241777	1.96335E-12	0.833565726	4.03837E-08	-0.21831237	0.805101198	-0.239327718	0.128646238	-0.155126097	0.390949444
AT1G07870	AT1G07870	1.26051176	0.003260609	1.935748988	2.30794E-09	0.942122751	0.003722822	-0.186149424	0.859442872	-0.206801766	0.71679006	0.085315548	0.754102564
AT5G39190	GER2	2.113774207	0.004039481	1.935717622	0.006455582	-5.466296509	0.248534793	2.320642358	0.604422229	0.544494891	0.674271991	-1.215240657	0.664511233
AT5G58620	AT5G58620	1.392751501	0.00080627	1.923733978	1.22566E-10	0.251502371	0.519834439	-0.28834021	0.726900342	-0.157697824	0.68853339	-0.279691152	0.072114744
AT3G10985	SAG20	1.535939576	1.27056E-07	1.914334259	2.27696E-41	0.871862015	2.90868E-11	0.305008352	0.680482607	0.393855807	0.007089019	0.422306929	0.000542497
AT3G06420	ATG8H	1.263297201	0.012091202	1.890296346	6.72276E-08	0.962686462	0.015397991	-0.121915097	0.936848796	0.002776026	0.998724011	-0.201797949	0.459671328
AT2G46070	MPK12	1.23722178	0.024017452	1.882884378	3.58577E-10	0.908082442	3.50505E-05	1.770402128	0.248219642	-0.070770899	0.961441656	0.09788447	0.796629073
AT1G20440	COR47	1.82985956	4.94904E-06	1.845301515	2.41388E-24	0.824280809	3.65407E-08	1.218679838	0.548879103	-0.440674456	0.002149624	-0.402401938	0.051147513
AT3G10410	SCPL49	1.522556601	1.27071E-06	1.84176557	2.52081E-15	0.866179884	0.0026097	-0.45769884	0.273353269	-0.211721587	0.344099076	-0.30248743	0.007233411
AT5G39080	AT5G39080	1.253868165	0.003842647	1.838846211	5.43542E-09	0.88372092	7.38983E-09	0.298703969	0.854231696	0.218669891	0.641167673	-0.088112539	0.777404017
AT1G76180	ERD14	1.645058955	1.39543E-06	1.824004676	2.54944E-28	0.569716015	1.4372E-05	0.150119658	0.935083701	-0.071346981	0.856243901	-0.453991584	0.05011774
AT4G23880	AT4G23880	1.26866963	0.007323118	1.822704908	4.8382E-05	0.359555801	0.362237238	-0.195890735	0.936447266	0.101603905	0.919307727	-0.6260295	0.020374369
AT3G47080	AT3G47080	1.24147539	0.006889781	1.808292285	7.59091E-07	0.608902012	0.067011473	0.381958753	0.715343553	-0.254956704	0.526650507	0.323044526	0.129968089
AT4G21350	PUB8	1.754173413	1.43304E-08	1.792062383	2.41218E-10	0.499192166	0.102270292	0.580526252	0.737650473	0.309132333	0.525334184	-0.630214652	0.002943886
AT3G53230	AtCDC48B	2.80230255	3.9259E-14	1.786623069	5.27674E-08	0.995883474	0.024868543	-0.676461344	0.624489864	0.009465957	0.992130114	-0.332096785	0.126109015
AT5G04760	AT5G04760	1.256978248	0.017104265	1.781182192	7.92593E-08	0.779964358	0.01925668	0.262830185	0.913152929	0.109058086	0.921538071	0.210531544	0.552310453
AT5G62130	AT5G62130	1.453959381	0.001629666	1.770572791	2.30747E-09	0.518149945	0.100390847	-0.296687861	0.836779419	-0.107576988	0.883729772	-0.442131703	0.026481881

AT2G01008	AT2G01008	2.255268846	0.019875089	1.770105331	0.01214325	0.801131666	0.60276543	0.934825075	0.684527973	-0.206086021	0.929498792	-0.35440363	0.772751863
AT5G44210	ERF9	1.273354832	0.038024719	1.752676707	4.58594E-06	0.323184748	0.561622098	-0.030672241	0.987679313	0.277177612	0.590426369	0.062641987	0.895230824
AT3G02150	PTF1	1.200610753	0.002583276	1.743802235	1.16978E-06	0.907732524	0.000946521	0.88625969	0.610291087	-0.117295662	0.925076802	0.291271421	0.285079317
AT2G18170	MPK7	1.840234276	2.1464E-06	1.741859038	1.78998E-10	0.80444998	0.000839765	0.361761861	0.597104923	0.121992991	0.713346329	-0.257836733	0.130301026
AT1G22710	SUC2	1.310266569	0.000432287	1.731741969	2.85445E-11	0.727813872	0.003157942	0.25733659	0.855010607	-0.148065332	0.618760168	-0.350530587	0.019188618
AT1G06473	None	2.265531672	1.20126E-06	1.727215158	3.46876E-09			-0.27468426	0.661895574	-0.164641491	0.744570338		
AT5G43850	ARD4	1.137717633	0.000501619	1.722740728	7.28252E-10	0.458339853	0.003033448	0.551022676	0.629389168	0.241247732	0.458747396	-0.312816456	0.060534758
AT1G79430	APL	1.625206574	0.001221215	1.717550015	1.05551E-12	0.869510427	0.000281531	0.721983137	0.430181703	0.418464897	0.319353019	0.172774748	0.523357401
AT4G05320	UBQ10	1.250074374	4.67483E-05	1.703788401	1.63947E-17	0.989722802	1.09579E-22	0.488151969	0.696374342	-0.259221005	0.071751927	-0.369589905	0.003233982
AT1G73680	ALPHA DOX2	1.320206704	0.000288731	1.697640495	2.18218E-12	0.788322952	0.000169994	0.454361993	0.719664849	-0.243603618	0.482903102	-0.262494661	0.191432448
AT2G05075	None	1.077102959	0.027685504	1.693650028	0.0005922			0.046563501	0.988471189	0.593656382	0.271482762		
AT4G38932	AT4G38932	2.057799644	1.88773E-14	1.690359336	5.09646E-15	0.558309607	0.001548247	-0.122989333	0.922389968	0.389009192	0.143937525	-0.373890456	0.106012179
AT5G06980	AT5G06980	1.428207799	0.001260773	1.688237544	6.48921E-15	0.876597276	1.40477E-15	2.112605256	0.100032418	0.564823753	0.006064026	0.03937654	0.852645628
AT2G19310	AT2G19310	1.232169575	0.010224686	1.685606777	1.57355E-09	0.884613567	0.000364657	-0.404922599	0.653026084	0.162638195	0.771376139	-0.459941478	0.019500254
AT5G10650	AT5G10650	1.345279529	0.002538385	1.677681085	6.06381E-12	0.851798184	5.06269E-05	-0.09581591	0.937178328	0.210718037	0.591845798	-0.523321698	0.006170302
AT1G21670	AT1G21670	1.555320995	0.000114844	1.676623373	3.44909E-08	0.704010595	3.248E-05	1.306093123	0.437249233	-0.267494047	0.550143794	-0.323202897	0.056584492
AT4G14020	AT4G14020	1.946540264	0.009777428	1.672035595	0.000277094	-0.621024696	0.040366644	0.654383737	0.640704059	0.763684304	0.082940872	0.804844283	0.003838708
AT1G11910	APA1	1.217083904	1.23614E-06	1.668621729	6.61927E-14	0.672021716	6.49982E-05	-0.19233024	0.842936823	-0.328698735	0.03250456	-0.739083083	1.08985E-12
AT1G69295	PDCB4	1.231046664	6.83298E-07	1.667643085	1.595E-09	0.698679232	0.006846137	-0.742605236	0.013621408	-0.493555885	0.009781566	-0.288821416	0.038218183
AT4G11360	RHA1B	1.499339154	0.000276323	1.648402178	1.16189E-06	0.873243014	1.06375E-07	-0.068021055	0.970302657	-0.481601965	0.108180357	-0.160473387	0.478024454
AT5G42146	AT5G42146	2.309130052	0.011959176	1.640382344	0.012794177	0.787898744	0.260784095	0.881950407	0.6903809	0.809132134	0.244822575	0.099935719	0.876735126
AT1G12220	RPS5	1.60028017	3.07701E-05	1.639330544	3.71166E-08	0.814343093	0.002996806	0.758444711	0.637815859	-0.203207345	0.793767617	-0.339175433	0.122339185
AT4G01600	AT4G01600	1.530794275	0.012513155	1.63565923	0.001023004	0.821414029	0.007875924	0.370586837	0.781802383	0.665427418	0.393043989	0.560231407	0.018420884
AT3G11930	AT3G11930	1.347721477	1.71426E-06	1.634715669	3.16977E-24	0.484127752	5.39705E-05	0.500019048	0.680416479	0.423683513	0.054082776	-0.487672656	0.014148026
AT1G04620	HCAR	1.242620019	0.003251758	1.631526668	2.09415E-13	0.752268158	7.40706E-05	0.610611011	0.665505209	0.081285407	0.862605995	0.616385231	1.56923E-05
AT3G54363	AT3G54363	1.654581909	0.000490582	1.621779487	0.001486416	0.630665178	0.1539533	0.238776213	0.859820185	0.683324741	0.234846898	0.212443414	0.596285901
AT3G49590	ATG13	1.352242789	0.001168887	1.621484895	8.84569E-15	0.957003628	6.02916E-08	0.910759725	0.509458958	0.191210242	0.66451881	0.4393755	0.024390511
AT4G18280	AT4G18280	1.91467221	0.001717187	1.618430507	0.003623854	0.811025622	0.194989622	0.18982082	0.963509602	-0.422696852	0.599487284	-1.307894549	0.123050746
AT5G60790	ABCF1	1.569339919	2.31E-10	1.607062692	5.79231E-10	0.583347168	0.015003296	-0.319816066	0.715330311	0.116503212	0.596442477	-0.513211617	1.78534E-06
AT3G59050	PAO3	1.091297083	0.002631579	1.604595839	8.14946E-07			0.169821078	0.842161979	-0.017252451	0.979747929		
AT5G45440	AT5G45440	1.441393383	0.008767755	1.597760783	0.012193459	0.569155323	0.303912724	0.019995372	0.993989575	-0.046003818	0.977360449	-0.455703026	0.081880104
AT1G78380	GSTU19	1.888804049	2.86673E-11	1.586704428	1.14271E-21	0.923757205	1.02083E-07	0.255536858	0.807334996	0.547300741	0.000221079	-0.187372177	0.31435997
AT5G64430	AT5G64430	1.096381109	0.00212683	1.585999541	1.522E-07	0.936170546	3.01249E-11	0.005543975	0.997285903	-0.360080334	0.157494921	-0.397938163	0.040672615
AT3G02140	TMAC2	1.318012314	0.00926375	1.585437307	3.19979E-05	0.835953865	6.99139E-05	0.475121343	0.717727383	-0.059413963	0.944784464	-0.596204661	0.003986656
AT5G67330	NRAMP4	1.189112201	0.000133481	1.585106125	1.07159E-07	0.868867562	4.10537E-05	0.424594337	0.774949086	0.176544104	0.663211305	-0.373561491	0.015363351
AT3G52850	VSR1	1.159712605	6.70989E-05	1.57679155	6.34903E-12	0.362510592	0.106809233	0.292810804	0.792939242	0.028174263	0.946158029	-0.266077822	0.070248539
AT2G23150	NRAMP3	1.320729675	0.002946813	1.560603811	0.001946529	0.444988755	0.315510742	-0.349205503	0.701530914	-0.334268036	0.380571063	-0.531411288	0.010834248
AT2G14878	AT2G14878	2.049478678	7.24206E-20	1.559511618	7.47081E-17	0.724901422	0.000128586	-0.270873478	0.706790319	-0.057622705	0.876125588	-0.519376237	0.002831605
AT3G23490	CYN	1.619492048	1.66001E-07	1.537140013	1.68622E-14	0.78501602	3.28445E-09	0.723400629	0.577104002	0.207004274	0.496377799	0.067719322	0.746644187
AT1G29395	COR413IM1	1.522890037	0.000635086	1.533858058	1.44012E-07	0.725792988	0.024770093	2.411568797	0.152918235	-0.133708358	0.897002087	-0.824757506	0.000258611
AT4G21570	AT4G21570	1.077561445	0.013095857	1.527436487	8.06278E-07	0.207985178	0.574818048	0.088375263	0.944330983	-0.175731518	0.542006423	0.261374691	0.104121441
AT1G10090	AT1G10090	1.167706176	0.005339243	1.527035684	3.45346E-09	0.576209113	0.041871254	0.775839134	0.657374576	-0.26075736	0.447017804	-0.335704047	0.042338225
AT4G17370	AT4G17370	1.446002134	0.001026535	1.525813775	2.78636E-06	0.277881459	0.470281771	0.627667037	0.648467857	0.389825365	0.233813969	0.108757684	0.77280831
AT5G42850	AT5G42850	1.267278482	1.23935E-06	1.522585884	1.57355E-09	0.362695765	0.248393405	0.14302281	0.838133701	0.085997659	0.855059422	-0.169811873	0.542618837
AT5G21020	AT5G21020	2.148884978	5.37879E-25	1.52059635	2.69744E-35	0.7971375	1.99501E-16	0.22971218	0.680482607	0.607797406	2.50325E-05	-0.695252352	1.45287E-07
AT4G28230	AT4G28230	1.569948391	0.000414821	1.518854662	4.18134E-11	0.909389952	0.00517468	1.106350585	0.326457456	-0.364170343	0.444342627	0.503811014	0.030442469
AT5G18600	AT5G18600	1.485679554	1.95413E-08	1.518843415	5.08141E-11	0.804951679	0.004351761	0.293808544	0.795199419	0.789799667	0.000742404	-0.899423816	0.002065459
AT4G37180	AT4G37180	1.547220278	3.22188E-05	1.512571641	9.65744E-12	0.819623063	2.32647E-07	0.903035584	0.402774567	0.192959901	0.612139308	0.376736393	0.005496344
AT2G16595	AT2G16595	1.831097382	0.009345885	1.511386542	0.013449734	0.073990858	0.907940078	-0.247248779	0.845932274	0.183491248	0.779367665	-0.23640487	0.291423003
AT4G02360	AT4G02360	2.150298613	0.002548657	1.508708528	0.008885267	1.464897599	0.135644334	-0.430017023	0.895706838	-0.693663105	0.515657999	-0.593796911	0.349322141
AT1G01360	RCAR1	1.77964646	2.05914E-07	1.498253432	2.04455E-05	0.597402907	0.049040497	-0.035109905	0.984084429	0.106496719	0.892756446	-0.820075412	3.54448E-05
AT2G17570	cPT1	1.519025179	0.002234569	1.49791143	0.000166481	0.848556891	0.007520996	0.624348688	0.563758673	0.288677367	0.579669854	-0.483786555	0.013130996
AT5G06530	ABCG22	1.150507036	0.004563722	1.492798923	1.36173E-05	0.846801313	2.8296E-05	0.766840477	0.66808962	-0.724684648	0.018795715	0.082489603	0.797932423
AT5G48412	AT5G48412	2.217236833	2.88441E-16	1.482055908	5.39231E-15			-0.158473098	0.871383083	0.16088869	0.511503982		
AT1G70810	AT1G70810	2.123110653	0.004941513	1.476278244	0.028625433	0.900400289	0.076316299	0.179291323	0.94576123	0.574895897	0.494228383	-0.873241744	0.007476801
AT5G67060	HEC1	1.526008318	0.048751583	1.473228008	0.000369595	0.634871931	0.067447935	-0.085457978	0.965260868	0.030249354	0.970474742	0.134448835	0.685503683
AT2G36950	AT2G36950	1.716748975	8.97467E-06	1.472738212	3.5538E-10	0.881047542	8.11189E-06	-0.120929452	0.947372736	-0.057366684	0.926851583	-0.887999608	8.90844E-06
AT3G57062	AT3G57062	1.103509459	0.045653173	1.468647746	0.003799647	0.010428491	0.984282258	0.194913415	0.911431059	0.139031371	0.878467109	-0.064100926	0.895158165
AT5G55860	AT5G55860	1.374707529	7.12486E-05	1.46573924	3.84662E-09	0.828553835	0.000111057	0.34453337	0.664635521	-0.042130626	0.949699423	-0.483753182	0.018969958
AT1G66240	ATX1	1.27777418	4.27148E-05	1.464630969	1.37765E-13	0.900142695	5.04731E-05	-0.335931555	0.663046749	0.079008258	0.843462618	-0.607866726	0.002471603
AT5G65495	AT5G65495	1.583820227	0.032186519	1.460978888	0.004958191	0.486396959	0.393687602	0.392726675	0.704132415	0.181680317	0.818088034	-0.641766654	0.008559225
AT4G34230	CAD5	1.536228904	1.43119E-05	1.454750204	1.932E-09	0.344134904	0.313759524	-0.554302647	0.673165787	0.100244054	0.758461089	-0.625540352	9.56792E-05
AT1G29260	PEX7	1.734129826	4.70451E-07	1.451183868	7.20935E-14	0.976289562	1.22875E-05	0.647028815	0.59142349	0.228160493	0.50786628	-0.05402869	0.819981057
AT5G18130	AT5G18130	1.420722963	0.000141215	1.448574742	2.16478E-11	0.91877653	6.42159E-08	1.369750528	0.45375294	-0.093471873	0.848060883	-0.929809902	5.54537E-13
AT2G27150	AAO3	1.113279455	0.027607015	1.443605511	1.86959E-06	0.908238071	0.00035897	-0.034873102	0.985077447	0.032456141	0.966304751	-0.335730281	0.122203522
AT3G54960	PDIL1-3	1.564279666	3.7206E-07	1.437107997	7.07847E-05	0.06113064	0.897821055	-0.894559728	0.035613542	-0.372384237	0.109186116	-0.138835663	0.434697061
AT5G40760	G6PD6	1.289224305	1.58169E-05	1.434593637	7.52649E-10	0.602240781	0.058322068	-0.354219013	0.696427231	-0.173827781	0.562368535	-0.155640928	0.278946995
AT3G10250	AT3G10250	1.339397426	1.31063E-06	1.428383244	2.76611E-09	0.807577161	4.01161E-05	0.31058444	0.753910599	-0.054765103	0.918028302	-0.331855122	0.040711032
AT5G16200	AT5G16200	1.144554283	0.020648505	1.427678387	0.005241882	-0.912359765	0.077912417	-0.552698802	0.925849553	-0.150517547	0.929239543	0.294064298	0.529001329
AT4G08980	FBW2	1.26182171	1.45955E-06	1.422851584	1.42856E-08	0.925930401	9.14681E-10	0.170734994	0.898391389	0.17754629	0.529833936	0.009988439	0.960735364
AT1G08570	ACHT4	1.425328167	4.97168E-06	1.421436649	6.74193E-08	0.413864574	0.038616111	0.261829823	0.823401939	0.08288166	0.838708765	0.180587524	0.297082319
AT1G06200	AT1G06200	1.442172659	1.90725E-06	1.408742824	7.44576E-19	0.982144938	8.17983E-13	0.418313487	0.657369817	0.195903158	0.467896265	-0.204338342	0.137733582
AT4G01280	AT4G01280	1.371189185	8.64353E-05	1.408533366	4.83058E-09	0.854822776	0.000263473	0.472050863	0.637815859	0.17989484	0.711773969	-0.338106822	0.038062818
AT3G12250	TGA6	1.127741364	0.025548547	1.405918546	0.00164002	0.957699125	4.57948E-05	0.377433656	0.719629226	-0.05678329	0.961513155	-0.405744476	0.015467012
AT2G31200	ADF6	1.084740495	0.000676347	1.398718128	1.20484E-08	0.418020014	0.178068708	0.100680023	0.93664894	0.110480086	0.792965164	0.172908898	0.479812536
AT5G05880	AT5G05880	1.357379574	0.00249383	1.39582316	0.003741245	0.441061151	0.488851194	-0.157261563	0.892607537	0.646555768	0.115382159	0.729596761	0.000261728
AT3G48800	AT3G48800	1.383227647	0.002422141	1.395701711	0.004774287	-0.093856273	0.836329338	0.188093496	0.943633329	0.185939021	0.782369045	-0.65273682	0.056191618
AT4G39090	RD19	1.534754397	4.3657E-06	1.386857009	2.10129E-11	0.82088644	0.05143824	0.288736102	0.836212936	-0.199607933	0.283155947	-0.391221253	6.29524E-05
AT5G63625	None	2.217997184	0.019392148	1.386126132	0.041447262			1.172767709	0.664635521	0.407396545	0.796907255		
AT5G23460	AT5G23460	1.228872946	0.044818479	1.385050092	0.001186009	0.456440295	0.458498435	0.248834371	0.899163675	0.031782466	0.974078437	-0.259524879	0.512034786
AT1G16250	AT1G16250	1.445072075	0.000223673	1.380547881	4.5664E-06	0.83347455	5.10566E-06	0.848652958	0.620403615	0.541665763	0.157627029	-0.487685975	0.070063189
AT3G04710	TPR10	1.684369382	0.012743932	1.38043012	0.001441064	0.655419333	0.081396439	-0.183904961	0.942727755	0.06310299	0.925388746	-0.229269166	0.266001972
AT2G45720	AT2G45720	1.847818334	1.12205E-05	1.37804746	0.015363874	0.815227091	0.012994215	-0.262725507	0.842936823	0.066828409	0.947242295	-0.665599003	0.000450039
AT1G01730	AT1G01730	1.515426885	4.22551E-06	1.375297159	4.39238E-07	0.935710854	2.7737E-06	0.001044214	0.999285124	-0.100038666	0.79023526	-0.295948849	0.032065301
AT4G03200	AT4G03200	1.049639626	0.002538385	1.367934925	1.3929E-09	0.869487218	4.94132E-06	0.547306462	0.648919095	0.294253641	0.348918221	0.291931684	0.196807381
AT3G28050	UMAMIT41	1.452861656	1.75455E-10	1.367229899	9.30983E-11	0.930596488	4.32793E-10	-0.16602435	0.885840009	-0.027486649	0.950828272	-0.57892596	2.8261E-05
AT2G22430	HB6	1.522020356	3.8118E-08	1.365116041	1.03406E-17	0.66224423	0.001065568	0.512850447	0.43725222	0.301739209	0.051294254	-0.164472165	0.386994353
AT3G12530	PSF2	1.420974071	0.003073638	1.362969335	0.000201219	0.82357867	0.03930066	0.609554805	0.676109289	0.250603234	0.611423968	0.120972576	0.711493724
AT4G30960	SIP3	1.238472478	0.000243536	1.362919693	1.62955E-08	0.417885957	0.018042104	-0.100696297	0.93480387	-0.003865953	0.993586481	-0.620521302	7.13362E-05
AT3G16190	AT3G16190	1.293681122	2.71505E-06	1.361404577	4.03708E-11	0.996411171	5.4667E-06	0.586047115	0.614294893	0.203403119	0.516852689	0.048238296	0.840220331
AT5G13750	ZIFL1	1.184581495	0.010394186	1.360422189	0.015842865	0.804304512	0.014939986	-0.888223982	0.2451362	-0.153092649	0.805955561	-0.226848457	0.440888363
AT1G13930	AT1G13930	1.219046146	0.015569033	1.358261242	4.00805E-11	0.346883367	0.124873349	1.672070985	0.334544782	-0.388333386	0.230607776	-0.195171109	0.522236388
AT4G34180	AT4G34180	1.437675819	1.15396E-08	1.357621132	8.79581E-13	0.82526504	2.65597E-05	-0.17889613	0.840363187	-0.282114082	0.385602602	-0.703913432	2.63823E-06
AT1G50600	SCL5	1.216335821	0.001673371	1.353430078	4.84894E-14	0.976169967	6.33231E-13	0.54447125	0.691895802	-0.07326276	0.839992273	-0.272070267	0.051994371

AT2G27490	ATCOAE	1.611242298	0.000340785	1.352810209	0.000243392	0.848486416	0.023511909	0.196974728	0.943633329	0.539922971	0.199674929	-0.252866669	0.529001329
AT5G57060	AT5G57060	1.124390053	0.013092645	1.351037852	0.000405099	0.428640485	0.453210678	-0.524039314	0.665778009	0.235123708	0.716628523	-0.131911951	0.71308565
AT1G09740	AT1G09740	1.886722838	1.87666E-10	1.349461173	2.11397E-06	0.438790028	0.057798078	-0.086513591	0.952762809	-0.673447487	0.003657892	-0.674570966	0.000982451
AT5G02380	MT2B	1.536041603	2.25369E-12	1.346002042	3.95317E-37	0.80474032	0.015236061	0.643520577	0.445447639	0.459387875	0.020961467	-0.160971514	0.903335217
AT1G36730	AT1G36730	1.452145037	2.25187E-06	1.337828822	7.74186E-25			0.391306087	0.735907718	0.023268216	0.95731077		
AT2G23120	AT2G23120	1.591078083	0.006061764	1.332829577	1.02085E-07	0.69170602	0.007902664	1.218526001	0.449207924	0.104718245	0.867566449	0.014404976	0.989597198
AT3G48530	KING1	1.581905144	2.39962E-08	1.332606698	1.68805E-17	0.518027248	0.010332193	0.866216223	0.537325911	-0.105796207	0.69079026	-0.269203906	0.043907952
AT3G14200	AT3G14200	1.394340957	0.000872447	1.332034452	0.005319065	0.46019727	0.350666171	0.262967639	0.782181302	0.059174988	0.9202103	0.194354695	0.47093389
AT5G58070	TIL	1.030999288	0.013276865	1.323517976	3.14574E-07	0.261804753	0.135968214	0.735818865	0.622746033	-0.054861021	0.929239543	-0.128445075	0.424255611
AT5G16360	AT5G16360	1.808588896	6.29066E-05	1.321320956	0.004332167	0.774706842	0.110815322	-0.561837579	0.674766805	-0.270362973	0.512974126	-0.617097138	0.006328738
AT2G13965	None	1.42434891	0.006915167	1.319798901	7.32118E-05			1.072863694	0.613859108	-0.234696668	0.736648978		
AT5G28830	AT5G28830	1.274383129	0.005569987	1.314197105	0.000839746	0.931226277	5.12838E-05	-0.000489816	0.999867318	0.194957588	0.76862131	-0.624809192	0.011636892
AT2G45960	PIP1B	1.018856791	0.001081874	1.305100053	5.64561E-15	0.52566936	9.72199E-07	0.090241401	0.956962717	-0.367785745	0.001702347	-0.520562368	9.81999E-06
AT3G14750	AT3G14750	1.263515792	0.001453197	1.300507661	3.848E-07	0.939208879	0.001363169	0.922030301	0.629761053	0.207576957	0.684831837	0.260516269	0.244048785
AT4G36840	AT4G36840	1.013438223	0.007818142	1.298437146	0.000604426	0.178710868	0.646155962	0.830324269	0.598901706	0.017859197	0.984765765	0.009728272	0.97995131
AT1G25520	AT1G25520	1.277937636	0.000416499	1.297574269	5.38969E-05	0.693548826	0.052794428	-0.287356879	0.753910599	0.276225285	0.547993779	-0.639507534	0.008976616
AT1G60730	AT1G60730	1.190745718	0.001759542	1.289603396	7.95885E-06	0.339621851	0.57695454	-0.381893096	0.86582927	-0.552633205	0.133305554	-0.573722387	0.000280761
AT2G19810	OZF1	1.078032316	0.014532217	1.287744345	2.12724E-06	0.724368497	0.00013189	1.422324205	0.393926565	0.353053681	0.186040855	-0.03929163	0.895286666
AT2G15980	AT2G15980	1.197627241	0.030743855	1.285029072	0.039978153	-0.81607693	0.327416325	-0.161847536	0.941453781	0.063801149	0.957434731	-0.846718431	0.028454881
AT3G13910	AT3G13910	1.252521542	0.006289725	1.283955661	0.01019035	0.71481517	0.195074184	0.249907301	0.885628632	0.153749282	0.753697842	-0.30315972	0.196939952
AT1G71697	CK1	1.312205192	0.00220396	1.283327164	3.22424E-05	0.694240885	0.00629508	-0.026207532	0.981893161	0.121488451	0.831451868	-0.541842316	0.042044016
AT3G03330	AT3G03330	1.412327677	0.000274577	1.282603694	2.90522E-09	0.826586735	6.26352E-07	0.453205975	0.719629226	0.183571122	0.654339197	-0.226229042	0.110120598
AT5G16550	AT5G16550	1.151402668	0.000131768	1.279169477	0.003285137	0.425609774	0.142408232	-0.513983386	0.349541385	-0.274843463	0.374534812	-0.608500037	8.81401E-06
AT1G08800	AT1G08800	1.662564952	4.70268E-08	1.278888653	0.000326354	0.889186585	0.001151483	-0.246053062	0.794754875	-0.480520761	0.04830872	-0.971243817	7.39143E-06
AT3G21750	UGT71B1	1.144060859	0.008041759	1.276536278	0.000973701	0.8529696	0.055894208	-0.451218878	0.618907586	0.109530156	0.820927434	0.098318577	0.630364052
AT5G58800	AT5G58800	1.005833038	0.009386672	1.274125804	3.99764E-05	0.879201127	5.22492E-06	1.026717019	0.591481547	-0.053378567	0.944342934	0.230967276	0.312422473
AT2G24940	MAPR2	1.962148571	2.50384E-16	1.273008484	3.71853E-13			-0.085319827	0.921880854	0.120916934	0.709696358		
AT3G51880	HMGB1	1.810359441	3.47873E-07	1.272329625	2.11661E-29	0.940992384	2.21438E-18	0.656808371	0.642268593	0.073207185	0.831684104	-0.702501489	0.215145544
AT4G23630	BTI1	1.165071333	6.07816E-08	1.271731715	3.67508E-09	0.333179163	0.175345668	-0.350908341	0.380161231	-0.198086274	0.423442669	-0.231101949	0.0487893
AT4G02860	AT4G02860	1.084402122	0.001691604	1.269882454	4.60358E-06	0.588394344	0.025786013	-0.346747949	0.532231419	-0.276338457	0.3051444	-0.250493686	0.124073453
AT3G45590	SEN1	2.125441284	0.014260931	1.266990907	0.020427281	0.872474061	0.052663032	0.287259005	0.854170198	0.493946139	0.591803993	-0.03430366	0.934992181
AT1G73380	AT1G73380	1.085501579	0.01391925	1.265205453	2.13298E-07	0.831708564	0.000267069	0.63089356	0.627500883	0.287007867	0.429416497	0.097735134	0.644577682
AT1G02816	AT1G02816	1.196997907	5.71139E-05	1.264786061	0.000133005	0.96593679	0.001330856	-0.305836339	0.653363031	-0.275156212	0.493875814	-0.19765245	0.417536098
AT5G53220	AT5G53220	1.109867197	0.039094449	1.261936724	0.015632642	0.890971955	0.110600886	0.161520131	0.883538452	0.352674977	0.662608549	-0.085493922	0.810597859
AT3G54130	AT3G54130	1.196297678	0.005199384	1.26117787	0.006309315	0.958534319	0.003360377	0.284973063	0.800906229	0.079820682	0.924627441	0.224923226	0.407265506
AT1G48550	AT1G48550	1.284372553	0.005242217	1.260180123	7.3613E-09	0.630604815	0.006366455	1.274449044	0.322558315	0.30432521	0.430032117	0.027683789	0.936148146
AT1G22190	AT1G22190	1.045502133	0.00874741	1.258093343	7.65296E-07	0.213769039	0.181643925	0.675044519	0.662758454	-0.230490971	0.39386988	0.354322742	0.049842427
AT1G15230	AT1G15230	1.328120705	0.000181419	1.251842978	3.11585E-08	0.522808835	0.082291205	0.073594014	0.94895451	0.384017186	0.275351974	0.001667394	0.995979128
AT5G58650	PSY1	1.174315873	0.025295238	1.227023073	0.001019372	0.58278665	0.04665877	0.64642205	0.66417735	0.487337469	0.312819999	-0.307105223	0.381711424
AT5G12120	AT5G12120	1.107139564	0.00021205	1.222741328	1.88476E-15	0.855688906	2.07614E-12	0.891918376	0.505950107	-0.188769129	0.55787595	0.041060719	0.815256026
AT4G02970	AT7SL-1	1.143482341	0.017125179	1.218256946	6.21262E-07	0.499083756	0.223503713	0.105799254	0.928193379	0.272761228	0.490474456	0.590809399	0.036673569
AT5G47060	AT5G47060	1.436426415	0.000864366	1.216198787	0.00910424	-0.102134279	0.876371208	-0.772962406	0.616300683	0.045245856	0.938970422	-0.422077274	0.032162778
AT5G17900	AT5G17900	1.328929026	0.000188654	1.215365143	1.52962E-14	0.237730988	0.584319658	0.874553222	0.473099145	0.233705017	0.470295104	-0.312247087	0.270365533
AT3G22480	PDF2	1.549365747	1.43632E-06	1.207744712	8.23179E-07	0.58908415	0.000630953	0.441636447	0.667782687	0.289419298	0.360033758	0.268382773	0.101877757
AT2G22860	PSK2	2.639106157	6.17998E-14	1.206102504	0.000953648	0.900547487	0.000964575	-0.014308304	0.994162268	0.463148307	0.260685017	-0.545175226	0.002927356
AT2G38410	AT2G38410	1.439895699	5.37416E-05	1.195060411	2.86674E-08	0.932426172	7.00297E-07	0.265619377	0.847660958	-0.153602219	0.709696358	-0.438830934	0.003564742
AT1G17520	AT1G17520	1.070217814	0.004973967	1.19326339	7.0106E-05	0.882959917	0.000162388	0.682262602	0.559125018	0.050665381	0.947508153	-0.038133601	0.900704599
AT1G01650	SPPL4	1.040622562	0.012089074	1.191174552	3.81269E-05	0.796092622	0.000302976	-0.150659503	0.908064459	-0.327482248	0.212664929	0.043323478	0.834517012
AT4G05631	AT4G05631	2.218768196	4.24E-08	1.190003288	0.011491861			-0.126479101	0.970302657	0.622356748	0.233813969		
AT1G56290	AT1G56290	1.311486533	0.003448912	1.18806966	0.000157389	0.777405243	0.000340582	0.049426839	0.982183273	0.184138404	0.781706477	-0.386226237	0.039925808
AT2G04890	SCL21	1.834759152	7.41777E-05	1.185885237	0.000135056	0.8774742	6.64861E-05	0.116474952	0.923622633	0.3067427	0.424511917	-0.330413281	0.133985171
AT5G21940	AT5G21940	1.149972454	0.000286869	1.181258156	0.000134821	0.258902992	0.070970854	0.25955117	0.816446062	0.387062595	0.029329363	0.067690414	0.741859289
AT4G32340	AT4G32340	1.548362515	0.00785228	1.180835768	2.75913E-06	0.192093555	0.500700357	0.79093565	0.594193754	0.316120536	0.487021579	-0.037633029	0.916394659
AT3G05880	RCI2A	1.40582169	0.000661816	1.179854476	2.50978E-08	0.431798692	0.13407108	-0.477752932	0.25826973	-0.22681505	0.407635739	-0.720334506	7.99663E-05
AT4G01960	AT4G01960	1.145851663	2.83447E-05	1.176310075	1.90254E-06	0.789847291	0.006227713	0.011830569	0.99377782	0.251977252	0.470607346	-0.092401159	0.694466763
AT3G63010	GID1B	1.527074598	0.002036548	1.173609982	0.003442182	0.402469466	0.333369762	-0.091074889	0.971297857	0.147240825	0.851691904	-0.457604023	0.07890576
AT5G58020	AT5G58020	1.423411819	1.15178E-06	1.172303301	1.53407E-08	0.643629414	0.010812731	0.386578823	0.784469624	0.180340658	0.564913091	-0.445054459	0.01440009
AT5G28320	AT5G28320	1.151227758	0.008767034	1.169673597	0.00093337	0.615804024	0.249045006	0.96045097	0.647947756	0.074753196	0.933843556	-0.128188704	0.761927143
AT1G21190	LSM3A	1.589904608	3.44553E-05	1.169642619	9.25383E-05	0.404320997	0.183822981	0.216568571	0.821043512	0.495552394	0.096500101	-0.385036377	0.055626975
AT5G51570	AT5G51570	1.701590649	0.000439863	1.16911	0.001130266	0.40836475	0.322410796	-0.408623864	0.619462504	-0.135149578	0.7740683	-0.505383283	0.004232198
AT1G52347	AT1G52347	1.360541863	0.00236633	1.167232579	0.003784826			0.492420545	0.759771605	0.030889493	0.975386078		
AT3G24840	AT3G24840	1.865408798	0.003089364	1.164472473	0.035434639	0.121882646	0.876421535	-0.308698529	0.742807747	-0.342741053	0.478838042	-0.18019802	0.550420112
AT5G62070	IQD23	1.012998817	0.0070302	1.159633156	0.005531409	0.056008525	0.904234087	-0.171905064	0.836614072	-0.268406991	0.500891025	-0.197525454	0.199496512
AT3G21790	AT3G21790	1.040520129	0.006403411	1.157511951	1.01897E-07	0.898559945	7.08252E-06	1.315607158	0.55995834	0.02544275	0.979610562	0.167430265	0.429123865
AT3G48680	GAMMA CAL2	1.484760329	2.99805E-09	1.157425232	1.91149E-11	0.743306762	4.86945E-07	0.5199204	0.613859108	0.194596113	0.33502325	0.099880902	0.663311146
AT1G47056	VFB1	1.114011643	0.009501259	1.145778133	3.93239E-07	0.720670959	0.004158167	0.188415306	0.94654169	0.092903789	0.87385307	-0.257526108	0.27850998
AT1G19660	AT1G19660	1.091282726	0.001298964	1.14433044	8.08447E-08	0.964963192	4.28613E-14	0.788378858	0.640445007	0.308684929	0.164549923	-0.213161948	0.372130385
AT5G63370	AT5G63370	1.087730287	0.000330378	1.137265967	1.22706E-07	0.890258741	2.14269E-16	0.5489378	0.680482607	0.089237594	0.83365656	-0.242585755	0.148392197
AT3G05120	GID1A	1.165910674	0.014004488	1.131665075	9.80363E-05	0.356973737	0.137584232	-0.084059729	0.966109877	0.101762186	0.785089338	-0.235986653	0.17799253
AT5G64180	AT5G64180	1.012082256	0.001834475	1.127819841	7.9908E-07	0.501751581	0.002521675	0.389596957	0.616300683	0.111697081	0.793774805	-0.333095922	0.122810309
AT1G09415	NIMIN-3	1.063678504	0.044026425	1.126982968	0.000619684	-0.961343548	0.012143998	0.60624832	0.574375676	0.159806074	0.835674303	-0.156441811	0.654863071
AT1G28580	AT1G28580	1.227392359	2.26061E-08	1.12014353	1.8415E-08	0.674677619	4.25898E-05	-0.176061257	0.76449563	0.000599015	0.999129744	-0.121869736	0.379734297
AT2G46080	BPS2	1.080573955	0.001821274	1.119292977	0.000653737	0.689046445	0.001450179	0.291307134	0.712045709	0.161042751	0.676169382	-0.43356113	0.003100225
AT5G07470	PMSR3	1.207261759	4.2051E-07	1.114739427	4.40845E-07	0.828418121	9.40927E-08	0.188928953	0.86998549	-0.074546412	0.851431725	-0.230723929	0.150809311
AT4G32210	SDH3-2	1.552625941	0.00052268	1.11380035	0.003386196	0.542696522	0.117367087	-0.212568105	0.834638751	0.076342198	0.907678975	-0.184934138	0.4846429
AT3G26360	AT3G26360	1.03252441	0.002190961	1.104937202	1.48187E-05	0.443913681	0.040114551	0.262542848	0.756939148	0.520805436	0.102835055	-0.027490317	0.918008373
AT3G13380	BRL3	1.469288554	0.008797091	1.104874816	0.003692732	0.56829102	0.221680467	-0.899292008	0.430181703	-0.251742853	0.684508482	-0.936979731	6.35784E-05
AT5G03200	LUL1	1.238963362	0.00010019	1.103524841	5.40931E-07	0.892454207	2.21999E-05	0.116392257	0.94974555	0.030529383	0.971701608	-0.051857642	0.867604777
AT2G47600	MHX	1.216841134	0.002448788	1.101443192	0.00154575	0.98695926	4.13296E-07	-1.086743702	0.182784775	-0.247264601	0.355974588	-0.500683583	0.007320248
AT4G35040	bZIP19	1.078716432	0.001153098	1.099030507	0.000429815	0.546400801	0.010198132	0.104904095	0.936758504	-0.079366235	0.862495589	-0.290595006	0.051929979
AT3G02360	AT3G02360	1.009793375	3.15156E-05	1.097562267	1.62513E-11	0.674706221	0.000234408	-0.080030745	0.940518648	-0.01378066	0.972421502	-0.112389057	0.421954352
AT4G32320	APX6	1.006743741	0.006157248	1.095693982	2.90686E-05	0.977949159	1.48909E-10	0.677681211	0.603726755	-0.336604726	0.290601273	0.182415777	0.3498426
AT5G19740	AT5G19740	1.393806037	0.010806446	1.093256656	0.00443994	0.979541972	0.00026457	-0.711392513	0.610227484	-0.199282331	0.471663512	-0.427757379	0.029541058
AT4G10080	AT4G10080	1.147642844	0.00196291	1.092834484	1.10053E-09	0.51660784	0.004673095	0.874860634	0.563758673	0.319923765	0.209134688	-0.356674211	0.190523866
AT5G42570	AT5G42570	1.410804111	0.001023615	1.092466953	8.70448E-05	-0.096621667	0.919211379	-0.517752363	0.289915023	-0.288447144	0.359234864	-0.452766683	0.002685039
AT1G79410	5-10月	1.561175229	5.81641E-06	1.089930646	0.00390807	0.233921476	0.566026333	-0.303069061	0.709338752	-0.140607404	0.758375211	-0.706385953	2.74833E-05
AT2G01540	AT2G01540	1.501994485	6.86617E-05	1.088625275	5.0292E-06	0.502635761	0.027052794	0.094603307	0.932578056	-0.106370295	0.863621528	-0.136644849	0.498505889
AT2G16710	AT2G16710	1.291460885	0.008827364	1.086475674	0.007032017	0.544774164	0.01344158	0.10695781	0.916241006	-0.133410354	0.818982935	-0.264684915	0.232535592
AT5G40340	AT5G40340	1.294806286	0.000605118	1.08257266	7.40787E-05	0.719506189	0.00413279	0.339652608	0.726943566	0.058676686	0.905953395	-0.635988172	0.007491237
AT3G27090	AT3G27090	1.155187415	1.51451E-05	1.081394241	1.58884E-16	0.963598015	2.89218E-21	0.489258072	0.633171846	0.133712591	0.554072186	-0.564696332	9.72839E-08
AT1G51600	ZML2	1.229827518	3.1407E-05	1.079580914	2.1309E-07	0.905004088	0.000111667	0.609159931	0.180725221	0.257869799	0.484585201	0.033773934	0.879522531
AT1G75810	AT1G75810	1.44430117	0.00080871	1.073098161	0.017048787	0.094152955	0.8649166	-0.161149717	0.94632944	-0.257466776	0.755587242	-0.588147733	0.056111431
AT5G23590	AT5G23590	1.135486271	0.001093852	1.070514517	5.15478E-05	0.942760685	4.05763E-06	0.209593627	0.842161979	0.325734561	0.185410599	-0.09962049	0.730643373
AT2G27200	AT2G27200	1.299209136	0.021331362	1.070334438	0.000576327	0.769760616	0.005043368	0.073059303	0.953045007	-0.223745675	0.670885618	-0.145409445	0.555246218

AT2G41070	EEL	2.074496699	0.005735697	1.069085886	0.012672663	0.963056159	0.008913014	-0.400327775	0.797973064	-0.014438545	0.977067667	0.017721242	0.931594029
AT5G09590	MTHSC70-2	1.190972661	0.001040345	1.067414695	5.16045E-06	-0.048239771	0.914591345	-0.120653195	0.947241707	-0.092353232	0.824058587	-0.332684494	0.042559605
AT1G76560	CP12-3	1.088722156	0.038664501	1.062598391	0.013871451	0.510474557	0.12041398	0.356849228	0.796607769	-0.01078284	0.992775435	-0.632340783	0.193675575
AT5G66250	AT5G66250	1.052276506	0.010096142	1.06019393	5.30159E-05	0.956746764	1.18828E-09	0.634593843	0.598901706	0.274281014	0.343212533	-0.200568507	0.300479639
AT3G08520	AT3G08520	1.254049926	3.44557E-06	1.059513989	1.00538E-10	0.603519437	0.008349017	0.272061786	0.62738272	0.52472875	0.043532871	0.08063221	0.824357435
AT1G18260	EBS5	1.064352715	0.00129027	1.059224288	3.53419E-05	0.764511014	0.000393667	0.117040675	0.92946974	-0.050624563	0.924140734	-0.589326988	0.000769669
AT3G57340	AT3G57340	1.123856534	6.14032E-05	1.053075712	1.13777E-07	0.959530637	1.19001E-08	0.781654762	0.452963543	0.273638155	0.267463237	-0.010450223	0.963175545
AT1G70360	AT1G70360	1.715002325	0.03839471	1.050910237	0.011844718	0.339913602	0.42155226	-0.349385851	0.805968219	0.331727043	0.637101903	-0.474296914	0.153434995
AT5G37260	RVE2	1.406274442	4.79889E-05	1.050839939	0.029075241	0.409168295	0.254616351	-1.103047228	0.1682865	-0.406094863	0.057650203	-0.363317628	0.119282882
AT1G20100	AT1G20100	1.023482977	0.00721145	1.050510541	0.000370631	0.703125826	0.00020646	0.319091316	0.668028007	-0.027766333	0.950034363	0.052616775	0.834601312
AT3G07760	AT3G07760	1.036103242	0.002319318	1.048325509	2.96154E-07	0.512452579	0.038011903	0.597496253	0.62738272	0.12221794	0.707408013	-0.302965085	0.026569603
AT3G17020	AT3G17020	1.336846898	4.25601E-06	1.045193042	2.68598E-15	0.629262893	5.89958E-05	0.233430837	0.794188368	-0.050412171	0.905571805	-0.5329318	0.021953354
AT2G23090	AT2G23090	1.289973607	6.75123E-05	1.044563776	9.03945E-05	0.894090862	9.61165E-15	-0.10969852	0.928888991	0.248348629	0.498823832	-0.369768419	0.026957864
AT4G39100	SHL1	1.168504834	0.000677711	1.043723549	5.97082E-10	0.801940024	8.02119E-07	0.705367184	0.55977909	0.041351263	0.930270964	-0.290250191	0.106434118
AT4G14615	AT4G14615	1.198046369	0.000112468	1.043491334	7.912E-07	0.051373638	0.892776575	0.446908561	0.330164829	0.253176851	0.493234927	0.247862374	0.115956288
AT2G33700	PP2CG1	1.0820286	0.005445908	1.03810552	0.004383295	0.10756148	0.825656666	-0.105337808	0.936447266	-0.304052792	0.446788505	-0.43460713	0.006880292
AT1G31830	PUT2	1.543669436	5.0852E-07	1.037411771	5.80499E-05	0.85511206	0.002022034	-0.109442487	0.917312631	0.011456811	0.986818647	-0.311068193	0.056401791
AT5G55140	AT5G55140	1.677204524	2.66704E-05	1.036740185	1.2277E-06	0.916202847	1.38944E-08	0.302264269	0.709909038	0.168365354	0.692574508	-0.135682228	0.505592628
AT2G37200	AT2G37200	1.260953353	0.001560528	1.029805209	0.00019182	0.734712466	0.015293651	0.013606176	0.992570276	0.024870074	0.968000197	-0.047592668	0.843752657
AT3G51530	AT3G51530	1.002918587	0.034599599	1.029568852	0.003577673	0.679498393	0.028697351	0.213954485	0.858510704	0.11433315	0.871060407	0.236617325	0.353484247
AT3G01470	HB-1	1.083043767	0.000804302	1.022290944	1.0439E-07			-0.124375063	0.892607537	-0.303034296	0.074898598		
AT5G16470	AT5G16470	1.625053413	5.60929E-08	1.021225784	7.24558E-13	0.886973103	7.01651E-12	0.193366006	0.808237728	0.197477926	0.398166246	-0.399005233	0.017977728
AT1G02860	NLA	1.198246759	0.000196236	1.020453098	0.018225174	0.993503718	6.40593E-10	-0.292683449	0.847546649	-0.274519083	0.52838553	-0.351510933	0.021352599
AT1G56280	DI19	1.31557148	4.9254E-07	1.020056336	1.76784E-13	0.470530619	1.12107E-05	0.560277954	0.607117655	0.188934397	0.234902133	-0.62724696	6.03467E-11
AT5G14680	AT5G14680	1.227472274	4.99136E-07	1.019842059	0.000412311	0.944937739	1.13902E-08	0.589206397	0.62738272	-0.047058976	0.930954102	-0.346010252	0.013712297
AT5G12140	CYS1	1.004364561	1.13766E-05	1.019174082	6.03984E-07	-0.572805313	0.000547743	-0.225437814	0.818515078	0.089672834	0.788704297	-0.261645893	0.309812644
AT2G17870	CSP3	1.288997714	0.000559167	1.019028741	0.018671743	0.080383311	0.856949008	0.091768667	0.954839264	0.32984489	0.412791131	-0.766857045	0.24840968
AT4G05070	AT4G05070	1.1430507	0.006289725	1.018038821	0.003493904	0.115631042	0.605026797	0.684479336	0.647435191	0.056778684	0.91147162	-0.49937267	0.074477694
AT1G07530	SCL14	1.074550518	0.000393078	1.017532943	3.02901E-07	0.753773556	5.46249E-08	0.403179317	0.697407195	0.111347652	0.683738162	0.080852705	0.719365777
AT4G21510	FBS2	1.125838019	0.015962143	1.015632098	0.000511046	0.284504417	0.349896561	1.175173501	0.458341345	0.413466736	0.458804094	-0.217132749	0.575379961
AT5G07220	BAG3	1.258510325	0.000202297	1.014841875	1.00525E-05	0.91772239	7.38355E-12	-0.071002862	0.948630245	-0.172475895	0.4584002	-0.958853391	4.42823E-09
AT1G30880	AT1G30880	1.386709571	2.09688E-13	1.012376219	8.76995E-13	0.955896458	1.12047E-12	0.370269478	0.606948021	0.372692033	0.012526034	-0.053608622	0.76087978
AT3G62880	ATOEP16-4	1.020764937	0.032828657	1.011389162	0.018411687	0.926524638	0.016470448	0.792401352	0.470585322	0.173357779	0.823358742	-0.333827465	0.216645715
AT5G57110	ACA8	1.137200109	0.005120221	1.011075496	0.000124519	0.930598064	0.003110583	-0.737383696	0.313609174	-0.319973911	0.119428864	-0.068238677	0.748934697
AT4G21620	AT4G21620	1.955327283	0.003480693	1.007778072	0.005046884	0.145746555	0.777652816	-0.867770892	0.491585303	-0.076167822	0.886139087	-1.374200148	0.123881284
AT3G07590	AT3G07590	1.111731322	0.013699403	1.007554248	0.012577855	0.405589617	0.517777741	-0.17834899	0.947241707	0.334670804	0.428068121	0.199513444	0.306896538
AT1G78550	AT1G78550	1.065178727	0.02912114	1.002824545	0.019454582	0.495361098	0.373279542	0.097349646	0.958212376	0.016837461	0.986818647	0.202342621	0.539557172
AT1G75720	AT1G75720	7.209368781	0.023934172	-5.957796102	0.032969047	-1.076863557	0.661691353	0.37038583	0.964220939	-0.414104303	0.812750957	0.122139623	0.907973202
AT4G26320	AGP13	8.199472143	0.001443865	-7.846199597	0.000580456	-0.494431392	0.858123609	-1.734400626	0.665778009	0.017148878	0.991419859	-0.195786242	0.714700217
AT4G17970	ALMT12	-1.010820905	0.021084685	1.31391899	0.003575109	-0.657840158	0.302362647	0.045149775	0.987613184	0.0506949	0.973034706	0.078800186	0.901239449
AT4G08870	ARGAH2	-1.419611638	0.025270749	1.06095623	0.003096754	0.101092217	0.853800171	0.832999254	0.677952867	-0.182813708	0.868126439	-0.483402441	0.379743034
AT4G19170	NCED4	-1.680380281	5.05231E-06	-1.005662011	0.003336479	-0.912644	6.92441E-10	1.964609719	0.393567324	0.128389222	0.927507051	-0.7282264	0.000461035
AT3G15690	AT3G15690	-1.040647818	2.20514E-05	-1.006051063	0.000802229	-0.81374558	2.65101E-07	0.055074886	0.962474985	0.15399266	0.646759677	0.266701624	0.124531908
AT5G17670	AT5G17670	-1.492821591	6.63328E-05	-1.008519413	1.73204E-05	-0.195620387	0.313342042	1.173499341	0.518492932	0.702423555	0.088819089	0.424063952	0.008477353
AT2G26710	BAS1	-1.312645153	0.001967889	-1.013522384	0.001802314	-0.274980239	0.445196462	0.005251846	0.997285903	-0.177042598	0.809528287	-0.653152991	0.003300655
AT5G28450	AT5G28450	-1.425933091	0.014889469	-1.014085188	0.010093478	-0.553321819	0.000110693	1.847207765	0.469832585	0.931268687	0.458380546	0.755924422	0.050233641
AT2G31370	AT2G31370	-1.082173266	0.004077745	-1.019673373	5.68474E-08	-0.907154432	2.55563E-06	-0.214553826	0.716004594	-0.25611656	0.415944499	0.091413599	0.62035353
AT5G02120	OHP	-1.780968315	2.73667E-05	-1.023524484	6.96624E-05	-0.946327176	9.87311E-07	0.864845378	0.604415921	0.341130656	0.563104532	0.518930442	0.04191787
AT1G14270	AT1G14270	-1.426289035	5.81641E-06	-1.027666982	2.91466E-05	-0.459779936	0.028596204	0.276692181	0.748160003	0.15227985	0.682180924	0.728311411	1.08851E-07
AT3G02830	ZFN1	-2.014995901	3.13599E-09	-1.033948324	4.35734E-06	-0.485281149	0.02287106	0.391881464	0.696374342	0.002766996	0.99783138	0.753292496	7.79965E-05
AT3G48820	AT3G48820	-1.457567291	0.000983011	-1.034731761	0.011057996	-0.326968848	0.305640935	-0.311637454	0.767987589	-0.051041811	0.934318497	0.14066371	0.525088565
AT3G48200	AT3G48200	-1.590146225	2.93521E-05	-1.035721422	0.000350827	-0.619972349	0.010343746	0.111457043	0.929506087	0.270993214	0.477463307	0.250168612	0.191274428
AT2G37620	ACT1	-1.241631444	0.026256329	-1.036431085	0.009321338	-0.868912513	4.12979E-06	-0.002827335	0.998419999	-0.017782183	0.977736057	0.528147786	0.001741424
AT2G26080	GLDP2	-1.451773141	6.35049E-07	-1.042046429	3.57298E-08	-0.060517089	0.826918292	0.619053356	0.514439527	0.002236871	0.996165588	0.202539353	0.317982991
AT1G12800	AT1G12800	-1.856861052	2.60403E-14	-1.048692099	7.66935E-08	-0.993928795	5.75671E-10	0.266332111	0.793776337	0.094632553	0.82334304	0.23531486	0.133561096
AT2G01260	AT2G01260	-1.058267729	0.00728243	-1.049436938	0.00225868	-0.490693618	0.076523089	-0.528246964	0.653915725	-0.056873835	0.945235704	-0.103330727	0.683297186
AT3G54460	AT3G54460	-1.191575413	4.28931E-05	-1.049536788	6.11749E-05	-0.58514182	0.002651704	0.465771088	0.66524984	0.013103161	0.986818647	0.060911205	0.804433334
AT3G05910	AT3G05910	-1.415064581	3.08944E-05	-1.050649249	7.19065E-10	-0.540139169	0.005570799	-0.605778253	0.593831668	-0.258831564	0.306223133	-0.199360432	0.102348537
AT1G34210	SERK2	-1.115915426	1.33288E-05	-1.053280782	0.003332693	-0.650246081	0.033691519	-0.026397844	0.985396223	0.079626942	0.883566526	-0.017122973	0.958043778
AT2G42320	AT2G42320	-1.064375945	0.006107198	-1.055873678	0.000176053	-0.93848038	4.41061E-07	-0.157123085	0.86582927	0.021070898	0.962321358	0.248580608	0.099845285
AT4G10470	AT4G10470	-1.441622771	0.000273351	-1.057174377	0.044705889	-0.474397786	0.193507277	0.320734939	0.839420713	0.316797271	0.57878639	0.788185264	0.000144328
AT3G11170	FAD7	-1.743840781	8.93051E-16	-1.059665491	4.72658E-06	-0.959027159	9.5655E-15	0.322509578	0.731041294	0.039258379	0.935173532	0.559823803	0.009278405
AT2G40020	AT2G40020	-1.469965944	0.007602625	-1.067202895	0.015487749	-0.875518162	0.009958698	0.137024672	0.965260868	0.566708142	0.568034781	0.44442292	0.265894945
AT5G03040	iqd2	-1.473676718	3.13273E-07	-1.070300488	6.19293E-11	-0.37772037	0.019502573	-0.4075645	0.606948021	-0.505481269	0.001319427	-0.316297896	0.036606235
AT1G33610	AT1G33610	-1.583763753	5.40755E-05	-1.072009682	1.5962E-05	-0.975608502	0.009194004	0.270767398	0.936845219	-0.84353138	0.067981813	0.823662834	0.00054443
AT2G17530	AT2G17530	-1.050795059	0.002377978	-1.078596407	0.000341809	-0.357961485	0.247076595	-0.398639625	0.674766805	0.003883384	0.99559583	0.256378821	0.164401902
AT1G31420	FEI1	-1.023149851	1.72914E-05	-1.078721023	1.21408E-08	-0.893824374	1.58679E-05	-0.311733053	0.707962062	-0.172116951	0.515835758	0.015773243	0.95717911
AT2G19880	AT2G19880	-1.468823142	3.50653E-06	-1.080218432	0.003467613	-0.953643756	4.32711E-05	-0.852488754	0.584586994	-0.573243729	0.013140762	-0.263164917	0.1382503
AT2G47940	DEG2	-1.556536184	2.41261E-15	-1.081243552	7.73712E-06	-0.72029648	4.61516E-09	0.583077858	0.598901706	0.160366883	0.56041738	0.558702504	2.76076E-05
AT1G32060	PRK	-1.521755363	0.000284883	-1.08195081	9.38781E-12	-0.328508103	0.03323195	2.580032052	0.073310574	0.192810673	0.542580864	0.999944546	7.27275E-07
AT2G18890	AT2G18890	-1.128148894	0.004691472	-1.083544014	0.016167651	-0.319616888	0.233363506	-0.231261486	0.906874792	0.101308881	0.907642877	0.247363184	0.402980058
AT3G17470	CRSH	-1.154684386	0.000307219	-1.095525526	0.004816716	-0.460900165	0.073546655	0.607748124	0.659288623	0.382725745	0.511188955	0.61110895	0.011927587
AT3G43610	AT3G43610	-1.128023581	0.005041445	-1.095729155	0.001649071	-0.598725666	0.00477857	-0.327422622	0.712887303	0.029612421	0.963419708	0.507506296	0.000761551
AT1G51610	AT1G51610	-1.099385516	0.007332101	-1.098543481	8.9084E-05	-0.617661562	0.0255471	-0.229902688	0.844060401	-0.261747276	0.566059569	0.349989765	0.059975992
AT3G15570	AT3G15570	-1.669050094	1.18705E-05	-1.100760662	0.000254876	-0.293616316	0.174917333	0.593813914	0.783323192	0.038325804	0.982896351	0.089266718	0.835198075
AT1G09160	AT1G09160	-1.145938629	5.19073E-05	-1.103272605	0.002864076	-0.723820261	0.001214923	-0.18973708	0.905793887	-0.029934381	0.954260932	0.148648342	0.348215224
AT1G18340	AT1G18340	-1.115177313	0.046311885	-1.104036906	0.01908896	-0.408817523	0.278612322	0.15073715	0.8965227	-0.213262011	0.684725911	0.393081088	0.082022499
AT3G15360	TRX-M4	-1.753774664	1.40676E-11	-1.104294185	7.69624E-12	-0.951149422	1.97439E-23	0.923161332	0.48876871	0.251562794	0.480483712	0.102567668	0.657443093
AT4G25120	SRS2	-1.50539317	0.002139808	-1.106220092	0.022104261	-0.69620036	0.287428038	-0.229305374	0.915552048	-0.400082104	0.316265052	0.080656574	0.83883114
AT4G22540	ORP2A	-1.194163343	7.5769E-05	-1.107537285	1.28196E-11	-0.334867604	0.030103013	-0.081845932	0.93556951	-0.059817541	0.896222696	0.143576563	0.346532013
AT2G26900	BASS2	-1.241496905	1.29492E-08	-1.113574019	1.42077E-10	-0.930543531	4.32793E-10	0.205200027	0.794754875	-0.009857962	0.986774932	0.766063187	1.75596E-06
AT5G11880	AT5G11880	-1.204751094	2.1771E-05	-1.114862986	0.0003679	-0.836762949	3.38515E-07	-0.07934642	0.931898506	-0.217566692	0.368046063	0.362975019	0.038539057
AT4G01040	AT4G01040	-1.178572651	0.001585442	-1.120594347	9.41146E-07	-0.694782185	0.015752207	0.004841285	0.99779774	0.085006247	0.897458174	0.455726357	0.003453448
AT3G19680	AT3G19680	-1.618110472	1.24894E-05	-1.121499467	2.72304E-05	-0.911079222	0.002873567	0.239512154	0.905109619	-0.570331814	0.010714218	0.094477947	0.777262044
AT5G59250	AT5G59250	-1.531199173	4.15656E-06	-1.123369747	2.24503E-08	-0.577938306	0.00026365	1.556078095	0.422258087	0.284238552	0.683019892	0.562571455	0.004437129
AT1G69700	HVA22C	-1.333279515	0.014546168	-1.12521941	0.017874436	-0.823425236	0.002404943	0.387999129	0.677620385	0.725754223	0.000531541	0.929957184	3.91027E-07
AT3G53320	AT3G53320	-1.196917036	0.005161505	-1.127186767	0.043854946	-0.477094732	0.241493769	0.045895745	0.988471189	0.158955492	0.828385541	0.329154064	0.185851002
AT5G44740	POLH	-1.014151649	0.043029886	-1.127756953	0.003488748	-0.701745665	0.090040071	0.316640138	0.766274785	0.401764003	0.363657818	0.563050155	0.011634542
AT2G39140	SVR1	-1.841439762	7.38477E-07	-1.128706676	6.48241E-05	-0.521775249	0.015735353	-0.16250482	0.821455875	-0.120498257	0.863621528	0.294717354	0.096807574
AT5G35170	AT5G35170	-1.5954997	4.19237E-05	-1.13130167	4.32667E-09	-0.724855873	7.23094E-08	1.723893025	0.207276291	0.226164999	0.597179621	0.917538678	4.41025E-11
AT2G05210	AtPOT1a	-1.626289943	8.14995E-05	-1.131429151	0.000624526	-0.64275443	0.003803046	-0.08538715	0.958564896	0.302345989	0.447139821	0.72567187	6.43838E-05
AT3G14415	GOX2	-1.368471384	2.99573E-06	-1.131870103	6.18147E-19	-0.511963248	3.9442E-06	0.484756829	0.661123793	0.027828557	0.958669515	-0.196943775	0.133125141
AT5G04530	KCS19	-1.159086323	6.75604E-06	-1.132109596	0.004317723	0.142315711	0.462893126	-0.156864679	0.820789421	-0.199346051	0.571036133	0.154119868	0.294078126
AT1G14720	XTH28	-1.000739936	0.000772854	-1.133420413	1.57512E-06	-0.9842697	1.39714E-08	-0.079565163	0.95840698	-0.340798599	0.096474964	0.268079448	0.060541064

AT4G32260	PDE334	-1.447588272	7.07927E-06	-1.136996974	1.76305E-13	-0.654970349	1.32444E-11	1.086990006	0.484632088	0.191851462	0.520848536	0.516073069	0.000335336
AT2G22970	SCPL11	-1.278848991	0.001364255	-1.140778169	0.008904089	-0.402039988	0.123036289	-0.328990878	0.691824202	-0.522890748	0.035470898	-0.09618361	0.609661052
AT4G31850	PGR3	-1.304100183	8.80327E-05	-1.14694634	0.00320257	-0.424171072	0.024777881	0.28881455	0.828183742	0.159778512	0.861957219	0.049513091	0.846076347
AT1G54350	ABCD2	-1.429663906	3.71188E-05	-1.156141402	0.009629539	-0.769644332	0.026157533	0.635631973	0.653363031	0.22485634	0.659063827	0.91778138	6.13673E-05
AT4G26860	AT4G26860	-1.17062041	0.000302844	-1.159960676	7.8694E-07	-0.433240295	0.010062642	0.988985619	0.49571945	0.08040068	0.898600693	0.583662363	0.000447784
AT3G13690	AT3G13690	-1.269308717	5.37398E-05	-1.160402597	4.24429E-08	-0.972456566	3.91475E-08	0.283778406	0.692491609	0.003317964	0.996289463	-0.004105967	0.987491195
AT2G29690	ASA2	-1.429525326	0.0002306	-1.160763914	3.75308E-05	-0.617448222	0.012602449	-0.284320776	0.781016963	-0.252687505	0.515629348	0.110258552	0.691189766
AT5G10900	AT5G10900	-1.49057344	0.01139105	-1.163546583	0.036241307	-0.35830983	0.66520863	0.437285594	0.707962062	0.219682365	0.788611213	0.14136332	0.731011594
AT1G65190	AT1G65190	-1.666653831	0.018669013	-1.165589954	0.01984281	0.278767605	0.889794681	1.002612955	0.814118697	0.191687638	0.947746874	-0.763323593	0.575639295
AT3G14930	HEME1	-1.325443876	3.99666E-09	-1.175190337	7.79554E-08	-0.92056886	5.76066E-12	0.338064269	0.765335087	0.420726286	0.058461349	0.698342436	8.15572E-05
AT1G48610	AT1G48610	-1.366471637	0.007189483	-1.17824744	0.000574039	-0.287980311	0.416696868	-0.435692596	0.858742486	0.166070746	0.784214866	0.133694146	0.602354246
AT1G65470	FAS1	-1.705155713	0.001005899	-1.179113958	0.031368528	-0.658325733	0.196829672	-0.07422872	0.974201155	-0.094204866	0.869846959	-0.068945752	0.790914771
AT4G14360	AT4G14360	-1.317328558	2.69771E-06	-1.182396405	0.021568249	-0.749868028	5.11064E-05	-0.588647212	0.601921549	-0.484286311	0.070895289	-0.169989114	0.278773762
AT4G11900	AT4G11900	-1.808085861	0.002957753	-1.182792904	0.005607159	-3.442698025	0.535162474	0.5272894	0.88065104	0.527949088	0.871794848		
AT2G42870	PAR1	-1.247344501	0.000492443	-1.183031941	0.035431946	0.13503231	0.527432391	0.58598755	0.517374656	0.685067775	0.002739639	0.017176936	0.959022434
AT2G35155	AT2G35155	-1.682176018	0.000209299	-1.183414734	0.00340888	-0.776919602	0.006292737	0.199872658	0.859313107	0.077957513	0.90578753	0.297706515	0.372981655
AT5G03650	SBE2.2	-1.104376766	0.000373956	-1.1872643	3.3038E-10	-0.785612721	1.68217E-08	0.640632613	0.589531904	-0.113526208	0.792969578	0.253024006	0.140913981
AT5G07770	AT5G07770	-1.268809903	0.013983871	-1.190055431	0.002015287	-0.839929234	0.020199303	0.094378927	0.956852966	-0.597728	0.081630061	0.149290994	0.602217596
AT4G39400	BRI1	-1.133553314	1.31421E-06	-1.192602328	2.84406E-11	-0.716723771	7.56395E-08	-0.339278956	0.60160892	-0.169623787	0.586043627	-0.121623186	0.513027724
AT2G22670	IAA8	-1.119574375	0.001447081	-1.199353507	8.3799E-14	-0.971876556	1.80912E-20	-0.173849273	0.839580847	0.293719695	0.096958965	0.235919734	0.044688465
AT5G14210	AT5G14210	-1.139130363	0.000190656	-1.199507322	3.16085E-06	-0.908581031	6.96806E-07	0.093631244	0.952785336	0.201689673	0.616763356	0.595221518	0.004081876
AT5G09260	VPS20.2	-1.134529587	0.016841115	-1.206684419	0.008313506	-0.345941891	0.1615785	0.086695165	0.960320548	0.14045951	0.820469547	-0.098095331	0.689368074
AT1G48360	AT1G48360	-1.131885532	0.026172589	-1.211385599	0.000417124	0.197617812	0.70478638	0.361692481	0.665673432	0.470129967	0.337356272	0.269281892	0.192122272
AT1G52870	AT1G52870	-1.710119636	3.20432E-05	-1.212061279	5.84524E-07	-0.394877352	0.003647089	1.264857038	0.495602725	0.072878852	0.930422599	-0.334113299	0.226067983
AT4G05330	AGD13	-1.292356415	0.002558078	-1.215126297	9.80363E-05	-0.863176654	0.003219602	0.168808725	0.928888991	0.506611267	0.165947898	0.533531558	0.024046617
AT2G38120	AUX1	-1.140653339	2.06286E-05	-1.217544465	2.89657E-07	-0.194606045	0.416696868	-0.19738696	0.765335087	-0.315922817	0.056102446	-0.259567462	0.040823397
AT1G32540	LOL1	-1.262223468	2.77473E-05	-1.236285446	2.32395E-08	-0.636688737	4.12794E-05	0.008085939	0.996693402	-0.186280872	0.680666228	-0.330088024	0.124646754
AT5G10150	AT5G10150	-1.166031349	0.004103214	-1.239583394	4.80286E-10	-0.320279248	0.186216092	1.38754183	0.586846304	0.574623916	0.342476394	0.636661184	0.007114638
AT4G17260	AT4G17260	-1.007830254	0.046783875	-1.247053021	0.002481414	-0.842527751	0.018637683	-0.335300564	0.808237728	-0.401990451	0.490301085	0.325067277	0.124041514
AT2G25250	AT2G25250	-1.388456269	0.001443865	-1.2515615	1.14917E-05	-0.747935675	0.026828736	0.532942931	0.706203289	-0.87229433	1.4278E-06	0.625067527	0.000167502
AT1G14580	AT1G14580	-1.952438582	5.92548E-11	-1.251869682	0.001233718	-0.863998659	3.41007E-06	-0.258108353	0.825077044	-0.439465917	0.306003649	0.644570282	0.010169208
AT4G30950	FAD6	-1.845565105	8.15183E-23	-1.285130307	3.53249E-08	-0.969309746	9.26563E-18	0.568053058	0.45375294	0.332083216	0.041396188	0.7857896	1.54778E-08
AT2G42300	AT2G42300	-1.414763062	8.81059E-08	-1.289471227	2.79969E-05	-0.682793914	0.00059957	-0.074486252	0.953801756	-0.070286162	0.897477433	-0.102111739	0.61796844
AT1G80640	AT1G80640	-1.26453066	0.011117628	-1.293447865	0.001860038	-0.563293466	0.002536086	0.424962657	0.708641472	0.210931818	0.631435158	0.547805893	0.009401247
AT3G45260	AT3G45260	-1.611156907	3.35006E-05	-1.296320944	0.000225679	-0.263347567	0.486276183	1.100664772	0.164934579	-0.261043636	0.721412596	0.75526682	0.000277287
AT1G79060	AT1G79060	-1.393692535	0.01016764	-1.297709421	0.003433042	-0.53460794	0.264762361	-0.213179756	0.875558325	-0.118727442	0.861478038	0.506017026	0.003755245
AT5G65730	XTH6	-1.230173585	0.038609016	-1.298082609	7.49193E-11	-0.688269077	0.019075994	2.404530334	0.184430557	-0.295826753	0.767320273	0.150419321	0.707309909
AT4G36540	BEE2	-1.179750469	6.61162E-05	-1.298617355	2.96069E-06	-0.292297141	0.1447853	-0.161428202	0.871661081	-0.005182265	0.992519652	-0.068689296	0.697137175
AT1G68400	AT1G68400	-1.115545703	0.008449777	-1.300298906	0.000713732	-0.826837846	0.014356748	0.105053429	0.907100964	-0.242592976	0.582547704	0.014274285	0.953861513
AT2G01170	BAT1	-1.233898113	0.001467384	-1.300312755	0.000709367	-0.550510637	0.025479079	0.102546744	0.936758504	-0.094839208	0.878941396	-0.070022731	0.790058212
AT3G23530	AT3G23530	-1.334567983	9.5439E-07	-1.301631037	0.000139442	-0.906468196	6.28814E-07	0.203835646	0.835259894	0.317213246	0.373477183	0.760616156	1.70846E-08
AT5G06770	AT5G06770	-1.538122234	0.014742161	-1.304492909	0.009621073	-0.682601774	0.057526363	0.474702942	0.726900342	-0.07711884	0.929498792	0.064801332	0.869897802
AT5G47800	AT5G47800	-1.008739546	0.014114313	-1.304755802	0.001204449	-0.497629574	0.186630698	-0.305646214	0.838984335	-0.004966565	0.997309584	0.201886196	0.583261004
AT3G61580	SLD1	-1.345563507	2.23324E-11	-1.306373306	6.21386E-21	-0.920401705	2.37192E-20	-0.098213969	0.917097586	0.009895445	0.983764323	0.208521296	0.332641753
AT4G10000	AT4G10000	-1.328853402	0.017320528	-1.31020642	0.002211112	-0.679583371	0.039069705	0.943784319	0.646729152	0.450669835	0.535087539	0.104922269	0.761927143
AT4G03110	RBP-DR1	-1.644905974	5.18364E-06	-1.310914782	2.76223E-10	-0.574079452	0.070131851	0.170654524	0.871661081	0.118108383	0.835618895	0.238188063	0.162633967
AT1G67900	AT1G67900	-1.450613962	7.83431E-05	-1.31396897	2.68148E-16	-0.636332381	1.04105E-05	1.130793784	0.562656211	-0.024035959	0.979199677	-0.541980241	0.000363894
AT3G63370	OTP86	-1.455651817	0.04189038	-1.314760631	0.030993478	-0.437151704	0.292292797	0.807881557	0.764510534	-0.029763733	0.985120284	0.00885664	0.985093313
AT1G27210	AT1G27210	-1.349063316	2.14537E-05	-1.316789108	0.000668173	-0.267793527	0.311118368	-0.002826493	0.998026502	0.119850819	0.700183076	0.947566968	2.69815E-11
AT1G31160	HINT 2	-2.083783636	5.67775E-07	-1.318669883	0.000113893	-0.51869091	0.177286734	0.293439781	0.73148555	0.147228579	0.841586178	0.621386572	0.000142417
AT5G24290	MEB2	-1.102020502	0.036502912	-1.327898931	0.008837253	-1.039404863	0.13455322	-1.011341854	0.523707451	-0.293045073	0.537471928	-0.82506139	0.001271741
AT5G56890	AT5G56890	-1.570047505	0.00016277	-1.332877206	1.20851E-08	-0.879532958	0.000306377	-0.615138683	0.632278424	-0.538608993	0.023493808	-0.013135644	0.96739024
AT5G23860	TUB8	-1.225074959	0.000118523	-1.335254886	7.55297E-05	-0.888026733	2.01112E-08	-0.058356928	0.956216588	-0.06611139	0.876276652	0.384117112	0.032996802
AT2G39870	AT2G39870	-1.078455766	0.000247948	-1.335817501	4.47351E-13	-0.014494074	0.9733794	0.054077891	0.964169173	-0.041804247	0.924868537	0.33455839	0.061216706
AT1G14840	MAP70-4	-1.055582786	0.019569562	-1.340180302	2.19329E-06	-0.337936253	0.078188928	-0.225793158	0.856756323	0.072184414	0.885435975	0.476960374	0.000700403
AT2G44230	AT2G44230	-2.246509114	1.43196E-06	-1.344749262	2.4304E-06	-0.610983232	0.052305448	-0.146241979	0.928725719	-0.459658239	0.263502113	-0.468078404	0.010021703
AT5G16540	ZFN3	-1.473040319	0.000177444	-1.356651559	3.16725E-10	-0.065572019	0.866988322	1.253794885	0.386146892	-0.211069971	0.804862892	0.794439046	3.87303E-05
AT1G33470	AT1G33470	-1.050493369	0.007021643	-1.358160284	1.15322E-05	-0.527443631	0.013077373	-0.142697706	0.950313494	-0.066847008	0.8599648	0.266248862	0.187196908
AT4G27230	HTA2	-1.168313057	0.00026541	-1.384759411	0.000212467	-0.721558349	0.005975157	-0.180760628	0.877496967	0.093565556	0.813646907	0.252939126	0.164994868
AT1G21730	AT1G21730	-1.005557764	0.000406482	-1.388395056	1.35054E-08	-0.297876602	0.430200185	-0.048341197	0.972495118	-0.062315693	0.897458174	-0.063518464	0.763113464
AT2G30575	LGT5	-2.258802806	9.13275E-06	-1.388611656	0.000601699	-0.636397655	0.102327797	0.087364595	0.968467209	-0.050538307	0.956608575	0.135441851	0.644040554
AT1G33240	GTL1	-1.144002873	4.05582E-05	-1.388965302	3.67604E-11	-0.704422985	1.0736E-05	-0.113228197	0.926124658	-0.252429256	0.353432914	-0.626084453	3.88637E-06
AT5G42100	BG_PPAP	-1.425261835	0.000175146	-1.392834379	1.42638E-14	-0.854893888	0.000129208	-0.447612672	0.616300683	-0.501829276	0.019262498	0.225183252	0.343850552
AT5G14060	CARAB-AK-LYS	-1.691401389	4.75658E-09	-1.412216376	4.19197E-17	-0.768035713	1.25538E-05	0.463729594	0.470503933	0.233644482	0.448596009	-0.008952842	0.969973433
AT4G02900	AT4G02900	-2.042351685	2.30882E-05	-1.419698413	0.000156601	-0.193392318	0.568775605	0.093427164	0.966811844	0.031992752	0.97926944	0.741578366	0.001384404
AT5G26270	AT5G26270	-1.23719346	4.07578E-05	-1.426826336	1.71793E-06			0.133551163	0.944029748	-0.7934092	0.006737401		
AT3G15030	TCP4	-1.186157611	0.028851976	-1.435594895	0.003717361	-0.818713683	0.001905653	-0.33806943	0.640445007	-0.144257515	0.768261019	0.202356876	0.489426303
AT5G66005	AT5G66005	-1.741527368	0.002240757	-1.436136306	0.032030931	-1.101847404	0.058862476	0.295536911	0.855081587	0.621877067	0.354547118	0.726889508	0.000941772
AT3G55950	CCR3	-1.860763554	0.02823917	-1.439093824	0.022998925	-2.903874477	0.14910162	-0.934275186	0.619569853	-0.490896451	0.599487284	0.170344833	0.6693427
AT5G09315	None	-1.478956501	0.035636196	-1.439221675	0.036056087			-0.578798945	0.721140128	0.083593625	0.957639075		
AT3G53310	AT3G53310	-2.098237391	0.001263743	-1.440474108	0.003888208	-3.184964406	0.114377005	0.726743233	0.644839236	0.284516509	0.8182882	0.013624951	0.97989514
AT1G05805	AT1G05805	-1.08688585	0.000106092	-1.445528043	4.32859E-10	-0.80125943	0.005252968	0.908416167	0.279591669	-0.060095408	0.912702486	0.199409517	0.261221637
AT5G10620	AT5G10620	-1.625586582	0.01130915	-1.451413618	0.035132991	-0.801127699	0.187318778	0.190429061	0.980732812	0.571368033	0.691313385	0.867633001	0.093757375
AT3G59780	AT3G59780	-1.835922194	1.5247E-09	-1.453130601	6.20563E-21	-0.97580707	1.38092E-11	1.146947497	0.430181703	0.456733738	0.029374753	0.646435062	0.001474712
AT1G76110	AT1G76110	-1.637658914	0.001894879	-1.458366408	0.000126659	-0.418329519	0.185143779	2.077014751	0.370740012	-0.082080144	0.971700698	-0.376013454	0.299209529
AT1G26130	AT1G26130	-1.335476173	0.048585642	-1.45849573	0.002069811	-0.321748315	0.496027339	-0.173955896	0.932578056	0.416600951	0.491938784	-0.16627487	0.582923936
AT5G62410	SMC2	-1.52730612	0.015585338	-1.462362045	0.000569499	-0.563835472	0.168325764	-0.1030104	0.953493824	-0.103568426	0.879603903	-0.066658284	0.769565959
AT1G67400	AT1G67400	-1.502175161	0.000983011	-1.462776733	0.002142074	-0.83411668	0.005134241	0.724543601	0.678599943	0.033705209	0.983751188	0.693021864	0.016201788
AT2G48030	AT2G48030	-1.056296811	0.005282564	-1.466866185	1.68261E-05	-0.861420989	0.115315861	-0.136476892	0.90505206	-0.424750167	0.24518545	-0.606958544	0.007586797
AT4G20870	FAH2	-1.008390584	0.000424404	-1.470789039	1.13882E-13	-0.49675824	0.047134138	-0.155285578	0.895706838	-0.076485158	0.868188276	0.172910318	0.470539394
AT2G20250	AT2G20250	-1.898821011	0.027279973	-1.47097801	0.008511015	0.322174271	0.418190798	1.494039886	0.619354152	0.666665943	0.638324999	0.803914866	0.110079625
AT2G34510	AT2G34510	-1.886397363	1.59702E-06	-1.476304226	8.70167E-15	-0.570013823	0.008440368	0.41515321	0.769724647	-0.34800619	0.209073558	0.181203926	0.581209617
AT1G28670	ARAB-1	-1.461923466	0.003420466	-1.476234901	0.030566184	-2.039877314	0.111350564	-0.311911784	0.877411995	-0.228908352	0.348335571	0.746257061	0.002640459
AT5G38520	AT5G38520	-1.939798047	2.38466E-10	-1.477122988	4.41316E-12	-0.868445098	1.84612E-08	0.806839712	0.554617999	0.305302369	0.381380382	0.39211477	0.030868569
AT2G25100	AT2G25100	-1.102799343	0.011348769	-1.486756319	0.006003766	-0.615978078	0.074628216	0.34632931	0.712887303	0.515600371	0.377885595	0.534715154	0.01914316
AT1G49730	AT1G49730	-1.391235236	1.05221E-08	-1.488898687	1.29879E-10	-0.760503086	4.24662E-06	0.213834518	0.831849152	0.19856233	0.575903861	0.541011253	0.004966907
AT5G28770	BZO2H3	-1.224997322	7.20524E-05	-1.494929111	3.43453E-14	-0.6086343	9.9171E-05	0.572185955	0.459472858	0.319306402	0.185949923	0.600153952	0.000161089
AT2G02070	IDD5	-1.184797492	0.000246104	-1.501256732	5.02777E-10	-0.408764588	0.071430598	0.280041717	0.721771703	0.161611444	0.606718088	0.718824478	2.08889E-05
AT3G22780	TSO1	-1.176074906	0.01061685	-1.503934236	1.18304E-09	-0.852797797	0.00025845	0.003634638	0.997835552	-0.204533112	0.563142427	0.078488773	0.756224353
AT1G45180	AT1G45180	-1.361606417	0.000365043	-1.512556538	0.000908344	-0.854942606	0.00249094	-0.711693752	0.66524984	0.563842252	0.233539745	-0.037069063	0.912115778
AT2G43360	BIO2	-1.189569897	8.24653E-05	-1.514470354	3.09574E-08	-0.74143174	0.000551708	0.108090927	0.904735573	-0.017040137	0.979081111	0.31890611	0.1078334
AT2G33850	AT2G33850	-1.706519429	0.016331592	-1.515088885	0.000651817	-0.492140876	0.792350154	0.538594138	0.718595611	-0.169685616	0.924868537	0.266184956	0.644577682
AT5G12950	AT5G12950	-1.154219813	0.00014647	-1.519419598	3.22594E-06	-0.442301329	0.046056266	0.210342006	0.867277077	-0.488584345	0.072595425	0.432540909	0.003633457
AT5G49030	OVA2	-1.481062626	9.85132E-05	-1.523576673	4.53692E-08	-0.980049354	5.04117E-08	-0.191499077	0.824328526	-0.071719182	0.867744589	0.240481402	0.12432351

AT1G09440	AT1G09440	-2.567067638	0.001175813	-1.52647438	0.012730285	-0.710802652	0.227277754	0.710261616	0.680482607	-0.195776151	0.884988464	0.972649027	0.000769305
AT1G31580	ECS1	-3.676085929	1.5536E-09	-1.528279414	7.78251E-06	0.451414385	0.908984144	3.745182908	0.076422125	3.376379983	0.500891025	1.993245803	0.355089129
AT2G31070	TCP10	-1.386259802	8.78979E-07	-1.529859867	0.009076535	-0.899080636	0.00049981	0.275152815	0.795199419	0.282324073	0.502091749	-0.125350966	0.728527329
AT2G06850	XTH4	-1.373361384	2.40403E-09	-1.536556339	1.07285E-08	-0.629350914	7.75703E-07	-0.633792936	0.494852954	-0.7042169	4.97923E-06	0.583941109	5.10203E-05
AT2G23600	ACL	-1.369671515	1.60476E-09	-1.536709641	3.27001E-12	-0.898932281	0.000882267	0.253418068	0.808237728	0.005270074	0.99453777	0.147140365	0.488377499
AT2G31010	AT2G31010	-1.174633042	0.014106321	-1.539341604	2.84419E-05	-0.440124633	0.087711221	0.283985175	0.820406788	0.1010315	0.896222696	0.874412658	5.122E-08
AT4G16860	RPP4	-1.749022229	0.000134105	-1.542491746	1.2491E-05	-0.185707223	0.661568473	2.970384863	0.071069389	0.856344321	0.364405615	-0.069459212	0.87295679
AT1G72930	TIR	-1.513994128	0.048596902	-1.545079814	0.003231026			2.029452682	NA	-1.460295991	0.262547221		
AT1G73570	AT1G73570	-2.810726821	1.62474E-06	-1.546895108	0.004424443	-1.108148366	0.064152749	-0.124663484	0.975645787	0.02258366	0.988079805	-0.120178096	0.714909394
AT2G20680	MAN2	-1.776588638	0.01023057	-1.55197061	0.009317474	-0.758817735	0.017693804	-0.698613811	0.585524552	0.201351305	0.7207124	0.408862297	0.027582536
AT4G36550	AT4G36550	-1.316881388	0.000354964	-1.565690126	6.72082E-05	-0.556525875	0.085809956	0.005709532	0.997796217	-0.183793013	0.768565149	0.661914153	0.004026176
AT3G56160	AT3G56160	-2.012555951	6.62895E-07	-1.566776317	7.91778E-06	-0.376279338	0.046596701	0.188277235	0.824658442	0.717490374	0.006249521	0.915093108	6.83472E-11
AT1G48600	PMEAMT	-1.436048731	0.023924696	-1.575801228	5.54011E-05			-0.507498923	0.73148555	-0.191989471	0.475390745	2.036995974	0.761927143
AT3G46200	NUDT9	-2.022220761	0.00926375	-1.582551846	0.005520905	-0.812501962	0.091516126	-0.414783698	0.805751383	0.299923928	0.694731487	0.8443307	0.000117231
AT5G64630	FAS2	-1.802152719	0.014977441	-1.586175463	0.020244521	-0.848514382	0.035310266	-0.064106814	0.982323936	0.006774292	0.994809352	0.508048252	0.010169208
AT1G69530	EXPA1	-1.201920887	0.002281826	-1.588619464	8.1839E-10	-0.526291061	0.078213744	-0.580449871	0.650183354	0.02010714	0.974623822	-0.320535	0.022400246
AT3G61310	AT3G61310	-1.374649404	0.005060152	-1.590015169	1.65516E-05	-0.857668785	0.009728941	-0.054627782	0.979321443	0.066431161	0.93404597	0.219589774	0.385346864
AT4G02130	GATL6	-1.04633892	0.034675929	-1.590815305	0.000113078	0.034411695	0.939872684	0.164230954	0.93403749	0.234182295	0.575644885	0.67204257	0.007854607
AT2G07680	ABCC13	-1.525295208	0.000105152	-1.590821679	0.009778305	-0.237666437	0.418165035	0.133902053	0.937079728	-0.008369006	0.995618796	0.45666502	0.015602438
AT2G35650	CSLA07	-1.342672241	8.50383E-06	-1.605980274	2.60783E-12	-0.934071843	0.000187151	0.434465027	0.627163215	0.289279778	0.466319315	0.709555548	1.9728E-05
AT1G32730	AT1G32730	-1.454593861	0.002319318	-1.608122584	0.001792195	-0.896932527	0.057590994	-0.380440209	0.904735573	0.482887879	0.496686298	0.167029948	0.633127872
AT1G18360	AT1G18360	-1.191153532	0.036482608	-1.61766371	3.93239E-07	-0.936940144	0.000151805	-1.05285619	0.477936046	-0.567771135	0.298603419	0.552289082	0.005218315
AT3G50110	PEN3	-2.230083206	0.000691569	-1.620711269	0.026978862	-1.204862923	0.125693632	-0.681171964	0.901065817	0.024191959	0.988123438	0.201547491	0.556485777
AT2G29890	VLN1	-1.130023886	0.045292679	-1.629540021	5.10851E-06	-0.192709428	0.647158166	0.149385298	0.932578056	0.178616126	0.737309895	0.880459163	4.85382E-08
AT2G37450	UMAMIT13	-1.857399308	5.18677E-05	-1.631229777	0.000345148	-0.648129118	0.002182674	0.657781456	0.563758673	0.06178201	0.941582568	0.705092304	3.73287E-05
AT1G79460	GA2	-1.143665595	0.01886452	-1.635801815	9.33177E-05	0.228768947	0.549685289	-0.182714978	0.932705464	0.114481348	0.855385068	0.047509957	0.851655433
AT2G32010	CVL1	-1.935338918	3.01664E-08	-1.636817755	6.43941E-06	-0.972836906	1.43568E-07	-0.187766524	0.885844797	0.080395317	0.924627441	0.357496415	0.123223222
AT3G54920	PMR6	-1.192464909	0.003146684	-1.642745535	5.41597E-07	-0.483368406	0.007646371	-0.155785697	0.932705464	0.269634607	0.12182076	0.669499037	1.02426E-08
AT3G59110	AT3G59110	-1.135610381	1.41074E-05	-1.675164229	1.67197E-13	-0.934219879	3.3511E-09	0.294716765	0.713846558	0.019903587	0.975427751	0.032597649	0.905095413
AT2G38695	AT2G38695	-1.510626685	0.012402409	-1.678081155	0.002260038	-0.602418754	0.132800578	0.022108292	0.995985035	0.086174152	0.950071203	0.365808706	0.325530522
AT3G53760	GCP4	-1.387094294	0.013757323	-1.680471131	0.002244348	-0.588940954	0.299045176	-0.067967154	0.982749202	0.032274009	0.970360188	0.472513881	0.015191692
AT5G05820	AT5G05820	-1.404935895	0.001876921	-1.694876801	0.000474625	-0.8367127	0.00165383	-0.378137304	0.731041294	-0.18062117	0.733142657	-0.265952092	0.398239933
AT1G69900	AT1G69900	-1.403555076	0.007351552	-1.70112328	0.007908238	-0.940934906	0.008216319	0.398395931	0.820789421	-0.49015814	0.642559119	-0.957704979	0.002445677
AT3G13000	AT3G13000	-1.650551326	4.4231E-08	-1.702172446	1.46398E-08	-0.953920893	6.16491E-05	0.117007215	0.948923938	0.380833063	0.399531927	0.537566602	0.000582887
AT4G25830	AT4G25830	-1.662906677	0.000217716	-1.708477975	0.002917969	-0.680565268	0.032632187	-0.618531417	0.664635521	-0.226497062	0.628005681	0.226243657	0.427237867
AT5G45560	AT5G45560	-2.006735405	0.000922448	-1.715838636	0.007499501	-0.919063034	0.025786013	-0.534595781	0.88065104	0.280487535	0.616572022	0.413837975	0.06289521
AT5G36940	CAT3	-1.001347854	0.022233105	-1.718822491	8.55631E-09	-0.722266275	0.002174087	-0.302868437	0.76611969	0.058737278	0.951101524	-0.632442765	0.006589081
AT5G54630	AT5G54630	-1.409126404	6.38691E-05	-1.741201106	1.48114E-07	-0.848975124	9.40777E-05	0.476141092	0.610227484	0.341303003	0.343748902	0.402331288	0.039519699
AT2G21080	AT2G21080	-1.420705203	0.049835301	-1.753070011	1.90356E-05	-1.752596439	0.445204477	0.574894675	0.72835818	0.145098776	0.869033869	0.749427397	0.714401361
AT1G67630	POLA2	-1.303598521	0.022712402	-1.758357958	8.31394E-06	-0.418230422	0.297939728	0.175891204	0.950313494	0.065982449	0.934226173	0.145247764	0.632185318
AT2G34600	JAZ7	-2.945009762	0.000168846	-1.759399382	0.001642117	-0.719336926	0.226794846	0.190549563	0.972205981	1.078490778	0.550143794	0.789816391	0.354419853
AT4G14560	IAA1	-1.781918542	0.000147483	-1.773725041	1.1528E-08	-0.936203651	2.34081E-07	0.473104093	0.7594526	-0.326081505	0.516852689	0.399966034	0.066859394
AT3G52290	IQD3	-1.829590371	0.000426326	-1.77671471	0.004321199	-0.74676991	0.120731264	-0.579781181	0.667958595	-0.177384504	0.76508716	0.624183641	0.003039401
AT1G22490	AT1G22490	-1.451282292	0.013699403	-1.796711513	0.001727063	0.074756939	0.880383976	1.648830918	0.479937209	-0.402310072	0.842628389	0.651056018	0.211375413
AT1G18560	AT1G18560	-1.229908496	0.027368625	-1.804597163	0.017038563	-0.10673947	0.872762878	-0.044653753	0.990915497	-0.436495226	0.527510672	0.052271896	0.896065922
AT2G21340	AT2G21340	-1.828665722	2.71211E-07	-1.813842738	3.27952E-08	-0.719477124	7.18392E-06	-0.134362146	0.917312631	0.070748002	0.926264746	0.038573826	0.887135533
AT4G08950	EXO	-1.637067376	2.35999E-05	-1.815891948	0.011593683	-0.976780268	0.311786997	0.437962999	0.622671196	-0.403771521	0.063641582	0.698367013	0.403759919
AT4G32980	ATH1	-1.639170315	0.014443854	-1.816636567	5.41597E-07	-0.980766375	0.000244575	0.457310733	0.714583753	0.252681873	0.700183076	0.806047969	1.50135E-06
AT3G52840	BGAL2	-1.407768262	1.20708E-06	-1.83297642	1.367E-16	-0.935745388	1.17142E-06	0.280745223	0.704225272	-0.201416528	0.562716873	0.178619197	0.232434649
AT4G17810	AT4G17810	-2.120424891	1.20126E-06	-1.843802393	0.000197139	-0.599637385	0.02702868	0.338322532	0.883697487	-0.183149247	0.872795006	-0.558291417	0.04003514
AT5G23060	CaS	-3.053023194	2.85447E-26	-1.868141146	4.55553E-23	-0.973731876	4.2801E-05	0.25745378	0.804372175	0.281993952	0.823358742	0.784728836	9.13034E-07
AT3G02920	RPA32B	-2.176383558	0.0294624	-1.880342523	0.015206687	-0.503565713	0.305658652	-0.297648868	0.886424693	-0.483768696	0.369984812	0.337341765	0.196807381
AT5G01630	BRCA2B	-2.006342912	0.000155605	-1.88682337	0.009796296	-0.397434019	0.361980874	0.098774965	0.97809523	0.055468452	0.959289777	0.62690167	0.005508644
AT3G21550	DMP2	-1.663422924	0.034183565	-1.906214483	0.005076501	0.381743187	0.413317108	-0.824953781	0.652047062	-0.550390892	0.2553851	-0.719066455	0.001312057
AT3G16700	AT3G16700	-2.721687994	2.36915E-06	-1.916751323	0.020992502	-1.337339053	0.06278662	-0.76083649	0.67307941	0.013821476	0.992611192	0.310252294	0.348207296
AT1G02335	GL22	-1.566761742	0.015973428	-1.966244804	0.017067027	-0.978400245	0.15054111	-0.340475763	0.916964723	-0.715957341	0.081630061	-0.550448605	0.035261618
AT5G49740	FRO7	-2.604962129	1.22722E-05	-1.980263663	7.74032E-06	-0.658022429	0.002887573	1.517061612	0.649356026	-1.611592946	0.227955565	-0.836192143	0.027656093
AT4G14150	PAKRP1	-1.831436301	0.048542208	-2.020101978	0.01644588	-1.112410003	0.659564394	-0.776213253	0.795199419	0.372328028	0.708363406	0.647374837	0.000802648
AT5G26280	AT5G26280	-1.256605404	6.21227E-09	-2.032409343	6.20599E-10	-0.742001112	0.000339195	-0.483809072	0.481581974	-0.914562432	6.20601E-05	-0.899756591	1.86105E-09
AT3G23730	XTH16	-1.214743655	0.02623786	-2.039731759	4.51652E-05	-0.970179749	0.012421352	-0.30324416	0.757543455	-0.374526226	0.295617122	-0.314004053	0.636985214
AT5G67100	ICU2	-1.760007715	0.008810618	-2.058798534	0.009235574	-0.874682497	0.009345117	-0.181830911	0.956315558	-0.031736581	0.97361709	0.243208999	0.269331769
AT5G46240	KAT1	-3.29366368	5.46834E-17	-2.070153818	1.859E-11	-0.967997204	0.008719335	0.208752204	0.923622633	0.486693228	0.494073375	0.343702121	0.426074177
AT5G06930	AT5G06930	-1.635473779	0.005109587	-2.070872526	1.57209E-10	-0.926681885	0.028168978	-0.460522838	0.65378996	-0.409001657	0.258496511	-0.230956704	0.288281617
AT2G27770	AT2G27770	-1.171672926	0.032579032	-2.087382327	5.16046E-07	-0.969213099	0.003257766	0.117118768	0.93623088	0.106728054	0.913724709	-0.489721492	0.052168387
AT2G18300	HBI1	-1.469915882	3.72892E-07	-2.099871532	2.69573E-12	-0.75129992	7.55178E-05	0.416542329	0.657854795	-0.189565683	0.425195266	0.119002254	0.490934846
AT2G29980	FAD3	-1.818395221	0.001285055	-2.130952815	6.66113E-16	-0.991802927	0.146761702	-0.384299406	0.849119707	-0.185355707	0.519240109	0.230321087	0.107110653
AT3G06750	AT3G06750	-1.817680452	4.38623E-06	-2.142509895	3.01833E-17	-0.90717406	2.18418E-05	-0.070440546	0.965260868	-0.293416683	0.556330533	-0.103744676	0.765456877
AT1G50890	AT1G50890	-2.346911663	5.083E-06	-2.150109136	0.00233268	-0.878489327	0.038889661	0.121772591	0.964619407	-0.037373036	0.983720308	0.533887131	0.14646834
AT5G44130	FLA13	-1.547889028	1.09407E-06	-2.150458255	0.033619739	-0.483567662	0.100436996	-0.244324623	0.842936823	-0.892943058	0.001974719	-0.188349422	0.485566389
AT4G18780	IRX1	-1.942671949	0.000977378	-2.169573414	0.001099297	-0.887516219	0.148562059	-0.529795012	0.718989301	-0.899327576	0.028557616	-0.398283755	0.158356202
AT5G45650	AT5G45650	-2.239672099	0.0097857	-2.174621771	0.012527471	-0.899518534	0.290470633	-1.176744331	0.664635521	-0.437418104	0.684689832	0.981664391	0.004559492
AT3G05140	RBK2	-2.878379937	3.61268E-06	-2.200940822	0.007511956	0.052682416	0.965724222	0.02791095	0.990924696	0.281729829	0.824058587	-0.69612439	0.441743596
AT2G31650	ATX1	-1.504964629	0.004005065	-2.209025697	0.000487115	-0.611525249	0.087217974	-0.04811724	0.982734229	0.020636542	0.986774932	-0.012635379	0.969252469
AT1G61300	AT1G61300	-3.32153809	3.9847E-09	-2.210697837	0.001874682	-0.890575827	0.005920187	1.632361297	NA	-2.043097864	0.676008799	0.468818502	0.018229122
AT2G37430	ZAT11	-2.015908312	0.021715836	-2.21291087	0.026568903	0.968605775	0.610823786	-3.070760687	0.61026714	0.026695187	0.994163341	1.063553762	0.09386099
AT4G11100	AT4G11100	-1.888218752	9.86633E-05	-2.2552911	0.009920637	-0.934738856	1.49482E-06	-0.209764476	0.957721682	0.761031796	0.037059656	0.784359414	5.4351E-05
AT3G54930	AT3G54930	-2.290848569	0.006411227	-2.257235495	0.006227764	-0.916652137	0.091917422	0.408047171	0.856750536	0.349162511	0.699161654	0.815982699	0.003818493
AT5G66770	AT5G66770	-1.868286692	0.009876491	-2.348431481	6.135E-08	-0.299332933	0.321097387	-0.493055208	0.761894565	-0.186817267	0.744330774	-0.154387026	0.531612971
AT5G03355	None	-2.521593532	1.9017E-07	-2.378493061	2.1446E-06			-0.093951755	0.958708047	-0.46089719	0.436175425		
AT5G44400	AT5G44400	-3.033312631	0.03589308	-2.395814097	9.86278E-06	-1.315742433	0.134715397	0.152104508	0.966082349	-0.599155131	0.195152749	0.112502297	0.828777655
AT1G65800	RK2	-4.794301337	3.2126E-06	-2.450925412	0.000320796	-1.535852954	0.063460539	2.58720064	0.691895802	2.249172321	0.691313385	0.990070186	0.462364542
AT5G16190	CSLA11	-2.183689624	0.034371974	-2.464351845	0.000487507	-0.939864157	0.668657337	-0.258068085	0.97071238	-0.106189362	0.947746874	0.533096854	0.253327214
AT1G01225	AT1G01225	-1.854486386	0.004318658	-2.473388745	1.95236E-07	-1.351513378	0.082128157	-0.066329241	0.975645787	0.154124395	0.870101981	0.293523879	0.386858054
AT4G16140	AT4G16140	-1.663518038	0.010660656	-2.496391674	1.3196E-09	-1.379467218	0.093370002	-0.467405145	0.782971455	0.054533176	0.937520456	0.846882146	0.000465972
AT2G29680	CDC6	-2.866908442	0.000292765	-2.515681228	0.005687582	-0.888352927	0.042773351	0.089586898	0.990938961	-0.302462819	0.766394043	0.22191796	0.519969668
AT3G15390	SDE5	-2.0342846	0.003677773	-2.522849456	0.00520626	-0.442833662	0.587487997	-0.546242156	0.77590923	0.081517123	0.949384996	0.379978832	0.149997008
AT4G19820	AT4G19820	-2.031023481	0.000502359	-2.546352352	5.02043E-05	-1.149082257	0.153496556	3.923610815	0.266366767	-0.853372547	0.819132051	1.356912403	0.114138026
AT1G02640	BXL2	-2.244936398	3.7206E-07	-2.599022702	5.29712E-12	-0.289450295	0.138753919	-0.536877405	0.55369123	-0.10515053	0.786582627	0.488355554	2.10995E-05
AT5G44635	MCM6	-1.693466642	0.025343683	-2.770920135	2.10413E-05	-0.971726437	0.022651025	0.206549188	0.963509062	-0.050760414	0.955714995	-0.071170346	0.814315114
AT5G41080	GDPD2	-1.864559821	0.000266085	-2.962703732	0.000681232	0.592177163	0.011025912	1.79266983	0.446425625	-0.399603953	0.554224402	0.777934576	0.00281838
AT3G42800	AT3G42800	-1.581531411	0.012139417	-3.001667081	0.036275466	-0.53677862	0.434576566	0.438605023	0.806588363	-0.873841161	0.118355095	-0.138296422	0.753986354
AT5G03350	AT5G03350	-3.207819894	1.0072E-06	-3.006048207	1.54132E-06	-1.636113196	0.493270455	-0.01175978	0.997790351	0.349363363	0.647824775	1.869277236	0.086616314
AT3G56300	AT3G56300	-2.166207405	0.010345685	-3.221853659	0.01283283	-1.446389285	0.079985815	-0.557838676	0.930502911	-0.124387314	0.944446213	0.864871014	0.003516106
AT4G13575	AT4G13575	-4.884722216	2.08277E-12	-3.619111651	5.36849E-22	-0.175052106	0.731195446	3.790208939	0.268371614	-6.64605887	0.224073613	-1.036005103	0.265946987

AT4G29740	CKX4	-2.311370138	0.00099329	-3.743557552	0.002426264	0.44933173	0.592206583	-2.95753434	0.343493265	-0.821187189	0.683421984	-0.653809266	0.286750465
AT1G78450	AT1G78450	-3.675723396	0.007227647	-3.825287918	0.038224486	-0.898583844	0.301071494	1.798036293	0.869849969	1.320542647	NA	1.130675559	0.767696123
AT4G16880	AT4G16880	-5.135251304	7.95616E-08	-4.028670124	0.00064224			-4.347100853	0.702246294	0.214618966	0.986818647	-5.206295806	0.281492188
AT5G07800	AT5G07800	-2.196380431	0.011990796	-4.032121068	0.028305846	-0.781723277	0.4589589	-0.271362948	0.908636304	-0.374554072	0.625207384	-0.143843229	0.670052825
AT1G66100	AT1G66100	-5.560399345	0.004614244	-4.104641608	0.000211444	6.203044767	0.17483003	5.571909122	0.637815859			-1.07478505	0.840361736
AT5G18020	SAUR20	-1.830480759	0.038135313	-4.176909208	0.024240675	-2.352181343	0.054540212	-0.682047698	0.903381971	-0.427850941	0.773564408	-0.451339282	0.655577041
AT3G46370	AT3G46370	-9.181569362	4.33763E-07	-4.407839028	0.019730543	0.64253239	0.124226029			-0.851356915	0.938900761	-1.436583526	0.677050764
AT4G09530	AT4G09530	-7.87278841	0.023811896	-6.570823337	0.045486566	-4.087945298	0.453957696	-0.884205995	0.962819636	-2.414740633	0.65641871	-1.083590841	0.669990446
AT5G51470	AT5G51470	-6.015505198	0.003329266	-6.84204822	0.025767389	-2.342107295	0.548896798	0.96384167	0.957738363	0.003477624	0.999843118	-2.202582188	0.201659274
AT2G01870	AT2G01870	-4.882274867	0.02494135	-6.963222364	0.022155581	-0.869076504	0.113869066	-0.138932499	0.993804788	-1.350490669	0.824002281	1.055377867	0.09771885
AT1G64260	AT1G64260	-5.990209028	0.011069652	-7.101432048	0.015520079	-0.199086857	0.551262982	0.853716857	0.770868476	-0.17612881	0.950034363	0.126412341	0.553659062
AT1G71760	AT1G71760	-7.48224678	0.005834911	-7.242388184	0.006870159	-0.853598149	0.719722303	-0.465257833	0.966082349	0.610337168	0.880643463	0.786792755	0.208618666
AT3G10780	AT3G10780	-7.426757669	0.04856962	-7.246108002	0.007149385	0.67646785	0.246114423	5.016277172	0.429222482	-6.116572787	0.27856344	0.200202132	0.867117452
AT5G66710	AT5G66710	-6.192543546	0.004585757	-7.262044351	0.006815996	-1.956125475	0.500483869	-0.100746845	0.993804788	-1.335250987	0.723835701	1.693716736	0.105709141
AT5G07570	AT5G07570	-8.362036142	8.2212E-05	-7.700488102	0.001129624	-1.296187969	0.743855924	-1.67212412	0.798808149	-0.381816659	0.899954945	-0.129926765	0.912330402
AT4G19970	AT4G19970	-7.817980356	0.002110858	-7.916293896	0.000966957	0.477687172	0.853656332	-0.867953298	0.895706838	1.250214869	0.58538838	-0.629478689	0.538342261
AT2G46760	GulLO6	-4.775549175	0.001192308	-8.870140685	1.22432E-06	-1.161710807	0.558282275	-0.794101224	0.892359255	0.070423988	0.977721011	-0.806212256	0.25277856
AT3G47400	AT3G47400	-8.210225416	0.00884436	-8.882513462	1.05274E-06	-1.52205546	0.214127724	-1.849631441	0.652047062	-1.605614256	0.099101096	0.421936745	0.400301014
AT5G24105	AGP41	-7.538373305	0.006040473	-8.903884997	8.83152E-07	-2.389208839	0.221479892	3.26790259	NA	-0.229273501	0.985862786	0.1759922	0.891669695
AT5G24770	VSP2	-2.32800447	0.018849723	-9.040190115	5.61111E-07	-2.041014208	0.232990493	2.148438944	0.828386835	-1.134483773	0.499322416	-0.587429725	0.241913307
AT2G39880	MYB25	-2.81293473	0.0009183	-9.721312956	3.75672E-10	-1.563644481	0.091484785	-3.672044537	0.472697787	0.726863197	0.528718647	0.533843765	0.159838739
AT2G43610	AT2G43610	2.152817062	0.035020999	-1.289759621	0.638950591	-5.360514078	0.000654911	-2.248631845	0.019675654	-1.614505648	6.89551E-06	-3.054781565	0.000354512
AT3G16150	ASPGB1	1.689859779	0.044818479	-0.020956823	0.989512899	1.784438055	1.26935E-05	-2.734682374	0.039793784	0.072750926	0.97926944	-1.955128967	1.36326E-09
AT4G25400	AT4G25400	9.217844335	6.30419E-07	-5.772144107	0.276503596	-6.419208438	0.020020882	-4.957003992	0.003248606	-0.359053504	0.864675247	-2.846068753	2.9578E-12
AT1G74930	ORA47	-2.041747917	2.95763E-05	-0.974515739	5.02613E-05	-2.423081758	0.004264542	2.759184325	0.036952766	-0.950541929	0.19374197	3.898699955	1.67878E-49
AT4G21400	CRK28	1.353268035	0.000499762	0.3358011	0.301878813	1.662918692	5.46053E-15	1.070106623	0.039793784	0.572923181	0.005430336	0.259579998	0.179894346
AT1G53460	AT1G53460	1.154308086	0.017868736	0.674210025	0.058891325	1.120022915	0.000209344	1.014718254	0.026396562	0.377802593	0.362631268	-0.056982995	0.865945075
AT5G45280	AT5G45280	-1.649607572	3.84428E-06	-0.45554248	0.346073889	-2.108461229	1.24274E-18	-1.42525538	0.048477694	-0.999282256	1.91079E-07	0.342466012	0.008587919
AT1G30720	AT1G30720	1.432505276	0.00089687	0.253616478	0.762460134	1.460436741	1.21405E-07	0.708056673	0.714772015	-1.828323114	7.8915E-06	-1.523391912	1.04476E-07
AT2G21045	AT2G21045	3.373150592	0.009812774	-0.227291095	0.875332256	-1.874495364	0.002385399	-1.775799735	0.663046749	-1.388941827	0.032013489	-2.110610549	2.96589E-14
AT3G01190	AT3G01190	6.356287319	1.03122E-06	-0.321832544	0.943194995	-6.862487132	0.005024705	-1.82522305	0.646729152	-3.647970599	2.35484E-18	-4.840492902	2.30719E-08
AT1G56430	NAS4	-2.521886675	0.018123514	-0.910721541	0.431341694	-2.715925822	0.023819999	-2.147572086	0.615358268	-1.940134393	0.011022037	-1.615793476	2.049E-06
AT1G76930	EXT4	2.31431693	6.16147E-17	1.941717187	0.077199606	2.630000269	0.003507083	-1.353313792	0.377008424	-1.479552569	0.005864299	0.667363944	0.000545119
AT5G25980	TGG2	-1.81402963	0.001105737	0.432142965	0.795579281	6.823614643	0.026404543	1.469044104	0.732322487	2.801663143	8.72297E-08	-2.851812313	0.485954896
AT3G45650	NAXT1	-1.312682432	0.009670039	-0.734167093	0.162337695	-1.245954672	0.008892394	-0.935456557	0.034723544	-1.188356676	0.002616749	-0.322656209	0.192943039
AT1G79620	AT1G79620	-1.842061748	0.041360778	-1.583712186	0.102414291	-1.133915859	0.031389715	-0.772579866	0.65378996	-1.008947907	0.002554745	-0.719693521	0.017011655
AT2G38080	IRX12	-2.433068769	0.000247925	-0.991773415	0.040244757	-1.921503361	0.000231483	-0.841691861	0.544222418	-1.036945332	0.007443264	-0.564367317	0.029089024
AT1G80240	DGR1	-2.548523012	6.90593E-07	-3.599803957	0.306311229	-4.13432386	0.001274633	-0.297762955	0.891851015	-1.771095309	0.000121281	-1.261713418	0.051043283
AT1G58235	AT1G58235	1.259293331	2.81454E-06	0.866328597	0.005269477	1.225643168	1.87753E-09	-0.311060259	0.66808962	-0.165667216	0.605282789	-1.024844765	5.3015E-10
AT3G30210	MYB121	9.396665326	2.46259E-07	3.114192985	0.475286352	2.548334236	0.001397684	-2.149777724	NA	-0.421942627	0.846389857	-1.082141972	5.62656E-08
AT5G43580	UPI	3.531134795	2.03269E-18	0.326841807	0.727336861	3.010538151	0.01100574	0.153568808	0.963390011	0.022892482	0.980663768	-1.111464507	0.021797159
AT1G12200	FMO	1.401625407	0.000465928	0.782851771	0.004826594	1.044886797	1.86593E-08	-0.524702561	0.637815859	-0.094267876	0.8193214	-1.114912886	5.86499E-24
AT5G62920	ARR6	1.342327578	3.96832E-05	0.818567021	0.084021178	2.080553299	4.22014E-22	-0.239593806	0.78630406	-0.128789131	0.78520308	-1.16291112	1.07045E-13
AT5G01610	AT5G01610	2.016322067	0.000448661	0.355232882	0.727719313	1.704842946	0.016860751	0.644016096	0.637815859	0.257065539	0.794004103	-1.235463086	0.024993715
AT4G31380	FLP1	1.409207104	0.00020156	0.555196969	0.118762992	1.444237443	3.35969E-09	0.007217149	0.997449367	0.268893496	0.344099076	-1.292205065	2.36493E-14
AT3G19390	AT3G19390	1.35132785	0.00131646	0.166425177	0.880257351	2.19176402	2.48319E-13	-0.792077727	0.598901706	-0.343873261	0.55798645	-1.385948064	9.69391E-07
AT4G01670	AT4G01670	2.92020566	5.65393E-05	1.081037871	0.152850175	1.065393647	0.018130069	0.72066515	0.826599845	-0.884327069	0.396940123	-1.391703138	0.003564742
AT1G62035	ath-MIR171c	8.669699923	9.85132E-05	3.838155037	0.099576739	6.627763468	3.1096E-05	-1.530148609	0.742761608	-0.803657093	0.616572022	-1.398811835	0.008423902
AT5G48110	AT5G48110	1.713243214	0.002224808	0.675497226	0.23957502	1.539940137	0.00117509	-0.315036789	0.770575751	0.012786851	0.989503241	-1.497152523	1.02367E-11
AT3G26440	AT3G26440	1.608909558	0.012263336	-0.117385238	0.897577355	1.308729787	0.027659474	-0.560202894	0.747544101	-0.056101905	0.950394071	-1.539676273	1.20008E-08
AT1G15040	GAT1_2.1	3.155362855	6.18357E-13	1.103872023	0.07872436	2.079765305	3.99115E-18	1.418336763	0.52463539	-0.584694881	0.556508441	-1.548997337	1.26221E-08
AT4G35750	AT4G35750	1.72461149	2.5544E-17	0.602588891	0.000811332	1.156003083	5.10845E-09	0.439822131	0.695103554	0.127982974	0.721412596	-1.551526424	8.74246E-19
AT5G40960	AT5G40960	3.130040481	0.021618325	0.864992837	0.386633495	2.514565003	0.001214923	-0.450396928	0.835055333	0.285125245	0.788769673	-1.754652843	7.69428E-06
AT1G15380	GLYI4	1.915650663	1.38447E-08	0.78925649	0.005367725	1.524031468	4.04499E-31	-0.387359888	0.886485915	-0.495642627	0.125239905	-2.100436723	2.97009E-29
AT2G25770	AT2G25770	4.990095487	7.23242E-12	2.107433555	0.085410566	3.58527314	9.91238E-17	0.805759826	0.760347123	-0.624577877	0.433938829	-2.331933399	1.29546E-09
AT2G16190	AT2G16190	8.959968874	1.78994E-05	2.410856934	0.236057046	4.054316817	6.62891E-05	-0.647054344	0.922389968	0.009140674	0.999129744	-2.431129023	0.02492011
AT5G65080	MAF5	1.567887279	0.040671779	0.930596889	0.45861172	1.735215371	0.002232185	-1.648746639	0.433704774	0.25532446	0.901217481	-2.609573839	1.47067E-05
AT3G46690	AT3G46690	1.713265874	0.048930241	1.703879924	0.134563554	2.444037967	1.3432E-07	0.87912882	0.706563034	0.011269607	0.996000267	-2.956400387	4.33593E-08
AT4G21120	AAT1	3.251338792	0.000612826	2.442674415	0.245627465	3.750728102	0.001135472	-0.696221534	0.696784826	-0.254701171	0.811222926	-3.268474078	5.88849E-23
AT3G45700	AT3G45700	8.768972482	0.003237248	0.589128053	0.907514396	-6.1075681	0.049235961	0.016113722	0.993618504	-0.539115057	0.552737263	-1.681134082	1.97464E-05
AT1G10480	ZFP5	4.323384169	0.00106403	3.182990715	0.05207685	-7.291851732	0.001005713	-0.857646858	0.723308816	-1.193554658	0.067570199	-2.561007122	3.48982E-13
AT1G31050	AT1G31050	4.094215964	0.004201675	-0.295279378	0.840318376	-5.717971531	7.54784E-05	-1.342196676	0.735220801	-0.491016508	0.47166418	-2.375427778	1.35965E-21
AT3G04570	AHL19	1.949263106	0.01808408	-0.183686896	0.90574659	-8.207525954	3.64081E-06	-1.373898718	0.635731984	-0.486390604	0.296836932	-2.941611091	2.27599E-05
AT4G13310	CYP71A20	1.954763554	0.04371811	0.829894808	0.487054444	-4.109120474	0.026094776	-1.65456543	0.598901706	-0.203628001	0.872795006	-2.979801128	4.89363E-16
AT3G24310	MYB305	4.871395652	0.003301139	1.68460986	0.210763072	-6.042030345	0.006663166	-1.833371784	0.515156234	0.123285528	0.933631495	-3.403294192	0.000815374
AT1G54000	GLL22	4.842707438	5.43407E-05	-1.421600346	0.457867474	-2.836791536	0.038875051	-1.973609137	0.413866874	-0.143156416	0.848181428	-2.442466407	5.74181E-39
AT2G32270	ZIP3	9.609017152	1.54826E-07	1.347578285	0.586242507	-5.421811578	0.041074716	-2.076564872	0.622600949	-0.940344957	0.404527441	-3.274130887	5.42948E-13
AT2G41480	AT2G41480	3.342047656	6.43605E-05	-0.862727838	0.786146042	-1.772428653	0.022753268	-2.843516695	0.62738272	-0.915576926	0.000119918	-1.849840115	2.56173E-49
AT1G65390	PP2-A5	-3.401490057	1.04222E-25	-3.989053638	0.106012385	-3.894219475	1.65171E-16	1.440116361	0.765661972	-0.534286225	0.843540715	3.774152412	1.59642E-14
AT5G41740	AT5G41740	-3.682621734	1.07484E-25	-1.793234533	0.219134395	-2.796659135	0.000235015	-1.163328825	0.585524552	-1.310854349	0.147894848	3.243759084	4.67191E-30
AT1G16410	CYP79F1	-2.859151085	3.07199E-05	-0.77594776	0.562278728	-3.118247438	0.004440803	-1.268533449	0.806948566	1.787422896	0.621001856	2.975531637	2.59268E-07
AT1G28370	ERF11	-1.387370016	0.023734354	-0.701175781	0.40442208	-1.223035742	0.030394294	0.84684332	0.726900342	-1.276359517	0.256505051	2.82396217	1.37632E-11
AT3G56400	WRKY70	-1.349287289	0.027317715	-1.034263651	0.071213734	-1.232372424	0.000214375	1.480114541	0.422258087	0.096927408	0.944320981	2.401560102	2.73342E-40
AT1G20510	OPCL1	-2.095103803	4.53286E-10	-0.924446582	0.006908015	-1.983817672	1.96847E-10	-0.106737106	0.970881852	-0.281242289	0.68153437	2.070431579	1.0873E-52
AT2G29310	AT2G29310	-1.505637056	0.003929029	-0.756037776	0.008596243	-1.081325151	4.05714E-09	1.981673746	0.261605458	0.359944134	0.680666228	2.022321988	1.18167E-15
AT1G78370	GSTU20	-3.273862286	0.020648505	-0.540360975	0.736113487	-3.330380788	5.34625E-05	0.813638669	0.690375404	0.517877168	0.731432711	1.956915487	7.00932E-45
AT1G17380	JAZ5	-1.419140282	0.021862716	-0.616371101	0.325436449	-1.592235259	0.040767581	-1.06888077	0.554617999	-0.386599399	0.821510242	1.944432979	3.55229E-11
AT3G13600	AT3G13600	-1.881413013	6.23378E-06	-1.743015276	0.271567187	-2.406477987	0.003356358	-0.174876108	0.910686362	-0.339007297	0.596277828	1.719963417	5.78762E-09
AT4G23020	TRM11	-1.302053507	0.029667803	-1.027123863	0.25681456	-1.792239674	5.47162E-05	1.108359655	0.640704059	-0.269513831	0.864675247	1.645632636	2.87609E-08
AT4G27440	PORB	-1.712696408	4.86638E-19	-1.286877566	0.083575521	-1.766303853	1.72218E-33	0.979845891	0.242292325	0.687618872	1.25277E-07	1.641911032	1.81561E-39
AT1G60260	AT1G60260	-1.893731882	0.000127853	-0.491967188	0.472063276	-1.045563811	0.000395437	1.004438968	0.603726755	1.560616435	0.058461349	1.60422688	1.01937E-14
AT3G06880	AT3G06880	-2.809488588	0.002228893	-2.552965869	0.36185984	-2.599339968	2.06261E-11	0.142540068	0.972495118	-0.017027489	0.985172934	1.526726488	7.17008E-23
AT1G51110	AT1G51110	-1.806290953	6.03631E-05	-0.974197567	1.79266E-06	-1.167647853	3.50644E-09	1.637070403	0.296836814	0.405057721	0.619118822	1.449545867	2.45231E-11
AT4G23180	CRK10	-1.489126576	0.00035726	-0.425579885	0.018081299	-1.414615945	6.69468E-05	0.388966167	0.869264259	-0.666777348	0.035605525	1.398594391	1.22634E-14
AT3G28080	UMAMIT47	-3.707010558	0.006736058	-2.901412707	0.05585476	-1.714337771	0.004991769	-0.994120416	0.918503553	0.669949654	0.830996968	1.369646897	0.020374369
AT1G78460	AT1G78460	-1.477813206	2.23898E-10	-1.256524933	0.118686852	-1.080554671	0.001290573	1.420714547	0.149173732	-0.119393789	0.959320035	1.329618208	2.47654E-16
AT1G19670	CLH1	-1.987500103	0.021327479	-1.079490938	0.065733071	-2.461344841	1.30409E-10	0.783130725	0.726900342	0.592879617	0.374534812	1.278364204	8.46484E-09
AT5G17170	ENH1	-1.517632864	1.19465E-05	-0.532878782	0.010945261	-2.026375706	1.40547E-30	0.858118141	0.554617999	0.372181585	0.337228984	1.256775753	1.98739E-14
AT3G52180	SEX4	-1.480495505	0.008449777	-0.852870283	0.019140965	-1.378166942	3.13348E-08	0.060121276	0.973178391	0.658303379	0.061893219	1.241246545	6.53383E-16
AT5G51100	FSD2	-1.671747824	0.001723685	-0.989620305	0.007508079	-1.06904822	0.001385973	0.429293995	0.652857308	0.199538403	0.698582521	1.23975851	2.79248E-19
AT3G05730	AT3G05730	-2.547532249	0.036987569	-1.024269716	NA	-3.287003808	2.46681E-68	-0.559968972	0.870136387	-0.303210393	0.916169477	1.210492635	1.29052E-11
AT4G17560	AT4G17560	-1.192303477	4.15982E-08	-0.891128352	0.000103231	-1.452732654	2.2705E-28	0.658086874	0.343493265	0.505641262	0.003528517	1.185429806	1.62836E-25
AT2G30890	AT2G30890	-2.363636427	0.031338953	-3.068967824	0.204148337	-3.504737035	0.011795149	-0.891133477	0.8667671	-0.487788058	0.714991703	1.171828732	0.037731645
AT5G10100	TPPI	-1.968802873	0.003347472	-0.5156982	0.258530189	-1.328822156	0.010300038	-0.278481215	0.808442563	0.101712662	0.89611977	1.165270683	1.94111E-06

AT5G61420	MYB28	-2.074440121	0.01809542	-2.509797846	0.073379235	-2.156361988	0.004517416	-0.083538055	0.981525175	-0.184194492	0.807054326	1.135459631	1.85939E-13
AT3G28930	AIG2	-1.694519098	0.000319002	-0.180283549	0.817233287	-1.85322805	0.003239802	-0.04882989	0.984928214	-0.446652127	0.545156804	1.118244692	4.33279E-05
AT4G38950	AT4G38950	-1.581530147	0.013178935	-2.283424992	0.165930228	-2.607393594	6.39235E-10	-0.261992243	0.907707664	0.106310493	0.933770468	1.1181291	1.8691E-12
AT1G78820	AT1G78820	-1.52595891	3.63829E-06	-0.677104186	0.012205526	-1.54661245	3.331E-12	-0.046443307	0.981845063	-0.482486786	0.22924511	1.117858044	4.7583E-12
AT5G35740	AT5G35740	-2.812140763	0.000377407	-1.005981204	0.577487316	-2.245694595	1.45116E-05	-1.221208034	0.567190016	0.275443332	0.675049685	1.108652212	0.00022076
AT4G37800	XTH7	-1.790616733	4.17259E-11	-0.144054858	0.867790997	-1.411675066	6.03612E-20	-0.535032358	0.602119156	0.553602102	0.114406998	1.093396664	7.67766E-11
AT5G21920	YLMG2	-1.128753624	6.46678E-05	-0.10311277	0.82176617	-1.093298086	6.77892E-11	0.774077244	0.62053317	0.134823225	0.819806715	1.080001931	5.43707E-09
AT5G42070	AT5G42070	-1.524539765	0.015966707	-0.535394536	0.105405608	-1.171764405	5.24835E-06	1.15075249	0.569286428	0.673396793	0.183982731	1.079929954	0.000106099
AT3G61950	AT3G61950	-3.127484235	0.023603956	-1.757210993	0.121859617	-2.075933245	0.001380955	0.181180334	0.982734229	0.497217505	0.770730129	1.068963908	0.0099057
AT3G13120	AT3G13120	-1.44551538	1.00407E-10	-0.870218476	6.37793E-05	-1.082906823	6.58881E-17	0.527315697	0.550417276	0.310864876	0.209073558	1.062351048	1.70153E-10
AT1G56540	AT1G56540	-1.457336844	0.006627632	-0.196396109	0.82972225	-1.700508234	8.43287E-05	-0.100106714	0.972495118	0.086180899	0.956826005	1.06178953	0.000310572
AT3G23400	FIB4	-1.408661258	0.000198141	-0.910319048	2.94382E-11	-1.318857287	9.31825E-10	0.542765766	0.613859108	0.271161106	0.557703713	1.033059638	3.80865E-15
AT5G55930	OPT1	-2.964708069	0.003125269	-1.498799309	0.058691223	-1.683793773	0.019911555	0.728232271	0.721140128	0.651325166	0.570854222	1.030264972	0.021266708
AT5G40950	RPL27	-1.28547627	2.71941E-11	-0.721594063	2.10378E-08	-1.653642738	4.06147E-34	0.477777029	0.577728436	0.397054618	0.044701841	1.017283056	5.67149E-10
AT1G78630	emb1473	-1.537516557	8.03619E-19	-0.778593843	3.12568E-07	-1.128031796	3.51943E-33	0.673225835	0.465456099	0.331268847	0.128759545	1.008083398	5.99816E-13
AT1G06830	AT1G06830	-1.986179279	0.007467569	-1.371113624	0.427002427	-2.364998948	0.007609396	0.371172491	0.884201248	0.66246019	0.331042184	-1.005185639	0.041871092
AT2G44290	AT2G44290	-2.207231161	0.000326242	-0.490867242	0.279334269	-1.213823506	0.016872126	0.238448356	0.950313494	-0.851641197	0.352819474	-1.468234551	0.014436487
AT1G71200	AT1G71200	-7.999257907	0.000707118	0.272734406	0.944922771	-2.878525538	0.026144235	-2.113194437	0.716554635	-1.327889673	0.666285275	-1.966694217	0.025951822
AT2G29130	LAC2	-4.116549192	0.000955308	-1.802812054	0.356243355	-4.848894069	0.004554428	-0.17647382	0.981893161	-1.225615905	0.377996082	-2.065689153	3.19762E-12
AT1G77960	AT1G77960	-5.918687872	0.000206487	-2.997062896	0.165900251	-1.87637956	0.002082659	6.768657656	0.579644733	-4.954503713	0.550184567	-5.86761996	0.036606235
AT5G03550	AT5G03550	8.183873969	0.011644505	0.065279863	0.989804887	6.922053349	0.003240732	-1.162147185	0.814118697	0.296420995	0.886464423	-1.142828866	0.255911851
AT1G79130	AT1G79130	8.287789954	0.000231687	5.020985215	0.077029982	4.087811115	0.034602096	1.822970341	0.902565251	-4.02940546	NA	-0.009528033	0.997852969
AT4G02810	FAF1	2.421732993	0.007606206	1.323706192	0.569904334	3.739483311	1.42632E-11	0.766427351	0.653363031	-0.308203033	0.868188276	-0.41466416	0.267803767
AT2G28056	ath-MIR172a	8.547465075	0.004889293	6.753627624	0.194328386	3.56199511	0.038594047	-0.140465632	0.97809523	-0.122294521	0.958701896	-0.444023258	0.27137584
AT2G34610	AT2G34610	8.318748867	0.000435501	3.47862722	0.458559728	3.259514022	5.31336E-05	-2.450502714	0.604422229	-0.548216695	0.830492367	-2.762675061	0.166570147
AT5G20240	PI	2.926130373	0.000374673	-0.446545155	0.762385403	3.190032649	7.38388E-11	2.540917672	0.604422229	-0.928949449	0.608161551	-0.239065011	0.806305096
AT4G36740	HB40	10.48095152	3.69721E-11	2.549401458	0.143890383	3.036055988	0.000330452	1.563088637	0.207276291	0.612184769	0.542006423	-0.173834151	0.782278679
AT2G40670	RR16	2.319208713	0.006797584	1.232012422	0.087478397	2.933850703	2.1474E-07	0.117792934	0.963305035	0.134613712	0.890198216	0.397908864	0.354766069
AT4G15258	SNOR37-2	6.546129757	0.002474037	-0.428280256	0.913380163	2.741508837	0.000550024	0.026431167	0.997285903	1.337031508	0.618270343	-0.532241939	0.483966771
AT1G25530	AT1G25530	2.590456113	0.000200735	1.936980588	0.453288505	2.540549943	1.78945E-19	-0.566398363	0.760771281	0.025668441	0.977935511	-0.214834118	0.103394386
AT2G43510	TI1	2.519635702	0.01184923	0.903519497	0.257607203	2.201616957	0.000569327	-0.907295337	0.847168445	0.141514317	0.922377562	0.171385873	0.687397949
AT3G56260	AT3G56260	1.92238534	0.045146805	2.403709458	0.116961519	2.14776261	0.000105762	-2.038524199	0.057938647	-0.28165067	0.828433141	-0.009068693	0.989307115
AT3G09910	RABC2b	4.175857026	0.001056367	1.720040378	0.241522862	2.074348192	1.1189E-07	-1.363065692	0.511554278	-0.245479823	0.844918508	-0.106331318	0.724645316
AT1G23120	AT1G23120	2.306606479	0.00370055	1.161658149	0.243004458	1.868736811	1.22069E-25	-0.643141275	0.587300556	0.080024589	0.947801595	-0.516268841	0.00863867
AT5G09220	AAP2	1.474170702	2.21196E-05	-0.312174528	0.465247914	1.861570479	0.003826443	-0.111864674	0.951334464	0.054867494	0.899429705	-0.213560222	0.18736611
AT3G54720	AMP1	1.061103658	0.000760944	0.894603669	0.000102477	1.782759225	6.42893E-13	0.727281585	0.62738272	0.249016393	0.471497157	-0.170322496	0.367327511
AT2G20142	AT2G20142	1.42979409	0.041798464	0.975695318	0.450075403	1.769326076	0.006458561	-0.184017839	0.925849553	-0.445751798	0.548029196	0.609451708	0.002224231
AT2G42980	AT2G42980	3.255337787	0.034085966	1.903889493	0.137517519	1.75015419	0.025079822	-1.052328714	0.839957919	-0.37808151	0.932254226	-0.269020884	0.761927143
AT2G17036	AT2G17036	1.239460751	0.04774532	0.890126519	0.001684219	1.739932705	1.28773E-09	2.260995922	0.146113635	0.490145069	0.524307711	0.678450181	0.010023416
AT4G24450	PWD	2.112728921	0.002339372	0.784459839	0.086099792	1.739189351	5.12395E-13	0.848328552	0.340875713	0.088664466	0.934609223	0.469610736	0.021798346
AT3G47300	SELT	1.624884741	0.000701453	0.764582685	0.097139987	1.71461264	4.31962E-19	-0.867232725	0.451431824	0.023496081	0.980633826	-0.651504065	1.72406E-05
AT1G62480	AT1G62480	1.300918319	2.03125E-06	0.36047789	0.043510381	1.712815477	1.01898E-31	-0.369578806	0.477988145	-0.150189708	0.490131911	-0.185433795	0.494261571
AT1G09140	SR30	1.105115756	2.42125E-05	0.978602415	6.90369E-05	1.670656289	2.59298E-25	0.666176069	0.576396288	0.229641525	0.485888437	0.128728599	0.528989295
AT3G57300	INO80	1.02383247	0.004779427	0.88024577	3.42027E-07	1.666224714	3.3586E-45	1.098701831	0.281913411	0.167888703	0.474364938	0.205785238	0.140539219
AT3G17225	AT3G17225	1.488879472	0.040333667	1.072416223	0.199433211	1.665816715	0.003161656	-0.011057101	0.996799452	0.49474634	0.690504472	0.474928345	0.3498426
AT2G18440	GUT15	1.003474943	0.000721543	0.474293656	0.002508356	1.663087418	1.70002E-77	0.470679094	0.646729152	0.418776228	0.011141694	-0.22344544	0.444054194
AT1G53690	AT1G53690	3.162821096	3.71188E-05	1.35703162	0.544440572	1.642308527	4.03904E-07	-0.259955151	0.93556951	-0.241483332	0.837269281	-0.341352929	0.660939303
AT2G29420	GSTU7	3.000177216	0.02273132	1.45348649	0.294956481	1.636143169	2.27735E-13	0.71871642	0.604422229	0.964065203	0.09968568	-0.3334455	0.254595774
AT5G05860	UGT76C2	1.071337276	0.001364255	0.786221045	0.019000932	1.596051848	7.48715E-15	-0.239004844	0.684184988	0.252074112	0.424511917	-0.276215244	0.09375178
AT2G14170	ALDH6B2	1.249722258	0.001859558	0.793160224	1.11953E-05	1.582371849	1.29703E-21	1.557456065	0.343779816	0.149183133	0.558061075	0.189563118	0.213188885
AT4G31290	AT4G31290	1.434738855	1.07577E-08	0.496826081	0.016128325	1.571991045	1.29096E-23	1.046548176	0.292318054	0.749872184	1.42338E-08	0.577713411	1.11628E-06
AT3G48100	RR5	1.102735171	0.000618211	0.00181243	0.997838226	1.534717761	9.54796E-13	-0.176658606	0.829537284	-0.202414895	0.503819458	-0.749591628	1.93719E-06
AT5G41270	AT5G41270	1.245935678	0.003232938	0.87233608	0.019489754	1.534050049	1.05153E-09	0.272588857	0.813799839	0.322666579	0.462524306	0.00647226	0.988297148
AT1G02840	SR34	1.355830429	2.18216E-12	0.839748363	2.75457E-09	1.505484236	7.7942E-42	0.633291441	0.521675277	0.508188313	0.000794764	0.096839499	0.684873307
AT5G46050	PTR3	1.68439764	0.004671193	1.225769232	NA	1.486021042	0.026997823	-0.104000267	0.951840095	0.174491137	0.828846482	0.034873598	0.931132053
AT1G18330	EPR1	1.27706099	0.000996764	0.981561723	1.90941E-06	1.443445451	7.90146E-12	1.718039242	0.203276234	0.118643179	0.713858938	-0.471493876	0.000184843
AT3G57170	AT3G57170	1.035414596	0.002091152	0.965741862	0.000345921	1.437635353	3.2722E-26	0.594093911	0.596296903	0.193163718	0.563990873	0.47371244	0.002622993
AT1G01640	AT1G01640	1.874424245	0.00357416	1.05663038	0.115518293	1.434159277	0.001713199	-0.583889405	0.695103554	-0.299552855	0.327553421	-0.796468802	0.000280718
AT5G46840	AT5G46840	1.406734342	3.37766E-11	0.691151919	0.000115683	1.421252141	1.81753E-21	0.331709767	0.598901706	0.281641856	0.151285272	-0.269639523	0.099958957
AT4G14900	AT4G14900	1.273601705	4.45241E-06	0.939369804	1.20879E-09	1.418187835	2.23814E-19	0.06429693	0.972495118	-0.220619161	0.286362954	-0.435825611	0.000568784
AT1G05890	ARI5	1.138540954	0.000161919	0.949527696	8.53666E-09	1.408737554	4.02921E-27	0.316773549	0.760553279	0.015275159	0.974021484	-0.440549588	0.001649788
AT5G43930	AT5G43930	1.017128063	0.00372471	0.934784795	1.14636E-05	1.407331182	7.47997E-17	0.665410962	0.653363031	-0.038276997	0.936116842	-0.099809493	0.653449905
AT1G10890	AT1G10890	1.262721257	0.000583231	0.835603181	8.16907E-06	1.396104243	6.06067E-16	0.820959592	0.34017207	0.354095434	0.157028396	0.15308856	0.562577219
AT1G55810	UKL3	1.508037565	6.83234E-05	0.60561231	0.001342175	1.384603366	5.80095E-12	1.488320038	0.204145338	0.246076604	0.264748823	0.470764675	0.000201738
AT3G15620	UVR3	1.461434457	0.001604875	0.231710466	0.429495844	1.377986558	7.86476E-11	1.258307752	0.477936046	0.20359276	0.621484795	-0.067399681	0.761927143
AT3G23150	ETR2	1.787193682	0.00021925	0.992631254	6.37793E-05	1.373697489	3.10848E-05	-0.065484729	0.961727612	0.505974444	0.034660889	0.543569789	0.000804505
AT2G34590	AT2G34590	1.357110674	0.000191189	0.712475497	1.91333E-05	1.373110582	3.22589E-17	1.088118666	0.425005763	0.258566873	0.284897888	-0.172571767	0.232083273
AT1G07300	AT1G07300	1.808502363	0.019816185	0.666999265	0.230208409	1.370514205	0.001070824	0.249935493	0.838133701	-0.031081273	0.977308664	-0.360255817	0.225818743
AT1G67580	AT1G67580	1.060609756	0.000210204	0.86253053	4.3007E-08	1.348804856	1.01909E-29	0.701329512	0.598901706	0.236390809	0.272722436	-0.269014892	0.230102946
AT5G16110	AT5G16110	1.301921734	3.4954E-05	0.800353693	1.02711E-11	1.334790384	8.42141E-16	0.374801213	0.774421775	0.052177021	0.869846959	-0.820904508	1.36519E-12
AT2G36930	AT2G36930	1.089231908	2.59551E-05	0.783851912	9.53925E-06	1.33468768	2.13594E-18	0.204840323	0.820941695	0.127480355	0.588786155	-0.018065131	0.930657572
AT2G24530	AT2G24530	1.295774492	0.002231063	0.746481173	0.162358652	1.334638464	0.000152134	0.419226296	0.689293428	0.402552244	0.484585201	0.152577732	0.658193338
AT5G23080	TGH	1.120562886	2.45156E-05	0.610473712	0.003037565	1.330735381	9.54219E-18	0.60861281	0.676109289	0.270661499	0.298693478	-0.182415168	0.307430527
AT3G16050	PDX1.2	1.356834055	0.035528819	0.981227778	0.080080834	1.312700598	0.036269697	0.000107507	0.999880715	0.620552233	0.183982731	0.132697276	0.69516335
AT1G73630	AT1G73630	1.833890042	0.000400707	0.589474219	0.420374903	1.30746083	0.000489231	0.041752566	0.982055475	0.573305992	0.195022122	-0.275637294	0.486287512
AT2G44900	ARABIDILLO-1	1.133181649	0.003733138	0.786466758	4.74795E-05	1.30058258	7.64904E-11	0.951464604	0.373396563	0.292601048	0.295937959	-0.203602953	0.250559605
AT3G13670	AT3G13670	1.171916188	1.78612E-05	0.817543044	8.45031E-10	1.298397544	2.71758E-29	0.283004998	0.761621094	-0.062566835	0.848060883	-0.332265869	0.019617804
AT5G20350	TIP1	1.051136331	0.001613249	0.917661928	1.09964E-11	1.283857202	2.18735E-11	0.65407977	0.64702911	-0.067383585	0.844918508	-0.42194478	0.001934807
AT1G17410	AT1G17410	1.011381114	0.04675717	0.866163314	0.062723702	1.282755914	1.05122E-11	0.303107242	0.859442872	0.564823269	0.115937316	-0.080944679	0.759380031
AT1G53780	AT1G53780	1.098564724	0.000284883	0.998709956	1.52375E-06	1.282749451	1.41911E-17	0.511827458	0.663046749	0.158625771	0.61085978	-0.245518767	0.0871747
AT1G64040	TOPP3	1.610940306	8.89457E-08	0.902331456	4.34938E-07	1.273970713	2.14993E-19	0.461045779	0.677861669	0.299307235	0.177117809	-0.296291254	0.050213867
AT5G66360	DIM1B	1.352680885	0.00361783	0.656390636	0.110614355	1.27349633	0.000584682	0.925406017	0.607986058	0.469759806	0.191049475	0.035762653	0.903810867
AT4G18140	SSP4b	1.344086247	0.001525243	0.86683937	8.8316E-05	1.270116028	8.64574E-19	0.628103042	0.642268593	0.421782685	0.161848929	-0.042516222	0.898780501
AT5G26240	CLC-D	1.02588153	0.002744383	0.790976772	0.000528027	1.265841542	1.70045E-14	0.377247228	0.676109289	0.117844102	0.729103822	-0.018923265	0.936148146
AT3G55770	WLIM2b	1.612756869	9.40987E-11	0.949762285	4.54188E-15	1.253811725	1.18147E-22	0.342196143	0.726553713	0.153134493	0.42662725	-0.398930951	0.000901341
AT1G29970	RPL18AA	1.52609399	1.06654E-07	0.954882778	6.47764E-06	1.251923369	4.9072E-13	0.136817345	0.936758504	-0.096989633	0.791823839	-0.757116684	9.07991E-06
AT2G19540	AT2G19540	1.323150747	9.40404E-05	0.895710935	6.06169E-07	1.245973468	3.71067E-17	0.100263428	0.933923133	0.162058563	0.496280372	-0.005869002	0.979855671
AT3G29035	NAC3	2.522885997	1.19212E-08	0.979310406	0.122980162	1.243824883	0.007512713	-0.541920505	0.763069171	-0.147330384	0.732834061	-0.86061051	1.14776E-06
AT5G58040	FIP1[V]	1.17805046	6.02829E-05	0.859162037	1.08506E-08	1.237461842	1.16807E-21	0.320086021	0.784469624	-0.078001823	0.811017735	-0.573084307	2.06902E-05
AT3G61198	AT3G61198	1.341519524	0.014106377	0.492582635	0.258205282	1.232649127	0.00125092	1.983227138	0.343493265	0.341435358	0.655417993	0.819316925	4.88067E-05
AT3G45610	DOF6	1.460555528	0.002267183	0.974494193	0.123678972	1.222606268	0.000648806	1.381759821	0.186021173	0.537002721	0.381034551	0.916158081	5.12992E-05
AT5G59440	ZEU1	1.145493451	0.000583184	0.60883035	0.013705634	1.207141345	1.59968E-08	0.49408538	0.572584637	0.370279463	0.047902811	0.082189574	0.651462259
AT1G33790	AT1G33790	1.076102624	0.004886708	0.712096282	0.003498362	1.205686581	4.42429E-06	0.953235321	0.501273844	0.325017395	0.340858268	0.583737296	0.000564805
AT3G61600	POB1	1.173022219	4.16866E-05	0.91315962	5.44219E-16	1.204557197	5.87327E-24	0.96467017	0.465456099	0.204915787	0.342870836	0.101347981	0.483300265

AT5G09270	AT5G09270	1.801990638	0.00013954	1.206467301	0.142467445	1.204398562	0.000434918	0.012449165	0.993718604	0.439078013	0.341342649	-0.141482149	0.579556091
AT5G47720	AACT1	1.679998129	7.41747E-09	0.998204148	0.000228945	1.192238524	7.10106E-10	-0.351666616	0.741369316	-0.25025885	0.517863855	-0.72881575	1.89812E-06
AT1G53030	AT1G53030	1.442182047	0.0009798	0.988558295	0.002936212	1.186953114	6.42069E-06	0.495978612	0.632376028	0.445713246	0.397816908	-0.076518522	0.825500327
AT4G12680	AT4G12680	1.709870876	0.000757589	0.918794258	0.065695468	1.183685874	2.67322E-06	0.962729003	0.537763881	0.646790507	0.168946553	0.310397166	0.193505372
AT3G13277	AT3G13277	1.304762341	0.047237683	0.949548219	0.197608809	1.180990712	0.019964002	-0.125302063	0.935645553	0.172452137	0.84618077	-0.780027044	0.001816118
AT2G45620	AT2G45620	1.226481455	1.59702E-06	0.8442511	1.96191E-10	1.174426769	7.30419E-16	0.285985066	0.808237728	-0.141326794	0.590113348	-0.607709664	1.32585E-05
AT3G10030	AT3G10030	1.424198609	8.59924E-08	0.593916018	0.002615945	1.172839212	1.62063E-16	0.577228336	0.632278424	0.270430291	0.270404042	-0.178834256	0.35346788
AT3G51730	AT3G51730	1.106121763	0.001025197	0.772642429	0.002604517	1.171046051	5.77912E-10	1.116650925	0.562683359	0.151440984	0.53525964	-0.52041394	0.000483818
AT4G19190	AT4G19190	1.188610462	0.000321142	0.978702274	6.31997E-08	1.168148244	1.6167E-13	0.923764598	0.598901706	0.028031751	0.963411941	-0.384439954	0.034564624
AT1G77180	SKIP	1.342004264	1.56059E-07	0.985136596	7.47028E-18	1.166080157	8.03254E-25	0.502184091	0.691895802	0.079099062	0.789091143	-0.437610769	0.015363351
AT2G39705	RTFL8	1.284329888	0.000672435	0.505428471	0.24666383	1.164899279	0.000316901	0.435782203	0.619462504	0.657236261	0.01028372	-0.75688977	6.77022E-05
AT1G43580	AT1G43580	1.267336547	6.31089E-06	0.814245365	3.56887E-05	1.162095617	1.93504E-09	0.463129989	0.532231419	0.109587432	0.72834265	-0.042930552	0.859268475
AT4G32610	AT4G32610	1.231559361	0.000142302	0.70412741	0.000949029	1.160084235	2.31687E-14	0.352878902	0.647947756	0.15024555	0.606680463	-0.189804013	0.406486689
AT5G26980	SYP41	1.182851272	0.009086595	0.873313596	0.00242593	1.159352128	3.38493E-07	0.569267099	0.626508471	0.524923244	0.086698345	-0.031798494	0.902986568
AT5G52040	RS41	1.060164903	3.06476E-05	0.883090507	7.14374E-10	1.158971067	8.34024E-26	0.665171186	0.535142931	0.26953628	0.17086402	0.031586454	0.886316196
AT1G64230	UBC28	1.298313164	2.38444E-07	0.936374437	1.70193E-10	1.151465334	6.00872E-16	0.514236033	0.564319446	0.162383982	0.509573067	-0.216189116	0.075821684
AT5G44320	AT5G44320	1.243147389	4.4042E-06	0.591418147	0.00016232	1.146037995	3.12106E-16	0.343786191	0.706790319	0.110378353	0.645262033	-0.172783437	0.166570147
AT5G12330	LRP1	1.756544549	0.041962854	1.070652743	0.209137193	1.13849404	0.039073256	-1.272047697	0.275076337	0.383258937	0.404527441	-0.610831236	0.000106701
AT5G27860	AT5G27860	1.514542139	0.002445914	0.997806345	0.001777126	1.133115712	2.14076E-19	0.643414965	0.565040399	0.508577426	0.048520127	0.119336079	0.719900879
AT5G18310	AT5G18310	1.397887747	2.85676E-07	0.660905928	0.005439268	1.133006655	5.58694E-08	0.59922321	0.45375294	0.357714418	0.163590133	0.089934608	0.711515593
AT3G48185	AT3G48185	1.746194134	0.01190785	1.300903795	0.103792883	1.12768632	0.024824567	0.120131418	0.948636922	-0.176528705	0.819508138	-0.177344033	0.591050931
AT4G00238	AT4G00238	1.40975816	9.47371E-07	0.672283763	0.034268011	1.123043992	8.45663E-07	0.145441879	0.883652513	0.528773515	0.017767425	-0.331056679	0.069684523
AT4G09830	AT4G09830	1.497905254	5.06321E-10	0.696921461	4.83086E-05	1.118365737	4.53385E-10	0.297846068	0.773251874	-0.136765712	0.537666508	-0.262953379	0.046239771
AT5G51410	AT5G51410	1.003212512	0.000432572	0.934416706	2.89657E-07	1.112120064	4.90037E-09	0.976877161	0.330164829	0.22628352	0.423389572	-0.180834184	0.434986978
AT4G02005	AT4G02005	1.794025707	0.017116451	0.095379477	0.885628479	1.10832363	0.000891884	1.534418963	0.149725841	0.628895247	0.288680005	-0.310303544	0.347558345
AT5G64930	CPR5	1.189833807	0.000689864	0.828892855	0.000906616	1.10723684	9.44208E-13	0.591174756	0.691824202	0.038759676	0.949845179	-0.612387393	8.6333E-06
AT3G12050	AT3G12050	1.423016281	6.99393E-07	0.818845631	0.000716826	1.097487781	1.85391E-09	0.480876396	0.632278424	0.19796718	0.564401409	-0.191077189	0.471176327
AT1G76920	AT1G76920	1.029796513	0.003406551	0.713970546	4.07552E-05	1.096936038	3.01094E-12	0.100969475	0.939540455	0.146069139	0.641639	-0.416821435	0.005218315
AT2G18196	AT2G18196	1.320745897	0.003448316	0.61129194	0.106264445	1.092779534	0.000375256	0.028434404	0.991271129	-0.284314456	0.578542261	-0.874185794	8.09538E-08
AT1G76980	AT1G76980	1.292015694	0.028525504	0.657827076	0.50054717	1.087391223	0.020592732	-0.574301075	0.746270632	-0.337417064	0.587774995	-0.859303271	0.000262231
AT2G42740	RPL16A	1.019012758	0.00021887	0.123664714	0.753263514	1.085446706	9.2419E-11	0.210213016	0.720336606	0.480432704	0.019985311	0.416216617	0.006350047
AT3G09880	ATB' BETA	1.116015091	0.000519079	0.53399661	0.019307779	1.084579751	4.77731E-15	0.508539878	0.691824202	0.073692779	0.863949393	-0.452974134	0.001096218
AT2G02760	UBC2	1.001933257	0.000164038	0.806686826	3.75308E-05	1.083825295	2.48225E-13	0.353461912	0.727618493	0.134298935	0.556330533	-0.349629195	0.002700949
AT5G44860	AT5G44860	1.439077409	3.4954E-05	0.800581915	0.002162667	1.082512296	1.97187E-06	0.067141358	0.963509602	0.16349368	0.731476924	-0.383639316	0.03677538
AT2G37340	RS2Z33	1.000912476	1.73394E-06	0.58495102	0.000527475	1.078511627	1.16122E-25	0.442399234	0.602119156	0.215160488	0.367698844	0.262489449	0.029127425
AT5G63910	FCLY	1.156109416	2.49378E-05	0.757937924	0.00154575	1.069486991	5.10566E-06	0.036391147	0.979334259	-0.222424326	0.482865838	-0.380959416	0.001736889
AT3G54540	ABCF4	1.177804594	1.64485E-06	0.807563887	8.76707E-07	1.060014005	6.57813E-21	0.588558336	0.611321113	0.0545586	0.895976532	-0.069354219	0.662386124
AT3G48860	AT3G48860	1.024810735	0.00107112	0.831366427	0.000198315	1.057443795	2.07965E-05	0.227063397	0.886809278	0.160378574	0.632232464	-0.044348867	0.831007961
AT3G59800	AT3G59800	1.180365935	0.003838793	0.839154251	0.020684466	1.052538351	1.40604E-05	0.899984602	0.605164263	-0.323306747	0.57526134	0.022576191	0.962284666
AT1G48430	AT1G48430	1.326906651	2.78851E-05	0.729743256	2.05086E-06	1.051743303	6.45528E-07	0.95127965	0.373396563	0.133121964	0.706074374	0.31706845	0.04459263
AT4G17730	SYP23	1.595077978	2.0973E-11	0.939315138	2.41724E-11	1.049601374	3.4119E-12	0.336016249	0.62738272	0.339758977	0.045923958	-0.416282246	0.004011634
AT5G27990	AT5G27990	1.169072483	0.000518737	0.893105848	1.93035E-05	1.048608267	3.41275E-09	0.036325131	0.973864665	-0.028967047	0.952804023	-0.115502221	0.509347091
AT5G59950	AT5G59950	1.299231845	2.1419E-07	0.761198686	1.11614E-06	1.046648461	7.92479E-14	0.570172045	0.587695712	0.36311471	0.012645087	-0.212754166	0.210117907
AT5G19000	BPM1	1.286271055	0.000185128	0.848974299	6.5912E-05	1.04659991	6.25006E-12	0.376038882	0.714877681	0.261331079	0.297489043	-0.118962742	0.532604475
AT5G18065	AT5G18065	1.923081933	0.018426327	0.733372872	0.431419545	1.045285948	0.037029932	-0.473775536	0.718095285	0.347757367	0.640493482	-0.02147408	0.961926376
AT2G20120	COV1	1.130379196	0.0005515	0.649157714	6.42133E-06	1.043539425	1.15261E-07	0.58903103	0.613859108	0.040867548	0.917795278	0.098670996	0.573915735
AT1G43700	VIP1	1.154662263	0.00018874	0.934305978	2.03095E-06	1.042139856	1.49441E-16	0.488947396	0.682116008	-0.019192428	0.968882556	-0.568283905	0.000269413
AT1G74860	AT1G74860	1.088967797	0.006611865	0.768386654	0.0110217	1.039101058	0.000134412	0.226186908	0.847660958	0.165950483	0.809692025	-0.367803601	0.039475256
AT1G18773	AT1G18773	1.293942163	0.018880791	-0.204299231	0.697250744	1.038096755	8.31669E-05	0.648159654	0.612475768	0.475882254	0.53583656	-0.20363331	0.465070609
AT2G44410	AT2G44410	1.035461353	0.005423202	0.927624446	4.64788E-06	1.033335537	7.03363E-08	0.508137103	0.657950393	-0.02492831	0.963495178	0.113355853	0.519097607
AT1G05570	CALS1	1.395096004	3.24357E-06	0.988607757	0.000124704	1.022870104	7.51384E-05	-0.134775316	0.904735573	-0.309622586	0.246817246	-0.168568638	0.370267841
AT1G52740	HTA9	1.410041001	5.93772E-09	0.86406033	5.60886E-13	1.016166469	2.12277E-14	0.305208056	0.747061282	0.2659007	0.168575478	-0.030718619	0.894445048
AT5G28640	AN3	1.506754053	1.14604E-05	0.716402557	0.020044331	1.013330402	2.19612E-06	0.022783073	0.991243691	0.467755311	0.015126104	0.136916163	0.609363854
AT3G42150	AT3G42150	1.085379076	0.000830364	0.502919406	0.049680726	1.011783803	7.1956E-06	0.69320641	0.61896016	0.221436454	0.5094879	0.2608989	0.170973348
AT3G63310	BIL4	1.169859355	0.000124762	0.665972276	0.003103314	1.010136374	3.81956E-10	0.314332932	0.767112256	-0.393624662	0.091416969	-0.2053962	0.253770503
AT4G02715	AT4G02715	1.365136896	9.65967E-08	0.990816902	1.17648E-12	1.002153869	1.85391E-09	0.403723147	0.677620385	0.320118075	0.112283574	0.037489186	0.838875738
AT1G23870	TPS9	1.062866641	0.003031471	0.981166395	1.23666E-11	1.000622099	3.02758E-21	0.746837427	0.622850451	0.046806288	0.905220501	-0.265453039	0.109522448
AT2G17710	AT2G17710	1.468416177	0.005282564	0.213680572	0.709091962	-1.076062122	0.043682592	-0.518056064	0.794188083	-0.108740933	0.816135888	-0.252983532	0.009385385
AT2G18938	AT2G18938	4.620831072	0.000497057	2.379040966	0.546583075	-6.350319929	0.023712492	-0.656288015	0.788737401	0.818406466	0.573217541	-1.099037302	0.305383492
AT2G43050	ATPMEPCRD	1.988090725	0.004069965	-0.339835435	0.630179527	-2.004653522	3.42901E-09	-0.738377329	0.680482607	-0.229870872	0.560774678	-0.689101735	0.001196923
AT1G15960	NRAMP6	-1.205135827	0.01113833	0.010439638	0.987827125	2.388859457	5.43626E-10	0.811460947	0.674232756	0.742249777	0.20702246	0.856941349	0.001271342
AT5G17520	RCP1	-1.170704838	0.004309506	-0.312700343	0.599357886	-1.003501787	0.004440278	0.210358237	0.861051144	0.636945936	0.312067095	0.673282006	0.063194157
AT1G69390	MINE1	-1.168264559	8.22856E-05	-0.79082556	0.01031444	-1.004560702	1.32371E-05	0.718383757	0.498228536	0.172926411	0.742435213	0.400038172	0.023967852
AT1G17270	AT1G17270	-1.340869028	0.042835015	-0.802739781	0.413650984	-1.008513908	0.047754876	-0.316699543	0.887767455	-0.40065244	0.508067557	-0.116703442	0.722084549
AT3G49680	BCAT3	-1.538784573	2.45547E-06	-0.84723087	0.002876229	-1.008521299	2.40948E-07	0.213032145	0.820186281	-0.014224542	0.98143759	0.581488989	3.60353E-05
AT5G55710	TIC20-V	-1.465481917	2.23142E-06	-0.993866491	0.002063188	-1.008835622	4.52474E-05	0.40785162	0.726257149	-0.393926908	0.44947764	0.242899782	0.460023872
AT2G37940	AtIPCS2	-1.071814653	0.00012499	-0.763354836	0.010712713	-1.009774807	5.14973E-05	-0.573620075	0.345714534	-0.176986547	0.587713321	-0.039719226	0.832914053
AT1G26150	PERK10	-1.624372023	1.08948E-07	-0.745119245	1.11953E-05	-1.013066402	8.99605E-05	0.561170304	0.542342434	-0.027648777	0.963495178	0.483267262	0.002445831
AT3G29320	PHS1	-1.0411842	1.47209E-05	-0.844760958	0.001506372	-1.013082043	6.95028E-12	0.630962623	0.563668365	0.308138357	0.126218761	0.922742751	9.34268E-12
AT2G29510	AT2G29510	-1.079408322	0.000934922	-0.954651329	3.14508E-05	-1.01704314	6.77655E-07	0.061075436	0.962474985	-0.040685185	0.929498792	0.438785624	0.008936726
AT1G67840	CSK	-1.224971061	0.001514549	-0.749993925	0.000493303	-1.02260513	9.97618E-10	0.258764691	0.727618493	0.0630881	0.934318497	0.099116589	0.695826195
AT5G11610	AT5G11610	-1.256425006	0.023471096	-0.765205698	0.154320798	-1.024155608	0.005773917	1.551884035	0.598901706	-0.507031549	0.729103822	-0.326235647	0.708742759
AT1G03680	THM1	-1.469241814	2.96054E-06	-0.974721323	6.71961E-14	-1.024645206	3.98943E-23	1.213730337	0.389773786	0.279107216	0.505171247	0.520039676	0.014593012
AT2G41420	WIH2	-1.023201527	0.00752893	-0.7055635	2.74516E-05	-1.027165149	2.74567E-07	-0.4114402	0.611612407	-0.216257101	0.400086588	0.132516109	0.483828511
AT5G12860	DiT1	-1.252254252	2.10109E-11	-0.381456102	0.020928888	-1.032704373	2.23245E-08	-0.313900915	0.775188288	0.129626421	0.70708338	0.047064915	0.84664268
AT2G06050	OPR3	-1.159631465	0.000353536	-0.132753713	0.691570993	-1.035296726	0.001092426	-0.081375375	0.939540455	0.102621368	0.850188481	0.465681047	0.001278557
AT5G37360	AT5G37360	-1.279353263	0.002371944	-0.207026564	0.530699978	-1.0396511	0.0012403	0.611974569	0.663291724	0.177749576	0.801085181	-0.025033051	0.93972357
AT5G11450	PPD5	-1.193250936	1.28611E-06	-0.779029369	0.001756033	-1.041163454	2.20761E-12	0.355783503	0.697914029	0.36113777	0.153996968	0.786215517	5.77336E-08
AT5G56170	LLG1	-1.004507021	0.001584337	-0.621920857	0.005556764	-1.04377981	1.15149E-08	-0.547271373	0.62738272	-0.43036891	0.07338077	0.093434527	0.655257134
AT1G60950	FED A	-1.120971467	0.00279704	-0.713524711	4.23083E-08	-1.046048203	4.91922E-22	1.226927864	0.426758148	0.087865736	0.729469634	0.326098259	0.067243352
AT2G35500	SKL2	-1.370559563	4.97168E-06	-0.758390167	0.03557449	-1.049374821	1.19189E-08	0.184850694	0.922207233	0.034926005	0.962199154	0.826722711	6.46218E-10
AT1G47380	AT1G47380	-1.181230949	0.003005404	-0.674372482	0.228824716	-1.051887893	0.000169839	-0.830864948	0.615358268	0.091733423	0.88865708	0.041247202	0.861863413
AT3G26310	CYP71B35	-1.688131131	0.04280646	-0.917776179	0.143645952	-1.052084993	0.009156091	0.859237612	0.646324084	-0.482790863	0.711979287	0.099844194	0.860120387
AT1G04200	AT1G04200	-1.087029152	0.01397182	-0.932753621	0.01085037	-1.059043643	0.007200424	-0.265306868	0.822093569	-0.312161412	0.400218863	0.470898414	0.000480808
AT1G26090	AT1G26090	-1.676630617	0.009054181	-0.524697835	0.279334269	-1.061368352	0.013456476	-0.214715756	0.949776066	-0.147810376	0.895894402	0.59300612	0.023542968
AT3G25470	AT3G25470	-1.018403369	9.71253E-05	-0.587561843	0.071213734	-1.061495875	2.12147E-11	0.046799898	0.979951138	0.034412569	0.93904418	0.467382565	0.000414162
AT3G08505	AT3G08505	-1.649854442	0.005303866	-0.846054319	0.133389877	-1.062222331	0.012515676	-0.364791027	0.851805999	0.202749851	0.84792397	0.335313204	0.403249327
AT5G48370	AT5G48370	-1.206044585	0.044532357	-0.442206023	0.311461713	-1.063986726	0.001349972	-0.789954777	0.428836781	-0.091792447	0.932464417	0.163988953	0.62257975
AT5G63920	TOP3A	-1.548833967	0.033369825	-3.481659666	0.096849096	-1.069631964	0.008146578	-0.100575263	0.982356953	0.322684785	0.599487284	0.541300196	0.025581823
AT4G34190	1-9月	-1.376575286	4.82039E-08	-0.445109519	0.00819666	-1.0699331	7.2849E-14	0.212441944	0.799533905	0.526296559	0.058461349	0.533885496	0.000503551
AT1G17050	SPS2	-1.30842787	0.016494074	-0.221265288	0.679604274	-1.071738277	0.005282475	-0.032200738	0.993101238	0.112844144	0.918873929	-0.420357158	0.084002233
AT5G14320	EMB3137	-1.085472667	6.02338E-07	-0.861293641	7.95967E-05	-1.080301256	2.25416E-19	0.684477636	0.416170703	0.451074096	0.038074571	0.74971632	5.176E-08
AT2G21385	AT2G21385	-1.469818539	2.93937E-09	-0.679514618	0.079112066	-1.084771937	5.07207E-08	0.310607353	0.704470077	0.59692889	0.01922901	0.833107477	2.25232E-06
AT4G28740	AT4G28740	-1.513610565	0.000717452	-0.048758533	0.941736906	-1.088677337	2.95152E-05	-0.114400188	0.973896678	0.113219514	0.940884108	-0.093289357	0.84010635

AT2G44920	AT2G44920	-1.128278892	0.002347266	-0.889577275	4.97218E-07	-1.094934113	2.9711E-15	1.180760082	0.202198274	0.544425981	0.058132889	0.757640842	1.35645E-06
AT5G16710	DHAR3	-1.63653568	4.15774E-08	-0.766460388	0.000213298	-1.097247882	1.4848E-17	0.537707361	0.478067535	0.078309958	0.896222696	0.712490196	4.34446E-06
AT3G01120	MTO1	-1.220294859	0.002033235	-0.748656469	0.001063914	-1.097526572	4.24699E-15	-0.506799575	0.721140128	-0.067617183	0.846389857	0.142254786	0.411653018
AT4G28706	AT4G28706	-1.565741272	2.3991E-06	-0.620850004	0.012914769	-1.100791261	4.96193E-06	0.873425987	0.537732757	0.269699125	0.556988648	0.907799532	2.91987E-09
AT2G04700	AT2G04700	-1.041542124	4.239E-05	-0.7443373	4.73916E-06	-1.114327246	2.20711E-12	0.144299327	0.92165066	0.248319246	0.359502101	0.222856039	0.190919618
AT1G20640	AT1G20640	-1.503960816	0.001804395	-0.893145288	0.092467	-1.115006379	3.17946E-05	0.225355567	0.922363029	0.107884715	0.88362445	0.186666316	0.565940227
AT1G66840	PMI2	-1.147223533	0.046061377	-0.917452311	0.087782636	-1.123861954	0.016821563	0.049996207	0.990915497	0.588063336	0.561819089	0.267239928	0.593729953
AT2G25840	OVA4	-1.716322087	8.51338E-05	-0.715535611	0.050291654	-1.125721229	6.79138E-08	-0.246564754	0.874384556	0.35234508	0.309618466	0.668651481	7.4101E-05
AT3G57180	AT3G57180	-1.714015668	7.47981E-05	-1.969968894	0.437356899	-1.128853346	6.49982E-05	-0.409700629	0.79986937	-0.096194881	0.921848437	0.468239001	0.007644646
AT5G50280	EMB1006	-1.129159534	0.005003494	-0.92862282	0.031040822	-1.130009447	0.000176386	0.1982835	0.913152929	-0.026460872	0.973216634	0.251097277	0.285814104
AT1G61380	SD1-29	-1.262345918	0.023417644	-1.016392902	0.178368686	-1.130766065	0.013747653	-0.327459467	0.917008703	-0.599688302	0.233539745	-0.1935659	0.525542738
AT5G19370	AT5G19370	-1.211298112	0.001259895	-0.910277775	0.007726019	-1.133862138	1.53215E-09	-0.054974393	0.973566858	0.187107308	0.614546122	0.717987916	1.31966E-05
AT4G37310	CYP81H1	-2.241824778	8.42427E-10	-0.254244022	0.596596063	-1.138858551	0.000148197	0.010331893	0.996799452	0.409658998	0.500891025	0.834192735	5.47851E-05
AT3G26570	PHT2;1	-1.883124655	2.25309E-07	-0.785349761	1.3251E-07	-1.139928366	9.11951E-11	0.449533519	0.586831374	0.186527032	0.511188955	0.631519815	0.005782228
AT4G19100	PAM68	-1.347666499	0.024786491	-0.801835143	0.319740098	-1.143215525	0.000111667	0.008162511	0.997515054	0.539333797	0.512257495	0.484137499	0.034382054
AT5G52060	BAG1	-1.197734186	5.01238E-06	-0.770233398	0.001747273	-1.143355358	1.70902E-10	-0.790451985	0.602119156	-0.469902923	0.043902091	-0.2533477	0.145473718
AT5G08650	AT5G08650	-1.694709206	8.68251E-11	-0.952691547	9.78374E-07	-1.145650622	6.76245E-19	0.740858398	0.611321113	0.230151048	0.400204562	0.523171104	0.001066575
AT1G63610	AT1G63610	-1.831261928	1.42402E-08	-0.795735239	0.001108356	-1.148818538	1.3448E-08	0.129547337	0.945609936	-0.052436082	0.939586734	0.654294971	2.61798E-05
AT3G24530	AT3G24530	-1.258430327	0.009707055	-0.694982833	0.151255279	-1.149109755	0.00022022	-0.622589551	0.607117655	-0.491198629	0.10891184	0.232641648	0.326214991
AT1G09310	AT1G09310	-1.021016178	4.86886E-06	0.353891465	0.171572362	-1.152147873	8.04659E-18	-0.727326227	0.28860531	-0.520280619	4.10826E-05	-0.506661795	0.000580106
AT2G23170	GH3.3	-1.279189055	1.82525E-05	-0.905572631	0.073028721	-1.163256386	2.44972E-05	-1.072701196	0.214402425	0.409797131	0.189767942	0.923131545	1.31634E-08
AT4G02790	EMB3129	-1.227144797	0.002046179	-0.592494306	0.112471912	-1.164558187	7.58794E-10	0.131949897	0.93556951	0.367510359	0.318380163	0.748271872	1.89812E-06
AT3G63510	AT3G63510	-1.225097706	0.03025009	-0.653408354	0.104463239	-1.164702907	8.64157E-08	-0.187935925	0.882425056	-0.110601779	0.888839095	0.718237487	0.000203152
AT1G63970	ISPF	-1.500552477	1.78306E-05	-0.939094644	0.005066606	-1.165107953	5.43305E-06	-0.23961849	0.835453631	0.353642153	0.38803856	0.098442996	0.702965815
AT1G53430	AT1G53430	-1.057185921	0.001095233	-0.757240921	0.001711042	-1.168117348	2.99499E-14	-0.217011847	0.798808149	-0.480035098	0.04690642	0.051762754	0.82928832
AT3G26490	AT3G26490	-2.496635203	0.001031217	-1.263168385	0.128502705	-1.171602445	0.049259844	0.116528757	0.95447214	-0.31676573	0.778769276	0.461098815	0.164906448
AT3G17330	ECT6	-1.413209143	0.008874226	-0.86527935	0.031650094	-1.176260664	0.002796602	-0.719409809	0.726553713	-0.430059766	0.267111063	0.266455527	0.219392828
AT3G29360	UGD2	-1.019608972	0.003113492	-0.569965705	0.057288138	-1.178432558	3.61494E-10	-0.80721579	0.498301283	-0.353227067	0.042112855	0.310935327	0.055250953
AT5G45750	RABA1c	-1.166380134	4.92549E-06	-0.540118868	0.005589433	-1.186428398	4.95556E-10	-0.221137603	0.807092469	-0.156120456	0.71778863	0.531058159	0.000172326
AT3G17390	MTO3	-1.369166312	0.000596192	-0.922866238	0.233212084	-1.187271287	1.29133E-13	-0.887246415	0.413284704	-0.317199447	0.020351278	0.023379118	0.939860436
AT1G54500	AT1G54500	-1.186883009	5.11909E-05	-0.579329384	0.000162428	-1.192231435	1.55124E-08	-0.248576579	0.760764535	-0.156726109	0.719551702	0.038682778	0.876146783
AT1G04000	AT1G04000	-1.477025553	0.002011002	-1.09913534	0.148408422	-1.193066359	0.000114229	0.20149959	0.876000878	-0.377940575	0.455963467	0.121237506	0.718310107
AT4G34570	THY-2	-1.650964245	0.002613943	-0.857787819	0.2155141	-1.194238079	0.00023546	-0.184125302	0.952021801	0.418018694	0.451867216	0.436509718	0.029513439
AT2G27680	AT2G27680	-1.790883597	1.5536E-09	-0.702494985	0.016654929	-1.196398453	1.72388E-16	0.379868907	0.721140128	0.330165401	0.186000387	0.392932724	0.024006892
AT1G13250	GATL3	-1.582126645	5.2342E-05	-0.696083699	0.140979014	-1.198941746	0.000108654	-0.496315172	0.616300683	-0.404319719	0.326550255	0.236101151	0.31620871
AT3G62530	AT3G62530	-1.465947473	5.30288E-07	-0.837594329	0.001713425	-1.205032058	1.98263E-18	0.379980343	0.685495817	0.330125162	0.086684216	0.499089172	0.00051688
AT3G25040	AT3G25040	-1.386879156	0.043748603	0.029020141	0.969523989	-1.20825183	0.000152958	0.463016779	0.877177314	0.464848404	0.661503366	0.758333023	0.372463517
AT2G20570	GPRI1	-1.404505046	2.39634E-08	-0.863137996	0.00464873	-1.208942216	7.10307E-11	0.649395075	0.616806988	0.431097282	0.740033328	0.077185797	0.721734262
AT4G16265	NRPB9B	-2.36565945	0.010659577	-1.295470251	0.174151489	-1.210529909	0.001558535	-0.155536971	0.962474985	0.440193738	0.463584085	-0.00491424	0.988820398
AT5G10470	KAC1	-1.3168064	1.62522E-05	-0.574992713	0.024819465	-1.214191058	2.46479E-10	0.097091951	0.924803201	-0.24614836	0.629819822	0.074815443	0.781685589
AT4G30860	SDG4	-2.506728586	0.005878549	-2.120598363	0.179519171	-1.218326108	0.002970154	0.10174845	0.981591431	0.054012832	0.959723084	0.295023472	0.143983704
AT3G59970	MTHFR1	-1.518584228	7.63526E-05	-0.616131392	0.005406886	-1.219052678	1.40044E-14	-0.407483888	0.749317454	-0.273903164	0.106990472	0.691843368	4.2526E-05
AT5G24300	SS1	-1.63958173	1.88258E-11	-0.994405621	4.73484E-06	-1.219692536	5.05613E-21	0.432475371	0.576334999	0.09780461	0.809692025	0.221409963	0.158868349
AT4G04850	KEA3	-1.685797769	2.22641E-05	-0.736782573	0.086264084	-1.222704217	0.000216335	0.053995458	0.978783612	0.118773146	0.838708765	0.281710661	0.203617804
AT4G17300	NS1	-1.708856806	8.28849E-07	-0.660751805	0.058474749	-1.224656517	1.85457E-06	-0.192878288	0.909213799	-0.071816926	0.909436033	0.303718728	0.0746612
AT5G50110	AT5G50110	-1.76722909	0.037338905	0.243015767	0.682860979	-1.227732372	0.001418425	0.066617332	0.980517937	-0.005802784	0.997269331	0.238093486	0.382778077
AT3G60620	CDS5	-1.272896466	0.021361327	-0.495705763	0.139380177	-1.230420192	0.000509674	-0.376680133	0.859422872	-0.277347876	0.632768673	0.351492082	0.166964714
AT4G26540	AT4G26540	-1.624432039	0.000318594	-0.974736772	0.139224454	-1.23209185	0.001137077	-0.594386444	0.659255524	-0.260346117	0.63240298	-0.650356831	0.007117019
AT2G41680	NTRC	-2.124823628	2.13913E-15	-0.88545044	9.36895E-06	-1.234211885	1.71206E-11	0.06113701	0.963509602	-0.025341716	0.963963546	0.400688278	0.004817434
AT4G38020	AT4G38020	-2.250966847	7.3925E-07	-1.16088148	0.126736902	-1.23559622	0.00077556	0.091280088	0.966694285	0.376318128	0.588242002	0.740734442	0.011894322
AT5G30510	RPS1	-1.604744191	4.8601E-17	-0.892957385	9.58779E-06	-1.246017387	7.91155E-35	0.347110517	0.657374576	0.364578752	0.034795261	0.703283469	1.3412E-09
AT4G32915	AT4G32915	-1.176610892	0.001748521	-0.668317529	0.063480974	-1.250922364	3.5724E-05	0.227396092	0.820789421	-0.097467553	0.889007467	0.308363211	0.249350515
AT4G29060	emb2726	-1.236608243	1.96402E-10	-0.860723765	0.000272962	-1.255182907	1.3641E-21	0.210202137	0.774537456	0.27633566	0.120900727	0.284587678	0.010879932
AT5G38290	AT5G38290	-1.576250217	0.000245216	-0.894645075	0.176331093	-1.255801767	2.93271E-08	0.293192051	0.870759043	0.155401831	0.825786977	0.887796507	8.16535E-07
AT1G32900	GBSS1	-2.04595802	1.72923E-10	-0.789742229	0.001727063	-1.25842216	4.14522E-06	1.405067233	0.491082274	0.843908257	0.23426505	0.745416677	0.000807456
AT2G24860	AT2G24860	-1.253823995	0.032504388	-0.83315771	0.019634199	-1.258773826	1.24608E-05	0.196376302	0.897311543	0.000807094	0.999664963	0.129080935	0.761927143
AT3G19800	AT3G19800	-1.993474054	3.93455E-08	-0.8317078	0.00049823	-1.260179483	1.43253E-06	-0.03724932	0.991920965	0.535411537	0.401934539	0.715260182	0.008601086
AT2G30390	FC2	-1.384300449	1.59825E-06	-0.828727713	0.002575173	-1.265295766	3.41541E-15	0.291131728	0.72835818	0.227538823	0.563104532	0.668524999	7.22764E-07
AT2G21530	AT2G21530	-2.369594664	3.17973E-17	-0.952550824	1.94747E-05	-1.266331612	7.46287E-18	0.14089893	0.951514049	0.112617689	0.892756446	0.658604105	0.001304506
AT1G18040	CDKD1;3	-1.422724702	0.021665047	-1.315390381	0.220076386	-1.267091963	0.001114936	-0.029782842	0.990335664	-0.056304298	0.947746874	0.338020897	0.24377487
AT1G51100	CRR41	-1.230724945	0.007088307	-0.026147256	0.969706081	-1.268397624	0.017239861	0.460326914	0.706790319	0.169841461	0.843414743	0.207637369	0.447097079
AT3G45050	AT3G45050	-1.434417809	5.33809E-05	-0.706517108	0.167667503	-1.2690901	1.01197E-05	0.093418071	0.966082349	0.596730494	0.176850826	0.790203998	2.82391E-05
AT5G38980	AT5G38980	-1.425999315	0.000187968	-0.891423133	0.016184388	-1.270429248	2.20378E-07	0.711273738	0.289219283	0.281385923	0.53525964	0.958742002	2.06723E-06
AT3G55740	PROT2	-1.573565878	0.001091474	-0.195510961	0.689594595	-1.2741468	1.31743E-06	-0.727438467	0.305469701	0.296117044	0.624949045	-0.232938766	0.263617761
AT3G09210	PTAC13	-1.095691452	0.000180908	-0.524771678	0.003476282	-1.277921945	1.1295E-18	0.807589965	0.326457456	0.43029635	0.125191522	0.203573258	0.238968902
AT1G59990	EMB3108	-1.741816981	0.000737275	-0.868071182	0.059368389	-1.290220647	4.23758E-05	0.171757109	0.902675758	0.075810557	0.878467109	0.828527897	2.82078E-07
AT2G44650	CHL-CPN10	-1.295889719	4.10171E-06	-0.735470791	0.003578352	-1.290490348	7.42519E-14	0.267394155	0.727834308	0.236299547	0.484585201	0.606543254	1.84988E-05
AT3G54210	AT3G54210	-1.353986875	3.3117E-13	-0.955719449	9.56998E-06	-1.293915731	1.62726E-29	0.383678813	0.604415921	0.34087614	0.166489666	0.797603466	2.71202E-07
AT5G09650	PPa6	-1.800726654	7.76641E-05	-0.91213029	0.004848012	-1.294146238	3.16334E-13	0.134926139	0.966114207	0.272373791	0.305406977	0.848319618	1.01599E-10
AT5G51890	AT5G51890	-2.197887643	2.10109E-11	-0.948266947	0.007832375	-1.297513117	2.25929E-06	-0.215861017	0.905793887	-0.660541599	0.169512681	-0.414848984	0.218772756
AT5G06270	AT5G06270	-1.064485662	0.008671411	-0.806305778	0.222070958	-1.301482096	6.4612E-08	0.289100203	0.820845372	0.147341723	0.86306445	0.668573255	0.005648997
AT1G65920	AT1G65920	-1.495811543	0.021217107	-0.863372279	0.222412481	-1.302639455	0.002977977	-0.474317608	0.822056681	-0.127994673	0.839258237	0.479901043	0.009117497
AT3G55040	GSTL2	-2.005018653	1.5437E-11	-0.804532737	0.037131384	-1.303953504	3.92108E-12	0.789703719	0.511763779	-0.10819896	0.911846326	0.761949693	0.003000594
AT2G41990	AT2G41990	-3.955212768	9.60281E-22	-3.906077281	0.310899196	-1.307669128	0.00023041	0.445530964	0.89609717	-0.559892575	0.513381523	0.583576967	0.041434831
AT3G22890	APS1	-1.085907608	0.000277873	-0.787973376	0.000476184	-1.317270718	6.98161E-19	0.074993285	0.965260868	-0.163030412	0.583522802	0.497111491	7.30822E-06
AT2G39290	PGP1	-1.127506061	0.014697224	-0.806210087	0.008563785	-1.324346372	5.58941E-05	-0.299256038	0.721917053	0.189399979	0.667541358	0.143067866	0.591278442
AT1G04640	LIP2	-1.676253521	1.22394E-10	-0.552412086	0.069215575	-1.324764804	2.5123E-09	0.301522146	0.66924865	0.515102642	0.348299018	0.8990052	5.88344E-06
AT3G06730	TRX z	-1.083763056	0.006801629	-0.864311628	0.007653195	-1.32602071	1.67468E-05	-0.00223273	0.998880437	0.076833483	0.883566526	0.733883257	5.0885E-07
AT1G49380	AT1G49380	-1.156989498	0.001329765	-0.866426185	0.021305226	-1.329088653	2.72475E-14	0.536074302	0.721967244	0.691056295	0.019886905	0.434113786	0.049667474
AT1G63940	MDAR6	-1.295698443	0.024216935	-0.043363541	0.970817369	-1.329965705	2.1716E-17	-0.456900874	0.845608826	-0.190610647	0.400769075	0.295895431	0.023276998
AT5G17870	PSRP6	-1.182708018	1.07559E-05	-0.820608573	0.001214478	-1.33161681	1.96935E-33	0.475709576	0.320232025	0.231642352	0.48196212	0.951775239	1.18849E-07
AT3G61750	AT3G61750	-1.623652293	0.020005806	-0.661650775	0.347063931	-1.339933357	0.001692714	-0.059196461	0.984800288	-0.177180922	0.876125588	0.222964804	0.510784783
AT5G62840	AT5G62840	-1.328976176	0.010171026	-1.053375388	0.197107036	-1.342181099	3.65843E-05	-0.427660656	0.877636325	0.296763475	0.798358357	0.792278488	0.050276285
AT4G02530	AT4G02530	-1.328891706	1.96964E-06	-0.756861573	3.748E-06	-1.346749631	3.68338E-19	0.536238426	0.610292743	0.353647876	0.400729172	0.420165942	0.001852417
AT4G39800	MIPS1	-1.518841142	7.87529E-07	-0.712922514	1.60685E-08	-1.363581716	2.81241E-14	-0.364962283	0.688446852	-0.285758998	0.224579636	0.35151018	0.016737724
AT4G03400	DFL2	-1.421604732	0.01024058	-0.83895908	0.010403444	-1.366671939	0.001848103	-0.758868302	0.567190016	-0.172722633	0.84694947	-0.449531184	0.077290661
AT3G62570	AT3G62570	-2.373849501	4.7346E-05	-2.216719493	0.078464519	-1.367418504	0.042199148	-0.449565752	0.847175786	-0.12970649	0.884228042	0.487264911	0.070796502
AT1G75350	emb2184	-1.194197963	0.000194538	-0.974764325	3.99715E-06	-1.372214341	1.68875E-22	0.807816307	0.099695852	0.479343809	0.010735268	0.833246554	1.13462E-06
AT1G01630	AT1G01630	-1.127220865	0.001202271	-0.679703723	0.010256255	-1.372628902	8.50333E-09	-0.252097432	0.833592548	-0.235744689	0.469904353	-0.144041024	0.524394566
AT2G25830	AT2G25830	-1.505882251	2.77473E-05	-0.477224745	0.262911307	-1.375405697	4.10393E-07	-0.262483658	0.722776207	-0.129588443	0.826752121	0.526201494	0.00331225
AT1G26230	Cpn60beta4	-2.187270685	0.000413553	-0.739518601	0.199627524	-1.382069654	2.80984E-06	0.441607147	0.847546649	0.420644409	0.659920291	0.749030896	0.003564742
AT2G28000	CPN60A	-1.034444616	0.016298178	-0.450705283	0.218448918	-1.382440064	7.78688E-24	0.034541069	0.982749202	0.389800855	0.040669566	0.577965299	0.000428532
AT3G47650	AT3G47650	-1.536270627	6.56517E-13	-0.849911718	0.017167392	-1.384503567	1.44028E-15	0.224816992	0.790756089	0.22660142	0.488929906	0.399118062	0.016929411
AT1G33590	AT1G33590	-1.20314686	8.55261E-05	-0.83013056	2.46233E-06	-1.388674988	5.09993E-08	0.395963416	0.763885599	-0.547840371	0.005499128	0.986425811	4.17122E-09

AT3G54640	TSA1	-1.49891155	0.001186107	-0.358916753	0.522379261	-1.395662904	3.3871E-08	0.004444695	0.998026502	-0.380946941	0.315834547	0.156627715	0.417012057
AT5G63570	GSA1	-1.249483438	6.62895E-07	-0.893204866	0.009577735	-1.418168706	2.81825E-32	-0.085161501	0.950268917	0.188375339	0.491144609	0.655112843	5.86525E-09
AT4G17360	AT4G17360	-2.99792622	0.000675546	-1.404213821	0.104575813	-1.423645037	5.54305E-05	0.613216689	0.66808962	0.186187755	0.922870616	0.464902355	0.241226741
AT3G19620	AT3G19620	-3.648735255	0.003812911	-1.143505341	0.172982367	-1.431583841	0.005271301	-2.869988205	0.562656211	-1.539167679	0.404837745	-0.130615343	0.86116735
AT3G01980	AT3G01980	-1.376043791	0.003860509	-0.538153739	0.161675881	-1.43566617	5.31336E-05	0.204217787	0.895706838	-0.25052145	0.584384403	0.573784769	0.005385498
AT5G13510	EMB3136	-2.090842194	0.000181538	-0.973687569	0.023508194	-1.438426834	1.47325E-22	0.980718598	0.633171846	0.002588164	0.999129744	0.868034613	0.000260445
AT4G12910	scpl20	-1.853833329	0.001893393	-0.958364405	0.113294533	-1.451697294	0.00316683	-1.239372186	0.259297385	-0.401768589	0.5094879	-0.759166035	0.000922162
AT3G02380	COL2	-1.911791912	2.96524E-05	-0.724988494	0.025928678	-1.457032365	0.02753008	0.498483597	0.640704059	0.122881382	0.870887081	0.529863165	0.006913186
AT3G46970	PHS2	-1.533624453	1.2491E-08	-0.704676842	0.001776819	-1.459024406	1.77762E-12	0.517599224	0.282825999	0.368131694	0.188580093	0.702825208	0.000229272
AT1G21350	AT1G21350	-1.875933426	5.27805E-06	-0.876853906	0.002899984	-1.470338519	1.93122E-10	0.079172153	0.957032589	0.06140326	0.938703599	0.850386507	1.90234E-05
AT4G04750	AT4G04750	-3.637637508	0.004769789	-1.706813034	0.0984756	-1.485483185	0.030340499	0.209984068	0.980863668	0.284428771	0.919937283	0.929938781	0.001386669
AT4G27720	AT4G27720	-1.158613562	7.96684E-05	-0.508204722	0.066400629	-1.490400915	1.73629E-07	-1.337366833	0.319520931	-0.61790391	0.004630277	0.571296929	0.000551231
AT5G27560	AT5G27560	-1.883077242	6.13471E-07	-0.753287619	0.009735485	-1.495546266	1.11507E-19	0.789815512	0.326457456	0.010603503	0.98996705	0.566800563	0.000896964
AT4G18440	AT4G18440	-1.600403827	0.01237331	-0.863722862	0.014050402	-1.501052527	2.86361E-13	-0.033056101	0.990938961	-0.044436419	0.957156296	0.047112189	0.842774086
AT2G31110	AT2G31110	-2.096750234	2.09926E-06	-1.503159128	0.073533779	-1.509299244	0.022392736	0.007621845	0.997796217	-0.32319215	0.548823276	0.384619221	0.257132773
AT3G27060	TSO2	-1.308445687	0.023914247	0.511730419	0.132231933	-1.511397392	5.34271E-14	-0.45042789	0.828875452	-0.289004982	0.319847377	0.051534503	0.821231112
AT4G02850	AT4G02850	-1.374927652	0.036027243	-0.826395531	0.15019317	-1.512912058	0.002988553	0.543930308	0.714552244	0.040612116	0.983751188	-0.680158378	0.119623366
AT1G32500	ABCI7	-1.174711082	1.01218E-06	-0.400702898	0.270122555	-1.517542886	4.20484E-14	-0.071147577	0.967195835	-0.000758523	0.999364514	-0.062415443	0.800238759
AT3G54400	AT3G54400	-1.342383543	0.004884948	-1.060427692	0.287662317	-1.524346808	1.55881E-12	-1.012432831	0.548879103	-0.62973285	0.001203988	0.534276746	0.010160175
AT5G48310	AT5G48310	-2.704412892	0.012367431	-1.518149598	NA	-1.525494854	0.04333815	-0.060477042	0.987576531	-0.112108013	0.900609444	0.578902328	0.004072935
AT3G21320	AT3G21320	-2.528596134	0.003959325	-0.847522495	0.678135401	-1.536221372	5.10719E-05	-6.677007251	0.564319346	-1.016895169	0.723920667	-1.170097167	0.086513045
AT3G53560	AT3G53560	-1.46497059	3.43474E-06	-0.469080673	0.120030839	-1.536896992	1.83488E-13	0.32451964	0.659221802	0.268650739	0.5094879	0.58947622	0.000327729
AT5G66920	sks17	-1.247019035	0.00084968	-0.927495557	0.042249428	-1.55129757	8.76887E-14	-1.096026785	0.122649421	-0.862424848	7.8915E-06	-0.293507397	0.18242685
AT2G28500	LBD11	-1.643585154	0.008523315	-0.659078496	0.51088817	-1.561708347	0.029395659	-0.842330337	0.62738272	-0.163778991	0.899553662	0.801949773	0.004547621
AT2G45100	AT2G45100	-1.850775398	0.001167962	-0.711939215	0.329101819	-1.566948629	0.004619004	-0.363161198	0.809641478	-0.18991855	0.830492367	0.854945043	0.000134939
AT5G01770	RAPTOR2	-2.073620141	0.000628131	-1.605252127	0.067806078	-1.567462576	0.004407675	0.106862825	0.968655039	0.398216317	0.545081536	0.580469393	0.048620187
AT1G29460	AT1G29460	-2.377651981	0.000157965	-0.522234183	0.726090063	-1.568989878	0.027589781	-1.121310085	0.576080473	-0.082469189	0.963495178	-0.425881386	0.415871983
AT1G71880	SUC1	-1.345782135	0.015621322	-0.461236199	0.735355456	-1.580433988	0.025543107	-0.141550225	0.968021479	-0.00521219	0.994163341	0.23214934	0.174005358
AT3G20040	ATHXK4	-1.728165674	0.017650818	-2.619710275	0.067719416	-1.585775499	0.027778598	-0.182458632	0.93556951	0.094672456	0.94946668	-0.063715634	0.882180894
AT5G02760	AT5G02760	-2.144442342	3.14746E-14	-0.754268405	0.498025292	-1.603502119	6.60942E-22	-0.428662975	0.676109289	0.193525091	0.540774196	0.158110458	0.570044515
AT1G06620	AT1G06620	-1.432581199	0.020692544	-0.110739968	0.926669956	-1.603511325	0.044541533	-0.696378304	0.720626114	0.099225778	0.932299626	-0.667370757	0.0614364
AT5G07590	AT5G07590	-2.018886971	0.002619114	-0.76968505	0.0877112	-1.603894922	1.3773E-07	-0.331079033	0.91867804	-0.217192638	0.746294175	0.646550239	0.016970717
AT3G01960	AT3G01960	-1.729481001	0.040328605	-1.176461152	0.522427051	-1.60882195	0.002988189	0.120052831	0.989294835	0.259010398	0.913903461	-0.000144	0.999875041
AT3G04650	AT3G04650	-1.596679933	0.002596271	-0.458882165	0.474217385	-1.61146581	1.52213E-08	-0.094892128	0.971297857	-0.393734947	0.32411496	-0.034680959	0.912330402
AT5G17990	TRP1	-1.336986727	0.000541129	-0.230960957	0.577156671	-1.611908806	1.25214E-08	-0.031061991	0.990335664	0.344278356	0.338776976	0.61804016	0.000520813
AT1G62780	AT1G62780	-1.682665506	4.14232E-07	-0.687534667	0.166805289	-1.615083478	1.15853E-09	-0.267842961	0.88065104	0.234617766	0.63650345	0.660160759	4.67691E-06
AT5G54100	AT5G54100	-1.894974297	0.000212769	-0.726177141	0.450789895	-1.617184229	0.015130638	-0.605634737	0.697914029	-0.509164554	0.364511686	0.286120693	0.289064795
AT5G40160	EMB506	-1.330975572	1.06929E-05	-0.875495084	0.030217146	-1.620249592	1.61719E-21	0.491206022	0.695103554	-0.005406541	0.994940679	0.868294209	1.09535E-08
AT5G57270	AT5G57270	-1.214572071	0.009008095	-0.547335631	0.240733932	-1.622964363	3.13736E-08	-0.264022935	0.879193082	-0.360278398	0.440414957	-0.000322052	0.999263202
AT2G28605	AT2G28605	-1.198728461	0.001806505	-0.623360743	0.15255638	-1.627340234	4.26907E-12	0.588821683	0.657881954	0.360489297	0.563242017	0.517738548	0.039124732
AT5G53420	AT5G53420	-1.739156074	0.001467384	0.105001073	0.913084797	-1.631213087	0.000328716	-0.284710288	0.935083701	-0.127626613	0.862129805	0.00475844	0.988467984
AT4G21210	RP1	-1.763661638	5.49586E-14	-0.580925044	0.026746167	-1.640838095	6.74574E-14	0.28990941	0.751308246	-0.083651493	0.91048199	0.033569741	0.921077297
AT4G08590	ORTHL	-1.675942602	0.01938916	-2.017670741	0.236615887	-1.647542296	0.005108342	0.231991815	0.906972928	0.278309885	0.771935979	-0.009113584	0.98446865
AT3G43720	LTPG2	-1.418162045	0.000901994	-0.892076353	0.005674688	-1.666313552	6.8864E-13	-0.975937752	0.62053317	-0.265314915	0.407598637	-0.509715806	0.004945902
AT2G15290	TIC21	-1.40453627	0.000179742	-0.915606962	0.00015528	-1.668183061	2.49881E-26	-0.036092021	0.978807026	-0.167001423	0.645084436	0.431577196	0.000553513
AT3G45140	LOX2	-3.244513204	4.29854E-10	-0.287868568	0.646884706	-1.675662935	0.015736639	-1.188513136	0.645515722	0.890794443	0.47930267	-0.922958598	0.264427128
AT5G23510	AT5G23510	-1.102282917	0.013762388	-0.902317364	0.156613463	-1.699730152	7.29837E-07	0.060150005	0.97809523	0.103226824	0.923637745	-0.375099089	0.186031485
AT5G39210	CRR7	-1.95843215	0.000223396	-2.06823157	0.056565697	-1.701923712	2.81801E-08	-0.185031819	0.969068856	0.059846178	0.966653721	0.907504166	0.016138171
AT1G03310	DBE1	-1.765050009	1.45842E-07	-0.774176365	0.120030839	-1.705015727	5.2317E-11	0.037260952	0.985767196	0.455166454	0.265412977	0.335299848	0.067635517
AT5G50160	FRO8	-2.287477368	6.86881E-09	-1.366010328	0.052505801	-1.727139355	9.86482E-11	1.141876459	0.577205285	0.183792999	0.895021414	0.918978007	0.000246232
AT2G02590	AT2G02590	-1.674564531	0.007748175	-1.142998016	0.180631694	-1.731045032	0.000110013	0.372123832	0.895085848	0.29256061	0.812750957	0.571825345	0.060449769
AT5G44530	AT5G44530	-1.329876644	0.005222001	-0.421806002	0.379254784	-1.734690023	3.0866E-07	0.726632854	0.71459397	-0.518663301	0.4684032	0.892494303	0.002645909
AT1G14345	AT1G14345	-1.571750089	0.000151376	-0.86712846	0.045959197	-1.743898378	0.005421383	0.467140512	0.71455431	-0.475833899	0.35655851	0.334741184	0.213255347
AT1G69523	AT1G69523	-4.131990571	0.00062455	-2.265400318	0.566635546	-1.759351466	0.014992146	-1.234397883	0.926730546	0.227380283	0.957434731	0.96405983	0.669671849
AT5G24380	YSL2	-2.063131891	8.79552E-05	-1.02918289	0.256003473	-1.784990663	1.16945E-07	-1.158933125	0.354523081	-0.584476329	0.42891931	-0.977392745	0.000162284
AT3G20790	AT3G20790	-1.484997458	0.035608877	-0.763477419	0.053094035	-1.801334085	6.4863E-21	-0.296236089	0.907707664	0.642290778	0.006770981	0.665855131	1.18224E-06
AT2G24395	AT2G24395	-1.03138745	0.039185187	-1.480393526	0.112788976	-1.802293218	2.73015E-06	-0.610632534	0.66808962	0.083457222	0.934226173	-0.097900244	0.857036312
AT2G36870	XTH32	-1.645872828	0.032504388	-1.006982458	0.075102728	-1.808803325	6.20952E-07	-0.993694595	0.584219873	0.053248549	0.932299626	0.920834864	2.1258E-07
AT5G05730	ASA1	-1.246107525	0.002812187	-0.586980091	0.040874604	-1.814520703	4.6994E-24	-0.331997344	0.718989301	-0.37092238	0.269079164	0.198016863	0.260160974
AT4G34090	AT4G34090	-1.551723706	3.34916E-05	-0.9937961	0.005031368	-1.819453556	1.16099E-16	0.684654382	0.667180536	0.260048411	0.640493482	0.866741297	7.8159E-05
AT1G55490	CPN60B	-1.62697799	6.65884E-07	-0.946739818	0.001418386	-1.824426985	3.47552E-28	-0.051257411	0.980517937	0.324090063	0.081662368	0.856875673	3.65333E-10
AT2G32550	AT2G32550	-3.773511085	0.006578294	-0.986906863	0.608789463	-1.844262177	0.001043649	-1.602973346	0.840596718	-0.006564539	0.999500879	-1.334881502	0.209008157
AT5G06590	AT5G06590	-2.099594727	0.013433041	-3.376216059	0.123742495	-1.874357623	0.004799439	-0.195429189	0.979221735	0.192730636	0.864675247	0.141974147	0.733176778
AT1G15140	AT1G15140	-1.652049569	1.96934E-10	-0.951978197	0.002725211	-1.882820835	1.03459E-22	-0.122574326	0.951334464	-0.212053153	0.690120555	0.330907395	0.097783556
AT3G04550	AT3G04550	-1.722878985	1.51298E-08	-0.588033719	0.119234331	-1.896168688	1.90503E-12	0.071122557	0.965260868	-0.560949686	0.092965001	0.196807156	0.33720483
AT3G52380	CP33	-1.133034363	0.000175004	-0.407633663	0.194053282	-1.897544294	4.52716E-21	0.229578225	0.847660958	0.399835987	0.053023261	0.562412537	2.58144E-07
AT3G56290	AT3G56290	-1.779262206	0.006255429	-0.713409226	0.277259693	-1.922531037	1.07256E-05	0.113692151	0.971723999	0.16551176	0.954260932	-1.070979937	0.37186331
AT1G04040	AT1G04040	-1.533632494	0.007878567	-1.596401532	0.192046158	-1.934337615	8.19992E-21	-0.751675253	0.704225272	-0.53715999	0.085087943	-0.114050452	0.659099801
AT3G52940	FK	-1.295430436	0.003700494	-0.943784345	0.029022641	-1.934603868	1.02063E-06	-0.449672687	0.759702709	-0.376056639	0.323767852	0.187244191	0.469764742
AT3G25760	AOC1	-1.251623587	0.037559629	-0.703064251	0.215701909	-1.947894363	3.38801E-09	0.901309516	0.694819594	0.436767218	0.633717846	0.669564423	0.269401617
AT5G01790	AT5G01790	-2.319193873	0.002915929	-1.75353195	0.287416171	-1.948827683	0.005218649	-0.851492747	0.379538351	-0.220342631	0.864675247	-0.177682316	0.653449905
AT5G25460	DGR2	-1.112522209	0.007660717	-0.935029195	0.003909179	-1.972438836	3.18639E-36	-0.807743299	0.553691236	-0.229166643	0.158582747	-0.018936842	0.91133621
AT4G22910	FZR2	-2.092984015	1.41887E-05	-0.877996954	0.299233534	-1.97875815	6.48871E-05	-1.169478133	0.499936609	-0.332902217	0.542006423	0.268737654	0.48985135
AT5G41880	POLA3	-2.36084054	0.013998154	-2.208729818	0.114765499	-2.000312955	4.8414E-06	-0.594858418	0.674766805	0.16258769	0.822344596	0.262305236	0.424414266
AT2G33180	AT2G33180	-1.901251438	1.51789E-05	-0.932657216	0.011622177	-2.00553423	2.76285E-06	-0.530073501	0.890745298	0.072513918	0.936116842	0.349373596	0.288210517
AT4G20760	AT4G20760	-1.551311601	0.000480956	-1.030123239	0.11285956	-2.007957436	5.35427E-09	-0.039671898	0.991943214	0.107440694	0.929787432	0.282477534	0.319349348
AT1G18590	SOT17	-1.895428096	0.013733925	-0.26057843	0.833519665	-2.011906702	0.025528671	-0.029435461	0.996799452	-0.262312957	0.772601532	0.776779645	0.000198543
AT2G47010	AT2G47010	-2.015805144	0.021151177	-2.989618572	0.050097918	-2.014748021	0.000111517	-1.404315125	0.726900342	0.293277477	0.84790968	0.529239552	0.065456682
AT2G16630	AT2G16630	-2.261873018	0.010096142	-1.460996077	0.199627524	-2.026785116	0.000659586	-1.760428377	0.498460747	-0.762923414	0.039484958	-0.976335164	1.22083E-10
AT4G18375	AT4G18375	-1.700550081	0.013998154	-1.392882781	NA	-2.048357905	0.000150015	-0.142048734	0.953045007	0.514513259	0.524821373	0.578130487	0.020764807
AT2G23770	LYK4	-1.273500136	0.042415673	-0.904831729	0.09898054	-2.077958638	0.00023041	0.077134923	0.972495118	-0.556391164	0.499322416	0.375308048	0.309818731
AT4G13260	YUC2	-2.388137841	0.000499837	-2.503097385	0.067763297	-2.080549356	7.55406E-10	-1.946369198	0.247896754	0.332572019	0.856243901	-0.216635729	0.76995062
AT5G03760	ATCSLA09	-1.552195132	0.010012978	-1.025445294	0.080329023	-2.085370427	1.97621E-10	-1.184752664	0.622600949	-0.777265587	2.23709E-05	-0.081806116	0.663609016
AT2G28560	RAD51B	-2.306714816	0.035353778	-2.670678137	0.073783481	-2.097388446	0.001540635	-0.195001382	0.966211576	-0.47145405	0.835639362	0.100200647	0.851062215
AT5G48790	AT5G48790	-1.2755663	0.013958752	-0.840875338	0.000206708	-2.12431238	2.87361E-25	1.107882936	0.379838512	0.690885795	0.050954277	0.98692284	0.000178694
AT5G48540	AT5G48540	-2.451624864	0.000202297	-0.611413453	0.460011796	-2.134350019	0.009824515	-1.817961476	0.205816502	-0.52903485	0.41464716	0.804043735	0.019798159
AT3G03770	AT3G03770	-1.872608995	0.010133096	-0.713211438	0.824983671	-2.137688297	5.90627E-07	-0.793673558	0.874570817	-0.504681762	0.308252797	-0.111923976	0.753635308
AT2G38320	TBL34	-3.426298002	0.011438173	-0.612336575	0.784747957	-2.153571398	0.022454093	-0.006201353	0.999462317	-0.745574171	0.271977035	-0.118393839	0.830419747
AT4G05190	ATK5	-1.663969906	0.012858894	-3.273084677	0.132947093	-2.181686743	8.12657E-09	-0.36048592	0.926124658	0.055594899	0.943180766	0.620561982	0.000524398
AT4G29400	AT4G29400	-1.557340745	0.0173683	0.089492835	0.9255767	-2.187553361	7.5814E-11	-0.666276181	0.731041294	0.282610902	0.735365463	0.485770496	0.036317621
AT1G35560	AT1G35560	-1.152035257	0.044438872	-1.31422458	0.068885234	-2.203424143	1.94698E-05	0.302367284	0.893088744	0.092997203	0.933194561	-0.489690073	0.270479124
AT2G18328	RL4	-2.025205404	0.013849244	-0.275491264	0.832667476	-2.248815119	4.33159E-05	1.006462464	0.691895802	0.278664129	0.813650241	0.652462683	0.047562962
AT5G14760	AO	-2.157148079	0.018669133	-0.710443476	0.227930107	-2.271084673	1.27515E-08	-0.809654431	0.691824202	0.375944212	0.648350657	-0.342150943	0.289365185
AT4G01883	AT4G01883	-3.25639025	2.8577E-06	-0.776419924	0.096734307	-2.293739941	2.28482E-08	-0.271813839	0.884882727	-0.044899002	0.978424424	-0.035542713	0.938293102

AT3G55990	ESK1	-2.519774374	0.00234546	-1.764017279	0.143147486	-2.355421629	9.40966E-05	-2.030215161	0.107094826	-0.728113764	0.244822575	-0.623302144	0.003561054
AT1G75150	AT1G75150	-2.439877201	0.047456989	-1.633904622	0.066065522	-2.371906106	0.003626064	0.087827975	0.991149733	-0.118394648	0.927935348	-0.158463875	0.580293788
AT1G23340	AT1G23340	-2.181040672	0.035364783	-1.838742473	0.247282424	-2.412791749	5.86471E-09	-0.928102012	0.855081587	-0.068502718	0.964274323	0.210498169	0.567571222
AT5G18060	SAUR23	-2.166490758	0.00059544	-2.569789145	0.070033541	-2.479834953	8.45663E-07	-0.578973661	0.920450733	0.484922293	0.498449509	-0.191038033	0.549576525
AT2G30424	TCL2	-2.314208826	6.82841E-05	-1.560265982	NA	-2.581977164	5.45842E-09	0.15349432	0.972402645	-0.069485049	0.97211695	0.875082306	0.0285489
AT1G65490	AT1G65490	-4.496813849	1.06496E-11	-0.047357872	NA	-2.600850818	1.49009E-08	-3.879499977	0.731041294	1.812334014	0.845062586	-0.397665723	0.802292684
AT2G22330	CYP79B3	-1.032669895	0.047164669	-0.944477041	0.001083597	-2.617977964	1.66275E-30	0.252750315	0.901436908	-0.213423088	0.801344983	-0.363537653	0.030109392
AT5G55570	AT5G55570	-8.383213934	0.000159344	-5.75560313	0.278021734	-2.638757905	6.42909E-06					-0.61235869	0.761927143
AT1G03055	D27	-3.415123575	0.033442676	-1.586054935	0.593965144	-2.699476245	0.038757494	3.661012601	NA			-5.595888515	0.052845251
AT4G07960	CSLC12	-2.300846388	0.010351858	-3.341720136	0.081603841	-2.705583104	0.002357566	-1.236045914	0.521393002	-0.072076803	0.962799626	0.717039336	0.006473502
AT4G14400	ACD6	-4.640238902	0.006225707	2.44480379	0.259544897	-2.721089452	0.016363666	-3.493134469	0.664635521	6.205735795	0.188884212	-0.722045986	0.624479415
AT5G57220	CYP81F2	-1.718739328	0.0042018	-0.779436472	0.029890658	-2.759887388	0.00047161	0.114036675	0.982529985	-1.223054927	0.219497123	0.292803652	0.569262556
AT5G10570	AT5G10570	-2.511025667	4.44413E-08	-2.334352051	0.317661048	-2.799980718	1.0369E-06	0.884762927	0.665832077	0.508546625	0.710881196	0.365100663	0.520301157
AT5G18030	AT5G18030	-2.08381376	0.000122732	-1.427893241	0.457566389	-2.853726787	7.17349E-16	-0.78047007	0.787044709	0.003127052	0.998561976	-0.218748779	0.516832204
AT1G26560	BGLU40	-2.056975672	0.006939565	-0.95047859	0.309322591	-2.87530529	5.6614E-09	-0.894672836	0.847546649	-0.698550034	0.033365224	-0.483713435	0.005685286
AT5G63060	AT5G63060	-3.620931263	1.0135E-06	-1.316820614	0.131626815	-2.896185641	6.30233E-09	0.215829076	0.977839623	-0.091670245	0.965212969	0.408177154	0.431225844
AT1G54385	AT1G54385	-1.765972098	0.019689964	-2.187464615	0.142368505	-2.953644894	9.01866E-07	0.124316683	0.968504529	-0.058811189	0.958669515	0.459916608	0.054686644
AT1G04800	AT1G04800	-1.271810856	0.046974052	-0.041099699	NA	-3.013006982	1.84468E-08	1.042261189	0.66524984	0.666723717	0.216118636	1.030976597	0.244380669
AT3G10570	CYP77A6	-1.923683301	0.026350887	-1.241817563	0.431141963	-3.040499194	0.000124375	-0.355356315	0.96432609	-0.014799994	0.996289463	0.009111603	0.991481208
AT1G29450	AT1G29450	-2.095938589	0.000524746	-0.674729496	0.742141724	-3.049914827	0.000797061	-0.032522159	0.993903763	-0.076970582	0.962807909	-0.636275943	0.239617906
AT5G10170	MIPS3	-1.756385263	0.014791507	-1.2041487	NA	-3.406225789	1.09885E-21	-0.671165533	0.795048376	-0.442715874	0.464148269	-0.550975241	0.018978862
AT5G03130	AT5G03130	-8.258606678	0.000295024	-6.509882982	0.054136445	-3.617542469	0.021189812					2.451749531	0.710015308
AT5G51720	NEET	-3.815243798	1.34241E-11	-2.529470479	0.280326586	-3.835238765	0.007117706	-1.065577845	0.755333613	0.949175345	0.53583656	0.241475925	0.7929073
AT1G65870	AT1G65870	-8.181134157	0.000213058	-6.519995352	0.150669769	-3.850550216	0.006424786	3.182879942	0.741727789	6.346132687	0.233594737	0.121532719	0.910866066
AT1G49830	AT1G49830	-5.505087487	0.015627762	-1.313029202	0.322152025	-3.92580811	0.027569828	-0.454464008	0.95447214	0.199080186	0.968001113	1.285836389	0.089644755
AT1G13650	AT1G13650	-11.2828525	2.98618E-13	-3.755059458	0.053942138	-3.991968899	3.48242E-05	4.200333411	0.709586436	5.878756286	0.447017804	-0.664358275	0.780581324
AT4G39950	CYP79B2	-1.676177609	0.007748175	-0.382893028	0.590313805	-4.126255792	1.19567E-10	-0.647099219	0.637815859	-0.844421475	0.010534029	-0.505796988	0.000630515
AT4G39940	AKN2	-2.16140865	0.000179778	-0.285801943	0.653250674	-4.135002293	7.32847E-23	0.03700897	0.989667011	0.007790487	0.995134383	0.357349193	0.039256796
AT1G52290	PERK15	-3.647488465	1.76105E-08	-3.573629983	0.097490743	-4.43315143	0.006774062	-0.335089104	0.955538427	-0.510696484	0.323767852	0.214570518	0.685312586
AT2G15020	AT2G15020	-9.232829061	1.39239E-06	-3.17782419	0.116568761	-4.481631064	0.00264739	-4.61151454	0.685495817			-5.765580927	0.087287033
AT3G14210	ESM1	-1.74172029	2.02077E-05	0.34433259	0.821496623	-4.776466396	4.206E-47	0.083409934	0.968175076	-0.0828096	0.962421525	1.306948452	0.577660508
AT1G04110	SDD1	-4.949869234	2.5388E-06	-1.856239078	0.28634519	-4.954327015	8.6774E-05	-0.11088766	0.989481773	-0.928296495	0.638629847	1.02977994	0.074050036
AT2G46450	CNGC12	-1.154368278	0.00485019	-0.512979618	0.3031959	-5.156175067	0.00539038	0.471077187	0.567190016	0.281430654	0.518256458	1.129397228	0.245867841
AT3G18660	PGSIP1	-4.073730223	0.001606482	-0.790331366	0.457199967	-5.404189152	1.06386E-05	-1.083985416	0.656918679	-0.647934775	0.564561434	0.165595263	0.813653513
AT4G23200	CRK12	-4.281384811	0.020677566	-3.350013771	0.343954931	-5.61890493	5.28564E-07	0.23012905	0.990938961	1.325341796	0.872528185	1.404422164	0.799033011
AT4G34510	KCS17	-8.674726487	2.5397E-05	-0.162080946	0.951724881	-5.767590628	0.020705454	1.351536487	0.801691587	0.874565771	0.629318502	0.827500746	0.191898471
AT1G52040	MBP1	-7.39964854	0.049391728	-2.361314555	0.336701039	-6.371758207	0.024579211	0.311557819	0.967737794	2.446366711	0.077868942	0.713973549	0.028675355
AT5G37420	AT5G37420	-7.525919169	0.008557413	-2.411384985	0.395076621	-6.678564424	0.013298393	5.69183886	0.633628949	5.720724666	0.407412033	-0.228473746	0.901004011
AT4G14650	AT4G14650	-7.600545529	0.006451662	-0.286945609	0.94564379	-6.951157385	0.007974086	-1.684660145	0.676109289	0.289876663	0.851437944	-0.477118997	0.178495085
AT1G12030	AT1G12030	-7.09671205	0.020218639	-3.144852759	NA	-7.335307289	0.000550024	-3.873481431	0.731041294	2.669601638	0.619871203	-0.822926419	0.70479722
AT2G37900	AT2G37900	-7.2030153	0.000717452	-1.458648015	0.56208598	-7.483095088	0.000251291	0.084996376	0.991559366	0.854875935	0.63650345	1.06489787	0.057060602
AT4G22880	LDOX	-5.060825291	0.043061747	-5.796171402	0.204700468	-7.516245205	0.000887552					2.751146087	0.604318632
AT3G18900	AT3G18900	-8.950345414	2.19555E-05	-0.615762208	0.78547714	-7.744576539	0.000174479	0.349150772	0.964175862	-1.173804333	0.199674929	0.191198126	0.849076526
AT4G00970	CRK41	-2.800987676	9.83909E-05	-1.076585644	0.245627465	-8.772716899	6.84715E-08	0.99302963	0.880948584	-0.363315379	0.955523511	0.874946023	0.673129186
AT1G65060	4CL3	-6.375623399	0.000710016	-1.524506116	0.452938547	-8.815054633	2.10286E-07	-1.560650244	0.820789421	-0.496107498	0.853649569	0.583549086	0.430399703
AT5G54720	AT5G54720	-4.564268449	6.50433E-05	-1.962997317	0.638471032	-8.8598757	4.07844E-08	-1.33724935	0.764587933	-2.35773397	0.390414646	0.8832564	0.500417545
AT2G37100	AT2G37100	-4.392059672	0.019639335	-2.729112192	0.076722627	-9.311551215	8.94541E-10	-1.007938877	0.827927269	-0.247973473	0.899175176	0.758671271	0.088591649
AT2G48140	EDA4	4.204650145	0.021357333	-0.292215112	0.907513104	-1.007768232	0.667480426	-2.225398804	0.021051627	-1.377610679	0.022433723	-1.517250572	1.6025E-09
AT2G29290	AT2G29290	-1.96139555	3.7314E-05	-0.895363923	0.00263606	-0.142156907	0.519834439	3.049120486	0.034661846	1.255348971	0.001020639	1.565913726	0.000283233
AT3G14940	PPC3	1.350341139	0.000993607	1.050783	0.069606784	0.768672614	0.074344622	-1.276977272	0.044081353	-1.415037222	1.73061E-08	-0.670720756	0.001932008
AT3G22235	AT3G22235	-2.235583238	0.01332687	-1.783857045	0.059272397	1.034377969	0.849987443	9.684346436	7.12871E-07	3.888695728	0.000574492	-0.357990528	0.918540608
AT3G26460	AT3G26460	5.414175449	0.005467236	0.530944575	0.85073826	-1.61479772	0.37810977	-3.121177	0.003032424	0.380794674	0.822809738	-2.69882502	9.02189E-33
AT3G28360	ABCB16	4.989696329	0.012802394			-1.152531012	0.640069008	-9.567983234	2.67483E-05	0.274173777	0.957156296	-4.614208557	0.001858195
AT1G78340	GSTU22	3.251193954	0.012317254	0.247904839	0.923036393	-1.132962291	0.586147636	-2.700206983	0.013621408	-0.676592656	0.239929079	-2.633239871	0.005190655
AT3G02850	SKOR	2.834047146	0.001580083	-1.262558275	0.542003972	-1.677094616	0.050098228	-2.426500249	0.002516721	-1.746084688	0.172208668	-2.064372799	1.45172E-07
AT3G06390	AT3G06390	2.633605149	1.66103E-05	-0.457341553	0.736335478	-0.260606917	0.894311921	-2.02029447	0.026846356	-0.438758498	0.462927792	-2.220666855	1.22652E-49
AT2G20260	PSAE-2	-1.042581326	0.015093145	-0.689458596	3.33977E-05	-0.365325523	0.012968468	2.680984947	0.017266958	0.801159406	0.158543269	1.112369479	2.16542E-25
AT1G29930	CAB1	-1.189074521	0.003607217	-0.96927514	1.00985E-07	-0.536520608	1.89114E-07	3.900231967	0.000808898	1.285910658	0.214036947	2.126075965	2.29678E-55
AT5G43570	AT5G43570	8.54761413	8.7109E-05	1.585926554	0.798846412			-5.830703016	0.022675761	0.027845454	0.998079347		
AT5G41570	WRKY24	7.928550269	0.003332527	-4.984338097	0.355127868			-8.353424067	0.001277004	-0.543407618	0.899553662	-1.243268684	0.160110944
AT5G11290	AT5G11290	7.357221499	0.01308707	2.12989913	0.330273274			-8.063534093	0.017266958	-1.066314492	0.607875274		
AT4G16990	RLM3	-1.058713399	0.018814102	-0.764701628	3.4532E-05			2.193618976	0.022940238	0.515370645	0.086779373		
AT3G14420	GOX1	-1.086646021	0.008793087	-0.348818006	0.01407593	0.228981969	0.095220229	2.825007072	0.046799286	0.160544657	0.632350312	0.169567925	0.41724
AT3G61470	LHCA2	-1.416918477	0.000537091	-0.90414722	8.54858E-08	-0.854195332	6.12344E-23	2.71389188	0.030255469	0.621631758	0.003688639	0.958201183	4.68404E-11
AT5G15160	BNQ2	-1.57371229	0.018730026	-0.839192046	0.135925927	0.160211282	0.729844661	2.909619324	0.003309303	0.853883345	0.500990625	0.89754742	0.038065065
AT3G20840	PLT1	6.870245943	0.048915672	-5.77920289	0.27579039	-6.200891888	0.191245949	0.276202622	0.971297857	2.422483941	0.04094721	-2.337407707	0.000973717
AT5G64120	AT5G64120	1.212808434	0.001941122	1.720732915	0.289221842	0.733914397	0.331642114	-0.672123216	0.774188277	-1.407818447	0.00339994	1.215835487	2.55542E-11
AT2G18370	AT2G18370	3.29722082	0.030158861	-0.269732063	0.950818885	1.608290524	0.781007005	-2.006663773	0.548879103	-1.207879378	0.00875737	-1.033051483	4.45199E-06
AT5G48657	AT5G48657	7.468345916	1.34296E-05	2.274566643	0.232189406	-0.385478024	0.934985662	-1.42751983	0.717709314	-2.292066594	0.014385114	-1.227464603	0.049317277
AT2G38380	AT2G38380	2.788585407	0.002377978	-1.498360137	0.147728837	0.482350613	0.839052823	-2.046623303	0.380380211	-1.390561021	1.00504E-05	-1.375092397	1.09452E-13
AT2G37130	AT2G37130	3.906336125	0.000137716	0.42601372	0.164251342	1.318927362	NA	-0.453514203	0.84196754	-1.054089273	0.001732895	-1.426741104	5.79456E-36
AT5G64100	AT5G64100	2.175946862	6.81235E-07	-0.932386949	0.444214724	0.096877541	0.942630597	-2.113940525	0.377008424	-1.770162004	9.98593E-12	-1.500276081	8.10326E-19
AT1G05700	AT1G05700	8.020170113	0.01726911	0.378065999	0.951357175	-5.910589783	0.156691827	-3.303670465	0.568009156	-1.819985787	0.020547537	-1.553315665	0.038450921
AT2G28960	AT2G28960	8.556313385	5.64915E-05	-1.32802342	0.697293368	-6.054497388	0.069843345	-2.816460555	0.477936046	-1.956314663	0.004347678	-1.610088255	0.000377635
AT3G22800	AT3G22800	8.367685489	0.000170828	-1.022261381	0.850441839	-5.479371957	0.298691805	-1.094576577	0.798808149	-3.207362979	7.8915E-06	-1.636858071	3.9239E-05
AT3G32980	AT3G32980	2.055760773	6.01031E-12	0.422598158	0.325436449	0.225670449	0.79780856	-1.49265744	0.086419025	-1.223184726	1.59838E-13	-1.64287098	5.11904E-65
AT2G44380	AT2G44380	2.21662137	0.000750413	-0.36349127	0.887223034	-3.983672221	0.466753727	-0.226128657	0.962872831	-1.981959934	2.57716E-05	-1.685549225	0.002326397
AT1G13590	PSK1	4.559565908	0.028883107	4.036201293	0.244589059	0.616757669	0.906415937	-0.852729576	0.867643421	-1.534080227	0.042444463	-1.812616134	0.014456807
AT2G46750	GulLO2	3.535539299	7.9296E-07	-1.723514722	0.056430926	-0.884771031	0.743855924	-1.687617545	0.386146892	-2.371995198	0.000693656	-1.823124448	4.99674E-07
AT3G54040	AT3G54040	3.26618828	1.60649E-07	0.584655502	0.623202641	-1.353974589	0.410194004	-1.930728864	0.084017009	-1.516141783	3.42747E-09	-1.840857054	4.6151E-12
AT1G61050	AT1G61050	8.122066783	0.001485049	-6.509419296	0.213745976	3.408375782	0.459472033	-0.846445626	0.88581519	-2.497401449	0.002301529	-1.867883867	0.000179285
AT4G25090	AT4G25090	8.305548603	0.0003256	-1.806547511	0.598677598	-2.130721143	0.284827995	-2.055859066	0.610227484	-2.037582361	1.23561E-05	-1.890850282	1.32514E-07
AT5G44380	AT5G44380	2.905596362	8.13711E-08	0.118087269	0.911208777	0.839551912	0.48734869	-1.562849452	0.565728752	-1.563058202	2.04314E-11	-1.906111948	5.15833E-16
AT5G22890	AT5G22890	1.491886767	0.012173536	-0.834682118	0.299074998	-1.03356495	0.191509282	-0.899807988	0.741732129	-1.051573758	0.020351278	-1.977558487	1.95096E-11
AT3G53480	ABCG37	2.559453335	1.03964E-09	-0.600984153	0.3085224	-0.021139778	0.987093797	-1.162494965	0.489831977	-1.458750956	5.90459E-12	-2.063210106	9.44775E-21
AT4G15390	AT4G15390	4.436853413	0.006465318	0.331747925	0.855355791	1.165515606	NA	-1.748938578	0.606294014	-2.614492167	1.37882E-12	-2.167425491	8.30505E-46
AT1G72200	AT1G72200	1.74040902	0.044563389	1.159663922	0.172982367	1.500071761	0.25379172	-0.680118645	0.697407195	-1.034706195	0.005790177	-2.176969106	4.31279E-11
AT1G30700	AT1G30700	2.275113543	0.010379899	0.61863122	0.71625331	-3.570955751	0.08691624	-2.089546443	0.11301143	-2.551969419	4.72023E-05	-2.181878406	9.45836E-05
AT3G14680	CYP72A14	4.253005875	8.75099E-05	1.027851742	0.090956451	1.692415297	0.191094565	-0.694584078	0.468917108	-1.491275441	0.00076913	-2.183387274	1.92951E-08
AT5G09530	PELPK1	1.957572025	0.002964122	-0.883835126	0.436567271	-0.568608704	0.515138609	-2.956257403	0.535600467	-1.215619379	0.000578853	-2.262490597	8.71448E-83
AT2G44010	AT2G44010	2.339559559	0.025424766	-1.876816471	0.368551611	-1.311940434	0.512519685	-1.076938119	0.667824887	-1.223283118	0.037137651	-2.369542845	5.18336E-18
AT2G22510	AT2G22510	1.751537262	0.031301618	-2.688606315	0.071157862	-0.171571198	0.96198573	-2.209936106	0.691895802	-1.114480481	0.010617052	-2.428830691	3.14425E-48
AT3G04060	NAC046	2.155271475	0.008419532	1.076972211	0.304758051	1.217769146	0.125827581	-0.795040884	0.733019473	-1.499175426	0.030288046	-2.432799338	4.13598E-06
AT5G23220	NIC3	9.898621163	1.41751E-06	1.842116536	0.199907996	2.057644168	0.589246793	-1.399730907	0.255573307	-1.107804747	0.00514006	-2.661199118	3.00316E-16

AT5G62480	GSTU9	3.192653279	0.019793492	4.643198152	NA	1.141866295	0.387264961	-3.452842906	0.189012735	-2.029449341	0.000178235	-2.744236056	2.1869E-07
AT5G43350	PHT1;1	2.718710319	0.03025009	-0.282797306	0.821496623	0.016288604	0.993348353	-1.6104893	0.417861924	-1.873368813	4.73042E-09	-2.764406547	1.00375E-30
AT3G01420	DOX1	3.700657087	2.91009E-13	0.196597187	0.718897972	-0.916011524	0.63268579	-2.210313063	0.172638291	-1.248267754	8.01697E-06	-2.984799048	1.19004E-41
AT4G15380	CYP705A4	8.757099198	1.58398E-05	-1.167769283	0.69328171	2.403035696	0.518322499	-2.326988111	0.602119156	-1.80095578	0.014502397	-3.233372946	5.34785E-09
AT3G13435	AT3G13435	8.248113272	0.000583851	-6.377569208	0.088447994	-3.374634797	0.544074387	-1.19367365	0.794188368	-2.007722023	0.001900498	-3.305502566	1.42618E-05
AT1G51860	AT1G51860	5.280107065	0.01134938	2.729579972	0.292373264	-0.061442481	0.98510866	-1.830511375	NA	-2.195075696	0.002359244	-3.555936131	1.98552E-06
AT1G07560	AT1G07560	8.093274114	0.000835436	-0.452354621	0.907826886	1.040805341	0.768555902	-2.249093318	0.602123416	-2.263045234	0.024803317	-3.573664714	0.025000409
AT5G03570	IREG2	7.587016834	0.00580389	1.368502741	0.829447911	-3.99997266	0.464644883	-5.107802182	0.06577519	-2.848758848	0.008003167	-5.562445655	2.63902E-08
AT5G55250	IAMT1	-1.617896694	0.030258282	-0.53840788	0.290507004	-0.709907162	0.030271654	1.565849707	0.48447674	2.580993519	0.007865792	1.071738257	0.038792914
AT3G55980	SZF1	-1.042350416	0.025028547	-0.976778586	3.86345E-06	-1.043378799	0.059311876	1.262751894	0.420106526	-1.215445921	6.83384E-12	2.802978979	7.5275E-106
AT4G01950	GPAT3	-1.557985386	0.000734741	-0.512780037	0.228611944	-0.225095426	0.852033036	0.174133926	0.903381971	-1.899397572	1.52397E-28	1.237139876	1.69814E-24
AT5G42250	AT5G42250	-1.378668273	0.006462731	-0.961945042	0.035260545	-2.969559705	0.116582232	-0.436197191	0.726214276	-1.204376307	0.033395254	-2.606398352	5.88304E-10
AT1G02310	MAN1	7.558912198	0.008095455	2.42999304	0.551558129	1.848657505	0.731195446	-6.937331186	0.228732299	7.060976446	0.027212433	-0.394446026	0.786091796
AT5G57123	AT5G57123	9.238193331	0.000964952	-3.553772892	NA	6.255472822	0.165720882	-0.669556747	0.912371649	1.650727656	0.020395623	-0.475946777	0.25693805
AT5G44572	AT5G44572	1.064327789	0.017013666	-0.102423707	0.82577943	2.148871011	0.649858806	1.349681754	0.10756991	1.039959209	0.01149978	-1.080789972	0.878292965
AT3G13677	AT3G13677	1.290097625	0.015214986	0.042977018	0.958397157	0.025084213	0.96097311	-0.023914026	0.992411026	1.004693214	0.024638847	0.141238787	0.75209065
AT3G23800	SBP3	2.701300505	0.038677385	0.849502037	0.734268289	2.539353602	0.18336844	-1.807920237	0.747920884	-1.056787756	6.99455E-06	-0.843896826	5.43358E-06
AT4G11290	AT4G11290	3.564258608	0.000123488	-0.495818195	0.702985603	0.753591773	0.426091005	-0.918382514	0.684184988	-1.220592947	4.76103E-10	-0.739995094	1.17754E-12
AT4G07820	AT4G07820	3.147527461	0.002696136	1.132200283	0.69332934	-2.242707603	NA	-3.389096952	0.182876684	-1.518964851	0.000395037	-0.162802684	0.69851789
AT3G26470	AT3G26470	3.146831183	0.000326332	1.258286267	0.256123751	0.755765202	0.90084865	-1.014506406	0.567190016	-1.578470501	0.000750618	-0.873800296	0.001927628
AT4G30170	AT4G30170	5.130769744	0.033638795	-0.699881677	0.49026372	4.940403669	NA	-0.622861235	0.810393493	-1.761884438	0.011141694	0.094493895	0.633459467
AT4G08770	Prx37	9.77791382	5.0852E-07	0.627546031	0.84810514	1.00941512	0.383201428	0.26302572	0.929450746	-1.762967762	0.012030989	0.191662275	0.219676107
AT1G49570	AT1G49570	9.499161195	2.52398E-07	5.594496733	0.120900444	-4.498825745	0.406088392	-0.160403618	0.982183273	-1.871404231	0.029145664	-0.376811843	0.610972275
AT1G67148	AT1G67148	7.391084872	0.011859934	-0.32853236	0.961158262	0.543713908	0.659797046	-1.812828419	0.648262785	-1.901771454	0.026146688	-0.921632743	7.66235E-05
AT1G21310	EXT3	2.025063691	0.019816185	1.437006161	NA	0.345271048	0.631904616	-1.339749924	0.532959875	-2.067666425	3.65965E-07	-0.446156228	0.047073909
AT1G64160	DIR5	7.392808313	0.013101579	-4.591953932	NA	-5.086714879	0.340601224	1.538380436	0.588988003	-2.120541619	0.027661555	-0.814887617	0.625892126
AT4G35380	AT4G35380	3.881777448	0.005452283	-3.107897743	0.191218122	-0.344918821	0.892776575	-1.289510398	0.765952517	-2.175729346	0.008050127	-3.37695793	0.096696735
AT5G45080	PP2-A6	7.562031812	0.04532585	-2.561365622	0.293279215			-3.569787813	0.099695852	-2.315828001	2.45277E-05		
AT4G16260	AT4G16260	3.29256779	0.012376278	-0.174133378	0.911252286	1.090248191	0.358240908	-0.385997354	0.870759043	-3.151818257	0.001551085	-0.373668261	0.096182532
AT1G51840	AT1G51840	7.975683788	0.02149287	2.036111417	NA	-4.169754161	0.444084641	-0.703355461	0.917916755	-3.278296467	0.029866247	-1.316501915	0.265063991
AT1G22380	UGT85A3	-4.314083319	0.042934971	-0.966214055	0.411323479	-0.560962942	0.661890699	-6.293315071	0.606294014	6.910648933	0.043902091	-1.148602249	0.558097467
AT3G57210	AT3G57210	-7.384376686	0.008890492	-6.161761925	0.219954486			0.062903708	0.997253752	6.884312592	0.046900432	-0.090559901	0.988233344
AT2G25510	AT2G25510	-2.157857553	0.000583184	-0.103727699	0.94152605	-0.026444284	0.989576544	-1.028076231	0.677952867	4.565050951	0.002263173	-3.046806565	NA
AT4G23600	CORI3	-2.174227789	0.023909708	0.933226824	0.087478397	-0.531241448	0.411552648	-2.126050909	0.638094248	3.184250506	0.009591483	0.562644615	0.092642537
AT3G10720	AT3G10720	-1.317855463	0.000311962	-0.491831318	0.001733336	-0.806927222	0.0001642	-0.251113336	0.863668961	-1.037357887	1.32754E-09	0.662621868	7.98026E-08
AT2G28315	AT2G28315	-2.406389237	0.021311817	-2.088960184	0.60411819	-1.092994449	0.344185824	-1.082825264	0.62738272	-1.712012164	0.000212192	-0.26804452	0.536863766
AT1G70130	AT1G70130	2.298277106	0.024706371	0.215290997	0.955791894	-4.71157708	0.382173607	-2.911046345	0.365746137	1.168083409	0.514843759	2.775693285	0.038573562
AT1G64820	AT1G64820	7.841723588	0.002621243	2.084232862	0.517259319	2.408881848	0.263702666	2.98131972	0.760059267	-0.052938366	0.99699153	2.485147146	0.000280201
AT3G29000	AT3G29000	1.906803687	0.01023057	0.433795364	0.554449861	0.510156882	0.613613871	0.381863677	0.890718713	-0.5060293	0.779117201	1.278889783	0.000582887
AT3G60550	CYCP3;2	2.404406947	0.039362336	0.421129483	0.783216119	1.034128881	0.623373261	-1.146744792	0.646729152	-0.408980836	0.516305535	1.264097824	1.1088E-06
AT5G13870	XTH5	1.037569417	0.047370351	0.712000622	0.086848879	0.542755859	0.177229317	-0.114779364	0.936758504	0.229802995	0.599327211	1.046087348	4.06624E-07
AT1G30080	AT1G30080	5.10822627	0.016331592	2.945170235	0.453678327	-5.923184481	0.066697465	-2.224832616	0.588988003	-0.582257472	0.479007201	-1.013527213	0.001198937
AT4G30270	XTH24	1.168728936	0.002124237	-0.296154975	0.340553155	0.384320807	0.59723779	0.684217994	0.604422229	-0.233016685	0.287120998	-1.021892287	6.05726E-13
AT3G49690	MYB84	5.755976762	0.00423866	-0.019170039	0.992252355	2.701821726	0.451756668	-1.252718508	0.751883565	-0.623924852	0.531929726	-1.024471239	0.034648171
AT2G01660	PDLP6	1.798369904	0.000986444	-0.330069766	0.666946174	-0.760134729	0.221399128	-0.51765407	0.572999928	-0.321745873	0.491449838	-1.088737846	1.26134E-10
AT2G43060	IBH1	1.225336352	0.002010874	0.734245711	0.043183044	-0.014080155	0.977183027	-0.360466121	0.833592548	-0.358441067	0.246238177	-1.101797361	9.89947E-06
AT2G45510	CYP704A2	2.555036633	0.000179602	0.747035213	0.151232773	0.893594411	0.041457386	-0.044171563	0.982055475	-0.271749447	0.515629348	-1.105742566	2.62907E-13
AT1G31770	ABCG14	1.688555852	0.000122805	0.031172255	0.946007207	0.629936231	0.151984857	-0.885306585	0.619569853	-0.513369979	0.03541087	-1.107456302	2.11983E-11
AT3G51910	HSFA7A	1.432704272	9.11363E-07	0.557338507	0.196730959	-0.38352175	0.243885429	-0.059436067	0.968021479	0.265021102	0.433987964	-1.108471448	0.000281011
AT4G29110	AT4G29110	1.367325004	0.010660656	0.440190027	0.492042604	0.413293724	0.506537492	0.363336644	0.869697319	-0.693295113	0.513584451	-1.12453643	0.006269563
AT1G76820	AT1G76820	1.355007469	0.00116739	0.654129941	0.067062843	0.904738264	0.000139574	0.507024875	0.704225272	0.625373524	0.27271809	-1.128541208	1.24666E-10
AT4G06744	AT4G06744	2.358298209	0.014284075	0.997508775	0.126486085	1.15068875	0.133164401	-0.645477888	0.676109289	-0.484411899	0.047888714	-1.133422403	5.93707E-08
AT4G20390	AT4G20390	1.426869596	0.034394855	-0.42426527	0.649023209	0.771202228	0.300437624	-1.060218271	0.561207188	-0.971738667	0.028965666	-1.139447587	4.69044E-11
AT3G53200	MYB27	8.998928374	5.43059E-06	2.375830745	0.462982996	-5.151864406	0.22386822	-0.455735718	0.950313494	0.198927648	0.89611977	-1.15562675	0.003598693
AT3G08970	ATERDJ3A	1.706855886	0.014745519	1.941499314	0.292955885	0.97679052	0.48027595	-0.66452522	0.743764644	0.142914715	0.927080141	-1.156960708	0.001271741
AT5G61260	AT5G61260	2.267853812	0.044831745	0.668136328	0.753304847	0.234022345	0.890006463	-0.836041432	0.704225272	0.606452034	0.536681853	-1.191614063	0.030749034
AT5G66390	AT5G66390	1.905534381	0.001326108	0.527288444	0.334422414	0.009502072	0.992882683	-1.779804142	0.107090587	-0.804006572	2.79103E-06	-1.199788846	7.54365E-11
AT5G65210	TGA1	1.212735845	0.00029287	0.765811206	0.03881807	0.380119477	0.024557449	-0.612324074	0.579644733	-0.371855496	0.125748593	-1.209624965	2.22361E-15
AT5G03700	AT5G03700	1.372813573	0.000819988	0.498788744	0.144161173	-0.036758338	0.953713635	-0.695090677	0.471997679	-0.075398752	0.89546988	-1.223141001	2.77631E-16
AT5G41040	RWP1	1.320205222	0.049494728	-0.205269667	0.853833931	-0.011655149	0.994037176	-1.006225239	0.417861924	-0.677238159	0.085181279	-1.232923969	2.61807E-10
AT4G00910	AT4G00910	2.308709315	0.013262193	-0.447085005	0.898485807	-2.775329292	0.055363573	-1.012266322	0.646729152	-0.197046088	0.895021414	-1.242852557	0.025331948
AT2G36320	AT2G36320	1.527730401	1.29817E-14	0.817437991	0.003360817	0.476132885	0.057481545	-0.467649937	0.623259615	-0.117997553	0.619626918	-1.24360219	2.89435E-20
AT1G69600	ZFHD1	1.772045187	0.001155098	0.511745242	0.619555336	0.678109314	0.235323102	-0.829838796	0.697407195	0.167499171	0.831427457	-1.254321658	1.41341E-06
AT5G15290	CASP5	2.581192431	0.009408997	-0.995725668	0.7950779	-0.346816167	0.881293548	-1.807137666	0.386146892	-0.463416656	0.550184567	-1.25509855	0.001395735
AT5G65040	AT5G65040	2.156919135	7.90198E-06	-0.412974515	0.704099225	-0.582685481	0.420280569	-0.873464642	0.611321113	0.452864906	0.542006423	-1.274814941	8.18245E-07
AT4G32650	KAT3	1.919293395	0.000786731	0.443286348	0.583687707	1.229565306	0.06062159	-0.612316181	0.700915574	-0.311535751	0.423442669	-1.292325853	2.91737E-08
AT5G17430	BBM	8.156777688	0.000465928	-1.814143362	0.669330472	-5.85386944	0.170575766	-1.166083281	0.878942115	0.713366189	0.550924248	-1.293181168	0.042128536
AT4G31100	AT4G31100	1.550318944	0.013101154	0.016953128	0.990789184	-0.343274016	0.815329546	-0.718972638	0.514675172	-0.144657334	0.871060407	-1.293870849	1.46019E-09
AT3G22240	AT3G22240	2.345840364	2.42191E-06	-0.718762416	0.223102388	0.794022014	0.03639576	-1.314247289	0.497907624	-0.574808363	0.056781643	-1.301049095	6.73336E-20
AT1G66173	AT1G66173	1.712579137	0.018530845	-0.049456496	0.973527921	0.485189006	0.195345646	-0.50062463	0.822327646	0.530926267	0.640493482	-1.306226054	5.34648E-05
AT5G22300	NIT4	1.321864974	0.033115868	0.658330357	0.249007628	0.691238777	0.161579967	-0.588210401	0.732322487	0.228055401	0.843491589	-1.316178827	0.015886342
AT5G36180	scpl1	4.624026712	0.034233955	0.135290881	0.972706681	7.504368317	NA	-1.119976967	0.73994806	-0.453053547	0.350799539	-1.316320492	1.69107E-09
AT5G20960	AO1	1.119890816	0.00622284	0.24607686	0.451921956	0.172351605	0.694062628	-0.858106479	0.632278424	-0.64932154	0.000744912	-1.318602355	1.13073E-16
AT4G04830	MSRB5	2.522746606	1.66682E-13	0.284379313	0.845775655	0.358099699	0.803417316	-1.040322079	0.602119156	-0.275983558	0.56843464	-1.331993471	5.39948E-12
AT5G60850	OBP4	1.0264782	8.47157E-05	0.896683512	6.66493E-06	0.990061475	2.06261E-11	0.158909851	0.926124658	0.045281366	0.926546158	-1.336951101	1.49751E-10
AT4G25070	AT4G25070	7.820203053	0.003516125	2.185983887	0.513015701	0.536315643	0.637338337	-0.528658807	0.962474985	-1.386352943	0.119428864	-1.350945457	0.010989741
AT1G78780	AT1G78780	2.578173255	0.030948949	1.480711383	0.260938593	-2.98869257	0.173719368	-1.333276652	0.62738272	0.1410456	0.94576228	-1.352861756	0.000297025
AT3G55515	RTFL7	9.346033652	0.000891822	6.075472437	0.249924206	4.125042983	NA	0.235941545	0.972495118	0.045976235	0.976693515	-1.363332259	3.52932E-06
AT4G12080	AHL1	1.166174994	0.001931503	0.651479119	0.238388286	0.655919363	0.193701528	-0.713294901	0.653026084	-0.331868503	0.401367422	-1.371899634	1.30248E-17
AT3G41768	AT3G41768	1.300442509	0.000168846	0.384768929	0.117039851	-0.233038735	0.187366103	-0.753695657	0.58656299	0.513898289	0.586649565	-1.374842289	0.005063483
AT2G19110	HMA4	1.320449471	1.91977E-06	0.109000231	0.758604757	0.295314562	0.434166957	-0.746673018	0.463461794	-0.209071184	0.477423267	-1.385799553	4.03168E-28
AT1G65690	AT1G65690	2.061515267	0.00601807	0.63844486	0.667443162	-2.365028812	0.209065253	-2.496043597	0.504097788	-0.346908399	0.635237253	-1.389346809	1.84946E-06
AT3G44550	FAR5	8.294639472	0.000238013	-1.449902978	0.666204815	3.087618152	0.358816271	-1.867961779	0.648919095	-1.100887317	0.207299863	-1.407006998	3.12878E-06
AT1G27030	AT1G27030	1.610922704	0.008077013	-0.448592375	0.444924378	-0.087904461	0.820690988	-0.174010487	0.963509602	-0.364041921	0.281955469	-1.427884053	2.59825E-26
AT4G22212	AT4G22212	2.398862004	0.020920645	-0.767602979	0.716748916	0.294842112	0.758472891	-1.794481695	0.343493265	-0.012560031	0.991628492	-1.43167666	6.58962E-12
AT2G35380	AT2G35380	4.088229766	0.001817308	-2.157700384	0.459680415	-0.359009134	0.904268221	-2.744306413	0.404349383	-0.254906011	0.755493303	-1.454963371	4.32615E-07
AT4G23700	CHX17	3.260880148	4.56575E-05	-0.318784471	0.832885745	-1.009493729	0.569491682	-1.333023966	0.563804992	-0.523361034	0.631435158	-1.491029677	7.67315E-08
AT5G10210	AT5G10210	1.650483916	0.017449485	-0.787254145	0.679217956	0.335246003	0.731195446	-0.666307796	0.732322487	-0.091075023	0.950394071	-1.503359744	4.85821E-07
AT2G44370	AT2G44370	9.339115317	2.34079E-07	2.517865235	0.184362493	-2.04132316	0.519897762	-2.986169094	0.45115866	-1.055427119	0.35655851	-1.50576132	0.001253927
AT1G24575	AT1G24575	1.660818239	0.040949683	0.67678681	0.314124822	0.745693362	0.214350028	-0.122006038	0.969967158	0.104008491	0.886579506	-1.506559869	1.23885E-11
AT1G33030	AT1G33030	7.328488553	0.014106377	3.999632979	0.191426718	-0.167900641	0.975724473	0.045115244	0.99607627	-1.481136418	0.449974648	-1.531462719	0.024005872
AT3G21770	AT3G21770	2.692346503	1.99966E-06	0.30930446	0.686977015	-0.350639197	0.48228094	-1.218786025	0.650302848	-0.479645581	0.070974858	-1.54587084	7.58712E-27
AT1G78000	SULTR1;2	1.628778981	0.000350479	-0.426670931	0.512523913	-0.541963061	0.452640789	-0.614268691	0.736631974	-0.828380729	0.018938657	-1.584059755	2.11672E-27
AT1G15210	ABCG35	2.691008932	0.000474259	-1.278735209	0.147553539	-0.082562196	0.936535888	-0.875733381	0.672844426	-0.720137712	0.002368604	-1.585744853	4.14782E-24

AT5G63595	FLS4	7.553279578	0.048930241	2.901806044	0.616592061	1.607724832	0.602181952	-0.21098172	0.976635648	-0.550299283	0.566059569	-1.608319531	1.81773E-05
AT2G40080	ELF4	2.149247841	0.007494305	0.704444709	0.536982421	-0.409124244	0.692835766	-1.153552337	0.073730624	-0.799955601	0.321321609	-1.615220416	3.86521E-05
AT2G29060	AT2G29060	1.906928314	0.001931503	0.483866772	0.462105387	0.555419392	0.313759524	-0.715142655	0.640467755	0.058917532	0.95946592	-1.623902617	2.46242E-07
AT1G80180	AT1G80180	1.058640572	0.00795184	0.920247081	2.79617E-06	0.439538457	0.000324132	0.882328179	0.634717017	-0.188770403	0.508011601	-1.624259143	6.91405E-13
AT5G20550	AT5G20550	7.622693443	0.049974855	-2.285518443	0.60545144	-0.442402206	0.885150831	-0.490198182	0.936758504	-0.53034751	0.579669854	-1.639028233	2.69785E-07
AT1G19960	AT1G19960	1.71221777	0.025452614	-0.519398378	0.82856755	-1.045263692	0.090250211	0.287023103	0.935220363	1.159642995	0.346839563	-1.642755039	0.000809951
AT5G07680	NAC080	2.680712242	0.000297869	0.307042347	0.746880964	0.893416706	0.236098003	-0.604916998	0.565712252	-0.437118	0.505417058	-1.649736123	2.08833E-10
AT5G57480	AT5G57480	2.093066679	0.038551723	0.309926782	0.907826886	-1.326710468	0.087217974	-1.595598944	0.064369348	-0.460157513	0.437782126	-1.674914583	1.12255E-05
AT5G25110	CIPK25	2.949018421	0.03508893	-1.118989534	0.227739807	-0.994655759	0.157664841	-0.738449878	0.705076831	-0.723397607	0.152264493	-1.684223417	1.96644E-09
AT5G09290	AT5G09290	8.420713727	0.000947101	0.947447922	0.797796781	1.020608941	0.869899322	-1.186878816	0.782088381	-0.515283426	0.689109924	-1.68724887	0.000809165
AT2G27550	ATC	7.749839343	4.80299E-07	-4.486087168	NA	-1.994015588	0.564634462	-0.59548628	0.769907555	-0.091483803	0.933105027	-1.690690511	4.41834E-10
AT4G01350	AT4G01350	3.468363146	0.008544709	1.194007877	0.566660999	-1.069729213	0.59723779	-0.836830702	0.784493718	-0.59939108	0.262877013	-1.702708325	1.21212E-12
AT4G14580	CIPK4	2.068447166	3.314E-08	0.402977104	0.681996047	0.197176674	0.769692158	-0.558713948	0.710296405	-0.17681764	0.808204461	-1.703543819	1.76967E-17
AT1G55610	BRL1	1.481687633	0.00460021	0.738194768	0.366789804	-0.527024899	0.540353982	-0.644578435	0.66808962	-0.34288447	0.529108457	-1.70585325	3.64167E-18
AT1G51420	SPP1	10.27239479	2.32396E-11	0.752105782	0.806182502	-0.52257683	0.834662109	-0.887846974	0.699108913	-0.812738039	0.269079164	-1.709920675	0.001119047
AT1G61590	AT1G61590	1.777530052	0.010045556	0.330702004	0.701715699	-0.717566564	0.423190215	-1.296200435	0.309698198	-0.814266765	0.003657892	-1.718557591	1.89368E-12
AT4G11190	AT4G11190	3.231403281	0.038413106	0.378254111	0.809091009	1.592047722	NA	-1.068897264	0.691824202	-0.431458036	0.093724774	-1.72437429	2.50807E-35
AT5G40730	AGP24	2.293109759	2.14537E-05	1.222729434	0.1519642	-0.939592336	0.298971618	-1.138458924	0.558566659	-0.592079566	0.002103486	-1.725065778	0.011876985
AT3G20960	CYP705A33	3.498301004	0.005773005	0.577485107	0.526920474	0.924118273	0.187696847	-0.513435558	0.831161184	-0.10397438	0.893744923	-1.748467468	2.82556E-13
AT1G12010	AT1G12010	1.83921662	1.80513E-06	0.004222613	0.99432117	0.8139824	0.470253428	-0.16004742	0.942727755	0.217162086	0.703919657	-1.807769146	1.3599E-08
AT4G16008	AT4G16008	1.176601477	0.003610518	0.873949531	0.002542555	0.79320919	0.005108342	0.536337131	0.779496598	-0.136749003	0.778769276	-1.81200319	8.96242E-12
AT2G28780	AT2G28780	1.537263083	0.005955825	-2.511951248	0.298942593	-2.942658825	0.421354799	-1.587992189	0.445362737	-0.661426825	0.282859045	-1.812663486	7.08377E-22
AT5G23190	CYP86B1	7.791825866	0.031692682	-6.202461832	0.218909953	-1.076500839	0.851925577	-2.466905348	0.499733644	-0.13484208	0.929239543	-1.814368715	6.48969E-11
AT2G29620	AT2G29620	8.361602308	0.00021205	6.594914132	0.207021441	4.948012855	0.355538637	-0.320822757	0.967195835	0.580572775	0.743097639	-1.81523974	0.030868569
AT5G65790	MYB68	4.210308768	0.00296729	0.300774066	0.841647002	0.866142959	0.675319516	-1.026017015	0.420106526	-0.557259773	0.423442669	-1.818306139	2.19025E-15
AT1G05260	RCI3	2.163397085	0.043682917	0.370206552	0.806531082	-1.181144416	0.535162474	-1.48062126	0.777104408	-0.85913094	0.000394412	-1.830347273	3.55609E-53
AT4G20820	AT4G20820	2.099701123	0.000893116	0.541713514	0.547218349	-0.504960008	0.243776817	-0.227934904	0.907260297	-0.166342407	0.868982135	-1.83061683	1.74697E-11
AT1G67110	CYP735A2	3.225288468	0.000327457	1.352355318	0.151614144	1.886627012	NA	-0.890378254	0.582068518	-0.73560216	0.079186571	-1.834997273	4.71219E-30
AT2G48130	AT2G48130	4.432902603	0.013114597	-0.375469917	0.919061855	-3.182303927	0.175469006	-1.796183738	0.65378996	-0.953460416	0.137096093	-1.839797962	2.72278E-17
AT5G28080	WNK9	3.974619558	0.001277126	0.558326877	0.673446362	0.414349795	0.625467473	-1.392538856	0.603941405	-0.285291381	0.638684873	-1.842969493	4.19824E-40
AT1G03840	MGP	9.295059115	1.63137E-06	-2.445693027	0.560348617	0.127531018	0.977203483	-0.694534462	0.784493718	-0.102393773	0.973543025	-1.84476254	0.002360712
AT1G64590	AT1G64590	7.92886555	0.021432621	-6.621300073	0.05185929	-5.77566385	0.096278468	-1.726471499	0.697407195	0.562918019	0.891499092	-1.849061436	0.007613466
AT5G10280	MYB92	3.381239781	0.01293789	0.187061197	0.892428751	0.394006792	0.76171361	-1.200141614	0.856800122	-0.328993474	0.547993779	-1.853569549	2.92364E-25
AT4G28940	AT4G28940	7.577861026	0.047151543			0.815496959	NA	-2.341408285	0.587695712	-0.27352117	0.907309781	-1.866875977	3.62318E-06
AT4G35390	AGF1	4.67703105	0.043682917	0.361025846	0.941640409	-0.22002325	0.969487671	-1.902740783	0.641679337	-1.010215781	0.392120814	-1.880396703	0.002650385
AT1G07610	MT1C	1.938283046	1.89153E-07	0.441846938	0.528465168	0.412076281	0.417843377	-1.099563681	0.589531904	-0.654034895	0.09955352	-1.888963066	0.001898963
AT5G07322	AT5G07322	2.514660306	0.005968685	0.788965196	0.741484683	-0.778163962	0.394823035	-1.11125459	0.685178869	-0.272227719	0.700183076	-1.893458675	4.24323E-13
AT5G05320	AT5G05320	1.517402239	0.009187828	0.45081002	0.464362213	0.306850796	0.680969106	-0.042308479	0.987613184	-0.057647764	0.958007814	-1.899927181	3.07429E-17
AT5G02090	AT5G02090	3.598621914	6.50505E-07	1.059622918	0.180078605	-0.817357835	0.136802778	-0.780379632	0.661873187	0.131271437	0.906954561	-1.90178088	2.69977E-10
AT5G13910	LEP	7.814465628	0.028690075	-5.138178927	0.33816757	-2.648405744	NA	-1.808319283	0.681538103	0.136038373	0.942567361	-1.913248672	5.87933E-05
AT4G39795	AT4G39795	2.447571279	0.00285554	-0.832529035	0.511011346	-2.558704971	0.176288453	-0.785371521	0.877665269	0.181159296	0.87549967	-1.917215442	3.40092E-10
AT4G11880	AGL14	7.675624852	0.038260697	-2.610176647	0.423632749	-3.967003132	0.468268694	-1.628367673	0.680482607	0.364527364	0.885377618	-1.925130846	0.000244439
AT1G77885	AT1G77885	1.059232236	0.048497153	-0.508618053	0.477874901	-0.006339578	0.99280792	-1.6399411	0.45375294	-0.036669142	0.97325662	-1.936528539	2.67876E-16
AT2G47130	SDR3	3.626712845	0.000400539	0.410311171	0.673725998	-1.699673797	0.084764278	-0.37040611	0.799604234	-0.080044653	0.944784464	-1.949146611	6.4147E-16
AT1G02520	ABCB11	3.287553313	7.20664E-09	0.52594383	0.608152041	1.658327986	0.148968617	-1.372270618	0.360555684	-0.527267519	0.398922866	-1.955696298	1.55248E-16
AT5G59780	MYB59	1.818574707	6.10231E-14	0.569216961	0.003436602	-0.621627593	0.123173219	-0.539843012	0.705076831	0.039259711	0.954684273	-1.965793258	2.3715E-58
AT3G14280	AT3G14280	2.888315164	5.59245E-06	-0.285220937	0.803684447	-0.267957639	0.731516858	-0.861598797	0.836430582	-0.465103299	0.423241879	-1.975417523	5.83165E-28
AT1G65510	AT1G65510	2.406272534	1.45875E-05	1.119488646	0.148806163	0.765254068	0.42729072	-1.138262424	0.353346746	-0.397510082	0.366101624	-1.984424012	2.48175E-12
AT2G23540	AT2G23540	3.214647274	0.002529838	-3.649115987	0.081906816	1.340547165	NA	-2.085767089	0.168399238	-0.457666737	0.39039795	-1.98814194	3.896E-58
AT5G50760	AT5G50760	2.362773023	0.005151998	-0.117560906	0.92600671	-0.0120419	0.990286483	-1.336766505	0.732322487	-0.718599388	0.283161645	-1.992672308	4.37053E-15
AT4G38080	AT4G38080	2.721668968	2.48947E-07	-1.021379096	0.083995021	0.981270496	0.177719418	-1.771382124	0.254941695	-1.022594982	0.1790983	-1.992695763	2.26326E-42
AT5G51780	AT5G51780	1.40976503	0.001887906	-0.422271629	0.546249886	-0.352026425	0.64766522	-0.976386524	0.430181703	0.221724258	0.668927554	-1.993889738	5.67171E-18
AT3G21710	AT3G21710	3.333410584	0.001451849	0.245543343	0.868617429	-1.729208808	0.642882344	-0.726887762	0.892607537	-0.964598536	0.038878892	-1.999945674	2.55542E-11
AT3G18400	NAC058	5.111768907	0.006555953	-0.392119065	0.918282679	-0.723643896	0.815604719	-2.281643866	0.575580003	-0.682982116	0.601022413	-2.014258608	1.91643E-13
AT2G31020	ORP1A	3.279131316	0.020127088	0.711713958	0.75750311	0.24650486	0.835530304	-1.505636391	0.481011352	-0.869491302	0.1812604	-2.014531899	8.47611E-09
AT5G38550	AT5G38550	8.319834423	0.001679293	5.206822031	0.330858911	-4.353942638	0.423752099	-0.078459058	0.976635648	-1.735091135	0.08501642	-2.020158499	0.000146507
AT1G08670	AT1G08670	8.715653997	0.000247925	-0.470547901	0.929298011	0.450555461	0.902925112	-2.203290043	0.611039849	-0.714015906	0.42662725	-2.023625677	0.00205613
AT5G38450	CYP735A1	8.332292768	0.001005841	-3.591666303	NA	5.072009726	0.341892172	-2.847293723	0.627163215	-1.295773788	0.631530565	-2.030348449	0.000836858
AT5G06090	GPAT7	8.179437295	0.00045278	4.820761594	0.100622599	1.147903943	0.844684779	-0.29677142	0.969967188	-0.417369737	0.846818973	-2.037066982	0.003497351
AT5G46845	ath-MIR160c	4.439816734	0.00017749	2.158464738	0.317643971	1.66594832	0.179105125	-0.666631658	0.680416479	0.336027636	0.719986639	-2.053755016	1.38168E-05
AT4G30450	AT4G30450	3.466899383	2.54057E-11	0.377684486	0.756938797	0.08893791	0.929354074	-1.074844482	0.685334859	-0.014460219	0.992180597	-2.08513932	1.25437E-05
AT4G11650	OSM34	4.073909709	2.92594E-09	0.768607458	0.32011156	1.37657232	0.65979946	-0.636174536	0.767355318	-0.953808307	0.048834515	-2.142109083	1.66206E-37
AT3G51350	AT3G51350	2.252526511	0.012263336	1.429664234	0.218989881	-1.587959628	0.434423568	-0.618809536	0.665673432	-0.63510671	0.474596951	-2.194644196	3.72958E-07
AT1G29280	WRKY65	1.586570881	0.011673575	-0.13754618	0.868838443	-0.57307304	0.599816363	-1.024226768	0.626994653	-0.68712606	0.012283914	-2.203588374	2.37743E-38
AT3G16450	JAL33	1.017446609	0.011608761	0.08023703	0.733294316	0.622871422	0.654177633	-0.090009394	0.966082349	0.022203962	0.965786265	-2.208938387	3.89601E-24
AT2G16005	AT2G16005	8.265033781	0.010084779	-3.176438033	NA			-0.214806469	0.980070246	0.149741835	0.950989651	-2.256770099	7.56941E-05
AT4G32860	AT4G32860	2.552864903	0.009268068	0.327830398	0.832850375	-0.63543181	0.704308217	-1.105034962	0.593379395	0.055542509	0.965288092	-2.264483864	1.12358E-24
AT5G45380	DUR3	1.938476197	0.000313656	0.59079996	0.586190692	1.167778191	0.133750036	-0.859112195	0.677978366	-0.313190784	0.711316589	-2.288463577	4.8521E-32
AT3G46130	MYB48	1.456710542	0.009501142	0.188446874	0.827760652	-0.850676435	0.611677889	-1.048042349	0.639875152	-0.062304564	0.939586734	-2.295317755	4.84374E-54
AT1G73280	scpl3	8.649486902	0.000159299	-0.856933282	0.814050636	0.07185994	0.989491688	-0.224016456	0.975645787	-1.112418092	0.35782048	-2.302792907	0.000176077
AT5G06740	AT5G06740	9.067464638	5.71648E-06	1.140438241	0.75746763	0.826108921	0.802071542	-1.259312573	0.767355318	-1.251026316	0.131118879	-2.309511817	9.77879E-06
AT1G08320	TGA9	3.024678419	0.02354026	-0.643627012	0.56966914	-1.676436904	0.285496623	-0.898428883	0.758715149	-0.551380237	0.258496511	-2.313243306	1.6884E-18
AT4G16563	AT4G16563	1.337946939	0.01715368	0.087259813	0.940039895	1.336127881	0.111580793	-0.472517509	0.821797134	-0.733266488	0.176633494	-2.353839096	1.62682E-12
AT3G44290	NAC060	3.735526806	0.02848413	2.787988543	0.068552633	0.923294337	0.698391736	-2.040562048	0.359834404	1.251477862	0.402232289	-2.387064918	0.000938021
AT2G35000	ATL9	7.327111163	4.77583E-05	0.30277327	0.930686245	0.016854531	0.995492744	-1.862034859	0.663291724	-0.519554145	0.666064463	-2.393501306	2.12936E-07
AT3G04530	PPCK2	9.477908774	4.53062E-07	1.172642899	0.757003448	-4.567171562	0.398424355	-0.684449347	0.906077605	-0.540790258	0.613407287	-2.416122046	7.83112E-11
AT1G67030	ZFP6	2.672107487	0.036336269	0.187088847	0.89562865	-0.510281223	0.729632661	-1.029233249	0.637815859	-0.383325901	0.56302106	-2.448313068	2.95232E-34
AT1G17800	AT4G17800	1.919375771	9.74488E-05	-0.007915141	0.991532036	-0.665665846	0.466756371	-1.170182727	0.471997679	0.303278734	0.548448609	-2.461910843	3.74951E-30
AT4G22070	WRKY31	7.979545392	0.001614821	2.789549751	0.628863761	0.888754417	0.788898792	-1.78818004	0.686315536	0.185990033	0.930662149	-2.475941247	0.002095714
AT1G71692	AGL12	3.339854176	0.013908868	2.399094577	0.221853707	-0.396233024	0.762462004	-1.355412125	0.469878733	0.69564223	0.295272258	-2.476219057	0.021922478
AT4G37010	CEN2	2.298556845	0.020593966	1.335686475	0.238609621	-0.75682345	0.713429359	-0.894399397	0.828875452	-0.553559597	0.437782126	-2.480260947	2.76885E-11
AT1G18860	WRKY61	9.539680488	2.64962E-08	2.219887565	0.710265504	-1.383779064	0.730790909	-0.044542905	0.997245719	-1.576440299	0.238414153	-2.48149902	1.50135E-06
AT3G61270	AT3G61270	7.480128193	0.012609799	-2.954084081	0.363882234	-2.399144834	0.494112572	-1.242077436	0.728894922	0.524660173	0.732098793	-2.489947744	5.89251E-05
AT3G01260	AT3G01260	3.455145235	0.004332775	-2.463247114	0.190129457	0.072099622	0.971852646	-1.709056434	0.143428011	-0.611253093	0.292535313	-2.498103485	1.19511E-45
AT2G29330	TRI	4.603675926	4.00108E-05	-0.848668354	0.771077714	-1.141171377	0.518726396	-0.705321309	0.895706838	-0.885132335	0.136659092	-2.542514739	3.55229E-11
AT5G44920	AT5G44920	4.724120165	6.63328E-05	0.87009696	0.477113114	1.230360271	0.695351542	-0.945781223	0.837258505	-0.081731796	0.921971948	-2.548092661	1.43651E-06
AT5G10580	AT5G10580	4.070131351	1.15441E-11	-0.916825597	0.329058498	0.027950023	0.996023703	-2.197900031	0.25501825	-0.691666292	0.096569468	-2.571902029	1.72287E-72
AT1G49310	AT1G49310	3.322679338	1.43196E-06	1.336034774	0.104491787	1.238358409	0.115059031	-0.495073438	0.809598664	-0.406258231	0.529822481	-2.586813459	2.39122E-16
AT5G63600	FLS5	3.674145301	8.43459E-20	0.34082641	0.410232212	0.029369591	0.983639587	-0.85241791	0.704470077	-1.072829517	0.099161951	-2.587817444	4.90769E-33
AT2G01520	MLP328	2.852999104	0.000399604	-2.195885134	0.154774875	0.396463636	0.836718313	-3.254392871	0.051975957	-1.048450526	0.37021645	-2.593790245	5.36429E-57
AT5G66870	ASL1	7.450882378	0.012954032	-0.557039006	0.877725449	2.994395767	0.464644883	-0.754057031	0.809598664	-0.625105052	0.511659422	-2.618630497	1.30154E-14
AT2G37750	AT2G37750	4.334429933	4.12832E-17	0.775929046	0.235453593	-0.70968706	0.41887282	-1.164669717	0.610227484	-0.488177116	0.269079164	-2.618838534	5.03569E-30
AT4G15400	BIA1	7.341346362	0.000151446	1.97995782	0.493901913	0.991425567	0.667944115	-0.470988237	0.95660961	-1.958213686	0.063531748	-2.624872721	5.86461E-07
AT1G68150	WRKY9	10.13994238	1.77529E-10	1.491823588	0.43303311	-0.24044383	0.939914065	-1.363800808	0.7265412	-1.456802471	0.115965322	-2.661348952	4.38522E-10

AT1G35330	AT1G35330	7.975104255	0.021933754	-3.176438033	NA	-3.585343201	0.516230268	-0.344406065	NA	-1.62639281	0.364175721	-2.661775451	0.024795008
AT1G22065	AT1G22065	3.551255488	0.006253824	-2.386707687	0.385484079	1.151437976	0.416862201	-0.941110053	0.701052583	-0.364276158	0.579526976	-2.678530983	1.68026E-25
AT5G23830	AT5G23830	3.479173028	0.024426785	-2.518278121	0.353231516	0.656334353	0.839497121	-1.522520752	0.691895802	-0.533917005	0.437782126	-2.710411432	1.5157E-28
AT1G70880	AT1G70880	2.702556037	0.000641501	-0.53994386	0.813381602	-0.308824053	0.812922538	-0.512456542	0.847546649	-1.012893679	0.443659868	-2.733718194	1.60477E-12
AT2G36295	AT2G36295	7.793551917	0.0042018	-3.875540153	NA	-2.353593325	NA	-0.188995837	0.987665908	-1.053032323	0.72855886	-2.739574539	0.008755174
AT1G66280	BGLU22	3.914957651	4.64645E-07	-0.461607245	0.755699073	-0.491779056	0.709249602	-2.096574614	0.343493265	-0.41582401	0.181124968	-2.742039816	6.334E-104
AT3G27884	AT3G27884	4.923208262	0.020069105	-0.613749886	0.910195579	0.867146324	0.707044624	-2.237727382	0.594299218	-0.366674412	0.804786811	-2.743280577	9.9384E-56
AT3G20940	CYP705A30	7.654540094	0.045607625	-3.176438033	NA			-1.168046626	0.791617906	-1.866016797	0.315645869	-2.759802521	0.000180371
AT3G24240	AT3G24240	3.197646425	0.048599195	-2.028424748	0.412503803	-0.918031058	0.345806735	-0.484807642	0.845321467	0.429094638	0.64301291	-2.782240817	5.28219E-27
AT4G19980	AT4G19980	7.389524574	0.01323974	3.720853937	0.499975464	-5.57987565	0.218374753	0.899339224	0.929134073	0.673859953	0.779416896	-2.805252114	0.008016037
AT5G26310	UGT72E3	7.606467073	0.007543665					-8.338645061	0.15552114	-2.25022145	0.215476608	-2.81288444	2.41895E-07
AT5G48000	CYP708A2	4.265059626	0.009920565	-0.72817946	NA	0.033887688	NA	-1.726110091	0.769724647	-0.476578819	0.247887542	-2.813991478	9.43252E-37
AT3G04360	AT3G04360	8.570260437	6.86617E-05	0.847700274	0.86911886	2.596755846	0.586260855	-1.247363547	0.767112256	-0.550885745	0.747890859	-2.818165495	2.64711E-08
AT1G53940	GLIP2	3.536757265	0.04532585	-2.171372364	0.419053533	0.306567052	0.924206514	-1.168203096	0.785453357	-1.379992919	0.054073078	-2.830637441	1.5093E-26
AT5G59530	AT5G59530	3.206412745	0.015849747	-1.25365578	0.203258589	1.357286624	0.318232214	-0.575126037	0.813181718	-0.383186437	0.613826202	-2.831474018	1.24274E-11
AT3G14185	AT3G14185	6.6568206	1.72059E-05	1.882917419	0.42098276	1.251294183	0.510150325	-0.877474038	0.875648986	-0.448216173	0.891499092	-2.85167168	0.000154954
AT4G18510	CLE2	8.291215989	7.38317E-08	1.876448188	0.370628719	-3.213771363	0.335006603	-1.949513479	0.176712738	0.018683991	0.992281921	-2.898082088	1.78054E-15
AT3G21352	AT3G21352	7.914769638	0.025966161	3.768017673	NA	2.069311432	0.636648751	2.214367303	0.818515078	0.960509851	0.758120679	-2.906704327	0.00172977
AT1G21528	AT1G21528	8.533279012	7.34464E-05	0.576809	0.898743089	1.028456996	0.816787004	-1.069045405	0.837258505	2.579619423	0.500487453	-2.907241565	0.000101282
AT5G66690	UGT72E2	6.342001962	1.62144E-07	-1.513316452	0.619555336	-1.45505061	0.575770259	-1.261086354	0.848696702	-0.639098581	0.211913228	-2.913987524	1.079E-28
AT4G15270	AT4G15270	8.392337797	0.000203916	1.946031298	0.75099289	-5.97491444	0.13588278	-1.121923999	0.796607769	-1.597625436	0.468044244	-2.925140542	1.60311E-06
AT1G70850	MLP34	3.528091168	0.000255521	0.406151828	0.439349182	-0.526082153	NA	-1.350119063	0.619569853	-0.391049059	0.63650345	-2.93505775	4.79602E-68
AT5G13150	EXO70C1	7.891603046	0.002082431	7.083715037	0.086913335	0.270457746	0.958168982	-1.866738838	0.660697037	-0.217048119	0.885377618	-2.952189218	0.002488918
AT3G12977	AT3G12977	3.33940021	0.038009305	0.990260997	0.701771624	-0.022041924	0.990461216	-0.809022411	0.871618665	-1.399977496	0.0512972	-2.961674396	1.52196E-30
AT2G35270	GIK	6.966813125	3.04755E-07	0.143583874	0.970618499	-1.419457857	0.665697665	-2.640093813	0.448215878	-0.78573568	0.554552826	-2.983769535	1.59262E-11
AT2G02120	PDF2.1	3.716875333	0.012917743	1.275560168	0.607837548	0.296713813	0.848831555	-2.819771313	0.430181703	0.397081111	0.693137369	-3.070201276	2.5645E-41
AT4G15990	AT4G15990	1.477102814	0.000655603	0.692818826	0.011448186	-0.889826128	0.017635385	0.609917628	0.774188277	0.056813271	0.956275704	-3.103238469	1.49553E-23
AT5G26290	AT5G26290	7.067724745	2.04483E-06	-0.543075354	0.692499673	-1.050220108	0.690009831	-0.664008001	0.704225272	0.514502745	0.448519218	-3.112191953	2.48822E-19
AT4G15340	PEN1	2.02331296	0.006398789	-0.468928544	0.834112307	-0.761124727	0.842237571	-1.323887361	0.613855588	-0.727800057	0.084413786	-3.204251738	0.001308206
AT2G20080	AT2G20080	7.801353308	0.028737485	2.107955204	0.725558826	6.610200892	0.107867045	-1.265779018	0.585524552	-1.11045081	0.186040855	-3.23134219	5.54316E-07
AT5G43540	AT5G43540	4.793989673	0.007948901	-0.31073493	0.903996989	-1.456066579	0.570425526	-1.431276978	0.714552244	-0.291610386	0.782369045	-3.275533806	3.20714E-38
AT1G08990	PGSIP5	5.215746536	0.012296335	-4.253899481	NA	-3.326825067	0.265108633	-2.958876171	0.392763018	-1.678405619	0.161334136	-3.298143758	2.41341E-09
AT5G59520	ZIP2	2.116169755	0.020930358	-0.717265899	0.731306963	-2.185146045	0.154625487	-1.487717692	0.646729152	-0.76601205	0.136821643	-3.357106411	1.33721E-28
AT5G47990	CYP705A5	10.57574109	2.06889E-07	-2.059762882	0.733576829	0.633188264	0.855076928	-2.26832009	0.692108738	-0.085995793	0.924808173	-3.41304329	6.69942E-28
AT2G45360	AT2G45360	7.619303708	0.042688099	6.747734404	0.157225093	0.363215409	0.883075636	-6.068671663	0.616300683	-5.025462607	0.136808239	-3.422416227	0.042660463
AT5G44610	MAP18	3.319120245	9.84309E-05	0.579109839	0.847313057	-0.87010022	0.512702109	-1.72847778	0.759753443	-0.743935396	0.221396166	-3.472803343	8.07775E-09
AT2G05440	GRP9	5.108472078	5.09019E-26	0.004281361	0.996544923	-0.89166102	0.430010137	-1.531892449	0.583036307	-0.012474458	0.996096785	-3.503800803	0.001193616
AT1G33750	AT1G33750	8.527838288	0.005103558	-5.092099046	0.343592375	-6.493473289	0.067719528	-0.790033264	0.884201248	0.683512838	0.663717238	-3.544287919	3.46331E-06
AT3G28345	ABCB15	3.076363666	5.27883E-09	-0.586918452	0.393349853	-0.465503334	0.746723019	-1.362723596	0.567989442	-0.548488366	0.38827458	-3.558600291	2.73342E-40
AT2G14820	NPY2	7.599187248	0.008894565	-7.168325587	0.058432024	-5.338825754	0.282993844	0.294380865	0.980732812	1.925503764	0.576608643	-3.721028693	0.004733869
AT3G14260	AT3G14260	2.491938617	0.006992753	1.060204606	0.81058459	-5.800194113	0.09317458	-2.472763148	0.54270472	-1.648230661	0.086779373	-3.721925851	0.032308535
AT1G74500	BS1	7.162706819	0.02628938	-0.732909509	0.903497425	-5.418290937	0.190163512	-3.417364023	0.671767466	3.366675545	0.258496511	-3.743243448	0.000235136
AT3G21520	DMP1	8.469823869	0.006163496	1.824454599	0.758921897	-0.832542299	0.837504531	2.352724679	0.804495913	5.270230278	0.478400288	-3.770327954	0.0403576
AT3G51360	AT3G51360	7.786457391	0.004054865	2.498931846	NA	0.596989172	0.909399887	-2.238814617	0.715935596	-2.853644411	0.220100806	-3.878009663	0.00083438
AT3G27150	AT3G27150	9.045231273	8.71108E-06	-3.363580594	0.328142969	-1.140833544	0.67997173	-2.556168826	0.616300683	0.225469746	0.934226173	-3.986981741	1.50985E-35
AT5G48100	TT10	8.227237822	0.001304112	0.447846773	0.945819696	2.026206623	0.62341452	1.287963602	0.885200785	0.659956789	0.962199154	-4.221398893	0.000538844
AT3G48340	CEP2	7.702422888	0.010241214	1.300048764	0.797367342	1.49576354	0.760754636	-4.152122833	0.067229778	-0.416881453	0.759562292	-4.376735871	9.00844E-06
AT4G15280	UGT71B5	7.139172494	0.028206645	-3.553772892	NA			-6.734857897	0.553691236	-1.46265202	0.543446569	-4.574913021	0.010252642
AT3G50640	AT3G50640	8.723616782	0.002760496	-5.21583593	0.330273274			-0.166970256	0.982055475	-0.050166882	0.986181089	-4.689650914	5.89661E-06
AT1G66270	BGLU21	4.687909022	0.002120329	-1.743421166	0.503712282	-2.441254312	0.322127153	-1.105433222	0.62738272	0.4058942	0.456417144	-5.082016913	6.934E-106
AT5G46890	AT5G46890	8.03256241	0.022123392	-0.899262106	0.888989745			-1.988441767	0.646729152	-3.17561716	0.254724672	-5.322134688	0.016949833
AT1G61080	AT1G61080	7.145302189	0.030254705	-0.495070349	0.940395392			-7.337007362	0.058170834	2.256240623	0.612310929	-5.762098164	0.006263267
AT4G15740	AT4G15740	7.017406765	0.037153768	1.717978147	0.74309325			-1.072439473	0.909509184	-1.49995783	0.790926204	-6.355788575	1.11237E-07
AT1G30135	JAZ8	-3.668468153	0.009408997	-1.871796404	0.280547819	-0.705713819	0.685436983	-5.303997518	0.652047062	1.804053348	0.868126439	6.989845758	1.59931E-17
AT1G19210	AT1G19210	-2.142371314	0.000471053	-1.10905572	0.090418733	0.070894036	0.974092637	1.869919129	0.348017657	-0.225877907	0.952599873	6.123851872	6.2009E-126
AT3G44350	NAC061	-6.916366953	0.031520061	-2.591101112	NA	-1.619401708	0.787911947			6.851573814	0.10851461	6.062371611	0.007938354
AT1G56250	PP2-B14	-1.405739245	0.030449515	-1.613531453	0.072866859	-1.211420431	0.481778289	-4.895208516	0.106739726	-2.667591955	0.325217058	4.363215243	3.97931E-10
AT3G46090	ZAT7	-3.057043074	0.000207087	-0.781053004	0.495998438	-0.504170172	0.817453193	-1.099214189	0.847915568	-0.485540002	0.959396129	3.007363445	0.00667559
AT4G11280	ACS6	-1.065013414	0.01647718	-0.661985892	0.001781161	-0.212711794	0.821536319	1.473441768	0.272560088	-0.92061189	0.000148098	2.976761822	1.08933E-97
AT4G14450	AT4G14450	-1.907961166	0.03078093	-1.503471423	0.135271917	-1.300486721	0.32616655	2.372933803	0.627500883	-2.292897419	0.118968926	2.568482515	0.004897817
AT4G23220	CRK14	-2.099215888	0.03419535	0.468260694	0.659387534	-0.871327725	0.591203351	-2.829033314	0.405880771	-1.184302814	0.602615521	2.467507279	0.000113674
AT4G34150	AT4G34150	-1.239244139	0.011500731	-0.681373003	0.003446119	-0.824340743	0.018006665	0.450104076	0.823849926	-0.748329512	0.001132723	1.871307521	4.40251E-63
AT3G23170	AT3G23170	-1.184314287	0.013149113	-0.963649092	0.003964251	-0.564506812	0.042514274	1.14934132	0.548879103	-0.582201544	0.181834326	1.866426244	1.13859E-38
AT5G65300	AT5G65300	-1.161740831	0.039700129	-0.608365109	0.136326571	-0.879173565	0.7386381	0.795769803	0.637815859	-0.313493454	0.756378496	1.79114669	1.99835E-09
AT4G13770	CYP83A1	-2.785835607	0.037054615	-1.369625006	0.225575531	-2.740290233	0.116418774	-0.024424115	0.993527973	0.087698434	0.983568996	1.734334829	6.67645E-19
AT1G24330	AT1G24330	-2.010664034	0.008724717	-1.312873771	0.053160693	-0.382017563	0.685525339	0.970960932	0.485140053	0.282652303	0.84694947	1.608989226	2.80473E-06
AT5G24210	AT5G24210	-2.710903182	7.72143E-07	0.205166919	0.806182502	0.397344612	0.001297453	-0.381756031	0.962474985	1.072139945	0.60427973	1.563144719	1.53943E-17
AT1G56520	AT1G56520	-1.514458923	0.005867324	-0.202367296	0.706509601	-0.913690197	0.001343804	1.052229705	0.637815859	0.08207293	0.956955597	1.528042532	5.83627E-10
AT3G16140	PSAH-1	-1.62628329	1.94113E-05	-0.936670646	6.32123E-10	-0.239020352	0.095055298	2.28566766	0.064550355	0.591405166	0.078060542	1.415698539	6.75979E-16
AT1G55450	AT1G55450	-1.577722155	0.000131916	-0.696884555	0.004800925	-0.425744615	0.572879014	0.485738129	0.809502311	-0.870589262	0.244448816	1.409606569	4.06456E-21
AT3G22880	DMC1	-1.727007425	0.027042415	-2.824143699	0.248895344	-1.256210193	0.053164172	0.419821378	0.704225272	0.09905821	0.916204809	1.363753441	4.48331E-11
AT1G63750	AT1G63750	-1.202890917	0.001944946	-0.248165423	0.486061413	-0.476256366	0.072373126	0.273226444	0.886772005	-0.37173846	0.516239281	1.308661111	7.28718E-09
AT4G01310	AT4G01310	-1.181886703	6.62895E-07	-0.605662881	0.010149836	-0.860893705	1.86589E-16	0.951970402	0.282214136	0.630027922	0.000771532	1.2767206	7.37518E-26
AT4G37610	BT5	-1.056271371	0.040101718	-0.81469395	9.10487E-06	-0.580780674	0.002970154	1.409245211	0.408402881	-0.276805128	0.578542261	1.265527501	4.04095E-13
AT3G55630	DFD	-1.914235015	0.000285704	-1.087274755	0.152429821	-0.829835578	0.000785988	0.0227175	0.993804788	0.396083153	0.579014551	1.235895475	6.07913E-07
AT5G13630	GUN5	-1.59753628	5.76786E-07	-0.854537241	4.51915E-12	-0.832625264	6.82445E-09	1.595479195	0.150731881	0.592464474	2.20436E-05	1.230886594	2.3201E-21
AT2G02450	LOV1	-1.119958554	0.003039397	-0.614721089	0.138762609	0.203795957	0.413640048	0.452825781	0.837258505	0.217249195	0.849103006	1.222069655	6.70388E-12
AT1G66980	SNC4	-1.062288891	0.007232153	-0.706711665	2.27396E-05	-0.057177347	0.807508661	1.243459425	0.103567007	0.301753123	0.50473905	1.210482619	6.44922E-09
AT5G44060	AT5G44060	-1.295489529	0.01997818	-0.568656269	0.129366766	-0.660227172	0.300437624	0.432046281	0.705076831	-0.482193594	0.400769075	1.192025123	0.000325116
AT5G35790	G6PD1	-1.27933211	2.10959E-05	-0.971190164	1.20405E-06	-0.464726265	0.009702517	0.850286589	0.309230058	0.623351078	0.001592389	1.178222563	8.73803E-19
AT4G25290	AT4G25290	-2.429508151	3.60855E-05	-0.854230478	0.139186038	-0.984415051	0.009676212	0.617005524	0.644839236	0.842132402	0.093724774	1.173898466	2.95125E-06
AT5G51820	PGM	-1.123226502	0.000329858	-0.848457584	0.015024159	-0.871067709	3.6053E-05	0.344984368	0.691824202	0.155284124	0.638629847	1.170570871	1.82551E-22
AT5G19220	APL1	-1.253078648	0.001734721	-0.871696491	0.003623854	-0.383170282	0.033347269	2.109567822	0.165975469	0.436306102	0.459415277	1.100340081	1.23998E-05
AT5G47190	AT5G47190	-1.315101169	7.47315E-10	-0.869777859	0.005786345	-0.924937082	2.18551E-17	0.627393981	0.597380545	0.668661546	3.81206E-06	1.094860405	2.48822E-19
AT1G64510	AT1G64510	-1.097996947	8.48099E-07	-0.714147727	0.001144218	-0.850689821	9.28566E-15	0.501032942	0.639605292	0.416995641	0.142463471	1.090553113	4.07477E-16
AT5G66675	AT5G66675	-1.255196772	0.001087546	-0.479737789	0.061779927	-0.416431184	0.076907853	0.052991324	0.978807026	-0.08323281	0.894710906	1.078860614	7.17404E-12
AT3G56940	CRD1	-1.082327259	0.005125699	-0.569084506	3.50682E-08	-0.214767847	0.102270292	2.508045141	0.060059967	0.450082037	0.023970427	1.076624401	3.57357E-08
AT1G49010	AT1G49010	-1.302780034	0.005605516	-0.630240072	0.170874153	-0.710599615	0.015500656	1.127576942	0.594489802	0.14600283	0.87530671	1.070957796	3.68855E-06
AT4G30280	XTH18	-2.465713237	3.74475E-07	-1.628007028	0.123868351	-1.364661598	0.104058872	-0.986914569	0.022857137	-0.985026179	0.00034334	1.035451045	1.80605E-05
AT2G23100	AT2G23100	-1.808306703	0.000710784	-1.037331599	0.064216227	-0.164898973	0.770806433	0.253673913	0.908636304	-0.243959029	0.784525395	-1.001085245	6.40617E-05
AT1G72140	AT1G72140	-1.502095795	0.016841839	-0.470599639	0.772177181	-0.973380945	0.179170732	-0.726872014	0.680482607	-0.296728713	0.796532054	-1.342013256	7.34873E-05
AT1G74670	GASA6	-2.625471573	4.16406E-11	-0.936788558	3.3529E-06	-0.721103883	0.0040226	-0.546941466	0.261605458	-0.147406763	0.747458101	-1.458506842	6.73871E-26
AT1G72180	AT1G72180	-1.116058923	0.000276323	-0.491013277	0.043505796	-0.593190791	0.000275704	-0.533234339	0.598689518	-0.764590604	0.000983778	-1.486276509	2.22621E-15
AT2G36970	AT2G36970	-2.273510943	0.027300053	-0.359276756	0.710151622	-1.713241343	0.440215294	-0.970201656	0.706790319	0.280487373	0.895021414	-1.53090814	0.04191787

AT4G11210	AT4G11210	-6.90841863	0.043586689	-2.017860871	0.659082201	-1.510351763	0.706153047	-1.928173988	0.66808962	0.507301003	0.687877555	-1.687541064	2.80093E-06
AT5G60650	AT5G60650	-7.699126946	0.00235138	-1.019314062	0.855355791	0.354695817	0.893021382	-0.87605991	0.874187681	-1.235177077	0.162394808	-1.748926632	0.017912858
AT3G48700	CXE13	-6.665917181	3.55606E-09	-2.416955958	0.170994673	-0.185536913	0.85688552	-0.210148901	0.982356953	-0.646780978	0.798599938	-2.778903737	0.003213868
AT5G56840	AT5G56840	-7.435148609	0.01113833	-6.560481141	0.143397629	-5.391564048	0.307512202	-4.086917128	0.636072176	-2.357190094	0.725806449	-4.558980135	0.000119391
AT3G44326	AT3G44326	9.985026011	1.06606E-07	1.75352943	0.668904414	0.015325445	0.997135163	-1.288954954	0.74755066	-0.095988455	0.957156296	-0.768519094	0.05245004
AT5G19890	AT5G19890	9.454547715	6.04495E-07	1.396633441	NA	2.773109154	NA	-0.720791992	0.894653614	-2.278078923	0.188884212	-0.740402574	0.248221707
AT1G69920	GSTU12	9.111704953	2.14682E-06	-5.235910174	0.328418104	4.734883457	0.379465519	-2.382013224	0.68789331	-2.015823746	0.10830565	0.597976214	0.597865032
AT3G01795	None	9.045451761	0.000957969	-0.303513943	0.90574659			-0.031598941	0.997449367	1.255045531	0.481635224		
AT2G21900	WRKY59	8.998326034	0.001385791	4.345256869	NA	-4.433215038	0.414155513	-1.798749604	0.788737401	-1.509912405	0.686400884	-2.063162513	0.1737946
AT1G32350	AOX1D	8.875074382	6.45793E-05	4.789211323	0.134265619	3.619352335	0.066884929	-4.120090123	0.474958589	1.913672923	0.817578778	-0.093042789	0.978860866
AT3G05920	AT3G05920	8.846236634	3.38828E-05	0.324612005	0.942332968	0.792620718	0.804175457	-1.547347553	0.79089306	-0.598238403	0.046984347	-0.297240009	0.411361258
AT1G60750	AT1G60750	8.777033188	1.54297E-05	-0.328429755	0.929298011	-1.206847305	0.67072139	-4.107982665	0.062555197	-0.376470789	0.857077081	-0.358659274	0.784157323
AT2G44000	AT2G44000	8.539601346	0.000174216	0.767751199	0.855320256	-0.750620153	0.847376036	-3.054403717	0.637614678	-2.218032287	0.347566674	-1.13236257	0.447993128
AT2G01770	VIT1	8.538936832	0.00684508	-3.761655451	NA	-3.322614377	0.550728949	-1.275368496	0.282829999	-0.01323075	0.993586481	-0.1948324	0.812084632
AT2G34317	None	8.510552983	0.005199512	1.578703131	0.709358034			-3.714699484	0.10756991	-2.021821364	0.25012821		
AT1G32880	AT1G32880	8.49235837	8.42464E-05	2.996842901	NA	0.683481593	0.878089188	-4.861151553	0.672844426	-5.227747414	0.525384544	1.406099278	0.679724019
AT5G64810	WRKY51	8.463199175	0.000219696	-0.402471915	0.940423707	2.631776134	0.30321571	-2.273976164	0.609674706	0.336531092	0.870402458	-1.022832132	0.463513149
AT2G03360	AT2G03360	8.453509486	0.000149799	4.024230278	NA	-2.648405744	NA	-1.664261936	0.685495817	-0.422342971	0.796567249	-0.063726173	0.949429742
AT1G44180	AT1G44180	8.447590681	0.000146627	6.628084864	0.204289868	-1.468231882	0.481261541	-0.572170073	0.928193798	-0.82935395	0.612310929	-2.24609456	0.253327214
AT3G12470	AT3G12470	8.425449092	0.000269035	2.762591953	0.311226507	-5.530112958	0.134310058	-2.215843547	0.820406788	1.117616597	0.736648978	-0.294796009	0.768617623
AT5G37940	AT5G37940	8.391183855	0.007047986					0.019183882	0.99779774	1.537555549	0.537002257	0.762418294	0.710015308
AT5G07390	RBOHA	8.370775503	0.000301376	3.967605489	0.419757542	5.602956397	0.194974087	-1.684653808	0.697407195	-0.83800531	0.564561434	-1.063718353	0.131934508
AT2G07642	None	8.330614476	0.000402752	1.040719925	0.749470035			-0.535574159	0.964175862	-2.421644925	0.533838944		
AT5G40000	AT5G40000	8.305578884	0.00045436	-0.406494166	0.921154475	3.187085306	0.565081887	-1.96266031	0.676109289	-0.197338729	0.975104725	2.392781293	0.178779695
AT1G22670	AT1G22670	8.269643631	0.010242859	7.306948751	0.06246275	-5.42170602	0.264051783			0.622832979	0.962056972	1.135779278	0.704175734
AT4G25380	SAP10	8.249604317	0.000601473	2.634257311	0.452303244	-3.585343201	0.516230268	-0.26646123	0.982356953	-0.723604482	0.917626906	-0.835440655	0.672512218
AT5G47000	AT5G47000	8.215114111	0.000552793	-7.032634727	0.071900703	-5.945258908	0.082350351	-0.26191937	0.990184359	0.491306425	0.959952977	-2.234495356	0.161169186
AT2G02240	MEE66	8.215060735	0.000513688	2.003404654	0.608198323	1.134926743	0.654737871	-0.489972501	0.943927962	-0.605953719	0.906954561	0.916770745	0.596430528
AT3G12880	AT3G12880	8.129880915	0.000599898	2.209679858	0.293491026	2.967432811	0.063292964	-1.548709109	NA	-1.058991172	0.872528185	0.310827147	0.888538076
AT2G45930	AT2G45930	8.110840104	0.00143536	-1.854707831	0.645827721			0.051796319	0.995194798	1.295795263	0.323767852	1.177961515	NA
AT3G60966	AT3G60966	8.089154634	0.01647412	3.818615709	NA	2.274379069	0.47501306	0.923366503	0.876015705	-0.650437673	0.766478769	0.129958901	0.940951769
AT3G47660	AT3G47660	8.073742506	0.016535734	1.323176575	0.685501977	0.619869969	0.69070398	-3.380030163	0.646747644	-0.953334129	0.869626899	1.685148487	0.248696594
AT4G05840	None	8.036570318	0.017868736	3.839363505	0.359540701			-5.837732596	0.62738272	0.834532442	0.775703028		
AT3G10240	AT3G10240	8.013828358	0.01912703	0.665536182	0.919370886	1.358434881	0.829698046			-5.387885547	0.512974126	-3.684443735	0.145120461
AT4G05715	None	7.998610226	0.019066033					2.846742377	0.659255524				
AT4G30640	AT4G30640	7.991323949	0.004503466	-0.068565104	0.990942336	-0.000951354	0.999751719	-2.096294736	0.62738272	0.191555599	0.947748856	-0.331689127	0.632532875
AT5G03204	AT5G03204	7.973261161	0.001298964	6.703848987	0.147727856	-0.051703438	0.980019738	-0.19659671	0.992216145	4.913946044	0.554072186	0.766659702	0.62744417
AT1G73270	scpl6	7.961857395	0.0012483	-4.742700232	NA	-5.835637671	0.078240582	-1.129675516	0.79986937	-0.959043433	0.715948092	-3.214498964	0.062053169
AT3G13432	AT3G13432	7.954135025	0.021125344	0.876840533	0.893781642			-2.224307788	0.704004387	-2.058834342	0.338254673	-2.382211983	0.32024229
AT3G52080	chx28	7.805676509	0.032418415			0.070403266	0.990286483	-6.169958387	0.405880771	-5.376112455	0.513456138	3.078681523	0.62744417
AT2G21420	AT2G21420	7.780811342	0.002808904	3.437254237	0.51864127	3.214066468	0.563907217			-3.877608262	NA	3.827581105	0.510936831
AT2G04805	None	7.780027761	0.033704273	-2.876163836	NA			-0.513047218	0.829607708	-0.064570485	0.97926944		
AT5G06250	DPA4	7.768919683	0.034834576	0.8992463	0.828733221	-0.011380193	0.997546424	2.171076439	0.204145338	0.827377234	0.952661388	0.674140486	0.134266181
AT2G27930	AT2G27930	7.753900757	0.007748175	-6.458900736	0.067683675	2.592648204	0.298472943	-1.642832176	0.684041748	-0.9102056	0.22311972	0.663786152	0.170480714
AT1G66450	AT1G66450	7.73952949	0.037559629	0.73154758	0.892509551	-2.58669937	NA	-0.56781059	0.924948308	-0.132914911	0.927507051	-1.426616831	0.112129571
AT1G44608	AT1G44608	7.732422226	0.033300479	0.943823525	0.807165399	1.960172281	0.214500785	-3.319793329	0.204270555	-1.090480011	0.168946553	-0.712358771	0.191274428
AT3G55210	NAC063	7.732184213	0.003372149	1.322815173	0.759958174	-2.648405744	NA	-4.928463417	0.66808962	-5.639066929	0.491449838	4.826494131	0.052141055
AT5G24410	PGL4	7.714807204	0.035654723	1.513057186	0.774884962	4.380438758	0.419578731	-2.514282047	0.503058648	-2.020948569	0.085434663	-5.433098779	0.210866459
AT1G69470	AT1G69470	7.700205316	0.007306017	2.676443799	0.427746251	2.153405396	0.553598299					-0.908505385	0.495835543
AT5G18661	AT5G18661	7.696125328	0.00638602	2.547102564	0.301158195	4.327246017	0.426259265	-0.981602231	0.866511604	-3.684978705	0.10971002	-1.909412812	0.172520569
AT4G17785	MYB39	7.694771736	0.0359295	-2.032417285	NA	-2.353593325	NA	-0.472443272	0.967195835	-7.11442742	0.058461349	-0.911479788	0.511911351
AT4G16160	ATOEP16-2	7.680271622	0.013579089	2.511691386	0.056841735	1.706679942	0.400740549	2.29535205	0.73148555	-0.871794116	NA	-0.321141544	0.828022313
AT5G04205	None	7.672836048	0.014968148	-2.38531432	NA			1.156713657	0.9487076	-0.128529569	0.988021272		
AT3G01516	AT3G01516	7.660745427	0.004762171	-6.02436495	0.1381815	-4.659589779	0.388183694	-0.874576306	0.66524984	-0.082224591	0.945436408	-0.536804832	0.152038413
AT3G50940	AT3G50940	7.62300633	0.007818142	0.674719985	0.882726036	0.799639692	0.73212911	2.265146518	0.677952687	0.424539738	0.924140734	-1.254597248	0.439961558
AT4G00600	AT4G00600	7.610720078	0.010053476	4.975671495	NA	1.815387117	NA	-5.239376593	0.653363031			1.928156229	0.77280831
AT5G63660	PDF2.5	7.609155894	0.043877437	-5.051461836	0.348127112	-0.600296217	0.90090152	-1.374758872	0.076422125	-0.395273081	0.594112049	-0.312965896	0.435559662
AT3G49480	AT3G49480	7.595785711	0.009796765	2.738778745	0.628733808	-3.633033911	0.510150325	2.903611677	0.809492797	-0.934954716	NA	0.340497907	0.951347321
AT1G56555	AT1G56555	7.590405257	0.006026487	1.914336992	0.74138954			-4.446520998	0.696374342	-3.117262929	0.391506399		
AT3G53820	AT3G53820	7.564787961	0.046438105	5.064781048	0.346189762	-4.433215038	0.414155513	-4.446520998	0.696374342	-3.515617394	NA	-2.627417824	0.371291656
AT1G61930	AT1G61930	7.561839152	0.014546832	0.38213992	0.953928951	4.148859113	0.445672623	-6.8059717	NA	0.636063281	0.888051403	-0.160455466	0.915976652
AT4G11655	AT4G11655	7.551626902	0.007494305	-2.55453064	NA	-4.648399821	0.389481017	-0.224059125	0.977607985	-2.261744555	0.338125494	-1.451799056	0.28988835
AT1G67290	GLOX1	7.551317754	0.047206435	5.124969061	0.061136529	0.815496959	NA	-5.779009005	0.629761053	0.667263373	0.941261327	0.39000964	0.930514869
AT1G26790	AT1G26790	7.477452211	0.009781513	-4.591953932	NA	1.511572084	0.639338151	0.843041169	0.915552048	0.020928988	0.99766313	1.122400583	0.778521154
AT1G18510	TET16	7.442652094	0.024335648					-4.066128103	0.717709314			2.469806537	0.681264476
AT4G10860	AT4G10860	7.436287406	0.010847625	1.185724665	NA			-5.775770899	0.629761053	-0.43075978	0.961513155	2.194323713	0.128840016
AT3G47030	AT3G47030	7.395625999	0.011179658	-0.314910761	0.95817051								
AT5G59390	AT5G59390	7.384907892	0.01461805	3.23743934	0.125114544	2.609674877	0.208383828	-0.193367932	0.982356953	-2.181829712	0.334330187	-2.297366526	0.068103222
AT1G08903	None	7.373547297	0.019829795	2.455612356	0.650946443			-1.148261516	0.794188368	-0.657163452	0.811237669		
AT5G38910	AT5G38910	7.355764136	0.013698121					0.851783142	0.954974702	-1.942334302	0.818586652	-0.050238488	0.991296529
AT4G11170	AT4G11170	7.315483784	0.022527786	3.548511695	NA	3.22572152	0.529963262	-2.357238451	0.565728752	0.051502375	0.983185114	0.470250266	0.630172638
AT5G63270	AT5G63270	7.180087382	0.021808259	5.878862555	0.267759424			-3.703226371	NA	-1.075408427	0.935104348	-3.910320644	0.135043215
AT3G50200	AT3G50200	7.171943796	0.022580042	0.782323214	0.746401863	-5.518789436	0.233705882	-0.810623495	0.934000827	0.712611066	0.73834094	-0.558075442	0.847459501
AT5G62080	AT5G62080	7.162075707	0.025918308			1.860572935	0.759729464	6.772984811	0.579644733	-5.77143003	0.461651524	-1.545875838	0.669845049
AT2G36260	AT2G36260	7.152891866	0.037342061	3.587048631	0.391293567			3.299188355	0.770575751	-5.859668614	0.374204613	3.609488397	0.556583235
AT5G41663	ath-MIR319b	7.152555603	0.025424766	-1.555878094	NA	-2.648405744	NA	1.099935611	0.951485287	1.288953909	0.710782334	0.363234206	0.836654871
AT1G62580	NOGC1	7.12470538	0.002124582	-0.576678619	0.911031518			1.196578043	0.827744348	-0.933376793	0.831572245	0.545686113	NA
AT5G40010	AATP1	7.114279699	0.032083109	-0.44788467	0.935492382	-4.709153354	0.382234414	-3.419728077	0.559215018	-1.069241914	0.934609223	-1.045017815	0.604911976
AT3G54240	AT3G54240	7.103315329	0.033803104									-2.226156597	0.738428166
AT1G70260	UMAMIT36	7.075363363	0.033115868	-6.543433743	0.144840341	-6.110308208	0.119393414	-1.926782563	0.652047062	-1.438106392	0.620431294	0.49496972	0.621024452
AT4G18980	AtS40-3	7.060902641	0.033914187	6.011019131	NA	-4.459045057	0.4113278	0.402480301	0.964220939	0.785388113	0.951101524	0.155352245	0.95399871
AT1G79580	SMB	7.056718799	0.034580654	-4.723239303	NA	-4.71157708	0.382173607	0.610520553	0.931898506	-1.083188432	0.645084436	-2.650121086	0.069293943
AT4G25000	AMY1	7.026604834	0.034233955	-2.543056742	0.378403875	-2.242707603	NA	-0.684495041	0.9572994	-0.206196404	0.925831983	-0.608667506	0.622032827
AT2G26610	AT2G26610	7.006241573	0.043115515	7.075582148	0.170060868	-1.108056393	NA	-5.705481965	0.632278424	4.418165677	NA	1.364330033	0.662802557
AT1G52330	AT1G52330	6.996468452	5.40096E-05	-0.572039324	0.922060792	-0.195968174	0.962700052	-0.342938863	0.962474985	0.432752689	0.689027214	-0.835508504	0.120066567
AT2G07741	AT2G07741	6.913337749	0.04774532	1.421707572	0.716333445			-1.335567226	0.881491928	-1.640596121	0.711567545		
AT5G26690	AT5G26690	6.897187748	0.044796663	7.132543979	0.079414437	1.012809914	0.789297179	-1.926586411	0.771494464	0.164442972	0.986818647	-1.009908494	0.46579554
AT5G26150	AT5G26150	6.704444745	0.007911366	4.509690938	0.316103626	-0.696624968	0.792057077	-6.56386973	0.583897164	0.448194027	0.957609656	-3.240136462	0.225850344
AT2G26820	PP2-A3	6.070434754	0.016326401	3.058706994	0.591201483			-2.340390565	0.715099832	-1.869950876	0.308270854		
AT2G35670	FIS2	6.026619343	0.000710016	3.334721686	0.311701496	3.760221429	0.105098019	-4.289184143	0.705076831	6.825526551	0.123803529	1.309159059	0.627280037
AT3G28620	AT3G28620	5.931167131	0.0024653	0.727289716	0.667239715	1.499105861	0.386064994	1.515334483	0.766274785	0.281976052	0.95452954	0.022001329	0.985575436
AT2G07773	AT2G07773	5.897542601	0.008538618	-4.570368553	NA			-1.380668319	0.91867804	0.683510846	0.924543846	-4.425818518	0.450770561
AT5G37485	AT5G37485	5.774685068	0.009955404	1.37081983	0.533127389			0.8155426	NA	-0.375874068	0.931660636		
AT5G25640	AT5G25640	5.47924167	0.023103572	-1.321603471	0.800229605	0.593892853	0.713765884	-0.83279265	0.894700903	-0.103478669	0.977721011	-0.237964912	0.662236256

AT2G07728	AT2G07728	5.327218943	0.004063213	4.783807469	0.134844751	-1.108056393	NA	-0.230082961	0.978043477	0.724362154	0.775432933	-2.821006508	0.076027373
AT3G47200	AT3G47200	5.274845778	0.002812177	1.716481281	0.738801352	0.202760105	0.882499818	-1.357491394	0.731041294	-0.410132245	0.633120941	-0.823752459	0.000139633
AT4G31330	AT4G31330	5.195016602	0.001402556	-0.126006607	0.976592039	-0.183425757	0.945136989	-1.000967261	0.637614678	-0.734836667	0.53525964	-0.933493584	0.034984261
AT3G46730	AT3G46730	5.008891737	0.013855838	1.616650755	0.516982537	-0.680837684	0.46470787	-0.135039991	0.980070246	0.56465462	0.905209075	-1.56827279	0.193506326
AT2G28420	GLYI8	4.978583958	0.021123253	0.798690265	0.764612718	-1.103102511	0.610339589	-0.199166163	0.986012712	-0.546892966	0.924868537	-1.052468632	0.250781381
AT4G10843	None	4.815245731	0.038399087	1.670216687	0.229062709			-0.609614864	0.935551609	0.449791469	0.834150524		
AT2G18000	TAF14	4.792404895	0.020029711	-0.616955726	0.819430334	-1.981926143	0.414273168	-1.201547074	0.79990092	-0.392489636	0.770008565	-0.14291772	0.831943502
AT2G01175	AT2G01175	4.785299896	0.019413402	2.481571968	0.382152221	-0.750243226	0.809701832	0.873566386	0.782701965	0.430770901	0.865593881	-0.124487164	0.905488956
AT3G04115	None	4.767824398	0.010916403	2.879973638	0.101830109			-1.674225963	NA	-0.719456715	0.630207891		
AT1G52343	AT1G52343	4.610294499	0.01127649	3.096596837	0.08010244	1.631422884	NA	0.474531514	0.854730188	0.459562815	0.618270343	-1.988042102	0.066971522
AT3G28600	AT3G28600	4.594141782	0.014254466	4.306087201	0.105812132	2.509230223	0.452570091	-0.862203864	0.93556951	-2.613800108	0.503946392	-0.635633323	0.289175087
AT5G46871	AT5G46871	4.574028331	0.024065953	0.854136069	0.766022251	1.815387117	NA	-1.32104585	0.774188277	0.743415019	0.673297356	2.559238023	0.204389125
AT5G51451	RGF5	4.569495975	0.04309198	0.645472706	0.610769576	-0.10382021	0.966242667	0.065354449	NA	-0.194336488	0.942567361	1.035570724	0.149881903
AT3G24900	RLP39	4.460731508	0.006797584	1.094520034	0.685714658	-0.236579313	0.948386376	-1.71570452	0.826650676	7.198450496	NA	0.13884126	0.920355944
AT2G36650	AT2G36650	4.421943226	0.027433453	1.133048091	0.736800872	0.155211882	0.962252946	0.088606374	0.991886227	0.309906763	0.918766875	-0.806439961	0.359440074
AT5G64590	AT5G64590	4.281946673	0.034819656	0.449317305	0.888528145	2.000643722	0.652422463	-1.888387282	0.652047062	-0.365880803	0.854148094	1.177961515	NA
AT3G51750	AT3G51750	4.015396935	0.007244963	2.164643594	0.074628571	-1.466604115	0.154645118	-1.037282771	0.876015705	-0.910280047	0.605502385	-0.507424977	0.56864071
AT3G46700	AT3G46700	3.99433958	0.042326683	1.484232867	0.316103626	6.294188528	0.157886521	-0.460455222	0.820406788	0.078450507	0.952732555	-1.434983025	0.052271466
AT4G37140	MEE69	3.953792624	0.043727766	-0.401059474	0.919513183	-1.187793672	0.745879114	-1.263728822	0.927699035	-0.659301797	0.929498792	0.25777274	0.864771454
AT5G24910	CYP714A1	3.822930415	0.001431554	1.397152463	0.121061162	1.915553195	0.097321083	-0.02968702	0.993687851	-0.083708181	0.949463042	0.093263697	0.809288683
AT4G31351	AT4G31351	3.750892126	0.024020442	1.133305603	0.41783681	-5.802413016	0.172997545	-0.242490049	0.972495118	-0.226936282	0.876125588	0.77436494	0.71308565
AT1G09647	None	3.661130421	0.045653173	1.925598753	0.168502922			-0.970477028	0.920836073	0.350264613	0.933194561		
AT2G40200	AT2G40200	3.599821783	0.004579031	1.597009209	0.427002427	1.586048853	0.298781716	0.239578766	0.852449542	0.423264169	0.53583656	-0.529923251	0.067546034
AT1G12630	AT1G12630	3.588255599	0.048690103	0.172299441	0.957278713	0.869461254	0.511946084	-0.869999211	0.888051184	-5.416878679	0.358196097	-0.660112199	0.366416733
AT2G32660	RLP22	3.53960318	0.008315314	0.265085639	0.859276802	0.640202505	0.514630728	0.599390371	0.857472027	0.019028472	0.994764946	-1.400485426	0.15845484
AT3G59220	PRN	3.4409688	5.48764E-05	1.161095095	0.250978388	-0.196056183	0.947251726	-1.279411371	0.743032554	-0.390784946	0.560637517	-0.278246151	0.393594342
AT5G59940	AT5G59940	3.436436461	0.000383701	0.458956513	0.871633466	0.060553821	0.972171219	0.234289764	0.939540455	-0.658971011	0.725163011	-2.834800021	0.129156983
AT2G29410	MTPB1	3.428640173	0.002708386	1.591200124	0.127351336	2.33869604	0.182984319	0.938800363	0.604422229	-0.918102233	0.498449509	0.351566931	0.735373105
AT3G20898	AT3G20898	3.27417009	0.027412687	-0.040237273	0.989526668	-2.690060977	0.134655094	0.390936285	0.912756407	0.959005989	0.312492273	-0.261177821	0.683373259
AT3G59700	HLECRK	3.19256915	0.011369421	2.403262709	0.082296538	1.921205847	0.061959496	-0.487258508	0.860750349	-0.920548929	0.202619495	-0.883287453	0.014275758
AT2G14160	AT2G14160	3.136564393	0.033766227	-0.169312035	0.948570401	1.974831804	0.669856639	4.045314963	0.266366767	-1.825529638	0.683406479	2.093620192	0.756708586
AT2G02850	ARPN	3.058346541	0.007153418	1.017011942	0.400612762	-1.399937858	0.616713539	-0.731579591	0.870136387	-0.167174052	0.786729721	0.225271935	0.461950754
AT4G13660	PRR2	3.02966792	1.22406E-12	1.04531549	0.109524771	0.663290445	0.401849692	-0.628751893	0.676109289	-0.286332065	0.319908376	-0.885218582	2.85755E-06
AT2G07662	None	2.991015375	0.01562081	2.106580649	0.124159711			-1.222963543	0.778726911	0.042816324	0.985787245		
AT5G53980	HB52	2.975281972	2.40813E-05	0.345468166	0.702590264	-0.325889505	0.622803532	-0.78956344	0.735038565	0.150489311	0.677593345	-0.270334591	0.36502435
AT3G20865	AGP40	2.955530657	0.003242904	0.836031645	0.614665945	-1.275764221	0.493152811	0.730596033	0.855081587	0.963447243	0.434411297	0.242641462	0.743552743
AT3G15357	AT3G15357	2.953304378	0.043532209	2.628867504	0.121918554	0.51426779	0.646222228	-0.074746502	0.984525706	-0.422486704	0.722116343	-0.790271366	0.252764936
AT1G30730	AT1G30730	2.924961755	5.24688E-06	0.318486025	0.805524372	1.164185887	0.311193474	-1.43568691	0.386146892	-0.26504316	0.872528185	-0.93501725	0.119282882
AT4G04810	MSRB4	2.888966733	0.000254086	-0.030071105	0.984617573	1.119463201	NA	-0.64121743	0.786649032	-0.073046449	0.953839888	-0.76719834	0.018137788
AT4G08555	AT4G08555	2.863585233	0.001366167	1.240412645	0.263254523	1.447119091	0.209728981	-0.795540546	0.74868355	-0.492162455	0.707175415	0.837023821	0.126428763
AT5G59090	SBT4.12	2.824490533	0.000550419	0.441471372	0.512805574	-0.653650126	0.492093251	-1.454493088	0.511554278	-0.739633882	3.39517E-05	-0.4453293	0.00717757
AT2G19580	TET2	2.735077317	0.027929185	0.688237518	0.803159356	0.87070052	0.69211533	-0.952023955	0.644839236	0.004300938	0.998625192	-0.662213763	0.016080731
AT3G63280	NEK4	2.600533956	0.002979389	0.619477511	0.282528618	0.805204328	0.049956558	-0.323005902	0.706790319	-0.286097029	0.593079802	-0.377266696	0.027272604
AT2G44790	UCC2	2.53363342	4.47972E-13	-0.461903524	0.467246125	0.763947302	0.054197482	-0.943754645	0.62738272	-0.933751118	1.01311E-09	-0.574009668	5.93448E-06
AT5G18270	ANAC087	2.526598503	0.003216456	-1.787485405	0.30930998	1.177623177	0.22517316	-1.519047015	0.408213729	-0.257224249	0.741935896	-0.626432507	0.234911907
AT1G07187	None	2.521173309	0.006925801	-0.11173301	0.920752431			4.280047583	0.197650176	0.834726742	0.815592903		
AT5G42090	AT5G42090	2.472563968	0.003874299	1.045006607	0.505009596	0.320117911	0.309323826	0.00800567	0.998732347	1.019550518	0.265754101	-0.906671204	1.00898E-09
AT1G30650	WRKY14	2.393016962	0.000997331	-0.926606096	0.288321173	-0.450857314	0.713699901	-0.924462838	0.704225272	-0.530449469	0.241208566	-0.585580609	0.006303365
AT4G14465	AHL20	2.356700115	0.00045021	0.767951897	0.256179416	-0.72792728	0.52500442	-0.725318148	0.797097845	-0.335329603	0.107269164	-0.831099517	1.97733E-11
AT2G04800	AT2G04800	2.354134628	0.000997331	-0.389469991	0.75539002	4.286651146	0.430047478	-0.666560342	0.741727789	-0.037511461	0.976227734	-3.354701917	0.330265139
AT4G01440	UMAMIT31	2.334392469	0.000934243	1.247086446	0.182290739	-0.413847036	0.616539116	-0.434095947	0.724234753	-0.649867192	0.231454365	-0.867880978	0.000272896
AT3G25730	EDF3	2.333240962	0.000122366	0.773178996	0.251819284	0.613052316	0.431644659	-0.513500089	0.787239413	-0.018893886	0.980533189	0.06552511	0.831550853
AT5G06990	AT5G06990	2.290180021	0.038212538	2.909448864	0.06085876	0.998160703	0.716207539	-1.349082171	0.674766805	-0.049587902	0.989242512	-0.103643481	0.921780637
AT3G15800	AT3G15800	2.232557753	0.039140968	1.225366992	0.324463318	1.351438434	0.111935187	0.817469109	0.642268593	-0.03158746	0.989242512	0.169382549	0.838875738
AT2G36120	DOT1	2.162840369	2.56787E-05	0.0735884	0.914040617	0.001657363	0.998042972	-1.013196813	0.667958595	-0.112955338	0.936554664	-1.420322814	0.099788022
AT1G61370	AT1G61370	2.158566547	0.00055852	0.862043436	0.338425474	-0.089457902	0.846028847	-1.330558645	0.218297828	-0.427327837	0.613678565	0.087403717	0.763774037
AT4G14130	XTH15	2.119430044	1.53518E-24	1.752231949	0.063337284	0.961173777	7.68507E-10	-0.37548643	0.675671944	0.213575624	0.23157283	-0.123839455	0.687450253
AT5G55090	MAPKKK15	2.073737435	0.023909708	-0.64729187	0.721493317	1.072066509	0.400048268	-0.352274215	0.890241742	-0.111181725	0.961615657	-0.554134475	0.251659074
AT4G11350	AT4G11350	2.069436843	0.022142221	0.930268999	0.448398578	1.04499477	0.327904934	-0.722982863	0.901575434	-0.460494018	0.680312145	-0.529555268	0.091043757
AT2G14900	AT2G14900	2.068108264	0.008545221	0.282826746	0.765570525	0.607497143	0.409776771	-0.717143405	0.665778009	0.107190515	0.65368445	0.566275136	0.007057564
AT1G58410	AT1G58410	2.044811834	0.025446156	0.012897169	0.991007282	0.111726731	0.932348482	-0.920571419	0.226580455	-0.108447435	0.909221761	-0.840911504	0.002966818
AT1G04920	SPS3F	2.043739028	0.032164215	1.360668429	0.248302141	0.526454742	0.799033433	0.057687463	0.996799452	2.790373049	0.144543426	0.838553791	0.439877089
AT3G62270	AT3G62270	2.040857996	0.001879456	-0.796758301	0.273295291	-0.466340123	0.640967065	-1.108785166	0.616538576	-0.724556678	0.022744601	-0.729496137	1.84889E-05
AT3G06035	AT3G06035	2.034657761	0.012258275	0.173242596	0.888763742	-2.125352866	0.129003423	-1.338674157	0.589531904	-0.083439869	0.938703599	-0.3433362	0.242081485
AT4G31408	AT4G31408	2.032512054	0.002291259	1.297116081	0.054027568	1.840422325	0.485119037	0.161051177	0.951485287	0.222996046	0.799682552	1.392657747	0.733695779
AT1G14687	HB32	2.006063274	0.004404426	1.355172772	0.127747156	0.059276996	0.941342712	-0.213935496	0.962872831	0.035366105	0.975427751	-0.578316775	0.026668843
AT5G65870	PSK5	2.002005862	0.042641054	0.904196894	0.311461713	1.011361087	0.344568018	-0.812729021	0.704225272	0.009409534	0.992575938	-0.426162179	0.118096427
AT3G14780	AT3G14780	2.001928185	0.024628498	-0.14256678	0.907755503	0.674646731	0.368921589	-2.518687568	0.501211524	0.537946632	0.658178574	-0.780135924	0.140742391
AT5G20420	CHR42	1.990059208	0.006792573	0.861004261	0.182783765	0.615646334	0.35709046	-0.17387666	0.933020011	0.053763979	0.963419708	0.026590532	0.958043778
AT3G01350	AT3G01350	1.985549282	0.027778715	0.147207596	0.944941601	-0.3597659	0.780809506	-0.59310883	0.736950721	-0.342194232	0.852137227	-0.862943465	0.088516353
AT3G41762	AT3G41762	1.892774503	0.029681838	3.397010596	0.11181077	1.514186352	0.115259164	-1.083381677	0.637815859	0.157467327	0.930083708	-0.583874513	0.115294665
AT2G28910	CXIP4	1.87371336	4.90665E-10	0.807262169	5.27177E-08	0.808350308	3.8503E-08	0.121841462	0.936758504	0.239903288	0.380571063	-0.960573021	0.092890692
AT2G32280	AT2G32280	1.844838493	0.020846393	-1.370362086	0.154036715	-0.441841174	0.694787538	-0.817049242	0.86916879	-0.010642126	0.991628492	-0.381815708	0.08979867
AT1G14870	PCR2	1.842985891	2.40721E-05	0.775942823	0.057456791	0.704954642	0.017993071	-0.138995868	0.963647885	-0.540677851	0.099448789	-0.731795535	0.050556172
AT5G14000	NAC084	1.808195779	0.000439561	0.563736874	0.501943754	0.750687686	0.351481044	-0.218494713	0.918165358	0.529562041	0.043844505	-0.868036492	2.65339E-05
AT3G55230	AT3G55230	1.766207691	0.015966707	-0.208444412	0.794613263	-0.061997034	0.956947319	-1.60046152	0.092759749	-0.589537746	0.002832318	-0.936398616	3.91475E-08
AT2G35540	AT2G35540	1.765307782	9.06056E-05	0.960465754	0.020909969	0.269203348	0.632646919	0.71511556	0.613859108	0.17868648	0.829152807	-0.606573921	0.135290303
AT4G22710	CYP706A2	1.764126332	0.000317831	0.525201361	0.486869533	0.274074247	0.968396887	-1.302396942	0.552832084	-0.969126913	0.017774439	-0.676460459	0.151898399
AT3G21260	GLTP3	1.759363074	0.000539887	0.884970512	0.007260973	0.177062433	0.573742504	1.152097814	0.108407947	0.551169447	0.029374753	-0.937004999	4.90919E-05
AT3G49601	AT3G49601	1.757126794	3.75216E-07	0.777420712	4.60358E-06	0.811366847	5.06958E-05	0.870429739	0.518021029	0.1371789	0.770483892	-0.380551503	0.207232443
AT1G67105	AT1G67105	1.754875553	0.000684078	0.211605876	0.768207562	1.069792151	0.130374741	0.177204907	0.953275639	0.135171633	0.858274732	-0.978464865	3.34096E-13
AT2G33410	AT2G33410	1.744436246	4.12896E-11	0.781574943	0.000672439	0.781301857	3.74283E-05	-0.244736893	0.74868355	0.22397669	0.495576001	-0.470239775	0.042032803
AT1G67265	RTFL21	1.74053473	0.000494246	0.713603639	0.153912232	0.300265015	0.353462581	0.278954405	0.695554054	0.341597053	0.31350844	-0.45720724	0.132153085
AT3G09270	GSTU8	1.739094292	0.006801629	0.770058558	0.404677029			-0.555291197	0.709790565	-0.065120228	0.919937283	-0.168947199	0.868390224
AT5G46590	NAC096	1.728000609	0.048529346	0.52309571	0.874150892	0.210971852	0.843573913	-2.229269388	0.598901706	0.527008634	0.674895041	0.232973441	0.777660396
AT3G06490	MYB108	1.722143698	0.008523315	0.620190306	0.62821357	-0.2814123	0.816337036	-0.661818384	0.826454915	1.246778729	0.155471451	0.497079298	0.24984202
AT5G15120	AT5G15120	1.718701827	0.01647718	0.842890385	0.111457174	0.44003882	0.320090018	0.053765088	0.982183273	-0.07269793	0.923631194	0.802510082	1.82558E-07
AT5G20165	AT5G20165	1.71383627	3.47592E-05	0.799887716	0.003293721	0.453734878	0.0240239	0.35210245	0.647947756	0.227533992	0.562253128	0.248777945	0.258017322
AT1G02900	RALF1	1.700558884	0.01555037	-0.580833678	0.598041208	-0.406745533	0.573903994	-0.946762012	0.657637951	-0.227530538	0.614717559	-0.797241386	2.64053E-05
AT1G09480	AT1G09480	1.698410643	0.028169515	0.249649335	0.713875335	0.67496347	0.354621947	0.57694219	0.643303174	0.014718886	0.992128549	-0.595939631	0.155249915
AT1G77280	AT1G77280	1.685802515	0.003330078	0.826962875	0.026791373	0.607239664	0.17747575	-0.222126919	0.8904839	-0.106404793	0.876654188	-0.535695264	0.009816451
AT3G18020	AT3G18020	1.683241524	0.03690326	0.282231213	0.786124385	-0.683031932	0.30460058	0.779635901	0.687176366	0.39380242	0.714322744	-0.287454918	0.554229605
AT3G57600	AT3G57600	1.669061387	0.017116451	0.705074128	0.41291054	1.201842289	0.147884764	2.185599458	0.576142947	1.279888207	0.509638584	0.878372746	0.544374174
AT5G20700	AT5G20700	1.668245135	0.001979356	0.996490001	3.1514E-07	-0.003282694	0.994398745	-0.749644865	0.644113891	-0.001731597	0.997749369	-0.650568379	1.32821E-05

AT1G77170	AT1G77170	1.646596649	0.008338495	1.206005681	0.092210316	0.700529288	0.373815549	0.214821954	0.951768175	-0.143162068	0.894924841	0.121973503	0.780422638
AT5G63830	AT5G63830	1.646519438	0.018123514	0.768454783	0.055529312	0.413088384	0.318529825	0.548893281	0.666155455	0.063486758	0.951393667	-0.125331622	0.638875589
AT5G58375	AT5G58375	1.641912027	3.34582E-06	0.898613843	0.000171571	0.883700485	0.000525502	-0.100469954	0.93735704	-0.008697546	0.992163494	-0.40933797	0.075272875
AT3G10190	AT3G10190	1.639841728	0.008449777	0.521345294	0.248745292	0.646923099	0.118436606	-0.255864191	0.876276863	0.155645203	0.737214865	-0.012215331	0.961518035
AT4G23420	AT4G23420	1.637091073	0.004589816	0.942705362	0.173752824	0.500889871	0.472658038	-0.575699719	0.706790319	0.226379449	0.812750957	-0.730215644	0.011453423
AT1G12440	AT1G12440	1.634780076	1.05126E-06	0.946126552	3.53155E-10	0.865198432	8.0799E-10	1.075077717	0.532231419	0.156406052	0.491768843	-0.339990997	0.006515661
AT1G20900	ESC	1.629512029	0.010744878	-0.256435183	0.804677888	-0.077600151	0.946540611	-1.21394276	0.532231419	-0.20850936	0.485963765	-0.348281874	0.042182179
AT2G33480	NAC041	1.624308471	0.024643989	0.737503556	0.151379895	-0.084454816	0.904069478	-0.036381697	0.982749202	0.169392647	0.708114422	-0.730210625	0.009494269
AT3G63110	IPT3	1.609884024	0.045653173	1.300137133	0.053418055	-0.66985475	0.445359174	-0.233388981	0.915508397	0.162442127	0.794547505	-0.619726013	0.00100579
AT1G16480	AT1G16480	1.609328376	0.020930358	-0.088427707	0.929092989	-1.214475975	0.128621485	-0.850483998	0.867112468	0.76204128	0.500891025	-0.426617538	0.218834929
AT2G20940	AT2G20940	1.597384964	0.022955307	1.286435933	0.057231528	0.609318766	0.219110393	-0.142074169	0.947241707	0.345421967	0.503113055	-0.01426562	0.966348787
AT3G02800	PFA-DSP3	1.5790793	0.040892756	1.163199523	0.182539024	-0.074804909	0.946044165	-0.851068386	0.482795025	-0.377772699	0.693137369	0.589969279	0.13499218
AT5G49440	AT5G49440	1.572912073	1.38928E-11	0.795529698	2.68227E-05	2.829905263	0.213886224	-0.232559267	0.709586436	0.109135216	0.72447373	-0.85167229	0.718024439
AT1G10210	MPK1	1.560404392	0.001260201	0.985086986	0.001083597	0.919310074	0.005419976	0.51082954	0.664635521	0.10475748	0.874178263	-0.195149496	0.421822804
AT1G78930	AT1G78930	1.547833889	0.005452283	0.153367383	0.864267618	0.124647879	0.811831879	0.458845435	0.665778009	0.065994193	0.952784918	-0.033717571	0.92203865
AT5G10960	AT5G10960	1.53743055	1.44883E-07	0.886320872	2.09263E-13	0.719087146	1.60156E-05	0.363106216	0.72835818	0.295903515	0.154073947	-0.568921214	0.002074387
AT2G31560	AT2G31560	1.531302858	0.023269677	0.769157591	0.312204977	0.984853527	0.092553477	-0.941051522	0.374426008	0.249624914	0.792830015	-0.139836888	0.644754765
AT1G34180	NAC016	1.525520241	0.046067404	1.343228065	0.15340719	0.987761229	1.39617E-05	-1.8976624	0.159803622	-0.269808795	0.865064302	-0.752689586	4.2983E-08
AT4G39190	AT4G39190	1.519366035	0.028536262	0.118263376	0.89578614	0.820022785	0.034115741	-0.174688941	0.919009651	-0.23015601	0.775734843	-0.596632871	0.033904333
AT2G12550	NUB1	1.513572973	0.048238749	0.691020043	0.160396236	0.329724347	0.490582102	-0.398496164	0.621238589	0.108954362	0.871642493	-0.564300431	0.026139244
AT1G44810	AT1G44810	1.50897642	1.2668E-05	0.554857934	0.018782574	0.760988232	0.00206874	0.326405631	0.712887303	0.308297315	0.199899627	-0.250738044	0.347673603
AT2G20550	AT2G20550	1.499791335	0.005687734	0.695717089	0.137953854	0.592090988	0.140324885	0.072691816	0.962572752	0.030460929	0.972421502	-0.008998216	0.974950673
AT3G21740	APO4	1.495843132	0.000375001	0.756203657	0.020241869	0.151830157	0.693265522	0.563601814	0.610227484	0.189344756	0.656106268	0.511414775	0.012576256
AT3G44798	AT3G44798	1.495190943	0.000318363	0.79882366	0.022594625			-0.128747135	0.906077763	0.202462907	0.741311457		
AT5G07070	CIPK2	1.494220508	0.036711712	0.557668177	0.58195626	-0.234860142	0.623915738	0.390748079	0.850083667	-0.94062631	0.20619067	0.304026988	0.315628316
AT3G03520	NPC3	1.493036788	0.011406001	0.682342779	0.047671255	0.238440997	0.573217775	-0.683204234	0.740082034	-0.473876324	0.028482593	-0.494468195	0.013182613
AT1G03970	GBF4	1.492431039	0.00643843	0.992197163	0.014873251	0.84913896	0.050643459	-0.069549131	0.979620414	0.128329925	0.871112567	-0.012102106	0.97927969
AT2G19350	AT2G19350	1.490831416	0.003480693	0.698575952	0.075819074	0.979157014	0.005429082	0.595315209	0.652047062	0.188571594	0.784261917	-0.450704391	0.05850501
AT1G03940	AT1G03940	1.489228343	0.010096142	0.304235204	0.701920702	0.11390774	0.873970772	-0.523924559	0.767697725	0.270594125	0.741744418	-0.511224543	0.178288367
AT3G47120	AT3G47120	1.482688798	0.000219542	0.54766363	0.178998669	0.055169819	0.921610788	0.471806923	0.760059267	0.20811274	0.770136717	-0.584383489	0.018649836
AT4G34680	GATA3	1.479981648	0.006772297	-0.423008372	0.433778082	-0.540534366	0.214152484	-0.236945497	0.847660958	0.210838519	0.801878575	-0.920341509	2.79013E-07
AT3G01400	AT3G01400	1.478549888	1.47284E-05	0.886879845	0.002940273	0.846100619	8.93431E-07	0.363401473	0.799533905	-0.247506953	0.474847187	-0.861964874	7.67315E-08
AT3G56030	AT3G56030	1.475238923	0.036917243	0.515433217	0.520793163	0.504193663	0.218988257	-0.27943468	0.907707664	-0.714122864	0.284682645	-0.543314127	0.102612934
AT3G47965	AT3G47965	1.464287778	0.000213402	0.908268989	0.004518726	0.865069233	0.800000347	0.926898617	0.532231419	-0.034937781	0.969622714	-1.020134435	0.455198411
AT1G80830	NRAMP1	1.453537516	8.04046E-05	0.56785414	0.067376507	0.956190928	0.006224621	-0.516020734	0.745375315	-0.397956907	0.127559438	-0.895974248	4.25091E-07
AT1G04010	PSAT1	1.451983055	0.015751448	0.685380219	0.09892662	0.907069548	0.014337518	0.403078059	0.712887303	-0.176577072	0.866906715	0.087124732	0.796908831
AT2G40230	AT2G40230	1.450847433	0.02732061	-0.189546875	0.85594543	0.468862503	0.553693559	-0.915557034	0.268371614	-0.07965013	0.939203962	-0.917251318	0.000797458
AT3G62460	AT3G62460	1.449274261	0.023895948	0.424760843	0.304437514	1.538981995	0.068412058	0.12920688	0.929026631	0.347243586	0.474544596	-0.134039384	0.865883226
AT2G33830	AT2G33830	1.446048966	9.60495E-06	0.619261316	6.18557E-08	0.590664508	0.286136072	0.575446885	0.657637951	0.159844626	0.417651934	-0.167887068	0.379811469
AT1G56220	AT1G56220	1.44507072	0.000287762	0.991982062	1.48069E-06	0.658192685	3.80747E-05	1.033787635	0.433621295	0.293758235	0.054308609	0.413085123	0.017369097
AT5G18900	AT5G18900	1.434331257	0.000363448	0.933467496	0.000700417	0.160261038	0.611267994	0.077298571	0.950356778	-0.128418504	0.771376139	-0.361932055	0.026957864
AT1G34418	AT1G34418	1.430341872	0.005741934	0.659999552	0.013626477	0.822342001	0.002857245	0.004557958	0.997796217	0.474196293	0.040223026	-0.636755988	0.024646087
AT3G55460	SCL30	1.424723716	1.33246E-08	0.872091611	5.77611E-08	0.942694808	1.24013E-11	0.369379553	0.715330311	0.224461669	0.291060321	0.167306221	0.229088213
AT5G14440	AT5G14440	1.420892099	0.000607193	0.579946223	0.05554891	0.99506044	2.77742E-08	0.25502464	0.705188505	0.161505245	0.691313385	-0.247739105	0.719365777
AT4G18260	AT4G18260	1.418933406	0.014003554	0.260909122	0.775110744	0.207859977	0.531783057	-0.254839822	0.93556951	0.01460709	0.985852126	-0.005182018	0.985546163
AT1G61040	VIP5	1.409685445	5.30915E-09	0.668486743	0.001491401	0.92409542	1.53695E-07	0.196946852	0.845215023	0.181415919	0.532046002	-0.196467087	0.278237473
AT1G28760	AT1G28760	1.398204841	0.039381947	0.577739836	0.460011796	-1.073838625	0.099749106	0.488088087	0.771670925	0.144613461	0.923979325	-0.039875659	0.925942539
AT2G02230	PP2-B1	1.395496195	0.004800177	0.942302086	0.013541869	0.433612262	0.383650698	0.327118165	0.857647179	-0.327079422	0.509409433	-0.780225845	0.013687278
AT5G25890	IAA28	1.392204721	0.040656286	0.148179958	0.83666283	-0.461101949	0.198232639	-0.243509249	0.911720308	0.06252788	0.896187586	-0.203896962	0.295607317
AT3G05945	None	1.388844211	0.007332101	-0.387674449	0.557506388			1.054374476	0.6208322	-0.706948137	0.452730925		
AT2G28370	AT2G28370	1.387634361	9.98788E-07	0.678018271	0.000688984	0.64400308	0.000763318	0.126104263	0.930136802	0.133799197	0.600775782	-0.285719842	0.032282637
AT4G17940	AT4G17940	1.382989187	0.001852995	0.231654757	0.517906854	-0.033384459	0.940945165	-0.309488452	0.827628464	0.378104427	0.351440557	-0.692038806	0.358881286
AT3G26580	AT3G26580	1.375109412	0.00065058	0.797658957	4.80388E-06	0.861489885	3.6004E-07	1.219486221	0.527270121	0.164527033	0.605561645	-0.528409297	0.321619561
AT4G36990	HSF4	1.368585492	7.07927E-06	0.841614133	0.068662271			-0.517581028	0.726635812	0.06517592	0.9056262		
AT5G64880	AT5G64880	1.36606065	3.20432E-05	0.879767065	8.31394E-06	0.590362491	0.010014947	0.184465216	0.836588087	0.220891826	0.576874111	-0.450462932	0.017829301
AT3G07220	FHA2	1.365889804	1.83897E-05	0.584114033	0.017361908			0.658046194	0.579506696	0.302250242	0.20731129		
AT5G23680	AT5G23680	1.365166892	0.004453881	0.882709996	0.096476961	0.302529041	0.446971729	-0.76988959	0.389042646	-0.222547261	0.822587771	-0.595874219	0.068401684
AT5G61640	PMSR1	1.360950959	0.002854709	0.940237388	0.005890791	0.558313948	0.043346686	0.096995278	0.962819636	0.119464409	0.868646909	-0.348076348	0.106246557
AT4G10610	CID12	1.360411447	3.59351E-10	0.926479908	1.50305E-08	0.992631249	2.99221E-13	0.244875515	0.751883565	0.301849084	0.098683167	0.512614608	7.79965E-05
AT5G62900	AT5G62900	1.355606377	0.00728243	0.601711943	0.114348127	0.991009761	0.006248063	0.146223167	0.8904839	0.06040771	0.934226173	-0.496542427	0.01085748
AT5G49210	AT5G49210	1.350716279	8.2128E-08	0.684319708	0.026677383	0.910343652	1.52218E-07	0.389705121	0.672844426	0.401698055	0.135635347	0.077243048	0.777037681
AT3G62290	ARFA1E	1.34844719	1.22635E-10	0.729625734	6.48041E-06	0.694943868	1.32973E-09	0.064768759	0.953774208	0.128404815	0.512974126	-0.659959049	1.36239E-12
AT4G00590	AT4G00590	1.33809381	0.00062455	0.913804461	0.007946906	0.93344301	0.003864391	0.053422431	0.972495118	0.126930713	0.885236354	-0.212198877	0.399873974
AT4G32070	Phox4	1.336644667	3.93409E-05	0.900582681	0.003494011	0.591218733	0.064099441	-0.543135757	0.6107328	-0.211034426	0.575644885	-0.571626457	0.007053319
AT3G18320	AT3G18320	1.331131245	0.023909708	0.962705353	0.040001917	2.193818549	0.167109593	2.831838514	0.376362272	-0.642188332	0.500487453	-2.042572455	0.761927143
AT3G57910	AT3G57910	1.329590426	0.028031899	0.622263022	0.370732966	0.468765569	0.511854725	0.612776161	0.719188014	0.18584296	0.8641541	-0.431634872	0.249611497
AT3G49360	PGL2	1.328204205	3.2877E-05	0.631331169	0.013896569	0.520420556	0.078269229	-0.253699245	0.664635521	-0.149886954	0.58137711	-0.875300247	2.78762E-08
AT3G58840	PMD1	1.327970057	0.01030144	0.355220098	0.416373154	-0.471211902	0.366175561	-0.090275761	0.971297857	0.310716013	0.419417854	-0.853462146	0.010262061
AT5G40100	AT5G40100	1.321103557	0.023400398	-0.433299736	0.635770302	-0.116071178	0.853274885	0.106774939	0.957410897	-0.31801013	0.432579383	-0.26215363	0.294702404
AT5G45130	RHA1	1.317712757	3.49183E-07	0.883790303	2.75913E-06	0.649233041	0.000848612	0.276732374	0.741589735	0.057879324	0.870887081	-0.352951208	0.006792869
AT1G67860	AT1G67860	1.315658384	0.003607899	0.760275142	0.107406554	1.175216609	0.161878831	0.935319186	0.149173732	-0.141917792	0.888051403	0.717522305	0.06753878
AT4G14110	COP9	1.314156952	0.000538498	0.715136419	0.010945762	0.915065957	1.554E-07	0.332954233	0.751883565	0.520905855	0.007288944	0.134867642	0.513799224
AT1G64140	AT1G64140	1.313778823	0.000114374	0.977760037	1.71127E-11			0.294135024	0.841434341	-0.054797045	0.900659794		
AT5G05950	MEE60	1.312237332	1.0346E-05	0.632001202	0.007509022	0.717816916	0.000700121	0.308792533	0.706790319	0.446965227	0.050031519	-0.095146321	0.693821764
AT1G09320	AT1G09320	1.311990247	0.00052166	0.823531322	0.025487853	0.852440489	0.001324007	0.335507705	0.767355318	0.298354314	0.488358661	-0.561214566	0.031533111
AT4G03940	AT4G03940	1.302148072	0.035630552	0.19711157	0.859276802	1.233584555	0.164596254	0.444560169	0.784580159	-0.221409377	0.856243901	-0.32254412	0.565730134
AT5G50335	AT5G50335	1.29649323	0.035426816	1.038698553	0.120421997	0.64616901	0.1348711	-0.508494023	0.771422703	0.691705606	0.006948692	0.716934586	0.002885873
AT5G53650	AT5G53650	1.29633543	0.032504388	0.41453729	0.279826924	0.908034126	6.37316E-05	0.108891638	0.940849055	0.732443964	0.024430994	-0.24324064	0.469884365
AT5G37475	AT5G37475	1.293473573	9.20725E-05	0.910842189	0.000358301	0.490423868	0.009598864	-0.034812563	0.972270573	0.183484487	0.494394022	-0.011256522	0.963938492
AT1G60420	AT1G60420	1.291256593	0.000902913	0.976947509	9.70766E-06	0.916986859	7.46752E-05	0.150808179	0.899163675	0.129873855	0.715239621	-0.116622552	0.484056494
AT1G69850	NRT1:2	1.291113493	1.01017E-05	0.565382789	0.013852812	0.619418362	4.18375E-05	0.173209113	0.872669065	-0.061217801	0.910428886	-0.79918964	9.78045E-10
AT1G79190	AT1G79190	1.290264078	4.95474E-05	0.656601387	0.051094835	0.720512015	0.000179484	0.220513669	0.872697499	-0.289069153	0.384645119	-0.054288814	0.81611626
AT1G13690	ATE1	1.289235605	0.012804212	0.585114943	0.033195528	0.590048816	0.001894646	1.107375436	0.465456099	0.338928005	0.437384676	0.118734715	0.566851802
AT5G38895	AT5G38895	1.286422224	0.005812859	0.876829007	0.087184495	0.63863023	0.024319666	0.367008152	0.792048637	0.099435547	0.879321883	0.035096078	0.946507734
AT3G06610	AT3G06610	1.280036998	0.000330293	0.569249023	0.146255729	0.49913523	0.021646065	-0.041830844	0.971723999	0.225657898	0.488929906	-0.165252213	0.424094986
AT1G16810	AT1G16810	1.278602242	0.000270439	0.416334146	0.063627767	0.661569103	9.2684E-05	0.586461696	0.521675277	0.051910359	0.919937283	-0.296800666	0.311770311
AT4G15910	DI21	1.277624857	0.007308449	0.746170696	0.024079162	0.6408575	0.146926528	-0.230778849	0.903733738	0.571190897	0.000374129	-0.854822306	0.000444229
AT3G04350	AT3G04350	1.277497205	2.94734E-08	0.892076772	0.000147153	0.978758574	7.0149E-09	0.015980659	0.991559366	0.141154291	0.663912053	-0.943602702	1.36522E-16
AT1G43890	RAB18	1.269862452	1.08224E-05	0.924368416	4.28882E-06	0.720138115	0.001306133	0.213294928	0.835453631	0.288760737	0.176653084	-0.244105446	0.210413506
AT2G32240	AT2G32240	1.269084388	2.11108E-06	0.778699895	0.000318607	0.679938785	0.012448207	-0.470310546	0.669547085	-0.479902601	0.002250063	-0.918713097	2.92114E-10
AT5G65240	AT5G65240	1.267979935	0.000755996	0.438478785	0.429349938	0.317335986	0.482062524	-0.127963791	0.89537963	-0.287521021	0.529108457	-0.361974651	0.117246939
AT1G27670	AT1G27670	1.267385115	0.000223673	0.785688113	0.039319829	0.620763815	0.036373473	-0.052509916	0.980732812	-0.157115033	0.701818608	-0.118443509	0.668532147
AT1G64490	AT1G64490	1.266491829	0.01139105	0.485736512	0.329101819	0.715617763	0.056226835	-0.070611068	0.971297857	0.326533674	0.445236462	-0.166406167	0.577066851
AT5G56460	AT5G56460	1.258233642	0.024761732	0.443669932	0.467297714	0.271029739	0.551019821	-0.27798592	0.784493718	0.094963153	0.866014538	-0.502000626	0.001973962

AT1G56200	EMB1303	1.25622585	6.54205E-06	0.826200991	8.809E-10	0.509158046	4.8414E-06	0.439046445	0.656115309	0.209944927	0.535349266	0.029295688	0.923345813
AT5G27690	AT5G27690	1.252618817	0.038225185	0.939647095	0.018967121	0.725412482	0.072612922	1.191689988	0.292351702	0.467405986	0.523723041	0.183742426	0.717893195
AT1G26800	AT1G26800	1.250952601	0.000919481	0.931828779	0.009163267	0.719571656	0.020779198	0.243652139	0.810169925	0.344343892	0.417672111	-0.390865144	0.085559695
AT4G11300	AT4G11300	1.244601133	0.028899026	0.580717002	0.319967492	-0.144307542	0.838356904	-0.202013638	0.858420491	0.221019409	0.77082751	-0.531062673	0.188768288
AT4G03030	AT4G03030	1.244548581	7.46925E-07	0.806603526	0.000158716	0.789637788	4.21855E-06	0.224285677	0.896927975	-0.143907425	0.704291796	-0.956819251	4.69249E-05
AT2G29990	NDA2	1.241456986	0.017116451	0.03422807	0.956886122	-0.817342161	0.01569751	0.045905727	0.992231761	0.055844893	0.95452954	0.180596988	0.555242943
AT2G16930	AT2G16930	1.240196832	0.000404311	0.766706212	0.000745976	0.824775153	0.000254629	0.598637832	0.604422229	0.099578344	0.857006472	0.197813604	0.195671344
AT1G31812	ACBP6	1.238292386	1.10567E-05	0.601209868	0.000460231	0.654408799	6.78748E-07	0.140918704	0.914546909	0.221371089	0.254786774	-0.426957435	0.004976418
AT5G54470	BBX29	1.237934202	0.02685423	-0.092884676	0.908625565	0.201048418	0.720139876	0.066879752	0.981525175	-0.704208844	0.269079164	0.185422683	0.719130737
AT5G53160	RCAR3	1.235959572	2.17057E-05	0.771473807	6.19904E-11	0.764312599	2.43076E-05	0.593916837	0.152330984	0.509578923	0.000158501	0.110523509	0.458837921
AT2G19460	AT2G19460	1.23490471	0.00106039	0.358308572	0.590223232	0.045274522	0.934365292	0.23824214	0.813180163	0.128376469	0.856337781	-0.00254649	0.994616214
AT5G41020	AT5G41020	1.234410579	0.005282564	0.445872713	0.311194247	0.607424338	0.002242986	0.147317016	0.881491928	-0.333400279	0.485644927	-0.806976368	0.00069978
AT1G33520	MOS2	1.233184651	1.44189E-06	0.338341398	0.414650411	0.164712944	0.61916997	0.113343717	0.953923605	0.023548738	0.970305421	-0.815490838	0.161122891
AT5G26110	AT5G26110	1.232125722	0.000226387	0.539232556	0.122518142	0.790642689	0.001025397	-0.001933872	0.998795143	0.187255686	0.641719502	-0.174697921	0.348667155
AT1G16916	AT1G16916	1.229028776	0.016705626	-0.509170593	0.3889554	-0.350229455	0.3814539	0.230611892	0.855210277	0.964343515	0.024919065	0.235806389	0.548166507
AT3G48330	PIMT1	1.228887195	0.000492443	0.628235172	0.072979755	0.609607475	0.016801373	0.380003193	0.691824202	0.506833095	0.152286365	0.20790199	0.363837231
AT5G24870	AT5G24870	1.227373291	0.003882946	0.544939261	0.17174157	0.394117351	0.254338268	-0.088743685	0.944029748	-0.146693703	0.824058587	-0.369269245	0.209436419
AT1G54320	AT1G54320	1.226140497	1.7152E-06	0.988558592	7.74148E-08	0.586091068	0.00260868	0.016422336	0.991438225	-0.153094373	0.55787595	-0.408136886	0.000501582
AT1G49850	AT1G49850	1.225974212	1.06458E-05	0.501502553	0.036943062	0.432358989	0.119071764	0.150106413	0.933020011	0.000610836	0.999659387	-0.207818367	0.335319317
AT3G16640	TCTP	1.224002949	6.49846E-07	0.535648109	0.000124358	0.253880833	0.03180367	0.251772889	0.677000788	0.404336677	0.00638449	-0.378946265	0.016142654
AT1G26450	AT1G26450	1.222242191	0.027275117	0.188703094	0.839429343	0.952314447	0.058343598	-0.108713491	0.937178328	-0.189051318	0.751599754	0.206994446	0.386994353
AT3G59640	AT3G59640	1.221562905	0.045607953	0.108189978	0.881907063	0.067723238	0.899005135	-0.024177565	0.991271129	-0.120225112	0.896222696	-0.021972799	0.963430966
AT3G05380	ALY2	1.214991918	0.000373216	0.789833824	0.009322234	0.874713033	4.18254E-07	-0.219516455	0.731445776	0.017308085	0.976570624	-0.276458094	0.119775757
AT4G40060	HB16	1.211629667	5.52809E-08	0.987338275	1.74082E-09	0.417476643	0.046363877	0.01082142	0.992593412	0.049997248	0.89611977	-0.718983762	1.05117E-13
AT1G28050	BBX13	1.207690442	0.028050323	0.092857822	0.889830491	-0.154476473	0.71048857	-0.230228835	0.837184533	0.252513393	0.491449838	-0.205355774	0.365559863
AT2G16600	ROC3	1.207252401	0.00011024	0.914801221	7.7265E-07	0.308207488	0.084612797	0.194730217	0.700347186	0.434634351	0.005888777	0.066648537	0.719503216
AT4G18905	AT4G18905	1.207031639	0.00031764	0.769157195	0.000807249	0.460517864	0.009032731	-0.071252703	0.93058272	0.076456002	0.850188481	-0.161057164	0.234096555
AT2G17990	AT2G17990	1.206675522	0.007494305	0.396285768	0.215035547	0.227181938	0.52256039	-0.202898653	0.842959841	0.20100532	0.52853693	-0.68875783	0.002111509
AT5G60340	AAK6	1.206215386	8.50717E-07	0.980884971	1.69766E-12	0.907516834	1.14646E-09	0.613542991	0.343493265	0.465974101	0.001134929	0.276245682	0.087809541
AT2G44310	AT2G44310	1.205345717	1.56992E-05	0.913148971	0.002252201	-0.336050084	0.216232892	-0.16455452	0.870080205	0.395527604	0.13025603	-0.449615692	0.039145799
AT4G20880	AT4G20880	1.204419443	0.001223838	0.807257591	0.053796983	0.667180523	0.02548783	-0.223191839	0.771422703	-0.259731166	0.356096017	-0.785128924	0.001037779
AT1G35516	AT1G35516	1.202584266	2.43507E-06	0.362036691	0.164506976	0.735536732	2.96526E-07	0.220959374	0.824310279	0.282801208	0.224978038	0.182391237	0.450529821
AT1G76860	LSM3B	1.202211196	5.00231E-05	0.626620785	0.001485407	0.413716312	0.038696507	0.088539261	0.953801756	0.071108825	0.887567656	-0.12131087	0.680305572
AT5G53400	BOB1	1.199648976	0.001835006	0.714422295	0.009503197	0.630903439	0.005866537	-0.236623943	0.80141891	0.361724911	0.191308765	-0.181206573	0.247655837
AT5G24800	BZIP9	1.193661001	4.71739E-05	0.824035514	0.000724093	0.668228028	8.86476E-07	0.75286663	0.640704059	0.369789172	0.069374895	-0.224190636	0.183000207
AT5G62290	AT5G62290	1.193439487	5.64502E-05	0.658860904	0.060517312	0.620943724	0.003277495	0.386507402	0.712887303	0.34713587	0.292089408	0.059303592	0.811308424
AT1G11360	AT1G11360	1.191846363	0.000179363	0.561597364	0.061376191	0.33403551	0.101579412	0.098874994	0.951228965	0.325524552	0.180665309	-0.346712949	0.107666449
AT5G12410	AT5G12410	1.186567269	4.56575E-05	0.660101171	0.002277885	0.790938107	3.94166E-06	0.412456152	0.48633216	0.301684657	0.232320876	-1.3928E-05	0.999938661
AT4G27780	ACBP2	1.185852424	0.002863172	0.706384116	0.043668984	0.788365193	0.030854504	-0.412997867	0.652140399	-0.213144833	0.619982969	-0.618809039	0.004700323
AT1G68300	AT1G68300	1.18427635	1.70511E-05	0.925614696	5.75505E-05	0.919617276	1.86605E-05	0.063665318	0.963305035	-0.152896935	0.640668232	-0.576861881	0.001633807
AT3G53410	LUL2	1.183955353	0.010706425	0.49718192	0.199002591	0.522668038	0.109176357	0.417815535	0.66808962	0.018566796	0.986818647	0.008321772	0.980218804
AT5G12010	AT5G12010	1.183376357	0.00034786	0.871914826	0.046222337	-0.178415074	0.685633688	-0.52215419	0.619616142	-0.307169888	0.376826371	-0.561505402	0.000731715
AT1G08110	AT1G08110	1.18287509	0.000148099	0.920619754	3.90708E-13	0.679069419	8.52499E-08	0.632225571	0.590788206	-0.123370569	0.615893075	-0.226696317	0.063293998
AT1G29785	AT1G29785	1.181935954	0.014460764	-0.057123035	0.930686245	-0.173971324	0.74762439	-0.556328531	0.601921549	0.844076534	0.00248889	-0.363418892	0.286025974
AT1G76700	AT1G76700	1.180686594	0.000585944	0.87452187	0.000407738	0.833897386	7.19856E-06	0.552106666	0.706790319	0.07869153	0.885377618	-0.400470736	0.052972753
AT5G24570	AT5G24570	1.180611364	0.004704106	0.610208907	0.027284126	0.381679797	0.532384249	-0.275965919	0.809502311	-0.563512042	0.052855195	-0.790858122	0.008146495
AT1G36980	AT1G36980	1.176392166	0.000374673	0.99460256	7.30346E-08	0.831733803	2.46695E-09	0.219880425	0.867387495	0.160699987	0.556330533	-0.499200153	4.85799E-05
AT1G29030	AT1G29030	1.171999097	0.021461605	0.289031751	0.635876923	0.365538412	0.357125301	-0.193770484	0.851805999	-0.322460007	0.408833515	-0.020957898	0.938557714
AT1G76690	OPR2	1.169990471	0.017355316	0.283929682	0.762697723	0.487430009	0.344331606	-0.554132882	0.66808962	-0.300529407	0.651307456	-0.364497743	0.2545807
AT5G51980	AT5G51980	1.165188505	0.040656286	0.576182476	0.118072346	0.065606438	0.882676503	0.150504221	0.827716472	0.287487648	0.407469184	0.425959697	0.019003036
AT2G39940	COI1	1.164791519	0.000148211	0.819216064	0.001322765	0.751616456	0.000284187	0.140245427	0.906561784	-0.115473685	0.729493271	-0.215810148	0.188842043
AT1G64750	DSS1(I)	1.163069924	0.001759542	0.497011815	0.041669875	0.232484231	0.199702434	0.100028767	0.902565251	0.513148648	0.008751696	-0.16629023	0.444220408
AT4G12990	AT4G12990	1.162993061	0.024418743	0.34947132	0.672212611			0.29022436	0.833817066	-0.1895703	0.810684437		
AT1G09960	SUT4	1.162577582	0.015556028	0.989213092	0.03750405	0.471473888	0.411726273	-0.506821548	0.643223507	-0.036776031	0.967793437	-0.474781931	0.03522325
AT2G22080	AT2G22080	1.159436065	0.00136626	0.484266687	0.104097999	0.132001671	0.628185833	0.500211871	0.637303685	0.221889089	0.499031618	-0.469942244	0.100009986
AT2G24240	AT2G24240	1.156515782	0.01279873	0.809945232	0.102334695	0.801006945	0.000572349	0.599682698	0.66524984	0.051371809	0.957156296	-0.848046852	0.000313778
AT5G43822	AT5G43822	1.155894015	0.024526599	0.930730363	0.123095279	0.489123249	0.31457981	0.55944938	0.73461774	0.494959782	0.409394157	0.126307109	0.698705066
AT4G39550	AT4G39550	1.152491947	0.017864701	0.065321385	0.93580134	0.037002975	0.952606907	0.452164017	0.653363031	0.196845754	0.779557887	-0.1398456	0.620437128
AT4G14410	bHLH104	1.152292729	2.38347E-07	0.932369425	6.53135E-06	0.833052998	1.45246E-06	0.177698557	0.748481821	0.198130296	0.405816794	-0.557728156	0.003314201
AT1G51200	AT1G51200	1.150577368	9.50149E-07	0.653454209	3.09705E-05	0.512738964	4.75259E-05	0.112050104	0.903468584	0.14987824	0.533170432	-0.76017769	3.84879E-10
AT4G29420	AT4G29420	1.148711363	0.016177558	0.569708879	0.048906211	0.567231883	0.070976698	1.255027762	0.459522829	0.463358067	0.363073224	-0.222576827	0.372823058
AT4G37870	PCK1	1.148453134	1.79995E-09	0.621116463	0.000117807	0.845131778	0.000457598	0.199885738	0.741086025	0.024451687	0.943490436	-0.309705438	0.006864777
AT5G58210	AT5G58210	1.146069429	0.039653574	0.207727954	0.576949754	0.918101598	0.000102239	0.206573057	0.885120518	0.012766784	0.989147453	-0.229795575	0.338340797
AT1G34630	AT1G34630	1.144918077	0.00423866	0.978429983	0.003328648	0.711533159	0.000818647	-0.361035308	0.669910795	0.252023597	0.510442568	-0.27741542	0.121165626
AT3G03160	AT3G03160	1.142017354	1.55869E-05	0.540331316	0.023095991	0.13928351	0.657411784	-0.266819592	0.706790319	0.133234378	0.658556264	-0.349118644	0.020083866
AT1G54380	AT1G54380	1.140546433	0.005955825	0.345675552	0.354177666	0.812261559	0.000932038	0.404243088	0.585601259	0.379909158	0.142654235	0.022743735	0.939354546
AT3G07870	AT3G07870	1.139073297	0.001963793	0.849826683	0.010149836	0.883913443	2.01847E-05	0.628851128	0.637660913	0.02563683	0.970929514	-0.652510358	3.09294E-05
AT1G27650	ATU2AF35A	1.138400236	4.3657E-06	0.68679967	4.96346E-06	0.795463468	5.28678E-07	0.02985877	0.979193949	0.373169983	0.035243174	-0.398705451	0.030364053
AT5G35200	AT5G35200	1.136706858	7.36778E-06	0.854288833	2.28643E-05	0.73599535	7.19138E-06	0.076396126	0.947241707	-0.154674145	0.554072186	-0.39919877	0.000584168
AT5G58950	AT5G58950	1.136203838	2.02219E-05	0.777938976	1.53568E-06	0.982635082	2.8335E-13	0.196256359	0.891620687	-0.118228605	0.568299134	-0.808234258	2.47187E-09
AT5G64250	AT5G64250	1.135015271	0.000209299	0.931990829	1.92255E-06	0.346181586	0.106773602	-0.320763232	0.735907718	-0.008197969	0.992281921	-0.562442649	0.001693982
AT5G21482	CKX7	1.133853119	0.010484694	0.827759862	0.093533273	0.897792522	0.00214167	-0.444609079	0.710654662	-0.076147497	0.955760402	0.161128408	0.623835762
AT2G33310	IAA13	1.131177602	2.88166E-05	0.348872559	0.216164596	0.160900921	0.49647718	-0.184605138	0.866477795	0.450795294	0.162392805	-0.804441615	0.00108384
AT4G02370	AT4G02370	1.130458237	0.00066226	0.75731917	0.005502785	0.894254465	3.70725E-05	0.064191911	0.961157568	-0.16636784	0.529391821	-0.875347178	4.37403E-06
AT5G60540	PDX2	1.12990482	0.00043939	0.520650355	0.016402052	0.77220667	2.06493E-05	0.162207389	0.830606688	0.298364052	0.179582117	-0.03219348	0.87978831
AT5G20520	WAV2	1.129029428	8.23991E-08	0.700652825	9.74654E-06	0.905471928	4.89014E-09	0.362756845	0.652473285	0.415907174	0.001003755	-0.088329731	0.66365854
AT3G57390	AGL18	1.128389447	0.002545929	0.94639501	7.70924E-05	0.900280087	3.66381E-05	0.609538266	0.619462504	0.325931182	0.397816908	-0.042642784	0.867370638
AT5G58470	TAF15b	1.12665421	0.008971785	0.460070696	0.084049834	0.565299747	0.007662514	-0.067262872	0.966082349	0.194624981	0.544538932	-0.503353694	0.392204912
AT4G24990	ATGP4	1.125241108	0.000113776	0.763803122	0.000166383	0.804049248	0.000335503	0.474598491	0.653363031	0.354345979	0.141230165	-0.628733415	0.005752484
AT1G02340	HFR1	1.124930631	0.013461955	0.810355166	0.000822182	0.544183595	0.032009517	-0.335225452	0.821797134	0.013266547	0.971701608	-0.343491698	0.017356195
AT2G21060	GRP2B	1.12485973	0.018420777	-0.319736231	0.52200125	-0.246295265	0.518172305	-0.574101162	0.647947756	0.475203779	0.157407643	-0.878125927	0.339758914
AT1G20920	AT1G20920	1.124848374	0.002844172	0.474364135	0.059915235	0.854711676	1.19567E-10	0.544480238	0.667027577	0.353852647	0.169823672	-0.324674402	0.091078224
AT2G32260	CCT1	1.124234644	3.67823E-05	0.737105886	0.000118633	0.760144373	5.89596E-05	0.16188492	0.895706838	-0.034438832	0.951805765	-0.404011769	0.032395248
AT5G47120	BI1	1.123411467	0.000745552	0.375366196	0.172737369	0.08449405	0.775308313	-0.439329813	0.474958589	-0.307398931	0.127102132	-0.369340716	0.01903686
AT2G32600	AT2G32600	1.12307836	0.002670107	0.874331023	5.70671E-07	0.902971368	7.23094E-08	0.621615185	0.616300683	0.060975869	0.895021414	0.055223227	0.823421499
AT4G33420	AT4G33420	1.119011075	0.001981428	0.182765421	0.682275313	-0.582510755	0.086371304	-0.657532575	0.419631806	-0.341340666	0.072740261	-0.469963866	0.002208453
AT5G02600	NAKR1	1.117992094	0.009643383	0.795582594	0.012541951	0.749368349	0.021671589	1.037783274	0.474740724	0.05281994	0.94946668	0.771805741	0.002349936
AT1G59900	E1 ALPHA	1.116478895	3.54072E-06	0.967963052	5.18999E-19	0.940305147	1.38808E-13	0.63825575	0.62738272	0.103201738	0.639388142	-0.252759175	0.022980912
AT1G56700	AT1G56700	1.116225825	1.01461E-06	0.800901476	0.000372486	0.642387863	1.79173E-05	0.025749112	0.982664567	0.14494002	0.599487284	-0.213521546	0.140552899
AT2G04410	AT2G04410	1.115581727	0.000118911	0.648781367	0.001107235	0.163661905	0.439177283	-0.293123146	0.647947756	-0.004902302	0.992618878	-0.666314379	0.004662512
AT2G17130	IDH2	1.113704422	0.04297017	0.104941567	0.828946186	0.183622986	0.620313382	-0.315433725	0.657369817	-0.092333256	0.871060407	-0.215199182	0.453488959
AT2G20490	NOP10	1.110923256	1.65526E-05	0.601464218	0.005065829	0.722231999	3.54747E-06	0.111620172	0.899858481	0.074087399	0.856695283	0.222124313	0.288239911
AT5G15750	AT5G15750	1.110709142	0.001306957	0.766514067	0.008748125	0.445459105	0.236056507	0.462865075	0.580334332	0.049586334	0.934596763	0.046452796	0.864733016
AT5G09225	AT5G09225	1.108541603	0.003441401	0.51450015	0.048910711	0.717326255	0.000266035	0.315389651	0.720336606	0.333286319	0.330168475	0.115653553	0.732831304

AT1G49170	AT1G49170	1.105408804	0.02304182	0.506407718	0.288321173	0.587314093	0.031840175	0.338814071	0.702246294	0.186107027	0.802919605	-0.134241558	0.65693965
AT5G06850	FTIP1	1.101862786	0.017996693	0.556115329	0.226460119	0.657510476	0.099905452	-0.075537509	0.973855042	-0.306754946	0.484700858	-0.682231823	0.001517571
AT4G32240	AT4G32240	1.100913059	0.000112089	0.826566989	0.020936622	0.542897337	0.048499943	0.33936229	0.691824202	0.401026518	0.113310986	-0.703614564	0.001526966
AT2G25690	AT2G25690	1.100714434	0.028455415	0.301180335	0.550812574	0.074684478	0.84766831	0.061306632	0.970881852	-0.485916682	0.303995259	0.046017967	0.87134476
AT1G12080	AT1G12080	1.100470431	8.04895E-05	-0.651630393	0.040633618	0.261400376	0.407406992	-0.443921535	0.644839236	-0.322568087	0.239373546	0.218913695	0.402980058
AT5G20610	AT5G20610	1.09999741	0.000122366	0.9707564	3.42286E-06	0.819725774	2.56404E-07	0.090991456	0.954137138	-0.277438319	0.362760375	-0.328651677	0.030508898
AT1G44760	AT1G44760	1.099449771	0.006885573	0.411387632	0.157118804	0.888719029	0.001720516	0.073902176	0.92946974	0.722347653	5.48866E-06	0.731032811	7.24871E-07
AT5G65430	GRF8	1.099273487	0.000186051	0.403095386	0.014781901	0.921732119	1.46341E-12	0.621615909	0.601921549	0.147608721	0.52648618	0.067252895	0.763113464
AT3G28950	AT3G28950	1.096834414	0.01971717	0.007639098	0.991616417	-0.023291662	0.968755108	-0.058310392	0.982623916	0.069848189	0.927080141	-0.888624135	0.000534385
AT2G03890	PI4K GAMMA 7	1.096257115	1.01062E-05	0.432938391	0.010100561	0.769885033	2.95906E-07	0.236238088	0.783641982	0.305407447	0.108180357	-0.587497283	1.76687E-05
AT2G01650	PUX2	1.094438345	0.000533917	0.480178695	0.167239801	0.168073373	0.624601236	-0.017713971	0.990915497	-0.351245028	0.331350639	-0.451899271	0.016444561
AT2G39100	AT2G39100	1.092522651	0.008615364	0.837976413	0.080390255	0.622737059	0.078790198	0.281540278	0.769502096	0.143885246	0.839885043	0.068439757	0.823490342
AT1G68070	AT1G68070	1.090528507	5.50509E-05	0.995501555	0.011043428	0.336224733	0.228359841	-0.130113792	0.910340522	0.141645416	0.731005856	-0.0771547	0.778538173
AT1G61870	PPR336	1.089234436	0.005878549	0.387626927	0.186663498	0.595251269	0.074933709	0.269085358	0.680482607	0.245673775	0.484700858	-0.080684973	0.715673175
AT1G61250	SC3	1.088811996	0.000139727	0.855896588	9.59861E-07	0.466128798	0.0015344	-0.177129906	0.904735573	-0.169327707	0.44501051	-0.247323124	0.063567741
AT4G37790	HAT22	1.08843803	0.000678298	0.994495521	2.10413E-05	0.842498251	5.98573E-10	-0.230574906	0.798570031	-0.031221381	0.957130081	-0.885834772	5.85055E-06
AT4G29820	CFIM-25	1.087935875	0.003873669	0.614292446	0.163676389	0.767964227	0.019911555	-0.40270091	0.676109289	-0.029688913	0.966085856	-0.684160757	6.36454E-05
AT5G08139	AT5G08139	1.087301463	0.003405734	0.664525496	0.25703168	0.601497083	0.186535669	-0.412624051	0.751495303	0.445444952	0.411113318	-0.530689758	0.051842312
AT5G27700	AT5G27700	1.085147118	1.64491E-07	0.558034377	0.000243935	0.769784243	1.27068E-09	0.145377002	0.820789421	0.477201461	0.00030078	0.294402545	0.08096539
AT1G21760	FBP7	1.084667013	0.000644535	0.652934645	0.00582103	0.766861838	9.06401E-06	0.932204128	0.49268988	-0.022985505	0.972421502	-0.043766409	0.867604777
AT5G52550	AT5G52550	1.08459699	0.002762556	0.990185238	0.000604962			0.709534216	0.611321113	0.239308749	0.498449509		
AT4G24590	AT4G24590	1.083634596	0.000639835	0.615462377	0.099465148	0.601676564	0.010760023	0.01007763	0.995194798	0.47800054	0.072740261	-0.286928204	0.379257434
AT5G53540	AT5G53540	1.083195442	0.000609268	0.895115519	4.63259E-06	0.38620582	0.057089716	0.598289635	0.604422229	0.186224502	0.559593178	0.227606572	0.224087845
AT3G21865	PEX22	1.081787223	2.88166E-05	0.795568904	0.000344652	0.608599366	9.3193E-06	0.503322217	0.636577463	0.321392026	0.05776587	-0.092514441	0.680912677
AT2G25910	AT2G25910	1.079709495	3.91787E-05	0.650238032	0.001492989	0.959736087	3.33565E-07	0.064335865	0.960890507	-0.004206345	0.994163341	0.161034357	0.279228599
AT5G46760	MYC3	1.079485785	3.8076E-07	0.480704785	0.015152614	0.101720345	0.680112013	-0.125498067	0.932418897	0.268886485	0.277703192	-0.597569532	0.002342357
AT5G39570	AT5G39570	1.078922256	0.000242013	0.950033613	8.81412E-09	0.264840052	0.203295175	0.169429262	0.904735573	-0.068682795	0.845640294	-0.599969945	0.009303893
AT3G42790	AL3	1.076776505	3.40264E-05	0.665729674	0.000153095	0.888356188	1.65992E-12	0.271697023	0.704522562	0.213188185	0.373412714	-0.097813867	0.544355521
AT2G46310	CRF5	1.07608083	0.002427165	0.559885963	0.268334812	0.620390048	0.104020235	-0.422059991	0.601921549	-0.11527785	0.831572245	-0.603360949	5.26857E-05
AT1G78895	AT1G78895	1.073475862	0.008596334	-0.221528633	0.639013956	0.109426268	0.761302817	-0.066103406	0.972495118	0.385619102	0.481870906	-0.239105956	0.36439213
AT3G14180	ASIL2	1.072384495	0.00166923	0.564734187	0.057955566	0.718165699	0.042055305	-0.289167013	0.751883669	0.400621097	0.058730626	-0.589164498	0.00575083
AT5G58590	RANBP1	1.071227194	0.000270197	0.17345464	0.6568004	0.552394108	0.002411698	0.047759096	0.972495118	0.042895059	0.934609223	-0.090178579	0.750030356
AT2G15000	AT2G15000	1.07021576	0.04223978	0.077370776	0.879731332	-0.755411942	0.030680941	-0.503571683	0.726900342	0.240501604	0.667064993	0.417276959	0.048213653
AT3G11900	ANT1	1.069529951	0.030905297	0.559166679	0.037512263	0.614321128	0.025151195	0.670540555	0.675615589	0.250882848	0.591890271	-0.695755162	7.67603E-05
AT4G35230	BSK1	1.066725977	0.00011855	0.558820426	0.002576182	0.964721134	8.76887E-14	0.477003621	0.647947756	0.05578108	0.855204799	-0.346197324	0.023348863
AT5G01240	LAX1	1.065669351	0.005308097	0.958050034	2.83984E-09	0.588543192	0.002083062	1.220867386	0.510634068	-0.115101614	0.723913117	-0.5154656	9.46378E-05
AT5G63760	ARI15	1.065372158	0.00195741	0.447869622	0.253411802	0.94090469	0.001073598	0.426836718	0.726934566	-0.027296384	0.964180223	-0.65764861	0.00420161
AT3G07030	AT3G07030	1.065037955	0.006911886	-0.356208944	0.449905287	0.256287068	0.522796149	-0.250149141	0.877665269	-0.145039963	0.85220051	-0.709932976	0.322121927
AT5G54430	PHOS32	1.063997462	5.28796E-05	0.79111218	3.05312E-06	0.791530189	1.15025E-07	0.154267832	0.861673753	-0.019499729	0.959952977	-0.141452878	0.411321258
AT3G63390	AT3G63390	1.062563985	0.005401646	0.496736345	0.057800427	0.43986899	0.053368337	-0.339642079	0.769523039	0.667801783	0.003726829	-0.774614812	0.000115847
AT3G63220	AT3G63220	1.062463195	0.012403777	0.924842209	0.025454242	0.881238207	0.005734669	0.439846865	0.764557332	0.196407829	0.717765433	0.011886141	0.96926048
AT5G02100	UNE18	1.061826402	0.002240757	-0.042913557	0.936296257	0.660598293	0.268575682	0.178918302	0.850231847	-0.402132023	0.126532237	-0.758500873	0.000105197
AT4G11240	TOPP7	1.061717038	4.52198E-07	0.563813247	0.000164995	0.455071185	0.005010719	0.073498456	0.941411184	0.209738534	0.330077786	-0.160391058	0.269644502
AT5G05550	AT5G05550	1.060943074	0.003607899	0.963895094	0.013244775	0.784670272	1.77586E-05	0.534914432	0.712887303	0.843345656	0.042712549	0.437229441	0.024312279
AT5G61510	AT5G61510	1.059277319	0.00643843	0.62436104	0.019724051	0.556948705	0.011992492	0.607513707	0.548879103	0.204569547	0.575399675	0.216078781	0.323671836
AT1G60650	AtRZ-1b	1.055959482	0.020348516	0.240255773	0.595587503	0.914095821	0.005190288	0.159678284	0.932578056	0.109995979	0.896222696	-0.251530037	0.408056794
AT1G72175	AT1G72175	1.055011008	0.00989443	0.666147968	0.03097999	0.504804892	0.236056507	0.232286045	0.906874792	0.278888394	0.524973096	-0.364778422	0.150013518
AT1G22790	AT1G22790	1.054734858	0.000580859	0.436091684	0.146416951	0.347854811	0.161620877	0.194724331	0.894633383	0.313248391	0.331126199	0.117099728	0.723146422
AT1G15220	CCMH	1.052809478	0.0234583	0.259758259	0.527104433	0.60742178	0.074800503	-0.034463247	0.988471189	0.387357063	0.473541564	-0.226571416	0.285350046
AT1G49650	AT1G49650	1.052217312	0.001812565	0.80793553	0.002877789	0.264051082	0.687283493	0.475142396	0.536751295	-0.12479894	0.779421808	0.004335571	0.991194733
AT2G29020	AT2G29020	1.051767469	0.003353802	0.597089192	0.020025448	0.479445511	0.036500824	-0.075510176	0.936758504	0.313944654	0.167946677	0.042653263	0.844917584
AT3G24100	AT3G24100	1.051280174	0.015286478	0.675288808	0.010810271	0.143354856	0.444084641	0.173925453	0.848507895	0.017205806	0.980354555	-0.714707692	0.000593195
AT4G21790	TOM1	1.050947714	0.000124777	0.93716503	7.94007E-08	0.967611947	1.23801E-11	0.526799569	0.647883443	0.084177958	0.798599938	-0.416547275	0.000525158
AT5G39850	AT5G39850	1.050578442	0.007748175	0.699275216	0.026536033	-0.010513022	0.97926065	-0.297219384	0.680482607	-0.098819711	0.802042097	-0.948710324	8.67925E-10
AT3G49870	ARLA1C	1.050444095	0.000339233	0.908464626	4.1845E-05	0.69716331	3.00187E-05	0.210837439	0.774132304	0.095237796	0.752969619	-0.281963487	0.061069628
AT5G53800	AT5G53800	1.048905018	0.015139411	0.821592776	0.006650507	0.486358813	0.066806827	0.026974113	0.982734229	0.260933902	0.459901739	-0.512552773	0.099266962
AT3G14570	GSL04	1.047654257	0.043604045	0.918346816	0.009667141	0.706947996	0.05977022	-0.517416	0.657854795	-0.507659876	0.104373576	-0.512146488	0.109522448
AT4G21192	AT4G21192	1.045365785	0.008351991	0.281247761	0.497141357	0.330368247	0.313759154	0.512745139	0.604422229	0.391283533	0.456448413	0.422070619	0.115261467
AT4G40040	AT4G40040	1.044847545	2.62288E-05	0.893890797	1.13982E-09	0.585956856	4.33508E-06	0.366423621	0.73461774	0.115179457	0.573481432	-0.198401901	0.214012122
AT1G78150	AT1G78150	1.044698168	1.26074E-06	0.614683737	5.45731E-06	0.646135251	6.49643E-07	0.2424033	0.627500883	0.225345396	0.180670778	-0.153309385	0.243606578
AT2G02160	AT2G02160	1.043529325	0.000331705	0.548531714	0.000209892	0.641673421	9.15884E-07	0.388123396	0.676109289	0.110281811	0.737409054	-0.534765027	0.007530851
AT5G58290	RPT3	1.041080648	0.000130132	0.926920512	3.36777E-14	0.988665135	9.4394E-15	0.331052911	0.680595832	-0.08691616	0.748569551	0.087124514	0.657145208
AT4G34870	ROC5	1.04097392	0.00485019	0.763189673	3.02398E-06	0.042416658	0.841492602	-0.253325303	0.815973749	0.249899967	0.160507774	0.156634079	0.490132648
AT3G51980	AT3G51980	1.04084369	0.018849723	0.677207094	0.14014599	0.147842424	0.77329369	-0.318688245	0.647947756	-0.241538642	0.517194506	-0.259728938	0.193672247
AT1G61620	AT1G61620	1.040829713	0.00053015	0.987313869	2.12685E-09	0.960339519	4.17249E-08	0.529704431	0.619116349	0.140363249	0.589526013	-0.225647815	0.086306779
AT5G55160	SUMO2	1.04066457	0.000465931	0.335618489	0.217428858	0.17093563	0.344505272	0.251355425	0.691895802	0.277280962	0.185915504	-0.016792308	0.938277099
AT1G31120	KUP10	1.03932636	0.019449716	-0.082198401	0.90439585	-0.442542443	0.117108639	-0.940098376	0.613859108	-0.45530068	0.109186116	-0.703819076	2.6594E-05
AT3G47160	AT3G47160	1.039187657	0.006523846	0.367673462	0.218301374	0.069072029	0.827569325	0.43935574	0.657637951	0.185698447	0.515326675	-0.474183951	0.066213476
AT1G12650	AT1G12650	1.03705191	0.003073638	0.334187929	0.341385493	0.623858436	0.018415956	0.294216764	0.66524984	0.369506452	0.230355117	0.208049874	0.223594321
AT5G23340	AT5G23340	1.036804478	0.001656264	0.513592953	0.025341717	0.815783663	0.000177144	0.382158218	0.753936613	0.447054274	0.143920603	-0.200453459	0.248701321
AT3G45240	GRIK1	1.036517746	0.002074606	0.979838115	3.68533E-05			0.465948873	0.613859108	0.254130591	0.398922866	-2.974466693	0.645082103
AT5G10710	AT5G10710	1.035953175	0.017809345	0.937007928	0.001840148	0.972760718	2.2697E-06	0.555757266	0.647947756	0.095101307	0.882211291	0.186823762	0.3498426
AT2G38830	AT2G38830	1.035315485	0.035023791	0.591606066	0.28183966	0.364501719	0.392192513	0.695828019	0.633628949	0.656564552	0.273439868	0.298011677	0.286754713
AT1G68550	CRF10	1.031051671	0.007652597	0.526116777	0.312408264	-4.916336039	0.359268731	0.160744689	0.906972928	0.277880039	0.334374346	-0.030232491	0.993906767
AT2G42310	AT2G42310	1.030183737	0.000104721	0.36764741	0.024706823	0.406148969	0.006354667	0.044249056	0.964175862	0.4197227	0.046804287	0.278197894	0.113293187
AT5G20920	EIF2 BETA	1.029273654	1.77021E-05	0.832143401	7.33513E-09	0.897784247	6.88068E-08	0.294312626	0.709586436	0.186807403	0.526650507	0.004974102	0.988801827
AT4G00355	ATI2	1.027593252	3.30985E-05	0.801236469	1.42207E-05	0.696561459	0.000338051	0.435623305	0.691824202	0.217748591	0.333574619	-0.031359959	0.868390224
AT3G23710	Tic22-III	1.023077414	0.02365491	0.928926212	0.001985981	0.759931199	0.001274539	0.735549473	0.55451616	0.249457324	0.478415556	0.209937863	0.234096555
AT2G47790	AT2G47790	1.022911677	0.000141573	0.722965314	0.000392113	0.960799534	1.09852E-08	0.548074426	0.433558429	0.289535176	0.265754101	0.258398305	0.112543797
AT3G63210	AT3G63210	1.022868638	0.014970229	0.916011981	0.001628616	0.305194492	0.174557796	-0.081047135	0.957474117	-0.358835153	0.305729659	-0.768927062	2.69315E-08
AT1G15340	MBD10	1.022863232	0.003125105	0.765675753	5.43868E-06	0.553847008	0.000157801	0.486332843	0.698438613	0.05038162	0.914111276	-0.386041301	0.491926726
AT4G30935	WRKY32	1.021478045	0.007543665	0.153790235	0.709660357	0.594082721	0.050016253	0.159471968	0.824310279	-0.070647881	0.928898731	-0.162584864	0.359440074
AT5G45500	AT5G45500	1.021445576	2.90053E-05	0.677543777	0.000127594	0.792775564	1.78367E-07	-0.017373374	0.990915497	0.06094641	0.883566526	-0.440009707	0.002135717
AT3G52560	UEV1D-4	1.021305565	3.14815E-05	0.716914632	3.4218E-06	0.716624115	1.48834E-06	0.033637196	0.972495118	0.160044645	0.516852689	-0.207759366	0.212338855
AT1G18690	XXT4	1.021262577	0.000674549	0.220250652	0.513698328	0.798673604	0.027524386	0.84884743	0.616300683	-0.224343055	0.604209898	-0.239957017	0.287397439
AT2G32415	AT2G32415	1.020168391	0.004245478	0.813155093	0.001106498	0.752882076	0.014666047	0.248206292	0.731041294	0.287612863	0.51736341	0.298053297	0.126573638
AT3G02770	AT3G02770	1.018616385	0.001964602	0.828662482	0.009062033	-0.217570413	0.4695086	-0.165268405	0.829142257	0.225932258	0.579418774	0.163271407	0.51141875
AT1G32810	AT1G32810	1.017228154	0.007942176	0.595449927	0.010092505	0.769012714	2.52408E-05	0.408034867	0.735038565	0.07069512	0.896222696	-0.461724266	0.003826579
AT2G19480	NAP1;2	1.01683888	3.15178E-07	0.427914246	0.002941376	0.75093211	1.33019E-12	0.022268964	0.982356953	0.059291147	0.857349753	-0.430903872	0.001487546
AT1G68140	AT1G68140	1.016053809	8.83416E-05	0.731681765	0.000905099	0.862669118	6.77649E-06	0.390536287	0.681052047	0.284645472	0.130563677	-0.097575943	0.553659062
AT3G52240	AT3G52240	1.014533427	0.004689015	0.842388357	4.94142E-09	0.95340682	1.10391E-08	0.701821933	0.616300683	0.013394534	0.976570624	-0.340700229	0.022722361
AT5G23405	AT5G23405	1.012085787	0.016298178	0.536376484	0.31177237	0.455639327	0.204194628	1.019185224	0.272375515	0.40357948	0.228145578	0.304473954	0.143232463
AT4G05000	VPS28-2	1.010498727	0.023603956	0.356259593	0.425623038	0.690750795	0.005568844	0.472052726	0.680248595	-0.041874326	0.945176019	0.280713989	0.135933768
AT5G56010	HSP81-3	1.009809331	0.004760503	0.738457594	7.95904E-05	0.470695567	0.022325788	-0.392210711	0.760755998	-0.055453383	0.853148499	-0.434714948	0.003020751

AT1G24560	AT1G24560	1.008376339	0.001806505	0.39151639	0.098580961	0.661980373	0.000830887	0.365498047	0.759046954	0.091452873	0.828846482	-0.63773234	0.000272896
AT2G44820	AT2G44820	1.008305028	0.040461107	0.678454895	0.109355347	0.715144371	0.027650189	0.229873629	0.835453631	0.103162192	0.854284413	-0.391984544	0.288368834
AT2G09795	AT2G09795	1.008207698	0.029095592	-0.3441877	0.667228396			0.000985802	0.999867318	0.208205893	0.710782334		
AT5G27720	emb1644	1.005882557	0.004273037	0.525598461	0.015083987	0.304990042	0.101061216	0.439567207	0.657374576	0.065063453	0.858766684	-0.194959771	0.339647252
AT3G13772	TMN7	1.005320076	0.000518255	0.740096137	7.10587E-06	0.677692799	9.15152E-06	-0.0809593	0.952724258	-0.432797215	0.032013489	-0.307285178	0.038188159
AT5G47620	AT5G47620	1.004812216	0.005730835	0.95510096	2.67657E-06	0.352727156	0.135855243	0.320717784	0.66808962	0.196707734	0.41344799	-0.083403829	0.723391633
AT2G38370	AT2G38370	1.002967524	0.014970229	-0.034952863	0.962803303	0.51376772	0.097755833	0.26601213	0.783584962	0.66187725	0.015230347	0.28721887	0.138697634
AT1G54410	AT1G54410	1.002849047	0.015563966	0.085443488	0.769856709	0.034230227	0.876371208	0.073391263	0.924925439	-0.007488311	0.988079805	-0.690687802	0.34828082
AT1G01210	AT1G01210	1.002542483	0.012178392	0.396473405	0.470316359	0.273017081	0.481775334	0.088260671	0.954839264	0.378038011	0.377885595	-0.21838634	0.35721095
AT1G26830	CUL3	1.001826657	0.003527197	0.7641868	3.72662E-05	0.783501263	1.07892E-07	0.690852413	0.526841194	0.031128254	0.944236118	-0.067076763	0.733013689
AT5G57815	AT5G57815	1.000698489	0.011184935	0.583286423	0.034373971	0.03053921	0.907860542	0.404177586	0.647947756	0.199802899	0.654201154	0.104250023	0.700506187
AT5G60580	AT5G60580	1.000617975	0.000417155	0.695010689	0.004024987	0.778504638	0.000191829	-0.35273807	0.627184543	0.074477403	0.899086634	-0.427387887	0.004076361
AT2G26240	AT2G26240	1.000517548	0.001422845	-0.465760831	0.18970906	0.262732763	0.29816821	-0.165569097	0.885844797	0.098234774	0.858274732	-0.362677162	0.050363073
AT1G54830	NF-YC3	1.000483023	0.003605388	0.86357304	0.00238699	0.522689693	0.016214329	0.283597359	0.777838956	0.064263523	0.924627441	0.000556785	0.997971229
AT5G47380	AT5G47380	-1.000460241	0.047057733	-0.295035572	0.339217301	-0.533694198	0.15198056	0.570914331	0.651780566	0.436791629	0.570610039	0.798255996	0.00028201
AT1G22430	AT1G22430	-1.002158613	0.000760506	-0.628426491	0.005242839	-0.022789755	0.899451091	0.546729535	0.65378996	0.387500323	0.091680639	0.592113856	1.19462E-06
AT1G67560	LOX6	-1.002878667	0.00165163	-0.752625046	0.000793451	-0.761264121	0.010376467	-0.082999018	0.95447214	-0.20684864	0.511188955	0.359109118	0.039697776
AT1G64680	AT1G64680	-1.003616224	0.035127617	-0.533245843	0.032014699	-0.672372194	8.7436E-05	1.048487242	0.476722275	0.252836856	0.67569451	-0.149167211	0.640716898
AT2G25800	AT2G25800	-1.005388478	0.001614821	-0.545759205	0.00537004	-0.179334161	0.383201428	-0.052279633	0.972495118	-0.186165367	0.506760368	0.061423217	0.795305706
AT5G45060	AT5G45060	-1.006098278	0.035471184	-0.577719812	0.04844483	-0.26788109	0.426091005	-0.372634512	0.793776337	0.105315425	0.909090302	-0.1498858	0.683373259
AT5G27360	SFP2	-1.006839038	0.018387079	-0.713941584	0.113014266	-0.555844801	0.057596586	0.767586009	0.616300683	-0.170612502	0.793767617	0.10518547	0.762616382
AT5G22510	INV-E	-1.007229418	0.000277873	-0.053049274	0.837273247	-0.613747578	0.000108856	0.536485759	0.561715693	0.002309064	0.99699153	0.204142821	0.278398091
AT4G17615	CBL1	-1.010023716	0.020930358	-0.079069768	0.866342084	-0.51487357	0.254617925	-0.394989203	0.769724647	-0.077513281	0.929622352	0.230895294	0.312812655
AT2G33250	AT2G33250	-1.011069427	0.022364053	-0.763342082	0.096137892	-0.771317253	0.000303205	0.276259247	0.862046764	0.317695541	0.678137129	0.181427293	0.636208738
AT5G45390	CLPP4	-1.012349317	2.49768E-06	-0.614686191	0.001140177	-0.993387504	3.1682E-08	0.347832757	0.662219342	0.250000387	0.295815926	0.639917348	6.60204E-07
AT1G56590	ZIP4	-1.012946607	0.004104236	-0.167097849	0.712004921	-0.187020838	0.625088368	-0.418803104	0.644839236	0.082204047	0.905535933	0.176899181	0.53614093
AT1G03520	AT1G03520	-1.014395412	0.007338951	-0.489866609	0.193538729	0.132021636	0.669805914	-0.127523347	0.928193379	-0.273746716	0.496280372	-0.196531769	0.456864698
AT4G20010	PTAC9	-1.015853936	0.017116451	-0.451228108	0.331549025	-0.736377808	0.004264325	0.317465915	0.691824202	0.317034547	0.503946392	0.153182872	0.613197531
AT3G19720	ARC5	-1.017201665	0.001667518	-0.562967464	0.000868653	-0.323488946	0.061336498	0.843236991	0.523551884	0.261478822	0.367749288	0.452956745	0.01391785
AT5G55990	CBL2	-1.017652637	0.011427717	-0.580237463	0.160647103	-0.617488448	0.080334672	-0.143222949	0.918157431	0.00892483	0.992021888	0.315242837	0.109087566
AT5G64840	ABCF5	-1.01820569	0.002203441	-0.189077346	0.465032195	-0.399005622	0.059436133	0.646643655	0.647435191	0.233596817	0.254592273	-0.238713881	0.107561055
AT2G29390	SMO2-2	-1.018855807	0.001298964	-0.287886773	0.441530372	-0.373942434	0.230358333	-0.506148422	0.575257546	-0.210210163	0.640365963	0.600601229	0.00018245
AT3G20070	TTN9	-1.019899112	0.018956221	-0.410373058	0.429126797	-0.688004937	0.076317882	0.942011781	0.11427399	-0.105795187	0.879224165	0.141446435	0.643017179
AT1G70740	AT1G70740	-1.019990847	0.019023975	-0.4911781	0.13953743	-0.645454428	0.102640984	-0.339547351	0.757212383	-0.292318028	0.532355117	0.797243778	0.000249493
AT4G33110	AT4G33110	-1.020983358	0.029904298	-0.442601044	0.220019596	-0.358553034	0.296208618	0.431498458	0.685652926	0.130979098	0.865842332	0.541578779	0.001887683
AT5G44070	CAD1	-1.025355414	0.001355116	-0.590756052	0.017806458	-0.375334337	0.268538045	0.033725241	0.983132237	-0.38495242	0.308165197	0.375894306	0.026891359
AT2G44490	PEN2	-1.026217837	0.000224233	-0.713168694	0.012613947	-0.953098271	8.36016E-12	0.159337305	0.89609717	-0.193442982	0.515330631	-0.1235345	0.555882933
AT1G79560	FTSH12	-1.02810214	0.021461669	-0.287023597	0.58167279	-0.817719211	0.00177035	-0.070827847	0.973864665	0.020297561	0.971701608	0.23818649	0.11607034
AT3G13490	OVA5	-1.030847313	0.000251152	-0.210534739	0.468479533	-0.006306491	0.98033627	0.184004815	0.828875452	0.127235672	0.727051106	0.377119086	0.007551217
AT2G40400	AT2G40400	-1.032032794	0.000227932	-0.2555336	0.320256812	-0.556722198	0.000226841	0.442670363	0.598901706	0.286111011	0.399530708	0.007875648	0.979855671
AT1G01860	PFC1	-1.032406061	0.04237757	-0.369417608	0.605156927	-0.446033256	0.115538578	0.572040831	0.704225272	0.111590512	0.928310357	0.316932927	0.253672375
AT4G01090	AT4G01090	-1.032807718	0.004287115	-0.294465265	0.366684986	-0.033457175	0.925668253	-0.084101812	0.967199692	-0.701252165	0.000674342	0.468589725	0.022797217
AT5G16715	EMB2247	-1.034688702	0.000705395	-0.631447299	0.028555293	-0.147208771	0.483644116	0.122778415	0.92001591	0.164062762	0.630851499	0.597152994	3.39805E-05
AT2G19680	AT2G19680	-1.035651812	0.016034229	-0.557558882	0.337890052	-0.563589105	0.061615414	-0.119380842	0.93556951	0.187673919	0.689231427	0.112161281	0.649588495
AT2G40270	AT2G40270	-1.03662766	0.005265054	-0.539285166	0.012706379	-0.805631817	7.94618E-05	0.260337659	0.822093569	-0.566833579	0.062377578	0.407880063	0.009763554
AT1G51680	4CL1	-1.037908374	0.0249222	-0.245038244	0.547218349	-0.939339871	0.000180867	-0.588099952	0.696148644	-0.31102474	0.05978971	-0.222518052	0.256766304
AT1G08470	SSL3	-1.038873582	0.016267218	-0.596872691	0.208769928	-0.819342957	0.127328702	-0.46771802	0.717709314	-0.130627803	0.827181504	0.284808039	0.351033845
AT4G00370	ANTR2	-1.041882082	1.28257E-05	-0.546778867	0.036440006	-0.408380182	0.00986856	0.506934329	0.616300683	0.011078609	0.986818647	0.319782446	0.073458981
AT2G32230	PRORP1	-1.042980214	0.048599195	-0.342325707	0.533252885	-0.94930591	1.14503E-06	0.224588266	0.820789421	0.282285563	0.270268074	0.621695788	8.0739E-06
AT2G01110	APG2	-1.044283453	0.004808303	-0.384743235	0.115813904	-0.740330647	2.83137E-08	0.522869562	0.653363031	0.283684096	0.39892496	0.231343383	0.107110653
AT4G14070	AAE15	-1.045169602	0.000451089	-0.319030383	0.214363433	-0.74096287	0.001263033	0.347231944	0.721140128	-0.004697077	0.995225758	-0.078330032	0.731748502
AT2G26910	ABCG32	-1.049599236	9.09302E-07	-0.800461628	5.95721E-08	-0.361632548	0.007751938	-0.101077609	0.91867804	-0.441557069	0.014101575	-0.14740315	0.372773271
AT4G37920	AT4G37920	-1.049766801	0.004353112	-0.869937492	0.00267114	-0.725100461	7.17589E-05	0.060757819	0.976183443	0.370913327	0.306248333	0.43139248	0.088591649
AT1G61430	AT1G61430	-1.049894281	0.035284677	-0.213666736	0.761737018	0.76114313	0.001216107	-0.426281694	0.729284891	-0.087965093	0.930188697	0.574887387	5.85785E-05
AT1G22060	AT1G22060	-1.056521618	0.000163938	-0.955513416	7.91704E-05	-0.405754654	0.092752909	-0.244282954	0.79601276	-0.080936357	0.871370942	0.065140958	0.765967725
AT1G69550	AT1G69550	-1.056807986	0.00925901	-0.64922461	0.218632269	0.509905295	0.244385067	0.25341409	0.826687709	0.348048073	0.538897638	-0.020338452	0.969252469
AT4G35360	AT4G35360	-1.05755426	0.002363297	-0.141831061	0.710265504	0.211573031	0.408561209	-0.316677314	0.764352616	0.248219521	0.49344706	0.097443233	0.67017756
AT2G20930	AT2G20930	-1.057985483	0.000263756	-0.42142829	0.132645299	-0.817886826	0.003316548	-0.013452713	0.993804788	-0.040676156	0.951805765	0.254245239	0.131226872
AT5G56730	AT5G56730	-1.059250321	0.03258682	-0.731093729	0.140449228	-0.152010646	0.73067473	-0.17753331	0.919341928	0.00649928	0.995196598	0.213991128	0.414064674
AT4G35250	HCF244	-1.061367006	0.003246835	-0.465591623	0.035333713	-0.753461278	4.26413E-08	1.134020684	0.420106526	0.39075707	0.232088784	0.543092804	0.000915858
AT1G79600	AT1G79600	-1.062806023	0.000406482	-0.267403014	0.260960464	-0.346803436	0.013946555	0.539116169	0.721967244	0.05917641	0.917795278	-0.308818173	0.131524239
AT3G61670	AT3G61670	-1.064118476	0.003634423	-0.788735081	0.002604517	-0.603113781	0.028203129	-0.065734173	0.969268687	0.005573441	0.99349199	0.340765101	0.107374629
AT5G59750	RIBA3	-1.064201194	0.026334623	-0.417975606	0.380216412	-0.905026799	3.73561E-05	0.493441906	0.610291087	0.378667442	0.493875814	0.377225426	0.117680853
AT3G13180	AT3G13180	-1.06673941	0.000112089	-0.246371598	0.477380708	-0.575946614	0.000703076	0.350780949	0.726214276	0.26677215	0.530836115	0.824131175	3.22018E-09
AT3G10270	GYRB1	-1.066825062	0.001035493	-0.610400243	0.162506116	-0.829835904	0.061060863	0.272685628	0.801461921	0.079209051	0.901005702	0.416881664	0.011916594
AT5G51200	EMB3142	-1.068640533	6.65374E-06	-0.986790178	2.65359E-05	-0.69537008	3.34251E-07	0.621709335	0.563758673	-0.32485969	0.114379534	0.0953794	0.626317813
AT5G17050	UGT78D2	-1.068901613	0.009774109	-0.088918583	0.877658877	-0.858130938	0.095547672	0.340961405	0.77590923	-0.181924533	0.91048199	0.275162028	0.33341775
AT4G34730	AT4G34730	-1.069629002	0.000415293	-0.51109464	0.054633989	-0.857321577	8.10261E-07	0.455095967	0.720012409	0.15704306	0.727051106	-0.117085064	0.578140895
AT5G47370	HAT2	-1.069931183	8.10866E-05	-0.763897456	0.004343548	-0.021290341	0.91992051	0.231434657	0.66808962	-0.1081914	0.784731396	-0.238333604	0.100727351
AT3G01060	AT3G01060	-1.070283616	0.02732061	0.28932899	0.318045257	-0.563996532	0.124649153	0.821294923	0.589474071	0.660139815	0.112390029	0.077713856	0.845934082
AT5G26820	IREG3	-1.072523971	0.005414496	-0.559287386	0.016641367	-0.531827617	0.003921778	0.421358043	0.660443668	-0.058736184	0.922383664	0.401812241	0.015776154
AT3G14790	RHM3	-1.073190573	0.000585583	-0.923700734	0.005076119	-0.230113302	0.266155449	-0.39223218	0.694676172	-0.293651928	0.345300689	0.02061251	0.940783936
AT1G06240	AT1G06240	-1.073494327	0.031367999	-0.930893227	0.063989939	-0.417622455	0.334110404	0.556211258	0.653363031	0.539597502	0.437231376	0.346315921	0.303044835
AT1G58080	ATP-PRT1	-1.073790832	4.28127E-07	-0.207884894	0.335459081	-0.341950203	0.065925792	0.138473197	0.867333727	0.011197617	0.984765765	0.171760447	0.238968902
AT3G58140	AT3G58140	-1.074960859	9.71253E-05	-0.704189424	0.025332112	-0.965018278	1.34965E-11	0.299810907	0.787698816	0.140302399	0.743050453	0.304741891	0.040562343
AT5G44150	AT5G44150	-1.075055085	0.0410012	-0.452471169	0.462982996	-0.704757579	0.131760113	0.076890385	0.952724258	0.165368702	0.818414231	0.325975001	0.299661755
AT5G07000	ST2B	-1.076087907	0.001745819	-0.653989985	0.061024886	-0.003581102	0.990341275	0.284928457	0.732113981	-0.363533289	0.182805783	-0.3298299	0.032967466
AT5G63470	NF-YC4	-1.076384509	0.008315314	-0.41038844	0.160539808	-0.041381607	0.876914496	0.182045487	0.917312631	0.327678667	0.529080536	0.080600752	0.734979737
AT2G34460	AT2G34460	-1.076842554	0.023480489	-0.22412289	0.450789895	-0.539160236	0.004760962	1.579619534	0.315604719	0.270775184	0.67431887	0.463411074	0.025552844
AT1G63260	TET10	-1.081186099	0.000906072	-0.741301764	0.020090981	-0.795465364	0.008869167	0.143187613	0.902801933	0.103464687	0.883566526	0.064620397	0.834081601
AT1G62430	CDS1	-1.082199304	0.000130155	-0.522140868	0.15968405	-0.447352345	0.312638307	-0.24620883	0.769603147	-0.12621201	0.815610645	-0.076505675	0.728193532
AT5G66570	PSBO1	-1.082269777	0.008271191	-0.341001145	0.041154319	-0.474091244	1.36294E-07	2.496014891	0.065594488	0.441270744	0.042524084	0.631913583	0.000285778
AT1G29690	CAD1	-1.083196181	0.00884436	-0.61364256	0.023622281	-0.640996289	0.020514413	-0.743365866	0.389773786	-0.456195848	0.159548469	0.587382313	0.003385066
AT3G03630	CS26	-1.086368653	0.007936055	-0.530062069	0.150335484	-0.763626599	0.000667106	-0.078190396	0.978345558	-0.126399297	0.831451868	0.747977497	4.77424E-06
AT3G22150	AT3G22150	-1.08691456	0.019261727	-0.609624177	0.026978862	-0.415919644	0.309081349	-0.018400095	0.991791107	0.353275962	0.36223266	0.230038783	0.235426908
AT5G14460	AT5G14460	-1.087098839	0.005401646	-0.658543447	0.006720618	-0.733384103	0.003202705	-0.01354581	0.993513121	0.345404828	0.25004987	0.272246502	0.063107855
AT4G33220	PME44	-1.087299286	0.00012541	-0.376549581	0.266856406	-0.958414983	0.000407644	-1.195214983	0.274120453	-0.754251038	1.08737E-06	-0.446016222	0.000116939
AT2G46100	AT2G46100	-1.087933787	0.002940539	-0.713831138	0.195263528	-0.812143677	0.001150524	0.334470932	0.81577697	0.105834857	0.922482268	-0.54960964	0.082697811
AT4G34830	MRL1	-1.088451926	3.19183E-05	-0.18609012	0.47552302	-0.248074327	0.260833763	0.254840507	0.835111797	0.25335369	0.364511686	0.41780207	0.002663576
AT3G19170	PREP1	-1.089653275	6.22741E-07	-0.398319178	0.063000639	-0.311342812	0.161873025	0.23559408	0.742086143	0.022267584	0.95818046	0.036771575	0.877524271
AT1G18670	IBS1	-1.089660401	0.00036957	-0.520909947	0.095853857	-0.652863429	0.000690677	-0.074558788	0.95447214	-0.174532123	0.613672437	0.060726381	0.777259321
AT4G28730	GrxC5	-1.09052669	0.04468276	-0.091187734	0.876482205	-0.927625384	0.000519367	-0.470096384	0.676109289	0.507743557	0.279620754	0.282608731	0.256272909
AT4G02540	AT4G02540	-1.090703937	0.000277724	-0.986282364	9.9321E-05	0.179261686	0.853445624	0.554156154	0.385221483	0.004675546	0.994809352	0.208184378	0.725114275
AT4G03150	AT4G03150	-1.093107767	0.008158131	-0.863886872	0.080604912	-0.375412705	0.35208246	1.413663558	0.324030693	0.145924486	0.904726557	0.563094484	0.038674883

AT5G49970	PPOX	-1.098471677	0.001117732	-0.369390456	0.216005003	-0.419585148	0.050914192	0.405112998	0.616300683	-0.244954593	0.554658146	0.434615634	0.00560624
AT1G19800	ABCI14	-1.099680966	0.003781458	-0.68563604	0.050669494	-0.645401669	0.017476425	0.188990666	0.830747236	-0.042723151	0.947508153	0.31517555	0.082497111
AT5G65010	ASN2	-1.102059098	0.009510476	0.147320334	0.634009618	-0.239570207	0.289679566	0.029204749	0.989993885	-0.004052501	0.996204637	0.426218867	0.002039267
AT5G46420	AT5G46420	-1.103366713	3.98384E-07	-0.558156478	0.001682246	-0.669588694	3.71626E-09	0.575987958	0.524357407	0.222381696	0.38944897	0.329670483	0.050217709
AT1G49840	AT1G49840	-1.105115106	0.021383715	-0.893031916	0.001630471	-0.758520521	0.006148003	0.146358	0.924925439	0.255700453	0.540482493	0.078211964	0.796458524
AT1G53730	SRF6	-1.105119761	0.001015542	-0.783804013	0.016305615	-0.654128324	0.009794577	-0.364633702	0.616300683	-0.471480423	0.066562382	-0.00955902	0.972388595
AT2G35410	AT2G35410	-1.105469606	4.57408E-05	-0.503094743	0.104463239	-0.650975128	0.000553514	0.575847767	0.494091063	0.12064405	0.82877322	0.786881369	1.59236E-05
AT2G39190	ATATH8	-1.105610461	3.96117E-05	-0.501259418	0.002221086	-0.431073063	0.011456816	0.39350143	0.672844426	-0.012431414	0.986818647	0.540288806	0.004192213
AT5G17530	AT5G17530	-1.107038796	0.001498757	0.21083314	0.494911149	0.083266783	0.799033433	-0.11557609	0.930238321	0.107446104	0.869431803	0.17676221	0.372954076
AT1G70250	AT1G70250	-1.107281646	0.021398054	-0.610208972	0.489551172	-1.332954584	0.093329068	0.23257438	0.906077763	0.119409485	0.929498792	-0.203844836	0.678972792
AT1G77060	AT1G77060	-1.108082298	0.001189624	-0.615619833	0.003144491	-0.550292428	0.000561751	0.607684453	0.469878733	0.402441768	0.161558082	0.870712972	2.68286E-07
AT3G54660	GR	-1.109316095	1.70091E-07	-0.486088574	0.001908978	-0.792545862	5.25942E-07	0.197056556	0.743849982	0.167272155	0.478838042	0.176774375	0.176818081
AT2G35260	AT2G35260	-1.111617207	0.006588753	-0.515109177	0.000926991	-0.572479026	0.001470256	1.749184918	0.13090699	0.144788047	0.719986639	0.64069723	2.23359E-06
AT1G11720	SS3	-1.112229781	1.01771E-05	-0.521817398	0.045277039	-0.387533593	0.036634204	0.525367717	0.62738272	0.461861996	0.092523083	0.756074301	2.01295E-08
AT5G12150	AT5G12150	-1.112359965	2.77473E-05	-0.604815798	6.74572E-05	-0.931121014	7.92479E-14	-0.003779299	0.997790351	-0.063312361	0.826821818	0.181422576	0.303816665
AT1G12250	AT1G12250	-1.114150613	0.046084318	-0.6959739	0.040449122	-0.956170243	8.93011E-14	0.494229116	0.722781473	0.502803346	0.230927385	0.848360106	6.48087E-06
AT1G16880	ACR11	-1.121450487	9.99451E-08	-0.534406513	0.009710903	-0.719420985	1.71713E-06	0.15602686	0.874176467	0.131543324	0.680253239	0.274296396	0.087218715
AT1G76730	COG0212	-1.121482272	0.005271866	-0.906716601	0.00044234	-0.452457713	0.016971996	0.565783571	0.62738272	0.289860251	0.556330533	0.564308099	0.002038304
AT5G12050	AT5G12050	-1.122654837	6.44652E-05	-0.605557219	0.02994402	-0.501880767	0.031443856	1.077457329	0.313609174	0.377516247	0.168222434	0.391230601	0.075579123
AT5G53580	PLR1	-1.123845306	0.000506147	-0.647325495	0.008666443	-0.939800045	0.000111667	0.152726673	0.870396305	0.403067791	0.155596055	0.382729131	0.021797159
AT5G67470	FH6	-1.123872349	0.005930114	-0.609337689	0.339093282	-0.81212939	0.000968569	-0.749061148	0.474740724	-0.290831028	0.506477549	-0.56810553	0.02306099
AT3G13980	AT3G13980	-1.127014653	0.035833161	-0.847550751	0.050634717	0.102077281	0.811831879	0.082584537	0.970302657	-0.702544349	0.041479375	-0.797629927	3.32427E-07
AT2G35370	GDCH	-1.128370065	6.94343E-05	-0.597473633	0.000587834	-0.224381385	0.11797325	1.192083848	0.228806712	0.588779743	0.003773583	0.724462067	2.89379E-08
AT1G01430	TBL25	-1.129394498	0.000163643	-0.453758128	0.164731038	-0.345852784	0.093249816	-0.351286966	0.665673432	-0.575295194	0.000198996	-0.254264965	0.238847315
AT4G32880	HB-8	-1.12940601	0.000117842	-0.895364443	7.22114E-05	-0.010360327	0.981790411	-0.284561323	0.653363031	-0.283319856	0.295776096	-0.430664768	0.032019992
AT5G23130	AT5G23130	-1.131731113	0.002416379	-0.740342375	0.041076779	-0.363426906	0.473072716	-0.204342572	0.855010607	0.080034305	0.918766875	0.118226847	0.699344098
AT4G13220	AT4G13220	-1.13256436	0.031483402	-0.801029568	0.125114544	-0.99215036	0.057464303	-0.299260331	0.78630406	0.124540022	0.905907141	0.316695769	0.267217976
AT1G18620	TRM3	-1.135720829	0.006033445	-0.530793776	0.004145742	-0.094698846	0.583509325	0.618832067	0.66524984	-0.117405006	0.796178186	-0.092749532	0.681350366
AT4G25130	PMSR4	-1.138720944	2.01134E-05	-0.232133036	0.406753591	-0.996274948	2.25792E-11	0.550612548	0.602119156	0.110341037	0.724055703	0.382681309	0.010967546
AT3G23700	AT3G23700	-1.142227526	0.001504201	-0.763739308	8.83152E-07	-0.591193268	5.38606E-05	0.828997041	0.546687098	0.240075136	0.42662725	0.206174984	0.270087795
AT5G13460	IQD11	-1.142895086	0.000274558	-0.836442507	0.003146931	-0.801621123	0.006140708	-0.283548863	0.759046954	-0.392423406	0.115937316	0.215173975	0.247533591
AT1G55690	AT1G55690	-1.145541827	0.008513952	-0.709014758	0.019347637	-0.415889107	0.103547642	-0.302235458	0.876428092	-0.307218316	0.266105366	-0.179293977	0.342126031
AT4G16890	SNC1	-1.145860997	0.002090298	-0.831660754	0.000113225	-0.01154212	0.972965179	1.074417027	0.430181703	0.143682218	0.777094046	0.468610477	0.119868757
AT5G46110	APE2	-1.146145833	0.002052869	-0.734940815	2.84659E-12	-0.139643135	0.22133675	2.294186141	0.06223917	0.461395465	0.096913953	0.48093789	0.001094459
AT4G14550	IAA14	-1.146289482	0.029299313	-0.833484578	0.010194226	-0.337919313	0.135637472	0.804791948	0.582068518	-0.006418673	0.994950288	-0.148662439	0.567571222
AT3G58620	TTL4	-1.146387834	0.013728066	-0.94796996	0.005591403	-0.696425998	0.037633098	-0.334180879	0.760059267	0.237926545	0.531182014	0.357825907	0.018354083
AT2G03310	AT2G03310	-1.146674457	0.027368625	-0.955736847	0.095067529	-0.833283573	2.86998E-05	1.865173527	0.379538351	0.576977656	0.611338103	-0.695483204	0.044688037
AT1G80650	RTL1	-1.148347135	0.041476896	-0.8266925	0.005065829	-0.211941285	0.488928855	0.520421637	0.67307941	-0.051992559	0.958669515	0.40963018	0.182249958
AT2G38740	AT2G38740	-1.149120189	0.007474793	-0.82926883	0.166459391	-0.819521833	0.003321754	-0.630070319	0.731041294	0.150573178	0.828846482	0.016711072	0.972921616
AT2G20610	SUR1	-1.150619652	0.003086034	-0.382364768	0.37329024	-0.516291366	0.013077373	-0.188001071	0.907283981	-0.184470974	0.616763356	0.284858541	0.139396802
AT2G45010	AT2G45010	-1.151400647	0.003887642	-0.58881548	0.065698316	-0.605178209	0.019230253	-0.26277348	0.780423734	-0.186406485	0.67277392	0.522421481	0.005283621
AT1G50450	AT1G50450	-1.152893268	0.001443149	-0.293723076	0.368911829	-0.295658356	0.203047813	0.894104214	0.514451369	-0.082677978	0.924868537	0.564468521	0.003100225
AT2G20290	XIG	-1.154071811	0.037209636	-0.626454798	0.131987724	-0.580183179	0.1247347	-0.540443028	0.769502096	-0.245379803	0.580634074	0.014244064	0.972457306
AT1G53500	MUM4	-1.154592883	0.020815661	-0.585516857	0.376156102	-0.880514776	0.000101714	-0.938750689	0.486378875	-0.201237238	0.730592522	0.108559101	0.637571052
AT4G26370	AT4G26370	-1.161279058	0.000269054	-0.773429156	2.8012E-06	-0.540275864	0.000117292	0.734394439	0.598901706	-0.220656839	0.563104532	0.337550477	0.025542946
AT4G19710	AK-HSDH II	-1.162131459	0.005623044	-0.809677092	0.007761484	-0.771158494	0.00038911	-0.101534185	0.962336309	0.029361594	0.958438477	0.690544014	7.59307E-05
AT5G07630	AT5G07630	-1.162297201	0.001963793	-0.766829526	0.006429616	-0.104046928	0.706330819	0.320725399	0.664635521	-0.052365878	0.937319087	0.565633046	0.002445677
AT4G20310	AT4G20310	-1.162889727	0.047816699	-0.242183796	0.758604757	0.009645524	0.986461882	0.642248744	0.676109289	-0.061461068	0.95818046	0.202698563	0.539634856
AT5G52520	OVA6	-1.169300379	0.000131916	-0.813525452	0.000608771	-0.010395212	0.969001857	0.276213305	0.706790319	0.07656036	0.894530102	0.271784261	0.098732509
AT5G42765	AT5G42765	-1.170808652	0.001457164	-0.905392275	0.002902696	-0.749546558	0.003237636	0.253251638	0.895706838	0.243949048	0.566973384	0.467516434	0.014298302
AT5G39020	AT5G39020	-1.17632961	0.026427731	-0.85417711	0.330218076	0.687752355	0.540839961	-0.383512846	0.956315558	-0.781425678	0.612245594	-0.329445034	0.548317689
AT3G58160	XIJ	-1.177821692	0.042137341	-0.252743705	0.720474629	-0.162751731	0.779617842	0.807404465	0.501217475	0.391693418	0.53583656	0.294838934	0.300139705
AT1G62250	AT1G62250	-1.17930577	0.014872902	-0.800519847	0.109524771	-0.358251966	0.077791698	-0.090930693	0.967199692	0.231410331	0.794822197	0.120766301	0.63213429
AT5G62720	AT5G62720	-1.185117181	0.005249036	0.083170879	0.760517867	-0.717693201	0.000517023	0.443503947	0.602119156	0.073937677	0.95555756	0.140104348	0.436378672
AT5G53450	ORG1	-1.190560308	0.000225921	-0.931538373	1.73204E-05	-0.846285767	2.20229E-07	0.652275595	0.606948021	0.143246334	0.755752983	-0.092031132	0.687802012
AT5G48900	AT5G48900	-1.190694902	0.005917927	-0.500293716	0.175649023	-0.339911698	0.218227429	-0.888222133	0.577728436	-0.451918734	0.011035748	0.115868521	0.517142827
AT3G04910	WNK1	-1.195879026	2.99573E-06	-0.789740502	2.55514E-07	-0.646902567	1.63365E-06	-0.112737414	0.912363588	-0.27015409	0.2149371	0.03841787	0.833007341
AT5G13410	AT5G13410	-1.196692937	0.003842647	-0.134302681	0.709358034	-0.876423824	0.000198513	0.459203818	0.743050938	0.424003288	0.347875594	0.318402599	0.168055274
AT3G47570	AT3G47570	-1.198490123	0.000897003	-0.688632133	0.016481917	0.080085893	0.841949503	-0.438488956	0.674232756	-0.137159703	0.820469547	0.044903767	0.855158201
AT4G01690	PPOX	-1.199391276	1.75808E-08	-0.70390197	0.001681812	-0.744113696	0.000105775	0.32858165	0.66524984	0.23238688	0.413832097	0.208425146	0.107455497
AT4G33140	AT4G33140	-1.203628301	0.047078035	-1.231718663	0.201521887	-0.385140157	0.457180996	-0.256151094	0.837258505	0.208945437	0.802042097	0.501973319	0.015316589
AT1G68830	STN7	-1.2046317	0.010546184	-0.266980745	0.301607136	-0.645318371	0.001470256	1.458377464	0.379538351	0.324955449	0.549887825	-0.148861748	0.467690423
AT1G66330	AT1G66330	-1.205283358	0.000989583	-0.558899515	0.191774516	-0.620570814	4.9304E-05	0.216073592	0.800410801	0.315163606	0.274431207	0.210846312	0.266453963
AT1G27680	APL2	-1.206915764	0.018152504	-0.788397087	0.018671743	-0.276482931	0.555045761	-0.351641665	0.804372175	-0.138323469	0.864675247	0.488510045	0.021317356
AT5G11250	AT5G11250	-1.20771998	0.002754464	-0.986285078	0.047076997	-0.784620014	0.196164701	-0.296558513	0.824938073	-0.032500174	0.967846409	0.601433259	0.106296535
AT3G49290	ABIL2	-1.208066099	0.016553665	-0.734849932	0.004721072	-0.661667736	0.118208606	0.275031128	0.762028951	0.153594907	0.780834039	0.463072069	0.147648189
AT4G18810	AT4G18810	-1.208153929	0.000517207	0.099178332	0.804509802	-0.63989195	0.10406643	1.049389426	0.56497814	0.219058444	0.678528221	0.474322769	0.077004164
AT3G51820	G4	-1.211687317	1.07015E-06	-0.591388469	0.000429963	-0.96198252	3.50589E-09	0.2862981	0.758662788	0.206188041	0.498823998	0.566702205	0.000241859
AT5G13120	Pnsl5	-1.216283679	2.68987E-08	-0.612775928	0.020498843	-0.957790522	1.38328E-12	0.264665697	0.731041294	0.20941086	0.513601757	0.400492047	0.010707715
AT4G25960	ABCB2	-1.216291092	3.65394E-07	-0.828468719	0.000706813	-0.393219663	0.022992308	-0.638022104	0.385351075	-0.617012337	0.037100036	-0.118628143	0.575521738
AT1G73060	LPA3	-1.216604293	4.66709E-05	-0.594809209	0.015282924	-0.413009711	0.071115045	0.569989046	0.482795025	0.311282193	0.262547221	0.532308231	0.000340721
AT1G17360	AT1G17360	-1.217560661	0.006258242	-0.076767106	0.851682787	-0.627643124	0.00702553	-0.432421441	0.637815859	-0.263223724	0.51084463	0.161632227	0.42849546
AT3G63190	RRF	-1.217800658	4.37166E-07	-0.880531711	1.08085E-05	-0.83208427	3.24696E-12	0.24201451	0.76184275	0.153975015	0.573996347	0.678810533	1.35896E-08
AT1G01080	AT1G01080	-1.218788736	6.13978E-08	-0.822685273	0.003277386	-0.695497903	0.000672344	0.600882424	0.443483247	0.311034564	0.381034551	0.738050594	7.97013E-05
AT5G47840	AMK2	-1.21958498	6.82908E-05	-0.751846093	1.66984E-06	-0.625272826	1.6431E-06	0.705903841	0.588988003	0.051190878	0.934318497	0.408172424	0.001320606
AT3G14390	AT3G14390	-1.220164803	2.32103E-05	-0.873223782	0.000247211	-0.952386068	7.23971E-06	-0.094610574	0.936758504	-0.143688475	0.660372631	0.46295677	0.018449686
AT1G06850	bZIP52	-1.224396437	0.015139411	-0.866604278	0.048291997	-0.769785044	0.323207005	-0.659662348	0.62738272	0.059566996	0.930422599	0.460311646	0.021930387
AT5G60920	COB	-1.225274594	2.2E-05	-0.913424165	1.74892E-07	-0.388659951	0.012566019	-0.6221436	0.372975709	-0.505594841	0.005231588	-0.264672726	0.201214388
AT5G28750	AT5G28750	-1.225965876	2.26382E-06	-0.608885936	0.026356621	-0.656885017	9.22735E-05	0.567039752	0.601921549	0.661791125	0.009686411	0.323947811	0.221463422
AT1G62750	SCO1	-1.227928573	0.000170386	-0.72093726	2.24009E-08	-0.955463769	2.35111E-14	0.876264899	0.517612286	0.176719963	0.511188955	0.396698754	0.016702222
AT1G13820	AT1G13820	-1.228116629	0.000563302	0.117317545	0.74934387	-0.567713297	0.005200647	1.042731481	0.330487988	0.178068694	0.771376139	0.403930376	0.039946441
AT1G29070	AT1G29070	-1.229530658	2.48077E-06	-0.325028441	0.019453524	-0.710971487	1.42377E-09	0.73156931	0.457206007	0.398960397	0.058461349	0.781436002	5.26945E-09
AT4G20910	HEN1	-1.231959337	0.013020382	-0.486733967	0.322708739	-0.214970668	0.638751261	0.271479327	0.888277803	-0.102365654	0.896410026	0.197805037	0.501521062
AT2G28305	LOG1	-1.232521597	0.03924285	-0.636199931	0.017095016	-0.134613099	0.684813862	1.045290647	0.634709839	0.234580657	0.784525395	0.825514651	8.27167E-05
AT2G31170	SYCO ARATH	-1.232756308	0.000895143	-0.664941928	0.235747967	-0.873855527	0.001298085	0.153534866	0.908903757	0.140744941	0.784223487	0.51456017	0.005540472
AT5G64940	ATH13	-1.235982762	0.000357705	-0.63416808	2.37096E-05	-0.186320361	0.452601624	1.199529976	0.28061619	0.416763511	0.039484958	0.681136654	1.6547E-07
AT5G02790	GSTL3	-1.237270427	1.57955E-07	-0.37215127	0.131012267	-0.55423142	3.90686E-05	0.258606444	0.818724846	0.261502812	0.422674488	0.575740553	5.91525E-06
AT5G44870	LAZ5	-1.240990683	0.003754313	-0.817878506	0.108908676	-0.26475219	0.357023711	0.600923354	0.641679337	-0.312860347	0.798599938	0.131275226	0.794612983
AT4G23550	WRKY29	-1.242070586	0.010141822	-1.859398308	0.100191635	-0.845032206	0.027589781	0.130673084	0.967195835	-0.241491569	0.794004103	-0.152754131	0.738428166
AT4G35920	MCA1	-1.243280443	1.98886E-06	-0.817898215	0.002343246	-0.798743228	3.82994E-05	0.134188922	0.866262307	-0.151245002	0.578542261	0.200951337	0.324767749
AT5G01530	LHCB4.1	-1.246335763	0.001818687	-0.723586577	9.69098E-08	-0.509390826	1.35015E-06	2.079457841	0.114681201	0.501880593	0.019886905	0.660863022	9.2523E-05
AT1G05200	GLR3.4	-1.246426041	0.000766723	-0.522603017	0.04710896	-0.087669202	0.883075636	0.116557369	0.929590087	0.127433638	0.810706099	0.305792573	0.217807647
AT1G44350	ILL6	-1.247540951	0.000550185	0.236426831	0.696307747	-0.779436141	0.00126763	-0.377990366	0.644839236	-0.274823004	0.577315828	-0.997114263	6.794E-11
AT5G03880	AT5G03880	-1.249420212	1.24925E-06	-0.499897888	0.00766651	-0.798518071	2.20061E-10	0.469929421	0.66524984	0.022788064	0.964567705	0.232616626	0.144758806

AT1G16650	AT1G16650	-1.250190879	0.040101718	-0.563953943	0.296870878	0.176081142	0.710630857	0.327918102	0.742807747	-0.239634357	0.752215159	-0.077520208	0.807880563
AT1G63350	AT1G63350	-1.251466339	0.024091041	-1.134812088	0.122304516			1.217003186	0.49571945	-0.001004772	0.99989203	-2.042572455	0.761927143
AT1G02560	CLPP5	-1.252769669	1.79605E-06	-0.473394803	0.000606753	-0.70548524	8.48196E-09	0.298500213	0.746429074	0.041109867	0.930422599	0.358496981	0.043982929
AT5G38660	APE1	-1.253163789	0.00022541	-0.424222053	0.01913892	-0.734834013	4.48877E-10	0.74211782	0.552008826	0.159049866	0.690120555	0.166685811	0.370217869
AT5G66130	ATRAD17	-1.257131768	0.019356668	0.468867788	0.471242573	-0.778388364	0.121493074	0.45958976	0.754617282	-0.264099949	0.773814133	-0.122482054	0.742676022
AT2G16280	KCS9	-1.257478551	9.65306E-05	-0.914800732	0.006769845	-0.777127554	0.00598464	-0.466835199	0.603326479	-0.383508271	0.213715912	-0.356057996	0.050390966
AT5G66470	AT5G66470	-1.258290302	8.44577E-05	-0.530185836	0.045655685	-0.697195344	0.000140038	0.138370212	0.87876612	0.076883104	0.885326824	0.522304295	0.000316852
AT3G60750	AT3G60750	-1.258753475	3.93777E-09	-0.653687185	0.000400568	-0.978475038	3.28458E-24	0.090462959	0.939540455	-0.144960933	0.475376987	0.338516893	0.039589087
AT5G54160	OMT1	-1.259726709	2.80194E-05	-0.31458991	0.266217038	-0.505910009	0.001407286	-0.159433643	0.861673753	-0.455451393	0.02032355	0.128540587	0.375611064
AT1G69200	FLN2	-1.259880184	0.007638943	-0.856205579	0.179545821	-0.93996511	0.000335619	0.122340042	0.957410897	-0.192155618	0.672741445	0.70553622	7.51304E-06
AT1G14030	LSMT-L	-1.261972676	0.000104585	-0.363815982	0.391745092	-0.804682657	0.000202625	0.6356492	0.66524984	0.272702699	0.681170023	0.85533508	2.3258E-09
AT5G09660	PMDH2	-1.266981217	0.001566456	-0.601050555	3.75134E-05	-0.22783397	0.01997992	2.310573086	0.133245343	0.434277542	0.032844586	0.508698213	0.002063527
AT3G06070	AT3G06070	-1.267528254	0.015044605	-0.845793747	0.002680717	-0.602743621	0.058089533	0.296049838	0.785348132	0.058615595	0.933194561	0.447733889	0.040662673
AT5G04310	AT5G04310	-1.26883919	0.027291155	-1.208278571	0.270104097	-0.385858609	0.713638128	-0.519565599	0.883652513	-0.709224123	0.215154282	0.332863588	0.509276551
AT5G01920	STN8	-1.272702475	2.6986E-06	-0.696816977	0.010278711	-0.083189861	0.669576478	0.767528232	0.579644733	0.356942083	0.158543269	0.27221983	0.079436783
AT3G25805	AT3G25805	-1.273403512	0.004596571	-0.646745201	0.178429635	-0.705802874	0.006764139	0.318653681	0.765693011	0.18046795	0.778684649	0.726725053	0.000174866
AT3G25690	CHUP1	-1.275998575	7.27074E-05	-0.541498817	0.006776968	-0.597193482	1.58448E-06	0.334646831	0.769713722	-0.096598599	0.79996985	-0.59980793	1.90676E-06
AT5G48545	HINT3	-1.277624836	0.001813028	-0.643875408	0.023496734	-0.698696645	0.001948133	-0.073115785	0.958600664	-0.354909312	0.267247575	0.361571618	0.04425902
AT2G41830	AT2G41830	-1.278996777	0.026641127	-0.537471208	0.439842686	-0.688356755	0.167307065	-1.237394632	0.343493265	-0.519809553	0.190118429	-0.457881474	0.071355337
AT1G31230	AK-HSDH I	-1.27955705	0.00026541	-0.711784385	0.024549543	-0.716738015	0.000107348	0.164112735	0.890241742	-0.011998381	0.985120284	0.597934217	0.000213971
AT4G04880	AT4G04880	-1.280516622	0.037520077	-0.189214803	0.749457723	-0.487751964	0.147900225	-0.004822363	0.998419999	0.301941234	0.564931556	0.455781228	0.049936818
AT3G59300	AT3G59300	-1.282443676	0.032746219	-0.058626029	0.918171613	-0.613823428	0.182019334	0.067606569	0.960530601	-0.314492154	0.53525964	-0.106708133	0.656995047
AT3G07670	AT3G07670	-1.282465943	2.91058E-06	-0.49147463	0.047359005	-0.695417756	8.34994E-05	0.330773971	0.832523868	0.362861197	0.259031822	0.61483769	0.000381763
AT4G26690	SHV3	-1.284874974	0.000124777	-0.840383607	5.75185E-06	-0.99160035	3.27587E-06	-0.731232255	0.131910635	-0.805060305	0.003794022	-0.417213029	0.056104684
AT2G22250	AAT	-1.287501933	0.003821467	-1.195337506	0.081082904	-0.354675608	0.291446643	-0.162866619	0.944369177	0.144529815	0.751599754	0.219075965	0.225453838
AT5G45170	AT5G45170	-1.289690877	0.000116651	-0.470993939	0.110159546	-0.782320443	0.000236736	0.226716369	0.784469624	0.230738585	0.603112997	0.159215769	0.462413249
AT1G76450	AT1G76450	-1.292867992	4.30025E-05	-0.531067684	0.000908137	-0.611343835	0.000194158	0.85149246	0.34017207	0.152830707	0.723913117	0.70432338	1.2705E-07
AT3G02570	MEE31	-1.294143547	7.49966E-06	-0.561873145	0.013601508	-0.803692229	9.9246E-06	-0.390825401	0.672844426	-0.290290487	0.398166246	0.096704853	0.685281737
AT1G08520	ALB1	-1.297338154	2.13291E-09	-0.663635626	1.43424E-05	-0.742179198	7.32433E-09	0.355684044	0.66524984	0.058844271	0.872795006	0.669546568	9.46506E-09
AT4G00400	GPAT8	-1.299621333	6.25599E-07	-0.797605725	6.02832E-06	-0.535445484	0.006507848	-0.329209897	0.680248595	-0.651451236	0.000929749	0.408185879	0.002140888
AT5G60800	AT5G60800	-1.299844844	0.048413683	-0.98963629	0.125174328	-0.946309278	0.059108956	-0.720535438	0.765335087	0.539699937	0.473771238	0.547013328	0.232254665
AT1G09490	AT1G09490	-1.303085449	2.2543E-05	-0.317957536	0.327924555	0.038535657	0.898947545	0.194110321	0.76277889	0.090581452	0.87530671	0.8876442	7.72329E-09
AT1G17220	FUG1	-1.304847822	2.17273E-05	-0.844751882	9.4704E-07	-0.925310146	1.72393E-09	0.366311735	0.739606027	-0.02178723	0.963419708	-0.102415809	0.505465058
AT1G32160	AT1G32160	-1.306667737	0.000123541	-0.467364887	0.026678037	-0.795383136	8.97927E-05	0.447537305	0.676109289	0.253756647	0.565049692	0.269287327	0.133767446
AT5G15760	PSRP3/2	-1.307043735	0.020520794	-0.601438613	0.349059392	-0.419768318	0.397523495	-0.832201419	0.632278424	0.119998853	0.934609223	0.422415234	0.278057136
AT5G27450	MK	-1.308155538	0.011058815	-0.853886524	0.073121983	-0.352096564	0.129003423	-0.187093911	0.90808103	0.187369308	0.672741445	0.557722476	2.4139E-05
AT3G19020	AT3G19020	-1.313244163	0.017656013	-0.939143604	0.241642622	-0.670506538	0.234770367	0.086798682	0.982034793	0.308207893	0.750566091	-0.041252808	0.914949036
AT3G15840	PIFI	-1.314956956	6.65824E-05	-0.425378578	0.036275466	-0.576840942	0.001965341	1.051290924	0.606948021	-0.175051675	0.63279222	-0.509733045	0.004818474
AT1G59840	CCB4	-1.315602504	0.010785545	-0.609657308	0.330192657	-0.657803584	0.002933589	1.037285955	0.359381588	0.015950346	0.990379828	0.80966271	3.2301E-05
AT2G23690	AT2G23690	-1.31845362	0.039667299	-1.905605965	0.054353742	-0.323573765	0.624168347	0.208659696	0.926826962	0.082595944	0.963495178	0.655190095	0.11051385
AT3G21420	LBO1	-1.324609174	0.040668489	-1.50566142	0.057480145	0.153209206	0.855731155	0.498878377	0.895706838	0.793667165	0.456284977	-0.339441833	0.462503442
AT1G50250	FTSH1	-1.326546269	1.10551E-05	-0.732480058	0.000481661	-0.644610847	0.000328802	1.045262583	0.541116454	0.059806503	0.915825933	0.049575512	0.830182946
AT5G08740	NDC1	-1.327614013	3.31792E-07	-0.221030001	0.569121336	-0.843878079	2.18568E-07	0.276278992	0.726635812	0.014694414	0.982995784	0.441277031	0.021377883
AT5G36930	AT5G36930	-1.327638456	0.039576774	-0.815524535	0.208473679			0.740051701	0.441510658	0.609205094	0.373165367		
AT1G61500	AT1G61500	-1.332088683	0.017364286	-0.700901799	0.140338386	-0.19271564	0.609139032	-0.009965485	0.996799452	0.019615837	0.989211341	0.324059522	0.156871475
AT1G75010	ARC3	-1.336015683	0.000152849	-0.570058475	0.07226292	-0.52821863	0.035020514	0.186087837	0.858622982	0.51232571	0.20731129	0.325576749	0.073893978
AT1G14700	PAP3	-1.336306704	0.000196205	-0.207631527	0.652614125	-0.469045526	0.092446839	-0.137101594	0.95625417	-0.045602858	0.940778985	0.1147892	0.637532233
AT3G25660	AT3G25660	-1.337232158	1.41448E-09	-0.891305235	0.000532505	-0.618530769	8.55929E-05	0.391721992	0.552538268	0.31812066	0.187294793	0.598707117	1.68388E-07
AT2G39850	AT2G39850	-1.338414255	0.026740231	-0.288416534	0.806182502	-1.143864445	0.053368337	0.266911709	0.917312631	-0.740588555	0.399136391	0.682036166	0.002487692
AT5G40850	UPM1	-1.341656549	0.049700308	-0.572779786	0.282052143	-0.612409976	0.000175146	-0.13087728	0.954608771	-0.12976626	0.709061816	0.097365396	0.613956434
AT4G05090	AT4G05090	-1.345265736	0.000793955	-1.285878952	0.085367903	-0.676776013	0.015580713	-0.524492862	0.579644733	0.129657893	0.864675247	0.101376818	0.743309994
AT5G66210	CPK28	-1.345710672	0.000267707	-0.904895256	6.99647E-05	-0.643541209	0.012448207	-0.145488976	0.917312631	-0.374477874	0.142775997	0.877948136	3.59712E-16
AT2G17800	ARAC1	-1.354485013	0.001740707	-0.711776425	0.0479184	-0.883778977	0.000328569	-0.162859517	0.924835736	0.205787564	0.55798645	0.203010296	0.281672276
AT5G23120	HCF136	-1.359821689	7.65368E-06	-0.736862989	4.34593E-07	-0.549307155	2.55515E-08	0.976264641	0.589474071	0.394473027	0.074733867	0.15040581	0.320850282
AT3G21200	PGR7	-1.361497057	6.64096E-06	-0.563497624	0.032576947	-0.996164043	1.27645E-05	-0.01253164	0.993737713	0.041541154	0.945132997	0.644698879	1.76966E-05
AT2G39250	SNZ	-1.361555654	0.011179658	0.125912574	0.838157496	-0.439847386	0.405910666	0.737996547	0.800334079	-0.29928846	0.824058587	-0.825199233	0.077986884
AT4G14210	PDS3	-1.363051006	4.20473E-06	-0.597606087	0.001518593	-0.79069897	5.22422E-07	0.268128696	0.797780778	-0.060277304	0.900659794	0.258088998	0.108823843
AT5G09870	CESA5	-1.365482847	0.000722721	-0.859450155	0.02279729	-0.944305843	0.001246687	-0.99365454	0.311063931	-0.479307496	0.024128731	-0.108249059	0.553529664
AT2G29670	AT2G29670	-1.367044368	2.77113E-05	-0.672478345	0.025885333	0.023234255	0.931037596	1.137172663	0.561207188	0.043566621	0.957156296	-0.435642547	0.005527178
AT1G18870	ICS2	-1.368586211	0.015989343	-0.582650487	0.208116055	-0.990681233	0.133123355	0.694634926	0.790038336	-0.801629334	0.273198176	0.46590261	0.344490818
AT2G15620	NIR1	-1.370631454	0.004227972	-0.241696889	0.626271806	-0.571255824	0.006334119	-0.500299682	0.649408835	-0.4512929	0.046955932	-0.386533631	0.001670841
AT5G54920	AT5G54920	-1.371197115	0.007047986	-0.744304388	0.180046766	-0.635638027	0.078269229	-0.306652245	0.894017469	-0.042725967	0.966553832	0.259921266	0.306134475
AT5G02670	AT5G02670	-1.371432525	0.000585944	-0.664731965	0.175376545	-0.396774058	0.426712153	0.368466796	0.720012409	0.412095404	0.484700858	0.909531133	1.86101E-05
AT5G19460	NUDT20	-1.373166766	5.76856E-05	-0.764884651	0.038564075	-0.504879049	0.104131402	-0.011785229	0.994162268	0.523282587	0.180670778	0.46528457	0.023454705
AT4G31550	WRKY11	-1.375777793	0.000709551	-0.815691451	0.000532505	-0.838567108	0.019642751	0.055636316	0.977840464	-0.565732637	0.086684216	0.21517253	0.230682773
AT3G18390	EMB1865	-1.377338143	2.3312E-11	-0.978628829	5.53693E-07	-0.798927218	1.48752E-06	0.330757852	0.637815859	0.030966567	0.947746874	0.276446923	0.052477563
AT1G79500	AtkdsA1	-1.37923244	2.2922E-05	-0.842986613	0.034145547	-0.56930734	0.053475968	0.02854342	0.990509604	-0.023372375	0.975427751	0.918275897	6.36304E-10
AT5G10750	AT5G10750	-1.380141297	0.000154341	-0.451673722	0.212884557	-0.320382287	0.374120612	-0.049631203	0.981573179	-0.509875884	0.166216897	0.13561471	0.645082103
AT5G60600	HDS	-1.383723978	9.31055E-05	-0.653532569	3.77269E-06	-0.521775225	3.38493E-07	0.785533241	0.522528829	0.027558214	0.947801595	0.156571679	0.329740823
AT5G63040	AT5G63040	-1.384491891	0.012903225	-0.934322262	0.033271544	-0.720115502	0.01420909	0.456690642	0.629389168	-0.311632852	0.660224389	0.137727024	0.717805353
AT4G39350	CESA2	-1.384750726	4.37166E-07	-0.996203653	8.34158E-05	-0.428074162	0.00537116	-0.300661208	0.575257546	-0.08359082	0.815551772	0.115453062	0.500237347
AT1G20810	AT1G20810	-1.384950653	3.05454E-06	-0.469211068	0.196057139	-0.794674241	0.000112487	0.598145786	0.591266866	0.29921603	0.445683075	0.470155905	0.012694451
AT3G59870	AT3G59870	-1.390967429	0.000288731	-0.867493296	0.031304765	-0.874827483	0.00379495	0.190140841	0.921425607	0.031089382	0.976570624	0.638899863	0.026204679
AT5G08050	AT5G08050	-1.39393813	0.000219147	-0.812176581	0.000138868	-0.791533445	4.41922E-08	0.564019059	0.681118337	0.32651287	0.486024672	0.488179748	0.024740445
AT5G57590	BIO1	-1.397173662	0.011897303	-0.755892296	0.143468707	-0.144895204	0.704054918	0.114823728	0.904181528	-0.18539969	0.805159659	0.505547248	0.010068291
AT1G50732	AT1G50732	-1.398219437	0.018593498	-0.917134011	0.062735235	-0.729268071	0.006896857	2.220606363	0.242136666	1.359105722	0.206582188	0.559476589	0.504020571
AT1G31330	PSAF	-1.398349809	0.000398473	-0.976894393	5.46329E-10	-0.785777527	2.43534E-17	2.533544017	0.058058012	0.540198386	0.291056347	0.872688711	7.44331E-10
AT1G70550	AT1G70550	-1.398648407	0.017199659	-0.996580751	0.024079162	-0.434505347	0.37974386	-0.636431299	0.619569853	0.02472655	0.977185557	0.06387332	0.815256026
AT5G58140	PHOT2	-1.408217624	0.000302366	-0.441594137	0.006518723	-0.71628391	2.38456E-05	1.513001219	0.422303722	0.216916448	0.577315828	0.558901151	0.000975167
AT2G48120	PAC	-1.412218204	0.000126263	-0.410749087	0.533127389	-0.800462419	0.062118366	0.333053004	0.753811706	-0.187840279	0.801700319	0.551297776	0.002795509
AT3G54610	HAG1	-1.412985126	0.001199251	-0.689746852	0.307077564	0.274618271	0.396422687	-0.266221038	0.846215946	0.132657903	0.831264881	0.209944589	0.408729429
AT4G04350	EMB2369	-1.412992527	2.80541E-07	-0.794800679	0.002682945	-0.581801959	0.010755987	0.059110662	0.968021479	0.398438539	0.240758122	0.33444226	0.04992625
AT2G29650	PHT4;1	-1.415242642	0.000432045	-0.635324348	0.005110088	-0.865847103	0.000137847	1.049465783	0.480814999	0.495093633	0.128818223	-0.007660217	0.982806977
AT5G12930	AT5G12930	-1.417044128	0.013755335	-0.022125772	0.976926062	-0.34220172	0.547854296	-0.202659166	0.961565092	-0.554718578	0.548231503	0.001394696	0.996747944
AT1G26520	AT1G26520	-1.419235506	0.019023975	0.259625522	0.732303678	-0.70546325	0.166087469	-0.125499557	0.961565092	-0.10572748	0.915081785	-0.080389774	0.81670246
AT5G09820	AT5G09820	-1.419354663	0.023649248	-0.958132114	0.105718714	-0.767899807	0.004625788	0.015381286	0.996799452	0.554789815	0.178708196	0.614009406	0.015785064
AT5G14100	ABCI11	-1.424376454	0.020846393	-1.159165129	0.103342525	-0.077797269	0.86974482	-0.44821197	0.748333812	-0.140611318	0.878562017	0.403167894	0.159575853
AT2G20890	PSB29	-1.425263327	3.98854E-13	-0.778232558	4.45695E-05	-0.978502031	9.20725E-21	0.426573977	0.663046749	0.152928563	0.672727761	0.662166166	2.88837E-05
AT1G17850	AT1G17850	-1.43312116	0.000421414	-1.11045017	0.154152904	-0.245114485	0.513033572	0.932201243	0.558178127	0.411969505	0.476594025	0.753464771	4.28298E-06
AT1G49630	PREP2	-1.433411519	0.001736787	-0.798578499	0.078710549	0.392344633	0.391582814	0.246789994	0.843525143	0.010417762	0.988694176	0.407161393	0.03562668
AT4G33500	AT4G33500	-1.435200963	8.11195E-05	-0.399325312	0.110945671	-0.637871646	0.009111993	0.573300699	0.408213729	0.080043549	0.893802094	0.711188538	4.37904E-05
AT2G37240	AT2G37240	-1.436105616	0.006009276	-0.046642765	0.947868198	-0.831356627	0.060197613	0.717408127	0.767881165	0.157600687	0.919937283	0.237236459	0.573915735
AT1G73530	AT1G73530	-1.437066124	0.000129017	-0.348094281	0.413909957	-0.7656743	0.000805344	0.877631099	0.334544782	0.605619071	0.167549093	0.685321126	0.000292911
AT1G11860	AT1G11860	-1.439351438	8.44933E-10	-0.847771871	3.61228E-06	-0.782093381	2.47212E-12	0.628984803	0.588988003	0.229990901	0.176633494	0.239516817	0.0406663

AT2G43945	AT2G43945	-1.441836014	6.60076E-06	-0.849223301	0.000346192	-0.517170055	0.036705744	0.59333634	0.653363031	-0.106648123	0.875837042	0.188186589	0.429150414
AT4G22550	LPPbeta	-1.442407616	0.04309198	-0.186532617	0.874341306	-0.201237184	0.73067473	-0.610812058	0.805996468	-0.835990375	0.074230713	0.044775628	0.920019822
AT1G50020	AT1G50020	-1.445835251	2.88741E-05	-0.540571314	0.13309299	-0.611569568	0.017464801	0.090061149	0.948761383	-0.160686871	0.867849993	-0.285508195	0.184692189
AT5G04190	PKS4	-1.451788628	0.006714602	-0.992658335	0.00050281	-0.550914383	0.017996329	0.316816985	0.798532157	-0.078144765	0.883796441	0.729345312	0.000334481
AT4G13100	AT4G13100	-1.454681216	0.00354867	-0.597529851	0.324654614	-0.50289566	0.551753983	-0.341556539	0.895706838	-0.015919684	0.986860362	-0.235899636	0.458254215
AT1G08540	SIG2	-1.463974649	2.56547E-09	-0.796692457	0.000172139	-0.655836191	7.47509E-06	0.045193614	0.981525175	0.070475639	0.900091313	0.281070947	0.142688772
AT1G68780	AT1G68780	-1.46665791	0.016346527	-0.492885207	0.371163991	-0.493785509	0.232837315	0.556625492	0.637614678	0.130770462	0.886505805	0.152632442	0.635194607
AT5G40500	AT5G40500	-1.467392346	0.045729826	-0.285647644	0.575614699	-0.890371467	0.000106604	1.804668258	0.579644733	0.113622897	0.963419708	0.685710717	0.28032243
AT2G28470	BGAL8	-1.468872931	5.02567E-07	-0.950673366	0.000481585	-0.065215012	0.829056733	0.383785648	0.741369316	-0.537010087	0.022387951	0.05371404	0.823421499
AT1G56500	AT1G56500	-1.46941215	4.27538E-06	-0.887457723	5.4349E-05	-0.239923229	0.232110567	0.733785477	0.627960887	0.021618472	0.975386078	0.216901489	0.341487484
AT1G78830	AT1G78830	-1.475817415	6.21873E-05	-0.574128261	9.80897E-05	-0.809851618	0.000468626	-0.086756891	0.967195835	-0.714466581	0.000422369	0.276062129	0.073626739
AT3G14330	AT3G14330	-1.476117449	0.000120738	-0.874610775	0.071629054	-0.782911943	0.003158752	-0.029193987	0.992379592	-0.155000154	0.828933168	0.395938218	0.030094485
AT5G22860	AT5G22860	-1.487094044	0.001162756	-0.932391071	0.026681358	-0.511256753	0.221295307	-0.169371061	0.925800135	-0.401246436	0.27946574	0.361962868	0.377993158
AT1G05620	URH2	-1.487418983	0.045326624	-1.148258919	0.072436606	-0.570117574	0.099487912	-0.863557194	0.697744369	-0.075124495	0.882725602	0.357787628	0.027115511
AT3G29400	EXO70E1	-1.491451061	0.012234466	-1.539203019	0.076836567	-0.870619948	0.018347604	-0.923037341	0.023094271	-0.128504888	0.869183581	-0.289708094	0.311277432
AT1G32440	PKp3	-1.492562953	0.00012503	-0.795698427	0.007701432	-0.222320189	0.36319714	0.210424845	0.90560947	-0.23433028	0.570965538	-0.219975869	0.301801895
AT1G56510	WRR4	-1.49479594	0.011448637	-0.022092479	0.970866334	-0.865685782	0.713429359	2.149104695	0.326457456	0.373880645	0.803422956	1.513796403	0.12591332
AT5G02940	AT5G02940	-1.494993998	4.33902E-06	-0.59836883	0.017302753	-0.342617318	0.069757422	0.309116818	0.810393493	0.49523522	0.349208222	0.550639589	0.001163609
AT4G17740	AT4G17740	-1.495620484	4.88518E-05	-0.940887481	0.000794437	-0.962931419	1.88473E-06	0.176018169	0.916448617	0.117356211	0.843366485	0.53281461	0.002847814
AT3G19180	PARC6	-1.504489375	0.010777901	-0.995716798	0.000864259	-0.577255393	0.062069471	0.011821176	0.994744545	0.261562658	0.516852689	0.658553382	0.000119214
AT2G32400	GLR5	-1.508088124	0.016306936	-1.564687581	0.066435138	-1.241724256	0.115384342	-0.153657223	0.958708047	-0.405063525	0.530853324	0.001833181	0.99687508
AT1G74040	IMS1	-1.50858495	4.2055E-06	-0.519226596	0.17174157	-0.542894828	0.016195725	-0.125562941	0.930511033	0.064782854	0.913153064	0.146616754	0.53494968
AT2G19940	AT2G19940	-1.510498883	3.11537E-09	-0.892005977	0.000866678	-0.832276478	7.38494E-05	0.101194531	0.921986527	-0.179784269	0.592311926	0.609776832	9.53294E-05
AT1G13100	CYP71B29	-1.511661184	0.002654524	-0.879576828	0.176475212	-1.102062182	0.11713594	0.103981458	0.951485287	-0.737585138	0.218018511	-0.703510417	0.005071796
AT3G27925	DEG1	-1.513250795	0.000100226	-0.91278613	1.48567E-05	-0.573842945	9.36704E-05	0.636472827	0.672844426	0.055333145	0.934596763	0.222364388	0.426506536
AT4G16370	OPT3	-1.516120807	8.69656E-06	-0.795577075	3.85605E-05	-0.805214697	0.000278365	-0.451201086	0.565728752	-0.543211162	0.002981125	-0.429502484	0.002420413
AT5G61300	AT5G61300	-1.518001011	0.010053476	-1.063095006	0.112660691	-0.858287861	0.116900341	-0.405705017	0.86582927	-0.028478152	0.983264238	0.646140192	0.019036634
AT5G09790	ATXR5	-1.519328622	0.003708807	-0.97552804	0.057514617	-0.694653846	0.199448033	0.083408063	0.962474985	0.332079259	0.610778533	0.612535325	0.002109776
AT1G77260	AT1G77260	-1.52012231	3.66512E-06	-0.29730001	0.485500818	-0.628078546	0.044093136	-0.724930631	0.221083366	-0.516840198	0.122550672	-0.430297964	0.024445831
AT1G10360	GSTU18	-1.529853771	0.000439037	-0.547373685	0.092101896	-0.198800622	0.444958454	0.683826067	0.774188277	-0.248044478	0.834690411	-0.056641286	0.861906214
AT3G51450	AT3G51450	-1.531144596	0.010660656	-1.178000201	0.14299464	-1.157156217	0.081681986	0.684779157	0.795944563	0.230574735	0.899954945	0.552167387	0.053868358
AT5G19190	AT5G19190	-1.532090139	0.001703116	-0.525232411	0.009270121	0.117341592	0.762256368	0.532291229	0.680482607	0.024348644	0.964483468	-0.249610635	0.207942572
AT1G09580	AT1G09580	-1.541818205	0.018952994	-0.784732353	0.124530759	-0.461240507	0.249820298	-0.158370502	0.93556951	-0.044391353	0.963419708	0.459040722	0.01614417
AT5G49010	SLD5	-1.543158257	0.022151188	-1.264292282	0.129085418	-0.66343383	0.300263901	-0.382640719	0.892607537	0.100465317	0.940108495	0.44296377	0.108545116
AT4G18570	AT4G18570	-1.546794443	1.16793E-05	-0.752250546	0.000718869	-0.449453039	0.006394819	-0.015102765	0.990545661	-0.163252267	0.704725736	0.353973008	0.085561052
AT1G68540	TKPR2	-1.548972971	0.010084779	-0.093331117	0.922637484	-0.899736153	0.07008741	0.263497967	0.917312631	-0.326653719	0.652699183	0.214153473	0.597282028
AT1G71810	AT1G71810	-1.551387201	0.003921537	-0.911371664	0.082323726	-0.887638813	0.0003636	0.426567765	0.644839236	0.237173174	0.727051106	0.060206352	0.863959231
AT1G80030	AT1G80030	-1.560415605	9.7123E-09	-0.690234381	0.003133369	-0.997501929	1.82399E-06	0.389786719	0.657881954	0.135801692	0.790926204	0.11968052	0.615621823
AT4G25720	QC	-1.561971845	0.008577597	-0.644251029	0.220053884	-0.636211934	0.101908618	-0.393679051	0.751885934	-0.145626279	0.829112414	0.189167587	0.560073826
AT3G43540	AT3G43540	-1.565033052	5.70064E-07	-0.854963887	0.000364084	-0.396402595	0.057265613	0.828545329	0.195928186	0.203134349	0.613407287	0.838028601	4.20876E-09
AT1G16260	AT1G16260	-1.568622921	4.82606E-05	-0.458505785	0.141558938	-0.891502433	0.591183547	0.597319975	0.664635521	0.3380324	0.495989166	1.997188017	0.297007507
AT2G04842	EMB2761	-1.573127536	9.25359E-06	-0.916418246	0.003712017	-0.708404841	2.94934E-07	0.817727247	0.435291031	0.045364164	0.93380026	0.26297061	0.097187586
AT5G06870	PGIP2	-1.575536619	0.000107689	-0.30167478	0.388648691	-0.485236369	0.013468616	0.640725782	0.665778009	-0.044682113	0.958669515	0.548924102	0.008794181
AT4G00050	UNE10	-1.578072674	0.003547799	-0.705805995	0.413600468	-1.364446789	0.108212094	0.323529233	0.938741823	-0.117500461	0.957133906	0.542448272	0.30157313
AT1G35340	AT1G35340	-1.580126299	3.93777E-09	-0.793003764	3.53116E-05	-0.636677537	1.97387E-05	0.447585433	0.743023983	0.015802751	0.985862786	0.854058111	1.55246E-06
AT5G10320	AT5G10320	-1.581485896	0.004280141	-0.725912326	0.265656803	-0.26376555	0.557068091	-0.337218536	0.820385931	0.805901254	0.084083261	0.427279841	0.047310751
AT4G38420	sks9	-1.592392813	1.2878E-05	-0.891435997	0.004108922	-1.943884873	0.102270292	-0.228972349	0.853294054	0.294337044	0.581594843	0.759683197	0.014517591
AT1G74710	EDS16	-1.593506923	0.004467477	-1.358139984	0.171736076	-0.231849974	0.849998901	0.140745297	0.94363635	0.30811966	0.773603021	0.520844032	0.088629275
AT1G70610	ABCB26	-1.605617142	0.000217981	0.140365778	0.792907242	0.33737943	0.102913936	0.185571554	0.861319622	0.505747716	0.346839563	0.136728575	0.538798272
AT5G50915	AT5G50915	-1.608637634	0.032120544	0.722250194	0.353624892	0.003342381	0.997099041	-0.606232291	0.653363031	-1.092725368	0.05120669	-0.553736748	0.080326185
AT3G47560	AT3G47560	-1.612911852	4.25114E-05	-0.925111679	0.0063037	-0.782174775	0.010753007	0.687602986	0.608298816	0.52813918	0.172183841	0.665294602	0.000566021
AT1G67740	PSBY	-1.622853556	8.10866E-05	-0.774213789	4.16796E-05	-0.910438639	6.01483E-11	1.775371598	0.222445022	0.23470791	0.782369045	0.714503084	1.7855E-05
AT1G27385	AT1G27385	-1.625031522	0.000135104	-0.638185775	0.028098802	-0.82648673	0.000755786	0.187159815	0.876015705	0.19688083	0.762086306	0.17729343	0.567632283
AT2G26260	3BETAHSD/D2	-1.627609536	0.028128825	-0.687420626	0.346053002	-0.183732112	0.654744222	-0.44991206	0.67527834	0.381873485	0.425058911	0.316381907	0.127993262
AT4G10060	AT4G10060	-1.630057437	3.72347E-07	-0.802946825	0.000994833	-0.431079702	0.041174978	0.365309628	0.696374342	-0.050617047	0.935515226	0.881715033	6.39172E-10
AT4G23940	FtsHi1	-1.631036053	0.002010874	-0.894022246	0.022851541	-0.675551443	0.008400988	-0.265015167	0.885840009	0.029641419	0.966304751	0.525936128	0.004632515
AT1G71440	PFI	-1.636207978	3.98384E-07	-0.675410436	0.062641706	-0.139817218	0.601729741	0.126661924	0.93556951	-0.131355997	0.805955561	0.333343797	0.060536362
AT1G44446	CH1	-1.640796743	0.000494769	-0.620219547	0.015270147	-0.830877303	5.34628E-06	1.421853228	0.267160791	0.46307212	0.267594627	0.62451289	1.74275E-05
AT2G21330	FBA1	-1.643508335	6.81025E-06	-0.510195057	0.000538866	0.076008209	0.627341468	2.385883777	0.077570991	0.556944927	0.040299214	0.94718183	6.41696E-09
AT5G05200	AT5G05200	-1.644334848	2.01817E-08	-0.948736266	5.33738E-10	-0.664224247	0.000136419	0.273004002	0.837258505	-0.187589709	0.61326813	-0.133682248	0.542360041
AT1G66520	pde194	-1.646077233	0.013855838	-1.327022842	0.10511396	-1.037274793	0.070016739	0.204184131	0.947082547	0.042642869	0.978655483	0.635887868	0.028149571
AT3G47450	NOA1	-1.662149831	2.54425E-09	-0.368834141	0.31269532	-0.981185737	1.87296E-06	0.152919397	0.932578056	0.02046016	0.985440963	0.452725689	0.014716922
AT3G25770	AOC2	-1.667562612	1.37269E-05	-1.035471288	0.052173667	-0.72108389	0.301199	2.022576686	0.616300683	0.456862756	0.927107216	-4.043660022	NA
AT2G34840	AT2G34840	-1.67254338	0.001892197	-0.301646326	0.798509428	-0.703086128	0.15456243	-0.590627648	0.729908776	0.212463974	0.782363328	0.547563407	0.00892318
AT2G33255	AT2G33255	-1.676385519	0.00157606	-0.317874756	0.460011796	-0.913440064	0.02079379	0.201327652	0.922582072	0.427027015	0.474364938	0.587773529	0.003101879
AT3G09710	IQD1	-1.685901494	0.001289147	-1.063424049	0.118457873	-0.663090947	0.179042583	-0.630411147	0.637614678	-0.523979724	0.3051444	-0.419243592	0.057855538
AT5G59020	AT5G59020	-1.694758402	0.009662876	-0.753130511	0.35952125	-0.158627735	0.823069766	-0.571202367	0.807059483	0.036004891	0.980764662	-0.322815714	0.30548127
AT5G04140	GLU1	-1.695961791	3.45016E-12	-0.912794482	7.65496E-14	-0.910206647	4.18839E-10	0.845625881	0.50075057	0.312861716	0.129558821	0.399363991	0.022001069
AT1G72450	JAZ6	-1.697810811	1.47788E-05	-0.724328737	0.001248329	-0.895627104	5.9047E-05	0.442835024	0.632278424	-0.176335283	0.776078497	0.488207914	0.002109776
AT1G02720	GATL5	-1.702807557	0.008249771	-1.143366162	0.158122566	-0.702570423	0.129323029	-0.765203014	0.731041294	-0.147800829	0.865593881	0.372528066	0.202411149
AT1G74640	AT1G74640	-1.70972432	2.1771E-05	-0.949920937	0.001666297	-0.817729938	0.005647126	0.594183242	0.524004533	0.134103919	0.832272733	-0.009371894	0.980400268
AT1G80850	AT1G80850	-1.718808031	0.009774109	-0.740411795	0.346398333	-0.858412083	0.066518436	0.758138116	0.662219342	0.583102566	0.632768672	0.963124175	0.00669931
AT5G64580	EMB3144	-1.738324553	0.000121544	-0.949063819	0.000577063	-0.915874947	1.5372E-05	-0.027708074	0.991559366	-0.080123612	0.900659794	0.330053201	0.064533826
AT2G40960	AT2G40960	-1.740601099	0.003980013	-0.896804138	0.081590462	-0.426329023	0.057664817	0.339206538	0.84196754	0.472927085	0.478838042	0.973131025	1.36163E-09
AT4G21890	AT4G21890	-1.754216999	0.021674974	-0.814467449	0.399784494	-0.133208265	0.888739221	-0.589483602	0.652047062	0.297661401	0.663168669	-0.010837433	0.984723501
AT4G15510	PPD1	-1.757317754	1.23491E-05	-0.85363317	0.025902453	-0.757095219	0.023768311	0.861707222	0.598901706	0.041384897	0.971701608	0.834548134	0.000514497
AT1G19650	AT1G19650	-1.774287171	0.008135408	-0.876381352	0.036270445	-0.426971399	0.063244613	-0.162450773	0.936758504	-0.101368948	0.910605748	0.339266781	0.248375692
AT1G36180	ACC2	-1.786109355	0.036597145	-0.248310873	0.837051039	-0.494237018	0.412811167	-0.422616002	0.770575751	-0.302830212	0.694731487	-0.149636223	0.647517608
AT1G19450	AT1G19450	-1.795579151	0.001467384	-1.670766357	0.535278948	-0.547713684	0.070265974	-0.793606519	0.676109289	-0.496521509	0.198445026	-0.351676403	0.107012249
AT3G20860	NEK5	-1.796814252	0.001530709	-1.419310576	0.387279741	-0.785903215	0.273352518	-0.167816436	0.942584765	-0.187392335	0.799682552	-0.090993767	0.823002303
AT1G33770	AT1G33770	-1.806689282	0.003843176	-1.884808001	0.237464965	0.104887065	0.932140413	1.05801405	0.70850021	0.369467098	0.857963506	1.118069814	0.380317411
AT3G50820	PSBO2	-1.80961979	2.88166E-05	-0.670310477	0.000225357	-0.893013883	7.4839E-06	1.817861187	0.36114754	-0.126217664	0.770526843	-0.459608545	0.028875199
AT2G17695	AT2G17695	-1.811996685	1.92761E-05	-0.8078258	0.152429821	-0.36421115	0.227228064	0.286591744	0.861588972	-0.179319386	0.866906715	-0.333497555	0.293302884
AT1G11790	ADT1	-1.814417054	0.003838793	-0.460569391	0.433319799	-0.900610116	0.003617861	-0.324914844	0.858622982	0.094935515	0.900194273	0.72470458	0.000928632
AT5G20850	RAD51	-1.818517214	0.039933011	1.593548254	0.062484503	-1.060372085	0.247533747	-1.502704618	0.704004387	0.75981901	0.202005359	0.283404585	0.350291472
AT3G55400	OVA1	-1.820256695	2.12511E-06	-0.832399799	0.000282903	-0.918252349	1.86628E-06	0.095278367	0.95660961	0.170364678	0.624737627	0.403001718	0.020101248
AT5G58120	AT5G58120	-1.824210569	0.002770495	-0.932581564	0.03210561	-0.282802627	0.418381875	1.245815315	0.567190016	-0.763404975	0.5094879	0.438725887	0.02013077
AT1G70410	BCA4	-1.827006073	4.31053E-13	-0.80401638	5.28495E-05	-0.437487728	0.002441562	-0.050915688	0.970946609	-0.317358883	0.27946574	0.561768357	1.54203E-06
AT4G32770	VTE1	-1.83701792	0.000170386	-0.925761217	0.019737304	-0.414432986	0.38335487	1.241375783	0.66924865	-0.78599716	0.543168404	0.386671509	0.451772006
AT5G07660	SMC6A	-1.842965073	0.041600408	-1.278127522	0.162079904	-0.425504204	0.716732692	1.025615302	0.631596962	0.243178244	0.850121117	0.43476035	0.092079908
AT2G15080	RLP19	-1.860885323	1.91048E-05	-0.865837316	0.008857079	-1.013867332	0.728610613	2.971544246	0.553691236	-1.236183885	NA	-0.45683215	0.853194019
AT1G34340	AT1G34340	-1.876426309	1.90266E-08	-0.708350131	0.041811378	-0.44123961	0.353491414	0.630029228	0.672844426	-0.226500027	0.698022181	0.533092769	0.00928098
AT2G34930	AT2G34930	-1.891905577	3.29532E-06	-0.748816196	0.023031261	-0.352254457	0.19848717	0.097325397	0.966082349	-0.376062243	0.508853027	0.462534259	0.081807333
AT3G16650	AT3G16650	-1.954055182	0.003646847	-1.145017988	0.330466739	-0.49803706	0.525404807	-0.619836592	0.75047646	0.460623169	0.576608643	0.074915925	0.799688042

AT4G25100	FSD1	-1.957471691	5.57262E-11	-0.109243865	0.92912178	0.179190027	0.150450984	0.030893369	0.988471189	0.831863102	0.301120228	0.931114633	8.04875E-05
AT2G46650	CB5-C	-1.989182583	0.043532209	-0.483453564	0.532398619	-1.614937601	0.519834439	0.07988421	0.987266226	-0.00487988	0.998107647	0.719791687	0.025213859
AT1G17920	HDG12	-2.01490095	0.037097372	-0.501035741	0.594646473	-2.533633123	0.221295307	-0.580300313	0.676109289	0.354636412	0.645548269	0.996140871	4.34862E-05
AT4G10150	AT4G10150	-2.015010592	0.003472402	-2.032389209	0.248905037	-2.206031468	0.124055215	-0.594792428	0.697914029	-0.172257986	0.869890097	-0.152858326	0.786979089
AT3G61220	SDR1	-2.01865363	0.024744407	-0.306065381	0.718026944	-0.904584545	0.093249816	-0.099537425	0.985626278	1.147600078	0.707672204	0.105949479	0.888538076
AT5G60490	FLA12	-2.054156082	0.01656862	-1.773593959	0.335529552	-0.636412447	0.721165087	-0.722865376	0.888872615	-0.604829531	0.416307461	0.605148717	0.155017462
AT1G11070	AT1G11070	-2.058747033	0.009088019	-1.470880377	0.384083167	-0.684500075	0.17830697	-0.733032181	0.92031926	-1.527730723	0.802042097	-0.760870662	0.214246898
AT2G40460	AT2G40460	-2.064932826	0.00087386	-1.703509647	0.051258282	-0.987976122	0.121406169	-1.002598793	0.66924865	-0.923348781	0.27229745	-0.691152321	0.022375186
AT5G52900	MAKR6	-2.091688032	4.84971E-10	0.265009908	0.469550897	-0.842115714	0.000131327	-0.721424715	0.248219642	0.433419057	0.314574969	0.009639974	0.979377934
AT1G18710	MYB47	-2.097501803	0.009744723	0.737114867	0.560322983	-1.777216722	0.084148963	-2.170574946	0.658780788	0.319277752	0.939665158	-0.224188717	0.773980771
AT3G61680	AT3G61680	-2.130369929	0.001941122	-2.4429705	0.526142276	-1.271009103	0.129381216	0.026786704	0.992231761	0.107816924	0.941042784	-0.168325377	0.640406028
AT4G16940	AT4G16940	-2.146933747	0.023269677	0.664332371	0.608933822	-0.14161573	0.972965179	-0.475961709	0.820789421	-0.548988432	0.781573394	-0.03163502	0.986892016
AT3G04980	AT3G04980	-2.183711302	0.012140513	-0.876195333	0.268582233	-1.334044117	0.058455909	-0.477787677	0.850582824	-0.228782418	0.821418926	-0.351335555	0.450529821
AT1G77390	CYCA1;2	-2.210111991	0.007638943	-0.357408522	0.794162532	-1.082771674	0.105852458	-0.671885621	0.66924865	-0.50570235	0.716620878	0.705427769	0.19603394
AT4G09350	NdhT	-2.231463901	0.017627532	-0.900887031	0.145511565	-2.382152599	0.297865548	-0.749438656	0.917312631	0.343457149	0.857897799	0.427035804	0.610636545
AT4G24460	CLT2	-2.233961787	0.000262115	-1.124582907	0.058034781	-0.49691438	0.155425517	-1.197156885	0.603376116	0.027864364	0.98143759	0.102984856	0.781032743
AT1G35180	AT1G35180	-2.263285474	0.002186809	-1.320916871	0.104575813	-0.819150282	0.170067228	0.152387629	0.966811844	-0.254398256	0.819806715	-0.049673247	0.937625425
AT2G20980	MCM10	-2.296495101	0.008084101	-2.058385416	0.240891092	-2.16068539	0.09390509	-1.596968779	0.478317143	-0.150025733	0.913903461	-0.084345861	0.797777166
AT1G65486	AT1G65486	-2.31759504	0.017627532	-0.867085172	0.665406574	-1.242373256	0.589460533	-5.792988818	0.629389168	1.374682028	0.651975596	1.12345319	0.513027724
AT2G37030	AT2G37030	-2.359641627	0.016587993	-1.284199506	0.523480262	-1.226497923	0.117945303	-5.349126464	0.647947756	0.253972758	0.957291513	-1.703706529	0.339509893
AT4G32000	AT4G32000	-2.365476403	0.000490875	-0.975816191	0.146650767	-0.791075681	0.168126271	0.029286077	0.990335664	-0.447648661	0.366626004	0.129311601	0.766591434
AT1G45191	BGLU1	-2.410859358	0.043346321	-1.561234634	0.111737475	-0.85950099	0.41755374	0.450077001	0.858622982	0.710821195	0.46570375	0.732890042	0.122844419
AT1G58370	RXF12	-2.420298396	0.015393661	-1.269515386	0.116931434	-2.047034325	0.103928668	-0.294526207	0.966434558	-0.863106578	0.038816205	-0.211661328	0.476138996
AT5G05270	CHIL	-2.446872096	6.52091E-06	-2.03476682	0.054095315	-3.094020239	0.363605308	-0.991207162	0.66808962	-0.183762933	0.925388746	-0.459244211	0.295781365
AT4G23240	CRK16	-2.468221163	0.006702337	-0.693123699	0.5437093	-0.633294835	NA	-2.377245032	0.715810757	0.161470389	0.950790869	-0.229990073	0.887306315
AT3G25880	AT3G25880	-2.592726372	0.0329884	-0.034202974	0.98913201	-2.080127413	0.141850875	-0.417139457	0.962474985	1.862983824	0.085472556	0.641407564	0.523345318
AT1G64400	LACS3	-2.593616176	0.016051851	-0.058035825	0.980651529	-1.342487591	NA	-0.687449107	0.704132415	-0.004590499	0.998107647	-0.164364384	0.725766391
AT1G29730	AT1G29730	-2.730899878	0.005762877	-1.090095828	0.566126049	-0.291643442	0.750635684	1.130419099	0.534532914	1.27835801	0.780741529	-0.604688712	0.299067875
AT3G28270	AT3G28270	-2.757570472	0.003954399	-0.795242188	0.728572248	-0.206508878	0.946974074	-5.612322002	0.637614678	5.469500172	0.507785667	-6.09685695	0.058659194
AT5G24205	AT5G24205	-2.774639209	0.024583809	-2.067208177	0.278128121			0.987214419	0.872161839	1.16444892	0.775228493		
AT3G54600	DJ1F	-2.837400836	0.003399982	-1.200714099	0.188840917	-1.614028054	0.063987961	-0.401098171	0.851719458	0.028611355	0.985986938	0.840760405	2.60112E-06
AT1G09890	AT1G09890	-2.847711329	0.027242742	-1.125581241	0.333822072	-0.71046008	0.519341383	-0.292043454	0.887763386	-0.600000105	0.570854222	0.315482252	0.435079399
AT1G29430	AT1G29430	-2.857171928	0.001404888	-2.890280419	0.168308245	-1.068365967	0.28396006	-0.429071574	0.792048637	0.000174155	0.999968179	-0.684916945	0.138467937
AT5G11590	TINY2	-2.881538021	0.017160931	-0.521266418	0.898319385	-0.92986373	0.613541057	-0.872191918	0.93058272	0.77523036	0.811222926	1.067177053	0.168752532
AT1G62630	AT1G62630	-2.907003312	8.26453E-05	-2.777457495	0.053185651	-2.429077867	0.150515403	1.77435492	0.759090766	-0.057661025	0.994307202	1.594631673	0.254716514
AT1G12370	PHR1	-2.991972917	0.042323913	-0.982862239	0.637672608	-2.112353951	0.161505588	-0.411824808	0.935645553	-0.091910113	0.971280036	0.340654001	0.573202852
AT4G00460	ROPGEF3	-3.078192001	0.015400471	-2.345450529	0.183564123	-1.658583726	0.322513735	-0.554323353	0.847193283	0.759286348	0.50442122	-0.074976173	0.930701086
AT5G44568	AT5G44568	-3.170396631	0.037136582	1.042978056	0.621251681	-0.173709013	0.847566287	-0.82092322	0.879905736	0.776501769	0.430532999	0.567023085	0.070486573
AT5G03390	AT5G03390	-3.324057571	0.020882049	-2.841549674	0.457782627	-1.178613966	0.139337341	-0.511581393	0.93556951	0.061541916	0.972421502	0.997203076	7.49705E-06
AT2G04080	AT2G04080	-3.354421828	0.024909894	-1.520206278	0.590936102	4.278692779	0.051891916	-0.776300206	0.697691387	-0.723797933	0.639793247	0.553072387	0.602217596
AT4G15975	AT4G15975	-3.677314627	1.34402E-05	-0.85216777	0.668408224			-0.59679078	0.93157093	0.120199541	0.96653787	1.177961515	NA
AT5G50930	AT5G50930	-3.871330803	0.030059128	-1.414012139	0.469550897	-0.220814517	0.855785106	0.478676781	0.789727513	0.06056439	0.977308664	0.142698375	0.765456877
AT5G43890	YUC5	-3.944344903	0.000823319	-2.664020326	0.487054444	-2.417750341	0.50523716	0.030192157	0.994162268	0.080183449	0.988021272	2.032786226	0.053581619
AT1G65250	AT1G65250	-3.951707579	0.009445731	-2.351031149	0.228600397	-0.729806308	0.179845704	-0.551950582	0.94585537	0.952166072	0.547527307	-0.606617355	0.11879827
AT5G44420	PDF1.2	-4.42701937	0.02512817	-2.666833386	NA	1.211564633	0.532607563	-0.552609514	0.937566901	4.110862423	0.376459115	-3.127747373	0.082318375
AT1G62981	AT1G62981	-4.454455677	0.027503247	-1.380891502	0.484518204	-0.332582614	0.795012002	-1.853553167	0.661895574	-0.943168924	0.708363406	-0.408171948	0.675481185
AT3G52740	AT3G52740	-4.789554411	0.012893128	0.403185442	0.84634218	0.132790639	0.95029404	7.577476301	0.373396563	5.767261102	0.399136391	-0.924823444	0.714546216
AT2G23050	NPY4	-4.793844166	0.018276892	-4.679433448	NA	-3.766520032	0.296260558	-0.122979451	0.985078099	0.314652768	0.883566526	-0.956161713	0.184351198
AT2G22600	AT2G22600	-4.903532715	0.017125179	-1.025149587	0.788235953	-4.632769432	0.39119055	-1.313401289	0.792048637	0.777137827	0.633717846	0.626772218	0.64187558
AT3G45430	AT3G45430	-4.988682002	4.04808E-05	-1.725683765	0.370141269	3.236995383	0.272002305	-1.190248397	0.786149293	-0.575913469	0.71169254	0.687267042	0.433540583
AT3G06655	None	-5.110173075	0.001275751	-3.191835566	0.120975237								
AT1G76190	AT1G76190	-5.114963129	0.034233955	-5.791870441	0.193992601	-6.024571756	0.057273924			-0.171541668	0.964229576	1.109760204	0.872321093
AT5G13930	TT4	-5.191602294	0.00117712	-1.079115862	0.573821689	-0.509317274	NA	-2.365167335	0.717215462	-0.412948551	0.869250171	-1.006269485	0.534270204
AT2G47880	AT2G47880	-5.226534672	0.027883072	-0.991806904	0.72552979	0.140257403	0.838623271					1.072981214	0.621775432
AT1G08443	None	-5.401220084	0.020920645	1.063895627	0.869962814			-2.700350308	NA	5.689507166	0.419833127		
AT4G01270	AT4G01270	-5.432124677	0.018494353	-0.645258509	0.838276727	-1.100263081	0.559816449	-1.65784829	0.680482607	0.09610376	0.963495178	0.338269308	0.423344988
AT1G16070	TLP8	-5.93069813	0.000963005	-2.264732613	0.163342904	-0.983786651	0.245708062	-0.310283957	0.876503587	0.121118255	0.923631194	0.751502373	0.013906105
AT4G06115	None	-5.955520302	0.000543372	-1.111945564	NA								
AT5G36920	AT5G36920	-5.981582738	0.009847471	-1.836259409	0.391149034	0.026749597	0.99402928	0.263452837	0.982734229	0.829290557	0.863621528	-0.104134536	0.929387479
AT1G24147	AT1G24147	-6.188198383	0.009432053	-3.176438033	NA	0.018456012	0.995027037	-7.009703251	NA	3.535813429	NA	-4.564904366	0.262861919
AT2G21890	CAD3	-6.647909737	0.044985034	-0.011325088	0.99795667	-5.877237009	0.079634913	5.731194486	0.632278424	4.857491874	0.55798645	1.844065027	0.414300689
AT2G26010	PDF1.3	-6.677051297	0.044077669	-1.303008771	0.799736406	0.451721985	0.913444807			2.828456204	0.779388526	-2.012685178	0.761927143
AT5G38120	4CL8	-6.915274228	0.036435246	-3.875540153	NA								
AT1G56650	PAP1	-7.045993493	0.023975709	-4.157764304	NA	-3.448038984	0.148539459			-2.710124576	NA	-1.957094039	0.764682005
AT5G36700	PGLP1	-7.04650084	0.035468913	-5.871580537	0.267800994	-0.828882226	0.741945775	3.513934685	0.748808009	-0.871794116	NA	0.962338286	0.713446827
AT5G55780	AT5G55780	-7.114609533	0.023913647	-4.360777742	NA	-2.43257692	0.460200837	-5.513353854	0.640704059	-4.427973516	NA	-1.269679691	0.070517482
AT2G38600	AT2G38600	-7.200139836	0.022750122	2.705513921	0.535861253	-4.899870335	0.361140347	-6.56106766	0.593379395	-1.871411517	0.825142465	0.912259617	0.451534921
AT3G01700	AGP11	-7.395746878	0.010909754	-5.82169028	0.271700729	-0.737577919	0.843341603	-4.709284728	0.680482607	2.089910845	0.703811392	0.477379702	0.768617623
AT1G20080	SYTB	-7.479137968	0.005390077							0.806414823	0.922334223	2.304809417	0.127694018
AT1G44318	hemb2	-7.482910002	0.041879647	-3.591666303	NA	4.734883457	0.379465519			-6.152193501	0.269079164	-1.238603616	0.858149424
AT3G52600	CWINV2	-7.499196329	0.009519124	5.82828386	0.271605621	3.414265332	0.532109885	-6.650766931	0.587695712	5.190337075	0.529833936	-0.9205664	0.871619987
AT5G14565	ath-MIR398c	-7.579346545	0.005735697	0.814675795	0.886220172	-4.650987649	0.389306313			6.344482766	0.229220954	-2.029663157	0.288583997
AT1G14315	AT1G14315	-7.621694459	0.040656286	-5.012675121	0.35211144	3.14576655	0.573217775			-3.707938446	NA		
AT5G55507	AT5G55507	-7.660315144	0.014301629	3.022069515	0.602587578			5.628310675	0.637614678	5.664603858	0.42978986	0.858242271	0.878292965
AT1G20750	AT1G20750	-7.676926023	0.005419176	1.594045705	0.67681765	4.915707362	0.358816271					1.687673863	0.804433334
AT1G66930	AT1G66930	-7.68330974	0.028795349	-3.804812146	0.114054795	-5.819027782	0.165498404	0.137127167	0.971723999	0.059275537	0.984765765	-0.128173102	0.893400872
AT5G07700	MYB76	-7.761444232	0.001709746	-6.741048691	0.111727275	-4.959703219	0.354764451			5.757862021	0.478838042	2.773470756	0.669113881
AT2G37720	TBL15	-7.793838615	0.002792387	-4.374637525	NA	-3.001110281	NA					-5.206295806	0.281492188
AT4G19570	AT4G19570	-7.833670604	0.003954399			-2.732919615	0.116410386	-3.862355172	0.731041294	0.530123256	0.958701896	-1.407529537	0.368835
AT1G55390	AT1G55390	-7.875350464	0.020920645	-5.608256073	0.291724403	-0.135451609	0.976287367	-3.925646906	0.66808962	-3.319469462	0.219842059	0.058776386	0.979790495
AT5G39220	AT5G39220	-7.900499138	0.001984806	0.042856385	0.989690207	-3.967003132	0.468268694	1.228328302	0.924835736	1.00175628	0.660253473	1.15106682	0.197457656
AT1G20680	AT1G20680	-7.973386375	0.001627147	0.996319433	0.679680832			2.205191024	0.721140128	1.293218038	0.613589516	0.545686113	NA
AT2G03520	UPS4	-8.045469771	0.004910637	-4.363559867	NA	3.68304403	0.204281875					0.656263233	0.929846352
AT5G03395	None	-8.184857353	0.013762388										
AT2G24010	scpl23	-8.292654734	0.000143787	-0.053109623	0.992252355	-5.198091203	0.32831486	0.972986658	0.935083701	0.528513975	0.917877035	-2.113033026	0.137937859
AT1G77860	KOM	-8.524336306	3.97692E-05	-3.054826722	0.20643017	-2.356782072	0.213183363	-2.750991949	0.66808962	-1.847914306	0.17164089	-0.538611234	0.622102072
AT3G23110	RLP37	-9.219183239	5.06028E-07	-1.23364169	0.557848567	-2.577948541	0.05945984	0.089773027	0.991559366	0.874671242	0.565321135	0.130213703	0.908248878
AT1G54860	AT1G54860	6.230278982	0.26707237	7.952391719	0.001090846	7.322647934	0.008128006	-8.205641301	0.004376318	-1.723671731	NA	-5.144868878	0.001144452
AT1G23330	AT1G23330	0.462575716	0.494165583	1.427644096	0.017217223	1.030701607	0.009551741	-1.76385337	0.009579107	-0.024795367	0.977935511	-1.24304585	2.43693E-10
AT3G44260	CAF1a	-0.650455192	0.183515175	-1.178073275	2.74426E-05	-1.315632939	1.54048E-13	2.003064698	0.021051627	-0.520685665	0.215758478	3.009450491	2.84416E-82
AT1G36060	AT1G36060	0.854921026	0.089164218	1.49389054	0.000331336	2.061266774	4.19685E-15	1.005110585	0.03662156	0.65137375	0.000863919	0.527980205	0.00644645
AT1G24580	AT1G24580	2.651824074	0.119744403	4.982257777	4.96965E-13	2.26859506	4.52355E-07	-8.325791624	0.002115343	-0.630971191	0.915081785	-1.618562238	0.061596179
AT4G13195	CLE44	1.582372023	0.500393778	-8.02551519	0.000567599	-3.439151173	0.003530471	-2.221649207	0.032525887	-0.668595669	0.499014848	-0.274973731	0.692641673
AT5G12030	HSP17.6A	1.721491629	0.576262454	2.780124182	0.044358459	5.93975152	0.000740679	-1.062395473	0.663229141	-2.129136566	0.017302289	-1.99847707	0.002169944

AT5G53710	AT5G53710	3.800291568	0.050158776	10.88348865	3.05404E-14	4.172868422	7.02636E-07	-5.357530527	0.601444432	-2.97052782	0.049028491	-2.246911183	0.005994386
AT4G21680	NRT1.8	1.611588374	0.431253858	2.198480082	0.001506372	2.639852038	0.001074969	-1.875256566	0.637124836	-1.377944464	0.009414101	-1.125139325	0.017962879
AT3G21670	AT3G21670	-0.311149042	0.656106765	2.603595158	9.2185E-13	1.594727505	1.17573E-11	-1.631505319	0.387029898	-2.369414149	3.65965E-07	-2.693556827	2.07021E-52
AT1G10560	PUB18	0.474769919	0.345184872	1.385641965	2.4353E-05	-1.119611737	1.02334E-07	1.530385563	0.548879103	-1.517372467	0.01996611	1.084019542	0.016483073
AT1G07135	AT1G07135	-0.615155649	0.291359243	-1.440730841	2.06026E-09	-1.271094052	7.06737E-09	1.419778084	0.340427521	-1.096454641	0.000531296	2.80582547	4.76236E-06
AT1G13609	AT1G13609	-4.063370825	0.23824052	-6.000653176	0.026183176	-5.675911665	0.000335096	-2.838264352	0.641679337	-7.560847351	0.000228249	-4.020504183	0.000258782
AT4G03820	AT4G03820	1.639047403	0.134140433	1.748893799	0.017738459	1.833549714	2.87456E-06	-0.677074805	0.653363031	-1.043644847	0.005005015	-0.362238249	0.126109015
AT5G01870	AT5G01870	-0.091986145	0.951720459	-2.660196617	1.4272E-05	-2.655235533	0.022571748	-0.520552374	0.685430071	1.120554902	9.88369E-05	-0.660840339	0.000279909
AT3G49620	DIN11	1.095762769	0.1331609	2.6914294	0.000369525	2.31567673	0.004760962	0.593973719	0.820186281	-0.470289847	0.446205494	1.534377415	2.09019E-06
AT3G50770	CML41	1.760678652	0.10659852	3.630463528	0.008857354	2.871273184	3.75383E-11	1.631876096	0.581277369	0.478271719	0.796321518	1.194993855	0.014493122
AT1G31040	AT1G31040	0.688012945	0.328089429	1.160585517	0.000914482	1.113117069	0.014824707	0.404529359	0.589531904	0.485710756	0.098024026	1.17037987	9.67987E-13
AT2G18550	HB21	2.461207588	0.152529365	5.037605483	0.000141682	4.009149937	1.59536E-25	-0.018907321	0.993618504	-0.134557019	0.935705727	1.084693635	0.003676947
AT1G69610	AT1G69610	2.046361731	0.204401537	1.467291805	0.043056537	1.487312444	0.0061568	-0.160891388	0.940518648	-0.255522668	0.854315137	-1.027264645	0.018763822
AT1G15840	AT1G15840	1.076520729	0.29549967	2.068842391	0.001509653	1.172234628	0.002425071	0.097824852	0.97384104	-0.566841878	0.469711207	-1.046076401	0.000111258
AT1G76705	AT1G76705	1.00101935	0.638552432	1.9147899	0.011818886	1.760193096	0.019593849	1.327841044	0.604422229	-0.145557005	0.934609223	-1.081640198	0.031187195
AT1G53790	AT1G53790	0.793828521	0.336652631	1.552571275	0.039420267	2.856040784	0.036335681	-0.228586304	0.937178328	-0.007373434	0.99569371	-1.10582003	0.016929411
AT3G27440	UKL5	2.778828125	0.112716455	4.707316822	0.004561195	2.912809811	1.8891E-05	1.200301789	0.704004387	-1.119959193	0.219838372	-1.171633715	0.021720406
AT4G09760	AT4G09760	0.434392905	0.356607418	1.426460989	6.03844E-08	1.067263565	1.80117E-12	0.799444892	0.608157116	-0.158400671	0.792871229	-1.231097599	1.57761E-13
AT1G61810	BGLU45	-0.074678977	0.95860723	3.029491203	0.024566512	2.040675313	1.18248E-06	-1.060762873	0.813580653	0.005353953	0.999095849	-1.238247445	0.000181657
AT5G52640	HSP90.1	1.854584839	0.203704194	2.760307098	1.57085E-06	2.540341824	2.48868E-07	-0.353571774	0.822213665	-0.351407194	0.493875814	-1.283542021	5.01533E-09
AT2G47890	AT2G47890	1.121948151	0.057195008	1.57319937	3.6861E-07	1.015575005	0.00048188	0.31069428	0.909760998	-0.600060817	0.431646471	-1.404506222	1.29601E-09
AT5G15240	AT5G15240	0.830477928	0.356084969	1.892539924	7.44523E-05	1.185948421	0.003862521	-0.252191478	0.965260868	-0.548616532	0.589186505	-1.415792838	0.001412258
AT1G63440	HMA5	2.064670202	0.421540379	1.705044766	0.000314668	1.50477853	3.15639E-05	-1.106372608	0.317624416	-0.761747671	0.019645646	-1.446243634	1.15313E-13
AT5G59700	AT5G59700	1.23556586	0.066321849	1.26978778	0.038397949	1.717623223	1.53924E-05	-0.576136224	0.706952662	-0.643760539	0.288283378	-1.460618873	3.16025E-11
AT1G03070	AT1G03070	0.991848556	0.76383829	2.917170933	0.031571098	4.278077979	0.000539386	-0.614537124	0.955547263	-0.075218328	0.980488802	-1.547899291	0.024131537
AT2G30550	AT2G30550	0.295027318	0.558155318	1.588673155	3.37492E-05	1.101306075	0.005010719	-0.677308984	0.434853795	-0.594132988	0.012236978	-1.588790831	5.26767E-12
AT4G40070	AT4G40070	0.723677447	0.123008206	1.664448941	0.004017173	1.094507791	0.036571739	-0.588888597	0.664635521	-0.954930283	0.045923958	-1.74409178	2.24257E-05
AT1G79900	BAC2	1.355245277	0.143476029	3.619592753	0.005452993	2.859092649	0.000339421	-2.537476747	0.058361763	-0.026068881	0.991853866	-1.785784523	6.78004E-09
AT1G46554	AT1G46554	1.244149225	0.061725881	1.200579633	0.004311309	1.342211613	0.00174566	-0.155631112	0.905793887	-0.020943641	0.988694176	-1.802287888	8.02755E-17
AT4G35090	CAT2	0.928494413	0.047133077	2.263930962	6.43732E-34	2.207754914	1.87673E-91	1.624169418	0.513982534	-0.411741066	0.154520447	-1.84019414	2.62453E-49
AT1G55740	SIP1	0.970467068	0.168021971	1.617736727	0.001015442	1.418070784	0.00088981	-0.157271561	0.958708047	0.04337872	0.977935511	-1.878081487	3.49521E-13
AT2G25090	CIPK16	0.785488443	0.418331474	2.728014126	0.004749096	1.757938075	7.22465E-06	-1.226766674	0.782971455	-1.372116723	0.237792964	-1.976552982	5.54905E-07
AT2G23030	SNRK2.9	1.256546765	0.11768119	1.81729464	0.004353203	2.192475258	7.67232E-09	-0.463871552	0.775442514	-0.576518783	0.408099027	-1.982425986	1.38627E-08
AT1G53470	MSL4	1.929905151	0.085215419	2.171200675	0.002608695	3.715993502	1.10315E-06	-1.106972078	0.465456099	-0.68619188	0.331126199	-2.155901687	1.3359E-12
AT5G24090	CHIA	1.405246234	0.225753093	2.402753787	0.023890495	2.480634274	0.000774861	-1.17478528	0.524880861	-0.798819859	0.140027778	-2.314929483	1.09136E-29
AT4G06534	AT4G06534	0.971221155	0.200220949	2.452999902	3.97052E-09	2.01443794	8.49332E-05	1.437609243	0.62738272	-1.035337845	0.473771238	-2.757044507	0.014933321
AT4G06536	AT4G06536	2.606687495	0.065823029	1.92119423	0.000158007	2.528542916	1.7452E-05	1.54559638	NA	-2.256172504	0.303704885	-2.994339148	0.015427504
AT1G69790	AT1G69790	0.187653053	0.884517224	2.64476317	0.023118066	2.164016361	0.003649252	-2.798901704	0.473778212	-0.830592087	0.430253928	-3.060819062	2.48587E-11
AT5G09930	ABCF2	-0.569286802	0.892278642	8.97061535	0.001027551	2.797876743	0.033816207	-1.46425182	0.929103958	-3.733209853	NA	-3.071982574	0.010989741
AT4G24000	CSLG2	1.065623301	0.818661478	5.954458876	9.08331E-07	3.535371384	0.032661074	-3.359762101	0.697082252	4.041406001	NA	-4.025549501	0.049104822
AT5G01300	AT5G01300	1.048241627	0.693060033	7.011082753	0.005575627	4.998238036	4.25732E-05	-1.390113017	0.75520587	0.098747746	0.984765765	-4.607581299	0.01465103
AT3G17520	AT3G17520	1.378398687	0.466673762	6.395325778	3.59803E-08	6.00230433	0.018729424	-5.076473303	0.092759749	-0.414079863	0.888839095	-4.748929501	1.23346E-24
AT5G25190	ESE3	-1.330209403	0.116325701	1.219394526	0.035203997	-1.037519569	0.047453879	-1.129996789	0.433558429	-0.165720351	0.684563426	-1.061764862	1.42565E-09
AT2G42540	COR15A	-0.780539265	0.076824189	1.886640122	1.15233E-06	-2.135728775	0.005539926	-1.066790742	NA	2.050623303	0.732723094	-5.242230149	0.013270638
AT3G02410	ICME-LIKE2	-1.379414841	0.654259763	-4.299772358	0.04690678	1.36387106	0.021619488	-0.808684129	0.937313144	0.175805993	0.976570624	1.524940464	0.01693164
AT2G28970	AT2G28970	4.456967053	NA	-6.682940813	0.041816584	3.479757868	0.002589825	-1.596164847	0.810393493	-0.551174015	0.725057451	-1.867404376	0.00683448
AT5G26260	AT5G26260	0.791250564	0.416218882	-1.733397141	7.78726E-06	1.071772967	0.005763551	-1.150204768	0.409171348	-0.770003234	1.67415E-05	-1.923352578	3.44782E-56
AT1G09240	NAS3	1.043874735	0.698757543	-3.098864733	0.0313056	2.918713463	0.019797186	1.564668185	0.602119156	1.50735224	0.39354127	-1.933403145	0.011519263
AT3G02940	MYB107	-0.893243788	NA	-6.838725965	0.024051468	7.546992968	0.007006812	0.29847689	0.936758504	-1.016195694	0.674387453	-2.212346993	0.048507512
AT5G47230	ERF5	-0.925526776	0.064792536	-1.379448425	0.007653195	-1.292953856	6.08288E-07	0.606574737	0.639875152	-0.605223293	0.293529779	3.385511677	1.0272E-163
AT5G54490	PBP1	-1.026600925	0.06040389	-1.370278671	0.010973493	-1.725072197	7.60284E-05	-0.275904858	0.850582824	-0.716474027	0.401419116	2.592831695	2.70027E-17
AT3G50800	AT3G50800	-1.019043007	0.18190867	-1.652673676	0.009301137	-1.532010018	8.03234E-06	1.524694808	0.240541784	-0.474064415	0.678149824	2.480501869	1.09633E-20
AT1G56150	AT1G56150	-1.184140344	0.051712702	-1.36372707	0.00134645	-1.386180633	0.003819152	1.368834938	0.183477648	0.838077506	0.258496511	2.159663264	3.29611E-19
AT3G46620	RDUF1	-0.831515546	0.062090056	-1.349540423	2.92234E-08	-1.105429323	2.22065E-05	0.895503482	0.399892108	-0.214955038	0.511188955	1.979528255	7.74716E-38
AT1G32928	AT1G32928	-0.290923429	0.661517739	-1.272544287	4.7126E-05	-1.058995397	0.003019497	1.542520605	0.114681201	0.117050255	0.897458174	1.780651094	5.73987E-29
AT1G72950	AT1G72950	-2.571898084	0.116773259	-5.353544466	0.000514535	-1.565884501	0.035732699	-6.845261298	0.279591669	-0.685336284	0.883410478	1.604409666	0.000374233
AT3G49260	iqd21	-0.971638625	0.000202689	-1.013517292	1.179E-06	-1.19025538	6.12465E-13	0.756999974	0.517374656	0.582215433	0.1393487	1.531870496	1.55952E-29
AT2G27402	AT2G27402	-2.613554659	0.113885703	-4.41146873	0.000102175	-4.250972081	1.08449E-05	0.42085729	0.912756407	0.353378582	0.295937959	1.453184783	2.97697E-16
AT2G41100	TCH3	-0.849415696	0.123042265	-1.286301357	2.2087E-07	-1.264309004	1.36599E-14	1.288283299	0.55451616	-0.648359908	0.111039984	1.380410327	1.17112E-25
AT1G55670	PSAG	-0.937820385	0.051427648	-1.180025095	3.27769E-11	-1.279535746	3.25413E-36	2.290853628	0.134938826	0.979685927	0.017928061	1.271480599	9.18635E-11
AT3G57450	AT3G57450	-0.632657542	0.226255768	-1.393256224	2.41347E-07	-1.188544908	3.03475E-06	0.530210696	0.616300683	-0.205780276	0.514972377	1.219384016	1.13997E-09
AT1G15570	CYCA2;3	-1.389954514	0.095713599	-2.654321154	1.93035E-05	-1.926816335	2.3908E-09	-0.346715994	0.905158299	0.269125931	0.529391821	1.184731151	2.42511E-12
AT2G37390	NAKR2	-1.498790535	0.139303974	-1.717848332	0.020869943	-2.451345654	4.02746E-06	0.110169732	0.97283519	0.610022902	0.499322416	1.171362484	5.8936E-06
AT5G63140	PAP29	-1.329779508	0.083235046	-2.253913891	0.000340914	-1.799556954	6.00904E-14	-0.336216121	0.681538103	0.365194917	0.168222434	1.130069309	2.21696E-22
AT1G13790	FDM4	-1.008152743	0.156138135	-2.952953818	0.000525775	-1.733827437	0.018347604	0.503095093	0.850321406	0.434393919	0.638422258	1.113055451	8.82768E-06
AT4G37110	AT4G37110	-1.495935083	0.065631603	-1.47579476	0.016063469	-2.209259257	1.64234E-13	-0.15129376	0.947082547	0.225639722	0.609403216	1.065505945	3.34074E-11
AT1G31550	AT1G31550	-2.283090226	0.178844627	-1.61922711	0.043614626	-2.390324489	3.32766E-06	3.350765154	0.620656096	1.640050548	0.650894952	1.042644159	4.7871E-05
AT1G52400	BGLU18	-1.27119924	0.148270042	-1.872740012	3.29652E-05	-1.185805391	0.012331977	0.704623906	0.780552255	0.241298266	0.895246951	1.027311166	1.02226E-05
AT2G29180	AT2G29180	-0.863385623	0.04780325	-1.263446499	0.001596957	-1.402544872	7.73897E-10	0.766947763	0.691895802	0.782502042	0.090455663	1.002576617	1.85851E-08
AT2G44460	BGLU28	-1.569031868	0.745378776	-7.180403688	0.012295256	-6.869158323	0.005028767	-0.611551647	0.967195835	-0.528621091	0.708283869	-1.026105266	0.02208144
AT1G08430	ALMT1	-1.962742118	NA	-3.454757597	6.30454E-07	-4.542239212	0.000355684	-0.754442624	0.706790319	-0.656817297	0.176850826	-1.030987313	0.000169421
AT3G20015	AT3G20015	0.434353281	0.531926046	-1.232885772	0.002796316	-1.148698236	0.014561278	-1.196296828	0.627500883	-0.275717809	0.56208899	-1.154219332	6.15801E-16
AT4G20230	AT4G20230	-2.034237962	0.319074855	-4.865836406	0.00676058	-3.227274507	0.005241821	-1.052484162	0.816439804	-0.701392552	0.50048846	-1.197150943	0.007330727
AT4G31910	BAT1	-0.628626644	0.456480199	-1.445937488	0.021850527	-1.357378702	0.008893052	-1.315897326	0.454321515	-0.516690756	0.176633494	-1.845990166	4.63165E-46
AT3G25190	AT3G25190	0.915857991	0.056400805	-2.406091571	0.000100877	-2.458162674	1.69177E-05	-1.953960815	0.143428401	-0.449753165	0.542576874	-1.932216704	4.27931E-14
AT4G23720	AT4G23720	-0.648128899	0.873283065	-7.687970784	0.001667327	-6.840680612	0.00996071	-5.111604685	0.143428011	-0.18206505	0.936930362	-2.105847771	0.001494764
AT2G16760	AT2G16760	-0.99930489	0.730254892	-6.646212166	0.043235322	-6.827112772	0.008609043	-1.719872737	NA	-0.787296797	0.646791252	-2.165289684	0.000100067
AT3G56980	bHLH39	0.657217017	0.41333403	-3.694400263	0.007577128	-5.720376098	2.28092E-05	-2.356534626	0.570475688	-0.725866704	0.532355117	-3.021999071	3.98399E-20
AT1G47395	AT1G47395	0.037221128	0.974458714	-1.736332428	0.006112039	-3.377604246	1.29818E-09	-0.475858846	0.759702709	-0.460712222	0.727458862	-3.170490969	6.54459E-10
AT3G56970	bHLH38	-5.380961502	0.09899812	-9.925483036	6.66964E-11	-6.065400616	0.020338489	-7.449111368	0.060861487	-3.589753392	0.146467869	-4.028847225	1.17689E-06
AT2G30766	AT2G30766	-3.878143495	0.071326322	-8.430614927	2.02381E-05	-5.01184107	0.002742661	-4.998842802	0.665832077	4.549090345	0.519557487	-4.356889291	5.94048E-07
AT5G66780	AT5G66780	2.908673792	0.154934837	11.41651339	6.5448E-06	10.94475006	4.54967E-07	-9.047529768	0.058305704	-0.31362575	0.949699423	-1.282166691	0.173919718
AT1G27461	AT1G27461	3.778660162	0.128051715	11.76349119	1.81954E-16	10.04451919	0.031535795	-5.74989862	0.63192307	4.120646097	NA	-3.465380228	0.301393291
AT4G31830	AT4G31830			8.397041928	0.000136636	7.976700826	0.001214923	-3.862355172	0.731041294	6.844487252	0.110157263	-4.547450668	0.178495085
AT5G58465	ath-MIR390b	0.666955535	0.909864575	5.16092022	0.017903845	6.585715698	0.009505926	0.039457837	0.998026502	0.529809518	0.945176019	-0.394090665	0.806930655
AT4G33465	SCRL22	6.650268574	0.186309519	7.139753369	0.021965984	6.250238667	0.033455727	-2.192461302	NA			-3.246636424	0.608744561
AT5G33355	AT5G33355	3.486629342	0.223738608	9.047110944	4.15773E-06	6.124067409	0.034931827	-3.803117872	0.210735808	-2.482267046	0.103973183	-4.629849836	0.346015974
AT1G72760	AT1G72760	1.834295747	0.124139537	3.306353528	1.45094E-05	5.686800286	2.5663E-11	1.697115669	0.712887303	-2.238293306	0.287620198	0.322124746	0.709773652
AT4G16270	AT4G16270	2.209030863	0.293507219	6.300948247	1.96089E-05	5.48531374	0.008207603	-0.182776443	NA	0.805691954	0.830492367	-0.949581945	0.645082103
AT1G01480	ACS2	2.370825774	0.183364852	4.136946133	0.004346055	5.43599979	7.16946E-10	0.146229793	0.982529985	1.258839134	0.371127498	-1.847904795	0.119775757
AT3G15670	AT3G15670	3.786995241	0.057145401	10.6531024	3.21052E-14	5.247764179	1.35326E-08	-6.589681107	0.579506696	-0.290811504	0.963963546	-2.547357543	0.120857319
AT3G22840	ELIP1	2.404144774	0.331020991	10.86267085	8.09372E-13	4.898835936	0.000136934	-0.128423925	0.986613165	-0.983067803	0.60004464	0.143743499	0.864014836
AT3G18550	BRC1	0.397344268	0.87133725	8.767650853	2.48389E-05	4.750592028	0.00034811	2.298803138	0.301149642	-1.380586654	0.511503982	0.964204841	0.321710223
AT2G29380	HAI3	0.443332513	0.905746628	5.909124244	4.49206E-05	4.379158866	1.764E-06	-0.51632596	0.957250593	2.60087111	0.550718639	-1.126668046	0.572809065

AT2G46670	AT2G46670	2.650940097	0.058617708	5.70516038	4.18533E-07	4.241572952	1.06059E-06	0.781719289	NA	-2.919207811	0.601650081	1.014226914	0.511982725
AT3G22740	HMT3	1.454083893	0.170151357	2.01090822	0.019180468	4.00885423	1.30196E-09	-1.070193145	0.791390498	-0.207486764	0.929498792	-0.445881951	0.636208738
AT1G52560	AT1G52560	1.945315998	0.339653767	4.675942644	0.016163623	3.487284794	0.002086568	-2.898716996	0.640704059	2.688727768	0.284359663	-1.077959716	0.142818027
AT1G32560	AtLEA4-1	4.071934545	0.272643421	6.179948787	0.000513358	3.293695303	0.001098867	1.911687205	0.680595832	0.55933586	0.879031376	-2.12343955	0.091913795
AT2G16660	AT2G16660	1.512584177	0.206817636	1.750975938	0.027272758	3.132188199	3.85915E-05	-0.517314824	0.598455054	-0.068325415	0.927151022	0.020796489	0.945241305
AT4G39360	AT4G39360	3.57224347	0.110837092	4.542563343	0.00038184	3.009311299	0.011981041	-2.23147094	0.665832077	-1.007993855	0.700995813	-1.114442852	0.255058142
AT3G07340	AT3G07340	0.53722533	0.414205693	1.235986242	0.001158198	2.964270955	6.60451E-38	-0.456079472	0.731041294	-0.356278645	0.735176002	-0.313847378	0.389554269
AT4G11890	ARCK1	2.221228878	0.22436711	3.696854412	1.48319E-11	2.930300506	4.68972E-05	0.344071941	0.967187969	-0.200776251	0.964180223	-0.096212165	0.937560253
AT1G21550	AT1G21550	2.511739501	0.103068265	2.804144991	4.32603E-05	2.912135676	4.79911E-08	1.602259505	0.721040326	1.087962802	0.592879849	1.089133414	0.338715376
AT2G36750	UGT73C1	1.229921259	0.863086651	7.826737272	0.001937641	2.840530939	0.028314662	0.687992217	0.971297857	-1.389274391	NA	-1.20235345	0.429508612
AT2G35950	EDA12	0.968089632	0.356411925	2.129084325	0.021070894	2.766288676	0.000450888	0.141728165	0.969967158	0.152091659	0.929498792	0.170529508	0.675481185
AT5G20230	BCB	0.931874578	0.103305965	1.352463578	5.22044E-12	2.728301172	9.71839E-48	4.016522893	0.184799885	0.345952011	0.599757243	0.854061155	9.26732E-07
AT3G03240	AT3G03240	3.601374236	0.156740751	7.848224494	0.0204808	2.647001444	0.027353424	-1.654744346	0.692385923	-0.7673928	0.49658841	-0.709091314	0.165850881
AT5G28010	AT5G28010	1.434962069	0.143365778	1.877815805	0.017302753	2.643684105	0.00289765	-0.580387997	0.820186281	-0.046074874	0.975386078	0.049078714	0.908248878
AT2G46970	PIL1	4.125681594	0.20051295	2.160054716	0.002611662	2.588526799	0.012283435	-0.667855011	0.799598167	-0.162273401	0.719544808	-0.527916853	0.000734938
AT1G19610	PDF1.4	1.312413207	0.076823038	2.237020569	4.52907E-05	2.497726086	3.39814E-06	0.218615998	0.921880856	-0.33506887	0.78408754	0.036110378	0.963145211
AT5G10946	AT5G10946	2.821330867	0.249501265	1.112297875	0.02506862	2.444511726	1.91462E-19	0.224126199	0.914095872	0.728433977	0.071751927	-0.685377148	0.081313632
AT2G29090	CYP707A2	1.581989963	0.446039526	2.44933888	2.74168E-05	2.372473444	0.003100229	1.153778202	0.632509641	-0.18622091	0.950400259	0.628988913	0.236498952
AT2G16750	AT2G16750	0.301452317	0.755903721	1.18188549	0.033217624	2.368655421	2.32644E-19	-0.40027676	0.760971046	-0.469886137	0.261998738	-0.043181994	0.88764063
AT3G28210	PMZ	1.321262852	0.070372763	1.521444517	0.036571438	2.342067716	0.003598045	0.595748411	0.781016963	-1.161225566	0.244244745	0.382863518	0.538879702
AT3G04000	AT3G04000	1.124551472	0.218994712	2.230200427	0.000577063	2.256034121	1.27189E-06	0.138405807	0.966082349	0.402731485	0.639543122	-0.244484424	0.47126727
AT5G20380	PHT4;5	0.461686312	0.297325037	1.034397359	0.000275713	2.144027543	6.552E-21	0.149688632	0.943118218	-0.021152983	0.973023368	-0.071749066	0.708566231
AT4G22950	AGL19	2.361915599	0.215994667	1.996038305	0.037406301	2.137472337	0.020216609	1.123672143	0.658916837	2.348753419	0.050454592	0.785910169	0.247983311
AT2G36080	ABS2	0.712676843	0.120086116	1.332639313	8.97709E-05	2.098649525	1.41013E-15	-0.310441156	0.691824202	0.107755398	0.79996985	-0.178217432	0.439142943
AT1G51140	FBH3	0.94229582	0.092837302	2.577967988	6.21194E-16	2.08782336	3.6388E-05	-0.351880071	0.767112256	-0.078470321	0.905220501	-0.659760001	0.000226246
AT4G21440	MYB102	2.076601818	0.050152116	4.739772755	0.000588859	2.055446225	0.006559245	0.234878984	0.982734229	0.713084226	0.77937019	0.055294055	0.97995131
AT5G37670	AT5G37670	1.321170455	0.263905113	1.971825643	0.028629035	1.985340876	0.030288614	0.027236802	0.992836576	-0.178823458	0.925402712	-0.780922096	0.135700274
AT5G03190	CPUORF47	0.901934671	0.018663951	1.994364371	3.32937E-06	1.96686195	6.8386E-16	0.46845826	0.747187452	0.028678133	0.969516868	0.371973059	0.067170574
AT3G19270	CYP707A4	2.113321153	0.260981305	2.782758237	0.000174634	1.938609607	0.002896287	2.314613287	0.404019457	-6.966791252	0.081267691	-0.828697815	0.378305947
AT2G04040	DTX1	0.900455438	0.707352427	2.208840845	0.005893964	1.926452904	0.002290232	1.035151678	0.680482607	1.717365499	0.246182324	-0.449499419	0.749040762
AT1G17870	EGY3	0.132996617	0.863086651	2.204815978	2.26819E-05	1.906343436	0.002054018	-0.706519209	0.720336606	-0.508217466	0.572236533	0.065685149	0.891596693
AT4G28020	AT4G28020	0.693524494	0.240252354	1.254919019	0.010559222	1.888861993	3.25937E-11	1.223506996	0.286531805	0.050135913	0.966304751	0.29498141	0.215120729
AT3G53160	UGT73C7	0.633582682	0.722531432	2.826215425	9.51399E-07	1.876063815	0.004778552	-1.54904199	0.697407195	0.564418492	0.732399618	0.079103887	0.92470567
AT4G34890	XDH1	0.762388878	0.030299207	1.233033245	5.55242E-06	1.85048278	9.38839E-87	-0.140278058	0.944803625	-0.194451942	0.434258931	-0.168334826	0.338335293
AT3G47340	ASN1	0.866714065	0.064765488	1.071566065	1.42589E-13	1.781633332	1.55023E-26	2.870366586	0.073310574	0.188067397	0.86223157	0.351897964	0.10890804
AT3G62590	AT3G62590	2.340031906	0.170612885	2.110467481	0.000119897	1.771152919	0.014410765	-1.030427639	0.306026996	-0.128109005	0.930903945	-0.835991853	0.000575302
AT2G35820	AT2G35820	0.882386717	0.015893409	1.072003863	3.34027E-09	1.750206621	1.51997E-52	0.903861446	0.409180759	0.234707872	0.593861533	-0.006965806	0.970042231
AT5G67480	BT4	0.984439874	0.065372881	1.634574687	1.75108E-10	1.749548039	1.94709E-23	0.728863318	0.588156522	0.04049682	0.933631495	-0.44754017	0.017368843
AT4G15236	ABCG43	-0.2407353	0.920555722	2.020406914	0.042324457	1.724417932	0.004491716	-0.740629354	0.658158748	0.104844216	0.963495178	-0.239783829	0.786091796
AT5G15860	PCME	0.966112606	0.001569676	1.64596314	2.07764E-12	1.714371094	6.55184E-15	-0.345797835	0.669689637	-0.028315738	0.964513621	-0.241692132	0.20526308
AT4G18650	AT4G18650	3.014245484	0.205445818	3.152368092	0.009570842	1.687855781	0.002300261	-2.201934331	0.720012409	-1.249337725	0.616572022	-0.229693083	0.895683424
AT3G58750	CSY2	0.9627114	0.004266701	1.147902998	5.92009E-09	1.65730244	4.96439E-35	0.615120978	0.647947756	0.041237493	0.935705727	0.048860102	0.811585798
AT1G68500	AT1G68500	3.304370098	0.159728641	3.318685325	3.38221E-05	1.645814808	0.000847885	0.065821905	0.990391014	0.118366546	0.935637155	-0.580399691	0.392718192
AT5G13490	AAC2	-0.052016748	0.935769115	1.20963561	0.000442954	1.630255641	0.000295259	-0.510102993	0.691895802	0.143117784	0.769545948	-0.706402419	0.006937011
AT3G59530	LAP3	1.098905116	0.376178446	2.432762999	9.75354E-05	1.62163188	0.001165245	-0.330989702	0.90485789	0.212172954	0.870101981	0.201104865	0.645082103
AT3G27870	AT3G27870	0.93272372	0.044341842	1.925786563	1.43239E-08	1.613244163	6.4019E-08	-0.019864739	0.989159859	0.330302047	0.487203495	0.035606483	0.901004011
AT4G18210	PUP10	0.558730067	0.557368222	1.656356013	0.005956855	1.580142384	2.53297E-05	-0.208983709	0.926713558	-0.21662873	0.84694947	-0.310753086	0.293726349
AT3G20300	AT3G20300	0.874778337	0.036358345	2.151517262	2.92194E-06	1.545999644	3.80114E-07	0.051563813	0.982734229	-0.193148942	0.684515288	-0.924447868	2.99089E-09
AT5G50240	PIMT2	0.671462254	0.488357723	1.973059467	0.03392738	1.542371238	0.007365974	-1.323039709	0.591481547	-0.008463579	0.997588168	-1.189195729	0.060300375
AT4G31240	AT4G31240	0.858051175	0.042233491	1.211528722	0.019312065	1.538666498	9.63769E-09	0.21940928	0.820789421	-0.106765222	0.898728824	-0.688804553	5.21331E-05
AT3G52340	SPP2	0.915365301	0.020477006	1.76567522	5.60699E-20	1.517438486	5.14578E-28	0.755349178	0.413866874	0.485508158	0.006064026	0.211665473	0.286025974
AT4G33950	OST1	0.933554125	0.016866881	1.2307247	0.000152484	1.499581626	3.27872E-09	-0.218477935	0.745375315	-0.157185832	0.690459445	-0.172266814	0.367098955
AT4G36760	APP1	0.725141198	0.01792698	1.536449829	8.0104E-13	1.481032419	2.50366E-23	0.240989612	0.848507895	-0.170896798	0.515397723	-0.106154896	0.648085745
AT2G39050	EULS3	0.792751592	0.379888724	1.286571047	0.001587119	1.480370139	0.00038911	-0.973183434	0.492642336	-0.16858503	0.850188481	-0.8682876	0.002057463
AT1G10865	AT1G10865	0.645274057	0.436596931	1.294595521	0.00049823	1.475285967	2.22711E-11	0.392762853	0.716554635	0.262892508	0.721412596	-0.271638064	0.133880451
AT3G59140	ABCC10	0.636090168	0.519395998	3.144786266	4.33847E-07	1.473236535	0.013936671	-0.506660193	0.823318742	0.271528819	0.714991703	-0.048103126	0.912934203
AT4G36260	STY2	0.737640155	0.379142749	1.060160422	0.007617555	1.47103146	0.00143195	0.539764346	0.585524552	0.366334841	0.548029196	0.087101097	0.860866297
AT5G05310	AT5G05310	0.397516323	0.634360081	1.11454211	0.009220006	1.455658735	2.62511E-12	0.743667837	0.619462504	-0.38317222	0.377377981	0.194004541	0.53604339
AT1G69280	AT1G69280	0.564020583	0.661040932	1.738496884	0.003363751	1.452320061	0.034846818	0.246560101	0.93556951	0.215329057	0.854847579	0.229966789	0.710015308
AT2G25450	AT2G25450	0.795990028	0.036874098	1.763419872	6.88287E-19	1.424881464	1.0589E-54	1.347987548	0.353314166	0.048156214	0.924627441	-0.614300996	0.000114155
AT3G10740	ASD1	0.654957934	0.04129205	1.220483546	4.59211E-10	1.400604289	3.13497E-19	0.272515723	0.864987393	-0.125627354	0.656772532	-0.362827483	0.003845919
AT1G28960	NUDX15	0.905177538	0.005152345	1.362745519	4.44112E-07	1.398120026	2.30896E-18	0.059577176	0.972495118	-0.153241864	0.606138332	-0.119766458	0.559201891
AT3G01590	AT3G01590	0.777323401	0.081762749	1.654891536	3.55229E-10	1.3969539	1.10741E-13	0.554007051	0.665778009	-0.016797126	0.979610562	0.097354782	0.587589462
AT5G51970	AT5G51970	0.933559264	0.004054865	1.060601525	2.4325E-12	1.386512149	3.82311E-22	1.424431288	0.327697498	0.041570836	0.930662149	-0.178790229	0.442173722
AT1G13150	CYP86C4	2.289982268	0.12120731	1.4772477	0.001048824	1.385006828	0.003387933	1.387156108	0.294511423	0.186937696	0.89611977	0.570416192	0.224334625
AT1G12790	AT1G12790	0.194759565	0.779886613	1.23179223	0.000249702	1.377507452	1.21928E-10	0.490503674	0.731041294	0.042316209	0.963495178	-0.353362333	0.079762093
AT3G55610	P5CS2	0.862848061	0.002010874	1.07445773	5.00825E-06	1.377025102	6.64945E-36	0.294937115	0.8208148	-0.156142903	0.486297808	-0.950926969	1.29124E-13
AT4G34020	DJ1C	0.754780142	0.037683511	1.420167642	1.7589E-14	1.373498969	2.88816E-28	1.186598195	0.387045614	0.155088616	0.611423968	0.379612925	0.016842943
AT2G33590	CRL1	0.68781732	0.079298792	2.612791964	1.11951E-19	1.364657634	2.06493E-05	0.657927059	0.67307941	0.089490646	0.883410478	-0.085025359	0.779875273
AT5G19440	AT5G19440	0.907690436	0.005423202	1.877824336	3.78143E-12	1.359900896	9.67648E-08	-0.232444461	0.815085418	0.243378076	0.292482683	0.16139578	0.31772588
AT3G14010	CID4	0.928194647	0.000841118	1.323529572	9.74539E-16	1.353384719	8.45708E-28	0.36807264	0.744742191	-0.088995759	0.778769276	-0.192131361	0.249250184
AT4G05060	AT4G05060	0.878435202	0.035678671	1.184143835	8.23488E-10	1.342141333	9.59233E-23	0.869882683	0.562656211	-0.140923129	0.755752983	0.136968632	0.499389837
AT1G10170	NFXL1	0.688434211	0.005192376	1.155122706	5.65367E-10	1.338913109	8.27038E-24	-0.033423079	0.987613184	-0.113819876	0.783804237	-0.265247425	0.11436289
AT5G57240	ORP4C	0.954005958	0.087187848	2.283386969	2.11667E-20	1.322272836	1.60518E-14	0.271234338	0.885840009	-0.044044267	0.944236118	-0.285213807	0.212472587
AT5G03495	AT5G03495	2.236075259	0.197511644	2.246562526	0.039827557	1.31845327	0.028833146	-0.289744354	0.974340012	0.234390156	0.929498792	0.173589736	0.792740938
AT2G45670	AT2G45670	0.885629006	0.022927752	1.039365998	1.66021E-16	1.312310185	1.69257E-18	0.666430022	0.653363031	-0.202337997	0.318380163	-0.377839173	0.006974491
AT1G49050	AT1G49050	0.75594423	0.104083644	1.204913083	6.67095E-07	1.311994769	5.1345E-11	0.089821649	0.938659772	-0.302745374	0.511188955	-0.293829392	0.110343453
AT5G05600	AT5G05600	-0.673989531	0.2091129	1.018380367	0.035468855	1.30487773	0.04053594	0.215100749	0.930313852	-0.065391361	0.962017915	-0.456250304	0.257113412
AT2G28120	AT2G28120	0.550643022	0.398515052	1.904262051	8.35323E-09	1.298801485	5.84148E-11	0.206327016	0.911509594	-0.412916903	0.361184589	-0.73364698	0.00757866
AT3G08730	PK1	0.60754632	0.08717445	1.050810644	7.51849E-12	1.295802846	5.01792E-30	0.500589922	0.66524984	-0.120788592	0.676942605	-0.46566892	4.50074E-05
AT2G01020	AT2G01020	0.965192449	0.071992722	2.494296978	2.77042E-08	1.292062714	2.46148E-08	0.696439893	0.703833757	0.288183582	0.771934863	0.846188378	0.003730663
AT1G68570	AT1G68570	0.285943859	0.747697474	1.098625371	0.047359005	1.292042314	0.000409481	-0.701125641	0.574062337	-0.041004607	0.966322281	0.750517963	0.000209235
AT3G04500	AT3G04500	0.939431875	0.002159976	1.039723506	8.72795E-05	1.28222967	1.07707E-15	0.274048115	0.732322487	0.204957978	0.53525964	0.191388586	0.298412573
AT5G36160	AT5G36160	0.621669569	0.229638456	1.88554923	1.2665E-11	1.271856627	1.77746E-21	1.034435101	0.477936046	0.435091259	0.242508148	0.121006792	0.608744561
AT4G17840	AT4G17840	0.693599673	0.227244763	1.596819105	2.81432E-05	1.267793942	1.3576E-11	0.523479351	0.697057688	0.030715977	0.976044889	-0.700928504	0.017209498
AT1G12640	LPLAT1	0.660377647	0.103251419	1.090208875	2.36404E-05	1.258802444	4.15748E-13	0.219652093	0.769328597	-0.215923008	0.55787595	-0.404257387	0.006862611
AT5G57340	AT5G57340	0.909318908	0.044371242	1.082302892	9.8998E-10	1.253125971	1.36788E-13	1.054698437	0.300202167	0.277750686	0.381906601	0.619781717	6.30896E-05
AT2G41380	AT2G41380	1.085075468	0.160536825	1.593560465	0.01039228	1.251492827	0.003633879	-0.311517954	0.879613371	-0.007004819	0.994809352	0.043913101	0.921946213
AT4G10070	AT4G10070	0.995263732	0.001794366	1.43018924	2.58618E-11	1.250726973	3.26922E-09	0.517714775	0.712033791	-0.130064913	0.825713105	-0.10359376	0.632870798
AT1G01770	AT1G01770	0.994515943	0.011608761	1.105657928	0.000612715	1.239893082	1.08684E-18	0.334603676	0.796259461	0.317756655	0.371421607	-0.135688108	0.590279413
AT2G33150	PKT3	0.914742934	0.000905745	1.498503913	6.96692E-12	1.237205514	4.35084E-17	0.338483778	0.754554363	0.102645813	0.672329478	-0.054938234	0.719503216
AT4G20930	AT4G20930	1.069410997	0.070745901	2.154514455	4.1508E-09	1.236605655	2.66319E-05	0.525471716	0.663046749	0.382149098	0.36223266	0.55702337	0.004154057
AT4G36730	GBF1	0.839891579	0.012743932	1.06291339	1.79167E-12	1.234567687	1.85422E-14	0.96663422	0.558566659	0.376516602	0.023352521	-0.000279552	0.999012802
AT5G62090	SLK2	0.953694807	0.012480622	1.85158088	5.35182E-13	1.233889397	7.24786E-14	0.39849342	0.734168325	-0.104517196	0.7682641	0.031906919	0.887777229

AT2G36540	AT2G36540	1.461994894	0.448921756	2.768459993	0.000118633	1.226671798	4.17804E-06	0.049974766	0.988042725	-0.401549904	0.847151562	-0.65064684	0.036362621
AT1G55080	MED9	0.370155768	0.58063046	1.119624771	0.036511126	1.222920288	1.27645E-05	0.195118871	0.866385436	0.077622619	0.929239543	-0.254210821	0.288334221
AT5G13800	PPH	0.970888259	0.019995728	1.529598343	8.76352E-17	1.222603118	1.52134E-09	0.694446222	0.640704059	0.321473312	0.233539745	-0.299091382	0.034766221
AT5G63200	AT5G63200	0.848634212	0.018809087	1.631670338	4.81277E-13	1.211804967	2.04919E-16	0.367309517	0.716289725	0.173890404	0.58081406	0.03603202	0.891330254
AT1G09530	PIF3	0.82226736	0.011403434	1.603471626	6.46947E-12	1.209989212	6.55821E-14	0.304498643	0.726900342	0.449785548	0.004812535	0.968594203	7.41164E-17
AT1G73890	AT1G73890	1.325047018	0.128192174	1.64270387	0.005836394	1.207049389	0.016751255	0.063635486	0.984609535	0.556185618	0.531752878	0.403499207	0.409249159
AT4G24800	ECIP1	0.999270715	0.000304921	1.369916438	1.16789E-09	1.187410376	1.0876E-13	0.433354013	0.706790319	0.027604355	0.944687474	-0.165656983	0.277787403
AT5G28390	AT5G28390	0.616530241	0.309314492	1.411878578	4.13992E-09	1.185697952	1.64728E-05	1.333670078	0.498296358	0.162458233	0.846389857	0.100708427	0.757733808
AT1G25420	AT1G25420	0.647328448	0.0923776	1.116314693	8.31485E-07	1.181618082	6.17521E-14	0.160051783	0.894832627	0.308497349	0.24935218	0.217470531	0.174995142
AT5G04340	ZAT6	0.863226264	0.081936145	1.233979276	0.000327116	1.171735743	0.001063092	0.687233739	0.672344553	-0.576768973	0.201309169	0.040050672	0.908500767
AT3G54620	BZIP25	0.442889959	0.143782788	1.070610705	3.38496E-06	1.170121795	5.5503E-14	-0.166771183	0.880294241	-0.179887908	0.485655053	-0.279009728	0.04748072
AT5G05800	AT5G05800	0.978329019	0.001260773	1.128861973	2.22986E-12	1.170102956	1.11052E-13	1.000929206	0.413284704	0.155887988	0.565667698	0.09800518	0.573785288
AT5G54300	AT5G54300	0.772382901	0.298216422	1.149169708	0.037302865	1.166132871	3.22442E-05	0.342889096	0.885892922	0.053593713	0.97361709	-0.332829089	0.249257489
AT1G32870	NAC13	1.245843368	0.053007479	1.603881457	0.001241297	1.160670588	0.035958552	-0.894183466	0.53420839	0.340341611	0.665526143	-0.692743966	0.0014478
AT1G16090	WAKL7	0.697574559	0.422518711	1.037670783	0.021181388	1.159744758	0.002536292	0.13907992	0.952021801	0.108797705	0.934609223	0.323237105	0.343739516
AT5G57660	COL5	0.818478415	0.021461605	1.091099521	6.39383E-21	1.157291588	3.24108E-14	1.324802571	0.373396563	0.252623773	0.182187499	0.118658623	0.425285422
AT2G25140	CLPB4	0.919963947	0.013276865	1.216250626	9.94292E-05	1.156428793	0.000465308	-0.1940626	0.796134167	-0.307567727	0.441942321	-0.27717355	0.242359705
AT5G43920	AT5G43920	0.762123008	0.056785144	1.103288073	0.000829923	1.151846069	7.50001E-08	0.675964525	0.474958589	0.325590344	0.221431884	-0.145677612	0.452635414
AT4G14746	AT4G14746	0.471710869	0.303617475	1.476776853	0.000562543	1.150677105	0.000699399	-0.628401028	0.627731816	-0.413564962	0.234194935	-0.391100273	0.017586298
AT1G60160	AT1G60160	0.954584249	0.000516228	1.250175202	4.90136E-08	1.148523869	1.2144E-11	0.344579709	0.699108913	0.183977848	0.511659422	0.449875832	0.000222384
AT4G16760	ACX1	0.883330163	0.001083726	1.85531983	4.71762E-33	1.147338628	1.64426E-10	0.31273115	0.686926276	0.308129271	0.072389536	0.004296786	0.985220842
AT3G06500	A/N-InvC	0.740923589	0.066983595	1.259805539	0.000158007	1.13973623	8.80385E-13	0.635928397	0.680143812	-0.013010601	0.986218148	0.513574335	0.003640207
AT1G29240	AT1G29240	0.64454172	0.417504264	1.053191578	0.00926987	1.13870017	0.001296137	0.021088496	0.991271129	0.512661919	0.29098652	0.190349273	0.471495702
AT4G28150	AT4G28150	1.367524236	0.100918111	1.682306912	0.000921879	1.138468886	0.015811184	0.739858349	0.605522309	0.163801333	0.883116962	0.006837928	0.992516322
AT1G17640	AT1G17640	0.986474586	0.233418036	1.513886139	0.000527475	1.128799383	0.000351152	1.06103458	0.465456099	0.278526858	0.675165193	0.092786638	0.792024222
AT2G41945	AT2G41945	0.940412975	0.037691902	1.679345149	1.2817E-05	1.124389032	9.42337E-06	0.228567707	0.73450104	0.107519673	0.863621528	-0.323062333	0.089708764
AT3G27020	YSL6	0.774027791	0.017656013	1.354070053	8.08307E-10	1.123790672	1.42289E-15	0.730912621	0.637815859	-0.273761719	0.284359663	-0.194258686	0.235282223
AT1G04830	AT1G04830	0.550212881	0.250380896	1.010411627	0.000252357	1.119937674	1.12125E-10	0.300142567	0.842161979	0.161796859	0.758161633	0.190850657	0.376201052
AT2G26920	AT2G26920	0.958578911	0.009359284	1.159380888	9.31844E-07	1.119853007	3.07017E-08	0.45449312	0.731041294	-0.067668633	0.896721488	-0.008781635	0.970018299
AT3G57760	AT3G57760	0.709636848	0.191608545	1.735917011	0.000333951	1.117196826	0.005004404	1.17256376	0.586831374	-0.443280465	0.529833936	0.918459821	0.000303474
AT1G06430	FTSH8	0.782624374	0.075636437	1.616934177	7.85818E-14	1.106960814	1.1052E-09	1.59741475	0.218297828	0.128163562	0.794004103	-0.615552056	4.36165E-06
AT2G31130	AT2G31130	1.129538135	0.080765195	1.197304665	0.049345765	1.105829003	0.001022718	0.536886782	0.644839236	-0.218550932	0.806979855	-0.372647283	0.174857063
AT1G26930	AT1G26930	0.97039189	0.001993211	1.002339451	0.020842282	1.104286734	0.00142024	0.018639553	0.991271129	0.786394355	0.000567199	-0.044540421	0.903810867
AT1G56612	AT1G56612	0.626348116	0.405679548	1.25097761	0.001284547	1.103799903	0.000495755	0.295781325	0.776796299	-0.075130289	0.943180766	0.535962159	0.142560653
AT5G01880	AT5G01880	2.960803865	0.216856706	2.488262376	2.08614E-05	1.101667272	0.040536187	-0.916188311	0.637815859	0.637557481	0.493493798	-1.57580851	0.107540638
AT5G43260	AT5G43260	1.213744216	0.052364522	2.015451129	2.73169E-07	1.09600578	0.001601647	0.774527572	0.647435191	0.33945367	0.609403216	-0.51490317	0.141738976
AT1G31880	BRX	0.756345959	0.292105297	1.400523944	0.035602238	1.094036329	0.028889226	-0.274691797	0.876015705	-0.049153115	0.971700698	0.246070269	0.442977379
AT2G24180	CYP71B6	0.927656686	0.009473451	1.275066448	1.32713E-06	1.09129772	7.70216E-07	-0.087043858	0.950367876	-0.315829355	0.234682665	-0.695940137	5.05346E-07
AT4G27020	AT4G27020	0.616928589	0.103750643	1.358278949	0.000257035	1.090042771	1.91952E-07	0.159372665	0.930313852	-0.004071737	0.996495898	-0.434536526	0.072103381
AT5G13180	NAC083	0.964820444	0.000971402	1.287808939	2.58431E-20	1.08334476	1.6174E-17	0.495547102	0.644839236	-0.00727224	0.990464854	-0.189507215	0.21700465
AT5G65750	AT5G65750	0.472251426	0.153344869	1.256951761	4.90145E-11	1.083021667	2.11911E-07	0.024586835	0.981893161	-0.034558521	0.947746874	-0.06503669	0.775803854
AT3G09600	RVE8	0.729818054	0.21275708	1.471104644	1.7768E-05	1.076491625	0.003022128	0.867446592	0.62738272	0.064454431	0.929169201	-0.617612035	0.008234633
AT1G30400	ABCC1	0.814337588	0.00180968	1.087384293	6.85831E-06	1.069986954	4.10946E-19	-0.369488602	0.587695712	-0.248403288	0.207480361	-0.566885879	9.90224E-06
AT5G44250	AT5G44250	0.702881547	0.106033095	1.272490327	7.76737E-13	1.066790818	8.69426E-15	0.972075867	0.417321846	0.400794755	0.152264493	0.31893671	0.049428726
AT1G75490	AT1G75490	1.095056125	0.057436379	1.603099379	1.0701E-08	1.063964841	4.53385E-10	1.533070739	0.456578783	0.085482156	0.892516312	-0.493337678	0.020370299
AT3G01100	HYP1	0.70147924	0.132693606	1.174845649	0.00171685	1.063493878	3.47683E-05	-0.126234241	0.885844797	-0.428824088	0.119428864	-0.735121707	5.60193E-06
AT4G39140	AT4G39140	0.821748667	0.05562323	1.253902457	2.58439E-05	1.059970164	3.79551E-07	0.152822215	0.932158811	-0.223566258	0.579660469	-0.611083946	1.32585E-05
AT2G41410	AT2G41410	0.640089718	0.083366881	1.511125891	8.03434E-20	1.059574942	2.46279E-13	-0.05244435	0.963341405	0.042601518	0.916753332	0.226358918	0.141183893
AT2G01470	STL2P	0.853491675	0.008968369	1.110230772	5.35778E-07	1.055264233	6.78186E-09	0.380741283	0.704225272	0.217625051	0.481524151	-0.206221611	0.464264228
AT1G03760	AT1G03760	0.816824608	0.02022541	1.427040563	8.66161E-07	1.053657109	0.000478669	0.21787708	0.797097845	0.33093784	0.412920773	0.042678029	0.861911856
AT5G49700	AT5G49700	0.830924281	0.059807789	1.528365821	2.43221E-06	1.050567199	4.59441E-06	0.123407034	0.949913024	-0.048755798	0.924627441	-0.156966699	0.410396952
AT3G24740	AT3G24740	0.912705164	0.018114012	1.001989897	4.26473E-07	1.050113809	3.13527E-10	0.638863501	0.6657277	0.248872233	0.481806699	-0.205870615	0.475369379
AT1G53560	AT1G53560	0.908045088	0.051857091	1.720746689	1.18304E-09	1.048137384	1.90936E-15	0.108295431	0.960955304	0.235013956	0.53824515	-0.306852691	0.133898108
AT1G28190	AT1G28190	0.782521217	0.103309204	1.328399996	0.002823253	1.048040766	0.032375157	0.30597643	0.822720536	-0.021212352	0.983751188	-0.006488276	0.987386736
AT1G07310	AT1G07310	0.938658182	0.03322036	1.03858736	1.4722E-08	1.041307462	2.58188E-10	0.71483892	0.66808962	-0.316383035	0.256341356	-0.286395286	0.06875782
AT1G09570	PHYA	0.954003923	3.91155E-06	1.117393431	2.30821E-17	1.034222388	1.74364E-15	0.146883664	0.898229292	-0.045408241	0.89252962	-0.632704762	5.73507E-09
AT2G39725	AT2G39725	1.285292	0.065411814	1.465497023	0.003598728	1.033410176	0.028510817	0.96864846	0.155641191	0.626571661	0.292750658	0.332631863	0.440183822
AT3G53960	AT3G53960	-0.504193262	0.380672864	1.079301768	0.000516893	1.031582601	0.029742133	0.385599289	0.901814827	-0.024724079	0.990464854	-0.229312766	0.708566231
AT1G06110	SKIP16	0.910109401	0.003980013	1.473777436	7.79997E-14	1.030530778	3.36424E-12	0.32645372	0.782088381	-0.019002024	0.973023368	-0.012961517	0.948941317
AT4G21540	SPHK1	0.823594484	0.029683146	1.069846452	6.15112E-08	1.029612339	6.59814E-10	0.01632229	0.991559366	0.18812289	0.624678583	-0.419156552	0.012420732
AT1G54120	AT1G54120	0.961854676	0.233145719	1.992632653	0.002875573	1.029437702	0.001485715	-1.178607991	0.576334999	0.265176447	0.832357143	-0.782927635	0.004166186
AT4G15760	MO1	0.893112394	0.022773839	1.097000704	3.00176E-07	1.028151255	2.69143E-17	1.097524051	0.402802035	0.390807947	0.005266251	0.610288913	3.84995E-09
AT1G01830	AT1G01830	0.735004697	0.106575709	1.366240028	1.59851E-06	1.027752603	5.61142E-05	-0.161420671	0.89727291	0.124764723	0.796567249	-0.21408584	0.251086742
AT4G28300	AT4G28300	0.676075221	0.069910227	1.057578123	4.89525E-11	1.026768569	5.49916E-11	0.332526855	0.859820185	-0.282356261	0.239402781	-0.276905923	0.094783971
AT4G21650	AT4G21650	0.556698779	0.272643421	1.617869792	0.00162911	1.018048823	0.015222542	-0.476005661	0.715966217	-0.549925571	0.013046672	-0.140507022	0.443711591
AT5G09620	AT5G09620	0.460419539	0.127283479	1.188730504	1.73439E-07	1.015718034	1.60242E-06	-0.329081442	0.546625842	-0.356688656	0.189510493	-0.345376659	0.016021325
AT5G54170	AT5G54170	0.22756651	0.672712337	1.523132064	3.37492E-05	1.014994873	9.76026E-10	0.127108905	0.953045007	0.266543191	0.507785667	0.000725065	0.997496949
AT4G26700	FIM1	0.12359881	0.857108424	1.272536419	2.43266E-08	1.003156839	3.75987E-12	1.238810278	0.430181703	0.112301714	0.811038824	-0.481817593	0.000236175
AT4G10090	ELP6	0.398534147	0.423756745	1.111150601	0.007220795	1.000541022	0.002981826	0.578555318	0.429222482	0.201424704	0.727051106	0.479869651	0.074747804
AT3G07800	TK1a	-0.886528828	0.08276289	1.611280285	0.000333951	-1.035542863	0.04256837	0.067486514	0.980939287	-0.101435509	0.876703746	-0.341939808	0.096384573
AT5G47550	AT5G47550	-0.214511914	0.812676752	1.847192828	1.14204E-07	-1.19881893	2.7024E-05	-0.936273702	0.214402425	-0.027523416	0.97926944	0.46082502	0.170434184
AT5G23950	AT5G23950	1.398510098	0.647883951	4.540142496	0.023504186	-6.400841857	0.021819927	-0.780234243	0.908145137	-1.405787753	0.56843464	-2.80398148	0.115928794
AT3G05150	AT3G05150	-0.844647478	0.483630781	-2.442960496	0.001028158	2.396899866	9.02748E-26	-1.98991147	0.589531904	-0.621415345	0.072389536	0.036095813	0.910613018
AT3G30775	ERD5	-0.82663764	0.086847412	-1.573346511	8.7393E-14	1.213908854	7.08102E-15	2.525110833	0.100366568	0.118243539	0.802042097	0.167116539	0.358479862
AT4G13570	HTA4	-2.082606614	0.209746171	-1.523943913	0.013512337	1.027874602	6.4376E-05	3.331107377	0.50075057	2.953978425	0.770008565	-0.856244976	0.361165538
AT1G50490	UBC20	-0.877745969	0.335401492	-1.558756652	0.000429472	-1.002865393	0.0012309	-0.230801114	0.921812922	0.383610158	0.367098713	0.836983862	5.63447E-07
AT4G29590	AT4G29590	-0.954604399	0.172842592	-1.505527693	0.000174627	-1.007606718	0.000283869	-0.059047316	0.972495118	0.147803408	0.846410046	0.558774502	0.006499042
AT5G49100	AT5G49100	-0.44877868	0.540722705	-1.23686755	9.23184E-07	-1.009007293	8.93137E-05	-0.040256741	0.982034793	0.500189064	0.013187813	0.552376663	0.000388065
AT2G28740	HIS4	-0.516031767	0.472442053	-1.160650639	0.000532505	-1.010417244	8.80089E-05	-0.217425186	0.918501816	0.193914224	0.583032275	-0.32722365	0.298490444
AT2G43800	AT2G43800	-0.112188404	0.926534672	-1.201116368	0.020418625	-1.01477508	0.010062642	-0.148423474	0.93556951	-0.139117626	0.792838833	0.165554647	0.541541078
AT2G28930	PK1B	-0.590889979	0.137244516	-1.528062059	0.000135672	-1.017096228	0.000285099	0.165480194	0.90505206	0.092164694	0.864433396	0.680487921	2.00765E-05
AT4G28360	AT4G28360	-1.072393712	0.070370039	-1.107752016	0.000876922	-1.018625164	0.0002986	-0.550112141	0.6107328	0.047534091	0.934609223	0.261653216	0.097714154
AT5G48600	SMC3	-0.871608291	0.286054756	-1.331580817	0.006084945	-1.023457019	0.007437018	-0.103129365	0.980383411	-0.053815168	0.944784464	0.225158713	0.17960366
AT2G24230	AT2G24230	-0.576343256	0.320410658	-1.251029313	0.003124936	-1.02590925	0.006528013	-0.056502942	0.972495118	-0.251807098	0.482903102	-0.200123199	0.494943002
AT3G17680	AT3G17680	-1.374430568	0.092654226	-1.840475386	0.00311281	-1.028360592	0.012063415	0.315736071	0.776351562	0.669274336	0.159086213	0.99165506	1.61381E-09
AT4G13050	AT4G13050	-0.61600008	0.144980294	-1.212873688	0.00012938	-1.028851333	7.3703E-05	0.177773398	0.835453631	0.364083581	0.36328702	0.217435984	0.298101251
AT4G17870	PYR1	-0.931852506	0.021838706	-1.50567264	6.25014E-08	-1.036255561	2.65056E-06	-0.352411677	0.770575751	-0.316584296	0.287120998	-0.484621441	0.028542487
AT1G17140	ICR1	-0.562634509	0.372340116	-1.025343527	0.003640766	-1.037903156	1.70782E-05	-0.13771427	0.926124658	0.184913571	0.601982492	0.645763738	9.60164E-07
AT3G63160	F16M2_10	-0.777547559	0.165998737	-1.17757654	0.000990235	-1.041693847	2.63297E-07	0.53823546	0.700166133	-0.327122154	0.381166491	0.184588713	0.558442049
AT2G18290	APC10	-0.822402842	0.185119944	-1.036928186	0.00608194	-1.042029656	0.000344022	0.289568666	0.845932274	-0.046863564	0.958438477	0.085088388	0.807880563
AT3G45930	AT3G45930	-0.346510479	0.601270691	-1.690001794	0.000604426	-1.050683849	0.032938693	-0.152161035	0.963390011	0.193565898	0.589892257	-0.076399805	0.807195442
AT4G33740	AT4G33740	-0.690063118	0.015258129	-1.244179092	7.07692E-07	-1.056075901	0.00017154	-0.428765557	0.646729152	-0.299568685	0.162585503	-0.255424906	0.312482444
AT3G28910	MYB30	-0.690462163	0.104830481	-1.153323758	2.22316E-05	-1.056958591	2.01024E-07	-0.461998907	0.701052583	-0.154587642	0.568281463	0.610120167	2.4063E-07
AT2G42570	TBL39	0.435549294	0.73353643	-1.566371746	0.036365451	-1.058456298	0.022258297	-0.680921055	0.734168325	-0.167312728	0.579669854	-0.096272213	0.624818438

AT1G10020	AT1G10020	-0.975138819	0.004098912	-1.438665752	1.81106E-11	-1.061976835	0.003664258	-0.128927724	0.930511033	0.087087247	0.830448249	-0.215549724	0.196086927
AT5G40550	SGF29b	-0.819296598	0.182197195	-1.226350147	0.001464158	-1.063019357	0.016872126	-0.121823041	0.958708047	-0.037141419	0.970686809	0.21774759	0.385370312
AT1G77990	AST56	-0.259738027	0.866216826	-2.164309552	0.032782106	-1.064445426	0.044424686	0.520911869	0.726257149	-0.442226568	0.696800101	0.516123642	0.04785683
AT1G44900	MCM2	-1.812563252	0.074400955	-1.764411842	0.001713495	-1.064955015	0.023923423	-0.508354315	0.958708047	-0.035475814	0.962005746	0.026133939	0.945115939
AT3G57060	AT3G57060	-0.944974409	0.235075224	-1.228065147	0.009176318	-1.070863866	0.003488534	0.162108288	0.95641957	0.060793745	0.951101524	0.791605818	2.51912E-06
AT5G15530	BCCP2	-0.975531065	0.011966024	-1.528564136	0.001378611	-1.072380765	2.65597E-05	0.130387497	0.94654169	0.295941154	0.493875814	0.931834474	2.19027E-06
AT5G03150	JKD	-0.583088503	0.42087957	-2.103513514	1.94228E-08	-1.074302311	0.020398341	-0.902762377	0.565142454	-0.568322482	0.100750339	-0.009710847	0.969973433
AT2G38650	GAUT7	-0.928272023	0.060909348	-1.171149909	6.3023E-07	-1.082010217	2.64676E-06	-0.646357493	0.631570795	-0.299852738	0.186598083	-0.126899227	0.428228207
AT1G69620	RPL34	-0.688993335	0.11667035	-1.064077993	3.00582E-09	-1.087222093	4.08992E-26	-0.029291139	0.989622493	0.27455248	0.066439925	0.455319231	0.000128921
AT2G05990	MOD1	-0.933691007	0.000276753	-1.095122586	7.28696E-10	-1.08742581	1.24136E-10	0.125924193	0.914901791	0.092864677	0.770048135	0.659161485	1.79313E-09
AT2G40030	NRPD1B	-0.720131904	0.315360401	-1.125132654	0.003097333	-1.089613859	0.003178323	-0.196527082	0.894700903	0.131067128	0.835694557	0.346200682	0.087218715
AT1G62640	KAS III	-0.476476162	0.179787229	-1.140906948	0.000428315	-1.091796118	0.001008019	0.046822258	0.972495118	0.064115995	0.914915058	0.07980124	0.757730296
AT1G65010	AT1G65010	-1.011539963	0.124329249	-1.522643238	7.00705E-06	-1.095670853	1.29642E-07	-0.607370566	0.66808962	-0.019275856	0.966967951	-0.376155863	0.001823084
AT5G06670	AT5G06670	-0.978079111	0.068398694	-1.167672391	0.000251131	-1.099236113	5.12694E-06	0.68577816	0.625033288	0.239606924	0.654509491	0.671609797	0.001603001
AT2G30460	AT2G30460	-0.493510659	0.484464621	-1.87781249	0.002472936	-1.103636779	0.015216944	-0.274765554	0.83240074	-0.36276428	0.578542261	0.244867969	0.443613081
AT1G70895	CLE17	-0.801791287	0.182839027	-1.849554387	0.00089335	-1.10527392	0.048503125	0.626068782	0.587695712	-0.246567067	0.739714734	0.468932474	0.161455071
AT1G22540	AT1G22540	-1.135356585	0.081592393	-1.23332096	0.019909804	-1.118861604	0.000115014	-0.083521465	0.966099884	-0.089523075	0.930982464	-0.145337336	0.605306564
AT5G51670	AT5G51670	-0.732362778	0.181819993	-1.878385898	7.61212E-06	-1.122294529	0.000574558	-0.821508771	0.534010844	-0.48587347	0.215154282	-0.465020762	0.027488712
AT3G54750	AT3G54750	-0.978829801	0.171663106	-1.986928855	1.57633E-08	-1.122790847	4.04409E-05	-0.548397743	0.825077044	-0.18808055	0.684515288	0.298177823	0.105925735
AT1G76270	AT1G76270	-1.142577434	0.072584562	-1.876119378	0.026867137	-1.122872144	0.012994215	-0.469596712	0.846938555	0.179969863	0.74116076	-0.206770544	0.337602466
AT4G37100	AT4G37100	-0.435762797	0.524586619	-1.132956521	0.012576603	-1.126479612	0.012296727	0.066133555	0.971723999	-0.004673598	0.99569371	-0.439499969	0.022576481
AT3G16660	AT3G16660	-0.364832213	0.596029789	-1.276612525	0.043208593	-1.128543998	0.00018114	-0.626646573	0.733019473	0.101670659	0.95946592	-0.072881241	0.906321824
AT3G02580	STE1	-0.759028495	0.024333386	-1.031919698	0.002698824	-1.130069748	2.7358E-05	-0.358909292	0.732196157	-0.44833041	0.08213156	0.181699083	0.292262756
AT5G15580	LNG1	-0.842341505	0.256205051	-1.684486367	1.06304E-05	-1.136139252	0.003031734	-0.365568842	0.823579145	-0.101985233	0.813086742	0.204051799	0.355611518
AT3G18200	UMAMIT4	-1.539309318	0.166945185	-3.252994372	0.017569319	-1.136158205	0.043651712	0.119491986	0.972495118	-1.731035712	0.060428164	-0.680779322	0.115962117
AT4G34960	AT4G34960	-0.610756454	0.409098872	-1.29955805	0.003575109	-1.137907901	0.003918347	-0.401196333	0.820406788	-0.305333058	0.367749288	-0.04670069	0.857036312
AT3G51740	IMK2	-1.334058823	0.184758364	-2.153805023	9.16329E-11	-1.138945306	0.008194218	-0.228212865	0.909900566	-0.105273231	0.828846482	0.635203593	8.26621E-05
AT1G19950	HVA22H	-1.969615068	0.140891471	-1.909961203	0.018112863	-1.139047238	0.015060227	0.000425267	0.999867318	-0.414336461	0.518808901	0.457289064	0.162643972
AT5G16250	AT5G16250	-0.171232235	0.739098278	-1.262748339	3.76921E-05	-1.140674689	3.03212E-06	0.163022779	0.899162093	0.218972435	0.516305535	0.677678822	7.2872E-05
AT5G49760	AT5G49760	-0.971175966	0.000901994	-1.03761567	6.64467E-05	-1.141464455	1.09285E-12	-0.376309248	0.658641304	-0.480566761	0.006570732	-0.424804093	0.001346514
AT1G69780	ATHB13	-0.721907554	0.311850482	-1.964412725	9.02712E-06	-1.150402056	0.000142873	-0.404150663	0.720336606	-0.071606733	0.905535933	0.271174631	0.185306588
AT5G45670	AT5G45670	-0.706578183	0.706076738	-1.905384829	2.18835E-05	-1.150694406	0.000772343	-0.402521379	0.848749462	-0.103790286	0.812574037	0.608534631	0.000661994
AT1G20610	CYCB2;3	-0.883234909	0.331609471	-2.245048706	0.000164475	-1.156992669	0.005787988	-0.095659539	0.980608627	0.663612634	0.186167997	0.570943802	0.031196677
AT2G14890	AGP9	-0.857622941	0.106638633	-1.080869545	8.1516E-06	-1.161481516	2.68846E-08	-1.240443387	0.340427521	-0.694974411	5.47786E-05	0.142668692	0.543572432
AT5G50420	AT5G50420	-1.127500266	0.069352794	-1.229623478	0.012355388	-1.166705624	0.000307565	0.173989796	0.932578056	-0.201427575	0.72293719	0.243920573	0.235282223
AT2G23360	AT2G23360	-0.761600119	0.3049287	-1.297575184	0.002436389	-1.17085721	0.015734256	-0.381654218	0.842936823	-0.021599118	0.982621649	0.385055997	0.064094563
AT3G52640	AT3G52640	-0.909708438	0.012513155	-1.162432438	3.19979E-05	-1.172815973	3.98842E-05	-0.276292536	0.888092418	-0.393325149	0.227867455	0.042987154	0.878292965
AT3G01410	AT3G01410	-0.594484941	0.563683014	-2.595708153	0.000496142	-1.176622837	0.021051208	-0.016381009	0.996799452	0.258448499	0.697450631	0.20003	0.513146642
AT4G26760	MAP65-2	-0.933336551	0.301063915	-1.979036804	0.00098922	-1.1767189	3.54408E-05	-0.09887164	0.968587518	0.157683316	0.717563574	0.48206871	7.70382E-05
AT5G01890	AT5G01890	-0.936367488	0.304415798	-2.040093452	0.038070347	-1.176954484	7.60284E-05	-0.443024849	0.840363187	-0.482583149	0.085137231	-0.200529999	0.466586493
AT2G32590	EMB2795	-1.349543397	0.094731618	-3.10134568	0.000306931	-1.178649615	0.01569751	0.018979536	0.995786197	0.490982988	0.405629162	0.505488216	0.026058564
AT5G59770	AT5G59770	-0.933418236	0.058946429	-1.035138502	0.000283217	-1.178916467	0.003482415	0.644226372	0.583897164	0.24685969	0.666423542	0.504049739	0.121214667
AT1G14350	FLP	-0.130721643	0.858645958	-1.340276354	0.001895803	-1.180911192	9.6716E-05	-0.282544925	0.893088744	-0.224462057	0.409507617	0.064781851	0.713190307
AT3G08680	AT3G08680	-1.297474008	0.127195199	-1.424726017	3.53744E-08	-1.182765665	6.44006E-07	-0.668381769	0.657881954	-0.260183135	0.40839213	0.036675463	0.888816482
AT5G49215	AT5G49215	-1.0279781	0.308951157	-2.18376777	0.000221432	-1.186704291	0.015810553	-0.627025552	0.769724647	-0.50296723	0.298538335	-0.364534994	0.261340981
AT1G18840	IQD30	-0.913323169	0.126619013	-1.278323274	2.67154E-05	-1.187942997	1.32926E-05	-0.254182492	0.841532767	0.115305973	0.742744666	0.378259995	0.007598725
AT1G75820	CLV1	-0.94006078	0.015005714	-1.436869292	1.87578E-08	-1.197193903	2.56962E-05	0.330655683	0.706790319	-0.425447512	0.06120581	0.073942887	0.794715558
AT1G45130	BGAL5	-0.451406074	0.609112165	-1.101031538	0.037960836	-1.197941158	0.003215883	-1.25719239	0.46594725	-0.633288601	1.79961E-05	-0.393852803	0.001045333
AT1G55120	FRUCT5	-0.765881195	0.348437987	-2.487007752	0.035343212	-1.200083362	0.005217861	-0.670354676	0.708039647	-0.127713744	0.87272758	0.261978702	0.277599885
AT3G22790	NET1A	-0.307822412	0.81757665	-2.130309861	5.41935E-05	-1.203281374	4.21254E-05	-0.229451816	0.936758504	0.467374039	0.182807281	0.203222218	0.362392983
AT1G13170	ORP1D	-1.305292874	0.077940574	-1.89381623	0.004522795	-1.208130977	9.57312E-06	-0.281614652	0.905742158	0.094451511	0.846410046	0.045251323	0.860866297
AT1G61795	AT1G61795	-2.137101227	0.083859782	-3.34158743	0.009761567	-1.213678166	0.024002671	-0.54420602	0.804495913	-0.80623119	0.438575468	-0.855099076	0.015299145
AT3G54560	HTA11	-0.479209259	0.643688114	-1.799423592	0.001682246	-1.21436762	5.69122E-06	0.130040275	0.965260868	0.588564177	0.001299644	0.535154426	0.002239737
AT3G08600	AT3G08600	-1.102243876	0.080575638	-1.321964309	0.016159067	-1.21961864	0.004148465	0.585071163	0.672844426	-0.067394014	0.936190346	0.034646294	0.909844977
AT5G58480	AT5G58480	-1.385591635	0.085973022	-1.306850026	9.66209E-06	-1.225229431	0.0001236	-0.024274996	0.990938961	-0.411236377	0.379588207	0.665503187	0.004143073
AT1G20950	AT1G20950	-0.725143853	0.34242021	-1.271221765	3.74012E-06	-1.228691623	7.88316E-09	-0.575815872	0.719664849	-0.13848305	0.614010445	0.381545907	0.006581009
AT1G53040	AT1G53040	-0.063102479	0.957277161	-1.818036357	0.022928129	-1.230900905	0.011015972	-0.596232102	0.692108738	-0.051871803	0.93404597	0.206577973	0.259090882
AT5G40060	AT5G40060	-0.743471821	0.217388605	-1.542451642	0.00049823	-1.230947497	0.00256221	0.427019134	0.872669065	0.221428391	0.888052136	0.197655186	0.711493724
AT5G65310	HB5	-0.527844129	0.302007273	-1.180840947	0.000381006	-1.237753098	3.05814E-07	-0.334465017	0.799598167	-0.078258191	0.812921184	-0.029484008	0.866600625
AT1G05590	HEXO2	-0.522716973	0.378499936	-1.479671347	0.00079621	-1.238861864	0.005593393	-0.357047787	0.672844426	-0.374559577	0.429416497	-0.100709253	0.701958746
AT2G26695	AT2G26695	-0.909924923	0.065119476	-2.101658569	0.001348816	-1.243119991	1.26467E-05	0.659795886	0.667958595	0.267501491	0.672727761	0.039395354	0.948941317
AT1G33040	NACA5	-0.865252221	0.161690492	-1.25902295	0.002116288	-1.249076038	1.79851E-09	-0.546090453	0.681538103	-0.02198008	0.971032958	0.229904113	0.2178367
AT3G62020	GLP10	-0.997462324	0.249704965	-2.309593904	0.004721072	-1.251407259	0.02912562	-0.490618876	0.855010607	-0.559617968	0.200290659	-0.362170398	0.205096434
AT3G26700	AT3G26700	-1.021950503	0.231987802	-1.157846729	0.038455476	-1.25531669	0.001245197	-0.474832357	0.769907555	0.232071224	0.614063594	0.282662625	0.182249958
AT1G76540	CDKB2;1	-1.329703782	0.123868295	-1.784904203	0.029294239	-1.257336685	0.004244714	-0.346384875	0.871661081	0.060233653	0.923989605	0.512639506	0.00022742
AT1G08960	CAX11	-0.738778344	0.280735082	-1.73301161	0.012931505	-1.257436524	0.003409102	-0.673009328	0.559215018	-0.543149146	0.208074892	-0.486951928	0.076117776
AT2G41820	AT2G41820	-0.544642287	0.626859735	-1.716398662	3.96083E-05	-1.259652693	2.89011E-05	-0.704116342	0.665778009	-0.209207489	0.592718867	-0.351275508	0.010488382
AT5G51560	AT5G51560	-0.64983419	0.367054299	-1.923256361	0.000580316	-1.272771218	1.25231E-05	-0.167465895	0.906862455	-0.12906415	0.789832518	0.41529082	0.055066583
AT5G07580	AT5G07580	-0.816186992	0.077133387	-1.542538467	1.1307E-06	-1.27883788	8.31249E-08	0.064490362	0.963787534	-0.025990393	0.969433294	-0.777093895	4.43896E-05
AT3G44790	AT3G44790	-2.891381527	0.641966396	-7.323148264	0.012455414	-1.284369444	9.49755E-07	-2.366342373	0.790112221	0.284150521	0.924557924	-0.069774551	0.863344122
AT1G31710	AT1G31710	-0.268279962	0.690258214	-1.195493019	0.011159316	-1.286386194	0.016066154	-0.498278518	0.723070421	-0.302834133	0.391809138	-0.18400262	0.500058566
AT3G01860	AT3G01860	-0.819996054	0.168263822	-1.434763478	0.036960993	-1.296283663	0.001306133	0.075405744	0.972495118	-0.93379785	0.274020042	-0.13485072	0.673745505
AT2G02790	IQD29	-0.450805899	0.474402926	-1.080646609	0.025310114	-1.304247637	0.000517023	-0.272915556	0.748179179	0.213107351	0.581594843	0.088566269	0.710965578
AT5G48450	sks3	-0.676617003	0.419613061	-1.136876436	0.026434969	-1.30802906	0.011182785	-0.534436673	0.797657205	-0.355495667	0.323767852	-0.175429076	0.543905909
AT1G51060	HTA10	-0.707743719	0.25070383	-1.349670941	0.000941087	-1.309674426	1.74315E-07	-0.443826456	0.805348024	0.056719777	0.885377618	0.125898378	0.555735164
AT1G68520	BBX14	-0.995164924	0.00728243	-1.290826108	5.9556E-07	-1.312132287	7.98011E-21	0.589510011	0.627094298	0.216389691	0.550968731	-0.591854004	0.011256385
AT1G73020	AT1G73020	-0.84708581	0.248571516	-1.112055203	0.00477494	-1.31243595	0.000230866	-0.200032315	0.810371664	0.135866194	0.798470713	0.373462963	0.085061081
AT5G11510	MYB3R-4	-0.738565384	0.504546049	-1.741139754	0.041373204	-1.31420695	0.002043903	-0.491866564	0.828028997	-0.153401213	0.838103967	0.429084196	0.047775674
AT4G32710	PERK14	-1.307116643	0.097981288	-1.690868187	0.018284	-1.322960003	0.000168474	-0.638578705	0.711255369	-0.210711999	0.69110225	0.522931542	0.035323009
AT4G30560	CNGC9	-0.719341228	0.549053496	-2.833726506	0.046364941	-1.324889438	0.007539875	-0.411176756	0.72131695	-0.292017484	0.742744666	0.17726928	0.620437128
AT4G30340	DGK7	-0.720013407	0.270083636	-1.209482774	0.000704537	-1.329630658	0.000566588	-0.390393725	0.757212383	-0.258822744	0.576874111	-0.235284554	0.300681192
AT2G38580	AT2G38580	-0.804337137	0.372397395	-1.225595907	0.001521346	-1.331934402	0.000890646	-0.376364339	0.878770658	-0.143513207	0.762086306	0.346758279	0.076911776
AT4G11450	AT4G11450	-0.875487768	0.240747498	-1.600767879	0.003852101	-1.334884921	0.033871527	-0.179277187	0.953045007	0.369587706	0.485857955	0.363556237	0.109134612
AT5G06390	FLA17	-1.0443056	0.087532823	-1.38784383	3.21611E-07	-1.34222505	1.35533E-09	-0.736234085	0.602119156	-0.52126566	0.083096348	-0.175447129	0.208623519
AT3G05330	ATN	-1.0970126	0.105474232	-2.015790186	0.005308292	-1.347656176	0.010322757	0.182134629	0.953923605	0.036959272	0.968719007	0.606134535	0.001301164
AT1G75780	TUB1	-0.210765822	0.916975907	-2.055905204	0.041549762	-1.351621877	0.002098628	-1.490422657	0.522087553	-0.273937755	0.406394097	0.968111857	2.86762E-13
AT3G14310	PME3	-0.789470002	0.218927493	-1.995462945	1.69684E-07	-1.354750308	2.89261E-07	-0.753489209	0.647947756	-0.859764254	6.74989E-11	-0.145609501	0.326395605
AT5G61130	PDCB1	-0.969962199	0.161609117	-1.842279794	1.52008E-08	-1.357659319	1.30337E-09	-0.166422084	0.924835736	-0.256891607	0.475581847	0.34148615	0.141255714
AT1G02730	CSLD5	-0.871000343	NA	-2.640088214	1.4112E-10	-1.36150132	0.001410287	-0.286603523	0.867112468	-0.092314595	0.847697457	0.516983038	0.001406328
AT4G36180	AT4G36180	-0.167482188	0.903324611	-1.389902655	0.000602379	-1.364521901	6.80532E-05	-0.356580708	0.855427008	-0.256109691	0.459623683	-0.013891258	0.963912358
AT4G21270	ATK1	-1.314016143	0.195511078	-3.269221116	9.27357E-14	-1.368212527	0.007036453	-0.484926008	0.77715426	0.027961404	0.97926944	0.795424083	1.19718E-05
AT1G22950	AT1G22950	-1.014370102	0.131235497	-1.622427719	0.000779834	-1.385201768	0.002283941	-0.433991351	0.79501376	0.108774275	0.862023974	0.070213583	0.77280831
AT3G63200	PLP9	-0.750515426	0.38452737	-1.623652266	7.31983E-06	-1.387282762	4.27764E-07	-0.460823683	0.774776885	-0.155969987	0.691184156	-0.136265756	0.578666484
AT4G38660	AT4G38660	-0.464782294	0.622496634	-1.692118072	0.000855363	-1.390005082	5.33766E-06	-0.515495025	0.864304727	-0.06932033	0.899553662	0.472911489	0.006297976

AT4G00950	MEE47	-0.86779515	0.064421615	-1.20394354	0.000226401	-1.395008371	5.63788E-05	0.479896005	0.678599943	0.451590842	0.313339304	0.072230478	0.818734747
AT2G47500	AT2G47500	-0.958745379	0.08072709	-1.677337507	8.01038E-08	-1.401945078	6.2101E-07	-0.3359319	0.851457903	-0.141987216	0.79023526	0.535980209	0.000118928
AT3G19050	POK2	-0.654794693	NA	-2.995276215	4.43769E-07	-1.40847841	0.000133664	0.192441952	0.941415027	0.018413722	0.987053629	0.30793663	0.155017462
AT2G20635	AT2G20635	-1.241473445	0.099441456	-2.475424689	1.48525E-07	-1.408849419	0.00376441	0.228541016	0.915353837	0.564255722	0.259031822	0.720153899	0.00029222
AT1G14180	AT1G14180	-1.315116648	0.083563185	-1.907757538	0.000314672	-1.410732106	0.012224123	0.184205887	0.936758504	-0.006182737	0.996256673	0.503317205	0.028221162
AT5G16240	AT5G16240	-0.921735068	0.11225395	-1.391559488	0.000296734	-1.410947788	2.90317E-07	1.269370083	0.147390465	0.089070467	0.939586734	-0.479003531	0.313842725
AT3G46320	AT3G46320	-0.67676663	0.469810481	-1.24765074	0.007726724	-1.41885396	7.41092E-06	-0.529833682	0.742454197	0.101783786	0.860971852	-0.049783972	0.867251916
AT5G67200	AT5G67200	-0.127420409	0.924232001	-1.497380924	0.003495628	-1.435855281	0.005121016	-0.213649535	0.947834751	-0.134638929	0.834816971	-0.138893822	0.65369276
AT2G29050	RBL1	-1.346796933	0.151048952	-1.554381929	9.80092E-05	-1.440499308	3.417E-07	-0.294496884	0.918652486	0.404840773	0.198671181	0.582178003	0.000158241
AT3G02030	AT3G02030	-1.041807821	0.214253351	-1.833493236	0.019066005	-1.444286314	0.006128853	-0.13174353	0.972077881	-0.102937579	0.947624125	0.436532475	0.21029417
AT2G24630	ATCSLC08	-1.215117616	0.065228916	-1.671559069	0.001063485	-1.444382359	1.69215E-05	0.140400523	0.971773393	0.20667038	0.702914033	0.558430483	0.025948437
AT2G30200	EMB3147	-0.720956127	0.031600709	-1.144023257	3.3218E-05	-1.444741059	4.58248E-09	-0.003857864	0.99779774	0.029051932	0.954083501	0.516082342	0.000124845
AT1G04030	AT1G04030	-0.761068972	0.462303741	-1.570569828	0.019459325	-1.446846086	0.037541991	-1.188488702	0.615358268	-0.11500308	0.897051319	0.196919822	0.61794044
AT2G28620	AT2G28620	-1.172830055	0.396385473	-2.762721875	2.6165E-07	-1.451955272	0.009835535	-0.658668588	0.812877249	0.006578862	0.994307202	0.545121868	0.002831605
AT2G39180	CCR2	-1.137065672	0.080091734	-1.676000251	0.016838554	-1.462238885	0.012331977	-0.463149101	0.830658776	-0.376712216	0.505696968	0.208299877	0.522186053
AT5G17660	AT5G17660	-1.252741809	0.100213718	-1.183517921	0.004697045	-1.466678635	0.003633879	-0.328771449	0.871661081	0.28176561	0.673806122	0.555969382	0.014734802
AT5G62580	AT5G62580	-1.293687219	0.079957642	-2.023613164	1.36652E-07	-1.474498099	0.000134862	-0.518179106	0.753910599	-0.105393969	0.809573476	0.194334241	0.378974515
AT1G04020	BARD1	-1.208810508	0.326223499	-2.006450857	0.012623521	-1.482570653	0.002719261	-0.393953646	0.899163675	-0.274411945	0.669993341	0.196739946	0.506283058
AT2G16400	BLH7	-0.567116931	0.177315272	-1.136490801	3.38309E-05	-1.485024918	5.57386E-10	-0.401899333	0.751308246	0.014476744	0.982621649	0.344079733	0.027544849
AT5G59650	AT5G59650	-1.935184331	0.050106994	-3.783339289	0.036537141	-1.485688524	0.005026733	0.658556836	0.911737208	-0.190498973	0.917626906	0.361454862	0.389784048
AT3G13520	AGP12	-0.885102921	0.065023193	-1.956161722	2.15288E-11	-1.493342664	5.00368E-13	-0.868470483	0.602119156	-0.332554141	0.088460217	-0.142856557	0.369323178
AT1G72250	AT1G72250	-1.612714043	0.059279264	-2.299347398	0.014908792	-1.506402663	0.000122564	0.132996011	0.952966451	-0.041069493	0.96180004	0.60701574	0.000511225
AT3G06740	GATA15	-0.985343997	0.166509647	-1.126342448	0.04797952	-1.513158034	0.001296249	-0.419978111	0.859699136	-0.083498213	0.922486937	0.129030881	0.648811573
AT5G54590	CRLK1	-1.102246384	0.418331474	-1.845596707	0.000533752	-1.514119583	0.01290251	-0.056159259	0.987671055	0.15137324	0.871587976	0.232105008	0.439215612
AT5G67390	AT5G67390	-0.501728129	0.513515802	-1.320970835	0.044923287	-1.520116575	0.000111667	-0.958227244	0.66524984	-0.343742845	0.41344799	-0.508930184	0.023373811
AT5G56040	AT5G56040	-0.735606777	0.379704967	-1.173561709	0.001659914	-1.520454071	4.04838E-07	-0.755967415	0.604422229	-0.193931456	0.614546122	-0.5849088	0.001175997
AT3G28760	AT3G28760	-1.504988187	0.162319283	-1.825916164	0.012794177	-1.52710821	1.20061E-06	-0.196513996	0.953774208	0.87655311	0.085181279	0.93566853	5.36205E-06
AT3G03130	AT3G03130	-0.560007042	0.782072629	-2.534477392	0.000211262	-1.527464918	0.016991388	0.44147775	0.947241707	0.491045564	0.284218275	0.496980252	0.005349042
AT3G15550	AT3G15550	-1.387892303	0.200462829	-3.239852462	4.88388E-09	-1.543917713	0.024791923	-0.001930818	0.999785291	-0.184734321	0.851227968	0.712862494	0.001872633
AT5G18080	SAUR24	-0.768112753	0.287242671	-2.018103803	0.012597465	-1.544770245	0.000875152	-0.17957334	0.951485287	0.108182431	0.933194561	0.176537749	0.636231621
AT3G61610	AT3G61610	-1.14634307	0.173550145	-1.78293584	8.3198E-05	-1.547859515	1.44573E-05	-0.493404039	0.738198292	0.253587904	0.550184567	0.642839343	0.000245546
AT4G17090	CT-BMY	-0.822926282	0.176316066	-1.008359779	0.04380177	-1.558140407	4.10576E-11	0.675378194	0.847168445	-0.293797023	0.738511482	0.812248959	5.92239E-05
AT1G05950	AT1G05950	-1.244613998	0.172234464	-2.75906965	0.007529433	-1.558745329	0.002677231	-0.078540153	0.982183273	-0.144986437	0.856890412	0.182266093	0.619103946
AT5G22880	HTB2	-0.407179394	0.558124923	-1.34612904	1.18398E-10	-1.56247071	8.41431E-20	-0.159181315	0.949241707	0.202690896	0.447139821	0.116410756	0.690632579
AT1G26945	KDR	-1.118132389	0.159533299	-2.388766749	1.07978E-05	-1.578839238	0.00109545	-1.087882479	0.647947756	-0.517450069	0.050846981	-0.381130561	0.02773602
AT3G46940	DUT1	-1.458451173	0.172864909	-1.493991395	0.016613033	-1.581327915	0.00323148	-0.124374636	0.990915497	-0.353622608	0.38407314	-0.539560139	0.016033777
AT1G69770	CMT3	-1.724275869	0.393526939	-2.15611659	1.24267E-06	-1.583473843	5.91411E-06	-0.213637579	0.9487076	0.133983073	0.828846482	0.474188969	0.003390978
AT4G08685	SAH7	-0.913491614	0.182683492	-1.782663698	0.001121151	-1.589979119	5.04394E-17	-0.608467081	0.662219342	-0.208027879	0.428068121	0.282372565	0.248496009
AT4G00490	BAM2	-0.893057881	0.217388605	-1.179686764	0.048885429	-1.597782004	0.000710039	-0.321013447	0.870136387	0.069314077	0.929134363	0.585274613	0.001591961
AT1G52750	AT1G52750	-1.010049545	0.335487231	-1.984676208	0.003153455	-1.605144824	0.006896857	-1.108743692	0.616300683	-0.656638561	0.187081257	-0.895012651	0.000554185
AT3G04690	ANX1	-0.884742783	0.353350645	-1.653592474	0.0361089	-1.618372916	0.007723954	0.486143982	0.696184854	0.287298767	0.723940828	0.035058789	0.945848985
AT5G10310	AT5G10310	-1.576570135	0.095674039	-4.028590221	0.004919011	-1.618545685	0.008958328	0.592633622	0.66524984	0.42696112	0.782635294	0.556316267	0.160493733
AT4G29730	NFC5	-0.4991658	0.649986738	-1.725544616	0.01101455	-1.623587283	0.002441562	0.078643225	0.981525175	0.221906846	0.81403589	0.513857057	0.058082881
AT4G01730	AT4G01730	-1.487040067	0.163075202	-2.811063965	0.000136649	-1.624892788	0.036471051	-0.24847212	0.971795498	0.320410368	0.593691419	0.555905062	0.035827937
AT5G39990	AT5G39990	-0.335537186	0.741283947	-1.018625144	0.026820633	-1.627646979	0.00016994	-0.922900899	0.576142947	-0.308350924	0.318968615	-0.041831526	0.875058094
AT4G31840	ENODL15	-0.807514734	0.680492892	-3.504514144	8.75682E-05	-1.635039829	3.54142E-05	-0.1700337	0.961727612	0.119704319	0.838286194	0.474999229	0.000690776
AT5G47100	CBL9	-0.20157492	0.783008708	-1.202301386	0.025349047	-1.635252671	0.001038073	0.222438194	0.872721351	0.204292173	0.780461868	-0.225855877	0.440466791
AT5G37000	AT5G37000	-0.007100663	0.997444393	-2.100400764	0.037138653	-1.636609696	0.000390714	-0.191159406	0.962872831	0.310435018	0.605282789	0.26281929	0.272119166
AT4G20940	GHR1	-0.700546305	0.463149685	-1.619211952	0.00069576	-1.637687789	0.000697932	-0.00042682	0.999867318	0.028479463	0.976044889	0.541799473	0.019122187
AT3G09035	AT3G09035	-0.460711157	0.422805576	-1.009624432	0.009533145	-1.643505364	3.2284E-07	-0.065068898	0.981414167	0.417260771	0.29658389	-0.146300059	0.616969754
AT3G23890	TOPII	-1.235093889	NA	-3.131789197	6.72983E-07	-1.645098084	8.79818E-11	-0.190705272	0.95660961	0.321871371	0.771376139	0.48189119	0.00228511
AT5G60930	AT5G60930	-1.257667946	0.17640558	-4.087809246	1.62818E-08	-1.655647802	0.001006267	-0.175176356	0.951502645	0.017687405	0.986774932	0.480717904	0.00520383
AT1G16020	AT1G16020	-0.825440398	0.173089986	-2.047867071	8.21761E-05	-1.662983844	9.79698E-08	-0.157969811	0.886829223	-0.251946792	0.584384403	0.089198043	0.711887158
AT2G06925	PLA2-ALPHA	-1.262225285	0.073452782	-1.377973044	0.006948826	-1.665241533	3.67026E-11	0.052085231	0.987576531	0.014750416	0.988021272	0.758074488	0.000222376
AT3G18730	TSK	-1.989218508	0.095334451	-2.443413169	0.014086176	-1.671954636	0.015362703	-0.571818316	0.821433118	-0.188075567	0.866290971	0.007377048	0.987386736
AT1G47400	AT1G47400	-0.343004193	0.791145164	-2.923457477	0.045506528	-1.687062567	0.018197241	-0.808675126	0.887722484	-0.487941137	0.761114072	-2.92547213	0.107119446
AT3G13560	AT3G13560	-0.606092063	0.613750995	-2.51491796	5.86162E-08	-1.688120614	3.61826E-06	-0.669360975	0.786453057	-0.237566412	0.564980934	0.451746631	0.059688014
AT2G16270	AT2G16270	-0.803325627	0.417360595	-2.091346536	0.000890149	-1.689402967	0.000585716	-0.098496318	0.972495118	0.587626538	0.433732558	0.414135848	0.055107595
AT5G41620	AT5G41620	-0.602491537	0.527248615	-2.282748451	0.02833602	-1.691506381	0.015903513	-0.169694314	0.974758564	-0.144909234	0.875307769	-0.362668527	0.204708785
AT5G55730	FLA1	-1.36469744	0.091621201	-1.430235054	0.000411719	-1.692769339	2.46734E-05	-1.015969091	0.563758673	-0.464827271	0.02814195	0.33822206	0.026331823
AT2G22795	AT2G22795	-0.2311644	0.753506382	-1.231266406	0.000220891	-1.694721308	2.5004E-09	-0.396456388	0.780811436	-0.425740296	0.127559438	-0.495067634	0.373163475
AT2G28950	EXPA6	-1.550441145	0.248099299	-2.295932151	1.24507E-12	-1.704537353	2.37982E-27	-0.70842965	0.664635521	-0.136605469	0.529833936	0.160716538	0.294304175
AT2G22610	AT2G22610	-2.078728245	0.087103457	-2.319833377	0.003661641	-1.707644804	0.000241932	0.137627656	0.976183443	0.065896779	0.957133906	0.561405749	0.002921225
AT1G08560	SYP111	-0.446004995	0.670664124	-2.556073034	5.05073E-10	-1.711900725	1.05047E-07	-0.042898943	0.989481773	0.434807555	0.266001052	0.471629295	0.00973342
AT5G57210	AT5G57210	-0.957903612	0.039677552	-1.88546044	0.004482885	-1.723539166	2.6135E-06	-0.224271667	0.902565251	-0.545015608	0.18824835	0.168287997	0.531423074
AT3G58650	TRM7	-0.372289513	0.800627102	-4.056002283	0.000153457	-1.727642595	0.004019368	-0.1936246	0.958663864	0.027018395	0.984250213	0.386440133	0.079490366
AT1G04520	PDLP2	-0.666243008	NA	-2.102214954	0.001627663	-1.731648704	0.000538893	-0.206610364	0.982749202	-0.137377218	0.824058587	0.405146084	0.006789558
AT5G18050	SAUR22	-1.018923315	0.250896796	-1.697188396	0.007434056	-1.736536699	1.1061E-05	-0.009642097	0.997796217	-0.032987265	0.972994743	-0.281892655	0.311059226
AT1G17430	AT1G17430	-0.671018438	0.495059936	-2.897885094	2.38439E-06	-1.745574028	1.09034E-05	-0.752167662	0.636161844	-0.3756227	0.515629348	-0.841411917	5.77563E-05
AT5G20740	AT5G20740	-0.26231241	0.80715233	-1.696205749	0.000559528	-1.74805043	0.002783808	-0.420949632	0.823579145	-0.01541193	0.984765765	0.436838007	0.016138171
AT5G56120	AT5G56120	-0.595587713	0.697499986	-2.814143813	0.033668459	-1.751560552	0.000348086	-0.045690675	0.988429284	0.605804642	0.207883732	0.687780423	0.004040379
AT4G22010	sks4	-1.339518394	0.073565065	-2.025941043	4.49796E-06	-1.756508235	3.38618E-11	-1.174632908	0.598901706	-0.568949413	0.003310922	0.489207708	0.019815811
AT1G03780	TPX2	-1.73131379	0.062695378	-3.575172619	4.96588E-06	-1.771638743	0.017694123	-0.076540111	0.984525706	0.132696092	0.846389857	0.868376922	2.77719E-07
AT1G34355	PS1	-0.720486609	0.607742463	-1.543948524	0.004046668	-1.774623517	0.005055145	-0.071494052	0.989273278	0.388183018	0.53525964	0.746101567	0.002304825
AT4G23800	3xHMG-box2	-0.929912507	0.345026093	-2.823875358	1.25645E-06	-1.774747856	4.22039E-09	0.041238576	0.991271129	0.448226316	0.104573951	0.593058843	9.18964E-05
AT4G30400	AT4G30400	-0.925263522	0.308834148	-1.491055295	0.001031972	-1.795211194	0.005850955	-0.419027368	0.876044849	-0.027773025	0.976570624	-0.552533189	0.004153703
AT1G63360	AT1G63360	-0.828427871	0.187160903	-1.018855325	0.004060883	-1.798617326	5.05093E-05	0.447002069	0.789793891	-0.016555865	0.992618878	-0.103350374	0.939126879
AT3G45230	AT3G45230	-0.935920029	0.20778809	-1.885673086	0.000843283	-1.800381827	0.000859229	-0.363185309	0.837694719	-0.557017107	0.161600965	-0.242455594	0.317787061
AT4G00020	BRCA2(IV)	-1.639622948	0.108015949	-2.176957257	0.002153139	-1.807951017	0.03734734	-0.217634036	0.966811844	-0.507008988	0.324033632	0.456627673	0.022060739
AT5G65360	AT5G65360	-1.209394078	0.060170742	-1.313257184	4.79042E-06	-1.809739125	5.00017E-16	-0.473935766	0.801461921	-0.056260919	0.915825933	0.238151902	0.237293642
AT3G16180	AT3G16180	-0.310199434	0.754468472	-1.32070777	0.000681232	-1.821444975	9.57802E-13	-1.036970165	0.592967663	-0.215996298	0.494905358	0.280937467	0.022343777
AT2G32380	AT2G32380	0.342254586	0.797701755	-2.489157508	0.002072492	-1.827093974	0.000198196	-0.934459452	0.709203242	-0.256661384	0.62054369	0.015798884	0.9726666
AT2G40480	AT2G40480	-0.258166913	0.835640939	-1.200249471	0.026970707	-1.832838693	3.74356E-05	-0.341919998	0.810393493	-0.386005224	0.222566815	-0.050438081	0.835980131
AT3G46900	COPT2	-6.125442888	0.270207033	-7.597692847	0.002073393	-1.833502394	3.75119E-06	-3.56730759	0.195928186	-1.831381873	0.160762404	-0.700325613	0.305070989
AT1G36940	AT1G36940	-0.779493118	0.453562364	-2.571125916	2.35087E-06	-1.837708765	0.003054417	-0.433859086	0.794188368	-0.019487405	0.983466557	0.544578417	0.000688487
AT5G38060	AT5G38060	-0.783633997	0.286791515	-2.018497054	0.006522053	-1.838618608	2.26035E-05	-0.385207807	0.919561093	0.129742639	0.934596763	0.146753267	0.763355544
AT5G06150	CYC1BAT	-1.130653753	0.307682975	-4.403757738	0.019369782	-1.844906085	2.58327E-05	0.014796604	0.997449367	0.278084588	0.570610039	0.943569697	5.21624E-12
AT5G15510	AT5G15510	-0.673374265	NA	-2.346433944	0.025464517	-1.853760144	0.000952689	-0.324285304	0.898391389	0.410563171	0.373165367	0.619373675	0.001606983
AT2G37420	AT2G37420	-1.063296105	0.325140075	-3.366127715	0.00510724	-1.860825844	0.00243781	-0.227254427	0.947082547	0.371892897	0.515641671	0.739498617	0.000201173
AT1G52280	RABG3d	-0.939416108	0.161653538	-1.620331479	0.002460152	-1.868614965	2.58703E-06	-0.329933414	0.84545588	-0.185015136	0.610572948	0.072602862	0.758042057
AT5G59870	HTA6	-1.609209025	0.256265469	-2.139695928	3.14841E-07	-1.879558751	2.59523E-12	-0.47697207	0.836779419	0.200465615	0.50913003	0.583808858	2.16452E-05
AT5G45700	AT5G45700	-1.044791828	NA	-4.467592062	3.44009E-05	-1.888522805	0.000618996	-0.055415674	0.993804788	0.765146321	0.067774495	0.525493593	0.004555072
AT3G01710	AT3G01710	-1.076073839	0.597438265	-2.312374629	0.000212467	-1.898333175	0.000954967	0.582004075	0.82384916	0.254215716	0.728206184	0.904303256	1.46762E-05
AT5G53880	AT5G53880	-1.018869933	0.135997059	-2.229697578	0.00026954	-1.90098921	0.000286572	-0.144258433	0.963305035	-0.112605389	0.858766684	0.386557542	0.34939366

AT4G35880	AT4G35880	-1.189727297	0.310470189	-1.849395269	0.001792195	-1.933125946	0.000131172	-0.824713664	0.865681813	-0.220698926	0.677459535	0.267402549	0.207511099
AT5G46740	UBP21	-1.367998766	0.19230941	-1.24528961	0.019312065	-1.939387168	0.003694572	-0.847493066	0.640704059	-0.44524787	0.487040982	-0.108416317	0.790405508
AT4G37630	CYCD5;1	-1.190078896	0.178732993	-1.62234603	0.020842282	-1.95717518	6.42657E-05	-0.084484782	0.978043477	-0.304378317	0.712912545	0.455652705	0.166780406
AT5G64780	AT5G64780	-1.928947295	0.225850619	-1.49554695	0.001112903	-1.957943149	0.000377275	1.094244106	0.746429074	-0.981879634	0.561308113	-0.080247181	0.921488272
AT2G33400	AT2G33400	0.158891	0.892677274	-2.080117787	0.000228411	-1.965775888	0.009626511	0.6391216	0.644839236	0.222038012	0.734495346	0.566649299	0.001597213
AT3G11110	AT3G11110	-3.005900842	0.221422083	-2.430000755	1.35772E-06	-1.980119937	4.88532E-06	3.89649674	0.676109289	6.117326905	0.292619435	2.190562184	0.743686193
AT5G22940	F8H	-1.042225077	0.320426341	-2.805937545	0.000423728	-1.981249373	0.002382945	-0.497224163	0.872302914	0.232113348	0.625207384	0.366487537	0.11567147
AT4G36360	BGAL3	-1.273165305	0.057264369	-1.042082715	0.001518769	-1.987730569	2.14497E-11	-0.97237037	0.503058648	-0.460720307	0.002280808	0.117284462	0.548560383
AT1G21880	LYM1	-1.797089269	0.057779757	-2.61514508	0.000777432	-2.00021693	4.5951E-07	-0.782130012	0.875701693	-0.385370326	0.561308113	0.272187776	0.308241155
AT3G10310	AT3G10310	-0.45207033	0.792734374	-2.948623577	0.036511126	-2.002290451	0.006879988	-0.097871762	0.989882569	0.347909953	0.677459535	0.587734354	0.007773479
AT2G25060	ENODL14	-0.724732766	NA	-2.505754814	1.24254E-08	-2.004257923	6.83056E-09	-0.165346885	0.966973374	0.132848695	0.712261785	0.620339452	7.4868E-08
AT5G66330	AT5G66330	-0.961078217	0.673617969	-3.318184763	0.014480757	-2.019071079	0.00016994	-0.973199716	0.718595611	-0.754344433	0.295568883	0.050236211	0.912396184
AT5G62550	AT5G62550	-1.096925752	0.257815951	-2.312721867	9.86278E-06	-2.020435776	2.88064E-06	-0.348116542	0.899858481	-0.015960825	0.983764323	0.495370439	0.024737911
AT5G65683	WAVH2	-0.777627347	0.466570968	-2.815350286	1.49452E-09	-2.023976443	0.001491737	-0.382041662	0.871383083	-0.613435132	0.063472951	-0.01770399	0.954012675
AT4G02800	AT4G02800	-1.016737623	NA	-3.166858724	8.29947E-05	-2.03282141	4.96852E-06	-0.470884211	0.809502311	0.340212441	0.496377799	0.614913041	0.001135626
AT3G12685	AT3G12685	-1.007265664	0.231196557	-1.235017688	0.03403659	-2.037113952	0.008727142	0.023464463	0.992699161	0.505427318	0.588325617	0.842775404	0.014422586
AT3G48160	DEL1	-0.239146097	0.89724489	-2.213525676	0.012976107	-2.039473625	0.014199684	-0.263967848	0.969967156	-0.350389571	0.747270887	0.160647238	0.708238201
AT3G55660	ROPGEF6	-0.52407246	0.709562348	-3.723177648	0.001346557	-2.039478773	0.037253846	-0.39941961	0.889161775	0.358775704	0.616392696	0.996888238	2.4297E-07
AT5G14610	AT5G14610	-0.961289869	0.051595571	-1.321284808	4.92625E-05	-2.048037488	9.75437E-05	0.266736853	0.855081587	-0.053501494	0.946158029	0.50915649	0.002543324
AT1G52000	AT1G52000	-0.125893878	0.939161007	-2.112118046	6.67866E-08	-2.053974867	4.15981E-21	0.434612103	0.847546649	0.644405915	0.050513286	0.831169288	3.10667E-09
AT1G23790	AT1G23790	-0.581679603	NA	-4.203178836	0.005584079	-2.074652289	0.003085456	-0.040464343	0.993737713	-0.401892821	0.638845866	0.823543779	0.001051102
AT5G48320	AT5G48320	-1.062802868	0.258247437	-1.624508946	0.043870962	-2.079708958	0.003215816	1.534230652	0.563804992	-1.22238858	0.363849408	0.905927992	0.047689234
AT1G54040	ESP	-1.800915728	0.174740908	-3.752113804	0.001329903	-2.087991511	2.70591E-21	-4.61294846	0.138904674	-0.577863062	NA	0.295422127	0.12591397
AT5G50740	AT5G50740	-1.36118295	NA	-4.653283506	5.3704E-06	-2.116714475	9.62192E-05	0.340586114	0.944029748	0.634972439	0.17086402	0.369495319	0.313009643
AT3G25980	MAD2	-0.550417072	0.621976105	-4.086849252	7.57284E-05	-2.123130294	4.34593E-06	-0.192837407	0.93403749	0.302317955	0.515629348	0.593872395	0.001386669
AT3G49750	RLP44	-0.745701067	0.527728649	-1.968417501	0.032789241	-2.125374418	9.15366E-05	-0.603976556	0.917312631	-0.034041286	0.980663768	-0.400117729	0.088431796
AT2G35190	NPSN11	-0.857390074	0.67250724	-1.663527374	0.003293721	-2.138560629	3.35725E-09	-0.863802252	0.680482607	-0.092240565	0.820892331	0.347535995	0.015790934
AT1G04250	AXR3	-0.279803337	0.761295964	-1.467634621	0.000738414	-2.155721662	4.39982E-07	-0.842936333	0.661895574	-0.521063095	0.002028309	-0.919450428	6.53989E-09
AT3G17360	POK1	-0.961285927	0.422626273	-1.834577459	4.27494E-05	-2.218407456	0.000222019	0.164728984	0.981893161	0.183425357	0.788769673	0.476851788	0.025646196
AT1G63300	AT1G63300	-1.060115343	0.109271669	-1.701517756	0.003556309	-2.221882202	8.31156E-11	-0.563472645	0.676109289	0.090671956	0.922870616	0.130406681	0.645082103
AT1G47210	CYCA3;2	-0.666259775	0.509228614	-1.599634738	0.001506372	-2.226549008	1.27526E-06	-0.459619956	0.753381979	-0.231124061	0.592718867	0.287392884	0.082076777
AT3G02120	AT3G02120	-1.220775237	0.233657367	-3.482451045	8.60173E-11	-2.227740665	5.33766E-06	-0.068916657	0.985484378	0.160873545	0.799785412	0.45392771	0.022060739
AT5G55520	AT5G55520	-1.123564271	NA	-3.018917004	0.020241869	-2.22888624	7.10735E-07	0.299622424	0.894945882	0.148750756	0.891818582	0.687367278	0.000425213
AT3G50890	HB28	-0.719678663	0.589663303	-10.16993929	4.29281E-12	-2.239520132	0.000515025	-0.233208785	0.968655039	0.067564116	0.927107216	0.358274594	0.039097847
AT5G66230	AT5G66230	-0.780269774	0.556768109	-3.97140967	0.035485856	-2.240999521	8.95963E-09	-0.367444207	0.896533613	0.176372378	0.737773763	0.96202502	1.22399E-06
AT3G12610	DRT100	-0.456208107	0.798734135	-1.237405529	0.019084357	-2.249019434	9.96839E-31	-1.230573143	0.594074706	-0.50991193	0.003251814	0.021987926	0.931594029
AT3G61820	AT3G61820	-0.671527384	0.29793529	-1.077911252	0.018952989	-2.261491312	5.32525E-06	-0.682904521	0.692108738	-0.510746157	0.066481086	-0.267911091	0.337812898
AT1G11545	XTH8	-0.858125542	0.652187962	-1.592688633	0.000299991	-2.274127974	0.000847885	-1.50377776	0.628417613	-0.491149902	0.015028896	0.365804417	0.009321332
AT4G15830	AT4G15830	-1.043327026	0.331158724	-3.063873479	0.015974145	-2.277925584	3.87947E-05	-0.074503457	0.983305599	0.307912058	0.565321135	0.538556697	0.000709346
AT4G34060	DML3	-1.388588506	0.621591774	-7.382213777	0.038977265	-2.283097173	0.014785954	-1.135391015	0.909120533	0.0247844	0.998079347	-0.560222775	0.821231112
AT1G11120	AT1G11120	-1.307853683	0.107392864	-3.500533404	9.74959E-06	-2.296173914	0.001125431	-0.662003551	0.637614678	0.097466488	0.923631194	0.100395923	0.734399536
AT1G44110	CYCA1;1	-1.018819155	NA	-3.092290808	0.000182178	-2.300652527	9.40316E-07	-0.40451497	0.852133107	0.446059226	0.445236462	0.696034599	9.39277E-06
AT5G02890	AT5G02890	-1.689936614	0.34744306	-2.878166919	0.031486254	-2.307586106	0.001896623	-0.506255931	0.835442106	0.058329004	0.957261724	0.369019282	0.118019998
AT1G59540	ZCF125	-1.184018169	0.714024714	-1.994215171	0.00378893	-2.309560006	0.00239284	-0.55237608	0.926124658	-0.131964087	0.886676119	0.495744167	0.021047032
AT1G72890	AT1G72890	-0.337127353	0.665047835	-1.639504045	0.000488447	-2.311416202	0.004183596	0.404338462	0.80141891	0.27913973	0.838734926	-0.67519387	0.21860835
AT5G67270	EB1C	-1.083915013	0.3539449	-2.117095784	0.010306141	-2.322740688	1.89294E-06	-0.303318363	0.965260868	0.230052937	0.728724954	0.392143995	0.043231626
AT2G25735	AT2G25735	-1.300195364	0.078198733	-1.695602726	7.98747E-05	-2.33883127	5.96175E-12	0.942463951	0.637614678	-0.236481307	0.926800209	0.283006927	0.819960054
AT3G07320	AT3G07320	-1.167393553	0.101428397	-1.067154409	0.000631366	-2.339023986	1.43125E-06	-0.789404812	0.665778009	-0.530620378	0.086779373	0.07881548	0.732288848
AT2G33560	BUBR1	-0.573545008	0.714394678	-2.852726146	3.60826E-05	-2.342990751	0.001255484	0.014179133	0.997515054	0.276486701	0.76508716	0.336459838	0.224438289
AT4G22490	AT4G22490	-1.399977609	0.398434726	-3.964542423	6.52739E-19	-2.353476152	2.93731E-09	-0.222918084	0.946164167	0.807938088	0.265437817	0.886351209	9.23574E-14
AT4G29240	AT4G29240	-0.150693483	0.930511772	-2.802731275	0.047165847	-2.362082568	0.010025637	-0.212180233	0.950356778	0.122666088	0.872234469	0.539289564	0.036640588
AT3G56440	ATG18D	-0.80754987	0.498861127	-1.900948536	0.003360817	-2.38600036	0.002668318	-0.650357392	0.909924166	0.489291986	0.583966301	0.92298058	0.014166247
AT5G66610	DAR7	-1.028742944	0.522439167	-2.396903867	0.000191787	-2.410903506	0.000278056	0.40390056	0.807059483	0.530181556	0.448596009	0.690166182	0.05371814
AT5G21100	AT5G21100	-1.411544019	0.053229623	-2.103920585	0.004325429	-2.48779252	1.54696E-06	-0.289583658	0.915850405	-0.154444579	0.87076682	0.890523294	1.45983E-05
AT3G53750	ACT3	-1.510766543	0.062526926	-2.048224818	0.001400031	-2.493817701	1.47037E-15	-0.655263941	0.708185012	0.015812741	0.979610562	0.888455985	2.13607E-11
AT4G03100	AT4G03100	-1.628512679	0.168249432	-1.758925297	0.002690589	-2.497245224	3.4974E-05	-0.071433703	0.981893161	0.167204172	0.834745508	0.310167248	0.158699751
AT2G26760	CYCB1;4	-0.656631949	0.579903259	-3.095150866	3.61601E-06	-2.522641716	0.000205544	-0.627680887	0.912756407	0.234095688	0.716620878	0.318196794	0.27765919
AT3G02640	AT3G02640	-1.296542128	0.232356144	-3.735774392	1.42331E-12	-2.537495871	2.05713E-11	-0.382169627	0.766274785	-0.00272347	0.997749369	0.517151554	0.000265331
AT3G23670	KINESIN-12B	-0.82477086	0.513793841	-2.661882962	2.11731E-06	-2.540156566	0.002373254	-0.074988907	0.984800288	0.48153569	0.425062136	0.69235238	0.001085895
AT5G37630	EMB2656	-1.051597495	0.215032762	-1.845182112	0.000400106	-2.54733478	3.76153E-05	-0.529885721	0.930511033	-0.080426111	0.929622352	0.364664476	0.176991167
AT2G47370	AT2G47370	-1.633803746	0.058617708	-8.774307992	5.62781E-06	-2.548683962	0.011096354	0.2024407	0.982055475	-0.961857017	0.832852351	-0.308619161	0.828623408
AT3G11520	CYCB1;3	-0.924365158	0.469317461	-2.123125859	0.010810271	-2.563855205	0.000242161	-0.41417875	0.872669065	-0.343730249	0.619834919	0.514714183	0.070248539
AT3G59550	SYN3	-2.425668353	0.202743341	-2.89415686	0.013811954	-2.570672061	0.000489589	-0.517133992	0.932705464	-0.693764458	0.383106164	-0.139228383	0.747552861
AT1G53140	DRP5A	-0.696164287	0.55326564	-2.520135754	0.000119912	-2.570942786	5.85723E-07	-0.185284881	0.979832545	-0.066738482	0.950400259	0.587803605	0.003337521
AT1G54010	AT1G54010	-1.631739124	0.233838272	-1.956175787	0.03349798	-2.59071711	5.67874E-14	-0.772954656	0.685495817	-0.214395507	0.511503982	0.754206957	0.000239815
AT4G26660	AT4G26660	-1.404901696	0.333922614	-2.364866685	0.001321956	-2.604440107	0.004413868	-0.37706234	0.954839264	-0.130497787	0.872528185	0.528534161	0.009835813
AT3G45390	AT3G45390	-2.461812555	0.478678155	-7.995579757	0.000302966	-2.644477224	2.10444E-05	-0.67820793	0.916990613	1.000709972	0.674900862	-0.378123257	0.598024022
AT4G20430	AT4G20430	-1.513793251	0.116083389	-2.555209376	5.35493E-13	-2.660497816	6.3309E-12	-0.427714644	0.8351738	-0.224542178	0.70029725	0.783441387	0.000243006
AT1G06080	ADS1	-2.513200504	0.065419122	-1.818794581	0.003813164	-2.669012762	7.63689E-10	-0.695588593	0.721967244	0.292579702	0.32606276	0.200951921	0.364601396
AT4G27595	AT4G27595	-0.715595717	0.41895179	-1.225635726	0.043812972	-2.695657617	1.5873E-09	-0.839401185	0.66524984	-0.164829875	0.787771995	-0.597631101	0.001286209
AT5G51600	PLE	-0.365978191	0.718042074	-2.013405702	0.001344127	-2.710105721	3.5568E-06	0.30965986	0.876044849	0.124858913	0.879603903	0.613767163	0.001356028
AT1G12620	AT1G12620	-0.616853529	0.383443279	-1.815790283	0.005395442	-2.833080151	0.004699073	-0.147382784	0.950201026	-0.030262312	0.983566153	-0.462207242	0.566627637
AT3G58850	PAR2	-1.168866892	0.15535629	-1.826430289	1.81831E-06	-2.841537838	3.14285E-13	-0.193773127	0.918503553	0.577906502	0.013499936	-0.163679117	0.679319715
AT1G05440	AT1G05440	-1.15576468	NA	-8.392752326	2.64294E-05	-2.936734632	0.003945614	-0.220468784	0.928888991	0.731995395	0.341313856	0.45223962	0.193672908
AT3G48425	AT3G48425	-1.220519304	0.177717316	-1.960723019	0.002243104	-3.131109895	3.98959E-06	0.210914954	0.91224978	0.126563273	0.902992004	0.376186249	0.157688708
AT2G44740	CYCP4;1	-0.947586894	0.80046884	-1.873833904	0.025480769	-3.211325885	3.33636E-12	-1.089694369	0.692562785	-0.282034768	0.647571386	-0.311446923	0.183048339
AT1G08730	XIC	-3.701637014	0.277197367	-8.604680561	3.71282E-05	-3.277436772	0.044797987	1.738735057	0.722658205	0.68727456	0.897458174	0.441948969	0.622471367
AT5G67280	RLK	-1.324409503	0.179787229	-1.12459577	0.02463162	-3.288783759	2.0411E-14	-0.717702229	0.604422229	-0.472997587	0.117868539	0.375952469	0.133252527
AT1G70220	AT1G70220	-0.36043309	0.946554216	-7.335354205	0.01369241	-3.367686705	0.022658371	0.42223631	0.979951138	0.923447021	0.713515945	-1.572143361	0.314357019
AT5G23400	AT5G23400	-1.516311375	0.202672638	-1.68731877	0.047944786	-3.443179907	0.000617337	-0.540541429	0.841139133	-0.254282757	0.714984255	0.154428619	0.680391362
AT4G15160	AT4G15160	0.13092796	0.967558012	-9.159400073	8.88376E-08	-3.646178539	0.037447207	-4.033518209	0.455598103	-0.491497953	0.127496273	0.155130502	0.632522432
AT5G44500	AT5G44500	-0.253904278	0.786620854	-1.236903913	0.021728528	-3.939259425	0.041525387	-0.100488971	0.962474985	-0.027343912	0.974957125	0.323813495	0.445987955
AT4G30650	AT4G30650	-1.118060222	0.231918634	-1.490058503	0.020079361	-4.099728067	1.55925E-15	-0.18226711	0.980359148	0.023171972	0.986099562	-0.293680683	0.323278458
AT5G28630	AT5G28630	-1.121413645	0.094590108	-2.33881335	0.000297866	-4.450674583	1.17147E-12	-1.170323074	0.631570795	-0.617911002	0.664563024	0.067971995	0.913359499
AT1G24148	AT1G24148	-3.848225763	0.286630648	-2.161243546	0.001862691	-4.67032411	3.89315E-08	2.932856673	NA	4.760782423	0.566059569	-1.603068282	0.502100171
AT1G12100	AT1G12100	-1.504125969	0.719515894	-8.35120812	2.72594E-05	-5.214871147	3.88292E-05	-0.218665516	NA	2.15870237	0.555532957	1.888982967	0.145409887
AT4G25950	VATG3	-5.961832816	0.290702795	-7.264576897	0.007829775	-6.139949134	0.040259004			5.570258497	0.498449509	-3.961807178	0.512034786
AT2G18620	AT2G18620	0.719661621	0.862343103	-6.801571084	0.025488477	-6.334496792	0.026878379	-0.378647041	0.961702889	0.072571872	0.98407766	-0.954143443	0.172252321
AT1G23350	AT1G23350	-4.142513151	0.079957642	-7.063634134	0.011866186	-6.337127978	0.035923436	-5.485320817	0.642268593	1.618629846	0.620431294	-1.643636431	0.620819521
AT1G06090	AT1G06090	-0.056908816	0.985952479	-9.863963327	8.59262E-10	-7.027443678	0.005726953	-0.018355312	0.99779774	0.380624696	0.765891356	-0.853163298	0.024650868
AT2G40470	LBD15	3.113537158	0.617213437	-7.659539951	0.001204462	-7.042468428	0.006160242	-1.182153984	0.937384913	-0.097955459	0.976570624	-1.24365837	0.199443085
AT3G44970	AT3G44970	-2.304320677	0.160698744	-2.815085417	0.003701592	-7.084451304	0.006617486	-6.098263603	0.616300683	4.369805847	0.397816908	-1.210143	0.82094289
AT1G10770	AT1G10770	-0.141511755	0.968863476	-7.418765981	0.036275466	-7.092084807	0.001882736					0.545686113	NA
AT5G58280	AT5G58280	-1.563385453	0.711653065	-7.758137033	0.00296247	-7.281005172	0.001161611	-0.064260363	0.993955856	-0.274695415	0.938675699	1.177774375	0.053356244

AT5G52930	AT5G52930	-2.3267497	0.277338216	-8.382512075	5.3961E-05	-7.635963695	0.000353257	-1.677263232	0.825077044	0.098899352	0.985120284	0.71669	0.377766728
AT1G75250	RL6	-1.856569257	0.525101376	-8.933067425	6.12194E-06	-7.670163173	8.9819E-05	3.216172523	0.721140128	1.640538866	0.788769673	-1.387132731	0.425314887
AT3G61090	AT3G61090	-3.130842469	0.070740262	-4.682862974	0.02082882	-8.019662046	1.4195E-05	2.739641658	0.823368717	-1.007496639	0.884532096	1.66775207	0.1493765
AT1G80745	AT1G80745	-6.851203767	0.052277535	-6.57293038	0.049385144	-8.316625659	2.36368E-06	-0.929552475	0.95625417	-2.550800133	0.45316439	0.5121139	0.729188227
AT4G36700	AT4G36700			-7.056208656	0.014166558	-8.693286336	8.28833E-08	1.962779342	0.890903688			-2.441984156	0.202387207
AT5G25610	RD22	-0.599483554	0.052413518	1.692925738	2.34206E-11	0.757678216	0.358357134	-1.932992488	4.56935E-11	-1.362530648	3.9814E-16	-1.989220609	1.78477E-26
AT1G49960	AT1G49960	3.286863797	0.060333855	2.668479312	0.019724051	-0.126850141	0.971231439	-6.557786672	0.000198626	-0.786250401	0.597798092	-2.308231215	8.21319E-15
AT3G49230	AT3G49230	3.145168155	0.270198608	8.240435894	0.001532937	0.348896733	0.924628388	20.79065606	6.36558E-05	1.466476872	0.845100928	0.574512082	0.229659605
AT3G12750	ZIP1	1.34398049	0.089252015	1.377852457	0.014873791	-0.02485197	0.98114823	-1.276573078	0.0090643	-0.212647821	0.817744169	-0.461022034	0.029354631
AT5G55020	MYB120	2.069943622	0.431585969	2.77253421	1.39005E-05	2.969114619	0.200296696	-7.794565184	0.01227281	0.546269582	0.938376901	-1.86991968	0.32862821
AT1G55290	AT1G55290	3.406494391	0.163278943	3.715917276	0.025591602	0.06872943	0.992022419	-7.987579707	0.012858082	2.364766504	0.572682273	-0.436046107	0.896302442
AT5G49920	AT5G49920	1.239214954	0.474378112	3.930319263	0.040883369	0.989896707	0.691882213	-8.455284186	0.009017776	-1.916878292	0.443659868	0.838307999	0.300139705
AT4G31950	CYP82C3	-1.317638189	0.852530818	8.399333248	0.005733005	-2.952387681	NA	-9.195549288	3.38287E-05			3.293167546	0.600118624
AT5G64620	C/VIF2	-0.476331799	0.365969683	-1.678286701	2.31185E-10	-0.38967852	0.292419283	-1.139145088	0.007483808	-0.403083932	0.10900746	-0.492659328	0.086034607
AT5G01490	CAX4	3.13179887	NA	-6.602630929	0.043814557			-8.804826006	0.000193989	-3.135167956	0.135568366	-2.497977031	0.203142166
AT3G61840	AT3G61840	-5.901639619	0.296235462	-6.658356252	0.037286545	0.20250523	0.966468022	-7.45529441	0.00450004	0.000240517	0.999968179	-0.262743933	0.838875738
AT5G50260	CEP1	7.465657592	0.055351324	-6.727709558	0.041018982	-0.298155996	0.951752165	-5.722125497	0.021231566	-0.191384954	0.958669515	-1.238259026	0.220660836
AT2G35770	scpl28	5.023551517	NA	-6.754898633	0.039632045			-9.479875955	1.23918E-06	-0.170131048	0.959473461	-0.646116293	0.453884032
AT1G12340	AT1G12340	-4.133385345	0.491413925	-7.082614507	0.015166914	0.267740273	0.4695086	-9.50548877	2.01516E-06	1.875663461	0.611660588	0.013508136	0.973737207
AT3G28510	AT3G28510	4.531793567	0.084670639	9.271466671	0.000395926	-0.596619249	0.88612199	-2.481817102	0.06242455	-2.473884469	0.011022037	-2.261914974	6.50528E-06
AT5G05500	MOP10	0.940863866	0.852604319	8.249998644	0.008571111			-2.242538772	0.826650676	-4.605364712	0.00150107	-3.819936693	0.004211271
AT1G51890	AT1G51890	3.379223229	0.160590679	3.456683121	0.036163405	2.358781331	0.18565956	-1.94105508	0.631991778	-2.455938949	0.00803865	-2.341493853	1.80411E-06
AT4G17340	TIP2;2	0.454784541	0.305359946	1.140459982	0.002874856	0.754740955	0.078269229	-1.065119789	0.255573307	-1.585740647	0.003010746	-3.590040902	3.92806E-74
AT2G46400	WRKY46	-0.830608648	0.198225001	-1.064220479	0.030856276	-0.608122548	0.148968617	-0.527365759	0.584219873	-2.074061894	0.005241583	2.793612566	2.56969E-79
AT2G22500	UCP5	-0.91023795	0.02354675	-1.286474916	1.88345E-05	-1.068238538	0.162467062	0.46088482	0.685495817	-1.282552487	2.37858E-07	2.195444847	8.6857E-66
AT1G70690	HWI1	-0.952648852	0.48110167	-3.229799284	6.57502E-05	-0.188033321	NA	-1.722141659	0.66808962	-1.30572842	0.005328469	-1.225348268	0.000589921
AT3G58940	AT3G58940	-0.520406559	0.690530683	-3.574056585	1.96335E-12	-0.805389789	0.623212619	-0.005202147	0.998732347	1.055930142	0.000547356	1.004713146	6.38654E-06
AT1G78865	AT1G78865	-0.966850824	0.523381947	-3.708660008	0.001378366	-1.215275727	0.16365312	0.701978444	0.790420536	1.174412242	0.003964955	1.380606054	1.03317E-13
AT3G49110	PRXCA	-0.508434835	0.739370515	-4.289884772	4.42483E-05	-0.738262763	0.667222126	0.34332527	0.923271698	-1.254616617	0.004347678	1.021626166	0.000245675
AT4G09780	AT4G09780			7.623085161	0.037204764	-4.385817118	0.419578731	-5.523927307	0.640704059	-8.504983461	0.000107866	-1.424330052	0.418843278
AT4G05097	None	-0.366498064	0.962381846	7.559240802	0.036428936					-7.099169665	0.025797515		
AT1G66700	PXMT1	8.819593197	NA	4.717058464	0.005331612	0.794982896	0.527760526	-2.857808977	0.627500883	-2.288782482	0.004669914	-0.153998229	0.400402529
AT1G13520	AT1G13520	0.521921932	0.811170069	2.959213677	0.026867137	-2.484099138	0.658069443	-4.385440649	0.268841422	-4.243242786	0.031757621	-2.621221016	0.10861734
AT1G78850	AT1G78850	0.164081485	0.761760446	1.026611669	1.014E-05	-0.150933258	0.616539116	-0.092897495	0.945609936	-1.176074629	0.001732895	-0.007147797	0.980083634
AT4G35810	AT4G35810	-1.219866657	0.411265545	-3.187892242	3.30267E-06	-0.517329159	0.674942188	0.422554099	0.95279399	2.625913121	3.60245E-08	0.708969161	0.01805251
AT5G38320	AT5G38320	-0.433225685	0.914900339	-8.080931995	0.000172306	-1.319938205	0.710105973	-0.168479589	0.982055475	1.998851779	0.010528954	-0.440374565	0.491211689
AT1G28710	AT1G28710	-1.222288004	0.101274602	-2.621975227	4.54453E-06	-0.230444768	0.748515462	0.101030723	0.992570276	1.333515873	3.84601E-08	0.417363357	0.014730669
AT1G75880	AT1G75880	-0.448196905	0.596633712	-1.398679532	0.002546809	-0.860434309	0.124058243	0.785390269	0.658780788	1.108354773	0.040223026	-0.092564993	0.916453331
AT3G26590	AT3G26590	-0.954714074	0.545923755	-4.869333283	0.006354284	-1.742115958	0.282728763	-2.379577495	0.561207188	-1.340185859	0.026828578	-0.509265555	0.270155294
AT4G25420	GA20OX1	-1.925486981	0.28809745	-3.911640716	0.047849543	-1.652044271	0.190859861	-2.365577495	0.691895802	-2.403229136	0.048834515	-0.094935719	0.920529379
AT2G47670	AT2G47670	5.82855554	0.303140656	-7.486413044	0.031840401	-1.186663936	0.177995469	1.853658509	0.465437463	-2.448908213	0.013770802	-1.293008427	0.609340774
AT4G00955	AT4G00955	-0.777826797	0.74686605	-7.340410868	0.042074644	-2.002768908	0.429325143	-1.193268129	0.807334996	-5.463379922	0.017657803	-0.892885238	0.191073606
AT4G12490	AT4G12490			6.87457972	0.009931073	2.980463815	0.547811518	0.742249337	0.968021479	5.486116696	0.477463307	5.383886867	6.76581E-06
AT5G01680	CHX26	-2.137913309	0.597060208	6.110904656	0.017723215	0.530414802	0.901927967			-0.223231513	0.984765765	4.532909223	0.015746477
AT5G24110	WRKY30	-2.29766323	0.416869968	2.198571751	0.011293332	0.670201909	0.750612688	-0.922382985	0.926463142	0.803352059	0.933194561	4.263560292	0.001277049
AT3G56790	AT3G56790	-0.134243843	0.899159016	1.470735072	0.019347637	-0.179248806	0.870261223	-0.605151404	0.782193202	-0.383256945	0.896241369	3.16158435	0.047013486
AT2G27270	AT2G27270	0.841556177	0.776081977	4.533565993	0.048329144	-0.786318417	0.791221097	-6.892594169	0.159803622	-6.11870682	0.27856344	3.010177199	0.038474317
AT5G45630	AT5G45630	0.3194665	0.722175235	2.331017489	2.1422E-10	0.732430317	0.092729976	2.199059078	0.719955906	-0.173221151	0.948625602	2.582603929	1.47148E-15
AT4G12480	EARLI1	-0.702430745	0.634935323	4.747882996	0.015074193	1.602397603	0.563907217	-0.685391542	0.916448617	-1.610973013	0.054073078	2.467482688	1.94481E-43
AT3G22830	HSFA6B	1.091414813	0.324945576	2.984174316	0.015217977	0.095941979	0.935858501	-1.420039925	0.691895802	-2.133015482	0.132749441	2.034012226	2.35782E-06
AT2G44070	AT2G44070	5.987871339	0.288572341	9.374417098	0.000275713			-0.769855897	0.936447266	0.586683048	0.78801231	1.987439572	0.001455243
AT1G74830	AT1G74830	1.316060934	0.416666294	3.400341855	0.015410235	2.389080451	0.298189133	-1.298825287	0.890241742	-0.363661591	0.958669515	1.860105509	0.010854589
AT4G27657	AT4G27657	0.625256505	0.344794406	1.260701302	0.000548105	-0.399511791	0.4137264	1.210007499	0.568009156	-0.408285393	0.596277828	1.84706861	1.42396E-11
AT5G62470	MYB96	0.539419851	0.378697873	1.281953785	4.05383E-07	0.338222134	0.267682908	1.191892525	0.579644733	-0.674199777	0.041396188	1.545305548	7.02961E-28
AT5G64310	AGP1	0.592790741	0.242515109	1.547065606	1.06944E-06	0.226414257	0.84014158	0.123847095	0.935083701	-0.539432874	0.00409111	1.404222123	1.6882E-22
AT4G36770	AT4G36770	2.247612022	0.285993754	2.917660454	0.005425589	2.156457342	0.194758128	2.112869886	0.76611969	-0.118252744	0.978139078	1.331386241	0.013108274
AT1G61890	AT1G61890	0.7969722	0.056844717	1.550185691	1.09154E-06	0.699892229	0.370006299	0.14789582	0.947010908	-0.443344712	0.198863935	1.247654656	1.319E-22
AT4G17250	AT4G17250	0.623936988	0.309049668	1.359719622	3.64344E-11	0.717634361	0.000679302	1.187215568	0.604422229	-0.015544069	0.98877807	1.186427833	2.87907E-09
AT4G26120	AT4G26120	0.998567587	0.242054289	2.02230958	8.48434E-05	0.362963833	0.751270695	1.026345596	0.554617999	-0.527897552	0.702332232	1.129391023	0.04068411
AT2G36800	DOGT1	1.091519025	0.112973761	2.003232677	3.0361E-07	0.852272581	0.02461852	0.497487706	0.735038565	-0.035185406	0.978281903	1.117223324	1.12799E-10
AT3G60670	AT3G60670	-0.051122363	0.975026773	1.837854358	0.047382268	1.108798972	0.283758276	1.123610159	0.496998136	1.274897547	0.104958611	1.096704411	0.000923314
AT1G06250	AT1G06250	1.701538885	0.093055446	1.520585538	0.002770379	0.459950334	0.466659381	0.506856502	0.802115332	-0.78785664	0.290963362	1.00484601	0.022216597
AT2G18193	AT2G18193	0.667798787	0.496347853	2.813824477	0.000153453	0.391903396	0.53520185	-0.728692523	0.637815859	-0.882415128	0.046990814	-1.00381732	6.85522E-07
AT1G09970	LRR XI-23	0.962741161	0.000417721	1.42167545	2.83009E-13	0.745434455	5.63112E-05	-0.238181124	0.809598664	-0.265971905	0.294649168	-1.023720244	2.76314E-20
AT5G44585	AT5G44585	2.26564833	0.154511602	1.934309112	0.033911044	-0.971783261	0.435536147	-0.752359787	0.646729152	-1.017454188	0.158420892	-1.044345182	0.020112954
AT5G50100	AT5G50100	-0.057689919	0.948003005	1.251752165	0.003391871	-0.011869065	0.983724612	1.881325145	0.6208322	0.002434033	0.999543361	-1.051362839	0.038342558
AT5G66790	AT5G66790	0.692465396	0.101808171	1.111699695	5.57309E-06	0.847901349	0.001798632	0.46105818	0.758815467	-0.368729122	0.478521021	-1.059121884	2.20184E-06
AT5G66110	HIPP27	0.819666865	0.222683379	1.731301636	0.00926987	0.755770087	0.243776817	0.505099812	0.884882727	0.314409955	0.779367665	-1.088242528	0.003017144
AT4G36380	ROT3	0.597464428	0.174331433	1.557609061	0.006699611	0.2086368	0.510150325	-0.486588831	0.634112695	-0.665194143	0.001551085	-1.094215674	1.03875E-08
AT5G49660	XIP1	0.610995321	0.164778953	1.001812389	0.002123262	0.005631586	0.990094987	-0.373926339	0.689887596	-0.373392973	0.265754101	-1.097918221	2.49291E-09
AT3G16800	AT3G16800	0.744874196	0.077013793	1.385558751	0.000198315	-0.069825061	0.862237347	-0.914638644	0.212736692	-0.452751681	0.206811617	-1.105008993	1.98552E-06
AT1G07720	KCS3	-0.067606655	0.906367946	1.248291733	0.000254852	0.557508909	0.006239856	-0.865376879	0.356598451	-0.965804747	2.70372E-09	-1.1319994	2.07246E-14
AT1G14730	AT1G14730	0.984090818	0.065123854	1.118549533	0.019489754	0.81997227	0.173598206	-1.180966592	0.143428011	-0.632250355	0.034469941	-1.136451784	9.84232E-16
AT3G54140	PTR1	0.843123884	0.002013014	1.0016241	2.77298E-05	0.660563979	0.000777218	-0.543751878	0.569893363	-0.312924834	0.142881118	-1.156433219	4.56668E-24
AT2G27660	AT2G27660	0.729023849	0.763470291	2.4491177	0.035795922	-1.066677371	0.677855915	-2.505247529	0.496656379	-1.089796327	0.277755856	-1.207595728	0.012137991
AT3G53980	AT3G53980	-1.10888596	0.268368874	1.033606757	0.04491072	1.155254822	0.182373128	-0.38272263	0.920233522	-0.451755773	0.668878046	-1.216139768	1.75877E-24
AT2G37970	SOUL-1	0.795710652	0.561375674	1.863937159	0.015723403	0.761336534	0.324069692	1.667103035	0.565351364	0.02286102	0.997749369	-1.218553494	0.000256325
AT4G31370	FLA5	3.387327239	0.062396909	3.482236506	0.027591006	0.266735874	0.805708018	0.17137159	0.961157568	-0.12131445	0.938458484	-1.262669076	0.000597333
AT3G62410	CP12-2	-0.087876117	0.920697036	1.105918989	0.000157389	0.070037541	0.809701832	1.909438971	0.430181703	-0.279671065	0.699172252	-1.367886292	5.76411E-05
AT4G31230	AT4G31230	-0.817367132	0.601949921	3.385092408	0.049717823	0.074691874	0.979535236	-0.874118745	0.71427218	-1.683705331	0.312147668	-1.372411833	0.024380923
AT4G28040	UMAMIT33	0.376869455	0.516426003	1.078820297	2.91216E-08	0.357570719	0.021741848	1.8031198	0.373684124	-0.344852902	0.555733007	-1.385635971	1.06988E-11
AT1G21920	AT1G21920	0.979826687	0.001073654	1.049252941	1.66614E-05	0.609602664	0.001986802	-0.073909222	0.954861124	-0.315543389	0.115052992	-1.428636258	6.74019E-15
AT3G56080	AT3G56080	-0.596617553	0.494075613	1.187596412	0.01932001	0.956415772	0.000143024	-1.782567736	0.050118767	-0.881631675	0.158833385	-1.43366756	3.31307E-07
AT5G24120	SIGE	-0.129651515	0.898892011	1.138734742	0.018496193	0.131175358	0.813769755	2.619979575	0.25362179	0.566503561	0.717454016	-1.437106117	0.003086544
AT5G51440	AT5G51440	1.210414398	0.528680246	2.943767097	0.001043576	-0.067755331	0.976213832	-0.79163369	0.552538268	-1.132714478	0.073648126	-1.467652157	3.72183E-05
AT2G29460	GSTU4	1.138285281	0.603060872	4.14264847	0.001240781	1.077176681	0.384736164	-2.858169352	0.389591858	0.755606834	0.593339308	-1.474603701	0.020130194
AT5G53870	ENODL1	1.660000576	0.109957818	1.883469179	0.031318499	1.338841432	0.305526545	-0.251422777	0.926698256	-0.605983475	0.227633707	-1.477216227	0.000634811
AT5G40800	AT5G40800	4.02113896	0.061399344	7.88491954	0.023708538	0.439246452	0.861928306	-0.80606713	0.88256585	-0.723192217	0.556988648	-1.529719717	6.60652E-05
AT1G71910	AT1G71910	2.430609095	0.332942549	3.470714303	0.011810643	0.484702069	0.625236193	-0.287513278	0.972077881	-0.247842101	0.929498792	-1.532163118	0.013872019
AT4G23493	AT4G23493	2.94778429	0.120903826	3.707324244	0.015019421	1.114745488	0.275708675	-0.378661275	0.854730188	-0.500429148	0.653255372	-1.673368306	4.23633E-06
AT5G23750	AT5G23750	0.316565375	0.651256345	1.45794796	0.001468634	0.867075291	0.007045572	-0.332220358	0.735038565	-0.958355779	0.005266251	-1.720112438	1.28399E-08
AT2G43570	CHI	-0.794191878	0.616015417	3.555132097	3.80465E-05	0.672654879	0.620275696	-1.574034818	0.654905244	-0.190388305	0.913903461	-1.749879738	0.00523468
AT5G37500	GORK	0.745495072	0.28783548	1.346910003	0.013837681	1.236661919	0.097499663	-0.591160717	0.66524984	-0.705295205	0.079150458	-1.816019837	9.2258E-09
AT3G08860	PYD4	2.743620215	0.090285734	8.849936377	0.002367752	1.722425472	0.764196188	0.81911686	0.7265412	-0.333277079	0.798599938	-1.975038431	0.000367305

AT5G57550	XTH25	0.583286609	0.389855348	1.110361888	0.000627561	0.6571557	0.039735902	0.932641138	0.758662788	-1.482934976	0.083333251	-2.058720973	1.27716E-06
AT4G20000	AT4G20000	0.317026732	0.93052539	3.894046346	0.012811737	0.547524574	0.852836161	-3.614025299	0.186649778	0.397956257	0.857737893	-2.126318645	0.002695424
AT3G49700	ACS9	-6.462073343	0.246360339	7.641513682	0.03320283			-2.499869381	0.704225272	-1.317692835	0.372984939	-2.146021996	0.002413568
AT4G20110	VSR7	3.938847585	0.067652529	1.358842556	0.004802647	0.581695138	0.502044757	-0.99291274	0.663725004	-0.601506511	0.112330215	-2.265189597	4.3454E-17
AT1G07620	ATOBGM	1.288656284	0.453584412	4.589500729	0.028831114	1.450608541	0.645806593	-1.824892384	0.648919095	0.180176165	0.93404597	-2.414944477	6.45132E-07
AT2G26560	PLA2A	-0.127383271	0.882873187	1.168991834	0.001321956	-0.16815793	0.854350073	-0.951872733	0.385221483	-1.675799824	0.368015022	-2.507365923	0.000316764
AT1G58270	ZW9	0.621114025	0.161586696	1.435016355	7.91337E-11	1.31612261	0.140204306	0.40715434	0.728150704	-0.228692835	0.282851207	-2.601452119	5.79941E-08
AT5G59340	WOX2	-0.057254129	0.962232511	2.406212749	0.002746644	0.904997031	0.123357613	0.985431612	0.643189471	0.014413736	0.998107647	-2.628570764	0.003547062
AT3G57020	AT3G57020	0.466334667	0.486845319	2.476472403	3.96747E-09	0.911512625	0.052646082	-0.158259889	0.977607985	-1.05379232	0.405816794	-2.648092089	1.15431E-16
AT2G02300	PP2-B5	4.233683905	NA	7.868542748	0.021347162			-1.516989662	0.753910599	-0.207421894	0.954183802	-2.767216752	6.41325E-05
AT5G45920	AT5G45920	1.252279723	0.380531052	1.83454423	0.028602721	0.320410595	0.833610546	-1.692034717	0.66524984	-1.099547088	0.201309169	-2.778009796	1.86625E-13
AT5G44440	AT5G44440	1.900671326	0.158256821	1.556906479	0.028723778	-0.331431579	0.815329546	-1.479569033	0.172855349	-0.773419993	0.000162475	-2.919383083	7.54181E-51
AT5G13210	AT5G13210	2.336818879	0.385498306	3.895929474	3.23497E-21	0.102401052	0.935484086	-0.609606335	0.885755209	-0.251358848	0.851691904	-3.014854366	1.28866E-11
AT5G11977	ath-MIR156e	2.36604959	0.180312688	2.970485821	0.005560253	3.521583058	0.118132025	-0.572185488	0.90769891	-0.514159468	0.869846959	-3.521930526	0.005843152
AT5G24080	AT5G24080	1.129863928	0.654259763	8.717006174	9.58779E-06	1.602854935	0.401844047	-4.636773303	0.290161519	-1.509643323	0.676560651	-3.64808889	4.6952E-05
AT5G40382	AT5G40382	5.952993806	0.291779572	7.872349776	0.020244521	4.996347897	0.259612683	-0.462103575	0.981525175	-4.613738163	0.578542261	-3.7838204	0.007382925
AT1G30870	AT1G30870	-1.037456748	0.83847898	8.018423322	0.000507129	-5.247106702	0.322513735	-2.580367232	0.676109289	-2.573204582	0.312627722	-4.393252306	0.038026005
AT2G42530	COR15B	1.054107727	0.111368004	1.931070655	2.46106E-11	-1.762452342	0.085567017	2.294051908	0.619569853	3.750879606	0.086698345	-4.568199708	0.026086471
AT2G40170	GEA6	-3.242912684	0.603450636	4.838234533	0.037204764	0.655447909	0.902925112	-5.998735832	0.619569853	5.904706843	0.438575468	-5.415674185	0.000208828
AT5G44120	CRA1	1.921044625	0.695116324	7.581971076	0.0352571	5.808781649	0.255941809	-6.230200055	0.610227484	5.535487599	0.5013917	-10.79633478	1.41125E-06
AT3G21150	BBX32	-1.800020296	0.521320516	-8.12831994	0.000135158	-0.730254333	0.468268694	-5.053918176	0.66524984	-5.234182884	0.328939804	5.718578854	0.003185088
AT3G61190	BAP1	-0.65452982	0.313319667	-1.540241377	0.000116278	-0.889231575	0.001558454	1.376686385	0.475139116	-0.987250059	0.409507617	3.627203699	2.3333E-99
AT4G29610	AT4G29610	-3.829861508	0.059747963	-5.486771169	0.00012234	-5.485077594	0.143449796	-6.438161012	0.334544782	-1.569424988	0.664563024	3.260114885	0.00091699
AT5G17350	AT5G17350	-0.568820484	0.365534497	-1.454576112	5.84801E-07	-0.590937251	0.547896602	1.165354121	0.418113898	-0.796798097	0.120011613	2.979352299	2.21676E-46
AT3G10930	AT3G10930	-0.90375741	0.157176658	-1.706051247	1.14917E-05	-0.943120229	0.312165568	0.237750435	0.93556951	-0.567481382	0.464148269	2.92408217	3.74417E-30
AT1G61470	AT1G61470	-1.647989678	0.080557328	-1.670478108	0.01905226	-1.061935978	0.082308868	0.163539362	0.964169173	-1.970797036	0.308238589	2.781860246	4.60724E-24
AT2G14580	PRB1	-1.764572876	NA	-10.87248107	1.80029E-11	0.969402091	0.877546588	0.353435565	0.963509602	0.730837378	0.58538838	2.668829997	2.23359E-06
AT1G73550	AT1G73550	-3.711544429	0.07014583	-6.66075929	3.44445E-05	0.71345807	0.597945563	-0.286778472	0.984889186	0.085135188	0.991419859	2.521888564	0.011060683
AT2G27080	AT2G27080	-0.982972868	0.045280799	-1.333386922	5.95506E-07	-0.989379388	0.007725282	0.380981366	0.769907555	-0.739274517	0.006694896	2.482731116	1.30659E-50
AT3G50060	MYB77	-0.997375065	0.03553382	-1.136903956	0.00026466	-0.989775268	5.40126E-05	1.027608358	0.190412481	-0.512727564	0.258983627	2.353502676	1.00429E-70
AT3G28070	UMAMIT46	-1.446491155	0.281440464	-2.109600684	0.009570842	-0.707602971	0.32882226	-0.552592908	0.919009661	0.302517969	0.879603903	1.924099996	2.71341E-06
AT4G26255	AT4G26255	-1.512654469	0.537591544	-6.782791726	0.033017313	-3.801136243	0.488928855	0.37356529	0.963252175	0.274054385	0.948625602	1.913829864	0.022072874
AT3G13310	AT3G13310	-0.614182508	0.260934102	-1.224069434	0.000237082	-0.483836495	0.156678587	1.427121194	0.259637878	0.019661178	0.98143759	1.705395558	5.35533E-27
AT4G32800	AT4G32800	-1.184501042	0.089876154	-2.404868263	0.00012234	-1.048537956	0.064576332	0.50868131	0.709813898	0.016567865	0.992281921	1.603824342	1.46179E-10
AT1G66160	CMPG1	-1.052545841	0.102901324	-1.29049997	0.000109857	-0.249681063	0.52778063	0.561933104	0.771785129	-0.2666179	0.63650345	1.557634192	4.4556E-24
AT2G38995	AT2G38995	-1.460560831	NA	-8.35205058	0.00011435	0.2012382	0.960819369	1.155331158	0.834298409	-0.061041901	0.991628492	1.540154936	0.000629705
AT1G05575	AT1G05575	-0.704122984	0.396705641	-1.287581848	0.004919011	-1.13241331	0.068851341	0.279092933	0.824436763	-1.419955133	0.309618466	1.475241379	5.48626E-05
AT2G24350	AT2G24350	-0.817894977	0.722413279	-1.97683454	0.017302753	-1.270693411	0.103958021	1.097701169	0.647947756	-0.401763387	0.747270887	1.451535085	0.000109064
AT1G53920	GLIP5	-0.913849717	0.470003991	-1.857052697	0.015074193	-0.301629407	0.738923907	0.438404129	0.850602242	-0.039308622	0.987053629	1.393208487	3.17948E-08
AT4G28680	TYRDC	0.388693997	0.932054157	-7.746217011	0.001913794	-6.440378772	0.072785355	-0.24919264	0.982356953	-0.489505225	0.819770183	1.354577791	0.001140802
AT5G46880	HB-7	-0.771307772	0.387336117	-1.378355463	0.003676352	0.002910762	0.99585763	-0.6039091	0.613859108	0.392843073	0.507785667	1.334335529	4.58306E-13
AT4G39010	GH9B18	-0.434220054	0.881916739	-3.192116749	0.036056087	-3.031173057	0.060980516	0.716533386	0.665778009	0.936940674	0.556088295	1.271495426	0.000400383
AT1G77270	AT1G77270	-0.7893114	0.437891888	-1.834919494	0.001667327	-0.750997686	0.137144959	0.140708435	0.953801756	0.530010885	0.446427204	1.26877059	1.62008E-16
AT5G19340	AT5G19340	0.000839291	0.999112286	-1.320857492	0.014570886	-0.069234642	0.891852181	0.337357869	0.674766805	0.420351549	0.186485571	1.260429473	1.36835E-11
AT3G09800	AT3G09800	-0.702207953	0.138574067	-1.357802394	0.000400568	-0.929364362	0.00323148	0.260264751	0.858965347	0.547789761	0.268092126	1.243716205	9.16115E-13
AT4G33270	CDC20.1	-1.627395238	0.060909348	-2.699414316	1.73993E-12	-0.753106878	0.146298194	-0.185448221	0.954839264	0.226506629	0.678853314	1.215300362	2.96965E-13
AT3G52910	GRF4	-0.701250051	0.420267759	-1.165769107	0.019188844	-0.08936723	0.882630839	0.356475694	0.691824202	0.393726143	0.498449509	1.21268715	3.70005E-12
AT4G17000	AT4G17000	-1.049382136	0.278527931	-2.47487636	0.000259967	-0.966404359	0.658809405	-0.476437045	0.850582824	-0.382439704	0.670869081	1.191900994	0.001787828
AT1G14970	AT1G14970	-1.2549445	0.694951353	-1.998207931	0.002311298	-0.843097375	0.032135138	-0.338931069	0.887611849	0.385569825	0.514073245	1.172321022	6.60896E-09
AT3G57860	UVI4-LIKE	-0.259056418	0.799017702	-2.513395994	0.026975969	-1.079048595	0.189306769	0.006502178	0.997796217	0.863576293	0.036336072	1.138093047	1.26963E-07
AT1G26780	MYB117	-0.167248072	NA	-2.076970307	0.012653008	-0.259207735	0.832211771	-0.294828817	0.896556992	-0.147951076	0.846297688	1.137897648	5.83416E-08
AT1G25400	AT1G25400	-0.836235525	0.142105358	-1.639199626	6.69851E-07	-0.609767012	0.00376441	2.039604677	0.359497315	-0.830853565	0.131410428	1.137256844	0.000183615
AT1G31310	AT1G31310	-0.778899209	0.341401541	-1.746658399	3.55364E-05	0.37084618	0.439665	0.712338479	0.716554635	0.829385694	0.03653082	1.085058455	1.84253E-11
AT3G53232	RTFL1	-1.430946278	0.552220126	-4.005326688	0.045518054	-4.548676703	0.400413021	-0.303108748	0.948672371	0.520102063	0.578542261	1.080003735	0.047577682
AT3G06020	FAF4	-0.719323553	0.630244016	-4.092669715	0.007521542	-0.228621385	0.837656637	0.580182022	0.82297652	0.61645876	0.680252138	1.072090188	0.003826701
AT2G19620	NDL3	-1.406715598	0.082065019	-2.060017125	2.94845E-05	-0.867550388	0.04569976	-0.971933053	0.581307949	-0.015780233	0.989242512	1.070354197	1.10156E-08
AT4G30140	CDEF1	-1.376380867	0.534272697	-5.698717495	9.32545E-07	-2.77822998	0.052498319	-0.945526533	0.677952867	0.518774371	0.52838553	1.051930624	3.44117E-10
AT1G62500	AT1G62500	0.166585197	0.89304517	-1.898493891	7.2162E-07	-0.093347194	0.867693471	0.432407116	0.759378174	0.157629438	0.752481505	1.015754888	4.05192E-11
AT1G67650	AT1G67650	-1.541861938	0.193678278	-1.657581389	0.042300675	0.209557919	0.801048288	-0.67294626	0.726553713	0.064091347	0.973139779	-1.033114013	0.0499353
AT1G35250	AT1G35250	1.752552285	0.643216384	-6.723928539	0.038212463	3.739186775	0.168126271	-0.047705341	0.994744545	-0.353461669	0.846410046	-1.048633767	0.03358386
AT1G31885	NIP3;1	-2.36885392	0.231215881	-7.544946334	0.002687393	-0.51831847	0.787609215	-1.951879177	0.639605292	-0.709241517	0.512974126	-1.098238434	0.003900727
AT1G70890	MLP43	-0.577510933	0.037629926	-1.064520277	0.00298506	-0.913730674	0.19839632	-0.492962274	0.63593807	-0.347976778	0.08865775	-1.102663501	8.34336E-10
AT2G11810	MGDC	0.613007826	0.890630217	-7.527885067	0.005379585	0.188698579	0.797123855	-1.700942692	0.800906229	-1.301725826	0.430532999	-1.128194337	6.04909E-05
AT1G72850	AT1G72850	-1.422435081	0.6056242	-6.918968225	0.021866778	0.47649072	0.513540567	-0.285409025	0.972495118	0.115970662	0.982838333	-1.148421117	0.00093721
AT3G01290	HIR2	-0.09003456	0.895037608	-1.071664054	0.002092396	-0.034221474	0.919695967	0.050473297	0.988073831	-0.516801134	0.102362261	-1.154179427	4.95577E-08
AT4G20140	GSO1	-1.013432241	0.07489925	-2.047337183	6.33021E-05	-0.489781498	0.190599588	-0.729745773	0.563804992	-0.69428468	0.051433089	-1.162538041	5.44413E-08
AT5G38010	AT5G38010	0.444163072	0.890361956	-5.149319684	0.041373204	-2.660589878	0.244718114	-1.093332648	0.602119156	0.134209681	0.896798113	-1.195595197	0.000119526
AT4G12320	CYP706A6	-5.345825932	0.064792536	-4.435770453	2.3435E-05	0.441153882	0.044677362	-8.55632735	0.127893204			-1.28663625	0.000882674
AT4G25760	GDU2	0.399718482	NA	-8.679000379	5.32782E-06	-1.313081137	0.631625366	-0.854255034	0.86175641	-0.629506014	0.438575468	-1.301019076	2.22341E-10
AT5G03380	AT5G03380	-0.37706764	0.374948786	-1.073932395	3.87713E-06	-0.541768177	0.033138507	-0.649974156	0.448565635	-0.586553489	0.039908251	-1.301285353	7.3509E-09
AT1G66140	ZFP4	-0.060658994	0.933262537	-1.420872245	3.41337E-06	-0.687442501	0.021565793	-1.28971453	0.387045614	-0.257018861	0.496280372	-1.30428631	5.66273E-28
AT5G18840	AT5G18840	-0.716707352	0.792781259	-4.310557778	0.009557441	-0.798486363	0.02757675	-2.20615849	0.634709839	-0.772751535	0.771044627	-1.377816039	1.02884E-06
AT3G43190	SUS4	2.286321109	0.363058368	-7.61720565	0.001464158	-0.016209602	0.987261614	-1.365498577	0.732322487	-0.351364594	0.812243776	-1.410112113	0.007588823
AT4G19370	AT4G19370	-1.179515968	0.37149763	-7.923998716	0.000530737	-0.295113266	0.898105273	-0.900221238	0.854730188	-0.838674141	0.36366911	-1.429893821	1.01163E-05
AT4G17470	AT4G17470	-0.836997784	0.758271217	-7.838056819	0.000676787	-4.72633627	0.095518765	-0.345373837	0.965812438	-1.240645573	0.402055214	-1.454568282	1.75867E-07
AT3G45840	AT3G45840	3.076876074	0.370357824	-6.784847759	0.035059816	0.305785836	0.956870736	-1.036529658	0.829279066	-1.746524518	0.064904223	-1.489136625	0.000303498
AT1G62320	AT1G62320	-2.576136531	0.560905238	-7.694944826	0.001019372	0.347499768	0.844618601	-1.356289888	0.859819729	-2.62576389	0.118593098	-1.527072344	0.000376733
AT1G12980	ESR1	0.919433719	0.772101555	-6.661864846	0.039626214	1.840633903	0.571836492	-1.64994654	0.882425056	0.877749107	0.719840427	-1.624264121	0.026512944
AT2G45040	AT2G45040	6.924936047	0.129114988	-7.118561956	0.019737304	-4.987036802	0.351936488	-1.486895525	0.721140128	-1.583060243	0.536643581	-1.639075295	0.019187246
AT5G19520	MSL9	-0.384946212	0.847765378	-9.243729493	1.29936E-07	-0.525917012	0.811291601	-0.183438535	0.955404265	0.69772879	0.31350844	-1.731613826	1.82342E-06
AT5G50120	AT5G50120	1.169708127	0.619043506	-7.735529704	0.001031972	-0.016356848	0.995881762	-0.89489984	0.857531199	-0.508967658	0.712348236	-1.747856108	5.14425E-05
AT1G64210	AT1G64210	2.419866137	0.435038633	-7.510877971	0.002725839	-5.908651266	0.148539459	0.001229859	0.999867318	-0.264173576	0.90589961	-1.758244351	0.002355901
AT2G33550	AT2G33550	-0.317008448	0.659128282	-1.304211484	0.046364941	-0.907322968	0.031727118	-0.007687791	0.996911371	-0.074645726	0.956074131	-1.762233648	3.8839E-12
AT1G35515	HOS10	-0.041242176	NA	-6.68550534	0.034704799	-5.408194202	0.172022513	-3.816727957	0.459923668	-0.049625195	0.989337643	-1.809453917	0.042345069
AT1G17950	MYB52	0.8435879	0.861153624	-7.36683154	0.006596636	-2.333730843	0.620275696	-3.97825929	0.242292325	-0.242970423	0.944784464	-1.903940551	0.000334029
AT1G73330	DR4	2.243272378	0.225442385	-2.176006778	0.00243155	0.481538659	0.673642666	-0.681163871	0.754897495	-0.861489704	0.27238698	-1.951639231	2.55697E-38
AT3G62040	AT3G62040	0.964936238	0.113885703	-2.992258943	1.62513E-11	-1.778984492	0.493431475	-1.650822165	0.432817179	-0.828736055	0.003683221	-1.974701557	1.37265E-29
AT5G44550	AT5G44550	0.464699347	0.768795354	-1.620531068	0.045238964	-0.508733189	0.56448233	-2.126847148	0.704004387	-0.831268733	0.051294254	-1.99986069	1.40316E-49
AT1G79450	ALIS5	-0.417762438	0.838835816	-7.050213243	0.013896569	2.783969105	0.220818506	-1.643663014	0.563758673	-0.652343667	0.541086489	-2.058750837	3.1758E-05
AT1G17710	PEPC1	-1.716565207	0.41895179	-4.916352849	0.007318213	-0.332908982	0.839677401	-1.225713941	0.792048637	-0.603204124	0.624504205	-2.123040164	2.11233E-08
AT5G14070	ROXY2	0.37838611	0.500452155	-1.449391295	0.005218161	-0.863432218	0.047181661	0.665873752	0.837207287	-0.09589863	0.945035463	-2.145533537	8.91575E-11
AT1G05220	AT1G05220	2.124132983	0.599524835	-7.343995293	0.041983049	-1.629268176	0.64744727	-2.585231431	0.66924865	0.838935625	0.861731771	-2.510145897	0.010100015
AT4G36570	RL3	0.442695741	0.722932094	-2.139744762	0.019538327	-2.535249737	0.150076722	0.115141882	0.968891327	-0.889328879	0.111152817	-2.561087743	3.43866E-18
AT5G19790	RAP2.11	6.814568021	0.147572826	-6.962952289	0.022455516	-3.442698025	0.535162474	-0.634837177	0.972495118	0.402363832	0.952485383	-2.703362812	0.04655919

AT1G56680	AT1G56680	1.090600673	0.838199515	-7.376233257	0.004958191	-4.254472324	0.434481728	0.107866863	0.99299517	-1.158648993	0.646238543	-3.023007738	0.008915759
AT4G12050	AT4G12050	3.49828676	0.108952078	-7.192509206	0.009004535	1.085486755	0.605816428	-1.817897477	0.653363031	0.112831739	0.958438477	-3.985706389	4.5471E-19
AT5G17820	AT5G17820	2.487760009	0.144129396	-8.188938324	0.000131014	1.824611737	0.755895206	-3.37472158	0.20920389	-4.471522748	0.127102132	-4.286339255	0.022733502
AT5G59310	LTP4	-2.031894011	0.455347245	-8.302952078	0.005109255	2.625928537	NA	-3.241634265	0.282825999	-0.48409574	0.829112414	-4.528738643	6.71974E-07
AT5G54370	AT5G54370	-0.494255911	0.948827648	-8.453925212	0.005071859	-5.169138596	0.331642114	5.580983994	0.637815859	-1.951070151	NA	-8.719837362	0.01681949
AT2G39030	NATA1	-6.741861091	NA	10.55755306	1.17554E-08	-0.832506789	0.894311921			5.961970988	0.421936299	-6.323863896	0.0728361
AT5G59190	AT5G59190	1.640777043	0.397001499	10.05174463	3.26525E-09	2.363013728	0.267682908	-0.388119809	0.965013805	-0.403728739	0.931660636	-0.750209798	0.550291563
AT4G25590	ADF7	3.44014696	0.321996912	10.03738481	3.33835E-09	-1.739705102	NA	-4.005808992	0.721140128	5.425568429	0.510876298	-3.775794554	0.0871747
AT2G27300	NTL8	1.107881301	0.768795354	9.041741426	0.000768722	3.463830023	0.104820943	-1.078760771	0.907765638	1.125716644	0.752244106	-0.890921938	0.20676254
AT4G22960	AT4G22960	-2.444811134	0.452532265	8.986303638	4.08116E-08	0.111165603	0.979581166			1.654301106	0.666285275	0.756967404	0.496748679
AT1G58225	AT1G58225	0.6571154	0.930608464	8.887236281	0.001808165	2.517019472	0.190177154	0.38300602	0.982055475	-0.106084309	0.986774932	2.642833244	0.074983675
AT1G62620	AT1G62620	1.508508364	0.766743654	8.861677741	0.001451711	3.949340221	0.4695086	-4.502667498	0.691895802	-4.407483065	NA	1.770802759	0.794230514
AT2G07792	AT2G07792	2.169475529	0.431695435	8.822088645	0.000116829	-5.533204916	0.229165248	-0.48412716	0.974831553	-0.65655063	0.846410046	-2.506917876	0.236316688
AT5G07500	PEI1	5.384033117	0.344821053	8.751558204	0.001970098	3.75879835	0.13194949	0.392951439	0.982022128	-2.986069049	0.478385493	-1.880315828	0.208801423
AT5G61160	AACT1	6.86212614	0.212263471	8.693626533	9.13945E-06	-5.776119727	0.092553477	6.457049057	0.598901706	-7.028124527	0.074504799	4.443916637	0.333276402
AT1G54590	AT1G54590	5.659875004	0.318653886	8.611699357	3.03087E-05	-0.573621205	0.822038358	2.146366257	0.831161184			1.458585591	0.669447407
AT4G13390	EXT12	3.498267461	0.490153523	8.610570595	9.94292E-05	0.681921438	0.882898823	0.257155681	0.972897757	0.12021549	0.957812743	-0.604090614	0.784227322
AT1G32375	AT1G32375	1.356370906	0.71087012	8.557962507	2.67154E-05	-5.250633086	0.301871765			3.309319918	NA	0.357084601	0.886005509
AT4G05048	U49.1	0.961811656	0.833229279	8.510638433	0.000581738	1.369105786	0.144395263	0.21888041	0.987806306	-5.706172711	0.478838042	0.586352319	0.748441789
AT2G29470	GSTU3	5.891346668	0.297437494	8.43899575	0.004710105	1.620651512	0.792350154	-5.864386655	0.62738272	-1.471695535	0.802042097	1.385796246	0.55237472
AT1G49640	AT1G49640	1.288959899	0.855656945	8.317252082	0.010623301	2.169140867	0.593542315	-4.957504984	0.66808962	-0.759273921	0.896866257	0.878091659	0.865945075
AT5G44540	AT5G44540	2.074195235	0.714370669	8.260562631	0.008442189	-3.733182061	0.498163512			2.057388805	0.857006472	2.565146385	0.696978478
AT1G23070	AT1G23070	-1.786245442	NA	8.25446465	0.000180923	1.376594728	0.717076861	-4.404815965	0.697407195	0.650902178	0.929622352	-2.268712778	0.369854815
AT1G80330	GA3OX4	3.109031898	NA	8.199684283	0.00934296							1.687673863	0.804433334
AT3G09525	None	7.016375972	0.199836483	8.160072425	0.010791245								
AT3G17010	REM22	2.850624421	0.494334863	8.159353877	0.000478082			-7.024555019	0.477936046			-1.741657873	0.270451916
AT3G08700	UBC12	1.561580709	0.750979839	8.128788822	0.010898699	-1.739705102	NA	-4.944550507	0.66808962	1.019378839	0.92081979	0.591662031	0.858149424
AT3G13950	AT3G13950	5.359991114	0.346982987	8.058942107	0.000530346	-2.242707603	NA	-1.708998167	0.698438613	-2.817499604	0.060037937	-0.686729925	0.297007507
AT2G34030	AT2G34030	1.967876691	0.767391113	7.998463138	0.016002519	3.141438572	0.37587026	-5.026955832	0.66524984			0.422702331	0.891569281
AT4G21020	AT4G21020	0.881690398	0.90534663	7.957240433	0.01659938	4.31101523	0.087619577	-6.794447123	0.535600467	3.950755429	NA	-4.166025334	0.217405232
AT3G62380	AT3G62380	-3.344240056	NA	7.939955431	0.017240418								
AT3G57900	AT3G57900			7.921882914	0.001067237	-1.108056393	NA			4.410100294	NA	-1.422997025	0.833417297
AT2G35980	YLS9	3.415155738	0.213363593	7.869607152	0.01908896	1.795734795	0.689148452	-2.171266762	0.664635521	-2.113893564	0.466319315	0.396129924	0.744455405
AT3G06545	AT3G06545	5.132906385	NA	7.833462383	0.021987933			-6.17066845	0.612528262	-5.229958535	0.526108175	1.88108816	0.600347548
AT5G09570	AT5G09570	0.195875377	0.97974805	7.813102525	0.021774395			0.932411257	0.953923605	0.267914909	0.973023368	-2.139444919	0.475697024
AT3G02810	AT3G02810			7.747626948	0.001818054	-6.040818229	0.136876484	-8.280852268	0.1682865	-0.094567121	0.976380404	-0.564072541	0.645082103
AT5G51465	None	4.304716587	NA	7.719461662	0.029793594			-4.066128103	0.717709314				
AT1G09510	AT1G09510	1.507163248	0.681722244	7.689426602	0.031265386	-2.242707603	NA					3.258623214	0.604318632
AT4G04690	AT4G04690	5.962933292	0.215513755	7.642034114	0.033319906	0.826490155	6.83525E-06					0.394202244	0.034590358
AT3G08750	AT3G08750	0.530383059	0.754468472	7.631394958	0.003932306	4.689730363	0.059905985	-5.779635748	0.629761053	2.266887451	0.587484046	3.281648615	0.472440918
AT5G09610	PUM21	4.767702532	0.295408594	7.626672327	0.034678252	-4.97459813	0.353163742	4.639729649	0.685495817	0.705003978	0.905535933	-1.694961833	0.596870398
AT5G04010	AT5G04010	-1.954528275	NA	7.614098291	0.038109845	0.067638971	0.97926065	-6.398488808	0.312708513			-0.244471139	0.932835657
AT2G09575	None			7.58608023	0.035652868					-4.292127296	NA		
AT2G33175	AT2G33175	-0.151027957	0.972383736	7.55827556	0.037088229	0.790194057	0.898303395	-5.515150606	0.640704059	1.673381527	0.800724303	2.285084265	0.555246218
AT2G03130	AT2G03130	-0.726490551	0.852196368	7.54137282	0.040771183	4.739005022	0.379126869	-1.977019695	0.870136387	2.972692309	0.658556264	0.111230889	0.969252469
AT4G26050	PIRL8			7.469745871	0.045269589	2.068756928	0.448552304	-6.70921118	0.555633144	-4.02940546	NA	-2.554473889	0.697556879
AT1G07690	AT1G07690	5.305964226	0.352365134	7.46048422	0.005797856	-3.322614377	0.550728949	1.843404181	NA	-6.305133518	0.235635546	0.490633206	0.949628415
AT1G03390	AT1G03390	1.031874068	0.879443097	7.439944543	0.046358672	4.215339775	0.209826831					-2.95779581	0.217342421
AT3G62320	AT3G62320	-1.506767841	0.609728611	7.432497175	0.000627358	2.343708564	0.46540311	-1.03116372	0.942727755	0.357327362	0.956955597	1.16701509	0.356871381
AT2G07779	AT2G07779	6.89537479	0.133271315	7.402197845	0.010152388	0.398672036	0.841687919	1.882552228	0.87784337	1.963712658	0.53583656	-0.702871804	0.242214726
AT4G38540	AT4G38540	2.37960921	0.645274827	7.371895317	0.009669296	3.494190642	0.377766566	1.276666708	0.672844426	0.245942522	0.961811062	-0.722588693	0.683373259
AT4G14368	AT4G14368	-0.099677288	0.986439032	7.362468392	0.008889649			-6.053914434	0.616806988	1.212969762	0.878941396		
AT3G05390	AT3G05390	5.281942144	0.354468687	7.215875382	0.017501757	-3.967003132	0.468268694	0.69424813	0.96453852	-2.463948791	0.570854222	-2.633551706	0.066179631
AT2G34990	AT2G34990	1.500528253	NA	7.162261465	0.015130941	4.353075794	0.305648065	0.662966195	0.955198391	-0.589505279	0.905907141	0.839343976	0.774629089
AT1G15415	AT1G15415	1.845221362	0.784811824	6.979080804	0.035053475	2.039839478	0.52343868	-0.547715571	0.972495118	-0.405220616	0.902751377	0.273465684	0.875574127
AT3G11920	AT3G11920	-1.853887999	NA	6.919791061	0.029905458			-4.322140652	0.696374342				
AT1G71000	AT1G71000	2.080732261	0.681232123	6.792234566	8.3563E-07	2.520469661	0.398486503	-4.998542705	0.665832077	-1.174945221	0.885377618	-4.944694713	0.348215224
AT1G27565	AT1G27565	1.486881968	0.129430624	6.645871666	0.017746223	-1.002244599	0.332203311	-0.393886351	0.748978115	-3.683763643	0.162392805	-0.46496949	0.117521341
AT5G20370	AT5G20370	1.123376462	0.785130486	6.230332472	0.02079975	2.470993063	0.176795554	-5.250349016	0.653363031	-4.514293328	NA	2.783772109	0.091020719
AT4G12735	AT4G12735	-1.378185026	0.719902192	6.037803282	9.91857E-05	6.499492058	0.053751411	-1.661402244	0.709398459	-1.334335004	0.584854808	-2.391699206	0.069173637
AT1G26380	AT1G26380	0.959931673	0.857576039	5.92959048	0.00050281	-5.734666078	0.099439682	-1.134140619	0.806588363	-1.2689681	0.521243345	-0.133624489	0.931746173
AT4G27530	AT4G27530	3.824874127	0.302589432	5.670485411	0.001115507	1.179101745	0.611220998	-5.795986689	0.629389168	3.529999565	NA	-0.100878289	0.95198599
AT4G25330	AT4G25330	-4.1455653	0.305754839	5.647692813	0.003952861	0.110446441	0.979334313	3.688986812	NA	-5.513597564	0.503220327	-2.781647112	0.053641575
AT1G80660	HA9	2.04698384	0.566202108	5.549530169	0.009213207	-2.778225481	0.410194004	-0.239758644	0.978041029	-1.360008396	0.633384825	-0.405757998	0.825683711
AT3G05260	AT3G05260	5.595700078	0.324624779	5.501012586	0.001667327	2.354074329	0.317990601	-6.698703312	0.565728752	0.113292164	0.986818647	-0.32191421	0.751121995
AT4G08505	None	3.122116937	0.381683338	5.421353093	0.033426253			-0.317872625	0.96845901	-0.861116428	0.736648978		
AT1G48405	AT1G48405	2.141516294	0.249673509	5.411453872	1.50537E-05	1.004275527	0.664190606	-2.258321476	0.733077906	0.375183496	0.956151901	-0.018792091	0.9940385
AT4G14245	None	1.91256228	0.091176411	5.357471725	1.33695E-05			-0.370666169	0.961157568	0.746295071	0.630737501		
AT5G46350	WRKY8	0.30496286	0.886040777	5.342710708	0.001407286	1.449595062	0.455423923	-0.44172227	0.948830814	0.974839639	0.473734525	-0.645263887	0.180770772
AT1G01250	AT1G01250	1.706799341	0.156478103	5.264083095	1.90254E-06	1.647818978	0.06279882	1.973478568	0.841443038	-1.673616048	0.310305499	-0.824135076	0.662945246
AT4G18250	AT4G18250	-5.248535634	0.357811798	5.156625246	0.045033424	-3.329043115	0.364721283	-2.548537335	0.76337045	-1.850288349	0.446341993	0.614917408	0.458147011
AT2G19550	AT2G19550	1.299425617	0.821357297	5.148228147	0.001256846	2.010014527	0.726919248	-1.126106939	0.880545561	0.882601279	0.917278038	-0.343386433	0.830464898
AT5G24820	AT5G24820	3.091604174	0.308249228	5.117015969	0.025518965	2.284693494	0.123300642	2.168613681	NA	5.177053723	0.530853324	-2.974466693	0.645082103
AT1G75470	PUP15	7.183026459	0.093538699	4.943997	0.025792787	0.205376197	0.960445737					2.736233602	0.673857679
AT4G06265	None	0.393888623	0.922839875	4.906608322	0.015215539			-1.353245726	0.92566856	-1.693440069	0.694012377		
AT1G09500	AT1G09500	-2.22702939	NA	4.856309576	0.019054692	4.225750061	0.078790198	-7.829643901	0.107293781	6.071561746	0.391506399	0.368287965	0.883670512
AT3G21660	AT3G21660	4.376159931	0.227719436	4.74024024	0.00100443	1.626615796	0.187589073	-2.090930866	0.76022447	-1.507323252	0.816308593	-0.128607782	0.877625092
AT4G21930	AT4G21930	2.005520319	0.400008708	4.669014856	0.005748411	1.296981593	0.255914977	6.914071702	0.569286428	4.173384957	NA	0.210707912	0.915738486
AT2G26150	HSFA2	3.176502915	0.229241569	4.540571438	2.68159E-12	3.125662955	0.117855336	-1.077968769	0.622600949	-0.592810137	0.402596931	-0.280293525	0.47430986
AT2G02990	RNS1	-0.911157283	NA	4.533129669	0.018671743	1.311028306	0.74261784	-2.158137289	0.643189471	0.575337244	0.699972309	-1.045408954	0.157130759
AT2G01023	AT2G01023	7.055149731	0.053707258	4.522906255	0.042300675	2.092257998	0.251536347	0.826872723	0.935083701	0.879028628	0.745980368	0.816224491	0.454618181
AT4G34380	AT4G34380	0.46529905	0.880309423	4.473765724	0.017200806	1.496218947	0.632209012	-1.047888471	0.953801756	5.400055135	0.5094879	-1.615249493	0.608883797
AT5G06520	AT5G06520	1.183585624	0.36299122	4.458106783	0.019606877	5.210819997	0.326427657	-5.666393914	0.634935107	-5.170607409	0.531306852	4.91970834	0.209123886
AT2G43620	AT2G43620	-0.173645639	0.910922602	4.402008155	0.000900708	0.937704296	0.597700627	-0.830453894	0.628060145	-0.192918334	0.849369943	-0.20547668	0.843752657
AT3G26830	PAD3	-2.037249384	0.670358527	4.282598178	0.000576327	-1.027292101	0.671778857	0.847304148	0.879613371	-0.688459461	0.58137711	-0.983835436	0.202447364
AT2G07633	None	3.037996596	0.150201934	4.275820291	0.042649977			-1.030617758	0.910686362	1.158801324	0.59780496		
AT3G44440	AT3G44440	1.1669142	0.758964499	4.168616747	0.012514411	0.163902539	0.975088002	-0.984881347	0.949816063	1.578233534	0.733335658	-0.580994647	0.808215328
AT2G14060	AT2G14060	0.633425981	0.9331132	4.109461825	0.023848829	1.279349072	0.803437152	-5.902187641	0.627094298	-0.232839544	0.986774932	-0.386605932	0.955425129
AT3G51490	TMT3	1.332312923	0.271757967	4.071969312	0.006447846	1.226297084	0.279926563	-0.113849884	0.982749202	-0.307280936	0.927080141	0.805002174	0.251452315
AT3G52160	KCS15	1.842016498	0.660693126	3.923677446	0.031154286	0.344396733	0.789753602	1.961439972	0.791221745	-0.376815761	0.980488802	1.820606866	0.195012386
AT2G36780	AT2G36780	1.056098652	0.317451005	3.846413633	6.10587E-09	0.757276021	0.327377967	0.563299144	0.784493718	1.239958613	0.659063827	-0.715233454	0.378224865
AT3G11050	FER2	0.39805886	0.881154347	3.804260201	0.007317858	1.579801398	0.524433406	-1.186860884	0.797614335	-0.744249337	0.431964416	-0.109347239	0.854629678
AT3G29140	AT3G29140	1.628501395	0.101496146	3.750543021	0.004146928	1.342756215	0.82591609	1.051911042	0.683841765	0.123859487	0.934309657	0.531671958	0.687397949
AT2G04050	AT2G04050	2.429050821	0.536501593	3.691440777	0.000291572	1.019717193	0.813793623	0.842481283	NA	-0.843215147	0.601517674	-1.98049557	0.225300835

AT1G71490	AT1G71490	-1.151856406	0.387649756	3.648900006	0.013663852	0.756683104	0.42862352	0.705881949	0.761894565	0.33773899	0.894710906	0.53684448	0.193826961
AT3G15870	AT3G15870	2.357259136	0.537751357	3.554501066	0.022374548	-0.801367169	0.872476419	-6.570821677	0.066666426	-3.293649404	NA	1.305006849	0.81620306
AT4G23140	CRK6	-1.570865416	NA	3.546554137	0.002078648	1.48028047	0.786356558	-5.083879065	0.664075844	4.524658393	NA	-0.817300687	0.853140757
AT2G39200	MLO12	0.043674417	0.978101885	3.510365496	2.71174E-05	-2.007501792	0.307898602	-2.198313316	0.107439117	-0.72730021	0.391455509	-1.092349881	0.072502773
AT3G63050	AT3G63050	1.542160875	0.085427997	3.501510479	0.001137946	-0.033293431	0.981716626	1.176803556	0.71171834	-0.514237607	0.725933128	-0.942565369	0.173954722
AT5G25250	FLOT1	0.017871449	0.991557397	3.498276439	0.000671112	-0.333838818	0.875638618	-1.367382235	0.73461774	-0.512605879	0.571762044	-0.56744334	0.132132705
AT1G19565	None	0.273970195	0.924149011	3.473923232	0.023932705			0.03923966	0.996799452	1.741799184	0.587077522		
AT2G24535	None	3.006465564	0.249673509	3.473087133	0.036704747			1.190416513	0.627094298	-0.080875185	0.985585094		
AT5G17540	AT5G17540	-0.51381423	0.714394678	3.472712478	0.006713501	-0.969777942	0.412436653	0.549618079	0.828216935	0.424134479	0.796109622	1.219424108	0.099065645
AT5G48400	ATGLR1.2	1.865562287	0.3663105	3.433587328	0.020540942	0.916808485	0.755105263	-2.351178212	0.785453357	-0.844246584	0.838103967	1.003693092	0.364601396
AT5G25340	AT5G25340	0.464738856	0.715216915	3.095817093	0.039672326	1.904531855	0.195248923	0.235730341	0.972495118	0.820747428	0.64687986	-0.730490805	0.655577041
AT3G13672	AT3G13672	1.694491791	0.18847387	3.081060095	0.029269086	1.625859438	0.088005965	1.268212826	0.889744692	2.502704237	0.485888437	-0.49528892	0.741101936
AT5G61800	AT5G61800	1.437543642	0.234443151	3.022174376	0.000238673	0.734153622	0.492605162	-0.559700432	0.792939242	0.348082985	0.74754675	0.553391239	0.211790483
AT5G15720	GLIP7	2.163017273	0.279837848	3.007605322	0.002733586	2.555028235	0.055674222	0.286682436	0.980517937	0.473526286	0.798358357	0.628984041	0.224160742
AT1G20120	AT1G20120	1.947618192	0.29757313	2.968081713	0.049867121	-0.437129067	0.853387127	-0.627964108	0.953801756	1.14974478	0.855064321	-0.335279415	0.828599975
AT2G07638	None	1.136266702	0.194600672	2.908046114	0.000724554			0.00496285	0.998189671	0.380205732	0.727051106		
AT1G18990	AT1G18990	0.888251463	0.129035275	2.902677164	0.029930683	-0.064136027	0.919342834	-0.316997268	0.967195835	-0.962646412	0.67431887	0.153796715	0.863776511
AT5G42053	AT5G42053	1.676836502	0.626732032	2.82285624	0.007275354	-4.699898541	0.383290772	-6.015635582	0.619462504	3.263608744	0.69628703	0.478352871	0.850828754
AT1G42980	AT1G42980	0.948044861	0.335838769	2.819973046	0.006645821	2.221744388	0.099193853	0.666310608	0.928193798	-0.733992645	0.875543097	0.426341632	0.852645628
AT1G69485	AT1G69485	1.349460458	0.578486749	2.707667669	0.011705073	0.692742253	0.70478638	0.018335827	0.996867494	-0.140829664	0.976616225	0.222256491	0.765355438
AT4G11370	RHA1A	1.353054116	0.541490552	2.705653627	0.00226133	-0.513500217	0.591313089	-0.409667732	0.968021479	1.019150408	0.554239483	0.536832587	0.2144834
AT1G61860	AT1G61860	1.152808667	0.567362167	2.659313327	0.000723271	1.56290328	0.499013132	0.756284151	0.735695447	-0.891332336	0.403366939	-0.381063954	0.592827466
AT4G14140	DMT2	1.425224324	0.144177164	2.591659515	2.61889E-05	0.934164543	0.000350978	-0.521597598	0.784580159	-0.072563214	0.885996223	-0.892465396	1.76697E-12
AT4G37022	AT4G37022	2.071614747	0.265539273	2.558547939	0.018671743			1.050647517	0.841139133	-0.067866802	0.985120284		
AT5G04390	AT5G04390	1.535836367	0.641503812	2.550049607	0.034828935	2.365167841	0.125013349	-0.11338303	0.990915497	-1.240227731	0.812372215	0.441705212	0.892850348
AT1G02930	GSTF6	-0.313647829	0.599326551	2.502423729	7.04085E-13	-0.38896109	0.583345091	0.175104743	0.922005452	-0.975524473	0.057932212	0.224468342	0.463712364
AT1G71330	NAP5	2.103026091	0.190072871	2.482526387	0.00037591	0.98271778	0.055778913	-0.221228287	0.936427636	-0.307899259	0.869846959	0.099391985	0.768357562
AT4G18360	GOX3	2.25349753	0.088949145	2.446746997	0.001230479	2.251223291	NA	-0.906555571	0.758815467	0.280001389	0.570563609	-0.781365897	0.000323553
AT5G43450	AT5G43450	0.716967208	0.210828242	2.421288513	1.88173E-11	-0.16210043	0.625631498	1.331897257	0.54194953	-0.141117756	0.894043142	-0.155144145	0.671003167
AT5G24990	AT5G24990	2.037814976	0.242515109	2.389386037	0.000785739	0.422186646	0.709487451	0.144610676	0.944841108	0.634162819	0.521243345	-0.437314564	0.164994868
AT5G15950	AT5G15950	0.869050735	0.013733925	2.367279604	3.46163E-10			-0.62595926	0.675091988	-0.00907036	0.99699153		
AT1G10585	AT1G10585	-0.821042358	0.738096056	2.327759317	0.013563359	0.708576272	0.684938996	1.996227219	0.517002883	1.657729325	0.506340162	-1.070137546	0.343850552
AT4G23260	CRK18	-0.560898105	0.69868698	2.303678805	0.023760461	0.192099734	0.915828386	-0.392717956	0.954594754	-0.405153989	0.830164664	0.364593207	0.652967659
AT1G29195	AT1G29195	2.146531167	0.270083636	2.297356997	0.036537141	2.761203364	0.05004959	-1.211085946	0.73148555	-0.065788182	0.950790869	0.154391154	0.536318417
AT5G01595	AT5G01595	2.306054757	0.228469833	2.287382022	0.013239664	-0.2367335	0.924206514	-0.869351765	0.860561763	-1.247793658	0.711808808	1.466552313	0.180011513
AT1G73510	AT1G73510	0.166918921	0.918137227	2.235612149	0.003600908	2.457389622	0.161744548	-0.086468862	0.994162268	0.486991837	0.936190346	3.335325536	0.173548321
AT5G60250	AT5G60250	-0.876645291	0.36662449	2.235282122	0.000532505	0.549823963	NA	0.922980896	0.626700603	-0.268476732	0.871060407	0.312561054	0.705077442
AT2G29300	AT2G29300	-0.202699847	0.811239301	2.20643049	1.6554E-17	0.969085623	0.028328722	4.127709343	0.084005684	0.878038371	0.721384039	0.540105562	0.483524773
AT1G07390	RLP1	1.060927815	0.330872999	2.200663889	0.00021792	0.666305596	0.529305469	0.743705749	0.477936046	-0.542383743	0.550565037	0.129750397	0.829832959
AT5G01760	AT5G01760	0.279471007	0.885975526	2.195463783	0.02378589	0.071626171	0.977623147	-0.307475083	0.967195835	1.253036666	0.578542261	-0.705313707	0.368317217
AT3G48080	AT3G48080	0.9431051	0.478263594	2.152190045	0.039386936			-0.291755821	0.891886587	-1.494030522	0.073794382	0.850203234	0.905488956
AT4G39930	AT4G39930	0.284172803	0.857108424	2.150378117	0.018801768			0.703165108	0.911737107	-0.482563716	0.796390162	-4.13023522	0.490173832
AT3G25882	NIMIN-2	-0.508231372	0.691364274	2.113247039	0.017566366	-0.97147212	0.451302713	0.835974694	0.718095285	1.573308005	0.137320551	0.267960409	0.645082103
AT3G63380	AT3G63380	0.719855656	0.603543328	2.0722118	0.011233315	0.107425689	0.946876609	-1.620350098	0.619569853	-0.95984677	0.220100806	-0.673556524	0.143547356
AT1G02920	GSTF7	0.668131115	0.149313252	2.0613087	9.03895E-09	0.3957592	0.563907217	-0.470805181	0.774132304	-1.311163768	0.059053305	-0.309935262	0.071870766
AT1G16515	AT1G16515	1.351163663	0.558957823	2.020137582	0.029816467	0.899431122	0.374879228	-5.921865952	0.054617623	0.027891576	0.995441244	0.124046683	0.847313457
AT1G74410	AT1G74410	0.43581823	0.800627102	1.981214247	0.018891234	0.321352328	0.527319243	-0.08536684	0.978345558	-0.609521328	0.538214564	-0.818539847	0.000776063
AT3G24090	GFAT	0.759721204	0.257274442	1.976239997	0.0001824	0.435904996	0.601692303	-0.819167759	0.465456099	-0.346395157	0.58538838	0.049914355	0.891092995
AT5G11110	SPS2F	0.922737266	0.079386981	1.975181762	5.32782E-06	0.744051981	0.045695282	0.293610523	0.726900342	-0.142930386	0.801610644	-0.055869942	0.866159689
AT3G60160	ABCC9	2.072224099	0.22890752	1.964767858	9.66385E-05	3.949340221	0.4695086	0.02090229	0.994744545	0.463644064	0.465831089	0.310656294	0.785214861
AT3G05936	AT3G05936	0.821463416	0.550844695	1.963001904	0.005610679	1.150092565	0.255640358	1.582434185	0.675671944	0.649014325	0.881456747	0.074934988	0.966021038
AT5G67090	AT5G67090	0.069199269	0.968341417	1.944896518	0.036270445	1.696323959	0.216369157	-0.732230088	0.602123416	-0.866699649	0.306248333	-0.158618318	0.739424609
AT3G56320	AT3G56320	0.917694158	0.336008023	1.925154369	0.006050668	0.116248667	0.902528554	0.010238948	0.997285903	-0.391197577	0.594464993	0.114487542	0.753332443
AT2G41250	AT2G41250	0.975534842	0.007083849	1.923903916	4.78729E-10	0.418070917	0.22133675	0.385581212	0.706790319	0.50725218	0.083004186	0.246693315	0.288351697
AT1G25277	None	0.375294113	0.813748902	1.902676202	0.036648601			0.732854997	0.828875452	2.087337105	0.292437744		
AT1G64795	None	1.143776864	NA	1.897310862	0.038838758					0.108018157	0.966304751		
AT5G18780	AT5G18780	1.284900146	0.210052814	1.89224239	7.84111E-05	0.661226421	0.261286068	0.016008574	0.993482731	0.253816435	0.683019892	-0.852374299	0.004202111
AT4G34650	SQS2	0.001850502	0.998469772	1.890547825	0.003840374	0.319056509	0.617444015	0.248862098	0.914676286	-0.088976778	0.939595436	0.319998902	0.096379536
AT3G60520	AT3G60520	1.102266078	0.088000298	1.888324185	8.20307E-07	0.862907776	0.002441562	0.46455708	0.521675277	-0.079948037	0.927507051	-0.77936946	0.030215018
AT3G22450	AT3G22450	1.512350696	0.203459673	1.881204747	0.006276164	0.249999561	0.769497413	-0.170027518	0.936758504	0.138559392	0.951101524	0.578452467	0.240010196
AT3G44880	ACD1	0.706337229	0.093234319	1.875384748	6.96692E-12	0.887941033	9.84314E-08	0.450160076	0.721140128	-0.102952062	0.859129728	0.016792115	0.946507734
AT2G31751	AT2G31751	1.125229856	0.580518675	1.844147584	0.005518082	0.699641173	0.631221594	0.441289514	0.865378204	0.62520381	0.620540544	0.459721341	0.588654565
AT5G48780	AT5G48780	0.659112129	0.64032794	1.842893824	0.000541004			1.021494637	0.697407195	-0.190376471	0.923637745		
AT3G13880	AT3G13880	1.118985262	0.179750776	1.836595255	0.01629882	0.116140783	0.919188513	-0.086994051	0.975907193	0.658461724	0.55787595	-0.165773247	0.718407881
AT3G14070	CAX9	0.268919474	0.762990932	1.83303057	0.002913985	0.66907188	0.265081388	0.483920734	0.67307941	0.522270267	0.608503481	0.007597895	0.990196214
AT5G58660	AT5G58660	3.980703991	0.069956322	1.81681512	3.55173E-05	0.781849571	0.245372073	0.793613838	0.393926565	0.175260527	0.851018739	-0.739379218	0.076561769
AT4G27560	AT4G27560	1.76931104	0.266790951	1.801075784	0.001670069	0.521318313	0.742924267	1.03285848	0.647947756	0.656315211	0.613826202	0.333459086	0.368359667
AT3G03900	APK3	0.19385151	0.82958108	1.800961569	6.75652E-06	0.258045789	0.699078313	-0.286100017	0.908903757	-0.11600356	0.914111276	-0.026458761	0.946066841
AT5G62440	AT5G62440	0.866304588	0.047237683	1.795516055	3.75237E-12	0.736444599	0.015285549	-0.049510873	0.972495118	-0.213992671	0.54988571	-0.335877565	0.153901063
AT5G01700	AT5G01700	1.396347374	0.127887509	1.789988793	0.000391547	1.084265371	0.078887661	1.575140813	0.405917627	-0.876440379	0.143920603	-0.565614974	0.12609117
AT1G28765	None	1.805511558	0.203459673	1.78145714	0.00035891			0.230376115	0.953275639	-0.089808993	0.934609223		
AT3G05030	NHX2	0.693986295	0.180334137	1.779022346	0.000583229	0.467984254	0.198591513	0.001088896	0.999848181	-0.427641507	0.511659422	-0.505245039	0.019255075
AT3G58270	AT3G58270	0.263176618	0.861217048	1.767299236	0.014181774	0.855588128	0.263287397	-0.23952267	0.878942115	0.364971611	0.672847718	0.164357758	0.583130146
AT4G22270	MRB1	1.139420568	0.168180097	1.743172988	0.041789495	0.676474647	0.336079208	-1.450528171	0.482795025	-0.633874389	0.434411297	-0.738188333	0.029321406
AT1G13750	AT1G13750	0.711407902	0.341594178	1.739733104	0.004652437	0.915949019	0.384063356	0.621632486	0.69767913	-0.41215762	0.691085481	-0.465434476	0.418843278
AT1G62760	AT1G62760	1.367828733	0.160509979	1.712994466	0.04380177	-1.047427961	0.841279007	0.184886482	0.977477438	0.157267723	0.925076802	-3.242539869	0.095601495
AT5G57610	AT5G57610	0.888758007	0.041027076	1.698983924	3.81407E-10	0.684346915	0.137523256	0.200290622	0.893309057	0.226946275	0.728271334	-0.358095368	0.113372186
AT5G49110	AT5G49110	-0.204217359	0.822325282	1.693933684	0.007661377	0.048322058	0.947142532	0.462417422	0.731041294	0.252040483	0.820557135	0.532884956	0.092146119
AT2G20770	GCL2	0.288415152	0.710470684	1.681796049	0.018845441	0.409319804	0.50294384	-0.51601311	0.784628962	0.030047561	0.984765765	-0.581198419	0.04825321
AT5G40540	AT5G40540	0.622928134	0.229241569	1.676446869	4.3406E-05	0.53907536	0.145009892	-0.38278537	0.716554635	-0.319351597	0.555532957	0.288048846	0.281620971
AT5G59720	HSP18.2	2.130045457	0.2347693	1.669878841	0.023551565	-0.541033214	0.730316875	-0.298923137	0.968550892	-0.912975491	0.615893075	1.070775509	0.09742893
AT1G30500	NF-YA7	1.155456285	0.056423958	1.66674424	3.46876E-09	0.95929162	0.000133818	0.641250866	0.696184854	0.239236683	0.661209489	-0.493345686	0.022351386
AT4G18050	ABCB9	0.731711084	0.468749773	1.666009396	0.008556074	0.588048556	0.457597559	1.134629667	0.602119156	-1.615697673	0.478838042	-0.291323147	0.710648121
AT1G01715	None	0.401334712	0.618596856	1.664621578	0.001917418			0.044840906	0.986941732	0.418635203	0.606718088		
AT5G57670	AT5G57670	1.566900948	0.087393434	1.660745044	1.67996E-05	0.975486844	0.346083359	-0.667481569	0.647435191	0.442953077	0.092085165	-0.696943432	0.000377038
AT1G74360	AT1G74360	0.852485093	0.358050962	1.655152082	0.035852951	-0.283514612	0.740577152	-1.646769132	0.592459968	-0.580223071	0.686941393	-0.779421963	0.071870766
AT2G37150	AT2G37150	0.821681202	0.066564866	1.650107528	3.12976E-09	0.971272892	0.006944721	0.105632717	0.962872831	0.009231523	0.992281921	-0.234940481	0.319050291
AT4G35070	AT4G35070	0.721268117	0.249529687	1.647046211	0.001386246	-0.488264816	0.461929638	-0.377506472	0.840363187	0.510590073	0.396580875	-0.248843311	0.546123231
AT3G17770	AT3G17770	0.633865438	0.081741312	1.63930206	2.18442E-06	0.814398401	2.31752E-05	0.683943579	0.616300683	0.041597399	0.943490436	-0.243614206	0.222853666
AT2G31865	PARG2	-0.631236392	0.585655556	1.636550783	0.000244014	-0.660384173	0.511946084	-0.776102023	0.736215855	-0.432741704	0.584002075	0.383235218	0.515273935
AT2G18690	AT2G18690	0.972836425	0.065173314	1.629144218	0.005244518	-0.318189888	0.812523804	-0.469191103	0.792190337	-0.214198639	0.710391771	-0.694905287	0.104492093
AT5G16980	AT5G16980	1.211326752	0.057967662	1.621730412	8.26681E-05	-2.964401061	NA	2.192784937	0.437147152	1.187848646	0.07338077	-0.836044107	0.906589267
AT3G04030	MYR2	0.901876425	0.252859513	1.601476743	0.005065829	-0.309316652	0.657915915	0.266223467	0.916448617	0.347559498	0.781013423	-0.21837097	0.692641673
AT4G22530	AT4G22530	0.70557977	0.395502926	1.601063523	0.003906336	-0.195036135	0.846150848	-1.062473465	0.62738272	0.648227156	0.201027557	0.132959948	0.679319715

AT5G11650	AT5G11650	0.990291387	0.023370912	1.597824588	2.60092E-06	0.637652394	0.055363573	-0.340007557	0.742017486	-0.26251654	0.587443596	0.758749771	7.14638E-06
AT2G38860	YLS5	1.25799599	0.099708941	1.584718697	0.003476282	-0.129298183	0.898014022	-0.100738534	0.972495118	-0.423013897	0.445365274	-0.382606472	0.04392724
AT5G60680	AT5G60680	0.87850344	0.044418008	1.583713832	5.43339E-11	0.122916297	0.381625153	0.467381601	0.705076831	0.056061596	0.883729772	0.209478857	0.207735782
AT3G09350	Fes1A	1.357239572	0.058047998	1.579743979	5.40644E-06	0.671424118	0.047811077	-0.349538178	0.820002489	-0.387045064	0.324458776	-0.139419976	0.667523292
AT1G69870	NRT1.7	-0.559679541	0.220918623	1.57435153	9.24498E-08	-0.100036024	0.813769755	0.984611807	0.576080473	0.13202462	0.868188276	-0.117080208	0.799688042
AT5G49990	AT5G49990	0.882614657	0.04599506	1.570648885	1.5153E-08	0.999637926	0.000824156	-0.407328503	0.66501717	-0.421685085	0.252182317	-0.277279842	0.127354714
AT1G71140	AT1G71140	0.408717426	0.745484465	1.565413437	0.041445482	0.932642752	0.314502879	-1.353657248	0.736971614	0.648809283	0.639966929	-1.260383709	0.127038738
AT1G12880	NUDT12	1.236738949	0.13854803	1.559390447	0.036149169	1.012910067	0.194487755	0.607876011	0.674766805	0.374391197	0.582751486	0.606651499	0.217793935
AT3G06355	None	0.22681949	0.567479587	1.540401353	1.08043E-16			-0.048647619	0.969982605	0.577550621	0.006836936		
AT5G17860	CAX7	0.996698157	0.03589308	1.533855741	4.8554E-05	0.84641096	0.003867394	-0.065296961	0.979221735	0.047523833	0.961513155	-0.723184542	0.008224341
AT5G24940	AT5G24940	0.20577934	0.894211551	1.519844478	0.006062291	2.355189813	0.427272877	0.49569577	0.883495624	0.011904191	0.997309584	1.132564147	0.803690594
AT2G21320	BBX18	-0.120058081	0.914137352	1.505430729	0.019478763	-0.364945388	0.565509307	0.743510163	0.666155455	0.387533784	0.675282792	0.467622996	0.153339884
AT2G35940	BLH1	0.863425217	0.022156048	1.501593373	3.13393E-06	0.79237027	0.00068481	-0.523616415	0.621238589	-0.157680981	0.766314337	-0.671191521	9.35716E-07
AT5G20360	Phox3	0.210133805	0.721436768	1.498897093	0.000789795	0.108467654	0.881566622	-0.252814459	0.776796299	0.110079185	0.830492367	-0.538255982	0.006564198
AT5G64750	ABR1	0.91174913	0.222748577	1.495834011	0.024138692	1.129843978	0.132693727	-1.854243373	0.606948021	-1.632037062	0.428050845	0.086999381	0.87709707
AT2G45630	AT2G45630	0.972429889	0.012258275	1.488162244	3.95721E-05	0.683121846	0.143697597	0.129022525	0.941364115	-0.226887571	0.767529325	-0.252916741	0.344773491
AT5G39350	AT5G39350	0.618447338	0.556575072	1.468302807	0.022713219	-0.397131876	0.665759743	0.061180853	0.987325629	-0.296206943	0.761175288	-0.086035447	0.838551375
AT1G13960	WRKY4	0.867375591	0.0173683	1.462492147	3.97497E-09	0.908855368	1.58448E-06	-0.046264986	0.978097752	-0.127958171	0.743086296	-0.726963627	9.81999E-06
AT5G00980	None	1.309433826	0.24887026	1.45962466	0.029021765			0.401459589	0.800300732	0.245031192	0.82723652		
AT2G03500	AT2G03500	0.884567798	0.274218637	1.457983782	0.007298128	0.124939619	0.864902199	-0.323771076	0.957410897	-0.587982944	0.288981927	-0.044555281	0.929204909
AT5G37440	AT5G37440	2.07734316	0.188061692	1.452312984	0.040670147			-0.217207311	0.947082547	-0.600716766	0.556508441	1.81197656	0.789006341
AT2G26980	CIPK3	0.733132965	0.00885749	1.446755599	1.05659E-06	0.788827956	4.53056E-06	0.007860376	0.994744545	0.07178085	0.792838833	-0.629980507	3.86061E-06
AT5G19850	AT5G19850	0.294625637	0.674289419	1.446323747	0.001083597	-0.188273047	0.737410573	0.750065658	0.575078848	0.133751561	0.919527178	0.459984407	0.198399825
AT4G05050	UBQ11	0.993275581	0.010749695	1.438290913	6.02643E-12	0.560369184	0.00023779	0.586031686	0.66808962	-0.229420982	0.226076733	0.305738923	0.013495176
AT5G52570	BETA-OHASE 2	-0.117124147	0.891259982	1.43277012	6.80349E-07	-0.66849435	0.002844999	1.901665291	0.378972356	0.340748305	0.643058093	0.154087275	0.805652245
AT5G37540	AT5G37540	0.596774113	0.280698343	1.424478851	1.92981E-06	0.959545064	0.047942211	0.115812368	0.962872831	-0.727949415	0.013281256	0.908856319	0.000201173
AT1G14760	KNATM	0.713052592	0.405713922	1.423339431	0.028795486	0.868423121	0.205162635	0.836239371	0.587695712	-0.127907438	0.911423117	0.910110825	0.022249755
AT1G26770	EXPA10	0.385969512	0.431446354	1.41751151	0.000142111	0.622105427	0.038419885	-0.464487485	0.637614678	0.032056831	0.965131427	-0.193015417	0.235576194
AT5G17850	AT5G17850	0.997924297	0.047133077	1.41713909	0.004482885	0.422009253	0.460384622	-0.783449919	0.646729152	0.102956259	0.924808173	0.045442638	0.891699275
AT5G49480	CP1	0.975319201	0.001457164	1.413400468	5.08941E-05	0.735145698	0.010060068	-0.411547075	0.585524552	-0.227774876	0.554212839	-0.425676191	0.01527186
AT3G09370	MYB3R-3	0.904330945	0.099483635	1.407316794	0.00065708	0.126255961	0.748449443	-0.382877493	0.727618493	0.288281732	0.602615521	0.376886263	0.073257956
AT3G47360	HSD3	1.253967982	0.095674039	1.406697069	0.008686612	-2.648405744	NA	-0.087356903	0.965260868	0.476610016	0.302990007	-0.47206228	0.804433334
AT1G17940	AT1G17940	0.957178606	0.065793162	1.405330955	4.95492E-10	0.039330578	0.919777761	0.436912367	0.69015436	-0.47061469	0.119988448	0.15671691	0.547774688
AT2G35635	UBQ7	0.913962482	0.000264698	1.396122113	1.3013E-23	0.634588654	0.000154451	0.630915637	0.477936046	0.233524376	0.363015761	-0.168773884	0.335772657
AT4G33660	AT4G33660	1.348476797	0.055525627	1.395322014	0.004357867	0.904845973	0.005875282	1.037042833	0.67307941	-0.061856889	0.97724437	0.047690623	0.925942539
AT5G17300	RVE1	0.841320132	0.117602214	1.393820279	4.28882E-06	0.98156352	0.001297453	0.985391593	0.598901706	0.01693889	0.984002268	0.118288035	0.693072272
AT5G57840	AT5G57840	0.464018182	0.610990127	1.389721924	0.013234408	0.42155543	0.623373261	-0.288238472	0.797614335	-0.272550059	0.65255211	-0.822379294	0.008721078
AT1G66760	AT1G66760	0.801876047	0.053722653	1.389131584	2.24112E-06	0.076632059	0.841279007	0.66055271	0.598901706	0.099990942	0.819611825	0.6618228	1.66627E-06
AT5G47560	TDT	0.842239466	0.013719068	1.387051027	1.0542E-12	0.892974699	4.7911E-14	0.426422827	0.715935596	-0.32276295	0.084810184	-0.888344224	5.25903E-07
AT2G43930	AT2G43930	1.561425428	0.05470192	1.387028959	0.040989619	0.970729204	0.017121661	0.84783199	0.66524984	0.719254617	0.488543831	0.216153514	0.716482664
AT5G17380	AT5G17380	0.815203097	0.009568386	1.383284207	1.22104E-05	0.739545832	0.000802994	-0.373512576	0.642268593	-0.406720955	0.099807084	-0.516824678	0.001205659
AT5G64170	AT5G64170	0.535638614	0.326475206	1.377653783	7.60687E-07	0.653253771	0.00319107	0.997164787	0.565728752	0.893948865	0.000355122	0.235216715	0.22650191
AT5G01940	AT5G01940	1.238939012	0.129866853	1.377048218	0.035749265	1.255417069	0.109803671	0.806646314	0.514675172	0.562587167	0.570610039	-0.771394346	0.003079486
AT5G20070	NUDX19	0.739559323	0.043632351	1.37673959	2.29414E-05	0.286998889	0.418120427	0.136593486	0.924925439	-0.171820573	0.671308439	0.306597804	0.057221095
AT3G17365	AT3G17365	0.212852944	0.777202177	1.375861039	0.000134994	0.31996975	0.413578654	0.041986598	0.982734229	0.804360275	0.008266817	0.265447835	0.176081
AT1G16960	AT1G16960	0.329977234	0.69939111	1.368613631	5.71492E-05	0.373272264	0.514663546	0.865569096	0.632278424	0.309637068	0.820612674	0.392863375	0.558624855
AT1G02390	GPAT2	-0.918564312	0.178844627	1.364206254	0.002212243	0.169694066	0.801048288	0.207044719	0.94636964	-1.004995658	0.614088055	-0.639906217	0.247347621
AT4G27520	ENODL2	0.630733577	0.183888105	1.362905373	1.36313E-05	-0.345743672	0.215870646	0.065487097	0.980782333	-0.569682492	5.45009E-05	0.283347781	0.130760666
AT1G19180	JAZ1	-0.138471126	0.837555951	1.362043394	0.000336545	-0.143046224	0.763432294	-0.020799886	0.993556469	-0.304478894	0.482909192	0.554407137	0.001045436
AT1G02680	TAF13	0.778326834	0.211869621	1.350001112	1.56288E-05	0.724689683	0.000851131	0.637242527	0.695103554	0.16353691	0.785089338	0.179579839	0.592895838
AT1G28360	ERF12	0.736914593	0.382562585	1.349335606	0.041373204	0.398599328	0.605031759	-0.052092641	0.982749202	0.508523741	0.412791131	0.456615039	0.083779147
AT5G55050	AT5G55050	0.738631968	0.331000245	1.345783748	0.024803866	-0.496905001	0.39549612	-0.953688644	0.583897164	-0.323112079	0.722436301	-0.620286178	0.097116132
AT2G20495	AT2G20495	0.065555052	0.944004743	1.345745924	0.046163806	0.220094879	0.68706916	0.045463736	0.982356953	-0.328990476	0.676169382	-0.053442931	0.87784809
AT4G19450	AT4G19450	0.688052745	0.088466161	1.34290126	3.10008E-05	0.835491954	0.002125462	-0.093868655	0.933605124	-0.036140229	0.963495178	-0.14330064	0.591230596
AT1G66230	MYB20	0.555806559	0.445244033	1.342018123	0.010763377	-0.523030591	0.253832801	0.295295585	0.868407292	-0.126725884	0.913903461	0.451488957	0.034271823
AT5G20900	JAZ12	0.855794443	0.008337432	1.337117286	9.93292E-07	0.646698525	6.96771E-05	0.089620168	0.953657757	-0.168157505	0.554658146	-0.073978041	0.690682962
AT4G03960	PFA-DSP4	1.575082472	0.142708485	1.333011458	0.04710896	0.041247055	0.962926772	0.311914351	0.776796299	0.385590617	0.558208241	0.387295743	0.244670484
AT5G02320	MYB3R-5	1.271910993	0.161607205	1.325795029	0.000213457	0.741919867	0.024545642	0.589080939	0.680492035	0.263738065	0.624504205	0.083880142	0.806930655
AT1G80190	PSF1	0.88666773	0.065320716	1.319805072	7.26443E-08	-0.893688739	0.018535123	1.661792975	0.319520931	-0.180124701	0.786685207	0.1554839	0.576053987
AT4G22240	AT4G22240	0.068630353	0.879268267	1.316596436	2.35246E-05	0.364821595	0.164605855	0.039914486	0.980438692	-0.02566018	0.962199154	-0.377589681	0.073247983
AT5G10730	AT5G10730	0.968558829	0.062384104	1.311604365	0.000409554	0.306594078	0.425625631	0.356971227	0.631596962	0.144037848	0.832082438	0.200773814	0.393499062
AT1G69800	AT1G69800	0.685822693	0.178314825	1.311175277	1.14818E-05	0.904314396	0.00333019	0.22287571	0.804934387	-0.042507588	0.949933942	0.138943049	0.51472562
AT4G20830	AT4G20830	0.384780485	0.516312632	1.307589755	2.4304E-06	0.059679138	0.833049958	-0.445862338	0.532231419	-0.546664825	0.125191522	0.007078251	0.979157323
AT2G14260	PIP	0.732060011	0.079361915	1.306198527	5.02768E-11	0.499375561	0.015109797	0.728953107	0.596083291	0.267921557	0.314370878	0.232678434	0.109738814
AT5G18670	BMY3	0.940596861	0.049990711	1.30526299	0.001127964	0.428053412	0.031214788	0.041610735	0.984064159	-0.319268193	0.374494987	0.062519795	0.788446251
AT1G03610	AT1G03610	0.717680328	0.112690979	1.303145118	5.18166E-16	0.953913644	1.90652E-11	1.299008885	0.409171348	0.023629825	0.968001113	0.031393336	0.905468207
AT4G40042	AT4G40042	0.812069761	0.161484165	1.293742068	0.01984281	-0.131328823	0.801150944	0.464087148	0.672339447	0.24542747	0.704545524	0.063555105	0.889701819
AT2G41000	AT2G41000	0.270063723	0.676270318	1.289082043	0.016667004	0.364108025	0.551794316	-0.104007343	0.951485287	-0.128535503	0.921453678	-0.237977313	0.399793156
AT1G14230	AT1G14230	0.477785403	0.279837848	1.288782463	1.95863E-06	0.512089855	0.053944929	-0.292009012	0.640704059	-0.06468096	0.918766875	-0.473041751	0.008685512
AT3G52260	AT3G52260	0.786887018	0.129564491	1.285353193	0.002303958	0.981057	7.03494E-05	0.246654809	0.812177746	-0.154140346	0.769619898	0.205520611	0.408594621
AT1G26660	AT1G26660	0.678581632	0.302165345	1.280248616	0.005071081	0.623779865	0.090137346	-0.231554083	0.874677913	-0.046559579	0.957434731	0.196784915	0.376295368
AT5G47880	ERF1-1	0.888521859	0.018547659	1.272520455	1.06704E-05	0.987255178	1.00449E-05	-0.272395282	0.786452976	-0.171675269	0.570054815	-0.88249072	3.58473E-12
AT4G18130	PHYE	0.395160494	0.438141134	1.270948184	4.36813E-05	0.001332101	0.997844221	0.351031698	0.691895802	0.42190582	0.098576459	0.240948002	0.305056726
AT3G24170	GR1	0.496917912	0.081792404	1.267573645	1.55599E-10	0.832275997	8.25235E-06	-0.003182765	0.997796217	0.004489667	0.992611192	0.205695561	0.178384702
AT1G34420	AT1G34420	1.301320012	0.064578929	1.265400005	0.037534804	0.47779914	0.638201424	-0.058331868	0.982356953	-0.183359671	0.930861837	-0.70522979	0.186750845
AT2G47820	AT2G47820	0.532923714	0.506042992	1.264118008	0.015622774	0.863779266	0.0354955	-0.285174831	0.886397192	0.566347582	0.478838042	-0.175277812	0.587043893
AT2G35900	AT2G35900	0.411656127	0.428079311	1.262499784	4.68391E-07	0.590015973	0.012899433	0.045670595	0.971723999	-0.295807751	0.366910832	-0.424076722	0.002920388
AT4G13250	NYC1	0.995154674	0.007415882	1.262160837	2.20032E-10	0.7338846	2.5829E-05	0.145820263	0.930313852	0.020474147	0.964601576	-0.451370334	0.016684287
AT1G19570	DHAR1	0.765409447	0.000139785	1.2613793	1.66164E-17	0.871962035	4.50582E-05	-0.060097773	0.935083701	-0.081108161	0.81519289	0.032137266	0.869063235
AT1G42990	BZIP60	0.935965523	0.010849948	1.256292543	2.19329E-06	0.50397004	0.05634006	-0.231154275	0.810393493	0.046890266	0.934746814	-0.585612017	0.001211783
AT4G23500	AT4G23500	0.178515564	0.831662069	1.256003121	0.00438088	0.61569844	0.08501695	-0.85094453	0.58116222	-0.12589388	0.883566526	0.334853866	0.117490023
AT1G79160	AT1G79160	0.627898634	0.174972402	1.253891791	2.16221E-05	0.810379369	7.4992E-08	0.235552035	0.86023373	-0.132978167	0.790248164	-0.200018621	0.281672276
AT3G07700	AT3G07700	-0.082771091	0.897952162	1.25301937	4.93094E-07	0.756151035	2.05717E-05	0.61772909	0.62738272	-0.026958566	0.964378519	-0.556313802	0.006082174
AT4G16330	AT4G16330	0.669408438	0.094115854	1.252964372	8.43979E-09	0.881015047	0.000193032	0.924640049	0.575257546	-0.270569336	0.49449846	0.263721964	0.135290303
AT4G29330	DER1	0.723906053	0.070889087	1.252403577	0.000175408	0.380694106	0.15877248	0.027498126	0.981893161	-0.247942412	0.48159339	-0.245139991	0.231022317
AT2G01400	AT2G01400	0.523382347	0.584395985	1.25192058	0.031571098	0.276041308	0.540099428	0.151136065	0.95660961	-0.11840691	0.944687474	0.522271054	0.146341132
AT5G65470	AT5G65470	0.614573109	0.157231922	1.250481178	0.000892612	0.216319604	0.683488012	-0.757364389	0.035613542	-0.377470014	0.195152749	0.290764159	0.204928726
AT5G09540	AT5G09540	0.525323935	0.637889821	1.248808502	0.011642202	0.227733862	0.713619524	0.303442811	0.848886201	0.353612893	0.791556833	0.314648407	0.281701762
AT1G73880	UGT89B1	0.848647643	0.10697588	1.248059048	0.003924759	0.460085885	0.439199455	-0.580723157	0.546988467	-0.129243251	0.870702354	-0.8001749	0.001948405
AT5G56190	AT5G56190	0.973500938	0.00387877	1.244109043	0.000246388	0.525519995	0.061855943	0.238902396	0.837258505	-0.001205745	0.999095849	-0.061785422	0.791409046
AT3G53970	AT3G53970	0.695766093	0.01246859	1.24383412	1.17734E-08	0.802910506	0.000691288	0.194009592	0.838616558	0.228011249	0.484700858	-0.122290019	0.633852607
AT4G18160	KCO6	0.088939509	0.887880298	1.240967321	0.000136027	0.72566008	0.027595743	0.546854856	0.604422229	0.318372438	0.473097704	-0.283853571	0.237880575
AT3G22370	AOX1A	0.970397071	0.079070834	1.237050698	1.3392E-06	0.121828688	0.826365303	-0.394319905	0.769398386	-0.243035757	0.570965538	-0.869869293	2.23913E-05
AT5G62020	HSFB2A	0.370841778	0.598766798	1.23599453	0.000148005	0.285787227	0.408118891	0.666830603	0.616300683	0.03520621	0.964180223	0.940951979	3.15032E-06

AT5G57350	HA3	0.700689755	0.03471105	1.235079335	1.20845E-09	0.39885132	0.01153184	1.109831098	0.513982534	0.021905499	0.96808851	-0.134712239	0.47600239
AT2G32540	CSLB04	0.83613656	0.121729027	1.234673836	0.00856059	0.633842216	0.003979908	1.179915405	0.602119156	-0.217910388	0.755752983	-0.389457542	0.080168628
AT5G63000	AT5G63000	0.511398519	0.359155412	1.230002843	0.020185519	-0.669952383	0.055268397	-0.057601523	0.980596414	-0.103851746	0.893984711	0.082410703	0.765456877
AT5G35460	AT5G35460	0.590635213	0.133094176	1.228847598	4.60704E-05	-0.559774152	0.122537111	-0.061602091	0.951590959	-0.300488455	0.28882624	-0.388191638	0.02886108
AT2G39630	AT2G39630	0.712327552	0.117703269	1.225989457	1.4112E-10	0.551250953	0.024706587	0.24889497	0.798570031	0.094123126	0.857963506	0.083005578	0.736269029
AT2G36290	AT2G36290	0.994172203	0.005819344	1.225009593	0.003846438	0.633578769	0.017277208	-0.230907536	0.78928542	-0.072983399	0.912102846	-0.455171641	0.013609191
AT1G54110	AT1G54110	0.165125542	0.855616975	1.22469775	0.025921765	0.725478729	0.040103753	0.833372312	0.635915547	0.181203702	0.857077081	-0.023478538	0.960580919
AT1G16030	Hsp70b	0.932696362	0.0580708	1.221719732	0.022170379	0.788849205	0.117206965	0.22586929	0.855427008	0.151243509	0.833357931	-0.821955075	1.22675E-06
AT5G27460	AT5G27460	0.935586941	0.265256483	1.221113239	0.001488485	0.660713055	0.052722443	0.513981293	0.722658205	0.398924597	0.564980934	-0.330148696	0.201628635
AT4G36720	HVA22K	0.765452682	0.195879999	1.217418783	4.6784E-05	0.379794197	0.373471393	0.252521447	0.926730556	-0.139989884	0.875046829	0.260919236	0.358737617
AT2G27830	AT2G27830	0.317966187	0.407677874	1.21405114	5.6718E-08	0.483354881	0.001777953	0.765945002	0.289322585	0.250652094	0.321978721	0.416236496	0.020326155
AT4G27745	AT4G27745	0.896964369	0.051846621	1.213900601	0.000527475	0.688186852	0.012350702	-0.087888325	0.962872831	0.39724117	0.219378942	-0.304834235	0.333375308
AT1G79970	AT1G79970	0.764433243	0.019145198	1.213853649	3.03055E-10	0.946303994	3.06382E-07	0.221723157	0.8296049	-0.167450763	0.589526013	-0.033393382	0.881692412
AT1G62030	AT1G62030	0.920119663	0.039256157	1.209909246	0.002897073	0.635732654	0.200962739	0.549230727	0.82865311	0.195712877	0.834745508	0.463767162	0.081200499
AT4G34630	AT4G34630	0.560845823	0.254122871	1.209390908	0.020814269	-0.770351871	0.09326819	-0.197864145	0.911737107	0.630265601	0.200001282	-0.105479867	0.732560362
AT4G37760	SQE3	-0.082123746	0.924744138	1.206994664	4.52028E-05	0.71259115	0.000385621	1.880290153	0.317488294	-0.375183056	0.579109484	-0.660472081	0.013182613
AT1G56145	AT1G56145	0.868045523	0.109094318	1.205166778	0.017535674	0.395605935	0.334674315	-0.384271184	0.772801059	-0.53274089	0.126456313	-0.527171873	0.000596074
AT5G07080	AT5G07080	0.821839307	0.209403513	1.204566537	0.018276346	0.403116573	0.521854206	-0.59293456	0.66686842	-0.576692721	0.176850826	-0.471104603	0.034382054
AT5G06960	OBF5	0.785747937	0.079827759	1.201738497	0.00106008	0.822809416	0.001870385	0.009783575	0.994744545	0.086826057	0.865655253	-0.472428604	0.007061785
AT4G33700	AT4G33700	0.955653814	0.024308716	1.201527901	0.004318211	0.652039192	0.023103298	-0.642668114	0.619569853	-0.316619753	0.363657818	-0.166913434	0.421471443
AT4G24740	FC2	0.763993611	0.002281826	1.199253427	4.69218E-07	0.591132167	0.00078196	0.086241704	0.946164167	-0.096445461	0.76236983	-0.074658542	0.760150959
AT5G11430	AT5G11430	0.003704294	0.99681472	1.198130434	0.001269778	-0.015479998	0.984744755	0.320537429	0.868981104	-0.116199459	0.852739501	-0.183760859	0.451772006
AT1G15950	CCR1	0.438298477	0.288651778	1.194158321	3.58372E-06	0.621458214	0.000165483	-0.004531392	0.996892431	-0.510804051	0.058931207	-0.289129212	0.041793796
AT4G31860	AT4G31860	0.781755403	0.017873246	1.189949199	2.11743E-06	0.74493	9.41029E-05	-0.053998135	0.954137138	0.137503452	0.758375211	-0.403676682	0.018987447
AT3G60110	AT3G60110	1.008177047	0.078313685	1.189362076	0.010262132	-0.058155653	0.894760597	-0.450191005	0.715519489	-0.118654554	0.869406593	-0.614219986	0.003470494
AT2G37060	NF-YB8	0.626283023	0.220918623	1.186458891	0.000644333	0.456923751	0.373181618	-0.220880801	0.899163675	0.143623006	0.812068947	-0.250184696	0.390623516
AT5G65110	ACX2	0.854360532	0.026385242	1.181930427	5.10734E-06	0.764214148	7.84422E-12	0.602860335	0.62738272	-0.008965987	0.986774932	-0.229900337	0.176614112
AT1G75860	AT1G75860	0.51221357	0.228893225	1.181814842	0.000646879	0.437910909	0.227164397	-0.367739622	0.76611969	-0.23796641	0.540814611	0.245793253	0.158543575
AT5G27760	AT5G27760	0.41580767	0.210560111	1.17887635	4.49616E-05	0.449387547	0.109039631	-0.079234621	0.951514049	0.110247156	0.805320688	0.629266031	3.07886E-05
AT5G02010	ROPGEF7	0.816455551	0.099441456	1.178686307	0.00058727	-0.03846227	0.92978697	0.651105979	0.594299218	-0.050276881	0.950790869	0.186552383	0.477931227
AT1G49500	AT1G49500	0.649397196	0.171924238	1.177786433	3.38236E-11	0.809070776	7.28513E-09	0.955283685	0.507877122	0.046077693	0.916551162	-0.88616789	1.03448E-05
AT3G03640	BGLU25	0.817252062	0.278220342	1.176102	0.035023907	0.34410533	0.427173143	-0.559852824	0.731041294	-0.517815947	0.086698345	-0.734861673	4.35898E-06
AT1G05870	AT1G05870	0.610373593	0.046916435	1.171910492	0.000382141	0.372976005	0.013285219	0.013916676	0.992593412	0.058684147	0.91126852	0.021964882	0.928489429
AT3G02370	AT3G02370	0.471488048	0.57154597	1.169222117	0.049755306	0.933442821	0.091203442	0.455542371	0.66808962	0.439089516	0.453380046	0.240179108	0.661579768
AT2G40830	RHC1A	0.855069032	0.011438173	1.165939838	9.56773E-07	0.796809718	0.000494421	0.404942474	0.657374576	0.036383337	0.947746874	0.142117452	0.561289739
AT5G52420	AT5G52420	0.507772771	0.377532487	1.164590813	0.023936512	-0.269192054	0.487616523	-0.044946405	0.980437887	-0.398117542	0.377885595	0.018177788	0.953072699
AT4G38230	CPK26	0.375271488	0.639707406	1.164551856	0.041806846	0.653430941	0.310098161	0.019989274	0.990231864	-0.238017584	0.700183076	0.157329927	0.683373259
AT3G08690	UBC11	0.996275274	0.021733781	1.164212872	1.3142E-06	0.953102883	7.01639E-06	0.08470918	0.967199692	0.2805498	0.348918221	-0.353311882	0.041338603
AT5G38850	AT5G38850	0.690759375	0.12894512	1.162465312	0.000425891	0.705457273	0.051211284	0.344446369	0.890733612	0.169105168	0.824058587	-0.236510529	0.423764769
AT5G02490	Hsp70-2	0.358927267	0.487190676	1.159359281	0.02553135	0.793938487	0.071640849	-0.9919805	0.314063466	-0.31712611	0.312147668	-0.052143271	0.791147508
AT5G54660	AT5G54660	1.309876682	0.095674039	1.158532849	0.041732128	0.161179095	0.789285014	-0.077560402	0.963390011	0.108987605	0.923637745	-0.7142205	0.074190323
AT5G40470	AT5G40470	0.71845997	0.268978345	1.157511439	0.013001368	0.421307145	0.284302062	-0.244338182	0.823156238	0.271403043	0.712408612	-0.292520112	0.358479862
AT3G17070	AT3G17070	0.673216561	0.189156344	1.157500935	0.001404954	-0.430655018	0.224748072	1.233357727	0.498460747	-0.084637314	0.941042784	0.423808788	0.293173778
AT3G12800	SDRB	0.761712217	0.001451849	1.157444344	1.33217E-05	0.735021234	0.000546953	-0.018522233	0.982749202	0.094377613	0.772810255	-0.179878156	0.293077129
AT3G06180	AT3G06180	0.813360374	0.135071944	1.156813545	0.00208707	0.635911797	0.076911669	0.439296484	0.73148555	-0.402524888	0.526869951	-0.376383597	0.116254984
AT5G65207	AT5G65207	0.866298052	0.102932459	1.156593246	1.73964E-05	0.568009148	0.030791222	0.481299374	0.741727789	0.479539569	0.311450556	-1.205809211	0.228548647
AT3G10572	APEM9	0.922158172	0.094691917	1.15484781	2.79617E-06	0.992709778	2.73948E-06	0.948276955	0.433558429	0.524698028	0.051294254	0.261864966	0.173083876
AT2G32800	AP4.3A	0.252786823	0.680866043	1.15221001	8.40172E-05	0.608027192	0.000218438	0.437794526	0.810393493	-0.729652242	0.001347568	-0.202856323	0.254899728
AT3G27610	AT3G27610	1.000803552	0.082028757	1.151420524	0.004146928	0.410209527	0.458080935	0.268199424	0.864295062	-0.047744264	0.94489695	-0.140946419	0.715061125
AT1G70518	AT1G70518	0.770329937	0.181104091	1.150867737	0.021223216	0.903449832	0.009236271	-0.178072032	0.936758504	0.371931782	0.523331269	-0.212223704	0.438813138
AT3G14130	HAOX1	1.661318246	0.555256284	1.150638392	0.003862915	0.034253076	0.942489908	-0.427627893	0.73461774	0.470145906	0.363828665	0.361237791	0.187022029
AT5G13890	AT5G13890	0.92668582	0.07402549	1.15053802	0.001043358	0.839593371	0.000214374	-0.209676221	0.813678008	-0.801785994	0.005241583	-0.348160477	0.095026587
AT4G18530	AT4G18530	0.547359196	0.326673322	1.15026408	0.018399668	0.523753838	0.174576778	-0.043995733	0.976045283	-0.471457222	0.381380382	-0.73685893	0.000298854
AT4G23060	IQD22	0.351733495	0.440230912	1.146939323	7.32663E-10	0.741995278	0.00036622	0.040675186	0.977477438	0.411779347	0.022671441	0.131060547	0.498055994
AT1G70300	KUP6	0.763292426	0.061061554	1.142952451	0.000213195	0.715311644	6.23024E-05	0.18143395	0.907100964	-0.3762721	0.219024794	-0.413635693	0.065457997
AT5G06750	AT5G06750	0.982146788	0.019356246	1.142000662	0.005367725	0.05128211	0.927641135	-0.036988612	0.977607985	0.119084069	0.831572245	0.037419971	0.895161989
AT2G33060	RLP27	0.905789003	0.065241634	1.140905623	0.005066606	0.586601041	0.032137655	0.004971102	0.997591659	-0.282319205	0.486060958	-0.059312178	0.831817541
AT5G39410	AT5G39410	1.169424318	0.056959188	1.134360794	0.00024517	0.64353669	0.077912417	-0.162193709	0.869020685	-0.308538583	0.275351974	0.300138043	0.12668471
AT1G80040	AT1G80040	0.824354032	0.003420466	1.132149828	7.86749E-14	0.96878284	8.93071E-11	0.31605196	0.721140128	-0.09131833	0.78801231	-0.154164408	0.292522756
AT2G32090	AT2G32090	0.472339595	0.33216023	1.131565873	3.58137E-05	-0.113028233	0.551561438	0.974996357	0.48633216	-0.053128736	0.927507051	-0.294976458	0.122313548
AT5G54520	AT5G54520	0.916867176	0.029778909	1.130698396	0.008719417	0.905727968	0.001329801	1.043125204	0.511554278	0.103062345	0.923631194	0.112453339	0.69516335
AT1G04130	TPR2	0.772507032	0.181034918	1.130638664	0.007845351	0.421735115	0.260302229	0.049447288	0.982734229	0.279045215	0.657846079	0.138826354	0.674766726
AT5G25210	AT5G25210	0.712300957	0.199022929	1.130551838	0.001629655	-0.298508307	0.431171421	0.197753219	0.895706838	0.098193756	0.895385501	-0.031479006	0.944510228
AT5G09600	SDH3-1	0.767788288	0.043505849	1.124734983	0.022964015	0.497831763	0.107887215	-0.283152029	0.808237728	-0.161293602	0.756464891	-0.394505043	0.152038413
AT5G42200	AT5G42200	0.353914618	0.599420002	1.124433401	0.003498362	0.866730132	0.008685382	0.403991284	0.867112468	-0.121724041	0.897477433	-0.997494701	8.72763E-08
AT5G03080	LPPgamma	0.764561111	0.123416576	1.124427795	0.001225525	0.112261571	0.816417548	-0.439966336	0.653026084	-0.058000719	0.923979325	-0.278716952	0.384733625
AT5G39590	AT5G39590	0.819986126	0.033442676	1.12338548	9.67232E-12	0.691324986	5.01165E-07	0.047676893	0.964544073	-0.039860718	0.925039435	-0.579427684	1.96892E-07
AT2G32930	ZFN2	0.818376764	0.041087878	1.120283058	0.00351709	0.438581564	0.387155517	-0.277519943	0.74465111	-0.042994476	0.958119929	0.591183656	0.034820662
AT2G36630	AT2G36630	0.74176637	0.115333465	1.119081151	2.02318E-05	0.28396962	0.491213797	0.004768054	0.998026502	-0.24644665	0.566484832	-0.504547164	0.014244085
AT1G80440	AT1G80440	0.934754857	0.020973208	1.118181469	1.51061E-07	0.850047871	1.84358E-15	1.082776956	0.581307949	-0.214893979	0.412503533	-0.316313571	0.064451777
AT1G64710	AT1G64710	0.617485455	0.014149484	1.117202226	3.34493E-06	-0.474247489	0.010423621	0.055545367	0.954608771	-0.021785079	0.973216634	0.058176734	0.73344569
AT1G28540	AT1G28540	0.274256417	0.711653065	1.116987469	0.009259824	0.746741416	0.016931924	-0.153251251	0.926698256	0.183212362	0.804862892	-0.526552349	0.002471603
AT1G21590	AT1G21590	0.663852111	0.0344671	1.115895028	3.19116E-08	0.886046108	6.79774E-05	0.162277178	0.913184039	-0.147484066	0.616763356	0.116711586	0.58761038
AT3G10800	BZIP28	0.919464882	0.005359237	1.115331404	3.42478E-07	0.71961825	0.000215527	0.119653697	0.957721682	-0.311612847	0.323767852	-0.291418951	0.06289521
AT2G02730	AT2G02730	0.505521425	0.408111438	1.11529091	0.000902449	0.19698412	0.522796149	0.148367554	0.916448617	0.154142123	0.821586377	-0.160051072	0.598062524
AT5G06280	AT5G06280	0.532605092	0.263905113	1.112441273	0.001761658	0.24235384	0.5144924	0.016350784	0.993718604	-0.012824712	0.988694176	-0.948415729	4.97048E-06
AT1G56440	TPR5	0.819871567	0.093166007	1.112250436	0.004157623	0.821133005	0.007039732	-0.088644649	0.951485287	0.443011647	0.327045125	0.216021019	0.447570706
AT4G12300	CYP706A4	0.825820434	0.045247663	1.104724481	0.002908572	-0.710971759	0.013224393	0.188847885	0.874176467	0.052081024	0.946158029	-0.252665707	0.167196657
AT1G03800	ERF10	0.373188417	0.731668296	1.101421507	0.02547562	0.871202405	0.062132973	0.776787306	0.73461774	-0.24630469	0.851691904	-0.491135253	0.431137679
AT2G44970	AT2G44970	0.462330919	0.097895201	1.097275583	7.69947E-05	0.552792487	0.035877997	-0.18876702	0.840363187	-0.089301017	0.831323166	-0.025041762	0.921099137
AT4G04180	AT4G04180	-0.288510544	0.836813195	1.096986896	0.029870728	0.006887913	0.995519478	0.134830099	0.90769891	-0.654637396	0.254949018	0.051934516	0.876757738
AT4G00430	PIP1;4	0.27097766	0.471088306	1.095317954	0.000152508	0.319531998	0.213270297	-0.018979132	0.982749202	-0.139155058	0.540869414	0.097060391	0.566299013
AT1G79910	AT1G79910	0.330490775	0.672143512	1.095133234	0.032717476	0.257763682	0.707044624	2.320433261	0.142946924	0.166505466	0.932299626	0.698636877	0.484056494
AT4G33490	AT4G33490	0.861964112	0.143985932	1.095024015	5.64542E-08	0.832826784	0.000918579	1.028403921	0.532231419	0.061117363	0.937520456	0.017149231	0.945311232
AT3G26600	ARO4	0.805843616	0.035968184	1.093422269	0.014084	0.612475534	0.114470988	-0.070014295	0.962474985	-0.070454108	0.925402712	-0.395450185	0.077012507
AT5G64240	MC3	0.287138279	0.568124786	1.092036302	2.02988E-07	0.860587138	1.30439E-09	1.00931867	0.296836814	-0.09283085	0.866906715	0.034660009	0.905488956
AT3G45830	AT3G45830	0.86472123	0.003027253	1.091082322	1.62341E-05	0.934881738	2.1219E-06	0.089159591	0.950356778	0.271506089	0.430532999	-0.257953487	0.126038696
AT3G12730	AT3G12730	0.753100685	0.261774692	1.08901629	0.019876069	0.331404705	0.569907903	1.010703302	0.667824887	0.130283226	0.916204809	0.356233262	0.343890549
AT2G18700	TPS11	0.662257434	0.163623226	1.088516876	8.36221E-08	0.993241426	4.70615E-17	1.147935235	0.587695712	0.120496982	0.708292156	-0.455720974	0.001853683
AT5G17650	AT5G17650	0.387404748	0.450025297	1.088118707	0.018476295	0.058057167	0.930581457	-0.246645849	0.895136157	-0.211032487	0.724493694	-0.370579495	0.177005164
AT4G27680	AT4G27680	0.630538034	0.159741245	1.088097048	0.00019714	0.420099383	0.0708295	-0.259116974	0.770575751	0.023898357	0.974078437	-0.007756066	0.98044513
AT1G01140	CIPK9	0.352015237	0.504805655	1.0877539	6.99061E-06	0.338620856	0.339782251	1.037490404	0.521675277	-0.132914353	0.767740192	0.602664523	5.14425E-05
AT2G17840	ERD7	0.874205117	0.03471105	1.087102238	8.96657E-05	0.84858296	0.133205813	0.865639805	0.639605292	-0.438780488	0.10055888	0.022109845	0.936148146
AT5G39950	TRX2	0.700888882	0.066295333	1.08316278	2.14025E-07	0.157698129	0.703552303	-0.234978362	0.788737401	0.099462131	0.851437944	-0.525143036	0.006968046

AT5G22060	J2	0.753674924	0.001846722	1.082194818	2.67469E-05	0.514940371	0.005843967	-0.183047337	0.831610681	0.084338431	0.824002281	-0.201001225	0.153391717
AT4G23250	EMB1290	-0.827798785	0.09338351	1.079748375	0.007571669	-0.909560217	0.207404541	-1.698821148	0.142386732	-0.416483555	0.539713329	-0.462003543	0.124051705
AT2G38170	CAX1	-0.170402516	0.563075098	1.079542109	0.000730356	0.306949361	0.161505588	-0.371245394	0.675659451	-0.099397842	0.775718307	-0.595378602	1.866E-10
AT5G10610	CYP81K1	0.434368178	0.506265049	1.077678297	0.004048033	-0.169026728	0.681906541	-0.041234046	0.982055475	0.058837601	0.95230969	-0.145534543	0.596870398
AT1G01160	GIF2	0.775002493	0.090222024	1.077143715	0.000118633	0.664188868	0.000453294	0.589352163	0.639875152	0.177799551	0.698699911	0.079892253	0.664899519
AT1G21790	AT1G21790	0.807483329	0.09582349	1.074515149	0.011153389	0.288571896	0.306299275	0.093955287	0.961157568	0.137513986	0.895021414	0.003785378	0.992296702
AT1G47710	SERPIN1	0.774938153	0.080914076	1.073739171	0.000237357	0.717475536	0.007939953	0.276688512	0.65378996	0.082965436	0.856490679	0.159047248	0.275834988
AT5G58160	AT5G58160	0.602590182	0.281127822	1.0705259	0.011681718	0.927367878	0.005023642	0.092169946	0.966082349	-0.653383561	0.052518311	-0.414496504	0.05395373
AT3G60690	AT3G60690	0.899663704	0.052379868	1.069610146	5.33853E-05	0.640519912	0.002682457	0.581802519	0.624999199	-0.158419836	0.803276193	-0.691932444	0.000327369
AT1G79710	AT1G79710	1.069078989	0.09485034	1.068924824	0.014166558	0.411500282	0.279524845	-0.154940165	0.923167441	0.195824468	0.793971365	-0.004539689	0.993346502
AT4G15955	AT4G15955	0.358662698	0.617537146	1.067858432	0.00520626	0.549793958	0.22386822	0.247240854	0.901814827	0.081091178	0.934318497	0.084029173	0.807642544
AT2G36370	AT2G36370	0.258356439	0.716465829	1.066691697	0.008175529	0.465358054	0.159568526	0.002364142	0.999072892	-0.40526157	0.533114663	0.043592018	0.900092356
AT1G56610	AT1G56610	0.734335509	0.193793098	1.065392679	0.039538936	0.567047816	0.090573885	0.057682174	0.977422706	0.378308141	0.532297645	-0.234737151	0.366416733
AT1G64350	SEH1H	0.595973245	0.253444933	1.061156912	0.005280507	0.204083518	0.486900369	0.203275311	0.822327646	0.157701075	0.752244106	-0.279754577	0.348168732
AT5G56180	ARP8	0.865824065	0.039786769	1.060889853	4.94555E-05	0.906899718	1.15257E-07	1.044458677	0.587695712	-0.159143529	0.781670693	0.041193733	0.895683424
AT1G50570	AT1G50570	0.951639705	0.0005515	1.06087928	4.33699E-07	0.859515491	7.05114E-07	0.309398205	0.79601276	0.145852534	0.653680369	-0.150835692	0.442626614
AT2G23450	AT2G23450	0.88811345	0.025424766	1.060087971	7.06552E-05	0.975134875	1.24392E-07	0.183614944	0.922363029	-0.211583453	0.511188955	-0.496048178	0.00060082
AT1G78510	SPS1	0.229586349	0.729735032	1.059291482	0.005405212	0.135116367	0.66229444	-0.044543769	0.982734229	-0.459513355	0.290906703	-0.606383304	0.01351473
AT5G49280	AT5G49280	0.454976685	0.516345054	1.059270864	0.031226317	1.006023815	0.134500884	-0.244786094	0.866579819	-0.991475126	0.000637674	0.533078319	0.03534924
AT5G12890	AT5G12890	0.56321618	0.324131301	1.05852157	0.009807026	0.424340106	0.238700768	0.021210112	0.991271129	0.17476213	0.809692025	0.11968371	0.640812671
AT5G03290	IDH-V	0.778286467	0.004165802	1.049603134	5.69099E-09	0.863438753	3.12331E-07	0.057662841	0.958809354	0.138540304	0.568542894	0.031520686	0.856058206
AT1G30240	AT1G30240	0.428209777	0.516483604	1.049499151	0.013891652	-0.056785512	0.925529783	-0.156387044	0.950313494	-0.318354905	0.4554526	0.108324295	0.732926221
AT5G54930	AT5G54930	0.82946912	0.23750179	1.048314046	0.026023775	0.110997168	0.9026855	-0.434377367	0.809300038	0.059701399	0.958669515	-0.260016258	0.301393291
AT5G24670	EMB2820	0.588801148	0.096701672	1.047297358	0.00050281	0.996045536	1.62567E-08	0.658731728	0.527892502	0.282250698	0.366931793	0.29894099	0.032968665
AT1G17145	AT1G17145	0.686497227	0.106638633	1.045916306	1.00085E-05	0.651701277	0.002567337	0.957714734	0.306859729	0.118445927	0.824640727	0.402957522	0.036978455
AT3G56840	AT3G56840	0.524599159	0.299456906	1.045080227	0.021347162	0.434275289	0.221800157	0.047377777	0.972495118	0.091711558	0.905953395	0.100468866	0.714750078
AT5G11770	AT5G11770	0.666354089	0.012401157	1.044857651	3.79253E-12	0.733672276	9.21013E-08	-0.052942981	0.964000833	-0.143915508	0.599487284	-0.317342101	0.006755143
AT1G08845	AT1G08845	0.877704222	0.121932387	1.043557093	0.00619896	0.59203945	0.079178373	0.599708334	0.66808962	-0.024582071	0.98143759	0.080170681	0.765967725
AT1G07890	APX1	0.361725746	0.259831484	1.043412278	1.04549E-18	0.053497523	0.754808115	-0.352779201	0.705076831	-0.107065353	0.665301267	0.120763354	0.497174267
AT4G16765	AT4G16765	0.513808006	0.241189104	1.043083827	0.001105993	0.825416663	2.66377E-05	0.245883128	0.787101126	0.116839332	0.83684967	0.411771509	0.044772041
AT4G19640	ARA7	0.859147417	0.000233151	1.042815086	3.20087E-09	0.602957189	4.40373E-06	0.173463526	0.860750349	0.050857605	0.908564837	-0.416450781	0.013002714
AT5G45160	RL2	0.756935388	0.134832473	1.042421079	2.46233E-06	0.685567085	4.76928E-05	0.314635069	0.743023983	-0.019009834	0.97644	-0.131256379	0.476061956
AT1G27510	EX2	0.902811054	0.021496891	1.041925504	9.8998E-10	0.714425483	1.52273E-05	1.004500117	0.479092774	-0.053797478	0.885890089	0.022747714	0.925924994
AT1G16000	AT1G16000	0.712686931	0.156911471	1.039622804	0.009225383	-0.385711293	0.284261677	-0.120863431	0.935083701	0.351938518	0.397983364	0.235540221	0.351878218
AT5G27320	GID1C	0.978963888	0.003501405	1.039072866	0.007157839	0.781248702	0.002368311	0.352542299	0.711255369	0.048033138	0.932464417	-0.192948062	0.378279139
AT5G05850	PIRL1	0.280425273	0.603543328	1.037516057	0.04795328	0.949223165	0.024347151	0.020023452	0.990924696	0.189909245	0.831451868	0.638819881	0.004034423
AT3G59660	CDF2	0.95156512	0.018946703	1.034620293	0.004384602	0.218751724	0.448552304	0.696341594	0.62053317	0.043607337	0.934609223	-0.570458044	0.000265331
AT4G27690	VPS26B	0.73240327	0.037329874	1.033634299	5.69741E-05	0.242615903	0.336066461	0.357297603	0.69351933	0.131419191	0.835618895	0.389539704	0.011841196
AT3G59280	TXR1	0.809160399	0.046364556	1.033627138	0.000259967	0.22676106	0.454900648	-0.025794685	0.982734229	0.104866459	0.802042097	-0.194858044	0.386754555
AT5G01990	AT5G01990	0.483763726	0.416481193	1.032327538	0.002002606	0.686517943	0.032675673	-1.148927892	0.070093627	-0.596386526	0.17086402	-0.097823054	0.728193532
AT4G36930	SPT	1.03377205	0.09314241	1.030846741	0.005480584	0.661817335	0.097358831	-0.411516768	0.647435191	0.133567971	0.728271334	-0.269121764	0.095261494
AT5G08570	AT5G08570	0.534970784	0.085453628	1.024558824	1.69801E-08	0.711887198	9.27191E-05	0.227404966	0.776796299	0.050438373	0.915081785	-0.053705319	0.769546676
AT3G04240	SEC	0.496331515	0.125196009	1.024426682	0.000191558	0.826317105	9.53746E-11	-0.149120607	0.824296871	-0.222586099	0.212468123	-0.22182537	0.173258484
AT2G14720	VSR4	0.640526196	0.040917344	1.023919142	4.15266E-05	0.631345749	0.004377109	0.16879427	0.886344781	-0.274895575	0.234902133	-0.508614634	0.000144004
AT4G17245	AT4G17245	0.096166484	0.90206509	1.023233563	0.002847321	0.703421289	0.000747336	1.30612033	0.616300683	0.306142933	0.516484542	0.448126952	0.089939
AT2G41020	AT2G41020	0.651504198	0.057552915	1.022401936	0.009058937	0.865729709	1.04135E-05	0.416884611	0.697407195	0.260565045	0.578542261	-0.057839605	0.840253613
AT4G23920	UGE2	0.110775791	0.880998472	1.021476046	0.009905235	0.371224365	0.402261837	0.531573457	0.808503469	-0.135541209	0.922377562	0.013164495	0.977829609
AT5G04410	NAC2	0.939450514	0.000296369	1.02052879	9.47293E-09	0.839518633	2.39815E-07	0.300608368	0.704643732	-0.042619413	0.924868537	0.033543213	0.859268475
AT3G27390	AT3G27390	0.692311384	0.020780318	1.020222547	5.00825E-06	0.466125708	0.025193667	-0.057023468	0.962474985	-0.457190423	0.01770954	-0.305788327	0.013824592
AT2G16740	UBC29	0.883429063	0.048409179	1.020144144	5.01333E-05	0.55309369	0.217604355	0.425834773	0.703260431	0.480902373	0.331126199	0.701015757	0.005367301
AT5G47660	AT5G47660	0.446396333	0.52459168	1.019066061	0.011749634	0.031901638	0.946974074	0.032010027	0.987686631	0.120863487	0.849224444	-0.24157837	0.456895525
AT1G67600	AT1G67600	1.132568898	0.068396694	1.017121703	0.039422826	0.399250023	0.380565255	0.599845834	0.648919095	0.34507759	0.61043593	-0.246894398	0.438563841
AT5G26000	TGG1	-0.082497889	0.890630217	1.016067092	3.86886E-06	0.278646725	0.122575593	0.864612014	0.604422229	0.130349809	0.550718639	0.473092508	0.000796436
AT4G22750	AT4G22750	0.735438439	0.032418045	1.015025443	6.13378E-05	-4.459045057	0.4113278	0.67667324	0.598901706	0.121214544	0.654307041	2.640118533	0.19866292
AT5G05870	UGT76C1	0.732922612	0.149338346	1.01207521	0.014005619	0.882716785	0.001448811	0.222744447	0.858357172	-0.300409536	0.533838944	-0.110393891	0.659027551
AT4G13270	AT4G13270	0.762815432	0.146298537	1.011317008	0.005284454	0.187214111	0.53409358	-0.567403122	0.470585322	-0.181711624	0.668202307	-0.315572036	0.141586421
AT5G59420	ORP3C	0.795227545	0.014469645	1.008123779	4.28669E-08	0.555925948	4.6898E-06	0.302371394	0.797097845	-0.112259815	0.703592556	-0.434790134	0.000376733
AT1G03220	AT1G03220	0.183785559	0.74152252	1.006761337	0.000750622	-0.140337729	0.739608793	-0.820857177	0.142946924	-0.891617322	2.28868E-07	-0.547671548	1.04858E-06
AT4G32600	AT4G32600	0.428468372	0.334341793	1.006582375	5.10477E-05	0.180755273	0.455097084	0.253565747	0.769502096	-0.315020347	0.334330187	-0.587894077	0.000235168
AT1G06400	ARA-2	0.849264928	0.004886864	1.006053502	9.73419E-05	0.740720997	2.82318E-08	0.479896226	0.611336047	0.129706494	0.650894952	-0.088200893	0.608744561
AT2G24200	LAP1	0.614058437	0.008704945	1.005143944	1.49428E-10	0.26768447	0.078199513	-0.312325347	0.640704059	-0.02916501	0.943680364	0.038472489	0.826836059
AT3G13080	ABCC3	0.282895351	0.49392344	1.00346395	0.001956163	-0.34823197	0.195344786	-0.285280872	0.736215535	-0.112407636	0.721359573	-0.200925716	0.311381741
AT5G49450	bZIP1	0.668951272	0.192443759	1.002642597	1.58853E-09			1.019260965	0.48633216	0.268785511	0.202520597		
AT2G33390	AT2G33390	1.097059588	0.08385968	1.002506099	0.020268157	0.565253336	0.044461465	0.24404058	0.857816448	0.175534619	0.79111196	-0.682453614	0.002662481
AT5G51700	PBS2	0.127581442	0.844376933	1.002024725	0.000243935	0.705666487	0.027027036	-0.013635306	0.993804788	-0.245092447	0.522999379	-0.008961787	0.976668697
AT3G13340	AT3G13340	0.916056217	0.000765242	1.000913705	2.99254E-06	0.925388026	1.59517E-11	0.57301916	0.61532936	0.168788739	0.517194506	-0.013073865	0.946507734
AT1G64200	VHA-E3	-0.671791478	0.06565305	-1.000890243	0.000397984	-0.660767851	0.048323278	0.088259487	0.939540455	0.212805308	0.569674348	-0.574936021	0.002362907
AT1G57680	Cand1	-0.487244738	0.043618591	-1.001337802	1.57279E-05	-0.464923696	0.069299192	-0.124795107	0.902565251	-0.236137206	0.269079164	-0.4661611	0.001097425
AT3G60210	AT3G60210	-0.869882273	0.00602874	-1.0018494	0.018967569	-0.676366256	0.000333773	0.461516247	0.567190016	0.298699933	0.462559608	0.942179613	1.64811E-11
AT5G05890	AT5G05890	-0.716412902	0.026295068	-1.002807534	0.003555832	-0.607219987	0.006514842	0.206812305	0.873618624	0.160234926	0.753554187	0.492500183	0.008077628
AT5G26622	AT5G26622	-0.452786138	0.362313212	-1.007059522	0.033576068			-0.015700789	0.993101238	-0.263416648	0.509573067		
AT5G01710	AT5G01710	-0.717197689	0.021954131	-1.007097334	4.10493E-08			-0.136172813	0.901396065	-0.348580777	0.062445738		
AT3G15630	AT3G15630	-0.309716742	0.5074218	-1.007852342	0.000657452	-0.476291109	0.024582093	0.621855182	0.650183354	0.140836815	0.792766502	0.243989365	0.15549853
AT4G17190	FPS2	-0.712093231	0.222229302	-1.010845511	0.010152388	-0.696792237	0.032956231	-0.137720249	0.917312631	0.154431193	0.683203077	0.703804562	5.55639E-06
AT3G50560	AT3G50560	-0.367706704	0.579902272	-1.011131336	0.004132135	0.513551124	0.269237889	0.804779677	0.627500883	-0.023667534	0.982621649	0.595114701	0.00918986
AT5G38150	PMI15	-0.304077275	0.633969405	-1.011337854	0.003541956	-0.021143951	0.952029505	0.628837454	0.402774567	0.90141337	0.00875737	0.224963581	0.535483868
AT3G62720	XT1	-0.89911587	0.041373845	-1.015307958	9.25198E-06	-0.38365076	0.123033102	0.03890244	0.982749202	-0.207524281	0.507785667	0.436866246	0.001816135
AT2G25200	AT2G25200	-0.910489827	0.08072709	-1.015930922	0.001480039	-0.246992846	0.22453014	1.107900991	0.63459443	0.279266204	0.765238201	-0.494728716	0.094579558
AT4G04200	AT4G04200	-0.525325841	0.452619064	-1.017131192	0.01098763	-0.438891156	0.179194765	0.087200337	0.972495118	0.273245648	0.583115415	0.205635734	0.414097469
AT5G66420	AT5G66420	-0.429311259	0.681766097	-1.018596199	0.024581975	-0.965505944	0.00020916	-0.318565771	0.878717171	0.096809638	0.836814086	0.40262517	0.00407502
AT3G55920	AT3G55920	-0.710931516	0.231547839	-1.019795756	0.008227581	0.111797362	0.822452948	-0.220946433	0.885840009	0.121095996	0.861985137	0.276523258	0.204974586
AT1G18650	PDCB3	-0.92291089	0.036685692	-1.02202616	0.000496031	-0.038222705	0.914052728	0.329334398	0.696374342	0.48689421	0.091140662	0.675360695	0.000525158
AT3G12690	AGC1.5	-0.990570123	0.212461797	-1.024384161	0.033655415	-0.465378462	0.395893833	-1.429933452	0.061032701	-0.322694294	0.68240744	0.357388289	0.225774753
AT2G27060	AT2G27060	-0.868546745	0.107307529	-1.024663663	0.002759518	-0.716602056	0.022159967	0.113206225	0.947755104	-0.073442718	0.898780609	0.452614304	0.020159847
AT1G42480	AT1G42480	-0.592352698	0.279837848	-1.024947046	0.007405742	-0.479657859	0.115436331	-0.09089095	0.949488281	0.161201064	0.744946411	0.01563102	0.957915417
AT2G35520	DAD2	-0.469459219	0.473457601	-1.026693528	0.012336271	-0.913529684	0.00100862	-0.555804215	0.73994806	0.220788072	0.659063827	0.156661595	0.520171649
AT3G28860	ABCB19	-0.703108447	0.158276948	-1.027074139	3.06624E-14	-0.885163675	1.14167E-08	-0.734316372	0.602119156	-0.356781392	0.128550429	-0.053651704	0.778362063
AT5G12470	AT5G12470	-0.846013676	0.034085966	-1.027858606	7.66935E-06	-0.803002556	1.8251E-07	-0.106689372	0.948923938	0.124010518	0.67277392	0.018902855	0.925040874
AT1G16780	VHP2;2	-0.904654503	0.091191149	-1.030208689	0.044826047	-0.261116938	0.430711101	-0.08981794	0.961912118	0.32828335	0.449319013	0.088057633	0.712423931
AT5G65410	HB25	-0.303970533	0.543046662	-1.030596924	0.000141682	-0.426971331	0.082989623	0.509869116	0.484702336	0.1297649	0.775718307	0.519730715	0.008236687
AT1G75120	RRA1	-0.875279799	0.177880846	-1.031832197	0.026990466	3.56359E-05	0.99994077	0.314688872	0.855427008	0.749312178	0.063472951	0.623216526	0.012090572
AT1G77670	AT1G77670	-0.665036225	0.426351899	-1.031919718	0.028946625	-0.701376045	0.009403863	-0.079789306	0.972495118	0.105152397	0.819656454	0.453764264	0.011755772
AT5G19780	TUA5	-0.417823793	0.271136072	-1.032011072	0.000114623	-0.304610691	0.131520502	-0.343936054	0.676109289	-0.158620901	0.455517201	0.164640864	0.350258243
AT5G66320	GATA5	-0.92170486	0.005818255	-1.032966105	3.20459E-06	-0.445012635	0.181072915	0.131574159	0.899163675	-0.059944316	0.91964703	0.621604628	0.000350413

AT1G76550	AT1G76550	-0.828119802	0.030258282	-1.033817763	0.010155671	-0.538906367	0.078269229	-0.256691422	0.711996909	-0.022368549	0.966598167	0.448273322	0.00155007
AT1G21480	AT1G21480	-0.788765933	0.238924756	-1.034009645	0.011907106	-0.487463962	0.053364192	-0.342441915	0.895706838	-0.29968515	0.462653473	0.186231009	0.352275563
AT3G11760	AT3G11760	-0.247144009	0.644414813	-1.035761702	0.000111123	-0.402283844	0.114910734	0.253442329	0.782088381	0.069650571	0.922486937	0.248588731	0.140320118
AT2G46930	AT2G46930	-1.150397258	0.0813828	-1.03906923	0.014893798	-0.642211328	0.052471155	-0.435726412	0.711996909	-0.138618748	0.784731396	0.586585534	0.000160526
AT1G23000	AT1G23000	-0.666250465	0.39155992	-1.039456822	0.005030454	-0.155200225	0.743142394	0.030710774	0.990915497	0.5280893	0.070849441	0.816701016	2.49626E-08
AT2G37080	RIP2	-0.6803202	0.213304879	-1.039839583	2.05135E-06	-0.668169706	0.002216224	0.110184416	0.962474985	0.254032401	0.531745214	0.326554228	0.007390094
AT5G61460	MIM	-0.772963507	0.293056996	-1.04075465	0.032394145	-0.575221707	0.126575034	-0.324978625	0.865981336	-0.035129881	0.968000197	-0.153533701	0.541599124
AT3G18160	PEX3-1	-0.838699089	0.088678646	-1.040836374	0.031851741	-0.778238462	0.030288614	-0.07701226	0.976635648	0.083260581	0.904726557	0.189839265	0.48768752
AT3G22425	IGPD	-0.554479959	0.247325573	-1.042084755	0.023281794	-0.439165027	0.124914911	-0.320486501	0.831161184	0.028017316	0.976927728	0.033171884	0.912356436
AT5G19090	AT5G19090	-0.088058805	0.894653293	-1.042456134	0.001659811	0.035184119	0.884007123	-0.05795832	0.970881852	-0.030266966	0.943180766	0.067675051	0.800855041
AT5G03690	FBA4	-0.664937122	0.262742465	-1.043185334	0.015263938	-0.109044433	0.771719276	0.363978444	0.7265412	-0.013337427	0.990464647	0.665003773	0.000161822
AT4G14200	AT4G14200	-0.77096211	0.268911852	-1.04645728	0.007254639	-0.376588808	0.592117315	0.263442285	0.798599934	0.085564572	0.907644783	0.734079278	0.000195758
AT5G45510	AT5G45510	0.022303864	0.966714516	-1.047707432	5.35441E-05	-0.743012217	4.76794E-05	-0.573174409	0.546625842	-0.471284313	0.000290484	-0.911026823	2.70136E-11
AT4G16141	AT4G16141	-0.091898082	0.934250611	-1.050254815	0.000184961	-0.427873928	0.218453479	0.024057141	0.993871379	0.467774477	0.052518311	0.115797871	0.560055871
AT1G12120	AT1G12120	-0.794911474	0.002746399	-1.052506295	1.18839E-07	-0.50588382	0.024828071	-0.167427899	0.876015705	-0.078383511	0.798358357	-0.222474681	0.183348686
AT2G40820	AT2G40820	-0.084049738	0.926352794	-1.053089187	0.002137117	-0.809189085	0.010181893	-0.525341078	0.715330311	0.062526453	0.891797434	0.372661501	0.051855009
AT3G47890	AT3G47890	-0.236797701	0.694887344	-1.053534953	0.000381801	-0.123787321	0.594645179	-0.31534733	0.657369817	-0.02111181	0.976570624	-0.301935412	0.116063127
AT4G24090	AT4G24090	-0.895543889	0.002416379	-1.053623673	0.00017766	-0.576408213	0.00451831	0.273956106	0.86218218	0.224252392	0.691306135	0.218466234	0.322023144
AT3G50550	AT3G50550	0.123268296	0.911155886	-1.053824814	0.039454179	-0.167483084	0.808891499	-0.827975948	0.66924865	0.184410747	0.774137807	-0.314487032	0.193411721
AT2G04660	APC2	-0.322703686	0.678697642	-1.054053493	0.039425397	-0.748988627	0.082732415	-0.479977661	0.675189064	-0.019719941	0.985302578	0.218986583	0.383107635
AT4G03410	AT4G03410	-1.006776478	0.348270564	-1.05485336	0.003301902	-0.306026236	0.455014217	0.376473751	0.9164397	-0.606662325	0.316943478	-0.075517453	0.834624814
AT5G18410	PIR121	-0.601891797	0.093440302	-1.058661643	1.04611E-05	-0.594655704	0.002347796	-0.215282987	0.721459527	-0.196784509	0.535711681	0.284571287	0.060738681
AT3G49600	UBP26	-0.402223577	0.437350551	-1.059923922	8.09021E-05	-0.694517781	0.022934234	-0.040817372	0.982734229	0.011066761	0.986818647	-0.258128647	0.143603046
AT1G05460	SDE3	-0.287310062	0.616715961	-1.059966231	0.014166558	-0.645458936	0.034847247	0.183291778	0.895706838	-0.001944298	0.999095849	0.479346868	0.008615475
AT4G14760	NET1B	-1.129463668	0.086557534	-1.061824729	0.022687095	-0.879898163	0.000617668	-0.646673373	0.65378996	-0.380230576	0.283414394	-0.413578998	0.036579775
AT3G27906	AT3G27906	0.007568601	0.991774669	-1.062429664	0.000567599	0.63004274	0.09557237	0.298870402	0.685208378	0.089265602	0.871060407	0.173388813	0.554435555
AT1G16340	ATKDSA2	-0.678805764	0.20078301	-1.064130125	0.002023363	-0.374214238	0.324069692	0.3815433	0.689615852	0.304861107	0.494228383	0.554498522	0.000311807
AT5G61480	PXY	-0.899618654	0.088216877	-1.064323294	0.004611602	-0.49493605	0.209370474	-0.823330402	0.707962062	-0.538520111	0.005871987	-0.738183517	6.24118E-05
AT5G06050	AT5G06050	-0.925134159	0.105051393	-1.064427564	0.041075689	-0.667085227	0.139082201	-0.121290607	0.927744632	-0.287544822	0.439037232	0.155003192	0.416331316
AT3G60390	HAT3	-0.857263729	0.005819344	-1.064994603	0.000156006	-0.08812231	0.749252285	0.462548669	0.691824202	0.041497447	0.948497787	-0.025587529	0.927360829
AT4G25320	AT4G25320	0.496878952	0.40716079	-1.065332053	0.039573928	0.010211833	0.98510866	-0.567037561	0.441302192	-0.163996171	0.635351512	-0.489470756	0.024764863
AT5G41000	YSL4	-0.44944781	0.516353268	-1.068006697	0.019963426	0.095662203	0.837821873	0.063379179	0.973864665	-0.001600831	0.999129744	0.139808124	0.734577859
AT2G20900	DGK5	-0.350351536	0.377902219	-1.071895281	0.001174613	-0.485039696	0.061791674	-0.038829	0.978783612	-0.03888207	0.946158029	0.372049303	0.051224332
AT2G47230	DUF6	-0.045101566	0.956873262	-1.072115046	0.007673092	-1.098378563	0.109273165	0.37183609	0.76568568	0.686506567	0.049152364	0.652843891	0.000402896
AT1G20220	AT1G20220	-0.300223351	0.542552835	-1.072484273	0.000312925	-0.315773454	0.268408022	-0.101197669	0.961358666	0.10253922	0.830492367	-0.188858259	0.539181969
AT5G44930	ARAD2	-0.170971766	0.861499124	-1.072513642	0.02825447	0.196494981	0.76526442	0.706641755	0.597380545	0.075962336	0.945553607	0.270406882	0.561289739
AT5G61190	AT5G61190	-0.812026068	0.164145637	-1.07303579	0.001713425	-0.620282111	0.243581255	-0.273761453	0.899163675	-0.106903134	0.82785917	-0.102089741	0.667071589
AT1G50710	4-8月	-0.436508972	0.508314714	-1.074240001	0.007675649	-0.102582965	0.829674831	-0.068060416	0.970582282	0.113216402	0.813646907	0.412368988	0.002806128
AT2G35470	AT2G35470	-0.786307423	0.09209907	-1.075732391	0.000812983	-0.330332619	0.486917827	-0.107327598	0.914676288	0.153537646	0.703919657	0.342959089	0.165192298
AT2G34560	CCP1	-0.823935898	0.054188935	-1.080271929	4.27596E-05	-0.281058541	0.148533792	0.204952825	0.794330193	0.186949958	0.524879363	0.370112334	0.009538531
AT5G10020	AT5G10020	-0.914426567	0.168578591	-1.082007052	0.002280651	-0.711230251	0.033759826	-0.644628014	0.647947756	-0.240752673	0.485857955	-0.019539148	0.952324709
AT4G30850	HHP2	-0.702088287	0.180821457	-1.082732199	0.001682485	-0.284603678	0.603317397	-1.074170218	0.246235822	0.126071466	0.869033869	-0.617077681	0.000260305
AT2G35620	FEI2	-0.721311486	0.065411934	-1.084286256	0.020960033	-0.474851199	0.018186659	-0.313834873	0.745375315	0.009894985	0.988694176	0.35143753	0.019483342
AT3G02630	AT3G02630	-0.621020845	0.088246376	-1.086184976	0.001032865	-0.567291128	0.021629198	0.065686868	0.947563907	-0.122126144	0.737309895	0.103160264	0.576009155
AT3G60320	AT3G60320	-0.823022495	0.000130852	-1.087414481	4.48319E-13	-0.082639446	0.769724528	0.075766893	0.964923674	-0.289470904	0.139597196	-0.46273439	0.007675034
AT5G53210	SPCH	-0.49091606	0.519764282	-1.08845063	0.015364532	-0.066302395	0.919695967	0.330633606	0.815085418	0.475627704	0.510718611	0.791866714	0.000230667
AT5G46780	AT5G46780	-0.407396924	0.231603077	-1.089069906	2.92986E-05	-0.113901164	0.822586195	-0.431960853	0.65378996	-0.453853705	0.120900727	0.002558541	0.994530191
AT1G30440	AT1G30440	-0.804018347	0.064996634	-1.092223509	0.001775783	-0.842420932	0.000326705	-0.280159918	0.676109289	-0.169672329	0.617074584	-0.314236933	0.01602409
AT1G09630	RAB11c	-0.506442914	0.272193408	-1.092381931	0.003844345	-0.727587501	0.002192745	-0.032079903	0.982734229	0.057076846	0.93404597	0.202550298	0.283187232
AT5G56780	ET2	-0.391802196	0.383721868	-1.093026353	0.001060601	-0.667811939	0.220212604	0.666784011	0.618907586	0.246963161	0.655417993	0.30772666	0.429518275
AT1G14920	GAI	-0.589850364	0.218112607	-1.093091568	0.000914746	-0.36906744	0.034726451	-0.28951168	0.637815859	0.083047838	0.84792397	-0.173357464	0.380267975
AT5G43700	ATAUX2-11	-0.711374792	0.060356925	-1.094853885	2.59177E-05	-0.537651522	9.6802E-05	0.350646753	0.684257173	0.185775359	0.518995017	-0.371233317	0.037205747
AT3G54910	AT3G54910	-0.447404808	0.5074218	-1.096374364	0.000917567	0.089943604	0.848831555	0.525077466	0.567190016	0.289781073	0.571762044	0.801136537	0.000376733
AT5G60210	RIP5	-0.432265776	0.381343842	-1.096424423	1.3499E-06	-0.440584015	0.039808965	0.442486723	0.678205648	0.436856149	0.043532871	0.638420286	8.24869E-08
AT1G78060	AT1G78060	-0.100888582	0.899159016	-1.098384912	0.000569499	-0.960908517	0.000240734	-0.873772438	0.697407195	-0.636550157	0.00974561	-0.262683035	0.129553004
AT2G32450	AT2G32450	-0.332887427	0.51096648	-1.103049975	0.000776889	-0.182953192	0.519264307	0.4684167	0.66808962	0.016110498	0.986774932	-0.043443556	0.864372436
AT5G22400	AT5G22400	-0.366697889	0.573979049	-1.104505648	0.015189664	-0.388626487	0.297409396	-0.170974828	0.916159273	-0.037798803	0.965786265	0.039397939	0.880863262
AT5G55820	WYR	-1.236132047	0.304809911	-1.105281272	0.0143068	-0.756592479	0.137567199	0.148066573	0.955270508	0.353922555	0.481806699	0.451538843	0.012312676
AT1G12000	AT1G12000	-0.993810864	0.089182763	-1.105887368	0.002633709	-0.99167229	2.77473E-08	-0.584365586	0.659711758	-0.231316513	0.205719195	0.33329132	0.031187195
AT3G62370	AT3G62370	-0.725805514	0.061893785	-1.109966065	9.80092E-05	-0.990500718	5.17039E-05	-0.017978174	0.987671055	0.371370423	0.282828458	0.562410836	0.001771346
AT3G56640	SEC15A	-0.509055425	0.463618992	-1.110163953	0.011131916	-0.465379083	0.297832629	0.16797983	0.881491928	0.325945233	0.508677371	0.218735604	0.440627332
AT1G53300	TTL1	-0.629538136	0.120941871	-1.110800417	5.79284E-08	-0.985990632	2.92312E-07	-0.3582541	0.776929891	-0.152698784	0.704428764	0.41568343	0.009113616
AT2G30720	AT2G30720	-0.945347458	0.008827364	-1.112255347	0.00236543	-0.220028304	0.488928855	0.081013428	0.953923605	-0.133035677	0.833943938	0.371002242	0.116254984
AT3G55420	AT3G55420	-0.033267792	0.960104219	-1.112370584	0.000155058	-0.28247565	0.541743828	-0.43915252	0.805279046	0.117830844	0.747316158	-0.218962926	0.304518683
AT5G16870	AT5G16870	-0.85887509	0.052979895	-1.113425663	0.041669465	-0.431116215	0.332399409	-0.085963584	0.947537872	-0.265135995	0.59255443	0.199354577	0.502305672
AT2G16850	PIP2;8	-0.393215651	0.724735666	-1.113551355	0.02553135	-0.517748917	0.091194686	-0.347316881	0.90808103	0.144291112	0.666064463	0.585179037	0.004733574
AT2G46870	NGA1	-0.974997849	0.019703195	-1.115388119	0.012071156	-0.566879667	0.104542685	-0.038281419	0.990915497	0.135631258	0.859673416	-0.533513055	0.034452989
AT4G26610	D6PKL1	-0.900348465	0.004767586	-1.117602963	0.000164113	-0.2931967	0.058426178	0.342891942	0.691824202	-0.108296585	0.802042097	-0.007506458	0.973109329
AT3G27230	AT3G27230	-0.620047787	0.276640019	-1.117730753	0.044097184	-0.972240562	0.000697664	-0.686118005	0.649408835	-0.069527764	0.885890089	-0.037042924	0.867562283
AT4G09160	AT4G09160	-0.602896029	0.047951934	-1.119835226	0.000655349	-0.201691624	0.563907217	0.336592236	0.706790319	0.025470342	0.965729524	0.540609926	0.002989896
AT1G22910	AT1G22910	-0.862232425	0.035284677	-1.124952983	0.000236327	-0.631293263	0.004400963	-0.55813433	0.653026084	-0.188426529	0.559316902	-0.082897294	0.675867001
AT5G37790	AT5G37790	0.246198027	0.679299071	-1.125729328	0.017954801	-0.520470693	0.320941681	-0.912155794	0.533068231	-0.071268331	0.897418007	-0.37075993	0.081205669
AT2G42580	TTL3	-0.150865248	0.734918287	-1.128104697	2.97607E-07	0.361938663	0.105253826	0.871708972	0.434853795	0.055402477	0.915867449	0.312658491	0.02023948
AT1G22882	AT1G22882	-0.627009518	0.235911178	-1.128830716	0.007635982	-0.4090496	0.294373652	-0.604737431	0.478067535	-0.564254409	0.081630061	-0.528474639	0.002947058
AT2G22840	GRF1	-0.903755237	0.16050899	-1.130484157	0.018055517	-0.18412288	0.620647125	-0.023225496	0.989487724	-0.012438848	0.987053629	0.575497734	0.002833445
AT1G52190	AT1G52190	-0.654910254	0.024749491	-1.13289978	2.94888E-05	-0.901363723	1.19001E-08	-0.327230399	0.673165787	-0.205000127	0.794004103	-0.09460493	0.563688163
AT2G46920	POL	0.069310918	0.936581826	-1.133165511	0.000307334	-0.522372964	0.156278618	-0.576005167	0.442473475	-0.041967821	0.952661388	-0.422265085	0.020834212
AT4G18670	AT4G18670	-0.937681949	0.000291174	-1.135476606	0.000190458	-0.728128687	6.99997E-08	-0.505913717	0.323698908	-0.662331219	0.001732895	-0.174443882	0.458795209
AT1G06550	AT1G06550	-0.831727491	0.089880286	-1.136402976	6.79573E-05	-0.871686261	0.025565941	-0.484665566	0.666155455	-0.004525016	0.994163341	0.300635767	0.026517497
AT5G22920	AT5G22920	-0.750672239	0.092969832	-1.140733288	3.54015E-08	0.453378287	0.000395459	1.795398001	0.203276234	0.390315039	0.078890227	0.825445607	1.99184E-10
AT1G13030	AT1G13030	-0.176567322	0.854499768	-1.141737482	0.043505462	-0.563450577	0.236294604	-0.464550368	0.726635812	0.096584826	0.90798098	0.454181115	0.016409134
AT1G68060	MAP70-1	-0.356504948	0.450734651	-1.14205925	1.09063E-07	-0.668093851	0.00035897	-0.15044128	0.908372568	-0.088212666	0.799841888	-0.065238666	0.715258872
AT4G30080	ARF16	-0.642571622	0.186181095	-1.147412847	0.003734878	-0.313851693	0.362315907	-0.66361801	0.712887303	-0.387177646	0.342153895	-0.167153662	0.429727892
AT5G42140	AT5G42140	-0.640777636	0.20060204	-1.14757177	0.000354456	-0.137427807	0.7386381	0.334013247	0.720230968	-0.110754089	0.867384476	-0.057915289	0.853588587
AT1G53650	CID8	-0.70930234	0.15182294	-1.148416399	0.027427456	0.154371717	0.793801408	0.014129104	0.995788755	0.286784435	0.521243345	0.166712448	0.535123035
AT2G28150	AT2G28150	-0.389530245	0.218841211	-1.151486119	1.28447E-09	-0.641078946	0.014167443	0.444632596	0.576334999	0.184271005	0.655700009	0.302154767	0.053092559
AT3G28200	AT3G28200	-0.725523607	0.048904111	-1.151527759	5.26719E-09	-0.005773155	0.985943771	-0.655133693	0.019675654	-0.382125067	0.125753935	-0.247387277	0.196546731
AT3G18800	AT3G18800	-0.332452959	0.51825827	-1.152382451	0.004100727	-0.405337073	0.35908466	-0.256136775	0.867112468	-0.119690126	0.880748473	0.673792092	0.00100579
AT1G19980	AT1G19980	0.2149228	0.82800407	-1.153550129	0.03881807	-0.209421346	0.678383525	-0.44842068	0.66808962	-0.184159191	0.875476392	0.517152151	0.0458036
AT2G18940	AT2G18940	-0.629423706	0.190555563	-1.154350196	2.41947E-05	-0.644808134	0.008465771	0.108754839	0.917312631	-0.027074395	0.975427751	0.188561793	0.441078029
AT5G15210	HB30	-0.166941722	0.783496643	-1.156134402	0.000582901	-0.474534928	0.082423103	-0.342967149	0.757212383	0.27535018	0.536643581	-0.123071239	0.616969754
AT3G45090	AT3G45090	-0.292399339	0.756477616	-1.156314826	0.00177704	-0.88983245	0.009737463	-0.294440204	0.845234181	-0.177057843	0.598025021	-0.118443723	0.68354207
AT4G37260	MYB73	-0.776992577	0.04960571	-1.159249417	1.33998E-08	-0.887680095	2.19503E-06	0.636549713	0.567795183	0.084860716	0.812921184	0.486795252	0.00549272
AT2G41540	GPDHC1	-0.949507269	0.001851156	-1.159932831	0.0005431	-0.559984929	0.000663177	-0.254982438	0.810169925	0.142253449	0.680876398	-0.039897065	0.836886862

AT5G41180	AT5G41180	-0.40141008	0.666001052	-1.160001714	0.012360011	-0.705002012	0.212194792	-0.131558422	0.968891327	0.203257888	0.706765606	-0.103442958	0.695826195
AT5G63700	AT5G63700	-0.545065576	0.292712689	-1.161122956	0.007956096	-0.325265347	0.320246377	0.278594595	0.833592548	0.495821144	0.218925754	0.349860908	0.061308179
AT4G34560	AT4G34560	0.022080976	0.978709893	-1.161762103	0.000298746	-0.704092327	0.011744459	-0.110631747	0.962872831	0.15494559	0.654509491	-0.178438003	0.438717739
AT5G16720	AT5G16720	0.175740831	0.741656464	-1.163520064	0.010973008	-0.39085641	0.200157265	-0.075256179	0.967195835	-0.299583478	0.53525964	-0.420469497	0.023869104
AT1G01110	IQD18	-0.64977142	0.259076136	-1.166622516	0.037525106	-0.34873522	0.614593003	-0.274281966	0.906077763	-0.152689984	0.781706477	0.263556973	0.175852064
AT4G12310	CYP706A5	-0.912322574	0.099151381	-1.167675005	0.001127964	-0.550381751	0.052111545	-0.228516853	0.895706838	-0.665618349	0.125191522	-0.606642585	0.007409508
AT4G27760	FEY	-0.668081726	0.089886773	-1.170122183	0.002256104	-0.576372185	0.008540015	0.039188802	0.977743945	0.479414081	0.088665114	0.788142437	1.62189E-07
AT1G69690	TCP15	-0.497877451	0.318653886	-1.17551282	0.001314379	0.187961797	0.665703366	-0.567836428	0.496998136	-0.095345238	0.824167307	-0.199096927	0.259448629
AT5G11690	TIM17-3	-0.296154615	0.741047008	-1.176437867	0.026974256	-0.388039708	0.393412992	-0.374249232	0.823318742	-0.12398737	0.87076682	0.205473751	0.388258183
AT2G16440	MCM4	-0.956771985	0.319371119	-1.176457055	0.008837253	-0.974777641	0.028549051	-0.087950019	0.959104829	-0.493425434	0.087823294	0.102158649	0.710767349
AT4G01900	GLB1	-1.37015677	0.060298697	-1.178461319	0.038681899	-0.958469884	8.56383E-05	0.365830741	0.797614335	0.419228209	0.217289538	0.680869882	3.71686E-05
AT2G39890	PROT1	0.130944889	0.896020233	-1.179560031	0.017276095	-0.046250494	0.939986792	-0.418954541	0.844633595	0.042989318	0.943772149	-0.243808295	0.19648946
AT1G48230	AT1G48230	-0.598553896	0.150441219	-1.183550772	7.74689E-06	-0.358118759	0.04928908	-0.129154242	0.93556951	-0.228274863	0.45579132	0.021411087	0.925568481
AT5G26850	AT5G26850	-0.792337498	0.130865887	-1.184316672	2.4042E-07	-0.1755193	0.350034628	0.064993683	0.972205981	0.264311644	0.269079164	0.841538053	2.85937E-07
AT5G13610	AT5G13610	-0.693409769	0.290090218	-1.189611113	0.004450061	-0.907559983	0.004733258	0.352311006	0.777195979	0.170634039	0.863621528	0.187906642	0.529337289
AT1G08130	LIG1	-0.949787047	0.022915553	-1.189687507	9.34593E-05	-0.925659142	0.000247396	-0.135087447	0.930238321	-0.271031829	0.532358137	0.105126098	0.677198794
AT1G64530	AT1G64530	-0.730348989	0.027801327	-1.197805382	1.91356E-05	-0.64182872	0.015164025	-0.349268645	0.731041294	-0.180363838	0.700703125	-0.457509657	0.037799096
AT5G63810	BGAL10	-0.801048158	0.026680663	-1.199585241	9.517E-07	-0.60900257	0.045020537	-0.098437315	0.949913024	-0.490235884	0.003963365	0.303932174	0.044815522
AT1G12700	RPF1	-0.315664031	0.73226618	-1.203043968	0.022874563	0.164088327	0.753593328	0.782463642	0.475372579	0.00357782	0.998625192	-0.134556622	0.748811201
AT1G22530	PATL2	-0.468039213	0.075258388	-1.205440255	1.62479E-11	-0.208534596	0.4695086	-0.450380872	0.292351702	-0.566970741	0.001292684	-0.698000726	2.52156E-11
AT4G36150	AT4G36150	0.259184391	0.764687027	-1.208344731	0.006227495	-0.370279683	0.171302877	-0.315755956	0.913606209	0.177727186	0.827181504	-0.341807421	0.106692729
AT1G76680	OPR1	-0.330702578	0.536501593	-1.21015568	0.0024257	-0.980522678	1.50408E-05	-0.340271812	0.784665705	-0.337171137	0.17278441	0.476669854	0.000478722
AT5G14480	AT5G14480	-0.424534521	0.536057811	-1.210249253	0.023859728	-0.330592485	0.379941737	0.229369965	0.876698997	0.00610365	0.99569371	0.557863195	0.038048998
AT5G44040	AT5G44040	-0.606446769	0.291142604	-1.212647476	0.021665778	-0.098773952	0.717911903	0.120141482	0.939540455	0.110346451	0.875764034	0.064434243	0.75665223
AT2G35340	MEE29	-1.565271266	0.064289758	-1.213482608	0.049513174	0.301260757	0.504217341	-0.276156895	0.893780502	0.15244611	0.896500075	0.254746227	0.327601884
AT4G36120	AT4G36120	-1.087590617	0.105428437	-1.213704675	0.021175506	-0.379633479	0.488805567	-0.275744191	0.84002099	-0.469955147	0.31206941	-0.174300769	0.593884794
AT1G16520	AT1G16520	-0.301847935	0.723245705	-1.21388072	0.00476845	-0.330407617	0.414273168	-0.183879399	0.904735573	-0.052238433	0.935733669	0.196817426	0.28098258
AT3G19380	PUB25	-0.726378072	0.102010218	-1.214024631	1.92373E-05	-0.591165126	0.03013607	-0.084461378	0.965260868	-0.092276417	0.905907141	-0.677183928	0.000572282
AT1G49580	AT1G49580	-0.768690986	0.149716448	-1.214333509	5.20813E-05	-0.143054919	0.669115282	-0.72204218	0.666573155	-0.222580571	0.426752852	-0.192719784	0.327380909
AT5G65440	AT5G65440	-0.945114308	0.001475711	-1.215001201	3.65424E-07	-0.054176762	0.850248096	-0.106148206	0.940797093	-0.044102021	0.934609223	0.133910254	0.588381319
AT1G34640	AT1G34640	-0.259778862	0.768495368	-1.218288094	0.042300675	-0.400270872	0.53622253	-0.230384233	0.930073201	0.07285201	0.949621734	0.498328087	0.204928726
AT1G77850	ARF17	0.118952868	0.860392312	-1.219259675	0.008367957	-0.244172816	0.678960544	-0.602114645	0.616300683	0.017403943	0.984765765	-0.626089241	0.01674639
AT5G57180	CIA2	-0.746661557	0.09070264	-1.219870019	0.000119418	-0.888979357	2.04382E-07	0.306793417	0.717709314	0.268896689	0.4494586	0.550909252	0.0026404
AT3G18440	ALMT9	-0.364693913	0.463511939	-1.223310729	0.002735054	-0.355656957	0.332203311	-0.243136326	0.875648986	-0.437982832	0.219488221	-0.136404957	0.541554709
AT2G18160	bZIP2	-0.428960081	0.178800612	-1.22405865	3.67664E-10			0.208104162	0.746674731	-0.002175116	0.99766313		
AT4G35930	FBS4	-0.618997854	0.286630648	-1.228427623	0.035356196	-0.516457791	0.304750247	-0.459541761	0.714552244	-0.371369114	0.515330631	-0.233572796	0.413251097
AT3G18090	NRPD2B	-0.7572582	0.235911178	-1.22987995	0.018981996	-0.631523068	0.025776687	-0.250476057	0.925286116	0.463899575	0.315034185	0.567384401	0.013931124
AT5G50375	CPI1	-0.562448282	0.190555563	-1.230451839	0.009577735	-0.484975308	0.097056112	-0.125426829	0.954565337	0.36639217	0.472886194	0.569174172	0.000901341
AT3G54220	SCR	-0.725401185	0.13313928	-1.233334276	0.007544492	-0.528320768	0.049287003	-0.3528041	0.723262048	-0.276317334	0.301120228	0.036851327	0.891764412
AT1G69040	ACR4	-0.941743385	0.014914897	-1.234475301	1.46538E-11	-0.688119602	0.00023041	-0.556754004	0.441674694	0.09806893	0.75477566	0.113281206	0.484935474
AT1G15480	AT1G15480	0.254356261	0.782974577	-1.235181748	0.041488992	-0.224089287	0.548393519	-0.310777449	0.804495913	0.179048289	0.699294384	0.360299009	0.028886602
AT4G10400	AT4G10400	-1.147050033	0.098970359	-1.236882262	0.033932193	-0.473325979	0.266048416	0.496249305	0.712887303	-0.081931605	0.937182828	0.275553925	0.393594342
AT1G64080	MAKR2	-0.551073095	0.428079311	-1.239075307	0.009163267	-0.427391048	0.461373352	0.315902384	0.803209741	0.254815696	0.704291796	0.210280338	0.409795998
AT5G18620	CHR17	-0.440537522	0.388253149	-1.239634913	1.66851E-10	-0.418797206	0.031842306	0.120790676	0.92566866	0.15089878	0.680252138	0.060751655	0.760150959
AT1G55170	AT1G55170	-0.694167325	0.359327254	-1.23971184	0.008085464	-0.140477987	0.81316485	-0.759428344	0.610367887	-0.089303868	0.945313413	-0.190619972	0.586323896
AT1G09460	AT1G09460	-0.71761847	0.175643486	-1.240134612	5.83808E-05	-0.345301515	0.485589412	-0.617229444	0.313321941	-0.568114473	0.003901459	-0.533388172	0.036606235
AT3G56480	AT3G56480	-0.347219891	0.691700543	-1.240927799	3.71328E-07	-0.621705671	0.005935683	-0.61604795	0.650183354	-0.047487683	0.929239543	0.160659704	0.332870626
AT3G49250	DMS3	-1.475173528	0.05681823	-1.241318954	0.00395722	-0.742747294	0.097056112	0.010284094	0.997285903	0.370848886	0.544231331	0.371371347	0.108989417
AT4G32790	AT4G32790	-0.958122962	0.093730948	-1.24276097	0.021721075	-0.362326121	0.403059605	-0.191473121	0.921986527	-0.274302471	0.577315828	-0.285539285	0.395900036
AT1G15410	AT1G15410	-0.52499047	0.177315272	-1.243817003	1.5552E-05	-0.431516871	0.122348754	-0.005229566	0.996900825	0.031435211	0.965558632	0.154100674	0.492546195
AT4G03390	SRF3	-0.629042295	0.110558571	-1.245645643	9.19483E-08	-0.883331029	7.30285E-05	-0.463358843	0.682049925	-0.469755922	0.059053305	-0.255516903	0.147808654
AT5G09460	AT5G09460	-0.616718312	0.043979125	-1.246266485	6.41494E-05			-0.033552755	0.977477438	-0.100185491	0.721359573		
AT3G17920	AT3G17920	-0.732029028	0.082917245	-1.250475465	0.000326639	-0.314540357	0.348551861	0.04294908	0.97743145	-0.097734898	0.903043766	-0.191323861	0.459737676
AT2G37860	LCD1	-0.167040937	0.826301167	-1.256632929	0.012273182	-0.966914589	1.19493E-07	-0.018744792	0.993618504	0.105392644	0.793774805	0.155040122	0.384113408
AT5G04320	AT5G04320	-0.786887849	0.113598432	-1.257249044	0.004626309	-0.153070318	0.718533694	0.355560941	0.730563908	0.108669401	0.882725602	0.317390407	0.159443976
AT1G19990	AT1G19990	-0.919139738	0.32622398	-1.259982086	0.042358085	-0.512017348	0.30563731	0.116717171	0.969975497	0.453334511	0.402055214	0.177771456	0.608283271
AT4G35730	AT4G35730	-0.611824893	0.310338932	-1.266629013	0.011603492	-0.505572222	0.257295211	-0.261412135	0.84196754	0.000757351	0.999664963	0.588563285	0.002206799
AT2G35880	AT2G35880	-0.919703562	0.000170386	-1.267689573	3.2211E-09	-0.892123099	2.00761E-05	-0.490393253	0.525705888	-0.461155425	0.001735134	-0.07575537	0.616651133
AT5G48230	ACAT2	-0.344634667	0.365126464	-1.272923444	2.31479E-09	-0.15609344	0.4695086	-0.099770386	0.913641552	-0.024024817	0.962240526	0.181991385	0.203295059
AT4G37650	SHR	0.414261566	0.5732064	-1.278341117	0.004350903	-0.578653867	0.0620874	-0.818559495	0.62738272	0.069528804	0.90184129	-0.500089499	6.96106E-05
AT1G21910	DREB26	-0.842466335	0.229609546	-1.279948844	0.006522053	-0.939905475	0.177864123	1.045683138	0.465456499	-0.481003378	0.186485571	0.789752326	1.6351E-05
AT4G20350	AT4G20350	0.210669311	0.87169017	-1.281516251	0.033666074	0.988919502	0.146902214	0.562711322	0.648919095	-0.404400685	0.618550064	0.295297475	0.539986755
AT3G04860	AT3G04860	-0.153398084	0.819892089	-1.283170591	0.013073029	0.127313776	0.69411558	-0.207856543	0.878717171	-0.267921003	0.674900862	-0.537302973	0.002347064
AT5G52010	AT5G52010	-0.417317992	0.341165584	-1.283369566	0.007510082	-0.643962952	0.0637909	-0.314796807	0.759702709	0.192875356	0.775432933	-0.170447392	0.578666484
AT5G57780	P1R1	-0.497189844	0.503506581	-1.289387771	0.011278497	-0.507945646	0.078269229	0.020365811	0.994162268	0.389963792	0.254509041	0.607966177	1.95325E-05
AT5G27300	AT5G27300	-0.920413226	0.07054462	-1.292721118	0.00511209	-0.047099528	0.906522965	0.46776279	0.721140128	0.512585679	0.411935699	0.739364974	7.07102E-05
AT3G13960	GRF5	0.104747014	0.915565677	-1.293565153	0.012871879	0.148148958	0.705057556	0.270593664	0.823712918	0.479857375	0.201027557	0.38049661	0.058001519
AT4G02100	AT4G02100	-0.279385406	0.717234206	-1.296369329	0.004111411	-0.862051916	0.085661464	-0.216417307	0.935083701	-0.092086982	0.872795006	0.20908014	0.388724917
AT5G02820	RHL2	-0.454897496	0.43276143	-1.29852472	1.03823E-05	-0.471275668	0.267431813	-0.264088896	0.823579145	0.162134993	0.739480447	-0.012312527	0.963872804
AT3G60630	HAM2	-0.71025231	0.16488369	-1.301572299	0.009770343	-0.667070145	0.001600896	-0.254955068	0.720626114	-0.325758011	0.2553851	0.003470773	0.992804029
AT5G19770	TUA3	-0.917204382	0.000114801	-1.301784315	1.37765E-13	-0.662586357	3.83294E-05	-0.464874781	0.606948021	-0.278105379	0.099719688	0.052878117	0.777384495
AT2G39900	WLIM2a	-0.799493627	0.213850734	-1.301967368	0.007617555	-0.137301831	0.776169124	-0.278593314	0.866130347	0.113898574	0.724493694	0.291255819	0.057972251
AT1G75960	AT1G75960	-0.975826774	0.255236604	-1.30402201	0.047382268	-0.609481679	0.362009613	-0.322928884	0.930074641	-0.132131288	0.924938189	0.512614996	0.29213625
AT5G08000	E13L3	-0.075426217	0.956321965	-1.306142797	0.007499773	-0.314044718	0.472658038	-0.090857112	0.972495118	0.060797613	0.905220501	0.760606682	7.74564E-07
AT2G21090	AT2G21090	-0.256180648	0.861338532	-1.310389673	0.016875214	-0.467234956	0.51686753	-0.256532573	0.958179519	0.07459538	0.934574943	-0.013648101	0.970042231
AT5G45610	SUV2	-0.585523023	0.269347626	-1.311328739	0.001446001	-0.532358331	0.110096151	0.097912322	0.953774208	0.152761895	0.851437944	0.334858101	0.193672247
AT1G27500	KLCR3	-0.366116667	0.536057811	-1.312438268	0.022290983	-0.573848	0.027299926	0.281865599	0.831161184	-0.165708557	0.825713105	-0.192995384	0.355135126
AT1G52410	TSA1	-0.761010852	0.343629607	-1.313246958	0.029391845	-0.751572268	0.338501087	-0.192996427	0.938382038	0.044898347	0.950790869	0.089964084	0.751043136
AT4G39860	AT4G39860	-0.39365572	0.631402013	-1.313304821	0.000838873	-0.945915035	3.01978E-05	-0.124128488	0.968655039	0.152957718	0.770901826	0.38607234	0.077641746
AT2G25050	AT2G25050	-0.549700337	0.203344415	-1.314038027	0.004465692	-0.357127453	0.154987055	-0.50257154	0.663046749	-0.073795651	0.922486937	-0.195995825	0.372537586
AT2G32720	CB5-B	-0.570880509	0.403688438	-1.316463541	0.005732192	-0.6172085	0.056999131	-0.37626673	0.745375315	-0.168775916	0.606679379	0.246839755	0.209993103
AT3G12970	AT3G12970	-1.204349589	0.082095429	-1.319931688	0.013012229	-0.733199822	0.109568208	0.43397739	0.672844426	-0.294737014	0.672741445	-0.064243557	0.861871249
AT3G12150	AT3G12150	-0.755358962	0.03696358	-1.321585007	1.42437E-06	-0.574376994	0.016220467	0.190342545	0.829913345	0.271093028	0.561308113	0.084479068	0.786556318
AT3G10010	DML2	-0.229481359	0.633603905	-1.322782471	7.34206E-05	0.242863363	0.435969988	0.75880201	0.212736692	0.049440522	0.938640038	0.509055856	0.001770431
AT1G05670	AT1G05670	-0.188965869	0.794952744	-1.327291211	0.031880277	-0.450179762	0.420626257	0.517862493	0.66524984	0.326916043	0.640365963	0.113557526	0.730643373
AT1G66190	AT1G66190	-0.918101049	0.198494037	-1.329633743	0.042593364	-1.305930861	0.086191801	-0.483310742	0.813262169	0.238174624	0.780947109	-0.400994567	0.341866198
AT2G37585	AT2G37585	-0.96177958	0.103031624	-1.330277869	1.10787E-05	-0.230351343	0.502774472	-0.045190515	0.975645787	-0.060088638	0.931144582	0.03095206	0.920640175
AT5G46790	PYL1	-0.612284128	0.280488324	-1.331374281	0.015106262	-0.769783774	0.017091391	-0.415154388	0.706790319	0.075700161	0.875307769	-0.403884884	0.038489419
AT1G47290	3BETAHSD/D1	-0.981461927	0.108282384	-1.331948783	0.010663269	-0.880508614	0.010322757	-0.117666811	0.953045007	-0.074003186	0.907912238	0.394574723	0.064539726
AT3G02250	AT3G02250	-0.620496234	0.229613589	-1.337289774	0.000326621	-0.467397862	0.062008074	0.162373414	0.839420713	0.131481674	0.750474625	0.200270866	0.265577414
AT1G20840	TMT1	-0.622189358	0.213017091	-1.34251231	0.000159002	-0.134866418	0.722548919	-0.278067344	0.66524984	-0.529551988	0.066817393	-0.088832401	0.711515593
AT4G03550	GSL05	-0.432803309	0.29728592	-1.342956975	5.73473E-12	-0.324595052	0.142546482	-0.609595268	0.230088373	-0.273320989	0.26185804	-0.275392153	0.059219858
AT3G53380	AT3G53380	0.444098856	0.52947548	-1.345329534	0.000118995	-0.116458448	0.778025748	-0.264385949	0.78939283	-0.034894124	0.969007919	0.292668939	0.106937895
AT5G10400	AT5G10400	-0.731695894	0.27043599	-1.349073328	0.000105074	-0.518836167	0.611645933	0.029175912	0.992792909	0.339563004	0.22503462	0.031067791	0.95153083

AT1G77630	LYM3	-0.751163016	0.311121932	-1.349416895	0.016628993	-0.717738958	0.16926646	-0.525810989	0.703671882	-0.347055692	0.358428952	-0.062966102	0.879024324
AT5G60040	NRPC1	-0.299556121	0.612075454	-1.350977814	0.000904035	-0.198999966	0.606725184	-0.208776797	0.941364115	-0.002780617	0.99783138	0.211023586	0.401698665
AT1G21740	AT1G21740	-0.814031948	0.480009105	-1.352751415	0.031252667	-0.530116551	0.368546015	-0.300207243	0.976040445	0.158910951	0.802042097	0.133068857	0.537689863
AT4G25870	AT4G25870	-1.269595326	0.072270482	-1.35533469	0.000263955	-0.636097428	0.045742697	-0.07810731	0.982183273	0.352928058	0.341313856	0.045140204	0.883751418
AT1G51940	LYK3	-0.936005958	0.015138941	-1.35614934	0.00065294	-0.75121389	0.001154114	-0.299254979	0.719664849	-0.17138005	0.641522688	0.051144722	0.887714476
AT3G62150	ABCB21	-0.986681586	0.013466643	-1.360154473	1.65337E-08	-0.856740864	5.23751E-05	0.00737164	0.995194798	-0.599117453	0.067708278	-0.504396001	0.004410282
AT5G02370	AT5G02370	-0.812178376	0.16392321	-1.361171476	0.000131488	-0.95034672	0.105852458	0.539016574	0.676109289	0.060812405	0.957156296	0.743683511	0.000173518
AT2G27040	AGO4	-0.865165546	0.077623713	-1.361422021	3.38221E-05	-0.949522542	0.002867492	-0.311417942	0.886336463	0.015259016	0.98143759	0.426571416	0.013625805
AT3G47700	MAG2	-0.693351265	0.227719436	-1.362702528	0.011496307	-0.185485492	0.706237528	-0.540699104	0.770575751	0.104245077	0.916204809	-0.074864339	0.8161522
AT3G28040	AT3G28040	-0.672566722	0.055170469	-1.363012465	7.71324E-15	-0.913685487	2.75264E-06	-0.394468403	0.606948021	-0.186691867	0.559683277	0.311805889	0.205610908
AT4G37750	ANT	-0.543629176	0.342124213	-1.364269281	0.000517555	0.336301448	0.143718206	0.131436952	0.951485287	0.135919936	0.732309988	0.288219738	0.140320118
AT2G36340	AT2G36340	0.088406265	0.939161007	-1.366416775	0.027160574	0.086866731	0.87240166	-0.079035762	0.968504529	0.168546405	0.799125779	-0.51578841	0.010528669
AT1G54580	ACP2	-0.438640187	0.378081507	-1.367286375	2.02572E-05	-0.97151959	4.21346E-11	-0.288811864	0.870506552	0.361015762	0.017397275	0.359390781	0.011860144
AT1G60700	AT1G60700	-0.23327484	0.76784209	-1.368094954	0.001170558	-0.380849835	0.4695086	0.34013442	0.861588972	0.078173396	0.948116861	0.854856566	0.00098175
AT1G14290	SBH2	-0.879763064	0.003087756	-1.369233828	4.21707E-12	-0.779737562	7.55178E-05	0.336110995	0.759702709	-0.048186515	0.943757106	0.478418929	0.007167948
AT4G29720	PAO5	-0.247702372	0.751862258	-1.374908057	0.000802704	-0.494481946	0.055100528	-0.525498018	0.691895802	-0.422394058	0.200001282	-0.530290138	0.000140148
AT1G20410	AT1G20410	-0.600814619	0.510143725	-1.377689015	0.011250472	-0.132253836	0.69894638	-0.06518308	0.981578919	-0.166949116	0.765152482	0.146151372	0.449034307
AT2G21050	LAX2	-0.862688366	0.161316629	-1.379058545	5.02155E-06	-0.923416262	0.004561424	-0.306033129	0.871819231	-0.195480047	0.60011669	-0.265276749	0.205021194
AT1G48880	TBL7	-0.810506249	0.36182327	-1.37989905	0.03026962	-0.971917744	0.064716094	-0.470797932	0.849878329	0.029942096	0.985120284	0.182671536	0.697125935
AT5G22580	AT5G22580	1.016894752	0.569382446	-1.379949106	0.017848896	-0.495883653	0.40272043	-0.431644227	0.849063356	0.025013246	0.947746874	0.865170833	2.66921E-08
AT1G10750	AT1G10750	-1.355718046	0.051846621	-1.384098223	0.045690065	-0.358762658	0.651854951	-0.099762885	0.947241707	0.188933511	0.845336685	-0.464786753	0.042114491
AT5G18930	BUD2	0.128201329	0.902459138	-1.384753926	0.040818613	-0.97911921	0.077046008	-0.077359289	0.975645787	0.059115396	0.979081111	-0.026410752	0.964563269
AT5G20450	AT5G20450	-0.107911745	0.922043003	-1.386405306	0.040294031			0.489725098	0.692108738	0.135432062	0.909436033		
AT1G63480	AT1G63480	-0.871796667	0.339653767	-1.386517036	0.026678037	-0.095494227	0.862457636	0.14945989	0.899163675	0.400179589	0.505696968	0.390320696	0.054223443
AT5G44370	PHT4;6	-0.316608328	0.743804567	-1.388278057	0.017634393	-0.174227842	0.784810661	-0.269751437	0.834203073	-0.504590918	0.185313217	-0.330714818	0.131697112
AT1G77720	PPK1	-0.757578822	0.092996687	-1.388964183	0.003107315	0.008395523	0.988371325	-0.309185492	0.799598167	-0.205059158	0.680389077	0.107540094	0.682261652
AT4G13572	AT4G13572	-1.965561561	0.468465504	-1.394319134	0.002228495	-0.149876498	0.651484965	3.865479172	NA			-0.624425988	0.354157286
AT5G14730	AT5G14730	0.900685634	0.079941778	-1.396903598	0.034763764	-0.124101144	0.830572255	-1.153270864	0.433558429	-0.413473109	0.468003266	-0.805988977	0.000454261
AT5G16690	ORC3	-1.372536637	0.059268705	-1.397180267	0.028435326	-0.2677999	0.701699381	0.05021895	0.990915497	0.003140532	0.998849292	0.421615633	0.21546659
AT3G29375	AT3G29375	-1.061573114	0.156032672	-1.399082356	0.001243816	0.717267313	0.01442126	-0.419622544	0.663046749	-0.362714508	0.398044008	0.034333261	0.877162488
AT5G08415	AT5G08415	-0.783867345	0.011698861	-1.400172916	1.26333E-08	-0.893579996	0.000776532	0.210205676	0.842959841	-0.087901597	0.889895272	0.05272813	0.842535773
AT3G13190	AT3G13190	0.677804995	0.346982987	-1.401611496	0.001491401	-0.445369978	0.354398923	0.002817175	0.9987964	0.443797394	0.244825051	0.569262371	0.002135031
AT5G04770	CAT6	-0.724139972	0.127322026	-1.404109031	0.001925433	-0.242602682	0.660266961	-0.368131073	0.685495817	-0.040558702	0.953851164	0.110458379	0.753864702
AT1G16390	3-10月	-0.917641568	0.215994667	-1.406739623	0.013862382	-2.875973353	0.115082999	-0.27593357	0.84545588	-0.48209993	0.380762967	1.811495698	0.133955451
AT2G25737	AT2G25737	-0.619953295	0.416303748	-1.42458991	0.015530774	-0.527327176	0.204528351	-0.22316767	0.88065104	0.099542909	0.938376901	0.331134912	0.272980428
AT2G38970	AT2G38970	-1.121315123	0.293181795	-1.428197233	0.009511386	0.407103834	0.055710061	0.452644418	0.742807747	0.068842643	0.933077231	0.044317496	0.818125217
AT2G44830	AT2G44830	0.001139532	0.999583563	-1.430322	0.000837529	-0.602658662	0.127100309	-0.114893539	0.963305035	0.14853774	0.715410636	0.426586754	0.009287423
AT5G62520	SRO5	-0.633439077	0.461222059	-1.435812312	0.013706913	0.544131383	0.531789465	0.420106962	0.907707664	-1.167668608	0.415638203	3.022883819	0.08667291
AT4G36920	AP2	-0.3277841	0.409200657	-1.438205666	0.000135449	-0.47593123	0.305526545	0.193665289	0.783775657	0.321600052	0.357040463	0.192305155	0.445718493
AT2G23840	AT2G23840	-1.258795492	0.077017447	-1.438315702	0.0003371	-0.638220906	0.009403839	4.791036384	0.66524984	1.850847	0.326937807	0.373418853	0.572318101
AT2G47240	LACS1	-0.972961722	0.106032196	-1.44421365	7.68099E-06	-0.855014646	0.000267493	0.140705978	0.924925439	0.343558059	0.272234729	0.997756442	8.28819E-16
AT1G09820	AT1G09820	0.024121493	0.9872392	-1.449418279	0.036780283	-0.533358605	0.194487755	-0.847692005	0.663725004	0.321876989	0.64514601	-0.048835397	0.87531703
AT3G23880	AT3G23880	-0.21489552	0.668475598	-1.451937715	0.009309539	-0.16028793	0.394263238	0.160317776	0.876015705	0.466250721	0.037100036	0.301462496	0.078842925
AT3G58980	AT3G58980	-0.76531377	0.290294685	-1.458235579	0.044029167	-1.530617824	0.231896165	0.235549788	0.921639209	0.412789787	0.701511279	0.466045582	0.315956546
AT2G06040	AT2G06040	-0.712831029	0.15543532	-1.459209195	0.000558176	-0.579487446	0.125013349	-0.248168458	0.781040256	0.526761478	0.248762312	0.246156737	0.225163865
AT1G50360	VIIIA	-0.161295845	0.80855143	-1.45992246	7.32608E-06	-0.456574108	0.12256878	-0.284995286	0.776796299	-0.052808505	0.927080141	-0.129902791	0.564984661
AT5G66750	CHR1	-0.853384489	0.36857469	-1.461553826	0.013690456	-0.741008922	0.092553477	-0.262092984	0.910198054	0.324088991	0.489245379	0.566548046	0.00144681
AT5G44730	AT5G44730	-0.165877742	0.824857231	-1.471578941	0.00440109	-0.92083272	0.002848156	0.378426809	0.782181302	0.462145535	0.195376234	0.662426177	0.000549598
AT1G69330	AT1G69330	-0.358951408	0.610990127	-1.471977609	0.024206462	-0.80412764	0.009531556	-0.325767819	0.731041294	-0.156836436	0.847119924	-0.175153665	0.565187391
AT4G17910	AT4G17910	-0.675576615	0.305105679	-1.479885909	0.001392135	-0.462708724	0.433287615	-0.319550814	0.911720308	0.199596368	0.739817395	-0.110490734	0.660730283
AT5G42720	AT5G42720	-0.733739106	0.387834765	-1.48496938	0.019062544	-0.911765107	0.062558721	-0.493522353	0.706790319	-0.350214071	0.323767852	0.387715207	0.067600057
AT5G65970	MLO10	0.569563343	0.270788946	-1.486328927	9.60747E-05	-1.044969722	0.112502096	-0.758046135	0.611321113	-0.359888238	0.247798327	-0.951036642	2.29276E-05
AT5G53020	AT5G53020	-0.465149575	0.650138236	-1.48770704	0.000958827	-0.878105476	0.024471278	-0.364698667	0.855010607	-0.064143743	0.921888716	-0.425814691	0.058669273
AT1G57700	AT1G57700	-0.436265595	0.656220188	-1.493602271	0.001745483	-1.025201574	0.088187165	-0.501800225	0.836801579	-0.170161133	0.796508201	0.380894449	0.20286336
AT1G31150	AT1G31150	-0.810902768	0.137526978	-1.4937761	0.001014732	-0.737256025	0.051813257	0.130994284	0.935083701	0.024585795	0.9767935	0.424024184	0.033391414
AT3G54650	FBL17	-0.933334934	0.017320528	-1.494569755	2.33103E-05	-0.278449207	0.20982097	0.026361996	0.982749202	-0.067482233	0.891860506	0.539604234	0.000216727
AT1G57820	VIM1	-0.122766766	NA	-1.496527185	3.47776E-05	-0.382906569	0.190336949	0.158665415	0.927832298	0.272633006	0.475137305	0.596894888	3.30585E-05
AT5G06550	AT5G06550	-0.266152862	0.789160167	-1.498630505	0.028425071	-0.13733068	0.842237571	-0.125666201	0.958809354	0.588871511	0.055777087	-0.196517977	0.276246915
AT3G12550	FDM3	-0.589042846	0.459632352	-1.500252602	0.000126021	-0.850802138	0.034071845	-0.247916783	0.897433865	-0.227499573	0.67431887	0.211931542	0.431577584
AT4G22755	None	-0.536848768	0.401275543	-1.502217589	0.015083987			0.332170823	0.866579819	-0.070674091	0.945176019		
AT2G22770	NAI1	-0.71477184	0.305416687	-1.503641481	2.10413E-05	-0.707431882	0.142551555	-0.286024635	0.874751582	0.531049458	0.032971059	-0.575985749	0.010819455
AT3G01450	AT3G01450	-0.904962529	0.311500078	-1.504851021	0.017656462	-0.765532029	0.137625227	0.571623965	0.646729152	0.941498541	0.025586966	0.763763671	0.000315605
AT2G47160	BOR1	-0.29637974	0.554455069	-1.513263578	0.000908251	-0.784874869	0.000690468	-0.755743943	0.452963543	-0.365277737	0.066481086	-0.29068198	0.05205206
AT1G13180	DIS1	-1.041333631	0.055369682	-1.515024686	0.002762904	-0.066538072	0.894087755	-0.10489015	0.966082349	0.146069807	0.850540216	0.231608149	0.27160563
AT3G58790	GAUT15	-0.782826403	0.152359847	-1.515546582	0.000222961	-0.545342311	0.156151485	-0.182589645	0.895706838	-0.459116052	0.473771238	0.237200224	0.319158605
AT1G15490	AT1G15490	-0.838671647	0.116336093	-1.52329216	0.00013154	0.043979624	0.949629357	-1.075561843	0.314019385	-0.400221836	0.429416497	-0.218855337	0.327796023
AT5G08150	SOB5	-0.112124774	0.917554404	-1.524625856	0.000270282	-0.455505508	0.276390752	0.827341971	0.66524984	-0.254449612	0.878941396	0.643772574	0.141598375
AT5G14140	AT5G14140	-0.649748961	0.458891227	-1.546065776	0.012706379	-0.265620416	0.615178075	0.386456307	0.809492797	0.308619362	0.636492164	0.176425346	0.624189622
AT1G03830	AT1G03830	-0.66409647	0.381870662	-1.548629224	0.004550534	-0.574732672	0.100984141	-0.156081527	0.92894449	-0.019754168	0.984002268	0.213787194	0.227161821
AT5G45820	CIPK20	-0.970569083	0.073759951	-1.549924178	4.46489E-10	-0.750639203	2.23324E-05	6.808133397	0.425775966	6.0836118	0.301120228		
AT5G59010	BSK5	-0.661803339	0.335585451	-1.559562849	0.00050281	-0.546765831	0.040462925	-0.54112785	0.712887303	-0.213154179	0.573217541	0.114170359	0.555817233
AT1G16445	AT1G16445	-1.006252739	0.178035601	-1.569228855	0.029110681	-1.006303414	0.05555433	-0.930186908	0.637614678	-0.499111556	0.545156804	-0.008696278	0.984723501
AT2G45490	AUR3	-0.824712652	0.360167394	-1.570434328	0.011411377	-0.672506485	0.424263853	0.960783897	0.761187158	0.941833589	0.073794382	0.480421577	0.04978849
AT4G06075	None	-2.024537876	0.065419122	-1.588359478	0.003363751			2.791052024	0.587300556	1.065567829	0.756378496		
AT1G75520	SRS5	0.364350487	0.727630474	-1.588759214	0.008070562	-0.017073846	0.986142943	-0.181596697	0.961565092	0.457292146	0.510156577	-0.497889335	0.10834322
AT5G23420	HMGB6	-0.404470052	0.543614052	-1.595124736	5.52127E-07	-0.900792859	0.003379515	0.026693449	0.987824384	0.135182274	0.755493303	-0.072004538	0.755911033
AT1G10850	AT1G10850	-0.72302209	0.416218882	-1.596996963	1.48816E-05	-0.933583428	0.008705262	-0.785883899	0.709813898	-0.235043927	0.522909665	-0.07807042	0.733851256
AT1G22900	AT1G22900	-1.266260168	0.093581001	-1.598041561	0.029514274	-1.442243037	0.32950132	-1.000424079	0.574003303	-0.197799816	0.885326824	-0.378791501	0.552951241
AT5G12900	AT5G12900	-0.993474987	0.108934736	-1.598826411	7.99694E-05	-0.599194869	0.1010122	0.029752101	0.979952353	-0.262636419	0.491449838	-0.192252583	0.339618103
AT5G24750	AT5G24750	-1.182966913	0.053274964	-1.602921017	0.04574995	-0.011011658	0.983600618	-0.157540568	0.963956318	0.309053156	0.596277828	0.921618375	3.59203E-06
AT2G41940	ZFP8	-0.703192115	0.191612645	-1.606247511	2.14569E-05	-0.569770531	0.070905592	-0.111632236	0.946414416	0.002302678	0.99783138	0.114780951	0.714700217
AT1G64640	ENODL8	-0.522171776	0.635026317	-1.609032278	8.02158E-06	-1.005246973	0.141850875	-1.214900362	0.587695712	-0.635080503	0.002419489	-0.264198282	0.058759717
AT3G10080	AT3G10080	-0.709935391	0.345026093	-1.611637273	1.71422E-05	-0.951719267	0.008051432	-0.495357369	0.731041294	-0.252030703	0.57285663	-0.461575798	0.099178247
AT4G35270	AT4G35270	-0.931438859	0.056333046	-1.61600069	9.20217E-06	-0.844337474	0.054991552	-0.930291445	0.697407195	-0.190262745	0.76508716	-0.386298766	0.118661544
AT5G23940	PEL3	0.333368538	0.780638083	-1.618507575	0.000486768	-0.070880744	0.858886685	0.031169668	0.993618504	0.260010023	0.453380046	0.273495607	0.038365604
AT4G39320	AT4G39320	-0.855243843	0.263905113	-1.621900129	0.012295256	-1.085248602	0.279524845	-0.973790951	0.659255524	-0.651413219	0.082999383	-0.58515544	0.013930083
AT4G15790	AT4G15790	-0.719705488	0.306593431	-1.626858601	0.002293096	-0.316548155	0.534175806	-0.44507489	0.824436763	0.156346538	0.802042097	0.287744662	0.118595749
AT4G32890	GATA9	-1.324481254	0.160105866	-1.632475084	0.036024897	-0.67003972	0.236294604	-0.165197136	0.953923605	-0.331975851	0.485309912	0.11878431	0.73639711
AT3G44940	AT3G44940	0.035105319	0.978709893	-1.646487904	0.000226146	-0.826367712	0.063664177	-0.325157702	0.905793887	-0.048133187	0.951101524	-0.037552994	0.895783536
AT4G22860	AT4G22860	-1.600509619	0.052070372	-1.651251279	0.031571098	-0.93160105	0.174058006	0.232133885	0.963252175	0.437996267	0.516852689	0.918640892	3.80446E-06
AT1G11303	None	-1.596957791	0.177971178	-1.657022908	0.01210392			-0.415891123	0.720336606	0.609634004	0.395218257		
AT2G04650	AT2G04650	-1.132810844	0.157875793	-1.663003075	0.007417548	-2.479715161	0.056532202	-0.698944208	0.684184988	0.012296024	0.991628492	0.175896687	0.546694076
AT5G38110	ASF1B	-0.662020178	0.535777934	-1.664757098	0.002063565	-0.870202903	0.03054712	0.032020155	0.993556469	0.305189377	0.550184567	0.740149062	4.93596E-06
AT4G15920	SWEET17	-0.539591812	0.510994857	-1.667443504	0.013291125	-1.246582747	0.258523855	-0.740453347	0.637614678	-0.94061674	0.00070341	-0.97271031	4.30375E-07

AT1G80870	AT1G80870	-0.535150013	0.385587222	-1.670568439	0.033212418	-0.935331111	0.020020882	-0.651114224	0.676109289	-0.281132429	0.506154989	-0.609007807	0.001645476
AT5G46280	MCM3	-1.147837496	0.236771369	-1.680350752	0.004105255	-0.726623293	0.045850972	-0.164917985	0.966082349	0.090127608	0.891016552	0.151716082	0.587680674
AT1G49430	LACS2	-1.010660416	0.170797552	-1.684145953	3.52704E-08	-0.588367429	0.045719491	-0.830048106	0.482795025	-0.254076243	0.461925268	0.300791368	0.053092559
AT5G27920	AT5G27920	-0.807859817	0.00382502	-1.688796088	3.90972E-05	-0.082521173	0.777652816	0.152095831	0.914733185	0.004367267	0.996165588	0.487292609	0.00308796
AT5G66310	AT5G66310	-0.982387613	0.182029632	-1.68905319	0.039243619	-0.658995986	0.040585604	-0.413592901	0.766791062	-0.058371186	0.887277776	0.087960169	0.710342765
AT5G48360	AT5G48360	-0.1898456	0.826301167	-1.68911557	9.08908E-08	-0.714698083	0.005282227	0.160513887	0.921425607	0.311196706	0.466442631	0.642219267	0.000855452
AT1G11080	scpl31	-2.112898617	0.057174768	-1.696526121	0.004115293	-0.314691629	0.50891413	-0.906548256	0.705076831	-0.481187288	0.784214866	-0.187100483	0.359786882
AT1G13910	AT1G13910	-0.071789049	0.946768259	-1.698853888	0.009892421	-0.994648863	0.065342087	-0.568132525	0.664635521	0.199493959	0.821415128	-0.134789549	0.715195556
AT1G69370	CM3	-0.960416033	0.104111643	-1.703436353	0.003882482	-0.887150254	0.007082487	0.526102751	0.65378996	0.450440004	0.380682364	0.554133681	0.010784294
AT2G42840	PDF1	-0.620273015	NA	-1.710489923	4.94244E-24	-0.867932502	1.41579E-10	-0.62506093	0.708641472	-0.22813591	0.331296735	0.611607687	0.15772211
AT1G58070	AT1G58070	-0.539531909	0.459632352	-1.719764773	0.004111411	0.030926428	0.971232132	1.10465397	0.354109371	-0.164747586	0.866300056	0.269457466	0.609340774
AT2G26515	None	-0.855653044	0.564956791	-1.726496936	0.021568249			1.291395567	0.478317143	0.244696076	0.92081979		
AT5G61570	AT5G61570	-1.48948076	0.21180249	-1.730033439	0.030109578	-1.693632306	0.081530017	-1.093329246	0.64183064	0.061507903	0.959686797	-0.105971377	0.793696785
AT1G80370	CYCA2;4	-0.484889143	0.434215511	-1.732921982	0.001317231	0.266377042	0.680001788	0.798944446	0.577728436	0.643521606	0.204941626	0.531443889	0.027545111
AT2G43100	IPMI2	-2.500652615	0.142796883	-1.742057944	0.03011525	-2.316393616	0.321349687	0.432255857	0.9487076	-0.77466038	0.700995813	0.795418247	0.00085491
AT3G48440	AT3G48440	-0.499385726	0.605541763	-1.743584628	0.002734019	-0.716464937	0.154288797	-0.25400782	0.872669065	-0.274455929	0.735176002	-0.05656344	0.873433282
AT5G05240	AT5G05240	-1.857072891	NA	-1.746763424	0.007924607	-0.631857788	0.279366481	-0.108438417	0.957250593	-0.380206388	0.648463309	0.057141907	0.855988427
AT4G02060	PRL	-1.237783409	0.106638633	-1.748078967	3.34407E-08	-0.73420216	0.056973173	0.022407243	0.993804788	-0.083377751	0.896222696	0.023746566	0.946903519
AT5G20410	MGD2	-1.527984262	0.105664265	-1.748511471	0.011233315	-2.262736227	0.10100645	-0.856771949	0.631991778	-0.69588961	0.53558612	-0.419281929	0.420193558
AT2G01750	MAP70-3	-0.581669935	0.442409453	-1.750300834	9.65715E-05	0.06764792	0.886489138	-0.436495103	0.774301984	0.637556527	0.085456988	0.067177987	0.789765231
AT2G14860	AT2G14860	-0.987650047	0.313319667	-1.753601597	0.015884793	-0.513700658	0.514760353	-0.035656613	0.996799452	-0.076129627	0.963495178	0.360173888	0.240412427
AT3G05690	NF-YA2	0.315793728	0.752010811	-1.757170381	0.000529726	-0.844742051	0.069376336	-0.506768391	0.862230362	0.371039025	0.55798645	-0.539893015	0.002641508
AT5G04270	AT5G04270	-0.612157377	0.430165963	-1.761016189	0.000833772	-0.927747431	0.048705345	-0.145589961	0.944280349	-0.250107374	0.786585586	0.030236849	0.945870421
AT4G33400	AT4G33400	-0.297161425	0.706714014	-1.76530822	2.86947E-06	-0.63388827	0.204687118	-0.315324779	0.896533613	0.158714366	0.755775102	0.258558894	0.110982652
AT3G17460	AT3G17460	-1.42330932	0.124161417	-1.774572405	0.005551892	-0.43059743	0.473072716	-0.185950991	0.959825156	0.132584494	0.907644783	0.555302918	0.08386914
AT1G80280	AT1G80280	0.276737601	0.892278642	-1.776206599	0.000375115	-0.394539669	0.368921589	-0.512549349	0.810393493	0.015513323	0.98143759	0.31985059	0.098732509
AT5G04820	OFP13	0.558261194	0.424646983	-1.782108438	0.000138726	-0.978538488	0.001537897	-0.228752701	0.82814234	0.351197339	0.485857955	-0.273355633	0.308494201
AT5G05830	AT5G05830	-1.016128692	0.146216342	-1.790414208	0.000137388	-0.916684694	0.015050435	0.352887238	0.88581519	0.243389414	0.719219517	0.223483941	0.430883924
AT4G10800	AT4G10800	-0.00568006	0.996098493	-1.791324596	0.029671614	-0.765992978	0.29607528	0.304217197	0.926730556	0.509809699	0.531752878	-0.299451596	0.663868766
AT5G13960	SUVH4	-0.523113257	0.470658714	-1.792223686	0.000604565	-0.903472895	0.061613176	-0.242822939	0.963509602	0.597873823	0.09958401	0.205025723	0.32862821
AT3G07540	AT3G07540	-1.266949492	0.144912138	-1.793217042	6.91102E-08	-0.49889067	0.101499907	-0.642762956	0.618005886	-0.148791459	0.821418926	-0.045140931	0.878292965
AT1G11130	SUB	-1.110226119	0.188201713	-1.795171277	0.004049249	-0.564100067	0.109815655	-0.370536101	0.905084907	-0.480867828	0.063641582	0.181071512	0.55406104
AT2G41370	BOP2	-1.574653784	0.219833273	-1.795560725	0.000619684	-0.463406178	0.293679918	-0.236800732	0.914259529	-0.497373873	0.573996347	-0.101317008	0.786200819
AT3G13420	AT3G13420	-1.719801491	0.420998548	-1.807854652	0.038824323	-0.647660703	0.314083192	-1.088788741	0.819211867	-0.141108523	0.934226173	0.229656551	0.624955133
AT1G63080	AT1G63080	-0.658550315	0.46585282	-1.827300096	0.007774724	-0.509956698	0.514663546	0.467813089	0.869314843	-0.019745846	0.992611192	-0.186678062	0.663653401
AT3G63240	AT3G63240	-0.944249921	0.056959188	-1.82776504	0.00050783	-0.478662846	0.281396517	-0.575624556	0.707225978	-0.115136881	0.868188276	0.228513097	0.226759412
AT5G18700	AT5G18700	-1.405007714	0.212225079	-1.828651594	0.002090403	-0.826355279	0.029317298	-0.434711469	0.776796299	-0.034005632	0.974055498	0.524881265	0.014183421
AT2G02480	STI	-0.734491853	0.294342799	-1.831296662	0.002947094	-0.95932835	0.026117793	-0.739520505	0.760236762	-0.045947317	0.939428683	-0.198287617	0.330577103
AT3G53190	AT3G53190	-0.636174808	NA	-1.836873702	1.93984E-21	-0.753940618	0.028708757	-0.027118259	0.992593412	0.293667607	0.233701022	0.372853571	0.020496764
AT2G25530	AT2G25530	-1.21904733	0.170489914	-1.842911325	0.024604396	-1.04664982	0.151702698	-0.603458051	0.799315251	0.927284808	0.093661005	-0.024963057	0.965903331
AT1G63470	AT1G63470	-0.855866748	0.250896796	-1.849448029	5.64062E-06	-0.629080994	0.074721694	-0.426597793	0.794188368	0.301580747	0.57069623	0.391328693	0.047219541
AT5G10240	ASN3	-0.502622928	0.407119885	-1.852180931	2.82241E-09	-0.459165371	0.209778828	-0.394061282	0.846266574	0.018957908	0.975541657	0.15823577	0.473314689
AT2G36490	DML1	-1.07742593	0.067525167	-1.856111662	4.84549E-06	-0.478857654	0.037436994	0.055248337	0.97071238	0.216402017	0.55787595	0.709553406	3.85302E-05
AT4G04695	CPK31	-0.558169901	0.569314551	-1.86630836	0.005873388	-0.79560349	0.040531585	-0.566223652	0.855427008	-0.091325905	0.934318497	0.033516182	0.938293102
AT4G39235	AT4G39235	-1.116424946	0.068618722	-1.869727263	0.001398447	0.163921059	0.700360278	-0.181266306	0.959068856	0.066134273	0.964685707	-0.290300253	0.382079697
AT2G23700	AT2G23700	-0.435031705	0.720677613	-1.872785901	0.038819071	-1.096933376	0.167478174	-0.722206709	0.445120559	-0.096314257	0.864675247	0.142293731	0.650920534
AT5G08110	AT5G08110	-0.905175366	0.203383204	-1.87790726	4.76406E-08	-0.572896204	0.138579208	-0.714875151	0.684249334	-0.221224157	0.708887279	0.185129508	0.459878389
AT1G78650	POLD3	-0.294044612	0.703702664	-1.887850073	0.017151852	-0.847543239	0.004735265	0.090060077	0.958174755	-0.132757219	0.863713969	0.026398725	0.938469717
AT1G60810	ACLA-2	-0.652669514	0.331236028	-1.891184054	6.19057E-08	-0.490962932	0.006015632	-0.150223238	0.877665269	0.110801159	0.807923242	0.737602104	2.47284E-11
AT4G31360	AT4G31360	-0.954408251	0.205785229	-1.895320189	3.06588E-05	-0.947742123	0.043827504	-0.476142273	0.709616092	0.044270703	0.966304751	-0.409148617	0.157130759
AT2G25880	AUR2	-1.186509764	0.13419776	-1.896420174	0.005028944	-0.286451293	0.74126345	-1.125281429	0.721140128	0.286448638	0.674915955	0.629106236	0.000487323
AT3G17340	AT3G17340	-0.477818401	0.608893861	-1.897240018	0.000109153	-0.692843041	0.015930915	-0.445076501	0.732322487	-0.293308382	0.372809722	0.053366529	0.820998174
AT5G03615	None	-0.388916892	0.660115364	-1.905717442	4.10167E-13			2.881835398	0.054216083	0.006830231	0.99766313		
AT3G03350	AT3G03350	0.729780237	0.666001052	-1.910104333	0.039166332	-0.247256958	0.725993319	-0.746457963	0.714700562	0.334411467	0.73165232	0.071567129	0.900390808
AT3G25900	HMT-1	-0.644224947	0.573510285	-1.9133379	0.015270147	0.01053636	0.992268065	-0.885940742	0.644839236	-0.198329017	0.638629847	0.458239338	0.03453023
AT1G27120	AT1G27120	-0.448208427	0.649882611	-1.914019093	0.009756741	-1.518449116	0.05590489	-0.327072636	0.908903757	0.268857464	0.70395135	0.320147909	0.25879908
AT2G38460	IREG1	-1.499769256	0.064128602	-1.918449572	1.46398E-08	-0.930194632	3.19247E-05	0.820021336	0.652047062	-0.481433452	0.72508858	-0.625615851	0.164596715
AT2G02150	EMB2794	-0.689562542	0.646288791	-1.923438034	0.036379991	-0.805493828	0.358563291	0.314685628	0.876428092	0.238033846	0.802729715	-0.23965827	0.407688491
AT5G39760	HB23	-0.664941849	0.441196068	-1.927307042	5.35496E-06	-0.802329148	0.024746308	-0.864432691	0.62738272	0.110908133	0.866906715	-0.373226876	0.133576662
AT4G11080	3xHMG-box1	-1.060590493	0.242348482	-1.96275199	0.000148005	-0.899059425	0.236426787	0.258209619	0.950356778	0.125401363	0.887241345	0.680820295	0.0140603
AT1G50590	AT1G50590	-0.988785019	0.392228059	-1.96297854	0.017915504	-0.622255018	0.63455174	-0.866673332	0.637614678	0.088962488	0.963495178	-0.42266778	0.326004913
AT1G60630	AT1G60630	-0.065204672	0.944373487	-1.966016446	0.004967856	-0.528494373	0.421098487	0.209911862	0.964923674	0.085347293	0.928314082	0.366458463	0.164662083
AT1G62914	AT1G62914	-0.822840852	0.430741779	-1.970420436	0.029178145	-0.366331516	0.570425526	-0.388659313	0.843345258	0.410838474	0.682482982	-0.334270425	0.524633621
AT1G77460	AT1G77460	-0.735285411	0.365534497	-1.971373142	0.004059437	-0.930087047	0.00457008	-0.198089819	0.951851691	0.007385371	0.992519652	0.560638419	0.004465119
AT4G07400	VFB3	0.422799025	0.656220188	-1.971377753	2.77224E-05	-0.696006898	0.2323438	0.090729167	0.968416104	-0.279007996	0.78840313	-0.14688767	0.639429738
AT3G04310	AT3G04310	0.755454026	0.596224574	-1.984630005	0.021258941	-0.667147158	0.193335768	-0.483525462	0.769724647	0.633027894	0.485857955	-0.770793052	0.011991828
AT1G73360	HDG11	-0.47689921	0.701807703	-1.988334391	0.006982566	-0.81258428	0.329464801	0.096968757	0.977279226	0.266845502	0.838103967	0.826164586	0.003733058
AT4G19380	AT4G19380	-0.740543509	0.304415798	-1.991558245	0.002902793	-0.96491491	0.431784188	0.045675788	0.982749202	-0.27073327	0.738244261	0.087521828	0.833417297
AT3G51340	AT3G51340	-1.372024655	0.20464594	-1.993951317	0.004774005	0.215391443	0.843174713	-0.388173616	0.907707664	-0.350837683	0.786724861	0.086738044	0.906972637
AT5G41120	AT5G41120	-0.63209059	0.525535334	-1.995153695	0.005233067	-0.670792728	0.321466748	-0.670729467	0.812547619	0.352108199	0.643763442	0.541335469	0.046634881
AT2G03480	QUL2	-1.437539614	0.052368176	-2.003906246	0.002682149	-0.543763769	0.270933677	-0.953257392	0.704470077	-0.144483668	0.811041629	-0.072379068	0.770246672
AT3G14190	AT3G14190	0.112637986	0.930035011	-2.007003294	1.26398E-05	-1.631110102	0.486276183	0.059194488	0.987576531	0.234376142	0.70093096	0.487439026	0.033275722
AT4G18640	MRH1	-1.423685498	0.087985613	-2.017447017	0.008113583	-0.700658915	0.214152484	-0.342932317	0.81577697	-0.574475743	0.157494921	0.243572987	0.256766304
AT1G20480	AT1G20480	-1.084243668	0.060854784	-2.036785364	0.005102024	0.175933841	0.771566542	-0.144959857	0.954565337	0.097567565	0.957156296	0.811407591	0.009553486
AT5G50390	EMB3141	-0.950039434	0.021944446	-2.040465377	0.001011237	-0.72012809	0.113756782	0.640026752	0.539736869	-0.184675826	0.783448836	-0.008236374	0.988206943
AT5G40400	AT5G40400	-0.144302777	0.917523494	-2.04321715	0.00404526	0.404828101	0.491732111	-0.263978656	0.920233522	-0.741374129	0.237554778	-0.427212263	0.137733582
AT5G07140	AT5G07140	-1.142022407	0.226298119	-2.050322489	0.004314089	-0.843211152	0.16743539	-0.29477453	0.946164167	0.712051777	0.357977132	0.478367904	0.065162439
AT1G71870	AT1G71870	-0.027178653	0.977582662	-2.052561932	0.003033433	0.191490833	0.835423201	-0.643089914	0.760236762	0.563116929	0.363657818	0.076965181	0.863961739
AT1G64300	AT1G64300	-1.076765116	0.372340116	-2.053809762	0.045991861	-0.83258628	0.389981228	0.379836741	0.957474117	-0.426019292	0.758512148	-0.33531684	0.38993934
AT4G09510	CINV2	-0.917636132	0.186788491	-2.055312788	1.09719E-05	-0.854575758	0.011698992	0.366017931	0.724202396	0.147863963	0.791798458	0.425454929	0.074396
AT4G14310	AT4G14310	-1.146963509	0.155277188	-2.057524982	0.000386176	-0.854171278	0.020905785	-0.109966444	0.966082349	-0.285008204	0.478338322	0.366914963	0.071921713
AT3G11040	ENGase85B	-0.463554938	0.635773158	-2.065162238	0.000621751	-0.353046884	0.600505638	0.410572569	0.697407195	-0.221295928	0.749506811	0.407303836	0.123103433
AT3G49660	WDR5a	-0.618441192	0.53076928	-2.073834673	0.000444906	0.431452845	0.486104252	-0.197253439	0.947082547	-0.168389719	0.895385501	0.174997558	0.633459467
AT2G38570	AT2G38570	-0.933765166	0.258822134	-2.07458438	0.004967571	-1.010373732	0.109176357	-0.773630806	0.885755209	0.993436855	0.30299373	0.122037431	0.858765458
AT4G02290	GH9B13	1.354045322	0.56043581	-2.082032081	9.14529E-05	-0.825251647	0.114910734	-1.376546418	0.482795025	-0.471752689	0.019125497	-0.08298893	0.745726532
AT1G05205	AT1G05205	-0.6623598	0.621317444	-2.100883213	0.024692672	-0.788256671	0.01798964	-0.936576134	0.84461645	0.32212322	0.813363006	0.282383607	0.155237505
AT2G04235	AT2G04235	-0.699629025	0.371122918	-2.102093756	4.63404E-06	-0.850129727	0.215975039	-0.644918954	0.823579145	-0.052889279	0.964601576	0.531166369	0.056400318
AT3G45160	AT3G45160	0.2352097	0.864380668	-2.102961219	0.007242238	-0.56100612	0.574170459	0.468471492	0.863704625	-0.006496144	0.997749369	0.185641805	0.569262556
AT3G42670	CHR38	-0.276983177	0.762313285	-2.104005824	0.01283283	-1.161703074	0.055220279	0.022905297	0.991559366	0.498606142	0.211913228	0.203646762	0.451876822
AT2G43990	AT2G43990	-1.688749925	0.141176598	-2.111189242	0.016191328	-1.316218659	0.052727024	-0.701286433	0.863704625	0.64277186	0.302924516	0.205598022	0.543901294
AT1G60660	CB5LP	-0.487456227	0.406553351	-2.115283984	4.10215E-05	-0.945041775	0.002723009	-0.722405281	0.632534636	0.175684983	0.739298913	-0.084684725	0.830419747
AT4G29030	AT4G29030	0.401048398	NA	-2.127014527	2.96618E-08	-0.831103474	0.056751335	-0.136074311	0.972495118	0.164862991	0.826378011	-0.393575452	0.510620683
AT5G43990	SUVR2	-0.518589226	0.676104433	-2.133882388	0.000405698	-1.105629265	0.076814459	-0.123316795	0.963499596	0.52617866	0.360033758	0.80609077	1.24842E-05
AT5G03250	AT5G03250	0.855209806	0.652214896	-2.137840815	0.033498681	-0.094534112	0.91055655	-0.732426903	0.777083998	-0.206838806	0.9202103	0.437124782	0.253381668

AT3G48260	WNK3	-1.085159939	0.294691079	-2.138070446	0.001701156	-0.222158592	0.866228248	-0.703521734	0.696374342	-0.567153642	0.060706124	-0.890076136	0.000790341
AT5G18010	SAUR19	0.321834626	0.760597114	-2.151268071	0.021665778	-0.792459001	0.222822152	-0.280872446	0.936845219	0.178506607	0.8608857	-0.160069174	0.651292447
AT1G07370	PCNA1	-0.751301755	0.591422474	-2.165204751	0.003903889	-1.091743341	0.058718021	-0.120424744	0.975373509	-0.055493313	0.951316191	-0.054723602	0.867604777
AT2G42900	AT2G42900	-0.853985765	0.066750415	-2.168129408	2.23677E-08	-0.874972253	0.018029817	-0.240323246	0.83083887	-0.389679786	0.381649427	0.219396317	0.496284941
AT5G15150	HB-3	0.292405121	0.799030872	-2.182712581	0.000109527	-0.551145935	0.305258261	-0.38666765	0.791359656	-0.266423056	0.522944176	-0.063929435	0.788530683
AT1G68980	AT1G68980	-0.480408476	0.654831119	-2.201125686	0.001216797	-0.575630371	0.342196788	-0.082833185	0.973896678	0.370111772	0.629318502	-0.322717271	0.286522933
AT2G46780	AT2G46780	-1.095868449	0.144796054	-2.203024978	1.30666E-07	-0.811686685	0.081313009	-0.33936707	0.897609815	-0.170466657	0.694512158	0.36479976	0.109031658
AT1G23360	MENG	-0.790645282	0.155072408	-2.22046514	1.13431E-05	-0.554831201	0.028713091	0.094854015	0.963509602	0.501149856	0.16858815	0.477552539	0.016289992
AT3G13674	AT3G13674	0.040996942	0.98143943	-2.220514069	0.002486205	-1.603478547	0.316555409	-0.110667387	0.978041029	0.510354963	0.673329207	0.346647352	0.336250435
AT2G32765	SUMO5	-0.636257368	0.697573415	-2.227735694	0.003317483	-0.709467735	0.309678122	-0.473297692	0.862994029	0.392668321	0.53525964	0.578661467	0.017160334
AT2G16390	DRD1	-0.707459286	0.365534497	-2.235144758	6.41772E-11	-0.457368337	0.308835077	-0.01584527	0.993527973	0.55530231	0.177684996	0.683176795	5.96509E-05
AT2G17525	AT2G17525	-0.387490631	0.692241839	-2.239754585	0.002842319	-0.902200286	0.03615537	0.207541142	0.962881682	0.137007466	0.928751803	0.228122	0.56078459
AT4G34760	AT4G34760	-0.912193361	0.342349434	-2.243617639	5.45429E-07	-0.19099085	0.554704237	-0.046389152	0.984609535	0.368501708	0.186598083	-0.167070329	0.482330226
AT3G02930	AT3G02930	0.461865349	0.750185144	-2.256711142	0.000728389	-0.715904615	0.239570424	-0.287042961	0.931142856	0.088438579	0.910605748	-0.177933325	0.369603712
AT1G06360	AT1G06360	-1.585163765	0.0722803	-2.264815694	6.68174E-07	-0.342415383	0.141819627	-0.755587679	0.782971455	-0.088171919	0.883555449	0.387335282	0.00391384
AT1G04425	AT1G04425	-0.350011863	NA	-2.273507689	0.000537982			0.415267162	0.953045007	0.87636316	0.298368981		
AT1G31335	AT1G31335	-0.964510458	0.033803104	-2.281216819	0.001023004	-0.24508703	0.575616291	-0.895166264	0.352570033	-0.346505032	0.550143794	0.631971364	0.053716702
AT1G78260	AT1G78260	-1.258308275	0.068618722	-2.283832243	5.60255E-07	-0.918353857	0.108748272	-0.486358181	0.824677059	0.009495462	0.992281921	0.709167281	0.00251336
AT5G64110	AT5G64110	-0.865601705	0.357820627	-2.292375378	2.71066E-07	-0.39945483	0.656383395	-0.31023443	0.917296618	0.703997303	0.486060958	0.279086238	0.191274428
AT5G60150	AT5G60150	-1.015938448	NA	-2.299846019	0.001811802	-1.228477482	0.122099089	-0.733661212	0.888853109	0.102534209	0.950068828	0.658408004	0.026790906
AT1G28290	AGP31	0.250171572	0.902433732	-2.30214323	1.48856E-19	-0.780612845	2.31466E-07	-0.941753423	0.637815859	-0.558390848	0.00016199	0.288835721	0.178354985
AT3G18000	XPL1	-1.112048746	0.496764455	-2.313253952	0.00026954			-0.458136413	0.855143489	-0.241335344	0.424170545		
AT2G10931	AT2G10931	0.442272089	0.798330983	-2.337062277	0.033195092	-4.191543895	0.441624455	-0.07777421	0.981525175	-0.345664019	0.67282695	-0.416086559	0.958043778
AT3G62060	AT3G62060	-0.38048414	0.772564309	-2.386178785	0.009577735	-0.636112902	0.106809233	-0.925254527	0.588988003	0.119362534	0.867911751	0.670790611	0.001599909
AT2G05760	AT2G05760	-0.479229526	0.665597848	-2.400451671	0.040538908	-0.896659201	0.39987984	0.066335517	0.978443175	0.054637134	0.970129942	0.43712941	0.139888682
AT5G01110	AT5G01110	-0.746207799	0.376699042	-2.404822388	0.020570552	0.059482344	0.948315212	-0.05348325	0.990915497	0.060061478	0.966498605	0.055962865	0.921973859
AT4G16970	AT4G16970	-1.607020459	0.221663177	-2.410466208	0.001860771	-0.657151938	0.117584761	-0.020165594	0.994602444	0.054471145	0.964378519	0.261328168	0.343799147
AT2G31270	CDT1A	-1.336677319	0.123547524	-2.416102223	0.002575053	-0.857575753	0.040163132	-0.767736559	0.927465214	-0.428301566	0.377377981	0.019734491	0.963643927
AT1G51460	ABCG13	0.579037009	0.691344221	-2.438172041	0.001515685	-0.599373119	0.644864601	-0.222672849	0.970881852	0.39291732	0.645375892	0.222683156	0.499568376
AT3G44960	AT3G44960	-1.205189428	0.173916643	-2.450108179	0.016613033	-2.832349421	0.063745173	-0.335915917	0.963341405	1.099452996	0.157634587	0.360627044	0.518271304
AT3G21950	AT3G21950	-1.818846136	0.320488068	-2.450690174	0.001522775			0.691149387	0.843525143	-0.527530167	0.824058587		
AT5G05590	PAI2	-0.641550439	0.720197626	-2.471480086	0.00571116	-0.068153228	0.86661409	0.442854255	0.947241707	0.543233333	0.455181286	0.696171301	0.000417202
AT5G17160	AT5G17160	-0.110743262	0.95434083	-2.478636723	6.82248E-05	-0.89603759	0.099980107	-0.210083468	0.93447719	0.037067488	0.963874517	0.504854327	0.000179897
AT5G19670	AT5G19670	-0.8901094	0.192262131	-2.509786946	3.2612E-05	-0.482806684	0.260621153	0.043284736	0.98831332	-0.272909583	0.690561399	-0.055416388	0.846296808
AT2G07170	AT2G07170	-1.314037928	0.050105743	-2.566603629	7.09935E-07	-0.759975922	0.201929923	-0.121172195	0.972495118	0.279907678	0.570610039	0.708331882	0.000285778
AT1G08840	emb2411	-0.894349444	0.154659838	-2.636586005	1.137E-06	-0.405604985	0.439331113	0.17516906	0.963509602	-0.048499517	0.960463787	0.217509301	0.55499653
AT4G29310	AT4G29310	-1.040063829	0.21409699	-2.653502017	8.71725E-05	-0.946239124	0.028621012	-0.071342102	0.97809523	0.069714285	0.91692451	0.540356353	0.000251297
AT2G21880	RAB7A	-1.28078824	0.193241891	-2.679149742	0.001783973	-1.25342518	0.117945303	-0.863596457	0.724068414	-0.626816241	0.2436893	-0.37902956	0.147038971
AT3G63430	TRM5	0.175607514	0.904755827	-2.806789004	3.75134E-05	-0.979955176	0.11658413	0.09257835	0.972402645	0.08766369	0.924627441	0.633452171	0.001070617
AT2G45870	AT2G45870	-1.664259296	0.17319919	-2.841577604	0.013403996	-0.836199896	0.409004052	-0.412983824	0.951485287	0.164929969	0.931302326	0.326130336	0.247907896
AT5G45730	AT5G45730	-0.397083682	0.871128297	-2.8422968	0.046563008	-0.97360992	0.295735216	0.200979226	0.944280349	0.170563865	0.929498792	0.737460087	0.038188159
AT3G05600	AT3G05600	-1.087533673	NA	-2.845857394	1.02491E-06	-0.787640885	0.105231675	0.177886454	0.972495118	0.645079731	0.261998738	0.969370219	1.09894E-07
AT1G38131	AT1G38131	-0.350994259	0.891445738	-2.873353134	0.032426299	-2.736272354	0.051284149	-0.386749425	0.955547263	-0.288790985	0.838708765	0.469009837	0.252166
AT5G24580	AT5G24580	-0.747439253	0.357820627	-2.907284629	1.39514E-07	-0.916821733	0.043562608	-0.641648722	0.696374342	-0.413074955	0.219576651	-0.116730779	0.69386546
AT4G26260	MIOX4	-1.598699367	0.133856754	-2.927113674	0.025745469	1.576691939	0.218068815	1.357463601	0.680482607	1.051759235	NA	5.534306104	0.085980528
AT3G53350	RIP3	-0.699140968	0.349022249	-2.952437095	0.005242657	-0.777267247	0.426502078	-0.364883891	0.905793887	-0.092145925	0.913724709	-0.365428688	0.166616257
AT3G12040	AT3G12040	-0.72419138	0.469902368	-3.001452537	0.038838758	-0.480517406	0.231577522	-0.350959521	0.822720536	-0.539720652	0.533170432	-0.532062104	0.026891359
AT2G24700	AT2G24700	-0.416331617	0.767495199	-3.031211718	0.001522775	-0.94848528	0.243454488	0.346880636	0.841059666	0.568554055	0.323767852	0.919312803	1.02377E-07
AT3G23740	AT3G23740	-1.026941525	0.409098872	-3.036636446	0.018967036	-2.976649325	0.168546233	0.06678026	0.982749202	0.049456008	0.961441656	-0.166964478	0.733996267
AT1G05420	OFP12	-2.542894605	0.125642419	-3.083211662	0.036842046	-1.834900312	0.531789465	-0.078242543	0.987864525	-0.098367598	0.964742491	0.540687928	0.370593839
AT2G42800	RLP29	1.246270798	0.578170651	-3.104619635	0.021061187	-1.996504256	0.162998356	-0.390407903	0.967291937	-0.383226231	0.484106076	0.375375289	0.091078224
AT5G03870	AT5G03870	-0.253508675	0.915435765	-3.125003167	0.024955394	-0.220843189	0.859425143	-0.110189091	0.97809523	0.578548587	0.391551683	-0.152733774	0.674621968
AT5G38970	BR6OX1	-1.070978421	0.437154976	-3.131152023	0.006458813	-0.87075879	0.43915465	-0.234073027	0.959104829	-0.107825874	0.883566526	0.528997763	0.044917595
AT1G50910	AT1G50910	-1.029261091	0.455277439	-3.162748611	0.025403522	0.529122942	0.484385147	0.390726912	0.862037276	-0.6512904	0.584921772	0.123952451	0.774181406
AT4G00480	ATMYC1	-1.35623439	0.144062736	-3.224066464	7.45554E-06	-0.700767611	0.213569153	-0.004264042	0.9987964	0.280909548	0.640493482	0.961519597	1.16765E-08
AT2G17620	CYCB2;1	-0.529910556	0.805235353	-3.227505031	0.020688063	-1.49985246	0.238111393	-0.161053126	0.982529985	0.161971485	0.90184129	0.522261016	0.307008243
AT3G53650	AT3G53650	-0.901445762	0.664875834	-3.263019079	0.016562856	5.413759914	0.304852306	-0.101518257	0.989796361	0.258942747	0.820525033	3.515238442	0.569398004
AT4G31020	AT4G31020	-0.050188147	0.968254558	-3.33975378	0.017200806	-0.849194335	0.171834173	-0.342795024	0.895234351	-0.226215615	0.883566526	0.887720603	0.00542909
AT4G04460	AT4G04460	-0.833570403	0.661040932	-3.40767976	0.019606877	-2.011248211	0.235781096	-2.011795405	0.62738272	-0.252070635	0.88237076	-0.281314482	0.609217851
AT3G06030	NP3	-0.985918623	0.323552135	-3.411341306	0.000958827	-1.199729221	0.056188647	-0.235080843	0.951514049	0.335720114	0.552737263	0.481713966	0.11692334
AT1G09450	Haspin	-1.710572566	0.266692637	-3.413454566	0.004842406	-1.227963543	0.18699358	-0.197061613	0.961757542	0.033497866	0.978281903	0.974820121	9.76126E-06
AT1G03180	AT1G03180	-0.32455907	0.843017214	-3.429375804	0.008014054	-0.791257511	0.418038476	0.244369002	0.914259529	0.433411542	0.664015062	0.057900346	0.891024724
AT5G05400	AT5G05400	0.76112421	0.529666067	-3.468803181	0.041214762			-0.247319042	0.953923605	-1.204596428	0.145101222		
AT1G15760	AT1G15760	-0.070545729	0.983922132	-3.520296881	0.01375014	-0.398611875	0.767961324	-0.682465938	0.902565251	-0.316632743	0.768565149	-0.212757813	0.632156835
AT5G28610	AT5G28610	1.028742193	0.257815951	-3.524214509	0.008628858	-2.316918676	0.161350996	-1.264828215	0.754981183	-0.46795613	0.846389857	-0.303042951	0.748476765
AT3G04290	LTL1	-0.899605851	NA	-3.704699218	1.36325E-15	-0.969888136	0.056570921	-0.235935001	0.932418897	-0.168336012	0.711567545	0.644713657	0.000657662
AT1G01370	HTR12	-0.761323446	0.599049983	-3.709283495	0.002775993	-0.46158115	0.465755566	0.16360682	0.950356778	0.201206248	0.882725602	0.30432383	0.248250008
AT3G08770	LTP6	1.088853401	0.644914576	-3.710340569	2.54484E-09	-0.409981045	0.511669312	-0.092793825	0.982749202	0.222362531	0.591845798	0.309535418	0.063791502
AT3G12870	AT3G12870	-0.844000596	0.484122418	-3.742765438	0.00259051	-0.722200811	0.689402633	0.084413893	0.977840464	0.15432077	0.8800772	0.950585571	1.65885E-06
AT4G12917	AT4G12917	-1.915159129	0.327479051	-3.786781074	0.008171876	-4.1244848	0.14850782	4.875475042	0.672844426	4.120646097	NA	1.452406385	NA
AT4G09060	AT4G09060	-1.978563037	0.296966124	-3.922519201	0.026154006	-1.350581079	0.147471851	0.120808629	0.965471639	1.001810445	0.123393594	0.500400966	0.079292548
AT1G12380	AT1G12380	-0.682524236	0.589210178	-3.935184176	0.025052427	-0.476015775	0.711930835	-0.106334969	0.981591431	-0.369301279	0.5279315	0.664467874	0.000596074
AT1G55010	PDF1.5	-0.169936873	0.92740127	-3.964104877	0.001262644	-0.813795374	0.752748347	0.535698359	0.899519049	1.181701436	0.069050979	0.837916953	0.09365144
AT5G60720	AT5G60720	-1.671353781	0.377512014	-3.966873979	0.040033272	-1.403342378	0.392044903	-0.151373035	0.966021495	-0.192966269	0.920744976	-0.235572435	0.776213699
AT4G35620	CYCB2;2	-1.275088315	0.519496904	-4.028762033	0.000354623	-1.867339544	0.157577225	-0.182234166	0.964000833	0.325604547	0.605561645	0.705306223	0.005444933
AT3G51280	AT3G51280	-0.572852746	0.69982613	-4.073789192	3.08007E-11	-3.364861002	0.098879879	-0.170260205	0.966957004	0.45692819	0.469711207	0.609846564	0.000555303
AT3G05480	RAD9	-1.830400197	0.170728425	-4.086354589	0.024904601	-0.712694938	0.304852306	0.04232576	0.990915497	0.106167466	0.934609223	0.568692043	0.104345129
AT5G55680	AT5G55680	-0.767925784	0.691379374	-4.114784972	0.030310411	-1.01328584	0.671977282	0.015885443	0.996799452	-0.037953816	0.989242512	0.849637346	0.08897623
AT4G13690	AT4G13690	-2.240571157	0.622849484	-4.169747747	0.032898181	-1.485898979	0.440215277	-2.204916572	0.618005886	0.065842942	0.982621649	0.07853581	0.886045969
AT5G02515	None	-2.136116129	0.301385348	-4.202796035	0.032886439			2.100496669	0.637124836	2.331093274	0.187081257		
AT4G24265	AT4G24265	-0.650338557	0.858645958	-4.347938976	0.017723215	0.295161597	0.837172929	-0.100973107	0.989481773	-0.79264213	0.176386585	0.397315509	0.315052253
AT1G20060	AT1G20060	-1.685017366	0.20060204	-4.394482534	1.22808E-10	-0.97323039	0.337914957	-0.233673867	0.883972835	0.356731313	0.814963929	0.386045693	0.203026116
AT1G12550	HPR3	-2.189813102	0.406553351	-4.413335598	0.022374548	-2.396989536	0.061390904	0.399284829	0.878942115	0.083730281	0.972203917	0.504859973	0.521136361
AT5G52020	AT5G52020	-0.486769241	0.681246618	-4.500842148	0.01450448	-0.379133451	0.866296846	-2.207921421	0.62738272	-2.011931697	0.393297004	1.292811156	0.084348139
AT1G70985	AT1G70985	-3.099073024	0.062390944	-4.555360219	0.009888044	-1.286490466	0.430687744	-5.446415072	0.084005684	-0.799795694	0.954183802	-0.17048311	0.876499993
AT2G01900	AT2G01900	2.403860161	0.206810051	-4.566592061	0.046554857	-0.074482923	NA	-0.564329565	0.928771701	-0.804823496	0.243461821	-0.063073169	0.898187154
AT3G13680	AT3G13680	-0.185827944	0.936198049	-4.698887917	0.033957122	-0.540411264	0.853331771	-0.136500119	0.993804788	-3.031200657	NA	-0.461638226	0.830357013
AT4G26170	AT4G26170	2.082743863	0.506656426	-4.81245768	0.034132381	-0.70125875	0.580551893	0.157303895	NA	0.914891325	0.704428764	0.228567579	0.723343016
AT5G06790	AT5G06790	-0.299628647	0.937036926	-4.865596087	0.016893359	-1.543997789	0.16545761	-0.366702878	0.963252175	-0.876266055	0.525572109	0.217755464	0.691548955
AT2G35350	PLL1	0.212883391	0.861603351	-4.869951022	0.000871698	-1.760370674	0.229145213	-0.639145393	0.809502311	-0.710291272	0.064687133	-0.850373764	1.43333E-05
AT2G30820	AT2G30820	-2.006117192	0.437183069	-5.143448969	0.002559234	-1.821619335	0.417737103	-0.916819009	0.851805999	0.918289672	0.35863867	0.932660572	0.030442469
AT5G01910	AT5G01910	-0.836455609	0.721565878	-5.152656124	0.000624854	-0.902153983	0.276884437	0.227613179	0.936143398	0.785905064	0.279469979	0.616639455	0.010656286
AT5G65690	PCK2	3.866681934	0.126562262	-5.290935907	0.022875443	0.261419096	0.777652816	-2.509088423	0.665832077	1.351027969	0.686407369	-0.567216307	0.461950754
AT4G22517	AT4G22517	-1.757886866	NA	-5.426428615	2.35642E-35			-0.26924848	0.938833609	0.598037954	0.638629847		

AT1G67035	AT1G67035	-0.558112681	0.892900931	-5.591881823	0.018361943	-1.744290162	0.350257105	0.588224922	0.86582927	0.602616282	0.732399618	0.151207638	0.770384878
AT2G34960	CAT5	-1.465013678	0.773796032	-6.036955269	0.007234	-0.336499995	0.916464536	-6.438582389	0.599175866	-1.450857721	0.525384544	0.088349046	0.97989514
AT4G39790	AT4G39790	-1.029822757	0.432346441	-6.037043122	4.92639E-05	-0.990380211	0.628939732	-0.317952667	0.894017469	-0.129547519	0.936190346	0.260499998	0.589223809
AT1G56080	AT1G56080	0.758390487	0.626980076	-6.140001623	0.001489642	0.331438992	0.743246346	-0.34879197	0.959703883	-0.499737808	0.554239483	-0.121289295	0.761927143
AT4G26330	UNE17	0.870982364	0.892677274	-6.54614634	0.049628909	0.348925255	0.908984144	-1.437211612	0.856770017	-0.576556269	0.829854959	1.456282532	0.09606607
AT4G02170	AT4G02170	-2.233894971	0.37385721	-6.593288553	0.043289518	0.351968331	0.897309309	-6.704095634	0.264075534	0.77200021	0.947746874	2.147820066	0.293928598
AT3G48290	CYP71A24			-6.64780763	0.047451884			7.023055222	0.562387325				
AT4G01230	AT4G01230	-1.995906293	0.656106765	-6.663403479	0.038294816	-3.751881733	0.072985878	-6.615116598	0.57062251	0.333048451	0.963495178	1.115260171	0.521273404
AT1G56165	AT1G56165	-1.072342998	0.496819076	-6.675724989	0.000230738	-1.023227986	0.532293613	2.177375153	0.672844426	-0.312872772	0.919509807	0.178924178	0.832565351
AT3G26235	AT3G26235			-6.699481681	0.034285866								
AT1G71420	AT1G71420	0.640142856	0.645190287	-6.705675616	0.005678137	-2.167539598	0.104103388	-0.36355202	0.960527983	0.027832912	0.992519652	0.174720641	0.814315114
AT5G01175	AT5G01175	-1.961084117	0.573001344	-6.737542603	0.002600404	0.21379288	0.928925941	1.284944798	0.613859108	0.146768063	0.942981401	0.220274713	0.79941631
AT3G07260	AT3G07260	-6.558951781	NA	-6.7428997	0.029440269	0.281136122	0.563907217	-1.402677941	0.924803201	0.973655839	0.80706189	0.4389804	0.252690418
AT1G10790	AT1G10790	-0.763202372	0.918241212	-6.765843783	0.040509455	-5.213947094	0.219203647	1.413761954	0.832523868	-1.339165461	0.661503366	0.054463179	0.969443468
AT3G30725	GDU6			-6.788655833	0.039704292	4.734883457	0.379465519	-2.311776307	0.847546649	-0.841048915	0.914359122	-3.213669712	0.076098445
AT3G16712	AT3G16712	-3.191517075	0.09022378	-6.802564778	0.02496612	-4.05636002	0.17152898	-1.691974153	0.807242331	-0.513686284	0.879822636	-0.363000919	0.893228413
AT3G01015	AT3G01015	-3.585929062	0.433393484	-6.802607454	0.025370474	-2.842928772	0.314940826	0.678023477	0.968021479	0.470963265	0.941995647	1.123674115	0.459878389
AT5G37240	AT5G37240	-0.408496009	0.930035011	-6.807333753	0.026065948	5.888949012	0.238041347	0.467722679	0.968021479	1.924809929	0.70395135	1.250699175	0.492070377
AT1G63330	AT1G63330	-3.410173484	0.466864275	-6.814480828	0.036865295	-2.106480472	0.17820439	-5.505833429	0.577728436	1.410589299	0.900466581	-0.034446742	0.974488158
AT5G03795	AT5G03795	0.682719067	0.878556955	-6.834811943	0.046530787	2.357065668	0.252843784	-0.34580997	0.965260868	0.1245484	0.964223704	0.119525708	0.888538076
AT4G09405	None	-4.067388467	0.124831385	-6.842223487	0.025403522			-0.587971516	0.966980964	0.39220997	0.949384996		
AT5G49320	AT5G49320	1.005861441	0.834725161	-6.883349553	0.021223216	-0.632440699	0.779991752	0.043204741	0.994162268	-0.060800069	0.986558834	-0.403223792	0.656365922
AT4G35783	RTFL6	-1.800852081	0.398318158	-6.891587614	0.021850527	-1.096423994	0.225941574	-0.198210155	0.990924496	4.496767717	NA	-1.435054802	0.498505889
AT3G29173	None	0.254510058	0.96184164	-6.925693634	0.021586618			0.217426241	0.968021479	-0.079236026	0.987095576		
AT4G24600	AT4G24600	4.344190331	0.464185749	-6.945050522	0.03774582	-0.070348901	0.977183027	6.221665914	0.6107328	0.785363156	0.932254226	1.582819954	0.3765072
AT2G42885	AT2G42885	0.190761995	0.980195352	-6.954233672	0.024692672	0.161463363	0.969945712	2.278603947	0.864295062	4.122980551	0.06386383	0.128936241	0.891573213
AT2G14070	AT2G14070	-4.55852849	NA	-6.955310282	0.028249705	-0.424050718	0.657915915	6.186805615	0.611547142			0.189040922	0.969252469
AT4G13410	ATCSLA15	-6.265756926	0.237926525	-6.961797177	0.018783447	-2.885307344	0.515464307			3.166301284	NA	3.716597353	0.540059413
AT5G18710	AT5G18710	-0.747454144	0.863389629	-6.964276868	0.018726668	0.386048565	0.873937964	-0.689007261	0.918988754	0.302829517	0.896356173	-0.199583199	0.83571117
AT5G66740	AT5G66740	-1.493880642	0.629008633	-6.982355846	0.015501048	-4.52140956	0.403165211	2.068698012	0.828875452	-0.075816562	0.992180597	0.01774935	0.988467984
AT2G24610	CNGC14	2.16808748	NA	-7.014477483	0.001762258	-4.806752021	0.371732733	-0.562828463	0.92868457	-0.281299579	0.839885043	-0.892399943	0.597282028
AT5G55690	AT5G55690	0.918841463	0.90147635	-7.018584159	0.013618979	-0.322144409	0.944050677					1.137840493	0.801556905
AT3G06100	NIP7;1	-2.271626444	0.664977268	-7.035927746	0.013172807	-2.975229313	0.158475983	-7.540978434	0.336816969	0.132358629	0.97926944	0.357095392	0.807880563
AT5G51530	AT5G51530	-0.50407562	0.948196028	-7.036781507	0.025471216	-0.878045164	0.622748297	-5.181483417	0.657950393	-0.149522172	0.98143759	0.236485352	0.851308286
AT1G27045	ATHB54	-7.22273215	0.067449527	-7.06218572	0.01228848	-0.493057881	0.86502221	-0.285769	0.98493843	2.537735709	0.666064463	1.740926719	0.451772006
AT5G01150	AT5G01150			-7.071630119	0.017056277			-3.19193034	NA	0.396467846	0.956955597		
AT5G32460	AT5G32460	-3.072144401	0.272193408	-7.073501603	0.020168209	-0.630860329	0.016314213	-8.690796199	0.099695852			0.031563622	0.926686462
AT3G42725	AT3G42725	-2.271354161	0.438845556	-7.108574832	0.011210709	-0.937029631	0.682757756	0.413317367	0.954839264	0.21612997	0.885377618	0.047139107	0.950606257
AT3G13900	AT3G13900	3.462250402	0.362203681	-7.113493677	0.010033428	-5.831339581	0.082759653	-1.149202014	0.824296871	-2.377975228	0.342229601	-0.112020451	0.951866213
AT5G57735	TASIR-ARF	-0.77720747	0.812401376	-7.1191755	0.015935313	-2.14965222	0.583129725	0.363735937	0.968021479	0.464180218	0.865593881	-0.489950147	0.69708539
AT1G04290	AT1G04290	-1.342459238	0.598198344	-7.130626708	0.012672465	-1.950045967	0.683281404	1.746853154	0.871697206	-1.004727095	0.810706099	-0.665487588	0.846279716
AT4G00416	MBD3	-1.115317692	0.774304933	-7.143115773	0.026990466			-6.693833026	0.584586994	1.070311781	0.758194126	1.707035301	0.799688042
AT1G05035	None	-5.361300959	0.346632414	-7.162875962	0.008808988			-1.695086337	NA				
AT2G18450	SDH1-2	0.958903527	0.856739377	-7.174012673	0.013337721	-0.700514176	0.869611969	0.231339692	0.990915497	-0.386632407	0.922334223	-2.660853001	0.184203944
AT3G50230	AT3G50230	-1.343904116	0.732484338	-7.177027385	0.014131833	0.815496959	NA	-1.908445327	0.785453357	-0.97366724	0.79710327	0.234996788	0.781967853
AT5G02350	AT5G02350	0.393883754	0.952422369	-7.193736678	0.010773967			-0.283529015	0.969967158	0.057418584	0.983138034	-1.089600711	0.456864698
AT3G46890	AT3G46890	-3.204052473	NA	-7.237355626	0.007626122	-6.172001991	0.102531637	-0.341390666	0.977273207	-0.775819589	0.882739754	-0.285476795	0.814315114
AT5G36740	AT5G36670	-4.412805514	NA	-7.256445316	0.00926717	-2.675722486	0.590678712	2.006952839	0.782971455	-0.085881043	0.988264628	-0.77605987	0.651891619
AT3G05980	AT3G05980	1.088526648	0.557696794	-7.257050466	0.009611997	-0.990844541	0.826497675	-0.227937865	0.942731421	0.341946716	0.895021414	0.330367489	0.500430311
AT2G02750	AT2G02750	1.613029356	NA	-7.269060019	0.008009808	-0.050228672	0.972762045	1.346449925	0.791199667	0.814809998	0.766614324	0.073925365	0.900462475
AT2G13430	AT2G13430	6.699518452	0.184045935	-7.279594727	0.009456291	4.516043111	0.270080962					1.409877598	0.834601312
AT4G09430	AT4G09430	-1.6046309	0.816122178	-7.305400859	0.006070575	-5.516288817	0.234114742			0.211345104	0.989211341	-0.816024631	0.845269305
AT2G04005	None	3.941445652	NA	-7.327143455	0.006754857			-5.776362574	0.629761053				
AT1G78030	AT1G78030	-1.744339865	0.794952744	-7.33159165	0.009645304	0.400051374	0.939783531	-6.423263135	0.599339861	-1.254142492	0.783448836	-0.721735448	0.920917425
AT5G03090	AT5G03090	0.241641147	0.946951678	-7.332032732	0.005307406	-1.094950108	0.767091863	-1.044621442	0.859699136	-1.65988017	0.687436532	-2.31131295	0.101405976
AT2G30770	CYP71A13	-4.709992537	NA	-7.341855216	0.042444456	-1.092246488	0.560187995	7.756559432	0.314019385			-4.848107488	0.137857155
AT4G11400	AT4G11400	-0.255741731	0.956275432	-7.371489242	0.004137602	-2.346210214	0.424677866	-0.44371985	0.96262003	-0.414813977	0.860914379	-0.237789465	0.733871724
AT1G51055	AT1G51055	-1.970861831	0.659654569	-7.383957306	0.003916014	-5.885962519	0.090003832	-0.824903519	0.897050498	-1.663733083	0.709696358	1.099335124	0.329458485
AT5G45113	AT5G45113	-1.102824359	0.690526684	-7.387606197	0.005350872	0.155430827	0.883075636	-6.265013704	0.607117655	-0.07956743	0.996204637	-0.052873395	0.93288314
AT1G67370	ASY1	2.186356923	0.739098278	-7.42449966	0.003966779	1.121585197	0.635311358	-0.580962463	0.928343554	0.532418997	0.795808621	0.125671438	0.884962273
AT4G22900	AT4G22900	0.682172237	0.857784081	-7.438480938	0.036149169	0.53463654	0.780230058	-1.560989989	0.712887303	0.318827809	0.838286194	-0.96084327	0.027787968
AT1G55580	LAS	1.6393041	0.484837442	-7.465117046	0.033253956	-2.195103884	0.223050712	-1.34491136	0.732202251	-0.769526316	0.519043739	-0.912345395	0.024373144
AT2G10930	AT2G10930	-0.578311888	0.860578205	-7.471765029	0.003058978			1.093399756	0.864912011	0.185722788	0.940841103	-0.465953924	0.786620649
AT4G06080	None	0.828143693	0.907865147	-7.476480356	0.03296312			5.628310675	0.637614678	-0.251450784	0.971751472		
AT5G45240	AT5G45240	-2.496792525	0.378814527	-7.51662162	0.005082702			-0.049702677	0.995271955	0.478673681	0.847211544		
AT3G28335	None	-2.333854568	0.541416863	-7.534814935	0.002615804			1.298428198	0.918652486	-1.144539023	0.842628389		
AT5G35940	AT5G35940	-6.656686192	0.154869498	-7.568398802	0.002102641	-5.008976503	0.349775578	1.258875362	0.876428092	-0.38235681	0.915888621	-0.960215898	0.476315429
AT2G23190	CYP81D7	2.547006514	0.368359037	-7.570363429	0.027340599	4.451796127	0.379037885	-5.29019228	0.652047062			4.469302592	0.089369998
AT3G22540	AT3G22540	-0.493059114	0.920997827	-7.578678347	0.02663753	-5.653662828	0.129003423	-5.541484729	0.153148192	1.172842516	0.728724954	-0.777600569	0.465180723
AT2G44745	WRKY12	0.215167124	0.966480307	-7.586405325	0.002094306	1.122902688	0.306985228	0.566357061	0.821892411	0.287032164	0.917874146	0.353819689	0.332758783
AT5G56490	GulLO4	-4.051127154	NA	-7.589486298	0.002379203	0.286575748	0.918539688	1.015890804	0.912756407	-0.232784807	0.935637155	0.996302559	0.053641575
AT4G04710	CPK22	-6.728698262	0.141538766	-7.599431625	0.002334444	-1.031127863	0.740666832	-0.664854523	0.951554105	-0.94164013	0.498246857	0.113943143	0.916113226
AT3G48280	CYP71A25	-3.06211822	0.44555215	-7.604677505	0.002629963	0.162440369	0.898947545	-7.558928425	0.343493265			-0.680488787	0.661891749
AT3G42850	AT3G42850	-0.070592039	0.984849538	-7.611116975	0.002036738	6.037041962	0.207267168	2.257684053	0.62738272	1.113626243	0.703026572	2.354926266	0.221463422
AT1G07743	None	-0.84585365	0.838185656	-7.612141829	0.001818054			-0.503512553	0.969967158	-0.845525146	0.708283869		
AT4G36105	AT4G36105	-0.489774802	0.942117085	-7.652976857	0.001342175	-3.749463122	0.079004662	2.896874643	0.685495817	2.164772901	0.59029983	0.541227624	0.590574863
AT3G10290	AT3G10290	-4.057128325	0.070966773	-7.670598267	0.002734019	-1.63874893	0.631145143	0.562252716	0.92907878	0.858657963	0.299502215	0.226225028	0.689117509
AT4G04835	None	-1.774623686	0.601899706	-7.671120409	0.001127964			7.578367568	0.386146892	-4.322500116	NA		
AT1G04778	AT1G04778	-2.03412145	0.367636187	-7.706480285	0.00154575	-3.844064758	0.165847792	-1.338210468	0.81577697	0.895679099	0.491449838	-0.245489511	0.679259032
AT3G18590	ENODL5	0.897130383	0.903259609	-7.716339513	0.019817217					-0.431773511	0.976655483	-1.377867238	NA
AT1G52070	AT1G52070	5.862887199	0.299731107	-7.719001739	0.026368892	-3.585343201	0.516230268	-4.027181553	0.100032418	-2.699981007	0.646238543	-3.312927691	0.392167445
AT3G53450	LOG4	0.114214716	0.953708041	-7.744953382	0.000844763	-2.678496777	0.172594578	-1.375269963	0.796134167	0.504511136	0.820349765	-0.358587356	0.620067306
AT5G64667	IDL2	2.227112189	0.375428573	-7.75328905	0.000763364	-3.692546312	0.120395512	-0.364439541	0.963509602	-0.730499411	0.795788733	0.204240864	0.832405719
AT1G07373	None	-2.094018257	0.651008211	-7.804715495	0.00067588			-1.013930117	0.913085739	-1.748008532	0.550428211		
AT1G04880	AT1G04880	-0.677165297	0.923101938	-7.819559208	0.017806458	-1.372228087	0.536507552	1.247115913	0.804372175	-0.354711409	0.95452954	0.942121765	0.262287446
AT1G06420	AT1G06420	0.746436037	0.766387857	-7.821460116	1.10726E-05	-0.562091092	0.775106327	0.060605364	0.993737713	1.071083121	0.074494646	0.319042319	0.544288511
AT5G44620	CYP706A3	0.772018011	0.785794604	-7.84159511	0.000568537	0.02305909	0.988565389	0.487896686	0.946164167	0.506557608	0.740183289	0.489531122	0.057466177
AT3G47100	AT3G47100	-3.919421169	0.396234389	-7.874711781	0.000651697	0.352150297	0.915828386	-2.175657808	0.83240074	0.02851164	0.996340093	-0.638087248	0.500058566
AT3G26480	AT3G26480	-0.973595635	0.720197626	-7.880747783	0.000906616	0.73131344	0.89288352	0.827170691	0.883939861	-0.06596549	0.982995784	-1.72308644	0.514844223
AT5G37950	AT5G37950	3.785937349	0.107358373	-7.917220647	0.000545758	-5.774085111	0.177928023	-0.4611161	0.962474985	0.53777402	0.325804572	-0.270232115	0.490431889
AT1G05065	CLE20	-1.916699176	0.398230994	-7.945774294	0.000370764	-4.222668095	0.118829551	0.054787097	0.995045161	0.911573224	0.65361065	1.172742539	0.232482753
AT4G25160	AT4G25160	-2.157429265	0.517104316	-7.953835181	0.000311674	-1.197293095	0.372526558	-1.103366181	0.647947756	0.010744256	0.996289463	0.555229113	0.144531699
AT1G02070	AT1G02070	1.817150214	0.352056057	-7.958117962	0.000284211	-5.751279157	0.094802478	-0.477953741	0.945609936	-0.489138207	0.812750957	-0.230049703	0.844098547
AT1G09863	None	1.128165573	0.678117626	-7.973906344	0.000512182			-1.350953009	0.739104225	0.589110361	0.633148104		

AT2G35658	AT2G35658	-2.342565248	0.249673509	-8.004118122	0.002124338	-1.174775806	0.215426057	0.928848016	0.960961456	-0.398244828	0.943072833	0.999111056	0.106394804
AT5G52940	AT5G52940	-3.917864862	0.102646864	-8.029854639	0.00096948			0.649682909	0.969967158	2.563718308	0.673329207		
AT3G62960	AT3G62960	-5.917553352	0.294914377	-8.057170476	0.000263566	-3.322614377	0.550728949	5.573977872	0.637815859	6.198443561	0.355438697	1.977962436	0.620946901
AT3G26320	CYP71B36	-0.750921205	0.808481919	-8.061997787	0.001751289	-0.593565745	0.723512932	3.118015705	0.790596125	-0.733635546	0.906127081	-0.910416324	0.768555735
AT4G28560	RIC7	0.444060992	0.890227207	-8.079070546	0.000152484	0.615385176	0.786808669	0.304690111	0.918165358	-0.696320307	0.55798645	-0.647609899	0.276253553
AT1G53860	AT1G53860	-0.365955596	0.818565954	-8.106726759	0.000114343	-1.537964187	0.505892975	-1.154304818	0.542342434	0.808166474	0.358925545	-0.360112233	0.300649269
AT2G37560	ORC2	-1.075846057	0.445244033	-8.167225564	0.000163868	-1.69034736	0.107983697	-1.540927527	0.712887303	-0.053327374	0.98143759	-0.065608417	0.881908975
AT3G27730	RCK	-1.436286182	0.569280857	-8.172717522	0.000133893	-0.590092894	0.883288718	-0.810759579	0.884881786	0.520065904	0.802042097	0.875925877	0.074916922
AT1G47130	None	1.261485056	0.409098872	-8.189406115	0.000128706			-0.319764545	0.978041029	0.365980993	0.958669515		
AT3G57200	AT3G57200	-0.397955571	0.941244506	-8.258401697	0.000100877	-2.925496368	0.207861316	-0.163300478	0.982814189	0.306626754	0.854600248	-0.042232625	0.949893046
AT5G52290	SHOC1	-1.837051028	0.545760054	-8.327356518	7.75679E-05	0.300322872	0.899728701	-0.674293768	0.924925439	-0.264486639	0.957156296	0.439058036	0.683373259
AT3G11640	AT3G11640	0.037763083	0.993246068	-8.356861764	0.00012234	-0.678140526	0.687374149	1.276025943	0.878014682	-0.262627191	0.951111828	0.183586759	0.85083712
AT3G14540	AT3G14540	-1.715496342	0.445018984	-8.397235883	2.33649E-05	-0.217292918	0.957327805	0.455637442	0.950356778	0.115715376	0.983138034	0.250170782	0.893400872
AT3G60900	FLA10	-2.187004345	0.266224717	-8.414520347	3.40242E-05	-2.639138771	0.202076465	0.629177962	0.832843792	-0.036882405	0.988021272	0.712251801	0.040669586
AT2G18010	AT2G18010	-1.718216635	0.498861127	-8.460112386	1.37978E-05	-2.125543844	0.228848622	-0.092180086	0.990915497	-0.176771714	0.900609444	0.089333168	0.855158201
AT4G28430	AT4G28430	-0.684732343	0.802159631	-8.474723928	1.4321E-05	-2.305850085	0.282368111	0.336611958	0.963252175	0.396090631	0.713346329	0.703729279	0.018841369
AT1G14250	AT1G14250	-2.981223343	NA	-8.496689046	0.000153837	-5.96629567	0.064214942						
AT2G24680	AT2G24680	-1.251296813	0.725317004	-8.510377651	0.000270282	1.107622711	0.852836161	0.612926999	0.759702709	-0.177570975	0.923631194	0.829975025	0.48338674
AT2G18470	PERK4	-2.570104935	0.440548701	-8.529172147	0.000138491	-3.303153727	0.064716094	-2.940187746	0.413284704	-1.992326575	0.47020656	-0.643658662	0.469355591
AT5G54240	AT5G54240	-0.623247965	0.794952744	-8.616121822	1.77379E-05	-1.214708411	0.218988257	-0.589070227	0.655815891	-0.429244371	0.570854222	-0.652656055	0.128980557
AT2G34830	WRKY35	-1.667508389	0.648352019	-8.663184216	6.42896E-06	-0.779019107	0.718533694	-2.740283036	0.655815891	-1.698880924	0.265599244	-0.768750235	0.198283121
AT3G50120	AT3G50120	-2.62975095	0.388023642	-8.700472853	3.02603E-06	-2.113126575	0.364280638	-0.434041047	0.950356778	-1.258277889	0.067570199	-0.752489547	0.154704802
AT4G37685	AT4G37685	-0.234487685	0.924644156	-8.704529054	2.79617E-06	-0.181886245	0.901927967	-1.655522569	0.638079998	0.375298002	0.857138563	0.446713619	0.456630744
AT5G36790	AT5G36790	-3.861160133	0.057487883	-8.858767125	8.44275E-07			-5.915036667	0.626508471	5.104129189	0.53583656		
AT2G22140	EME1B	-1.621404825	0.306416998	-9.084434289	1.13524E-07	-0.15117371	0.893304021	-1.702253431	0.66808962	-0.570037259	0.684124932	0.180429125	0.758942832
AT4G35520	MLH3	-2.50228387	0.182567501	-9.09659274	3.49691E-07	-0.907979969	0.417637428	-0.075861337	0.991271129	-0.64677497	0.728206184	-0.02871625	0.964563269
AT4G39630	AT4G39630	-1.724589863	0.138292337	-9.099053079	1.89894E-07	-0.829659436	0.350309397	-0.028380144	0.992570276	0.455471246	0.531929726	0.791635284	0.003754206
AT5G07760	AT5G07760	-1.484010097	0.673985047	-9.165874763	6.57346E-08	-1.280704193	0.309415574	-0.34471379	0.971933573	-0.546262762	0.714634819	0.546926262	0.242717053
AT4G15320	CSLB06	-2.960024787	0.25423539	-9.38772968	1.26133E-06	-2.58669937	NA	0.31858608	0.968467209	2.712984519	0.130831592	0.720649128	0.549576525
AT2G38620	CDKB1;2	-0.292056396	NA	-9.425771817	1.19024E-08	-0.801778158	0.35179812	-0.323674593	0.963772337	-0.02555084	0.988021272	0.597068046	0.05205206
AT4G34360	AT4G34360	-1.427262794	0.133777632	-9.529167198	3.03223E-09	-1.203300845	0.436351654	-0.217356054	0.911720308	0.587092507	0.464679378	0.469820253	0.157992811
AT5G38930	AT5G38930	-1.108754046	0.523997735	-10.13855431	1.88173E-11	-0.745270583	0.808021058	-0.893954469	0.854333184	1.699428775	0.222566815	0.902827338	0.004286711
AT1G53830	PME2	-2.60541932	0.219101478	-0.977945919	0.735864838	-3.26926545	0.000637543	-3.504122054	6.19248E-06	-1.397914193	0.016481523	-1.13543465	0.004557658
AT3G07720	AT3G07720	0.282829884	0.55743614	0.280129464	0.574520031	-1.127059231	0.011384468	-1.191657929	0.025537001	-1.151116937	3.81206E-06	-1.244671175	0.07979398
AT1G72645	AT1G72645	0.255749292	0.749825639	-0.184722824	0.77014218	1.174355776	3.36745E-16	3.396180149	0.004235696	1.024769907	0.233539745	1.46940301	1.272E-05
AT1G66570	SUC7	5.815631709	0.304384198			3.760532851	3.08733E-17	-11.05667368	7.55974E-09			-1.435268236	3.17761E-07
AT5G05530	AT5G05530	2.199159085	0.454444822	2.00226276	0.089057893	1.945642097	0.020514413	-6.585861412	0.00495004	-0.137047536	0.976552764	-1.206354126	0.000912468
AT4G25470	CBF2	0.363559213	0.530892359	-0.187738156	0.29203733	-1.001600726	1.3119E-10	2.695768905	0.040105261	-0.234430771	0.736167095	3.239687899	2.1837E-113
AT1G15125	AT1G15125	-1.104149682	0.095350976	-0.873549986	0.040660598	-1.953563266	0.000123211	2.412038581	0.002034504	-0.817719456	0.658178574	1.274770964	0.000376733
AT3G12830	AT3G12830	-0.330515906	0.527793248	-0.977347715	0.172171123	-1.002255126	0.004054531	2.304193734	0.013621408	0.010141574	0.993725249	2.33493802	2.86066E-26
AT3G56710	SIB1	-0.478983461	0.577626207	-0.852515661	0.289402163	-1.248074583	0.002237839	1.175847283	0.034661846	-0.339394934	0.823666413	3.764228922	4.93004E-41
AT5G24030	SLAH3	-0.83892796	0.051445593	-0.180555732	0.749542511	-1.09801479	0.022725388	-1.47357234	0.016216352	-0.823471224	0.000752018	-1.278698055	3.6376E-06
AT5G59680	AT5G59680	-1.760926746	0.492404849	-2.250671595	0.450650554	-9.516370268	7.01333E-11	-5.751269016	0.010442407	-2.558026006	0.445236462	-4.00808416	2.42651E-16
AT5G36870	GSL09	-0.769544869	0.810161239	0.634810235	0.908904535	-6.834079122	0.005675515	-7.764678184	0.019206838	-2.556925696	0.211301091	-4.292719369	9.2131E-10
AT1G21890	UMAMIT19	1.07839494	0.866761102			-7.135175715	0.017135196	-8.522457309	0.001592467	1.969788129	0.578901234	-2.691943682	0.010919736
AT2G13360	AGT	0.411987601	0.461077937	0.557336534	0.00017667	1.125048637	1.69622E-22	3.302325441	0.032838202	0.167642607	0.612310929	-0.852296012	1.6431E-14
AT3G03480	CHAT	-4.887463845	NA	4.52332053	0.068084839	4.729481609	0.000130477	-7.732792331	0.040407114			-0.587319966	0.649881092
AT3G60950	AT3G60950	5.300659342	0.35264375	-3.761655451	NA	1.441508967	1.36519E-17	-9.228669196	4.95029E-09	-3.888938333	NA	0.455672472	0.007613466
AT3G61030	AT3G61030	5.819265876	0.304112084			1.356924494	6.91686E-14	-7.997205267	0.001231067	-4.054866351	NA	0.397854187	0.039300218
AT1G23130	AT1G23130	0.487798192	0.144861484	0.471691857	0.025495422	-1.854435264	2.03534E-41	3.786283636	1.19427E-05	2.03506438	0.070963193	-0.378230576	0.326214991
AT5G01060	BSK10	-4.709992537	NA			-6.806445073	0.034426292	-7.817048625	0.026029524	-1.62676333	0.576874111	-0.860368002	0.036074916
AT2G03020	AT2G03020	2.18669876	0.526931632	0.658367131	0.878862504	-4.751403814	1.66634E-07	-8.025938419	0.008978139	0.558870767	0.89791993	1.031792039	0.099958957
AT5G22460	AT5G22460	-1.445450054	0.736277684	0.498705599	0.771409648	2.830830171	0.00980582	-1.119441786	0.798808149	-1.150399432	0.004688335	1.57621882	1.98952E-13
AT3G12500	HCHIB	3.933738944	0.084782759	1.413599903	0.298758863	3.638475929	0.000710039	-1.306627647	0.536751295	-2.097656384	2.37319E-09	-1.68381823	3.24643E-13
AT4G16670	AT4G16670	1.314460975	0.687880262	2.273886474	0.322726384	1.722463806	3.34326E-10	-1.17286274	0.776796299	-1.187220268	0.019151736	-0.191177421	0.329458485
AT5G10880	AT5G10880	-5.970147766	0.289851886	-6.855294582	0.095994204	-6.505836819	0.021907154	-6.439238663	0.599399361	6.96257612	0.04012736	4.711212623	0.049436843
AT4G08300	UMAMIT17	0.400562088	0.707402458	-0.694143633	0.456607631	-6.418594059	2.11272E-06	-0.901443293	0.413866874	-1.092180035	0.002554745	-2.355093515	1.25978E-46
AT5G43180	AT5G43180	0.997219708	0.123822969	-0.592977066	0.795761936	-2.687559323	0.001865052	-2.252448904	NA	-1.118535316	0.046984347	-1.316439188	6.76367E-07
AT5G41900	AT5G41900	-1.233630328	0.361964149	-0.202034124	0.943338882	-3.749710193	0.000620269	-1.214248079	0.482795025	-1.491314944	0.005188687	-1.17287737	2.79295E-05
AT2G14100	CYP705A13	2.761960241	0.312918421	0.669905903	0.792297572	-7.892381352	6.64861E-05	-2.557964219	0.542342434	-2.371586273	0.03802904	-4.060304623	3.42715E-19
AT5G03270	LOG6	-1.514886722	0.755931633	-6.220946012	0.101035725	-6.229205656	0.036393786	-5.86052391	0.62738272	6.168236494	0.006836936	1.304488992	0.228163165
AT4G26830	AT4G26830	-1.74047057	0.486248826	-2.294036867	0.35259733	-8.609909624	1.62216E-07	-0.234475319	0.984064159	3.878474542	0.023746736	-1.003090581	0.470663197
AT4G36410	UBC17	-0.896194751	0.141689457	-0.979480905	0.033704102	-1.003517407	0.005580224	1.85551851	0.208579907	1.619255503	0.000487645	0.230719557	0.630552092
AT4G16120	COBL7	-0.797566371	0.237515389	-0.88527145	0.080204161	-1.379104887	0.014145379	-1.427977639	0.598901706	-1.013936594	0.001937821	-0.237241131	0.496468977
AT5G03260	LAC11	-1.685414694	0.065102014	-1.168201471	0.065632359	-1.295126628	0.048171417	-0.250740801	0.872669065	-1.015967015	0.001077297	-0.192886746	0.600457284
AT5G62220	GT18	-0.91031467	0.05810362	-0.351466988	0.593733305	-1.620482969	0.000214374	-1.893738508	0.561207188	-1.091412484	0.012438322	-0.477960834	0.037503571
AT1G64390	GH9C2	-0.326716867	0.614906517	-0.382109945	0.390031901	-1.205907076	0.000858848	-1.542955137	0.13090699	-1.091801658	1.25302E-20	-0.538288079	0.000302964
AT5G25930	AT5G25930	-0.981626946	0.035389177	0.006072	0.99438049	-1.655928497	0.002003886	-0.428783419	0.816446062	-1.192076096	0.001394431	-0.015588469	0.966991873
AT1G15550	GA3OX1	-1.089387128	0.626126798	-2.301248476	0.317985658	-1.929120261	0.035510263	-1.490489506	0.697407195	-1.229875896	0.00132665	-0.212077259	0.572142263
AT1G01900	SBTI1.1	-1.024711109	0.451610755	-0.450372466	NA	-1.733141438	0.011331921	-1.038721088	0.816446062	-1.300197274	0.043532871	-0.317638364	0.371291656
AT5G17420	IRX3	-1.132397883	0.250114502	-1.1875426	0.088188074	-1.453462334	0.019870456	-0.835707656	0.664635521	-1.439599245	1.49639E-05	-0.403670594	0.091565254
AT4G04745	AT4G04745	0.933372911	0.740501291	-0.040763008	0.994059452	-7.314129572	0.001465394	-0.985758055	0.865456099	-1.647281189	0.01991045	-0.510963113	0.308571886
AT5G48740	AT5G48740	-1.236653658	0.669452983	-1.536669495	0.505009596	-3.586153547	0.040731074	-1.296134192	0.760347123	-1.655108724	0.012526787	0.521102277	0.463882983
AT1G54215	AT1G54215	-5.541169131	0.329269542	-6.311439285	0.085874461	-6.447920803	0.017630287	-0.581568293	0.934711472	-6.347499753	0.025797515	-0.713219414	0.500856471
AT1G66500	AT1G66500	0.409420081	0.5074218	0.766828365	0.00010136	1.024169824	0.028907232	1.23457901	0.416170703	-0.367569015	0.398166246	2.102820569	9.99391E-17
AT2G39690	AT2G39690	0.709294938	0.378499936	0.986852667	0.04261371	1.330497437	0.001489597	2.140754487	0.541993938	0.947778369	0.453474968	1.805672216	0.001402561
AT2G33350	AT2G33350	-2.930258773	NA	7.406232758	0.055529312	3.492123311	0.009541395	-0.063561142	0.997515054	0.10936271	0.98877807	1.750056667	0.029549654
AT3G19550	AT3G19550			-4.13846489	NA	7.838920627	0.002210042	-4.868021144	0.672844426	-1.755726729	0.539582128	1.471685243	0.004177337
AT4G39670	AT4G39670	0.736526715	0.587563383	1.127752121	0.17341182	3.40235203	2.15411E-05	-0.982061492	0.691895802	0.096703295	0.968882556	1.425506286	0.004828619
AT4G14040	SBP2	0.209663014	0.703904147	0.056532829	0.849702366	1.357682779	2.73305E-13	1.850027507	0.070948623	0.212513394	0.592200811	1.062359049	5.28048E-14
AT2G42610	LSH10	3.384694986	0.099441456	-2.569965158	0.072877799	2.346567812	0.004941792	-0.429932968	0.871661081	0.209561145	0.462524306	1.057293773	3.89168E-12
AT5G44660	AT5G44660	0.246551793	0.595562239	0.589142707	0.007041621	1.126805424	1.29715E-08	1.16344618	0.379538351	0.177189042	0.733571412	1.048598573	4.38168E-11
AT1G32583	AT1G32583	2.349125684	0.177971178	1.27896682	0.159736403	1.695813161	0.000984672	0.090082312	0.976183443	-0.775457309	0.447139821	-1.003293974	3.65709E-06
AT3G04040	AT3G04040	-0.424873787	0.69839259	1.051813708	0.46028522	1.263923648	0.037732928	-0.675566048	0.907707664	1.073475754	0.39919608	-1.019137642	0.008963937
AT3G61450	SYP73	2.753185838	0.070745901	1.72551565	0.083085044	2.231597765	0.000249748	-0.363795189	0.936758504	0.242473616	0.878879956	-1.058591461	0.017654596
AT5G25450	AT5G25450	2.10783201	0.295621733	0.689124393	0.783753643	1.538223719	0.017135196	-0.604751598	0.792619066	-1.057270314	0.299340649	-1.068767899	0.001590005
AT1G31350	KUF1	0.520318845	0.350300528	-0.34056703	0.409554213	2.29695622	2.90482E-41	0.259698822	0.861051144	0.181223414	0.796907255	-1.097951892	2.94174E-16
AT3G05770	AT3G05770	2.445503419	0.391270537	3.107320726	0.286768718	6.760818209	0.031139617	-0.809717493	0.870396305	-0.037593304	0.985051085	-1.107364929	0.01800902
AT3G60720	PDLP8	1.975606095	0.218044287	0.652382441	0.499486417	1.594673682	0.034508104	0.452803244	0.7594526	-0.107742408	0.936190346	-1.17074344	0.001606083
AT5G24270	SOS3	1.70611908	0.058617708	0.17275931	0.817274002	1.39073573	0.006998007	0.01123131	0.997539086	0.155365751	0.8800772	-1.188999909	0.002477582
AT1G10470	ARR4	0.793926598	0.00636568	0.466266704	0.126638937	2.331339787	8.70578E-90	-0.210319757	0.787101126	-0.032116315	0.947746874	-1.189235609	3.98384E-18
AT2G07000	AT2G07000	6.675381029	0.177660057			2.281089577	5.27377E-06	-5.33171394	0.648919095			-1.195525522	0.002161532
AT1G72120	AT1G72120	1.863028629	0.297585295	1.56533995	0.163889415	2.161427946	0.000240698	-0.45488453	0.801691587	0.422808069	0.542006423	-1.239097153	7.43745E-06
AT4G18550	DSEL	3.316763868	0.205785229	4.074241191	0.066679792	7.425729334	0.00592967	-1.840200609	0.661563884	0.185606768	0.904726557	-1.260210601	0.011171116

AT5G16770	MYB9			-4.13846489	NA	2.066153215	0.020909913	0.189771724	0.991559366	-0.107810282	0.988021272	-1.271814603	2.35794E-05
AT3G63350	AT-HSFA7B	3.713372534	0.20778809	-6.730202855	0.061295558	6.59230778	0.045382211	0.626202965	0.715099832	-0.449866956	0.689600848	-1.281046183	0.004720678
AT5G02360	AT5G02360	5.655964325	0.319074855	-6.533483884	0.062712548	6.828766033	0.005789778	-0.140690399	0.987576531	-1.04700378	0.808484028	-1.282959246	0.01438805
AT4G00880	AT4G00880	6.136590223	0.275567586	-3.176438033	NA	1.220350148	0.000328072	-3.443034105	0.770575751	-2.288138356	0.601273744	-1.300775453	1.60027E-07
AT3G48390	AT3G48390	0.982661017	0.06495707	0.847118605	6.35815E-06	1.14453931	2.60401E-14	1.249344257	0.553691236	-0.339983746	0.385252516	-1.325350239	7.63785E-14
AT2G35170	AT2G35170	0.455372891	0.382751991	0.195580948	0.804988135	1.059595114	4.46259E-06	0.980216162	0.597380545	-0.41207245	0.493360816	-1.356013954	3.8839E-12
AT1G61820	BGLU46	0.789079221	0.297585295	0.315310456	0.605830228	1.219575583	0.005976365	-1.148005836	0.183329115	-0.804641894	0.010821699	-1.380634428	6.58389E-18
AT4G18610	LSH9	1.548548678	0.444072803	0.394772888	0.884456751	3.457887841	0.025251673	-2.079797555	0.613859108	-1.023101714	0.08501642	-1.389682561	8.42589E-06
AT1G56320	AT1G56320	2.768885496	0.089811388	0.293123815	0.965332241	2.664043606	0.032953254	-1.114142221	0.661736444	-0.25542422	0.885134295	-1.395196767	5.1629E-06
AT3G10320	AT3G10320	2.253052991	0.340815345	2.040109593	0.422514973	2.948388918	0.040472645	-1.517923908	0.499936609	-2.009762893	0.057158059	-1.407794774	0.005688546
AT2G38230	PDX1.1	0.946018286	0.040624377	0.918459327	8.36221E-08	1.515731255	4.88937E-25	2.292969383	0.183329115	-0.744483837	0.011093407	-1.409476036	4.12762E-20
AT1G65480	FT	2.052467223	0.267985964	-1.61452356	0.510489686	1.236538947	0.028719232	-1.147917436	0.365746137	1.365661464	0.227867455	-1.4655603	3.66771E-07
AT4G36450	MPK14	0.764124504	0.317451005	0.683294901	0.315031534	1.320615726	0.00058704	-0.062442634	0.973639349	0.287749204	0.641639638	-1.56962189	6.5443E-05
AT1G75450	CKX5	0.619475551	0.214642786	0.668529739	0.136339784	1.029488566	0.009639797	-0.899316935	0.294511423	-0.209928032	0.402950282	-1.622282935	6.69386E-44
AT1G61800	GPT2	1.990683814	NA	1.058471122	0.797558068	6.089904875	1.57489E-06	-1.018225239	0.834203073	0.281989629	0.828624466	-1.68083733	1.20129E-06
AT1G04220	KCS2	0.361166594	0.940507185	1.067643867	0.814331309	2.520338303	2.35342E-05	-0.291315099	0.967195835	0.212116495	0.919434066	-1.690899983	1.4755E-13
AT4G28530	NAC074	2.452445486	0.386662562	-0.743013378	0.786146042	2.438041803	0.018244436	-1.444334018	0.711255369	0.335898575	0.872528185	-1.69271201	1.92329E-09
AT1G20070	AT1G20070					4.444702042	2.95406E-10					-1.814767085	2.63316E-12
AT1G03570	AT1G03570					1.745289942	2.68477E-14					-1.842590693	2.30342E-10
AT5G28646	WVD2	3.57625681	0.177867981	0.906618663	0.736798563	1.861321588	0.012130155	-1.746632522	0.677620385	0.693029944	0.613826202	-1.906280277	4.1464E-18
AT1G16120	WAKL1	1.863311869	0.59667823	3.754782923	0.139503125	6.789134898	0.028894443	-2.830205338	0.632278424	0.656838243	0.805320688	-1.908716906	0.006022937
AT3G11415	AT3G11415	-1.1125501	0.879320402			4.001388254	0.010852541	-7.31064773	0.098303789	5.425568429	0.510876298	-1.915924958	0.04248055
AT3G61897	ath-MIR166b	6.740887587	0.222260919	-3.361240474	NA	7.181041635	0.011886546	0.016746565	0.988471189	-1.478596946	0.703258282	-1.994050391	0.04186877
AT1G23950	AT1G23950	-0.01129858	0.986160934	0.191448512	0.586319619	2.394299096	0.007186366	0.016746565	0.988471189	-0.096045958	0.806371006	-2.012051092	0.003497351
AT5G49290	RLP56	-0.445895267	0.692332809	-0.16131083	0.89251602	4.093944798	0.000254937	0.22277621	0.948597111	-0.043251648	0.984765765	-2.030899539	3.39333E-05
AT2G32530	CSLB03	1.46092828	0.124382766	1.592999901	0.125101837	1.811326749	6.09997E-05	0.08473223	0.979509283	-0.773190014	0.652173277	-2.085231673	2.35153E-06
AT4G04972	AT4G04972	4.616171969	NA			2.369684438	0.008902638	1.570789735	0.924835736	-5.980678993	0.31350844	-2.225213654	0.009311476
AT3G44120	AT3G44120	2.004580611	0.259976459	0.535570886	0.759093285	1.839503584	0.035772822	0.03118837	0.997449367	-0.223796533	0.968495475	-2.567488871	0.002842386
AT4G14780	AT4G14780	7.106798429	0.101053957	-5.440834787	0.30828466	7.453070009	0.006477466	-3.543705855	0.655815891	-0.957427108	0.755159043	-2.593932748	0.000112552
AT3G29570	AT3G29570	2.126870043	NA			6.689387683	0.00637212	-3.873481431	0.731041294			-2.679347283	0.032826551
AT3G53040	AT3G53040	2.12421405	0.485331922	3.454322536	0.13408384	13.49679157	2.58914E-10	-6.6771424	0.564319346	1.452339933	0.719986639	-3.174070451	0.000227224
AT3G07250	AT3G07250			6.28655218	0.227463234	6.164062553	2.26036E-13			3.535813429	NA	-3.182175583	0.022274471
AT1G23160	AT1G23160			-5.910639999	0.26434931	3.26809339	0.035166988	-7.586826526	0.326457456	0.490810799	0.930662149	-3.571645455	0.00158303
AT2G23240	AtMT4b	6.652426023	0.182108684	-2.353404057	NA	7.166576159	0.01911964	-5.252860951	0.653363031	-4.706767412	0.570555085	-3.677060738	0.02018322
AT5G55370	AT5G55370	6.647878957	0.180494511			7.786490181	4.97379E-05	-7.002057649	0.135283625	0.220052351	0.969622714	-4.206446798	3.46679E-08
AT3G58450	AT3G58450	7.384304467	0.073123536			6.410822833	0.014868669					-5.756376876	0.023528783
AT5G10120	AT5G10120					6.623376453	0.042202006	-6.063198499	0.616300683			-6.165586005	0.038076904
AT1G54870	AT1G54870	5.135882068	NA	9.473605452	0.050400287	7.85542386	6.08219E-05	-6.806572265	0.198640018	0.409940472	0.959952977	-6.993552395	3.78802E-07
AT1G04560	AT1G04560	7.442135896	0.058865668	2.651356793	NA	5.980063112	1.8428E-05	-5.388577563	0.595159861	2.080047258	0.711383732	-8.107191277	2.86969E-08
AT5G35660	AT5G35660			5.416375951	0.311226507	7.622853675	0.003580125					-9.758064984	0.002047641
AT1G47370	AT1G47370	-6.977408199	0.202891577	-6.364226761	0.180508515	-6.467332526	0.021837425			4.885997556	0.556508441	5.723035145	0.021909534
AT5G41750	AT5G41750	-1.054572821	0.326621865	0.865568447	0.325436449	-3.894823586	3.36679E-05	-0.425912857	0.823849926	-1.213640935	0.14763082	4.022214431	2.80897E-18
AT4G25490	CBF1	0.608561655	0.308834148	-0.0783921	0.792297572	-1.371895622	2.4533E-09	2.625557803	0.167653459	-1.02405857	0.154387444	3.729812968	1.39739E-10
AT4G11460	CRK30	-3.376401975	0.387336117	-1.170913622	NA	-3.110817144	1.32219E-17	-0.253610408	0.973864665	-0.80357172	0.885377618	2.997352745	3.30906E-08
AT5G47220	ERF2	-0.967957144	0.04619469	-0.406894249	0.227183714	-1.740466759	5.45129E-06	0.945680653	0.414486681	-0.060057981	0.963495178	2.885842268	2.02074E-22
AT1G63630	AT1G63630	4.817159862	NA			-6.563014284	0.012921985	-3.126920848	NA	-5.864555943	0.436704713	2.827674167	0.007872776
AT5G51190	AT5G51190					-1.123676373	1.18814E-05					2.698601431	6.0664E-102
AT4G12470	AZI1	0.979132685	0.445415744	1.083566463	0.540315673	-2.070724298	0.00420203	0.39489363	0.836779419	-0.534512181	0.445236462	2.576986662	1.30285E-39
AT5G23010	MAM1	-2.847335205	0.105664265	-1.980339548	0.160255167	-3.269741692	0.015016554	0.841954332	0.640740059	0.061155809	0.985585094	2.456142331	8.41014E-41
AT1G35210	AT1G35210	-0.549678111	0.410396471	-2.085874013	0.391305788	-2.217304492	0.00012159	0.483817966	0.825683018	-0.625484508	0.594846489	2.407627663	6.68812E-22
AT4G22513	AT4G22513	-4.596897629	0.431661015	-5.887474306	0.162632781	-5.104170332	0.0192963	-1.028326118	0.861051144	1.126513543	0.573217541	2.347047157	9.48896E-20
AT2G15042	AT2G15042	-4.227648281	0.079630604	-2.206635892	0.447807443	-1.337265673	6.84862E-05	-1.965526568	0.883412909	3.862218883	0.39950549	2.061705366	1.81505E-09
AT5G59730	EXO70H7	-4.065344747	NA			-1.521994954	0.020148704					2.046370691	1.05232E-19
AT4G30430	TET9	0.19013788	0.97172217	-3.137257615	0.221013546	-6.972482641	0.003554246	-4.962836267	0.66808962	-0.121724017	0.992611192	1.895622175	0.00034206
AT2G26740	SEH	0.89716812	0.034730601	0.828357506	4.04155E-07	-4.068404379	0.025945773	1.913942807	0.099695852	0.112132957	0.821418926	1.891658708	0.009303893
AT3G14760	AT3G14760	-1.157817021	0.214977743	-2.446396382	0.08939117	-1.584369121	0.000680655	0.905477353	0.703833757	-0.283817879	0.899553662	1.858317777	0.000392238
AT1G05550	AT1G05550	0.535834417	0.936914477	-5.07790712	0.345257625	-6.573833969	0.013922356	-6.916630941	0.507166524	0.891366199	0.818982935	1.838023129	0.008308959
AT2G34420	LHB1B2					-1.054430419	1.25053E-09					1.830212565	1.59075E-30
AT1G66400	CML23	-0.127693414	0.893881797	-0.112480247	0.887495804	-1.187532908	0.006039217	0.338817299	0.81670535	0.029579407	0.979247864	1.823042872	2.02254E-09
AT4G12030	BAT5	-2.183997614	0.094869688	-2.107249403	0.456861476	-3.269120028	0.002583088	0.472851299	0.823401939	-0.287484874	0.762931325	1.763863595	3.56541E-11
AT1G73805	SARD1	-0.92713441	0.291227646	-1.4181775	0.059764319	-2.305328136	0.000194859	1.18425006	0.62738272	-1.789018553	0.269079164	1.701287769	3.74388E-06
AT1G04660	AT1G04660	-0.938956062	0.465571275	-2.381427911	0.32589449	-8.145453244	6.85494E-05	-5.511260171	0.615454805	0.51255943	0.947746874	1.608600895	0.001226334
AT5G66520	AT5G66520					-1.15807336	0.00029915					1.530479779	7.90163E-05
AT5G04180	ACA3	-2.022250851	0.348058211	-3.826374769	0.084709865	-4.40959887	0.003913979	-0.335442222	0.977737576	-0.633993477	0.883729772	1.52749557	0.014274025
AT4G36500	AT4G36500	-0.679544935	0.149338346	-0.822094678	1.28803E-06	-1.398862868	6.83663E-07	0.679341955	0.66924865	-0.536115882	0.137145085	1.455948488	1.50812E-10
AT1G52100	AT1G52100	-0.823862149	0.105182973	-0.389315311	0.217304316	-1.69641625	8.10714E-12	0.29175577	0.718989301	0.97985084	7.76013E-08	1.453389709	2.55542E-11
AT5G20630	GER3	-1.785544894	0.172234464	-0.992594633	0.566660999	-3.271560556	1.34969E-38	-1.03214123	0.546687098	-0.442054647	0.041612958	1.426830179	2.8129E-12
AT5G08760	AT5G08760	-1.58939404	0.087743496	-0.0922567	0.929298011	-2.268752824	5.47009E-05	0.615139482	0.842959841	-0.17628171	0.944517409	1.394858122	0.010982843
AT3G16720	ATL2	-0.898978135	0.042949572	-0.671794848	0.024165509	-1.546565575	1.81174E-15	0.463216814	0.662219342	-0.681696861	0.016535834	1.352484566	9.47388E-26
AT4G23310	CRK23			-4.176821531	NA	-2.637530753	7.57326E-06	-6.909404345	0.514379829			1.344966921	0.010100015
AT4G22505	AT4G22505					-2.636089014	0.002248064					1.342868247	0.00158508
AT5G37770	TCH2	-0.675788522	0.111780519	-0.917009486	0.00154575	-1.190939992	0.00010139	0.410347764	0.659255524	-0.131749161	0.738367178	1.277989865	1.49114E-16
AT5G44600	AT5G44600	-0.804503363	0.34288677	-0.934450784	0.326094161	-1.205669626	0.004415258	0.368626752	0.828875452	0.978931608	0.018783152	1.270260726	5.52777E-09
AT1G11850	AT1G11850	0.640565344	0.458978863	0.240859549	0.782974719	-2.57241032	1.31533E-05	0.659647689	0.902675758	0.706712441	0.517194506	1.2535842	0.015439433
AT2G46510	AIB	-0.223490225	0.623142127	0.337007677	0.366605587	-1.119764251	0.000680785	0.107115829	0.911955093	-0.083556342	0.918426331	1.233895748	1.45193E-14
AT3G14630	CYP72A9	-0.61160942	0.431901255	-0.779112005	0.318767466	-2.118422752	7.81764E-05	1.898615079	0.696374342	0.869242566	0.644774623	1.200230592	0.021973893
AT4G03270	CYCD6;1	0.054893579	0.985842093	-0.511028871	0.756938797	-1.455157209	0.038376733	0.819420553	0.687176366	0.708325868	0.484106076	1.176016994	1.50804E-07
AT1G34380	AT1G34380	-1.036187497	0.280035081	-0.195669705	0.80829238	-1.872700053	0.0019925	0.682801148	0.757276638	-0.197233145	0.883566526	1.143982099	1.16819E-05
AT4G02330	ATPMEPCRB	-1.953551704	0.068108214	0.918361481	0.355654005	-1.504159956	0.001896108	-1.846951182	0.196045896	-0.740277001	0.014339444	1.14081204	1.27171E-15
AT3G58940	AT3G58940	-0.128189147	0.949460469	-2.183803396	0.136339784	-2.978257222	0.013051031	-0.436808536	0.947082547	0.27977258	0.831572245	1.112423272	0.001196658
AT1G21130	IGMT4	-0.343065349	0.576761569	0.201644266	0.195026808	-1.289432988	1.94398E-13	2.312066338	0.128407019	-0.091253508	0.857138563	1.071438227	4.63116E-12
AT1G20870	AT1G20870	-0.54573707	0.692604193	-0.071686721	0.979328921	-1.747274119	0.045619081	0.402607744	0.944057446	-0.291689165	0.917325189	1.070428857	0.014214264
AT4G03210	XTH9	-0.330944251	0.716467938	-1.620533665	0.10851692	-1.537597039	5.02372E-09	0.408709961	0.810393493	0.699629704	0.00076913	1.038651152	7.09814E-13
AT4G24750	AT4G24750	-0.649918608	0.010517499	-0.384357741	0.087309724	-1.075727949	1.97201E-10	0.361080642	0.666573155	0.565208255	0.008027695	1.038571406	4.16048E-16
AT3G23810	SAHH2	-0.811557417	0.616850378	-0.027591929	0.961894952	-2.258893013	8.42309E-25	-1.188263022	0.565728752	-0.377672695	0.01048166	1.013971503	1.64513E-11
AT1G13635	AT1G13635	-0.637380496	0.875205769	-2.758247503	0.422760933	-7.63988425	0.000203859	-0.965518504	0.857059659	-1.426175541	0.105439314	-1.012812408	0.015926527
AT4G09420	AT4G09420	-1.178301508	0.25714332	-1.008893886	0.278024625	-1.226268041	0.028671585	-0.360209905	0.928245224	-0.657267333	0.491144609	-1.033203113	0.027473053
AT3G06125	AT3G06125	-0.085552526	0.905602833	-0.790163094	0.038299696	-1.40316708	3.70022E-08	0.189952035	0.886772005	-0.025014894	0.974055498	-1.034741155	0.002098064
AT2G24762	GDU4	-0.479534189	0.777528879	-1.905777035	0.223891656	-2.766907503	4.81134E-07	-0.619796838	0.822327646	-0.302005267	0.595783013	-1.048833006	0.0228703
AT4G18430	RABA1e	1.280712155	0.798152281	6.474858629	0.057103259	-6.370002123	0.033147804	-1.586324545	0.685495817	-0.603104145	0.600536062	-1.085618275	0.004680675
AT4G02830	AT4G02830	1.829559654	0.587354024	1.720593162	0.463875953	-6.207721367	0.033926148	-2.189832505	0.051428123	-0.610206593	0.561519047	-1.08885829	0.004987989
AT3G50190	AT3G50190	0.348137588	0.910145261	-0.712286567	0.783990583	-6.446881689	0.025749403	-1.175001171	0.787678612	-0.093666979	0.974720863	-1.127849717	0.024031258
AT2G20750	EXPB1	-1.14890115	0.635095107	-0.746987317	NA	-4.593573159	5.17554E-05	-0.498140666	0.935898864	-0.910796211	0.109419665	-1.157560511	1.06099E-05
AT4G02630	AT4G02630	-1.212122626	0.357811798	-1.038479263	0.587517273	-4.885090804	6.35354E-08	-1.021653619	0.627184543	-0.498098638	0.556508441	-1.160087848	1.56355E-06

AT2G40260	AT2G40260	0.671177925	0.390140055	1.116478576	0.569458462	-2.151340948	0.004688549	-1.147551305	0.533068231	-0.67737875	0.511188955	-1.160120888	0.018229122
AT4G12390	PME1	-0.152802665	0.822761853	-0.983072159	0.174327256	-1.015349432	0.031658446	-1.043523303	0.433558429	-0.149477739	0.800883115	-1.182622257	3.6013E-13
AT4G27820	BGLU9	-0.802491674	0.28238224	-0.084278174	0.8780997	-1.030293539	0.006398323	-0.190445635	0.937499364	-0.31300158	0.798699426	-1.202539615	0.002355901
AT5G35732	AT5G35732	1.071208892	0.157176658	-1.567730743	0.272194371	-1.439605549	0.005998861	-0.511348197	0.817411494	0.33974656	0.519716763	-1.20960898	5.54555E-10
AT5G12420	AT5G12420	0.434597116	0.778740801	-0.405829256	0.784589986	-3.865122393	0.001721722	-1.876189763	0.173124482	-0.318045626	0.629318502	-1.248536786	3.67506E-16
AT4G13130	AT4G13130	1.574059193	0.794952744	1.25952684	0.703756629	-6.865321193	0.00482948	-0.592845182	0.922389968	-0.392209103	0.845286507	-1.318192262	0.004116615
AT1G74010	AT1G74010	3.497802269	0.171152167	-5.838500075	0.270384049	-6.440013613	0.018461326	-3.25012046	0.340875713	-0.439258522	0.737138931	-1.331120194	0.009835813
AT1G03820	AT1G03820	-0.121138029	0.964705665	-0.263494188	0.828301223	-2.917849165	0.0010472	-0.295670734	0.906841619	-0.422952604	0.182103611	-1.337887988	8.1165E-06
AT5G11540	GulLO3	-1.57259265	0.644890697	-2.390944986	0.396674716	-5.215661281	0.002207564	-0.472546121	0.944057446	-1.295043034	0.170484591	-1.338319274	0.014921404
AT2G38490	CIPK22	0.219430778	0.967147181	-4.162388174	0.449530875	-6.93212601	0.003759182	-1.489995652	0.770575751	-0.933582477	0.646759677	-1.353396201	0.01248171
AT2G24580	AT2G24580	0.395709493	0.542523834	-0.347606022	0.678866741	-1.511502339	0.000710072	-0.897450223	0.554617999	-0.620781939	0.2553851	-1.36320049	4.14534E-09
AT1G67870	AT1G67870	0.408220369	0.259011984	0.085366424	0.728196689	-1.492451972	2.08038E-06	0.277341398	0.840723231	-0.167664728	0.568739186	-1.38041023	4.69044E-11
AT2G04570	AT2G04570	-1.299627652	0.053353106	-0.77465708	0.212000774	-2.647539852	3.1893E-10	-1.192992349	0.240541784	-0.615313145	0.23763681	-1.386298033	3.3527E-15
AT3G23410	FAO3	-1.071275953	0.143698714	-1.721964488	0.050451482	-1.661798116	0.002288182	-0.648129571	0.708039647	-0.267642444	0.859145245	-1.40042346	0.002831605
AT4G19030	NLM1	2.352578044	0.521039619	-1.802638281	0.673975837	-6.486833564	1.06783E-06	0.494183145	0.832157671	0.785754343	0.467113472	-1.417154225	0.000358362
AT4G22980	AT4G22980	0.931744702	0.482760363	-1.587682843	0.341104478	-1.981675012	0.032707797	1.069729477	0.728429327	0.191848326	0.952784918	-1.444362601	0.012252116
AT3G22620	AT3G22620	1.246119954	0.154845347	0.584283951	0.494040544	-1.432930711	0.047134138	-1.523539978	0.343493265	-0.817680576	0.006836936	-1.445185374	2.12939E-23
AT1G52200	AT1G52200	0.105655073	0.860578205	0.446568413	0.802686832	-1.762140847	7.24922E-09	-0.278517694	0.842161979	-0.358392456	0.511253459	-1.455384165	1.9242E-11
AT5G19970	AT5G19970	0.938119153	0.240852223	-0.283555172	0.800919486	-1.189908965	0.024188337	-0.15067326	0.963509602	-0.710184899	0.271416954	-1.48292946	1.75404E-07
AT1G01070	UMAMIT28	1.208358792	0.058222136	0.653357693	0.756346813	-1.597131399	0.015002782	-1.066146511	0.604422229	0.175900154	0.84150489	-1.485062088	7.57211E-18
AT3G62930	AT3G62930	-1.484828791	0.200804436	-0.20561096	0.818220075	-2.093756382	0.005466379	0.095244574	0.982055475	-0.994434279	0.250286441	-1.488709929	2.71187E-06
AT5G04660	CYP77A4					-3.242796174	0.000184084					-1.526472296	0.010990235
AT1G64370	AT1G64370	0.198224826	0.652968819	-0.284132615	0.348840745	-1.501222979	5.60049E-06	-0.240402842	0.855431266	0.194365877	0.529391821	-1.527774366	6.42651E-19
AT3G23430	PHO1	0.802434605	0.051728068	-0.955231408	0.028580403	-1.548094372	1.54161E-08	-1.111360477	0.626508471	-0.737274995	0.173837217	-1.54327801	3.95687E-18
AT3G25130	AT3G25130	0.019899272	0.995808248	-0.895970086	0.701257749	-3.813470471	0.023322964	-0.521325033	0.932925673	-0.461579235	0.589186505	-1.576382518	8.40606E-07
AT3G25710	BHLH32	-0.154425902	0.748082706	-0.315484074	0.634866318	-1.382740238	2.01907E-06	-0.764116275	0.616300683	-0.397721601	0.30327919	-1.586905194	7.34308E-26
AT1G17180	GSTU25	1.304856199	0.845766617	0.786304771	0.768707211	-6.947940676	0.008151303	-1.859220851	0.640704059	-0.388008959	0.550428211	-1.59398792	0.003721295
AT3G13400	sks13	-0.774859571	0.842497187	0.195616726	0.914257362	-1.878158555	0.000425104	-1.442433584	0.62738272	0.249950679	0.936554664	-1.618570328	0.001204673
AT4G01750	RGXT2	-0.342562245	0.897310354	-0.496678706	0.54123455	-3.553816501	0.007387338	-1.340628912	0.731041294	-0.614819952	0.400375471	-1.690322661	7.97685E-11
AT2G40900	UMAMIT11	0.361696965	0.560518927	-0.35150866	0.538575608	-1.248899386	0.009595654	-0.971621357	0.641679337	-0.333911245	0.409507617	-1.697858602	1.13193E-24
AT3G22600	AT3G22600	1.411862519	0.113956383	0.552862817	0.605540689	-1.580546149	0.003324535	-1.739987734	0.316926557	-0.975226951	0.001535604	-1.76681291	3.05029E-28
AT4G01390	AT4G01390	1.611560956	0.496416317	-2.494769525	0.304649951	-8.467965293	4.36928E-07	-1.75751557	0.662219342	-0.357204158	0.75658734	-1.783764019	2.49298E-06
AT2G47000	ABCB4	-0.417645656	0.258853925	-0.662063734	0.179972104	-1.482002903	7.63559E-06	-1.085207526	0.107439117	-0.648767214	0.000815238	-1.805362726	1.93224E-48
AT4G37940	AGL21	0.90753532	0.811494429	0.339811507	0.930404286	-6.411068988	0.023530751	-0.874498948	0.664635521	-0.721572441	0.515629348	-1.844633087	1.32585E-05
AT1G66950	ABCG39	6.953215393	0.132897598	-4.253899481	NA	-6.558059809	0.01310078	-0.632483246	NA	-0.504096125	0.924868537	-1.85577163	0.001279558
AT5G63450	CYP94B1	-0.239844924	0.744274129	0.464807787	0.511011346	-1.30321217	0.008580073	1.001614534	0.610227484	0.321073977	0.797291564	-1.895183898	5.536E-08
AT3G56360	AT3G56360	-0.731533593	0.216506091	-0.543501097	0.045431609	-1.421366077	2.9559E-06	0.00366765	0.998375447	0.053093069	0.958438477	-1.918106522	6.35208E-05
AT2G42350	AT2G42350	0.703796388	0.622849484	-1.977489825	0.363575035	-7.241598278	0.001137077	-0.85194079	0.66808962	-2.347265678	0.05341887	-1.946790727	0.000869508
AT4G31730	GDU1	0.6068158	0.515977929	-0.879723735	0.267800994	-1.887715592	6.60102E-06	-0.998978579	0.731041294	-0.203972389	0.70277046	-1.955682462	1.38004E-26
AT5G16023	RTFL18	-4.821666152	0.254007982			-2.586889484	0.049758826	-1.00004709	0.841532762	0.1234427	0.952322031	-1.976156143	0.000676101
AT5G16570	GLN1;4	-0.306093498	0.948997247	-1.165072885	0.71294196	-6.742230159	0.011216665	-1.555763895	0.531516474	-1.060498596	0.529391821	-1.984872406	3.56285E-06
AT2G29740	UGT71C2	1.139015111	0.190385429	-0.788539368	0.502155838	-2.279080457	0.000333434	-0.345471987	0.750125148	-0.367945402	0.631435158	-1.990729768	6.97771E-17
AT1G44800	SIAR1	0.762652944	0.112946699	-0.530444286	0.065037083	-1.228418913	0.036145714	-1.529890435	0.13090699	-0.507450817	0.034261248	-2.005766099	1.8265E-70
AT2G01890	PAP8	-0.669230814	0.405761816	-0.850744054	0.07023353	-1.812797536	8.81375E-05	-1.049460518	0.527892502	-0.296188272	0.550479828	-2.039528652	2.04643E-24
AT2G41660	MIZ1	1.075531619	0.247503718	-0.775614621	0.320073222	-1.581555909	0.035100138	-2.028031643	0.62738272	-1.194207176	0.108570709	-2.157840738	1.01824E-14
AT4G29690	AT4G29690	0.046508744	0.991544266	-2.775894551	0.458286134	-6.554940326	0.01724331	-1.838756537	0.643357707	0.313569989	0.804230791	-2.205373066	1.16567E-06
AT5G48940	AT5G48940	3.833121955	0.093826459	-1.479949448	0.472694811	-7.344360367	0.000941243	-0.018504664	0.99779774	0.966444993	0.177453251	-2.206396942	2.24902E-07
AT4G21903	AT4G21903	2.043616997	0.637529539	-2.40699105	0.430770584	-6.57467252	0.015122426	-2.858350559	0.619569853	-1.383057315	0.524219167	-2.461721714	6.30942E-06
AT5G40510	AT5G40510	2.322616531	0.412525841	2.155940989	0.513479503	-7.0619178	0.003300785	-1.505227936	0.702246294	-1.301430865	0.106408321	-2.586572691	4.92612E-09
AT3G25790	AT3G25790	4.696539729	NA	-5.721384902	0.281155108	-4.928958071	0.016633464	-7.478509669	0.077042066	-2.900820205	0.390095752	-2.653232755	3.25198E-06
AT2G39370	MAKR4	1.318050175	0.532265183	-0.626094988	NA	-6.970802252	0.003364946	-1.973148024	0.632278424	-0.336436568	0.798599938	-2.727509253	7.85075E-11
AT2G46685	ath-MIR166a	-3.455215133	0.445554504	1.924104042	0.71960671	-6.690808853	0.009993243	0.279270311	0.982356953	-0.401681762	0.930861837	-2.80138508	0.002645909
AT1G52800	AT1G52800	-1.109772515	0.700044629	-0.193126175	0.976493264	-7.805200843	0.002536471	-0.453280919	0.956296074	-0.001139705	0.999968179	-2.810820109	1.49849E-05
AT2G47140	SDR5	1.685178002	0.430062996	0.932701976	NA	-6.19815292	0.035479239	-1.334426038	0.795199419	-0.595552913	0.802042097	-2.870335975	0.003764609
AT1G68880	bZIP	2.809996104	0.065497448	-0.018861283	0.990655327	-2.03750859	0.010747361	-1.52714693	0.457619105	0.186292194	0.865593881	-2.928696269	3.75738E-43
AT1G52830	IAA6	-0.734196277	0.784357485	-3.378802426	0.064097534	-8.147604992	4.49688E-06	-2.243595191	0.707962062	-1.488516226	0.508120478	-3.090202576	0.000542852
AT2G27000	CYP705A8	2.938101151	0.250896796	-0.952720988	0.708599249	-5.907047503	0.012777109	-0.368149648	0.961157568	-0.003454163	0.999095849	-3.165758347	1.32509E-08
AT1G35625	AT1G35625	3.770901877	0.318296183	-6.005790621	0.253180668	-6.65431895	0.010024125	-2.190133149	0.715099832	-0.163788887	0.962364022	-3.351140927	0.000675339
AT5G28520	AT5G28520	5.258566845	0.357350363			-6.120463232	0.04841946	-6.446337223	0.598901706	0.962866079	0.944351305	-3.55499713	0.007150324
AT1G64920	AT1G64920			-3.761655451	NA	-6.710471145	0.04363444			-1.330951259	0.88237076	-5.239834924	5.96889E-07
AT4G22210	LCR85					-6.442716009	0.020061019	-5.503360692	0.641552314			-6.139186567	2.32401E-13
AT1G52680	AT1G52680			7.163718334	0.163894042	10.46600308	0.024106251						
AT2G45580	CYP76C3	5.756120256	0.309427609	5.416375951	0.311226507	8.216671135	4.30874E-05	5.044845883	0.66524984			-3.551145768	0.567278555
AT1G75830	LCR67			6.48066178	0.216235729	7.354319169	0.007435446	-4.240317945	0.706790319	-5.972091554	0.316459036	-3.664112675	0.293077129
AT4G25200	HSP23.6-MITO	0.290589732	0.944917689	1.260110623	NA	7.309788036	0.001305801	-5.097402913	0.663046749	-2.021070663	0.635351512	-1.181305338	0.546841096
AT4G00350	AT4G00350					7.16938969	0.015941824	-4.709284728	0.680482607	3.890233918	NA	0.825276113	0.906849279
AT4G00210	LBD31	3.383318152	NA	-4.748600036	NA	7.138716843	0.016605717	-0.146092675	0.985077447	-0.967084064	0.465426536	-1.036539087	0.074946916
AT3G56891	AT3G56891	0.329688091	NA	-5.541109678	0.298605949	7.021427247	0.001910781	-4.519784755	0.260795959	1.674595701	0.80706189	-0.463020217	0.714217977
AT5G40645	AT5G40645	4.910080283	NA	6.490566015	0.2155141	7.003797836	0.01857227	0.32204547	0.979362915	1.32316961	0.805380892	-0.371626957	0.849218537
AT5G60320	AT5G60320	1.504782086	0.785130486	4.031469431	NA	6.971950591	0.020873826	-6.129794355	0.615358268	1.317823445	0.899018771	1.975059594	0.303995708
AT4G17585	AT4G17585					6.944140389	0.021074838					1.456458497	0.398826939
AT1G78720	AT1G78720	2.769972114	0.345956696	-0.369369579	0.939333377	6.918240334	0.002678586	2.652518235	0.613859108	-2.416752355	0.563990873	0.184453323	0.968768927
AT4G22460	AT4G22460					6.87585011	0.034381622	-2.864628523	NA			-0.752616982	0.028698876
AT2G02030	AT2G02030	3.941445652	NA			6.785067557	0.030947739						
AT2G22460	AT2G22460	1.01425555	0.889207645	-0.580705521	0.929298011	6.71889069	0.008350533	-4.606586329	0.685495817	-3.031200657	NA	1.264890224	0.490934846
AT5G14130	AT5G14130	0.164237099	0.975054583	1.822795128	0.712888255	6.670502298	0.040685047	-4.628401639	0.271829457	-1.78291614	0.226976579	-1.096472329	0.106559585
AT1G66110	AT1G66110	-4.636248157	NA	-0.020163787	0.994418253	6.662432261	0.000485263	1.581354984	0.919773518			0.39512112	0.871619987
AT1G20180	AT1G20180	5.528536566	0.331020991	6.383984215	0.20643017	6.657383116	0.039808965	-3.291923963	0.602119156	-0.067614536	0.988694176	-1.908073403	0.396996488
AT2G32885	AT2G32885	0.815218127	0.846040316	2.614556146	0.36185984	6.656605711	0.038320146	2.71260191	0.706952662	0.972038256	0.790248164	0.221847396	0.931985216
AT4G32630	AT4G32630	3.726246192	0.312982783	1.627220145	0.616811796	6.655501001	0.008440368	-5.563385291	0.637815859	1.832130703	0.810706099	0.043644519	0.989597198
AT2G15500	AT2G15500	-0.519143539	0.939500202	6.402623245	0.222412481	6.653806567	0.043235119	2.22617956	0.855010607	6.463962618	0.282043163	4.063410015	0.448011915
AT1G12150	AT1G12150					6.648752265	0.038373651					-1.980925018	0.401130132
AT5G52500	AT5G52500					6.59877518	0.010540303					-4.763612891	0.309239427
AT3G26934	AT3G26934	-2.930258773	NA			6.496192183	0.015191407					4.891705743	0.080957028
AT4G09870	AT4G09870	1.156616053	NA			6.455786135	1.12053E-05					0.407471228	0.674701796
AT4G35690	AT4G35690	3.459440076	0.245148462	2.831721937	0.122565613	6.026862669	0.003395084	-1.60315903	0.911720308	-0.935341685	NA	0.85556483	0.607512355
AT1G72100	AT1G72100	5.716324947	0.313319667	6.450762689	0.1945833	5.933002396	0.040890199			-2.833092005	0.753699142	-2.562258388	0.125566732
AT2G46950	CYP709B2	1.430558731	0.55743614	2.099166221	0.128424477	5.82626376	4.43146E-12	-4.08236345	0.319072725	0.095979972	0.963419708	0.741698207	0.042992381
AT1G69880	TH8	4.360717475	NA	3.367546552	0.053062821	5.688636159	0.009354081	-1.223586087	0.767987589	-0.580854421	0.178726868	-0.72547908	0.168060922
AT2G18540	AT2G18540	5.5869848	0.325482345	2.680062676	0.645186607	5.569934168	0.000105832			2.74674029	NA	-0.138927184	0.973316881
AT5G28442	AT5G28442	-0.130437071	0.94864768	-0.537129722	0.620975341	5.348137822	0.008869167	-0.760592058	0.817796099	0.292173652	0.839560047	0.525348083	0.781967853
AT2G38240	AT2G38240	0.249706697	0.973333102	1.24474156	NA	5.273713285	0.023925225	-2.108716459	0.820789421	6.08529011	0.389152097	-2.812820653	0.239865932
AT2G31085	CLE6	3.200111902	0.585655556	0.37282079	NA	5.123969436	0.000316737	-1.899778715	0.645398888	-0.165348136	0.946158029	-1.857859376	0.148250462
AT5G44310	AT5G44310	7.23446138	0.082203797	3.918470663	0.184891312	5.101989509	2.21812E-10			5.780307094	0.39039795	-1.0773989	0.797712529

AT4G34470	SK12	0.590032211	0.707402458	2.459387907	0.191849166	4.946233485	0.034131315	0.614633693	0.957394163	-0.01765499	0.998738887	-0.425668004	0.860993089
AT5G51580	AT5G51580	4.835996915	NA			4.86829588	0.000265547	-2.192461302	NA			-0.984007339	0.069759495
AT3G60150	AT3G60150	0.417892768	0.606037644	1.112467609	0.05922289	4.622854901	0.0389736	0.748142236	0.683449697	-0.064618451	0.966304751	0.565405382	0.668532147
AT5G17260	NAC086	-1.025569325	0.858282276	0.12218508	0.975957737	4.579768433	0.02298482	0.370418647	0.963509602	0.918512896	0.594966702	0.513582886	0.793550636
AT5G10945	ath-MIR156d	1.259856987	0.568753274	2.702956723	0.473180019	4.300635704	1.3287E-07	-0.037780974	0.994162268	0.868742842	0.543343218	-1.170955885	0.079025133
AT3G43110	AT3G43110	2.400032016	0.433015454	3.149309663	0.507787364	4.299260051	0.043666758	-1.627755935	0.830658776	-1.278185437	0.621001856	-1.151935195	0.126109015
AT5G04380	AT5G04380			3.017706639	0.479054175	4.296882176	0.001019703			2.730108096	NA	4.142899364	0.421796108
AT1G68170	UMAMIT23	1.317707444	0.648337966	0.239536192	0.965676281	4.20528957	4.81752E-05	1.539954958	0.606948021	-0.145140837	0.952303785	0.943016355	0.024544191
AT1G52770	AT1G52770	1.643886998	0.809933488	-5.87455115	0.267759424	4.051141394	0.000172429	2.279268519	NA	-1.139552242	0.891797434	0.972618143	0.002631465
AT1G66795	ath-MIR157b	5.908643371	0.295872049	6.642387532	0.156615152	4.000232072	0.022392736	-4.416620964	0.411130518	0.120491849	0.985120284	-0.357259677	0.729140709
AT1G74890	ARR15	0.752074757	0.758173469	-1.895195122	0.211026057	3.614435435	0.001014868	1.685886773	0.313609174	-0.650278155	0.680876398	-0.335417827	0.458795209
AT2G21235	AT2G21235	-0.857222977	0.908023822	-5.702164045	0.283049535	3.584591481	0.013156358	2.497172576	0.845274118			1.713978706	0.701720435
AT1G19230	AT1G19230	0.233370915	0.925644446	1.42611451	0.473577943	3.462773899	0.014611949	-0.248315239	0.954974702	-1.267876463	0.163436416	-0.959263252	0.047899626
AT1G18830	AT1G18830	0.37752573	0.956654223	1.24555337	0.726360135	3.350803203	0.009699664	-4.716520456	0.355648666	1.069707072	0.859192131	0.192817533	0.898456199
AT5G26147	ath-MIR156f					3.291487579	0.039108226					-1.499196489	0.478024454
AT1G04500	AT1G04500	1.205648786	0.669322447	0.877090517	0.815442713	3.258567195	0.011414865	2.031367713	0.276496238	1.346533918	0.713259931	0.757570008	0.633569812
AT5G41660	AT5G41660					3.218919288	0.027939543			4.716613333	0.570054815	0.178463776	0.862377582
AT5G66700	HB53	2.628493212	NA	-0.418975764	0.833982833	3.083720077	0.001973505	-0.682171733	0.901031341	0.165708553	0.891016552	0.42351665	0.119602266
AT3G12955	AT3G12955	2.984365151	0.189777491	3.243188362	0.172986026	3.080147414	0.005095256	-0.015396614	0.999006241	-3.093265217	0.236928233	0.8604328	0.671915895
AT1G18280	AT1G18280	3.501316496	0.132693606	-0.581788531	0.870230461	3.032114275	1.88702E-05	0.590849824	0.923622633	-2.03355654	0.282753298	-1.206993449	0.058920053
AT1G43910	AT1G43910	1.028538642	0.695756899	1.824118731	0.154812137	3.006328229	1.56823E-05	-1.640094228	0.676109289	0.017546123	0.992519652	0.186658529	0.687886436
AT1G37140	MCT1	3.912846536	NA	4.01296404	0.104697374	2.999019367	0.04540387	1.112652105	0.845234181	0.087397255	0.99569371	-0.595481066	0.606466457
AT4G31610	REM1	-1.456443729	0.806134891	-4.975200483	0.356104166	2.905319673	0.00076391	-0.726275799	0.891533981	-0.778142972	0.407444638	-0.605141643	0.088581898
AT2G25095	ath-MIR156a	1.676755538	0.398434726	0.770256679	0.464327308	2.883253192	0.0003612	-0.193955878	0.953045007	0.62125619	0.534429577	-0.650223236	0.164169241
AT1G05560	UGT75B1					2.841885313	7.15053E-07					0.417919249	0.514856652
AT1G24735	AT1G24735	2.568982	0.251041591	1.987439806	0.320503015	2.793667331	0.015942633	0.059096672	0.996799452	0.871461969	0.707010797	-1.267355805	0.286765105
AT2G35550	BPC7	1.950656877	0.292424745	1.018564394	0.33432887	2.783150173	0.018600467	-1.329742504	0.214881581	-0.06218607	0.976570624	-0.761492634	0.215145544
AT3G47870	LBD27	-1.545125527	NA	6.905326837	0.182539024	2.757213054	0.001344708			3.131163454	0.68232732	0.630120062	0.512831064
AT1G66170	MMD1	-0.815907405	0.775261717	-0.041170355	0.990942336	2.735011723	0.012330554	0.131623156	0.991542137	0.94246123	0.908594277	1.610532213	0.303135263
AT2G40050	AT2G40050	3.13179887	NA	5.343401819	0.318517823	2.701104381	0.047382165	6.287763961	0.606948021	0.071286571	0.996204637	0.137505787	0.919696947
AT5G26170	WRKY50	0.712809487	0.652328333	0.84111929	0.322432115	2.684423178	4.29834E-07	1.271034949	0.204145338	-0.325819016	0.816809207	0.872124106	0.009493431
AT4G10845	None					2.57990344	0.001839482					-0.487983755	0.646992921
AT3G22910	AT3G22910	0.329475966	0.831680879	0.391863915	0.770238672	2.535351329	7.59698E-06	-2.977872296	0.416717256	-0.603485773	0.698012604	1.122789789	0.072185283
AT1G52315	AT1G52315	0.375127608	0.900538403	-0.360557568	0.804247356	2.520292312	0.035364478	-0.855043208	0.90270351	0.563287016	0.758399047	0.987367821	0.127993262
AT5G52190	AT5G52190					2.460176572	1.582E-35					-0.235488317	0.550631606
AT5G20670	AT5G20670	-3.455682031	0.108650231	1.346609298	0.768707211	2.424867568	0.002676138	-2.244314457	0.596083291	-1.593171631	0.258496511	-0.918950944	0.047723963
AT5G08210	ath-MIR834	0.726647538	0.87082448	1.52825506	0.806698584	2.401099917	0.02912562	0.512663556	0.954565337	1.486329069	0.534429577	0.900127372	0.715258872
AT3G62090	PIL2	0.075259495	0.990985016	3.720438395	0.117719614	2.36539655	2.09344E-10	-0.42715793	0.899519049	-0.716092103	0.515629348	-0.154839082	0.420684331
AT1G06148	AT1G06148	0.961174776	0.587736319	1.238744619	0.625737228	2.340349169	0.024142936	0.880704556	0.865477998	0.551215423	0.851431725	-2.067489755	0.082446369
AT5G07380	AT5G07380	1.377235826	0.301385348	2.047914884	0.146956252	2.320256879	0.002685366	-0.252542673	0.929506087	-0.496404659	0.719986639	-0.201284156	0.711420989
AT4G33666	AT4G33666	-0.965174778	0.835732851	6.303634403	0.076400821	2.317593334	6.92987E-31	1.021450474	0.932561267	-0.786065031	0.93866942	0.290521959	0.553216562
AT1G72070	AT1G72070	4.951171168	0.068921167	5.266784785	0.325767534	2.164049577	0.048624201	1.949356158	0.892607537	-0.880995731	0.937302004	-0.46350698	0.118956926
AT3G11550	CASP2	1.085891517	0.226298119	-0.166899719	0.897372713	2.161599334	4.73834E-06	-0.651647211	0.52993453	-0.721463976	0.034469941	-0.872299957	0.001105315
AT5G03840	TFL1	1.32569445	0.798152281	-0.141835082	0.975124251	2.156704504	0.006906028	-0.752664547	0.888051184	-0.963367397	0.549721675	0.695207478	0.047474296
AT1G54050	AT1G54050	0.576905071	0.504805655	0.459864044	0.532813361	2.139409172	0.010029665	0.422833595	0.828170856	-0.411478366	0.731699443	0.484742007	0.347001116
AT5G64190	AT5G64190	2.391916793	NA	1.489030241	0.157211688	2.117248325	0.001517116	0.38317423	0.792939242	0.058101143	0.976783113	-1.072821689	0.073138687
AT2G38220	APD3	-2.621717902	0.681232123	-0.421959024	0.931592925	2.114406817	2.40856E-06	7.22297796	0.459923668			0.896570159	0.528358783
AT1G02700	AT1G02700	0.811958849	0.725545918	1.337391048	0.138538524	2.101870592	0.023656241	1.912364065	0.355991528	0.490925724	0.729469634	-0.093412749	0.903653063
AT3G62510	AT3G62510	0.425306329	0.927029866	2.361569174	0.357909155	2.090746555	0.00186527	0.851014932	0.78527074	-0.67442338	0.924627441	-0.500254664	0.531612971
AT3G04184	AT3G04184	0.761764	0.398155145	0.414052036	0.610052754	2.057052029	0.019032077	1.013733783	0.596296903	0.510814324	0.719271526	0.094607567	0.934943825
AT5G45990	AT5G45990	1.94452744	0.493488039	-0.908635692	0.825632002	2.052270525	0.034105765	0.935426075	0.921639209	0.553858762	0.894043142	-0.720823596	0.512725931
AT1G48320	DHNAT1					2.048892006	0.00747843					0.871985826	0.302184349
AT5G28490	LSH1	-0.627359976	0.775261717	0.18592574	0.882610442	2.024150916	0.009147976	-1.027358039	0.66808962	-0.194113142	0.772141111	0.522442927	0.102250292
AT3G02390	AT3G02390					1.99130526	1.9058E-05					0.848128789	0.042054397
AT1G23450	AT1G23450	2.321819558	0.396255704	1.183707676	0.309364667	1.972111391	0.035874982	0.345524998	0.911720308	0.308873672	0.885563354	-0.179371373	0.855893682
AT2G45320	AT2G45320	0.911886373	0.03619454	0.899171254	0.042369231	1.965088999	1.37742E-19	0.703117968	0.67307941	0.162188098	0.866906715	0.04579045	0.892343554
AT2G29480	GSTU2	2.715058697	NA			1.962904093	8.19904E-06	-3.710612614	NA			-0.721178555	0.031255001
AT2G35750	AT2G35750					1.941809104	1.67042E-08			2.153164503	NA	0.8666101	2.45842E-05
AT2G15310	ARFB1A	0.853742753	0.40338726	1.886538357	0.057634834	1.926876919	0.000485263	0.540722088	0.696374342	0.570892982	0.505833226	0.018900913	0.976431114
AT5G28050	AT5G28050	0.592836483	0.150114991	0.744876547	7.59683E-05	1.924671183	1.16574E-36	0.993655791	0.519283767	0.156025244	0.53525964	0.238094978	0.114488325
AT3G27025	AT3G27025	0.845488645	0.753506382	0.649962543	0.710248622	1.904005351	0.001805211	-2.591613142	0.653363031	-0.096950214	0.9653838	0.385030629	0.469621059
AT4G00200	AT4G00200	0.84277706	0.149887355	0.910564051	0.049917376	1.892672898	1.16339E-10	-0.016150001	0.992570276	0.574648366	0.31770781	-0.286127442	0.295607317
AT2G39110	AT2G39110	0.705873078	0.769760628	0.365215556	0.885086412	1.890694568	5.30278E-16	-0.939981262	0.682962505	-1.020673152	0.10891184	0.003455835	0.992804029
AT5G18255	AT5G18255	-0.078353924	0.949690135	0.753068362	0.354908038	1.887674058	0.001172769	0.670337428	0.699340866	0.271180496	0.751599754	0.217178434	0.58158109
AT2G17430	MLO7	1.059883415	0.144327596	0.597219006	0.33545998	1.855320707	0.001644269	0.929082396	0.541293542	0.334812901	0.611423968	-0.39082529	0.510936831
AT4G39364	AT4G39364	-0.265372326	0.940716282	1.349845299	0.637337505	1.837996	0.040467707	-0.815190475	0.924948308	1.342437756	0.649898068	0.605642962	0.468707594
AT3G13450	DIN4	0.770436089	0.091524236	0.632343497	0.001608987	1.822072701	1.27436E-41	2.337546023	0.155832685	0.092885132	0.855204799	0.663994843	4.87771E-08
AT1G28940	AT1G28940	0.781341782	0.897738708	2.937531055	0.451735576	1.798902489	0.030314375	-3.573885544	0.599175866	-1.043361029	0.775246054	0.510410798	0.522173257
AT4G15710	AT4G15710	2.271036231	0.369131153	2.534346396	0.05224477	1.785329292	0.040374394	1.176027432	0.663046749	0.246597424	0.896866257	0.480807649	0.542618837
AT5G57565	AT5G57565	0.201152805	0.798361804	0.205886803	0.549976193	1.78295055	3.9366E-33	1.077980388	0.372330299	0.360854099	0.41464716	0.056720536	0.897444316
AT2G20815	QWRF3	0.760387664	0.388287493	1.300768307	0.054402823	1.771628799	2.79199E-07	0.440503649	0.706790319	-0.244953845	0.712660934	0.20525965	0.575978195
AT3G45440	AT3G45440	2.228515332	0.299731107	0.897614882	0.805492773	1.770811107	0.002327426	0.071458114	0.995194798	-0.886042052	0.653948393	0.427819936	0.193672247
AT1G02230	NAC004	-0.184901605	0.926556874	-2.052102184	0.689600145	1.743884613	0.0223202	-1.363112736	0.588156522	-0.598393103	0.794004103	-0.988658617	0.090657074
AT2G45910	AT2G45910	0.824575701	0.036787526	0.818633447	0.000480723	1.731414034	2.84993E-39	0.211817629	0.893988754	-0.126742043	0.796189324	-0.609479473	0.000219094
AT2G38110	GPAT6	0.532975502	0.691700543	-1.325047681	0.354139102	1.703916865	0.007902664	-0.966902329	0.675189064	-0.78091329	0.23980685	0.441227806	0.050260163
AT4G25707	AT4G25707	1.278983501	0.236552888	0.98412695	0.070291464	1.699384101	0.000112197	0.74051883	0.62738272	0.484299205	0.53583656	0.600148636	0.036539543
AT5G04520	AT5G04520	0.650117758	0.175885863	0.731090996	0.112167841	1.696921876	5.99414E-14	-0.063912829	0.963509602	0.07420361	0.923509746	-0.099585049	0.729401954
AT1G36070	AT1G36070	0.719320332	0.124120872	0.779257274	0.003328648	1.691108287	8.32133E-16	0.527754609	0.691824202	-0.080785848	0.871060407	-0.506795416	0.001309436
AT2G34070	TBL37	0.953946941	0.030474671	0.83701825	0.000694486	1.679373758	2.65906E-20	0.078125952	0.929506087	-0.042736598	0.944687474	0.210541159	0.141351932
AT1G49530	GGPS6	1.264322257	0.464606121	0.610521966	0.481167685	1.678946267	0.001311566	0.40763337	0.840363187	0.082638341	0.965131427	-0.129120702	0.814647393
AT1G10690	AT1G10690	1.115681176	0.240834319	1.051338604	0.121989108	1.670666098	0.000155526	0.375611784	0.893988754	-0.225592645	0.872795006	-0.060326724	0.906544453
AT4G39180	SEC14	0.887367997	0.235435774	0.74519528	0.064868064	1.669744494	0.030614035	1.208839382	0.151807634	0.086786116	0.919937283	0.37987223	0.541765988
AT4G39361	AT4G39361	1.4262129	0.515566723	0.897099365	0.592931167	1.66729	0.000312483	0.571180433	0.794188368	0.478711901	0.791689922	0.542288575	0.192943039
AT2G43400	ETFQO	0.579412891	0.225753093	0.734871823	3.1246E-05	1.65320123	1.99953E-28	1.66450992	0.23877538	0.262904718	0.433183088	0.341480766	0.06264741
AT1G67070	DIN9	0.436709378	0.64257914	0.886382362	0.1005941	1.631846332	1.54604E-06	1.979166552	0.393926565	0.276529823	0.816953676	-0.254776785	0.69386546
AT1G65110	AT1G65110	2.153535046	0.367204741	1.331000639	0.685053259	1.625096109	6.96771E-05	-0.594263264	NA	-1.63909533	0.193967676	0.417184059	0.10890804
AT4G23900	AT4G23900					1.624451609	0.004251119	3.94199062	NA			0.812224083	0.001057088
AT5G18245	AT5G18245	0.203602308	0.891682817	-0.074255656	0.937781078	1.60746184	0.001839482	0.654005663	0.721140128	0.523677124	0.5763915	0.413594843	0.27240914
AT5G42010	AT5G42010	0.260532116	0.878809942	1.143690805	0.611731647	1.599812515	0.039121975	-1.137848428	0.805348024	-1.118875789	0.334261823	-0.872223383	0.048761083
AT1G18270	AT1G18270	0.487489429	0.202952096	0.045022514	0.897436009	1.55924498	2.29235E-20	0.891207989	0.627094298	-0.190729518	0.531929726	-0.207663749	0.366994106
AT3G22750	AT3G22750	0.771845546	0.190166506	0.929730575	0.020498843	1.558841899	5.38909E-20	0.653518529	0.568009156	0.223873947	0.616572022	-0.20497774	0.336891668
AT5G42825	AT5G42825					1.556381318	1.6453E-15					-0.365730729	0.030746078
AT3G57520	SIP2	-0.144137217	0.807661862	0.090593177	0.82339931	1.5543272	1.21285E-31	2.657651261	0.149337129	-0.143703658	0.769619898	0.079791608	0.791409046
AT1G53785	None					1.543377878	7.15914E-09					-0.709729974	0.005013406

AT1G27170	AT1G27170	0.561711039	0.654052363	1.256662056	0.089190316	1.535447976	0.011100472	-0.53598351	0.803788858	-0.376219717	0.592718867	-0.863799266	0.002957081
AT1G72800	AT1G72800	1.209620184	0.243781713	1.727577518	0.06244784	1.532195697	0.014001849	-0.237232346	0.926730556	-0.315583501	0.813646907	-0.128144326	0.766156189
AT1G80380	AT1G80380	0.064264334	0.918347866	0.387963527	0.154882945	1.531963448	1.9132E-24	0.998744044	0.619462504	-0.048751663	0.90360881	-0.732492707	4.84206E-06
AT1G68290	ENDO2					1.516427059	0.026138069	-5.286207544	0.652473285	-2.296449527	NA	-0.543262187	0.077157855
AT4G25810	XTR6	0.051603354	0.956028071	-0.142429123	0.864546714	1.50834405	0.003435498	1.3127931	0.422258087	-0.762315299	0.006555842	0.676558275	0.005956868
AT5G61850	LFY	1.65565263	0.366861619	1.201219614	0.2506451	1.491748886	0.047997282	1.132135816	0.594299218	0.126762041	0.958669515	0.12499754	0.871657535
AT4G16280	FCA	0.932913185	0.013849244	0.858103346	3.70058E-06	1.488181701	4.79564E-27	0.582557578	0.685495817	0.159991164	0.571318331	-0.06082023	0.759016936
AT1G33640	AT1G33640					1.48764518	0.044435394					-0.281987184	0.75665223
AT5G28030	DES1	0.433893507	0.875833593	1.168179589	0.364620992	1.483579077	0.002463613	1.118019365	0.647947756	-0.011431462	0.99699153	0.378616628	0.600457284
AT2G13960	AT2G13960	1.837303531	0.101292199	1.170139401	0.12205805	1.476810296	0.000198152	0.37889554	0.890842517	0.500435363	0.698398994	-0.197603252	0.694764607
AT4G29230	NAC075	0.090880854	0.947900337	0.135454286	0.866922987	1.475272175	0.00766387	-0.307209629	0.796607769	0.297898721	0.688409418	-0.784917912	0.012396791
AT5G49640	AT5G49640					1.460268965	3.1068E-17					-0.264648878	0.244714775
AT1G69310	WRKY57	-0.23125142	0.826301167	0.098228006	0.926555944	1.454640199	0.027724365	-0.601888219	0.66524984	-0.011087822	0.993373477	0.019265528	0.967139311
AT5G48570	AT5G48570	0.640971014	0.507787563	0.767443067	0.328621592	1.450218618	0.026516085	-1.220760636	0.653363031	-0.175464042	0.834876738	-0.17015954	0.655577041
AT4G29990	AT4G29990	0.355133428	0.714897497	1.111726654	0.064675975	1.44706892	5.86425E-06	0.341020959	0.734187528	-0.275671128	0.589186505	0.329262833	0.149081166
AT1G48330	AT1G48330	1.33786634	0.156919471	0.340172449	0.770238672	1.43752791	0.001187807	-1.353471548	0.604422229	0.410814273	0.709610691	-0.192775559	0.689800627
AT3G59020	AT3G59020	0.780663044	0.00089687	0.868165161	2.19972E-05	1.435925582	2.68308E-35	0.180419323	0.828875452	-0.083512496	0.731606335	-0.112673201	0.575639295
AT1G09710	AT1G09710	0.663972985	0.088156774	0.378586164	0.330494084	1.435508922	1.86444E-13	0.622991652	0.66808962	0.337879056	0.452730925	-0.083797358	0.761927143
AT3G61070	PEX11E	0.185677591	0.72520844	0.843175896	0.000577225	1.432193088	4.51593E-21	0.67044355	0.644613901	-0.244141441	0.449974648	0.015299581	0.948941317
AT1G80050	APT2	0.631896151	0.149860261	0.10958171	0.785411454	1.431953735	6.22504E-12	-0.20059457	0.874570817	-0.135236654	0.824058587	-0.508207543	0.003157967
AT3G01650	RGLG1	0.320518789	0.440960258	0.305074182	0.324654614	1.431278307	2.76453E-10	-0.649598069	0.359157502	-0.243101028	0.485857955	-0.310508379	0.042076415
AT1G68872	AT1G68872	0.671208645	0.497082719	0.148168141	0.840815416	1.426386558	0.03340879	0.134221462	0.953721764	1.062521278	0.10891184	0.064464334	0.90523233
AT3G23870	AT3G23870	4.01768032	0.166150362	-2.873300516	0.624159882	1.416429036	0.009505926	-1.190486591	0.887521452	-0.712949616	0.72447373	-0.78973287	0.315816228
AT2G01320	ABCG7	0.635839576	0.060090511	0.816134613	2.64502E-06	1.413427643	5.41282E-30	0.765427589	0.62053317	0.44839963	0.045144176	0.108734994	0.59033654
AT2G15695	AT2G15695	0.974067073	0.017438181	0.913392862	3.14574E-07	1.410803297	2.07539E-26	1.374728205	0.281913411	-0.120458803	0.777094046	-0.256467923	0.150023604
AT3G11770	AT3G11770	0.745207329	0.01683997	0.9577227	0.000228143	1.407723394	1.07568E-07	0.150667216	0.878425628	-0.119393353	0.708887279	0.02805743	0.903469307
AT5G63260	AT5G63260	0.196493646	0.765417989	0.518369866	0.049200207	1.400389217	1.55264E-20	-0.06751694	0.971723999	0.149450214	0.745877821	0.219954418	0.200050554
AT3G56450	ALPHA-SNAP1	-0.270392185	0.791840266	0.637182219	0.406703643	1.398333091	0.000370268	0.490360838	0.691824202	0.484131899	0.513381523	-0.101429353	0.782157432
AT3G47730	ABCA2	0.768540754	0.011248932	0.776598122	0.000582363	1.396359058	2.20952E-19	-0.284719091	0.706790319	-0.526092366	0.009570362	-0.885681317	7.0937E-09
AT1G77230	AT1G77230	0.634505122	0.369131153	0.822642408	0.243454356	1.393966741	0.000900323	0.204155221	0.896927975	0.081528372	0.937392682	-0.500330284	0.082134746
AT3G62310	AT3G62310	0.748925011	0.005053079	0.724580339	0.000215362	1.391596929	3.45155E-23	0.641032231	0.575257546	0.002853706	0.995352013	0.156161021	0.315507406
AT2G36350	AT2G36350	0.697838936	0.15535629	0.177529477	0.639758525	1.389560102	2.23832E-07	-0.107682125	0.908634744	0.150538497	0.646238543	0.116825486	0.669620875
AT1G51520	AT1G51520	0.89456393	0.120875263	0.646197961	0.444651421	1.368123322	0.011168737	0.107351976	0.963509602	0.066212501	0.956739399	-0.226078709	0.469478567
AT2G01450	MPK17	0.46650183	0.208847372	0.92409821	2.40779E-08	1.364694478	4.01059E-18	0.607252761	0.58116222	0.336342542	0.013828968	0.326211397	0.053406512
AT1G49832	AT1G49832	1.088756569	0.621904193	0.597617102	0.667661419	1.362329319	0.033608003	0.398555175	0.935083701	0.861251848	0.567773209	0.066080451	0.929713443
AT2G39681	TAS2	1.049542509	0.093351625	-0.622784682	0.179294655	1.360423065	3.86926E-07	0.544014542	0.593379395	0.987837235	1.11302E-05	0.432710925	0.207305165
AT4G02000	AT4G02000	-0.64681113	0.75463429	0.824093172	0.330628028	1.358488298	0.033249894	-0.428135034	0.91394044	-0.40372587	0.798599938	-0.095049012	0.909683962
AT3G06850	BCE2	0.542630882	0.166177115	0.022568089	0.955679844	1.356561409	1.18056E-12	1.796149071	0.294511423	0.266343758	0.298693478	0.14604282	0.41273591
AT4G21500	AT4G21500	0.93855697	0.178035601	1.620376047	0.087107858	1.350656487	0.004216791	-0.754524691	0.602119156	0.116178258	0.92420515	-0.504187864	0.084348139
AT1G51740	SYP81	0.617847441	0.284250772	0.845486302	0.0001743	1.350370588	8.47165E-10	0.787604633	0.62738272	0.320638776	0.375724552	0.333534638	0.076560142
AT3G12510	AT3G12510					1.347899128	0.004342228					-0.572652257	0.132153085
AT5G16500	AT5G16500					1.345384644	6.97351E-20					0.121446356	0.567172849
AT5G67520	APK4	-0.122790628	0.930035011	1.172189671	0.167930127	1.345043209	0.003352618	-0.48069009	0.880294241	-0.0210803	0.986818647	-0.102607752	0.728299066
AT3G29180	AT3G29180	0.497268243	0.101556369	0.404527444	0.031880277	1.339345071	6.3258E-19	-0.083955791	0.93556951	0.010288096	0.982346996	-0.221236504	0.164796023
AT3G62950	AT3G62950	0.602913798	0.40080392	0.069875273	0.919311145	1.336734046	3.93276E-11	6.685897587	0.079118837	0.566603179	0.739714734	-1.14599156	0.05971456
AT4G19420	AT4G19420	0.493024791	0.378814527	-0.183835361	0.526855004	1.336529754	1.03816E-15	-0.29838806	0.830747236	-0.259318753	0.769279109	-0.734267185	4.10482E-05
AT1G30040	GA2OX2	-0.320538433	0.665927557	0.426511451	0.298942593	1.335698229	2.44941E-05	-0.275112562	0.870136387	0.183724723	0.822587771	0.244797173	0.178580317
AT1G33110	AT1G33110	0.343197384	0.566512148	0.610257702	0.010355276	1.334458169	4.26207E-10	0.241063532	0.847660958	0.384569104	0.19679265	0.112342263	0.646992921
AT3G02500	AT3G02500	-0.235901726	0.840212622	0.64633707	0.516982537	1.325826985	0.00233754	0.584542444	0.693976671	0.156988932	0.920357345	-0.024176538	0.967594282
AT1G03935	AT1G03935	3.017786482	0.370574205	1.101660004	0.695864495	1.32464182	0.015362703	-0.275688989	0.979362915	0.609560089	0.848060883	-0.782072627	0.060792029
AT1G33350	AT1G33350	-0.062808158	0.967147181	0.392716339	0.721752265	1.323880927	0.026290785	0.047912486	0.982734229	-0.274875503	0.831572245	0.254654929	0.500398281
AT5G55700	BAM4	0.725192813	0.114016696	0.304367208	0.045307914	1.319149888	1.47118E-09	1.689242247	0.266872879	0.388950034	0.082999383	-0.3047536	0.170129
AT2G01940	SGR5	0.29581119	0.584395985	0.34546734	0.298942593	1.317970435	2.17944E-22	0.155327031	0.904735573	-0.157783623	0.685177794	-0.121986207	0.48338674
AT5G24460	AT5G24460	0.029043527	0.963763107	0.672755	0.032733853	1.312273065	1.29762E-07	0.608049201	0.692108738	-0.468722155	0.215575766	-0.461496217	0.006523538
AT5G41580	AT5G41580	0.459908648	0.478814145	0.903946399	0.005965081	1.311891931	2.99171E-10	0.431368516	0.70850021	0.183233116	0.805320688	0.456614127	0.02265316
AT5G35320	AT5G35320	0.885764065	0.01758289	0.904185865	0.001914836	1.310789401	1.1402E-10	0.102445612	0.949561769	0.022717792	0.976570624	-0.206584481	0.335845261
AT1G60430	ARPC3	0.858469976	0.243849775	0.849408169	0.010530034	1.309110003	2.47051E-10	0.705731829	0.587695712	-0.045071749	0.965477972	-0.154304728	0.615955027
AT3G05210	ERCC1	0.853244397	0.092654226	0.942272131	0.000837271	1.306440976	2.40347E-11	0.321795199	0.818968121	-0.278530623	0.427798338	-0.117474692	0.567528751
AT1G26580	AT1G26580	0.982989701	0.001821927	0.948363748	1.43348E-05	1.304796553	4.44471E-42	0.718032149	0.584219873	0.508963511	0.000609866	0.319172907	0.125504897
AT1G54290	AT1G54290	1.036991199	0.051642471	0.959713914	0.002248347	1.301414921	1.44772E-11	0.240707811	0.867360547	-0.326306142	0.556665874	-0.733460835	0.001531235
AT3G59270	AT3G59270	-0.392478491	0.887321068	-0.397228684	0.740575988	1.30119926	0.014299938	2.301583354	0.313973003	0.929334398	0.514480278	0.917166681	0.009472417
AT4G29380	VPS15	0.974058776	0.003011354	0.79270347	1.49486E-05	1.296811962	1.80556E-11	0.430286821	0.726635812	-0.004183147	0.994809352	-0.394883071	0.021828513
AT4G02430	SR34b	0.752359923	0.076894027	0.480064187	0.077409547	1.295253577	3.07982E-14	0.754327374	0.572525232	0.213715526	0.606116282	0.192628755	0.52586572
AT4G36850	AT4G36850	-0.063716246	0.933564753	0.484165755	0.005498464	1.290128185	2.34404E-21	2.494085125	0.076815321	0.200462144	0.412791131	-0.432988309	0.000919647
AT2G48020	AT2G48020	-0.166942077	0.785239423	0.653339915	0.046834917	1.288219134	5.01542E-19	0.316874072	0.872669739	0.300720263	0.509638584	-0.975313902	6.73806E-19
AT1G79880	La2	0.972694444	0.018324052	0.868527462	0.003104489	1.28602717	2.28277E-12	0.569183905	0.519228952	0.119197981	0.798599938	0.182237098	0.265577414
AT3G07520	GLR1.4	0.829979868	0.292882356	0.964983924	0.023305677	1.284028861	3.48565E-05	0.363378379	0.854231696	0.091721781	0.927254488	0.115251975	0.786567889
AT5G42950	AT5G42950	0.611011782	0.013949401	0.822252148	6.16547E-10	1.28248248	2.66146E-23	0.187058372	0.876015705	0.084749955	0.811243353	0.010861416	0.964161933
AT1G56170	NF-YC2					1.281716792	1.02183E-05					-0.27661429	0.16804094
AT1G50040	AT1G50040	0.314473831	0.610204677	0.010972105	0.980651529	1.281309472	1.13924E-05	1.73750889	0.429222482	0.608562595	0.245604961	-0.318812945	0.510936831
AT5G02840	LCL1	0.861539242	0.051564835	0.764013125	8.34158E-05	1.280424318	9.97713E-30	1.302415763	0.319520931	0.241123957	0.272394607	-0.172754761	0.191274428
AT3G30180	BR6OX2	0.794554373	0.010238502	0.921967473	0.000290761	1.277737396	8.24231E-25	0.037413022	0.979832545	-0.249198404	0.282272743	-0.396656465	0.00254946
AT1G27590	None					1.266436414	4.8414E-06					0.012196339	0.972028341
AT2G11270	AT2G11270	6.241727464	0.266100077	2.143455938	0.614415104	1.265667034	0.001314431	-7.343166781	0.071373	0.156310494	0.988694176	0.055130622	0.902568843
AT1G70510	KNAT2	0.255646888	0.825204264	-0.369842007	0.745209868	1.26420259	0.000386523	-0.488503997	0.697104641	0.029778089	0.97926944	-0.107798377	0.761927143
AT1G02670	AT1G02670	-1.23949007	0.147670904	-0.399675912	0.592709278	1.263970382	1.11626E-05	0.099788002	0.974385857	0.034118536	0.989503241	0.330619523	0.513146642
AT5G16120	AT5G16120	0.592838879	0.106598166	0.960346901	2.141E-07	1.261379902	4.89878E-17	0.722027415	0.565728752	0.459949919	0.001725378	-0.06366038	0.808951662
AT5G12270	AT5G12270	1.173457434	0.215245808	0.322779093	0.73674204	1.260687111	0.004613587	0.855282007	0.65378996	-0.127653689	0.934609223	0.319660398	0.465873
AT3G57040	ARR9	0.561617881	0.211683302	0.534486087	0.002492037	1.257245818	1.16122E-25	0.563996827	0.622600949	0.221285795	0.398976453	-0.910582613	8.95551E-10
AT1G60200	AT1G60200	0.880961277	0.023450489	0.945718389	7.80873E-07	1.257243616	1.29268E-28	0.851660534	0.572525232	-0.020957952	0.964180223	0.01029716	0.962284666
AT5G48655	AT5G48655	0.627421566	0.126619013	0.960725449	9.02734E-06	1.252344914	7.49113E-15	0.426408716	0.684631883	0.161553143	0.708887279	-0.078229747	0.727554925
AT5G55970	AT5G55970	0.734945913	0.120454656	0.532015891	0.080080834	1.250247401	3.16897E-09	1.603470042	0.385954218	-0.142606863	0.834008361	-0.605898868	0.053581619
AT5G46410	SSP4	0.887565255	0.002988298	0.500952488	0.054960984	1.241706115	1.24812E-18	0.641742125	0.616300683	0.228363956	0.412991167	-0.161315213	0.288941937
AT3G46670	UGT76E11					1.231230841	0.001121772					-0.675895127	0.088497217
AT5G03770	KDTA	0.450473707	0.476375946	0.26410474	0.679025682	1.230730784	1.028E-05	0.580038196	0.542342434	0.104268883	0.907678975	0.428128718	0.07342487
AT1G27300	AT1G27300	0.806619077	0.039056896	0.996147842	0.000685894	1.226405215	3.17107E-10	0.540580412	0.680482607	0.043598818	0.957156296	-0.033658709	0.913915991
AT2G47800	ABCC4	0.305004908	0.79471575	1.253759345	0.096925968	1.222138297	0.000596124	-1.403645086	0.052258989	-0.506280997	0.101685107	-0.41980309	0.026445902
AT3G61415	SK21	0.972022541	0.052106571	0.795813603	0.005319426	1.217628082	8.65648E-10	0.634393414	0.619354152	0.558872302	0.108180357	0.041525202	0.859658378
AT5G57510	AT5G57510	1.915232812	0.142856572	0.999080745	0.26465214	1.214039441	0.015339679	-1.104825464	0.685208378	0.206848028	0.964180223	2.411410055	0.070007302
AT2G26030	AT2G26030	0.835434881	0.031372601	0.777276337	0.075353185	1.212633801	6.42893E-13	0.399114577	0.758815467	0.423752208	0.172338689	0.341726248	0.195829778
AT4G00040	AT4G00040	0.08319262	0.855564757	0.407255066	0.287857287	1.212565198	0.001909169	0.613892215	0.477936046	0.149327386	0.715453459	0.181299065	0.531357944
AT5G35210	PTM	0.558172869	0.079540623	0.48459557	0.021232191	1.205960633	6.552E-21	0.72996744	0.558566659	0.053686024	0.90184129	-0.031173726	0.895344997
AT3G46210	AT3G46210	0.534992935	0.227953262	0.226618485	0.491057517	1.205031846	2.50776E-10	0.263444587	0.67890445	0.279554741	0.473771238	0.3565334	0.01071942
AT1G12240	ATBETAFRUCT4	-0.041629406	0.955497432	0.700344156	0.000788395	1.20046355	2.3718E-27	2.314494535	0.172638291	-0.171966436	0.626901279	0.641144295	6.777E-06

AT5G23700	AT5G23700	0.764201139	0.46330644	0.568362699	0.599573882	1.199465354	0.001838168	2.350287281	0.62738272	-1.91531287	0.484585201	-0.781667773	0.194826078
AT2G42330	AT2G42330	0.828968708	0.162557643	0.217248397	0.511046037	1.197404507	1.26189E-06	0.815251431	0.574062337	0.560475947	0.106911026	0.059823616	0.845269305
AT4G38480	AT4G38480	-1.16362039	0.083538352	0.013063674	0.986258404	1.197264272	7.88951E-05	-0.8142777	0.867112468	-0.406418492	0.550143794	0.176757063	0.593884794
AT2G32795	AT2G32795	-0.177266109	0.831400281	0.563564624	0.442637915	1.197189583	0.000216335	0.691723275	0.601182558	-0.295775322	0.825549954	-0.427529027	0.255308217
AT4G20850	TPP2	0.880180037	7.41777E-05	0.743380563	4.07038E-08	1.196560322	7.32978E-24	0.438914627	0.66808962	0.104581119	0.682180924	-0.296658495	0.059219858
AT1G16820	AT1G16820	1.206518879	0.26903587	-0.214901956	0.873167092	1.188171532	0.00028047	0.436779533	0.80338535	1.174956533	0.563104532	0.370287586	0.429684088
AT1G09910	AT1G09910	0.568776886	0.151580885	0.914411581	2.72514E-06	1.185437671	0.000101112	0.886898417	0.541068634	0.021094645	0.979610562	0.107647303	0.761157425
AT5G53730	AT5G53730	0.692983183	0.292519146	0.599067158	0.260271918	1.184325677	0.001279972	-0.357761544	0.784226966	-0.101926214	0.883566526	-0.869759224	6.47265E-07
AT3G07215	AT3G07215	0.714390872	0.404410007	0.773588983	0.090058974	1.181874682	3.56524E-08	0.295535571	0.828875452	0.43497107	0.279139044	0.200695755	0.653449905
AT5G13010	EMB3011	0.796077936	0.006465318	0.732598911	2.74716E-05	1.180564665	5.40445E-23	0.499739586	0.68625412	-0.043660153	0.920286072	-0.410361551	0.004974465
AT1G21580	AT1G21580	0.46812809	0.099072071	0.187939391	0.463011914	1.179144572	6.35353E-15	0.594517519	0.640704059	0.014440249	0.981554934	-0.197152351	0.191741929
AT5G66030	ATGRIP	0.854232071	0.001654201	0.583967113	9.82817E-05	1.178687576	1.18178E-28	0.301401558	0.67527834	0.068737608	0.802042097	-0.286668025	0.054438767
AT5G24350	AT5G24350	0.59015831	0.11008047	0.318959579	0.267584542	1.177753338	3.19616E-08	-0.009022446	0.994550104	-0.226663816	0.518256458	-0.307599414	0.074839881
AT3G06210	AT3G06210	0.461652969	0.422777576	0.53330176	0.029531675	1.175214443	8.28976E-07	0.587870221	0.647947756	-0.290364886	0.511821543	-0.595079194	0.006303365
AT1G18745	AT1G18745	0.025172194	0.991544266	-0.262825456	0.923246481	1.166124195	0.000304987	-0.764182711	0.866579819	0.302562384	0.879590575	0.189677586	0.510245115
AT3G22960	PKP-ALPHA	0.995345206	0.000610581	0.579515683	8.29291E-07	1.165422168	2.95458E-17	0.94892529	0.478067535	0.187494981	0.242591101	-0.132982596	0.376793342
AT4G21450	AT4G21450	0.867623316	0.00988566	0.77666341	6.61294E-09	1.163067202	1.60042E-16	0.615093951	0.615186535	0.177617676	0.403366939	-0.155918172	0.264704207
AT3G07420	NS2	0.538718618	0.429485814	-0.041055887	0.95817051	1.160807287	0.000469235	-0.053898815	0.982356953	-0.754719912	0.046522241	0.402787259	0.049365129
AT1G27752	AT1G27752	0.791478039	0.008093667	0.47074595	0.012969628	1.160767067	3.25896E-09	0.036761562	0.980475256	0.142049053	0.714322744	-0.324544316	0.025799271
AT1G62310	AT1G62310	0.346711506	0.52768402	-0.227311688	0.451393009	1.158053571	9.72326E-07	0.729202477	0.541116454	0.108042939	0.825713105	0.012019716	0.965679406
AT1G21610	AT1G21610	0.421135973	0.311875314	0.611728114	0.002063188	1.157107722	2.28423E-13	1.139273867	0.558566659	-0.04985434	0.914915058	0.068209724	0.758114758
AT4G21110	AT4G21110	0.933940475	0.009876491	0.982043638	1.88283E-08	1.156389395	1.78178E-14	0.530609347	0.581277369	0.288639186	0.245128903	0.119969817	0.487681047
AT4G38250	AT4G38250					1.155551617	9.731E-10					-0.495238401	0.00018077
AT1G27520	AT1G27520	0.673092108	0.073931482	0.694065636	0.005758663	1.154857523	5.32808E-13	0.933746829	0.50075057	0.115795046	0.812750957	0.184762227	0.32279546
AT1G66050	VIM2	-0.787205121	0.565500096	-0.552820221	0.630179527	1.153045021	0.038703654	-0.715911442	0.685495817	-0.724439515	0.564980934	0.299022522	0.576882241
AT1G55920	SERAT2;1	0.83839497	0.017433248	0.743316715	0.010714705	1.151859441	2.39122E-07	0.592385874	0.568009156	0.309566546	0.317458952	0.119210968	0.54011105
AT4G39890	RABH1c	0.080758581	0.925931189	0.05102192	0.951433959	1.151755876	0.048447769	0.500529092	0.731041294	-0.220445998	0.863960047	0.455157985	0.167909104
AT1G70590	AT1G70590	0.758831094	0.050372852	0.606881057	0.111599384	1.151566886	1.66112E-13	0.612326972	0.685495817	0.279428317	0.467546241	0.055479113	0.859856058
AT3G27260	GTE8	0.812826503	0.011613693	0.935622599	2.38258E-09	1.146134315	9.49722E-21	0.654036646	0.665778009	-0.201523958	0.428068121	-0.242133514	0.135250345
AT2G04160	AIR3	-0.363662819	0.534272697	0.518432214	0.17451503	1.14561506	2.13179E-05	-0.310009625	0.767987589	0.211880812	0.800469173	-0.089454429	0.839187777
AT4G36980	AT4G36980	0.543629497	0.102646864	0.68178898	0.000152102	1.144634689	1.31484E-11	0.740212575	0.517612286	0.171433965	0.558444545	0.258857539	0.092945148
AT1G07480	AT1G07480	0.884342713	0.027358491	0.698933139	0.063943712	1.141506574	9.63846E-13	-0.001770048	0.999311751	0.084950321	0.908594277	-0.304750776	0.048235849
AT5G64680	AT5G64680	0.705458238	0.137755488	0.268597062	0.481302528	1.140985932	8.16075E-08	0.435768333	0.589112149	0.114273878	0.827003175	-0.006138798	0.980118833
AT4G21710	NRPB2	0.605402734	0.011100351	0.480914456	0.004482885	1.139181343	6.41279E-18	0.565787422	0.584219873	0.113277783	0.707829653	0.10457832	0.552877675
AT3G09840	CDC48	0.908492409	9.66534E-06	0.923042391	7.12447E-19	1.136489176	7.83272E-27	0.343620876	0.726934566	0.053786329	0.831600439	-0.190675761	0.186318194
AT1G70620	AT1G70620	0.149080331	0.730518191	0.086106628	0.682866594	1.135672367	3.11203E-12	0.806686121	0.448889099	0.133780529	0.655417993	0.287233592	0.012965534
AT3G41979	AT2G01020					1.133701356	0.016892978					0.786768748	0.007379243
AT3G15290	AT3G15290	0.775464235	0.023455142	0.93206918	0.000233973	1.132158005	6.34554E-08	0.366426649	0.721579398	0.266400854	0.434411297	0.063412206	0.867604777
AT5G58730	Mik	0.967843107	0.087683454	0.418963235	0.442637915	1.12754007	0.005224404	-0.436384432	0.66524984	0.098333049	0.897458174	-0.661378618	0.00133143
AT5G05750	AT5G05750	0.68163027	0.025573709	0.757167718	3.87961E-05	1.126217191	3.57885E-10	0.471766205	0.618907586	0.26522561	0.382371301	-0.720836266	1.50804E-07
AT1G55590	AT1G55590	0.4525123	0.346683514	0.59228004	0.012981989	1.125790928	8.68665E-08	0.275197189	0.845839675	0.140479845	0.760117948	0.028923805	0.920529379
AT1G79610	NHX6	-0.128014285	0.878809942	0.620401342	0.362734137	1.123993434	0.031048019	-1.256609247	0.300378357	-0.106980131	0.913946561	-0.577323179	0.001795748
AT4G24230	ACBP3	0.241994442	0.676533746	0.321848465	0.129757572	1.123417097	4.32602E-25	1.702001061	0.313609174	0.195815297	0.541137876	-0.30471436	0.288179519
AT5G61530	AT5G61530	0.874675215	0.003980013	0.772287911	0.000924591	1.123251487	6.59176E-08	0.32388578	0.653363031	-0.161792345	0.571318331	-0.38604925	0.017381723
AT1G18450	ARP4	0.791306703	0.001440541	0.795200107	0.000161484	1.122582383	1.28249E-15	0.72241039	0.387045614	0.229930192	0.258510236	0.307416249	0.009041656
AT1G01670	AT1G01670	-0.071231283	0.967108711	0.094942368	0.972706681	1.1215534	7.75478E-05	0.047318693	0.9905016	0.043596497	0.987310107	-0.561909205	0.009920837
AT5G25560	AT5G25560	0.762298817	0.010133096	0.550369472	0.018025772	1.119555743	9.2776E-16	0.68903805	0.422258087	0.287646214	0.198938844	0.289211512	0.072370541
AT4G36050	AT4G36050	0.883535161	0.107185623	0.628481765	0.198074732	1.118610999	5.76403E-05	0.625523447	0.62738272	0.221135158	0.753699142	-0.0854719	0.815256026
AT3G51090	AT3G51090	0.724045303	0.287320311	1.124422106	0.081980865	1.118383401	0.049760855	0.027718617	0.989481773	0.15091356	0.870101981	-0.021261001	0.962335808
AT4G12570	UPL5	0.716842482	0.008547328	0.055797561	0.841362436	1.116782539	1.7567E-11	0.643602385	0.539736869	0.055383681	0.90438859	-0.27115504	0.072856276
AT3G26100	AT3G26100	0.762127313	0.00712963	0.642543873	0.009428808	1.115832356	6.41608E-22	-0.173914551	0.798808149	0.053859761	0.878562017	-0.391253702	0.040480506
AT3G07650	COL9	0.615784323	0.203003501	0.68756528	0.002282358	1.115087524	0.000763318	-0.468295309	0.605522309	-0.127068014	0.770526843	-0.05109995	0.807151807
AT2G46200	AT2G46200	0.386038589	0.468001732	0.735995952	0.014497278	1.114523221	4.10133E-06	0.040910806	0.978451523	-0.180975094	0.753902445	-0.415167207	0.024262621
AT3G61420	AT3G61420	0.552354271	0.193213628	0.916624716	0.002492037	1.113158158	1.39603E-10	1.133613862	0.505950107	-0.04090124	0.944351305	0.252657504	0.161879447
AT4G34412	AT4G34412	0.555057177	0.301936662	0.807763432	0.159850821	1.111036784	0.00186527	0.316117694	0.769696781	0.044527488	0.963419708	0.44975307	0.056793175
AT2G02570	AT2G02570	0.944438084	0.003183592	0.78429275	0.001623525	1.109929435	7.68523E-13	0.920251229	0.379538351	0.176053655	0.524173345	-0.039216526	0.895783536
AT4G18120	None					1.109818625	5.2373E-13					0.193282173	0.281271757
AT2G41530	SFGH	0.684918838	0.047380565	0.743504653	0.000485688	1.109803819	1.63036E-11	0.148804342	0.828875452	0.01426359	0.980633826	0.428069462	0.032496771
AT5G19330	ARIA	0.643393697	0.009305399	0.324830335	0.119234331	1.108731862	8.93071E-11	0.418880347	0.653363031	0.151147077	0.540482493	-0.117095653	0.396520133
AT4G36690	ATU2AF65A	0.448421865	0.127892435	0.306072163	0.081976962	1.108451058	1.11599E-16	0.680948631	0.542178894	0.230117122	0.378030276	0.162578836	0.41343626
AT1G74456	AT1G74456	0.129821447	0.949533165	0.419631965	0.797276875	1.107711261	0.024226757	-1.276118832	0.765335087	0.09799292	0.973216634	0.289643109	0.583297417
AT5G64270	AT5G64270	0.660946432	0.0097857	0.701171394	8.49608E-09	1.105981198	1.57352E-17	0.480974876	0.66808962	0.031911461	0.940108495	-0.208558288	0.189572027
AT5G47040	LON2	0.28208404	0.5321325	0.700862434	7.57122E-05	1.105623043	2.51457E-29	0.972450548	0.588156522	0.022075991	0.96106411	-0.182372583	0.28720387
AT3G54500	AT3G54500	-0.069565718	0.926875823	0.46261997	0.087478397	1.103426036	6.85999E-11	2.410583521	0.086459345	0.605183158	0.000977815	0.43033683	0.000519893
AT5G49650	XK-2	0.723365934	0.026944333	0.706633043	1.83616E-05	1.102614719	6.42893E-13	0.462128082	0.62738272	0.222839633	0.484227201	0.152838401	0.512034786
AT3G13460	ECT2	0.776335671	5.48764E-05	0.344997147	0.026212788	1.101976868	5.44097E-25	0.303428983	0.680248595	0.227940954	0.068183619	-0.301201703	0.00779651
AT3G54380	SAC3C	0.762489273	0.022078954	0.899673652	0.001811802	1.100363078	1.28556E-10	0.344451745	0.646729152	0.232904303	0.499031618	-0.117094318	0.620205636
AT5G11600	AT5G11600	1.067195724	0.065683943	0.799060417	0.015772692	1.096047516	3.90804E-05	0.759869015	0.640716302	-0.126067677	0.84694947	-0.308733442	0.345593825
AT1G50630	AT1G50630	0.951700045	0.01715368	0.429757343	0.306035027	1.095123194	0.00019344	-0.878417349	0.379538351	-0.640144917	0.013382132	-0.995796919	3.2989E-12
AT1G30410	ABCC12	0.647770318	0.077274725	0.471927388	0.141618385	1.094056025	2.07992E-05	0.30363279	0.697914029	-0.029116062	0.963495178	-0.001845881	0.995307161
AT2G01830	WOL	1.461710312	0.073234076	0.008759279	0.989804887	1.09113069	9.28363E-05	-0.712571639	0.598901706	-0.414228746	0.085764383	-0.884559655	2.14371E-11
AT3G60300	AT3G60300	0.933854801	0.000171107	0.620781833	2.99717E-06	1.088723795	1.7211E-10	0.403242503	0.611321113	0.20841842	0.329008218	-0.301924553	0.00637671
AT3G42640	HA8	0.802480705	0.115078169	0.372239121	0.357008311	1.087129829	6.47043E-09	0.54191327	0.58097193	0.131676009	0.792838833	0.330955555	0.117880697
AT5G14170	CHC1	0.498213576	0.117192805	0.337674095	0.027065353	1.085053409	2.43137E-15	0.51675728	0.653363031	-0.01280973	0.98143759	0.11756295	0.516832204
AT3G53540	TRM19	0.585235833	0.110800017	0.27692004	0.162449403	1.084696741	1.62653E-14	1.11093881	0.383660928	0.247830529	0.469904353	0.413197218	0.014404104
AT4G36648	AT4G36648	0.438986536	0.360165821	0.213726826	0.350415728	1.084363748	7.84422E-12	-0.027242146	0.982734229	0.378039701	0.05125618	-0.40274711	0.035259647
AT5G17440	AT5G17440	0.903319867	0.001585442	0.920705205	3.71888E-07	1.08415357	7.07697E-10	0.502232044	0.672844426	0.258755211	0.442923576	-0.288513118	0.097622952
AT1G69220	SIK1	0.301645244	0.324052858	0.418637529	0.090464382	1.080442429	1.64093E-16	0.131419043	0.907707664	0.016757525	0.976380404	-0.170030783	0.270365533
AT4G25710	AT4G25710	0.840547811	0.11990625	-0.221855798	0.636843204	1.079750379	0.000271886	-0.447844762	0.694819594	-0.07400785	0.906137209	-0.140922159	0.608979536
AT5G47430	AT5G47430	0.58367867	0.0853672	0.97469592	1.04611E-05	1.079389518	2.76494E-23	0.297647265	0.766336214	0.177588467	0.524173345	-0.125463847	0.456775466
AT1G24180	IAR4	0.806073524	0.020181017	0.581760529	0.045413176	1.078531245	1.42407E-13	-0.290627182	0.637815859	0.158538717	0.568939289	-0.283320965	0.065468767
AT2G05270	AT2G05270	4.545921689	0.119438257	7.163718334	0.163894042	1.077131779	0.006022798	-6.191476273	0.611321113	-6.372561228	0.206811617	-0.052440859	0.914022828
AT5G04600	AT5G04600	0.903633734	0.000228456	0.684881246	0.000411719	1.076173403	3.87224E-13	0.324766384	0.6657277	0.224584814	0.258531028	0.418280855	0.000235074
AT3G05250	AT3G05250	0.591564167	0.279837848	0.962665085	0.001397089	1.074874998	0.000358835	0.444522618	0.784817453	0.449355853	0.315425441	-0.160590569	0.509365018
AT5G47830	AT5G47830	0.679703803	0.156505624	0.927992779	0.01499151	1.07322342	9.449E-05	0.072971516	0.964220939	0.404836657	0.331240352	0.275155403	0.134205794
AT1G02370	AT1G02370	0.504417539	0.462794455	0.301979039	0.608608769	1.072250861	0.021256114	0.120596602	0.907707664	0.046592584	0.963411941	-0.309695657	0.135953173
AT5G50780	AT5G50780	0.862122775	0.08248012	0.603817593	0.120963336	1.071764911	4.78114E-05	0.652212117	0.691895802	-0.187226798	0.71169254	-0.577441367	0.007446695
AT1G73980	AT1G73980	0.57841333	0.135566426	0.924350248	9.70766E-06	1.067955006	9.35687E-09	0.444018184	0.726900342	0.005518424	0.992519652	0.269282819	0.116347503
AT2G25760	AT2G25760	0.765689397	0.006426058	0.723366031	0.000332063	1.065560689	3.41058E-13	0.233634754	0.802172683	-0.115284402	0.660782083	-0.269726516	0.046175064
AT3G15850	FAD5	0.555768793	0.228161487	0.373162189	0.019508264	1.063489204	2.62538E-21	1.356685366	0.405880771	0.154726968	0.603941128	-0.260767116	0.056531119
AT5G09330	NAC082	0.131668697	0.767687451	0.180465971	0.362707033	1.062980998	2.68329E-14	-0.090814469	0.907100964	-0.108149371	0.730231	-0.162235288	0.406960376
AT5G15330	SPX4	0.866996401	0.044614046	0.858408941	2.47112E-05	1.062928506	4.17134E-08	0.217019858	0.919978446	0.308859282	0.487031589	0.424915415	0.049769039
AT5G42520	BPC6	0.769651484	0.008810618	0.665686362	0.002066239	1.061265158	3.5788E-11	0.326795846	0.734168325	0.014184869	0.975427751	-0.258150619	0.040544985
AT4G21660	AT4G21660	0.991456414	0.002176909	0.707884199	4.10613E-06	1.057193048	2.80598E-22	0.572047191	0.602864055	0.138728862	0.595418694	-0.06014975	0.76995062

AT1G03210	AT1G03210	0.585956973	0.206970805	-0.283770254	0.644039825	1.057125435	0.002587922	0.05737995	0.978807026	-0.183408721	0.727083628	0.09333144	0.734055256
AT1G74910	AT1G74910	0.249893378	0.565695419	0.582406924	0.000107719	1.054159164	7.79472E-22	0.761838458	0.62738272	0.05563629	0.880097385	-0.032027271	0.852575771
AT5G59080	AT5G59080	0.5361199	0.334009946	0.733020606	0.000269614	1.05313027	2.66464E-18	1.576294612	0.309698198	0.473286906	0.057036111	-0.617452111	0.000193472
AT5G05690	CPD	0.826926877	0.021647296	0.974059272	3.5538E-10	1.053107629	8.83854E-17	1.076258352	0.514379829	0.040791296	0.900466581	-0.308350847	0.031751371
AT2G02770	AT2G02770			-2.175702898	NA	1.05307005	8.64522E-06					0.52622982	0.003079486
AT2G46900	AT2G46900	0.767532961	0.004201508	0.602703537	0.000241367	1.052347317	1.16573E-09	0.522593087	0.616300683	0.165832226	0.507785667	-0.022338833	0.912018611
AT1G76490	HMG1	0.963303759	0.000108553	0.644315278	5.7557E-06	1.052245204	1.8944E-18	0.457936982	0.709790565	-0.100541498	0.652699183	-0.436168261	1.13683E-05
AT3G15010	AT3G15010	0.397084614	0.340228105	-0.036221098	0.915911242	1.050305327	1.95622E-10	0.452898886	0.663725004	0.610603386	0.001771424	0.007848427	0.980063892
AT5G43100	AT5G43100	0.807630908	0.013101154	0.596921843	0.01031444	1.050104037	8.28833E-08	0.300828648	0.776796299	-0.117412171	0.763639797	-0.40611487	0.013743885
AT3G57410	VLN3	0.375112742	0.200567197	0.587324732	9.45101E-05	1.04968715	6.95621E-22	0.1904749	0.851449159	-0.162393012	0.447158692	-0.233091993	0.076510929
AT3G53030	SRPK4	0.7603894	0.044949109	0.530700417	0.062104785	1.049577254	2.91746E-09	0.610593021	0.656079416	0.082361375	0.893802094	-0.22527193	0.209193967
AT3G59940	AT3G59940	3.431704201	NA			1.048627549	4.49895E-30					-0.076457635	0.675481185
AT1G05840	AT1G05840	0.614510123	0.112370972	0.559509064	0.00250564	1.047811902	8.35749E-09	0.51037045	0.66808962	0.152897925	0.628323907	-0.315131696	0.033306433
AT2G34750	AT2G34750	0.431209018	0.126466505	0.440750282	0.010149836	1.047311907	1.07895E-12	0.25409041	0.691895802	0.163086719	0.491449838	-0.059452505	0.77280831
AT3G45730	AT3G45730	0.707308202	0.270597653	0.386473643	0.294560671	1.047181283	0.000614583	2.13794663	0.1476628	-0.17555966	0.762855447	-0.640400925	0.104344277
AT1G11400	PYM	0.674352804	0.06810495	0.380103731	0.10703869	1.046107112	4.17921E-09	0.407101718	0.676109289	0.313505318	0.170461792	-0.0993044	0.577297199
AT5G25800	AT5G25800	0.379718595	0.648981931	-0.098015862	0.898319385	1.0460069	0.000888596	0.080274932	0.95196005	0.132873665	0.793774805	-0.355702097	0.083313665
AT3G01850	AT3G01850	0.317668946	0.368239227	0.32496154	0.169984007	1.044968644	6.90211E-10	0.501643762	0.647947756	0.028936836	0.962637134	-0.002591532	0.990657871
AT5G24740	AT5G24740	0.37927106	0.333922614	0.328804692	0.20246594	1.044805219	1.52291E-06	-0.088158657	0.932578056	0.115126213	0.790326516	-0.0412837	0.877319097
AT2G38185	APD1	0.132710904	0.906772678	0.910103208	0.076483553	1.043511942	0.005647242	0.547727766	0.695103554	0.065421122	0.958669515	0.263591624	0.45640227
AT2G18760	CHR8	0.752153723	0.208060355	0.420243473	0.251614904	1.042334452	9.87551E-05	-0.02424997	0.989159859	-0.193049618	0.738491049	0.115550193	0.752788409
AT2G27950	AT2G27950	0.572107214	0.105152358	0.618219828	0.004190525	1.041611812	2.30038E-08	0.1685813	0.818968121	-0.134936468	0.765384319	-0.380688596	0.018815716
AT2G04550	IBR5	0.792376208	0.051712702	0.574567582	0.00393958	1.040989566	1.34018E-09	0.746944049	0.626315677	-0.007222928	0.992575938	0.093880989	0.643400911
AT1G09770	CDC5	0.854761055	0.000217787	0.497423187	0.002293096	1.038188339	4.75185E-16	0.455890336	0.657369817	-0.029785938	0.938376901	-0.160025542	0.344403125
AT5G09880	AT5G09880	0.594945615	0.085536767	0.461471218	0.045923317	1.036841159	1.01381E-11	0.284699316	0.807972118	0.170810583	0.501063227	-0.059317764	0.824403551
AT2G45660	AGL20	-0.601140558	0.060837863	-0.069407208	0.842903971	1.036812696	3.61305E-12	0.392717115	0.8397484	0.172327279	0.780514837	-0.315968714	0.138963508
AT5G17980	AT5G17980	0.010822395	0.989956248	-0.767024933	0.559158239	1.036746717	0.02011586	-1.189404887	0.221083366	-0.070814334	0.946159599	-0.589922034	0.009002755
AT5G25060	RRC1	0.801477948	0.001959799	0.859234535	7.0688E-07	1.035692704	2.62586E-14	0.529149388	0.679462719	-0.032886134	0.942839979	-0.061913725	0.794463934
AT3G59770	SAC9	0.768037967	0.012351338	0.751681261	2.31941E-05	1.034547881	2.04019E-14	0.12800178	0.932561267	-0.058266522	0.865593881	-0.257718592	0.142703223
AT4G21550	VAL3	1.253736019	0.069329161	0.65340273	0.359156795	1.034436294	0.02682447	0.819798336	0.618907586	1.000062916	0.083002578	0.98215564	6.44236E-05
AT3G06080	TBL10	0.298790852	0.598766798	0.238877622	0.24561455	1.034260267	7.22521E-27	1.289144609	0.465456099	-0.221231468	0.495989166	-0.595524031	0.00275777
AT1G50200	ALATS	0.624833124	0.01040358	0.551394615	7.65296E-07	1.03364084	2.4138E-29	0.428622293	0.678433242	0.103220395	0.708747273	-0.045395685	0.828383646
AT5G14500	AT5G14500	0.723936807	0.068398694	0.797285693	0.062380351	1.032340717	6.19965E-06	0.509291681	0.459472858	0.236879561	0.606447207	0.12910466	0.644690125
AT2G41500	LIS	0.638300024	0.010278568	0.599021164	0.009159661	1.032164953	1.63166E-10	0.385098281	0.565728752	0.148816001	0.590113348	0.359119769	0.003527971
AT2G02060	AT2G02060	0.717481448	0.250896796	0.529434182	0.191831144	1.030668722	0.000537758	1.157431409	0.465456099	0.508482081	0.493493798	0.917274578	0.000995807
AT3G11720	AT3G11720	-0.525833906	0.389794757	-0.0890368	0.910046768	1.030443742	9.98454E-05	-0.014705421	0.996799452	-0.290180026	0.542006423	-0.390397243	0.082158357
AT3G52420	OEP7	0.46296709	0.54624231	0.841657375	0.10894911	1.030070552	0.042893188	-0.349645012	0.744391446	-0.119272415	0.903473685	-0.307213597	0.347673603
AT5G14390	AT5G14390	0.726172969	0.003000108	0.578736267	0.000762831	1.029889503	7.42825E-08	-0.216589945	0.712887303	-0.061191701	0.831572245	-0.499601522	3.50467E-06
AT2G45380	AT2G45380	0.575261578	0.038972284	0.846226842	0.000312724	1.026404855	2.43137E-15	0.234699425	0.704225272	0.142032053	0.61159811	-0.141290765	0.527525696
AT1G10610	AT1G10610	0.734991792	0.096433173	0.952054618	0.006233765	1.022155203	7.41092E-06	0.180031734	0.924076399	-0.204417192	0.666285275	-0.473146816	0.037097744
AT4G35785	AT4G35785	0.556579418	0.0722803	0.553308244	0.007754876	1.021303206	1.4217E-05	0.494786041	0.602119156	0.078888689	0.817744169	0.34904534	0.005970878
AT5G23450	LCBK1	0.739388461	0.012376278	0.822036193	1.06772E-08	1.01986576	1.01821E-15	0.441037435	0.726257149	0.016417496	0.968232343	-0.305381355	0.045184152
AT5G15550	AT5G15550	0.695377825	0.047237683	0.454432132	0.178286659	1.019722843	6.86111E-07	0.082790458	0.918503553	-0.008540793	0.988694176	0.311174524	0.066278208
AT2G15790	SQN	0.67630179	0.357820627	-0.563051764	0.349174089	1.018983901	0.012173339	-0.025936094	0.994162268	0.471638688	0.243652435	0.129492358	0.687276721
AT2G17930	AT2G17930	0.191025831	0.651781434	0.46757841	0.06056996	1.0188079	1.38012E-12	0.093337252	0.932705464	-0.174013366	0.548231503	-0.11225908	0.648632942
AT4G26190	AT4G26190	0.618488919	0.118202068	0.476153163	0.026820633	1.016943014	1.66156E-10	0.174208888	0.869369848	-0.063351928	0.895021414	-0.373555259	0.022567983
AT3G15400	ATA20	1.000630847	0.226255768	0.205013293	0.846397862	1.016616299	0.018838399	1.65971915	0.379538351	0.017907585	0.992611192	0.554171124	0.091589996
AT5G64200	SC35	0.934494934	0.000685486	0.91716105	7.01539E-11	1.015638134	4.35963E-10	0.424361924	0.667958595	0.042411446	0.918831356	0.107170045	0.601258629
AT4G37480	AT4G37480	0.9077827	0.010742343	0.897749447	0.000257035	1.015442867	7.69478E-06	1.116716239	0.419304064	0.250007374	0.56473855	-0.274274214	0.180254399
AT3G44330	AT3G44330	0.5193275	0.063773627	0.400286272	0.042649977	1.014231935	1.74612E-15	-0.063502208	0.945609936	0.140242353	0.556988648	0.265698966	0.03007083
AT4G16892	AT4G16892	0.869130082	0.626732032	0.605432754	0.814761995	1.013426037	0.004447019	-0.871780089	0.691824202	-0.162174262	0.930193157	0.345919495	0.147542107
AT5G56900	AT5G56900	0.556973859	0.093351625	0.808753139	0.000683898	1.013102544	3.61305E-12	0.361076861	0.731041294	0.223712302	0.447139821	-0.110273142	0.623120343
AT1G30970	SUF4	-0.046372939	0.936496305	-0.120448108	0.766576792	1.013048127	7.89943E-06	0.702069221	0.611226747	0.097863225	0.859291563	0.069629787	0.751055607
AT3G56960	PIP5K4	0.756953035	0.441909447	0.914929086	0.129590004	1.012677064	0.007192622	0.38843086	0.746623995	0.299819789	0.613407287	0.358585862	0.069039599
AT5G58410	AT5G58410	0.933321453	0.006411227	0.646726086	0.045404079	1.009294582	1.85265E-07	0.110405922	0.886611043	0.080962469	0.822224461	-0.045129561	0.849578852
AT5G14080	AT5G14080	0.810363692	0.060216927	0.376435754	0.236827839	1.00795368	0.000256371	0.276877904	0.71427218	0.248299777	0.554658146	-0.111873973	0.627545411
AT3G53500	RS2Z32	0.831585827	0.002867861	0.888680479	3.67203E-13	1.007399552	2.09307E-20	0.603508049	0.606948021	0.225146102	0.345320512	0.384919966	0.001136875
AT3G02300	AT3G02300	0.277937826	0.498459218	0.549479575	0.068171041	1.007102808	1.40636E-16	0.270637884	0.787239413	0.420025199	0.056949658	0.28917093	0.180855906
AT5G03830	AT5G03830	0.05126578	0.956764347	-0.487277486	0.369235077	1.007046947	1.55776E-07	1.268309197	0.180725221	0.726249721	0.1172018	0.72650585	0.000465972
AT3G07273	AT3G07273	1.2959694	0.176531339	0.975427817	0.104034047	1.006198716	1.03464E-09	0.663324061	0.685495817	0.131713295	0.868646909	-0.407679723	0.017833006
AT1G65580	FRA3	0.83656892	0.003004341	0.879756491	3.30258E-08	1.004063658	9.86403E-08	0.426014542	0.731041294	-0.034924796	0.952031435	-0.416157911	0.020006822
AT5G11170	UAP56a	0.439548988	0.059322185	0.643850472	0.00016588	1.004023719	2.1716E-17	0.368218986	0.598901706	0.382255725	0.004733845	0.443873349	1.39603E-05
AT2G30260	U2B"	0.992505916	0.001891084	0.882155723	1.96034E-08	1.001651947	1.10579E-10	0.758259576	0.558018169	0.17611517	0.499171407	0.358923057	0.032216278
AT2G28290	SYD	0.393886141	0.246005045	0.464464109	0.053816133	1.000613011	5.70946E-20	0.417085813	0.691824202	-0.014716323	0.976497975	-0.068055899	0.797993405
AT1G76260	DWA2	-0.783015678	0.206331927	-1.14355527	0.156807295	-1.000483511	0.049504141	-0.743129378	0.751824543	0.035150795	0.968495475	0.384043134	0.032951884
AT5G16000	NIK1	-0.142075321	0.75793169	-0.955395278	8.02158E-06	-1.000771929	8.47574E-05	-0.132239093	0.907765638	-0.086846734	0.840107151	-0.298795125	0.018014611
AT4G32700	AT4G32700	-1.274193283	0.133856754	-1.406771324	0.09690821	-1.002828813	0.049141005	-0.416556672	0.847546649	-0.302442755	0.562199149	0.133245509	0.756224353
AT1G31070	GlcNAc1pUT1	-0.264121354	0.776909888	-0.695580858	0.097786572	-1.003825758	0.00849573	-0.366012997	0.809924196	0.048382358	0.927080141	0.313187437	0.026503114
AT2G14750	APK	-0.587170755	0.284122008	-0.160958723	0.77014218	-1.003842991	0.00273282	-0.088315347	0.980444746	0.026559849	0.969038524	0.046538375	0.862827603
AT4G30530	GGP1	-0.407594778	0.302887171	-0.15235982	0.702143888	-1.005289141	2.76234E-07	-0.054076456	0.982749202	-0.115898879	0.846978493	-0.192291846	0.261510792
AT1G05690	BT3	-1.336161988	0.143817357	0.092544687	0.917370859	-1.007871803	0.019020286	0.407411037	0.958708047	0.276077423	0.915888621	-0.929578597	0.130652247
AT1G60000	AT1G60000	-0.922217184	0.001806505	-0.547025163	0.05438301	-1.008228683	1.28846E-07	0.391226398	0.646729152	0.343925713	0.438335545	0.661240006	4.59261E-06
AT3G53700	MEE40	-0.006524664	0.99256625	0.25939476	0.508023536	-1.009417223	0.000719861	-0.026638374	0.990938961	0.141363018	0.855204799	0.175914694	0.591135097
AT2G37500	AT2G37500	-0.877832223	0.056048205	-0.527125402	0.069630327	-1.009759166	1.34044E-05	0.000147336	0.999867318	0.342042905	0.186598083	0.651366878	1.00195E-06
AT4G01700	AT4G01700	1.897421955	NA	1.158308545	0.34160045	-1.011166572	0.032486602	-0.019832909	0.997385614	-0.946202997	0.375724552	0.003880518	0.993208295
AT1G56340	CRT1a	-0.715147849	0.126317139	-0.842063383	0.000274283	-1.011396568	1.41008E-09	-0.504771843	0.721140128	-0.28410986	0.088342315	0.001173431	0.99534153
AT1G74100	SOT16	-0.913582915	0.021843539	0.094026098	0.883207332	-1.011895717	0.000491025	-0.135443879	0.930880201	-0.330073156	0.244409692	-0.245061889	0.129212098
AT1G72480	AT1G72480	-0.403528078	0.620914794	-0.902579708	0.02022765	-1.012473851	0.008902638	-0.228748679	0.910428643	-0.127942964	0.772354903	0.150752852	0.440082254
AT1G07790	HTB1	-0.441254625	0.318296183	-0.481303404	0.098603716	-1.014133445	4.65838E-08	-0.149481748	0.936758504	-0.074323861	0.895436863	0.098455912	0.641667287
AT4G32350	AT4G32350	-0.562107367	0.236926543	-0.720412006	0.21424319	-1.014533355	0.002874818	-0.123467104	0.922389968	-0.167150589	0.763413934	-0.505965691	0.010916746
AT2G22870	EMB2001	-0.089420758	0.90017624	-0.80576291	0.079618487	-1.015099738	0.002091535	0.114991242	0.953045007	0.084691372	0.921538071	0.290334353	0.283282445
AT1G28340	RLP4	-0.974493897	0.000797305	-0.705712964	0.017641685	-1.015919966	4.98534E-05	-0.489899985	0.619462504	-0.502456319	0.043902091	0.027206567	0.906712049
AT4G15430	AT4G15430	-1.234413717	0.145218881	-1.237770267	0.103831706	-1.016163227	0.016417215	-0.40652617	0.651780566	-0.541336484	0.43672083	-0.014152087	0.970042231
AT3G48350	CEP3	-0.938064263	0.436260106	-0.404585634	0.648074217	-1.01693026	0.00781512	-0.730354016	0.616300683	0.122691501	0.921030796	-0.999223812	0.000166649
AT3G48500	PDE312	-0.336597611	0.569701221	-0.292359197	0.532813361	-1.017407194	2.34255E-07	0.138218286	0.899191689	0.126178518	0.732724552	0.41355244	0.002317231
AT5G40140	AT5G40140	0.207851967	0.842124815	-0.840372692	0.151152225	-1.017739688	0.040260336	-0.995454264	0.637815859	0.357236996	0.4554526	-0.461929553	0.021173701
AT2G40280	AT2G40280	-0.539230723	0.250896796	-0.38635907	0.389275159	-1.021969744	0.000114804	-1.306422284	0.181549308	-0.394480949	0.243652435	0.034091387	0.887162367
AT2G46110	KPHMT1	-0.775955861	0.439790375	-1.763917266	0.295716908	-1.022635514	0.022255396	-0.303440931	0.926730556	0.466345853	0.519043739	0.627029625	0.026922357
AT2G31360	ADS2	-0.103992593	0.85212188	-0.547595196	0.002889572	-1.023452916	3.96651E-10	-0.433836981	0.474958589	-0.119069723	0.603531978	-0.350876071	0.076854548
AT5G43745	AT5G43745	-1.096969015	0.08524909	-0.334477707	0.642187791	-1.023591108	8.03268E-07	-0.625712586	0.798570031	0.030406241	0.965667906	0.613247541	0.001632849
AT4G13170	AT4G13170	-0.938715861	0.051712702	-0.828768555	0.000200164	-1.023592615	2.28266E-06	-0.136236028	0.955547263	0.064594327	0.882236352	0.273576584	0.09386099
AT5G47770	FPS1	-0.988803935	5.40755E-05	-0.887807081	6.03844E-08	-1.024194658	4.02109E-16	-0.043699019	0.966973448	-0.000132918	0.99989203	0.286046855	0.046706483
AT3G25545	AT3G25545	-0.111803381	0.882140511	0.031452124	0.950039021	-1.025360894	0.009591841	-0.263763281	0.894700903	-0.389562816	0.405526156	-0.053436204	0.856266389
AT1G22360	UGT85A2	-0.054323622	0.937194036	-0.635500321	0.067746505	-1.026909841	0.006443583	-0.374543875	0.768328791	-0.151590911	0.540899829	-0.592687056	0.00011571

AT3G55960	AT3G55960	-0.804242509	0.050514128	-0.508890034	0.066750981	-1.027227961	4.08854E-09	-0.213305577	0.741998356	-0.206084977	0.608938746	0.195391887	0.34900433
AT4G39960	AT4G39960	-0.422800261	0.436548025	-0.64296304	0.146969251	-1.027666577	0.008465771	-0.275231107	0.881491928	0.00202141	0.998561976	-0.314900079	0.184351198
AT5G60030	AT5G60030	0.646058824	0.321121302	-0.085638759	0.907664875	-1.028511514	0.022864306	-0.048105786	0.982749202	0.141779455	0.851437944	-0.680772761	0.053748735
AT4G17880	MYC4	0.266509225	0.658005927	-0.999707777	0.04909948	-1.029270287	0.014816721	-0.079976042	0.968587518	-0.110244045	0.847697457	-0.323931481	0.198930236
AT1G74380	XXT5	-0.77721444	0.071552583	-0.820015497	0.001048173	-1.031567635	6.20741E-07	-0.933795007	0.46594725	-0.295807621	0.402366475	-0.199006161	0.187133724
AT5G63650	SNRK2.5	-0.293288366	0.776081977	-0.140477429	0.884314612	-1.032686079	0.02530328	-0.548011972	0.698438613	0.037832682	0.930394509	0.637396282	3.10613E-07
AT2G45270	GCP1	-0.878066957	0.023063332	-0.502426989	0.145252105	-1.032936986	0.000453624	0.19515193	0.868525831	-0.012604916	0.988079805	0.122401007	0.697526428
AT5G43440	AT5G43440	-0.726788232	0.232859029	-0.207753037	0.621719311	-1.033385065	0.000848461	1.342928561	0.383660928	0.085082123	0.934318497	-0.277558722	0.491926726
AT5G08400	AT5G08400	-0.01875358	0.986984579	0.204700855	0.767858599	-1.033686462	0.00598422	-0.526907628	0.68299661	-0.326341302	0.58081406	0.733979659	0.000769144
AT1G73830	BEE3	-0.877261656	0.020280296	-0.96877803	0.166114464	-1.034225222	3.103E-05	0.291376066	0.800300732	0.359987245	0.168660897	0.475547216	0.007207913
AT1G66820	AT1G66820					-1.037137405	9.49848E-05					0.227530948	0.588020922
AT1G13860	QUL1	-0.887866528	0.099543829	-1.140437394	0.131863661	-1.038739086	0.009465295	-0.528558254	0.706790319	0.037232582	0.962199154	-0.07286708	0.798222366
AT3G24715	AT3G24715	-1.152167316	0.054188935	-0.202591474	0.778654169	-1.040067566	0.040320921	-1.329267708	0.242770523	-0.305472871	0.645173966	-0.344036244	0.185344727
AT4G27240	AT4G27240	-1.057365103	0.150287896	-1.001537205	0.223891656	-1.040497926	0.003532734	-0.259443164	0.885840009	0.290662204	0.52838553	0.428183857	0.012140653
AT5G66640	DAR3	-2.008137051	0.065320716	-0.073827209	0.939070838	-1.041837313	0.026035713	-0.004205921	0.998326123	-0.487529083	0.72447373	0.56034375	0.105669456
AT1G18570	MYB51	-0.805110531	0.106507893	-0.283395746	0.537372637	-1.042255323	0.004882516	-0.148570306	0.865029651	-0.666672998	0.000838733	0.891097594	6.29713E-08
AT4G31590	CSLC5	-0.620968222	0.198307848	-0.499428495	0.17661564	-1.042828726	5.03589E-06	-0.807529745	0.461515802	-0.448733444	0.100538468	-0.114407333	0.620368396
AT5G37480	AT5G37480	-0.007148915	0.994603462	-0.254022976	0.717251281	-1.043297881	0.006798125	-0.002999765	0.999072892	0.07546292	0.924140734	-0.095109626	0.711493724
AT2G31340	emb1381	-0.564866719	0.368100522	-0.625492613	0.186816318	-1.043438377	0.002090155	-0.619043312	0.712887303	0.234487514	0.724480035	0.553513405	0.009443509
AT2G12400	AT2G12400	-0.412974511	0.394947701	-0.402675082	0.23056654	-1.045625908	1.40799E-05	-0.94024415	0.377008424	-0.488111373	0.042773824	-0.206902179	0.243236144
AT3G21400	AT3G21400	-0.567980246	0.591503164	-0.284698828	0.761393724	-1.046052466	0.026724252	0.325090541	0.835453631	0.48664011	0.511659422	0.808214403	6.29524E-05
AT1G69680	AT1G69680	0.408625408	0.729455307	0.193935787	0.820588517	-1.046160986	0.035299197	-0.098467665	0.965812438	0.228200519	0.806656038	0.163334273	0.69816555
AT4G18930	AT4G18930	-0.814216096	0.051138139	-0.824841739	0.013914605	-1.049637155	0.006876093	-0.156765841	0.871661081	-0.343884169	0.423442669	-0.234305123	0.404029055
AT1G21980	PIP5K1	0.098199782	0.858404535	0.013691257	0.976592039	-1.050633368	1.53794E-06	-0.659749043	0.681315477	-0.339530419	0.14099198	-0.772849951	3.98095E-11
AT1G16800	AT1G16800	0.12424882	0.903526096	-0.887543524	0.162898562	-1.053050514	0.001476109	-0.09572209	0.972043985	0.195613442	0.727140875	-0.214042485	0.372928323
AT2G36470	AT2G36470	0.198414369	0.835151646	0.486565932	0.571941444	-1.053973756	0.041626114	0.055252756	0.97809523	-0.065247833	0.95555756	-0.025998544	0.951374475
AT5G57030	LUT2	-0.348837974	0.354171467	-0.170416018	0.561209991	-1.054581733	6.96301E-09	0.348027033	0.695103554	0.175302349	0.686407369	0.466646265	0.000910518
AT1G22070	TGA3	-0.459020241	0.334175913	-0.666174121	0.101384231	-1.055313715	0.001206486	-0.187625563	0.809300038	-0.044557454	0.954183802	-0.529251126	0.006007444
AT2G40550	ETG1	-1.130191515	0.11730376	-0.795717503	0.046222847	-1.056754705	0.011023615	0.239567473	0.918652486	-0.291853522	0.558208241	0.063813696	0.846076347
AT3G54430	SRS6					-1.057460939	0.008744821					0.074315054	0.800940027
AT5G11740	AGP15	-0.314419923	0.351400494	-0.589293137	0.018803081	-1.059622583	8.81343E-08	-0.623204119	0.517452709	-0.11653327	0.708114422	-0.374947862	0.433215945
AT2G14880	AT2G14880	-0.72587061	0.035654723	-0.478395271	0.080295461	-1.059802549	0.000644884	-0.194547614	0.920233522	-0.037823804	0.947746874	0.567652551	0.001573684
AT2G43850	AT2G43850	0.087485479	0.938520791	-1.972342703	0.064326263	-1.060045509	0.007925657	-0.410997099	0.696427231	-0.578052188	0.301120228	0.001231894	0.997491107
AT5G50150	AT5G50150	-0.667730269	0.286915577	-0.683728544	0.158245015	-1.061018566	0.012218921	-0.440219799	0.685208378	-0.038599044	0.955714995	0.223263521	0.198911212
AT2G33210	HSP60-2	-0.507930224	0.59809306	-0.727450556	0.216164596	-1.061021133	2.58581E-05	-0.18007171	0.951514049	-0.107954114	0.817827569	0.215204471	0.149580443
AT2G16460	AT2G16460	-0.592776818	0.305751196	-0.6799849	0.317985658	-1.06181857	0.017835781	-0.281223315	0.809632507	0.080040717	0.954116505	0.260188574	0.353872773
AT3G22142	AT3G22142					-1.062144458	0.000255606					-0.173826344	0.818002025
AT2G44160	MTHFR2	-0.688529132	0.025563338	-0.492100489	0.004874749	-1.062162345	6.55115E-17	-0.265499507	0.73209996	-0.123073347	0.566059569	0.246005588	0.098108122
AT5G61040	AT5G61040	-0.602510276	0.331780189	-0.759225075	0.180631694	-1.063688923	0.006717879	-0.544205701	0.676109289	-0.07649724	0.935266104	-0.09472756	0.773569854
AT5G50460	AT5G50460	-0.261531222	0.710100481	-0.694451348	0.121346871	-1.065401193	8.88685E-08	-0.418892176	0.793956546	0.114497098	0.773789213	0.121661322	0.581274794
AT2G28720	AT2G28720	0.031121895	0.973083341	-0.937628178	0.022896597	-1.067252536	3.83374E-06	0.412678288	0.800300732	0.114482147	0.891347914	-0.725183104	0.023892431
AT1G07220	AT1G07220	-0.20409885	0.775221978	-0.468681149	0.31510004	-1.072439238	0.007565335	-0.727392861	0.643394187	-0.529970527	0.207975462	-0.086984489	0.758992991
AT4G13930	SHM4	-0.739982017	0.281440464	-0.723195964	0.005514358	-1.072458028	5.77455E-11	-0.667215376	0.672844426	-0.357236271	0.011547431	0.154664449	0.36448492
AT1G24530	AT1G24530	-0.775046092	0.007818142	-0.786550033	0.177849813	-1.072808458	0.000217548	0.200281398	0.840351964	0.105624084	0.864864207	0.956853323	2.28207E-05
AT1G63220	AT1G63220	-0.405633002	0.69939111	-1.249818238	0.067406083	-1.072844454	3.19247E-05	-1.035776218	0.584219873	-0.138798038	0.809654035	0.086129587	0.731765969
AT4G38330	AT4G38330	-0.695027163	0.571620273	-0.533368236	0.528232795	-1.074504609	0.031618457	-0.198111306	0.95660961	0.765676971	0.437384676	0.398102086	0.346034696
AT2G34640	PTAC12	-0.817802263	0.107575253	-0.954231952	0.034333841	-1.075175551	2.22349E-06	-0.115074228	0.963292526	0.087477181	0.824246731	0.515811353	8.98824E-06
AT5G58520	AT5G58520	-0.599862539	0.142970885	-0.689700687	0.128637543	-1.076422749	0.015369985	-0.505021934	0.685495817	-0.055016942	0.937392682	-0.00130866	0.996255728
AT2G26930	CDPMEK	-0.822867837	0.019104471	-0.951878855	0.009348712	-1.076584219	9.07698E-06	0.276861721	0.781016963	-0.034344415	0.966304751	0.512296765	0.01089384
AT2G25310	AT2G25310	0.085702965	0.886546602	-0.356757787	0.175865551	-1.076822681	0.000193288	-0.334157994	0.691239307	-0.090115958	0.804786811	-0.254145293	0.047713279
AT1G25390	AT1G25390	-0.335885572	0.454444822	-0.659827172	0.04909948	-1.076829559	0.005621816	-0.527120527	0.584219873	-0.316130708	0.467113472	-0.095533763	0.762529404
AT5G66410	PLP3b	-0.936513567	0.095015419	-0.497293742	0.241522862	-1.077453835	0.000256592	-0.560978684	0.776796299	0.037115659	0.958126572	0.370577478	0.032403863
AT2G41890	AT2G41890	-0.763618452	0.147974667	-0.797175481	0.035099145	-1.081095459	4.44199E-05	-0.705256869	0.660697037	-0.123262278	0.872528185	0.006967978	0.984249318
AT4G11010	NDPK3	-0.067504683	0.936581826	0.371297747	0.380416109	-1.082186114	1.47596E-08	-0.430011092	0.823318742	0.315409307	0.152238433	0.428104733	0.002161565
AT1G58440	XF1	-0.72636175	0.030906044	-0.427146105	0.191584509	-1.082243167	3.01215E-05	-0.401653482	0.653026084	-0.452042603	0.087042206	-0.124032179	0.646992921
AT4G34710	ADC2	-0.535986014	0.066520439	0.006750539	0.981437572	-1.082635675	1.14002E-07	-0.001666923	0.999298203	0.034886285	0.924327265	0.371273313	0.00623699
AT3G05675	AT3G05675	0.506897991	0.507937078	-0.448887916	0.590951808	-1.082658118	0.026489083	-0.540361024	0.702246294	0.029007163	0.980488802	0.16164863	0.587043893
AT2G43290	MSS3	-0.121000379	0.851413535	-0.545135718	0.117507111	-1.082795687	6.69039E-07	-0.72052677	0.498013754	-0.215613844	0.485309912	-0.122899883	0.544355521
AT2G23180	CYP96A1	-0.657467694	0.318530672	-0.20608311	0.768207562	-1.083385481	0.027365129	-0.266744825	0.963390011	0.464897468	0.373045197	0.182635325	0.638429995
AT4G09570	CPK4	-0.846075584	0.010599312	-0.353336015	0.521993585	-1.087191898	0.00010224	-0.692660654	0.165577607	-0.465957576	0.159233933	0.840373722	4.72688E-08
AT4G24700	AT4G24700	0.304111283	0.82958108	0.310887912	0.791720507	-1.087637178	0.032676842	-1.907391402	0.784665705	0.294750679	0.916551162	-0.504118206	0.445973742
AT3G25890	CRF11	0.305491065	0.726976273	-0.274978317	0.676332404	-1.088478452	0.000409165	0.091490926	0.964169173	0.194679333	0.583878077	0.061392482	0.796908831
AT1G31010	OSB4	-0.262436513	0.777447935	0.245949313	0.786716723	-1.089102839	0.037785997	-0.01273777	0.997245719	-0.205795268	0.809692025	-0.106925125	0.801934126
AT1G76360	AT1G76360	-0.973644032	0.039688999	-0.942450724	0.00023459	-1.090030399	1.52956E-07	-0.083706788	0.942141085	-0.231520936	0.725609398	-0.195996369	0.463983459
AT3G62600	ATERDJ3B	-0.317143835	0.632987689	-0.513220867	0.078559405	-1.090518509	6.45101E-06	-0.48500218	0.751015129	0.116287399	0.717185262	-0.39613875	0.040917914
AT1G62180	2-4月	-0.485639516	0.769121541	0.078999813	0.922063591	-1.092997819	0.001157155	-0.772850662	0.597380545	0.040192402	0.962421525	-0.631245572	5.4351E-05
AT1G77690	LAX3	0.545595886	0.294691079	-0.658077775	0.054538114	-1.093441482	0.000183891	-0.766386006	0.278458537	-0.15656998	0.570054815	-0.325462291	0.036148512
AT1G79890	AT1G79890	-2.187113708	NA	-1.175071116	0.270618878	-1.095029798	0.003864409	-0.761704892	0.886091691	-0.571901057	0.536643581	0.109232626	0.747137096
AT3G60290	AT3G60290	0.310246577	0.848312391	-0.479525567	0.452244523	-1.095094262	0.002729538	0.921678716	0.548879143	-0.229321439	0.89880166	0.363124804	0.321364236
AT4G17180	AT4G17180	-1.288732726	0.135774791	-1.384368959	0.05442068	-1.095159652	0.022753268	-0.066020129	0.984928214	-0.007703795	0.994809352	-0.082997897	0.796908831
AT5G65610	AT5G65610	2.381352659	0.631700583	1.895101446	0.677627705	-1.098568423	0.043171677	-4.240317945	0.706790319	0.532515158	0.918426331	0.020846566	0.972388595
AT2G32920	PDIL2-3	0.593863018	0.24625377	0.163597505	0.651682233	-1.099537451	8.51828E-06	-0.713647902	0.588988003	-0.178313042	0.5094879	-0.254418347	0.106336565
AT2G22100	AT2G22100	-0.111507081	0.944131239	-0.829990296	0.439807302	-1.100116848	0.026658784	-0.1113238	0.966082349	-0.049191108	0.959952977	-0.420687332	0.088676533
AT4G19120	ERD3	-0.373075907	0.631700583	-0.164549534	0.82924137	-1.100383156	0.007410562	-1.192929199	0.447715856	-0.529058799	0.048200607	-0.662884967	5.81952E-09
AT3G07568	AT3G07568	0.331699518	0.904683096	-0.236525531	0.930871605	-1.100697817	0.024161946	-0.580629627	0.899252848	0.845034055	0.301150815	0.248024276	0.533639437
AT1G69160	AT1G69160	-0.969251611	0.01879298	-0.792517729	0.000918331	-1.106776501	4.47871E-12	1.251764487	0.228792905	0.246207716	0.588646494	0.309782663	0.174005358
AT5G35670	iqd33	-0.111825402	0.910743654	-0.431105038	0.615563307	-1.106904109	0.046777874	-0.208090703	0.931898506	0.387479034	0.524769439	0.798953227	0.001224338
AT1G67880	AT1G67880	-0.538264957	0.407850206	-0.187051465	0.74327068	-1.107687728	0.009350436	-0.030910474	0.987671055	-0.268220958	0.570854222	0.766011311	3.69751E-06
AT1G13260	RAV1	-0.197381842	0.676104433	-0.835221073	0.000752248	-1.11147202	1.8944E-18	0.113917192	0.934711472	-0.359654139	0.01770954	0.534409929	5.9851E-05
AT4G21960	PRXR1	0.012399056	0.983922132	-0.253486729	0.332212779	-1.111639535	0.044618363	-0.451268949	0.691941372	-0.481014244	0.011211792	-0.320897732	0.006679649
AT1G48460	AT1G48460	-0.346557517	0.786760776	-0.171454709	0.845892791	-1.113019877	0.031501613	-0.225163708	0.790756089	0.465573176	0.319126358	0.175564965	0.547314839
AT1G77090	AT1G77090	-0.889752255	0.118254641	0.074863198	0.881736896	-1.1148511	0.000210527	0.018112172	0.992411026	0.2717803	0.693633289	0.233707369	0.417110901
AT3G23050	IAA7	-0.730427039	0.019816185	-0.697318596	0.000198132	-1.11551182	1.05774E-13	0.031579239	0.976195901	-0.171301751	0.475390745	-0.04214898	0.851340178
AT5G13400	AT5G13400	-0.854533603	0.064239043	-0.378000116	0.483341914	-1.11583612	0.010138576	-0.644923997	0.438062471	-0.416397869	0.281175492	-0.40320796	0.117179852
AT1G61260	AT1G61260	-1.011157359	0.074259877	-0.366133925	0.466923818	-1.116622361	0.008653087	-0.719177501	0.606948021	0.009449403	0.993849308	0.061543696	0.857036312
AT1G63130	AT1G63130	-1.225437043	0.174836627	-0.379874917	0.573401511	-1.116688984	0.022347932	0.26865217	0.882425056	0.194789969	0.813086742	-0.012180882	0.979473037
AT3G44730	KP1	-1.386067663	0.070920858	-1.92422324	0.204175799	-1.120835179	0.02978321	-0.613623403	0.750687341	-0.231611441	0.694510885	-0.657115954	0.000942419
AT5G02410	ALG10	-0.504813179	0.5394706	-0.894921485	0.239190304	-1.121596317	0.001732861	-1.021487402	0.816446062	-0.617237547	0.158420892	-0.079292317	0.790710524
AT2G22170	PLAT2	-0.360066799	0.199536598	-0.478684762	0.236455252	-1.123368975	3.06747E-08	-0.659682904	0.135734471	-0.520433904	0.002554745	0.005071333	0.984590646
AT5G18820	Cpn60alpha2	-1.07989275	0.255988601	-0.597930512	0.371215678	-1.125039069	0.029574481	-0.161543479	0.905158299	-0.360279041	0.567773209	0.307657676	0.218285766
AT1G20830	MCD1	-0.737387106	0.099003529	-0.738751494	0.124215418	-1.127712021	0.000661976	0.515670791	0.680482607	0.041951312	0.964180223	0.345912726	0.149536974
AT2G43180	AT2G43180	-0.837636019	0.098713049	-0.318404153	0.536747283	-1.128391243	0.000124857	-0.022549181	0.993737713	0.297220748	0.65213715	-0.133092583	0.751043136
AT5G52910	ATIM	0.084281291	0.917299809	0.213082536	0.763236749	-1.12877356	0.014320668	-0.345261319	0.886772005	-0.142311723	0.872234469	0.66379664	0.000406364
AT2G26680	AT2G26680	-0.368240822	0.60671	-0.381223771	0.423082407	-1.129952999	0.000357738	-0.494516853	0.577205285	-0.116164202	0.805660767	0.010917737	0.969252469

AT3G59400	GUN4	4.608173391	0.16707985	2.524396199	0.668441762	-1.130387513	2.74442E-10	-0.349222362	0.978043477	-6.470800231	0.105439314	0.827199487	0.000523223
AT4G14770	TCX2	-0.912622718	0.030645568	-1.79211194	0.505647142	-1.131073464	0.015106457	-0.328811201	0.84268148	0.031099447	0.964180223	0.557381487	0.000530563
AT1G36390	AT1G36390	-0.600283753	0.456745185	-0.804710725	0.177406704	-1.132723351	0.001452524	0.187573996	0.873147282	0.296879602	0.623982577	0.425704566	0.05726891
AT3G47860	CHL	-0.79048085	0.070740262	-0.603360579	0.207638339	-1.136981459	1.33238E-05	0.391916766	0.679033189	0.242115485	0.563104532	0.683723394	2.86419E-06
AT1G55260	AT1G55260	-0.990830913	0.026806813	-0.290221361	0.511046037	-1.140889269	0.004426192	-0.718713303	0.663046749	-0.146133674	0.706765606	0.187117758	0.373475927
AT1G20930	CDKB2;2	-0.502568118	0.64371527	-0.799010663	0.060785937	-1.14097601	0.000270739	0.068214468	0.968021479	0.284346689	0.588204188	0.896868262	1.59937E-10
AT5G58970	UCP2	-0.814348957	0.440751266	-1.095515009	0.22230865	-1.14141304	0.002828947	-0.144386988	0.951210681	-0.733762273	0.071991501	0.33183314	0.137213069
AT1G73650	AT1G73650	-0.568069744	0.258599383	-0.603377262	0.107888821	-1.145774808	3.71414E-05	0.473587795	0.606948021	-0.090911452	0.918646458	0.227603541	0.407576368
AT1G02650	AT1G02650	-0.866264146	0.246980733	-0.611581899	0.317423143	-1.146518621	0.013380932	1.623934114	0.2905498	-0.626092567	0.626594727	1.527282266	0.104492093
AT5G49170	AT5G49170	-0.485140426	0.564469691	-2.340048445	0.12521783	-1.147431574	0.00996071	-0.564000254	0.822720536	0.455118699	0.364175721	0.822880009	0.000362744
AT4G25900	AT4G25900	-0.777081369	0.058617708	-0.298816029	0.405234232	-1.14823784	7.61763E-05	-0.73115489	0.45375294	-0.291168649	0.43793265	-0.432049216	0.008805798
AT3G15060	RABA1g	0.007927648	0.991149836	-0.495603233	0.287174916	-1.148265505	0.027620558	0.46346778	0.715099832	-0.251386122	0.654509491	-0.411845342	0.323677785
AT2G03550	AT2G03550	-0.84558254	0.014794775	-0.332502878	0.379529344	-1.149051314	0.014073517	0.64625471	0.647947756	0.459797356	0.393043989	0.633907256	0.059206097
AT5G05190	AT5G05190	-0.643740904	0.338778694	0.189782614	0.82888974	-1.149572661	0.007661584	-0.302392733	0.867112468	-0.596685015	0.318968615	-0.097800191	0.820515822
AT2G41110	CAM2	-1.56453497	0.072605832	-2.084575485	0.181775246	-1.149772158	5.36774E-06	-0.902243589	0.692108738	-0.806845444	0.232646545	0.401999504	0.041911703
AT5G23920	AT5G23920	-0.471489457	0.319628734	-0.121946538	0.749182172	-1.150281049	1.23311E-07	-0.441742789	0.613859108	-0.435911789	0.094613963	0.119695913	0.61860441
AT1G23970	AT1G23970	-0.064548115	0.937458757	-0.765361459	0.224678208	-1.150463533	0.004935815	-1.012626069	0.437207517	-0.064354608	0.944178317	0.055032298	0.863669815
AT4G38860	AT4G38860	-0.559362381	0.679232209	-0.856666028	0.215679018	-1.150894379	0.001484635	-0.04517372	0.991438225	0.640096324	0.083096348	-0.036671675	0.902568843
AT2G39360	AT2G39360	-0.278244	0.586711287	-0.304979223	0.511735201	-1.155168194	0.0019925	-0.804247639	0.308658904	-0.460468353	0.34321022	-0.845624504	9.39277E-06
AT5G46910	AT5G46910	-0.551489347	0.326350242	-0.472405732	0.25691222	-1.156095791	0.000896722	-0.683214028	0.564319346	0.067475805	0.940221624	0.542903456	0.022322615
AT1G64330	AT1G64330	-0.029159777	0.973333102	-0.298257172	0.567139906	-1.156146929	0.00249094	-0.573431476	0.691824202	-0.128987015	0.796178186	-0.493412476	0.016727919
AT1G50575	AT1G50575	-0.755911378	0.324253366	-1.184260565	0.062735235	-1.156733528	0.012766896	-0.3475741	0.869069601	0.345004398	0.43480166	0.29455443	0.253755214
AT3G17120	AT3G17120	-0.355792636	0.668360809	-0.715861069	0.042205889	-1.158752187	0.034843276	-0.752784985	0.692108738	-0.045730385	0.934609223	0.206583739	0.225408568
AT1G71970	AT1G71970	-0.975646379	0.000125186	-0.979242581	0.001288972	-1.160560232	4.09897E-09	0.418173604	0.632278424	-0.056848439	0.924627441	-0.364350339	0.088472573
AT1G11290	CRR22	-0.78213392	0.372201645	-0.262535625	0.820265411	-1.162215544	0.016184532	0.037095638	0.996799452	0.357436682	0.710593111	0.625217959	0.031228406
AT5G59350	AT5G59350	-0.067493195	0.953518517	-0.178740766	0.781915365	-1.163894082	0.005721627	-0.6629418	0.657374576	-0.155639266	0.79109963	-0.087038499	0.808438467
AT2G20630	PIA1	-0.174973692	0.747470362	-0.005857433	0.989490222	-1.164387829	1.76462E-11	-0.498807462	0.696184854	-0.226734183	0.352772432	0.100364836	0.564984661
AT4G35350	XCP1	-1.126953634	0.347295741	-0.912187088	0.112534692	-1.167092869	0.035051487	-0.762913523	0.816446062	-0.701872204	0.024837418	-0.257647249	0.269978452
AT3G08630	AT3G08630	-0.583115805	0.355936141	-0.594353227	0.443851732	-1.16777312	0.000226362	-0.743482516	0.637531704	-0.09497431	0.873994427	-0.078852203	0.754134839
AT5G20080	AT5G20080	-0.632858206	0.376918873	-0.127650253	0.793190862	-1.168160928	7.49491E-08	-0.337850161	0.878770658	-0.002817866	0.995617102	0.473373592	0.000151732
AT4G14890	FdC1					-1.169186833	1.99001E-10					0.728313433	8.67744E-05
AT5G26667	PYR6	-0.299743418	0.654419394	-0.55491558	0.215269214	-1.169344391	1.65155E-10	-0.602036344	0.681538103	0.026510155	0.954083501	0.363074798	0.055091402
AT1G31920	AT1G31920					-1.170004974	2.75547E-08					0.211304275	0.494261571
AT3G49220	AT3G49220	-0.166978017	0.695206885	-0.251895578	0.219593602	-1.170045277	1.47E-05	-0.873303187	0.574062337	-0.635576735	0.000189738	0.149125493	0.425809972
AT5G28150	AT5G28150	-0.140682565	0.879443097	-1.086961393	0.051802748	-1.171488665	0.000309827	-0.493846366	0.84223801	0.246057961	0.613407287	-0.430779065	0.078482174
AT3G02660	EMB2768	-0.754988029	0.194113973	-0.915903579	0.046057109	-1.17752444	7.02601E-05	-0.407392749	0.795199419	0.520025774	0.160507774	0.096418279	0.754176524
AT5G55510	AT5G55510	-0.87854696	0.168139571	-0.84620973	0.055161182	-1.177939485	0.000469913	-0.099594115	0.963956318	-0.183018284	0.809056368	0.50664185	0.041430432
AT5G01950	AT5G01950	-0.879184872	0.004233438	-0.759157897	0.010589975	-1.182979877	1.49579E-08	-0.107609506	0.904122885	-0.007501815	0.992021888	0.046722605	0.864416493
AT1G29980	AT1G29980	-0.742596877	0.562595348	-0.631113584	0.375772743	-1.185111104	0.000426029	-1.039239427	0.532118649	-0.386713396	0.105491787	-0.194180201	0.356542999
AT2G46530	ARF11	-0.656119224	0.158594539	-0.623981735	0.052320928	-1.18541744	1.63375E-05	-0.283172813	0.682142539	-0.020017607	0.976616225	-0.536521787	0.002161565
AT3G06910	ULP1A	-0.797352783	0.416218882	-0.937303434	0.117456456	-1.188978013	0.003664258	-0.436719849	0.805751383	0.336036968	0.613578722	-0.040439302	0.904410352
AT3G12560	TRFL9	-0.327336154	0.570335651	-0.806985916	0.118337431	-1.189736197	0.000526668	-0.4158341	0.882425056	0.242016761	0.60011669	0.263573166	0.165276086
AT5G09240	AT5G09240	-1.497050873	0.165553306	-1.492066702	0.208839557	-1.189793222	0.002314743	-0.832585451	0.657950393	-0.104656269	0.913421083	0.35746237	0.323524679
AT2G04530	CPZ	-1.015428258	0.204738344	-0.56672766	0.386116634	-1.193391737	2.26544E-05	0.272964154	0.703833757	0.103102588	0.911074193	0.605652816	0.015594622
AT1G29960	AT1G29960	-0.865815492	0.254180232	-1.81948633	0.24065851	-1.194056491	0.015106142	-0.078841554	0.978979608	0.01116222	0.992611192	0.27211844	0.448303834
AT2G29560	ENOC	-0.263756774	0.738096056	-0.536822195	0.188377857	-1.195703136	0.005747456	-0.195040191	0.882124977	-0.010360011	0.986818647	0.062724168	0.784355787
AT1G57600	AT1G57600	-0.482933759	0.480091818	-0.97858473	0.130910923	-1.196055824	0.003111134	-0.013731799	0.995142373	-0.232599784	0.727585697	0.355236446	0.094295772
AT3G51290	AT3G51290	-1.386458019	0.088466161	-2.857468513	0.238302495	-1.197860061	0.002473627	-0.075692315	0.978807026	0.303581796	0.564980934	0.783003038	5.23727E-05
AT4G28310	AT4G28310	-1.214448977	0.357642513	-1.468841287	0.400075583	-1.198611779	0.034273634	-0.45570516	0.944057446	0.139402919	0.895894402	-0.515040066	0.017094494
AT2G29630	THIC	-0.47491302	0.286606431	-0.441482866	0.047381967	-1.199473375	9.02472E-06	0.887270067	0.51312948	0.001390257	0.998764738	0.489092789	0.006797873
AT5G59690	AT5G59690	-0.305402718	0.27504783	-0.773942532	0.000155921	-1.200743692	1.1731E-14	0.082223594	0.946164167	0.416727322	0.005882326	0.231649308	0.157099675
AT4G21880	AT4G21880	-1.538134459	0.09172801	-2.114718292	0.164735337	-1.203480097	0.008571987	-0.196399873	0.949913024	0.5107532	0.487963816	0.527790187	0.028269462
AT4G33360	FLDH	-0.269792761	0.747202061	-0.418845197	0.448584841	-1.20432107	0.000175444	-0.866007191	0.6100493	0.006429457	0.992611192	0.029729263	0.900506166
AT1G18265	AT1G18265	-0.658326999	0.676044314	-1.152641555	0.269158639	-1.208139166	0.024995207	2.867878646	0.592459968	0.571580279	0.865842332	0.270912201	0.834601312
AT5G49020	PRMT4A	-0.41550265	0.470470417	-0.7642133	0.003964251	-1.211557092	1.70399E-07	-0.143223357	0.952665809	0.149547983	0.782369045	0.28941288	0.095936524
AT4G30660	AT4G30660	-1.431236159	0.779208419	0.37327029	0.910195579	-1.211775683	0.02381559	1.287912065	0.855010607	-1.24031753	NA	-0.486558687	0.224117227
AT2G28900	OEP16-1	-0.641733206	0.110863414	-0.68129076	0.003297965	-1.213200568	1.51579E-06	0.15906147	0.929309252	0.050137904	0.928678672	-0.169473745	0.518667214
AT3G42950	AT3G42950	-0.414683055	0.563807373	-0.903797486	0.024853174	-1.214759722	0.00495589	-0.693161321	0.680248595	-0.464130586	0.122203706	-0.026310709	0.918008373
AT1G14460	AT1G14460	-0.981200052	0.021933754	-0.902212191	0.029271062	-1.21515406	7.86059E-05	-0.219766859	0.885755209	0.070644111	0.911074193	0.15200922	0.505509201
AT4G25835	AT4G25835	-0.96047779	0.060832271	-0.769433367	0.079636116	-1.215276903	2.94875E-05	-0.421323392	0.711996909	-0.372039788	0.537002257	-0.159862367	0.462724925
AT5G39320	UDG4	-0.482955656	0.608084003	-1.177479904	0.087529616	-1.216761407	7.58656E-09	-0.939733787	0.56002253	-0.064907982	0.897540079	0.639716436	5.66245E-05
AT2G30070	KT1	-0.750199201	0.093739708	-0.743419848	0.074532644	-1.217913025	1.00891E-08	-0.006167136	0.997796217	0.329288287	0.416330952	0.821354146	4.10369E-08
AT4G28400	AT4G28400	-0.413658202	0.195418219	-0.806233884	0.015493501	-1.219722109	9.00197E-09	-0.295831226	0.709586436	-0.180483882	0.633800749	0.779796356	1.20915E-07
AT3G05490	RALFL22	-0.8893225	0.01715368	-0.845494142	0.000251732	-1.220002163	1.39967E-06	-0.472380307	0.397155994	-0.28370775	0.170484591	-0.342980077	0.03902651
AT2G15050	LTP	0.069074787	0.912646363	0.669740217	0.008313506	-1.220334786	0.02895093	1.285163393	0.399905212	0.282502671	0.349500586	-0.708763647	0.069789087
AT4G38280	AT4G38280	0.207988242	0.737025845	0.463285148	0.28026848	-1.223251081	0.006774062	0.638696081	0.435922072	0.085271197	0.895976532	0.542751213	0.275265406
AT3G23820	GAE6	-0.694180143	0.006108782	-0.503242518	0.023661994	-1.224177301	1.83875E-21	-1.119941442	0.219098116	-0.543055217	0.00076913	-0.163562003	0.325504779
AT1G72670	iqd8	-0.63600006	0.656728421	-2.342385544	0.069759645	-1.22597226	0.014832656	0.256527752	0.917097586	0.176943727	0.854600248	0.355303268	0.115716485
AT5G39840	AT5G39840	0.513191646	0.606845279	-1.069136628	0.163342904	-1.226949615	0.030255106	-0.28287778	0.936143398	-0.225192998	0.774149402	-0.288821533	0.257939837
AT5G11950	LOG8	-0.865643677	0.106300923	-0.534451054	0.20125548	-1.228472464	0.008207603	-0.159021845	0.957161604	0.219627735	0.636145007	0.436296377	0.014589034
AT1G54180	BRX-LIKE3	-0.615329193	0.432164509	-1.004471945	0.121977974	-1.229321185	0.010738642	0.12578375	0.959586532	-0.016595834	0.989337643	0.302073793	0.397915306
AT5G66000	AT5G66000	0.430951674	0.701552003	-0.350545531	0.820826373	-1.230248295	0.041443892	0.566477676	0.629761053	-0.06689159	0.963495178	0.21531926	0.550952411
AT5G62350	AT5G62350	-0.276785307	0.292440215	-0.989537534	1.80654E-07	-1.232108816	5.76063E-07	-0.181556655	0.718141493	-0.342057207	0.055883031	-0.170015334	0.250205222
AT1G28660	AT1G28660	-0.596128628	0.199511245	0.00623457	0.995020274	-1.232312759	0.004923175	0.059329054	0.977477438	0.084133718	0.852739501	0.362952414	0.11884146
AT1G26650	AT1G26650	0.29120559	0.582988459	-0.479368622	0.257590084	-1.232557607	0.003346982	-0.44151501	0.6657277	-0.306303534	0.391017149	-0.287805394	0.297085666
AT5G11790	NDL2	-0.997293293	0.042764322	-0.983956023	0.010980488	-1.232802748	2.58089E-06	-0.481342549	0.751308246	-0.265104903	0.473771238	0.04033272	0.866309021
AT1G74700	TRZ1	0.233734117	0.838241968	-0.606971146	0.550943367	-1.234374636	0.013856166	-0.303034664	0.899163675	-0.070979076	0.964567705	0.206423022	0.615134051
AT5G19750	AT5G19750	-0.219520174	0.835661403	0.002998065	0.996373269	-1.235944876	0.009311975	-0.307378445	0.834203073	0.125926033	0.838103967	0.42419213	0.002599423
AT5G17630	AT5G17630	-0.964266092	0.154174592	-0.546162855	0.114015077	-1.236750821	6.31664E-06	-0.143087386	0.939540455	-0.133873561	0.79996985	0.131384544	0.538007672
AT1G15050	IAA34	0.574188473	0.770698195	-1.415644198	0.721887364	-1.238809514	0.021022873	-3.759253988	0.521675277	0.891517991	0.810706099	-0.305005624	0.868514097
AT1G43620	UGT80B1	-0.805356025	0.119141948	-0.941779622	0.16527318	-1.239351869	1.11383E-05	-0.608198706	0.598901706	0.056034887	0.954114031	-0.095860935	0.688724161
AT5G07900	AT5G07900	-1.082522809	0.230175167	-0.288620843	0.71854982	-1.240265631	1.68058E-05	0.529076066	0.66524984	-0.305046014	0.764722506	0.086315454	0.814315114
AT3G07390	AIR12	-0.973488369	0.015847579	-0.507547212	0.100957535	-1.245493097	3.26134E-09	-1.042462838	0.144996349	-0.322216128	0.29888981	0.434112712	0.002210967
AT5G49910	cpHsc70-2	-0.772980155	0.008414216	-0.105042605	0.638270014	-1.245967375	4.90398E-12	-0.09181176	0.964220939	-0.01332329	0.978424424	0.136119101	0.615105526
AT4G27270	AT4G27270	-0.727200189	0.366244117	-0.96703324	0.137130899	-1.246479521	0.004251119	-0.149708937	0.947563907	-0.044736355	0.964274323	0.787513492	0.000392015
AT5G65830	RLP57	0.176687877	0.833821933	-0.749120092	0.283720805	-1.248588031	0.025633727	-0.352304955	0.726713101	-0.728821589	0.176850826	-0.841718068	0.001708188
AT1G56260	MDO1	-0.681608707	0.444531686	-1.369926415	0.055706481	-1.25025613	0.034726451	-0.518538173	0.743769324	0.188436281	0.878918096	0.609443925	0.158603967
AT5G65840	AT5G65840	-0.941603127	0.044614046	-0.648370825	0.055282844	-1.250644776	3.50119E-05	-0.221255712	0.902565251	0.297596605	0.689695224	0.270432449	0.448109183
AT3G49730	AT3G49730	-0.324710453	0.842497187	-0.722515225	0.522427051	-1.257747824	0.010039897	0.156181183	0.982734229	0.131125606	0.929239543	-0.213871831	0.509643638
AT1G79760	DTA4	-0.351579323	0.720732143	-2.09099186	0.231625483	-1.25800944	0.038376733	-0.040393339	0.996463041	0.496784085	0.683729785	-0.650913957	0.041434831
AT2G32690	GRP23	-0.910428794	0.021361327	-0.665881185	0.014712033	-1.258887005	3.52648E-07	-0.780702445	0.317624416	-0.870684491	8.9575E-08	-0.911850022	0.036238559
AT3G13470	Cpn60beta2	-0.81121791	0.198821517	-0.544252555	0.256538077	-1.259538683	0.000159698	-0.161315825	0.938857514	0.15634895	0.743115665	0.237490943	0.35433008
AT1G14170	AT1G14170	-0.461981968	0.453564638	-0.656889777	0.064216227	-1.260675989	4.18493E-05	-0.23309799	0.932578056	-0.020221085	0.974319184	0.182717211	0.411518379
AT1G35430	AT1G35430	-0.339441067	0.67975358	-0.794084465	0.217326004	-1.2617593	0.003636809	-0.21218969	0.893481012	0.035287684	0.975716846	-0.345187427	0.184188999

AT5G62170	TRM25	0.059295147	0.939840178	-0.579763053	0.1831716	-1.266720771	0.013302744	-0.802943414	0.6107328	-0.542166465	0.108303825	0.16240475	0.50828951
AT1G22750	AT1G22750	-0.90224272	0.059394663	-0.557068595	0.286763295	-1.268044486	6.6714E-06	-0.348437758	0.851334862	0.078818941	0.950790869	0.046365362	0.917856939
AT1G29840	AT1G29840	-1.094209165	0.262770747	-1.059700504	0.169159586	-1.268998783	0.008185686	0.276943347	0.901188008	0.283687022	0.775873682	0.418364613	0.144537313
AT4G17430	AT4G17430	-0.453360153	0.513794629	-0.510927547	0.425623038	-1.269754807	0.003083529	-0.607959664	0.646899683	-0.091484965	0.930954102	-0.39435123	0.123050746
AT2G41770	AT2G41770	-0.735023366	0.168984849	-0.732638458	0.24561455	-1.271377545	0.000317614	-0.975282183	0.425005763	-0.371707334	0.423389572	-0.287706488	0.129748911
AT1G60890	AT1G60890	-0.505690408	0.664850545	-0.527141678	0.389189636	-1.271826427	0.000166851	-0.555318794	0.664635521	-0.091360004	0.905535933	-0.052908006	0.819344276
AT5G23360	AT5G23360	-0.812539413	0.220806651	-1.240943552	0.169984007	-1.272941476	0.0270834	-0.003621504	0.9987964	0.006787835	0.997128661	0.011684109	0.979855671
AT3G16290	EMB2083	-0.970456022	0.023502501	-0.414514896	0.160539808	-1.275228637	8.11524E-13	0.040556204	0.982734229	0.280336404	0.408064187	0.372287384	0.051924334
AT5G55620	AT5G55620	-0.873052156	0.047795038	-0.717413265	0.089051238	-1.276439085	5.03248E-07	0.11109346	0.972495118	-0.348937931	0.584309227	-0.311872342	0.392969215
AT1G20720	AT1G20720	-1.648454616	0.37868743	-3.091120752	0.367282473	-1.277192925	0.006662327	0.321500638	0.936758504	-0.170343149	0.894274701	0.441597789	0.180254399
AT2G40815	AT2G40815	-0.306345343	0.794316117	-1.687416615	0.075239214	-1.285503852	0.000319112	-0.023820276	0.994744545	-0.033606838	0.983764323	0.445055763	0.107381502
AT5G59410	AT5G59410	0.363982309	0.639761144	-0.10630481	0.886953052	-1.285558657	0.043376673	0.219457499	0.876015705	0.683629824	0.102885339	0.142905892	0.669990446
AT5G59500	AT5G59500	-0.681799516	0.213104442	-0.267451489	0.567581875	-1.2867671	1.5731E-05	-0.257724916	0.948672371	-0.005502956	0.995217542	0.340070208	0.098571684
AT5G50250	CP31B	-0.894659751	0.011857246	-0.600625627	0.016651966	-1.286798968	4.30907E-06	0.320037466	0.67890445	0.162390397	0.741681948	0.499708904	0.00126634
AT3G47690	EB1a	0.203437728	0.870637933	-0.686770169	0.576149143	-1.286981001	0.016701553	-0.32557828	0.894170799	0.202465463	0.765891356	0.339517243	0.11857672
AT4G12720	NUDT7	0.01727354	0.983226242	0.10599468	0.762308691	-1.28763384	2.08157E-08	0.290803843	0.83327628	-0.602742643	0.011956993	0.848503645	4.26028E-09
AT1G67660	AT1G67660	-0.84275799	0.313174392	0.173437638	0.716670363	-1.288161985	0.011100472	-0.252253266	0.930073201	0.010629259	0.99349199	-0.048051084	0.912232978
AT3G56000	CSLA14	-0.692903488	0.382208466	-0.47151639	0.554828208	-1.291038622	0.007193544	0.023526218	0.993737713	-0.169669638	0.843306602	-0.986822642	0.002778208
AT1G24240	AT1G24240	-0.459962948	0.597280869	-0.980382705	0.161901435	-1.291692955	0.026824611	-0.497774516	0.66924865	0.156945127	0.819806715	0.382747622	0.236642
AT3G16560	AT3G16560	-0.479107789	0.598766798	-0.534781018	0.400652455	-1.295980122	5.30109E-05	-0.577773169	0.691824202	0.042256542	0.958669515	-0.295647589	0.196400444
AT1G15000	scpl50					-1.296052433	1.39435E-10					0.024828245	0.921307057
AT1G53240	mMDH1	-0.621277784	0.156370979	-0.274954744	0.363575035	-1.29983966	2.45913E-16	-0.559062069	0.692562785	-0.054587761	0.866906715	0.457757184	0.003014621
AT1G33480	AT1G33480	-1.149243819	0.088028899	-0.512039957	0.577156671	-1.299914711	0.001521589	-0.276054329	0.911737107	0.227458686	0.814963929	0.133727495	0.702124456
AT3G29280	AT3G29280	-1.02012877	0.416218882	-0.907448998	0.383710798	-1.301952849	0.02467935	0.167533585	0.937910278	1.097213462	0.069240001	0.477287416	0.109900708
AT1G11300	AT1G11300	0.265916832	0.864380668	-0.784255509	0.722665196	-1.305888485	2.71557E-05	0.249254622	0.939383152	1.164477558	0.176633494	0.959648535	2.49298E-06
AT4G36810	GGPPS1					-1.306139113	0.000908978	-4.61151454	0.685495817	2.951013079	NA	-0.333336227	0.07979398
AT5G08350	AT5G08350					-1.311143916	0.004160628					0.27888114	0.311697367
AT2G37920	emb1513	-0.566344304	0.166204564	-0.618703945	0.096886687	-1.311887101	5.69244E-05	0.937456406	0.246589247	-0.166797914	0.864675247	0.230913826	0.47291281
AT3G11950	AT3G11950	-1.104636615	0.083366881	-0.783551045	0.241522862	-1.312143634	0.004573681	-0.299424671	0.832523868	0.038927087	0.971701608	-0.472457293	0.074345462
AT1G04050	SUVR1	-0.224573839	0.882140511	-1.217526998	0.222412481	-1.314508043	0.010203556	-0.181124762	0.883652513	0.107145545	0.930422599	0.116182286	0.760938865
AT1G19710	AT1G19710	-0.898441706	0.051511775	-0.500895971	0.369400893	-1.315171935	0.00493561	-0.565362299	0.66524984	0.228316099	0.611810682	-0.063661113	0.828652364
AT4G02110	AT4G02110	-1.292445775	0.263905113	-0.286822473	0.763236749	-1.31694827	0.015747483	-0.704642851	0.770580754	-0.070734472	0.956955597	0.024017047	0.950592079
AT1G65295	AT1G65295	-0.445611478	0.450153092	-1.068196191	0.071157862	-1.322070584	3.93002E-06	-0.729841043	0.584654981	0.143721315	0.769736176	-0.404759923	0.148838681
AT3G23160	AT3G23160	0.162310308	0.880366152	-1.060134292	0.071213734	-1.32229381	0.012110609	-0.751851943	0.766274785	-0.064090845	0.944128957	-0.75649102	0.005048558
AT2G30870	GSTF10	-0.018617254	0.966625921	-0.36610154	0.152463992	-1.324117362	2.35856E-10	-0.22221586	0.876919895	-0.191028575	0.487378356	-0.309281204	0.098352798
AT1G01420	UGT72B3	-0.362798949	0.668475598	0.281532724	0.682866594	-1.332021797	0.00357241	0.768976491	0.66686842	0.053231885	0.963495178	0.107660501	0.843770796
AT4G00905	AT4G00905					-1.336261908	0.024989272					0.416253605	0.210440529
AT1G26840	ORC6	-1.853801812	0.069513237	-0.095228246	0.968034359	-1.337911828	0.026648796	-1.560466997	0.691895802	-0.311796094	0.818088034	-0.176927173	0.677668919
AT4G23490	AT4G23490	-0.173950322	0.883855785	-0.35317377	0.647352073	-1.340396013	0.002978573	-0.644512595	0.726214276	-0.291438221	0.526108175	0.120860249	0.702370138
AT1G61000	AT1G61000	-2.039003031	0.198054011	-1.109615258	0.101821659	-1.341711336	0.007312216	0.059498869	0.980307293	0.49192222	0.400946216	0.134659087	0.668588147
AT1G63850	AT1G63850	-0.341752442	0.461130207	-0.538882714	0.046174071	-1.344420374	6.34499E-05	0.164456428	0.87460883	0.411030308	0.214036947	0.601013245	0.000181637
AT4G17050	UGLYAH	-0.889898407	0.005878549	-0.996900981	0.000137376	-1.346988875	4.22706E-08	-0.064844765	0.965260868	-0.389010264	0.1899614	0.290319793	0.132840319
AT1G69030	AT1G69030	-0.977102585	0.037405967	-1.066575971	0.251013605	-1.347012031	4.80862E-05	-0.579205516	0.644839236	0.070269408	0.933264689	0.092781423	0.761927143
AT2G29550	TUB7	-0.626730951	0.183176313	-0.926030734	2.25653E-08	-1.348282872	7.95817E-15	-0.256053861	0.872302914	-0.184454946	0.428388656	0.294636069	0.046316539
AT1G09040	AT1G09040	-0.355629419	0.70205241	-0.661301533	0.176484863	-1.351728581	0.014026481	0.129163699	0.93556951	0.161522327	0.86923938	0.474597123	0.10699273
AT1G48270	GCR1	-0.309186304	0.796113962	-0.370068549	0.669041128	-1.356420713	0.007827521	-0.774387003	0.386146892	-0.386680051	0.594846489	-0.066836273	0.848191362
AT1G30420	ABCC11	-1.175208118	0.167144517	-0.217402011	0.850714079	-1.363570169	0.04411953	0.236334994	0.935083701	-0.048491629	0.959320035	0.422254521	0.11579668
AT4G30410	AT4G30410	-0.650373941	0.296966124	-0.735438366	0.501514349	-1.363965817	0.004213235	-1.234008454	0.455598103	0.021971905	0.98143759	-0.3642034	0.114082795
AT1G53350	AT1G53350	-0.67936864	0.194914411	-0.722344906	0.158585346	-1.364086283	0.000542092	0.886916959	0.317624416	0.550392007	0.424170545	0.79870873	0.002641508
AT3G17420	GPK1	0.302412238	0.746270416	-0.76716829	0.395474667	-1.366570654	0.01984461	-0.580868204	0.708039647	-0.424830099	0.180665309	-0.242832465	0.206549448
AT3G11650	NHL2	-0.73484089	0.133773212	-0.604810053	0.165972615	-1.368341908	0.004685598	0.51029277	0.642268593	-0.485351595	0.383106164	0.294134464	0.248657527
AT5G67370	CGLD27	-0.898651304	0.072756164	0.051726059	0.941640409	-1.370364544	4.00101E-07	0.867491184	0.62738272	0.089335782	0.956653533	-0.263051317	0.534270204
AT1G01690	PRD3	-1.111068749	0.217906787	-1.031773389	0.135021461	-1.374987658	0.044143136	-0.697155744	0.699729916	0.137167382	0.890487133	0.148298317	0.529878313
AT4G25600	AT4G25600	-0.513183425	0.365534497	-0.82495359	0.165413337	-1.377651144	0.005404878	0.292762166	0.825815508	0.31981557	0.700995813	0.512477974	0.062073807
AT5G56600	PRF3	-0.848729125	0.079298992	-0.237964225	0.54677197	-1.384937189	8.76888E-09	-0.084138323	0.975645787	0.057942057	0.950068828	0.068882588	0.853247354
AT2G27970	CKS2	-0.594380423	0.266706366	-1.040543091	0.122306639	-1.385631561	3.34047E-05	0.0750563	0.972495118	0.095889375	0.854600248	0.36646427	0.059372609
AT2G21590	APL4	-0.049184611	0.963441891	0.081814987	0.919061855	-1.386235745	0.000908978	-0.040230642	0.985630298	0.092779137	0.929239543	0.187783014	0.412977646
AT5G40830	AT5G40830	-0.69018089	0.438456152	-1.40368692	0.079414834	-1.388538657	0.001833769	-0.838770923	0.685495817	-0.242037022	0.575644885	0.102993776	0.69851789
AT4G14330	AT4G14330	-0.562707783	0.681246618	-2.879859017	0.198553266	-1.390076686	0.010424325	-0.194536408	0.924925439	0.218143014	0.74053286	0.518955301	0.014488927
AT3G54030	BSK6	-0.788675133	0.151963145	-0.423247951	0.299389714	-1.395621892	9.93345E-10	-0.702747388	0.619462504	-0.298077408	0.376736667	-0.00172735	0.993748509
AT1G16630	AT1G16630	-2.07226934	0.253390621	-1.43342297	0.380072477	-1.396227023	0.031726116	-0.239814404	0.972495118	-0.069508941	0.951111828	0.319953272	0.128404738
AT3G19970	AT3G19970	-0.926771989	0.124030303	-0.485147347	0.471556954	-1.401652894	0.003030167	-1.013432048	0.340427521	-0.276191888	0.671914451	-0.431980941	0.131172627
AT1G49200	AT1G49200	0.241927612	0.945980546	-1.394580809	0.680507097	-1.402043366	0.003945614	-0.537012735	0.947241707	1.381641032	0.751599754	-0.488036035	0.028542487
AT3G22210	AT3G22210	-4.981360071	0.385289293	0.841087733	0.897577355	-1.408571919	5.31208E-06	0.919426208	0.961358666	-0.637720023	0.957130081	0.808866509	0.008404585
AT1G79850	RPS17					-1.4098342	2.80732E-44					0.624992332	0.000545119
AT1G67950	AT1G67950	-1.034805476	0.097061238	-0.264087369	0.691853355	-1.411392129	0.003596103	-0.620307073	0.81670535	0.226697614	0.762086306	0.579520508	0.000638251
AT5G56250	HAP8	0.110793962	0.918135268	-1.491873151	0.131783441	-1.414108008	3.85915E-05	-0.488699053	0.613392082	-0.116747339	0.883796441	-0.196637273	0.4763224
AT4G22730	AT4G22730	0.239604272	0.878121585	0.533923633	0.663698866	-1.414947441	0.030889544	-1.095837064	0.532959875	-0.799794842	0.099652256	-0.915085824	0.001375038
AT5G67150	AT5G67150	-1.037060596	0.203419482	-1.715348407	0.183672925	-1.416730054	0.014816725	0.129949236	0.972495118	-0.011118328	0.992762808	0.999067257	0.000124204
AT5G62140	AT5G62140	-1.410237658	0.06294047	0.183806917	0.870574021	-1.418476116	7.66355E-05	-0.004990097	0.998189671	-0.73371323	0.315034185	-0.018885035	0.972960625
AT5G07610	AT5G07610	-0.611613822	0.554496871	-0.130544681	0.863123566	-1.418658453	0.002450806	-0.29041884	0.884491938	-0.069874464	0.941154653	-0.424059095	0.16878314
AT2G18990	TXND9	-0.442827232	0.519039308	-0.815221024	0.172079729	-1.41949416	6.8309E-05	-0.184406567	0.928193379	0.050106652	0.946435828	0.081277383	0.762616382
AT3G50685	AT3G50685	-0.575045781	0.365220967	-0.83598085	0.027240639	-1.421809089	9.03717E-16	1.129690928	0.382849115	0.590806035	0.356290239	0.682325989	0.018246999
AT3G51710	AT3G51710	-1.387468815	0.068618722	-0.414937355	0.76685235	-1.427875423	0.049587048	-0.561005311	0.795199419	0.160360797	0.869846959	-0.541714889	0.041745543
AT4G22850	AT4G22850	-0.800664392	0.368100522	-0.979717527	0.192094661	-1.427983272	0.005875142	-0.243137901	0.921590494	-0.01550453	0.989259366	0.374968894	0.120634847
AT1G78100	AUF1	0.044273223	0.935970811	-0.367384995	0.368315148	-1.429998859	6.51686E-07	-0.705354362	0.280109421	-0.236623849	0.511659422	-0.971766819	4.89513E-06
AT2G42960	AT2G42960	-0.259298411	0.789968388	-0.282519317	0.645099884	-1.431848956	1.28023E-05	-0.183395652	0.929287022	0.026126072	0.976380404	0.55238567	0.001318373
AT2G38360	PRA1.B4	-0.706571491	0.452096561	-0.021724792	0.980651529	-1.431866026	0.01569751	-1.811452697	0.186649778	-0.803607711	0.002508933	0.056616221	0.852849337
AT5G10430	AGP4	1.356280558	0.799030872	-5.337726288	0.288235745	-1.433354242	0.000281802	-1.31660418	0.707962062	-0.193713506	0.935733669	-0.2721277	0.03358386
AT4G12880	ENODL19	-0.103519601	0.929121333	-1.025595763	0.226379267	-1.434546523	0.007149093	-1.047114622	0.643189471	-0.512639975	0.000697945	-0.225815664	0.149330851
AT3G25600	AT3G25600	-0.538738034	0.399804555	-0.799914297	0.046882052	-1.434852463	5.89833E-06	-0.317898359	0.743023983	-0.449385231	0.437231376	0.118195648	0.691273531
AT5G63780	SHA1	0.110276336	0.846930644	-0.076394474	0.89149533	-1.436354963	3.60295E-11	-0.16212959	0.847660958	-0.172870668	0.680523811	0.298307449	0.227408252
AT2G27130	AT2G27130	-1.333105638	0.288795636	-1.914627283	0.21885022	-1.436811327	0.021688584	-1.205220379	0.582068518	-0.159155721	0.880643463	0.177932332	0.516024542
AT4G12840	AT4G12840	-1.020150294	0.378814527	0.010426724	0.992356298	-1.442636333	0.047709625	-0.148483607	0.956216588	0.376099944	0.615893075	0.399091047	0.13840286
AT1G55535	AT1G55535	-0.339909353	0.78851868	-0.592824232	0.528491537	-1.443857522	0.012685354	-0.818467833	0.613859108	-0.698382369	0.037262273	-0.234108291	0.38493804
AT1G11000	MLO4	-1.215382266	0.060060869	-0.77618196	0.144625441	-1.444014163	0.000361193	-0.347500739	0.870136387	-0.44632496	0.282272743	0.620038251	0.001097425
AT5G52540	AT5G52540	-1.183796318	0.096877589	-0.937287241	0.052702605	-1.450036827	1.03672E-10	-0.530739425	0.905793887	0.049151615	0.952912486	0.309104861	0.05460798
AT3G62160	AT3G62160	-2.390386104	0.144456453	-1.212743197	0.202004941	-1.457573488	0.004465379	-0.465760644	0.916159273	-0.528798351	0.62939182	0.821145047	0.056981616
AT3G25100	CDC45	-0.92582269	0.629701975	-2.652976937	0.102578409	-1.45803591	0.034056896	0.308958564	0.965260868	0.350579816	0.666423542	0.027503605	0.953476821
AT5G37010	AT5G37010	-0.93797255	0.423394579	-2.015041216	0.484026023	-1.458710781	0.019147719	0.175840222	0.981040592	0.118659311	0.883729772	0.355696469	0.072905455
AT1G29630	AT1G29630	-0.783144932	0.537340087	-1.478212294	0.096137892	-1.459308144	0.001756806	0.136685106	0.956605792	-0.459923128	0.420851067	0.138275484	0.681350366
AT5G55760	SRT1	-0.631285719	0.557268753	-0.943005393	0.173329196	-1.45992194	0.000272479	0.078505814	0.977477438	0.004764229	0.99766313	0.411883455	0.187489462
AT5G07810	AT5G07810	-1.151046287	0.36241248	-1.208761248	0.184840667	-1.461144097	0.021159901	-0.293982341	0.931277674	-0.446556684	0.58567217	0.440132075	0.138406523
AT3G50340	AT3G50340	-0.548160907	0.320668858	-0.115959361	0.83666283	-1.461321669	0.001581365	-0.476526238	0.632278424	-0.061026084	0.949191441	0.403428854	0.167543758

AT1G41830	SKS6	-0.787982659	0.081780715	-0.084253235	0.854304674	-1.462517479	2.53008E-09	-1.060386804	0.562656211	-0.726882865	9.05753E-06	0.097519348	0.718310107
AT2G15280	AT2G15280	-1.134063785	0.053665493	-0.893076373	0.045959197	-1.467277694	0.000653853	-0.067872925	0.974831553	-0.003129887	0.997749369	0.754047797	0.000962195
AT3G16420	PBP1	-0.549496317	0.110923098	-0.738165092	0.002104426	-1.471103037	8.38393E-28	-0.484426109	0.548879103	-0.154470966	0.49939525	-0.571797792	0.000258568
AT4G00525	AT4G00525	0.332366608	0.836756444	0.152276067	0.939559076	-1.471239816	0.019797186	-0.826933067	0.881491928	0.726500885	0.735176002	0.17169909	0.720737185
AT1G17620	AT1G17620	-0.943083219	0.028420183	-0.820493494	0.029996377	-1.474213423	5.97274E-06	-1.011914816	0.50075057	-0.699766282	1.11302E-05	-0.167989224	0.381406453
AT4G13950	RALFL31	0.61322956	0.444072803	0.216200346	0.812091478	-1.475434131	0.010600298	-0.391349176	0.79604451	-0.009077769	0.994307202	0.062823895	0.861418164
AT1G29500	AT1G29500	-1.073462532	0.107293385	-0.207104466	0.821950193	-1.475474148	0.00015575	-1.093751759	0.282825999	-0.462736809	0.497836761	-0.640596319	0.008433662
AT5G18430	AT5G18430	-1.35980467	0.492922399	-0.464227847	0.724759335	-1.47926235	0.012992406	1.644676957	0.388440005	-0.17538614	0.958669515	1.069369525	0.228427668
AT5G39550	VIM3	-0.626382277	0.520879809	-2.142388676	0.153691189	-1.487933453	0.003837891	0.31490272	0.916964723	-0.204237572	0.752551043	0.068061983	0.853403897
AT5G03120	AT5G03120	-1.044367903	0.104983584	-0.890954586	0.350943098	-1.493869439	0.007045572	0.342908516	0.825908066	-0.320523086	0.473873568	-0.235487952	0.600732361
AT5G48470	AT5G48470	-0.796356291	0.231918634	-0.282961659	0.737568337	-1.496525222	1.37467E-07	-0.26007609	0.862230362	-0.06691448	0.934318497	0.858398467	2.96535E-07
AT5G09320	VPS9B			-3.553772892	NA	-1.496578124	0.01611021	-2.929008469	NA	4.535610705	NA	0.179225783	0.606213154
AT4G39380	AT4G39380	-1.497173046	0.061015484	-1.273605739	0.079414834	-1.499372422	0.018505694	-0.122887851	0.985904881	0.190861351	0.903379455	0.818080711	0.005015106
AT2G46380	AT2G46380	-0.772222197	0.230769961	-1.192869001	0.07015645	-1.504565163	0.006771405	-0.34912003	0.868423705	-0.290433077	0.681233319	0.452704723	0.120459784
AT1G22410	AT1G22410	-1.071864257	0.070834353	-0.592991513	0.09475926	-1.511064833	4.48721E-10	-0.540510899	0.726214276	-0.328027754	0.088665114	0.024023557	0.889366297
AT1G21120	IGMT2	0.23146576	0.912646363	2.176555639	0.167132845	-1.51109474	3.13991E-13	-2.912161558	0.076008337	-1.802380926	0.083096348	0.956435226	1.42683E-05
AT2G29320	AT2G29320	-0.915295279	0.223577398	-0.71532838	0.118337431	-1.511798243	4.56982E-05	2.122503043	0.622600949	-0.697081206	0.67431887	0.053434041	0.930514869
AT3G16920	CTL2	-0.752122985	0.342037549	-0.718918606	0.360604903	-1.514066222	0.000430428	-0.71778089	0.753910599	-0.701910177	0.305023554	-0.742377101	0.006809118
AT3G51660	AT3G51660	-1.099428929	0.207149969	-0.037597463	0.954041371	-1.515220689	0.004497159	-0.925845656	0.849119707	-0.073655378	0.963963546	0.043720071	0.921480203
AT1G02820	LEA3	-0.627421404	0.258713403	0.75456923	0.258007358	-1.518091853	0.02006265	-0.146947735	0.957573086	0.571973918	0.524173345	-0.740557867	0.092204407
AT5G15770	GNA1					-1.518695295	0.02824867					-0.483353277	0.334627903
AT3G04640	AT3G04640	-0.891247679	0.086371539	-0.656785988	0.057174474	-1.521852386	1.0221E-06	0.675260056	0.754353989	-0.727117825	0.270646891	1.146792829	0.177265141
AT2G26270	AT2G26270	-0.816829575	0.281440464	-1.257614	0.2155141	-1.527485046	0.003563516	0.306956942	0.867112468	0.097583462	0.932299626	0.75305559	0.001374816
AT3G43810	CAM7	-0.471726567	0.362308459	-0.864944897	0.010041444	-1.527506314	1.98656E-16	-0.546088505	0.719955906	-0.13328679	0.568739186	0.434362988	0.005326066
AT1G70430	AT1G70430	-1.304657076	0.348058211	-0.990732683	0.588270393	-1.527783646	0.003307376	-0.474620189	0.794600974	-0.346865918	0.684563426	0.693098087	0.001331492
AT4G26780	AR192	-0.241556388	0.838271619	-0.25846063	0.753247029	-1.530442468	0.004893201	-0.011082705	0.997539086	-0.426897659	0.277703192	-0.028809249	0.933908479
AT1G17560	HLL	-1.262507881	0.220952922	-1.676720792	0.312474532	-1.530488862	0.034587819	-0.110141791	0.974956709	0.204560986	0.78078663	0.636332644	0.000619269
AT1G13600	bZIP58	-0.334766748	0.786951518	-2.348899973	0.188048231	-1.531210481	0.022934234	-1.031227511	0.822720536	-0.907903035	0.360480945	-0.259103256	0.321346073
AT5G47870	RAD52-2	-0.407504986	0.470490522	-0.55365748	0.197764635	-1.531504156	0.00495589	0.182212187	0.813515173	-0.04595921	0.957156296	0.608810319	0.027309882
AT2G36880	MAT3	-0.957519783	0.178844627	-0.535202491	0.135435446	-1.535400351	5.8621E-11	-0.782647311	0.696374342	-0.421092354	0.001290985	-0.025885812	0.89349372
AT2G38730	AT2G38730	-0.80564299	0.233850145	-0.55781509	0.520388711	-1.536703163	1.29376E-05	-0.081024913	0.983589298	0.046729689	0.966598167	-0.192655599	0.672111195
AT5G15630	IRX6	-1.443218014	0.168232425	-2.967530303	0.391776461	-1.540166888	0.04105013	-0.527161696	0.646729152	0.243186964	0.819229747	0.077150814	0.839723943
AT5G07030	AT5G07030	0.696802209	0.471574792	-0.924865084	0.042629388	-1.540247433	0.000230659	-1.246139878	0.489831977	-0.943473524	4.72674E-08	-0.181495418	0.500747254
AT1G25220	ASB1	-0.850079606	0.139058273	0.283006666	0.469671123	-1.542520718	1.67468E-05	0.16808965	0.951334464	-0.052034778	0.946435828	0.198607407	0.462958548
AT5G59920	ULI3	-0.975359881	0.038647438	-0.796107727	0.03302041	-1.543577654	0.000179484	-0.000105218	0.999880715	-0.727477725	0.27174856	0.132589378	0.747781992
AT1G64670	BDG1	-0.777978553	0.352989235	-0.71821976	0.106126059	-1.546524464	8.53666E-06	-0.526357221	0.714699776	0.235222693	0.507785667	0.316607091	0.227451182
AT5G02080	AT5G02080	-0.054025893	0.968718266	-0.10872803	0.964906388	-1.547810961	0.002085335	0.22757376	0.953774208	-0.351011927	0.799206081	0.608199373	0.113649281
AT4G30330	AT4G30330	-0.766928286	0.359414109	-0.413766085	0.383528829	-1.548185766	5.41135E-06	0.294703193	0.853012539	0.390516386	0.445236462	0.591318201	0.001477325
AT2G17972	AT2G17972					-1.551903095	7.32451E-07					0.196285222	0.679068519
AT5G24760	AT5G24760	-1.117230554	0.22478376	-1.087758952	0.148361128	-1.554466171	0.001881605	-0.375163458	0.893576528	0.174601175	0.677420627	-0.070746041	0.773455043
AT5G39380	AT5G39380	0.477706323	0.639958175	-0.326174088	0.803818195	-1.55905687	0.003537626	-0.390442387	0.714675034	0.130450775	0.886696146	-0.062394928	0.8421914
AT4G36660	AT4G36660	-0.651153499	0.280943528	-0.944467204	0.082122522	-1.563210611	0.021576537	-0.674361596	0.661895574	-0.322389208	0.373165367	-0.097337898	0.760087127
AT3G54710	CDT1B	-1.990458764	0.073182763	-2.702454855	0.051820445	-1.564267672	0.009145498	0.203863762	0.94659229	-0.324855711	0.670885618	0.428730159	0.138617347
AT2G28410	AT2G28410	-0.016247256	0.98735123	-0.746067936	0.275889269	-1.564600559	0.036183078	-0.017447794	0.993804788	-0.030220182	0.976044889	0.365323692	0.313574787
AT4G39040	AT4G39040	-0.918832045	0.015374867	-0.527846533	0.257849355	-1.565225528	4.21715E-12	0.332760026	0.767112256	0.434823874	0.173673356	0.977458489	7.38141E-10
AT1G25375	AT1G25375	-0.917874195	0.226399493	-0.828838989	0.074689293	-1.570956693	1.1941E-05	0.299160473	0.88980598	-0.334615111	0.573984194	0.479012527	0.052664676
AT3G44050	AT3G44050	-1.450279343	0.153846132	-3.935118272	0.251001408	-1.574013793	0.003095238	-0.444465793	0.935083701	0.347227437	0.511253459	0.523562351	0.013700295
AT1G59560	ZCF61	-1.52352944	0.190536872	-1.84177746	0.058766538	-1.574465612	0.020526891	-0.093481888	0.981525175	0.170305055	0.859991285	-0.150624496	0.794715558
AT2G38810	HTA8	-0.832417909	0.241392656	-0.455620147	0.51088817	-1.574910535	3.28243E-05	0.09159986	0.974758564	0.669777109	0.120350378	0.492298881	0.018479642
AT4G30060	AT4G30060	-0.929443956	0.263135636	-0.575468116	0.526591936	-1.576748702	0.009584976	-1.554895183	0.306859729	0.092844272	0.905535933	0.108685211	0.790399188
AT5G17165	AT5G17165	2.753000348	0.192086808	-1.727478704	0.483821862	-1.577150832	0.000223267	0.717555099	0.855975888	0.516764682	0.801085181	0.644278676	0.026785997
AT5G01170	AT5G01170	-0.410261754	0.627158471	-0.37246954	0.51149317	-1.578867405	0.001457871	-0.061314491	0.981658745	-0.169284667	0.810111895	-0.785984066	9.26659E-05
AT1G74940	AT1G74940	0.164186125	0.834725161	-1.026927961	0.096016745	-1.580032817	2.06095E-10	0.175271791	0.809598664	-0.073809959	0.920228005	-0.293294254	0.23313103
AT3G63170	FAP1	-0.564734122	0.170805195	-0.364865546	0.157641	-1.583019603	7.05758E-10	0.019529938	0.990915497	-0.154854683	0.743097639	0.356248259	0.025233017
AT3G03820	SAUR29	-0.606262192	0.464185749	-0.969616909	0.650652096	-1.585997891	0.010062642	-0.84538722	0.79705977	0.490400926	0.56051112	-0.155329586	0.691679796
AT3G59880	AT3G59880	-0.70189592	0.801175614	0.229092649	0.837065693	-1.59032758	0.027650189	-1.014314109	0.832229205	0.270071212	0.846410046	-0.800101105	0.006400497
AT3G53830	AT3G53830	-0.534112764	0.55743614	-0.097589327	0.900102468	-1.590431077	0.000203287	0.840960609	0.693517591	1.850238036	0.217754547	1.133921184	0.133833183
AT5G42330	AT5G42330	-1.05434897	0.382381542	-1.314576821	0.085761739	-1.59394781	0.003618453	-0.597777846	0.92001591	0.377080844	0.560363834	0.42912897	0.032968665
AT5G02260	EXPA9	0.299506512	0.777377414	-0.580967458	0.388891346	-1.596459611	0.001020945	-1.660562199	0.051975957	-0.639143266	0.000951489	-0.491574972	0.227184224
AT2G46210	SLD2					-1.597441345	0.010283893					0.831251443	0.011991828
AT3G26115	AT3G26115	-0.587673744	0.424446991	-0.693822095	0.465032195	-1.60204246	0.041446106	0.685283056	0.71459397	-0.212753624	0.845302268	0.567279772	0.263173567
AT2G27690	CYP94C1	-0.590501063	0.568718111	-1.232688749	0.090032728	-1.603247122	0.015280292	-0.11711696	0.972543651	0.152935784	0.925388746	0.077744723	0.864092702
AT4G00165	AT4G00165			-2.55453064	NA	-1.608556689	1.10599E-08					-0.618657362	0.041669544
AT1G04730	CTF18	-1.727392705	0.052904473	-1.567252394	0.321897729	-1.613993961	9.62192E-05	0.237085534	0.89418557	-0.552297657	0.311915151	0.334145379	0.250289455
AT3G61360	AT3G61360	-0.381261438	0.897363741	-2.836974049	0.229780027	-1.628020621	0.018234893	-0.40623086	0.954137138	-0.499414686	0.735176002	-0.060154436	0.889096661
AT3G59900	ARGOS	-0.961466407	0.042105811	0.04299336	0.970553203	-1.632499913	3.23931E-06	-0.41781494	0.795199419	-0.250597165	0.606522831	0.686369748	0.003266079
AT4G22485	AT4G22485	-3.776306884	NA	-4.265582747	NA	-1.634173001	0.000124857	-4.901903342	0.670807968	4.344469351	NA	0.576568569	0.19344618
AT1G04180	YUC9	-1.333404492	0.064456545	-0.683066016	0.487054444	-1.639808855	0.009004256	0.672712413	0.781016963	-0.841955143	0.790926204	-0.677994136	0.440889428
AT2G04790	AT2G04790	-1.458241835	0.073542377	-1.368942846	0.053508073	-1.641380899	3.194E-09	-0.248052436	0.939540455	0.374463875	0.849024218	0.827218801	0.068490824
AT2G41950	AT2G41950	-0.84043898	0.278195115	-0.57716541	0.377405467	-1.645569409	0.000219217	-0.916606518	0.632278424	0.218139458	0.823440824	0.463269822	0.270412842
AT4G12060	AT4G12060	-0.666564744	0.039255153	-0.308175095	0.26292419	-1.648676121	1.58632E-12	0.381059618	0.691895802	-0.023580786	0.972313832	0.37040817	0.115782912
AT2G31725	AT2G31725	-1.014822372	0.256655647	-1.0085502	0.226856566	-1.651179576	0.011943168	-0.075992307	0.972205981	-0.300383022	0.674271991	0.197821202	0.543045428
AT5G44565	AT5G44565	-4.709992537	NA	1.322232586	0.836001548	-1.653364168	3.86414E-07	-6.846676258	0.558178127	-5.885081928	0.348185596	0.838798301	0.149947935
AT3G14220	AT3G14220	-0.510191075	0.437538618	-0.757129831	0.062486322	-1.660273825	8.73108E-12	-0.577645784	0.652555847	-0.303079297	0.18824835	0.741290072	2.46242E-07
AT4G16515	RGF6					-1.664211233	0.003733255					-0.651965251	0.021762412
AT5G51920	AT5G51920	-0.05065944	0.985508803	0.064709669	0.960170762	-1.665361019	0.01169763	-1.086237091	0.602414093	-0.616339529	0.328764928	-0.714809201	0.016827467
AT2G20835	AT2G20835	-1.282648374	0.050469552	-0.398307489	0.415952464	-1.668541622	1.33462E-05	-1.167536328	0.707962062	0.221682652	0.857737893	0.205796063	0.747625326
AT1G70230	TBL27	-1.081144728	0.066898703	0.201360141	0.788113873	-1.668798424	0.000923634	-0.532782224	0.791359656	-0.539697965	0.1274505	-0.442009367	0.041167205
AT5G45400	RPA70C	-0.867794329	0.523678136	-0.844812861	0.247847565	-1.673504351	0.005721627	-0.06447272	0.985077447	-0.346694035	0.562199149	-0.131443725	0.635919678
AT2G04170	AT2G04170	-1.287759388	0.466305171	-0.56763077	0.741096982	-1.67625521	0.000615599	-0.785478659	0.706790319	-0.646778084	0.583966301	-0.972328821	0.003445364
AT5G02690	AT5G02690	-0.453249778	0.900776822	-0.596445719	0.722227133	-1.679520962	0.0128481	0.486088321	0.876015705	-0.063272635	0.972203917	0.135550545	0.84010635
AT4G31890	AT4G31890	-1.48733012	NA	-0.994417425	0.337223317	-1.685592906	0.001296161	-0.450623876	0.769724647	0.032379438	0.979395501	-0.094736598	0.718310107
AT4G12620	ORC1B	-0.839172805	0.60673625	-1.679358383	0.277259693	-1.68746049	0.03884458	-0.098186048	0.975290905	0.119064448	0.915825933	-0.043235496	0.901389644
AT4G21850	MSRB9	-0.137738984	0.962387552	-0.621725279	0.503620903	-1.689493927	0.011058343	-1.010505532	0.616300683	-0.673019524	0.010837232	0.311460968	0.02506924
AT3G14900	EMB3120	-0.430977961	0.752265289	-0.235830736	0.867188522	-1.696742121	0.000218489	-0.184395587	0.963341405	0.119936036	0.906635824	-0.12913629	0.691548955
AT2G14115	AT2G14115	0.483652781	0.686911772	-0.38766711	0.78547714	-1.702611538	2.45737E-05	-0.288306476	0.898391389	0.28419068	0.781137697	0.115752986	0.836522625
AT5G52950	AT5G52950	-0.937406886	0.523178176	-2.213501215	0.108846973	-1.708117966	0.02669274	0.166263544	0.982055475	-0.334272574	0.702677159	-0.109294184	0.82856522
AT3G24590	PLSP1	-0.731141561	0.061304859	-0.935349837	0.012733417	-1.708408044	1.59569E-19	0.226105212	0.824370324	-0.020010356	0.982621649	0.444404526	0.016011898
AT3G38400	EXLA2	-0.354495686	0.504466215	0.028271195	0.972613524	-1.709045254	8.67296E-05	-0.469165956	0.691824202	-0.574475331	0.06162656	0.562268844	0.060647576
AT3G31950	AT3G31950					-1.711241406	0.015610499					0.359939076	0.528358783
AT2G40475	ASG8	-0.808778437	0.052109814	-0.709953759	0.262911307	-1.715191338	0.024343572	-0.539782254	0.680482607	-0.099526032	0.915699339	-0.30821721	0.186750845
AT1G51405	AT1G51405	-0.916794523	0.266811404	-0.790326814	0.402253175	-1.718204518	0.000725008	0.009601568	0.999006241	0.762903958	0.461651524	-0.146756753	0.79941631
AT3G09050	AT3G09050	-1.320996464	0.088246376	-0.30869139	0.82588512	-1.718970695	2.96825E-06	1.333676283	0.427749503	0.110430117	0.964601576	0.339908115	0.146131812
AT1G44780	AT1G44780	-0.221664409	0.846391977	-0.777612226	0.2155141	-1.721567208	0.022303471	0.390131061	0.881491928	0.226132578	0.781706477	0.205889606	0.500674139

AT4G27030	FADA	-2.200335367	0.426239719	-0.713584921	0.301638458	-1.721991694	0.010051078	-0.560823296	0.899858481	-0.964224561	0.509840734	-1.004622192	0.697137175
AT5G03140	AT5G03140	-0.956805644	0.033485531	-0.758117033	0.296603245	-1.723381022	3.58907E-06	-0.596650359	0.702246294	-0.499890166	0.335733161	0.140593949	0.697010519
AT5G39900	AT5G39900	-0.210660509	0.891136818	-0.577478594	0.611044284	-1.723566385	0.000750612	-0.756871318	0.640704059	0.488725154	0.442615001	-0.319415341	0.204409562
AT3G08660	AT3G08660	-0.845293507	0.27642362	-0.970175102	0.070591466	-1.733266177	0.002681182	0.244197374	0.902565251	-0.052275187	0.979990818	0.086237283	0.855091071
AT1G59960	AT1G59960	0.132901234	0.90534663	0.096162146	0.906694979	-1.734827092	0.002300261	-0.37229628	0.876082394	0.005127073	0.996204637	0.373035777	0.037627233
AT5G44720	AT5G44720	-0.483810884	0.564982204	-0.65022432	0.232455511	-1.758074152	4.5615E-09	0.019189774	0.996799452	0.150372737	0.672984449	0.408307943	0.002820219
AT1G30600	AT1G30600	-0.744851766	0.398485226	-0.988903579	0.208790916	-1.762039192	0.000733572	-0.2006363	0.918988754	-0.313075171	0.577315828	0.642488598	0.000296831
AT1G68110	AT1G68110	-0.856518179	0.124759446	-0.21790611	0.800266246	-1.766652434	0.000410095	0.534433942	0.611226747	0.181906805	0.794004103	-0.14719702	0.708818341
AT2G23560	MES7	-1.099008014	0.309427609	-2.869178558	0.053816662	-1.770355755	0.002347955	-1.395665938	0.718989301	0.416019689	0.56302106	-0.190126712	0.627463234
AT3G28130	UMAMIT44	-0.744220084	0.297433663	-1.261762494	0.15019317	-1.770884678	0.000114682	-0.454639225	0.841624938	0.087964602	0.904202655	0.90181898	0.001690493
AT3G27290	AT3G27290	-0.477415346	0.793740977	-3.27817568	0.207949283	-1.771472356	0.008539179	-1.777256923	0.680482607	1.089539656	0.817199908	-0.410989223	0.407699171
AT4G22580	AT4G22580	-0.775990757	0.223811421	-0.6377295	0.307449354	-1.779578959	0.000352816	-1.578509208	0.1690382	-0.765561677	0.056465658	-0.612816812	0.025599717
AT2G26180	IQD6	0.090489337	0.956335774	-1.172698115	0.5402902	-1.781973705	0.033410664	0.335557598	0.855081587	0.650510428	0.375143623	0.465109554	0.085812192
AT5G46440	AT5G46440	0.059103572	0.98375039	-0.755244217	0.693164317	-1.784030033	0.011039912	3.31280129	0.256706039	0.541117609	0.866906715	-0.176952171	0.936532016
AT1G01390	AT1G01390	0.49674791	0.763107038	-0.464283697	0.709392243	-1.784147891	0.028990866	-1.220923066	0.769724647	-0.086548163	0.947746874	-0.688745074	0.013401329
AT1G25510	AT1G25510	-0.408948343	0.798361804	-2.932313771	0.080096155	-1.785116839	0.017973088	-0.489661486	0.936447266	-0.477971764	0.249123812	0.057358876	0.863669815
AT4G03010	AT4G03010	-0.554541028	0.505411334	-0.403858005	0.677835326	-1.794073136	0.022618489	0.089169951	0.977607985	-0.615950261	0.394417791	-0.011394146	0.982443856
AT3G43960	AT3G43960	-0.575062773	NA	-1.210955104	0.109788228	-1.800768775	0.004189752	-1.073091262	0.584219873	0.048656608	0.954114031	0.142214757	0.609742733
AT2G30960	AT2G30960	-1.445008804	0.161706693	-1.443908897	0.167930127	-1.80243766	0.007641514	0.403335776	0.867998738	-0.376860251	0.77337674	0.076989786	0.876613304
AT2G40330	PYL6	-1.348491127	0.159620695	-1.652101298	0.162079904	-1.809176777	1.2653E-06	0.488354499	0.940208794	-0.602439756	0.52315165	-0.12765428	0.80125764
AT2G39220	PLP6	-0.909775875	0.170052407	-1.14876838	0.102388559	-1.822650574	0.006447646	-0.515970745	0.804084932	-0.322844398	0.502091749	-0.969367298	7.71421E-08
AT3G10120	AT3G10120					-1.822912364	4.10583E-05					-0.318470424	0.407219504
AT2G01755	AT2G01755					-1.826698892	7.2346E-05					0.385245716	0.385542929
AT3G09540	AT3G09540	-1.556220193	0.091903705	-1.084169973	0.236790914	-1.829731855	0.003570999	-0.669152746	0.680482607	-0.563245899	0.565321135	-0.526924411	0.101765603
AT2G42760	AT2G42760	-0.542715573	0.352642997	-0.658342602	0.403256567	-1.829884499	3.70985E-05	0.51189444	0.74551082	0.103319204	0.944342934	0.403242984	0.308861171
AT4G21740	AT4G21740	-1.281756287	0.07842847	-0.370627809	0.700271184	-1.836816699	0.002385342	-0.721934135	0.768961401	0.127269074	0.915825933	-0.143577393	0.731884623
AT1G63100	AT1G63100	-1.044626994	NA	-4.243896482	0.189553019	-1.84165204	0.010432046	0.432459335	0.800906229	0.371906482	0.613826202	0.688319192	0.000556066
AT1G63400	AT1G63400	-0.989544759	0.334175913	-1.339401284	NA	-1.84689241	0.023613942	-0.785264606	0.691895802	0.311087163	0.806371006	0.162261383	0.785804633
AT3G58520	AT3G58520	0.498751786	0.711951164	1.082798546	0.26145859	-1.856980961	0.036068738	-0.424060213	0.879859511	0.069314728	0.95946592	0.166466286	0.656498794
AT1G28110	SCPL45	-1.109671126	0.094164935	-1.483834274	0.079815394	-1.857719954	0.00864897	-0.47114487	0.729344009	-0.476905646	0.151499956	0.181562183	0.460199112
AT4G39510	CYP96A12	-1.124199333	0.415535579	-0.847667501	0.294664401	-1.887783389	5.17539E-05	-0.034386454	0.993482731	0.32576011	0.617074584	0.597790818	0.005216304
AT1G67690	AT1G67690	-1.249509046	0.082455579	-1.432659463	0.059868574	-1.888521085	6.66881E-06	0.038224048	0.982749202	-0.167892358	0.851437944	0.469468286	0.019318417
AT5G63410	AT5G63410	-0.577654994	0.284808697	-0.926910248	0.03604322	-1.901719319	9.12295E-07	-0.769612194	0.616300683	-0.111104778	0.822587771	0.017489811	0.944510228
AT1G05210	AT1G05210	-1.271752159	0.55026332	-3.154176791	0.121037483	-1.907775383	0.014043311	-0.321377802	0.781888051	0.117266093	0.91956887	0.685895194	0.018939398
AT2G35210	RPA	-2.691944201	0.138758104	-3.321773906	0.128558961	-1.907924784	0.000923132	0.04643561	0.983589298	-0.947149401	0.199674929	-0.274658848	0.619764551
AT5G18475	AT5G18475	-0.839827884	0.356930172	-0.66314161	0.534069834	-1.910117015	0.001294765	-0.014676446	0.997796217	-0.243601795	0.905953395	0.090831753	0.865831576
AT5G03800	EMB175	-0.921792198	0.22376913	-0.935500964	0.199907996	-1.914719516	0.000171504	0.011578093	0.996799452	-0.321278185	0.632768672	0.01478899	0.97377227
AT2G38750	ANNAT4	0.339166362	0.741359224	-1.440306814	0.074852666	-1.915938212	0.000223993	-0.591172087	0.759702709	-0.017355258	0.978047702	-0.154424048	0.496718601
AT4G23790	TBL24	-0.907229796	0.238620059	-0.865994702	0.091014132	-1.928796121	5.23523E-05	-0.610670949	0.739946644	-0.217020953	0.66415299	0.288223145	0.092124214
AT1G63230	AT1G63230	-1.731690946	0.404907451	-0.168161372	0.884667101	-1.932930361	0.038445551	-0.797816834	0.875303851	0.252836244	0.888274101	0.394644342	0.596075422
AT1G74030	ENO1	-0.907753521	0.349119044	-1.193285723	0.297370149	-1.93506597	1.20054E-05	-0.823430077	0.765335087	-0.170106559	0.647824775	0.229222793	0.285680999
AT4G37450	AGP18	-1.313334046	NA	-0.95312317	0.210248346	-1.94184622	0.000519367	-1.171886331	0.550996121	-0.595803815	0.008495937	0.626759624	0.001051102
AT2G42110	AT2G42110	-1.000257338	0.460005333	-0.628115912	0.76685235	-1.947775375	0.021420964	-0.694332945	0.898391389	0.181209217	0.848060883	0.513819137	0.050450126
AT4G28250	EXPB3	-1.266004217	0.607762727	-0.752938463	0.378941793	-1.948249765	0.000306223	-0.955806953	0.673165787	-0.609040107	0.021121518	-0.944825948	1.48481E-06
AT4G04610	1-4月	-0.722766978	0.576982832	0.96826371	0.145496955	-1.948729807	0.002560495	-0.062086001	0.984889186	-0.684418553	0.026219667	-0.442497996	0.109045959
AT4G15070	AT4G15070	-1.631202563	0.15828273	-1.982543251	0.409530167	-1.949336899	0.000410568	-1.607077377	0.691895802	-0.566765193	0.624504205	-0.071018557	0.898091156
AT5G61200	AT5G61200	-0.989116196	0.232017714	-1.196471144	0.06421101	-1.954892771	1.31803E-06	-0.265156548	0.820136096	-0.121693451	0.913903461	0.050054703	0.897135004
AT3G52720	ACA1	-1.116854142	0.133271315	-0.986719441	0.093278908	-1.969411122	3.15687E-08	-0.754782708	0.680595832	-0.51108031	0.085472556	0.181441885	0.31061269
AT1G11740	AT1G11740	-1.136378156	0.101496146	-3.307495827	0.091500968	-1.970236561	3.6053E-05	-1.000645247	0.63192307	-0.383649528	0.613589516	-0.48358285	0.37717459
AT3G23635	RTFL13	-0.635855837	0.711127434	-1.788672936	0.648161757	-1.970718937	0.006021522	-1.743535413	0.674239406	-0.26696609	0.884432792	-0.053637312	0.936676881
AT4G10380	NIP5;1	1.18162279	0.355668119	-0.066354442	0.96897281	-1.978863303	0.01321753	-0.78468701	0.876561915	-0.954151874	0.052902597	-0.623384623	0.075821684
AT4G31500	CYP83B1	-0.451807438	0.485289524	0.502762977	0.411854363	-1.979819233	1.10457E-09	-0.554885934	0.740531841	-0.965958107	1.01436E-05	-0.993586799	1.51308E-12
AT4G18290	KAT2	-1.839719182	0.059288128	-2.294954948	0.358211492	-1.980225633	0.000215198	0.374039896	0.894170799	-0.345621657	0.660372631	0.99142501	0.043643298
AT1G21810	AT1G21810	-1.377283081	0.178844627	-1.373161882	0.107233532	-1.986356571	0.043460535	1.277716627	0.500463801	1.032355641	0.195022122	0.116971797	0.841891339
AT1G70950	AT1G70950	-0.588006169	0.362686461	-1.111167924	0.059308458	-1.993441145	2.75547E-08	0.436229892	0.662219342	0.338437115	0.563990873	0.466744246	0.057382525
AT3G01330	DEL3	-1.573501212	0.193346856	-1.144600478	0.522284766	-1.994924961	0.019405422	-0.472395593	0.859699136	0.649758481	0.431056406	0.116971311	0.780103696
AT3G52900	AT3G52900	-0.572719844	0.697824416	-1.405928657	0.131892269	-1.997815226	5.87396E-05	0.359160419	0.855010607	0.154470663	0.855614411	-0.599584808	0.027582536
AT3G22820	CLL1	0.16447961	0.941119586	0.005321088	0.998934353	-2.000039973	0.008451021	-0.105342614	0.990231864	-0.249376426	0.924938189	-0.043921319	0.947765748
AT3G09190	AT3G09190	-0.984033336	0.175165722	-0.633987042	0.554578103	-2.008663866	1.93434E-05	-1.609295516	0.436431133	0.840509219	0.242136082	-0.398320539	0.14689295
AT5G03995	AT5G03995	-1.468160636	0.650145462	-1.910152662	0.248377316	-2.009047581	0.000846342	-0.84574883	0.872669065	-0.082237561	0.966726249	-0.458782919	0.159206407
AT3G43800	GSTU27	-0.343048903	0.59185943	-0.654987857	0.377091042	-2.014026058	7.14602E-05	-0.841612051	0.706790319	-0.426508721	0.148740697	0.017290066	0.966991873
AT1G76310	CYCB2;4	-0.432151627	0.768965277	-3.186796045	0.391291772	-2.039521766	0.000612926	0.426083192	0.910428643	0.316917337	0.599501347	0.682674689	0.002589528
AT1G35530	FANCM	-2.869060966	0.122685542	-1.725441126	0.369400893	-2.043472793	0.02069436	-0.076342019	0.992231761	-0.522806966	0.625207384	-0.04572283	0.919757157
AT3G03060	AT3G03060	-0.995007088	0.252894521	-0.244074307	0.794906238	-2.044113242	0.000139919	-0.738927952	0.809502311	-0.11435527	0.884397165	-0.256358688	0.455336167
AT4G00110	GAE3	-0.176766494	0.810161239	-0.651096906	0.259322386	-2.046593931	5.34625E-05	-0.735285119	0.600201073	-0.574976966	0.172338689	-0.50767846	0.071080151
AT4G20362	AT4G20362	-0.046583369	0.983079283	-2.994812105	0.204917095	-2.065310426	0.010200636	-1.73984003	0.867112468	-0.354364685	0.891280709	-1.265457934	0.188482516
AT3G19040	HAF2	-1.134059647	0.352582795	-1.70580206	0.130116356	-2.068722765	0.000113834	-0.103067896	0.982664567	-0.044976575	0.989242512	0.638559092	0.14689295
AT3G25110	FaTA	0.469094874	0.702179348	-1.794931702	0.273542609	-2.087245224	0.013878891	-1.000642909	0.696374342	-0.068259734	0.927655438	-0.627560511	0.000208442
AT5G57970	AT5G57970	-0.171270435	0.920787057	-1.887733478	0.244833478	-2.091872973	0.006212411	-0.448738102	0.822720536	-0.080176709	0.910533024	0.081862793	0.788423251
AT2G18650	MEE16	-0.21537068	0.847337742	-2.412603217	0.271714527	-2.094496397	0.003026756	0.328054205	0.919978446	-0.055419758	0.977360449	0.298187013	0.578543995
AT5G39980	AT5G39980	-0.6048757	0.520336226	-1.332336215	0.106029083	-2.104969451	8.50777E-10	0.598489983	0.770575751	0.217995178	0.804786811	0.366073445	0.290317802
AT3G16670	AT3G16670	-0.690625695	NA	-1.59355576	0.310414954	-2.118403309	9.42927E-05	-2.556370917	0.598901706	-1.561167674	0.329900262	0.881986645	0.000105865
AT4G24710	AT4G24710	-0.083508729	0.964705665	-1.813535803	0.287163991	-2.12046872	0.008641412	-0.172825002	0.964169173	0.525463362	0.496377799	0.613935838	0.023610832
AT3G46110	AT3G46110	-1.844515605	0.324945576	-0.718654937	0.553456971	-2.125874429	0.009828925	-1.345620808	0.433558429	-0.535903693	0.625031289	0.172161205	0.694806823
AT4G33925	SSN2	-0.378667394	0.798876559	-0.912281826	0.493691935	-2.136582677	0.007630446	-0.859112447	0.864045341	0.508676131	0.638949843	0.751222268	0.089812207
AT2G41312	AT2G41312	-1.401489845	0.129114468	-1.361304596	0.320073222	-2.139673492	0.004758498	-0.583054786	0.923573229	-0.547482702	0.618760168	-0.748618346	0.09644703
AT3G18485	ILR2	1.172343657	0.821486767	0.505281229	0.828102683	-2.142612941	0.001790318	5.067103064	0.664635521	-0.415508116	0.924491567	-0.024059726	0.988233344
AT3G05800	AIF1	-0.573510978	0.357610108	-0.593236575	0.21488835	-2.153876218	1.10227E-05	-0.519338197	0.646729152	-0.109931881	0.906127081	0.065631391	0.890674644
AT1G77310	AT1G77310	-0.9321385	0.344087888	-0.829413993	0.420953117	-2.154059315	0.000411367	-0.447876019	0.76192737	-0.195190767	0.870543261	0.088647423	0.834374862
AT1G08280	AT1G08280	-0.608043552	0.522439167	-1.721239721	0.061130612	-2.157127989	4.18029E-05	-0.554772011	0.847546649	-0.014578906	0.988079805	-0.347322234	0.138672097
AT4G18460	AT4G18460	-1.234666906	0.273891974	-1.446366467	0.118494545	-2.169889235	0.001419831	0.035001099	0.996799452	0.287571899	0.852137227	0.473992241	0.234360003
AT1G08810	MYB60	-0.030916174	0.975672634	-0.203639005	0.745036256	-2.174523357	1.83753E-11	1.057188959	0.137572045	0.534382498	0.433732558	0.334969587	0.590373919
AT1G64450	AT1G64450	-2.369807659	0.228941002	-0.605419979	0.725066617	-2.194720766	0.000212355	-0.75666665	NA	-0.410328982	0.779424422	0.093832804	0.759638049
AT5G54970	AT5G54970	-1.587345392	0.11375427	-2.018803834	0.187847071	-2.194868829	0.000454039	-0.559362416	0.924925439	-0.483997316	0.15041691	-0.044997612	0.89749786
AT1G11440	AT1G11440	-0.394434077	0.591454051	-0.691528948	0.201293418	-2.209428585	3.68939E-05	-0.093790586	0.982356953	0.036519012	0.971701608	0.084862671	0.877669563
AT2G36200	AT2G36200	-1.393330352	0.098805924	-2.071994351	0.432271637	-2.218523939	1.63117E-12	-0.167943464	0.954309326	0.141765612	0.84792397	0.448341323	0.006827827
AT1G33170	AT1G33170	-1.511677348	0.076128947	-1.120666965	0.166408792	-2.219166656	7.72004E-12	-0.836515697	0.637815859	-0.285718686	0.36223266	0.490401665	5.09328E-05
AT1G55330	AGP21					-2.226147577	1.11213E-12					-0.154673245	0.455592845
AT5G66620	DAR6	0.403466981	0.717329136	-0.662519896	0.637672608	-2.230769076	0.006833622	-0.532376223	0.831610681	-0.468757331	0.41344799	-0.397527249	0.144624974
AT4G30290	XTH19	0.377682864	0.88580183	-0.771235588	0.463649349	-2.2313063	2.54582E-08	-1.715644441	0.245156267	-0.641527653	0.206811617	-0.259573832	0.080986233
AT3G20470	GRP5	0.446998334	0.684082936	-1.675679373	0.321897729	-2.237327679	0.000677989	-1.477479324	0.470078997	-0.439982085	0.119428864	-1.760534365	0.160344941
AT1G72270	AT1G72270	0.224916042	NA	-3.398939027	NA	-2.239910444	1.22248E-08			3.529999565	NA	0.530750009	0.11251502
AT5G42280	AT5G42280	-1.710269771	0.076630816	-0.40907404	0.529185602	-2.252274983	0.003494583	-0.645243249	0.735695447	-0.54454087	0.241900987	-0.148653308	0.746120302
AT3G48490	AT3G48490	-1.698557043	0.156201268	-0.75386718	0.709190436	-2.252732249	0.000244831	0.167749598	0.972495118	0.497622838	0.496280372	0.166367755	0.743309994

AT1G29440	SAUR63	-3.239697439	0.200316216	-7.086084567	0.068524855	-2.256750199	0.001007222	-0.183931929	0.988042725	2.846411827	0.388772141	-0.694725771	0.095479154
AT3G60440	AT3G60440	-1.37769816	0.225401234	-1.055663422	0.242773026	-2.283926447	4.09685E-05	-0.595288394	0.807595594	-0.315386755	0.541116807	-0.015472694	0.963037931
AT1G11700	AT1G11700	-0.504766255	0.582746251	-0.61840151	0.500671611	-2.286588052	5.94364E-06	-0.628852258	0.854655022	0.240616128	0.878729263	-0.676721743	0.105094308
AT1G18370	HIK	-1.257685518	NA	-3.482542426	0.085936394	-2.299992774	0.000848461	-0.135654769	0.968587518	0.454454977	0.363849408	0.560095571	0.001844183
AT1G32780	AT1G32780	-1.055988389	0.337851772	-0.805546059	0.596181063	-2.338433614	0.002495686	7.246313573	0.465456099	0.749950877	0.920279543	1.009086599	0.648256087
AT1G59780	AT1G59780	0.453668492	0.881154347	-6.481296573	0.055832004	-2.358533788	9.87311E-07	0.394693447	0.844544379	-0.424917737	0.814006716	0.292294019	0.718309329
AT2G47560	AT2G47560	-2.296162106	0.24010363	-1.453424993	0.706803985	-2.377125895	0.009522044	-0.945500754	0.923573229	0.576218088	0.919937283	-0.006156278	0.994167104
AT3G20130	CYP705A22	0.547087713	0.544195095	-0.405664632	0.643884043	-2.394178882	1.94072E-16	-1.159965873	0.150384452	-0.544072333	0.281935652	-0.326341379	0.159012352
AT1G24280	G6PD3	-1.888214214	0.12894512	-0.119974437	0.918234866	-2.420178189	0.000178388	-0.41286685	0.832523868	-0.621707914	0.058639925	-0.183149021	0.444829835
AT2G36690	AT2G36690	0.139235328	0.930035011	0.517909489	0.813933451	-2.423502123	0.008680204	0.082480497	0.981414167	-0.127843561	0.929239543	-0.915775087	0.025212218
AT2G26440	AT2G26440	-1.193906915	0.729064396	-0.966846337	NA	-2.441237318	0.00013678	-0.723284715	0.634717017	-0.541409946	0.148747654	0.713699717	0.015788173
AT2G37380	MAKR3	-1.451526286	0.354886098	-2.922830187	0.174727181	-2.459148333	0.001170659	-0.704398408	0.905404361	-0.238600166	0.883796441	0.590899405	0.226117001
AT4G19560	CYCT1;2	-3.118671002	0.058446162	0.201636512	0.918671643	-2.462952346	0.004234444	0.903129574	0.721140128	-0.459711566	0.900609444	-1.097134399	0.174621292
AT1G18250	ATLP-1	-0.642571557	NA	-2.986074663	0.171022352	-2.470367895	5.23783E-08	-0.378293241	0.917097586	-0.273523238	0.605215813	0.571351347	0.002107466
AT5G11300	CYC3B	-1.536721854	0.169040523	-1.571166553	0.104615608	-2.492925577	0.001524495	-0.546734301	0.853012539	-0.358016097	0.562717684	0.267245814	0.459904505
AT3G09780	CCR1	-1.397258505	0.088640261	-2.591066238	0.083503872	-2.494082293	6.66525E-07	0.186146287	0.932748047	-0.46937439	0.344315749	0.448829587	0.034208209
AT3G05740	RECQI1	-1.650723031	0.721611887	-1.609884983	0.369859319	-2.50711178	0.037293716	-0.143150115	0.964544073	-0.408060613	0.671669608	0.117548495	0.723755447
AT5G56580	MKK6	-0.663695182	0.622189527	-2.184957739	0.139380177	-2.50950377	0.000141855	-0.301665715	0.965708043	-0.180487892	0.91048199	0.663176153	0.004052712
AT2G17630	AT2G17630	-0.202075097	0.917538108	-0.807183237	0.28739423	-2.527146751	2.95091E-05	-0.71828854	0.638094248	0.163392726	0.752244106	0.134921385	0.492328013
AT2G17442	AT2G17442	-2.074636969	0.229241569	-2.258576606	0.331621077	-2.527599802	0.011438159	-0.57210771	0.926831771	1.034636321	0.254365422	0.555467248	0.103817135
AT1G78530	AT1G78530	-1.456616794	0.470808608	-2.628647288	0.233560559	-2.529155451	0.002955821	0.776388096	0.816446062	0.093862108	0.963419708	0.00044324	0.999727213
AT2G31730	AT2G31730	-1.135209825	0.23284432	-2.042196556	0.409879425	-2.531204744	1.19428E-09	-0.388065167	0.83883166	0.034245461	0.958669515	0.009209468	0.980083634
AT1G31290	AGO3	-1.041200921	0.086135306	-0.736197697	0.196376788	-2.560276754	0.019310325	0.70732024	0.721140128	-0.307098383	0.801085181	0.872955131	0.246520055
AT5G07460	PMSR2	-0.434180612	0.671424423	-1.088540871	0.148226935	-2.574773233	7.39703E-09	-0.130191294	0.967291937	-0.522610909	0.218351867	0.097914618	0.631243262
AT4G16980	AT4G16980	2.437190367	NA	-5.663910688	0.286906319	-2.577730608	2.15477E-28	-2.607242633	NA			-0.026984625	0.938476233
AT2G31141	AT2G31141	0.423723054	0.880998472	-1.231350485	NA	-2.582064223	0.047813422	0.183399008	0.97003735	-0.452678846	0.739960145	-0.155583091	0.857960784
AT3G02820	AT3G02820	-0.926003913	NA	-2.305777334	0.220207776	-2.584635759	1.42331E-08	0.016040596	0.996262282	-0.016211589	0.990969141	0.562561422	0.017356195
AT3G27200	AT3G27200	-0.947981051	0.421540379	-1.363014566	0.099914359	-2.596152241	0.029452955	-0.545997875	0.871661081	-0.52546839	0.376736667	-0.762614476	0.05370702
AT2G23590	MES8	-5.998919405	0.287463058	-6.409398466	0.170520063	-2.598066538	0.00677099			-6.861417308	NA	-1.606041426	0.439826612
AT2G40150	TBL28	-1.120519997	0.204738344	-1.542876567	0.384913336	-2.603650402	0.006430916	-0.739796037	0.71661163	-0.061867943	0.961864611	0.486746379	0.074159533
AT5G61000	RPA70D	-1.685739645	NA	-2.22907051	0.136724088	-2.608530658	0.025545615	-0.673850634	0.759046954	-0.051820725	0.951609136	0.054077563	0.834081601
AT3G15540	IAA19	-1.135790601	0.108523682	-2.085446178	0.414887118	-2.630789037	7.10365E-07	-1.0299492	0.704225272	-0.40999346	0.359502101	-0.883059269	0.08622694
AT3G25030	AT3G25030	-0.22240071	0.847047544	0.070085678	0.948240802	-2.640387199	0.028525161	0.334930522	0.915230329	0.462749788	0.544383104	0.141560163	0.698377023
AT1G71890	SUC5	-2.563245269	0.15343197	-1.731669385	0.378636971	-2.660191126	0.000688145	-0.064760709	0.990383459	-0.531599124	0.81276874	-0.562621478	0.131220708
AT3G50760	GATL2	1.378103625	0.255349455	0.948236292	0.380135332	-2.675006819	0.000857012	-0.986272082	0.393926565	-0.261147354	0.831138261	0.265865881	0.371146113
AT3G20150	AT3G20150	-1.723186584	0.101815334	-2.840319261	0.197546725	-2.678630976	2.31236E-06	-0.575658787	0.921148743	0.144764826	0.87530671	0.56218433	0.006813607
AT2G26360	AT2G26360	-0.5977438	0.871417073	-2.34620457	0.159736403	-2.703427261	1.6685E-07	-0.186033021	0.955307481	-0.527696933	0.591264783	0.031065354	0.938293102
AT2G43140	AT2G43140	-1.247520004	0.344787316	-1.237169784	0.69332934	-2.714157014	4.24101E-05	0.971649065	0.746623995	0.098307723	0.951707362	0.407865812	0.127334432
AT5G43250	NF-YC13	-2.198468632	0.317774089	-1.889206622	0.23722505	-2.724128451	0.03525085	-0.249780316	0.972402645	0.096372831	0.958969891	-0.12819881	0.789006341
AT5G45220	AT5G45220	2.704285574	0.559608451	-5.267203204	0.32516967	-2.73043316	0.028806898	-0.005238861	0.99972467	-3.68464722	0.099161951	-1.625322284	0.48442709
AT1G30370	DLAH	-0.094152338	0.955497432	0.457481185	0.75746763	-2.731916514	0.048978294	-2.707679036	0.641679337	-0.880920885	0.659627538	0.19754244	0.786091796
AT5G25140	CYP71B13	0.898760019	0.253173613	-2.335891058	0.143934328	-2.743813484	3.52371E-07	-0.203837238	0.94592221	-0.005686508	0.996483653	-0.88075173	0.000130074
AT2G01505	CLE16	-1.290925082	0.133597889	-1.10297854	0.616956307	-2.745123872	0.035440452	0.106214923	0.972043985	0.989564333	0.310782919	0.639733569	0.073845484
AT4G14750	IQD19	-0.361267893	NA	-2.131608506	0.163939006	-2.788078696	3.14814E-05	-0.943359302	0.554617999	-0.158333361	0.780041212	0.161890246	0.52276044
AT2G25220	AT2G25220	-1.180955808	0.217906787	-3.158919754	0.1245955	-2.802913911	0.011384722	-1.03467093	0.446425625	-0.743068921	0.236448885	-0.401739971	0.130153226
AT5G56610	AT5G56610	-0.238393544	0.934079045	-0.741918137	0.617409574	-2.808825933	0.001748874	0.251825236	0.947535842	0.415474024	0.712688665	-0.165767151	0.679259032
AT1G67750	AT1G67750	0.929903188	0.58753882	-0.927153767	0.639766979	-2.81506398	0.0270834	-1.841476732	0.267160791	-0.282900736	0.263088771	0.015564055	0.940118342
AT5G50990	AT5G50990	0.329662894	0.879443097	-1.754023312	0.251660551	-2.83836099	0.021022873	0.227894625	0.943633329	-0.133556947	0.940108495	0.672380164	0.131934508
AT5G49800	AT5G49800	-0.264989507	0.861092081	-1.476656512	0.164361842	-2.853636318	0.000110024	-1.054997438	0.807059483	0.004095578	0.996678199	0.128085994	0.669658345
AT1G28610	AT1G28610	-1.188356281	0.636502454	-0.648219424	0.707228124	-2.860452518	0.011196732	-0.102482278	0.991559366	0.801570006	0.81099022	-2.146053396	0.067482578
AT3G48320	CYP71A21	-1.952762619	0.363861928	-0.926179689	0.742201477	-2.864929357	0.008555169	-5.305613518	0.204594112	-2.858692768	0.635351512	0.125898182	0.936272977
AT1G60460	AT1G60460	-1.528466064	0.564956791	-1.630159715	0.577422004	-2.871047683	0.011813844	0.004116469	0.999867318	1.421750111	0.649499096	0.721000518	0.265402875
AT5G10390	AT5G10390	-0.928148544	0.41588113	-1.951930785	0.191120067	-2.88126256	1.79698E-28	-0.192137299	0.983989749	0.042040545	0.962056972	-0.047753708	0.856911153
AT5G14330	AT5G14330	-1.961806302	0.6002829	-0.769524286	0.734268289	-2.891762068	0.038208466	-2.871065167	0.346674285	-1.275165099	0.218754081	-1.049228003	0.119623366
AT4G22840	AT4G22840	-1.326593899	0.08834116	-3.392892161	0.31292573	-2.899047373	0.000164188	-0.805606718	0.626508471	-0.289702018	0.78078663	0.417450988	0.193778908
AT2G37090	IRX9	-1.839173942	0.364090592	-2.720585394	0.23929702	-2.919095243	0.019358509	-1.254131604	0.787101126	-0.938588922	0.112936963	-0.774713229	0.019065459
AT3G46650	AT3G46650	1.610145999	0.814886269	7.256166413	0.157408909	-2.929061198	0.032558686	-2.91652329	0.774188277	5.348132286	0.094442886	-0.810777045	0.192633275
AT1G09600	AT1G09600	0.068690459	0.98405443	-2.535063571	0.5202835	-2.937613134	0.035303506	-1.942570791	0.857125748	-0.766343951	0.881804712	0.395370241	0.492546195
AT1G72230	AT1G72230	-1.279774239	0.229264586	-1.223943078	0.080880783	-2.938200029	2.08281E-09	-1.216895761	0.590788206	-0.571148874	0.028086771	-0.399803057	0.073779089
AT3G52430	PAD4	-1.130839245	0.187507845	-0.85511856	0.235076905	-2.971841706	0.001576265	-1.260953371	0.611321113	-0.689057393	0.312942649	-0.394345062	0.279821152
AT4G25110	MC2	-0.237596142	0.937687453	0.425457875	0.85383281	-2.983061425	0.000231715	-1.572884579	0.768684787	-0.848747643	0.492826398	-0.506250821	0.450383987
AT4G02420	AT4G02420	-3.598555173	0.311719019	-0.953120873	0.370801393	-3.00499664	0.008520369	-0.281250717	0.972495118	-0.709553897	0.842628389	1.133878678	0.055955367
AT2G28140	AT2G28140	-2.762681523	0.43858637	-1.292384777	0.663959958	-3.052078768	0.048277508	-0.519027229	0.936758504	-0.23522004	0.867775012	-0.02396839	0.972388595
AT3G55290	AT3G55290	-0.725660444	NA	-0.954203245	0.628971413	-3.055569062	0.037447207	-0.776484296	0.885755209	0.322015443	0.82028988	-0.842536601	0.003470611
AT5G06480	AT5G06480	-1.080072964	0.475159207	-2.168790819	0.218316191	-3.062120616	0.005901662	0.707475826	0.712887303	0.640207927	0.649472568	0.438821685	0.351667472
AT2G19690	PLA2-BETA	-0.599770336	0.819892089	-1.430106871	0.465823866	-3.077679476	0.011450517	0.872768325	0.653363031	-0.800604438	0.630912609	0.469091766	0.407727595
AT1G65710	AT1G65710	-0.626844757	0.651848527	-2.737126447	0.059742073	-3.101429194	0.002771542	-0.56150099	0.924835736	0.017667329	0.989242512	0.488560826	0.091051251
AT1G10520	Pol{lambda}	-0.439518146	0.743662981	-0.887627655	0.463024656	-3.134686067	0.043374027	0.265272548	0.972077881	0.338024563	0.770008565	0.095088996	0.876146783
AT5G03360	AT5G03360					-3.17936493	2.546E-13	-4.451079662	0.696374342			0.079904406	0.875299673
AT3G18510	AT3G18510	-0.005923745	0.998421433	0.84950323	0.57146732	-3.194876786	0.020555657	-0.151308494	0.957721682	-0.963750136	0.284359663	-0.277787181	0.507141996
AT1G29110	AT1G29110	-1.057154982	0.589210178	-0.194337576	0.961296121	-3.199487102	1.16727E-05	-1.073839698	0.854730188	-0.292078879	0.951101524	-0.657156953	0.495041116
AT3G04140	AT3G04140	-0.107717161	0.926014869	-0.395375351	0.62705001	-3.207693358	0.001670413	-0.344199189	0.962609292	0.071991881	0.959578958	0.544977306	0.037118647
AT1G58300	HO4	-2.742950394	0.511800983	-3.001240233	0.314997009	-3.208426581	0.032675245	1.63336349	0.916964723	-6.657515785	0.140130981	2.665953073	0.162271863
AT1G75090	AT1G75090	-2.833357039	0.114156003	-0.968340034	NA	-3.211801336	0.000191829	0.094239508	0.980939287	-0.497428244	0.824725558	0.618321391	0.168987962
AT4G17760	AT4G17760	-2.472818175	0.190471655	-0.438192187	0.790946732	-3.261832745	0.041012363	-1.016603589	0.837694719	0.283170449	0.896187586	-0.086738154	0.849985022
AT2G22410	SLO1	-0.519324578	0.70870413	0.62105277	0.65714363	-3.267227145	0.003506417	-0.585354372	0.676109289	0.487093319	0.732143992	-0.389963183	0.210366067
AT1G68725	AGP19	-2.754819035	0.391322179	-3.138995476	NA	-3.281254054	0.049783293	-2.365711349	0.713002473	-0.330682228	0.911878353	-0.228350932	0.731765969
AT2G32630	AT2G32630	-0.019808885	0.991889547	-3.496997337	0.11470462	-3.364576726	0.010776702	-0.077029225	0.992593412	0.687373101	0.570555085	0.210859501	0.687578768
AT3G28960	AT3G28960	0.319082627	0.851723953	0.600697817	0.472709586	-3.396741553	0.023363116	2.970076475	NA	1.665515703	0.698586658	0.074523794	0.966568007
AT3G60540	AT3G60540	-1.487330407	0.585655556	0.478325339	0.850225011	-3.606901326	0.002053847	-7.434161821	0.06223917	-1.272928751	0.575644885	0.354574029	0.764897551
AT5G48660	AT5G48660	-0.661587209	0.529522584	-1.156711284	0.050782788	-3.644151198	0.017061059	-0.541193865	0.753910599	0.079076143	0.924938189	-0.026996143	0.943221794
AT3G12145	FLR1	-0.062608409	NA	-0.975837843	0.453894965	-3.655603492	0.003038955	0.440270179	0.856812526	0.112914228	0.893245359	0.503719543	0.031558169
AT5G22500	FAR1	-2.250039167	0.071234853	-0.851317539	0.398373967	-3.706137212	2.10713E-39	-0.49865601	0.742807747	0.369905701	0.031857176	0.605256045	0.00033849
AT1G74300	AT1G74300	-1.204262759	0.344821053	-0.482359122	0.665590927	-3.752984909	0.005463405	0.428008457	0.95447214	-0.817777804	0.710881196	-1.08733531	0.058819405
AT5G02430	AT5G02430	-0.27254875	0.882140511	-0.514309321	0.81559572	-3.758701375	0.017510916	0.033317497	0.994602444	0.005133196	0.997905896	0.914951691	0.006977916
AT3G44450	AT3G44450	-3.354405455	0.273021068	-2.549616028	0.320299853	-3.814493382	0.000186764	0.287167024	0.981591431	-0.273132564	0.963419708	0.717707065	0.361429224
AT3G59570	AT3G59570	0.22309814	0.909489786	-1.274568483	0.269876374	-3.816598766	6.50212E-05	-0.799026812	0.874288094	-0.430043596	0.628360098	0.434907775	0.140312107
AT1G14185	AT1G14185	-3.629855967	0.07402549	-2.560734741	0.288584465	-3.817916308	0.019354795	-0.245656209	0.874187681	0.005756108	0.998625192	-0.864420233	0.180397316
AT5G05330	AT5G05330	-2.516261702	0.058417214	-2.33487299	0.171809378	-3.919171885	5.77786E-12	-0.519606255	0.940852163	0.14221852	0.922334223	0.312791228	0.451207975
AT2G28250	NCRK	0.227622195	0.883708043	-0.609624344	0.520033684	-3.936040068	0.000485161	-0.617432402	0.913085739	-0.014836932	0.992180597	-0.587640013	0.017733579
AT1G20530	AT1G20530	0.751859302	0.790258663	0.141876985	0.92912178	-3.966443591	0.046777874	-0.582629002	0.913085739	-0.376147948	0.828813007	0.369575643	0.590267033
AT1G22110	AT1G22110	-0.672095358	0.489171332	-1.193917276	0.460606003	-4.04392125	0.043945034	-1.063736561	0.702246294	0.521388952	0.824058587	-0.904657587	0.332641753
AT1G21140	AT1G21140	-1.404737851	0.419613061	-4.194637053	0.081147887	-4.047850227	0.027976662	-0.83207128	0.867112468	0.396173657	0.756419204	-0.975801049	0.00182865
AT5G10260	RABH1e	-0.179164317	0.957893923	-1.293341602	0.71075276	-4.086844992	0.003157942	-0.262104326	0.972043985	-0.16163318	0.933802119	0.71167807	0.276076233

AT4G20160	AT4G20160	3.941445652	NA			-4.119347941	0.011659586	-1.399300957	0.934711472	3.721338465	NA	-3.513832574	0.091796715
AT1G53070	AT1G53070	-0.22227193	0.915356278	-1.260143411	0.343231026	-4.131509406	0.003357875	-0.761231022	0.704938131	-0.25410397	0.683530028	0.245139208	0.31400585
AT3G56100	MRLK	-1.337098407	0.487551743	-3.032753788	0.162399171	-4.208971312	0.00016028	-1.025905282	0.563804992	0.252879418	0.747478504	0.084373979	0.822063446
AT1G11450	UMAMIT27	0.84287898	0.775081848	0.146953196	0.920264171	-4.245245975	0.000268454	-0.551521286	0.770575751	-0.215024588	0.882725602	-0.673271016	0.030299242
AT4G11780	TRM10	-2.46681303	0.058500019	0.479299692	0.905033318	-4.305766606	0.006348531	0.543516054	0.971670869	-0.004797962	0.999460348	-0.050182744	0.959378462
AT5G54140	ILL3	-1.074116426	0.174744512	-0.061254622	0.978692427	-4.392414002	0.0027162	-0.776640258	0.890800373	-0.913764803	0.29888981	-0.553575972	0.123881409
AT2G26750	AT2G26750	20.85647803	NA	-0.532733774	0.935492382	-4.499592972	0.001691166	-4.367970888	0.701035069	1.593663064	0.739473195	2.035573418	0.421148776
AT5G60260	AT5G60260	1.970413938	0.706714014	-5.280649785	0.323736014	-4.717991625	0.016507148	1.397236485	0.885844797	1.37902015	0.90589961	1.445158677	0.069254957
AT1G55550	AT1G55550	-2.261863148	0.221444457	-2.467114878	0.117624268	-4.782096967	0.000343322	-0.106967998	0.982749202	1.526568297	0.246182324	0.661381761	0.464591171
AT1G07050	AT1G07050	-0.609398303	NA	-3.971461888	0.053866143	-4.793593221	0.000218518	-0.083276107	0.985928385	0.069772557	0.982621649	0.551984347	0.470663197
AT1G49230	AT1G49230	-0.078279754	0.990063098	-2.243972903	0.566649445	-4.863924765	2.50453E-05	-0.705171942	0.646171403	-0.304572935	0.686407369	-0.829758685	0.002028047
AT3G07960	PIP5K6	-1.698860314	0.10977024	-1.571932597	0.420523666	-5.370330771	0.000196078	-0.62218602	0.871661081	0.403287479	0.725408943	0.239725656	0.636183138
AT1G04540	AT1G04540	-0.850563257	0.503635584	-0.989291697	0.627840262	-5.534384827	4.79599E-05	-0.671737524	0.706856764	0.218903837	0.833881758	-0.649674148	0.361988869
AT4G12900	AT4G12900	-2.610614183	0.102932459	-2.270851732	0.187875374	-5.595134783	7.73579E-12	1.076446523	0.841532762	1.180887699	0.508853027	1.279329329	0.125104077
AT3G09020	AT3G09020	0.525249834	0.683640416	-0.894110593	0.60708268	-5.608017446	0.000294161	-0.558642662	0.926698256	-0.847581644	0.396807703	-0.865520146	0.226759412
AT3G03840	SAUR27	-1.449217874	0.459537505	-1.79253296	0.509171477	-5.614701289	4.53741E-06	0.006191023	0.999462317	-0.08096106	0.958701896	-0.36564477	0.410396952
AT3G03702	AT3G03702	3.13179887	NA	-4.268795478	NA	-5.640168267	0.022333704	-0.721041513	NA	2.38570681	0.636145007	1.888638383	0.084104334
AT4G26580	AT4G26580	-3.941893556	0.110558571	-0.805501844	0.726084464	-5.686317664	0.000315649	-0.959665762	0.829537284	-1.256375643	0.530853324	-0.415838083	0.64415185
AT2G42430	LBD16	0.84589579	0.709161123	0.445352555	0.868256264	-5.691629592	0.002587922	-1.765256996	0.341617797	-0.649113082	0.592718867	-0.710659003	0.479878036
AT4G28700	AMT1;4	-2.533790937	0.690971429	-0.894648943	0.891942924	-5.815301412	0.019975796	2.260334486	0.869314843			0.500877625	0.762697504
AT5G19100	AT5G19100	-1.25521879	0.809933488	-6.107002434	0.147055076	-5.895988957	0.042514274	-2.612779309	0.652047062	0.305175358	0.873118803	-0.282415335	0.453870377
AT3G05790	LON4	-2.08461514	0.67648401	-6.263382006	0.201838084	-5.961345581	0.008668855	1.845162621	0.803996013	-0.180686595	0.981701925	0.050573215	0.962584999
AT5G42655	AT5G42655	2.748091214	0.387418992	2.072265308	0.603662005	-6.114696532	0.042773351	-1.655720256	0.562387325	-1.205870772	0.177875101	-0.43715646	0.546841096
AT5G38250	AT5G38250			5.191608267	NA	-6.130327794	0.042179423	-3.873481431	0.731041294	6.593661601	0.171120809	-1.542136002	0.060039489
AT2G12405	AT2G12405					-6.145646045	0.042337012					0.675931674	0.513302522
AT3G59340	AT3G59340	-3.066488069	0.155939476	-4.201490177	0.215701909	-6.156347149	0.044847204	0.103271561	0.990231864	1.073370221	0.823781609	-1.852739635	0.155217411
AT2G39330	JAL23	-0.888177503	0.876836845	6.48066178	0.216235729	-6.176713106	0.038711491	0.535099973	0.963590756	3.048631178	NA	-0.481280525	0.803669614
AT3G20950	CYP705A32					-6.210030992	0.035266469					-2.662384887	0.342644259
AT1G72620	AT1G72620	-7.339931074	0.056419838	-6.590269348	0.145026684	-6.216408256	0.033024286	-6.934408847	0.168769726	5.705397375	0.485857955	0.250291339	0.965595352
AT5G36260	AT5G36260	-5.774293582	0.30770498	-2.137081511	0.650838553	-6.21921154	0.041941308	-0.691663561	0.911720308	-0.954719217	0.781706477	-0.840369368	0.16804094
AT5G45469	AT5G45469					-6.234933407	0.043728675					-1.828427288	0.262860644
AT4G26950	AT4G26950	-1.46305189	NA	3.08047001	0.583955901	-6.266306495	0.02978321	4.683535413	0.68299661	-0.883252633	0.82334304	0.404117582	0.897885446
AT4G30830	AT4G30830	-1.572738423	0.667328356	-0.777027133	0.828665561	-6.267030915	0.043874408	-0.513093101	0.944550204	-3.267834791	0.092917654	-1.970227711	0.12395728
AT3G18217	ath-MIR157c	-1.021973593	0.69939111	-6.480052837	0.060728431	-6.268861545	0.032285297	1.190926181	0.906972928	0.315489012	0.936333092	1.747784912	0.138406523
AT4G10950	AT4G10950	-1.51729562	0.794952744	-4.679433448	NA	-6.283816771	0.036863451	-6.52859983	0.585524552	-6.540887678	0.115937316	1.742650452	0.054891134
AT4G01520	NAC067					-6.291885354	0.032218323					-0.648504954	0.711142621
AT2G05330	AT2G05330	1.597493699	0.694887344	1.765686561	0.750966948	-6.292210845	0.028669313	-4.066128103	0.717709314	-0.516475206	0.929498792	-0.695078837	0.761927143
AT3G44805	AT3G44805			-4.66613555	NA	-6.300352548	0.032536423	-4.307082793	0.704225272			0.884903004	0.734979737
AT2G29263	AT2G29263					-6.302994729	0.028625414					-0.371984763	0.890006946
AT3G04900	AT3G04900					-6.309950835	0.007482893					-0.488783403	0.819960054
AT1G65481	AT1G65481	-1.977645312	0.669957897	3.930882791	NA	-6.327783361	0.032020621	-5.239376593	0.653363031	0.230719565	0.953591272	1.948464616	0.112207582
AT5G10572	SNOR77					-6.350688666	0.023273192			2.464811676	NA	-0.345404275	0.69421062
AT1G20015	AT1G20015	-5.340521569	0.348437987	3.592988133	0.304991732	-6.352824541	0.027206072	-0.984616008	0.951652663	1.25582005	0.817744169	0.219896628	0.92542086
AT5G60335	AT5G60335	-2.200942151	0.284712171	1.68357617	0.15377719	-6.358197961	0.029124593	0.297662964	0.967195835	-0.06553925	0.979308722	-0.731768233	0.484586163
AT3G54490	RPB5E	5.223428761	0.360167394			-6.390905415	0.028593381			4.178223344	NA	0.04203352	0.991296529
AT1G31720	AT1G31720	-1.125896659	0.684355548	-6.219597813	0.11069513	-6.404265143	0.022532211	-1.371992888	0.816446062	-0.510068062	0.934226173	-0.64781354	0.621173519
AT2G21770	CESA9	-1.984030402	0.138762772	-4.216135931	0.106954114	-6.412412531	0.021460859	-3.112306141	0.632376028	0.629305109	0.765246551	-0.540934001	0.575535736
AT5G55950	AT5G55950	-1.199427473	0.741787047	-1.306672844	0.71655539	-6.417693568	0.00185787	-0.15596023	0.982749202	-0.916862323	0.494306293	-0.230385792	0.689858975
AT1G61610	AT1G61610	-3.187311841	0.605853217	-2.139964672	NA	-6.432549501	0.018483856	0.129862789	0.994744545	-0.40757333	0.966304751	0.724350534	0.450165662
AT5G65600	AT5G65600	1.027684487	0.835661403	-1.437218407	0.793793522	-6.435392862	0.019442227	-0.113001631	0.990915497	-0.832762675	0.711979287	-0.146994341	0.900277696
AT1G04610	YUC3	-5.98363823	0.12190568	-6.445466698	0.172737369	-6.442042531	0.021519446	-0.106474479	0.990938961	0.036142895	0.992281921	-1.127546464	0.297102809
AT5G26190	AT5G26190	2.610085141	0.478790061	1.425799888	0.689034332	-6.445260788	0.018570984	-5.58731954	0.637815859	0.502923908	0.896984737	0.263522214	0.900311023
AT1G44130	AT1G44130	-5.340521569	0.348437987	5.975920769	0.258892844	-6.445622392	0.01798964	-5.99796884	0.619569853	-1.272573525	0.802042097	-0.398069772	0.836422976
AT1G23210	GH9B6	4.835996915	NA	5.191608267	NA	-6.453060467	0.019509826	-6.93967006	0.173181155	-1.134534888	0.851235177	0.861088849	0.669590322
AT2G43870	AT2G43870	-1.359598592	NA	2.630736082	NA	-6.455123057	0.017755261	-1.002525627	0.71171834	-0.899687615	0.806192405	-0.475340802	0.678193001
AT1G76955	AT1G76955	-6.169462283	0.259473009	-5.382881777	0.31388034	-6.458364611	0.018138711	-3.703226371	NA			-1.249494078	0.768357562
AT1G72220	AT1G72220	-1.928980108	0.665350824	-6.248607426	0.202178883	-6.478548769	0.001367866	-0.554715588	0.959104829	1.18710442	0.63881421	-0.763747899	0.32279546
AT4G27360	AT4G27360	-0.865711772	0.907547346	-4.839925905	NA	-6.483560545	0.020523821					-1.377867238	NA
AT3G46300	AT3G46300					-6.502958825	0.019967278					-0.767411591	0.453488959
AT4G05170	AT4G05170	-0.269202517	0.967594892	-2.711921395	0.59007636	-6.515179698	0.01650998	-1.17473469	0.804934387	-0.905861508	0.353512907	0.213656468	0.853588587
AT3G03910	GDH3	-3.495871817	0.482083074	-6.438259573	0.068407852	-6.557150939	0.021186776	-7.094846154	0.456578783	-0.11538022	0.989242512	0.154043684	0.871619987
AT1G69720	HO3			0.955619064	0.820250926	-6.563014284	0.012921985	-2.283491611	NA			0.545686113	NA
AT4G32785	None					-6.565481445	0.013095738					-0.804681873	0.081114584
AT3G19310	AT3G19310	-4.389271545	NA	-2.175702898	NA	-6.613793161	0.011371128	5.573977872	0.637815859	0.375088932	0.956955597	0.153141681	0.93288314
AT5G43660	AT5G43660	1.158142291	0.691379374	-1.885287028	0.563116089	-6.615725253	0.012266401	-2.24050741	0.735298872	-1.85389789	0.473144084	-1.9219302	0.137402661
AT2G45760	BAP2	-1.189824856	0.823834827			-6.627522025	0.04505295	-2.260890304	0.712887303			-0.020024411	0.992804029
AT2G37880	AT2G37880	0.586154198	0.881800662	-0.877697443	0.780727098	-6.63190962	0.045850972	-1.508435113	0.909352484	-1.556392839	0.705122473	0.979528713	0.319012592
AT5G17220	GSTF12	-4.77434033	0.090164179	-4.958945875	0.357794625	-6.646749741	0.010293766			-4.166738856	NA	-1.893614786	0.637136564
AT5G38000	AT5G38000	2.555569628	0.414899862	-4.670059497	NA	-6.656712945	0.012977873	-1.665306205	0.691824202	1.453515188	0.315034185	-0.932622796	0.14716965
AT1G70990	AT1G70990	3.087127495	0.283837555	-2.276626826	0.403956397	-6.669016067	0.009676212	-1.134811201	0.619569853	-1.19751542	0.112390029	-0.301729181	0.550308798
AT5G26790	AT5G26790	0.296025989	0.963763107	-1.744645682	0.758604757	-6.689039725	0.0093445	-0.252505615	0.978345558	-0.028358806	0.992618878	0.400969973	0.627986017
AT4G00890	AT4G00890			-3.760205642	NA	-6.705711057	0.007605937	-4.191630297	0.709586436			0.043205986	0.983516543
AT3G59010	PME61	-1.392382357	0.620186486	-0.578684716	0.840815416	-6.735465478	0.008239706	-2.421821165	0.607936383	-1.256038427	0.579526976	-1.604695018	0.070118135
AT2G03230	AT2G03230					-6.741595969	0.000408153					0.759543695	0.669113881
AT1G77765	AT1G77765	-0.879553861	0.905746628	-6.303239407	0.191261692	-6.774489619	0.033269552	-5.857034283	0.502514446	-3.06916931	0.417835615	-1.261397753	0.2545807
AT5G37478	AT5G37478	-0.461096915	0.887597229	2.321609107	0.699060901	-6.791076416	0.006814469	-0.447669715	0.953275639	0.6650471	0.708828204	-1.06047591	0.056747931
AT1G69820	GGT3					-6.794598594	0.031854624					0.843927675	0.418061838
AT3G24890	VAMP728	0.279639998	0.838219232	0.251170812	0.840815416	-6.798060347	0.006336886	-0.911643728	0.622600949	0.177748058	0.89084275	-0.491599265	0.669113881
AT1G20590	AT1G20590	5.434034078	0.339653767	-1.590061409	NA	-6.850995018	0.005096191					1.427887398	0.237634793
AT1G09350	GolS3	-0.653980263	0.8290244	1.533681048	0.492042604	-6.861825616	0.032352439	-5.618416013	0.637614678	-1.919090367	0.837072936	-1.950516911	0.229088213
AT5G03060	AT5G03060	2.118803216	0.618671796	1.429676295	0.617758126	-6.868380712	0.00538451	-0.979056953	0.918175819	-0.025941347	0.99575645	0.564390264	0.676630123
AT4G22370	AT4G22370	0.505894206	0.947709117			-6.872177805	0.00584569	-6.560553154	0.584586994	-1.164729483	0.791798458	-0.643518557	0.722330216
AT5G42800	DFR	-5.973627212	0.289521154	-4.13846489	NA	-6.887227156	0.004817419	-0.721041513	NA			1.320497304	0.848399623
AT5G25970	AT5G25970	-1.573262575	0.58404835	-2.670830907	0.258007358	-6.896859495	0.00446397	0.239747638	0.973420015	1.750060783	0.093570977	0.77330838	0.057629685
AT5G58180	ATYKT62	1.39139471	0.578713692	1.115992433	0.768201654	-6.931224018	0.005951603	-4.446520998	0.696374342	4.737406474	0.56843464	-0.416086559	0.958043778
AT5G03010	AT5G03010	-2.996733713	0.534553565	-2.356631243	0.44155731	-6.963309688	0.008449545	0.183626645	0.990231864	0.088178779	0.977308664	0.632968731	0.705374719
AT2G31230	ERF15	-1.577518926	0.38484057	0.425045003	0.866704526	-6.988436638	0.0231245	0.643377573	0.918503553	-0.420924858	0.862605995	0.379913192	0.753635308
AT5G39870	AT5G39870			-6.225450603	0.097419024	-7.00634228	0.002431929			0.036059702	0.998738887	-0.378089329	0.890006946
AT1G16360	AT1G16360	-5.648827596	0.319382726	0.396792487	0.916760049	-7.018108864	0.003917691	2.177638233	0.632278424	-0.288132007	0.954985687	1.540636115	0.073127497
AT4G08910	AT4G08910	0.314474487	0.945249215	-2.519634712	0.341488457	-7.030746709	0.003079925	0.958703692	0.929506087	-1.441045879	0.450245134	-0.676477475	0.425028958
AT4G01380	AT4G01380					-7.077920647	0.003749821					-0.089611549	0.966847061
AT1G08165	AT1G08165	2.957535937	0.339653767	-2.765506845	0.364102237	-7.084758508	0.00218077	1.628298891	0.763339794	-0.690542899	0.790248164	-2.723208252	0.139119421
AT4G14120	AT4G14120	-2.78007738	0.252368547	-3.172270428	0.187929194	-7.089193304	1.29619E-06	-0.213361119	0.978345558	-0.727126285	0.532451246	0.506013158	0.245130517
AT3G52550	AT3G52550	-0.291046736	0.953007157	-0.635652319	0.885283845	-7.10299388	0.010521875	1.378258307	0.872669065	-0.365641304	0.926355164	0.195199865	0.859324499

AT1G02360	AT1G02360	-1.622660965	0.210430609	-1.52520366	0.394305585	-7.10811866	0.001650019	-2.351309922	0.560885037	-1.320063104	0.211913228	-1.166315009	0.054946977
AT3G11870	AT3G11870	-0.112387561	0.989006833	-0.83996362	0.897532415	-7.114376033	0.001536922			5.198487002	0.529391821	0.490864238	0.906321824
AT3G03830	SAUR28	-1.625773811	0.277588188	-0.343868664	0.928901849	-7.126400723	0.002435294	0.254142753	0.974124568	-0.538764124	0.730592522	-1.084561633	0.283109869
AT2G35200	AT2G35200	0.040041908	0.993246068	-2.139964672	NA	-7.13337482	0.001756806	-0.435618401	0.953657757	0.168021477	0.967215096	-1.081147295	0.200684344
AT4G34810	AT4G34810	-1.466030261	0.700555836	0.608922983	0.82972225	-7.142213354	0.016272632	0.320028762	0.966082349	0.291655259	0.908842963	0.952419717	0.130548408
AT4G10560	MEE53	1.051317258	0.861153624	-0.502070861	0.918282679	-7.148199915	0.001565863	0.385199527	0.963509602	-1.048710658	0.372809722	0.733469013	0.384458536
AT3G55940	AT3G55940	-1.568799343	0.5689029	-1.309677752	0.791016329	-7.166217909	0.002495686	-1.775285313	0.679634667	-1.319889167	0.39996561	-0.008988304	0.992296702
AT1G49150	AT1G49150					-7.172542024	0.002178447					-1.614977841	0.42320037
AT1G07270	AT1G07270	-2.56436038	0.307682975	0.092822869	0.973459585	-7.237338584	0.00087768	-0.253150096	0.922389968	0.54603244	0.790326516	0.238490423	0.739945387
AT1G47265	AT1G47265	2.196609457	0.730820949	-2.591101112	NA	-7.255082442	0.000821904	-5.735724335	0.632278424	-4.794158971	0.563104532	-0.230819309	0.938069775
AT1G03980	PCS2	-1.039719397	0.790551307	-5.279161755	0.323830412	-7.263040413	0.001909003	-1.085525412	0.901575434	-0.69789484	0.753296492	0.2306999	0.837194067
AT5G16020	GEX3	-2.094489481	0.532983508	0.047084346	0.989490222	-7.272140605	0.000795337	2.168023193	0.873236091	-5.53955996	0.500990625	0.943272133	0.830082155
AT5G14750	MYB66	-0.216331887	0.947709117	-0.084769972	0.983029981	-7.273501421	0.0007419	-2.833334737	0.632278424	1.586385032	0.174037213	-0.171922797	0.840220331
AT4G15075	AT4G15075			-4.48555851	NA	-7.280791853	0.000816134			-5.634048579	0.423129898	-2.306358693	0.433616558
AT1G71691	AT1G71691	-0.527272832	0.915764206	-6.344482041	0.185328582	-7.284757874	0.001217937	-0.798406831	0.895197572	-1.431365745	0.854132788	0.910442699	0.506025826
AT1G29420	AT1G29420			3.028510462	NA	-7.334629919	0.000943707	1.202511839	NA			0.17371442	0.937560253
AT4G33810	AT4G33810	0.254934312	0.954859922	0.342183386	0.922004802	-7.336826314	0.000611864	-1.563994246	0.753936613	-1.400391147	0.103669281	-0.724005109	0.216647874
AT5G05840	AT5G05840	-0.807606933	0.913866668	-5.577050553	0.22535009	-7.336977031	0.001179034	-4.240317945	0.706790319	-5.191752364	0.529822481	-3.086619319	0.240412698
AT2G36355	AT2G36355	-7.105117936	0.085207889	-1.955202896	0.538919306	-7.351180697	0.000817953	0.364072042	0.965260868	-0.751988741	0.723913117	0.042830525	0.983273091
AT5G38270	AT5G38270					-7.375570458	0.008569377					-2.012685178	0.761927143
AT3G16360	AHP4	-0.992325187	0.89167441	-3.834138105	0.111303652	-7.388531307	0.000663117	-6.96326023	0.309076809	2.999765716	0.37113978	0.209303012	0.855927192
AT2G07599	AT2G07599	1.163998316	0.765943956	3.133504027	NA	-7.390148977	0.000751711	-0.061642892	0.996133946	-0.817007429	0.872598683	-0.460642317	0.847024966
AT5G62850	AtVEX1	-3.297489201	0.091540215	-1.790227904	0.542737828	-7.400314178	0.000565927	1.203150787	0.895234351	1.242086882	0.646137215	-1.233847812	0.703907702
AT5G58750	AT5G58750	-3.262285163	0.45124416	-4.896823138	NA	-7.424433662	0.000463451	-6.72970644	0.5523658	0.289661293	0.972994743	0.146097781	0.961366881
AT3G49930	AT3G49930	-0.603844785	0.834930756	-1.419982741	0.700686889	-7.453543631	0.000567286	-2.770219909	0.676109289	-1.610654216	0.497107266	-0.906371302	0.28959479
AT4G21380	RK3	-0.847723041	0.789968388	0.213284926	0.934788114	-7.499664361	0.000407037	-1.158391097	0.829913345	0.988756395	0.588544512	0.127951482	0.921077297
AT1G66810	AT1G66810	-2.300218312	0.214716661	-0.577954788	0.797523425	-7.507277903	0.000268936	-1.953240464	0.654905244	-1.257499837	0.204941626	0.343813658	0.714599864
AT1G73860	AT1G73860	-2.467356564	0.69939111	0.507937889	0.910195579	-7.557899872	0.000171895	-0.407294355	0.963252175	-0.949193462	0.87530671	-1.209075489	0.308604961
AT3G51220	AT3G51220	-1.786109253	0.569937445	0.361744069	0.82610291	-7.558066886	0.00017422	0.549210486	0.958600664	-0.219215844	0.947801595	0.995052103	0.038747411
AT5G52220	AT5G52220	-1.721331815	0.751827677	0.018262383	0.997613754	-7.567486044	0.000887042	-1.541200371	0.740531841	0.840546728	0.800469173	0.599270965	0.311261683
AT5G41460	AT5G41460	-0.103183882	0.950187878	0.38603863	0.810862864	-7.568802686	0.000349584	-1.522458163	0.696374342	-0.533552445	0.431788455	-0.021219673	0.97124045
AT3G09080	AT3G09080	-2.126515879	0.408617078	0.661366328	NA	-7.609678724	0.000180606	-0.684738523	0.912757899	0.538783053	0.808204461	0.664735537	0.257950784
AT5G28910	AT5G28910	-0.458494766	0.896020233	1.581676712	0.245851001	-7.620189035	8.00153E-06	-0.27400368	0.978041029	0.102099869	0.972994743	0.649280294	0.358843362
AT4G16807	AT4G16807	-1.212647347	0.607296095	-1.346062169	0.494350029	-7.622385838	0.000178688	-1.758495062	0.68625412	-0.042251682	0.982558752	-0.11811245	0.852123075
AT4G23340	AT4G23340	1.792102427	0.389651138	-4.711362079	0.072640879	-7.67050362	9.47169E-05	-1.456216126	0.726970774	0.126929013	0.939499352	-0.738251025	0.034486472
AT1G75530	AT1G75530					-7.684138724	8.8286E-05	-7.143365387	0.133245343	1.377421382	0.625228558	0.003678449	0.998996912
AT4G19590	AT4G19590	0.053394654	0.989381713	-6.24325012	0.203008128	-7.768988331	0.00014147	-0.923362876	0.925191863	1.245397445	0.412503533	-0.291400153	0.622233422
AT2G17470	ALMT6	1.857062616	0.458672966	-3.28003651	0.270104097	-7.773833194	0.000107134	1.447758658	0.913152929	-1.440912406	0.794004103	1.519436098	0.335614472
AT1G63320	AT1G63320	-3.192581456	NA			-7.774751993	6.08169E-05	-4.885058924	0.672844426	-4.806837927	0.56302106	0.21604094	0.83571117
AT2G33847	AT2G33847	-0.90309609	0.865690537	-1.105815021	0.799898763	-7.788118555	7.03697E-05	-1.998593529	0.755850436	-0.291337316	0.911878353	-0.311213815	0.688251776
AT5G61290	AT5G61290	-0.870834793	0.754468472	-3.051119876	0.19278484	-7.799126553	6.41722E-05	-2.927814487	0.659288623	-1.871452508	0.579669854	-0.165584525	0.912130488
AT1G19320	AT1G19320	-3.674011983	0.319751835	-2.125281218	0.406652507	-7.901211181	3.35458E-05	-6.460673721	0.598901706	-6.819599008	0.182940319	0.98527651	0.745042563
AT2G22810	ACS4	-2.721367097	0.17877403	-2.173619194	0.368258033	-7.945271061	2.3622E-05	1.121798765	0.909213796	-1.706017385	0.810616576	-1.696450087	0.517093481
AT5G51270	AT5G51270	-2.964717261	NA	-6.064154237	0.238983251	-7.953844938	3.39087E-05	-0.379198063	0.981578919	-3.958582212	0.154975902	-0.549091501	0.703660258
AT1G60450	GolS7	-1.564573586	0.484924351	-0.22366369	0.911339252	-8.062216654	8.04928E-06	-0.371352045	0.86582927	0.867380648	0.385100337	0.56007298	0.412432685
AT3G24230	AT3G24230	-6.733295706	NA	-6.994926156	0.076893392	-8.086405917	1.06694E-05	-0.140686131	NA	-0.154007876	0.968000197	-0.57776226	0.814632451
AT1G76610	AT1G76610	-1.709683065	0.053882022	-1.961144778	0.255237272	-8.248571865	1.58819E-05	-0.988920235	0.842161979	-1.419100949	0.586988099	0.312365469	0.835980131
AT3G61760	DL1B	-2.474963318	0.168794545	-2.363496889	0.20201346	-8.377310877	1.0027E-06	-0.042717375	0.997253752	-1.305636448	0.349208222	0.149724621	0.821652527
AT5G44050	AT5G44050	-1.895608308	0.363973999	-2.119611981	0.254374246	-8.661920423	9.89697E-08	-0.212907207	0.985077447	1.829096242	0.472438863	0.805138083	0.516947718
AT3G12820	MYB10	-0.239032416	0.938456204	-0.837777658	0.609972536	-8.906082527	3.23203E-08	-0.272754056	0.937910278	0.178575763	0.813363006	-0.134189101	0.891251224
AT4G34580	COW1	-1.23317003	0.515566723	-0.862367703	0.668343185	-9.188846904	1.18828E-09	-0.841560798	0.879859511	-0.364288641	0.813744651	0.800348868	0.126199959
AT3G01550	PPT2	-5.570215985	0.076046222	-4.504361041	0.140880569	-11.33317271	1.57055E-17	2.827127261	0.751308246	1.768233428	0.746959258	1.058489003	0.069411888
AT3G08940	LHCB4.2	-0.845075978	0.065419122	-0.4947818	0.006982566	-0.340121	-0.340121	4.185965017	0.000791358	1.373742405	2.35484E-18	1.507014592	5.69833E-21
AT4G33120	AT4G33120	2.928816438	0.114870133	-1.544389005	0.642371726	-4.699898541	-4.699898541	-2.12256276	0.047862576	-1.316593634	0.003941211	-3.090934105	2.14006E-10
AT2G38390	AT2G38390	1.050717287	NA	-0.937358962	0.685579108	4.263232335	4.263232335	-3.428130517	0.011225671	-2.256617134	0.001646983	-1.196748426	5.63253E-11
AT2G28990	AT2G28990	6.850087602	0.142628328	-6.32675152	0.075819074	-3.042601069	-3.042601069	-6.373373746	0.013621408	-3.002010583	0.048251654	-4.009060308	0.000119526
AT5G67400	RHS19	-1.674662717	NA			-4.718329781	-4.718329781	-8.162635677	0.003032424	-4.753670482	0.005923771	-1.79964154	0.040907681
AT5G23990	FRO5	-4.605234967	NA	21.08102402	NA	4.998274791	4.998274791	-8.463563154	0.002602154	-5.098638077	3.28262E-07	-4.799484364	0.000285778
AT2G19060	AT2G19060	4.812118796	NA					-9.27555431	7.63525E-06	-7.941981749	2.08169E-05	-6.307064088	1.31626E-12
AT4G19690	IRT1			0.905596328	0.80586931			-10.96323022	6.57314E-13	-4.354649776	9.37228E-17	-4.102760639	0.004374582
AT5G10990	AT5G10990	1.055089224	NA	-5.747638369	0.212865002	-2.964401061	-2.964401061	-7.563639988	0.031021407	7.444887828	0.032720325	1.225319113	0.403811713
AT1G14780	AT1G14780	1.567324557	0.183758349	0.513080315	0.572239813	0.95758647	0.95758647	-1.979578535	0.049926206	-1.045320676	0.005266251	-0.832941462	0.000339827
AT1G52120	AT1G52120			-3.913709836	NA			-8.094471256	0.00369309	-7.678761904	0.002422497	1.177961515	NA
AT5G57625	AT5G57625					3.361754846	3.361754846	-8.297304566	0.001368878	-4.392646361	0.000792658	-6.29735824	0.166570147
AT5G52710	AT5G52710					6.460830553	6.460830553	-8.326264595	0.008232965	-4.321055181	0.002862927	0.586288554	0.40844104
AT5G42600	MRN1							-8.694245111	0.000159296	-8.137458687	0.000334588	-3.567878339	0.564984661
AT1G33930	AT1G33930	-0.241654849	0.957312408	-2.435943519	0.147600944	-1.186351382	-1.186351382	8.627424986	0.000455687	0.909567467	0.704826102	2.432723084	8.2371E-06
AT3G55240	AT3G55240	-0.485353026	0.484419965	-0.113940139	0.892961229	-0.921879283	-0.921879283	2.932873549	0.029441299	2.655943431	0.122550672	1.722237087	1.44047E-07
AT4G27280	AT4G27280	-0.211171844	0.767803324	-0.953535303	1.0924E-06	-0.417413725	-0.417413725	1.993773458	0.01669116	-0.425801443	0.075375707	2.423722898	4.2782E-59
AT2G30170	PBCP	-0.90177276	0.042794213	-0.380550367	0.140567431	-0.581171816	-0.581171816	1.912783166	0.000871515	0.571058764	0.225171611	1.149966902	9.6808E-11
AT4G27652	AT4G27652	-0.061682751	0.946209204	-0.134719649	0.724521593	-0.695703971	-0.695703971	1.873899514	0.029489563	-0.313240142	0.610364465	2.439967058	9.94092E-48
AT5G63690	AT5G63690	1.094996515	0.310709119	1.424829768	0.147584039	0.543762475	0.543762475	1.399277543	0.030333721	0.582048236	0.394117937	1.003519686	0.000570133
AT3G12910	AT3G12910	-0.940702847	0.841828039	-1.104913024	0.842885945	1.896203147	1.896203147	-6.970150245	0.036952766	5.342861444	0.515629348	3.188938539	1.28482E-19
AT1G20310	AT1G20310	-1.369515804	0.572303345	-1.030395605	0.77014218	0.39249351	0.39249351	-7.603446812	0.034723544	0.967778517	0.871060407	2.741445732	0.008814723
AT2G39510	UMAMIT14			0.867770337	0.887858055			-9.097138101	1.22306E-05	-2.086019721	0.321494481	1.794694271	0.007899538
AT4G14370	AT4G14370	-0.239151357	0.879443097	1.527663084	0.232771298	-0.498134387	-0.498134387	-2.142249532	0.028903644	-0.455034842	0.74754675	1.353771018	0.012519241
AT5G07200	GA20OX3	-4.306021916	NA			-0.322853803	-0.322853803	-8.302331255	0.001368878	-0.23399271	NA	-1.13379709	0.008692323
AT5G46230	AT5G46230	0.915356928	0.706692845	-1.00628035	0.77395806	-0.567423079	-0.567423079	-2.296376061	0.02729194	-0.898355456	0.513456138	-1.136531995	0.002019851
AT5G35110	AT5G35110	0.412980601	0.896020233	-0.702932765	0.85594543	-2.964401061	-2.964401061	-8.467999605	0.00061223	-0.467129206	0.763716036	-1.235552156	0.042128952
AT4G12170	AT4G12170					3.370054164	3.370054164	-10.66299706	7.06239E-09	-5.993861666	0.400086588	-1.247890857	6.75579E-07
AT1G01120	KCS1	-0.807104378	0.009246389	0.176012668	0.665671364	-0.753555701	-0.753555701	-1.083905367	0.031021407	-0.946620044	8.99092E-08	-1.316201259	2.71228E-40
AT2G38060	PHT4;2	-1.011248942	NA	-5.964119934	0.259615073	0.607263058	0.607263058	-8.496749722	0.000474211	0.556172887	0.820734505	-1.329882993	0.017119575
AT1G28470	NAC010	0.070467397	0.973788838	-4.019268257	0.09279825	-0.270147355	-0.270147355	-2.606994868	0.007061489	-0.480440584	0.739968763	-1.3600846	0.000360386
AT1G30900	VSR6	-0.187016638	0.919101039	-0.700905762	0.604211142	-1.404521803	-1.404521803	-2.171535807	0.000764112	-1.000107915	0.219838372	-1.525409049	2.00692E-10
AT5G48850	ATSDI1	-1.456656259	0.766230417	1.832560722	0.584049866	-0.679834524	-0.679834524	-8.140638852	0.002778525	-0.210283684	0.944259795	-1.705465808	0.01163678
AT4G22214	AT4G22214	2.885955311	0.555167058	1.617002231	0.796862307	-5.610201732	-5.610201732	-7.441788899	0.03917779	-0.009186326	0.999129744	-1.707011556	0.015604456
AT5G04200	MC9	-0.290986718	0.87080219	0.494758339	0.830493652	-0.691167021	-0.691167021	-2.383934922	0.040951096	-0.472792177	0.305729659	-1.868660257	1.27199E-14
AT3G07970	QRT2	1.358255327	0.807922143	-4.39926821	NA	2.540606196	2.540606196	-8.71456718	0.000196309	-0.806712439	0.694512158	-2.045669934	1.44042E-06
AT3G62730	AT3G62730			-4.829418443	NA	-0.904461209	-0.904461209	-8.099742421	0.016167921	-2.332938559	0.426788377	-2.134122476	0.000745728
AT2G18800	XTH21	5.766775632	0.308593181	-4.761910288	NA	-4.584492934	-4.584492934	-7.778190587	0.021703759	0.59965631	0.873976865	-2.299654854	0.015609331
AT5G56080	NAS2	6.672944263	0.180381567	-0.707954158	0.900545964	-5.978294221	-5.978294221	-2.599137087	0.000390609	-1.129718511	0.391551683	-2.38766049	0.007408667
AT3G03650	EDA5	5.432773189	0.339653767	3.826878788	0.228113842	-4.169754161	-4.169754161	-8.770403956	0.000232034	-1.499945148	0.345005189	-2.482271496	0.00036413
AT4G09770	AT4G09770	4.594674208	NA	-5.697003203	0.283484602	-5.144954819	-5.144954819	-9.043987919	7.63525E-06	0.746192158	0.787345985	-2.709615178	0.009078388
AT4G13480	MYB79	4.456967053	NA			-3.585343201	-3.585343201	-9.064621845	0.000230431	1.180783796	0.923631194	-2.718883935	0.001519102

AT2G26480	UGT76D1			5.343401819	0.318517823	-5.900662457	-5.900662457	-8.98719811	0.000145928	-0.14478833	0.986774932	-2.873535978	8.93152E-05
AT2G16970	MEE15	4.495979824	NA					-8.658926114	0.000159296	-0.995454408	0.793495116	-3.048185006	0.000923314
AT2G31930	AT2G31930	4.720374787	NA			-3.846703073	-3.846703073	-8.383067138	0.005286142	2.488020309	0.727585697	-3.062184877	0.015984316
AT3G62210	EDA32			-3.723608976	NA	-5.107386302	-5.107386302	-7.895569325	0.014996721	-0.960164613	0.72293719	-3.111173892	0.000819251
AT4G10510	AT4G10510	0.165346408	0.979757022	-6.245132812	0.206367457			-8.788808264	0.000224281	-1.757619706	0.447017804	-3.135563819	1.40832E-07
AT2G37820	AT2G37820	1.297298363	NA	-5.360528298	0.316103626			-7.818902057	0.015660745	-1.879284945	0.694512158	-3.294611663	0.005216304
AT4G00070	AT4G00070	5.595700078	0.324624779	1.852008258	0.660676832	-5.600001462	-5.600001462	-8.174042826	0.001780367	-2.358337172	0.45858949	-3.466203178	0.010040285
AT5G59320	LTP3	-0.137149277	0.97446233	1.378039402	0.375772743	2.593992448	2.593992448	-4.125569944	0.000232034	-1.345778551	0.117868539	-3.635117098	1.33013E-07
AT4G37340	CYP81D3	4.196892303	0.09623779	-5.165788684	0.335449991	-5.29371904	-5.29371904	-5.330663252	0.000139611	-0.512129774	0.849967331	-3.978189961	1.24726E-05
AT4G25310	AT4G25310	-1.038296334	0.875391671	-5.784823833	0.275242324	-2.858537913	-2.858537913	-8.713498059	0.000764112	1.020486873	0.618699262	-4.051579051	2.31547E-08
AT2G46495	AT2G46495	1.542416554	0.800886887	2.052110626	0.508155445	1.824034791	1.824034791	-8.404569262	0.001780367	-2.716674863	0.420851067	-4.256044641	0.00606842
AT1G77950	AGL67	2.126870043	NA	6.816403062	0.125174328	-2.763139698	-2.763139698	-8.305391182	0.001058569	5.038041062	0.542006423	-4.431173617	4.17122E-09
AT4G27890	AT4G27890	-5.877178594	0.298216422			-2.479411406	-2.479411406	-9.298147082	1.40457E-05			-4.480455633	1.32783E-10
AT4G12550	AIR1			-5.460046351	0.306433138	0.114245417	0.114245417	-10.118511	1.95231E-07	-3.066231362	0.197345546	-4.50813772	7.91232E-27
AT4G25820	XTH14	6.265933808	0.263905113	2.270118548	0.70519861	-0.8886554	-0.8886554	-8.837292937	0.000114786	-0.44958221	0.939595436	-4.649914584	0.014967844
AT1G07550	AT1G07550	6.586829249	0.195693617	-6.261867841	0.203449364			-8.803090779	0.000505277	-1.9647367	0.456191169	-4.956492519	0.000633498
AT4G02270	RHS13					1.509335617	1.509335617	-7.692887717	0.02729194	-2.779880127	0.239373546	-5.377766085	0.029691673
AT3G32047	AT3G32047	0.434454736	0.954813473			6.679460022	6.679460022	-7.539104659	0.038510455	-0.803686317	0.953754747	-6.160682151	7.15514E-05
AT5G24100	AT5G24100	6.193782826	0.270083636	-3.553772892	NA	-2.002730959	-2.002730959	-7.997256118	0.00495004	-3.428396615	0.190070058	-6.392508024	0.000132781
AT5G26130	AT5G26130							-9.375664103	0.032525887			-7.016527594	6.25028E-05
AT5G13170	SAG29	-6.038493667	0.218108646	0.38490245	0.944922771	-0.075179621	-0.075179621	-9.797061533	3.3977E-06	-2.042957932	0.578542261	-7.174520361	0.000348444
AT3G63360	AT3G63360	-4.55852849	NA					-9.384901487	1.27886E-05	-4.954503713	0.550184567	-8.58103922	6.98099E-08
AT1G21395	AT1G21395					4.211937405	4.211937405	20.99790017	5.14003E-05			-2.974466693	0.645082103
AT4G35733	AT4G35733							20.86139625	6.05443E-05				
AT5G50140	AT5G50140	6.930262136	0.206565379	-4.051255346	NA			20.80771616	6.36558E-05				
AT4G04423	AT4G04423	-0.797805596	0.585082698	-0.055850132	0.988868832			20.26833295	0.00012471	1.399099568	0.866906715		
AT3G54065	None	-3.133209462	0.12836641	-1.659424177	0.506101041			9.895963626	0.03014848	-0.780884323	0.947746874		
AT5G04000	AT5G04000	0.971420824	0.419823709	-1.195168996	0.838823685	1.710148797	1.710148797	8.135031267	0.008334497	-1.203349079	0.72447373	-1.006752717	0.17419756
AT5G46490	AT5G46490	-0.622113451	0.544242741	-0.277629413	0.605664872	3.282600546	3.282600546	4.106341477	0.033328567	-2.260502268	0.383106164		
AT1G77210	STP14	-0.177438891	0.786905346	0.095224838	0.809356728	0.645449965	0.645449965	3.693914104	0.024208681	0.353024717	0.636285501	0.145878013	0.66018892
AT4G35770	SEN1	0.709175141	0.210769687	0.600859963	0.008624031	0.950993482	0.950993482	3.55761132	0.007024087	0.521446246	0.006064026	0.590913176	6.20459E-08
AT3G62550	AT3G62550	0.205147608	0.779886613	0.602766696	0.00338499	0.590819728	0.590819728	3.325713582	0.040438362	0.001857925	0.999129744	0.090248227	0.836253544
AT5G02160	AT5G02160	-0.484743155	0.383443279	-0.675689573	3.62217E-05	0.026308317	0.026308317	3.298242078	0.013621408	0.814691456	5.26178E-05	0.955702809	2.8624E-08
AT5G54770	THI1	-0.261280083	0.623409099	-0.048244649	0.826175404	-0.346346098	-0.346346098	2.926774283	0.02063208	0.081789594	0.811583203	0.112536912	0.406486689
AT1G68010	HPR	-0.64936624	0.168789045	-0.036419675	0.869962814	0.186278342	0.186278342	2.884604992	0.025169391	0.421768727	0.039686752	0.565399747	2.33021E-05
AT4G09900	MES12	-1.30046659	0.075009884	-0.08315173	0.855355791	-0.35104122	-0.35104122	2.744576458	0.014996511	-0.298454206	0.899553662	0.183003259	0.704092503
AT5G19940	AT5G19940	-0.263219424	0.647395988	-0.265949634	0.061714217	-0.307851381	-0.307851381	2.69407946	0.034723544	0.456000659	0.046194304	0.621752953	0.000691264
AT1G79040	PSBR	-0.958936967	0.030078375	-0.382802586	0.021576968	-0.837691838	-0.837691838	2.539114447	0.036952766	0.565534259	0.265754101	0.574490459	0.000673494
AT5G43470	RPP8	0.192273459	0.75510677	0.250574345	0.181630248	-0.008160314	-0.008160314	2.265066773	0.041186577	0.039956803	0.958119929	0.159454249	0.651051861
AT3G50480	HR4	0.147615338	0.849577528	0.269083093	0.585532227			2.24305306	0.015840375	0.604657057	0.066481086		
AT5G16810	AT5G16810	-0.427811714	0.336737651	0.611274665	0.061714217	0.322999346	0.322999346	2.141261892	0.039620238	0.660798581	0.108772614	0.875353015	1.27554E-05
AT5G46470	RPS6	-0.677158507	0.160917019	-0.367959689	0.237464966	-0.309318859	-0.309318859	1.868476865	0.039793784	0.098047234	0.905044588	0.446545782	0.019180135
AT4G30690	AT4G30690	-0.613249741	0.110662504	-0.585100915	0.001323565	-0.406465156	-0.406465156	1.055247974	0.034173018	0.469713374	0.035773796	0.676595466	0.000731495
AT1G06520	GPAT1	-0.026230554	0.977833245	-0.942170753	0.270121378	0.411060559	0.411060559	-1.07018492	0.023094271	-0.572841584	0.279139044	-0.641325336	0.015743437
AT5G11630	NOXY2	0.44919597	0.675232305	0.149203572	0.873960412	0.582601505	0.582601505	-1.128957925	0.014772649	0.29989676	0.653831304	0.296295728	0.3173191
AT1G68690	PERK9	0.133871037	0.877970597	0.715823066	0.155706618	0.132997227	0.132997227	-1.162964914	0.006700442	-0.334619058	0.608621897	-0.231806526	0.469579086
AT2G43120	AT2G43120	0.019111498	0.985842093	-0.823324243	0.203673022	-0.500035287	-0.500035287	-1.235066484	0.007918314	-0.326286023	0.511188955	0.269616327	0.197241721
AT3G25780	AOC3	0.918948764	0.053096755	0.424402602	0.363086924	-0.575712639	-0.575712639	-1.527214158	0.000290724	-0.489456472	0.360033758	0.969697019	2.85937E-07
AT1G02000	GAE2	-0.698969186	0.345026093	-0.452423101	0.357008311	-0.383132417	-0.383132417	-1.582621635	3.06565E-05	-0.575554672	0.258282444	-0.952406432	9.42361E-05
AT1G71110	AT1G71110	0.125507823	0.85636979	0.077040232	0.905508174	-0.47888813	-0.47888813	-1.841282838	0.032135808	-0.576017864	0.05060627	-0.392482207	0.035899047
AT4G39580	AT4G39580	0.079055864	0.954423621	0.552994872	0.530128133	-0.045308525	-0.045308525	-2.117156206	0.023094271	-0.435669068	0.806135068	0.161060687	0.840152285
AT1G09932	AT1G09932	-0.146292829	0.928155676	0.593204042	0.642505518	-1.006828017	-1.006828017	-2.657479056	0.034661846	-0.877308325	0.398994908	-0.044423863	0.937924382
AT3G17690	CNGC19	0.519639147	0.690820084	-0.45362465	0.84960019	-1.472637472	-1.472637472	-4.451170074	0.021051627	0.196558719	0.931302326	0.510324438	0.325243403
AT3G58190	LBD29			5.82828386	0.271605621	-3.967003132	-3.967003132	-5.222830313	0.000665765	-1.200705422	0.852093426	-1.364668811	0.349802908
AT3G16410	NSP4	1.572373879	0.521843942	0.503024541	0.873799929	0.107973602	0.107973602	-5.465207676	0.000612688	8.46932E-05	0.999968179	-0.913740448	1.56085E-08
AT1G10400	AT1G10400	1.307688552	0.618169643	2.394740859	0.364788837	0.611428955	0.611428955	-6.62656424	0.031910209	1.055830233	0.804786811	-0.069658027	0.944052403
AT2G19990	PR-1-LIKE	-0.122067853	0.978709893	-0.70172042	NA	-1.356047511	-1.356047511	-6.959694276	1.86617E-08	0.124832685	0.957130081	-2.856460803	0.059750525
AT4G25430	TRM23					-0.386272291	-0.386272291	-7.415129937	0.044537685	-0.309805635	0.952661388	0.023868716	0.988467984
AT5G39120	AT5G39120			1.043996991	0.85552235	3.797624877	3.797624877	-7.457778618	0.008657591	-4.407483065	NA	-2.124420451	0.243236144
AT1G12480	OZS1	2.192727553	0.50023514	0.722597581	0.884536821	0.066401994	0.066401994	-7.50543544	0.036659599	0.767400501	0.93295262	-0.199792085	0.898456199
AT2G31480	AT2G31480	5.291421123	0.353350645			-2.353593325	-2.353593325	-7.57769196	0.044537685	-2.441999891	NA	0.974876834	0.657224395
AT2G07585	None	-1.029105567	0.858534118	-0.994421834	0.836982411			-7.603450001	0.032389525	0.922874178	0.835297596		
AT1G27080	NRT1.6	1.506299094	0.700218767	3.070333015	0.062103545	1.401276833	1.401276833	-7.640819028	0.028146591	-1.912104255	0.53525964	1.643375642	0.561289739
AT3G20180	AT3G20180	-3.714732484	NA	-4.890819213	NA	-4.32704903	-4.32704903	-7.653263649	0.024641754	1.403794121	0.502953208	-0.73657311	0.455773918
AT2G34390	NIP2;1	5.710311994	0.313837929	3.666001774	0.374299202	1.446436412	1.446436412	-7.67330901	0.024641754	-1.814725246	0.570854222	-0.4601665	0.556521413
AT5G18636	AT5G18636	-0.04744486	0.994592158	1.938378384	0.249007628			-7.721204544	0.019675654	-1.931139161	0.565872781		
AT5G36240	AT5G36240	5.571609166	0.326621865			0.68659333	0.68659333	-7.75006977	0.042314036	0.44515039	0.975386078	0.006753113	0.987605059
AT3G58060	AT3G58060	5.362540152	0.346852975	-4.498825584	NA			-7.913836225	0.007550881	2.552765642	0.711773969	-2.835851714	0.059564635
AT1G61480	AT1G61480			-6.436663625	0.219211613	1.536108564	1.536108564	-7.95070612	0.006700442	-0.806460921	0.885828236	0.363617519	0.719120793
AT4G10020	HSD5	2.439380769	0.648269786	-1.121538496	0.766419997	5.011702086	5.011702086	-7.990250029	0.014996511	0.61835271	0.887737509	-0.200352758	0.93288314
AT1G59670	GSTU15					1.792591462	1.792591462	-8.017979732	0.034723544	4.418165677	NA	-0.445427548	0.460000516
AT2G34350	AT2G34350							-8.09989236	0.004462686	-2.672112564	0.230607776	-2.076487374	0.069932312
AT3G20730	AT3G20730	3.118990386	0.42087957	2.27630077	0.094143091	1.404434801	1.404434801	-8.127414485	0.008978139	-0.737285578	0.733750816	-0.075174366	0.896113947
AT1G22120	AT1G22120	2.577702017	0.460091669	4.816269265	NA	0.40776233	0.40776233	-8.141992567	0.006615804	-3.031200657	NA	-0.854370702	0.06346187
AT5G13320	PBS3	-2.107979687	0.653372238	-0.151898	0.957480058	-0.276193007	-0.276193007	-8.145036036	0.008334497	0.483381487	NA	0.699264574	0.589484191
AT3G53600	AT3G53600	-2.338560071	0.635055498	-4.875203671	NA			-8.214179899	0.003366984	-4.770598795	0.565321135	-3.117619646	0.302856566
AT2G18680	AT2G18680	-0.155866865	0.95823127	1.681029431	0.117214309			-8.219239146	0.001787773	-0.037908351	0.994809352	1.452406385	NA
AT2G14610	PR1	-0.487274824	0.924854279	4.680933429	NA	0.890505792	0.890505792	-8.275721889	0.014772649	1.295832504	0.894274701	-0.99531971	0.666907981
AT1G64405	AT1G64405	3.13179887	NA	-4.271757151	NA	2.32710382	2.32710382	-8.360387866	0.001998161	-0.934871341	0.799682552	-0.397336312	0.646992921
AT5G24860	FPF1	3.941445652	NA			2.083796485	2.083796485	-8.375592556	0.000898917	-0.813395071	0.686941393	-1.285880821	0.242453486
AT1G70170	MMP	0.81289209	0.846562203	1.969352206	0.746899209	-0.256395539	-0.256395539	-8.386290701	0.001277004	-4.925245279	0.237450962	-0.993130166	0.127160513
AT1G28650	AT1G28650	0.04607348	0.99493101					-8.490347745	0.000887648	-0.166510845	0.98996705	-2.773917138	0.104903893
AT1G55420	EDA11			4.053596333	0.457842486	7.088829457	7.088829457	-8.498164781	0.001058569	0.04210464	0.994163341	-3.183068369	0.117750995
AT1G55230	AT1G55230	5.643148484	0.320321289			-4.848369275	-4.848369275	-8.51857716	0.002135465	-5.378970033	0.491449838	-2.748309394	0.356937017
AT3G61390	AT3G61390	0.127934078	0.977053436	-5.016243409	0.351808035	2.165317836	2.165317836	-8.57170986	0.001772481	0.622907585	0.927107216	-2.968189336	0.108186855
AT1G04580	AO4					0.856401111	0.856401111	-8.697516616	0.001331882	0.713594758	0.952000282	-0.395726743	0.55700347
AT4G04490	CRK36	-0.148176746	0.97974805	1.768537756	0.307870752	-4.727561799	-4.727561799	-8.741835451	0.001005689	2.442936918	NA	-2.371415883	0.131496173
AT4G13890	EDA36	2.126870043	NA			-0.333721178	-0.333721178	-8.790267154	0.000196309	-2.736531841	0.613407287	-1.933654665	0.096151841
AT1G69930	GSTU11	-1.204456683	0.744826529	1.187600092	0.658545081	1.806889357	1.806889357	-9.022929497	0.000193989	-2.462099946	0.494228383	0.071889216	0.969453814
AT5G65320	AT5G65320	5.663800809	0.318296183	-1.396910792	0.826424466			-9.140503652	0.000183088	-0.768331017	0.787771995	-0.892187929	0.535187887
AT3G57260	BGL2	-0.19231734	NA	4.521824483	NA	-1.329729765	-1.329729765	-9.203547442	1.86861E-05	0.539775711	0.913903461	-7.049352097	NA
AT1G26390	AT1G26390	3.454782108	NA	4.167940509	NA	5.150456581	5.150456581	-9.322188042	0.000226468	-3.293649404	NA	-1.426136175	0.631407715
AT5G05340	PRX52	-0.766937299	NA	5.923444022	0.263600507	0.10936716	0.10936716	-9.355705635	0.001196867	-0.027363491	0.996204637	-0.604259106	0.849974116
AT4G26200	ACS7	-1.600172273	0.11225395	2.298711966	0.085006626	-3.443471303	-3.443471303	-9.616262143	3.3977E-06	-1.099498965	0.875307769	2.201590498	0.061958385

AT5G39100	AT5G39100	1.665659335	0.634127458	2.624293528	0.653714617	-4.799436413	-4.799436413	-9.668379196	1.1941E-05	6.08529011	0.389152097	-1.45819631	0.425144594
AT4G15242	AT4G15242	5.528536566	0.331020991	0.288290531	0.965676281	-0.366832944	-0.366832944	-9.69727051	8.7857E-06	-2.58194499	0.694330823	-0.076090514	0.697462519
AT5G27010	AT5G27010	1.156616053	NA	-4.498825584	NA	-0.557725388	-0.557725388	-9.948113794	8.40871E-07	-1.128816572	0.89611977	-0.091337353	0.714615614
AT1G09380	UMAMIT25	5.079689919	NA					-10.00344209	4.00221E-06	-6.842016944	0.178726868	-4.84215456	0.284358906
AT4G16215	AT4G16215	1.686386436	NA	-0.825358031	NA	-0.01700515	-0.01700515	-10.21237688	7.63525E-06	-0.30296613	0.977360449	-0.188506633	0.34786528
AT2G41850	PGAZAT							-11.02057863	7.06239E-09	-3.338179188	0.679295852	-2.042572455	0.761927143
AT2G05914	AT2G05914	3.521783493	0.243611404	3.396477871	0.55446246	-0.024834557	-0.024834557	-11.13388676	6.86788E-10	-0.285355674	0.958701896	-0.186926163	0.559807838
AT4G19240	AT4G19240	3.238888458	0.342805505	0.573402435	0.916456049	-0.052764955	-0.052764955	-12.08681569	1.97737E-13	6.27244538	0.333632035	-0.049508591	0.8685544
AT1G29100	AT1G29100	20.82411682	NA	4.722920559	NA	6.379914409	0.14311986	-1.100474737	0.906077763	8.433339738	0.000153607	-5.026265023	4.82542E-05
AT1G28480	GRX480	0.900667897	0.429252034	0.791576434	0.74170093	-0.171722483	0.895879743	-2.491889587	0.637614678	5.937109599	0.000174682	1.216050042	0.031234768
AT3G20395	AT3G20395	-0.182783893	0.802363037	0.36911466	0.610709932	-0.555355681	0.307512202	1.379779888	0.437207517	1.371343175	0.001806437	1.442533781	4.8869E-07
AT3G09870	AT3G09870	-2.591460511	0.184918404	0.560569521	0.82951655	-1.845570298	0.582195412	2.446124642	0.697407195	-4.041920138	0.019994893	4.133186425	1.06108E-30
AT5G42380	CML37	-0.354761943	0.818760835	-1.083505929	0.174172969	0.582242933	0.564487123	-1.684874032	0.451431824	-2.914597471	0.003207786	4.011783001	1.13273E-05
AT1G27730	STZ	-0.839789633	0.119206476	-0.85266259	2.60262E-08	-0.856523251	0.000772469	1.984611035	0.136169212	-1.122391111	0.00113082	2.963158542	2.14631E-93
AT1G61290	SYP124	-1.010889674	0.625492655	1.063545207	0.712359806	1.854306602	0.11568525	-5.08704053	0.625225271	-5.069465264	0.030825132	2.863359473	0.009796044
AT4G23810	WRKY53	-0.412860928	0.564290563	-0.315877451	0.747486559	-0.752174645	0.373613242	-0.664024278	0.705076831	-1.340048335	0.011182566	2.3486953	4.9628E-31
AT2G38470	WRKY33	-0.976045095	0.065290295	-0.476565872	0.008384836	-0.40582348	0.54729892	0.994645132	0.655815891	-1.617137554	4.72945E-14	2.2047173	3.62925E-51
AT5G57560	TCH4	-0.955174022	0.083711494	-0.281384815	0.360461965	-0.461379903	0.095163903	2.329448808	0.084215519	-1.252819135	1.19437E-12	2.130438075	5.02789E-39
AT5G57010	AT5G57010	-0.998552476	0.483102173	-0.040467786	0.986965768	-1.525928728	0.401454735	-3.882127747	0.10756991	-2.793175807	0.000181037	1.796927201	2.15288E-08
AT3G50930	BCS1	-0.561156705	0.409089886	-0.206036272	0.714230544	-0.984876396	0.004164125	-1.037598219	0.090721008	-1.21016905	6.62337E-05	1.732594381	5.00558E-37
AT5G27420	CNI1	0.785261378	0.128283036	0.577420093	0.037763549	0.391595448	0.363854374	0.39248139	0.844676044	-1.200795205	0.002437319	1.662365795	3.02587E-08
AT2G30210	LAC3	0.792640778	0.435905148	-0.364802148	0.773024438	-0.084504502	0.953360985	-1.325248219	0.240856916	-1.018803666	0.000228249	-1.006457777	3.67951E-07
AT1G24430	AT1G24430	6.668480083	0.18345166	5.338352945	0.318842237	0.772908446	NA	-2.204829987	0.597380545	-1.903336908	0.043902091	-1.021845291	0.000750564
AT4G40010	SNRK2.7	-0.301282725	0.87082448	0.351877539	0.812146561	-0.609372269	0.624058361	-0.422148602	0.732322487	-1.536444378	0.021982876	-1.145022868	6.97648E-06
AT2G25260	AT2G25260	3.911334925	0.175493283	0.763115124	0.786146042	-5.455504734	0.147921959	-0.975771634	0.658916837	-1.010525572	0.026694402	-1.256389344	0.000429466
AT4G35160	AT4G35160	-0.500485258	0.700555836	-1.423919579	0.340428189	-0.52485166	0.809021526	-1.420057522	0.377623412	-1.009193096	0.000553253	-1.324934016	5.17708E-08
AT5G40780	LHT1	-0.299366235	0.642462566	-0.266178447	0.762189797	-1.031871358	0.057266969	-0.456760512	0.735512818	-1.435712154	3.21975E-06	-1.449489245	1.03448E-05
AT3G58550	AT3G58550	-0.460489485	0.871216156	0.960072617	0.704678172	0.827567059	0.68164843	-1.87271032	0.469878733	-1.112642398	0.011544904	-1.557755335	5.94403E-18
AT1G51790	AT1G51790	1.40209652	0.493130592	1.082330701	0.598184284	-0.009255068	0.996095544	-1.57184016	0.420106526	-2.057371944	0.000951489	-1.578734287	0.000644893
AT1G02205	CER1	1.3540411	0.339653767	0.966415765	0.229780027	0.29557677	0.743142394	-1.616049678	0.255573307	-1.346024859	2.06493E-19	-1.633608725	9.41391E-26
AT5G25820	AT5G25820	1.215054796	0.695741487	1.31491491	0.548380422	-1.27058481	0.568890166	-1.555832134	0.69015436	-1.400740236	0.006382379	-1.637035755	4.53649E-07
AT4G00360	CYP86A2	-0.281983562	0.621859146	0.424097594	0.057800427	0.002594422	0.989319762	-0.578376168	0.647947756	-1.688959184	4.6867E-19	-1.647972317	1.04674E-38
AT5G53110	AT5G53110	2.886964937	0.063684701	1.493569527	0.520491926	1.293477545	0.414032159	-1.967357543	0.62738272	-1.589231369	0.003528517	-1.662479351	1.578E-05
AT5G63560	FACT	-0.007298345	0.997656929	0.022152038	0.990431447	1.569710811	NA	-1.640108693	0.785453357	-1.303172692	0.002325028	-1.663940288	8.7456E-26
AT1G50560	CYP705A25	1.245126201	0.124995004	0.940368089	0.48831288	0.061272226	0.975285072	-1.702358858	0.696374342	-1.512113463	0.011122394	-1.691986099	1.62376E-06
AT2G41800	AT2G41800	3.959596291	0.119914823	-1.888193845	0.641028272	-0.047696897	0.986142943	-1.362092027	0.732202251	-2.091604945	0.004688335	-1.725236525	0.00132779
AT3G13610	AT3G13610	2.664887458	0.279920293	2.815882712	0.475681635	-0.460422908	0.866988322	-3.402562243	0.253572572	-2.24596764	0.000977815	-1.740752355	0.000778044
AT2G24430	NAC038	3.225367124	0.170963406	0.575741342	0.780134425	-0.091112527	0.984968588	-2.441022676	0.616300683	-1.959725644	0.014195831	-1.775714508	5.92239E-05
AT5G41290	AT5G41290	1.156616053	NA	2.228533832	0.452503776	-5.941523691	0.144744566	-1.969994804	0.638079908	-3.033640061	0.002103486	-1.871069	0.019940209
AT3G45710	AT3G45710	0.1962745	0.885132359	-0.656352082	0.676332404	-1.175633228	0.471643381	-1.433512641	0.559215018	-1.130063347	0.003976804	-1.875710538	9.04834E-18
AT3G25930	AT3G25930	-0.031313748	0.989381713	-1.582712099	0.409852705	-1.137617583	0.563559598	-1.595901213	0.405880771	-1.33166263	0.004094084	-1.879835445	2.19505E-09
AT1G74460	AT1G74460	2.940432491	0.065016803	-3.021970104	0.219150322	-1.127565919	0.466372658	-1.745673153	0.657950393	-1.050265252	0.039595468	-1.899902518	2.11402E-21
AT3G51330	AT3G51330	0.435526392	0.446189306	0.504530155	0.151852821	0.037340752	0.890210018	0.532599005	0.751308246	-1.144070672	0.000259629	-1.965472368	3.3522E-12
AT1G51830	AT1G51830	4.132424985	0.118323583	0.621656582	0.829717102	-2.307320578	0.289279307	-0.871990657	0.709586436	-2.240226478	0.000217946	-2.015535769	5.06749E-06
AT3G11340	UGT76B1	1.895183491	0.069051866	3.698879855	0.370901645	-1.327215579	0.306887645	-1.578988517	0.319520931	-1.426140512	0.000117616	-2.033896007	5.91239E-13
AT3G27170	CLC-B	-1.636465347	0.092192216	0.729579224	0.422760933	-0.904266323	0.041890631	-1.162624084	0.548879103	-1.368256556	0.017834394	-2.062766906	6.79535E-08
AT3G23175	AT3G23175	3.296965583	0.177971178	2.206226818	0.522924428	-2.187436129	0.480244021	-1.646755557	0.670132494	-1.400685005	0.005269173	-2.073897908	4.7871E-05
AT1G18970	GLP4	6.828136132	0.15074356	-4.051255346	NA	-4.191543895	0.441624455	-4.027233043	0.341344612	-2.448875988	0.007677783	-2.080762681	7.98436E-06
AT1G78990	AT1G78990	1.064534848	0.69839259	0.794446901	0.419757542	-0.507256993	0.750405813	-1.45822229	0.712887303	-1.880996467	0.014385114	-2.109481184	1.33905E-13
AT3G55150	EXO70H1	3.11289252	0.540732769	-1.467302458	0.703756747	1.700285468	0.260689354	-1.312611122	0.872669065	-4.509272625	5.60845E-05	-2.161000599	0.017959196
AT3G11430	GPAT5	2.926352249	0.262800818	0.356106226	0.898743089	-0.76374876	0.81326973	-1.102705422	0.541116454	-1.284219841	0.025797515	-2.195760474	6.36892E-14
AT2G28160	FRU	1.42903449	0.616466449	-0.995133442	0.667228396	-3.205525046	0.051693406	-3.456339251	0.172638291	-1.743382511	0.005760791	-2.203120931	1.143E-06
AT1G55990	AT1G55990	2.839778522	0.227663943	1.19044411	0.611249039	-0.042264339	0.992300644	-1.226853557	0.445223268	-1.036383877	0.023991999	-2.254371021	8.88309E-29
AT5G11920	cwINV6	6.462763916	0.246433526	0.885109601	0.832667476	-5.188461677	0.142707071	-1.555430074	0.696839339	-1.771808178	0.006551316	-2.258433356	3.88779E-07
AT1G74770	AT1G74770	0.102745569	0.956373312	-2.674623794	0.25503282	-0.819218243	0.761498176	-1.385974893	0.527270121	-2.400211451	0.000266146	-2.281349594	1.20186E-07
AT1G61750	AT1G61750	3.30306457	0.149860261	-5.460046351	0.306433138	-5.841660059	0.164199733	-2.473187337	NA	-2.262423606	0.004883803	-2.323359749	1.04448E-05
AT5G15130	WRKY72	2.921207041	0.066659079	-1.189395584	0.687627114	-0.391464338	0.855938314	-1.501717919	0.09032468	-1.125891411	0.035605525	-2.325518294	1.87162E-08
AT5G06839	TGA10	1.772349246	0.413108628	-0.483508323	0.88394223	0.004418841	0.999047782	-3.370229679	0.336341964	-2.17540219	0.010209078	-2.358664686	6.48149E-07
AT5G01330	PDC3	1.278158254	0.741936498	-5.711708317	0.200621982			-1.639934754	0.674766805	-1.475310354	0.000112525	-2.403774007	2.28055E-06
AT4G05200	CRK25	0.751388056	0.809364636	0.907919665	0.794439613	0.896044363	0.771423795	-2.029541068	0.621192363	-2.213920579	0.046194304	-2.419931773	2.28805E-08
AT4G26010	AT4G26010	-1.296295919	0.747764649	-3.361240474	NA	0.436673268	0.939872684	-8.004170387	0.084215519	-3.113683828	0.00900571	-2.426041231	0.002320146
AT3G20380	AT3G20380	1.160007243	0.786319335	-2.988513122	0.223469231	-0.279451743	0.854874193	-2.752191042	0.441076488	-2.028543717	0.001364026	-2.427222193	8.20183E-21
AT1G24320	AT1G24320	-6.65444054	0.157868866	-6.206843161	0.238267828			-2.401063959	0.550996121	-2.093410544	0.015931654	-2.545738234	0.001162507
AT1G13300	HRS1	-0.995254428	NA	-2.26536935	0.147678892	-0.089694891	0.932269174	-1.321268745	0.835453631	-1.110205327	0.001398585	-2.557809569	1.22531E-26
AT2G18980	AT2G18980	3.075317449	0.19078764	-0.059871472	0.982531795	0.07825652	0.987403907	-3.253326215	0.565728752	-1.717748017	0.000716977	-2.661047117	2.62024E-10
AT4G33610	AT4G33610	0.900192537	0.411729032	-0.24545907	NA	1.68482989	0.11466842	-1.603070868	0.109029762	-1.059209543	0.011952942	-2.671858189	3.87375E-69
AT2G22630	AGL17	1.598695649	0.767783305	0.935155152	0.832845671	-3.916852234	0.105733743	-3.000654953	0.706790319	-3.200762957	0.046897684	-2.673796949	0.045871013
AT1G64480	CBL8	-4.780151407	NA			-4.569087875	0.39818496	-6.733498929	0.550996121	-8.240898849	0.000156727	-2.723015395	0.000450461
AT3G23190	AT3G23190	0.54746194	0.894162543	-0.627937277	0.85456374	-0.635857974	0.875638618	-1.744337879	0.66501717	-3.586151053	1.39218E-08	-2.789835387	9.65589E-05
AT3G09220	LAC7	2.593544345	0.0722803	-0.563528269	0.653097038	-1.875747678	0.39490366	-2.290292468	0.159498309	-2.122809562	0.000194258	-2.836183028	1.72103E-25
AT3G03500	AT3G03500	6.563672629	0.200316216	-6.490537971	0.155707179	3.797624877	0.488851194	-0.904586637	0.86582927	-2.172501021	0.044328688	-2.854488814	0.027528681
AT3G46330	MEE39	2.963447361	0.249704965	0.290059631	0.942332968	0.626852471	0.843296988	-1.222458907	0.786453057	-4.040094365	6.29912E-06	-3.306397268	4.03E-16
AT2G15370	FUT5									-7.708257014	0.004335575	-3.433996368	0.019238384
AT4G15393	CYP702A5	2.482435425	0.654419394	-3.723608976	NA			-1.36587399	0.844611065	-3.767734767	0.002301529	-3.545752506	6.64779E-08
AT4G13420	HAK5	5.814599351	0.304415798	22.54550677	NA	2.099918563	0.725215362	-2.575254432	0.602119156	-2.845650598	0.01550758	-3.546404069	0.002392871
AT3G24300	AMT1;3	2.381745736	0.353408009	-3.316959582	0.119234331	2.567180872	NA	-2.602600624	0.465456099	-1.8986984	0.000742404	-3.584814634	2.50769E-19
AT5G58010	LRL3	0.768225971	0.87258627	2.062261155	0.524010722	1.472271329	0.730401963	-2.760119649	0.653363031	-7.200712381	0.013828969	-3.793410159	0.0299717
AT1G19390	AT1G19390			6.490566015	0.2155141			-0.425226474	0.982356953	-7.066825688	0.023316141	-3.926997947	0.026476081
AT3G47040	AT3G47040	3.718535445	NA	-5.115600849	0.340926894	0.447976014	0.939783531	-3.260913618	0.598901706	-3.969415342	0.001398585	-4.283653926	0.004661274
AT2G44220	AT2G44220	-4.692608195	NA	-1.555878094	NA			-1.534088189	0.895706838	-4.64224659	0.000346838	-4.416204406	0.00452619
AT1G23720	AT1G23720	3.080687897	0.548419909	-7.127587354	0.063474748	0.548679841	0.902925112	-1.062539044	0.691895802	-2.772913484	3.37913E-06	-5.149629589	0.033843981
AT1G45015	AT1G45015	5.994853605	0.287876683	-1.121941465	0.777093349	-2.002730959	NA	-2.52285412	0.665778009	-2.008649632	0.025426925	-5.750068998	1.30004E-08
AT2G39530	AT2G39530	5.111906965	NA	1.237510432	0.824282915			-1.81516391	0.663046749	-2.538671662	0.011521702	-5.811899282	0.011633457
AT1G50090	BCAT7							1.789095486	0.833592548	-7.528848789	0.004419645	-5.953489913	0.030215018
AT1G64910	AT1G64910							-6.445269309	0.598901706	-7.684547458	0.003976804	-6.097905245	0.009611299
AT3G21340	AT3G21340	3.941445652	NA	-0.678811792	NA	-4.433215038	0.414155513	-6.229437821	0.433558429	-7.636449382	0.002879839	-6.835447016	0.001589017
AT3G21180	ACA9	4.456967053	NA			6.234070288	0.168956535	-3.776696482	NA	-7.536445427	0.004043622	-7.061291667	0.00369373
AT1G62980	EXPA18			2.402463948	0.684514664	5.076371217	0.341376062	-7.504291877	0.134386152	-8.625889778	2.59663E-05	-8.763581118	0.003485655
AT2G27535	AT2G27535	5.721316669	0.313013942					-0.530568309	0.944628225	8.968163607	1.59818E-06	-2.50311969	0.20738869
AT2G34230	AT2G34230	5.836866552	0.302165345	2.453530178	0.466953085	0.54462339	0.850394242	-0.702155005	0.964175862	8.041848127	0.000604453	1.552769522	0.060154955
AT2G09225	None	-1.358691846	0.676270318	-0.988747838	0.785084375			0.975079416	0.910659889	7.929370975	0.001125943		
AT2G47275	ath-MIR403	0.842336979	0.910112115	0.7367606	0.911153698	3.631123952	0.154308658	1.434292419	0.859442872	7.917519984	0.001928768	-1.859528867	0.212586769
AT3G44753	None	4.456967053	NA							7.904959959	0.00113082		
AT4G25885	AT4G25885	3.13179887	NA	-3.88709122	NA	5.532519125	0.292818218	0.842232783	0.93556951	7.822427468	0.001827997	0.545686113	NA

AT1G51820	AT1G51820	0.90445598	0.851720532	1.588918736	0.611983839	-0.005738204	0.999411864	6.635107968	0.588156522	7.72830545	0.014778479	1.995755775	0.673857679
AT5G35480	AT5G35480	-3.890022824	0.149177359	-2.236530686	0.172934279	-0.325281468	0.518873068	-3.17458129	0.725986946	7.600351368	0.00494956	1.205544093	0.246619733
AT1G58215	None	1.890096913	0.596718244	0.899215252	0.873177566			-6.501853841	0.587695712	7.589006533	0.003963365		
AT1G66960	LUP5							-0.573145914	0.972025023	7.565909532	0.009054015	-2.979290006	0.098632268
AT4G31685	None	-3.776306884	NA					-0.387348674	0.982749202	7.55925167	0.025797515		
AT3G09640	APX2	0.385026494	0.924304619	3.14497107	0.386366641	-0.724282963	0.87786857	-5.731935808	0.632278424	7.41489386	0.035397374	2.380052982	0.086952613
AT2G38152	AT2G38152	5.018731476	NA	-5.148842834	0.337071848	4.958816351	0.354398923	-6.965662904	0.48968933	7.405884893	0.034099576	-0.443096115	0.843046157
AT2G09710	None	2.516862281	0.461130207	-2.629884539	NA			2.112708753	0.87876612	7.387940615	0.00875737		
AT2G42150	AT2G42150	0.484756661	0.950206417	3.399734915	0.233212084	0.982619028	0.550728949	-7.702183692	0.2995105	7.35717914	0.04012736	-0.471623563	0.573785288
AT1G09917	None	-0.058682896	0.992392818	1.564195561	0.662195646			4.212975528	0.709586436	7.355655201	0.010097848		
AT5G58595	AT5G58595									7.29468655	0.025966041	-0.955547282	0.338810978
AT1G21850	sks8	5.359991114	0.346982987	-3.553772892	NA			-4.16347141	0.711255369	7.259762244	0.04927545	-0.416086559	NA
AT3G48310	CYP71A22	-1.739834089	0.057300005	-0.650951053	0.36993859	-0.367552715	0.242963292	-0.085411917	0.99607627	7.247030988	0.048251654	-0.878180157	0.723343016
AT5G16530	PIN5	-5.743094804	0.310470189			-3.780958982	0.491732111			7.021758402	0.040223026	0.921897913	0.826370774
AT2G26720	AT2G26720	-4.774989952	NA	-2.876163836	NA	-4.734158184	0.380168055	-4.626902805	0.685495817	6.971991067	0.036070805	3.569522806	0.252294675
AT3G03825	None	-1.872504572	0.630580323	-1.559904608	0.723972259			-4.505079083	0.692121623	6.145838693	0.012283793		
AT2G36985	ROT4	2.935703901	0.511300627	-5.388084317	0.059962031	-3.3027864	0.550728949	0.568075593	0.939540455	5.954788914	0.02326523	0.809451144	0.092366552
AT2G22821	AT2G22821	-2.435507556	0.326367919	-0.0909277	0.976592039	-3.731489567	0.130241529	0.083921174	0.996868544	4.846273445	0.02014193	-3.395725928	0.496718601
AT1G06977	None	-0.765034646	0.878809942	-4.96600625	0.357008311			0.369724015	0.982055475	4.443742064	0.043902091		
AT3G22231	PCC1	-2.921020928	0.29813833	0.356123586	NA	-1.896984324	0.213108287	2.007255857	0.770575751	3.870838233	0.007571125	-0.181506718	0.922100551
AT1G11112	AT1G11112	0.499829731	0.844173284	-2.511991603	0.360627815	-0.978014451	0.426273735	1.099856471	0.830747236	2.095164439	0.044701841	0.462116434	0.359451439
AT2G43520	TI2	1.387303799	0.178926379	0.888589797	0.208237684	-0.768404627	0.188511801	1.869356183	0.172638291	1.704687648	0.048587162	0.966924133	0.013161004
AT2G12170	AT2G12170	-1.283818769	0.279837848	-0.227940538	0.81559572			-0.282235838	0.917312631	1.658602099	0.013500187		
AT1G61450	AT1G61450	-1.276311071	0.241992947	-2.822786249	0.053491433	-2.229321104	0.301383177	0.427407045	0.885961583	1.503559826	0.012654908	0.471942597	0.507453653
AT3G52770	ZPR3	1.022571013	0.305742438	0.748707174	0.414887118	0.310729779	0.747633919	0.085790621	0.978783612	1.459631315	0.035821868	0.701946634	0.062240357
AT4G22760	AT4G22760	-0.012651696	0.99256625	0.969173494	0.288936795	0.705144496	0.353009616	0.602758617	0.726214276	1.432219088	0.014274267	0.184771161	0.719365777
AT4G28950	ROP9	-0.673121892	0.703458496	0.14106768	0.927278129	-2.047444906	0.128631719	-0.034210347	0.993080608	1.37501928	0.023675952	0.46738873	0.094617767
AT1G29270	AT1G29270	2.144793853	0.262742465	-0.378593621	0.804465117	1.563034979	0.190879686	0.368736933	0.921545622	1.140907988	0.007015924	0.455484695	0.058980187
AT2G22650	AT2G22650	0.533804838	0.254838428	0.493220899	0.184891312	0.889582939	3.7147E-05	0.871605247	0.640704059	1.135279123	0.000321073	0.963497551	8.23735E-08
AT5G27890	AT5G27890	-0.405827356	0.561856248	-0.725289157	0.090058974	0.866385006	0.021766161	0.896706331	0.439459487	1.119787808	0.003173408	0.643696192	0.020159847
AT2G27400	TAS1A	0.255065647	0.802330688	-1.110394353	0.114340171	-0.572671614	0.420663544	-0.105697437	0.975645787	1.107564839	0.000642158	0.316737868	0.357141435
AT5G40600	AT5G40600	-0.414239934	0.543614052	0.114478858	0.849840071	-0.076828017	0.894907926	0.605320747	0.614869324	1.105663169	0.040223026	0.638113195	0.019183642
AT4G14480	AT4G14480	-0.048764462	0.953107384	-0.03449774	0.960186234	-0.421785352	0.325659336	0.053462966	0.982749202	1.094928562	0.047888714	0.046720229	0.937276497
AT4G07455	None	0.571602791	0.382562585	-0.059201859	0.902777359			0.961507462	0.260339395	1.058712497	0.012755316		
AT5G03860	MLS	0.703294011	0.256705206	0.902367937	0.012536562	0.998890773	0.013418132	0.359589773	0.827716472	1.053572626	0.010584722	-0.156742388	0.661050559
AT3G17668	ENA	0.542195526	0.529625603	0.822873486	0.086657369	0.226670894	0.511946084	0.885454706	0.542186287	1.031938409	0.019659532	-0.180891923	0.66210259
AT4G06598	AT4G06598	-0.117971421	0.897466185	-0.373890754	0.459680415	-0.19391871	0.428167198	0.34207855	0.731041294	1.030320734	0.005538541	0.303976252	0.071383481
AT1G09467	None	1.451920356	0.157176658	-0.158942751	0.866922987			0.454049071	0.851334862	1.00846767	0.047065245		
AT5G22110	DPB2	-1.174491331	0.160258849	-1.715704616	0.134805415	-0.949254324	0.093970882	-0.579720565	0.691745399	-1.03015189	0.021497751	0.004367342	0.992804029
AT4G32460	AT4G32460	0.923369864	0.123229465	0.354111961	0.651888452	0.268366984	0.669022284	-1.807292923	0.152918235	-1.057619421	1.08471E-14	-0.123350897	0.617697122
AT1G02810	AT1G02810	0.667188427	0.662289149	-2.44494842	0.281483628	-0.298740618	0.897309309	-0.824414807	0.718989301	-1.096940001	0.014385114	0.87274417	0.000110301
AT5G11670	NADP-ME2	-0.193264152	0.573660301	-0.821319107	0.001196924	-0.950826798	2.461E-07	-0.585235335	0.644602447	-1.104571524	1.44602E-07	-0.663523735	1.35557E-09
AT2G43150	AT2G43150	1.228524507	0.208427883	-0.191716652	0.75628224	0.86630877	0.508980697	-0.681570642	0.777083998	-1.167693433	0.013770802	-0.647187977	7.69691E-06
AT5G60660	PIP2;4	0.31675096	0.84119697	-0.162457296	0.941759704	0.361662722	0.733499293	-0.954961005	0.717727383	-1.207508672	6.18266E-12	-0.984668656	7.03634E-06
AT3G52370	FLA15	-1.777206296	0.313088386	-0.818552824	0.53006929	-0.665242329	0.700505937	-1.596090933	0.681687594	-1.242535228	0.012630338	-0.267834304	0.289955696
AT1G09560	GLP5	-0.123411739	0.848385272	-0.580884319	0.190962648	-0.470764213	0.10837333	-1.109176516	0.296836814	-1.249840652	9.37228E-17	-0.982522806	1.34054E-12
AT1G62440	LRX2	0.865835606	0.597060208	0.10235985	0.953676661	-0.430309592	0.782633269	-2.022424124	0.109771855	-1.27144293	6.41492E-05	-0.67105739	0.028310892
AT4G33550	AT4G33550	0.329333584	0.915589343	1.884305524	0.119728615	-0.149093537	0.973201648	-1.057365005	0.805858204	-1.30040521	0.016862285	-0.421151639	0.148392197
AT5G62380	NAC101	-1.267958219	0.643269557	-2.063692237	0.372271241	-0.491686866	0.824625366	-1.095242032	0.809598664	-1.342389419	0.047065245	-0.230764953	0.705077442
AT3G51180	AT3G51180	-0.719275023	0.325168226	-0.039053756	0.970417834	-0.352264409	0.64451125	0.292307756	0.916241006	-1.395828871	0.044328688	-0.149272429	0.77280831
AT2G21140	PRP2	-0.275713905	0.690820084	0.405932509	0.481302528	-0.422158293	0.180361297	-1.417509835	0.125085126	-1.410871438	2.70372E-09	-0.875460671	2.23534E-12
AT1G10550	XTH33	-0.697312431	0.671215935	0.74203085	0.564097384	-1.696235611	0.238510172	-1.457169628	0.386146892	-1.412154736	1.50087E-05	-0.919653375	0.013023859
AT5G48430	AT5G48430	1.465212333	0.350839858	1.308744508	0.534556337	-3.394071639	0.129253355	-1.398013378	0.303024698	-1.448336154	0.046984347	-0.339538512	0.48486241
AT5G48070	XTH20	7.435313578	0.062161133	5.300264539	0.10953007	5.395665139	0.25490479	-1.938325789	0.62738272	-1.487954963	2.98293E-07	-0.78300148	0.015479672
AT1G65310	XTH17	0.056685116	NA	-0.99709958	0.374590335	-2.391548451	0.353163742	-0.624686575	0.834747602	-1.505273969	3.971E-12	-0.55287405	0.000136695
AT5G48290	AT5G48290	3.028045526	0.387834765	-0.753201769	0.878054411	0.752270991	0.898082883	-0.773578666	0.885840009	-1.51850993	0.010714218	-0.807074285	0.107173391
AT2G43590	AT2G43590	-0.949414917	0.289022497	1.910917165	NA	0.187605189	0.941267781	-1.465563917	0.807595594	-1.53383337	0.000319499	0.087630536	0.953069394
AT5G40590	AT5G40590	5.879906255	0.298216422	2.023333405	0.513048326	1.59712298	0.776753018	-1.787246449	0.665119721	-1.692183452	0.02424068	0.801524719	0.224017839
AT3G03530	NPC4	-4.54583291	0.189336523	-4.486087168	NA	-5.620209964	0.284277795	-1.459816453	0.717709314	-1.970310138	0.036036029	-0.690920453	0.334068287
AT2G45220	AT2G45220	2.07287812	NA	0.501492534	0.887727364	1.574957215	0.301486328	0.136340295	0.975907193	-1.976368475	0.000919738	-0.931753482	0.000467104
AT1G78860	AT1G78860	0.114381319	0.957590121	-1.3535644	0.347139861	0.24280746	0.839772638	-1.04400066	0.598901706	-1.976757444	1.37882E-12	0.016541451	0.946507734
AT3G15370	EXPA12	6.265874143	0.263905113			-2.648405744	NA	-2.088013519	0.647947756	-2.027520727	0.032971059	-0.494945075	0.762616382
AT3G02240	RGF7	3.057647703	0.474099263	1.677103292	0.470674531	0.571643032	0.9025159	-0.652983265	0.914660637	-2.136115993	0.038816205	-0.926481918	0.145525573
AT3G01345	AT3G01345	-0.564917692	0.633279735	1.625996529	0.257849355	0.612398382	0.19155569	1.267409679	0.509467924	-2.177956309	0.049929633	-0.784993547	0.004317113
AT5G46900	AT5G46900	21.79265772	NA	-5.395308256	0.312474532	1.450091902	0.809618896	-2.122875587	0.606517391	-2.198062614	0.000531296	-5.311855644	0.105925735
AT2G25810	TIP4;1	7.363294628	0.06745682	0.372579849	0.943594344	-2.212341769	0.644174188	-2.294839056	0.557888463	-2.237531669	0.003092915	0.100810405	0.791640653
AT4G12330	CYP706A7	4.027742266	0.105207088	-0.333751515	0.950039021	-5.145518534	0.334196031	-1.09881631	0.809492797	-2.327549336	0.022049394	2.700540725	0.339003026
AT5G45070	PP2-A8	3.518332926	0.243673951	2.732163335	0.18025076			-3.632865136	0.464366799	-2.717545641	0.000826602		
AT1G15625	None	5.432773189	0.339653767	-4.39926821	NA			-3.046344367	0.431282679	-3.046540057	0.037262273		
AT3G28550	AT3G28550	8.744996513	NA	-1.133345477	0.852840734	-0.123455853	0.9836973	-1.994163968	0.624615508	-3.106477815	0.029096286	-3.825432022	0.153573313
AT4G09080	TOC75-IV	-4.458474338	0.200984596	-0.250321166	0.923909876	-0.060885864	0.979581166	-1.907693773	0.664749823	-3.2554834	0.021989642	1.138366696	0.115288442
AT1G50050	AT1G50050	-5.244526985	0.35786256	-0.462399515	0.916773351	1.977067651	0.689523292	-0.29391887	0.971723999	-3.361330678	0.012255952	-3.779917637	0.082968892
AT3G29630	AT3G29630	-1.458552971	NA	-5.308070452	0.321100825			-1.61522622	0.881148813	-3.46782829	0.037100036	-3.135817749	0.058333719
AT4G09110	AT4G09110	6.384451536	0.251174117					-6.582013643	0.577728436	-3.572275188	0.038541019	-3.046852356	0.128784929
AT1G49030	AT1G49030					5.269668334	0.319976681	-1.180541454	0.829508496	-3.825963792	0.011056726	-1.669709123	0.083972637
AT3G11370	AT3G11370			-5.17700955	0.33432887			-6.374119888	0.091443847	-4.194545277	0.030861611		
AT1G53950	AT1G53950	5.384033117	0.344821053					0.093975026	0.994162268	-4.775976289	0.044328688		
AT1G12560	EXPA7					-1.108056393	NA	-6.783675619	0.197650176	-5.112093772	0.000742404	-6.188172732	0.110206512
AT5G46600	AT5G46600	0.129891819	0.983922132	0.639130432	0.904230982	-1.151022885	0.680985267	-1.23845179	0.893088744	-6.219651792	0.047902811	-1.332055772	0.113299553
AT5G38540	AT5G38540	6.28238192	0.262742465	-4.39926821	NA	-5.381902313	0.172491461	-4.622602053	0.245156267	-6.769179452	0.00974561	-1.177866833	0.051923435
AT3G20710	AT3G20710	3.454782108	NA	1.709593877	0.784654474			-5.862950022	0.62738272	-6.836371171	0.044328688		
AT2G34238	AT2G34238	4.835996915	NA	-5.228221573	0.329178684	0.592427525	0.919478525	-6.450423904	0.598901706	-6.938419034	0.038016346	1.311916531	0.546381291
AT5G41540	AT5G41540	-2.080575966	0.753598444					-5.006506075	0.665778009	-6.977647711	0.042117574		
AT1G63550	AT1G63550					0.155698386	0.969945712	-5.53875504	0.639875152	-6.989422043	0.031111468	-1.592162107	0.249190273
AT3G10750	AT3G10750	3.173170735	0.453277927	-0.833384749	0.882499279	0.02646944	0.996980515	-3.315999258	0.721140128	-7.055538515	0.027729674	0.572375445	0.757496632
AT3G13840	AT3G13840							-5.097402913	0.663046749	-7.177078891	0.018052978	-0.677826733	0.76604489
AT3G01572	AT3G01572	0.913619628	0.85482932	-7.238807491	0.056779088	0.267528718	0.919695967	-0.435089995	0.980081612	-7.30271804	0.013828969	-0.880139806	0.390741209
AT5G54040	AT5G54040	3.454782108	NA	-2.876163836	NA			-2.565384331	0.720626114	-7.306206449	0.011278476	-1.377867238	NA
AT2G24710	GLR2.3							-6.946200284	0.497907624	-7.346150225	0.03653082		
AT5G46960	AT5G46960					-3.322614377	0.550728949	0.585138107	0.975836766	-7.353511756	0.035797034	-0.951763645	0.843022062
AT2G40910	AT2G40910			-3.679017858	NA	0.723029958	0.889619853	-0.355853671	0.982734229	-7.35546124	0.010418974	-2.21151405	0.082318375
AT2G44110	MLO15	-0.296005248	0.969441835	1.731801401	0.780134425	5.669938712	0.278960536	0.148899628	0.991238447	-7.424481668	0.030288046	-5.287726628	0.173548321
AT1G07160	AT1G07160	-2.859631856	0.131326371	0.234876965	0.930855836	0.636897914	0.757792173	-2.425929263	0.611321113	-7.438526462	0.007573516	-0.293131579	0.905297287
AT3G44710	AT3G44710	5.368733387	0.346197649	-3.761655451	NA			-4.854473757	0.67307941	-7.512541595	0.006125864		

AT5G60010	AT5G60010							1.016414771	0.955209405	-7.518711411	0.005752538		
AT1G69240	MES15									-7.534513333	0.005241583	-5.79181618	0.080857591
AT5G40990	GLIP1									-7.67240254	0.019252548	0.882909848	0.832405719
AT1G78410	AT1G78410	-2.353935936	0.174505984	0.610512466	0.684856964	-3.185165748	0.221526907	-2.854060018	0.632278424	-7.680213514	0.004708376	1.641899004	0.056560265
AT3G13850	LBD22	-0.91464807	0.837818897	-3.913709836	NA	-4.32704903	0.426695818	-0.672686533	0.958809354	-7.689322001	0.014165114	1.59462625	0.506283058
AT4G19680	IRT2							-2.244427398	0.824370324	-7.791317075	0.001364393	0.047395957	0.988233344
AT3G09925	AT3G09925			-3.761655451	NA			-2.70334064	0.684861858	-7.953740629	0.000770237	-2.011539594	0.611046505
AT5G01900	WRKY62	5.436457002	0.339653767	-4.591953932	NA	1.279391133	0.751694304	-6.914047472	0.505950107	-7.967257554	0.000667383	-1.329720284	0.224866019
AT3G24600	AT3G24600	0.301795853	0.930035011	0.096329627	0.986707039	1.467862618	0.636298064	-6.427031496	0.600465892	-8.010041532	0.000919738	1.271099679	0.112543797
AT5G65980	AT5G65980	5.471266415	0.251670707	2.082134414	0.72872036	6.108605181	0.100681473	-2.993949669	0.697407195	-8.030164177	0.000395037	-1.629201842	0.054241233
AT5G58310	MES18	0.877207334	0.855496092	0.758158679	0.908096569	0.01791121	0.995492744	-2.427799773	0.73461774	-8.074978602	0.000531296	1.191243154	0.693072272
AT1G29830	AT1G29830			-1.539799123	0.783986664			-6.734176391	0.548879103	-8.13650724	0.000449414	-2.216080024	0.269973103
AT1G12040	LRX1							-0.731340542	0.966082349	-8.298376625	1.69434E-06	-7.884310041	0.120301559
AT1G26420	AT1G26420	-2.184898395	0.740779373	3.446358412	0.131782478	1.9167702	0.740419297	-2.322147043	0.637614678	-8.303942677	0.000170736	-1.37782297	0.291898217
AT5G15890	TBL21	2.197303023	0.557906115	2.059963628	0.564464658	-3.711657614	0.501349907	-6.718875964	0.562683359	-8.359619676	0.000202772	0.793725583	0.724715556
AT5G20470	HDK			-2.139964672	NA			-7.033796618	0.196045896	-8.550796952	1.34127E-05	-2.568278832	0.51993002
AT4G01535	AT4G01535			-4.898565306	NA	0.400312148	0.903274976	-6.756113677	NA	0.400312148	0.903274976	7.993221823	2.0421E-07
AT2G20350	AT2G20350	1.156616053	NA	-5.030431333	0.350293706							7.862894279	1.171E-06
AT2G28580	AT2G28580	-5.774293582	0.30770498	1.79032324	0.771087491	-1.000908022	0.871467426	-0.819238564	0.957721682	-1.000908022	0.871467426	6.944439737	0.00015331
AT4G29640	AT4G29640	1.259254802	0.842644696					-3.023408174	NA			6.808095685	0.000861158
AT1G75050	AT1G75050	-0.668107038	NA	-3.591666303	NA			-4.16347141	0.711255369			6.590177761	0.001526252
AT2G17850	AT2G17850	3.941445652	NA	-5.57868488	0.294646519			-5.701270161	0.632534636			6.459883295	0.011642064
AT5G21960	AT5G21960	-3.027152556	0.0597342	-2.976448918	0.241245404	-1.44211172	0.519412968	1.24285911	0.92984753	-1.44211172	0.519412968	6.360277157	0.000324333
AT5G50790	SWEET10	1.903847823	0.703277206	2.861508084	0.189171504	-5.783965704	0.104770859	-3.023408174	NA	-5.783965704	0.104770859	6.246389088	0.003704923
AT3G19085	AT3G19085	-0.41003934	NA	3.20351308	0.5289687	-3.00539233	0.53335352			-3.00539233	0.53335352	6.228913821	0.008668661
AT3G05975	AT3G05975	-4.55852849	NA					5.107974372	0.663046749			6.184534639	0.042802514
AT1G70390	AT1G70390			-4.051255346	NA			-7.269870992	0.418113948			6.149132804	0.027205801
AT1G72350	AT1G72350			6.876747928	0.184541871	2.951852217	NA			2.951852217	NA	6.056994135	0.018969958
AT1G58050	AT1G58050											6.007181694	0.021970953
AT1G61680	TPS14	3.718535445	NA	-1.696102729	0.786146042	-4.321639773	0.426712153	4.93845489	0.66808962	-4.321639773	0.426712153	5.964328956	0.013806719
AT1G76640	AT1G76640	-1.308128868	NA	0.184794628	0.97848721	-0.126932094	0.966960294	-1.764129882	0.812285774	-0.126932094	0.966960294	5.822883628	3.84019E-11
AT4G20970	AT4G20970							-4.854473757	0.67307941			5.780882315	0.026021919
AT5G25920	AT5G25920			0.47585789	0.942911299			-1.548709109	NA			5.71902559	0.026229698
AT4G31740	AT4G31740					-4.624622085	0.392044903			-4.624622085	0.392044903	5.697681067	0.024378984
AT5G42640	AT5G42640											5.602966832	0.029588266
AT1G22810	AT1G22810					0.022653296	0.988325398			0.022653296	0.988325398	5.519614561	5.74226E-23
AT1G21320	AT1G21320	-3.650467883	0.543614052	0.733815	0.877861114	1.236752694	0.697097372	0.777859316	0.96644259	1.236752694	0.697097372	5.448528141	0.010054354
AT4G01420	CBL5	-4.512225933	NA	6.020756943	0.25503282	3.488012422	0.528531154	-4.709284728	0.680482607	3.488012422	0.528531154	5.426569451	0.010847757
AT1G23500	AT1G23500	-5.101726022	0.372397395	-1.541274485	0.757803669	-3.884621112	0.346360748	6.194361035	0.611321113	-3.884621112	0.346360748	5.395134062	0.012856969
AT5G60350	AT5G60350	2.126870043	NA			4.849701515	0.366175561	-6.958450406	0.505881625	4.849701515	0.366175561	5.355482063	0.01467957
AT5G05300	AT5G05300	-0.245362657	0.954418772	0.816918134	0.823810442	0.61040749	0.838623271	2.102088755	0.781016963	0.61040749	0.838623271	5.165240743	0.001911401
AT4G01360	BPS3	-0.333463334	0.672838559	0.193940309	0.781915365	0.930682371	0.497325732	-0.006743338	0.9987964	0.930682371	0.497325732	5.081776461	5.57935E-09
AT2G32140	AT2G32140	0.474845863	0.870932148	-1.626961585	0.058693424	-4.431074623	0.414289384	0.123288342	NA	-4.431074623	0.414289384	4.921801487	0.033312341
AT2G44570	GH9B12	4.287027399	0.179614447	1.039816695	0.378243985	0.123022475	0.967669581	7.102820304	0.487759979	0.123022475	0.967669581	4.9117348	0.000377778
AT5G26630	AT5G26630	-2.779198857	0.608083588	-6.436663625	0.219211613			-0.087412778	0.996799452			4.911507271	0.009335425
AT3G55646	AT3G55646	-0.465354213	0.862066085	-0.030022847	0.982765851	-1.627539216	0.070063714	2.571777135	0.219098116	-1.627539216	0.070063714	4.61643034	9.33898E-06
AT1G50750	AT1G50750	-0.150773676	0.850014392	0.45345449	0.385319693	-0.23735276	0.656421668	-0.248605852	0.911737107	-0.23735276	0.656421668	4.614315862	1.0256E-132
AT4G19430	AT4G19430	5.908643371	0.295872049	6.748194672	0.194742577	4.412422616	0.415813719	2.313697274	0.861289322	4.412422616	0.415813719	4.57933327	3.70498E-06
AT2G20150	AT2G20150	2.853577111	0.502323387	0.160311646	0.981326376			-0.924765761	0.932578056			4.561998791	2.43275E-06
AT5G61610	AT5G61610	0.101130652	0.955853125	0.183706811	0.943891459	-0.597047545	0.806568344	0.140304664	0.993804788	-0.597047545	0.806568344	4.348104036	0.003066002
AT2G44930	AT2G44930	-4.09050399	0.183032204	-5.611815005	0.291428483	0.561303295	0.876421535	6.811103265	0.553691236	0.561303295	0.876421535	4.204835038	4.29421E-05
AT5G15800	1-9月	-0.766937299	NA	6.876747928	0.184541871	-5.643518978	0.201929923	5.637476411	0.637614678	-5.643518978	0.201929923	4.193811188	0.016750425
AT5G48050	AT5G48050	-4.997186725	0.383443279	-2.194969744	0.71445082			-3.999643691	0.721140128			4.158389367	0.044337271
AT5G65130	AT5G65130	1.141502115	0.875592918	-3.302458832	0.268582233	-4.368908833	0.421712624	0.961837914	0.929045669	-4.368908833	0.421712624	4.14681622	0.003572488
AT1G01560	MPK11	-1.202816047	0.088949145	0.72329454	0.430963314	-0.448024496	0.65649005	0.253857815	0.949241707	-0.448024496	0.65649005	4.079017872	5.16669E-15
AT2G32780	UBP1	-0.344474575	NA	-5.875020833	0.267759424	-3.700980483	0.502044757			-3.700980483	0.502044757	3.989228107	0.041577828
AT5G17120	AT5G17120					6.035878381	0.138304678			6.035878381	0.138304678	3.984629122	0.002207622
AT1G35183	AT1G35183			-5.842549694	0.270104097	-1.739705102	NA	-6.565117813	0.268420306	-1.739705102	NA	3.968425755	0.033577369
AT1G43800	FTM1	-1.184135585	NA	-3.053101743	0.179358269	3.463676106	0.052544718	-0.187476375	0.982249967	3.463676106	0.052544718	3.8928777	1.84522E-55
AT5G37400	AT5G37400	-0.871854453	0.387896939	0.156841763	0.902332184	-4.433215038	0.414155513	0.15096821	0.971297857	-4.433215038	0.414155513	3.780852622	0.021196351
AT5G51990	CBF4	0.284033765	0.842232884	1.420175696	0.149094134	-0.0103953	0.9931786	0.518842212	0.886772005	-0.0103953	0.9931786	3.694722318	0.041092856
AT4G33390	AT4G33390	0.671006779	0.893726408	-0.392486914	0.952635323	-2.704390577	0.244387631	-3.348775818	NA	-2.704390577	0.244387631	3.684798148	0.010708751
AT1G05880	ARI12							-5.676279699	0.634112695			3.594287008	0.048854529
AT3G01830	AT3G01830	-0.677116213	0.902670805	-1.919032551	0.542737828	0.510015874	0.803417316	5.273465165	0.653363031	0.510015874	0.803417316	3.46711995	1.68062E-06
AT5G60780	NRT2.3	0.25672511	0.960811474	1.022770766	NA	2.815429976	0.511669312	-0.381201998	0.961183622	2.815429976	0.511669312	3.454003799	0.00012618
AT1G57750	CYP96A15							4.962642632	0.66808962			3.375931254	2.40063E-09
AT3G23250	MYB15	0.706053703	0.501212944	0.575782647	0.44184448	-0.40563022	0.75878239	-0.812745293	0.702246294	-0.40563022	0.75878239	3.287799776	1.26301E-22
AT3G59030	TT12	0.355665544	0.887356207	1.923118202	0.500671611	-0.243308208	0.939825344	0.23503069	0.97809523	-0.243308208	0.939825344	3.28407489	0.031989796
AT1G27820	AT1G27820					-0.365433155	0.930478098	-0.721041513	NA	-0.365433155	0.930478098	3.283769937	0.006484572
AT2G22590	AT2G22590	-3.976212381	NA					-5.626171416	0.637614678			3.272586356	0.013751232
AT5G59820	RHL41	-0.542240039	0.387705276	-0.593858288	0.027428447	-0.730749362	0.003163199	0.906486895	0.581307949	-0.730749362	0.003163199	3.222763491	2.20523E-61
AT4G37290	AT4G37290	-1.903148563	0.573509305	-1.277896401	0.74630095	-2.454782743	0.147655555	-1.159418428	0.859442872	-2.454782743	0.147655555	3.186359443	0.000183691
AT4G15440	HPL1	-7.018240585	0.096711235	-1.599035295	0.579589401	-2.805135903	0.171337252	2.89523831	0.632278424	-2.805135903	0.171337252	3.156969917	6.77276E-05
AT5G64905	PROPEP3	0.199469654	0.964766891	-1.692833546	0.527762346	-0.839174852	0.675973981	-3.322961327	0.581307949	-0.839174852	0.675973981	3.114903992	0.000174468
AT1G71450	AT1G71450	-21.84136359	NA	-4.875203671	NA	-3.223321176	0.563907217	2.09036998	0.879613371	-3.223321176	0.563907217	3.111695217	0.010819455
AT5G37760	AT5G37760	-4.824010047	NA	-0.004514596	NA	0.187393301	0.939783531			0.187393301	0.939783531	3.058363824	0.000273249
AT1G09157	AT1G09157	0.878016352	0.557368222	2.627254552	0.191656533	-1.197148116	0.491085819	-6.488395916	0.591481547	-1.197148116	0.491085819	3.023550771	0.017097269
AT5G26920	CBP60G	-0.4277652	0.438845556	0.185660567	0.689233025	-0.678972461	0.094040754	0.249444398	0.916990613	-0.678972461	0.094040754	3.015632319	4.88961E-35
AT2G31100	AT2G31100	-0.058731766	0.985805532	-1.602813625	0.709758647	0.516739996	0.833049958	-5.241650972	0.653363031	0.516739996	0.833049958	2.997337643	0.001309617
AT1G05675	AT1G05675	-2.532411066	0.321996912	-0.266529835	0.800266246	-0.558088026	0.572478772	0.974011679	0.731041294	-0.558088026	0.572478772	2.935671443	0.000362377
AT3G26200	CYP71B22	0.205877432	0.948997247	0.024018813	0.987916618	0.211273554	0.863174507	0.178979483	0.980608627	0.211273554	0.863174507	2.928358757	4.34201E-48
AT4G10265	AT4G10265	0.870926323	0.824857231	1.14100074	0.752751916	-4.087945298	0.453957696	0.03755859	0.996858122	-4.087945298	0.453957696	2.896996322	2.73442E-07
AT1G33960	AIG1	-1.836937455	0.786478739	3.448840869	0.148024451	-3.310567945	0.07600967			-3.310567945	0.07600967	2.891357866	0.001113177
AT1G03880	CRU2											2.885653125	0.020134088
AT5G01015	AT5G01015	6.165655613	0.27254492	-4.460281122	NA	-0.677409493	0.8209707	-2.607242633	NA	-0.677409493	0.8209707	2.885220277	0.012511701
AT5G02720	AT5G02720					1.314688798	0.200962739			1.314688798	0.200962739	2.789312398	8.93152E-05
AT2G32210	AT2G32210	-0.841483286	0.1072281	-0.586111191	0.245618357	0.041341563	0.932433485	0.429245117	0.823368717	0.041341563	0.932433485	2.782512558	3.04375E-28
AT1G65860	FMO GS-OX1	-2.556488597	0.440399503	-1.875154634	0.484112335	-1.832122357	0.298662664	1.689881786	0.718595611	-1.832122357	0.298662664	2.780846803	5.32601E-11
AT2G21260	AT2G21260	4.456967053	NA	-2.175702898	NA	-1.830010518	0.506889479	0.856607965	0.962474985	-1.830010518	0.506889479	2.759946613	0.012181471
AT4G19850	PP2-A2	-0.343103597	0.964705665	-1.555878094	NA	3.18099952	0.452726601	0.750786795	0.965471639	3.18099952	0.452726601	2.704805988	0.039066178
AT1G30190	AT1G30190	-3.730384189	0.135191454	-2.822266311	0.092488687	0.049786532	0.979581166	-0.039162579	NA	0.049786532	0.979581166	2.692083316	3.61423E-06
AT2G22880	AT2G22880					-0.324926772	0.426712153			-0.324926772	0.426712153	2.685004969	1.21035E-30
AT1G66860	AT1G66860	5.368733387	0.346197649			2.631117117	NA			2.631117117	NA	2.640994408	0.005270895
AT3G56220	AT3G56220	0.259675669	0.951578428	-1.020289149	0.736393249	-2.164588169	0.287720376	0.791314285	0.905404361	-2.164588169	0.287720376	2.616618537	1.90766E-08

AT5G43290	WRKY49	0.195172833	0.930035011	-1.120659773	0.535428271	-1.126740749	0.600505638	-1.849944145	0.313973003	-1.126740749	0.600505638	2.587409151	6.70697E-05
AT1G76600	AT1G76600	-0.19856393	0.79315624	-0.4322348	0.182712513	-0.002018926	0.99688297	1.280188041	0.477936046	-0.002018926	0.99688297	2.568668266	3.77248E-89
AT4G25750	ABCG4	-0.510068752	0.836550078	-1.59160616	0.72882663	-0.469374413	0.67055554	1.653271123	0.707962062	-0.469374413	0.67055554	2.530868189	2.29082E-17
AT1G18740	AT1G18740	-0.863538421	0.065086781	-0.653760254	0.002010467	-0.68386058	0.285030973	1.463741403	0.175483631	-0.68386058	0.285030973	2.511941528	1.1134E-137
AT2G30020	AT2G30020	-0.540133014	0.309418844	-0.532648275	0.057315475	-0.098270998	0.933765776	1.615358599	0.23243323	-0.098270998	0.933765776	2.47175041	1.29141E-51
AT4G03050	AOP3					-3.453559977	0.116995126			-3.453559977	0.116995126	2.404653822	0.001963677
AT4G24110	AT4G24110	0.082520028	0.943663747	0.173576774	0.864097441	0.003198058	0.996715352	0.662797639	0.644839236	0.003198058	0.996715352	2.393371499	1.01413E-09
AT2G26400	ARD3	4.215741514	0.204993825	-1.30098023	0.751695527	1.618175177	0.620910111	1.642898483	0.706266671	1.618175177	0.620910111	2.376437897	5.53756E-13
AT2G47050	AT2G47050	-0.682451422	0.691379374	1.4492702	0.523651604	-1.882966852	0.368921589	3.456884721	0.662219342	-1.882966852	0.368921589	2.328323375	0.00601473
AT2G25460	AT2G25460	0.06103688	0.950098718	0.419228396	0.433062423	-0.399700168	0.517384039	0.992647379	0.76611969	-0.399700168	0.517384039	2.321563155	9.43505E-15
AT4G28405	AT4G28405	-1.374262347	0.585655556	-2.130875985	0.334488858	0.597014766	0.87107194	-0.714244212	0.953844689	0.597014766	0.87107194	2.270685102	0.028814278
AT2G17660	AT2G17660	-0.718980665	0.509755423	-1.18936276	0.129757572	-0.26990217	0.833326078	0.327776173	0.968467209	-0.26990217	0.833326078	2.262778505	0.000130027
AT1G63140	AT1G63140	0.471785533	0.95134967	-0.471588047	0.933051027	-6.03175939	0.053335801	1.255548334	0.898745998	-6.03175939	0.053335801	2.245057849	0.003550268
AT1G79510	AT1G79510	6.759233646	0.090122037			-1.215841481	0.113834301	-3.862355172	0.731041294	-1.215841481	0.113834301	2.231645275	0.000229272
AT1G30740	AT1G30740	2.627703255	0.417764104	-1.167737524	0.748394747	0.398353161	0.86978297	0.080555323	0.99694718	0.398353161	0.86978297	2.200904233	0.013718344
AT4G10260	AT4G10260	2.126870043	NA	3.550408275	NA			-2.130469655	NA			2.1792993	0.018081536
AT1G61130	SCPL32							3.688986812	NA			2.178848105	0.015271997
AT3G25250	AGC2-1	-0.531284829	0.730876924	1.383296098	0.152307647	0.383962345	0.907940078	-1.42969744	0.629761053	0.383962345	0.907940078	2.167951219	0.00030538
AT3G04410	AT3G04410	1.109800807	0.801053158	4.122374174	0.181900098	-0.276498093	0.905613517	-6.481965831	0.591545374	-0.276498093	0.905613517	2.137103793	0.000737867
AT4G24380	AT4G24380	-0.926012417	0.361170155	-0.12321659	0.91221712	-0.634752491	0.496558273	0.801916818	0.8351738	-0.634752491	0.496558273	2.116481546	5.93865E-15
AT1G44830	AT1G44830	-0.257182906	NA	0.307200044	0.894142914	1.270440605	0.206168443	-0.320503373	0.924925439	1.270440605	0.206168443	2.106100295	1.67748E-30
AT1G58420	AT1G58420	-1.138877251	0.441072987	-0.35475199	0.870815495	-1.560486264	0.385492416	-2.466039999	0.086459345	-1.560486264	0.385492416	2.087175258	3.5578E-09
AT1G16910	LSH8	0.691688413	0.926551246	-1.0491759	0.770387042	2.28135214	0.220902083	0.833587623	0.906561784	2.28135214	0.220902083	2.078655822	4.48917E-06
AT5G43620	AT5G43620	-0.234222324	0.748913859	0.347859085	0.162147412	0.479171363	0.151683671	1.128714096	0.359157502	0.479171363	0.151683671	2.072144932	3.45336E-32
AT3G11000	AT3G11000	-0.993828608	0.468509853	-2.688823087	0.075691585	0.088138497	0.957018438	0.740272147	0.704225272	0.088138497	0.957018438	2.061747029	1.67864E-05
AT3G48520	CYP94B3	0.280723866	0.713182083	0.168977485	0.815577011	0.45032654	0.702060518	0.469355074	0.835453631	0.45032654	0.702060518	2.022590452	1.37434E-15
AT1G30814	AT1G30814	-1.457449641	0.798876559	-4.460281122	NA	-0.367512787	0.94503851			-0.367512787	0.94503851	2.004732115	0.024747485
AT5G08460	AT5G08460			-3.553772892	NA	-0.210130299	0.880268746	-6.591784404	0.577728436	-0.210130299	0.880268746	2.001024077	0.000406364
AT1G06160	ORA59	-0.713214472	0.716612478	-1.320640476	0.650066467	-3.453357612	0.055384209	0.895242282	0.76277889	-3.453357612	0.055384209	2.000843401	0.001025675
AT1G10980	AT1G10980	2.705048597	0.570839905	-4.012984161	NA			0.369414816	0.977607985			1.978584353	0.004740087
AT2G26211	ath-MIR825	-0.406113724	0.902670805	-0.905032488	0.721612623	0.81923443	0.011384338	1.309482066	0.787101126	0.81923443	0.011384338	1.97649991	3.70515E-09
AT1G29680	AT1G29680	0.063246811	0.9629152	0.946320572	0.141898522	0.112153318	0.905777872	-0.097830822	0.984889186	0.112153318	0.905777872	1.953606775	0.007113283
AT1G34245	EPF2	-0.950507178	0.534842397	-0.197520729	0.88764165	1.248805876	0.080511644	-0.144876795	0.976183443	1.248805876	0.080511644	1.928065084	3.97781E-08
AT3G01840	LYK2	-0.573320877	0.697969722	-0.004385886	0.999012133	0.845116525	0.654515625	2.031259802	0.759046954	0.845116525	0.654515625	1.927626908	0.00058635
AT1G65890	AAE12	-4.752434735	NA	-5.051461836	0.348127112	-5.643845742	0.207279189	-5.776362574	0.629761053	-5.643845742	0.207279189	1.895270293	0.044560963
AT3G28500	AT3G28500	6.567726587	0.202672638	5.664949409	0.287163991	-2.952387681	NA	1.139563615	0.895706838	-2.952387681	NA	1.892599569	4.13582E-08
AT3G47750	ABCA4	-0.877194928	0.116121046	-0.552140763	0.212653558	0.466291088	0.249400485	1.358885906	0.37397762	0.466291088	0.249400485	1.885389636	0.001096218
AT5G01100	FRB1	-0.26682084	0.736418362	0.161630931	0.800229605	0.927168007	0.204171818	-0.96710619	0.529380338	0.927168007	0.204171818	1.881250218	3.24369E-11
AT1G50180	AT1G50180	-1.172545028	0.606650256	0.634998826	0.564485966	-0.923639982	0.288367376	0.128538685	0.991559366	-0.923639982	0.288367376	1.870746637	0.00973342
AT4G20920	AT4G20920	0.063260816	0.985842093	0.494862897	0.898667455	0.201089982	0.941962532	0.977641032	0.893088744	0.201089982	0.941962532	1.848017527	0.020496764
AT4G34210	SK11	2.114480739	0.655137622	4.194885002	0.166259123	-0.245549404	0.936947989	-5.130069771	0.662219342	-0.245549404	0.936947989	1.838580264	0.0425233
AT5G52760	AT5G52760	0.160887399	0.969070362	2.38132685	0.115620579	1.439787673	0.481935002	-2.678717952	0.65378996	1.439787673	0.481935002	1.828227407	1.58896E-06
AT5G55450	AT5G55450	5.663800809	0.318296183			0.674141769	0.376935087			0.674141769	0.376935087	1.824884885	3.70243E-09
AT1G68320	MYB62	-0.17440285	0.976794265	3.242067983	0.344851706	1.881560705	0.485502703	-6.419684685	0.348017657	1.881560705	0.485502703	1.820176073	0.035260798
AT1G62560	FMO GS-OX3	-2.204984194	0.059297862	-1.214805592	0.224351971	-1.30316448	0.426300621	1.087969637	0.659288623	-1.30316448	0.426300621	1.817593833	3.6813E-15
AT4G30230	AT4G30230	-3.761390803	0.134478609	-6.796918316	0.112269994	-0.637624358	0.864816024	-1.022698813	0.945609936	-0.637624358	0.864816024	1.795855201	0.019041245
AT1G09950	RAS1	-0.026221623	0.979035311	1.008139545	0.185621175	-0.607601962	0.204449049	-2.627065218	0.572999928	-0.607601962	0.204449049	1.794598211	5.63996E-09
AT3G49710	AT3G49710	-0.232753938	0.741283947	0.956588057	0.014220923	0.567423923	0.476287333	-0.090068202	0.975107394	0.567423923	0.476287333	1.779837187	1.69778E-18
AT2G36220	AT2G36220	-0.547738863	0.478790061	-0.491930678	0.291408307	-0.146600209	0.407137953	0.585792562	0.816446062	-0.146600209	0.407137953	1.770986957	3.10341E-39
AT2G32487	AT2G32487	0.291259141	0.966609221	-5.217389824	0.330196236	-2.245962374	0.519356056	1.112794136	0.950367876	-2.245962374	0.519356056	1.768857946	0.037745433
AT4G13210	AT4G13210	2.806244073	0.170022755	2.590070002	0.210761407	-5.087433881	0.2572465	-2.635609818	0.438561766	-5.087433881	0.2572465	1.767931984	1.20245E-10
AT5G52050	AT5G52050	-0.187098687	0.815432276	0.785324806	0.049479366	0.233369993	0.565883349	1.200118948	0.596896718	0.233369993	0.565883349	1.766438891	1.44354E-12
AT2G21510	AT2G21510	0.373934007	0.772289491	-0.079438842	0.972781238	0.663140446	0.463735953	1.860171285	0.716289725	0.663140446	0.463735953	1.732072902	0.034820662
AT2G45080	cycp3;1	0.529273463	0.833003819	-1.814559977	0.295003947	-0.520010637	0.692368528	0.73310903	0.73148555	-0.520010637	0.692368528	1.725344871	1.85723E-12
AT5G22250	CAF1b	-0.586832022	0.26567687	-0.935745728	0.004624111	-0.359705313	0.486947481	0.826312134	0.533068231	-0.359705313	0.486947481	1.71137711	3.82986E-23
AT5G50570	SPL13A					-0.84067391	0.698391736			-0.84067391	0.698391736	1.710727295	0.026922357
AT4G28703	AT4G28703	0.468778044	0.511800983	0.577053775	0.041323736	0.90523473	0.105546462	1.251048388	0.385351075	0.90523473	0.105546462	1.708692596	1.55218E-12
AT5G61600	ERF104	-0.152908427	0.818905931	-0.798181413	1.86084E-05	-0.415498456	0.138634232	1.250338225	0.272375515	-0.415498456	0.138634232	1.693410192	4.91957E-34
AT3G60890	ZPR2	0.378223553	0.940810605	-0.698344167	0.894142914	0.930881583	0.805395879	0.847087058	0.957634699	0.930881583	0.805395879	1.682467006	0.006359791
AT5G23480	AT5G23480	-0.96354613	0.498242178	-0.390525011	0.87594693	-2.062958742	0.581756635	-0.069853581	0.993687851	-2.062958742	0.581756635	1.673444439	0.004402627
AT1G43040	AT1G43040	6.509486227	0.214695548	-4.292327797	NA	-2.884900441	0.401865391	0.690245371	0.968021479	-2.884900441	0.401865391	1.668393557	0.005509275
AT2G44080	ARL	0.724744888	0.062709394	0.621219035	0.036249793	0.292917028	0.280627671	0.906296416	0.160653628	0.292917028	0.280627671	1.664515107	1.80411E-22
AT1G32920	AT1G32920	-0.455706696	0.390062813	-0.900138676	1.62142E-07	-0.785427756	0.00024183	1.458463528	0.195538287	-0.785427756	0.00024183	1.664052522	5.84328E-22
AT2G41120	AT2G41120	-1.294669128	0.360325624	0.648482163	0.512403677	-1.256038666	0.61652672	0.752939044	0.781398199	-1.256038666	0.61652672	1.653914119	0.000606064
AT5G10890	AT5G10890	-1.654941319	0.445763312	-1.834041332	0.104615608	-0.127906264	0.924207089	1.257602944	0.822563633	-0.127906264	0.924207089	1.645929647	0.001082388
AT3G27540	AT3G27540	-0.911338372	0.279482723	-1.312655776	0.083799997	-0.47400692	0.706933465	-0.205807451	0.958179519	-0.47400692	0.706933465	1.642756148	2.60584E-12
AT4G31800	WRKY18	-0.129586188	0.84899775	-0.090188894	0.82973778	0.329069549	0.377850142	-0.758623929	0.561007867	0.329069549	0.377850142	1.638394082	4.72042E-18
AT3G15210	ERF4	0.192246767	0.760506001	0.457474309	0.059298277	-0.610934725	0.004177569	1.233311996	0.430181703	-0.610934725	0.004177569	1.628257782	3.0892E-45
AT1G03010	AT1G03010	3.57241851	0.195486614	1.243137017	0.459892184	-0.647538087	0.857529032	0.352984053	0.963590756	-0.647538087	0.857529032	1.625621201	7.56941E-05
AT5G59550	RDUF2	-0.790004243	0.078770682	-0.630265702	0.005229531	-0.80431637	0.010226948	0.656018522	0.50075057	-0.80431637	0.010226948	1.622618862	2.4684E-43
AT1G77640	AT1G77640	-0.744525972	0.295589568	-2.537570049	NA	-0.515921896	0.588004179	0.64867792	0.640445007	-0.515921896	0.588004179	1.613487227	2.07805E-13
AT4G22520	AT4G22520					-1.101317598	0.62087674			-1.101317598	0.62087674	1.601671757	6.07544E-10
AT3G05932	AT3G05932	1.927187184	NA			1.123642276	0.24345825			1.123642276	0.24345825	1.600040426	0.02659105
AT2G05810	AT2G05810	1.001778823	0.890995974	1.24474156	NA	-5.17328131	0.331274236	-2.607242633	NA	-5.17328131	0.331274236	1.59488024	0.034590358
AT1G14430	AT1G14430	-0.635137788	0.607989768	-2.324180412	0.105044015	-0.952097332	0.146625702	-1.454181193	0.70850021	-0.952097332	0.146625702	1.585440501	2.0204E-09
AT2G30040	MAPKKK14	-0.291300777	0.65364245	0.721057055	0.049252356	0.352732948	0.393589856	-0.476018903	0.552449115	0.352732948	0.393589856	1.583296912	1.10811E-23
AT3G03190	GSTF11					0.396232749	NA			0.396232749	NA	1.569156519	0.008183243
AT2G41010	CAMBP25	-0.678453151	0.350348201	-0.082915699	0.803215464	-0.887783817	0.027821383	0.257162841	0.828875452	-0.887783817	0.027821383	1.546666747	4.43383E-25
AT3G18960	AT3G18960	0.537467302	0.234231814	0.534851862	0.184507458	0.432478629	0.353491414	0.345024303	0.740082034	0.432478629	0.353491414	1.546444979	5.87722E-18
AT1G53080	AT1G53080	-2.618803319	0.161099998	-1.435942777	0.523327021	-0.367881016	0.842677102	-1.266304437	0.784469624	-0.367881016	0.842677102	1.542170404	0.005817015
AT2G32770	PAP13	-2.854242208	0.09582349	-1.740959244	0.52200125	-1.661768176	0.335567787	1.646095514	0.697179773	-1.661768176	0.335567787	1.523357442	0.020656518
AT1G32640	MYC2					-0.3121114	0.321270089			-0.3121114	0.321270089	1.522391588	1.90642E-25
AT4G23190	CRK11	-0.833419415	0.067808295	0.390571848	0.376969258	-0.676812496	0.208516962	-0.235776171	0.817660485	-0.676812496	0.208516962	1.519950531	1.28784E-13
AT2G17040	NAC036	-0.026889221	0.985952479	0.57580598	0.550670797	-0.202795139	0.833610546	0.402012641	0.885975979	-0.202795139	0.833610546	1.516248037	0.000163218
AT3G56880	AT3G56880	-0.278591906	0.595085726	-0.076337843	0.791678736	-0.34655312	0.128443407	0.996714248	0.47702471	-0.34655312	0.128443407	1.507599965	1.29768E-25
AT4G21590	ENDO3	3.061984804	NA	0.400459711	0.951433959	-4.056423485	0.458088827	-0.626636845	0.917296618	-4.056423485	0.458088827	1.500065345	8.63561E-07
AT1G24140	AT1G24140	-0.399424423	0.862240293	0.92893173	0.680857983	-0.556438921	0.832455422	-2.392214874	0.180725221	-0.556438921	0.832455422	1.499153231	0.023571914
AT4G00870	AT4G00870	1.483112855	0.797138292	3.589059813	0.50922175	1.86751587	0.714646433	0.261138671	0.972495118	1.86751587	0.714646433	1.47896245	1.40009E-05
AT4G04260	AT4G04260	-1.106311625	0.879443097	-4.13846489	NA	-0.258849677	0.771490282	-5.268333145	0.653363031	-0.258849677	0.771490282	1.473443163	0.02813005
AT2G31150	AT2G31150	0.078339694	0.890630217	-0.446547525	0.184413145	0.402235444	0.107079031	0.769790212	0.548879103	0.402235444	0.107079031	1.471024426	4.99204E-29
AT2G12465	LCR50					-1.856471523	0.276128233			-1.856471523	0.276128233	1.470128823	0.00162928
AT2G39650	AT2G39650	0.146262137	0.859267284	-0.244925635	0.663345939	-0.575307263	0.233112499	-0.081659447	0.963787534	-0.575307263	0.233112499	1.468758325	1.34054E-12
AT3G19710	BCAT4	-1.860197172	NA	-1.330472314	0.086487631	-1.158522649	0.538634461	0.6406429	0.850602242	-1.158522649	0.538634461	1.463117129	2.14212E-12
AT2G26190	AT2G26190	-0.799631272	0.09961765	-0.477516392	0.018239861	-0.368652268	0.259091295	0.513080727	0.704225272	-0.368652268	0.259091295	1.456267832	2.06881E-25

AT5G52750	AT5G52750	-0.04257805	0.954911771	0.002827389	0.998169029	0.860277681	0.189418221	1.234838156	0.632278424	0.860277681	0.189418221	1.454584543	2.78791E-09
AT5G26030	FC1	-0.53898137	0.214695548	-0.503498295	0.087209151	-0.830075918	0.014963935	-0.351265026	0.684527973	-0.830075918	0.014963935	1.443854703	3.40015E-33
AT1G71050	HIPP20	1.778047624	0.365392154	1.428260832	0.16441612	0.270691476	0.812354977	0.974431673	0.88065104	0.270691476	0.812354977	1.439033612	0.005791144
AT3G13175	AT3G13175	0.997460107	0.525847931	-1.354921118	0.41783681	0.306418198	0.755105263	0.13812532	0.972077881	0.306418198	0.755105263	1.419665776	7.41898E-16
AT2G40140	CZF1	0.109939768	0.851568785	-0.458160785	0.09066087	-0.449262308	0.04409622	0.352081583	0.810393493	-0.449262308	0.04409622	1.417508649	1.48603E-32
AT2G30420	ETC2	0.126129663	0.948306808	-1.421473896	0.301878813	-0.324525541	0.703725914	0.796877298	0.935083701	-0.324525541	0.703725914	1.41602273	0.017733579
AT1G22260	ZYP1a	-1.942776858	0.517008616	-2.056788043	0.476490104	-2.741906637	0.176288453	2.696773712	0.665673432	-2.741906637	0.176288453	1.413097044	0.043470912
AT1G27770	ACA1	-0.879708969	0.050831935	-0.125855688	0.660584007	-0.546509186	0.06601668	-0.516886212	0.459923668	-0.546509186	0.06601668	1.410976917	2.36309E-29
AT5G03310	AT5G03310					0.669861945	0.350846098			0.669861945	0.350846098	1.408175359	0.014129709
AT5G24590	TIP	-0.316889273	0.5739017	-0.218999877	0.547790864	0.174199288	0.55581616	0.652298259	0.646729152	0.174199288	0.55581616	1.398477089	3.70236E-27
AT1G21326	AT1G21326	-0.011925555	0.989532784	-1.11465308	0.164520368	-1.96634753	0.125947001	2.222393557	0.477936046	-1.96634753	0.125947001	1.397818403	0.027403469
AT4G19050	AT4G19050	2.751997257	0.408314523	0.457722859	0.944677939	0.233036649	0.908736982	-0.591362642	0.925849553	0.233036649	0.908736982	1.395443177	6.01435E-06
AT5G39580	AT5G39580	-0.496757945	0.431141806	0.197934579	0.918809825	0.014674794	0.983639587	1.00242115	0.220281975	0.014674794	0.983639587	1.394142301	1.12556E-13
AT4G10270	AT4G10270					0.813810329	0.73067473			0.813810329	0.73067473	1.393185607	0.002713224
AT1G59740	AT1G59740	0.768307065	0.195824653	0.994936859	0.004047089	-0.018577069	0.979535236	-1.217510484	0.532231419	-0.018577069	0.979535236	1.390866731	0.001973675
AT3G49160	AT3G49160	-0.258636212	0.683999397	-0.508488081	0.197307939	0.651866533	0.002567337	1.064777862	0.057938647	0.651866533	0.002567337	1.389647923	2.27559E-19
AT4G03205	hemf2	-0.418619158	0.340802173	-0.614701719	0.030788621	-0.20267527	0.322406971	0.736154892	0.420106526	-0.20267527	0.322406971	1.388915333	6.39098E-16
AT1G47990	GA2OX4	-0.946162131	0.218240473	-0.800732016	0.268039629	-1.259616873	0.127213221	1.574345103	0.460519108	-1.259616873	0.127213221	1.382885119	6.6882E-05
AT3G45640	MPK3	-1.002414982	0.051712702	-0.90981934	1.67067E-05	-0.884408851	1.29067E-07	0.484426972	0.7777051	-0.884408851	1.29067E-07	1.379399472	8.81078E-28
AT5G14200	IMD1	-2.197658513	0.174559724	-1.015094537	0.417203286	-1.712496124	0.360366356	0.423083538	0.859442872	-1.712496124	0.360366356	1.379269788	1.9683E-20
AT1G60270	BGLU6	-1.759523697	0.115377331	-0.940900977	0.439842686	-0.76318659	0.199813538	-1.184914547	0.792048637	-0.76318659	0.199813538	1.368982372	1.96134E-05
AT3G52525	OFP6	1.110057945	0.313995886	-0.069728754	0.957278713	-0.677885509	0.414077955	0.825923367	0.830747236	-0.677885509	0.414077955	1.368712444	0.005722889
AT5G58390	AT5G58390	-1.879207589	0.544670342	-1.071428466	0.629208388	-0.888760742	0.658138759	0.052060896	0.991399664	-0.888760742	0.658138759	1.361180721	5.13005E-09
AT3G53530	NAKR3	0.271181336	0.836208955	-0.048309704	0.958077566	0.369649052	0.345249457	0.965329607	0.706790319	0.369649052	0.345249457	1.359090725	9.53892E-13
AT4G12870	AT4G12870	0.585223649	0.765721518	-1.301342984	0.50695547	-2.572779053	0.277514687	0.036415333	0.992399529	-2.572779053	0.277514687	1.355280868	1.02846E-10
AT4G17500	ERF-1	0.103020362	0.883986195	0.20991298	0.26795201	-0.691416897	0.000461303	1.020634987	0.518492932	-0.691416897	0.000461303	1.353104787	7.14877E-17
AT5G45540	AT5G45540	-0.065119679	0.990985016	-0.003317552	0.998169029	0.152073958	0.906559769	-0.890572424	0.87862916	0.152073958	0.906559769	1.352049264	0.000487665
AT3G49530	NAC062	-0.32274086	0.545097731	-0.41396297	0.171956701	-0.188755885	0.628429997	-0.028307586	0.98831332	-0.188755885	0.628429997	1.344914458	1.89861E-32
AT1G33475	AT1G33475	-0.26381456	0.750979839	-0.605796623	0.382152221	-0.320669921	0.457027947	0.690639154	0.604422229	-0.320669921	0.457027947	1.328122853	0.000129104
AT5G45520	AT5G45520	-0.564769717	0.909518502	1.063711671	NA	-1.083271381	0.468336511	3.648826923	0.619354152	-1.083271381	0.468336511	1.326101468	0.04330258
AT5G04690	AT5G04690	-0.482895372	0.909130329	1.681570911	0.582587062	1.98494812	0.051100054	-0.989183894	0.850582824	1.98494812	0.051100054	1.325628888	0.006301074
AT3G63480	AT3G63480	-1.343705117	0.262171958	-1.764676616	0.46636953	-0.17348384	0.870623783	-0.262357826	0.901814827	-0.17348384	0.870623783	1.316926685	9.25268E-06
AT2G21220	AT2G21220	-0.151249864	NA	-0.916488264	0.699353206	-1.987670183	0.115855604	-0.192878781	0.947241707	-1.987670183	0.115855604	1.313021217	0.000273907
AT2G31790	AT2G31790	-0.963057156	0.012280469	-0.068010174	0.927855266	-0.951241411	0.001405397	1.318244476	0.230088373	-0.951241411	0.001405397	1.310192199	2.91619E-09
AT3G51895	SULTR3;1	-0.516191708	0.575615345	-1.183269095	0.079045499	-0.150953312	0.793099398	-0.769509663	0.618907586	-0.150953312	0.793099398	1.29241134	2.70897E-20
AT3G08000	AT3G08000	1.403966158	0.516866311	-0.164379698	0.910195579	-0.765471291	0.309678122	0.828422291	0.696374342	-0.765471291	0.309678122	1.292125658	0.000538401
AT1G05835	AT1G05835			1.419081486	0.822763627	-0.731947382	0.248552057			-0.731947382	0.248552057	1.280502026	7.95501E-14
AT5G08790	ATAF2	0.373846952	0.540107375	0.786450569	0.00428307	0.835017664	0.0003541	0.519917813	0.691824202	0.835017664	0.0003541	1.276701196	3.13126E-26
AT5G59330	AT5G59330	-1.632896058	0.615837196	-6.225475596	0.236812567	4.998274791	0.350257105	0.120477516	0.992379592	4.998274791	0.350257105	1.276495853	0.000265267
AT4G02200	AT4G02200	-0.988137851	0.437183069	-0.77163107	0.547387809	-0.780625152	0.193127686	-1.910890517	0.664075844	-0.780625152	0.193127686	1.276098706	2.0778E-07
AT3G12890	ASML2	1.116537842	0.651888397	-1.814209748	0.749542511	2.237686599	0.100626361	1.761808049	0.616300683	2.237686599	0.100626361	1.271593904	0.036945335
AT2G16060	HB1	0.485771161	0.725317004	1.717362821	0.30896129	0.353421992	0.598672743	-0.247389505	0.924076399	0.353421992	0.598672743	1.271030549	4.07476E-07
AT3G53130	LUT1	-0.768171176	0.022942168	-0.895198711	0.02560979	-0.815624802	0.001044836	0.790180883	0.228792905	-0.815624802	0.001044836	1.268107546	2.16234E-16
AT3G22120	CWLP					-0.976544908	4.51862E-05	-4.709284728	0.680482607	-0.976544908	4.51862E-05	1.260734354	8.62341E-11
AT2G30370	CHAL	-0.421263709	0.834925132	-1.248037703	0.263254523	-0.18040339	0.831985973	-0.426749276	0.809300038	-0.18040339	0.831985973	1.260538588	2.0583E-07
AT5G36000	AT5G36000	-2.001868887	0.0746637	0.542855909	0.70351996	-0.52916559	0.510150325	0.054401212	0.991559366	-0.52916559	0.510150325	1.258706523	0.017388396
AT1G55050	AT1G55050	-1.515721254	0.179067149	-3.279185023	0.15391511	-1.319095769	0.231896165	-0.281595526	0.93403749	-1.319095769	0.231896165	1.254797105	0.00973342
AT3G47480	AT3G47480	-0.973817681	0.785070894	0.927915456	0.590948376	-1.275720526	0.230021511	-0.97401688	0.859138601	-1.275720526	0.230021511	1.250138311	0.034993401
AT5G64870	AT5G64870	1.000522264	0.078775141	-0.556839863	0.205156546	-1.25964354	0.084906272	0.834120517	0.269029707	-1.25964354	0.084906272	1.246874667	9.58877E-10
AT2G38870	AT2G38870	-0.422329534	0.61697383	0.014914798	0.986057919	-1.702222055	0.491138117	-0.610245186	0.653363031	-1.702222055	0.491138117	1.239817847	0.00023387
AT3G03030	AT3G03030	-0.34919635	0.646812045	-0.264513028	0.553511356	-0.254055405	0.520348478	-0.256308236	0.912949458	-0.254055405	0.520348478	1.237569558	5.26056E-05
AT5G20635	AGG3	0.196443326	0.893152452	-0.065643695	0.951173551	0.194530152	0.793099398	-0.329779026	0.898144093	0.194530152	0.793099398	1.235688406	2.19489E-09
AT1G50740	AT1G50740	-0.637769041	0.339349237	0.100445921	0.812899276	-0.100013171	0.775632982	-0.343327854	0.745973303	-0.100013171	0.775632982	1.232227249	1.03492E-15
AT3G27650	LBD25	1.487542339	0.18059861	1.230900939	0.665671364			0.455037529	0.875648986			1.227910237	0.000317287
AT3G26230	CYP71B24	1.18077957	0.24625377	0.78134314	0.255130425	0.130698515	0.926272904	1.816677123	0.417690799	0.130698515	0.926272904	1.226852606	0.041258975
AT1G62975	AT1G62975	-0.109169368	0.90534663	0.356585613	0.667239715	0.146140658	0.780789515	0.704911986	0.574062337	0.146140658	0.780789515	1.221105441	3.57206E-05
AT1G01130	AT1G01130	0.624229203	0.397027453	0.235836987	0.807612868	-0.023871196	0.9743903	0.139857846	0.947537872	-0.023871196	0.9743903	1.220066392	0.001909627
AT4G29360	AT4G29360	-0.737808326	0.145663222	-0.949125887	0.018521844	-0.385834033	0.23665355	0.412646643	0.783038559	-0.385834033	0.23665355	1.215041245	2.07846E-09
AT3G02020	AK3	-1.617620693	0.612982118	-1.521163515	0.054350217	-1.333357292	0.625101718	0.371361255	0.854730188	-1.333357292	0.625101718	1.213829721	0.000145573
AT5G07150	AT5G07150					-0.257473563	0.780572939			-0.257473563	0.780572939	1.212559093	0.000153256
AT2G32860	BGLU33	0.083319169	0.905380006	1.110082113	0.08704919	0.271371319	0.645481965	1.086419742	0.282858748	0.271371319	0.645481965	1.212267167	3.17177E-11
AT2G17830	AT2G17830	0.023362179	0.976992404	0.864918468	0.051223997	0.185277095	0.862486811	0.710853736	0.625364787	0.185277095	0.862486811	1.21193324	1.14775E-08
AT1G27100	AT1G27100	-0.938990505	0.029274503	-0.797137072	1.57183E-07	-0.685361408	0.000102541	0.754880504	0.640704059	-0.685361408	0.000102541	1.210166953	2.70752E-21
AT3G51080	GATA6	-0.802643147	0.240506471	-0.203766619	0.700271184	0.764248778	0.01131058	0.825206505	0.224145565	0.764248778	0.01131058	1.209091457	2.08421E-07
AT4G39900	AT4G39900	0.18332023	0.598198344	-0.179087255	0.580144375	-0.03621117	0.905613517	0.671221065	0.021051627	-0.03621117	0.905613517	1.208198709	8.89214E-22
AT5G42530	AT5G42530	-1.211200584	0.152822773	1.426160021	0.052148516	0.006570929	NA	-0.252083787	0.958708047	0.006570929	NA	1.205332621	0.005695548
AT1G03270	AT1G03270	-0.65781884	0.618791076	-0.104728238	0.942332968	-1.634398553	0.227428637	0.780963951	0.691895802	-1.634398553	0.227428637	1.197757464	0.000209075
AT5G60880	BASL	-0.30486955	0.910125225	-1.294131421	0.519799784	0.283372965	0.760770085	0.992596871	0.542510701	0.283372965	0.760770085	1.194087345	0.007467607
AT4G39410	WRKY13	-0.398167607	0.793215853	-1.856439385	0.319588645	0.998284008	0.21974559	1.098882831	0.567190016	0.998284008	0.21974559	1.18812444	0.029549654
AT2G45050	GATA2	-0.215005474	0.780170921	-0.80762533	0.217240628	-0.814994846	0.022349751	-0.427547995	0.837258505	-0.814994846	0.022349751	1.184967815	2.88767E-18
AT2G40000	HSPRO2	0.301568567	0.633439831	0.779788493	1.57057E-05	0.227848183	0.315467085	1.694825354	0.246894084	0.227848183	0.315467085	1.183489117	8.05925E-17
AT4G34790	AT4G34790	-1.826942189	0.331609471	-1.647580023	0.313601797	-1.338932648	0.08417355	0.181124468	0.966434558	-1.338932648	0.08417355	1.181461938	1.80327E-07
AT3G16510	AT3G16510	0.09378937	0.926325013	0.199353001	0.679680832	-0.453042014	0.154987562	0.731134108	0.722619004	-0.453042014	0.154987562	1.181146546	0.00018245
AT3G44720	ADT4	-0.709019878	0.081936145	-0.065327836	0.894142914	-0.250961534	0.439331113	-0.225824649	0.899858481	-0.250961534	0.439331113	1.178199234	3.0619E-09
AT4G24540	AGL24	1.108768214	0.37064493	0.279300448	0.854186879	0.396135283	0.541357685	0.351782594	0.87813535	0.396135283	0.541357685	1.176843044	1.67187E-13
AT5G13140	AT5G13140	-0.878717309	0.00424955	-0.794717027	0.004766428	-0.754646843	3.87077E-06	-0.021007195	0.986012712	-0.754646843	3.87077E-06	1.174619533	6.10601E-11
AT3G06640	AT3G06640	-0.20097255	0.952422369	-0.714373624	0.770937752	-0.041763431	0.972708394	1.895572705	0.409180759	-0.041763431	0.972708394	1.171884637	0.034654532
AT1G68585	AT1G68585	0.742507767	0.524695566	0.041108514	0.975124251	0.659506643	0.236752322	0.910717801	0.563758673	0.659506643	0.236752322	1.159393671	0.009153122
AT2G14080	AT2G14080	-0.128251862	0.834507102	0.194246942	0.436715192	0.442018057	0.041919083	1.310031278	0.394836051	0.442018057	0.041919083	1.155333256	8.95745E-16
AT1G10640	AT1G10640	-1.124857816	0.407043574	-2.345297908	0.114676068	0.10902979	0.876208256	-0.018002985	0.995194798	0.10902979	0.876208256	1.151878171	3.02585E-13
AT4G29880	PIRL7	0.582643129	0.742069256	1.016255716	0.334568445	1.778545242	0.443177919	0.992949613	0.763885599	1.778545242	0.443177919	1.151464408	0.045846229
AT1G74490	AT1G74490	-1.908722709	0.238370431	-1.283090387	0.508023536	-1.422121277	0.354368844	-1.269532691	0.751308246	-1.422121277	0.354368844	1.149485576	3.54501E-06
AT1G24020	MLP423	-0.850415463	0.786620854	-1.524034079	0.115525868	-0.38480206	0.507129608	0.089133723	0.982734229	-0.38480206	0.507129608	1.147037032	1.6849E-11
AT2G46720	KCS13	0.349289779	0.634254916	0.009701913	0.986057919	-0.339660183	0.394263238	1.10893547	0.498296358	-0.339660183	0.394263238	1.14695608	9.51733E-11
AT1G80760	NIP6;1	0.026591845	0.983079283	0.602343495	0.437617896	-0.177828196	0.82701	0.708198127	0.601921549	-0.177828196	0.82701	1.145571848	8.35386E-09
AT5G52100	CRR1	-0.715202887	0.329257494	-0.057244522	0.922789871	0.124130999	0.623915738	1.965861477	0.327697498	0.124130999	0.623915738	1.145547917	4.79191E-07
AT2G37260	TTG2	-1.117618579	0.435639513	-0.776282577	0.262655337	-1.106709201	0.537483719	1.20525168	0.78630406	-1.106709201	0.537483719	1.142033093	0.002433344
AT5G25490	AT5G25490	-0.018202713	0.988976522	0.372672703	0.784747957	0.38489238	0.536163465	-0.261462304	0.93556951	0.38489238	0.536163465	1.141271692	1.79633E-08
AT1G37150	HCS2	0.053542073	0.972983219	0.305913968	0.848489082	-0.1112512	0.947624151	0.319865136	0.939540455	-0.1112512	0.947624151	1.13374902	0.017440127
AT1G70760	NdhL					-0.928170523	1.03067E-09			-0.928170523	1.03067E-09	1.130648546	0.010819192
AT5G42700	AT5G42700	-1.042643807	0.63651753	-1.567523456	0.468479533	-2.00930235	0.414273168	0.010065461	0.9987964	-2.00930235	0.414273168	1.128691122	0.049090978
AT1G19020	AT1G19020	-0.024404948	0.978652988	-0.281887912	0.453717488	0.344166065	0.552253572	0.935135115	0.627303788	0.344166065	0.552253572	1.121556106	0.004054884
AT5G23020	IMS2	0.51540491	0.896020233	0.188095121	0.942966265	1.118839601	NA	0.035842742	0.993804788	1.118839601	NA	1.119370867	4.40368E-21
AT1G48660	AT1G48660	-1.503352802	0.700914645	-5.818609799	0.271991558	0.761035497	0.829959753	-0.725076375	0.932561267	0.761035497	0.829959753	1.118789429	0.01085748

AT5G24490	AT5G24490	-0.786172836	0.023501837	-0.518016724	0.015499102	-0.426258795	0.034818072	1.939024058	0.067229778	-0.426258795	0.034818072	1.117428592	3.13689E-24
AT3G28750	AT3G28750	1.127452422	0.427124873	0.911101903	0.740361217	0.509812839	0.648429144	0.32118749	0.930136802	0.509812839	0.648429144	1.115931905	0.0171594
AT4G29670	ACHT2	-0.738297774	0.053540786	-0.602967984	0.017048787	-0.440037746	0.200095443	0.276264593	0.804495913	-0.440037746	0.200095443	1.114172442	1.6555E-19
AT5G39670	AT5G39670	1.099154811	0.71196926	0.732907959	0.896238592	-0.807009389	0.396338252	2.325641483	0.616300683	-0.807009389	0.396338252	1.113882294	0.000171402
AT5G35580	AT5G35580	1.118173349	0.409867979	0.469655847	0.668904414	-0.679235594	0.456392703	-1.036813698	0.632278424	-0.679235594	0.456392703	1.113177889	4.29139E-06
AT4G22753	None					-0.407772969	0.48228094			-0.407772969	0.48228094	1.11284407	1.69584E-13
AT4G31060	AT4G31060	-0.798135358	0.158594539	-0.714134408	0.035053475	-0.596006072	0.078269229	1.078709871	0.100032418	-0.596006072	0.078269229	1.110971948	1.7047E-07
AT5G08260	scpl35	-0.020960881	0.98796255	-0.591281499	0.447063528	0.795410453	0.047181661	-0.250609944	0.930136802	0.795410453	0.047181661	1.110256332	1.99286E-14
AT3G18070	BGLU43	0.383441444	0.836700747	-2.795283102	0.065094226	-1.115462214	0.201780898	-0.608033307	0.916448617	-1.115462214	0.201780898	1.105602132	0.001454591
AT2G42360	ATL41					-0.588467234	0.452572599			-0.588467234	0.452572599	1.098040366	0.010839747
AT4G31805	AT4G31805	-0.947403805	0.227027015	-0.855644138	0.290507004	-0.573523991	0.549686602	0.359453549	0.820406788	-0.573523991	0.549686602	1.096320698	5.5096E-06
AT1G09070	SRC2	-0.202712676	0.745557522	0.060014427	0.751015865	-0.170517719	0.462019963	1.292605421	0.430181703	-0.170517719	0.462019963	1.093774751	1.11177E-24
AT4G39363	AT4G39363	0.451215084	0.923456684	2.068087626	0.424429062	2.004630058	0.081864669	-0.351407502	0.964000833	2.004630058	0.081864669	1.093274011	0.022854657
AT5G64060	NAC103	-0.344296954	0.830467273	1.699311498	0.205416656	-2.208609352	0.350257105	0.879110906	0.697407195	-2.208609352	0.350257105	1.091591785	0.006296504
AT2G47920	NET3C	-0.873368754	0.392331993	0.125428702	0.928290454	0.47639651	0.468268694	1.120427804	0.477936046	0.47639651	0.468268694	1.091231339	0.012644087
AT3G58780	SHP1	0.141910574	0.96827212	-2.195485982	0.374138705	-1.478936947	0.568156383	-1.134287253	0.647947756	-1.478936947	0.568156383	1.089041177	0.009921654
AT4G21310	AT4G21310	0.754286224	0.891262936	0.090107762	0.980898503	0.387804093	0.675770575	0.168188359	0.982529985	0.387804093	0.675770575	1.087669644	0.000146112
AT5G48485	DIR1					0.299492453	0.293679918			0.299492453	0.293679918	1.085322706	2.25232E-06
AT1G21440	AT1G21440	-0.711939583	0.183724914	-0.623429309	0.070774761	0.136044513	0.640211764	0.44080636	0.721140128	0.136044513	0.640211764	1.083529703	5.12915E-17
AT5G14910	AT5G14910	-0.935849504	0.000721129	-0.614664665	0.001270645	-0.81956111	4.78098E-13	0.739845006	0.34017207	-0.81956111	4.78098E-13	1.083049091	4.09665E-16
AT3G58990	IPMI1	-1.661130642	0.149787079	-0.581240935	0.412926512	-0.878135494	0.775046633	0.396236046	0.893481012	-0.878135494	0.775046633	1.080476126	1.20423E-08
AT4G01540	NTM1	-0.392482419	0.75130762	-0.639030386	0.563492165	-0.647989745	0.380980177	-0.458297699	0.896927975	-0.647989745	0.380980177	1.071291343	0.000157692
AT5G47070	AT5G47070	-0.191014983	0.725672756	0.140319261	0.751680233	-0.520394192	0.149487274	-0.046130393	0.972614304	-0.520394192	0.149487274	1.071190896	4.52944E-15
AT3G55710	AT3G55710	-0.860842034	0.458891227	-1.233400764	0.063000639	-0.39987927	0.674433286	-0.630426408	0.935551609	-0.39987927	0.674433286	1.069712018	0.013535354
AT3G49920	VDAC5	-0.370916745	0.746389665	-0.126473605	0.870815495	0.063808144	0.930970769	0.021907401	0.994477124	0.063808144	0.930970769	1.066212808	1.18595E-05
AT1G49910	BUB3.2	0.235477573	0.968928688	-2.271800868	0.344097683	-0.219375029	0.917502996	-1.317019168	0.738198292	-0.219375029	0.917502996	1.065221045	0.000376853
AT1G17600	AT1G17600	1.13312393	0.67790539	-1.576212203	0.414117625	-0.413004797	0.630546208	-3.147252056	0.637815859	-0.413004797	0.630546208	1.064040915	0.002268177
AT1G05370	AT1G05370	-0.174874429	0.892309516	0.079044009	0.930084995	-0.698189089	0.337939265	0.143860066	0.976635648	-0.698189089	0.337939265	1.061793409	0.00021291
AT3G23760	AT3G23760	0.510163187	0.357499685	0.113081766	0.681693887	0.239838367	0.295367991	0.853460853	0.324288106	0.239838367	0.295367991	1.060427825	7.241E-06
AT1G14750	SDS	-0.794718784	0.771096517	-3.37390889	0.220207776	-1.29413638	0.517639823	-0.038478617	0.993609003	-1.29413638	0.517639823	1.056278499	0.044322429
AT5G64570	XYL4	-0.448094084	0.3946851	-1.08158267	0.194894478	0.287277159	0.301080723	-0.043542733	0.973864665	0.287277159	0.301080723	1.056056961	9.93058E-14
AT1G58848	AT1G58848	-0.199224744	0.885622873	0.381635898	0.821496623	0.100588895	0.876371208	-0.078071316	0.99341989	0.100588895	0.876371208	1.055378109	0.005047986
AT5G67260	CYCD3;2	0.119625239	0.841765087	-0.6105342	0.007765427	0.530541554	0.000469913	0.35551835	0.695263933	0.530541554	0.000469913	1.050348935	8.52663E-15
AT5G56810	AT5G56810	-0.973831954	0.251069782	-0.749739345	0.323547954	0.723158438	0.259091295	-0.263661359	0.948303914	0.723158438	0.259091295	1.049900146	0.015052613
AT3G11820	SYP121	-0.736753056	0.101684326	-0.45905219	0.147747317	-0.507608078	0.004660331	-0.022644211	0.990335664	-0.507608078	0.004660331	1.045047724	1.19746E-10
AT2G41180	SIB2					-0.665619491	0.32226611			-0.665619491	0.32226611	1.039707117	0.00422569
AT2G34170	AT2G34170	-0.457947416	0.287049365	-0.298447426	0.29641194	-0.437855604	0.04308133	0.949294466	0.606948021	-0.437855604	0.04308133	1.039349963	2.08899E-12
AT4G33920	AT4G33920	-0.514039678	0.263502982	-0.234499403	0.309385838	-0.332159548	0.361710588	0.596864047	0.6903809	-0.332159548	0.361710588	1.03648318	1.08681E-13
AT2G01918	PQL3	-0.90526055	0.410758102	-0.25947121	0.623024549	0.087571291	0.797900256	0.57401301	0.840351964	0.087571291	0.797900256	1.033012481	0.001946096
AT3G48900	AT3G48900	0.052143534	0.979785582	-0.562230599	0.520612585	0.69443886	0.398486503	0.676423955	0.690509114	0.69443886	0.398486503	1.032391005	0.001476995
AT4G30680	AT4G30680	-0.625230811	0.192797362	-0.679666843	0.24864545	0.329186186	0.395806482	0.88972623	0.106990328	0.329186186	0.395806482	1.03185152	1.17027E-06
AT4G01460	AT4G01460	0.113679354	0.830690134	0.225261299	0.572935969	0.276452904	0.357528439	0.73413378	0.610291087	0.276452904	0.357528439	1.029260045	5.79541E-10
AT4G01130	AT4G01130	-0.031721675	0.964782085	-0.176085359	0.719989184	-0.285033516	0.488805567	0.500631876	0.748924676	-0.285033516	0.488805567	1.027504728	2.30125E-07
AT1G20823	AT1G20823	0.137432467	0.88337212	0.654310635	0.286928078	-0.910410194	0.024706587	-0.074149445	0.97399901	-0.910410194	0.024706587	1.02699418	9.49412E-09
AT1G23090	AST91	-0.171467811	0.72090902	0.156810586	0.741096982	0.413808948	0.046671902	0.387593629	0.66524984	0.413808948	0.046671902	1.022952575	3.4791E-13
AT4G13880	RLP48	0.384798196	0.71331789	0.947671386	0.211117992	0.83202581	0.02890327	0.46566672	0.788737401	0.83202581	0.02890327	1.022283479	9.47028E-06
AT1G79840	GL2	-0.612351805	0.488896665	-0.177153133	0.776873173	0.213418429	0.83062768	-0.069021726	0.976635648	0.213418429	0.83062768	1.022242151	0.000449601
AT3G05160	AT3G05160	-0.017019475	0.979728666	0.092483426	0.778687509	0.143161317	0.574260279	1.095977969	0.582068518	0.143161317	0.574260279	1.020591948	2.90964E-06
AT2G32870	AT2G32870	-0.772525476	0.29143384	-0.64098574	0.195813337	0.302838928	0.174245583	1.193159174	0.317592245	0.302838928	0.174245583	1.017924874	2.92272E-08
AT3G63088	RTFL14	1.039866574	0.701552003	0.760811517	0.828245094	-0.103599497	0.970006823	-0.379940255	0.964169173	-0.103599497	0.970006823	1.017424884	0.012282841
AT4G24390	AFB4	-0.709913215	0.034024941	-0.330635177	0.35211144	-0.097651507	0.772022628	0.192984002	0.904735573	-0.097651507	0.772022628	1.016887211	6.14338E-08
AT2G29260	AT2G29260	0.146136169	0.942494078	1.302848834	0.484715746	-0.108757327	0.930620796	-0.361039501	0.963509602	-0.108757327	0.930620796	1.016504969	0.036640588
AT1G68940	AT1G68940					-0.515166954	0.114417397			-0.515166954	0.114417397	1.016249189	0.000142327
AT5G16030	AT5G16030	-0.571919932	0.163014913	-0.572891431	0.009322234	-0.396956858	0.0028011	1.490280014	0.281913411	-0.396956858	0.0028011	1.012753343	1.72059E-19
AT4G01050	TROL	-0.521566267	0.256592162	0.014665991	0.939782651	0.14770259	0.259734266	2.341057147	0.056077181	0.14770259	0.259734266	1.011830065	2.563E-13
AT5G09976	AT5G09976	5.929224401	0.294010131			-3.223321176	0.563907217	1.424060528	0.906077763	-3.223321176	0.563907217	1.009047303	0.002046668
AT1G72530	AT1G72530	0.126784869	0.910701106	-0.293674947	0.824021444	0.367473133	0.55822826	0.291415649	0.883127616	0.367473133	0.55822826	1.003541462	0.005275504
AT3G48920	MYB45	2.415752809	0.516345054	-3.539349989	0.535812829	2.306166341	0.445672623	-0.513376317	0.841532762	2.306166341	0.445672623	1.003230223	0.000731157
AT5G24318	AT5G24318	0.659049075	0.368754877	0.026954378	0.978494875	0.472086976	0.296684297	-0.329818777	0.855486955	0.472086976	0.296684297	-1.000407585	0.000321066
AT4G05150	AT4G05150	0.952187374	0.000464028	0.762988296	1.00085E-05	0.769054612	0.000686324	0.191875857	0.893780502	0.769054612	0.000686324	-1.001073381	2.73097E-14
AT5G57090	EIR1	1.169690673	0.460091669	-0.884496128	0.531272228	0.986729432	0.535162474	-0.682129845	0.899191689	0.986729432	0.535162474	-1.005759429	0.003667453
AT1G64380	AT1G64380	0.100203463	0.897952684	0.320866683	0.474433808	-0.159279449	0.73067473	-0.776471764	0.450576814	-0.159279449	0.73067473	-1.006713572	9.26583E-06
AT3G03450	RGL2	0.603444591	0.661288993	-2.461245345	0.173234197	0.677276225	0.390295356	-0.482168131	0.837258505	0.677276225	0.390295356	-1.006763759	0.000162163
AT1G72830	NF-YA3	0.503833597	0.513793841	-0.103998476	0.89405405	-0.253436128	0.708429935	-0.578776937	0.609674706	-0.253436128	0.708429935	-1.006826282	6.73206E-06
AT4G34950	AT4G34950	-1.234044575	0.267153121	0.217098788	0.772177181	-0.881906592	0.134962859	-1.575684747	0.242292325	-0.881906592	0.134962859	-1.009367771	0.00029386
AT3G45680	AT3G45680	-1.642864972	0.639954057	-1.692207555	0.579701023	0.033529356	0.988325398	0.144800942	0.987071939	0.033529356	0.988325398	-1.010953877	0.026850206
AT3G50220	IRX15	-1.816888557	0.467743253	-0.756505549	0.726084464	1.461484904	NA	-1.335696987	0.73461774	1.461484904	NA	-1.012628562	0.021858622
AT3G47220	PLC9	-0.417318998	0.900720417	-0.402260868	0.88300857	-0.392545048	0.842237571	-0.862758818	0.663046749	-0.392545048	0.842237571	-1.012745095	0.000942327
AT1G71040	LPR2	0.34851174	0.487665378	0.293941863	0.403041239	-0.39494411	0.039824408	-0.281069921	0.806588363	-0.39494411	0.039824408	-1.013643883	2.87814E-14
AT3G12700	NANA	-0.11601464	0.922725437	-1.197868939	0.085145767	-0.761655437	0.137376346	-1.485983292	0.427749503	-0.761655437	0.137376346	-1.014410313	9.26839E-11
AT3G45600	TET3	0.420240223	0.278012624	0.006373593	0.990431447	-0.607356697	0.011409179	-0.602908525	0.646747644	-0.607356697	0.011409179	-1.015006037	8.38496E-10
AT1G28130	GH3.17	-0.467184077	0.149629367	-0.00715605	0.984810149	-0.468959841	0.028539234	-0.322616527	0.777143749	-0.468959841	0.028539234	-1.017130572	2.09087E-11
AT5G23230	NIC2					0.566809184	0.519834439			0.566809184	0.519834439	-1.01986292	0.014858425
AT1G28570	AT1G28570	-0.329683057	0.823490042	0.470682449	0.587216419	-0.646636893	0.439833035	-0.408894225	0.749436809	-0.646636893	0.439833035	-1.021152222	1.03012E-06
AT5G59070	AT5G59070					-1.043280381	0.083699594			-1.043280381	0.083699594	-1.021421468	0.016384769
AT3G27270	AT3G27270	1.096932554	0.412286261	1.154807528	0.256950311	0.79813533	0.513142941	-0.628224916	0.912756407	0.79813533	0.513142941	-1.023728783	0.000399809
AT1G74250	AT1G74250	0.226833692	0.63136523	0.068094324	0.883760379	-0.072275932	0.836329338	-0.385401705	0.765335087	-0.072275932	0.836329338	-1.026179711	0.034733247
AT5G54145	AT5G54145	0.525137883	0.277235076	-0.054969109	0.943336663	0.507161776	0.128918595	0.037378994	0.987953474	0.507161776	0.128918595	-1.026236245	0.000676957
AT5G48920	TED7	-2.035872507	0.128909039	-2.269136844	0.222932221	-0.421770803	0.833610546	-1.08064302	0.810169925	-0.421770803	0.833610546	-1.028727927	0.002463044
AT3G47420	G3Pp1	1.38992937	0.10117898	0.669948354	0.33816757	-0.094703028	0.846491222	-0.276403147	0.865123396	-0.094703028	0.846491222	-1.031545291	4.74604E-14
AT1G15170	AT1G15170	-0.278131363	0.738409571	0.486984415	0.376464643	-0.111192259	0.833961269	0.626734697	0.653363031	-0.111192259	0.833961269	-1.031969599	0.010239739
AT1G76890	GT2	-0.505469254	0.226766955	-0.389797683	0.153422623	0.22585267	0.67997173	0.064227524	0.972495118	0.22585267	0.67997173	-1.032357167	0.033478847
AT5G14120	AT5G14120	0.068016214	0.897320913	0.132546189	0.638471032	0.285651635	0.035503271	0.997169361	0.590931998	0.285651635	0.035503271	-1.034103376	1.85539E-13
AT1G14240	AT1G14240	0.640592721	0.849820791	-2.230347556	0.506601636	-0.649055349	0.897300312	-0.039977937	0.993721829	-0.649055349	0.897300312	-1.037199793	0.021047032
AT1G01180	AT1G01180	0.370971829	0.469098323	-0.549563918	0.167121487	-0.442241221	0.107068338	0.394102616	0.735907718	-0.442241221	0.107068338	-1.03828481	7.13874E-09
AT2G16570	ASE1	1.226326592	0.333457117	0.844262632	0.57570781	-0.3003222	0.777599635	-1.148540299	0.782088381	-0.3003222	0.777599635	-1.038699909	1.16333E-06
AT2G32020	AT2G32020	-1.359016205	0.135359652	-0.681607639	0.172052889	-0.658077278	0.408145617	-1.113990003	0.823318742	-0.658077278	0.408145617	-1.039244597	0.01959554
AT5G06300	LOG7	1.104402631	0.111385221	0.450593259	0.470268798	-0.556898483	0.330984632	-0.277135055	0.916241006	-0.556898483	0.330984632	-1.041544372	0.007860948
AT5G17330	GAD	0.721298638	0.065463398	-0.432469453	0.255078317	-0.76197414	0.504126442	-0.9255631	0.592490849	-0.76197414	0.504126442	-1.041698433	8.88308E-23
AT2G37460	UMAMIT12	-0.22502627	0.862455177	0.574530198	0.749075764	0.427810867	0.689620245	-0.160781249	0.938659772	0.427810867	0.689620245	-1.042584991	3.70247E-09
AT3G20935	CYP705A28	0.86791697	0.792734374	1.895835098	0.506399919	-0.975124373	0.76526442	-1.292271606	0.795199419	-0.975124373	0.76526442	-1.047521919	0.048565995
AT4G16840	AT4G16840	0.88952942	0.35786256	-0.661987121	0.512362447	0.144531739	0.81869976	-0.296622235	0.830747236	0.144531739	0.81869976	-1.047797491	3.8017E-05
AT2G40120	AT2G40120	-0.515129364	0.529584645	-0.662786846	0.357008311	-0.799106101	0.481871169	-0.810298034	0.759702709	-0.799106101	0.481871169	-1.047861847	1.96134E-05
AT1G26820	RNS3	0.558957959	0.842067821	-0.547899542	0.747486559	1.005918521	0.392862748	-1.943659276	0.647947756	1.005918521	0.392862748	-1.048246007	0.00275618

AT5G63800	MUM2	-0.701110147	0.023063332	0.102114713	0.840814152	-0.412604194	0.137612421	-0.313101565	0.743050938	-0.412604194	0.137612421	-1.048382784	3.46262E-11
AT4G30250	AT4G30250	-0.310085833	0.919767621	-0.998040857	0.333868199	1.097179106	0.448796001	0.986639739	0.861558235	1.097179106	0.448796001	-1.04860523	0.003606873
AT3G49210	AT3G49210	-0.140105564	0.901237264	4.28352E-05	0.999960916	-0.58169463	0.25648921	-0.778921543	0.706790319	-0.58169463	0.25648921	-1.049027252	0.000136894
AT1G27620	AT1G27620	1.551087674	0.098126339	0.363491651	0.734772063	0.239167607	0.855938314	-1.188692138	0.300202167	0.239167607	0.855938314	-1.050736913	2.26307E-05
AT1G68795	CLE12	1.795339893	0.473644215	1.767465787	0.73757663	-2.985870133	0.365618237	-1.302005011	0.779600001	-2.985870133	0.365618237	-1.052188324	0.048063644
AT1G70830	MLP28	0.478296192	0.325140075	0.203986508	0.194024504	-0.075235724	0.925381643	-0.84668872	0.547026175	-0.075235724	0.925381643	-1.053459045	7.333E-10
AT1G18980	AT1G18980	0.030041728	0.996306924	6.297897059	0.231144701	2.655446589	0.527728535			2.655446589	0.527728535	-1.053600564	0.009014264
AT3G27190	UKL2	1.033713675	0.227830272	0.274082213	0.624159882	0.226755482	0.502044757	-0.334169892	0.780655515	0.226755482	0.502044757	-1.054077281	5.73486E-12
AT2G05320	AT2G05320	0.475461406	0.410322619	-0.549339376	0.580529237	-0.723935777	0.293786473	-0.158326149	0.956315558	-0.723935777	0.293786473	-1.054587944	0.001477325
AT4G03330	SYP123	-5.054400824	0.107659552	1.917119972	0.753471877	-1.730079401	0.428099675	-0.653051785	NA	-1.730079401	0.428099675	-1.05910658	0.018822353
AT3G27960	KLCR2	-0.049637146	0.942313594	-0.34375725	0.588962061	-0.888770254	0.012266401	-1.00562624	0.287797874	-0.888770254	0.012266401	-1.059691734	1.0255E-11
AT3G19553	PUT5	-0.454856281	0.227719436	-0.59949116	0.019201293	-0.857691438	0.000515042	0.212147033	0.872508582	-0.857691438	0.000515042	-1.062333903	9.39157E-08
AT1G48690	AT1G48690	2.982232584	0.280616553	-1.05347656	0.798508123	0.82483084	0.860679009	0.445395445	0.870759043	0.82483084	0.860679009	-1.062480724	0.011468186
AT1G28400	AT1G28400	-0.314562834	0.664977268	-0.855608286	0.008840736	-0.690570745	0.038607822	-0.564879792	0.740082034	-0.690570745	0.038607822	-1.063819735	9.23858E-09
AT2G17820	HK1	0.938709587	0.000957603	0.148059852	0.719456248	0.514703109	0.147655555	-0.486705806	0.554617999	0.514703109	0.147655555	-1.064848163	1.62738E-10
AT3G21530	AT3G21530	-0.47949048	0.555604126	-0.832142323	0.683444375	-0.868686477	0.044441987	-1.026858074	0.637815859	-0.868686477	0.044441987	-1.066590237	0.00275777
AT4G00940	AT4G00940	-0.324499057	0.798876559	0.492158555	0.737569998	-0.590314469	0.620313382	-0.05867548	0.988335664	-0.590314469	0.620313382	-1.069864876	0.004973705
AT1G31950	AT1G31950	0.439368132	0.738001278	-1.249503445	0.247540917	-0.979559066	0.233654121	0.762612318	0.637815859	-0.979559066	0.233654121	-1.069941972	0.003252413
AT1G51170	UCN	0.150083368	0.851295469	-0.481233018	0.644814917	-0.201134631	NA	-0.499408896	0.697407195	-0.201134631	NA	-1.072613898	1.03223E-05
AT2G41330	AT2G41330	-0.707291596	0.375906215	-0.1673315	0.856039419	0.147977257	0.85359159	-0.232523838	0.914901791	0.147977257	0.85359159	-1.072796314	6.82527E-06
AT1G22030	AT1G22030	-0.790402832	0.409200657	-1.71720156	0.107707978	-1.392025358	0.09525263	0.191753125	0.955547263	-1.392025358	0.09525263	-1.073243035	0.004720271
AT2G27010	CYP705A9	3.005543734	0.424702902	-0.550003888	0.9213441	0.628694461	0.354159668	-0.468581202	0.942141085	0.628694461	0.354159668	-1.075078221	1.36056E-06
AT5G65660	AT5G65660	-0.359640812	0.377846953	0.330820997	0.269924621	0.431605169	0.066109407	-0.882796128	0.336088856	0.431605169	0.066109407	-1.076255629	1.51789E-14
AT2G22790	AT2G22790	-0.380340771	0.681722244	-0.625579875	0.400497572	-0.856843354	0.23748647	0.503730008	0.712045709	-0.856843354	0.23748647	-1.076641548	0.003235952
AT4G25390	AT4G25390	0.091793842	0.907968972	0.391958779	0.633762434	-1.000256801	0.096069156	-0.453427804	0.816446062	-1.000256801	0.096069156	-1.079011909	0.000751258
AT5G21280	AT5G21280	0.249569655	0.737070016	0.1824677	0.759617746	-0.441966414	0.474062925	-0.500008014	0.644839236	-0.441966414	0.474062925	-1.081617762	1.10746E-05
AT1G06640	AT1G06640	-0.025348595	0.967050888	0.193921508	0.37389734	-0.794750188	0.068373635	-0.094935627	0.938659772	-0.794750188	0.068373635	-1.082286695	5.1824E-12
AT1G69050	AT1G69050	1.613835704	0.413883229	0.359932654	0.85090769	-0.866508319	0.680310698	-0.025113205	0.993871379	-0.866508319	0.680310698	-1.083616349	0.003417555
AT3G47980	AT3G47980	-1.330039806	0.249218629	-2.306412919	0.235453593	-2.326889436	0.287584922	-0.271778514	0.93555914	-2.326889436	0.287584922	-1.089538175	0.005028484
AT1G10682	AT1G10682	0.908499721	0.008895533	0.202900274	0.483456871	0.330642007	0.101326442	-0.321747094	0.753645324	0.330642007	0.101326442	-1.092221784	4.54231E-07
AT1G77400	AT1G77400	-0.669444761	0.639187797	-1.608206096	0.069729503	-0.937386902	0.212300476	-0.131749784	0.968655039	-0.937386902	0.212300476	-1.094781084	0.0008667
AT2G48090	AT2G48090	0.943747502	0.711547924	2.073195145	0.239270881	1.900153403	0.146902214	-0.590528445	0.920392797	1.900153403	0.146902214	-1.095676688	0.00126077
AT5G52120	PP2-A14	-0.282295301	0.700884002	-1.312903795	0.070566626	-0.633527896	0.186413519	-0.343174153	0.719188014	-0.633527896	0.186413519	-1.096216395	0.000143411
AT1G06980	AT1G06980	0.08926912	0.932723124	-0.81307343	0.079278554	-0.184298944	0.835163343	-0.931240019	0.286535095	-0.184298944	0.835163343	-1.096388011	9.64562E-10
AT4G29140	ADS1	0.325267846	0.754468472	-1.147556113	0.515490853	-0.112644282	0.866988322	-0.982572245	0.665832077	-0.112644282	0.866988322	-1.102478401	1.50205E-11
AT1G61560	MLO6	1.857861773	0.430204196	0.528572054	0.896100784	-0.879989264	0.282368111	-1.861289103	0.527892502	-0.879989264	0.282368111	-1.105645993	0.00011141
AT2G35910	AT2G35910	0.116730872	0.945980546	-0.692955679	0.756342205	-0.988903928	0.182050453	-1.694142977	0.465456099	-0.988903928	0.182050453	-1.108588663	0.006750892
AT5G11420	AT5G11420	-0.306328657	0.592887746	-0.018211517	0.945943497	-0.198585513	0.189312765	0.716934699	0.672844426	-0.198585513	0.189312765	-1.109361731	1.62557E-31
AT3G13790	ATBFRUCT1	-0.222667678	0.668475598	-0.893235783	0.007904209	-0.258397031	0.709467638	-0.791320281	0.348017657	-0.258397031	0.709467638	-1.113008048	5.80197E-08
AT5G26660	MYB86	2.968079187	0.439147173	-2.249234854	0.605391946	3.533875423	0.368213558	-2.375921139	0.6015277	3.533875423	0.368213558	-1.113048637	0.021196351
AT3G26180	CYP71B20	0.354540561	0.55193911	0.664695675	0.004611602	0.351546195	0.039657347	1.414365758	0.530016256	0.351546195	0.039657347	-1.113712512	1.03707E-05
AT1G02430	ARFD1B					0.928818755	0.409424921	-3.938649031	0.726900342	0.928818755	0.409424921	-1.115134812	0.015851355
AT1G72360	ERF73	0.881805213	0.204601959	-0.379513403	0.546324898	-0.72760537	0.310723482	0.100787597	0.962474985	-0.72760537	0.310723482	-1.118928791	9.93911E-07
AT4G39070	BZS1	0.587939176	0.416062601	-0.346232586	0.549694815	0.205148623	0.667715393	-1.004830786	0.386146892	0.205148623	0.667715393	-1.121638213	1.78229E-08
AT5G06800	AT5G06800	0.620721669	0.400311988	-0.505370913	0.400075583	0.037773525	0.956844888	-0.778959441	0.260339395	0.037773525	0.956844888	-1.12236142	2.49291E-09
AT1G66480	AT1G66480	1.358719301	0.123130802	1.101379077	0.086425313	0.143189231	0.866296846	-0.346771708	0.850570445	0.143189231	0.866296846	-1.126374945	0.000147158
AT2G32300	UCC1	1.078100962	0.271672665	-0.309904495	0.847313057	-0.80610698	0.495123677	-1.601436152	0.128676662	-0.80610698	0.495123677	-1.126829697	5.42948E-13
AT4G31110	AT4G31110	0.905125688	0.269572659	0.225204249	0.862918578	0.897753607	0.437900726	-0.668028793	0.629085847	0.897753607	0.437900726	-1.128872144	4.89574E-09
AT4G35060	HIPP25	1.121248241	0.373593458	0.093310947	0.929298011	-0.691397234	0.441624455	-1.091141456	0.79987193	-0.691397234	0.441624455	-1.129245904	0.000116793
AT1G61740	AT1G61740	-0.080213926	0.832478748	0.062758419	0.877821415	0.092562665	0.639397891	-0.430518116	0.581307949	0.092562665	0.639397891	-1.130531578	3.9706E-16
AT1G44970	AT1G44970	0.132450928	0.947774299	-0.297721669	0.914040617	-1.103073209	0.476287333	-1.135006071	0.611336047	-1.103073209	0.476287333	-1.131161263	8.40441E-10
AT3G27027	AT3G27027	2.131329807	0.058617708	-0.405213643	0.780221119	0.2699414	0.670956456	-0.820507257	0.694290176	0.2699414	0.670956456	-1.131535755	6.2898E-05
AT5G56050	AT5G56050	0.299683514	0.936239933	-2.178416444	0.426124795	0.067866426	0.967511145	-2.019073836	0.626700633	0.067866426	0.967511145	-1.132062832	0.001800452
AT5G25620	YUC6	1.573337523	0.36872108	-1.32516698	0.107191683	-0.431239539	0.542098243	0.014673104	0.996868544	-0.431239539	0.542098243	-1.132470478	2.44154E-08
AT4G34880	AT4G34880	2.838919036	0.340380926	2.508049071	0.439349182	0.202381979	0.938694663	-1.38411416	0.579644733	0.202381979	0.938694663	-1.133122239	0.001267393
AT1G51800	IOS1	-0.02964233	0.991889547	2.527989216	NA	0.213632952	0.957066336	-1.878194517	0.648501763	0.213632952	0.957066336	-1.134614757	0.002043886
AT1G02620	AT1G02620	-0.586986003	0.511974683	-0.733918157	0.104615608	0.487157942	0.167385684	1.296675589	0.648249035	0.487157942	0.167385684	-1.135142422	0.003636755
AT4G30670	AT4G30670	2.08691536	0.191184346	0.653044838	0.501525443	0.925336521	0.35709046	-1.369733685	0.616300683	0.925336521	0.35709046	-1.135352813	1.27726E-05
AT4G10310	HKT1	0.054507275	0.953506507	-0.45789022	0.667228396	-0.120796566	0.920316832	-0.240523596	0.935083701	-0.120796566	0.920316832	-1.137547792	0.002388048
AT1G53680	GSTU28	1.437037235	0.331020991	2.656939645	0.139224454	1.346129206	0.065405344	-0.889675821	0.885120518	1.346129206	0.065405344	-1.140015925	0.038365604
AT5G60950	COBL5	-0.042884735	0.980195352	0.270205187	0.918354317	1.467694198	0.054307216	-1.450066422	0.712563324	1.467694198	0.054307216	-1.142159637	0.003020495
AT5G28620	AT5G28620					-0.242864449	0.302279369			-0.242864449	0.302279369	-1.142753137	2.59532E-08
AT3G26612	AT3G26612	0.765297948	0.26903587	0.386112208	0.648262698	-0.219442441	0.811560163	0.895861935	0.606948021	-0.219442441	0.811560163	-1.144961403	0.007556604
AT1G23530	AT1G23530	1.53735195	0.430240798	0.428158939	0.841761444	0.20069705	0.774644973	-1.394342857	0.26835646	0.20069705	0.774644973	-1.146769425	2.72735E-05
AT1G15310	ATHSRP54A	-0.325734111	0.690294528	-0.664726546	0.233557231	-0.074784056	0.917002675	-0.727986461	0.622850451	-0.074784056	0.917002675	-1.148662078	0.000175098
AT3G19370	AT3G19370	0.204784811	0.777447935	0.025710349	0.978274786	-0.639844357	0.376972913	-0.818947351	0.684041748	-0.639844357	0.376972913	-1.151169311	1.03297E-07
AT1G70140	FH8	2.591375495	0.069465684	0.91114649	0.28737729	0.616773522	0.320520597	-0.769157254	0.878014682	0.616773522	0.320520597	-1.153090632	1.04262E-05
AT5G63590	FLS3	1.673602755	0.616850378	-0.215792059	0.956095042	1.835983441	0.746025446	-1.594021841	0.681052047	1.835983441	0.746025446	-1.157581274	4.05325E-08
AT1G23760	JP630	0.32841119	0.721020172	-1.96056781	0.247174427	-1.067598878	0.28485393	-0.691330427	0.777838956	-1.067598878	0.28485393	-1.16048313	2.2459E-11
AT1G52030	MBP2	-0.052031402	NA	-2.809863354	0.233092772	-0.888062132	0.774702389	1.686488009	0.718141493	-0.888062132	0.774702389	-1.165500778	2.57931E-05
AT4G36430	AT4G36430	9.632135004	0.056383668	2.549503846	0.186169721	-0.50261525	0.893304021	-1.308768027	0.707962062	-0.50261525	0.893304021	-1.168186235	4.30646E-11
AT3G22886	ath-MIR167a	0.03634032	0.994562076	-0.609689316	0.914524086	-0.36897996	0.834209105	-6.512653179	0.587820092	-0.36897996	0.834209105	-1.168946241	0.025212218
AT4G26890	MAPKKK16	0.085616717	0.990985016	-1.279838007	0.798495606	1.393920567	0.717850018	0.303674377	0.968416104	1.393920567	0.717850018	-1.169669172	0.027260721
AT1G74660	MIF1	0.461287873	0.650698929	-1.196784994	0.174446272	-0.972598237	0.125062435	-0.97846312	0.685495817	-0.972598237	0.125062435	-1.169863926	6.45316E-05
AT4G19950	AT4G19950	0.925486091	0.30770498	0.127698384	0.924671479	-0.689298685	0.418557734	0.026104443	0.990915497	-0.689298685	0.418557734	-1.17006807	1.20224E-06
AT5G06190	AT5G06190	0.923056888	0.827671598	2.125106317	0.387067138	0.973459887	0.197896565	-0.705973237	0.914115136	0.973459887	0.197896565	-1.172359653	0.001391072
AT2G26650	KT1	0.651066031	0.020844002	0.153022353	0.617865136	0.135437859	0.648429144	-0.374400136	0.597380545	0.135437859	0.648429144	-1.173695318	5.47478E-17
AT4G20480	AT4G20480	-0.354315772	0.412525841	-0.35329671	0.291544204	0.556269913	0.238751976	0.184735741	0.801461921	0.556269913	0.238751976	-1.173986662	3.7656E-05
AT5G55170	SUMO3	-0.234154074	0.942906453	-1.796725785	0.58646005	-3.170905869	0.095055298	-0.377296446	0.936845179	-3.170905869	0.095055298	-1.175022122	0.007013636
AT2G18210	AT2G18210	1.791627964	0.113981234	-0.860128968	0.706139201	-1.308751129	0.41470933	-0.861903438	0.856756323	-1.308751129	0.41470933	-1.179377763	0.002187294
AT2G03010	AT2G03010	1.363584319	0.341129948	0.850181561	0.791461094	0.906106629	0.08134847	0.707924014	0.751308246	0.906106629	0.08134847	-1.180731891	0.021128848
AT5G23660	SWEET12	0.441664026	0.282921879	-0.178096617	0.696596998	-0.98605951	0.048233266	-0.866665197	0.478067535	-0.98605951	0.048233266	-1.181774877	2.73774E-11
AT4G18830	OFP5	2.036100181	0.327479051	0.836013588	0.647852731	0.030572026	0.960027527	-0.296268569	0.976073408	0.030572026	0.960027527	-1.18236604	2.10083E-05
AT2G21840	AT2G21840	0.717514861	0.72513189	-1.529710856	0.590099662	1.003813809	0.73277749	-0.561219697	0.926730556	1.003813809	0.73277749	-1.184279712	0.049598109
AT4G36060	bHLH11	0.584225152	0.586231466	-2.735253191	0.06023465	-1.643007027	0.23189513	-1.449162483	0.511554278	-1.643007027	0.23189513	-1.184503542	4.05226E-05
AT3G26500	PIRL2	1.004699888	0.279788531	0.837838794	0.482421047	0.024565118	0.97900791	0.579533914	0.792048637	0.024565118	0.97900791	-1.185006868	0.008868117
AT1G24130	AT1G24130	-0.271903794	0.939264378	-0.371874742	0.942397584	-1.690331097	0.134310058	-1.354063233	0.613859108	-1.690331097	0.134310058	-1.185915391	0.002616991
AT3G19660	AT3G19660	3.824501078	0.287203431	2.007054938	0.134995485	0.275313799	0.876460982	-0.772965262	0.644839236	0.275313799	0.876460982	-1.186175819	0.012930742
AT1G18390	AT1G18390	-0.437231485	0.458649412	-0.706791878	0.1387818	-0.268818818	0.58085566	-0.796853297	0.56497814	-0.268818818	0.58085566	-1.186429482	2.37648E-06
AT5G65800	ACS5	7.461319212	0.064996634	-5.699236538	0.283302378	4.907112755	0.112918896	-1.20759198	0.767662883	4.907112755	0.112918896	-1.189307684	4.35849E-07
AT4G32480	AT4G32480	0.131934637	0.850003552	0.393657133	0.189484518	0.220237861	0.309323826	0.647301095	0.721140128	0.220237861	0.309323826	-1.190794296	1.23417E-09
AT1G72300	PSY1R	-0.477097378	0.274795278	-0.475857752	0.112994569	-0.519456514	0.04665877	-0.023906954	0.991271129	-0.519456514	0.04665877	-1.191209772	8.84638E-09
AT5G47050	AT5G47050	0.509560673	0.283075215	-0.544095163	0.274940621	-0.15280621	0.727196239	-0.74435993	0.644839236	-0.15280621	0.727196239	-1.193224401	4.10075E-18
AT5G60760	AT5G60760	1.783443698	0.398870207	-0.595989115	0.677080743	0.872388176	0.135198788	-1.224082407	0.758815467	0.872388176	0.135198788	-1.194853743	2.71202E-07

AT3G49830	AT3G49830	2.326026747	0.265335134	1.234371366	0.427769971	-1.204751424	0.232209977	-0.88615962	0.632278424	-1.204751424	0.232209977	-1.1964633	0.009966429
AT1G08310	AT1G08310	3.184531379	0.238638209	0.23337192	0.897008917	1.976519694	0.058571654	-1.010571135	0.827716472	1.976519694	0.058571654	-1.198410461	0.006750892
AT3G01950	AT3G01950					-0.126243295	0.900886508			-0.126243295	0.900886508	-1.201208403	0.000329871
AT1G79310	MC7	1.470366846	0.450025297	1.143546316	0.616623179	-1.952324614	0.471374816	-0.331908226	0.887058556	-1.952324614	0.471374816	-1.201239139	0.007059439
AT4G15765	AT4G15765	1.116362759	0.206970805	-0.090825853	0.941640409	0.60381841	0.586435301	-0.844822512	0.731041294	0.60381841	0.586435301	-1.201430062	5.13563E-08
AT3G30849	None					-0.727364686	0.464644883			-0.727364686	0.464644883	-1.204402921	1.2705E-07
AT2G47200	AT2G47200					2.463575438	0.178815071			2.463575438	0.178815071	-1.2055433	0.009652273
AT5G50200	WR3	1.425827812	0.21589116	0.468943268	0.559358042	-0.798838377	0.656573964	-0.475291832	0.677620385	-0.798838377	0.656573964	-1.207942436	8.61481E-06
AT1G11580	PMEPCRA	0.634700984	0.206587707	-0.958101081	0.001160928	-1.004554931	0.102270292	-0.590114413	0.691824202	-1.004554931	0.102270292	-1.208437219	3.73186E-24
AT5G66590	AT5G66590	-0.412582081	0.362404064	0.408966525	0.321770361	0.422785732	0.190064869	-0.839498501	0.079047322	0.422785732	0.190064869	-1.21282286	5.15702E-17
AT5G49360	BXL1	0.118123083	0.836550078	0.626603679	0.002534704	0.881147102	2.45698E-07	0.570799073	0.709813898	0.881147102	2.45698E-07	-1.213261554	4.46611E-17
AT5G22930	AT5G22930	-0.437018911	0.840483992	-1.659202594	0.09713356	-0.787436198	0.853800145	-0.461900839	0.742807747	-0.787436198	0.853800145	-1.214251908	0.000622048
AT3G45220	AT3G45220	6.884984217	0.134217691	2.184083746	0.71655539	1.834267798	0.098113352	-1.556676079	0.853012539	1.834267798	0.098113352	-1.214672856	0.02871812
AT1G60989	SCRL7	1.030038767	0.352365134	0.055489084	0.961614902	-0.114463109	0.931101383	-0.419212057	0.876015705	-0.114463109	0.931101383	-1.217827258	0.000498794
AT5G61010	EXO70E2	-0.353410062	0.533297696	-0.933871861	0.114911754	-0.351321537	0.540099428	0.241970153	0.928888991	-0.351321537	0.540099428	-1.220690216	8.49409E-05
AT4G15230	ABCG30	0.703696735	0.402891653	-0.996290733	0.211141196	-0.606821182	0.268392563	-0.787868858	0.498460747	-0.606821182	0.268392563	-1.221940209	4.28455E-08
AT5G57620	MYB36	1.171385676	0.562562538	-1.531736144	0.597465279	-2.750127507	0.156947465	-0.580228936	0.797097845	-2.750127507	0.156947465	-1.225556481	7.12123E-05
AT2G24140	AT2G24140	2.09563645	0.331684513	-5.480834859	0.099195753	-1.381255975	0.648048474	-0.240176632	0.973864665	-1.381255975	0.648048474	-1.229263742	0.001527504
AT5G01830	SAUR21	-0.301870318	0.747470362	-1.015031215	0.067312842	0.114328756	0.821828822	-0.881607034	0.648919095	0.114328756	0.821828822	-1.23343328	2.35717E-05
AT5G40450	AT5G40450	0.316968459	0.327479051	-0.30386406	0.073408394	0.263400755	0.015968599	-0.0140588	0.994162268	0.263400755	0.015968599	-1.234301047	1.61954E-23
AT1G60680	AT1G60680	0.923771032	0.292095436	0.226788109	0.815577011	-0.036811735	0.980869098	-0.705313071	0.776796299	-0.036811735	0.980869098	-1.237153079	4.52732E-13
AT5G18290	SIP1;2	0.010808653	0.991544266	-1.221321239	0.115109335	-0.046717057	0.970526572	0.020853943	0.993101238	-0.046717057	0.970526572	-1.239452352	0.017654596
AT2G03200	AT2G03200	1.985784291	0.234715116	-2.324264973	0.416431345	-0.573550232	NA	-1.619427982	0.486317517	-0.573550232	NA	-1.240001942	1.12006E-07
AT1G67150	AT1G67150	2.457035985	0.317066132			-5.956992411	0.14809234	-2.855402013	0.627689165	-5.956992411	0.14809234	-1.244526643	0.006398744
AT1G75030	TLP-3	0.487620427	0.806380855	0.427965398	0.71854982	0.775657036	0.542196082	-0.8897392	0.705340124	0.775657036	0.542196082	-1.244531677	0.000111258
AT1G11410	AT1G11410	-0.930554599	0.188481312	0.490728863	0.416290064	-0.095317023	0.895465743	-0.071657065	0.974340012	-0.095317023	0.895465743	-1.246278987	8.42917E-08
AT1G53270	ABCG10	3.652974606	0.139655928	3.080638187	0.11978648	1.367303368	0.447847961	-1.059294177	0.613859108	1.367303368	0.447847961	-1.252502238	0.004344947
AT1G68238	AT1G68238	-1.3209249	0.188542655	-0.78657803	0.394729866	-2.91944847	0.152280716	0.208982815	0.938741823	-2.91944847	0.152280716	-1.252505718	0.000804505
AT1G72510	AT1G72510	0.475509761	0.256633874	0.319615926	0.465475738	-0.080093473	0.796949486	-0.484694342	0.62738272	-0.080093473	0.796949486	-1.255250857	4.56672E-20
AT1G76500	SOB3	1.521895626	0.078826442	-0.784583192	0.445457934	0.421141885	0.602594608	-0.366761673	0.876015705	0.421141885	0.602594608	-1.255835844	2.1925E-10
AT5G41590	AT5G41590	2.450825379	0.243797549	0.765165381	0.512805574	1.287122198	0.225825086	-0.329263371	0.935898864	1.287122198	0.225825086	-1.256396704	0.044438842
AT4G16230	AT4G16230	-0.396821978	0.931967385	-6.068341728	0.117899325	-1.446392555	0.605026797	1.228828486	0.893576528	-1.446392555	0.605026797	-1.258793335	0.037244443
AT5G15830	bZIP3	-0.096721225	0.917943932	-1.310716059	0.163482329	-0.655311843	0.413328848	-0.712050248	0.572365365	-0.655311843	0.413328848	-1.261626343	2.95475E-13
AT2G21100	AT2G21100	1.661923591	0.463550603	0.163120917	0.965795256	-0.581877517	0.866988322	-0.948779158	0.697104641	-0.581877517	0.866988322	-1.263794291	1.16942E-06
AT4G30110	HMA2	0.75407276	0.333981509	0.839487128	0.226104403	0.302156798	0.773110211	-0.631931753	0.73994806	0.302156798	0.773110211	-1.265080097	4.73995E-07
AT1G73290	scpl5	4.835996915	NA	-2.876163836	NA	-1.108056393	NA	-4.064318458	0.396407907	-1.108056393	NA	-1.265189742	0.045542883
AT5G65160	TPR14	-0.036100676	0.980336065	-0.990607058	0.653253574	-0.972249103	0.332990826	-0.683178984	0.894347553	-0.972249103	0.332990826	-1.26898744	0.029236849
AT2G33205	AT2G33205	0.412142123	0.928150491	-0.712167	NA	-1.998936573	0.433904652	-1.637548823	0.693517591	-1.998936573	0.433904652	-1.271152625	0.015686179
AT5G37740	AT5G37740	0.655438803	0.180929138	-0.005488758	0.992544898	-0.119914956	0.793489655	-0.959151632	0.406418036	-0.119914956	0.793489655	-1.273203932	4.85488E-16
AT5G47635	AT5G47635	2.878763339	0.539551213	-5.138178927	0.33816757	2.278856153	0.180044113	-1.306399908	0.874584357	2.278856153	0.180044113	-1.274008089	0.028865929
AT4G24140	AT4G24140	-1.202114975	0.119311697	-2.110765033	0.186760802	-0.985893467	0.549855394	-1.031048872	0.820789421	-0.985893467	0.549855394	-1.275110817	3.61976E-08
AT3G57160	AT3G57160	-0.468707563	0.756646548	-0.995925204	0.41109307	-0.317596482	0.863608504	-0.315430658	0.92099795	-0.317596482	0.863608504	-1.275871098	0.010125608
AT3G15510	NAC2	-0.132125248	0.808547683	-0.728027163	0.02553135	-0.228599786	0.414043264	-0.259211291	0.807059483	-0.228599786	0.414043264	-1.275972568	1.02849E-11
AT1G31240	AT1G31240	1.009181557	0.555256284	1.267607832	0.176092099	0.351779614	0.735558652	0.98297548	0.856770017	0.351779614	0.735558652	-1.281010068	0.027461053
AT2G23430	ICK1	0.058730122	0.953696902	0.079513871	0.924671479	-0.839019129	0.003635299	0.213499656	0.937367978	-0.839019129	0.003635299	-1.281682198	0.002631465
AT1G12160	AT1G12160	2.458830011	0.190166506	0.953014531	0.672989534	1.789212966	0.762975261	-0.661459244	0.781069404	1.789212966	0.762975261	-1.285362564	0.002066927
AT5G58360	OFP3	0.955376067	0.808849727	-0.451607437	0.943194995	1.245523883	0.662419062	-0.769261316	0.90560947	1.245523883	0.662419062	-1.288335577	0.010386828
AT3G12720	MYB67	2.8660943	0.456467021					-2.374440063	0.712887303			-1.289753366	0.021136341
AT3G05890	RCI2B	0.319984038	0.836208955	0.507019896	0.391816656	0.153511689	0.766825571	-1.196108637	0.640704059	0.153511689	0.766825571	-1.293976962	2.69812E-10
AT2G24550	AT2G24550	0.108148306	0.87149223	0.107165159	0.854975652	-0.718047421	0.008347285	0.177230124	0.910428643	-0.718047421	0.008347285	-1.295535886	3.56142E-11
AT3G46880	AT3G46880	1.25735147	0.855564757	-6.579545749	0.154812137	-2.546327835	0.371517726	-0.509479938	0.943501162	-2.546327835	0.371517726	-1.298059732	0.037387931
AT3G21330	AT3G21330	0.293133103	0.52961534	0.697040354	0.282849712	-0.548750069	0.027612716	0.130524115	0.935532809	-0.548750069	0.027612716	-1.302214381	4.05325E-08
AT5G44578	AT5G44578	-0.313798814	0.872485744	1.213798125	0.676332404	-0.421071977	0.838269275	-1.148048086	0.781069404	-0.421071977	0.838269275	-1.302563717	1.17904E-07
AT4G02410	AT4G02410	-0.328703654	0.68870151	0.486862343	0.244833478	-0.410980826	0.682487301	-0.74034124	0.601921549	-0.410980826	0.682487301	-1.304071609	0.027159016
AT1G11735	ath-MIR171b	-1.701788452	0.61697383	-0.860399954	0.872982515	0.035195609	0.973961505	-1.639837439	0.704225272	0.035195609	0.973961505	-1.306009264	0.000233496
AT1G33440	AT1G33440	0.548854232	0.62154103	-0.060271732	0.962340572	0.748217559	0.158652977	0.358231569	0.857472027	0.748217559	0.158652977	-1.30677542	7.06248E-06
AT4G10240	bbx23	6.689387775	0.226765184	6.748194672	0.194742577	-0.302544156	0.921741659	-0.540442098	0.930313852	-0.302544156	0.921741659	-1.311461152	8.77599E-05
AT1G80310	MOT2					0.702274903	0.002164083			0.702274903	0.002164083	-1.3191446	1.46171E-15
AT5G02640	AT5G02640	1.018590408	0.379026046	1.203427171	0.321130575	1.897256681	0.058463135	-0.282235797	0.914259529	1.897256681	0.058463135	-1.319203369	0.000641913
AT2G02680	AT2G02680	0.846661684	0.743494745	-1.512379414	0.641335865	1.820296746	NA	-1.14299519	0.79089306	1.820296746	NA	-1.322683613	1.2637E-05
AT4G17220	MAP70-5	-0.490588011	0.876852852	1.661836793	0.577659163	-0.543136789	0.826497675	-0.98535566	0.616300683	-0.543136789	0.826497675	-1.323940083	0.001414516
AT2G42280	FBH4	0.455178979	0.348437987	0.010465295	0.988868832	0.462213304	0.038525248	0.445511186	0.665119721	0.462213304	0.038525248	-1.325788786	1.01278E-12
AT2G34020	AT2G34020	3.134598627	0.352582795	-1.274231405	0.694348273	-5.3951404	0.270902528	-1.079956816	0.534532914	-5.3951404	0.270902528	-1.328134505	3.22832E-05
AT5G05960	AT5G05960	1.180920471	0.266284229	0.247008155	0.694458582	0.848107671	0.310270457	-0.34772123	0.905084907	0.848107671	0.310270457	-1.32954625	4.97537E-11
AT1G74020	SS2	0.97181608	0.122960295	0.040486165	0.96441214	-0.622713061	0.276441321	-1.115304555	0.491415157	-0.622713061	0.276441321	-1.331315136	2.21938E-18
AT4G31520	AT4G31520	0.492779781	0.711087984	0.072902937	0.974275566	-0.300719986	0.694079023	-1.491922303	0.613859108	-0.300719986	0.694079023	-1.337802269	0.013751232
AT2G39210	AT2G39210	0.369556685	0.410659178	0.131951616	0.792839385	-0.258007546	0.654470783	0.073214516	0.951485287	-0.258007546	0.654470783	-1.339175764	6.32536E-16
AT1G17030	AT1G17030	6.540106094	0.203989398	2.995663829	0.600552247	-0.408318156	0.94503851	-2.646696094	0.612475768	-0.408318156	0.94503851	-1.340244155	0.016748553
AT3G43850	AT3G43850	-0.332706388	0.614856499	-0.357213647	0.350658191	-0.305862111	0.097563203	0.789144648	0.66034304	-0.305862111	0.097563203	-1.34281127	3.02587E-08
AT3G21780	UGT71B6					1.140734095	0.42485059			1.140734095	0.42485059	-1.344541425	0.035664965
AT2G43865	AT2G43865					0.981774153	0.615610139	-4.792276676	0.676109289	0.981774153	0.615610139	-1.344582205	0.039980123
AT1G22160	AT1G22160	0.77180537	0.342968262	0.073729507	0.928451507	0.490154979	0.339782251	0.362755754	0.857472027	0.490154979	0.339782251	-1.346497318	2.50942E-05
AT1G62660	AT1G62660	0.70265597	0.133271315	0.445276639	0.307443819	0.126518296	0.775632982	-0.949863424	0.344860927	0.126518296	0.775632982	-1.348396015	1.53483E-16
AT1G80820	CCR2	0.765050941	0.379077519	0.10770086	0.903804629	0.505141685	0.577447254	-1.308055429	0.420106526	0.505141685	0.577447254	-1.349831001	2.08434E-09
AT5G66985	AT5G66985	0.822935685	0.834551872	-3.61168822	0.112487564	-2.646068659	0.258236079	-1.70295911	0.669734807	-2.646068659	0.258236079	-1.351994986	0.030212939
AT5G05790	AT5G05790	0.258457523	0.75566658	-0.697248047	0.393499406	-1.452139572	0.052663032	-0.116533638	0.951652663	-1.452139572	0.052663032	-1.352292208	0.014207615
AT5G06510	NF-YA10	0.25136424	0.93988726	0.106044089	0.969729551	-2.37544944	0.322500867	-0.241608594	0.93556951	-2.37544944	0.322500867	-1.353173604	0.000589782
AT3G47790	ABCA8	3.417823609	0.205866724	0.134996645	0.980651529	1.311626013	0.585081784	-2.152528768	0.092759749	1.311626013	0.585081784	-1.35393421	1.8942E-05
AT1G22440	AT1G22440	2.228728774	0.099920997	0.2680834	0.862918578	-0.920051802	0.609205802	-0.696183811	0.795048376	-0.920051802	0.609205802	-1.354827889	1.01148E-09
AT3G49330	AT3G49330	0.953555113	0.798876559	-1.061446525	0.772293002	-0.768090244	0.837172929	-0.196184293	0.978345558	-0.768090244	0.837172929	-1.356645489	7.02264E-05
AT4G26150	CGA1	-0.327904564	0.717934169	0.368485726	0.528350075	0.344932342	0.458423879	-0.127946752	0.976275631	0.344932342	0.458423879	-1.357910148	2.65901E-05
AT5G66670	AT5G66670	-1.524491459	0.571620273	1.073253549	NA	-0.503907597	0.871759136	-1.263064485	0.788737041	-0.503907597	0.871759136	-1.358127387	0.030109392
AT5G63970	RGLG3	0.632746583	0.331609471	0.894079538	0.161946736	0.370335892	0.527319243	-0.94393594	0.6278704	0.370335892	0.527319243	-1.358316406	2.32542E-09
AT2G36380	ABCG34	0.754625993	0.060314066	0.289968656	0.225955971	0.577385173	0.030122827	-0.483780123	0.62738272	0.577385173	0.030122827	-1.358356505	1.32775E-17
AT5G28660	AT5G28660					-1.231658503	0.574818048	-6.122936828	0.615454805	-1.231658503	0.574818048	-1.364576214	0.010419301
AT3G60490	AT3G60490	1.290567508	0.352365134	-0.408830397	0.862396779	1.275989358	0.734099232	-0.026002388	0.993804788	1.275989358	0.734099232	-1.370387261	0.049977645
AT2G04450	NUDT6	-1.729071377	0.236886943	-0.559913212	0.83393857	-3.261166836	0.094993248	-0.174805026	NA	-3.261166836	0.094993248	-1.3713849	2.60915E-07
AT4G35030	AT4G35030	-0.539144529	0.622849484	-1.30997485	0.315778522	-0.040179571	0.963329013	-0.388273645	0.906874792	-0.040179571	0.963329013	-1.373500213	1.68995E-05
AT4G19960	KUP9	-1.089057514	0.175263074	-0.171195803	0.787412227	-0.225219649	0.575906828	-0.066868979	0.985414921	-0.225219649	0.575906828	-1.374188761	2.90379E-08
AT5G37690	AT5G37690	1.6533208	0.368100522	-1.874333685	0.635426813	4.435573524	NA	-1.136235476	0.693517591	4.435573524	NA	-1.375139552	6.01105E-11
AT1G03850	GRXS13	0.321861458	0.581956151	-0.24103035	0.505009596	0.573023302	0.003971263	0.76520896	0.697407195	0.573023302	0.003971263	-1.384070943	2.43585E-10
AT4G20460	AT4G20460	0.105375487	0.95823127	1.33926872	0.108200477	0.21516871	0.878119301	-1.517476003	0.718141493	0.21516871	0.878119301	-1.385192593	0.00080451
AT4G17280	AT4G17280	0.749251377	0.673700243	-0.808115099	0.787068883	0.229914533	0.87920389	-1.652363025	0.280737992	0.229914533	0.87920389	-1.38525928	3.46815E-14
AT3G05630	PLDP2	2.158108824	0.262784525	1.293245599	0.087478397	0.764523172	0.401844047	-0.799575982	0.677952867	0.764523172	0.401844047	-1.38566602	4.34862E-05

AT2G05910	AT2G05910	2.209065936	0.373996867	-0.291608105	0.927202865	1.850252598	0.437339833	-0.826792685	0.882425056	1.850252598	0.437339833	-1.386560756	0.004286711
AT1G76250	AT1G76250	0.153899005	0.915025919	-1.223011156	0.557044914	0.043479313	0.98177747	-1.090723644	0.662219342	0.043479313	0.98177747	-1.394543904	0.000288059
AT5G58780	cPT5	-3.204052473	NA	-2.591101112	NA	5.085392867	0.340353148	-1.476478097	0.731041294	5.085392867	0.340353148	-1.400452781	0.009416269
AT5G65070	MAF4	-0.515415929	0.587563383	-0.492641515	0.671586851	0.829355989	0.310875751	0.769592813	0.805968219	0.829355989	0.310875751	-1.400480547	0.007146814
AT1G08290	WIP3	4.779764171	0.098348677	1.358788758	0.609797401	-0.107911136	0.968750966	-0.77730286	0.70850021	-0.107911136	0.968750966	-1.401121749	0.000182377
AT5G40690	AT5G40690	0.387103103	0.717563959	-0.446772753	0.690779838	0.609701459	0.489090792	-1.157860674	0.562656211	0.609701459	0.489090792	-1.4014119	0.001301164
AT3G15990	SULTR3;4	0.787991819	0.267541701	0.60804062	0.425371786	-0.138011239	0.90932566	-1.066008299	0.204145338	-0.138011239	0.90932566	-1.405384197	8.53597E-09
AT3G28540	AT3G28540	2.383583595	0.164196083	-0.34611342	0.893874143	0.032783087	0.975211276	-0.655073736	0.8208148	0.032783087	0.975211276	-1.405495638	2.06334E-06
AT1G15190	AT1G15190	1.435920162	0.11814774	2.800742924	0.111090537	0.532703871	0.829923582	-1.793724523	0.66808962	0.532703871	0.829923582	-1.408344447	0.001129814
AT5G49070	KCS21	1.067533989	0.689489145	1.631497021	0.418176577	1.366260302	0.29497295	0.079467776	0.993101238	1.366260302	0.29497295	-1.413223844	0.005216304
AT5G49780	AT5G49780	0.716154296	0.742295415	-0.01885892	0.997968945	-0.731646413	0.733780927	-2.110588562	0.629407893	-0.731646413	0.733780927	-1.415066877	0.001516895
AT5G02400	PLL2	-0.819440071	0.822403923	1.224771707	0.758604757	-1.730626633	0.305258261	-1.556340305	0.697914029	-1.730626633	0.305258261	-1.415638095	0.000951987
AT3G59730	AT3G59730	0.424845528	0.955538677	-4.801221518	NA			-0.92066649	0.931327819			-1.420196596	0.010773184
AT1G34040	AT1G34040	4.088584834	0.067851621	6.026554986	0.254536337	4.849701515	0.366175561	-0.189255067	0.953275639	4.849701515	0.366175561	-1.426648098	0.041562802
AT2G01610	AT2G01610	-0.199448503	0.927793253	-1.18856623	0.607837548	-0.509746403	0.61016442	-0.27715822	0.967598896	-0.509746403	0.61016442	-1.428144689	4.916E-10
AT1G13830	AT1G13830	-0.628999023	0.892677274	-1.70879066	0.782980233	-0.668341169	0.836423786	-1.097911186	0.80141891	-0.668341169	0.836423786	-1.428660727	0.000918204
AT5G35370	AT5G35370	0.770807699	0.203726312	1.006002423	0.063299476	0.724461859	0.031389715	-0.368659265	0.616166756	0.724461859	0.031389715	-1.432319198	1.82556E-12
AT3G02620	AT3G02620	1.884758648	0.065633615	1.393502365	0.104218831	0.121484894	0.96198573	-0.320353911	0.8627468	0.121484894	0.96198573	-1.434253978	0.003547062
AT1G12290	AT1G12290	0.423712	0.616015417	0.76304999	0.582450724	0.536010889	0.486015462	0.132775047	0.965803105	0.536010889	0.486015462	-1.436876417	0.000250157
AT1G22230	AT1G22230	0.9041819	0.346779729	-0.119262013	0.936671353	-0.040230899	0.968310948	-0.895725875	0.62738272	-0.040230899	0.968310948	-1.439701846	1.94909E-07
AT3G09260	PYK10	-0.276366098	0.676749328	-0.959889191	2.65577E-07	-1.061786554	0.156652118	-0.79243821	0.605522309	-1.061786554	0.156652118	-1.442859877	1.74821E-49
AT2G45560	CYP76C1	-0.704276942	0.320410658	0.39723343	0.190173712	-0.281441329	0.334268954	1.175833995	0.62738272	-0.281441329	0.334268954	-1.442935968	1.14155E-05
AT3G16220	AT3G16220	-0.330043486	0.551400858	-0.399039052	0.210412872	0.083821693	0.774862503	1.06222106	0.589531904	0.083821693	0.774862503	-1.444568954	2.20178E-05
AT4G20235	CYP71A28	-0.627919075	0.766743654	-5.332003831	0.282684055	-5.261611557	0.200962739	0.142664967	0.984525706	-5.261611557	0.200962739	-1.447537139	0.000306684
AT3G48450	AT3G48450	2.218908689	0.511057359	0.191204252	0.969336953	-0.064343451	0.985484719	-2.00418184	0.626508471	-0.064343451	0.985484719	-1.452016401	4.15371E-05
AT1G01355	AT1G01355	4.278947524	0.175452721	5.31798644	0.155407595	1.214652585	0.254413871	-0.545263028	0.872934732	1.214652585	0.254413871	-1.454442449	0.004892271
AT3G44540	FAR4	4.194503527	0.06324136	0.075475213	0.990062038	1.003829163	0.753890836	-3.069235424	0.343851903	1.003829163	0.753890836	-1.456630044	7.70122E-08
AT5G57685	GDU3	0.858169905	0.145218881	-0.157746465	0.828665561	-0.621458739	0.395649201	-0.85037305	0.653363031	-0.621458739	0.395649201	-1.458088164	2.11848E-11
AT1G23140	AT1G23140	-0.227568691	0.95886949	-0.448003834	NA	-3.99997266	0.464644883	-1.344717421	0.602119156	-3.99997266	0.464644883	-1.459331764	0.045141893
AT2G39710	AT2G39710	0.251641286	0.879268267	0.412730893	0.779506403	-2.349683314	0.075446472	-1.191518344	0.352794607	-2.349683314	0.075446472	-1.460529035	8.31777E-07
AT2G01340	At17.1	-0.055475186	0.956873262	0.216131939	0.658719011	-0.482337372	0.358420975	1.185946987	0.470585322	-0.482337372	0.358420975	-1.463561165	0.001318767
AT4G08290	UMAMIT20	0.347469015	0.578938437	0.37567095	0.321897729	-0.710721025	0.015076934	-0.42107781	0.696374342	-0.710721025	0.015076934	-1.46731378	3.25318E-15
AT5G37980	AT5G37980					-1.212709515	0.775632982	-6.123450587	0.615454805	-1.212709515	0.775632982	-1.479491341	0.001371824
AT1G63530	AT1G63530	0.26927844	0.92730955	1.456824825	0.302571138	0.447558527	0.846491222	0.213680017	0.978216445	0.447558527	0.846491222	-1.482608587	0.00238372
AT5G49350	AT5G49350	1.41976465	0.573105566	5.873071989	0.218927972	-3.001110281	NA	-1.255744979	0.787606531	-3.001110281	NA	-1.484874485	0.009796044
AT4G22790	AT4G22790	-0.533816253	0.698048959	-0.457425124	0.778017206	-0.484330581	0.610313158	-0.527858924	0.773257427	-0.484330581	0.610313158	-1.486796471	7.02372E-09
AT3G54260	TBL36	-0.06525303	NA	-1.01460721	0.052803502	-1.017297009	0.290943144	-0.210966118	0.906077763	-1.017297009	0.290943144	-1.491913343	2.13313E-08
AT3G08040	FRD3	2.610848651	0.089910152	0.674493179	0.390329442	1.616551535	NA	-1.670779072	0.516270934	1.616551535	NA	-1.494641967	9.56498E-28
AT3G50840	AT3G50840	-0.234534938	0.76236827	0.858173243	0.078974899	0.65434973	0.33099271	0.544032123	0.662219342	0.65434973	0.33099271	-1.524139583	0.00973342
AT1G18880	NRT1.9	0.68507777	0.394615007	-0.561912863	0.363938568	-1.229238978	0.144836924	-0.715380477	0.696374342	-1.229238978	0.144836924	-1.524522857	1.05212E-33
AT2G30840	AT2G30840	3.820746636	0.093351625	0.167852671	0.966238103	2.382295607	0.361980874	-0.213223838	0.918988754	2.382295607	0.361980874	-1.530849511	2.28775E-05
AT4G13790	AT4G13790	6.551970444	0.238438206	-5.332672059	0.318821513	-4.440404814	0.413578654	-1.591988113	0.702246294	-4.440404814	0.413578654	-1.534293244	0.04748072
AT4G16745	AT4G16745	0.866388346	0.044295146	0.759314893	0.032148283	0.194200217	0.768564373	1.319962548	0.428836781	0.194200217	0.768564373	-1.535847044	0.017011655
AT4G21600	ENDO5	0.846033681	0.375820781	-1.427116367	0.207361397	-1.222356851	0.246207352	-1.111529028	0.676109289	-1.222356851	0.246207352	-1.537407403	2.18318E-16
AT5G64490	AT5G64490	1.029995167	0.34744306	1.339351598	0.103104667	-0.003768198	0.996707154	0.40310579	0.914676288	-0.003768198	0.996707154	-1.538296701	0.000340053
AT4G34600	AT4G34600	0.975830625	0.171290702	0.274496645	0.712381119	-0.19774811	0.866988322	-0.630120005	0.694819594	-0.19774811	0.866988322	-1.543718612	7.78852E-19
AT1G11460	UMAMIT26	1.747526589	0.743494745	1.319594426	NA	-0.823801283	0.876208256	-4.187537977	0.343493265	-0.823801283	0.876208256	-1.548734023	0.03792315
AT1G34670	MYB93	3.264196537	0.12836641	-5.03225793	0.269476727	-0.083869886	0.975923031	-1.194483521	0.781888051	-0.083869886	0.975923031	-1.551339546	1.14237E-08
AT5G43780	APS4	0.987118654	3.05199E-05	0.78152695	1.86779E-06	-0.350808719	0.371732733	-0.792167408	0.492642336	-0.350808719	0.371732733	-1.554574413	2.96303E-42
AT2G22496	ath-MIR779	-1.345288853	0.849963696			-2.648405744	NA	-0.651310949	0.968021479	-2.648405744	NA	-1.558633632	0.011228874
AT5G14940	AT5G14940	2.251862379	0.122609609	0.767915641	0.263153256	-0.588921439	0.558450812	-0.361451929	0.897433865	-0.588921439	0.558450812	-1.558724351	1.71646E-07
AT5G52790	AT5G52790	1.11919442	0.778218711	0.265079818	0.925890995	0.288226067	0.880656543	-1.600531405	0.685495817	0.288226067	0.880656543	-1.564833404	6.19272E-05
AT1G47480	AT1G47480	2.035731966	0.094209803	-0.883005938	0.795358087	-0.806332933	0.604089215	-1.876714615	0.336979928	-0.806332933	0.604089215	-1.565216826	1.14696E-12
AT5G61430	NAC100	1.508677609	0.445753435	-0.699009829	0.57990314	-0.806910995	0.631718403	-0.193794876	0.979362915	-0.806910995	0.631718403	-1.56836928	0.003328594
AT1G17960	AT1G17960	0.683331176	0.770931518	1.00285393	0.354428638	0.25663657	0.851641949	0.738281685	0.901814827	0.25663657	0.851641949	-1.569062324	1.24189E-06
AT3G25640	AT3G25640	0.81027498	0.626383478	-0.201826302	0.893695497	0.134854744	0.91627703	-0.25300938	0.918531026	0.134854744	0.91627703	-1.573632228	1.13641E-05
AT5G67620	AT5G67620	2.684385087	0.454333731	0.627170894	0.877317897	1.679080287	0.324757935	-2.95120543	0.626508471	1.679080287	0.324757935	-1.57400527	0.002166815
AT3G18560	AT3G18560	0.961421049	0.063081014	0.893612356	0.046053512	0.507826307	0.064463484	0.156071089	0.951485287	0.507826307	0.064463484	-1.581138699	1.7926E-13
AT1G27940	ABCB13	0.109245317	NA	2.122647388	NA	-5.922658853	0.074592903	-4.763116245	0.541116454	-5.922658853	0.074592903	-1.585293585	0.030246082
AT3G01070	ENODL16	1.353289118	0.550940511	0.338566719	NA	-1.985289418	0.502044757	-1.630645012	0.377008424	-1.985289418	0.502044757	-1.588973236	0.020942771
AT4G40065	AT4G40065	-0.464824132	0.793525331	-1.788134858	0.297851442	-0.878983063	0.020789636	-2.288235973	0.143428011	-0.878983063	0.020789636	-1.59327883	3.29148E-10
AT3G18610	NUC-L2	1.478548321	0.069939244	0.94677852	0.156699506	0.779883728	0.150488495	-0.589397067	0.765952517	0.779883728	0.150488495	-1.593933076	6.54193E-10
AT1G22500	ATL15	0.609413373	0.626701705	-0.978751867	0.17503743	-0.85800121	0.228383173	0.035384039	0.990542206	-0.85800121	0.228383173	-1.59464472	1.58168E-19
AT1G67000	AT1G67000			-5.826612831	0.271411058	-0.242152497	0.944186177	-1.445929523	0.910553097	-0.242152497	0.944186177	-1.598262755	0.009023011
AT3G53690	AT3G53690	1.185004	0.481270483	-0.704749754	0.622058105	-0.625593331	0.767961324	0.027948142	0.996799452	-0.625593331	0.767961324	-1.600249295	0.000698274
AT5G24920	GDU5	0.141521072	0.906524522	-0.34792404	0.684051731	-0.28263139	0.664190606	-0.084861824	0.982356953	-0.28263139	0.664190606	-1.600701138	2.0778E-07
AT4G33330	PGSIP3	-2.319464164	0.139293664	-0.426502949	0.922519901	-3.235435171	0.111406926	-0.177796621	0.987744909	-3.235435171	0.111406926	-1.605960388	0.00116322
AT3G27400	AT3G27400	-1.310353907	0.214695548	-2.057073559	0.067713875	-0.034723836	0.984282258	-0.604603632	0.743032554	-0.034723836	0.984282258	-1.607829024	3.76711E-06
AT5G06330	AT5G06330	1.333374897	0.57906851	1.240590667	0.794902702	0.978470219	0.689696979	-2.327998101	0.565351364	0.978470219	0.689696979	-1.610029905	6.7846E-07
AT5G38020	AT5G38020	1.028616675	NA	-6.460033498	0.05891117	-0.069381269	0.977570247	-4.020975821	0.101067989	-0.069381269	0.977570247	-1.610569662	2.20564E-09
AT1G13080	CYP71B2	-0.674755952	0.305814639	-0.107697204	0.859241931	-0.793019785	0.002830503	-0.344530331	0.859699136	-0.793019785	0.002830503	-1.61082273	4.84261E-11
AT5G37990	AT5G37990	2.132660609	0.469098323	-0.24119106	0.950818885	-5.853550278	0.160074761	-2.643993333	0.099695852	-5.853550278	0.160074761	-1.611102058	2.42062E-10
AT5G18340	AT5G18340	0.923268155	0.480922658	-0.258523181	0.861315976	-0.160287017	0.95049795	-1.396475081	0.726553713	-0.160287017	0.95049795	-1.614329024	0.017885067
AT1G16310	AT1G16310	1.245229112	0.323552135	-0.655260718	0.726772512	-0.381351549	0.847376036	-1.740535739	0.101067989	-0.381351549	0.847376036	-1.620711231	3.35922E-10
AT2G38250	AT2G38250	1.265312891	0.534126665	1.359751319	0.198779171	2.279331025	0.059922473	-0.898270794	0.629389168	2.279331025	0.059922473	-1.623979052	0.001433841
AT3G02610	AT3G02610	1.323181948	0.673720177	-1.535071105	0.562969209	0.850710697	0.139470854	-0.20528521	0.968021479	0.850710697	0.139470854	-1.625213609	0.001146126
AT2G23620	MES1	-0.435151004	0.926254105	0.148211346	0.982686899	-5.568568449	0.225043153	-1.890681917	0.748808009	-5.568568449	0.225043153	-1.626037231	0.002930188
AT2G45830	DTA2	0.21703241	0.814692423	0.42114871	0.26852642	-0.139652756	0.709467638	-0.120189002	0.953045007	-0.139652756	0.709467638	-1.629121115	4.42309E-18
AT5G09978	PEP7			-6.038288106	0.140979014	4.327246017	0.426259265	-2.241652577	0.613392082	4.327246017	0.426259265	-1.630418464	0.020392393
AT2G22920	SCPL12	1.45394642	0.42620775	-1.706124013	0.404366736	-0.064435945	0.982790389	-1.048768197	0.808272271	-0.064435945	0.982790389	-1.632367048	3.57569E-14
AT3G13405	ath-MIR169a	0.178129967	0.97763751	5.463779989	0.124399338	0.501494398	0.862438588	-0.583038646	0.781380195	0.501494398	0.862438588	-1.63349542	0.00102322
AT1G68360	AT1G68360	1.796342182	0.065604615	0.351065534	0.886220172	0.807366616	0.234910598	-0.925834825	0.653026084	0.807366616	0.234910598	-1.633886505	6.32935E-12
AT2G30080	ZIP6	0.188031462	0.885975526	1.330741484	0.44601825	0.153535239	0.91027056	-0.25669425	0.899191689	0.153535239	0.91027056	-1.634555427	0.001366878
AT5G07475	AT5G07475	2.071306507	0.622004875	-6.480938609	0.156699506	-2.250666116	0.496936921	-1.927506049	0.633166756	-2.250666116	0.496936921	-1.636950715	2.47499E-09
AT5G13900	AT5G13900	5.825050004	0.303454156	-0.314257087	0.942851733	1.667599512	0.514365909	-2.685104815	0.479363164	1.667599512	0.514365909	-1.638159746	5.78573E-06
AT2G07806	AT2G07806	0.681076402	0.912375985	3.880965737	0.443232951	-0.500381681	0.502477602	1.163491992	0.758972478	-0.500381681	0.502477602	-1.638322949	0.000240664
AT3G04010	AT3G04010	0.162726156	0.897048429	0.633647676	0.378896663	-0.526671917	0.602594608	-0.367504472	0.825077044	-0.526671917	0.602594608	-1.639378709	0.000294517
AT3G16430	JAL31	0.090719328	0.911297746	-0.619417278	0.04734211	-0.870380882	0.175690662	-0.845247711	0.576396288	-0.870380882	0.175690662	-1.642182425	7.15942E-20
AT1G02220	NAC003	2.681859684	0.093139567	0.423308479	0.715581138	0.890811306	0.681152327	-0.171028258	0.981525175	0.890811306	0.681152327	-1.642284186	0.001863699
AT1G52880	NAM	0.246238894	0.82645511	0.331171284	0.680507097	1.201392598	0.070483337	-0.330977031	0.910686362	1.201392598	0.070483337	-1.649477812	4.28691E-05
AT2G38940	PHT1;4	-0.071546792	0.953506507	-1.282695064	0.215241292	-0.850947654	0.419578731	-0.903945559	0.747920884	-0.850947654	0.419578731	-1.65615964	4.93307E-10
AT3G09490	AT3G09490	1.813476531	0.322272931	0.117047643	0.965332241	-1.137990495	0.535162474	-0.865592883	0.854866009	-1.137990495	0.535162474	-1.65622596	1.88626E-05
AT3G52535	AT3G52535	1.0323984	0.328189259	1.394755204	0.210177793	-0.843680807	0.775632982	-0.112984939	0.982734229	-0.843680807	0.775632982	-1.657307514	0.003485655
AT4G16600	PGSIP8	6.854575639	0.140423043			-2.242707603	NA	-7.26152855	0.420106526	-2.242707603	NA	-1.658715179	0.047731902

AT1G69810	WRKY36	3.538834463	0.093826459	0.530524356	0.850225011	-2.271845638	0.256350275	-1.946780919	0.62738272	-2.271845638	0.256350275	-1.659318501	6.57321E-05
AT4G10250	ATHSP22.0	2.96416472	0.313013942	-0.233130804	0.972493789	5.883768284	0.138493746	-0.471653995	0.942622854	5.883768284	0.138493746	-1.660334746	0.000382396
AT5G43150	AT5G43150	0.208324482	0.819892089	0.730333676	0.114119462	0.796398928	0.119784139	-0.169504651	0.937367978	0.796398928	0.119784139	-1.66705784	1.65552E-07
AT3G51540	AT3G51540	1.79532903	0.406499625	1.379555436	0.337071848	1.688173756	0.423329778	0.238874869	0.976183443	1.688173756	0.423329778	-1.670480046	0.020032227
AT5G53320	AT5G53320	2.28625649	0.17813659	-0.984061139	0.420945033	-0.599264997	0.68106486	-1.057884481	0.809502311	-0.599264997	0.68106486	-1.67205328	0.000285778
AT1G10220	AT1G10220	1.522994028	0.714523161	0.850551126	0.877776907	1.222487313	0.708927277	-0.327564345	0.943927962	1.222487313	0.708927277	-1.674226021	0.020865913
AT5G42500	AT5G42500	6.117899554	0.277197367	3.202363031	0.573580997	1.463267847	0.780337674	-2.689742527	0.455598103	1.463267847	0.780337674	-1.676719551	0.000448938
AT4G25850	ORP4B	1.044990738	0.885761645	-5.664782145	0.286820194	1.218723311	0.502774472	-2.399449636	0.66524984	1.218723311	0.502774472	-1.680102411	0.000962195
AT3G57950	AT3G57950	3.614015253	0.409922778	3.645907156	NA			-0.977833104	0.871355456			-1.680134701	0.043231626
AT4G24130	AT4G24130	0.531321503	0.646137389	0.584353734	0.665671364	-0.668581143	0.735558652	-1.766349693	0.659255524	-0.668581143	0.735558652	-1.681735336	2.41712E-06
AT4G01430	UMAMIT29	0.961743929	0.327996753	0.514886407	0.532853963	0.737345863	0.391429502	-0.875129824	0.917296618	0.737345863	0.391429502	-1.684637611	1.02716E-41
AT4G22610	AT4G22610	5.590008925	0.309859202	-2.139964672	NA	-5.11999756	0.248076405	-2.712316974	0.44862491	-5.11999756	0.248076405	-1.688037493	0.000646153
AT1G73410	MYB54	2.998045054	0.487227793	-6.400485531	0.172280228	1.519704702	0.620275696	-0.85228572	0.876673849	1.519704702	0.620275696	-1.688652686	0.02102992
AT5G39090	AT5G39090	0.121664215	0.970026242	0.311419566	0.892180865	0.015980486	0.996095544	-0.479952361	0.967195835	0.015980486	0.996095544	-1.692099867	0.000386031
AT3G59690	IQD13	0.541257413	0.850291735	-1.021139777	0.849702366	0.270663289	0.866465719	-1.09838123	0.447191235	0.270663289	0.866465719	-1.6970156	0.001976715
AT5G16230	AT5G16230	1.024043958	0.669452983	-1.014451377	0.803159356	0.417988518	0.874254995	-0.918649145	0.830627308	0.417988518	0.874254995	-1.697836681	0.000131104
AT2G07785	AT2G07785	-0.766937299	NA	6.057469574	0.15700558	2.322991727	0.144339918	-0.721041513	NA	2.322991727	0.144339918	-1.698151868	0.022803944
AT2G38465	AT2G38465	1.607301154	0.086118052	1.545908714	0.064838176	0.997031278	0.239749753	-0.615357526	0.784493718	0.997031278	0.239749753	-1.699843746	0.000773422
AT3G42180	AT3G42180	-1.511951598	0.757225047	3.787858366	0.355478651	2.170897791	0.2152622	-1.259193302	0.770575751	2.170897791	0.2152622	-1.700431056	4.32153E-06
AT3G04370	PDLP4	6.584496499	0.092837302	-1.218072165	0.840111964	4.487297152	0.407055313	-1.396617464	0.732947801	4.487297152	0.407055313	-1.703077102	0.002858873
AT3G04220	AT3G04220	0.481217414	0.936523541	0.2224526	0.965487183	0.799670889	0.655610473	-0.165544181	0.982623916	0.799670889	0.655610473	-1.704391077	0.02108455
AT4G36610	AT4G36610	1.660165073	0.489150682	0.298601248	0.939992726	1.014788011	NA	-1.177937135	0.782181302	1.014788011	NA	-1.707545736	1.42984E-08
AT2G38500	AT2G38500	1.819776179	0.052458639	-0.238403044	0.89806042	0.919415468	0.348392681	-0.134322359	0.960890507	0.919415468	0.348392681	-1.709096695	0.000122922
AT2G16990	AT2G16990	0.394281985	0.599896224	0.88648147	0.144086689	-0.001128331	0.999411864	0.065063851	0.962872831	-0.001128331	0.999411864	-1.709464722	6.656E-15
AT1G49000	AT1G49000	0.860699576	0.287591494	-0.770362226	0.475369829	-1.361900179	0.408145617	0.409764877	0.910564013	-1.361900179	0.408145617	-1.724053779	8.25455E-05
AT4G20240	CYP71A27	-1.061179922	0.609587039	-3.737930167	0.174349573	-2.172278198	0.32831486	-2.239529664	0.576334999	-2.172278198	0.32831486	-1.724950496	5.95215E-13
AT5G66630	DAR5	1.613279482	0.087187848	0.669616909	0.616592061	1.319814169	0.310625031	-0.292985706	0.871661081	1.319814169	0.310625031	-1.729044376	3.41267E-07
AT2G44480	BGLU17	0.696541453	0.654772236	0.000874828	0.999862639	-0.679721422	0.866911366	-0.049819998	0.988471189	-0.679721422	0.866911366	-1.729688385	0.001609024
AT3G47290	PLC8	-0.971661621	0.000640942	-0.486446999	0.058719518	3.82334834	0.329164858	0.624023776	0.563758673	3.82334834	0.329164858	-1.730870295	0.011611844
AT1G54540	AT1G54540	2.46605138	0.603060872	2.545517042	0.517897177	1.20033446	0.746723019	-1.168668684	0.735512818	1.20033446	0.746723019	-1.732116783	2.23854E-07
AT3G01990	ACR6	-0.450174544	0.799720527	-0.408445568	0.728652089	-0.445689296	0.644703525	0.141562364	0.989159859	-0.445689296	0.644703525	-1.732212804	0.027380448
AT2G29995	AT2G29995	0.153990442	0.871249116	0.035684743	0.972062223	-0.644913022	0.354660392	-1.108295889	0.314063466	-0.644913022	0.354660392	-1.732489379	0.000117743
AT1G68850	AT1G68850	2.864711117	0.229241569	0.141475227	0.953923225	1.685789529	0.620379221	-2.919705063	0.354109371	1.685789529	0.620379221	-1.733072817	1.29527E-09
AT1G63245	CLE14	5.674180352	0.317451005	5.987353392	0.258007358			-1.27545815	0.792554498			-1.734143513	0.000963444
AT5G58860	CYP86A1	2.740277192	0.062695378	-2.368266502	0.13316505	1.010739324	NA	-2.026363379	0.709813898	1.010739324	NA	-1.741159594	4.05885E-38
AT1G29000	AT1G29000	0.971995752	0.884590287			-3.967003132	0.468268694	-0.835876671	0.895706838	-3.967003132	0.468268694	-1.75018825	0.041310739
AT5G15180	AT5G15180	1.821677074	0.59075794	2.318368555	0.110177548	-1.019663624	0.718533694	-5.143410261	0.128407019	-1.019663624	0.718533694	-1.751000422	0.003185732
AT2G38210	PDX1L4	0.476212191	0.624563564	1.010887635	0.087478397	0.586453853	0.119350139	1.955837555	0.696184854	0.586453853	0.119350139	-1.752171645	0.000950865
AT5G19810	AT5G19810	-1.258836846	0.636502454	-1.195074346	0.746264992	-0.832506789	0.894311921	-2.287368562	0.577728436	-0.832506789	0.894311921	-1.75604259	0.000241277
AT1G17190	GSTU26	0.097946757	0.915575321	0.214536704	0.880899505	0.206359781	0.817231208	-0.569942704	0.663046749	0.206359781	0.817231208	-1.758307679	6.85313E-13
AT3G49570	LSU3	-0.214348627	0.962387552	0.746640949	NA	-0.472887568	0.877883818	-1.14550342	0.753910599	-0.472887568	0.877883818	-1.767882984	0.008044832
AT3G27070	TOM20-1	-1.0043554	0.840205355					-1.758763877	0.782088381			-1.773250376	0.021765721
AT2G43535	AT2G43535	1.373051262	0.120681877	-1.375119004	0.052702605	-0.61151811	0.519199354	-0.507220023	0.774188277	-0.61151811	0.519199354	-1.791609054	2.35153E-06
AT1G08650	PPCK1	0.67346542	0.470221926	0.305045793	0.704197019	-0.747552875	0.065374171	-1.105402147	0.576396288	-0.747552875	0.065374171	-1.79320398	7.65706E-48
AT5G15900	TBL19	6.907905887	0.208308555	-4.729754511	NA	-1.350479627	0.810166199	-1.951503142	0.685495817	-1.350479627	0.810166199	-1.797905577	0.010101536
AT1G74458	AT1G74458	1.573145517	0.112946699	1.213020733	0.228112296	0.198842948	0.768186777	-0.258961603	0.818515078	0.198842948	0.768186777	-1.79990005	8.49553E-23
AT1G20700	WOX14	3.659606541	0.096711235	-1.388551761	0.563380853	0.366037392	0.886545113	-1.170980152	0.597380545	0.366037392	0.886545113	-1.803840737	5.49384E-07
AT4G28085	AT4G28085	-0.869199981	0.19609684	-0.036338149	0.980651529	-0.216179932	0.827938009	-0.291838392	0.938659772	-0.216179932	0.827938009	-1.807246542	2.09311E-05
AT5G08250	AT5G08250	6.642419826	0.185841493	-4.748600036	NA	0.217938852	0.961551614	-0.856383817	0.930511033	0.217938852	0.961551614	-1.810774777	0.002765922
AT2G04795	AT2G04795	0.53679765	0.572635073	0.134128974	0.891965028	-0.983987298	0.256568967	-1.158575658	0.553730067	-0.983987298	0.256568967	-1.811166869	0.001687522
AT3G56620	UMAMIT10	0.519642669	0.807513703	0.30927707	0.905905441	0.978243775	0.478215583	-1.994707083	0.62738272	0.978243775	0.478215583	-1.818978558	1.22391E-06
AT2G07676	AT2G07676	3.941445652	NA			-0.734837868	0.888196984	-3.710612614	NA	-0.734837868	0.888196984	-1.819264335	0.021899005
AT2G34940	VSR5	2.79358914	0.656174374	-6.841532889	0.103775285	-2.203184798	0.580822462	-1.862121404	0.665832077	-2.203184798	0.580822462	-1.820796604	4.15987E-05
AT1G72660	AT1G72660	0.463004709	0.915223154	1.148273509	0.744372328	2.68293005	0.287615276	-5.992023302	0.620104018	2.68293005	0.287615276	-1.827314304	0.02951164
AT1G48745	AT1G48745	-0.640817949	0.66204525	-1.045585401	0.266382192	-0.879886343	0.399689445	-0.869982683	0.653363031	-0.879886343	0.399689445	-1.833894596	1.94164E-07
AT1G76952	IDL5	1.295160973	0.7285476	-4.157820264	0.056916002	0.319919054	0.927546821	-3.151355875	0.604422229	0.319919054	0.927546821	-1.835327128	9.47969E-06
AT5G67450	ZF1	-1.305337607	0.242379599	-0.689939424	0.786764565	0.704770159	0.357298514	-1.522399557	0.55451616	0.704770159	0.357298514	-1.838428731	0.019003036
AT4G13290	CYP71A19	2.120212934	0.687385648	-3.875540153	NA	-5.892840378	0.07463631	-0.201084084	0.962474985	-5.892840378	0.07463631	-1.84649518	1.38474E-16
AT2G26290	ARSK1	-0.28646042	0.947813399	0.345302117	0.941640409	-4.288429215	0.430581617	-3.75649759	0.151842669	-4.288429215	0.430581617	-1.84704262	0.007128812
AT3G50350	AT3G50350	0.187421641	0.900353824	-1.12879293	0.058511522	-0.893835709	0.373775334	-1.396411634	0.716554635	-0.893835709	0.373775334	-1.850374418	2.07726E-06
AT5G22550	AT5G22550	-0.0762437	0.985508803	-0.950364998	0.724521593	-1.334749364	0.709467638	-0.756470202	0.674100217	-1.334749364	0.709467638	-1.862965276	7.11519E-05
AT5G59270	AT5G59270	6.531885743	0.09471376	-3.723608976	NA			-3.619203917	NA			-1.870670869	0.043259423
AT5G62260	AT5G62260	0.39772322	0.647765198	0.197273598	0.825077244	0.230981392	0.694088018	-0.545789013	0.626700633	0.230981392	0.694088018	-1.87561731	2.59765E-26
AT1G62710	BETA-VPE	-0.801085223	0.440849604	0.848340886	0.484518204	0.176343	0.904006245	-0.390353881	0.809825068	0.176343	0.904006245	-1.886710203	5.19403E-11
AT2G38790	AT2G38790					-0.616081761	0.29148125			-0.616081761	0.29148125	-1.886998103	1.70693E-06
AT2G14210	AGL44	4.815730707	0.119105485	1.560931959	0.327216105	0.253307516	0.431964911	-1.269049737	0.463297446	0.253307516	0.431964911	-1.88803152	4.80324E-17
AT5G19410	ABCG23	0.802177964	0.775785808	7.284778877	0.061147437	0.353325219	0.92906786	-1.673459771	0.693517591	0.353325219	0.92906786	-1.893191963	9.36788E-08
AT1G58280	AT1G58280	-1.217379697	0.407043574	-2.495214471	0.273183666	-0.894608732	0.484601477	-0.419318573	0.831610681	-0.894608732	0.484601477	-1.896126669	2.4106E-09
AT1G53990	GLIP3	2.428769969	0.385369171	-5.037474709	0.349603014	-5.233269921	0.308623268	-0.707166709	0.74822145	-5.233269921	0.308623268	-1.899632363	0.005888726
AT1G53540	AT1G53540	0.723671647	0.648230441	1.032024659	0.668402154	3.359110715	0.279687394	-7.099326352	0.457206007	3.359110715	0.279687394	-1.899774538	0.042701817
AT4G17215	AT4G17215	1.525142224	0.231230286	-0.079250444	0.96430604	-0.204081978	0.8978285	-1.869398955	0.648400166	-0.204081978	0.8978285	-1.902130109	9.00047E-12
AT4G10500	AT4G10500	0.920163354	0.727581979	3.563730404	NA	-0.175158948	0.960445737	-2.072731592	0.616300683	-0.175158948	0.960445737	-1.907501557	0.037624154
AT3G07195	AT3G07195	-0.995723543	0.502323387	-1.473977416	0.577487316	-0.501867339	0.901283074	-4.407915988	0.107090587	-0.501867339	0.901283074	-1.910777973	0.005238275
AT4G29800	PLP8	0.773869685	0.592213477	-0.867483742	0.82973778	-1.269099827	0.698474836	-1.918903803	0.66524984	-1.269099827	0.698474836	-1.91411347	4.13582E-08
AT4G21215	AT4G21215	-0.346048026	0.530443243	-0.460654211	0.270317882	-0.428519393	0.377693101	-0.37039657	0.754260213	-0.428519393	0.377693101	-1.914154651	5.0556E-26
AT5G43980	PDLP1	2.089585588	0.250122297	-0.378176262	0.876240776	0.484097757	0.83115322	-1.424529162	0.726553713	0.484097757	0.83115322	-1.925148423	2.55243E-05
AT1G51990	AT1G51990							-6.786311262	0.210076168			-1.928299602	0.010982843
AT1G10300	AT1G10300	0.560575766	0.860806431	-0.532346674	0.907826886	-0.024118466	0.995492744	-0.496045779	0.965260868	-0.024118466	0.995492744	-1.928349094	0.004052712
AT1G65850	AT1G65850	7.160290721	0.098805924	0.548866912	0.896064817	0.21829378	0.957312846	-4.027483761	0.498460747	0.21829378	0.957312846	-1.928855692	0.007372295
AT4G23680	AT4G23680	0.043266405	0.960197883	0.506982955	0.046965343	-0.826668371	0.000151919	-0.739745503	0.706266671	-0.826668371	0.000151919	-1.930358673	5.90198E-07
AT1G55940	CYP708A1	6.15681133	0.27341374	-3.145297424	NA	0.973256898	0.819336861	-5.102387321	0.589889774	0.973256898	0.819336861	-1.941607078	5.95462E-09
AT4G15330	CYP705A1	1.747742792	0.351453231	0.133281688	0.961956969	-0.03331971	0.992604378	-1.170164937	0.777104408	-0.03331971	0.992604378	-1.947004711	7.10234E-06
AT1G48800	AT1G48800			-4.460281122	NA	5.888949012	0.238041347	-6.31977838	0.604422229	5.888949012	0.238041347	-1.94975814	0.000286375
AT2G34000	AT2G34000	2.748358709	0.39552381	-5.616925467	0.291017228	-0.072315181	0.987023041	-0.353221123	0.962474985	-0.072315181	0.987023041	-1.953415121	9.10641E-06
AT5G51160	AT5G51160	2.481462005	0.508663075	3.434727842	0.081958987	2.460974963	0.491732111	-2.417920683	0.707962062	2.460974963	0.491732111	-1.961705717	0.000315279
AT2G01880	PAP7	5.494090109	0.334175913	-2.876163836	NA	-3.102511487	NA	-3.012480165	0.314063466	-3.102511487	NA	-1.9620068	0.007600744
AT1G14520	MIOX1	-0.927913993	0.09928281	-0.174324532	0.715756131	0.584878855	0.082264167	1.655758187	0.647947756	0.584878855	0.082264167	-1.96491167	0.000551377
AT5G55590	QRT1	1.862734731	0.571335518	0.097402985	0.974533195	-1.152064067	0.619043918	-3.217447161	0.600251767	-1.152064067	0.619043918	-1.967219935	0.002328181
AT4G31875	AT4G31875					-0.078475553	0.94503851			-0.078475553	0.94503851	-1.969437762	3.88605E-05
AT5G53390	FOP1	-1.32992794	0.718576709	0.397650496	0.951724881	-1.575031901	0.720809329	6.849989007	0.575257546	-1.575031901	0.720809329	-1.976436116	0.0045265
AT3G15300	AT3G15300	1.420023659	NA	1.965219554	0.245509253	-0.894117476	0.503870596	-1.29552336	0.558178127	-0.894117476	0.503870596	-1.979454468	1.19634E-11
AT2G35150	EXL7	1.024621464	0.834507102	3.883702712	NA	-0.192770894	0.952606907	-0.55877665	0.914147923	-0.192770894	0.952606907	-1.998952209	1.13906E-11
AT5G54190	PORA	-1.244604554	0.158103585	-0.779748179	0.682977985	-0.004454828	0.99788829	-0.505586822	0.742017486	-0.004454828	0.99788829	-1.999006808	2.33786E-21
AT3G55900	AT3G55900	-3.414453352	NA	3.728776902	NA	-0.647724736	0.861229132	-5.547918477	0.639605292	-0.647724736	0.861229132	-2.024402065	0.02699935

AT1G13420	ST4B	5.32487491	0.350695972	5.624517123	0.290775174	-1.396660876	0.67632096	-4.459180935	0.219949828	-1.396660876	0.67632096	-2.036171427	0.000537623
AT5G65030	AT5G65030	0.550339028	0.88867489	-0.366814662	0.808204607	0.449516337	0.670735957	-2.953900016	0.366253257	0.449516337	0.670735957	-2.037076693	1.52107E-13
AT1G48300	DGAT3	0.769030343	0.025092276	0.677273654	0.006365126	-0.147840154	0.497312078	0.178776722	0.909213796	-0.147840154	0.497312078	-2.047756962	7.14993E-75
AT5G10140	FLC	0.784194379	0.738786239	0.404435588	0.7954656	0.586081786	0.356750015	0.790375833	0.672844426	0.586081786	0.356750015	-2.05420663	0.002831605
AT4G14250	AT4G14250					0.103651732	0.972965179			0.103651732	0.972965179	-2.058709852	0.006027337
AT2G05540	AT2G05540	0.747880347	0.101750679	0.421555192	0.195710652	0.117336028	0.715769713	0.397290104	0.820186281	0.117336028	0.715769713	-2.059650832	0.000620405
AT2G43670	AT2G43670	3.359908631	0.22047313	0.213772454	0.96073843	-5.827713043	0.087877848	-1.128221105	0.885840009	-5.827713043	0.087877848	-2.060238449	2.91349E-10
AT5G44160	NUC	0.446590668	0.733737654	-0.111802594	0.962953586	-0.391608277	0.839994437	-1.799074964	0.653026084	-0.391608277	0.839994437	-2.06161328	4.90649E-10
AT4G28350	AT4G28350	0.024640191	0.984620075	-0.860026086	0.572862245	-1.916597687	0.303838003	-0.208340052	0.967187969	-1.916597687	0.303838003	-2.06169407	0.002834553
AT2G12646	AT2G12646	-0.073632428	NA	-6.405291921	0.17082959	-1.773705917	0.665380398	-0.368046739	0.964169173	-1.773705917	0.665380398	-2.06694732	0.003985995
AT1G03410	2A6	0.540029846	0.562603802	0.686936806	0.323442741	-0.068064358	0.940683427	0.76297761	0.776796299	-0.068064358	0.940683427	-2.08001918	0.023276998
AT4G16620	UMAMIT8	1.619371432	0.654577336	1.639807201	0.478778325	0.100277514	0.973922292	-3.394115753	0.542342434	0.100277514	0.973922292	-2.081559643	0.0011658
AT2G47360	AT2G47360	0.688809525	0.898892011	-1.404031484	0.713151836	1.218539942	0.773977548	-2.077371192	0.641679337	1.218539942	0.773977548	-2.084335192	0.00121829
AT1G03700	AT1G03700	2.783675296	0.324375973	0.6601253	0.919993643	-1.183751244	0.743246346	-4.435974464	0.306029459	-1.183751244	0.743246346	-2.084550782	0.004557658
AT2G40370	LAC5	1.625015725	NA	-1.912112217	0.553456971	-6.496145248	0.057798078	-1.337176219	0.735038565	-6.496145248	0.057798078	-2.090014959	8.35958E-11
AT5G10720	HK5	1.022050759	0.726242967	-5.442710858	0.128857184	-0.879504695	0.766814842	-0.739672778	0.892623197	-0.879504695	0.766814842	-2.091245807	3.73085E-09
AT5G13580	ABCG6	2.57269703	0.120915739	1.466884129	0.158097377	1.155312124	0.396444135	-1.576694213	0.685495817	1.155312124	0.396444135	-2.09532949	2.69171E-13
AT2G14960	GH3.1	-0.19592812	0.975054583	-5.753470471	0.278137081	-4.944806133	0.356286076	-3.490532041	0.20920389	-4.944806133	0.356286076	-2.108880606	0.004996413
AT2G30540	AT2G30540	-0.71557244	0.211330527	-0.112231442	0.849145367	-0.167206403	0.832711258	0.054601127	0.972043985	-0.167206403	0.832711258	-2.109568726	6.8482E-19
AT2G36090	AT2G36090	2.090754641	0.444072803	2.794335567	0.284305429	1.168215535	0.627255484	-0.839121852	0.871661081	1.168215535	0.627255484	-2.116193361	0.000806366
AT2G25980	AT2G25980	4.60125441	0.179332926	-2.68729924	0.246868606	-0.774621053	0.849887066	0.011904116	0.998326123	-0.774621053	0.849887066	-2.117731949	9.2523E-05
AT5G14160	AT5G14160	7.358273544	0.067454617	0.406472908	0.950818885	1.186301112	0.523394871	-6.674389429	0.301149642	1.186301112	0.523394871	-2.123613501	1.31943E-05
AT5G43630	TZP	0.061814076	0.945980546	0.252343594	0.629547357	-0.534689165	0.047167882	0.458625058	0.809598664	-0.534689165	0.047167882	-2.125269106	8.22908E-16
AT2G42060	AT2G42060	3.00599394	0.064643541	1.962497821	0.66773607	-0.278532734	0.960171181	-2.263396188	0.598901706	-0.278532734	0.960171181	-2.136050899	4.94708E-08
AT2G30130	ASL5	5.135882068	NA	-3.176438033	NA	-5.090668499	0.340353148	-2.054011541	0.749436809	-5.090668499	0.340353148	-2.141086869	0.049192397
AT5G09520	PELPK2	2.763594851	0.285834753	1.901451502	0.579148152	0.796257862	0.844111841	-1.039472073	0.726900342	0.796257862	0.844111841	-2.141368612	5.91854E-11
AT1G48510	AT1G48510	5.716206608	0.313319667	0.927753058	0.887778904	-6.118055316	0.113988502	0.155826692	0.99077971	-6.118055316	0.113988502	-2.148296907	0.002575268
AT1G07900	LBD1	1.491328481	0.30770498	3.124957475	0.184413145	0.903896204	0.509452598	-1.513941257	0.822720536	0.903896204	0.509452598	-2.148425295	0.049887598
AT4G34320	AT4G34320	5.816864504	0.304351831	1.630576967	0.743989008			-3.444465809	0.586750174			-2.148841385	0.020834212
AT2G48080	AT2G48080	1.430459776	0.604447879	-0.523104704	0.916627065	-0.293755282	0.962700052	-2.571574031	0.56497814	-0.293755282	0.962700052	-2.152463123	0.016360414
AT1G03020	AT1G03020	1.609781419	0.514792858	1.192753677	0.641448353	1.92009134	0.284573888	-0.059177976	0.994744545	1.92009134	0.284573888	-2.155611246	2.18229E-05
AT5G22355	AT5G22355	1.961104959	0.770101828			2.118175999	0.070797374	-1.953284224	0.647947756	2.118175999	0.070797374	-2.157333562	2.10332E-08
AT3G45070	AT3G45070	1.851621743	0.621488074	-4.900605911	NA	6.039647689	NA	-2.579929752	0.509467924	6.039647689	NA	-2.158465928	7.20264E-21
AT1G23200	AT1G23200	-0.973308726	0.509297031	0.606522344	0.644171283	-1.030586758	0.594455762	-5.177080277	0.056735704	-1.030586758	0.594455762	-2.161381986	0.017897312
AT5G44410	AT5G44410	0.14225647	0.878809942	-0.566163784	0.304106928	-0.325383927	0.441997917	-0.685282358	0.616300683	-0.325383927	0.441997917	-2.180518748	6.28174E-27
AT5G02780	GSTL1	6.492071439	0.102676764	3.409122713	0.168133237	-3.249958316	0.236294604	-0.815018973	0.89418557	-3.249958316	0.236294604	-2.184189041	1.95981E-05
AT4G40090	AGP3	1.330577528	0.850718081	-4.39926821	NA	1.124626917	0.769724528	-1.925605789	0.660852619	1.124626917	0.769724528	-2.188572843	0.009153122
AT5G47530	AT5G47530	6.79231989	0.154948398	0.708406028	0.887055461	-6.169267427	0.102614897	-2.311957056	0.575078848	-6.169267427	0.102614897	-2.193820033	2.89845E-05
AT1G01190	CYP78A8	2.046679458	0.278864512	0.582399557	0.63474492	-2.025946953	0.195108453	-1.785202859	0.653363031	-2.025946953	0.195108453	-2.197729414	9.21865E-26
AT5G58910	LAC16	3.718535445	NA	-3.591663003	NA			-2.111060403	0.83455438			-2.20173498	0.02992
AT3G44510	AT3G44510	6.115965848	0.179942101	-5.999034724	0.256525849	6.379914409	0.14311986	-2.69003568	0.446325688	6.379914409	0.14311986	-2.204345928	0.001476995
AT1G74080	MYB122	2.308461809	0.341639906	0.973634856	NA	-1.108056393	NA	-4.735013719	0.267160791	-1.108056393	NA	-2.20907449	9.65643E-05
AT3G13730	CYP90D1	0.022092104	0.982919296	-1.587951911	0.313522987	-0.070654483	0.919250378	-0.950011432	0.548822391	-0.070654483	0.919250378	-2.213708113	5.94432E-34
AT3G50260	CEJ1	0.953497747	0.072378506	0.396622877	0.639331373	0.0137521	0.988964218	-0.027946579	0.992836755	0.0137521	0.988964218	-2.218507447	5.13807E-11
AT5G62162	ath-MIR399c	-3.976212381	NA					-1.661093393	0.914660637			-2.22559211	0.039614735
AT2G39310	JAL22	0.865546972	0.013577949	0.649542122	0.002927006	-0.999847838	0.192911456	-0.156032437	0.936447266	-0.999847838	0.192911456	-2.243912562	5.78442E-27
AT5G07780	FH19			4.840730368	0.298293889	-4.321639773	0.426712153	1.475795237	0.905084907	-4.321639773	0.426712153	-2.257795306	0.009421196
AT3G13760	AT3G13760	2.857090394	0.269644059	1.41347303	0.736335478	-2.188899123	0.608481618	-0.614390536	0.915928155	-2.188899123	0.608481618	-2.269475627	5.63703E-06
AT4G36830	HOS3-1	0.015329548	0.996054752	0.375097965	0.919061855	-1.775163953	0.325969012	-1.389722149	0.39603659	-1.775163953	0.325969012	-2.273206789	7.0591E-06
AT4G14060	AT4G14060	21.38978861	NA	-4.81730311	NA	-4.191543895	0.441624455	-3.459125271	0.210076168	-4.191543895	0.441624455	-2.273607427	1.67878E-10
AT5G25160	ZFP3	1.269840483	0.53216225	-2.0393356	0.229988047	-0.625693333	0.612962012	-1.557161461	0.235958598	-0.625693333	0.612962012	-2.27412208	8.64257E-13
AT2G39175	ath-MIR160a	3.187683546	0.371037397	3.417028736	0.552173048	-4.267891515	0.433150989	-2.96985776	0.721140128	-4.267891515	0.433150989	-2.27609332	0.039980123
AT5G44460	CML43	3.232285099	0.274398645	0.70657555	0.891965028	-5.89072561	0.155601251	-2.001021886	0.628226763	-5.89072561	0.155601251	-2.279403808	6.30621E-14
AT3G50300	AT3G50300	1.074168537	0.729136791	-6.166921043	0.109091107	2.313838602	0.645346033	-0.720968329	0.901814827	2.313838602	0.645346033	-2.288980563	8.81401E-06
AT4G28840	AT4G28840	4.084010252	0.277197367	-0.739628837	0.869996955	-1.819061744	0.33831475	0.056079019	0.994744545	-1.819061744	0.33831475	-2.292365434	0.012902449
AT1G07500	AT1G07500					0.987162527	0.456760918			0.987162527	0.456760918	-2.293946466	0.02613858
AT3G25510	AT3G25510	0.97029489	0.711087984	0.543453916	0.839739664	2.351437683	0.510053661	-1.248000377	0.763339794	2.351437683	0.510053661	-2.303508905	1.35856E-08
AT3G22560	AT3G22560	-0.044033856	0.993246068	-4.012984161	NA	3.643066726	0.213997218	-7.318146786	0.405880771	3.643066726	0.213997218	-2.304793746	0.031168179
AT1G15580	IAA5	-0.338018583	0.945249215	-0.624606076	0.875216039	-0.813111865	0.783735459	-2.323120254	0.743849982	-0.813111865	0.783735459	-2.320727808	0.001183687
AT3G23450	AT3G23450	-0.210081108	0.802981578	-0.348617098	0.713776932	-0.822958098	0.009896011	-1.343619947	0.186649778	-0.822958098	0.009896011	-2.326676151	0.016371469
AT1G48930	GH9C1	6.79258431	0.154384093	0.917116104	0.819549444	4.793278395	0.30198675	-3.436873746	0.608254089	4.793278395	0.30198675	-2.32952416	0.013718771
AT5G12020	HSP17.6II	-2.885825176	0.339653767	-0.450896132	0.92420763	2.076851771	0.608568624	-0.630674526	0.931438503	2.076851771	0.608568624	-2.330890605	0.019633731
AT2G07727	AT2G07727	-0.944988034	0.504514005	1.57803301	0.187353131	1.160813469	0.502681357	0.648344787	0.93556951	1.160813469	0.502681357	-2.333067409	0.032996802
AT1G47510	5PTASE11	1.202294592	0.397415516	1.151673149	0.568469345	2.5672153	0.237294576	-1.225573069	0.928957326	2.5672153	0.237294576	-2.338432639	0.040711032
AT1G02770	AT1G02770	2.689572525	0.347210496	0.974034508	0.774524918	0.303944147	0.887809879	-3.738294716	0.488518707	0.303944147	0.887809879	-2.339276839	0.017592739
AT3G29034	AT3G29034	2.413860942	0.15588064	1.515106582	0.591302608	0.539112729	0.836682346	-1.665938289	0.672844426	0.539112729	0.836682346	-2.378291855	0.003511615
AT1G49860	GSTF14	-0.139861159	0.962926866	-1.547758687	0.328230593	-2.544930294	0.205066579	-1.099646861	0.426001186	-2.544930294	0.205066579	-2.381442161	2.0798E-28
AT1G14160	AT1G14160	3.805040682	0.21690726	4.234115945	0.42798443	3.051873796	0.274293707	-0.152681174	0.982749202	3.051873796	0.274293707	-2.388653873	2.38585E-08
AT2G37440	AT2G37440	4.91824117	0.077496129	0.317866253	0.956463112	-5.562134211	0.290183248	-0.460666529	0.948761383	-5.562134211	0.290183248	-2.389502045	0.038579198
AT3G01513	AT3G01513	2.493414594	0.263291904	-5.85333053	0.192062635	4.51614788	0.186763529	-1.666888962	0.281217577	4.51614788	0.186763529	-2.396979429	0.000438223
AT5G01250	AT5G01250	4.14605586	0.060333855	0.365567693	0.92869238	-4.807286179	0.32616655	-1.580351418	0.684321129	-4.807286179	0.32616655	-2.41101394	1.25705E-09
AT1G77520	AT1G77520	3.755780164	NA	3.855042856	0.425240111	4.18118527	NA	-2.804967806	0.457226926	4.18118527	NA	-2.415802682	2.80096E-31
AT1G56550	RGXT3	0.659939039	0.666906032	1.665903538	0.420584319	0.080341858	0.958564787	-1.452160482	0.351647666	0.080341858	0.958564787	-2.415917291	0.000322599
AT4G21230	CRK27	-1.30537024	0.73832452	-0.285409447	0.941640409	1.35897607	0.616321561	-0.820864403	0.870396305	1.35897607	0.616321561	-2.420398881	2.5257E-05
AT1G69526	AT1G69526	0.803593929	0.489829724	-0.436981301	0.856039419	-1.337250195	0.518322499	-1.122063666	0.676109289	-1.337250195	0.518322499	-2.44634275	1.2117E-08
AT4G37700	AT4G37700	6.308485875	0.256133506	-0.295280969	0.955461212	21.30468868	NA	-1.547553533	0.430181703	21.30468868	NA	-2.453395345	7.78004E-09
AT2G29750	UGT71C1	2.274870545	0.544867985	0.139970629	0.977716544	0.629855398	0.862072102	-1.641738503	0.585524552	0.629855398	0.862072102	-2.461734053	2.89163E-10
AT2G16385	AT2G16385	-0.406481383	0.954478576	-5.580787657	0.294477469	-4.639852615	0.390313142	0.101366338	0.990915497	-4.639852615	0.390313142	-2.461765723	0.000231995
AT4G11320	AT4G11320	2.39369565	0.088949145	-0.241789248	0.724521593	-0.894935151	0.502044757	-0.801345692	0.723572313	-0.894935151	0.502044757	-2.463282259	3.1436E-141
AT5G07650	AT5G07650	-4.412805514	NA	3.214801678	NA	-1.214668245	0.755334978	-8.085627113	0.202694291	-1.214668245	0.755334978	-2.464879224	0.000210706
AT2G28690	AT2G28690	0.745546582	0.916622144			-4.191543895	0.441624455	-1.451714533	0.837492311	-4.191543895	0.441624455	-2.468630308	0.001146623
AT1G30840	PUP4	2.12435827	0.215080075	0.735596944	0.50695547	-0.15159333	0.952329558	-0.297622252	0.924803201	-0.15159333	0.952329558	-2.474114774	3.40333E-14
AT1G15460	BOR4	5.862887199	0.299731107			-3.585343201	0.516230268	-0.828993041	0.88581519	-3.585343201	0.516230268	-2.481197558	0.01794297
AT1G62370	AT1G62370	1.595515057	0.440223628	1.653628962	0.179956013	1.183453654	0.440105891	-0.847614452	0.865681813	1.183453654	0.440105891	-2.481711504	7.5438E-07
AT1G48500	JAZ4	4.082167704	NA	-1.69264907	0.576294616	-5.803133208	0.183580262	-0.265227045	0.971297857	-5.803133208	0.183580262	-2.494448331	8.02018E-09
AT2G02630	AT2G02630					-2.242707603	NA	-1.763121156	0.790516534	-2.242707603	NA	-2.514026071	2.64358E-05
AT3G15240	AT3G15240	2.727572685	0.171290702	-2.552276423	0.302206667	-1.404685688	0.636119277	0.05599619	0.980596414	-1.404685688	0.636119277	-2.514254733	2.42062E-10
AT2G01580	AT2G01580	5.531307555	0.323626618	5.775866864	0.276503596			-2.611222044	0.51671888			-2.515754839	7.90542E-22
AT5G60480	HB26	4.720374787	NA			1.930250756	0.675409067	0.721521491	0.950313494	1.930250756	0.675409067	-2.515918012	0.046304881
AT3G25240	AT3G25240	1.108548545	0.879443097	2.531098209	0.667443162	-1.739705102	NA	-0.949261469	0.946164167	-1.739705102	NA	-2.51743575	0.001875846
AT1G66690	AT1G66690	-0.227295839	0.949533165	-1.205468669	0.605562946	1.017363947	0.442274012	-0.470039089	0.950356778	1.017363947	0.442274012	-2.535300494	0.012212065
AT3G03776	AT3G03776	5.123943612	NA	-0.678611792	NA			-7.618474189	0.075261499			-2.544351918	0.021213208
AT1G66800	AT1G66800	-5.163096104	0.366000783	1.127618511	0.855355791	2.610024648	0.463169333	-1.446874298	0.073041314	2.610024648	0.463169333	-2.545299788	7.3672E-15
AT2G07687	AT2G07687	2.98339058	0.254180232	0.533441138	0.856762817	-0.577053032	0.819376477	-4.253637653	0.455327624	-0.577053032	0.819376477	-2.554016523	4.50939E-05

AT1G13530	AT1G13530	-0.063521925	0.950895391	0.540411574	0.331506393	0.05641471	0.925737546	2.269548837	0.454321515	0.05641471	0.925737546	-2.554812525	4.96013E-05
AT4G15417	RTL1	5.994498018	0.287876683	2.008515654	0.673325418	3.678151494	0.320489172	-2.902696111	0.62738272	3.678151494	0.320489172	-2.563950329	0.002382358
AT2G39380	EXO70H2					-0.045648373	0.985067551			-0.045648373	0.985067551	-2.56945461	2.35325E-17
AT5G49770	AT5G49770	1.718919553	0.693561703	-1.787982627	0.75840407	-5.946269823	0.147814301	-1.482891932	NA	-5.946269823	0.147814301	-2.583123391	0.005540472
AT3G02885	GASA5	-0.205344881	0.975954414	-4.253899481	NA	0.759006772	0.898947545	-2.774669719	0.429222482	0.759006772	0.898947545	-2.595717174	1.53479E-05
AT2G45430	AHL22	4.729913744	0.146868139	-0.827318839	0.359540701	-2.702592307	0.059351513	-0.847453502	0.914733185	-2.702592307	0.059351513	-2.604538286	1.28901E-16
AT1G57570	AT1G57570			2.634865925	0.631891775	0.876810222	0.874656815	-1.029076597	0.842538979	0.876810222	0.874656815	-2.612539066	0.017701354
AT3G46270	AT3G46270			-3.176438033	NA			-6.317590927	0.604422229			-2.630069918	6.58296E-05
AT4G19920	AT4G19920	0.550076962	0.943257647	-5.740496478	0.279334269	-3.844489737	0.374145316	-0.756147689	0.941415027	-3.844489737	0.374145316	-2.640070941	0.001134123
AT4G16350	CBL6	-0.752366138	0.868676576	-0.904069338	0.719160414	0.40325064	0.778981063	-0.645527564	0.91823517	0.40325064	0.778981063	-2.650176985	0.0045265
AT5G48010	THAS1	8.549320953	NA	-3.063117559	0.242645902	1.979183572	0.454715154	-1.873659778	0.750687341	1.979183572	0.454715154	-2.661290109	3.89747E-35
AT1G71380	CEL3	-0.248940631	0.951701269	0.862561271	0.734185476	-0.058816605	0.98947497	-1.219771851	0.76756257	-0.058816605	0.98947497	-2.668872884	1.13343E-09
AT5G04950	NAS1	0.268006339	0.917756537	-0.068740617	0.962928971	-1.943659519	0.215843135	-1.097283627	0.807156857	-1.943659519	0.215843135	-2.669081619	8.64146E-16
AT5G43370	PHT1;2	-0.582671062	0.917129549	1.462219702	0.79174311	-1.79653462	0.527610212	-3.216250068	0.179960645	-1.79653462	0.527610212	-2.677663403	5.76651E-13
AT4G28790	AT4G28790	1.977040854	0.462722587	-2.630052705	0.352032097	0.097688533	0.937338071	0.065434235	0.989622493	0.097688533	0.937338071	-2.680464824	0.00565201
AT4G02180	AT4G02180	-5.305080438	0.352365134	-4.654549559	NA			-1.95550354	0.794733222			-2.684513679	0.000691264
AT5G47950	AT5G47950	7.425016249	0.06229437	-1.295691145	0.734271857	1.086907163	0.819932848	-1.812037666	0.691824202	1.086907163	0.819932848	-2.684664686	2.18705E-13
AT1G09090	RBOHB	1.497056303	0.428690872	-2.93576623	0.252770277	-0.818894591	0.816417548	-1.298160889	0.574003303	-0.818894591	0.816417548	-2.697154093	7.74819E-18
AT5G38030	AT5G38030	0.183123648	0.954464638	-1.226919628	0.772897405	-0.060650579	0.990006831	-2.502302815	0.558566659	-0.060650579	0.990006831	-2.703618969	4.92942E-11
AT2G37740	ZFP10	0.826459401	0.782958333	-2.913252	NA	-2.648405744	NA	0.298824679	0.968467209	-2.648405744	NA	-2.704085834	0.004557658
AT4G04990	AT4G04990	1.64033617	0.462410873	-6.368892624	0.18194474	-4.999294797	0.350593053	-2.386259769	0.586991571	-4.999294797	0.350593053	-2.706030681	0.004406581
AT1G73340	AT1G73340	1.624558787	0.812411905	0.033443459	0.996373269	-1.912821807	0.645806593	-3.914698895	0.416170703	-1.912821807	0.645806593	-2.711361161	0.046328642
AT1G68825	RTFL15	6.289148632	0.262163509	-1.573044728	NA	-4.476651713	0.070951268	-0.634848035	0.95447214	-4.476651713	0.070951268	-2.721937543	0.006188291
AT1G73220	1-10月	3.370328742	0.358454074	-3.004141853	0.246993988	-2.350497866	0.453147742	-2.77313879	0.433558429	-2.350497866	0.453147742	-2.725067533	5.86499E-24
AT3G09240	BSK9	1.959098456	0.644890697			-4.288429215	0.430581617	-0.553111283	0.936758504	-4.288429215	0.430581617	-2.72722075	0.012518091
AT4G00700	AT4G00700	2.994713677	0.051226789	0.357792671	0.885086412	-1.338726899	0.598999162	-1.406842745	0.716289725	-1.338726899	0.598999162	-2.736617231	1.09157E-18
AT2G46850	AT2G46850	5.609419093	0.323552135	-3.138995476	NA			-5.263262385	0.11648478			-2.739567243	0.011348468
AT2G19610	AT2G19610	6.479527986	0.218731641	5.07524062	NA	0.469824994	0.901927967	-0.142913899	0.991271129	0.469824994	0.901927967	-2.739758602	0.007476801
AT3G03670	AT3G03670	5.862887199	0.299731107			-3.967003132	0.468268694	-1.826349235	NA	-3.967003132	0.468268694	-2.741135891	0.029835869
AT1G77530	AT1G77530	2.226458565	0.28783548	-0.935201835	0.397857477	-0.772104315	0.788336678	-2.007687387	0.709782366	-0.772104315	0.788336678	-2.742385639	2.3628E-82
AT1G63005	ath-MIR399b					-2.002730959	NA	-0.37168574	0.982356953	-2.002730959	NA	-2.752863523	0.034804736
AT5G45200	AT5G45200	5.199428406	0.362404064	1.40042522	0.78219284			-6.231591675	0.393926565			-2.754371661	0.000963625
AT3G16390	NSP3	2.272662974	0.410032421	0.263440101	0.954535121	-2.515307648	0.340595628	0.156470877	0.966082349	-2.515307648	0.340595628	-2.755785705	3.62991E-10
AT1G16150	WAKL4	0.91629536	0.833229279	0.460455028	0.885283845	-1.894212697	0.26429457	-3.222283833	0.619462504	-1.894212697	0.26429457	-2.756519784	0.002458071
AT1G11540	AT1G11540	2.061855201	0.262553422	2.281803221	0.192404272	-0.435282212	0.896215186	-1.845610987	0.326457456	-0.435282212	0.896215186	-2.763727333	0.003158238
AT3G09162	AT3G09162	-0.514764789	0.698287764	-0.122730666	0.912388641	0.845826436	0.760151916	-1.567859747	0.527892502	0.845826436	0.760151916	-2.763902289	0.00197073
AT2G43390	AT2G43390	2.715058697	NA	-4.460281122	NA	1.506534241	0.801082726	0.041998667	0.996799452	1.506534241	0.801082726	-2.776623036	1.9837E-05
AT3G15250	AT3G15250	-0.407064136	0.952823865	1.306099903	0.837394994			-0.397649679	0.972708988			-2.777082929	0.024069648
AT1G73010	PS2	-0.017348052	0.995810341	1.057191383	0.644113479	-0.817534395	0.843100235	-1.416559586	0.718982932	-0.817534395	0.843100235	-2.78158684	1.08991E-13
AT3G30350	RGF4	0.389205902	0.950488284	-4.640799755	NA			0.043463854	0.996693402			-2.782575853	0.004202111
AT3G23637	DVL21	6.698274018	0.068271101	3.051594267	0.551888255	1.33752493	0.767634108	-0.542404218	0.946164167	1.33752493	0.767634108	-2.784588074	0.007294021
AT3G29410	AT3G29410	1.388313184	NA	-4.90049564	NA	-5.302237453	0.293001769	-0.077592025	0.991559366	-5.302237453	0.293001769	-2.789451285	0.000922684
AT3G01175	AT3G01175	1.294352037	0.534816421	-0.163690092	0.950818885	1.756493599	0.464644883	-0.334198725	0.966082349	1.756493599	0.464644883	-2.792849902	0.037745433
AT5G26220	AT5G26220	-2.825233727	0.439649895	0.594694519	0.928424536	-2.056670058	0.49647718	-1.16951311	0.935898864	-2.056670058	0.49647718	-2.803944336	0.001764975
AT5G51060	RHD2	6.431758668	0.249218629	-0.325582613	0.936341854	-5.685316506	0.197923798	-0.527361881	0.818074063	-5.685316506	0.197923798	-2.806586627	1.0099E-09
AT1G36640	AT1G36640	6.076771629	0.280742775	-4.839925905	NA	6.074275681	0.106206113	-0.124073006	0.994744545	6.074275681	0.106206113	-2.808019641	0.028409557
AT1G21100	IGMT1	0.951027222	0.03996868	0.158434167	0.77511952	-0.640476451	0.122254525	-0.866946116	0.591266866	-0.640476451	0.122254525	-2.813870424	4.27716E-55
AT1G44160	AT1G44160	4.820171107	0.141532638	0.270070902	0.88842381	-0.906946073	0.567584591	-0.709614469	0.674523124	-0.906946073	0.567584591	-2.827983724	6.72932E-20
AT4G37050	PLP4	5.434034078	0.339653767	-3.145297424	NA	-1.839339907	0.713115744	-1.284827262	0.864045341	-1.839339907	0.713115744	-2.82873532	0.005135903
AT4G12090	AT4G12090	0.587205671	0.880303268	1.535527558	0.674198241	-0.078341428	0.980447125	-0.674469423	0.915334525	-0.078341428	0.980447125	-2.830312822	1.19152E-08
AT5G51930	AT5G51930					0.370232924	0.932175941	-6.294418439	0.606242129	0.370232924	0.932175941	-2.840373333	0.001814351
AT1G68450	PDE337	2.811366378	0.277197367	-0.537204888	0.896164643	-4.640933822	0.390295313	-1.634175643	0.685495817	-4.640933822	0.390295313	-2.8442311	0.002800457
AT5G09720	AT5G09720	3.454782108	NA	-6.17387882	0.229698607			-0.887930309	0.885844797			-2.85168312	0.012022092
AT2G40802	None					5.633722337	0.282658002			5.633722337	0.282658002	-2.859029447	0.029373329
AT2G34210	AT2G34210			-5.271326506	0.324654614			-1.086129042	0.847660958			-2.878271118	4.43452E-10
AT4G14390	AT4G14390	-3.395087684	NA	1.016563603	0.876249216	-4.169754161	0.444084641	-1.488976051	0.835120667	-4.169754161	0.444084641	-2.886747627	0.000695086
AT1G29179	AT1G29179			-3.398939027	NA	-1.739705102	NA	-6.660229213	0.269611673	-1.739705102	NA	-2.903181693	0.018762279
AT3G50400	AT3G50400	3.605175588	0.124329249	-1.289163089	0.840814152	-2.275905979	0.350881324	-1.170644571	0.66686842	-2.275905979	0.350881324	-2.915626733	4.56312E-17
AT4G00080	UNE11	7.432107737	0.067901436	-5.292500328	0.322601539	-2.58669937	NA	-3.275062208	0.317592245	-2.58669937	NA	-2.920842127	0.002039267
AT3G54450	AT3G54450	0.849185962	0.802981578			2.062024252	0.700505937	2.546728963	0.841434341	2.062024252	0.700505937	-2.921563028	0.025606528
AT3G55734	ath-MIR393b	-5.519670118	0.331609471	-4.913938244	0.363070633	0.884999384	0.731565121			0.884999384	0.731565121	-2.930352146	0.048620187
AT4G26380	AT4G26380	4.92599202	NA	-4.157764304	NA	-3.001110281	NA	0.568608735	0.94375132	-3.001110281	NA	-2.943181359	9.81999E-06
AT2G15220	AT2G15220	3.723792572	0.161607205	0.426057966	0.939095475	-0.146275713	NA	-1.93065142	0.114681201	-0.146275713	NA	-2.959056621	0.002928294
AT4G23070	RBL7	-1.272535107	0.668060194	-0.505515827	0.921890447	-1.417187283	0.646222228	-0.734378348	0.965471639	-1.417187283	0.646222228	-2.963181852	0.009754482
AT5G42930	AT5G42930	1.995418919	0.520331647	4.410580992	0.143544319	-1.739705102	NA	-1.132266433	0.785453357	-1.739705102	NA	-2.975324496	2.09704E-05
AT5G20860	AT5G20860	5.205109934	0.362108033			0.910275954	0.883309287	-1.385043361	0.877665269	0.910275954	0.883309287	-2.97813831	0.000734938
AT5G22555	AT5G22555	6.402011492	0.235711555	4.337962747	NA	-3.322614377	0.550728949	-5.970642786	0.621192363	-3.322614377	0.550728949	-2.986873523	0.002425361
AT2G02795	AT2G02795	4.835996915	NA					-4.191630297	0.709586436			-2.991416141	0.011636892
AT5G41905	ath-MIR166e	0.67682174	0.91847454			0.815496959	NA			0.815496959	NA	-3.011058953	0.023954739
AT3G51171	AT3G51171			6.277029195	0.232780479	-2.648405744	NA	-3.541010623	NA	-2.648405744	NA	-3.011437928	0.018942693
AT3G05950	AT3G05950	6.603252696	0.19072712	7.329469038	0.057134576			-2.230418464	0.606948021			-3.014388745	0.011679564
AT2G15390	FUT4	0.712327518	0.864561057	-0.23529327	0.898519452	0.711640631	0.771567025	-0.734973451	0.647947756	0.711640631	0.771567025	-3.030068464	0.002168632
AT5G42590	CYP71A16	21.05994189	NA	2.669160375	0.649049321	0.815496959	NA	-1.660404032	0.681538103	0.815496959	NA	-3.030625729	2.13875E-07
AT2G26410	Iqd4	7.404909871	0.060716554			-3.801136243	0.488928855	-6.910830049	0.1476628	-3.801136243	0.488928855	-3.038557629	0.018649836
AT2G33280	AT2G33280	3.120241915	0.273415459	1.476582939	NA	1.928420051	0.152637606	-2.026665716	0.632278424	1.928420051	0.152637606	-3.040064971	0.000645901
AT3G26610	AT3G26610	3.590872139	0.193424738	-5.225134874	0.329514817	-5.653602708	0.205795992	-3.398659523	0.173287506	-5.653602708	0.205795992	-3.093987763	0.000239815
AT4G21830	MSRB7	6.627239723	0.188458489	-5.636488978	0.289294256	-5.391436654	0.307512202	-1.305800188	0.343779816	-5.391436654	0.307512202	-3.098909164	0.000593531
AT5G27940	WPP3	6.142096672	0.274951793	0.459446416	0.903979301	1.065511057	0.733538095	-0.230359071	0.965260868	1.065511057	0.733538095	-3.101195782	0.014427035
AT5G03545	AT5G03545	3.756635553	NA	-0.147036317	0.872057314	1.133700252	0.071339556	-0.980482364	0.685495817	1.133700252	0.071339556	-3.102931644	1.35614E-85
AT3G48400	AT3G48400					0.967304932	0.814558543			0.967304932	0.814558543	-3.116023635	1.1223E-05
AT1G05577	AT1G05577	5.862887199	0.299731107	-5.784682353	0.176914218			-0.050845458	0.997285903			-3.124036211	0.02773602
AT3G12220	scpl16	6.399766605	0.249083981	-2.035959108	0.684574832	0.038368288	0.994398745	-1.459065985	0.74868355	0.038368288	0.994398745	-3.13541425	0.000144141
AT2G07835	AT2G07835	2.634676379	0.359383596	2.347640715	0.614433534	-1.405097038	0.631904616	-0.351577915	0.967291937	-1.405097038	0.631904616	-3.143450239	0.000207931
AT4G25790	AT4G25790	4.971105034	0.350831046	-5.07679138	0.345291973	3.051620108	0.567322035	0.391472264	0.963305035	3.051620108	0.567322035	-3.147637937	0.000329991
AT1G18100	E12A11	7.438000685	0.061440725	-0.64359811	0.921890447	2.137395609	0.51686753	-3.028295256	0.474740724	2.137395609	0.51686753	-3.152464577	0.00212837
AT5G47740	AT5G47740	0.889557105	0.757819775	2.600997052	NA	-3.026504045	0.444084641	-1.698251011	0.70850021	-3.026504045	0.444084641	-3.156527251	2.04943E-05
AT1G53635	AT1G53635	5.135882068	NA	-4.012984161	NA	-1.648514566	0.761303145	-0.082089355	0.996693402	-1.648514566	0.761303145	-3.179978834	0.012443978
AT5G38005	AT5G38005	2.492516541	0.159019446	0.103696769	0.940544048			0.912919556	0.644839236			-3.182366059	0.006935878
AT2G45610	AT2G45610	-1.952984445	0.502749714	-0.872027817	0.759730965	0.064593703	0.966774516	0.361731442	0.981893161	0.064593703	0.966774516	-3.20052088	4.2371E-05
AT2G05180	CYP705A6	-1.615724836	0.811239301	0.508939162	0.916037976	0.649276032	0.843960216	0.087465096	0.994162268	0.649276032	0.843960216	-3.205483929	0.001103066
AT1G51190	PLT2	5.458093227	0.337851772	-0.986063414	0.857732279	-5.086714879	0.340601224	-4.080785969	0.647947756	-5.086714879	0.340601224	-3.247059281	0.012002821
AT3G24542	AT3G24542			-2.591101112	NA	-0.364372186	0.923530212	6.194361035	0.611321113	-0.364372186	0.923530212	-3.24790981	0.032168208
AT3G46710	AT3G46710	7.473645672	0.060193583	-5.971002593	0.258892844			-1.604510994	0.854215164			-3.257130786	0.008891369
AT3G50010	AT3G50010	-0.513168273	0.860222186	0.624438607	0.794159442	-5.581314788	0.12288559	-0.974645244	0.833592548	-5.581314788	0.12288559	-3.269719561	0.000405064

AT4G28930	AT4G28930	6.259747351	0.264515926	-5.043587665	0.349003803			-5.592041754	0.637815859			-3.281741899	0.037697602
AT1G75600	AT1G75600	5.079689919	NA	5.93286931	0.262911307	1.721734279	0.630105941	0.114212863	0.993101238	1.721734279	0.630105941	-3.293061798	0.034123399
AT4G33720	AT4G33720	6.886143975	0.144198684	6.787448854	0.191844983	-3.001110281	NA	-2.84363616	0.128407019	-3.001110281	NA	-3.316826676	2.87907E-09
AT5G59260	AT5G59260	0.329264455	0.76577338	-1.556863862	0.403444296	-1.642272949	0.445351749	1.71593966	0.266872879	-1.642272949	0.445351749	-3.329187556	0.000226338
AT2G05400	AT2G05400	7.399189561	0.062483509	7.026669312	0.173380333			-3.955834794	0.430181703			-3.341175677	0.038299896
AT1G27990	AT1G27990	-0.735055696	0.921537141	2.664181926	0.342010884	8.404082034	0.081711526	-7.939958149	0.24122237	8.404082034	0.081711526	-3.344650638	0.011886512
AT1G01580	FRO2	1.778175519	0.612554593	-0.621011973	0.788933755	0.761494197	0.513198545	-1.417736027	0.543346863	0.761494197	0.513198545	-3.350116709	3.34611E-05
AT5G60530	AT5G60530	-0.776720921	NA	-21.89439578	NA	-4.584492934	0.396348235	-0.2392373	0.964169173	-4.584492934	0.396348235	-3.354279356	0.00306601
AT2G10606	ath-MIR396a			-3.913709836	NA			-5.160914455	0.659994339			-3.381424165	0.011772958
AT1G26700	MLO14							-5.673146337	0.56497814			-3.396810252	0.047291541
AT3G14510	AT3G14510	6.193782826	0.270083636					-1.182830814	0.93556951			-3.39932309	0.014275758
AT2G34315	AT2G34315	3.941445652	NA			2.413584717	0.667938661	-5.472295337	0.619462504	2.413584717	0.667938661	-3.401457417	2.49207E-11
AT5G39720	AIG2L	6.604396862	0.234213881	5.319381292	0.320503015	5.862969052	0.142408232	-0.835118215	0.915508397	5.862969052	0.142408232	-3.402294754	0.002889915
AT4G12270	AT4G12270	2.046455152	0.734788213			-3.75030794	0.14205352	-5.632275363	0.637614678	-3.75030794	0.14205352	-3.433931603	0.000170445
AT3G29430	AT3G29430					-2.353593325	NA	-6.446337223	0.598901706	-2.353593325	NA	-3.523290099	9.65397E-08
AT3G07900	AT3G07900	6.788856489	0.153312708	-4.679433448	NA	4.327246017	0.426259265	-1.03422423	0.841135253	4.327246017	0.426259265	-3.53482434	0.000777516
AT5G12340	AT5G12340	0.954375217	0.807473652	3.101289172	0.472911729			-2.444466464	0.616300683			-3.54004569	0.00052848
AT3G29250	SDR4	4.512660851	NA	0.696088053	0.806182502	0.368217942	0.923295491	-2.40716376	0.583897164	0.368217942	0.923295491	-3.544492658	0.004948166
AT2G02610	AT2G02610					-3.322614377	0.550728949	-2.793995954	0.574167509	-3.322614377	0.550728949	-3.546263348	0.001232384
AT3G47050	AT3G47050					-4.624622085	0.392044903	-0.316601634	0.983589298	-4.624622085	0.392044903	-3.553560498	0.000631553
AT2G46740	GulLO5	2.228690928	0.255236604	0.096108239	0.972837902	0.09258874	0.956772632	-2.346991912	0.586991747	0.09258874	0.956772632	-3.567724008	1.50312E-07
AT1G33320	AT1G33320			-3.398939027	NA			-2.426521131	0.820789421			-3.57622049	0.038788283
AT5G45210	AT5G45210	5.983744028	0.288813237	-6.993347355	0.077353913	-3.001110281	NA	-1.252187701	0.766322553	-3.001110281	NA	-3.590607648	1.01379E-11
AT3G46810	AT3G46810	6.321922104	0.258822134			5.927421728	0.229874733	-3.259861309	0.702246294	5.927421728	0.229874733	-3.590962424	0.004956004
AT3G32040	AT3G32040	5.737494695	0.311460473	-4.960462249	0.357608394	-5.77466538	0.17818682	0.518476915	0.973864665	-5.77466538	0.17818682	-3.595800667	6.96106E-05
AT1G20860	PHT1;8			7.04030049	0.172437789	-4.087945298	0.453957696	-1.695086337	NA	-4.087945298	0.453957696	-3.609321166	0.008022153
AT4G22230	AT4G22230	3.13179887	NA					-0.818783739	0.888695133			-3.612109701	1.14448E-06
AT4G31710	GLR2.4	-0.325209964	0.960085602	5.331024152	0.053143056	-5.096276001	0.339872118	-5.276132839	0.653297341	-5.096276001	0.339872118	-3.623467412	0.008385632
AT4G13680	AT4G13680	4.051965755	0.339653767	2.488167575	0.467437503			-0.514201932	0.945536059			-3.627858314	0.007212013
AT3G06460	AT3G06460	-4.774989952	NA	-6.280964412	0.232239446	-0.383140241	0.953563665	-3.694702642	0.561207188	-0.383140241	0.953563665	-3.638801901	0.018596287
AT2G30340	LBD13	6.766923253	0.165678859	-5.267203204	0.32516967	-3.807506537	0.488805567	-2.277374037	0.602119156	-3.807506537	0.488805567	-3.639063312	0.00058635
AT5G10130	AT5G10130			-5.635323376	0.289380783	-4.528109461	0.402261837	-6.685603035	0.250491408	-4.528109461	0.402261837	-3.672145861	0.001305968
AT1G73300	scpl2	4.08905745	0.19078764	-0.881078502	0.860152116	0.19949656	0.952578368	-1.280934369	0.751883565	0.19949656	0.952578368	-3.675245931	0.025349972
AT1G50930	AT1G50930	6.224094931	0.267696789	6.413182904	0.200322124	-3.442698025	0.535162474	0.604940773	0.962474985	-3.442698025	0.535162474	-3.682174315	0.004907132
AT1G36340	UBC31	6.586344124	0.200784064			-3.633033911	0.510150325	-5.852613494	0.62738272	-3.633033911	0.510150325	-3.724176939	0.018711217
AT4G30120	HMA3	3.13179887	NA	-5.517491964	0.300585163	-4.219740522	0.43915465	-6.813201141	0.532231419	-4.219740522	0.43915465	-3.730066566	0.001041756
AT5G50300	AZG2	-0.08014658	0.979851851	-0.560679214	0.914040617	-2.67693615	0.39119055	0.110793837	0.982734229	-2.67693615	0.39119055	-3.752919302	3.01772E-05
AT3G57157	AT3G57157	0.990600514	0.799747593	-2.599730583	0.270011963	-3.664862489	0.102485617	-3.928113581	0.246894084	-3.664862489	0.102485617	-3.759107532	1.13098E-16
AT2G25780	AT2G25780	2.153583568	0.419304711	0.157745012	0.939267702	0.08191128	0.942630597	0.769081707	0.874176467	0.08191128	0.942630597	-3.771440054	0.04624188
AT2G04025	RGF3			-3.736395706	NA			3.19987699	NA			-3.774116816	0.008214452
AT3G46500	AT3G46500	6.592835696	0.093351625	-6.11394414	0.237193677	-0.845103931	0.772881279	-2.217177458	0.626700633	-0.845103931	0.772881279	-3.776970378	3.79159E-07
AT2G14510	AT2G14510	4.063105725	0.098307754	1.14253811	0.799736406	-3.700980483	0.502044757	-0.19306576	0.981893161	-3.700980483	0.502044757	-3.777566818	0.000469708
AT5G55360	AT5G55360			-4.578670363	NA	-1.108056393	NA	-5.215625129	0.655579278	-1.108056393	NA	-3.788597689	0.038373262
AT2G31081	CLE4							-5.868683716	0.62738272			-3.805111156	0.0017943
AT3G48201	ath-MIR861							-5.29019228	0.652047062			-3.817360974	0.019729454
AT1G03106	AT1G03106	3.938118842	NA	1.60836611	NA	-1.742547745	0.685928313	-4.16347141	0.711255369	-1.742547745	0.685928313	-3.818826808	0.000452077
AT3G11385	AT3G11385	5.432773189	0.339653767	-3.591666303	NA	-5.458524863	0.30092474	-0.049599803	0.997796217	-5.458524863	0.30092474	-3.832172044	0.005685286
AT1G66470	RHD6					-4.321639773	0.426712153	0.48491027	0.96157769	-4.321639773	0.426712153	-3.84671149	0.000160693
AT4G14630	GLP9	4.59289171	0.06019489	-0.496674333	0.922519901	-0.927512565	0.484881894	-3.118701735	0.296836814	-0.927512565	0.484881894	-3.886146984	1.68546E-16
AT5G06490	AT5G06490	2.126870043	NA			-3.585343201	0.516230268	2.343371241	0.818724846	-3.585343201	0.516230268	-3.922769425	0.034468428
AT5G26300	AT5G26300							-4.404815965	0.697407195			-3.942017855	0.00036381
AT1G63450	RHS8	6.628646938	0.232148963	3.621360939	0.275580244	0.158106302	0.970905939	-4.51349352	0.691895802	0.158106302	0.970905939	-3.968595874	0.001489953
AT1G54950	AT1G54950							-5.786557056	0.629407893			-3.978775898	0.039934986
AT5G59930	AT5G59930	-3.643453079	NA	-1.555878094	NA			0.511955656	0.944303948			-3.993345042	0.001047059
AT3G16440	MLP-300B	6.927382177	0.138785489	1.787784434	0.771409648	-5.311246515	0.186216092	-3.191430356	0.306384936	-5.311246515	0.186216092	-3.993705611	7.03707E-06
AT1G47620	CYP96A8	6.117899554	0.277197367			-3.967003132	0.468268694	-1.485307075	0.853263662	-3.967003132	0.468268694	-4.00526656	0.024731204
AT1G26240	AT1G26240	-1.086479082	NA					-5.705481965	0.632278424			-4.007108667	0.022322615
AT1G35630	AT1G35630							-6.154807496	0.613859108			-4.013959536	0.048056513
AT1G26250	AT1G26250					-0.015098744	0.993579907			-0.015098744	0.993579907	-4.032918991	1.37463E-43
AT2G05185	AT2G05185	-0.217089032	0.872278913	-0.475126949	0.500571539	3.269431555	0.404061068	0.654460453	0.822720536	3.269431555	0.404061068	-4.034634734	0.024868438
AT5G47980	AT5G47980	5.042678423	NA					-2.308865551	0.748924676			-4.048353612	0.002290215
AT3G09922	IPS1	5.231439086	0.359574059	0.114186333	0.985862473			-1.671546944	0.691895802			-4.068787837	1.17693E-15
AT3G09290	TAC1	6.033007173	0.284808697	-1.555878094	NA	-4.710035467	0.382173607	-1.387562359	0.916241006	-4.710035467	0.382173607	-4.081776015	0.001282458
AT2G43220	AT2G43220							-1.265809086	0.939540455			-4.084609957	0.00121979
AT2G42660	AT2G42660	5.69720005	0.315114148					-7.308065885	0.133245343			-4.097624377	0.000125149
AT4G08360	AT4G08360	1.617891235	0.284712171	0.480705293	0.872175554	3.462579006	0.4113278	-0.505651202	0.943633329	3.462579006	0.4113278	-4.129356156	0.004543323
AT2G14560	LURP1	0.426225235	0.885054087	0.507171992	0.812539944	-0.390416758	0.843100235	-4.245613559	0.344233894	-0.390416758	0.843100235	-4.151700981	5.85612E-06
AT2G44340	AT2G44340	5.042678423	NA			-3.807506537	0.488805567	-3.862355172	0.731041294	-3.807506537	0.488805567	-4.153210155	0.003819036
AT5G40420	OLEO2							-5.135734119	0.662219342			-4.194596296	0.049502845
AT5G63750	ARI13	-3.775494867	0.530443243	-4.801221518	NA	-2.58669937	NA	-4.552333497	0.691824202	-2.58669937	NA	-4.202861046	0.010403202
AT5G47450	TIP2;3	3.063441608	0.262171958	1.326964133	0.059079899	0.390990252	0.508980697	-1.691788157	0.447508842	0.390990252	0.508980697	-4.226887636	4.82665E-32
AT4G29340	PRF4	2.227842796	0.440548701			-1.739705102	NA	0.68043359	0.927378527	-1.739705102	NA	-4.23976186	8.91489E-06
AT5G53190	SWEET3	5.750957256	0.309987743			5.783279694	0.262336134	-2.079412812	0.722781473	5.783279694	0.262336134	-4.270263312	6.01701E-06
AT3G14362	RTFL10	2.126870043	NA			-5.122387479	0.336768877	-0.587219444	0.972402645	-5.122387479	0.336768877	-4.283665718	1.03012E-06
AT1G60090	BGLU4	-5.293322881	0.352720519					-6.461046996	0.598901706			-4.289267817	0.006350047
AT1G51640	ATEXO70G2					-4.915513415	0.35934542			-4.915513415	0.35934542	-4.297520654	0.003588417
AT5G06900	CYP93D1	-4.412805514	NA	1.856738514	NA	-1.01747133	0.831404469			-1.01747133	0.831404469	-4.307794392	0.021758586
AT5G10760	AT5G10760	-0.496745189	0.880998472	2.083714526	0.312474532	-1.025107372	0.668519047	-7.356413207	0.122155019	-1.025107372	0.668519047	-4.34118687	0.000376733
AT5G06630	AT5G06630					1.236092522	0.839677401	-4.893976703	0.671789204	1.236092522	0.839677401	-4.359205588	0.037909367
AT1G55240	AT1G55240	3.080616095	0.256265469	4.041281529	0.051440312	-4.979653868	0.352774567	-2.351481653	0.597104923	-4.979653868	0.352774567	-4.37095221	0.007551936
AT5G42610	AT5G42610					-3.661418292	0.507620608	-2.543780126	NA	-3.661418292	0.507620608	-4.398796687	7.55103E-09
AT3G48850	PHT3;2	3.441979868	0.568380665	0.260181559	0.960880749	-2.964401061	NA	-1.13661482	0.907260297	-2.964401061	NA	-4.417053366	4.2709E-05
AT2G21650	MEE3	-3.830925935	0.124253177	-0.084875073	0.986523423	-4.184196107	0.442520847	0.942220273	0.959068856	-4.184196107	0.442520847	-4.423850527	2.4834E-07
AT5G05900	AT5G05900	-5.047135099	0.321911829	2.846079326	0.477916573	-5.198091203	0.32831486	-1.361572459	0.806550871	-5.198091203	0.32831486	-4.461628188	0.028646456
AT5G09640	SCPL19	4.594674208	NA	-4.460281122	NA	2.082711547	0.569366572	-5.152602224	0.636072176	2.082711547	0.569366572	-4.481369879	0.0121832
AT2G02690	AT2G02690	5.609419093	0.323552135	-2.175702898	NA	0.856115294	0.878070106	-6.612720149	0.589474071	0.856115294	0.878070106	-4.489620908	0.004076361
AT2G34910	AT2G34910					-4.191543895	0.441624455	-0.721041513	NA	-4.191543895	0.441624455	-4.49674008	0.023699309
AT2G04038	bZIP48	6.830237871	0.148043001	-4.460281122	NA	-0.575038073	0.7982071	-0.569764722	0.958564896	-0.575038073	0.7982071	-4.507363141	0.000695086
AT1G50580	AT1G50580	5.432773189	0.339653767	-4.761910288	NA	-5.699447449	0.276156845	0.509602996	NA	-5.699447449	0.276156845	-4.517890307	2.17194E-06
AT5G50800	SWEET13	-0.400924172	0.953812298	-5.152860033	0.336810247	-3.223321176	0.563907217	-1.515918737	0.92566856	-3.223321176	0.563907217	-4.603791435	0.000163956
AT5G14020	AT5G14020	5.646299122	0.320056086	-5.399427233	0.312186187	-3.585343201	0.516230268	-0.318717657	0.978041029	-3.585343201	0.516230268	-4.607664891	8.98303E-06
AT3G53150	UGT73D1	3.136659088	0.227027015	1.849282567	0.660761304	0.684712777	0.904058182	-7.256497582	0.456340511	0.684712777	0.904058182	-4.635555842	0.001667185
AT2G28270	AT2G28270							-1.175902819	0.93556951			-4.688972227	2.48646E-06
AT3G54520	AT3G54520	6.248685073	0.265576029	-0.64626273	NA	4.086606314	0.45356011	-5.750098604	0.63192307	4.086606314	0.45356011	-4.699578275	0.038540138

AT5G06640	EXT10					-3.99997266	0.464644883	-7.155836644	0.099695852	-3.99997266	0.464644883	-4.719979688	0.001542933
AT2G25160	CYP82F1	21.11868061	NA					-1.269265681	0.632278424			-4.76556694	4.40806E-13
AT5G49270	SHV2	-4.92685669	NA			5.413759914	0.304852306	-6.900973855	0.510634068	5.413759914	0.304852306	-4.795299857	0.012906514
AT5G57420	IAA33							-5.896132131	0.627184543			-4.838744259	0.022964941
AT4G31940	CYP82C4			-4.591953932	NA			-7.480257672	0.385221483			-4.868522987	0.009421196
AT4G25250	AT4G25250	0.812534912	0.907880283	-6.104303092	0.237614438			-6.738209976	0.572365365			-4.880093683	0.001092411
AT2G45130	SPX3	-5.293322881	0.352720519	-0.678811792	NA			-6.840191704	0.534532914			-4.900060485	6.19417E-09
AT3G14710	AT3G14710	1.142250198	0.826527327	-4.913938244	0.363070633	1.636556177	NA	-1.717757086	0.830078697	1.636556177	NA	-4.908293106	0.018032696
AT4G08400	AT4G08400					-4.806752021	0.371732733	-0.210678333	0.991559366	-4.806752021	0.371732733	-4.917617548	0.049376793
AT1G57590	AT1G57590	0.43810141	0.949374547	2.408612127	0.684862398	-0.832506789	0.894311921	1.638385778	0.916448617	-0.832506789	0.894311921	-4.920136868	0.001434904
AT3G45330	AT3G45330			2.810399928	NA	0.027387621	0.996827525	-5.971239531	0.621192363	0.027387621	0.996827525	-4.957704749	0.014965148
AT1G53610	AT1G53610	3.718535445	NA									-5.000970203	0.017452185
AT4G08620	SULTR1;1	5.858559329	0.300195579	1.24474156	NA			0.597620495	0.973706963			-5.041842889	0.000100847
AT1G56630	AT1G56630	5.368733387	0.346197649					-1.564762942	0.910061478			-5.05772053	0.018351032
AT4G05540	AT4G05540	4.456967053	NA	5.413700435	0.311461713			-4.931481904	0.66808962			-5.07445125	0.043477971
AT1G33540	scpl18	1.156616053	NA	-5.546283109	0.297969732	-3.374634797	0.544074387	-7.886137598	0.254165498	-3.374634797	0.544074387	-5.076197444	0.010323032
AT5G41765	AT5G41765							-2.283491611	NA			-5.098774032	0.02719122
AT5G02340	AT5G02340					-2.353593325	NA			-2.353593325	NA	-5.105782097	0.033035603
AT1G14220	AT1G14220	5.952993806	0.291779572	-4.654549559	NA	-6.118168174	0.067825402	-5.784839125	0.268420306	-6.118168174	0.067825402	-5.12376012	0.01864954
AT2G36030	AT2G36030					-5.271390188	0.202170474			-5.271390188	0.202170474	-5.13177983	0.043841281
AT5G20790	AT5G20790	6.241727464	0.266100077	0.223143721	0.963385401	-2.002619449	0.7397377	-2.875684292	0.628417613	-2.002619449	0.7397377	-5.19204514	3.36552E-08
AT2G26370	AT2G26370	5.880662873	0.298216422	-5.700351932	0.283173235	5.808781649	0.255941809	-0.390994648	0.957738363	5.808781649	0.255941809	-5.218476418	0.005902568
AT3G54580	AT3G54580	7.292607849	0.080843759	-3.424152558	0.400075583	1.628216171	0.68996294	-0.768037276	0.733019473	1.628216171	0.68996294	-5.253722707	0.000221746
AT2G03630	AT2G03630	-4.692608195	NA	2.886505341	0.610854995			-4.124993846	0.712887303			-5.269242539	0.026331823
AT1G50610	PRK5											-5.297984095	0.029719174
AT1G21651	AT1G21651	-4.179902883	0.48530428	5.338352945	0.318842237			-5.563385291	0.637815859			-5.304453858	0.02252136
AT1G53640	AT1G53640	1.744829334	0.619763594	-5.253633048	0.326516785			-1.234472015	0.909363112			-5.306982819	0.025000385
AT5G24150	SQP1	-2.446094902	0.289304229	1.099387522	0.725558826	-0.460086586	0.897523434	-6.590104196	0.581196649	-0.460086586	0.897523434	-5.313248039	0.011328211
AT3G60270	AT3G60270	0.858616573	0.902670805	0.587271964	0.929037174			5.807203621	0.628720094			-5.318877956	0.011836481
AT2G45550	CYP76C4	2.363513282	0.716797663	6.96084028	0.178429635			-0.283467103	0.981525175			-5.365310925	0.002549854
AT5G27020	AT5G27020					-4.433215038	0.414155513			-4.433215038	0.414155513	-5.370652087	0.007652388
AT1G24230	AT1G24230					-0.588116002	0.928406277			-0.588116002	0.928406277	-5.375744913	0.019940209
AT1G30560	G3Pp3							6.023955062	0.619354152			-5.398209376	0.018381893
AT2G03720	MRH6			-1.590061409	NA			-2.864628523	NA			-5.416280693	0.028186644
AT1G80590	WRKY66	1.536966917	0.684321794	1.884683549	0.413096661			-6.774863483	0.547176358			-5.440232632	0.000256067
AT4G37850	AT4G37850					-4.144355925	0.447084424	-2.062665264	0.742807747	-4.144355925	0.447084424	-5.473160108	0.002868787
AT2G31082	CLE7			-5.695606491	0.283652882			-6.044587701	0.618005886			-5.497539143	0.001183611
AT4G12545	AT4G12545					2.961867924	0.497863092	-5.503828025	0.602119156	2.961867924	0.497863092	-5.512376524	8.26886E-31
AT2G04090	AT2G04090	0.805608932	0.890244766	-4.679433448	NA	2.21798689	NA	-2.291155622	0.62738272	2.21798689	NA	-5.566188742	0.033312341
AT2G29000	AT2G29000											-5.593387664	0.030766757
AT3G30580	AT3G30580			1.24474156	NA	-4.97459813	0.353163742	6.572392473	0.592520742	-4.97459813	0.353163742	-5.595015054	0.002457529
AT1G27140	GSTU14	4.910080283	NA	-2.175702898	NA			-1.950575233	0.772801059			-5.638496048	6.1155E-06
AT5G55350	AT5G55350					-3.801136243	0.488928855	-0.172879073	0.989159859	-3.801136243	0.488928855	-5.641857655	0.000909613
AT2G33070	NSP2											-5.650088088	2.7627E-05
AT1G34140	PAB1	1.40056266	0.841360256					1.049630194	0.953801756			-5.741155719	0.040231534
AT3G49845	WIH3	4.456967053	NA			-2.242707603	NA	-0.89218332	0.864757186	-2.242707603	NA	-5.753247392	1.15159E-07
AT1G56085	AT1G56085			5.191608267	NA							-5.756778032	0.049789418
AT5G45105	None											-5.76715277	0.01006647
AT3G10710	RHS12	-5.84317138	0.301385348	0.099751571	0.988696861			-6.215768965	0.388440005			-5.786088015	0.002209623
AT3G14880	AT3G14880	6.515226797	0.210562947	2.727384763	0.639017608	-2.648405744	NA	-6.775429997	0.183329115	-2.648405744	NA	-5.85036139	0.018694486
AT5G52390	AT5G52390	-6.724174759	NA			-2.648405744	NA	-1.384215714	0.782193202	-2.648405744	NA	-5.88071906	0.000169647
AT4G37990	ELI3-2	-1.809597744	0.607762727	3.924216414	0.190052712	-1.083733475	0.616713539	-6.728227305	0.582068518	-1.083733475	0.616713539	-5.939755463	0.000704528
AT2G07675	AT2G07675	3.186895379	0.566849637	1.424183374	0.685012219	-0.523355977	0.935484086	-4.999760003	0.652047062	-0.523355977	0.935484086	-5.957764613	0.003572488
AT4G27400	AT4G27400	-0.840503658	0.910813917	4.717218612	NA	6.239455343	0.083560614			6.239455343	0.083560614	-6.024098475	0.024266678
AT2G34202	ath-MIR399d							-5.251610535	0.653363031			-6.030674749	0.003150249
AT3G12230	scpl14							1.757847263	0.820789421			-6.039343557	0.049523081
AT3G59370	AT3G59370	2.384244613	0.694887344	-5.718084279	0.281483628	-4.584492934	0.396348235	-1.256372298	0.593505099	-4.584492934	0.396348235	-6.073174458	0.004192104
AT3G60470	AT3G60470	5.079689919	NA	4.400545508	NA			3.107107325	0.673165787			-6.121204345	0.008896021
AT5G24313	AT5G24313							-6.552166728	0.583897164			-6.172243796	0.009114474
AT1G61224	ath-MIR842	5.836866552	0.302165345	3.236246462	0.577487316	-3.322614377	0.550728949	0.212782907	0.990915497	-3.322614377	0.550728949	-6.192133854	0.021302175
AT1G33100	AT1G33100	3.941445652	NA					1.295056397	0.922005452			-6.207796286	0.036317621
AT2G23630	sks16							-7.252595657	0.174101569			-6.207796286	0.036317621
AT5G44770	AT5G44770											-6.208282332	0.010707715
AT1G64070	RLM1					-3.967003132	0.468268694			-3.967003132	0.468268694	-6.235062849	0.001089646
AT4G22080	RHS14							-6.421029243	0.6015277			-6.277257109	0.033072554
AT5G57540	XTH13			-2.913252	NA			-2.543780126	NA			-6.290281544	0.028481153
AT4G31970	CYP82C2	5.135882068	NA					-2.295853967	0.800300732			-6.298137311	0.027935422
AT3G16555	AT3G16555			0.824711119	0.893210754	-4.169754161	0.444084641			-4.169754161	0.444084641	-6.31248011	0.007429483
AT5G51490	AT5G51490	0.914346934	NA			-5.218253205	0.317035445	-0.221692689	0.991271129	-5.218253205	0.317035445	-6.320637479	0.030194238
AT2G28755	AT2G28755			5.721474013	0.281612599			-6.062073545	0.616300683			-6.338373976	0.010108746
AT3G20590	AT3G20590	2.126870043	NA	-4.253899481	NA	-5.941523691	0.144744566	2.212666109	0.726713101	-5.941523691	0.144744566	-6.344133249	1.26235E-05
AT5G11440	CID5	4.456967053	NA	-5.17700955	0.33432887	4.53342322	0.401492775	1.124418551	0.939540455	4.53342322	0.401492775	-6.386989316	0.005809912
AT1G26410	AT1G26410	4.943035281	NA	-0.093582438	0.985207836			-3.862355172	0.731041294			-6.416289608	0.012259574
AT3G17500	AT3G17500	1.156616053	NA			-3.001110281	NA			-3.001110281	NA	-6.416516782	0.023698478
AT3G22500	ATECP31	-4.802878425	NA			5.413759914	0.304852306	0.456097573	0.981578919	5.413759914	0.304852306	-6.435350953	0.021487272
AT3G56350	AT3G56350	5.376482665	0.345604796	-4.286237082	NA	4.53342322	0.401492775	-3.999643691	0.721140128	4.53342322	0.401492775	-6.442331183	0.000220782
AT3G25950	AT3G25950			-5.789231544	0.274768638			-3.610093876	0.66808962			-6.459721646	0.005783902
AT2G25150	AT2G25150	-2.4272238	0.436745235	-3.799086364	0.325007228	3.265848638	0.55758193	-1.479316836	0.782073003	3.265848638	0.55758193	-6.4600747	2.30325E-05
AT5G51520	AT5G51520					-5.090668499	0.340353148	-3.126920848	NA	-5.090668499	0.340353148	-6.470052492	0.009131511
AT3G50150	AT3G50150							0.308245346	0.984609535			-6.485971785	0.019053157
AT5G54790	AT5G54790							-5.998735832	0.619569853			-6.485971785	0.019053157
AT5G52260	MYB19	5.231439086	0.359574059					-7.15151405	0.446367117			-6.519890925	0.019630637
AT1G72980	LBD7			-4.176821531	NA	-2.964401061	NA	-6.597806956	0.575257546	-2.964401061	NA	-6.610177401	0.005216304
AT1G21230	WAK5			-3.163547123	NA			-5.915036667	0.626508471			-6.632245671	0.003184853
AT1G22090	emb2204			-3.572835016	NA							-6.672348677	0.00292994
AT1G48130	PER1											-6.673401215	0.011916594
AT4G22110	AT4G22110	7.037124481	NA					-0.337955556	0.982734229			-6.685683148	0.016474873
AT3G20360	AT3G20360	5.862887199	0.299731107	-5.636488978	0.289294256			3.270781675	0.663999152			-6.708169143	0.001481989
AT3G46400	AT3G46400							0.815754653	0.965471639			-6.733322204	0.012388353
AT1G22150	SULTR1;3			-6.411572765	0.221380384			-6.422860086	0.599840747			-6.743103976	0.009019729
AT5G62340	AT5G62340	6.662931335	0.229241569	-6.680992733	0.121019291	-1.196634119	0.747848981	-1.251872486	NA	-1.196634119	0.747848981	-6.774042937	5.39116E-11
AT1G19900	AT1G19900											-6.790585851	0.010360997
AT2G16230	AT2G16230					-0.325086005	0.960965405	-4.347100853	0.702246294	-0.325086005	0.960965405	-6.822200738	0.001037727
AT5G55240	ATPXG2			6.442169797	0.219122954			-3.928658632	0.726900342			-6.880776631	0.008064034

AT1G55430	AT1G55430	-6.321300904	0.258735538	0.762886339	0.907826886	-1.108056393	NA	-4.60160335	0.685495817	-1.108056393	NA	-6.8835325	0.006036189
AT5G35190	EXT13			5.87624709	0.267800994	4.327246017	0.426259265			4.327246017	0.426259265	-6.949863567	0.006216341
AT4G00680	ADF8	-5.411926248	0.341583685			-3.99997266	0.464644883	-7.777310652	0.292351702	-3.99997266	0.464644883	-6.953592333	0.000750045
AT3G59850	AT3G59850							-0.505695835	0.975645787			-6.987351626	0.004653992
AT3G07070	AT3G07070	6.973961521	0.203344415					-2.864628523	NA			-7.063435992	0.004077958
AT3G24210	AT3G24210					-5.034378277	0.258776909	-6.238885978	0.610227484	-5.034378277	0.258776909	-7.074118405	2.22785E-05
AT3G05155	AT3G05155	2.971234576	0.630645607	-5.701466479	0.283095227			-2.622648791	0.66808962			-7.307394607	0.000109464
AT3G24750	AT3G24750							-4.697871755	0.680595832			-7.342136769	0.000287208
AT1G19250	FMO1			5.480732866	0.304874357	4.148859113	0.445672623	-7.125453077	0.457619105	4.148859113	0.445672623	-7.362161363	0.000113002
AT5G51500	AT5G51500			2.409435164	NA	-3.001110281	NA	0.048367538	0.997790351	-3.001110281	NA	-7.415670352	0.000377285
AT5G14340	MYB40					2.951852217	NA	-6.237506607	0.610227484	2.951852217	NA	-7.418510782	6.68142E-05
AT1G52050	AT1G52050			-5.47704108	0.304752967	-3.99997266	0.464644883	-6.513156536	0.597380545	-3.99997266	0.464644883	-7.58394051	0.030212939
AT4G01630	EXPA17					-2.002730959	NA	-5.982877472	0.463148403	-2.002730959	NA	-7.791109915	0.024612058
AT3G54590	HRGP1					-0.763541118	0.856669534	-3.59732743	0.48633216	-0.763541118	0.856669534	-8.13777572	1.43021E-08
AT1G68250	AT1G68250	-5.273608137	0.354913301	1.398676344	0.782980233	-4.169754161	0.444084641	-5.897880016	0.627163215	-4.169754161	0.444084641	-8.150458656	0.010607949
AT3G12240	SCPL15							-6.508832807	0.281217577			-8.206261803	2.02494E-05
AT2G47540	AT2G47540							0.040335591	0.997245719			-8.220323741	4.11301E-06
AT4G09610	GASA2			6.878190921	0.184493817							-8.315659799	7.60901E-07
AT2G24980	EXT6			-4.591953932	NA			-3.847597861	0.655815891			-8.386257311	4.31673E-07
AT5G65158	PLAT3							-5.687649713	0.633628949			-8.578571469	3.742E-08
AT4G22666	AT4G22666	21.22520169	NA					1.20339207	0.945419933			-9.310213948	4.12913E-09
AT5G54740	SESA5											-10.01510212	0.038188159
AT4G28520	CRU3							-7.20252428	0.128407019			-12.4103275	0.007610985

Supplementary Table 2. PHYD-dependent and ecotype-related spaceflight-altered genes in the light and in the dark.The overlapped genes of different groups are highlighted in the same colors.

PHYD-dependent spaceflight-altered genes	
light	dark
AT1G08990	AT1G21395
AT1G29280	AT2G13360
AT1G73270	AT2G35770
AT1G77885	AT3G20180
AT2G05440	AT3G61840
AT2G27550	AT3G62550
AT2G29330	AT4G19530
AT2G36120	AT4G35733
AT2G37750	AT4G35770
AT2G44790	AT5G11630
AT3G01420	AT5G50140
AT3G06390	AT5G54770
AT3G13435	AT3G54890
AT3G14280	AT5G46490
AT3G28345	AT4G13495
AT3G53480	AT1G74930
AT3G55230	AT4G09900
AT3G62270	AT1G15125
AT4G01350	AT3G12830
AT4G17800	AT1G73540
AT4G30450	AT3G44260
AT4G38080	AT4G27280
AT5G09530	AT4G27652
AT5G17530	AT3G56710
AT5G20960	AT5G48850
AT5G25110	AT5G35110
AT5G47000	AT2G38060
AT5G52900	AT4G25310
AT5G59530	
AT5G63600	
AT5G66390	
AT1G02900	
AT1G22065	
AT1G31050	
AT1G34340	
AT1G61050	
AT1G69920	
AT1G78895	
AT2G04800	
AT2G21045	
AT2G21060	
AT2G21530	
AT2G43050	
AT2G46750	
AT3G04570	
AT3G07030	
AT3G16450	
AT3G21770	
AT3G22240	
AT3G28360	
AT3G45140	
AT3G46130	
AT3G46970	
AT3G54040	
AT4G11650	
AT4G25400	
AT4G30270	
AT4G31380	
AT4G39795	
AT5G02090	
AT5G10580	
AT5G24210	
AT5G25980	
AT5G38450	
AT5G43580	
AT5G47990	
AT5G48110	
AT5G51780	
AT5G55090	

Ecotype-related spaceflight-altered genes			
Ws-deficient in light	Col-0-deficient in light	Ws-deficient in dark	Col-0-deficient in dark
AT1G08990	AT1G04660	AT1G21395	AT1G04660
AT1G29280	AT1G64370	AT2G13360	AT1G64370
AT1G73270	AT2G18550	AT2G35770	AT2G18550
AT1G77885	AT2G29740	AT3G20180	AT2G29740
AT2G05440	AT2G47000	AT3G61840	AT2G47000
AT2G27550	AT4G09760	AT3G62550	AT4G09760
AT2G29330	AT4G35090	AT4G19530	AT4G35090
AT2G36120	AT1G01070	AT4G35733	AT1G01130
AT2G37750	AT1G02230	AT4G35770	AT1G01180
AT2G44790	AT1G02670	AT5G11630	AT1G02460
AT3G01420	AT1G04220	AT5G50140	AT1G02520
AT3G06390	AT1G04250	AT5G54770	AT1G03270
AT3G13435	AT1G04540	AT1G04580	AT1G03410
AT3G14280	AT1G05840	AT1G09932	AT1G03850
AT3G28345	AT1G06090	AT1G10400	AT1G03880
AT3G53480	AT1G07135	AT1G12480	AT1G05340
AT3G55230	AT1G07220	AT1G21400	AT1G05550
AT3G62270	AT1G08165	AT1G22120	AT1G05880
AT4G01350	AT1G08280	AT1G59670	AT1G06830
AT4G17800	AT1G08810	AT1G60190	AT1G07590
AT4G30450	AT1G10520	AT1G61480	AT1G09157
AT4G38080	AT1G10770	AT1G64405	AT1G09240
AT5G09530	AT1G10865	AT1G68010	AT1G09750
AT5G17530	AT1G11440	AT1G70170	AT1G09970
AT5G20960	AT1G11850	AT1G77210	AT1G10640
AT5G25110	AT1G14350	AT1G79040	AT1G13530
AT5G47000	AT1G18270	AT2G03020	AT1G13670
AT5G52900	AT1G18280	AT2G34390	AT1G14520
AT5G59530	AT1G21130	AT3G03480	AT1G15170
AT5G63600	AT1G22110	AT3G16410	AT1G15380
AT5G66390	AT1G23130	AT3G17690	AT1G16410
AT3G14420	AT1G23160	AT3G14420	AT1G17380
AT5G41570	AT1G24580	AT5G41570	AT1G17600
AT1G01360	AT1G25390	AT3G20730	AT1G18390
AT1G01730	AT1G26650	AT3G22235	AT1G19210
AT1G01860	AT1G29500	AT3G22961	AT1G21100
AT1G02520	AT1G30600	AT3G25780	AT1G21360
AT1G02640	AT1G30970	AT3G54065	AT1G21920
AT1G03520	AT1G31350	AT3G61470	AT1G22160
AT1G04310	AT1G31720	AT4G04423	AT1G22810
AT1G05260	AT1G33110	AT4G15550	AT1G23010
AT1G05700	AT1G33350	AT4G21400	AT1G27770
AT1G07610	AT1G37140	AT4G25430	AT1G27820
AT1G07873	AT1G43910	AT5G10990	AT1G28130
AT1G08320	AT1G44800	AT5G13320	AT1G29640
AT1G08500	AT1G49530	AT5G14470	AT1G30135
AT1G08800	AT1G52680	AT5G19940	AT1G30250
AT1G08903	AT1G52770	AT5G24860	AT1G30720
AT1G09163	AT1G52830	AT5G45280	AT1G30814
AT1G09415	AT1G53140	AT5G49920	AT1G30840
AT1G09480	AT1G59960	AT5G52710	AT1G31820
AT1G09490	AT1G61810		AT1G31950
AT1G09647	AT1G62640		AT1G33440
AT1G10360	AT1G63300		AT1G33960
AT1G12420	AT1G63850		AT1G34245
AT1G12800	AT1G64330		AT1G35183
AT1G13930	AT1G64390		AT1G35210
AT1G14270	AT1G65295		AT1G44160
AT1G14580	AT1G66110		AT1G44830
AT1G14687	AT1G66400		AT1G46554
AT1G14700	AT1G68570		AT1G46768
AT1G14870	AT1G68880		AT1G47395
AT1G15210	AT1G69220		AT1G48000
AT1G16070	AT1G70510		AT1G48300
AT1G17170	AT1G70990		AT1G48800
AT1G17850	AT1G72230		AT1G49310
AT1G18510	AT1G74010		AT1G50740
AT1G18860	AT1G74300		AT1G51170
AT1G19450	AT1G74910		AT1G52342

AT5G57123	
AT5G66690	

AT1G19490	AT1G75830		AT1G52690
AT1G19650	AT1G76610		AT1G53520
AT1G20900	AT1G77690		AT1G53920
AT1G21670	AT1G78100		AT1G55610
AT1G22430	AT1G80380		AT1G55740
AT1G22670	AT2G01320		AT1G56600
AT1G22710	AT2G01890		AT1G58190
AT1G23710	AT2G01940		AT1G58280
AT1G23850	AT2G03550		AT1G58420
AT1G26450	AT2G04160		AT1G60260
AT1G26800	AT2G12400		AT1G61130
AT1G27670	AT2G14100		AT1G61290
AT1G28230	AT2G16750		AT1G61470
AT1G28760	AT2G16760		AT1G61560
AT1G30080	AT2G17470		AT1G62560
AT1G30520	AT2G18540		AT1G62570
AT1G30650	AT2G21590		AT1G62660
AT1G30700	AT2G24580		AT1G62810
AT1G31160	AT2G26180		AT1G63630
AT1G31770	AT2G26760		AT1G64210
AT1G32060	AT2G27000		AT1G65510
AT1G32440	AT2G28250		AT1G65890
AT1G32470	AT2G28410		AT1G66860
AT1G33520	AT2G28970		AT1G67470
AT1G33750	AT2G29380		AT1G67870
AT1G35330	AT2G31360		AT1G68238
AT1G48360	AT2G32920		AT1G68320
AT1G48550	AT2G33350		AT1G71040
AT1G49310	AT2G33400		AT1G72120
AT1G49570	AT2G35820		AT1G72200
AT1G50020	AT2G36540		AT1G72510
AT1G50250	AT2G36690		AT1G72660
AT1G50450	AT2G37880		AT1G72910
AT1G51840	AT2G38220		AT1G72950
AT1G52870	AT2G38750		AT1G74770
AT1G55610	AT2G39110		AT1G75050
AT1G56500	AT2G39360		AT1G75750
AT1G56555	AT2G39370		AT1G76590
AT1G56600	AT2G40050		AT1G76640
AT1G59840	AT2G40260		AT1G76705
AT1G61080	AT2G40900		AT1G77270
AT1G61300	AT2G41660		AT1G78290
AT1G61500	AT2G42350		AT1G78820
AT1G61590	AT2G42430		AT1G79270
AT1G61870	AT2G42760		AT1G79310
AT1G62580	AT2G43290		AT1G79510
AT1G64160	AT2G43400		AT1G80160
AT1G64590	AT2G43800		AT1G80180
AT1G65250	AT2G45320		AT1G80760
AT1G65510	AT2G45660		AT2G01180
AT1G65690	AT2G45910		AT2G01660
AT1G66980	AT2G46950		AT2G02500
AT1G67030	AT2G47140		AT2G02930
AT1G67090	AT2G47800		AT2G05540
AT1G68150	AT2G48020		AT2G05810
AT1G69490	AT3G02410		AT2G07698
AT1G69600	AT3G03480		AT2G15042
AT1G70130	AT3G03830		AT2G15490
AT1G70260	AT3G04640		AT2G16990
AT1G70410	AT3G05800		AT2G18050
AT1G70610	AT3G06080		AT2G19620
AT1G70880	AT3G06125		AT2G20080
AT1G71440	AT3G06850		AT2G20350
AT1G71692	AT3G07215		AT2G20880
AT1G71697	AT3G07250		AT2G21220
AT1G71960	AT3G07720		AT2G22590
AT1G72900	AT3G08630		AT2G23030
AT1G74500	AT3G09020		AT2G23100
AT1G74670	AT3G09035		AT2G24260
AT1G74710	AT3G11720		AT2G24550
AT1G76110	AT3G12820		AT2G25150
AT1G77490	AT3G13450		AT2G25160
AT1G78000	AT3G13520		AT2G25770
AT1G78340	AT3G16420		AT2G25780
AT1G78780	AT3G16660		AT2G26211

AT1G79410	AT3G17120		AT2G27270
AT1G79460	AT3G17520		AT2G29060
AT1G79580	AT3G18217		AT2G29490
AT1G80180	AT3G19550		AT2G30550
AT1G80830	AT3G20015		AT2G30840
AT2G01008	AT3G20470		AT2G31100
AT2G01110	AT3G21670		AT2G32530
AT2G01660	AT3G22600		AT2G33380
AT2G01770	AT3G22620		AT2G33550
AT2G01870	AT3G22750		AT2G33580
AT2G02120	AT3G23430		AT2G35170
AT2G02450	AT3G23810		AT2G35300
AT2G02850	AT3G25110		AT2G35930
AT2G02930	AT3G25190		AT2G36320
AT2G03360	AT3G25600		AT2G36380
AT2G03855	AT3G25710		AT2G37170
AT2G05155	AT3G25890		AT2G38230
AT2G05510	AT3G26115		AT2G38400
AT2G07642	AT3G27025		AT2G38500
AT2G07680	AT3G28130		AT2G38800
AT2G07698	AT3G28960		AT2G39210
AT2G07728	AT3G30180		AT2G39310
AT2G07773	AT3G30775		AT2G39650
AT2G14820	AT3G43810		AT2G40390
AT2G15000	AT3G44260		AT2G41260
AT2G15980	AT3G44790		AT2G42530
AT2G16005	AT3G47730		AT2G42540
AT2G17695	AT3G47870		AT2G43535
AT2G18890	AT3G49220		AT2G44290
AT2G19110	AT3G50190		AT2G44480
AT2G20250	AT3G50340		AT2G45210
AT2G20550	AT3G50760		AT2G45560
AT2G20680	AT3G50770		AT2G45830
AT2G20890	AT3G53040		AT2G46400
AT2G21330	AT3G54500		AT2G46690
AT2G21340	AT3G57040		AT2G47130
AT2G21900	AT3G57450		AT2G47180
AT2G23120	AT3G57520		AT2G47270
AT2G26080	AT3G59270		AT2G47485
AT2G26820	AT3G59900		AT2G47750
AT2G28110	AT3G61070		AT2G47890
AT2G28960	AT3G61820		AT3G01420
AT2G29290	AT3G62510		AT3G01520
AT2G29440	AT3G63170		AT3G01830
AT2G29660	AT4G00110		AT3G02620
AT2G29670	AT4G01390		AT3G03470
AT2G31020	AT4G02000		AT3G04010
AT2G32010	AT4G02630		AT3G04410
AT2G32240	AT4G02830		AT3G05937
AT2G32280	AT4G03210		AT3G06020
AT2G33250	AT4G04610		AT3G08860
AT2G33480	AT4G04745		AT3G10930
AT2G34317	AT4G09870		AT3G12580
AT2G34500	AT4G10380		AT3G13600
AT2G35270	AT4G10845		AT3G15240
AT2G35370	AT4G12470		AT3G15510
AT2G36260	AT4G13950		AT3G16220
AT2G36295	AT4G16807		AT3G16250
AT2G36320	AT4G17840		AT3G16430
AT2G36330	AT4G17880		AT3G16690
AT2G37450	AT4G18650		AT3G16990
AT2G38380	AT4G21740		AT3G17790
AT2G39140	AT4G21960		AT3G18560
AT2G39250	AT4G23340		AT3G19553
AT2G39795	AT4G25470		AT3G20370
AT2G42870	AT4G25490		AT3G20590
AT2G43060	AT4G25900		AT3G21150
AT2G44010	AT4G27595		AT3G22830
AT2G44230	AT4G28400		AT3G22886
AT2G44370	AT4G30650		AT3G23480
AT2G45930	AT4G31500		AT3G24070
AT2G47130	AT4G31590		AT3G25790
AT2G47940	AT4G31610		AT3G26170
AT2G48130	AT4G31730		AT3G26180
AT2G48140	AT4G31830		AT3G26500

AT3G01350	AT4G34580		AT3G26612
AT3G01516	AT4G34710		AT3G27250
AT3G01795	AT4G35690		AT3G28007
AT3G02800	AT4G36850		AT3G28340
AT3G02830	AT4G37940		AT3G28345
AT3G03520	AT4G38400		AT3G29030
AT3G04115	AT4G38480		AT3G43850
AT3G04530	AT4G38660		AT3G44300
AT3G06035	AT4G39364		AT3G44720
AT3G06490	AT5G01170		AT3G45700
AT3G07090	AT5G01300		AT3G46690
AT3G07255	AT5G01870		AT3G48390
AT3G07760	AT5G02260		AT3G48510
AT3G08435	AT5G02430		AT3G48740
AT3G08970	AT5G03190		AT3G49710
AT3G10190	AT5G03770		AT3G50260
AT3G10780	AT5G03830		AT3G50640
AT3G11170	AT5G04380		AT3G50930
AT3G11930	AT5G04520		AT3G50970
AT3G12090	AT5G07030		AT3G51330
AT3G12470	AT5G07460		AT3G51350
AT3G13432	AT5G08210		AT3G51895
AT3G13910	AT5G09930		AT3G52180
AT3G14200	AT5G10946		AT3G52910
AT3G14260	AT5G11740		AT3G53480
AT3G14330	AT5G12420		AT3G54260
AT3G14440	AT5G20380		AT3G54580
AT3G14620	AT5G20670		AT3G56080
AT3G14680	AT5G20740		AT3G56360
AT3G15440	AT5G22880		AT3G56790
AT3G15500	AT5G23950		AT3G57770
AT3G15570	AT5G24460		AT3G57860
AT3G20898	AT5G25140		AT3G59310
AT3G21260	AT5G26147		AT3G59370
AT3G21510	AT5G26190		AT3G60140
AT3G22800	AT5G28030		AT3G60490
AT3G23180	AT5G28050		AT3G60720
AT3G23490	AT5G35660		AT3G61160
AT3G23840	AT5G35732		AT3G61430
AT3G24070	AT5G37770		AT3G61897
AT3G25660	AT5G38000		AT3G61900
AT3G25805	AT5G39380		AT3G61990
AT3G29000	AT5G40510		AT3G62030
AT3G43540	AT5G41460		AT3G62410
AT3G45260	AT5G41580		AT3G63480
AT3G46370	AT5G41660		AT4G01420
AT3G47030	AT5G41750		AT4G01535
AT3G47120	AT5G42655		AT4G01540
AT3G47400	AT5G43180		AT4G02330
AT3G48200	AT5G43660		AT4G03320
AT3G48680	AT5G44250		AT4G04260
AT3G48700	AT5G44310		AT4G04955
AT3G48800	AT5G44720		AT4G05150
AT3G49360	AT5G46910		AT4G08300
AT3G49480	AT5G47230		AT4G10260
AT3G49780	AT5G48940		AT4G11211
AT3G50200	AT5G51580		AT4G11650
AT3G50640	AT5G51970		AT4G12320
AT3G50820	AT5G52500		AT4G13030
AT3G50970	AT5G54140		AT4G13210
AT3G51350	AT5G54170		AT4G13530
AT3G51820	AT5G55700		AT4G14580
AT3G51910	AT5G57565		AT4G15610
AT3G52850	AT5G58180		AT4G15990
AT3G53200	AT5G60030		AT4G16000
AT3G53230	AT5G61290		AT4G16008
AT3G54240	AT5G62170		AT4G16190
AT3G54610	AT5G62350		AT4G16563
AT3G54920	AT5G63260		AT4G17340
AT3G54960	AT5G63780		AT4G20820
AT3G55210	AT5G65830		AT4G21120
AT3G55630	AT5G66620		AT4G21215
AT3G56160	AT5G67280		AT4G22513
AT3G56940			AT4G23050
AT3G57380			AT4G23310

AT3G57910			AT4G23450
AT3G58840			AT4G23493
AT3G59220			AT4G23810
AT3G59780			AT4G24480
AT3G60970			AT4G26010
AT3G63010			AT4G26520
AT3G63110			AT4G28040
AT4G00370			AT4G29110
AT4G00910			AT4G29610
AT4G01440			AT4G29640
AT4G01480			AT4G30270
AT4G02090			AT4G30280
AT4G02900			AT4G30430
AT4G05010			AT4G30460
AT4G05715			AT4G31370
AT4G05840			AT4G31380
AT4G07820			AT4G32480
AT4G10000			AT4G32800
AT4G10060			AT4G32940
AT4G10843			AT4G33390
AT4G10860			AT4G34210
AT4G11100			AT4G34410
AT4G11300			AT4G35750
AT4G11600			AT4G36450
AT4G11655			AT4G39700
AT4G12290			AT4G39970
AT4G13030			AT5G01015
AT4G13660			AT5G01040
AT4G14020			AT5G01610
AT4G14465			AT5G01680
AT4G14580			AT5G02720
AT4G15270			AT5G03890
AT4G15280			AT5G04080
AT4G15340			AT5G04150
AT4G15740			AT5G05110
AT4G16260			AT5G05320
AT4G17810			AT5G06320
AT4G18280			AT5G06760
AT4G18510			AT5G07010
AT4G18980			AT5G07650
AT4G19980			AT5G07680
AT4G21570			AT5G07690
AT4G23600			AT5G08460
AT4G23630			AT5G09440
AT4G23700			AT5G10100
AT4G23880			AT5G13080
AT4G24460			AT5G13210
AT4G24480			AT5G13370
AT4G25000			AT5G14070
AT4G25100			AT5G14120
AT4G25290			AT5G14940
AT4G25380			AT5G15960
AT4G27370			AT5G17120
AT4G28230			AT5G18660
AT4G31100			AT5G21960
AT4G31330			AT5G22550
AT4G31351			AT5G23750
AT4G31850			AT5G24120
AT4G32260			AT5G24210
AT4G32650			AT5G24530
AT4G32770			AT5G26030
AT4G32860			AT5G26146
AT4G32870			AT5G26310
AT4G32940			AT5G27940
AT4G33000			AT5G35370
AT4G33420			AT5G37500
AT4G33500			AT5G37760
AT4G34230			AT5G37980
AT4G35180			AT5G40780
AT4G36540			AT5G42250
AT4G36840			AT5G42380
AT4G37010			AT5G42530
AT4G39675			AT5G43150
AT5G01240			AT5G43630
AT5G01920			AT5G43840

	AT5G02120			AT5G44005
	AT5G02940			AT5G44440
	AT5G03040			AT5G46710
	AT5G03285			AT5G46880
	AT5G03570			AT5G47240
	AT5G03700			AT5G47910
	AT5G03890			AT5G49290
	AT5G04140			AT5G49360
	AT5G04205			AT5G49660
	AT5G04360			AT5G49690
	AT5G04530			AT5G50200
	AT5G05320			AT5G50300
	AT5G06230			AT5G50720
	AT5G06530			AT5G50790
	AT5G07070			AT5G51060
	AT5G07322			AT5G51790
	AT5G07630			AT5G52120
	AT5G08975			AT5G52310
	AT5G09440			AT5G54190
	AT5G09590			AT5G54840
	AT5G09660			AT5G56100
	AT5G09820			AT5G56795
	AT5G10320			AT5G56850
	AT5G11290			AT5G57010
	AT5G12010			AT5G59260
	AT5G12140			AT5G59340
	AT5G13080			AT5G59780
	AT5G13910			AT5G60850
	AT5G13990			AT5G61010
	AT5G14000			AT5G62260
	AT5G14100			AT5G63140
	AT5G15120			AT5G63450
	AT5G15960			AT5G63600
	AT5G15970			AT5G63800
	AT5G16200			AT5G64660
	AT5G16360			AT5G64850
	AT5G16540			AT5G66400
	AT5G17430			AT5G66490
	AT5G17460			AT5G66790
	AT5G17670			
	AT5G19220			
	AT5G20700			
	AT5G21020			
	AT5G22270			
	AT5G22300			
	AT5G22890			
	AT5G23060			
	AT5G23120			
	AT5G23830			
	AT5G24910			
	AT5G25890			
	AT5G26310			
	AT5G27450			
	AT5G28320			
	AT5G28750			
	AT5G35170			
	AT5G37485			
	AT5G37940			
	AT5G38140			
	AT5G38520			
	AT5G38550			
	AT5G38660			
	AT5G38910			
	AT5G39190			
	AT5G40010			
	AT5G40100			
	AT5G41080			
	AT5G41663			
	AT5G42060			
	AT5G42570			
	AT5G42630			
	AT5G42850			
	AT5G43570			
	AT5G44130			
	AT5G44210			

AT5G44380			
AT5G44610			
AT5G44870			
AT5G44920			
AT5G45440			
AT5G46590			
AT5G46760			
AT5G46871			
AT5G46890			
AT5G47060			
AT5G47370			
AT5G47650			
AT5G49730			
AT5G49740			
AT5G51980			
AT5G52310			
AT5G52520			
AT5G53980			
AT5G54840			
AT5G56010			
AT5G56460			
AT5G57480			
AT5G58070			
AT5G58620			
AT5G59090			
AT5G59250			
AT5G59520			
AT5G59780			
AT5G60340			
AT5G60790			
AT5G62070			
AT5G62480			
AT5G63130			
AT5G63270			
AT5G63790			
AT5G64100			
AT5G64580			
AT5G64840			
AT5G65010			
AT5G65040			
AT5G66470			
AT5G66490			
